# Quantitative trait locus analysis of body shape divergence in nine-spined sticklebacks based on high-density SNP-panel

**DOI:** 10.1038/srep26632

**Published:** 2016-05-26

**Authors:** Jing Yang, Baocheng Guo, Takahito Shikano, Xiaolin Liu, Juha Merilä

**Affiliations:** 1 College of Animal Science and Technology, Northwest A&F University, Yangling, Shaanxi, China; 2 Ecological Genetics Research Unit, Department of Biosciences, University of Helsinki, Helsinki, Finland

## Abstract

Heritable phenotypic differences between populations, caused by the selective effects of distinct environmental conditions, are of commonplace occurrence in nature. However, the actual genomic targets of this kind of selection are still poorly understood. We conducted a quantitative trait locus (QTL) mapping study to identify genomic regions responsible for morphometric differentiation between genetically and phenotypically divergent marine and freshwater nine-spined stickleback (*Pungitius pungitius*) populations. Using a dense panel of SNP-markers obtained by restriction site associated DNA sequencing of an F_2_ recombinant cross, we found 22 QTL that explained 3.5–12.9% of phenotypic variance in the traits under investigation. We detected one fairly large-effect (PVE = 9.6%) QTL for caudal peduncle length–a trait with a well-established adaptive function showing clear differentiation among marine and freshwater populations. We also identified two large-effect QTL for lateral plate numbers, which are different from the lateral plate QTL reported in earlier studies of this and related species. Hence, apart from identifying several large-effect QTL in shape traits showing adaptive differentiation in response to different environmental conditions, the results suggest intra- and interspecific heterogeneity in the genomic basis of lateral plate number variation.

Adaptation to different environmental conditions is often, but not always^1^, accompanied by genetically based morphological divergence in size and shape^2^,^3^,^4^. While common garden experiments^5^,^6^,^7^ can verify the heritable nature of such divergence, uncovering the genetic basis of these complex phenotypic traits can be far more challenging^8^,^9^. For instance, adaptive genetic divergence in body shape among fish populations residing in different environments has been repeatedly demonstrated^10^,^11^,^12^, but the genetic underpinnings of this divergence are still fairly poorly understood^13^,^14^. This is not surprising, because body shape is a complex trait, likely to be highly polygenic: large sample sizes, both in terms of number of individuals and markers, are needed to identify the causal loci influencing variation in such traits^15^,^16^. The quest for understanding the evolution of body shape is further complicated by the fact that different aspects of body shape variability can be under conflicting selection pressures, and genetic correlations caused by pleiotropy and linkage disequilibrium can constrain or facilitate allele frequency changes in a given locus depending on the prevailing selection presures^17^.

The stickleback fishes (Gasterosteidae) provide excellent model systems for studies of the genetic architecture of body shape divergence. The three-spined stickleback (*Gasterosteus aculeatus*) has in fact been proposed as a model to study the evolution of body shape in fish^17^; recently the nine-spined stickleback (*Pungitius pungitius*)–which diverged from the three-spined stickleback around 13 million years ago^18^–has also been emerging as a model for evolutionary investigations^19^. These two species are ecologically, and to a certain degree also phenotypically, very similar^20^,^21^. Early mapping studies in three-spined sticklebacks have focused on simple morphological traits, such as pelvic reduction^22^,^23^ and armor loss^24^,^25^,^26^, and have been followed by studies focusing on the genetic architecture of complex traits including body shape variability^13^,^27^,^28^,^29^,^30^. Similarly to three-spined sticklebacks, freshwater populations of nine-spined sticklebacks have repeatedly and independently evolved deeper bodies, reduced armor, shorter caudal peduncles, smaller brains, and different behavioral characteristics as compared to marine populations^31^,^32^,^33^. Quantitative genetic studies conducted in ‘common garden’ conditions suggest an additive genetic basis for these morphological divergences^7^,^22^,^34^,^35^,^36^,^37^. Thus, nine-spined sticklebacks not only provide another promising model to examine the genetics of body shape divergence as an adaptation to life in freshwater environments, but also offer a chance to explore whether the genetic basis (i.e. common genes and/or genetic pathways) of body shape divergence is similar to that in the three-spined stickleback. Addressing this question can provide important insights into the potential role of genetic and developmental constraints in the evolution of complex phenotypes.

Identifying the genomic regions that control phenotypic variation is the first step towards understanding the genetic underpinnings of adaptive divergence among populations^38^,^39^. Quantitative trait locus (QTL) mapping is a classical method used for this aim. In the past, QTL-mapping in non-model organisms has relied on low-density genetic maps, typically comprised of only a few hundred molecular markers. Advances in genomic technologies have made it feasible to explore the genetic architecture of phenotypic traits at a genome-wide scale in both model and non-model species^40^,^41^,^42^,^43^. There are now several approaches (e.g., multiplexed shotgun genotyping^44^, reduced-representation sequencing^45^,^46^, and restriction-site associated DNA sequencing [RAD-seq]^47^,^48^) that allow the discovery and genotyping of thousands of markers across any genome of interest, even in non-model organisms with limited or no genomic resources^48^,^49^. RAD-seq has been utilized to construct high-density linkage maps and to detect QTL in an increasing number of studies^14^,^50^,^51^,^52^. Although the power and precision of QTL-mapping critically depends on the experimental design and number of mapped progeny^53^, Stange *et al*.^54^ demonstrated that high-density maps can increase the precision of QTL localization and effect sizes, especially for small and medium sized QTL, as well as the power to resolve closely linked QTL.

The main goal of this study was to investigate the genetic architecture of morphometric divergence between marine and freshwater nine-spined sticklebacks, with the aid of QTL-mapping using thousands of SNPs obtained with a RAD-seq approach. To this end, we used 283 F_2_-generation full-sib offspring derived from a F_1_-generation inter-cross between phenotypically and genetically divergent marine and freshwater populations. We mapped genomic positions in a total of 49 traits, including 38 principal component (PC) scores for body shape, 10 anatomical morphometric traits (Fig. 1), as well as lateral plate number. The detected QTL were compared to those observed in earlier studies of sticklebacks to see whether the same or different QTL for homologous traits were discovered in different studies. We also conducted functional annotation of the QTL regions in order to identify candidate genes controlling variation in studied traits.

## Results

### Linkage map

The linkage map used in this study included 14,998 unique SNP markers distributed across 21 LGs matching the expected number of chromosomes (2n = 42;^55^) in the nine-spined stickleback, and was adopted from Rastas *et al*.^56^. The sex-averaged map spanned 2,529 cM, with 5.99 markers/cM^56^. In this map, 5,241 SNPs distributed on 4,791 reference sequences could be uniquely mapped to the three-spined stickleback genome, and were defined as informative SNPs^56^. To overcome computational limitations in QTL mapping due to high-density of makers, a coarse-mapping was first conducted with a simplified linkage map with 466 informative SNPs (Supplementary Table 1), which was followed by fine-mapping with additional SNPs around QTL regions identified by the coarse-mapping (see Methods for details).

### Morphological variation

The original measurements of morphological traits in the F_2_ progeny are given in Supplementary Table 2. Principal Component Analysis (PCA), based on landmark positions, was used to identify the independent axes of body shape variation. This analysis identified 38 PCs, of which the first three each accounted for >10% of the total body shape variation (Supplementary Fig. 1). PC1, accounting for 35.2% of the total variation, captured primarily variation in body depth and caudal peduncle length. Along this axis, the F_2_ progeny varied from individuals having shallow bodies and long caudal peduncles to individuals having deep bodies and short caudal peduncles (Fig. 2). PC2 and PC3, accounting for 15.7% and 10.4% of the total variation, respectively, captured variation not only in the body depth and caudal peduncle length, but also in shape variation corresponding to bending of the body downwards (PC2) and upwards (PC3; Supplementary Fig. 1). Sexual dimorphism along PC1 and PC3 was evident (*t*-tests, *t*_281_ ≥ 7.36, *P* < 0.001; Fig. 2), and thus, sex was used as a covariate to control the potential gender dependent variation in the subsequent QTL-mapping analyses. No sexual dimorphism was detected in PC2 (*t*-test, *t*_281_ = −0.87, *P* > 0.05).

The ten continuous traits that were used in the QTL-mapping showed obvious divergence between wild collected marine (HEL) and pond (RYT) fish (Supplementary Fig. 2). For example, marine sticklebacks had longer caudal peduncles, narrower bodies, shorter lower jaws and snouts than pond individuals (Supplementary Fig. 2). Likewise, marine individuals had on average more lateral plates than pond individuals (Supplementary Table 3). Sexual dimorphism was evident in most of the traits (results not shown;^31^), and thus sex was included as covariate to all QTL-analyses.

### QTL-mapping

With coarse-mapping we detected a total of 22 QTL (ten for PC scores of body shape, four for anatomical measures, and eight for lateral plate number) on 11 different LGs, that were significant at the genome-wide level (Supplementary Table 4). The significant QTL for anatomical measures were associated with lower jaw length, caudal peduncle length, body depth, and snout length, whereas no significant QTL were found for the six other measures (Supplementary Table 4). After adding more markers around QTL regions detected with the simplified map, the linkage map for fine-mapping was significantly improved in terms of marker density–approximately 2.1 markers/cM. With fine-mapping, more accurate (as judged from narrower CIs) marker positions for each of the 22 QTL regions were obtained (Table 1; Figs 3, 4, 5). Eighteen of the 22 significant QTL identified in the coarse-mapping became replaced by new and more accurate QTL makers in the fine-mapping results (Table 1; Supplementary Table 4).

#### QTL for body shape variation

While ten QTL markers on seven LGs showed significant association with nine PC scores of body shape variation in the coarse-mapping (Supplementary Table 4), seven of these markers were refined in the fine-mapping analyses (Table 1; Fig. 3). The percentage of variance explained (PVE) by the individual QTL varied from 3.50% to 12.90% (Table 1). A large effect QTL (PVE > 10%) was detected on LG7 (6.98 cM) for two PC scores (PC6 and PC11). In addition to the large effect QTL, another QTL was found on LG7 for PC3 (15.48 cM), which was also affected by a QTL on LG8 (76.07 cM). Two QTL on LG17 were associated with PC16 and PC20 (31.00 and 43.35 cM, respectively). QTL were also found on LG4 (78.06 cM), LG5 (27.59 cM), LG14 (87.17 cM), and LG15 (22.66 cM) for PC14, PC13, PC33, and PC1, respectively (Table 1).

#### QTL for anatomical measures

All of the four QTL for anatomical measures detected in coarse-mapping were also retrieved by the fine-mapping, which yielded significant (at genome-wide level) QTL for body depth, caudal peduncle length, lower jaw length, and snout length (Table 1; Fig. 4). Except in the case of lower jaw length, fine-mapping refined the QTL-positions obtained from the coarse-mapping. A QTL for body depth was on LG4 (56.87 cM) with the PVE of 8.60%, a QTL for lower jaw length on LG19 (105.58 cM) with PVE of 6.70%, a QTL for snout length on LG20 (46.8 cM) with PVE of 7.40%, a QTL for caudal peduncle length on LG15 (12.11 cM) with the PVE of 9.60% (Table 1). The F_2_ progeny with different QTL genotypes in these loci differed significantly in their mean phenotypic values (Fig. 6): individuals with AA genotype (pond allele) on marker 27323 had significantly longer lower jaws (ANOVA-LSD, *P* < 0.01) than CC-homozygotes (marine allele); on marker 13320, individuals with CC genotype (pond allele) had significantly shorter caudal peduncles (ANOVA-LSD, *P* < 0.01) than GG-homozygotes (marine allele; Fig. 6). Likewise, individuals with AA genotype (pond allele) on marker 11319 had significantly deeper bodies (ANOVA-LSD, *P* < 0.01) than GG-homozygotes (marine allele); on marker 21583, individuals with CC genotype (pond allele) had significantly longer snouts (ANOVA-LSD, *P* < 0.01) than TT-homozygotes (marine allele; Fig. 6; Supplementary Table 5).

#### QTL for lateral plate number

At the genome-wide level, coarse mapping detected four, two, and two significant QTL for left, right, and total lateral plate number, respectively (Supplementary Table 4). In fine mapping, each of these QTL markers were replaced by new markers (Table 1; Fig. 5). QTL on LG20 (53.97 cM) and LG21 (84.89 cM) were significantly associated with variation in the left side, right side, and total plate number counts with PVEs ≥8.00% (Table 1). QTL significant at the genome-wide level were also found for the left (but not right) side plate numbers on LG8 (65.63 cM) and LG12 (76.76 cM, Table 1; Fig. 5). However, when mapping was performed for each chromosome independently, a suggestive QTL for right side and total plate number was detected on LG8 (65.63 cM), together with a suggestive QTL for total plate number on LG12 (76.76 cM). A small but evident LOD-score peak for right side plate number was observed on LG12 (76.76 cM) though it did not pass the significance threshold (Supplementary Fig. 3). Genotypes of the marker 12832 for the QTL on LG8 were missing in both of the grandparents, and thus origin of the alleles could not be identified. The F_2_ progeny with different QTL genotypes in the remaining three loci differed in their mean plate number values (Fig. 7): individuals with CC genotype (pond allele) on marker 22134 had fewer plates (ANOVA-LSD, left plate number: *P* < 0.05; right plate number: *P* > 0.05; total plate number: *P* < 0.05) than TT-homozygotes (marine allele); on marker 11482, individuals with TT genotype (pond allele) had significantly fewer plates (ANOVA-LSD, left plate number, right plate number, and total plate number: *P* < 0.01) than CC-homozygotes (marine allele); on marker 18769, individuals with CC genotype (pond allele) had significantly fewer plates (ANOVA-LSD, left plate number, right plate number, and total plate number: *P* < 0.01) than AA-homozygotes (marine allele; Fig. 7). Although several QTL were detected for lateral plate number variation, none of these were found on LG4, which contains the *Eda*-gene known to be the major gene controlling lateral plate variation in the three-spined stickleback^24^,^57^.

### Candidate genes for morphological divergence

All of the QTL regions identified in the fine-mapping could be annotated with the aid of the three-spined stickleback genome (Table 1). These regions contained several genes with functions related to, for example, RNA or protein binding, and cellular protein catabolic processes (Table 1). Most of the detected QTL markers were located outside of these genes; only one QTL for PC6 and PC11 was located within the *Ccdc90b*-gene (Table 1). In total, 92 genes were identified within the (1.5) confidence interval for a given QTL for shape variation, and 49 genes were identified for lateral plate number variation (Table 1). Pathway identification with the KEGG database showed that these 49 genes participate in several pathways, but none of them is involved in the *Eda*-pathway (cytokine-cytokine receptor interaction pathway) controlling for lateral plate number variation in the three-spined stickleback^24^,^57^.

## Discussion

The most important findings of this study include identification of several fairly large-effect QTL for phenotypic traits of ecological, evolutionary and systematic importance. Hence, the results can advance our understanding of the genetics and evolution of traits of major adaptive and systematic significance. Furthermore, by identifying three novel QTL (located on LG8, LG20 and LG21 respectively) associated with variation in lateral plate numbers which differ from those detected in earlier study of this^58^ and related species^23^,^24^, the results suggest a heterogeneous genomic basis for a trait of major evolutionary and systematic significance. Lateral plate number is an important diagnostic trait in taxonomy and systematics in the genus *Pungitius*^59^,^60^, and hence, our findings are relevant for determining the utility of this trait for systematic inference. Apart from these findings and considerations, the results serve to illustrate the possibilities and challenges associated with QTL-mapping with large number of markers as generated by RAD-seq. In the following paragraphs, we will discuss each of the above mentioned points in light of our findings and related issues.

An earlier study of morphometric divergence between pond and marine nine-spined sticklebacks–including the two populations used in this study–has suggested this divergence has a genetic basis^31^. Among other things, marine nine-spined sticklebacks have narrower and longer caudal peduncles than the pond fish. The caudal peduncle is associated with maneuvering and locomotion performance in fish^61^,^62^,^63^. The elongate peduncle increases the amplitudes needed to drive the caudal fin and allows for the control over the angle of attack of the caudal fin^64^,^65^, and is likely adaptive for fish moving in open water environments and under high predation risk. There is also considerable activity in the caudal peduncle when a fish changes its direction of movement^65^. Here, we identified a significant QTL on LG15 for the variation of peduncle length between marine and pond populations, indicating that the allele from marine population contributes to elongation of the caudal peduncle. The results suggest that the detected QTL may provide a starting point to decipher the genetic underpinnings and molecular mechanisms of adaptive divergence in caudal peduncle length in sticklebacks. Given that the strength of predator-mediated selection on caudal peduncle length can be quantified easily in mesocosm settings in sticklebacks^66^, the discovery of fairly large effect QTL in caudal peduncle length might also provide an opportunity to study dynamics of genetic variation in QTL under directional selection.

Variation in the number of lateral armor plates in stickleback fishes has received considerable attention for at least two reasons. First, being a conspicuous, variable and easily studied trait, variation in plate numbers has been used as a diagnostic trait in stickleback systematics^59^,^60^. Second, at least in the three-spined stickleback, the adaptive value of variation in lateral plate numbers is fairly well understood^67^, and several studies have demonstrated the adaptive nature of temporal^68^,^69^ and spatial^24^,^70^,^71^,^72^ variation in plate numbers. While the genetic basis of lateral plate number variation in the three-spined stickleback is controlled by a major QTL in the locus close to the *Eda*-gene, together with several minor QTL^24^,^57^, different large effect QTL have been identified to control variation in lateral plate numbers in North American nine-spined sticklebacks^58^. In this study, we detected two large QTL (located on LG20 and LG21) for lateral plate numbers, which were different from those discovered in North American nine-spined sticklebacks (Table 2). None of the genes located near these two QTL are involved in the *Eda*-pathway (cytokine-cytokine receptor interaction pathway) according to current gene annotation information, suggesting that the genetic mechanisms controlling for lateral plate variation may be even more variable than previously anticipated. To this end, our findings give support to the view that similar morphological changes might be commonly achievable through different QTL and/or genetic pathways^73^,^74^, albeit more interpopulation crosses and families would be needed to verify such a conclusion.

If the genetic basis of variation in armor traits in sticklebacks frequently differs from one population and species to another, and is subject to recurrent losses and gains over short evolutionary time scales^69^, lateral plate phenotypes may carry little information about systematic relationships among different taxa. Hence, the application of this trait in *Pungitius* systematics (see: ref. 60 for a review) may not be warranted. We also note the QTL on LG8 that influences variation in lateral plate numbers on the left side of the body was near to the QTL region influencing body shape (Figs 3 and 5). Earlier quantitative genetic^30^ and QTL-mapping studies^13^,^27^ have observed genetic links between shape and armor traits. Such results might help to explain why body shape differentiation in sticklebacks is often accompanied by plate number differentiation during evolutionary adaptation to freshwater environments.

In this study, we identified ten significant QTL contributing to divergence in body shape, and an additional 12 QTL contributing to variation in anatomical morphological traits and lateral plate number. All of the detected QTL had fairly large PVE values (average PVE = 8.48%) and some can be considered as large effect QTL (PVE > 10%) according to conventional standards^75^,^76^,^77^. A notable feature of our results is that for most traits–with the exception of PC3 and lateral plate numbers–only one single QTL was detected for each trait. While such results could be interpreted to suggest that single genes with large effects, rather than many genes with small effects, contribute to the observed phenotypic variability, such a conclusion may not be warranted from our data. Namely, the possibility that many genes with small effects contribute to the shape variation cannot be dismissed, as QTL studies are biased towards detecting QTL with large effects^78^,^79^. For instance, although we used a large number of markers, the modest size of our experiment in terms of number of F_2_-progeny (from a single family) may not have allowed the detection of many small effect QTL^80^,^81^. Furthermore, our decision to use stringent genome-wide significance as a criterion for calling QTL lead to the exclusion of many (n = 95) QTL which reached significance only at a chromosome-wide level. We believe that their exclusion from further considerations was justified given the statistical, and thereby also biological, uncertainty associated with them. It should also be pointed out that variation in shape is a cumulative effect of variation in multiple principal components, and hence of multiple QTL, even if variation along each individual principal component axis would be coded by a single or few QTL. Considering all these points, our results are not at odds with the view that complex morphological traits, such as shape, are likely to often have a polygenic basis^82^,^83^,^84^.

Previous studies have shown that different aspects of shape and morphology, such as lateral plate numbers, have evolved in similar directions in different freshwater populations of sticklebacks^11^,^13^,^31^,^58^,^85^. Such parallel evolution of trait complexes would not be likely if there were strong antagonistic genetic correlations among traits selected to change in a parallel fashion. However, quantitative genetic studies of sticklebacks suggest positive genetic correlations among, for instance, lateral plate numbers and several shape traits^30^. The ultimate source of these genetic correlations is pleiotropy and physical linkage among loci influencing variation in different traits. In this study, we found that one QTL region on LG7 (6.79–6.98 cM) affected two (by definition independent) principal component scores (PC6 and PC11; Table 1). This observation suggests that the same genes or genetic regions can control different components of shape variation, a characteristic that might facilitate rapid population divergence in shape. Likewise, a short genomic region on LG20 (46.61–53.97 cM) was associated with variance in both lateral plate numbers and snout length, indicating that the same genetic factor(s) may govern (part of) the variability in these two traits. However, whether a single pleiotropic gene or multiple linked genes control variation in both traits cannot be assessed from our data. The same applies to the interpretation of QTL for each individual PC-axis: since the shape variation captured by each PC-axis captures variance in multiple landmark coordinate positions, a QTL for a given PC-axis can be inferred to have pleiotropic effects on multiple landmark positions.

For all of the 22 QTL we detected, the precision of the QTL locations were very accurate, as judged from the narrow confidence intervals around the QTL positions. This high precision is also apparent if we compare the average width of the confidence region in this study with those of the earlier QTL studies of sticklebacks (Supplementary Fig. 4). The high precision of the QTL regions in this study is likely due to the higher density of markers than any of the earlier studies, as well as the fine-mapping approach, which narrowed the confidence intervals for the QTL positions (compare CIs in Table 1 and Supplementary Table 4). Interestingly, the high precision of QTL locations allowed us to discover that most (21/22) of the QTL were located in non-genic regions. We can identify three possible explanations for this. First, it could be that the RAD-seq method is biased towards finding polymorphic SNPs adjacent to genes rather than within. For instance, if there are more restriction sites outside than within genic regions, this would lead to a bias in detecting more non-genic than genic QTL. However, comparing the distribution of restrictions sites in the three-spined stickleback genome does not suggest such a bias: the number of *Pst*I restriction sites on a given chromosome was significantly correlated with chromosome length (*r*_*s*_ > 0.98, *P* < 1.71 × 10^−16^), and about 63% of the restriction sites were located in the genic regions. Second, genic regions of a genome are known to evolve under more stringent constraints than non-genic ones^86^,^87^,^88^,^89^,^90^, and therefore, the likelihood of detecting polymorphic SNPs outside of genes may be increased. A third and mutually nonexclusive possibility is that the variation associated with the detected QTL is not controlled by a sequence polymorphism within the genes, but in the regulatory regions outside of the genes. Our data do not allow us to disentangle these alternatives. However, given the increasing evidence for the importance of non-genic regulatory elements in controlling phenotypic variation^91^,^92^,^93^, it is possible that the majority of the detected QTL represent regulatory polymorphisms.

Most of the genes identified in each QTL region were classified into broad GO categories and pathways. Unfortunately, the exact function of most of these genes is poorly known. For instance, the QTL for PC6 and PC7 were mapped within the *Ccdc90b* gene, whose function is still uncharacterized. However, Meis homeobox 2a (*Meis2a*) and limb bud and heart homolog (*Lbh*) genes in the QTL region for PC1 are known to be primarily involved in the formation of the viscerocranium and craniofacial morphogenesis in zebrafish^94^ and cichlid fish^95^, respectively. Similarly, Fgf receptor-like 1a (*Fgfrl1a*) gene in the QTL region for PC14 was found to be necessary for cartilage formation in zebrafish^96^. The gene *Fxr1* in the QTL region for PC3 is an RNA binding protein that plays a critical role in eye development and cranial cartilage derived from cranial neural crest cells^97^. Hence, *Meis2a, Lbh, Fgfrl1a,* and *Fxr1* might be involved in adaptive evolution of stickleback by contributing to the regulation of cranial shape. In addition, since bone morphogenetic protein 4 (*Bmp4*) in the QTL region for PC1 has been reported to play an important role in the formation of the dorsal-ventral pattern in zebrafish^98^,^99^, this gene might be responsible for shape variation in the nine-spined stickleback. Another gene, identified in the QTL region for lateral plate number variation, codes for tRNA methyltransferase 1 like (*Trmt1l*) protein and has a wide set of functions including metal ion and RNA binding. The *Trmt1l* gene has been reported as a significant regulator in motor coordination and exploratory behavior in murine studies^100^. However, although associations between lateral plate number and behavioural variation in sticklebacks are known, it is not immediately obvious how and why variation in *Trmt1l* gene is associated with plate number variability in our cross. In addition, we found that the QTL associated with caudal peduncle length was located close to thioredoxin-related transmembrane protein 1 (*Tmx1*) gene. Unfortunately, little is known about the precise function of these two genes: *Tmx1* gene is a member disulphide isomerase gene family regulating highly conserved enzyme-mediated disulphide bond formation affecting over one-third of all eukaryotic proteins^101^.

Most of the earlier QTL-mapping studies of sticklebacks have utilized low-density microsatellite marker-based linkage maps^13^,^17^,^23^,^27^,^28^,^58^,^71^,^102^,^103^,^104^,^105^,^106^,^107^. Here, we used a high-density SNP-based linkage map generated by RAD-seq technology, which allows genotyping a very large number of SNP markers for many individuals in a single step^40^,^47^,^48^. The RAD-seq approach has been utilized to construct linkage maps for QTL-mapping purposes in several other species^38^,^50^,^51^,^52^,^108^,^109^, including various teleost fishes^14^,^110^,^111^,^112^,^113^,^114^,^115^,^116^. However, most of these earlier studies have used a modest number of markers (range = 436–8,790; median = 2,011) compared to the linkage map used in our study. Using a high number of markers poses challenges for both linkage map construction and QTL mapping. As for the linkage map construction, many of the available software are not fully automated and require considerable user involvement in map construction (but see: e.g. ref. 56). As for QTL mapping, we used a two-stage approach (cf. coarse + fine mapping) to overcome the computational challenges associated with large marker numbers. Although this approach is not expected to improve neither the QTL detection power nor the proportion of explained phenotypic variance^54^, it allowed us to obtain narrow confidence intervals and hence increase the precision of QTL locations. This strategy in combination with the multiple QTL mapping approach allowed us to further filter out likely false QTL peaks detected by simple (univariate) interval mapping. In fact, the relatively low number of significant QTL reported in this study likely owes to the facts that (1) we reported only the QTL significant at the genome-wide level, and that (2) some of the QTL detected with interval mapping disappeared in multiple QTL mapping. However, given this stringent approach for QTL detection, we feel fairly confident that the QTL we reported are not only real, but also very precise.

We used Procrustes shape coordinates to map shape variability in our data. While these variables derived with geometric morphometric methods have many virtues^117^, they may be sensitive to distortions if alignment of individual subjects in the original photographs is not perfect. In fact, such distortions, known as “bending”, are common^13^,^29^,^30^,^31^ and apparent also in our data in both PC2 and PC3. Yet, once used for mapping, several QTL were detected for both of these PCs. Since it is unlikely that such QTL would have been detected if the actual variation in landmark locations had been obfuscated by bending effects, we believe that the landmark coordinates we used were still biologically informative. However, even if the validity of mapping results for variation in PC2 and PC3 coordinates were questioned, bending is unlikely to have influenced variation in all (ten) anatomical morphometric traits that were also used in this study.

In conclusion, we mapped several large-effect QTL, with a fairly high-level of accuracy, to different linkage groups in the nine-spined stickleback genome using a large panel of SNP-markers. These QTL influence variation in morphological traits of ecological and evolutionary significance, and the documented effects of allelic substitution in the QTL loci we detected align with the expectations based on the divergence in trait means among pond and marine populations in the wild. Hence, the results have taken us one step farther towards identifying genomic regions underlying differentiation among nine-spined stickleback populations living in contrasting environments. Comparisons to previous QTL studies conducted in this and related stickleback species show that the large-effect QTL detected in this study do not correspond to those detected in previous studies. This suggests that adaption to similar selection pressures may have been acquired using different genetic mechanisms.

## Methods

### Fish collection, crossing, and rearing

Grandparental sticklebacks (F_0_) from the Baltic Sea (Helsinki, Finland; 60°13′N, 25°11′E) and a pond (Rytilampi, Finland; 66°23′N, 29°19′E) population were collected in 2006 and transported to the aquarium facilities of the University of Helsinki. Details of crossing and fish rearing methods can be found from earlier studies^112^,^113^,^118^. In brief, a randomly selected female from the marine population and a male from the pond population were crossed artificially in July 2006. After F_1_ offspring from this cross reached sexual maturity, one randomly selected male and female were mated repeatedly to generate a F_2_ full-sib family comprised of seven successive clutches. F_2_ progeny were raised individually in 1.4 L tanks in zebrafish rack systems (Aquaneering Inc., San Diego, USA) at 17 °C for 187 days after hatching and then killed with an overdose of Tricaine Methanesulfonate (MS-222). Larval and juvenile sticklebacks were fed with brine shrimp nauplii and adults with frozen bloodworms. A total of 283 F_2_ progeny of seven clutches were available. To obtain morphological data, the F_2_ individuals were photographed with a digital camera for a lateral view. A fin clip from each individual was stored in 96% ethanol for DNA sequencing. The specimens were then fixed in 4% formalin for further phenotypic measurements. In addition, wild marine (16 individuals) and pond fish (63 individuals) used as reference material in this study were the same as used in Herczeg *et al*.^35^, but re-measured by the same person (JY) who also measured the F_2_ offspring to ensure comparable measurements. The experiments were conducted under the license from the Finnish National Animal Experiment Board (#STH379A). All experimental protocols were approved by the ethics committee of University of Helsinki, and all experiments were performed in accordance with relevant guidelines and regulations.

### Morphological analysis

**Figure 1:**
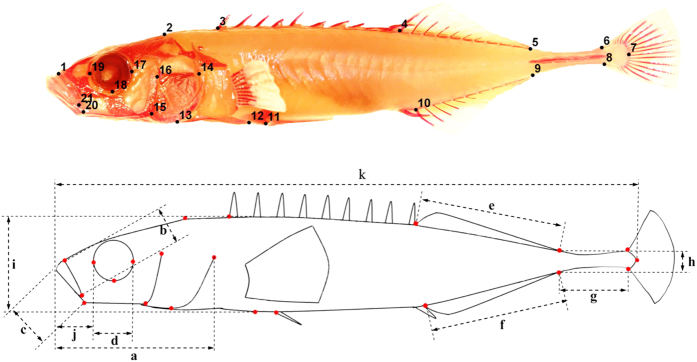
Landmark positions and definitions of anatomical measurements analyzed. Landmark positions: 1, Anterior extent of maxilla; 2, Posterior extent of supraoccipital; 3, Anterior insertion of first dorsal spine; 4, Anterior insertion of dorsal fin; 5, Posterior insertion of dorsal fin; 6, Origin of caudal fin membrane on dorsal midline; 7, Posterior extent of caudal peduncle; 8, Origin of caudal fin membrane on ventral midline; 9, Posterior insertion of anal fin; 10, Anterior insertion of anal fin; 11, Insertion point of pelvic spine into the pelvic girdle; 12, Posterior extent of ectocorocoid; 13, Anterior extent of ectocorocoid; 14, Posterior-dorsal extent of operculum; 15, Posterior-ventral extent of preopercular; 16, Dorsal extent of preopercular; 17, Posterior extent of orbit; 18, Ventral extent of orbit; 19, Anterior extent of orbit; 20, Anterior-ventral extent of preopercular; 21, Posterior extent of maxilla. Definitions of metric traits: a, head length; b, upper jaw length; c, lower jaw length; d, orbit diameter; e, dorsal fin base length; f, anal fin base length; g, caudal peduncle length; h, caudal peduncle width; i, body depth; j, snout length; k, standard body length. Measurement data and photos were collected by J. Y.

**Figure 2:**
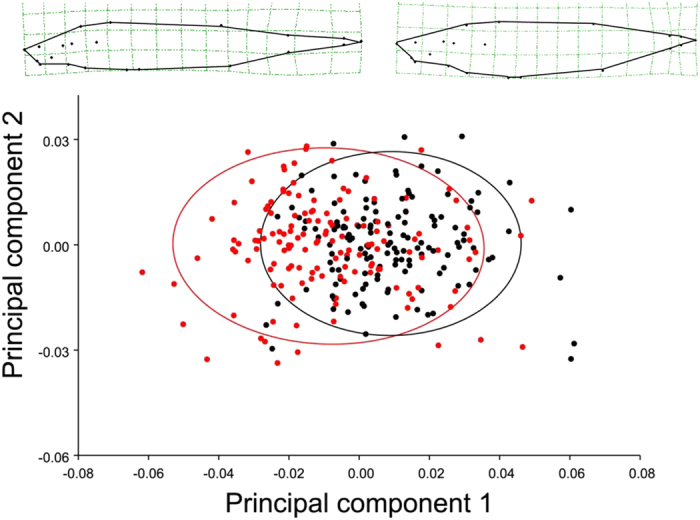
Sexual dimorphism in body shape in the F_2_ progeny used for mapping. Scatterplot of the first two principal component axe based on analysis of all landmarks. Black dots depict males, and red dots depict females. Wireframe graphs illustrate the body shape variation along the first principal component axis; black dots in the wireframes indicate the 21 landmarks used in shape analyses.

**Figure 3:**
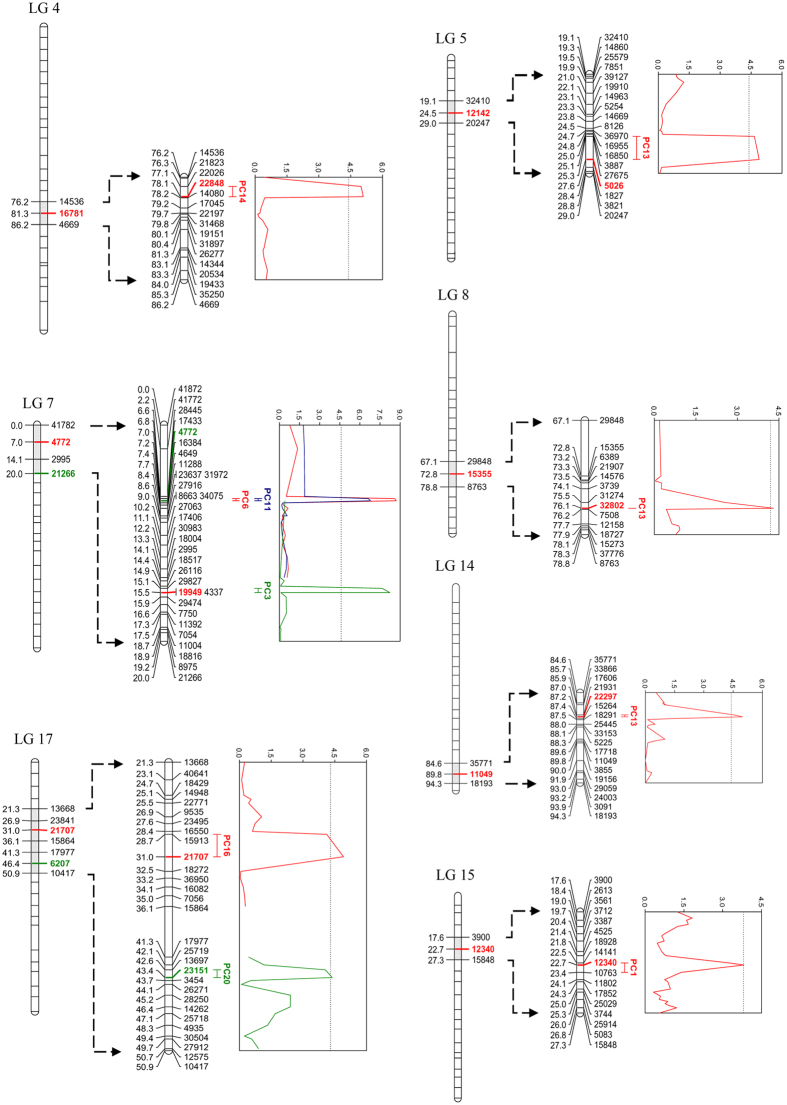
Significant QTL identified for body shape variance with fine-mapping. Significant QTL are marked by different colors. The QTL bars represent 1.5 unit confidence intervals. The graphs on the right side (Y-axis) of each linkage group show LOD score distribution, with dotted threshold line.

**Figure 4:**
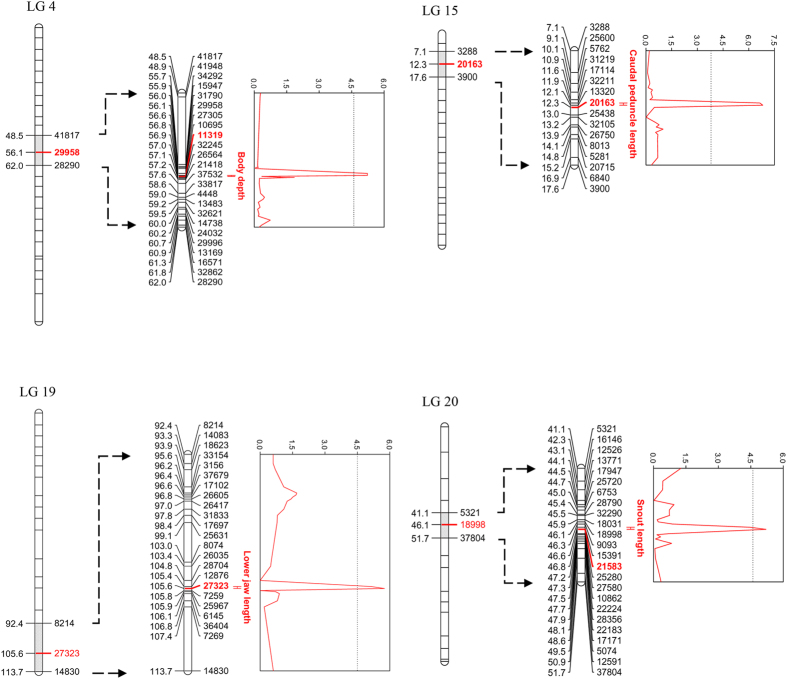
Significant QTL identified for anatomical morphological traits variance with fine-mapping. Significant QTL are marked by red color. The QTL bars represent 1.5 unit confidence intervals. The graphs on the right side (Y-axis) of each linkage group show LOD score distribution, with dotted threshold line.

**Figure 5:**
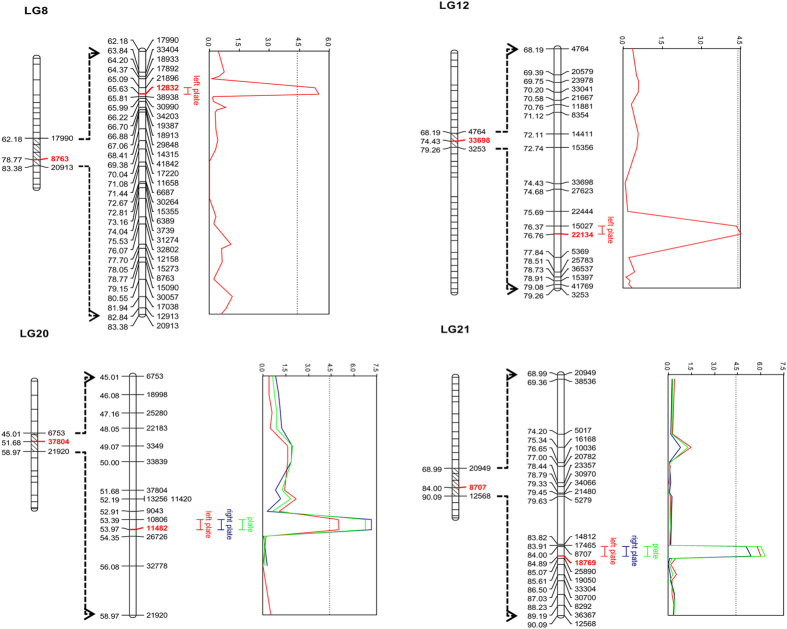
Significant QTL identified for lateral plate number variance with fine-mapping. Significant QTL for left, right and total lateral plate numbers are marked by different colors. The QTL bars represent 1.5 unit confidence intervals. The graphs on the right side (Y-axis) of each linkage group show LOD score distribution, with dotted threshold line.

**Figure 6:**
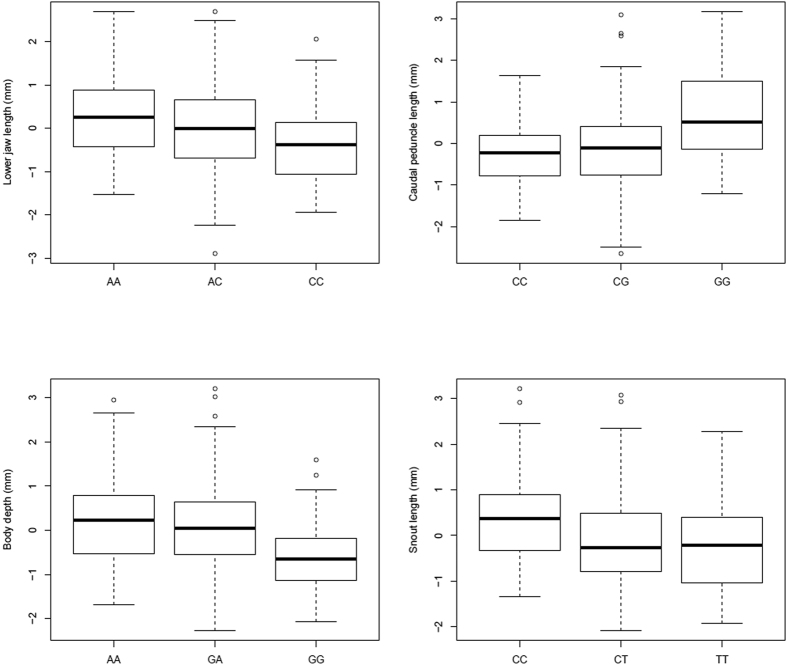
Median (bold line) lower jaw length, caudal peduncle length, body depth and snout length of nine-spined stickleback F_2_ progeny (n = 283) in different genotype classes for four QTL markers (a: 27323, b: 13320, c: 11319 and d: 21583). In each figure, genotype in left refers to pond genotype and that on right to marine genotype. Box indicates the lower and upper quartile values and whiskers represent the extreme values. Outliers are displayed by circles.

**Figure 7:**
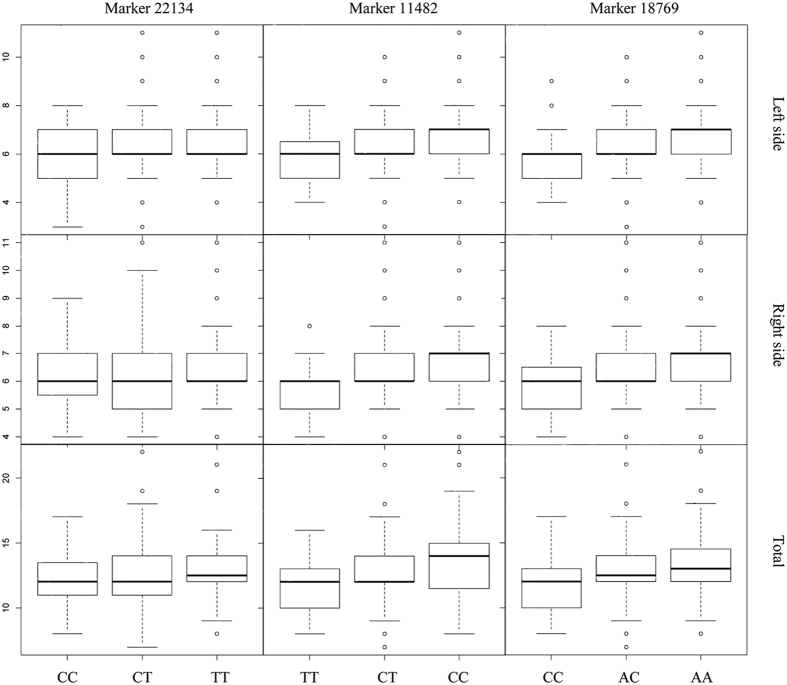
Median (bold line) of lateral plate numbers in the nine-spined stickleback F_2_ progeny (n = 283) in different genotype classes for three QTL markers (22314, 11482, and 18769). In each figure, genotype in left refers to pond genotype and that on right to marine genotype. Box indicates the lower and upper quartile values and whiskers represent the extreme values. Outliers are displayed by circles.

To quantify body shape variation, 21 landmarks (upper panel, Fig. 1) used in previous studies of three-spined^13^,^29^,^119^ and nine-spined sticklebacks^35^ were recorded from all F_2_ individuals using tpsDig v2.17^120^. MorphoJ^121^ was used to conduct geometric morphometric analyses of body shape variation. First, all the 21 landmarks were scaled and aligned through Procrustes superimpositions, and covariance matrix from the shape data was generated. Principal component analysis (PCA) was then applied to quantify shape variation among F_2_ progeny from the covariance matrix, working with partial warp scores. Since four degrees of freedom (cf. elimination of size, x and y coordinate positions, and orientation of specimens) were eliminated during Procrustes superimpositions^122^, variation along 38 (2*k*–4 = 38, where *k* = number of landmarks) PC axes were analyzed (see Results). PC scores were then used as separate traits to map QTL for shape variation. Although several earlier studies^13^,^27^,^123^,^124^ have used coordinates to map shape variation, our choice of using PC-scores was based on the fact that QTL inference based on shape coordinates can yield invalid inference^125^. Centroid size was also calculated with MorphoJ and used as covariate to control allometric effects during QTL mapping of PC scores. Apart from measurement of PC scores for the body shape, we also extracted information about variation in 11 anatomical measures (*viz*. head length, upper jaw length, lower jaw length, orbit diameter length, dorsal fin base length, anal fin base length, caudal peduncle length, caudal peduncle width, body depth, snout length, and standard body length) as defined in Fig. 1 (lower panel). In addition, the number of lateral plates on both sides of the body was counted from digital photographs. Each trait was measured or counted twice by the same person (JY), and the repeatabilities (R) of all measurements were high (R ≥ 0.89, *P* < 0.001 for all traits). Mean values of repeated measures were used in all analyses to minimize effect of measurement error. For QTL-mapping, the anatomical measurements were adjusted to variation in body size by regressing the trait values against standard body length and analyzing the residual variation. Sex of all F_2_ offspring was identified by gonadal inspection, and confirmed by genotyping a sex-linked microsatellite marker Stn19^126^.

### Genotyping and linkage map construction

Genomic DNA was extracted from ethanol preserved fin clips using the phenol-chloroform method^127^. RAD library construction and sequencing were performed by BGI HONGKONG CO., LIMITED. Briefly, DNA was fragmented by the restriction enzyme *Pst*I and DNA fragments of 300 to 500 bp were gel purified. Illumina sequencing adaptors and library specific barcodes were ligated to the digested DNA fragments and barcoded RAD samples were then pooled and sequenced on 24 lanes of the Illumina HiSeq2000 platform with 45 bp single end strategy. Adapters and barcodes were eliminated from reads and quality was checked using FastQC (http://www.bioinformatics.bbsrc.ac.uk/projects/fastqc/).

Details of SNP calling and linkage map construction for this data have been described in Rastas *et al*.^56^. In brief, the reads of parental individuals without any missing nucleotides (Ns) were pooled and identical reads were grouped together, and only reads occurring between 10 and 1500 times were kept and taken as reference contigs; reads of each individual were mapped against the reference contigs with BWA^128^, together with SAMtools^128^, producing a single bam file for each individual. Individual genotype posterior probabilities, taking into account the read and mapping qualities, were obtained from the output of ‘mpileup’ on the bam files. Only positions with two or more alleles and at least three reads for more than 158 individuals were considered. The parental genotypes were called by maximizing the likelihood of offspring and parent genotypes and then each offspring was called with respect to its called parental genotypes. Parental genotypes were called only if their likelihood was 100 times higher than the second best parental genotype combination and the offspring genotypes were called similarly as the parents. We are aware of that genotyping with RAD-seq could lead to biases, such as those introduced by mutations in cut sites^129^. However, as we used a F_2_ recombinant cross and removed RAD-tags with extremely low or high read numbers (see above), such biases are unlikely to have had any large effect on our results.

Linkage group (LG) assignment was obtained using the software Lep-MAP2^56^. First, SeparateChromosomes module was executed with LOD score limit 20 and minimum LG size of 10. Second, singular markers were added to the found LGs using JoinSingles module with LOD score limit of 10. Markers with more than 40 missing genotypes were removed from the LGs. Lep-MAP2 filters out markers by comparing the offspring genotype distribution and the expected Mendelian proportions with the dataTolerance of 0.01. Hence, all markers showing segregation distortion were filtered out and did not enter in the linkage map. The marker order was found using Lep-MAP2 allowing different recombination probabilities in both sexes. Ten independent runs were run and the marker order with best score^56^ was kept. Finally, markers with genotype error rate >0.1 were removed as well as markers from the map ends if they contributed over 10 cM (per marker) to map length. Likewise, if the parental coverage was above 500 (likely repetitive sequence) or below 20 (likely haplotype), these markers were removed.

### QTL-mapping

Due to the lack of nine-spined stickleback genome sequence, we annotated the linkage map by utilizing the three-spined stickleback genome resource (Ensembl release-75). We used BLAST search with an E-value cutoff at 1 × 10^−5^ based on the genomic divergence between nine- and three-spined sticklebacks^130^ to map the nine-spined stickleback reference sequences with SNPs onto the three-spined stickleback genome. SNPs that could be uniquely mapped on the three-spined stickleback genome are defined as “informative SNPs”.

QTL-mapping analyses were performed with a two-stage approach (i.e. coarse+fine mapping) to overcome computational limitations in QTL mapping with a large number of markers^131^. To identify QTL across the genome, we initially conducted coarse-mapping using a simplified linkage map consisting of 466 randomly selected informative SNPs at intervals of approximately 5 cM. After the coarse-mapping, fine-mapping was performed with additional SNPs located near/in the detected QTL region(s) to provide better accuracy for QTL positions. The additional SNPs were selected based on a linkage map with 14,998 unique SNPs^56^ and added to the simplified linkage map to create a high-density linkage map for the fine-mapping.

QTL mapping was conducted using MapQTL 6.0^131^ based on interval mapping (IM) with the following parameters: mapping step size of five, maximum of 200 interactions, and a functional tolerance value of 1 × 10^−8^. Sex was included as a factor to control the gender dependent variation in the mapped traits. Significance of LOD thresholds were determined with 10 000 permutations in Permutation Test (PT) process using a significance level of *P* < 0.05. To confirm the IM results and to detect additional QTL, Multiple QTL-mapping (MQM) analyses were conducted using suggestive QTL as cofactors. Newly detected significant QTL were added as cofactors, and a new round of MQM mapping was then performed. This was repeated until no new significant QTL were detected^128^. Based on the population variance among F_2_ progeny, the proportion of the phenotypic variance explained (PVE) by a QTL was calculated in MapQTL 6.0. These procedures were followed both for coarse- and fine-mapping rounds. 1.5-LOD support intervals were calculated to provide 95% confidence interval for each QTL^14^,^132^.

### SNP annotation and gene ontology analysis

The three-spined stickleback genome annotations were downloaded from Ensembl (release-75). BEDTools 2.17.0^133^ was used for annotation of the subset of SNPs identified by QTL-mapping to see whether the SNPs were located within or in proximity of known genes. Since the confidence intervals around detected QTL contained markers that we were unable to locate on the three-spined stickleback genome, we opted for the following approach. In brief, genomic intervals between the closest informative marker to the given QTL and the ends of its QTL confidence intervals were located in the three-spined stickleback genome. All the genes within these genomic intervals were then listed and candidate genes for given QTL were identified on the basis of gene ontology information. If gene ontology information did not retrieve any obvious candidate genes, none were called (but the identities of the genes within the search interval are reported). Gene ontology (GO) terms of the three-spined stickleback genes were retrieved with BioMart^134^ from Ensembl.

### Data accessibility

All data used in this study was given in the supplementary materials. The linkage map and genotype data are given in Supplementary Table 1. The original measurements of morphological traits in the F_2_ progeny are given in Supplementary Table 2.

## Additional Information

**How to cite this article**: Yang, J. *et al*. Quantitative trait locus analysis of body shape divergence in nine-spined sticklebacks based on high-density SNP-panel. *Sci. Rep.* **6**, 26632; doi: 10.1038/srep26632 (2016).

## Supplementary Material

Supplementary Information

## Figures and Tables

**Table 1:** Significant QTL detected with fine-mapping.

Trait	LG	QTL (Nearest marker)	Position (cM)	LOD	PVE (%)	1.5 CI (cM)	Genes with in 1.5. C.I.	
							No.	Genes
PC1	15	12340	22.66	3.81	3.5	22.47–23.37	17	***Meis2a**, C15orf41, ZNF770, Aqr, ACTC1(3 of 4), GJD2(2 of 2), STXBP6(2 of 2), Ddhd1a, Fermt2, **Bmp4**, Ypel5, Fut9a, Manea, Ppp1cb, PLB1(2 of 3), Lclat1, **Lbh***
PC3	7	19949	15.48	8.24	9.1	15.13–15.48	4	***Fxr 1**, Si: Ch211-14a17.7(5 Of 5), Ints2, Med13a*
	8	32802	76.07	4.3	4.9	76.07	14	*Rgs2, RGS13(1 Of 2), Uchl5, Glrx2, B3galt2, Aspm, Si: Ch211-198n5.11, Bcar3, Si: Rp71-1d10.8(1 Of 2), Depdc1a, Rpe65c, Fnbp1l, hps3, ttc14*
PC6	7	4772	6.98	8.72	12.9	6.79–6.98	1	Ccdc90b^*^
PC11	7	4772	6.98	6.79	10.8	6.79–6.98	1	Ccdc90b^*^
PC13	5	5026	27.59	4.89	7.8	25.29–27.59	11	*Si:Dkey-197c15.6, REXO4, KCNC3(2 Of 2), KCNA7(1 Of 2), Fgf21, Ppfia3, Zgc:195001(1 Of 2), Mybpc2b,ACPT, Lrrc4bb, HS3ST2(1 Of 2)*
PC14	4	22848	78.06	5.09	8.2	77.06–78.06	13	*Nitr13, **Fgfrl1a**, Maea, KLHL3, Hnmpa0l, Zgc:63568, Si: Ch211-255i20.3, Spon2b, Fam13b, Cxcl14, Lingo2a, Eif4ea, Adh5*
PC16	17	21707	31	4.91	7.8	28.74–31.00	6	*Suclg2, Fam19a1a, Eogt, Tmf1, Uba3, Fgd5a*
PC20	17	23151	43.35	4.38	7.1	42.63–43.35	14	*Evc2, MSX2, Stx18, Tacc1, Loxl2a, Rplp0(1 Of 2), Aggf1, R3hcc1, Golga7, Rplp0(2 Of 2), PXN(1 Of 2), MYL2(1 Of 2), CIT(I Of 2), Crybb3*
PC33	14	22297	87.17	4.98	8	86.99–87.17	12	*Tia1, DTWD2, Si:Ch1073-398f15.1, JMY(2 Of 2), HOMER1(2 Of 2), Dmgdh, ARSB, AP3B1(2 Of 2), Tbca, Otpa, Wdr41, Pde8b*
Lower jaw length	19	27323	105.58	5.75	6.7	105.42–105.58	14	*Calca, INSC, Zgc:113516, Sox6(1 Of 2), C11orf58, Ppp1r15b, Rps13, Pik3c2a, Si:Dkey-10o6.2, Tdg.1, Tdg.2, Nucb2b, Samm50, Api5*
Caudal peduncle length	15	13320	12.11	6.82	9.6	11.93–12.11	7	***Tmx1**, Atl1, Sav1, Nin, ABHD12B(2 Of 2), Pygl, Trim9*
Body depth	4	11319	56.87	5.35	8.6	56.81–56.87	5	*Xpnpep2, Trmt12, Zdhhc9, Sash3, Sytl4*
Snout length	20	21583	46.8	5.2	7.4	46.61–46.8	45	*Si:Ch73-22o12.1,Atp1a3b,Dedd1, Pou2f2a, Znf574, Erf, Gsk3ab, Cicb, Grik5, Ceacam1, Msh5, Abcb4, Rpp38, Rad54b, Epb41l4b, Cdh17, Gem, Rad54b, Si: Ch211-79l20.4, Ptpn3, Zgc: 153215, Tex10, Erp44, FRRS1L, Tmem245, Alg2, Scrt1a, Galnt1, Sec61b, Nr4a3, Invs, Stx17, Si:Ch211-197h24.6, Tmem67, Pdp1, Mf41l, Esrp1, Fam171a1, Nmt2, Crot, 13mbtl1b, Cnfn, Rundc3b, Tlr21, Pafah1b3*
Left side plate number	8	12832	65.63	5.45	8.7	65.09–65.63	28	***Trmt1l**, Mylk4b, Gmds, FOXQ1, Foxf2a, Foxc1b, Irf4b, DUSP22(1 Of 2), SLC22A23(1 Of 2), Tbc1d7, BPHL, Exoc2, DSP(1 Of 2), PSMG4, Dtymk, Agxta, Hdlbpa, Tns3.2, MARVELD3, C8orf82, Igfbp3(1 Of 2), Atg4b, Boka, Farp2, Naprt, PHLPP1(1 Of 2), Igfbp1a, Adcy1a*
	12	22134	76.76	4.5	7.3	76.37–76.76	6	*Pigt, Phactr3a, Ttll9, EPB41L1(1 Of 2), Cntn3a.1, Chl1a*
	20	11482	53.97	4.99	8	53.39–53.97	10	*Rusc1, Mf115, Polr3c, Dap3, Gba, Itga10, Crabp2a, Ca14, Prpf3, Rprd2b*
	21	18769	84.89	5.98	9.5	84.00–84.89	5	*Olfm3a, Abca4a, Tecrl2a, Arhgap29a, Prkdc*
Right side plate number	20	11482	53.97	7.16	11.3	53.39–53.97	10	*Rusc1, Mf115, Polr3c, Dap3, Gba, Itga10, Crabp2a, Ca14, Prpf3, Rprd2b*
	21	18769	84.89	5.34	8.6	84.00–84.89	5	*Olfm3a, Abca4a, Tecrl2a, Arhgap29a, Prkdc*
Total plate number	20	11482	53.97	6.75	10.7	53.39–53.97	10	*Rusc1, Mf115, Polr3c, Dap3, Gba, Itga10, Crabp2a, Ca14, Prpf3, Rprd2b*
	21	18769	84.89	6.27	10	84.00–84.89	5	*Olfm3a, Abca4a, Tecrl2a, Arhgap29a, Prkdc*

Candidate genes were listed in bold. “*” refers to QTL marker located within given gene.

**Table 2:** Comparison of QTL for shape and morphometric traits in nine-spined and three-spined sticklebacks.

Trait	This study	Other studies		References
	Nine-spined LG	Nine-spined LG	Three-spined LG	
Shape	4,5,7,8,14,15,17		1,2,4,7,9,11-14,16-21	Rogers *et al*.^29^
			1-5,7-9,12,13,15-21	Albert *et al*.^13^
			1,5,10,12,13,15-17,19-21	Liu *et al*.^34^
Lower jaw length	19	12		Shapiro *et al*.^58^
Caudal peduncle length	15			
Snout length	20		7	Rogers *et al*.^29^
Body depth	4		1,12,16,18	Rogers *et al*.^29^
Lateral-plate number	8,12,20,21	12		Shapiro *et al*.^58^
			4,7,10,21	Colosimo *et al*.^25^
			4,13,18,21	Cresko *et al*.^71^
			4,9,21	Liu *et al*.^27^
			13,21	Peichel *et al*.^23^
