# Supplementary material for: Quantitative trait locus analysis of body shape divergence in nine-spined sticklebacks based on high-density SNP-panel
Source: Sci Rep. 2016 May 26;6:26632. doi: 10.1038/srep26632 (PMC4880927; doi:10.1038/srep26632)
Supplement: Supplementary Information [file srep26632-s1.pdf]

Supplementary information

Quantitative trait locus analysis of body shape divergence in nine-spined sticklebacks based on high-density SNP-panel

Jing Yang[1,2], Baocheng Guo[2,*], Takahito Shikano[2], Xiaolin Liu[1,*] & Juha Merilä[2]

[1]College of Animal Science and Technology, Northwest A&F University, Yangling, Shaanxi, China

[2]Ecological Genetics Research Unit, Department of Biosciences, University of Helsinki, Helsinki, Finland

*Correspondence and requests for materials should be addressed to B.G. (baocheng.guo@helsinki.fi) or X.L. (liuxiaolin@nwsuaf.edu.cn)

Supplementary files legends

Supplementary Figure 1. The principal component analysis of body shape in nine-spined sticklebacks. a: Distribution of proportion of shape variance explained by 38 principal components. b: Body shape changes summarized by the first three principal components. The percentage of shape variation covered by each PC is shown. The light blue line represents a mean shape of F_2 progeny, and the dark blue line represents the shape variation.

Supplementary Figure 2. Divergence in ten metric traits (*viz.* lower jaw length, caudal peduncle length, body depth, snout length, upper jaw length, orbit diameter, dorsal fin base length, anal fin base length, caudal peduncle width and head length) between wild caught marine (HEL) and pond (RYT) nine-spined sticklebacks. Bold line indicates the median value and boxes the lower and upper quartile values. Whiskers represent the extreme values and outliers are displayed by circles. All values are estimated by adjusting individual trait values for differences due to sex and standard length. Asterisks indicate significant (*P < 0.05, **P < 0.01, ***P < 0.001) differences between the means.

Supplementary Figure 3. QTL-mapping results for lateral plate numbers. Shown are LOD-score distributions for left side, right side, and total plate count on LG8 and LG12. Dotted lines represent chromosome-wide threshold level for significance.

Supplementary Figure 4. Comparison of the confidence interval region in our study with those of the earlier QTL-studies of sticklebacks which have reported confidence

intervals for QTL they have detected.

Supplementary Table 1. Linkage map and genotype data used for QTL-mapping.

Supplementary Table 2. Original morphological measurements for all F_2 individuals.

Supplementary Table 3. Synopsis of morphometric variation nine-spined stickleback morphology among F_2-hybrids, pure marine and pure pond fish. Given are means, sample sizes (N), standard deviations, minimum and maximum values, and coefficient of variation for each trait.

Supplementary Table 4. Significant QTL regions detected with the coarse-mapping. LG, nearest QTL, position, LOD score, LOD threshold (genome-wide level), percentage of variation explained (PVE), 1.5 unit confidence interval (CI), and high density range used for fine mapping are shown for each trait.

Supplementary Table 5. Results of ANOVAs testing for differences in traits means between individuals with different genotypes for given QTL marker (Marker). Given are trait means, standard deviations (SD), minimum and maximum trait values, as well as denominator (ddf) and numerator (ndf) degrees of freedom with F-test values and associated P-values.

Supplementary Figure 1

a

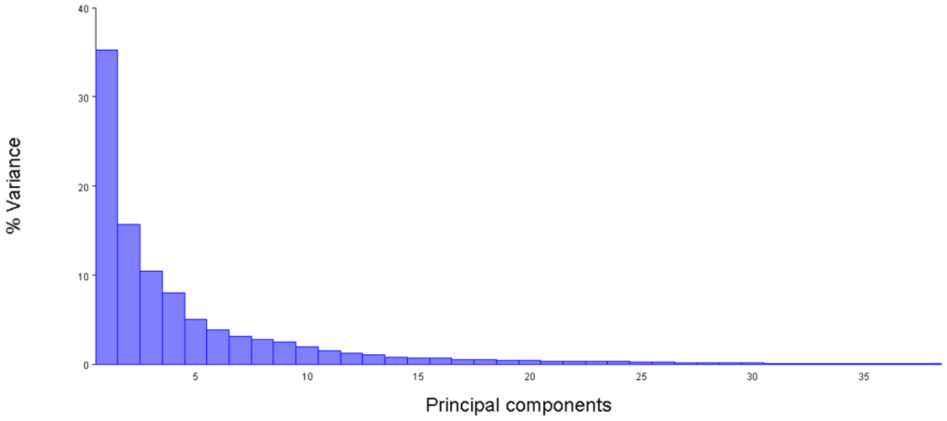

b

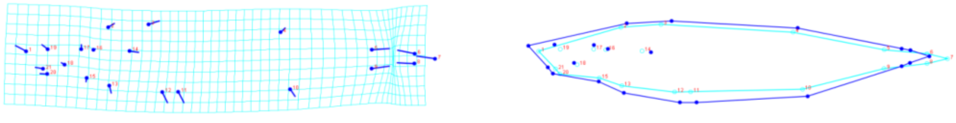

PC1 (35.228%)

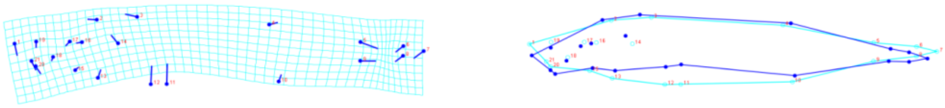

PC2 (15.657%)

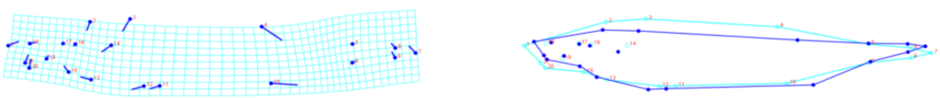

PC3 (10.445%)

Supplementary Figure 2

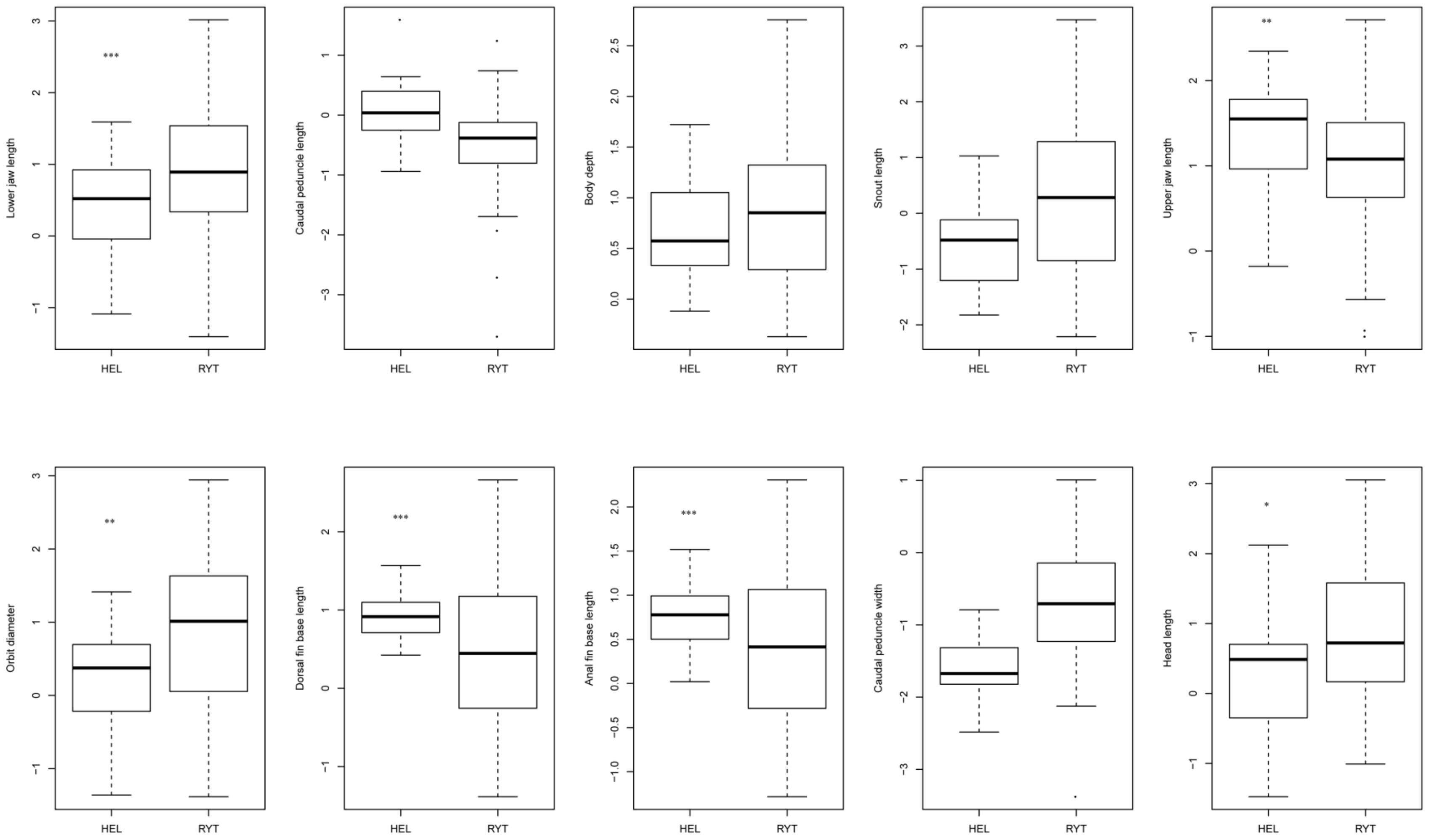

Supplementary Figure 3

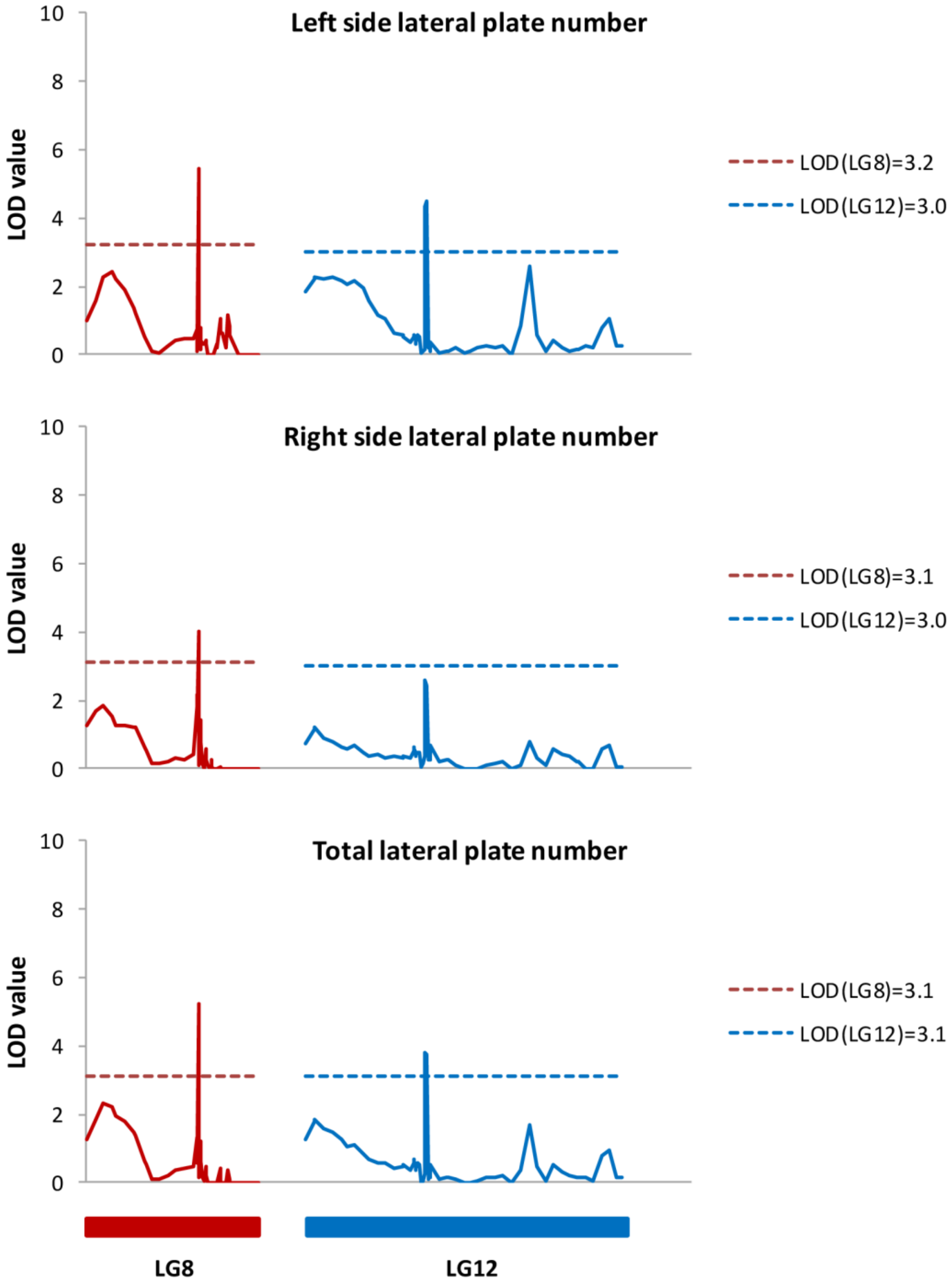

Supplementary Figure 4

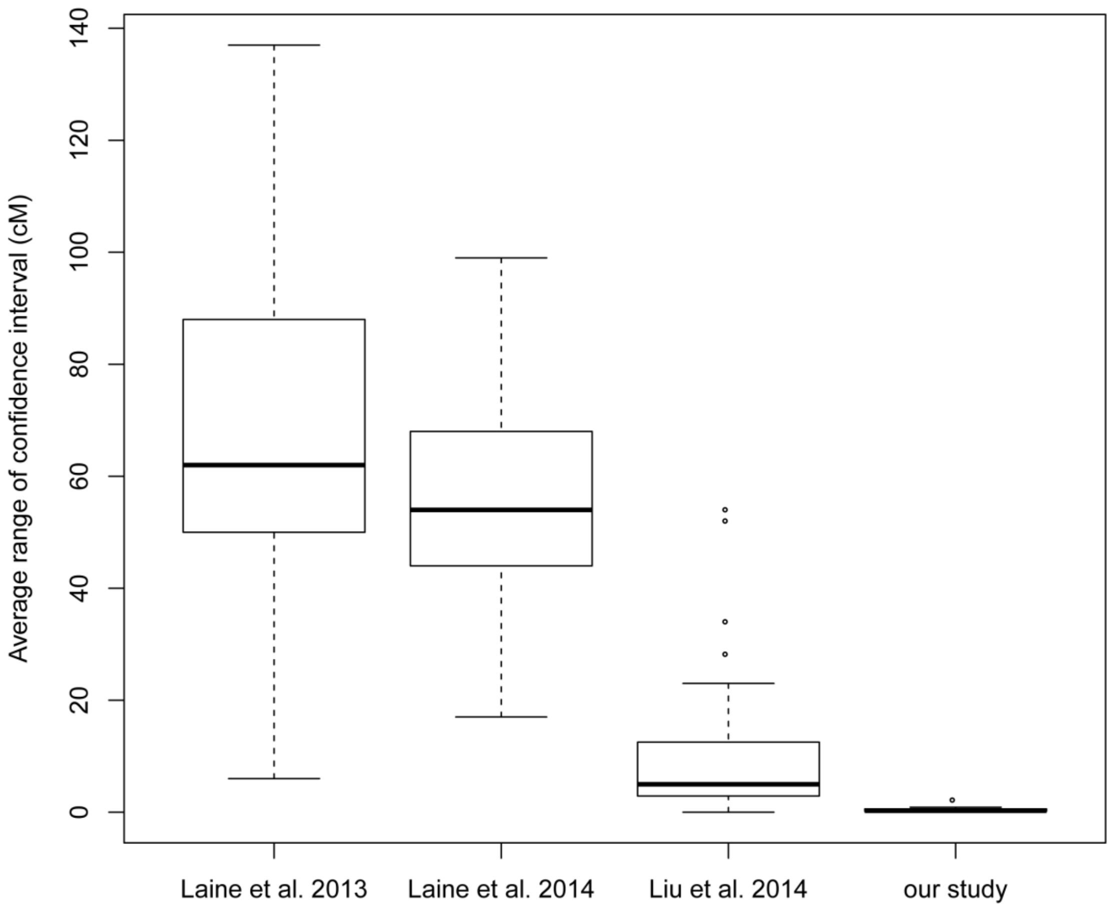

Supplementary Table 1: Linkage map and genotype data used for QTL-mapping.

Linkage Map			Genotype Data									
			Sample ID									
Group	Marker	Position	1-1	1-2	1-3	1-4	1-5	1-6	1-7	1-8	1-12	1-14
group1	16739	2.85	AC	AC	CC	AC	AC	AC	AC	AC	CC	CC
	25297	7.41	AG	AG	GG	AG	AG	AG	AG	AG	AG	GG
	30428	14.55	--	CT	CC	CT	--	CT	--	CT	CT	CC
	29240	17.78	AG	GG	AG	AG	AG	GG	GG	GG	AG	AG
	36457	20.28	AG	AG	GG	AG	AG	AG	AG	AG	AG	GG
	24595	25.03	CG	GG	GG	CG	CG	GG	GG	GG	CG	GG
	19469	27.63	CT	TT	CC	CT	CT	CT	--	CT	CT	CC
	1707	32.49	GT	TT	GG	GT	GT	GT	GT	TT	GT	GG
	10974	37.59	GT	TT	GG	GT	GT	GT	GT	TT	GT	GG
	17009	42.05	GT	TT	GG	GT	GT	GG	GT	GT	GT	GG
	17050	47.42	AG	AA	GG	AG	AG	GG	AG	AG	AG	GG
	35419	52.16	CT	CT	--	CT	CT	CC	CC	CT	CT	CC
	28067	57.87	AC	AA	CC	CC	AC	CC	AC	AC	AC	CC
	13287	62.86	AG	AG	AA	AA	AG	AA	AG	AG	AG	AA
	29184	67.32	CC	CT	CC	CC	CC	CC	CT	CC	--	CC
	13244	72.19	AG	GG	AA	AA	GG	AA	GG	AG	GG	AA
	23710	77.21	AT	TT	--	TT	AT	TT	AT	AT	AT	TT
	28262	82.48	CC	CT	CC	CC	CT	--	CT	CC	CT	CC
	4645	87.28	GT	TT	TT	TT	TT	TT	GT	GT	GT	TT
	15667	92.09	CG	CC	CC	CC	CC	CC	CG	CG	CG	CC
	22752	97.55	AT	AT	--	TT	AT	TT	AA	AT	AA	TT
	32944	102.29	CT	--	TT	TT	CT	TT	CC	CT	CC	TT
	26424	105.02	AT	AT	AA	AA	AT	AA	TT	AA	TT	AA
	12553	106.83	AT	AT	AA	AA	AT	AA	TT	AA	TT	AA
	19805	107.00	AG	AG	AA	AA	AG	AA	GG	AA	GG	AA
	27349	107.54	GT	TT	--	TT	TT	TT	GT	GT	GT	TT
	4512	107.71	GT	GT	TT	TT	GT	TT	GG	GT	GG	TT
	2138	107.88	AT	AT	TT	TT	AT	TT	AA	AT	AA	TT
	5548	108.96	CT	CT	TT	TT	CT	TT	CC	TT	CC	TT
	16543	109.31	CG	CG	CC	CC	CG	CC	GG	CC	GG	CC
	4712	109.67	CT	CT	CC	CC	CT	CC	TT	CC	TT	CC
	14233	109.74	CC	AC	AA	AA	AC	AC	--	AC	CC	AA
	34197	109.78	AG	AG	AA	AA	AG	AA	GG	AA	GG	AA
	25434	109.85	GT	GT	GG	GG	GT	GG	TT	GG	TT	GG
	32176	110.05	AG	AG	AA	AA	AG	--	GG	AA	GG	AA
	34617	110.46	GT	--	TT	TT	GT	TT	GG	TT	GG	TT
	10528	110.66	AG	AG	GG	GG	AG	GG	AA	GG	AA	GG
	19203	110.86	--	CT	--	--	CT	CC	TT	CC	TT	CC
	13500	111.06	AT	AT	AA	AA	AT	AA	TT	AA	TT	AA
	22416	111.17	AG	AG	AA	--	AG	AA	GG	AA	GG	AA
	8380	111.27	AC	AC	AA	AA	AC	AA	CC	AA	CC	AA
	31862	111.44	AG	AG	AA	AA	--	AA	GG	AA	GG	AA

	17541	111.62	CT	CT	TT	TT	CT	TT	--	TT	CC	TT
	3908	117.28	CG	CG	CC	CC	CG	CC	GG	CC	GG	CC
	14816	121.81	CT	CT	TT	CT	CT	TT	--	TT	CC	TT
	29901	126.51	CT	CT	CC	--	CT	CC	TT	CC	TT	CC
	6747	131.33	AC	AC	AA	AC	AC	AC	CC	AA	CC	AA
	17888	143.25	CC	TT	CT	CC	TT	CT	TT	CC	TT	CC
	15804	152.66	TT	CC	--	TT	CC	CT	CT	TT	CT	TT
group2	31439	1.23	--	AG	AG	AA	GG	AG	AG	AG	AG	AA
	4508	11.66	GG	GT	GG	GT	GG	GT	GT	GT	GT	TT
	32823	19.42	AG	AG	GG	AG	GG	AA	AG	AG	AG	AA
	10350	24.23	CT	CT	TT	CT	TT	CC	CT	CT	CT	CC
	23317	29.07	CG	CG	CC	CG	CC	GG	CG	GG	CG	GG
	3852	34.07	AG	AG	AA	AG	AA	GG	AG	GG	AG	GG
	28911	39.00	AC	AC	CC	AC	CC	AA	CC	AA	AC	AA
	27612	43.84	AG	AG	AG	AG	AG	GG	AG	GG	GG	GG
	13298	48.02	--	CT	--	CT	TT	CT	TT	CC	CT	CT
	6773	51.67	CT	CT	CC	CT	CC	CT	CC	TT	CT	CT
	13217	56.13	CC	CT	--	CT	TT	CT	--	CC	CT	CT
	8723	59.69	GG	AG	AA	AG	AA	AG	AA	GG	AG	AG
	2344	64.90	AC	AC	AA	AA	AA	AC	AA	AC	AC	AA
	7714	69.36	GG	AG	AA	AA	AA	AG	AA	AA	AG	AA
	22186	74.70	CG	CG	--	--	GG	CG	GG	GG	CG	GG
	12388	78.97	CG	CG	CC	CG	CC	CG	CG	CC	CG	CC
	3534	83.55	GG	AG	AA	AG	AA	AG	AG	AA	AG	AA
	18164	88.65	AA	AC	CC	AC	CC	AC	AC	CC	AC	CC
	30372	94.47	CC	CC	TT	CT	TT	CT	CT	TT	CT	TT
	22841	99.81	TT	TT	CC	CT	CC	--	CT	CC	--	CC
	14688	106.11	AA	AA	TT	AT	TT	AT	AT	TT	AT	TT
	6866	114.08	AG	AG	GG	GG	GG	AG	AG	GG	AG	GG
	29764	117.94	AT	AT	--	AT	--	TT	TT	TT	--	TT
group3	28203	0.00	TT	GT	GG	TT	TT	GG	GT	TT	GT	GG
	22537	5.09	--	AG	AG	GG	GG	AG	GG	GG	GG	AG
	2532	9.40	TT	CT	CT	TT	TT	CT	TT	TT	TT	CT
	20919	14.24	GG	AG	GG	GG	GG	AG	GG	GG	GG	AG
	14162	37.71	--	TT	GT	GT	GT	GT	GT	GG	GT	TT
	37398	42.43	--	GG	GG	GG	--	GG	GG	GG	GG	--
	5818	46.82	--	CC	CT	CT	CT	CT	CT	TT	CT	CC
	33307	50.91	TT	CC	CT	CT	CT	CT	CT	TT	CT	CC
	4401	55.24	AA	GG	AG	AG	AG	AG	AG	AA	AG	GG
	33621	60.23	GG	CC	--	CC	CG	CG	CG	GG	CG	CC
	12671	65.13	AA	GG	AG	GG	AG	AG	AG	AG	AG	GG
	33645	70.16	CC	TT	--	TT	CT	CT	--	CT	CT	TT
	17999	75.27	CC	TT	CT	TT	CT	CT	CT	CT	CT	TT
	2179	78.90	CC	CG	CC	CG	CC	CC	CC	CG	CC	CG
	34274	82.81	TT	AT	TT	AT	TT	TT	TT	AT	TT	AT
	18117	87.26	GG	GT	GG	GT	GT	GT	GT	GG	GT	GT
	13271	92.57	--	CT	CC	CT	CT	CT	CT	CC	CT	CT

	31752	97.66	CC	TT	CC	CT	--	CT	TT	--	TT	--
	17259	102.59	GG	GT	GG	GT	GT	GT	GT	GG	GT	GT
	12153	106.61	CC	TT	--	CT	CT	CT	TT	CC	TT	TT
	5530	111.61	TT	GT	TT	GT	GT	GT	GT	TT	GT	GT
	4202	114.87	--	CG	GG	CG	CG	CG	CG	GG	CG	CG
group4	38479	0.00	AG	AG	AA	AG	AA	AA	GG	AG	AG	GG
	15351	4.61	AG	AG	GG	AG	GG	GG	AA	AG	--	AA
	9411	9.84	AG	AG	AG	AG	GG	AG	AA	AG	AG	AA
	6434	14.72	CT	CT	CT	CT	CC	CT	TT	CT	CT	TT
	13077	19.55	AG	AG	AG	AG	AA	AG	GG	AG	AG	--
	14875	24.76	--	GT	GT	GT	TT	GT	GG	GT	GT	GG
	20908	30.58	AG	AG	AG	AG	AG	AG	AG	GG	GG	AG
	26929	35.09	TT	CT	CT	CT	CT	CT	TT	CT	CT	TT
	31954	40.12	--	AC	--	--	AC	AC	CC	CC	CC	CC
	11791	43.86	CT	CC	CC	CC	CC	CC	CT	CT	CT	CT
	34292	55.74	GG	GT	GT	GT	GT	GT	GT	GG	GT	GG
	15947	55.92	CC	CC	CC	CC	CC	CC	CT	CC	CT	CC
	14012	55.98	GG	CG	CG	CG	CG	CG	GG	GG	GG	GG
	31790	56.04	CC	AC	AC	AC	AC	AC	CC	CC	CC	CC
	29958	56.10	TT	TT	--	TT	--	TT	CT	TT	CT	TT
	27305	56.63	AA	AA	AA	AA	AA	AA	AG	AA	AG	AA
	10695	56.81	AA	GG	AG	AG	AG	AG	AG	AA	AG	AA
	11319	56.87	CT	CC	CC	CC	CC	CC	CT	CT	CT	CT
	32245	56.96	AA	AT	AT	--	AT	AT	AA	AA	AA	AA
	18586	57.02	CC	CT	CT	CT	CT	CT	CC	CC	CC	CC
	26564	57.11	AA	AG	AG	AG	AG	AG	AA	AA	AA	AA
	21418	57.17	CC	CT	CC	CC	CC	CC	CT	CC	CT	CC
	37532	57.57	--	AA	AT	AT	AT	AT	AT	TT	AT	TT
	33817	58.61	TT	AA	AT	AT	AT	AT	AT	TT	AT	TT
	4448	58.96	TT	CC	CT	CT	CT	CT	CT	TT	CT	TT
	13483	59.22	CC	CT	CT	CT	CT	CT	CC	CC	CC	CC
	32621	59.49	--	GT	GG	--	--	GG	GT	GG	GT	GG
	14738	60.03	--	CT	CC	CC	CC	CC	--	CC	CT	CC
	24032	60.19	AG	AA	AG	--	AG	AG	AA	AG	AA	AG
	29996	60.74	--	AA	AG	AG	AG	AG	AG	GG	AG	GG
	13169	60.92	AA	AG	AA	AA	AA	AA	AG	AA	AG	AA
	16571	61.28	--	TT	CT	CT	CT	--	CT	CC	CT	CC
	32862	61.81	CC	AC	CC	CC	--	CC	AC	CC	AC	CC
	28290	61.99	TT	AA	AT	TT	AT	AT	--	TT	AT	TT
	19493	62.08	--	AG	AG	AG	AG	AG	AA	AA	AA	AA
	8941	62.17	--	AC	AA	AA	AA	AA	AC	AA	AC	AA
	15855	62.34	TT	AT	TT	TT	TT	TT	AT	TT	AT	TT
	22823	62.52	AC	AC	CC	CC	CC	CC	AA	AC	AA	AC
	19354	62.70	CC	AC	AC	AC	AC	AC	CC	CC	CC	CC
	31000	62.87	AG	GG	AG	AG	AG	AG	GG	AG	GG	AG
	13423	63.05	AC	CC	CC	CC	CC	CC	AC	AC	AC	AC
	22747	63.23	AA	AG	AA	AA	AA	AA	AG	AA	AG	AA

	9506	63.41	--	CG	--	CG	CG	CG	CC	CC	CC	CC
	22053	63.63	TT	CC	CT	CT	CT	CC	CT	TT	CT	TT
	28694	64.29	CC	TT	CT	CT	CT	CT	CT	CC	CT	CC
	29737	65.19	CC	CT	CC	CC	CC	CC	CT	CC	CT	CC
	22643	65.37	--	AG	AA	AA	AA	AA	AG	AA	AG	AA
	20555	65.90	CC	TT	CT	CT	CT	CT	CT	CC	CT	CC
	34724	66.54	--	CT	TT	TT	TT	TT	CT	TT	CT	TT
	31101	66.97	--	TT	--	--	AT	AT	AT	AA	AT	AA
	12284	67.15	GG	AG	AG	AG	AG	AG	GG	GG	GG	GG
	23237	67.33	--	AC	AA	AA	AA	AA	AC	AA	AC	AA
	37249	67.86	--	CT	--	CC	--	--	CT	CC	CT	--
	15844	68.05	TT	GT	GT	GT	GT	GT	TT	TT	TT	TT
	11040	68.22	--	CT	--	TT	TT	TT	CT	TT	CT	--
	5573	68.39	AC	CC	AC	AC	AC	AC	CC	AC	CC	AC
	24594	68.75	--	AG	AA	AA	AA	AA	AA	AA	AG	AA
	8819	69.16	--	GT	--	GG	GG	GG	GT	GG	GT	GG
	33730	69.39	AT	TT	AT	AT	--	AT	TT	AT	TT	AT
	11875	69.57	AA	AG	--	AA	AA	AA	AG	AA	AG	AA
	19970	69.81	--	CT	CT	CT	CT	CT	CC	CC	CC	CC
	17613	69.99	--	CT	--	CC	CC	CC	CT	CC	CT	CC
	26031	70.17	AA	AC	AA	AA	AA	AA	AC	AA	AC	AA
	20789	70.35	CC	CT	CC	CC	CC	CC	CT	CC	CT	CC
	40117	70.54	--	TT	AT	AT	AT	AT	AT	AA	AT	AA
	7737	71.08	AG	AA	AG	AG	AG	AG	AG	GG	AG	GG
	9469	71.25	--	CG	CG	CG	CG	CG	GG	GG	GG	GG
	33712	71.42	CT	CT	TT	TT	TT	TT	CT	TT	CT	TT
	14536	76.16	AG	AG	GG	GG	GG	GG	AG	GG	AG	GG
	16781	81.33	AG	AG	AA	AA	AA	AA	AG	AA	AG	AA
	4669	86.16	AA	AG	AG	AG	AG	AG	AA	AA	AA	AA
	25154	91.41	--	AG	AG	AG	AG	AG	GG	GG	GG	GG
	32343	96.44	CT	CT	TT	TT	TT	TT	CT	TT	CT	TT
	33051	102.84	GT	TT	GT	--	TT	GT	GT	GG	GT	GG
	34468	104.13	TT	GT	GT	GT	GT	GT	TT	TT	TT	TT
	28260	109.43	GG	AA	AG	AG	AA	AG	AG	GG	AG	GG
	3016	113.20	CT	CT	CC	CC	CT	CC	CT	CC	CT	CC
	5154	119.65	GT	GT	TT	TT	GT	TT	GT	TT	GT	TT
	18033	132.28	AG	GG	GG	GG	GG	GG	AG	AG	AG	AG
	13442	1.19	AG	AG	AG	AG	GG	GG	AG	AA	AA	AG
	8752	4.39	--	CC	CC	CC	CC	CC	CC	CT	CT	CC
group5	24562	9.05	AA	AA	AA	AA	AT	AT	AA	AA	AA	AA
	16128	14.46	CG	CG	CG	CG	GG	GG	GG	CC	CG	CG
	32410	19.12	AC	AC	AC	AC	AC	AC	AC	AA	AC	AC
	12142	24.54	--	AG	AG	AG	--	GG	GG	AA	AG	AG
	20247	29.04	AG	AG	GG	AG	GG	GG	GG	AG	AG	AG
	17067	34.03	CT	CT	CC	CT	CC	CC	CC	CT	CT	CT
	9695	39.48	AG	AG	AA	GG	AA	AA	AA	GG	AG	AG
	8528	44.46	CT	CC	CC	CC	CC	CT	CT	CC	CT	CT

	8816	49.60	GG	GG	AG	GG	AG	AG	AG	AG	GG	GG
	27088	53.63	GG	AG	AA	AG	AA	AG	AG	AA	GG	AG
	9943	58.09	CT	CC	CT	CC	CT	TT	TT	CT	CT	CC
	14223	62.43	AT	AA	AT	AT	AT	TT	TT	AT	AT	AA
	14928	68.87	AC	AA	AC	AC	AA	--	AC	AC	AC	AA
	32644	72.17	--	TT	CT	CT	--	CC	CT	CT	CT	TT
	39858	77.93	AG	AG	AG	AG	GG	AG	AG	AG	--	GG
	24285	82.52	TT	CT	CT	CT	CC	CT	CT	CT	CT	CC
	28294	87.23	CT	CT	TT	CT	CT	TT	TT	TT	CT	TT
	31574	89.32	AG	GG	AG	AG	AG	AG	--	AG	GG	AA
	31004	9.92	AA	AA	AA	AG	GG	AA	AA	AG	AG	GG
	31599	14.15	--	CC	CC	CT	TT	CC	CC	CT	CT	TT
group6	30110	19.03	--	GG	GG	GG	AG	--	GG	GG	GG	AG
	26464	23.29	CC	CC	CC	CT	--	CC	CC	CT	CT	TT
	32335	28.49	GG	GG	GG	GG	CG	--	GG	CG	CG	CG
	31612	32.13	CC	CC	CT	CC	TT	CT	CC	CT	CT	--
	28298	37.02	--	CC	CT	CC	CC	CC	CC	CC	CC	CT
	9817	41.57	GG	AG	AG	GG	AG	AG	GG	AG	GG	AA
	29409	44.07	--	CT	CT	CC	--	CT	CC	CT	CC	TT
	4834	48.37	AA	AG	AG	AA	AG	AG	AA	AG	AA	AG
	3752	54.32	--	TT	CT	CC	CT	CT	CC	CT	CC	CC
	3518	59.30	GG	AA	AG	GG	AG	AG	GG	AG	GG	GG
	10072	62.51	GG	AG	AG	GG	GG	GG	GG	GG	GG	GG
	10470	67.52	TT	GG	GT	TT	GT	GT	TT	GT	TT	TT
	16995	72.77	CC	CG	CG	CC	CC	CC	CC	CC	CC	CC
	18145	77.21	CG	CC	CG	--	CC	CC	CG	CC	CG	CG
	28460	83.27	CC	TT	CT	CC	CT	CC	CC	CT	CT	CC
	21163	88.27	TT	CC	--	TT	CT	CT	TT	CT	CT	TT
	15853	92.90	--	TT	AT	AA	AT	--	AA	AT	AT	AA
	4099	98.77	AG	AA	AG	GG	AG	AG	AG	AG	AG	GG
	13597	102.33	--	TT	CT	CC	CT	CT	CT	CT	CT	CC
	3396	107.68	TT	TT	CT	TT	CT	TT	CT	TT	CT	TT
	27120	111.82	--	CC	AC	AA	AC	AC	AC	AC	AC	AA
	28445	6.60	GG	GG	GT	TT	TT	GG	GT	TT	GG	GG
	17433	6.79	--	GG	AG	AA	AA	GG	AG	AA	GG	GG
group7	4772	6.98	--	CC	CT	TT	TT	CC	CT	TT	CC	CC
	16384	7.17	TT	TT	AT	AT	AA	--	AT	AA	TT	TT
	4649	7.35	AA	AA	AC	CC	CC	AA	AC	CC	AA	AA
	11288	7.71	CC	CC	--	CG	CG	CC	CG	CG	CC	CC
	23637	8.41	AA	AA	AA	AG	AG	AA	AG	AG	AA	AA
	31972	8.42	TT	TT	TT	CC	CC	TT	CT	CC	TT	TT
	27916	8.60	TT	--	TT	CC	CC	TT	CT	--	TT	TT
	8663	8.95	GG	GG	GG	AA	AA	GG	AG	AA	GG	GG
	34075	9.04	CC	CC	CC	--	CT	CC	CC	CT	CC	CC
	27063	10.20	--	GG	GG	AA	AA	GG	AG	AA	GG	GG
	17406	11.14	--	AA	AA	GG	--	AA	AG	GG	AA	AA
	30983	12.16	TT	TT	TT	GG	--	TT	GG	GG	TT	TT

	18004	13.27	CC	CC	CC	TT	TT	CC	TT	TT	CC	CC
	2995	14.06	CC	CC	CC	GG	GG	CC	GG	GG	CC	CC
	18517	14.41	GG	GG	GG	TT	TT	GG	TT	TT	GG	GG
	26116	14.89	GG	GG	GG	AA	AA	GG	AA	AA	GG	GG
	29827	15.13	TT	TT	--	CC	CC	TT	CC	CC	TT	TT
	19949	15.48	--	TT	TT	GT	GT	TT	GT	GT	TT	TT
	4337	15.49	--	CC	CC	GG	GG	CC	GG	GG	CC	CC
	29474	15.85	--	GG	GG	AA	--	GG	AA	AA	GG	GG
	7750	16.59	GG	GG	GG	AG	AG	GG	AG	AG	GG	GG
	11392	17.34	CC	CC	CC	AA	AA	CC	AA	AA	CC	CC
	7054	17.54	CC	CC	CC	CG	CG	CC	CG	CG	CC	CC
	11004	18.70	AA	AA	AA	GG	AG	AA	GG	GG	AA	AA
	18816	18.87	--	CC	--	AA	--	--	AA	AA	CC	CC
	8975	19.23	GG	GG	GG	TT	TT	GG	TT	TT	GG	GG
	21266	19.95	AA	AA	--	CC	CC	--	CC	CC	AA	AA
	32811	20.67	CC	CC	CC	CT	CT	CC	CT	CT	CC	CC
	3850	21.03	GG	GG	GG	TT	TT	GG	TT	TT	GG	GG
	27767	21.93	GG	GG	GG	AG	AG	GG	AG	AG	GG	GG
	22278	22.13	TT	TT	TT	CC	CC	TT	CC	CC	TT	TT
	33479	23.51	CC	CC	CC	--	CT	CC	CT	CT	CC	CC
	22015	23.69	TT	TT	TT	AA	AA	TT	AA	AA	TT	TT
	16000	23.87	GG	--	GG	CC	CC	GG	CC	CC	GG	GG
	6651	28.03	--	TT	TT	GG	GG	TT	GG	GG	--	TT
	12798	33.12	--	AG	--	AG	AG	GG	AG	AG	GG	GG
	34200	37.74	GT	GT	GT	GT	TT	GT	TT	TT	GT	GT
	10424	40.31	TT	CT	TT	CT	CC	TT	CC	CC	TT	TT
	8107	44.80	AA	AC	AA	AC	CC	AA	CC	CC	AA	AA
	30374	47.95	CT	CC	--	--	--	CT	CC	CC	CT	CT
	28152	52.04	CC	GG	CG	CG	GG	CC	GG	GG	CC	CC
	14512	58.02	CT	CC	CT	CT	CC	TT	CC	CC	TT	TT
	27643	63.71	TT	GT	GT	GT	GT	--	GT	GT	TT	TT
	37370	68.85	CT	CT	TT	TT	--	TT	CT	CT	TT	CT
	18230	73.33	TT	AT	AT	AT	AT	TT	AT	TT	TT	TT
	6578	77.67	AC	CC	CC	CC	CC	AC	CC	AC	AC	AC
	19492	82.34	CT	TT	CT	TT	CT	CT	--	CT	CC	CT
	18354	87.28	AG	AA	AA	AA	AA	AG	AA	AG	AG	AG
	33458	92.09	AG	GG	GG	GG	GG	AG	GG	AG	AG	AG
	20457	0.79	--	CG	GG	CG	GG	CG	GG	GG	GG	GG
	20767	5.28	--	TT	CT	CT	CT	--	CC	CT	CT	CC
	14699	17.33	TT	TT	CT	CT	CC	TT	CC	CT	CT	CC
	24175	28.92	TT	TT	CT	CT	CT	TT	CC	CT	CT	CC
group8	17182	34.62	CC	--	CT	CT	TT	CC	TT	CT	CT	TT
	10996	38.68	TT	GT	GT	TT	GG	TT	GG	GT	GT	GG
	26129	42.55	--	CT	CT	CC	TT	CC	--	CT	CT	TT
	21888	47.31	AT	AT	AT	AA	TT	AA	--	AT	AT	TT
	25481	52.16	AG	AG	AG	--	GG	AA	GG	AG	AG	GG
	21900	57.38	AC	--	AC	AC	AA	CC	AC	AC	AC	AA

	17990	62.18	AG	AG	AG	AA	GG	AA	AG	AG	AG	GG
	33404	63.84	AG	AG	--	AA	GG	AA	AG	AG	AG	GG
	18933	64.20	CT	CT	CT	TT	CC	TT	CT	CT	CT	CC
	17892	64.37	AG	AG	--	GG	AA	GG	AG	AG	AG	AA
	21896	65.09	--	GT	GT	GG	TT	GG	GT	GT	GT	TT
	12832	65.63	CT	CT	CT	TT	CC	TT	CT	CT	CT	CC
	38938	65.81	AG	AG	AG	GG	AA	GG	AG	AG	--	AA
	30990	65.99	AG	AG	AG	GG	AA	GG	AG	AG	AG	AA
	34203	66.22	AG	AG	--	--	--	AA	AG	AG	AG	GG
	19387	66.70	CG	CG	CG	GG	CC	GG	CG	CG	CG	CC
	18913	66.88	--	AG	AG	AA	GG	AA	AG	AG	AG	GG
	29848	67.06	AC	AC	AC	AA	CC	AA	AC	AC	AC	CC
	14315	68.41	AG	AG	--	GG	AA	GG	AA	AG	AG	AA
	17220	70.04	AG	AG	AG	GG	AA	GG	AA	AG	AG	AA
	11658	71.08	AT	AT	AT	AA	TT	AA	TT	AT	AT	TT
	6687	71.44	CT	CT	CT	TT	CC	CT	CC	CT	CT	CC
	30264	72.67	CT	CT	CT	--	CC	CT	CC	TT	--	CC
	15355	72.81	CG	CG	CG	CC	GG	CG	GG	CC	CG	GG
	6389	73.16	CT	CT	CT	CC	TT	CT	TT	CC	CT	TT
	21907	73.34	CT	CT	CT	TT	CC	CT	CC	TT	CT	CC
	14576	73.52	CT	CT	CT	CC	TT	CT	TT	CC	CT	TT
	3739	74.07	AT	AT	AT	AA	TT	AT	TT	AA	AT	TT
	31274	75.53	AT	AT	AT	TT	AA	AT	AA	TT	AT	AA
	32802	76.07	CT	CT	CT	CC	TT	CT	TT	CC	CT	TT
	7508	76.24	CT	CT	CT	TT	CC	CT	--	TT	CT	CC
	12158	77.70	AT	AT	AT	TT	AA	AT	AA	TT	AT	AA
	18727	77.88	AG	AG	AG	AA	GG	AG	GG	AA	AG	GG
	15273	78.05	GT	GT	GT	TT	GG	GT	GG	TT	GT	GG
	37776	78.29	--	CT	CT	CC	CT	CT	TT	CC	CT	TT
	8763	78.77	CG	CG	CG	GG	CC	CG	CC	GG	CG	CC
	15090	79.15	AG	AG	AG	AA	GG	AG	GG	AA	AG	GG
	30057	80.55	CT	CT	CT	CC	TT	CT	TT	CC	CT	TT
	17038	81.94	CT	--	--	CC	TT	CT	TT	CC	CT	CT
	12913	82.84	AT	AT	AT	AA	TT	AT	TT	AA	AT	AT
	20913	83.38	AG	AG	AG	GG	AA	AG	AA	GG	AG	AG
	5189	88.28	AG	AG	GG	GG	AA	AG	AA	GG	AG	AG
	11484	92.59	--	GT	GG	GG	TT	GT	TT	GG	GT	GT
	28941	97.46	AG	GG	GG	--	AA	AA	AA	GG	AA	GG
	20000	100.13	AG	AG	AG	AG	GG	GG	GG	AG	GG	AG
	13117	0.07	TT	TT	CT	CT	TT	TT	TT	TT	CT	CT
	6390	5.83	AG	AG	--	AG	GG	GG	GG	AG	AG	AG
	14289	10.95	AG	AG	GG	GG	GG	AG	GG	AG	GG	GG
	15889	16.21	AG	AG	AG	AG	GG	AG	GG	AG	AG	AG
	6143	21.11	CG	CG	CG	CG	CC	CG	CC	CG	CG	CG
group9	8734	26.24	CT	CT	CT	CT	--	CT	TT	CT	--	CT
	15083	33.60	AC	AC	CC	CC	AC	AC	AC	AC	AC	CC
	17882	38.49	--	AA	AA	AG	AG	AA	AG	AA	AG	AA

	12987	43.14	AC	AC	CC	CC	AC	AC	AC	AC	AC	CC
	17762	48.09	AC	AC	AC	CC	CC	AC	--	AC	CC	AC
	1340	54.25	CT	CT	TT	TT	CT	--	CT	CT	CT	TT
	11895	59.14	AG	AG	AA	AG	GG	AG	GG	AG	--	AA
	9357	64.67	AG	AG	GG	GG	AA	AG	AA	AG	AA	GG
	13407	69.15	CT	CT	TT	TT	CC	CT	CC	CT	CC	TT
	27246	74.16	CT	CT	CT	CT	TT	CT	TT	CT	TT	CT
	33128	78.15	CG	CG	CC	CC	CG	CG	GG	GG	GG	CC
	12598	83.09	AG	AG	AA	AA	AG	AG	GG	GG	GG	AA
	11126	89.74	CC	CT	--	TT	TT	CT	CC	CC	--	TT
	1361	94.81	AT	AT	AT	AT	AT	AT	AA	AA	AA	AA
	31725	99.85	CC	AC	CC	CC	CC	AC	AC	AA	AA	AC
	38472	104.12	--	AG	--	--	GG	--	AG	--	AG	GG
	11261	109.00	CC	CT	CC	CC	CC	CT	CT	CT	CT	CC
	12571	114.14	CC	CT	CC	--	CC	CT	CT	CT	CT	CC
	37895	121.02	--	--	--	--	--	GT	GT	GT	GT	GG
	8020	0.19	CT	CC	CT	CC	TT	CC	CC	CT	CT	TT
	28975	6.88	AG	AG	GG	AG	GG	AG	AG	GG	GG	GG
	27447	18.31	AG	AA	AG	AA	GG	AA	AA	AG	AG	GG
	32517	24.99	GG	GG	GG	GG	CG	GG	GG	CG	GG	CG
	4341	29.23	AA	AA	AG	AA	GG	AA	AA	AG	AG	GG
group10	10280	33.00	GG	GG	AG	GG	AG	GG	GG	AG	AG	AA
	15348	38.72	GT	GT	GT	GT	GT	GT	GT	TT	GT	TT
	8908	43.04	GG	GG	AG	GG	AG	GG	GG	AG	AG	AA
	32629	46.61	GG	GG	AG	GG	AG	GG	GG	AG	AG	AA
	12609	51.75	GT	GT	GT	GT	GT	GT	GT	GT	GT	GG
	36887	56.12	--	TT	CT	CT	CC	TT	TT	TT	CT	CC
	22253	62.30	--	CC	CT	TT	TT	CC	CC	CC	CT	TT
	11505	67.35	CC	CT	CC	CC	CC	CT	CT	CT	CT	CC
	8757	75.02	AA	GG	AG	AA	AA	GG	AG	GG	GG	AA
	1970	79.06	CC	TT	TT	CC	CC	TT	CT	TT	TT	CC
	5322	84.51	--	GG	GG	AA	AA	GG	AG	GG	GG	AA
	10332	91.34	--	TT	TT	CC	CC	TT	CC	TT	TT	CT
	29177	96.75	GG	CC	CC	GG	GG	CG	GG	CC	--	CG
	12354	102.87	CC	TT	TT	CC	CC	CT	--	CT	TT	CT
	24482	0.00	--	AC	CC	--	--	AC	AC	AA	AC	AC
	35387	4.91	--	AG	AA	GG	AG	AG	AG	GG	AG	AG
	11299	8.90	TT	CT	CC	TT	CT	CT	CT	TT	CT	CT
	23713	13.72	AA	AG	AA	AA	AG	AG	AG	AG	AG	AG
	24183	19.19	AA	AG	AA	--	AG	AG	AG	AG	AG	AG
group11	28232	23.43	TT	TT	TT	TT	CT	CT	CT	TT	TT	CT
	27046	28.46	GG	AG	--	GG	AG	AG	AG	AG	AG	AA
	33692	33.34	AA	AG	--	--	AA	AA	AA	AG	AG	AG
	2211	38.03	TT	GT	TT	TT	TT	TT	TT	GT	GT	GT
	21829	41.04	TT	GT	TT	TT	TT	TT	TT	GT	GT	GT
	11528	53.51	--	AC	AA	--	AC	AC	AC	AC	AC	CC
	29065	58.57	--	AG	AA	AA	AG	AG	AG	AG	AG	GG

	19246	61.11	--	CG	--	CC	CG	CG	CG	CG	CG	GG
	25855	71.11	CC	CG	CC	CC	CG	CG	CG	CG	CG	GG
	7306	76.06	TT	CT	TT	TT	CT	CT	CT	CT	CT	CC
	7675	82.14	--	CT	CC	CT	CT	CT	CT	CT	CT	TT
	10906	87.37	AA	AT	AA	AT	AT	--	AT	AT	--	TT
	6515	91.15	AG	GG	AG	GG	AG	AG	GG	GG	GG	GG
	16630	96.72	CT	CT	CC	CT	--	CT	TT	CT	CT	TT
	27750	101.98	CT	CT	TT	CT	CC	TT	CC	CT	CT	CC
	21503	107.66	--	CT	CC	CT	TT	CC	TT	CC	CT	CT
	7257	112.27	AG	AG	GG	AG	AA	GG	AA	AG	AG	AG
	14124	117.34	--	CC	CC	CC	CC	CC	CG	CG	CC	CG
	18740	121.49	--	CG	GG	CG	CG	CG	CG	GG	CG	GG
	32234	125.79	GT	GG	GG	GG	--	GG	GG	GT	GG	GT
	11737	7.06	CG	CC	CG	CC	CG	CG	CC	CG	CG	CC
	36137	12.46	AA	GG	AG	GG	AG	AG	AG	AA	AA	AG
	8188	17.60	AC	AC	AC	AC	AC	AC	AC	AC	AC	AA
	7759	31.31	AT	AA	AT	AA	AT	AT	AA	AT	AT	AA
	13213	35.44	AA	GG	AA	GG	AG	AG	GG	AG	AA	AG
group12	25094	43.77	--	AT	TT	AA	AT	AT	AA	AT	TT	AA
	7115	48.88	AA	AG	AA	GG	AG	AG	GG	AG	AA	GG
	9129	53.22	AC	AC	AC	CC	CC	CC	CC	CC	AC	CC
	12006	58.18	TT	CT	TT	CC	TT	CT	CC	CT	TT	CC
	10047	63.92	GT	GT	GG	TT	GG	GT	TT	GT	GG	TT
	4764	68.19	GT	GT	GG	TT	GG	--	TT	GG	GG	TT
	20579	69.39	CT	CT	TT	CC	TT	CT	CC	CT	TT	CC
	23978	69.75	CG	CG	GG	CC	GG	CG	CC	CG	GG	CC
	33041	70.20	--	AG	AA	GG	--	AG	GG	AG	--	GG
	21667	70.58	CT	CT	TT	CC	TT	CT	CC	CT	TT	CC
	11881	70.76	GT	GT	GG	TT	GG	GT	TT	GT	GG	TT
	8354	71.12	CT	CT	CC	TT	CC	CT	TT	CT	CC	TT
	14411	72.11	CG	CG	CC	GG	CC	CG	GG	CG	CC	GG
	15356	72.74	AG	AG	AA	--	AA	AG	GG	AG	AA	GG
	33698	74.43	AC	AC	CC	--	--	AC	--	AC	CC	AA
	27623	74.68	CT	CT	CC	TT	--	CT	TT	--	CC	TT
	22444	75.69	GG	GG	AG	GG	AG	AG	GG	AG	AG	GG
	15027	76.37	--	--	CC	AA	CC	AC	AA	AC	CC	AA
	22134	76.76	CT	CT	TT	CC	TT	CT	CC	CT	TT	CC
	5369	77.84	AG	AG	AA	GG	AA	AG	GG	AG	AA	GG
	25783	78.51	CG	CG	CC	GG	CC	CG	GG	CG	CC	GG
	36537	78.73	GT	GT	TT	GG	TT	--	GG	GT	TT	GG
	15397	78.91	CT	CT	TT	CC	TT	CT	CC	CT	TT	CC
	3253	79.26	TT	TT	CT	TT	CT	CT	TT	CT	CT	TT
	23087	84.06	--	GG	GG	AG	GG	AG	AG	AG	GG	AG
	9239	89.81	CC	CC	GG	CC	GG	GG	CC	CG	GG	CC
	9376	93.61	AA	AA	AT	AA	AT	AT	AA	AA	AT	AA
	4444	98.59	AA	AA	AG	AA	AG	AG	AA	AG	AG	AA
	3195	101.70	CC	CC	AC	CC	AC	AC	CC	AC	AC	CC

	4347	116.66	TT	TT	CT	TT	CT	CT	TT	CT	CT	TT
	19408	121.32	TT	TT	CT	TT	CT	CT	CT	TT	CT	TT
	31498	126.13	--	GG	AT	AG	AT	AT	AG	GT	AT	GG
	3272	131.35	AA	AA	AT	AA	AT	AT	AA	AT	AT	AA
	4682	136.26	GG	GG	AA	AG	AG	AA	AG	AA	AA	GG
	36671	141.17	--	CC	AA	AC	AC	AA	AC	AA	AA	CC
	3058	146.15	AA	AA	AG	AA	AG	AG	AA	AG	AG	AA
	22370	150.36	--	GG	AA	GG	AG	AA	AG	AA	AA	GG
	29679	159.33	AA	AA	GG	AA	AG	GG	AG	GG	GG	AA
	26253	164.40	--	GG	--	GG	AG	AA	AG	AA	AA	GG
	23756	169.50	GG	GG	GT	GG	GT	TT	GT	TT	TT	GG
	24479	173.75	GG	GG	--	GG	GG	AG	AG	AG	AG	AG
	18781	178.70	--	CC	CT	CC	CT	TT	CT	TT	CT	CT
	1597	182.83	CC	CC	CG	CG	CG	GG	CG	GG	CG	CG
	6254	187.03	TT	TT	CT	TT	CT	CT	TT	CT	CT	TT
	3348	190.17	TT	TT	TT	CT	TT	CT	CT	CT	TT	CT
	180	4.59	CC	CC	CC	CC	CT	CT	CT	CC	CC	CT
	7430	9.59	GT	GT	GG	GT	GG	GG	GG	--	GT	GG
	14662	14.06	AG	AG	GG	GG	AG	AG	AA	GG	AG	AG
	34033	18.33	--	AG	AA	AA	AG	GG	GG	AA	AG	AG
	35828	35.44	AA	--	AA	AA	AG	AG	AG	AA	AA	AG
	31847	41.15	--	TT	GT	GT	--	GT	GT	GG	GT	GT
group13	30842	46.28	AA	AG	AA	AA	AG	AG	AG	AA	AA	AG
	4816	51.98	CC	CT	CC	CC	CT	CT	CT	CC	CC	CT
	8435	56.29	AG	AA	AG	AG	AA	AA	AA	AG	AG	AA
	20161	61.12	AG	AA	AG	AG	AG	AG	AG	GG	AG	AG
	12489	66.44	--	AA	AG	GG	AG	AG	AG	GG	GG	AG
	16009	71.60	CG	CC	CG	GG	CG	CG	CG	GG	GG	CG
	17658	76.25	CG	CC	--	GG	CG	CG	CG	GG	GG	CG
	8629	81.25	GG	AG	AG	GG	AG	AG	AG	GG	GG	AG
	16674	85.87	--	GG	AG	GG	GG	GG	AG	GG	GG	GG
	36089	90.63	--	CT	CT	TT	CT	CT	CC	TT	TT	CT
	26865	96.03	CC	CC	AC	CC	AC	CC	AC	CC	CC	CC
	34122	101.47	CG	CC	CG	CC	CG	CC	CG	CC	CC	CC
	11911	106.09	AC	CC	AC	CC	AC	CC	AC	CC	CC	CC
	33294	113.97	AC	CC	AC	AA	AC	AC	CC	AA	AA	AC
	29693	119.37	AA	AG	AA	AA	AG	AG	AG	AA	AA	AG
	6409	128.88	AG	GG	AG	AA	AG	AG	AG	AA	AA	AG
	18731	132.71	GG	AG	GG	GG	GG	AG	AG	GG	GG	AG
	16236	138.47	CT	TT	CT	CC	CT	CT	CT	CC	CC	CT
	29018	143.14	CT	TT	CT	CC	CT	CT	CT	CC	CC	CT
	27948	146.19	AG	AG	AG	AA	AG	AA	AG	AA	AA	AA
	39153	154.31	CT	CT	CT	CC	CT	CT	CC	CC	CC	CC
	13687	7.39	AG	AG	AG	--	AG	AA	AG	AG	GG	GG
	22059	12.65	--	AC	AC	AC	AC	AC	CC	AC	CC	CC
	9272	17.49	CT	CC	CC	CT	CC	CT	CT	CC	CC	CC
	34276	22.88	AG	AG	AG	GG	AG	GG	AG	AG	AA	--

	3070	27.14	AG	AA	AA	AG	AA	AG	AG	AA	AA	AA
	20828	33.44	AG	AG	AG	GG	AA	GG	GG	AG	AA	AA
group14	3303	38.24	AC	CC	AC	AA	CC	AA	AA	AC	CC	CC
	9776	42.32	AG	GG	AG	AA	GG	AA	AA	AG	GG	GG
	16679	47.28	GT	TT	GT	GG	GT	GG	GG	GT	TT	TT
	14957	53.37	AA	AA	AG	AG	AA	AG	AG	AG	AA	AA
	21860	58.85	CT	TT	CT	CC	CT	CC	CC	CT	TT	TT
	9926	63.04	--	CC	CT	CT	CC	CT	CT	CT	CC	CC
	22985	68.26	AG	AG	AG	AA	AG	AA	AA	AG	GG	GG
	22854	73.79	CG	GG	GG	CG	CG	CG	CG	CG	CG	GG
	34319	78.10	--	TT	TT	CT	CT	CT	CT	CT	CT	TT
	35771	84.61	AG	AG	AG	GG	AG	GG	GG	GG	AG	AG
	11049	89.77	AA	AG	AG	GG	AG	GG	GG	GG	AG	AG
	18193	94.28	CC	CG	CG	CG	CG	GG	GG	GG	--	CG
	9508	97.71	--	GG	AG	AG	AG	GG	GG	GG	AG	AG
	16466	3.90	AG	AG	--	GG	GG	AA	AG	--	AG	AA
	3288	7.09	CT	CT	TT	TT	TT	CC	CT	CT	CT	CC
	34576	7.27	--	AG	GG	GG	GG	AA	--	AG	AG	AA
	17923	7.67	CG	CG	CC	CC	CC	GG	CG	CG	CG	GG
	19068	7.86	AC	AC	CC	CC	CC	AA	AC	AC	AC	AA
	25600	9.07	AG	AG	AA	AA	AA	GG	AG	AG	AG	GG
group15	13463	9.34	GT	GT	GG	GG	GG	TT	GT	GT	GT	TT
	31789	9.61	AG	AG	GG	GG	GG	AA	AG	AG	AG	AA
	10501	9.67	--	AG	GG	GG	GG	AA	AG	AG	AG	AA
	14892	9.73	AG	AG	AA	AA	AA	GG	AG	AG	AG	GG
	8492	9.79	AC	AC	AA	AA	AA	CC	AC	AC	AC	CC
	5144	9.96	AG	AG	AA	AA	AA	GG	AG	AG	AG	GG
	5762	10.14	AG	AG	AA	AA	AA	GG	AG	AG	AG	GG
	18011	10.50	CT	CT	TT	TT	TT	CC	CT	CT	CT	CC
	12890	10.68	CG	CG	GG	GG	GG	CC	CG	CG	CG	CC
	31219	10.86	AC	--	AA	AA	AA	CC	AC	AC	AC	CC
	18522	11.39	CC	CT	TT	TT	TT	CC	CT	CT	CT	CC
	17114	11.57	GT	GT	TT	TT	TT	GG	GT	GT	GT	GG
	32211	11.93	CT	CT	CC	CC	CC	TT	CT	CT	CT	TT
	13320	12.11	CG	CG	CC	CC	CC	GG	CG	CG	CG	GG
	20163	12.29	AT	AT	TT	TT	TT	AA	AT	AT	AT	AA
	25438	13.00	CT	CT	CC	--	CC	TT	CT	CT	CT	TT
	32105	13.18	AG	AG	GG	GG	GG	--	AG	AG	AG	AA
	5067	13.72	AG	AG	AA	AA	AA	GG	AG	AG	AG	GG
	26750	13.89	--	AT	AA	AA	AA	TT	AT	AT	AT	TT
	8013	14.07	AG	AG	--	GG	GG	AA	AG	AG	AG	AA
	22456	14.34	AG	AG	AA	--	AA	GG	--	AG	--	GG
	23562	14.61	--	CG	CC	CC	CC	GG	CC	CG	CG	GG
	5281	14.78	AG	AG	AA	AA	AA	GG	AA	AG	AG	GG
	20715	15.19	CG	--	GG	GG	GG	CC	GG	CG	CG	CC
	35221	15.81	AG	AG	GG	--	AG	AA	GG	AG	AG	AA
	17201	15.91	AC	AC	AA	AA	AC	CC	AA	AC	AC	CC

36619	16.01	AG	AG	GG	GG	AG	AA	GG	AG	AG	AA
30042	16.21	CG	CG	CC	CC	CG	GG	CC	CG	CG	GG
29948	16.39	CT	CT	CC	CC	CT	TT	CC	CT	CT	TT
18110	16.73	AT	AT	TT	TT	AT	AA	TT	AT	AT	AA
6840	16.91	CT	CT	CC	CC	CT	TT	CC	CT	CT	TT
3900	17.63	AT	AT	--	AA	AT	TT	AA	AT	AT	TT
13507	18.21	AT	AT	TT	TT	AT	AA	TT	AT	AT	--
2613	18.40	CG	CG	GG	GG	CG	CC	GG	CG	CG	CC
3243	18.58	CT	CT	CC	CC	CT	TT	CC	CT	CT	TT
6891	18.77	AG	AG	AA	--	AG	GG	AA	AG	AG	GG
3561	18.95	--	AC	AA	AA	AC	CC	AA	AC	AC	CC
3712	19.65	GT	GT	TT	TT	GT	GG	TT	GT	GT	GG
10238	19.88	CT	CT	CC	CC	CT	TT	CC	CT	CT	TT
3387	20.44	CC	CG	GG	GG	CG	CC	GG	CG	CC	CC
36507	20.62	GG	GT	TT	--	GT	GG	TT	GT	GG	GG
4525	21.36	CC	CT	TT	TT	CT	CC	TT	CT	CC	CC
18928	21.80	--	AG	AA	--	AG	GG	AA	AG	GG	GG
14141	22.47	--	AC	AA	AA	AC	CC	--	AC	CC	CC
12340	22.66	TT	CT	--	CC	CT	TT	CC	CT	TT	TT
10763	23.37	CC	AC	AA	AA	AC	CC	AA	AC	CC	CC
11802	24.09	GG	AG	AA	AA	AG	GG	AA	AG	GG	GG
17852	24.26	TT	GT	GG	--	GT	TT	GG	GG	TT	TT
25029	24.98	TT	GT	--	GG	--	TT	GG	--	--	TT
3744	25.34	AA	AG	GG	--	AG	AA	GG	GG	AA	AA
6878	25.52	TT	CT	CC	CC	CT	TT	CC	CC	TT	TT
25914	25.96	TT	CT	CC	CC	CT	TT	--	CC	TT	TT
22116	26.23	GG	AG	AA	AA	AG	GG	AA	AA	GG	GG
13236	26.41	TT	CT	CC	CC	CT	TT	CC	CC	TT	TT
6421	26.58	CC	CT	TT	TT	CT	CC	TT	TT	CC	CC
5083	26.76	--	AG	GG	GG	AG	AA	GG	GG	--	AA
15848	27.30	--	CT	CC	CC	CT	CT	CC	CC	TT	TT
8480	32.12	CT	CT	TT	TT	CT	CT	TT	TT	CT	CT
27696	36.58	--	CT	CC	CC	CT	CT	CC	CC	TT	CT
5029	42.30	GG	AG	AA	AA	AG	AG	AG	AA	GG	AG
21010	48.62	CC	AC	--	AC	CC	AC	AC	AA	CC	AC
28360	52.86	CC	CG	CG	CC	CC	CC	CG	CG	CC	CG
20052	57.01	--	TT	GG	GT	TT	--	GT	GG	TT	GT
26264	62.91	AA	AA	GG	AG	AA	AG	AG	AG	AG	AG
19379	67.06	CT	CC	CT	CT	CC	CT	CC	CT	CT	CT
30482	69.38	GG	GG	GG	GG	GG	GG	GG	GG	GG	AG
24141	74.18	--	AA	AA	AG	AG	AG	--	--	--	AG
13839	77.62	--	CC	CC	CC	CG	CG	CC	CG	--	CG
25551	82.08	--	TT	TT	TT	TT	TT	TT	TT	CT	CT
20027	86.55	GG	GG	GG	GT	GT	TT	GG	GT	TT	GT
24517	0.52	--	GG	AG	AG	AG	GG	AG	AG	--	AG
18223	5.38	--	AG	AG	AG	GG	AG	GG	AG	GG	GG
30774	10.44	--	AC	CC	--	--	AC	AC	CC	AA	AC

	31715	17.13	--	AT	AA	AA	AT	AT	AT	AA	TT	TT
	27035	21.41	AT	AT	AA	AA	AT	AT	AT	AA	TT	TT
	23761	25.18	AT	AT	TT	TT	AT	AT	AT	TT	AA	AA
	17004	29.03	CT	CT	CC	CC	CT	CT	CT	CC	TT	TT
	2546	34.85	AG	AG	AA	AA	AG	AA	AG	AA	GG	GG
group16	11373	39.92	AC	AC	AC	CC	AC	CC	AC	CC	AA	AA
	14463	44.13	--	CT	CT	CC	CT	CC	CT	CC	TT	TT
	18955	50.62	AG	AG	AG	AG	AG	--	GG	AG	GG	GG
	5484	55.12	GT	GT	GT	GT	GT	TT	GG	GT	GG	GT
	24470	60.29	AG	AG	AG	AG	AA	AA	AG	AG	GG	AG
	9032	64.56	CC	CC	CG	CG	CG	CG	CC	CG	CC	CC
	6522	68.85	GG	GG	AG	AG	AG	AG	GG	AG	GG	GG
	5277	73.34	CG	CG	CG	CG	GG	GG	CG	CG	CC	CG
	28798	77.90	AG	AG	AG	GG	GG	GG	AG	AG	AA	AG
	32874	80.25	--	AG	AG	AA	AA	AA	AG	AG	GG	GG
	3574	83.10	AG	AG	AG	GG	GG	GG	AG	GG	AA	AA
	19217	87.24	GG	GG	GT	GT	GT	GT	GG	GT	GG	GG
	11525	92.50	AG	AG	AG	AA	AA	AA	AG	AA	GG	GG
	28903	102.24	TT	GT	GT	GT	GT	GT	TT	GT	TT	TT
	28160	104.21	CG	CG	--	CC	CC	CG	GG	CC	CG	GG
	23746	0.00	TT	CT	CT	TT	TT	TT	TT	TT	TT	CT
	13860	5.12	GG	CC	CC	CG	GG	GG	CG	GG	GG	CG
	28355	11.02	CC	CG	CG	CC	CC	CC	CG	CC	CC	CC
	2633	16.68	AA	AA	AC	AA	AA	AA	AA	AA	AA	AC
	13668	21.33	--	--	CT	CC	CC	CC	CC	CC	CC	CT
	23841	26.93	--	AA	AG	AA	AA	AA	AA	AA	AA	AG
	21707	31.00	CT	CC	--	CC	CC	CC	CC	CC	CC	CT
	15864	36.07	GG	CG	CG	GG	GG	GG	CG	GG	GG	GG
group17	17977	41.32	CT	CC	CT	CC	CC	CC	CC	CC	CC	CT
	6207	46.35	AG	AG	AG	AA	AA	AG	AG	AA	AA	AA
	10417	50.85	GT	GT	GT	GG	GG	GG	GG	GG	GG	GG
	20433	52.23	GT	GG	GT	TT	--	GT	GT	TT	TT	TT
	37506	52.96	--	CG	CG	GG	--	GG	GG	GG	GG	GG
	20317	53.20	CG	CC	CG	GG	CG	CG	CG	GG	GG	GG
	12728	53.76	AG	AG	AG	GG	GG	GG	GG	GG	GG	GG
	31691	53.91	CT	CC	CT	TT	CT	CT	CT	TT	--	TT
	25921	54.09	GT	TT	GT	GG	GG	GT	GT	GG	GG	GG
	6524	54.62	AG	AG	AG	GG	GG	GG	GG	GG	GG	GG
	19809	55.01	AG	GG	AG	AA	AG	AG	--	AA	AA	AA
	22317	56.64	CT	CC	CT	TT	CT	CT	CT	TT	TT	TT
	34323	56.97	AT	AT	AT	TT	--	TT	TT	TT	TT	TT
	22949	57.15	GT	GT	GT	TT	TT	TT	TT	TT	TT	TT
	19187	57.51	CT	TT	CT	CC	CT	CT	CT	CC	CC	CC
	6735	58.59	AG	AG	AA	AA	AA	AA	AA	AA	AA	AA
	23867	58.76	AT	AA	TT	TT	AT	AT	AT	TT	TT	TT
	13493	59.03	AG	AG	AG	AA	AA	AA	AA	AA	AA	AA
	4962	59.29	CT	CT	TT	TT	TT	TT	TT	TT	TT	TT

	12350	59.56	AT	AT	TT	TT	TT	TT	TT	TT	TT	TT
	14926	60.18	GT	TT	GG	GG	--	GT	GT	GG	GG	GG
	22910	65.74	AG	GG	AA	AA	AG	AG	AG	AA	AA	AA
	34632	70.72	CC	TT	--	CC	CT	--	CT	CC	CC	CC
	19251	74.65	CC	TT	CC	CT	CT	CT	CT	CC	CC	CC
	19895	86.10	CC	TT	CC	CT	CT	CT	CT	CC	CC	CC
	5899	91.57	GG	TT	GT	GT	GT	GT	GT	GG	GG	GT
	23558	96.01	CC	GG	CG	CG	CG	CG	CG	CC	--	CG
	16275	102.72	CC	AA	AC	AC	AC	AC	AC	CC	CC	AA
	11166	107.50	AA	GG	AG	AG	AG	AG	AG	AA	AA	GG
	22360	114.29	GG	AA	GG	AG	AG	AG	AG	GG	GG	AA
	14521	7.10	--	CT	TT	CT	CT	CC	CT	CC	CT	CC
	32379	11.98	AA	AC	AA	AC	AC	CC	AA	CC	AC	CC
	17648	14.14	--	GT	TT	GT	--	GT	TT	GT	TT	GT
	12382	19.60	--	GG	--	CG	CG	--	GG	CG	GG	CG
	22544	26.18	GG	AG	AG	AA	GG	AA	GG	AA	GG	AG
	15122	34.25	TT	TT	TT	CT	TT	CT	TT	CT	TT	CT
	32843	39.67	--	GT	GT	GT	--	GT	TT	TT	TT	TT
	13734	44.27	CC	CC	CC	CT	CC	CT	CC	CT	CC	CT
group18	19020	49.71	AA	AA	AC	AC	AA	AA	AA	AA	AA	AA
	4231	53.10	AA	AG	AG	GG	AA	AA	AA	AG	AA	AG
	27205	58.78	AG	AA	AG	AG	AA	AA	AA	AA	AA	AA
	24808	63.40	GT	GT	GT	GG	TT	TT	GT	GT	TT	GT
	21341	68.05	CT	CT	CT	TT	CC	CC	CT	CT	CC	CT
	5784	74.63	AC	AC	AC	--	AA	AA	AC	AC	AA	AC
	27683	80.22	AT	AT	AT	AA	TT	TT	AA	AT	TT	AT
	25593	86.28	GG	GT	GG	GG	GT	GT	GT	GT	GT	--
	13061	90.84	AG	AA	AG	AA	AG	AG	AA	AA	AG	AA
	13008	96.65	CG	CG	CG	GG	--	CC	CG	--	CC	CG
	16941	98.40	AG	AG	AG	--	AA	AG	AG	AG	AA	AG
	29440	0.00	CG	CC	CG	CC	--	CG	GG	CG	CG	CG
	24891	5.15	AG	AA	AG	AA	AG	AG	GG	AG	AG	AG
	36938	10.04	AG	AA	--	--	AA	--	AG	AA	AA	AG
	18747	14.90	AA	AA	AT	AA	AA	AA	AT	AT	AT	AT
	7042	18.66	--	AA	AA	AA	AA	AG	AG	AA	AA	AG
	14044	23.16	AA	AG	--	AG	AG	AA	AA	AG	AG	AA
	8105	28.19	AC	--	AA	AC	AA	AC	AC	AC	AA	CC
	31525	36.32	--	CC	CC	AC	CC	CC	CC	AC	CC	AC
group19	17180	41.34	AA	CC	CC	AC	CC	AC	AC	AC	--	AA
	24710	46.25	--	GT	GG	GT	GG	GT	GT	GT	GG	TT
	27888	51.35	--	AT	--	AT	AT	AT	AT	AT	AT	TT
	6677	64.12	AG	AA	AA	AG	AG	AA	AA	AG	AA	AG
	6732	71.98	AC	CC	CC	AC	AC	CC	CC	--	CC	AC
	16738	76.91	TT	AT	AA	AT	AT	AT	AT	AT	AT	TT
	8801	86.64	TT	CT	CC	CT	CC	CT	TT	CT	CT	TT
	8214	92.39	AA	AG	GG	AG	GG	AG	AA	AG	AG	AA
	14083	93.33	TT	CT	CC	CT	CC	CT	TT	CT	--	TT

	18623	93.89	CC	CT	TT	CT	--	CT	CC	CT	CT	CC
	33154	95.64	AA	AG	GG	AG	GG	AG	AA	AG	AG	AA
	3156	96.20	TT	CT	CC	CT	CC	CT	TT	CT	CT	TT
	37679	96.38	CC	CT	TT	CT	--	CT	CC	CT	CT	CC
	17102	96.58	--	CT	CC	CT	CC	CT	TT	CC	CT	TT
	26605	96.79	AA	AG	GG	AG	--	AG	AA	GG	AG	AA
	26417	96.99	CC	CT	TT	CT	TT	CT	CC	TT	--	CC
	31833	97.81	AA	AG	GG	AG	GG	--	AA	--	AG	AA
	17697	98.35	--	CG	CC	CG	CC	CG	GG	CC	CG	GG
	25631	99.06	--	AC	AA	AC	AA	AC	CC	AA	AC	CC
	8074	103.02	CT	CT	CC	CT	CC	CT	TT	CC	CT	TT
	26035	103.43	--	CT	CC	CT	CC	CT	TT	CC	CT	TT
	28704	104.81	AT	AT	TT	AT	--	AT	--	TT	AT	AA
	12876	105.42	CT	CC	CC	--	CT	CT	--	CC	CT	TT
	27323	105.58	AC	AC	AA	AC	AC	AC	CC	AA	AC	CC
	7259	105.76	GT	GT	TT	GT	GT	GT	GG	TT	GT	GG
	25967	105.93	--	CT	TT	--	CT	CT	CC	TT	CT	CC
	6145	106.11	CG	CG	GG	CG	CG	CG	CC	GG	CG	CC
	36404	106.83	CG	CG	--	CG	CG	CG	CC	GG	CG	CC
	7269	107.36	AC	AC	CC	AC	AC	AC	AA	--	AC	AA
	14830	113.69	CG	CG	GG	CG	CG	CG	CC	GG	CG	CC
	30345	5.29	AA	--	AT	AA	AT	AT	AT	AT	AT	AT
	28411	15.91	--	AG	AG	AG	--	AG	AG	AG	AG	GG
	36113	26.92	AA	AG	GG	AG	GG	AG	--	AG	AG	GG
	12534	36.04	AG	AG	AA	AG	--	AG	AG	AG	AG	AA
	5321	41.05	AG	AG	GG	AG	GG	AG	AG	GG	AG	GG
	16146	42.33	AC	AC	CC	AC	CC	AC	AC	CC	AC	CC
	12526	43.05	AG	AG	GG	AG	GG	AG	AG	GG	AG	GG
	13771	44.12	AG	AG	GG	AG	GG	AG	AG	GG	AG	GG
group20	17947	44.48	--	GT	TT	GT	TT	GT	GT	TT	GT	TT
	25720	44.74	GT	GT	--	GT	--	GT	GT	GG	GT	GG
	6753	45.01	AC	AC	AA	AC	AA	AC	AC	AA	AC	AA
	28790	45.37	CT	CT	CC	CT	CC	CT	CT	CC	CT	CC
	32290	45.54	CT	CT	--	--	CC	CT	CT	CC	CT	CC
	18031	45.90	GT	GT	TT	GT	TT	GT	GT	TT	GT	TT
	18998	46.08	AT	AT	TT	AT	TT	AT	AT	TT	AT	TT
	9093	46.34	CG	CG	CC	CG	CC	CG	CG	CC	--	CC
	15391	46.61	AC	AC	CC	AC	--	AC	AC	CC	AC	CC
	21583	46.80	CT	CT	TT	CT	TT	CT	CT	TT	CT	TT
	25280	47.16	CG	CG	CC	CG	CC	CG	CG	CC	CG	CC
	27580	47.34	CT	CT	TT	CT	--	CT	CT	TT	CT	TT
	10862	47.52	AT	AT	TT	AT	TT	AT	--	TT	AT	TT
	22224	47.69	--	CT	CC	--	CC	CT	CT	CC	CT	CC
	28356	47.88	AG	AG	AA	AG	AA	AG	AG	AA	AG	AA
	22183	48.05	AT	AT	TT	AT	TT	AT	AT	TT	AT	TT
	17171	48.60	AT	AT	TT	AT	AT	AT	AT	TT	AT	TT
	5074	49.46	AC	AC	CC	AC	AC	AC	AC	CC	AC	CC

	12591	50.88	GT	GT	GG	GT	GT	GT	GT	GG	GT	GG
	37804	51.68	AG	AG	--	AG	AG	AG	AG	AA	--	AA
	13256	52.19	GT	GT	TT	GT	GT	GT	GT	TT	GT	TT
	11420	52.19	AG	AG	GG	AG	AG	AG	AG	GG	AG	GG
	9043	52.91	CT	CT	CC	CT	CT	CT	CT	CC	CT	CC
	10806	53.39	AG	AG	GG	AG	AG	AG	AG	GG	AG	GG
	11482	53.97	CT	CT	CC	CT	CT	CT	CT	CC	CT	CC
	26726	54.35	AC	AC	AA	--	AC	AC	AC	AA	--	AA
	32778	56.08	AG	AG	GG	AG	AG	AG	AG	GG	AG	GG
	21920	58.97	CT	CT	CC	CT	CT	CT	CT	CC	CT	CC
	16492	64.03	AA	AG	AG	AG	AG	AG	GG	AG	AG	AA
	37632	73.88	TT	CT	CT	CT	CT	CC	CC	CT	CT	TT
	28420	78.32	GT	GG	GT	GT	GT	GG	GG	GT	GT	GT
	18786	82.59	AG	AG	GG	AG	GG	GG	GG	GG	GG	AG
	39051	92.50	AA	AT	AT	AA	AT	TT	TT	AT	AT	AA
	15832	97.66	AA	AA	AT	AA	AT	TT	TT	AT	AT	AA
	30684	102.16	--	AA	--	--	AC	CC	CC	AC	AA	AA
	29918	103.06	TT	TT	GT	TT	GT	GG	GG	GT	TT	TT
	35325	0.00	TT	TT	CT	CT	TT	TT	CT	CT	--	TT
	10548	5.12	--	AG	AG	AG	GG	AG	AA	AG	AG	GG
	18349	9.99	GG	GG	CG	CG	CG	GG	GG	CG	GG	CG
	27276	14.55	--	TT	CT	TT	CT	TT	TT	CT	TT	CT
	7536	18.82	GT	GT	GT	GG	GT	GT	GG	GG	GT	GT
	24810	27.69	GG	AA	GG	AA	GG	GG	AA	AG	AG	GG
	25753	34.40	--	AA	AC	AA	AC	AC	AA	AC	AA	AC
	30461	41.59	CG	GG	CG	GG	CG	CG	GG	CG	GG	CG
group21	23388	46.94	GT	TT	GT	TT	GT	GT	TT	GT	GT	GT
	18450	52.14	TT	CC	TT	CC	TT	TT	CC	CT	TT	TT
	35345	57.08	AG	AG	AG	AG	AG	AG	AG	AA	AG	AG
	36368	62.65	CT	CC	CT	--	--	CT	CT	CT	CT	CT
	20949	68.99	--	CG	CC	CG	CC	CC	CC	CC	CC	CC
	38536	69.36	AA	AC	AA	AC	AC	AA	AA	AC	AA	AA
	5017	74.20	AA	GG	AA	GG	AG	AA	AA	AG	AA	AA
	16168	75.34	CT	TT	CT	TT	TT	CT	CT	TT	CT	CT
	10036	76.65	CG	GG	CG	GG	CG	CG	CG	CG	CG	CG
	20782	77.00	GG	AA	GG	AA	AG	GG	GG	AG	GG	GG
	23357	78.44	--	CG	GG	--	--	GG	GG	GG	GG	GG
	30970	78.79	--	CC	--	CC	CG	GG	GG	CG	GG	GG
	34066	79.33	CT	CC	TT	CC	CT	TT	TT	CT	--	TT
	21480	79.45	AA	GG	AA	GG	AG	--	AG	AG	AA	AA
	5279	79.63	GG	AG	GG	AG	AG	GG	GG	AG	GG	GG
	14812	83.82	TT	CC	TT	CC	CT	TT	TT	CT	TT	TT
	17465	83.91	AG	GG	AG	GG	GG	AG	AG	GG	AG	AG
	8707	84.00	AA	GG	AA	GG	AG	AA	AA	AG	AA	AA
	18769	84.89	AA	CC	AA	CC	AC	AA	AA	AC	AA	AA
	25890	85.07	AT	TT	AT	TT	AT	AT	AT	AT	AT	AT
	19050	85.61	--	AA	GG	AA	AG	GG	GG	AG	GG	GG

33304	86.50	CC	AA	CC	AA	AC	CC	--	AC	CC	CC
30700	87.03	GG	AA	GG	AA	AG	GG	AG	AG	GG	GG
8292	88.23	AG	AA	AG	AA	AA	--	AG	AA	AG	AG
36367	89.19	CT	CC	CT	CC	CT	CT	CC	CT	CT	CT
12568	90.09	CG	CC	CG	CC	CG	CG	CC	CG	CG	CG
17387	95.28	CT	TT	CT	TT	CT	CT	TT	CT	CT	CT
36164	100.81	GG	CG	CG	CG	GG	GG	--	GG	GG	GG
16059	105.31	CT	CC	CC	CC	CT	CT	CC	CT	CT	CT
9260	106.91	--	GT	GT	GT	GG	GG	GT	GG	GG	GG

1-15	1-16	1-17	1-20	1-23	1-24	1-25	1-28	1-29	1-31	1-32	1-33	1-35
AC	AC	AC	AA	AC	AA	AC	CC	AA	AA	AA	AA	CC
AG	AG	AG	AA	AG	AA	AG	GG	AA	AA	AA	AA	AG
CT	CT	CT	TT	CT	TT	CT	CC	TT	TT	CT	CT	CT
AG	AG	GG	GG	AG	GG	AG	AG	GG	GG	GG	GG	GG
AG	AG	--	AA	AG	--	AG	GG	AA	AA	AG	AG	AG
CG	CG	GG	CG	CG	GG	CG	GG	CG	CG	GG	GG	GG
CT	CT	CT	TT	CT	CT	TT	CC	TT	TT	CT	TT	CT
GT	GT	GT	TT	GG	GT	GT	GG	GT	TT	GT	GT	GT
GT	GT	GT	TT	GG	GT	GT	GT	GT	GT	GT	GT	GT
GT	GT	GT	TT	GG	GT	GT	GT	GT	GT	GT	GT	GT
AG	AG	AA	AG	GG	AG	AG	AG	AG	AG	AG	AG	AG
CT	CC	CT	CT	CC	CC	CC	CT	CT	CC	CC	CC	CC
AC	CC	AA	AC	CC	AC	AC	AC	AC	AC	AC	AC	AC
AG	AA	AG	AG	AA	AA	AA	AG	AG	AA	AA	--	AA
CC	CC	CT	CC	CC	CT	CT	CC	CC	CT	CT	CT	CT
AA	AA	GG	AA	AA	AG	AG	AG	AG	AG	AG	GG	AG
TT	TT	TT	TT	TT	TT	TT	AT	AT	TT	TT	AT	TT
CC	CC	CT	CC	CC	CT	CT	CT	CC	CT	CT	CT	CT
TT	TT	TT	TT	TT	TT	TT	TT	GT	GT	TT	GT	TT
CC	CC	CC	CC	CG	CC	CC	CC	CC	CG	CC	CG	CC
TT	TT	TT	TT	AT	AT	AT	AT	TT	AA	AT	AA	AT
TT	TT	TT	TT	CT	CC	CT	CT	TT	CC	CT	CC	CT
AA	AA	AA	AA	AT	AT	AT	AT	AA	TT	AT	TT	AT
AA	AA	AA	AA	AT	TT	AT	AT	AA	TT	AT	TT	AT
AA	AA	AA	AA	AG	GG	AG	AG	AA	GG	AG	GG	AG
TT	TT	TT	--	GT	GT	TT	TT	TT	GT	TT	GT	TT
TT	TT	TT	TT	GT	GG	GT	GT	TT	GG	GT	GG	GT
TT	TT	TT	TT	AT	AA	AT	AT	TT	AA	AT	AA	AT
TT	TT	TT	TT	CT	CC	CT	CT	TT	CC	CT	CC	CT
CC	CC	CC	CC	CG	GG	CG	CG	CC	GG	--	GG	CG
CC	CC	CC	CC	CT	TT	CT	CT	CC	TT	CT	TT	CT
AA	AA	AA	AA	AC	CC	AC	AC	AA	CC	AC	CC	AC
AA	AA	AA	AA	AG	GG	AG	AG	AA	GG	AG	--	AG
GG	GG	GG	GG	GT	TT	GT	GT	GG	TT	GT	TT	GT
AA	AA	AA	AA	AG	GG	AG	AG	AA	GG	AG	GG	AG
TT	TT	TT	TT	GT	GG	GT	GT	TT	GG	GT	GG	GT
GG	GG	GG	GG	AG	AA	AG	AG	GG	AA	AG	AA	AG
CC	CC	CC	CC	CT	TT	CT	CT	CC	TT	CT	TT	CT
AA	AA	AA	AA	AT	TT	AT	AT	AA	TT	AT	TT	AT
AA	AA	AA	AA	AG	--	AG	AG	AA	GG	AG	GG	AG
AA	AA	AA	AA	AC	CC	AC	AC	AA	CC	AC	CC	AC
AA	AA	AA	AA	AG	GG	AG	AG	AA	GG	AG	GG	AG

TT	TT	TT	--	CT	CC	CT	CT	TT	CC	CT	CC	CT
CC	CC	CC	CC	CG	GG	CG	CG	CC	GG	CG	GG	CG
TT	TT	TT	--	TT	CC	CT	CT	TT	CC	CT	CC	CC
CC	CC	CC	CT	CC	TT	CT	CT	CC	TT	CT	TT	TT
AA	AC	AA	AC	AA	CC	AC	AC	AA	CC	AC	CC	CC
CC	CT	CC	CT	CC	TT	CT	TT	CT	TT	CT	TT	TT
TT	CT	TT	CT	CT	CC	CT	CC	CT	CT	CT	CC	CC
AG	GG	GG	GG	AG	--	AA	AG	AA	GG	AG	GG	GG
GT	GT	GG	GT	GT	GT	TT	GT	TT	GG	GT	GT	GG
AG	AG	AG	AG	AG	AG	AA	AG	AA	AG	AG	--	GG
CC	CT	CT	CT	CT	CT	CC	CT	CC	CT	CT	CT	CT
GG	CC	CG	CG	CG	CG	GG	CG	--	CG	CG	CG	CG
GG	AA	AG	AG	AG	AG	GG	AA	AG	AA	AG	AG	AG
AA	CC	AC	AC	AC	AC	AA	CC	AC	CC	AC	AC	AC
GG	AG	AG	AG	AG	GG	GG	AG	GG	AG	AG	AG	AG
CC	TT	CT	--	CT	CC	CC	TT	CT	TT	CT	CT	TT
TT	CC	CT	CT	CT	TT	TT	CC	CT	CC	CT	CT	--
CT	TT	CT	CT	CT	CC	--	TT	CT	TT	TT	CT	TT
AG	AA	AG	AG	AG	GG	GG	AA	AG	AA	AA	AG	AA
AC	AA	AA	AC	AC	CC	CC	AA	AC	AA	AA	AC	AA
AG	AA	AA	AA	AG	GG	GG	AA	AG	AA	AA	AG	AA
CG	GG	GG	GG	CG	--	CC	GG	CG	CG	GG	--	GG
CG	CC	CC	CC	CG	GG	GG	CC	CG	CG	CC	--	CC
GG	AA	AA	AA	AG	GG	GG	AA	AA	AG	AA	AG	AA
AA	CC	--	CC	AC	AA	AA	CC	CC	AC	AC	AC	CC
CT	TT	TT	TT	CT	CC	CT	TT	TT	CT	CT	CT	TT
CT	CC	CC	CC	CT	TT	CT	CT	CC	CT	CT	CT	CC
AA	TT	TT	TT	AT	AT	AT	AT	TT	AT	AT	--	TT
AG	GG	GG	--	GG	AG	GG	AG	GG	AG	GG	GG	GG
AT	TT	AT	--	AT	TT	AT	TT	TT	TT	AT	--	TT
TT	TT	GT	--	GT	GG	GG	GT	GT	GT	GG	--	GG
GG	GG	AG	AG	GG	AG	AG	GG	AG	AG	AG	AG	AG
TT	TT	CT	CT	TT	CT	CT	TT	CT	CT	CT	CT	CT
AG	GG	AG	AG	GG	GG	AG	GG	AG	AG	AG	AG	AG
GT	GG	GT	GT	GG	GT	TT	GT	GT	GT	TT	TT	TT
GT	GG	GT	GT	GG	--	GT	GG	GG	GT	--	GT	GT
CC	TT	CT	CT	TT	CT	CC	CT	TT	CT	CT	CC	CC
CC	CT	CT	CT	TT	CT	CC	CT	TT	CT	CT	CC	CC
GG	AG	AG	AG	AA	AG	GG	AG	AA	AA	AG	GG	GG
CC	CG	CG	CG	GG	CG	CC	CG	GG	GG	CG	--	CC
GG	AG	AG	AG	AG	AG	GG	AG	AA	AA	AG	GG	GG
TT	CT	CT	CT	CT	--	CT	CT	CC	CT	CT	TT	TT
TT	CT	CT	CT	CT	CT	CT	TT	CC	CT	CT	TT	TT
CG	CG	CC	CG	CG	CC	CC	CG	CC	CC	CG	CG	CG
TT	TT	TT	AT	AT	TT	TT	AT	TT	TT	AT	AT	AT
GT	GG	GT	GG	GG	GT	GT	GT	GG	GT	GG	GT	GT
CT	CC	CT	CC	CC	CT	CT	CT	CC	CT	CC	CT	CT

CT	CT	CT	CT	CT	CT	CT	TT	CC	CT	CC	TT	TT
GT	GT	GT	GG	GG	GT	GT	GT	GG	GT	GG	GT	GT
CT	CT	CT	CT	CT	CT	CT	TT	CC	CT	CC	--	TT
GT	GT	GT	TT	TT	GT	GT	GT	TT	GT	TT	GT	GT
CG	CG	CG	--	GG	CG	CG	CG	GG	CG	GG	CG	CG
AG	AG	AG	AG	AG	AG	GG	AG	AA	AA	AG	--	AG
AG	AG	AG	AG	AG	AG	AA	AG	GG	GG	AG	AG	AG
GG	AG	AG	AG	AG	AG	AA	AG	GG	GG	AG	AG	AG
CC	CT	TT	CT	CT	CT	CT	CT	CC	CC	CT	CT	CT
--	AG	GG	AG	AG	AG	AG	AG	AA	AA	AG	GG	AG
TT	GT	GG	GT	GT	GG	GT	GT	TT	TT	GT	GT	GT
GG	AG	AG	GG	GG	AG	GG	GG	GG	GG	GG	AG	AG
CC	CT	TT	CT	CT	TT	CT	CT	CC	CC	CT	CT	CT
AC	AC	CC	CC	CC	CC	CC	CC	AC	AC	CC	AC	AC
CC	CC	CT	CT	CT	CT	CT	CT	CC	CC	CT	CC	CC
TT	GT	GG	GT	GT	GG	GT	GT	TT	TT	GT	GT	GT
CT	CC	CC	CT	CT	CC	CT	CT	CT	CT	CT	CC	CC
CG	CG	GG	GG	GG	GG	GG	GG	CG	CG	GG	CG	CG
AC	AC	CC	CC	CC	CC	CC	CC	CC	AC	CC	AC	AC
CT	TT	TT	CT	CT	TT	CT	CT	CT	CT	CT	TT	TT
AG	AA	AA	AG	AG	AA	AG	AG	AG	AG	AG	AG	AA
GG	AG	AA	AG	AG	AA	AG	AG	GG	GG	AG	GG	AG
CC	CC	CT	CT	CT	CT	CT	CT	CC	CC	CT	CC	CC
AT	AT	AA	AA	AA	AA	AA	AA	AT	AT	AA	AT	AT
CT	CT	CC	CC	CC	CC	CC	CC	CT	CT	CC	CT	CT
AG	AG	AA	AA	AA	AA	AA	AA	AG	AG	AA	AG	AG
CT	CC	CC	CT	CT	CC	CT	CT	CT	CT	CT	CT	CC
AA	AT	TT	--	AT	TT	AT	AT	AA	AA	AT	--	AT
AA	AT	TT	AT	AT	TT	AT	TT	AA	AA	AT	AA	AT
CC	CT	TT	CT	CT	TT	CT	TT	CC	CC	CT	CC	CT
CT	CT	CC	CC	CC	CC	CC	CC	CT	CT	CC	CT	CT
GT	GG	--	--	GT	GG	GT	GG	GT	GT	GT	GT	GG
CT	CC	CC	CT	CT	CC	CT	CC	CT	CT	CT	CT	CC
--	AG	AG	AA	AA	AG	AA	AG	AA	AA	AA	AA	AG
AA	AG	GG	AG	AG	GG	AG	GG	AA	AA	AG	AA	AG
AG	AA	AA	AG	AG	AA	AG	AA	AG	AG	AG	AG	AA
TT	CT	CC	CT	CT	CC	CT	CC	CT	TT	CT	TT	CT
AC	CC	CC	AC	AC	CC	AC	CC	CC	AC	AC	AC	CC
AA	AT	TT	--	AT	TT	AT	TT	AT	AA	AT	AA	AT
AG	AG	--	AA	AA	AA	AA	AA	AG	AG	AA	AG	AG
AC	AA	AA	AC	AC	AA	AC	AA	AA	AC	AC	AC	AA
AT	TT	TT	AT	AT	TT	AT	TT	TT	AT	AT	AT	TT
AC	CC	AC	--	AA	AC	AA	AC	CC	AC	AA	AC	CC
AC	AC	CC	CC	CC	CC	CC	CC	AC	AC	CC	AC	AC
GG	AG	AG	GG	GG	AG	GG	AG	AG	GG	GG	GG	AG
CC	CC	AC	AC	AC	AC	AC	AC	CC	CC	AC	CC	CC
AG	AA	AA	AG	AG	AA	AG	AA	AA	AG	AG	AG	AA

CG	CG	CC	CC	CC	CC	CC	CC	CG	CG	CC	--	CG
CC	CT	TT	--	CT	TT	CT	TT	CT	CC	CT	CC	CT
TT	CT	CC	CT	CT	CC	CT	CC	CT	TT	CT	TT	CT
CT	CC	CC	CT	CT	--	CT	CC	CC	CT	CT	--	CC
AG	AA	AA	AG	AG	--	AG	AA	AA	AG	AG	AG	AA
TT	CT	--	CT	CT	CC	CT	CC	CT	TT	CT	TT	CT
CT	TT	TT	CT	CT	--	CT	TT	TT	CT	CT	CT	TT
TT	AT	AA	--	AT	AA	AT	AA	AT	TT	AT	TT	AT
AG	AG	GG	GG	GG	GG	GG	GG	AG	AG	GG	AG	AG
AC	AA	AC	AC	AC	AA	AC	AA	AA	AC	AC	AC	AA
CT	CC	CT	CT	CT	CC	CT	CC	CC	CT	CT	CT	CC
GT	GT	TT	TT	TT	TT	TT	TT	GT	GT	TT	GT	GT
CT	TT	CT	TT	CT	--	CT	TT	TT	CT	CT	CT	TT
CC	AC	CC	CC	CC	AC	CC	AC	AC	CC	CC	CC	AC
AG	AA	AG	--	AG	AA	AG	AA	AA	AG	AG	--	AA
GT	GG	GT	--	GT	GG	GT	GG	--	GT	GT	--	GG
TT	AT	TT	--	TT	AT	TT	AT	AT	TT	TT	--	--
AG	AA	AG	AG	AG	AA	AG	AA	AA	AG	AG	AG	AA
CT	CT	CC	CC	CC	CC	CC	CC	CT	CT	CC	CT	CT
CT	CC	CT	CT	CT	CC	CT	CC	CC	CT	CT	CT	CC
AC	AA	AC	AC	AC	AA	AC	AA	AA	AC	AC	AA	AA
CT	CC	CT	CT	CT	CC	CT	CC	CC	CT	CT	CC	CC
TT	AT	AT	AT	AT	AA	AT	AA	AT	TT	AT	TT	AT
AA	AG	AG	AG	AG	GG	AG	GG	AG	AA	AG	AG	AG
CG	CG	GG	GG	GG	GG	GG	GG	CG	CG	GG	CG	CG
CT	TT	CT	CT	CT	TT	CT	TT	TT	CT	CT	TT	TT
AG	GG	AG	AG	AG	GG	AG	GG	GG	AG	AG	GG	GG
AG	AA	AG	--	AG	AA	AG	AA	AA	AA	AG	AA	AA
AG	AG	AA	AA	AA	AA	AA	AA	AG	AG	AA	AG	AG
AG	AG	GG	--	GG	GG	GG	GG	AG	AG	GG	AG	AG
CT	TT	CT	TT	CT	TT	CT	TT	CT	TT	--	TT	TT
TT	GT	GT	--	GT	GG	GT	GG	TT	GT	GT	GT	GT
GT	GT	TT	TT	TT	TT	TT	TT	GT	GT	TT	GT	GT
AA	AG	AG	GG	AG	GG	AG	GG	AA	AG	AG	AG	AG
CT	CC	CT	CC	CC	CC	CT	CT	CT	CC	CT	CC	CC
GT	TT	GT	TT	TT	TT	GT	GT	GT	TT	GT	TT	TT
GG	GG	AG	AG	AG	AG	AG	AG	GG	GG	AG	GG	GG
--	AG	AG	AA	AG	GG	AA	AG	AA	AA	AG	AG	GG
CC	CT	CC	CT	CT	CC	--	CT	--	CT	CC	CC	CC
AT	AT	AA	AA	AT	AT	AA	AT	AA	AA	AA	AA	AT
GG	CG	CG	CC	CG	GG	CG	CG	CC	CC	CG	CC	GG
AC	AC	AC	AA	AA	AC	AA	AA	AA	AA	AC	AA	AC
GG	GG	--	AA	AG	GG	AG	AG	AG	AA	AG	AA	GG
GG	GG	GG	AG	GG	GG	GG	GG	AG	AG	AG	AG	GG
CC	CC	CC	CT	CC	CC	CC	CC	CT	CT	CT	CT	CC
AA	AA	AG	AG	AG	AG	AG	AG	AG	GG	AG	GG	AG
CT	CT	CC	CT	CC	CC	CC	CC	CT	CC	CT	CC	CC

AG	AG	AG	GG	AG	AG	AG	AG	GG	GG	GG	GG	AG
AG	AA	AG	GG	AA	AG	AG	AA	AG	AG	GG	--	AA
TT	CT	TT	CC	CT	CC	TT	CT	CC	CC	CT	CC	CT
TT	AT	TT	AA	AT	AA	TT	AT	AA	AA	AT	AA	AT
CC	AC	CC	--	CC	AA	CC	AC	AA	AA	CC	AA	AC
CC	CT	CC	TT	CC	--	CC	CT	TT	TT	CT	CT	CT
AA	AG	AA	GG	AA	--	AA	AG	GG	AG	AG	AG	AG
TT	CT	TT	CC	TT	CC	TT	CT	CC	CT	CT	--	CT
CT	TT	CT	TT	CT	TT	CT	TT	TT	TT	TT	TT	TT
GG	AG	GG	AA	GG	AA	GG	AG	AA	AG	AA	GG	AG
AG	GG	GG	AG	AA	AA	GG	AA	GG	AG	AA	AA	AG
CT	TT	--	--	CC	CC	TT	CT	TT	CT	CC	CC	CT
GG	AG	AG	--	GG	AG	AG	GG	AG	GG	GG	--	GG
CT	TT	TT	CC	CC	CT	TT	CC	TT	CT	CC	CC	CT
CG	CG	CG	GG	GG	GG	CG	GG	CG	CG	GG	GG	CG
CT	CT	TT	CC	CC	CT	TT	CC	TT	CT	--	--	CT
CC	CC	CT	--	CC	CT	CT	CC	CT	CC	CC	CC	CC
AG	AG	AA	GG	AG	AG	AA	GG	AA	AG	GG	GG	AG
CT	CC	TT	CT	CT	CT	TT	CC	TT	CT	--	CC	CT
AG	AA	AG	AG	AG	AG	GG	AA	AG	AG	AA	AA	AG
CT	CC	CT	CT	CT	CT	TT	CC	CT	CT	CC	CC	CT
AA	GG	AG	AG	AG	AG	AA	GG	AG	AG	GG	GG	AG
AG	GG	AG	AG	GG	AG	AG	GG	GG	GG	GG	GG	GG
GG	TT	GT	GG	GT	GG	GG	TT	GT	GT	GT	TT	GT
CG	CC	CG	CG	CC	CG	CG	CC	CC	CC	CG	CC	CC
CC	CG	CG	CC	CC	CC	CC	CG	CC	CC	CG	CG	CC
TT	CC	CT	TT	CT	--	TT	CC	CT	CT	CT	CC	CT
CC	TT	CT	CC	CT	CT	CC	TT	CT	CT	CT	TT	CT
TT	AA	AT	TT	AT	AT	TT	AA	AT	AT	AT	AA	AT
AA	AG	AG	AA	AG	AG	AA	GG	AG	AG	GG	GG	AG
TT	CT	CT	TT	CT	CT	TT	CC	CT	CT	CC	CC	CT
TT	CT	CT	CT	TT	TT	CT	TT	CT	TT	TT	TT	TT
CC	AC	AC	CC	AC	AC	CC	AA	AC	AC	AA	AA	AC
TT	TT	GG	--	TT	TT	GG	TT	GG	GG	GT	GG	TT
AA	AA	GG	AG	AA	AA	GG	AA	GG	GG	AG	GG	AA
--	TT	CC	CT	TT	--	CC	TT	CC	CC	CT	CC	TT
AA	AA	TT	AT	AA	AA	TT	AA	TT	TT	AT	TT	AA
CC	CC	AA	AC	CC	CC	AC	CC	AA	AA	AC	AA	CC
CG	CG	CC	CG	CG	CG	CG	CG	CC	CC	CC	CC	CG
AG	AG	AA	AG	AG	AG	AG	AG	AA	AA	AA	AA	AG
CC	CC	TT	CT	CC	CC	CT	CC	TT	TT	CT	TT	CC
CC	CC	TT	CT	CC	CC	CT	CC	TT	TT	CT	TT	CC
AA	AA	GG	AG	AA	AA	AG	AA	GG	GG	AG	GG	AA
CT	CT	CC	CC	CT	CT	CC	CT	CC	CC	CT	CC	CT
AA	AA	GG	AG	AA	AA	AG	AA	GG	GG	AG	GG	AA
--	GG	AA	AG	GG	GG	AG	GG	AA	AA	AG	AA	GG
GG	GG	TT	GT	GG	GG	GT	GG	TT	TT	GT	TT	GG

TT	TT	CC	CT	TT	TT	CT	TT	CC	CC	CT	CC	TT
GG	GG	CC	CG	GG	GG	CG	GG	CC	CC	CG	CC	GG
TT	TT	GG	GT	TT	TT	GT	TT	GG	GG	GT	GG	TT
AA	AA	GG	AG	AA	AA	AG	AA	GG	GG	AG	GG	AA
CC	CC	TT	--	CC	CC	CT	CC	TT	TT	CT	TT	CC
GT	GT	TT	TT	GT	GT	TT	GT	TT	TT	GT	TT	GT
GG	GG	CC	CG	GG	GG	CG	GG	CC	CC	CG	CC	GG
AA	AA	GG	--	AA	AA	AG	AA	GG	--	AG	--	AA
AG	AG	GG	GG	AG	AG	GG	AG	GG	GG	AG	GG	AG
AA	AA	CC	AC	AA	AA	AC	AA	CC	CC	AC	CC	AA
CG	CG	CC	CC	CG	CG	CC	CG	CC	CC	CG	CC	CG
GG	GG	AA	AG	GG	GG	AG	GG	AA	AA	AG	AA	GG
AA	AA	--	AC	AA	AA	AC	AA	CC	CC	AC	CC	AA
TT	TT	GG	GT	TT	TT	GT	TT	GG	GG	GT	GG	TT
CC	CC	AA	AC	CC	CC	AC	CC	AA	AA	AC	AA	CC
CT	CT	CC	CC	CT	CT	CC	CT	CC	CC	CT	CC	CT
TT	TT	GG	GT	TT	TT	GT	TT	GG	GG	GT	GG	TT
AG	AG	GG	AG	AG	AG	AG	AG	GG	GG	GG	GG	AG
CC	CC	TT	CT	CC	--	CT	CC	--	TT	CT	TT	CC
CT	CT	CC	CC	CT	CT	CC	CT	CC	CC	CT	CC	CT
AA	AA	TT	AT	AA	AA	AT	AA	TT	TT	AT	TT	AA
CC	CC	GG	CG	CC	CC	CG	CC	GG	GG	CG	GG	CC
GG	GG	TT	GT	GG	GG	GT	GG	TT	TT	TT	TT	GG
AG	AG	GG	AG	AG	AG	AG	GG	GG	GG	GG	--	AG
TT	TT	GT	GT	GT	GT	GT	TT	GT	GT	GT	GT	TT
CC	CC	TT	CT	CT	CT	CT	CT	TT	TT	TT	--	CC
CC	CC	AA	AC	AC	AC	AC	AA	AA	AC	AA	AA	CC
CC	CC	CT	CC	CC	CC	CC	CT	CC	CC	CT	CT	CC
GG	GG	CC	CG	CG	CG	CG	CC	CG	CG	CC	CC	GG
CC	CC	CT	CT	CT	TT	CT	TT	CT	CT	CT	CT	CC
GT	GT	TT	GT	GT	TT	GT	TT	GT	GT	TT	GT	GT
CT	CT	CT	TT	TT	TT	TT	TT	TT	TT	CT	TT	CT
AT	AT	TT	AT	AT	TT	AT	TT	AT	AT	--	--	AT
CC	CC	AC	CC	CC	AC	CC	AC	CC	CC	AC	CC	CC
TT	TT	TT	TT	CT	CC	CT	CC	CT	CT	CT	CC	TT
AA	AA	AA	AA	AA	AG	AA	AG	AA	AA	--	AA	AA
GG	GG	GG	--	GG	AG	GG	AG	GG	GG	AG	GG	GG
GG	CG	GG	--	CG	CG	CG	GG	CG	GG	GG	GG	GG
CT	TT	--	--	TT	TT	CT	CT	CT	CC	CT	CC	CC
TT	CT	CT	CC	TT	TT	CT	TT	TT	CC	CT	CT	CC
TT	CC	CT	CC	TT	TT	CC	TT	TT	CC	TT	CT	CT
CC	TT	--	TT	CC	CC	TT	CC	CT	TT	--	CT	CT
TT	GG	GT	GG	TT	TT	GG	TT	GT	GG	TT	GT	GT
CC	TT	CT	--	CC	CC	TT	CC	CT	TT	CC	--	CT
AA	TT	AT	--	AA	AA	TT	AA	AT	TT	AA	AT	AT
AA	GG	AG	GG	AA	AA	GG	AA	AG	GG	AA	AG	AG
CC	AA	AC	AA	AC	AC	AA	CC	AC	AA	CC	AC	AC

AA	GG	AG	GG	AG	AG	GG	AA	AG	GG	AA	AG	AG
AA	GG	AG	--	AG	AG	GG	AA	AG	GG	AA	AG	AG
TT	CC	CT	CC	CT	CT	CC	TT	CT	CC	TT	CT	CT
GG	AA	AG	AA	AG	AG	AA	GG	AG	AA	GG	AG	AG
GG	TT	GT	TT	GT	GT	TT	GG	--	TT	GG	GG	GT
TT	CC	CT	CC	--	CT	CC	TT	CT	CC	TT	--	CT
GG	AA	--	AA	AG	--	AA	GG	AG	AA	GG	AG	AG
--	AA	AG	AA	AG	AG	AA	GG	AG	AA	GG	--	AG
AA	GG	AG	GG	AG	AG	GG	AA	AG	GG	AA	--	AG
GG	CC	CG	--	CG	CG	CC	GG	--	CC	GG	CG	CG
AA	GG	AG	--	AG	AG	GG	AA	AG	GG	AA	AG	AG
AA	CC	AC	CC	AC	AC	CC	AA	AC	CC	AA	CC	AC
GG	AA	GG	--	AG	AG	AA	GG	AG	AA	GG	AG	AG
GG	AA	GG	AA	AG	AG	AA	GG	AG	AA	GG	--	AG
AA	TT	AA	TT	AA	AT	TT	AT	AT	TT	AA	AT	AT
TT	CC	TT	CC	TT	CT	CC	CT	CT	CC	TT	CT	CT
TT	CC	TT	CC	TT	CT	CC	CT	CT	CC	TT	--	CT
CC	GG	CC	GG	CC	CG	GG	CG	CG	GG	CC	CG	CG
CC	TT	--	TT	CC	CT	TT	CT	CT	TT	CC	CT	CT
--	CC	TT	CC	TT	CT	CC	CT	CT	CC	--	CT	CT
CC	TT	CC	TT	CC	CT	TT	CT	CT	TT	CC	CT	CT
AA	TT	AA	TT	AA	AT	TT	AT	AT	TT	AA	AT	AT
AT	AA	TT	AA	TT	AT	AA	AT	AT	AA	TT	AT	AT
CT	TT	CC	TT	CC	CT	TT	CT	CT	TT	CC	CT	CT
CT	CC	TT	CC	TT	CT	CC	CT	CT	CC	TT	CT	CT
AT	AA	TT	AA	TT	AT	AA	AT	AT	AA	TT	TT	AT
AG	GG	AA	GG	AA	AG	GG	AG	AG	GG	AA	AG	AG
GT	GG	TT	GG	TT	GT	GG	GT	GT	GG	--	GT	GT
CT	TT	CC	TT	CC	CT	TT	CT	CT	TT	CC	CT	CT
CG	CC	GG	CC	GG	CG	CC	CG	CG	CC	CG	CG	CG
AG	GG	AA	GG	AA	AG	GG	AG	AG	GG	AG	AG	AG
CT	TT	CC	TT	CC	CT	TT	CT	CC	TT	CT	--	CT
CT	TT	CC	TT	CC	CT	TT	CT	CC	TT	CT	CT	CT
AT	TT	AA	TT	AA	AT	TT	AT	AA	TT	AT	AT	AT
AG	AA	GG	AA	GG	AG	AA	AG	GG	AA	AG	AG	AG
AG	AA	GG	AA	GG	AG	AA	AG	GG	AA	AG	AG	AG
GT	TT	GG	TT	GG	GT	GT	GT	GG	TT	GT	GT	GT
AA	AG	GG	AG	GG	AG	AG	AG	GG	AG	AG	AG	AG
GG	AG	AG	AG	AG	--	AG	GG	AG	GG	GG	GG	AG
CT	TT	CT	CT	TT	CT	TT	CT	TT	TT	CT	CT	CT
AG	GG	AA	AG	GG	AA	GG	AG	GG	AG	AA	AA	AA
GG	GG	AG	GG	GG	AG	GG	GG	GG	AG	AG	AG	AG
AA	GG	AG	AG	GG	AA	--	AG	GG	AG	AA	AA	AA
GG	CC	CG	CG	CC	GG	CG	CC	CC	CG	GG	GG	GG
CC	TT	CT	CT	TT	CC	CT	TT	TT	CT	CT	CC	CC
CC	AC	CC	CC	AC	CC	CC	AC	AC	AC	CC	CC	CC
--	AG	AG	AA	AA	AA	AG	AG	AG	AA	AG	AA	AA

CC	AC	CC	CC	AC	CC	CC	AC	AC	AC	CC	CC	CC
AC	AC	CC	AC	AC	AC	CC	AC	CC	AC	CC	CC	AC
TT	CT	TT	TT	CT	TT	TT	CT	CT	CT	TT	TT	TT
AA	AG	AG	AA	AG	AA	AG	AG	GG	AG	AG	AA	AA
AG	AG	AG	GG	AG	GG	AG	AG	AA	AG	AG	GG	GG
TT	CT	CT	TT	CT	TT	CT	CT	CC	CT	CC	TT	TT
CT	CT	TT	CT	CT	CT	TT	CT	TT	TT	TT	CT	CT
CC	CG	CG	CC	CG	CC	CG	CG	GG	GG	GG	CC	CC
AA	AG	AG	AA	AG	AA	AG	AG	GG	GG	GG	AA	AA
TT	CT	CT	CT	CT	TT	CT	CT	CC	CC	CC	TT	TT
AT	AT	AA	AT	AA	AT	AA	AT	--	AA	AA	AT	AT
--	AC	AA	AC	AC	AC	AC	AC	AA	AC	AA	CC	AC
--	AG	AG	AG	GG	--	GG	AG	AG	GG	AG	GG	AG
CC	CT	CT	CT	CC	CT	CC	CT	CT	CC	--	--	CT
CC	CT	CT	CT	CC	CT	CC	CT	CT	CC	--	CC	CT
GG	GT	GT	GT	GG	GT	GG	GT	GT	GG	GT	GG	GT
CT	CT	CC	CT	CT	CT	CT	CT	CT	CT	TT	TT	CT
AG	GG	AG	GG	AG	AG	AG	GG	GG	AG	GG	GG	GG
AG	AG	AA	AG	AG	AG	AG	AA	AG	AG	GG	GG	AG
CG	GG	GG	GG	CG	CG	CG	GG	--	CG	CG	--	GG
AA	AG	AA	AG	AG	AA	AG	AA	AG	AG	AG	AG	AG
GG	AG	GG	AG	AG	GG	AG	GG	AG	AG	AG	AG	AG
GT	GT	GT	GT	TT	GT	TT	GT	TT	TT	GT	GT	GT
GG	AG	GG	AG	AG	GG	AG	GG	AG	AG	AG	AG	AA
GG	AG	GG	AG	AG	GG	AG	GG	AG	AG	AG	AG	AA
GT	GT	GT	GT	GG	GT	GG	GT	GG	GG	GT	GT	GG
TT	CT	CT	CT	--	TT	CT	TT	CT	CT	CT	--	CC
CC	CT	CT	CT	CT	CC	CT	CC	CT	CT	CT	CT	TT
CT	CC	CT	CC	CT	CT	CT	CT	CT	CT	CC	CC	CC
GG	AG	AG	AG	AG	GG	AG	GG	AG	AG	AG	AG	AA
TT	CT	CT	CT	TT	TT	CT	TT	CT	CT	CT	CT	CC
GG	AG	AG	AG	GG	AG	AG	GG	AG	AG	AG	AG	AA
--	CT	CT	CT	TT	CT	CT	CT	CT	CT	CT	CT	CC
CG	CG	CG	CG	CG	CG	CG	CG	CG	CG	CG	CG	GG
CT	CC	CT	CT	TT	CC	CC	CT	CT	CC	CT	--	CC
AC	AA	CC	CC	AC	--	AC	CC	AA	AC	CC	AC	AA
AG	GG	--	AA	AG	GG	AG	AA	GG	AG	AA	AA	GG
CT	TT	CC	CC	CT	TT	CT	CC	TT	TT	CC	CC	CT
AG	AA	GG	--	AG	AA	AG	GG	AA	AA	AG	AG	AG
GG	AA	GG	GG	AG	AA	AA	GG	AA	AA	AG	--	AG
CT	TT	CT	CT	TT	TT	TT	CT	TT	TT	CT	CT	TT
AA	GG	GG	AG	AG	GG	GG	AA	GG	GG	AG	AG	AG
AG	AA	AA	AA	AG	AA	AA	AG	AA	AA	AA	AA	AG
GT	TT	TT	GT	GT	TT	TT	GT	TT	TT	TT	TT	GT
GT	TT	TT	TT	GT	TT	TT	GT	TT	TT	TT	TT	GT
CC	AA	AC	CC	AC	AA	AA	CC	AA	AA	AC	AC	AC
GG	AA	AG	GG	AG	AA	AA	GG	AA	AA	AG	AG	AG

CG	CC	CG	CG	CG	CC	CC	GG	CG	CC	CG	CG	CG
CG	CC	CG	CG	CG	CG	CG	GG	CG	CC	CG	CG	CG
CT	TT	CT	CT	CT	CT	CT	CC	CT	TT	CT	CC	CT
CT	CC	TT	CT	CT	CT	CT	TT	CT	CC	CT	CT	CT
AT	AA	TT	AT	AT	AT	AT	TT	AT	AA	AT	AT	AT
GG	AG	GG	GG	GG	GG	GG	GG	GG	AG	AG	GG	GG
CT	CC	TT	CT	CC	--	CT	TT	CT	CC	CT	TT	CT
CT	TT	CT	CT	TT	CT	CT	CC	CT	TT	CT	CC	CT
CT	CC	CT	CT	CT	CT	CT	TT	CT	CT	CT	TT	CT
AG	GG	AG	AG	AG	AG	AG	AG	AG	AG	AG	AA	AG
CC	CC	CC	CC	CG	CC	CC	CC	CC	CC	CG	CG	CG
CG	GG	--	CG	GG	CG	CG	CG	CG	CG	CG	CG	GG
GG	GG	GG	GG	GT	GG	GG	GG	GG	GG	GT	--	GT
CG	CC	CC	CG	CG	CC	CG	CC	CG	CG	CG	CC	CG
AG	AA	GG	--	AA	--	AA	AG	AA	AG	AA	GG	AG
AC	AC	AA	AC	AC	AA	AC	AA	AC	AC	AC	AA	AC
AT	AA	AA	AT	AT	AA	AT	AA	AT	AT	AT	AA	AT
AA	AG	GG	AA	AA	AG	AG	GG	AA	AG	AA	AG	AG
TT	AT	AA	TT	TT	AT	AT	AA	TT	AT	TT	--	AT
AA	AG	GG	AG	AA	AG	AG	GG	AA	AG	AA	AG	AG
AC	AC	CC	--	AC	CC	CC	CC	AC	CC	--	AC	CC
TT	CT	CC	CT	CT	CC	CT	CC	TT	CT	TT	CT	TT
GG	GT	TT	GT	GT	TT	GT	TT	GG	GT	GG	GT	GG
GT	GT	TT	GT	GT	GT	GT	TT	GG	GT	GG	GT	GG
CT	CT	CC	CT	CT	CT	CT	CC	TT	CT	TT	CT	TT
CG	CG	CC	CG	CG	CG	CG	CC	GG	CG	GG	CG	GG
AG	AG	GG	AG	AG	AG	AG	GG	AA	AG	AA	AG	AA
CT	CT	CT	CT	CT	CT	CT	CC	TT	CT	TT	CT	TT
GT	GT	GT	GT	GT	GT	GT	TT	GG	GT	GG	GT	GG
CT	CT	CT	CT	CT	CT	CT	TT	CC	CT	CC	CT	CC
CG	CG	CG	--	CG	CG	CG	GG	CC	CG	CC	CG	CC
AG	AG	AG	AG	AG	AG	AG	GG	AA	AG	AA	AG	AA
AC	AC	AC	AC	AC	AC	AC	AA	CC	AC	CC	--	CC
CT	CT	CT	CT	CT	CT	CT	TT	CC	CT	--	CT	CC
AG	GG	GG	--	AG	AG	AG	GG	AG	AG	AG	GG	AG
AC	AA	AC	AC	AC	AC	AC	AA	CC	AC	CC	AC	CC
CT	CC	CT	CT	CT	CT	CT	CC	TT	CT	TT	CT	TT
AG	GG	AG	AG	AG	AG	AG	GG	AA	AG	AA	AG	AA
CG	GG	CG	CG	CG	CG	CG	GG	CC	CG	CC	GG	CC
GT	GG	GT	GT	GT	GT	GT	GG	TT	GT	TT	GT	TT
CT	CC	CT	CT	CT	CT	CT	CC	TT	CT	TT	CT	TT
CT	TT	TT	TT	CT	CT	CT	TT	CT	CT	CT	TT	CT
AG	AG	GG	GG	AG	AG	AG	AG	GG	AG	GG	GG	GG
CG	CC	CG	CG	CG	CG	CG	CC	GG	CC	GG	CG	GG
AA	AA	AT	AT	AA	AA	AA	AA	AT	AA	AT	AT	AT
AG	AA	AA	AA	AG	AG	AG	AA	AG	AA	AG	AA	AG
AC	CC	CC	CC	AC	AC	AC	CC	AC	CC	AC	CC	AC

CT	TT	TT	TT	CT	CT	CT	TT	CT	TT	CT	TT	CT
TT	TT	CT	CT	CT	TT	TT	TT	CT	TT	CT	CT	CT
GT	GG	AG	AG	AT	GT	GT	GG	AT	GG	AT	AG	AT
AT	AA	AA	AA	AT	AT	AT	AA	AT	AA	AT	AT	AT
AG	GG	AG	AG	AA	AG	AG	GG	AA	GG	AA	AG	AA
AC	CC	CC	AC	AA	AC	AC	AC	AA	CC	AC	--	AA
AG	AA	AA	AA	AG	AG	AG	AA	AG	AA	AG	AA	AG
AG	GG	GG	GG	AG	AG	AG	AG	AA	GG	AG	--	AA
AG	AA	AA	AA	AG	--	AG	AG	GG	AG	AG	AG	GG
AA	GG	GG	--	AG	AG	AG	AG	AA	AG	AG	--	AA
TT	GG	GG	GG	GT	GT	GT	GT	TT	GT	--	GT	TT
AG	GG	GG	GG	GG	GG	AG	AG	AG	AG	GG	AG	AG
TT	CC	--	CT	CT	CT	TT	CT	TT	CT	CT	CT	TT
GG	CC	CC	CG	CG	CG	GG	CC	GG	CG	CG	CC	GG
CT	TT	TT	TT	CT	CT	CT	TT	CT	TT	CT	TT	CT
CT	CT	TT	CT	TT	TT	CT	TT	TT	CT	CT	TT	CT
CT	CT	CT	CT	CC	CC	CT	CC	CT	CT	CT	CT	CC
GG	GG	GG	GG	GT	GT	GT	GT	GT	GG	GT	GG	GT
AG	AG	AG	AG	AG	AG	AA	AG	AA	AG	AA	AG	AG
AG	AG	AA	AG	AG	AG	AG	AG	GG	AG	GG	--	AG
AG	AG	AG	AA	AA	AA	AG	AA	AG	AG	--	AG	AA
GT	TT	GT	GT	GT	GT	TT	GT	TT	GT	TT	TT	GT
AG	AG	AG	AG	AA	AA	AG	AA	AG	AG	AG	AG	AA
CT	CT	CT	CT	CC	CC	CC	CC	CT	CT	CC	CT	CT
AA	AA	--	--	AG	AG	AG	AG	AA	AA	AG	AG	AA
AG	AA	AG	AG	AG	AG	AG	AG	AA	AG	GG	AG	AG
AG	AA	AG	AG	AG	AG	AG	AG	AA	AG	GG	AG	AG
CG	CC	--	CG	CG	CG	GG	CG	CC	CG	GG	CG	CG
CG	CC	CG	CG	CG	--	GG	CG	CC	CC	GG	--	CG
AG	AA	AG	AG	AG	GG	GG	AG	AA	AA	AG	AG	AG
GG	AG	--	GG	AG	GG	GG	AG	AG	AG	--	AG	GG
CT	CC	CT	CT	CT	TT	TT	CT	CC	CC	CT	TT	CT
CC	AC	CC	CC	AC	CC	CC	CC	AC	AC	AC	AC	CC
--	CG	CC	CC	CG	CC	CC	CC	CG	CG	CG	CG	CC
CC	AC	CC	CC	AC	CC	CC	CC	AC	AC	AC	AC	CC
AA	CC	AC	AC	AC	AA	AA	AA	AC	AA	AC	AC	CC
AG	AG	AG	AG	AA	AA	AA	AA	AG	AA	AA	AA	AG
AA	GG	AG	AG	AG	AA	AA	AA	AG	AA	AG	AG	GG
GG	AG	AG	AG	GG	GG	GG	GG	AG	GG	GG	GG	AG
CC	TT	CT	CT	CT	CC	CC	CC	CT	CC	CT	CT	TT
CC	TT	CT	CT	CT	CC	CT	CT	CT	CC	CT	CT	TT
AA	AG	AA	--	AG	AA	AG	AA	AA	AA	AG	AG	AG
CC	--	CC	CC	CT	CC	CT	CC	CC	CC	CT	CC	CT
AG	AA	AG	AA	AG	GG	AG	AG	AG	AA	AG	--	AG
CC	AC	AC	AC	AC	CC	CC	AC	CC	AC	AC	AC	AC
CT	CT	CC	CT	CC	CC	CT	CC	CT	CT	CC	CC	CC
AG	GG	AG	AG	AG	AA	AG	AG	--	GG	AA	AA	AG

AG	AG	AA	AG	AA	AA	AG	AA	AA	AG	AA	AA	AA
AG	GG	AG	AG	AG	AA	AG	AA	AA	GG	AA	AG	AG
AC	AA	AC	AC	AC	AC	AC	CC	CC	AA	CC	CC	AC
AG	AA	AG	AG	AA	AG	AG	GG	GG	AA	GG	GG	AG
GT	GG	GT	GT	GG	--	GT	GT	GT	GG	TT	TT	GT
AG	AG	AA	AA	AG	AG	AG	AA	AG	AG	AA	AA	AG
CC	CC	TT	TT	CC	CT	CC	CT	CT	CC	TT	CT	CT
CT	CT	CC	CT	CT	CT	CT	CC	CT	CT	CT	CC	CT
AA	AG	GG	AG	AA	AG	AG	AG	AG	AA	AG	AG	AG
CG	CG	GG	GG	CG	GG	GG	CG	GG	CG	GG	CG	GG
CT	CT	TT	TT	CT	TT	TT	CT	TT	CT	TT	--	TT
GG	AG	AA	--	GG	AA	AG	AG	AG	GG	AG	AG	AG
GG	AG	AA	AG	GG	AA	AG	AG	AG	GG	AG	AG	GG
GG	CG	CC	CG	GG	--	CG	CG	CG	GG	CG	CG	GG
GG	AG	--	AG	GG	AA	AG	AG	AG	GG	AG	AG	GG
GG	AA	--	AG	AG	AG	AG	AG	AA	AG	AG	AG	AA
TT	CC	TT	CT	CT	CT	CT	CT	CC	CT	CC	CT	CT
AG	AA	GG	AG	AG	AG	AG	AG	--	AG	AA	--	AG
CC	GG	CC	CG	CG	CG	CG	CG	GG	CG	GG	CG	CG
CC	AA	CC	AC	AC	AC	AC	AC	AA	AC	AA	AC	AC
--	GG	AA	AG	AG	AG	AG	AG	GG	AG	GG	GG	AG
GG	TT	GG	GT	GT	GT	GT	GT	TT	GT	TT	GT	GT
GG	AA	GG	AA	AG	AG	AG	AG	AA	AG	AA	AG	AG
GG	AA	GG	AA	AG	AG	AG	AG	AA	AG	AA	AG	AG
AA	GG	AA	GG	AG	AG	AG	AG	GG	AG	GG	AG	AG
AA	CC	AA	CC	AC	AC	AC	AC	CC	AC	CC	AC	AC
AA	GG	AA	GG	AG	AG	AG	AG	GG	AG	GG	AA	AG
AA	GG	AA	GG	AG	AG	AG	AG	GG	AG	GG	AA	AG
CT	CC	TT	CC	CT	CT	CT	CT	CC	CT	--	TT	CT
GG	CC	GG	CC	CG	CG	CG	CG	CC	CG	--	GG	CG
AC	CC	AA	CC	AC	CC	AC	AC	CC	AC	CC	AA	AC
TT	CC	TT	CC	CT	CT	CT	CT	CC	CT	CC	TT	CT
TT	GG	TT	GG	GT	GT	GT	GT	GG	GT	GG	TT	GT
--	TT	CC	TT	CT	CT	CT	CT	TT	CT	TT	--	CT
CC	GG	CC	GG	CG	CG	CG	CG	GG	CG	GG	CC	CG
--	AA	TT	AA	AT	AT	AT	AT	AA	AT	AA	--	AT
CC	TT	CC	TT	CT	CT	CT	CT	TT	CT	TT	CC	CT
GG	AA	GG	AA	AG	AG	AG	AG	AA	AG	AA	GG	AG
AA	GG	AA	GG	AG	AG	AG	AG	GG	AG	GG	--	AG
AA	TT	AA	TT	AT	AT	AT	AT	TT	AT	TT	--	AT
GG	AA	GG	AA	AG	AG	AG	AG	AA	AG	AA	GG	AG
AA	GG	AA	GG	AG	AG	AG	AG	GG	AG	GG	AA	AG
CC	GG	CC	GG	CG	CG	CG	CG	GG	CG	GG	CC	CG
AA	GG	AA	GG	AG	AG	AG	AG	GG	AG	GG	AA	AG
GG	CC	GG	CC	CG	CG	CG	CG	CC	CG	CC	GG	CG
GG	AA	GG	AA	AG	AG	AG	AG	--	AG	AA	GG	AG
AA	CC	AA	CC	AC	AC	AC	AC	CC	AC	CC	AA	AC

GG	AA	GG	AA	AG	AG	AG	AG	AA	AG	AA	GG	AG
--	GG	CC	GG	CG	CG	CG	CG	GG	CG	GG	--	CG
CC	TT	CC	--	CT	CT	CT	CT	TT	CT	TT	CC	CT
TT	AA	TT	--	AT	AT	AT	AT	AA	AT	AA	TT	AT
CC	TT	--	TT	CC	CT	CT	CT	TT	CT	TT	CC	CT
--	TT	AA	TT	AT	AT	AT	AT	TT	AT	TT	AA	AT
AT	AA	TT	AA	AT	AT	AT	AT	AA	AT	AA	TT	AT
GG	CC	GG	CC	CG	CG	CG	CG	CC	CG	CC	GG	CG
CC	TT	CC	TT	CT	CT	CT	CT	TT	CT	TT	CC	CT
AA	GG	AA	GG	AG	AG	AG	AG	GG	AG	GG	AA	AG
AA	CC	AA	CC	AC	AC	AC	AC	CC	AC	CC	AA	AC
TT	GG	TT	GG	GT	GT	GT	GT	GG	GT	GG	TT	GT
CC	TT	CC	TT	CT	CT	CT	CT	TT	CT	TT	CC	CT
GG	CC	GG	CC	CG	CG	CG	CG	CC	CG	CC	GG	CG
TT	GG	TT	GG	GT	GT	GT	GT	GG	GT	GG	TT	GT
TT	CC	TT	CC	CT	CT	CT	CT	CC	CT	CC	TT	CT
AA	GG	AA	GG	AG	AG	AG	AG	GG	AG	GG	AA	AG
AA	CC	AA	CC	AC	AC	AC	AC	CC	AC	CC	AA	AC
CC	TT	CC	TT	CT	CT	CT	CT	TT	CT	TT	CC	CT
--	CC	AA	CC	AC	AC	AC	AC	CC	AC	CC	AA	AC
AA	GG	AA	GG	AG	AG	AG	AG	GG	AG	GG	AA	AG
GG	TT	GG	TT	GT	GT	GT	GT	TT	GT	TT	--	GT
GG	TT	GT	TT	GT	GT	GT	GT	TT	GT	TT	GG	GT
AG	AA	AG	AA	AG	AG	AG	AG	AA	AG	AA	GG	AG
CC	TT	CT	TT	CT	CT	CT	CT	TT	CT	TT	CC	CT
CC	TT	CT	TT	CT	CT	CT	CT	TT	CT	TT	CC	CT
AA	GG	AG	GG	AG	AG	AG	AG	GG	AG	GG	AA	AG
CC	TT	CT	TT	CT	CT	CT	CT	TT	CT	TT	CC	CT
TT	CC	CT	CC	CT	CT	CT	CT	CC	CT	CC	TT	CT
GG	AA	AG	AA	AG	AG	AG	AG	AA	AG	AA	GG	AG
CC	TT	CT	TT	CT	CT	CT	CT	TT	CT	TT	CC	CT
TT	CT	CT	CT	CT	TT	CT	CT	CT	CT	TT	TT	TT
CC	TT	CT	TT	CT	CT	CT	CT	TT	CT	TT	CC	CT
AA	GG	AG	AG	AG	GG	AG	AG	GG	AG	GG	AA	AG
AA	CC	AC	AC	AC	CC	AC	AC	CC	AC	CC	AA	AC
CG	CC	CG	CC	CG	CC	CG	CG	CC	CG	CC	CG	CC
GG	TT	GT	GT	GT	TT	GT	GT	TT	GT	TT	GG	GT
GG	AA	GG	--	AG	AA	AG	AG	AA	AG	AG	AA	AG
TT	CC	TT	CT	CT	CC	CC	CT	CC	CT	CT	CT	CT
AG	GG	AG	GG	AG	GG	GG	GG	GG	AG	GG	AG	GG
--	AA	GG	AG	AG	--	AA	AA	AA	AG	AG	AG	AG
GG	CC	GG	CG	CG	--	CC	CC	CC	CG	CG	CG	CG
CT	TT	--	TT	CT	TT	TT	TT	--	CT	TT	CT	TT
TT	GG	TT	GT	GT	GG	GG	GG	GG	GT	GT	GT	GT
GG	AG	--	AG	GG	AG	GG	GG	GG	GG	GG	AG	AG
AG	AG	GG	GG	AG	AG	GG	GG	GG	GG	GG	GG	GG
AC	CC	AC	AC	AC	CC	AA	AA	AA	AA	AC	AC	AC

AT	AA	AA	AT	AT	AA	TT	TT	AT	TT	AT	AT	AT
AT	AA	AA	AT	AT	AA	TT	TT	AT	TT	AT	AA	AT
AT	TT	TT	AT	AT	TT	AA	AA	AT	AA	AT	TT	AT
CT	CC	CT	CT	CT	CC	TT	TT	CT	TT	CT	CC	CT
AG	AG	AG	AG	GG	AA	GG	GG	AG	AG	AG	AA	AG
AC	AC	AC	AC	AA	CC	AA	AC	CC	AC	AC	CC	AC
CT	CT	CT	CT	TT	CC	TT	CT	CC	CT	CT	CT	CT
AG	AG	GG	AG	GG	--	AG	AG	AA	AG	AG	--	AG
GT	GT	GG	GG	GG	TT	GT	GT	TT	GT	GT	GT	GT
AG	GG	GG	GG	AG	AA	AG	AG	AA	AG	GG	AG	AG
CC	CC	CC	CC	CG	CG	CC	CG	CG	CG	CC	CG	CG
GG	GG	GG	GG	AG	AG	GG	AG	AG	AG	GG	AG	AG
CG	CC	CC	CC	CG	CG	CG	CG	GG	CG	CC	CG	CG
AG	AA	AA	AA	AG	AG	AG	AG	GG	AG	AA	--	AG
AG	GG	GG	GG	AG	AG	AG	AG	AA	AA	GG	--	AG
AG	AA	AG	AA	AG	AG	AG	AG	GG	GG	AA	AG	AG
GG	GG	GG	GG	GT	GT	GG	GT	GT	GT	GG	GG	GT
GG	GG	AG	GG	AA	AG	AG	AG	AA	AA	GG	AG	AG
TT	TT	TT	TT	GT	GT	TT	GT	GT	GT	TT	GT	TT
GG	GG	CG	GG	CC	CG	CG	CG	CC	CC	GG	GG	--
CT	CT	CT	--	CT	TT	TT	CT	TT	CT	CT	TT	CT
CG	--	CC	GG	CG	--	CG	CC	CG	CC	CC	GG	CC
CC	CC	CG	CC	CC	CC	CG	CG	CC	CG	CG	--	CG
AA	AC	AC	AA	AC	AA	AA	AC	AA	AC	AC	AC	AC
CC	CT	CT	CC	CT	CC	CC	CT	CC	CT	CC	CT	CC
AA	AG	AG	AA	AG	AA	AG	AG	AA	AA	AA	AA	AA
CC	CT	CT	CC	CT	CC	CT	CC	CC	CC	CC	CC	CC
GG	GG	CG	CG	GG	CG	CG	CG	GG	CG	CG	CG	GG
CC	CT	CT	CT	CT	CC	CT	CC	CC	CC	CC	CC	CC
AA	AG	GG	GG	AG	AG	GG	AG	AA	AG	AG	AG	AA
GG	GT	GT	GT	GG	GG	GT	GG	GG	GG	GG	GG	GG
GT	GT	GG	GG	TT	GT	GG	GT	TT	GT	GT	GT	TT
GG	CG	CG	CG	GG	GG	CG	GG	GG	GG	GG	GG	GG
GG	CG	CC	CC	GG	CG	CC	CG	GG	CG	CG	CG	GG
GG	AG	AG	AG	GG	GG	AG	GG	GG	GG	GG	GG	GG
TT	CT	CC	--	TT	CT	CC	CT	TT	CT	CT	CT	TT
GG	GT	TT	TT	GG	GT	TT	GT	GG	GT	GT	GT	GG
GG	AG	AG	AG	GG	GG	AG	GG	GG	GG	GG	GG	GG
AA	AG	GG	GG	AG	AG	GG	AG	AA	AG	AG	AG	AA
TT	CT	CC	CC	CT	CT	CC	CT	TT	CT	CT	CT	TT
TT	AT	AT	AT	TT	TT	AT	TT	TT	TT	TT	TT	TT
TT	GT	GT	GT	TT	TT	GT	TT	TT	TT	TT	TT	TT
--	CT	TT	TT	CT	CT	TT	CT	CC	CT	CT	CT	CC
AA	AG	AG	AG	AA	AA	AG	AA	AA	AA	AA	AA	AA
TT	AT	AA	AA	AT	AT	AA	AT	TT	AT	AT	--	TT
AA	AG	AG	AG	AA	AA	AG	AA	AA	AA	AA	AA	AA
TT	CT	CT	CT	TT	TT	CT	TT	TT	TT	TT	TT	TT

TT	AT	AT	AT	TT	TT	AT	TT	TT	TT	TT	TT	TT
GG	GT	TT	TT	GT	GT	TT	GT	GG	GG	GT	GT	GG
AA	AA	GG	GG	AG	AG	GG	GG	AG	AG	AG	AG	AA
CC	CC	TT	TT	CT	CT	TT	TT	CT	CT	TT	CT	CC
CC	CC	TT	TT	CT	CT	TT	TT	CT	CT	TT	CT	CC
CC	CC	TT	TT	CT	--	TT	TT	--	CT	TT	--	CC
GG	GG	TT	TT	GT	GT	TT	TT	GT	GT	TT	GT	GG
CC	CC	GG	GG	CG	CG	GG	GG	CG	CC	GG	CG	CC
CC	CC	AA	AA	AC	AC	AA	AC	AC	CC	AC	AC	CC
AA	AA	GG	GG	AG	AG	GG	AG	AG	AA	AG	AG	AA
GG	GG	AA	AG	AG	AG	AA	AG	AG	GG	AG	GG	GG
CC	TT	CT	CT	CC	CC	TT	CT	CT	CT	CT	CT	CT
AC	AA	AC	AC	CC	CC	AA	AC	AC	AC	AC	AC	AC
GT	TT	TT	TT	GT	GT	TT	TT	GT	TT	TT	TT	GT
CG	GG	GG	--	CG	CG	GG	CG	GG	CG	CG	GG	GG
AG	GG	GG	AG	AG	AA	AG	GG	AG	AG	AG	AA	GG
CT	TT	TT	CT	TT	TT	TT	TT	TT	CT	CT	CT	TT
GT	TT	TT	TT	GT	GT	GT	TT	TT	TT	TT	--	TT
CT	CC	CC	CT	CC	CC	CC	CC	CC	CT	CT	CT	CC
AC	AA	AA	AA	AC	AC	AC	AA	AA	AA	AA	--	AA
GG	AA	AA	AG	AA	AG	AG	AA	AA	AG	AG	GG	AA
AG	AA	AA	AA	AA	AG	AG	AA	AA	AG	AA	AG	AA
--	TT	TT	GT	TT	GT	GT	TT	TT	GT	GT	--	TT
TT	CC	--	CT	CC	CC	CT	CC	CC	CT	CT	TT	CC
CC	AA	AA	AC	AA	AA	AC	AA	AA	AA	AC	CC	AA
AA	TT	TT	AT	TT	TT	TT	TT	AT	TT	AA	AA	AT
GG	GT	GT	GT	GT	GT	GT	GT	GG	GT	GG	GT	GG
AA	AG	AG	--	AG	AG	AG	AG	AG	AG	AA	AA	AG
--	CC	CC	CG	CC	CC	CC	CC	CG	CC	GG	CG	CG
GG	AA	AA	AG	AA	AA	AA	AA	--	AA	GG	AG	AG
CG	GG	--	CG	CG	CG	CC	CG	CG	CG	CG	--	CG
AG	GG	AG	AG	AG	AG	AA	AG	AG	AG	AG	AG	AG
AA	AG	AA	AA	AG	AA	AG	AA	AG	AG	AA	AA	AA
AA	AT	AT	AT	AA	AT	AA	AT	AT	AA	AT	AT	AT
AG	AG	AA	--	AG	AA	AG	AA	AG	AG	AG	AA	AA
AG	AA	AG	AG	AA	AG	AA	AG	AA	AA	AA	AG	AG
AA	CC	AC	AC	CC	AC	AC	AC	CC	AC	CC	AC	AC
CC	AC	AC	AC	AC	AC	CC	AC	AC	CC	AC	--	AC
CC	AA	AC	--	AA	--	AC	AC	AA	AC	AA	AC	CC
GG	TT	GT	GT	TT	GG	GT	GT	TT	GT	TT	GG	GG
AA	TT	--	--	TT	AA	AT	AT	--	TT	TT	AA	AA
AA	AG	AG	AG	AG	AG	AG	AG	AG	AG	AG	AA	AA
CC	AC	--	AC	AC	CC	CC	AC	CC	AC	AC	CC	CC
AA	TT	AT	AT	TT	TT	TT	AT	TT	TT	TT	AA	AA
CC	TT	CT	CC	TT	TT	TT	CT	TT	TT	TT	CC	CC
AG	AA	AG	GG	AA	AA	AA	AG	AA	AA	AA	GG	GG
CT	TT	CT	--	TT	TT	TT	CT	TT	TT	TT	CC	CC

CT	CC	CT	--	CC	CC	CC	CT	CC	CC	CC	--	TT
AG	AA	AG	GG	AA	AA	AA	AG	AA	AA	AA	GG	GG
CT	TT	CT	CC	TT	TT	TT	CT	TT	TT	TT	CC	CC
CT	CC	CT	TT	CC	--	CC	CT	CC	CC	CC	--	TT
CT	TT	CT	--	TT	TT	TT	CT	TT	TT	TT	--	CC
AG	AA	AG	GG	AA	AA	AA	AG	AA	AA	AA	GG	GG
CT	CC	--	TT	CC	CC	CC	CT	CC	CC	CC	TT	TT
AG	AA	AG	GG	AA	--	AA	AG	--	AA	--	GG	GG
CG	GG	CG	--	GG	GG	GG	CG	GG	GG	GG	CC	CC
AC	CC	AC	AA	CC	CC	CC	AC	CC	CC	CC	AA	AA
CT	TT	CT	CT	TT	TT	TT	CT	TT	TT	TT	CC	CC
CT	TT	--	CT	TT	TT	TT	CT	TT	TT	TT	CT	CC
AT	AA	--	--	AA	AA	AA	AT	AA	AA	AA	TT	TT
CT	TT	CT	CT	TT	TT	TT	CT	TT	TT	TT	CC	CC
AC	CC	AC	AC	CC	CC	CC	AC	CC	CC	CC	AA	AA
GT	GG	GT	GT	GG	GG	GG	GT	GG	GG	GG	TT	TT
CT	CC	CT	CT	CC	CC	CC	CT	CC	CC	CC	--	TT
CG	CC	CG	CG	CC	CC	CC	CG	CC	CC	CC	GG	GG
CG	CC	CG	CG	CC	--	CC	CG	CC	CC	CC	CG	GG
AC	AA	--	AC	AA	AA	AA	AC	AA	AA	AA	CC	CC
CG	CC	CG	CG	CC	CC	CG	CG	CC	CC	CC	GG	GG
TT	TT	AA	AT	AT	AT	AA	AA	AT	TT	--	--	TT
GG	GG	AA	AG	AG	AG	AA	AA	AG	AG	GG	AG	AG
GG	GG	AA	AG	AG	AG	AG	AA	--	AG	GG	AG	AG
AA	AG	GG	--	AG	AG	AG	AG	AA	AG	AA	AG	AG
GG	AG	AA	AG	AG	AG	AG	AG	GG	AG	GG	AG	AG
CC	AC	AA	AC	AC	AC	AC	AC	CC	AC	CC	AC	AC
GG	AG	AA	AG	AG	AG	AG	AG	GG	AG	GG	AA	AG
GG	AG	AA	AG	AG	AG	AG	AG	GG	AG	GG	GG	AG
TT	GT	GG	GT	GT	--	GT	GT	TT	GT	TT	TT	GT
GG	GT	TT	GT	GT	GT	GT	GT	GG	GT	GG	GG	GT
AA	AC	CC	AC	AC	AC	AC	AC	AA	AC	AA	AA	AC
CC	CT	TT	CT	CT	CT	CT	CT	CC	CT	CC	CC	CT
CC	CT	TT	CT	CT	CT	CT	CT	CC	CT	CC	CC	CT
TT	GT	GG	GT	GT	GT	GT	GT	TT	GT	TT	TT	GT
TT	AT	AA	AT	AT	AT	AT	AT	TT	AT	TT	TT	AT
CC	CG	GG	CG	CG	CG	CG	CG	CC	CG	CC	CC	CG
CC	AC	AA	AC	AC	AC	AC	AC	CC	AC	CC	CC	AC
TT	CT	CC	CT	CT	CT	CT	CT	TT	CT	TT	TT	CT
--	CG	GG	CG	CG	CG	CG	CG	CC	CG	CC	CC	CG
TT	CT	CC	CT	CT	CT	CT	CT	TT	CT	TT	TT	CT
TT	AT	AA	AT	AT	AT	AT	AT	TT	AT	TT	TT	AT
CC	CT	TT	CT	CT	CT	CT	CT	CC	CT	CC	CC	CT
AA	AG	GG	--	AG	AG	AG	AG	AA	AG	AA	AA	AG
TT	AT	AA	AT	AT	AT	AT	AT	TT	AT	TT	TT	AT
TT	AT	AA	AT	AT	AT	AT	AT	TT	AT	TT	TT	AT
--	AC	AA	AC	AC	AC	AC	AC	CC	AC	CC	CC	AC

GG	GT	TT	GT	GT	GT	GT	GT	GG	GT	GG	GG	GT
AA	AG	GG	AG	AG	AG	AG	AG	AA	AG	AA	--	AG
TT	GT	GG	GT	GT	GT	GT	GT	TT	GT	TT	--	GT
GG	AG	AA	AG	AG	AG	AG	AG	GG	AG	GG	GG	AG
CC	CT	--	CT	CT	CT	CT	CT	CC	CT	CT	CC	CT
GG	AG	AA	AG	AG	AG	AG	AG	GG	AG	GG	GG	AG
CC	CT	TT	CT	CT	CT	CT	CT	CC	CT	CC	CC	CT
--	AC	CC	AC	AC	AC	AC	AC	AA	AC	AA	AA	AC
GG	AG	AA	AG	AG	AG	AG	AG	GG	AG	GG	GG	AG
CC	CT	TT	CT	CT	CT	CT	CT	CC	CT	CC	CC	CT
AG	AG	GG	AG	AG	AG	AG	AG	AA	AA	AA	AA	AG
CC	CT	CC	CT	CT	--	CT	CT	TT	TT	TT	TT	CT
GG	GT	GG	GG	GT	GG	GT	GG	GT	GT	--	GT	GT
GG	GG	GG	AG	GG	AG	AG	AG	AG	AG	AG	AG	GG
--	AT	TT	AT	AT	AT	AA	AT	AA	AT	AA	--	AT
TT	AT	AT	AT	AT	AT	AT	AT	AA	AT	AA	AA	AT
CC	AA	AC	AC	CC	AC	AC	AC	AA	AC	AA	AA	AC
GG	TT	GT	GT	GG	GT	GT	GT	TT	GT	TT	TT	GT
TT	TT	TT	--	TT	TT	CT	CT	CT	TT	TT	CT	CT
AG	GG	GG	AA	GG	GG	AA	AG	AA	AG	GG	--	AA
GG	CG	CG	GG	CG	CG	GG	CG	GG	GG	CG	CG	GG
TT	CT	CT	TT	CT	CT	TT	CT	TT	TT	CT	CT	TT
GT	GT	GT	GG	GT	GT	GG	GT	GG	GT	GT	GG	GG
AG	GG	GG	AA	GG	AG	AA	GG	AA	AG	GG	AG	AG
AA	AC	AC	AA	AC	AC	AA	AA	AA	AA	AC	AA	AC
GG	CG	CG	GG	CG	CG	GG	GG	GG	GG	CG	CG	CG
TT	GT	GT	TT	GT	GT	TT	TT	TT	TT	GT	GT	GT
CT	CT	TT	CC	TT	CC	CC	CT	CC	CT	TT	CT	CT
AA	AA	AG	AG	AG	AG	AG	GG	AG	GG	AG	AA	AA
CT	CT	CT	--	CT	CC	CC	CC	CT	CC	CT	CT	--
CC	CG	CG	--	CC	--	CG	CG	--	CG	CC	CC	--
AC	AC	AA	AC	AA	AC	AC	AA	AC	AA	AA	AA	AC
AG	GG	AG	GG	AA	GG	GG	AG	AG	AG	AA	AG	AG
TT	TT	CT	TT	CT	TT	TT	CT	TT	CT	CT	TT	TT
CG	GG	GG	GG	CG	GG	GG	GG	CG	GG	CG	CG	CG
AG	AA	--	AA	GG	AA	AA	AG	AG	AG	GG	AG	AG
GG	CG	CG	CG	GG	CG	CG	CG	GG	CG	GG	GG	GG
CG	CC	CG	CC	GG	CC	CC	CG	CG	CG	GG	CC	CG
CT	CC	CT	CC	TT	CC	CC	CT	CT	CT	TT	--	CT
AG	GG	AG	GG	AA	GG	GG	AG	AG	AG	AA	AG	AG
AG	AG	GG	AG	GG	AG	AG	GG	AG	GG	GG	AG	AG
CT	CC	CT	CC	TT	CC	CC	CT	CT	CT	TT	CT	CT
GG	GG	AG	GG	AG	GG	GG	AG	GG	AG	AG	GG	GG
AG	GG	AG	GG	AA	GG	GG	AG	AG	AG	AA	AG	AG
AC	CC	AC	AC	AA	CC	CC	AC	AC	AC	AA	AC	AC
AT	TT	TT	AT	AT	TT	TT	TT	AT	TT	AT	AT	AT
AG	AA	AG	--	GG	AA	AA	AG	AG	AG	--	AG	AG

AC	AA	AC	AC	CC	AA	AA	AC	AC	AC	CC	AC	AC
AG	AA	AG	AG	GG	AA	AA	AG	AG	AG	GG	AG	AG
AA	AA	AG	AA	AG	AA	AA	AG	AA	AG	AG	AA	AA
CT	CC	CC	CT	CT	CC	CC	CC	CT	CC	CC	CT	CT
CG	CC	CC	CG	CG	CC	CC	CC	CG	CC	CC	CG	CG
CT	TT	TT	CT	CT	TT	TT	TT	CT	TT	--	CT	CT
GG	CG	CG	GG	GG	GG	CG	CG	GG	--	CG	GG	--
CT	CC	CC	CT	CC	CT	CC	CC	CT	CC	CC	CT	CT
GG	GT	GT	GG	GT	GG	GT	GT	GG	GG	GT	GG	GG

1-37	1-39	1-40	1-43	1-45	1-46	1-47	1-48	1-55	1-56	1-62	1-63	1-65
AC	CC	AC	AC	AC	CC	CC	AC	CC	CC	CC	CC	CC
GG	GG	AG	AG	AG	GG	GG	AG	GG	GG	GG	GG	GG
--	CC	CT	CT	CT	CT	CC	TT	CC	CC	CC	CC	CC
--	AG	AG	GG	--	AG	AG	GG	AG	AG	AG	AG	AG
--	GG	GG	AG	AA	AG	GG	AA	GG	GG	GG	AG	AG
CG	GG	GG	CG	CG	CG	GG	GG	GG	GG	GG	CG	CG
--	CC	CC	TT	TT	CT	CC	CT	CC	CC	CC	--	CT
GT	GG	GG	TT	TT	GT	GG	GT	GG	GG	GT	GT	GT
TT	GG	GG	TT	TT	GT	GG	GT	GG	GT	GT	GT	GT
TT	GG	GG	TT	TT	--	GG	TT	GG	GT	GT	GT	GT
AA	GG	GG	--	AA	AG	GG	AG	AG	AG	AG	AG	AG
CT	--	CC	CT	CT	CT	CC	CC	CT	CT	--	CT	CT
AC	CC	CC	AA	AA	CC	CC	--	AC	AC	CC	AC	AC
AA	AA	AA	AG	AG	AA	AA	AA	AG	AG	AA	AG	AG
CT	CC	CC	CT	CT	CC	CC	CT	CC	CC	CT	CC	CC
AG	AA	AG	GG	AG	AA	AA	AG	AG	AG	AG	AG	AG
TT	TT	AT	AT	TT	TT	TT	TT	AT	AT	TT	AT	AT
CT	CC	CC	--	--	CC	CC	CT	CC	--	CT	CC	CC
TT	TT	GT	GT	TT	TT	TT	TT	GT	GT	GT	GT	GT
CC	CC	CG	CG	CC	CC	CC	CC	CG	CG	CG	CG	CG
AA	TT	AT	AA	TT	TT	TT	--	AT	AT	AA	AT	AT
CC	--	CT	CC	CT	TT	TT	CC	TT	CT	CC	CT	CT
TT	AA	AT	--	AT	AA	AA	TT	AA	AT	TT	AT	AT
TT	AA	AT	TT	AT	AA	AA	TT	AA	AT	TT	AT	AT
GG	AA	AG	GG	AG	AA	AA	GG	AA	AG	GG	AG	AG
GT	TT	GT	GT	GT	TT	TT	--	TT	GT	GT	GT	GT
GG	TT	GT	GG	GT	TT	TT	GG	TT	GT	GG	GT	GT
AA	TT	AT	AA	AT	TT	TT	AA	TT	AT	AA	AT	AT
CC	TT	CT	CC	CT	TT	TT	CC	TT	CT	CC	CT	CT
GG	CC	CG	GG	CG	CC	CC	GG	CC	CG	GG	CG	CG
TT	CC	CT	TT	CT	CC	CC	TT	CC	CT	TT	CT	CT
CC	AA	AC	--	AC	AA	AA	CC	--	--	CC	AC	AC
GG	AA	AG	GG	AG	AA	AA	GG	AA	AG	GG	AG	AG
TT	GG	GT	TT	GT	GG	GG	TT	GG	GT	TT	GT	GT
GG	AA	AG	--	AG	AA	AA	GG	AA	AG	GG	AG	AG
GG	TT	GT	GG	GT	TT	TT	GG	TT	GT	GG	GT	GT
AA	GG	AG	AA	AG	GG	GG	AA	GG	AG	AA	--	AG
TT	--	CT	TT	CT	CC	CC	--	CC	CT	TT	CT	CT
TT	AA	AT	TT	AT	AA	AA	TT	AA	AT	TT	AT	AT
GG	AA	AG	GG	--	AA	AA	GG	AA	AG	GG	AG	AA
CC	AA	AC	CC	AC	AA	AA	CC	AA	AC	CC	AC	AC
--	AA	AG	GG	AG	AA	AA	--	AA	AG	GG	AG	AG

CC	TT	TT	CC	CT	TT	TT	CC	TT	CT	CC	CT	CT
GG	--	CG	GG	--	CC	CC	GG	CC	CG	GG	CG	CG
CC	TT	CT	CC	TT	CT	TT	CC	TT	--	CT	CT	CT
TT	CC	CC	TT	--	CT	CC	TT	CC	CT	CT	CC	CC
CC	AA	AA	CC	AA	AC	AC	CC	AA	AC	AC	AA	AA
TT	CT	CC	TT	CC	CT	CT	TT	CC	CT	CT	CC	CC
CC	CT	TT	CT	TT	CT	CT	CC	TT	CT	CC	TT	TT
GG	AG	AG	AG	--	AA	GG	AA	AA	GG	AG	AA	AA
GG	GG	GT	GT	GT	TT	GG	TT	TT	GG	TT	TT	TT
--	GG	AG	AG	AG	AA	GG	--	AA	GG	AA	AA	AA
TT	TT	CT	CT	CT	CC	TT	CC	CT	TT	CC	CT	CT
--	CC	CG	--	CG	GG	CC	GG	CG	--	GG	CG	CG
AG	AA	AG	AG	AG	GG	AA	GG	AG	--	GG	AG	--
--	CC	AC	AC	CC	AA	CC	AA	AA	CC	AC	AC	--
AG	AG	GG	AG	AG	GG	AG	GG	GG	AG	GG	GG	GG
CT	TT	CC	CT	TT	CT	CT	CC	CC	TT	CT	CT	CT
CT	--	TT	CT	CC	CT	CT	TT	TT	CC	CT	CT	CT
CT	TT	CC	CT	TT	CT	CT	CC	CC	--	CC	CT	CT
AG	AA	GG	AG	AA	AG	AG	GG	GG	AA	GG	AG	AG
AA	AA	CC	AC	AA	AC	AC	CC	CC	AA	AC	AC	AC
AA	AA	GG	AG	AA	AG	AG	GG	GG	AA	AG	AG	AG
GG	GG	CC	CG	GG	CC	CG	CC	CG	GG	GG	CC	CC
CC	CC	GG	CG	CC	GG	CG	GG	CG	CG	CC	GG	GG
AA	AA	GG	GG	AA	GG	AG	GG	AG	AG	AA	GG	GG
AC	CC	AA	AC	CC	AA	AC	AA	AC	AC	CC	AA	AA
CT	CT	CT	CC	TT	--	CT	CC	--	CT	TT	CC	CC
--	CT	--	TT	CC	TT	CT	TT	CT	CT	CT	TT	TT
--	AT	TT	AA	TT	AA	AT	AA	TT	AT	TT	AA	AA
AG	AG	GG	AG	--	AG	GG	GG	GG	GG	GG	AG	AG
TT	AT	TT	AT	--	AT	AT	AT	TT	AT	AT	AT	AT
--	GT	GG	GT	GT	TT	TT	GT	GT	GG	GT	GT	GT
AG	AG	AG	AG	--	GG	GG	AG	AG	AG	GG	GG	GG
CT	CT	CT	CT	CT	TT	TT	CT	CT	CT	TT	TT	TT
GG	AG	AG	AG	--	GG	GG	AG	AG	AG	GG	GG	GG
GT	GT	TT	GT	GT	GT	GG	TT	GT	TT	GT	GT	GT
GT	GT	GT	GT	GG	--	GG	GT	GT	GT	GG	GG	GG
CT	TT	CC	CT	--	CT	TT	CC	CT	--	CT	CT	CT
CT	TT	--	--	--	CT	TT	CC	CT	CC	CT	CT	CT
AG	AA	GG	AG	AA	AG	AA	GG	AG	GG	AG	AG	AG
GG	GG	CC	--	GG	CG	GG	CC	CG	CC	--	CG	CG
AG	AA	GG	AG	AA	AG	AA	GG	AG	GG	AA	AG	AG
TT	--	TT	CT	CC	CT	CC	TT	CT	TT	CC	CT	CT
CT	CC	TT	CT	CC	CT	CC	TT	TT	TT	CC	CT	CT
CG	CC	CG	CG	CC	CG	CC	CG	CG	CG	CC	CC	CC
AT	--	AT	AT	TT	AT	TT	AT	AT	AT	TT	TT	TT
GG	GG	GT	GG	GG	GG	GG	GT	GT	GT	GG	GT	GT
CC	CC	CT	CC	CC	CC	CC	CT	CT	CT	CC	CT	CT

CT	CC	TT	--	--	CT	CC	--	TT	TT	CC	TT	TT
GG	GG	GT	GT	GG	GG	GG	GG	GT	GT	GG	GT	GT
CT	CC	TT	TT	CC	CT	CC	TT	TT	TT	CC	TT	TT
TT	TT	GT	GT	TT	TT	TT	GT	GT	GT	TT	GT	GT
--	GG	CG	CG	GG	GG	GG	CG	CG	CG	GG	CG	CG
AG	AG	AG	AG	GG	--	AG	GG	AG	AG	AA	AG	AG
AG	AA	AG	AG	AG	AG	AG	AA	AG	AG	GG	AG	AG
GG	AA	AA	AG	AG	AG	AG	AA	AG	AG	GG	AG	AG
CC	TT	TT	CC	CT	CT	CT	TT	CT	TT	CC	CT	CT
AA	GG	AG	AA	AG	--	AG	GG	AG	GG	AA	AG	AG
GT	GG	GT	TT	GT	GG	--	GT	GT	GG	TT	GT	GT
GG	AG	GG	GG	GG	AG	GG	GG	GG	AG	GG	AG	AG
CT	TT	CT	CC	CT	TT	CT	CT	CT	CT	CT	CT	CT
CC	CC	CC	AC	CC	CC	CC	--	CC	CC	CC	AC	AC
CT	CT	CT	CC	CT	CT	CT	CT	CT	CT	CT	CC	CC
GT	--	GT	TT	GT	GG	GT	GT	GT	GG	GG	GT	GT
CC	CC	CT	CT	CT	CC	CT	CT	CT	CC	CC	CC	CC
GG	GG	GG	CG	GG	GG	GG	GG	GG	GG	GG	CG	CG
CC	CC	CC	AC	CC	CC	CC	CC	CC	CC	CC	AC	AC
CT	TT	CT	CT	CT	TT	CT	CT	CT	TT	TT	TT	TT
--	AA	AG	AG	--	AA	AG	AG	AG	AA	AA	AA	AA
--	AA	AG	GG	AG	AA	AG	AG	AG	AA	AA	AG	AG
CT	CT	CT	CC	CT	CT	CT	CT	CT	CT	CT	CC	CC
AA	AA	AA	AT	AA	AA	AA	AA	AA	AA	AA	AA	AA
CC	CC	CC	CT	CC	CC	CC	CC	CC	CC	CC	CC	CC
--	AA	AA	AG	--	AA	AA	AA	AA	AA	AA	AG	AG
--	CC	CT	CT	CC	CC	CT	CT	CT	CC	CC	CC	CC
TT	TT	AT	AA	TT	TT	AT	AT	AT	TT	TT	AT	AT
TT	--	AT	AA	TT	TT	AT	AT	AT	TT	TT	AT	AT
CT	TT	CT	CC	TT	TT	CT	CT	CT	TT	TT	CT	CT
CC	CC	CC	CT	CC	CC	CC	CC	CC	CC	CC	CT	CT
GT	GG	GT	GT	GG	GG	GT	GG	GT	--	GG	GG	GG
CT	CC	CT	CT	CC	CC	CT	CT	CT	CC	CC	CC	CC
AA	AG	AA	--	AG	AG	AA	AA	AA	AG	AG	AG	AG
AG	GG	AG	AA	GG	GG	--	--	AG	GG	GG	AG	AG
AG	AA	AG	AG	AA	AA	AG	AG	AG	AA	AA	AA	AA
CT	CC	CT	TT	CC	CC	CT	CT	CT	CC	CC	CT	CT
AC	CC	AC	AC	CC	CC	AC	AC	AC	--	CC	CC	CC
AT	TT	AT	AA	TT	TT	AT	AT	AT	TT	TT	AT	AT
AA	--	AA	AG	AA	AA	AA	AA	AA	AA	AA	AG	AG
AC	AA	AC	AC	AA	AA	AC	AC	AC	AA	AA	AA	AA
AT	TT	AT	AT	TT	TT	AT	AT	AT	TT	TT	TT	TT
AA	AC	AA	AC	AC	AC	AA	AA	AA	AC	AC	CC	CC
--	CC	CC	AC	CC	CC	CC	--	CC	CC	CC	AC	AC
--	AG	GG	GG	AG	AG	GG	--	GG	AG	AG	AG	AG
AC	AC	AC	CC	AC	AC	AC	AC	AC	AC	AC	CC	CC
--	AA	AG	AG	AA	AA	AG	AG	AG	AA	AA	AA	AA

CC	CC	CC	CG	CC	CC	CC	CC	CC	CC	CC	CG	CG
CT	TT	CT	CC	TT	TT	CT	CT	CT	TT	TT	CT	CT
CT	CC	CT	TT	CC	CC	CT	CT	CT	CC	--	CT	CT
--	CC	CT	CT	CC	CC	CT	--	CT	CC	CC	CT	CT
AG	AA	AG	AG	AA	AA	AG	AG	AG	AA	AA	AG	AG
--	CC	CT	TT	CC	CC	CT	CT	CT	CC	CC	TT	TT
CT	TT	--	CT	TT	CT	CT	CT	CT	TT	TT	CT	CT
AT	AA	AT	TT	AA	AT	AT	--	AT	AA	AA	TT	TT
GG	GG	GG	AG	GG	GG	GG	GG	GG	GG	GG	AG	AG
--	AA	AC	AC	AA	AC	AC	AC	AC	AA	AA	AC	AC
CT	CC	CT	CT	CC	CT	CT	--	CT	CC	CC	CT	CT
TT	TT	TT	GT	TT	TT	TT	TT	TT	TT	TT	GT	GT
CT	CT	CT	CT	--	CT	CT	CT	CT	TT	TT	CT	CT
CC	CC	CC	CC	AC	CC	CC	CC	CC	AC	AC	CC	CC
AA	AG	AG	AG	AA	AG	AG	AG	AG	AA	--	AG	AG
GT	GT	GT	GT	GG	--	GT	GT	GT	--	GG	GT	GT
TT	TT	TT	TT	TT	TT	TT	TT	TT	AT	AT	TT	TT
AG	AG	AG	AG	AA	AG	AG	AG	AG	AA	AA	AG	AG
CC	CC	CC	CT	CC	CC	CC	CC	CC	CC	CC	CT	CT
CT	CT	CT	CT	CC	CT	CT	CT	CT	CC	CC	CT	CT
AA	--	AC	AC	AA	AC	AC	AC	AC	AA	AA	AC	AC
CC	CT	CT	CT	CC	CT	CT	CT	CT	CC	CC	CT	CT
AA	AT	AT	TT	AA	AT	AT	AT	AT	AA	AA	TT	TT
GG	AG	AG	AA	GG	AG	AG	AG	AG	GG	GG	AA	AA
GG	GG	GG	CG	GG	GG	GG	--	GG	GG	GG	CG	CG
--	CT	CT	--	--	CT	CT	CT	CT	TT	TT	CT	CT
GG	AG	AG	AG	GG	AG	AG	AG	GG	GG	GG	AG	AG
AA	AG	AG	AG	AA	AG	AG	AG	AA	AG	AA	AG	AG
AA	AA	AA	AG	AA	AA	AA	AA	AA	AA	AA	AA	AA
GG	GG	GG	AG	GG	GG	GG	GG	GG	GG	GG	GG	GG
TT	CT	TT	CT	TT	--	TT	TT	TT	CT	CT	CT	CT
GT	GT	GT	--	--	GG	GG	GT	GG	GT	GT	GT	GT
TT	TT	TT	GT	TT	TT	TT	--	TT	TT	TT	TT	TT
AG	AG	GG	AG	GG	GG	GG	AG	GG	AG	AG	AG	AG
CT	CT	CC	CC	CC	CC	CC	CT	CC	CT	CT	CT	CT
GT	GT	TT	TT	TT	TT	TT	GT	TT	TT	GT	GT	GT
AG	AG	AG	GG	AG	AG	AG	AG	AG	AG	AG	AG	AG
GG	AA	AA	GG	AG	GG	AG	AA	AA	AA	AG	AA	AA
CC	CT	CT	CC	CC	CC	CT	CC	CT	CT	CT	CT	CT
AT	AA	AA	--	--	AT	AT	AT	AA	AT	AA	AA	AA
GG	CC	CC	CG	CG	GG	CG	CG	CC	CG	CC	CC	CC
AC	AA	AA	--	AA	AC	AA	AA	AA	AC	AA	AA	AA
GG	AA	AA	AG	AG	AG	AA	AA	AA	GG	AA	AA	AA
GG	AG	AG	GG	AG	AG	AG	--	AG	GG	AG	AG	AG
CC	CT	CT	CC	CT	CT	CT	CC	CT	CC	CT	CT	CT
AA	GG	GG	AG	AG	GG	GG	AG	GG	AA	GG	GG	GG
CT	CC	CC	CC	CT	CC	CC	CC	CC	CT	CC	CC	CC

AG	GG	GG	AG	GG	GG	GG	AG	AG	AG	GG	GG	GG
AA	AG	AG	AA	GG	GG	AG	AA	AA	AA	AG	AG	AG
--	CC	CC	CT	CT	CT	CC	CT	CT	CT	CC	CC	CC
AA	--	AA	AT	AT	AT	AA	AT	AT	AT	AA	AA	AA
AA	AA	AA	AC	AC	AC	AA	AC	AC	AC	AA	AA	AA
--	TT	TT	CT	CT	CT	TT	CT	CT	CT	TT	TT	TT
GG	GG	GG	--	AG	AG	GG	GG	AG	AG	--	GG	GG
CC	CC	CC	CC	TT	CT	CC	--	CT	CT	CC	CC	CC
--	TT	TT	TT	CT	CT	TT	--	TT	TT	TT	CT	CT
AA	AA	AA	AA	GG	AG	AA	AG	AG	AG	AA	GG	GG
AA	AG	GG	AG	GG	AA	AG	AG	AG	AG	GG	GG	GG
CT	CT	TT	CT	TT	CC	CT	CT	CT	CT	TT	TT	TT
GG	AG	AG	GG	AG	GG	GG	GG	AG	GG	AG	AG	AG
CT	CT	CT	TT	TT	CC	CT	CT	CT	CT	CT	TT	TT
CG	GG	GG	CG	CG	GG	CG	CG	GG	CG	GG	CG	CG
CT	CT	CT	CT	TT	CC	CT	CT	CT	CT	CT	CT	CT
CC	CT	CT	CT	CT	CC	CC	--	CT	--	CT	CT	CT
GG	AG	AG	AA	AG	--	AG	AG	AG	AG	AG	AG	--
--	CT	CT	TT	CT	CC	CT	TT	CT	CT	CT	CT	CT
AA	GG	AG	GG	AG	AA	AA	AG	AG	AG	AG	AG	AG
CC	TT	CT	TT	CT	CC	CC	CT	CT	CT	CT	CT	CT
GG	AA	AG	AA	AG	GG	--	AG	AG	AG	AG	AG	AG
AG	AG	AG	AG	GG	GG	GG	GG	AG	GG	AG	AG	AG
GT	GG	GT	GG	GT	TT	TT	--	GT	GT	GT	GT	GT
CG	CG	CG	CG	CC	--	CC	CC	CG	CC	CG	CG	--
--	CC	--	CC	CC	CG	CG	CC	CG	CC	CG	CG	CG
CT	TT	CC	TT	CT	CC	CT	CT	CT	TT	CT	CT	CT
CT	CC	TT	CC	CT	TT	CT	CT	CT	CC	CT	CT	CT
--	TT	AA	AT	AT	AA	AT	TT	AT	TT	AA	AT	AT
AG	AA	GG	AG	AG	GG	AG	AA	AG	AA	GG	AG	AG
CT	TT	CC	CT	CT	CC	CT	TT	CT	TT	CC	CT	CT
TT	CT	TT	TT	CT	TT	CT	CT	TT	CT	TT	CT	CT
AA	CC	AA	AC	CC	AA	AC	CC	AC	CC	AA	--	AC
--	TT	GG	GG	TT	GT	GT	GG	GT	GG	TT	TT	TT
GG	AA	GG	GG	AA	AG	AG	GG	AG	GG	AA	AA	AA
CC	TT	CC	CC	TT	CT	CT	CC	CT	CC	TT	TT	TT
TT	AA	TT	TT	--	AT	AT	TT	AT	TT	AA	AA	AA
AA	CC	AA	AA	CC	AC	AC	AA	AC	AA	--	CC	CC
CC	CG	CC	CC	CG	CC	CC	CC	CG	CC	CG	CG	CG
AA	AG	AA	AA	AG	AA	AA	AA	AG	AA	AG	AG	AG
--	CC	TT	TT	--	CT	CT	TT	CT	TT	CC	CC	CC
TT	CC	TT	TT	CC	--	CT	TT	CT	TT	CC	CC	CC
GG	AA	GG	GG	AA	AG	AG	GG	AG	GG	AA	AA	AA
--	CT	CC	CC	CT	CT	CT	CC	CC	CC	CT	CT	CT
GG	AA	GG	GG	AA	AG	AG	GG	AG	GG	AA	AA	AA
AA	GG	AA	--	--	AG	AG	AA	AG	AA	GG	GG	GG
TT	GG	TT	TT	GG	GT	GT	TT	GT	TT	GG	GG	GG

CC	TT	CC	CT	TT	CT	CT	CC	CT	CC	TT	TT	TT
CC	GG	CC	CG	GG	CG	CG	CC	CG	CC	GG	GG	GG
GG	TT	GG	GT	TT	GT	GT	GG	GT	GG	TT	TT	TT
--	AA	GG	AG	--	AG	AG	GG	AG	GG	AA	AA	AA
TT	CC	TT	CT	CC	CT	CT	TT	CT	TT	CC	CC	CC
TT	GT	TT	GT	GT	GT	GT	TT	TT	TT	GT	GT	GT
CC	GG	CC	CG	GG	CG	CG	CC	CG	CC	GG	GG	GG
--	--	GG	AG	AA	AG	AG	GG	AG	GG	AA	AA	AA
GG	AG	GG	AG	AG	AG	AG	GG	GG	GG	AG	AG	AG
CC	AA	CC	AC	AA	AC	AC	CC	AC	CC	AA	AA	AA
CC	CG	CC	CG	CG	CG	CG	CC	CC	CC	CG	CG	CG
AA	GG	AA	AG	--	AG	AG	AA	AG	AA	GG	GG	GG
CC	AA	CC	AC	--	AC	AC	CC	AC	CC	AA	AA	AA
GG	TT	GG	GT	TT	GT	GT	GG	GT	GG	TT	TT	TT
AA	CC	AA	AC	CC	AC	AC	AA	AC	AA	CC	CC	CC
CC	CT	CC	CT	CT	CT	CT	CC	CC	CC	CT	CT	CT
GG	TT	GG	GT	TT	GT	GT	GG	GT	GG	TT	TT	TT
GG	AG	GG	GG	AG	GG	GG	GG	AG	GG	AG	AG	AG
TT	CC	TT	CT	CC	CT	CT	TT	CT	TT	CC	CC	CC
CC	--	CC	CT	CT	CT	CT	CC	CC	CC	CT	CT	CT
AT	AA	TT	AT	--	AT	AT	TT	AT	TT	AA	AA	AA
CG	CC	GG	CG	CC	CG	CG	GG	CG	GG	CC	CC	CC
GT	GT	TT	GT	GG	GT	GT	TT	GT	TT	GG	GG	GG
AG	GG	GG	GG	AG	GG	GG	GG	AG	GG	AG	AG	AG
GT	GT	TT	--	--	TT	--	GT	GT	--	--	GT	GT
CT	TT	CT	CT	CC	CT	CT	TT	CT	TT	CC	CT	CT
CC	AA	AC	AC	CC	AC	AC	AA	AC	AC	CC	AC	AC
--	CT	CT	CT	CT	CT	CT	CT	CC	CT	CC	CC	CC
GG	--	CG	CG	CG	CG	CG	CC	CG	CG	GG	CG	CG
CC	TT	CT	CT	CT	CT	CT	TT	CT	CT	CC	CT	CT
GT	TT	TT	GT	TT	TT	TT	TT	GT	TT	GT	GT	GT
CT	TT	CT	CT	--	TT	CT	CT	TT	CT	CT	TT	TT
AT	TT	TT	AT	TT	--	TT	TT	AT	TT	AT	AT	AT
CC	AC	AC	CC	AC	AC	AC	AC	CC	AC	CC	CC	CC
TT	CC	CT	--	CT	CC	CT	CT	CT	CT	TT	CT	CT
AA	AG	AG	AA	AG	AG	AG	AG	AA	AG	AA	AA	AA
GG	GG	AG	GG	AG	AG	AG	GG	GG	AG	GG	GG	GG
GG	CG	GG	--	CG	GG	GG	CG	CG	--	CG	GG	GG
--	TT	CC	CT	TT	CT	CT	TT	CT	TT	TT	CT	CT
CT	TT	CC	CT	TT	TT	CT	TT	TT	CT	TT	CT	CT
CT	CT	CC	CT	CT	TT	CT	CT	CT	CC	TT	CT	CT
TT	CT	TT	CT	CT	CC	TT	TT	CT	TT	CC	CC	CC
GT	GT	GG	GT	GT	TT	GG	GT	GT	GG	TT	TT	TT
CT	CT	TT	CT	CT	CC	TT	--	CT	TT	CC	CC	CC
AA	AT	TT	AT	AT	--	TT	AT	AT	TT	AA	AA	AA
--	AG	GG	AG	--	AA	GG	AG	AG	--	AA	AA	AA
CC	AC	AC	AC	AC	CC	AA	CC	AC	AA	CC	CC	CC

AA	--	AG	AG	AG	AA	GG	AA	AG	GG	AA	AA	AA
AA	AG	AG	AG	AA	AA	GG	--	AG	--	AA	AA	AA
TT	CT	CT	CT	TT	TT	CC	CT	CT	CC	TT	TT	TT
--	AG	AG	--	GG	GG	AA	AG	AG	--	GG	GG	GG
GG	GT	GT	GT	--	GG	--	GT	GT	TT	GG	GG	GG
TT	CT	CT	CT	--	TT	CC	CT	CT	CC	TT	TT	TT
--	AG	AG	AG	GG	GG	AA	AG	AG	AA	GG	GG	GG
GG	AG	AG	AG	GG	GG	AA	--	AG	AA	GG	GG	GG
--	AG	AG	AG	AA	AA	GG	GG	AG	--	AA	AA	AA
GG	CG	CG	CG	--	GG	CC	CG	CG	CC	GG	GG	GG
--	AG	AG	AG	AA	AA	GG	GG	AG	GG	AA	AA	AA
AA	AC	AC	--	AA	--	CC	AC	AC	CC	AA	AA	AA
GG	AG	AG	AG	GG	GG	AA	AG	AG	AA	GG	GG	GG
GG	AG	AG	AG	--	AG	AA	AG	AG	AA	GG	GG	GG
AA	AT	AT	AT	AA	AT	TT	AT	AT	TT	AA	AA	AA
TT	CT	CT	CT	TT	CT	CC	CT	CT	CC	TT	TT	TT
TT	CT	--	--	TT	CT	CC	CT	CT	--	TT	TT	TT
CC	CG	CG	--	CC	CG	GG	CG	CG	GG	CC	CC	CC
CC	CT	CT	CT	CC	CT	TT	CT	CT	TT	CC	CC	CC
TT	CT	CT	CT	TT	CT	CC	CT	CT	CC	TT	TT	TT
CC	CT	CT	CT	CC	CT	TT	CT	CC	TT	CC	--	CC
AA	AT	AT	AT	AA	AT	TT	AT	AA	TT	AA	AA	AA
TT	AT	AT	AT	TT	AT	AA	AT	TT	AA	TT	TT	TT
CC	CT	CT	CT	CC	CT	TT	--	CC	TT	CC	CC	CC
TT	CT	CT	CT	TT	CT	CC	CC	TT	CC	TT	TT	TT
TT	AT	AT	AT	TT	AT	AA	AT	TT	AA	TT	TT	TT
AA	AG	AG	AG	AA	AG	GG	AG	AA	--	AA	AA	AA
TT	GT	GT	GT	TT	GT	GG	--	TT	GG	TT	TT	TT
--	CT	CT	CC	CC	CT	--	CT	CC	TT	CC	CC	CC
GG	CG	CG	GG	GG	CG	CC	GG	GG	CC	GG	GG	GG
AA	AG	AG	AA	AA	AG	GG	AG	AA	GG	AA	AA	AA
CC	CT	CT	CC	--	CT	TT	CT	CC	TT	CC	CC	CC
CC	CT	CT	CC	CC	CT	TT	CT	CC	TT	CC	CC	CC
AA	AT	AT	AA	AA	--	TT	AT	AA	TT	AA	AT	AT
GG	AG	AG	GG	GG	AG	AA	--	GG	AA	GG	AG	AG
GG	GG	AG	GG	GG	AG	AA	AG	GG	AA	AG	AG	AG
GG	GG	GT	GG	GG	GT	TT	GT	GG	TT	GT	GT	GT
GG	GG	AG	GG	GG	AG	AA	AG	GG	AA	--	AG	AG
AG	GG	AG	AG	AG	--	GG	AG	AG	GG	GG	GG	--
TT	TT	TT	CT	CT	CT	CT	TT	TT	CT	TT	TT	TT
GG	AG	GG	AG	--	AG	AG	AG	GG	AA	AG	--	AG
GG	AG	GG	--	GG	GG	GG	AG	GG	GG	AG	AG	AG
GG	GG	GG	AG	AA	AG	AG	AG	GG	AG	AG	AG	AG
CC	CC	CC	CG	GG	GG	GG	CG	CC	CG	CG	CG	CG
TT	TT	TT	CT	CC	CC	CC	CT	TT	CT	CT	CT	--
--	AC	AC	CC	CC	CC	CC	AC	AC	CC	AC	AC	AC
AG	AG	AG	AG	AA	AA	AA	AA	AA	AG	AA	AG	AG

AC	AC	AC	CC	CC	CC	CC	AC	AC	CC	AC	AC	AC
CC	CC	CC	CC	AC	AC	AC	AC	AC	CC	CC	CC	CC
CT	CT	CT	TT	TT	TT	TT	CT	CT	TT	CT	CT	CT
GG	GG	GG	AA	AA	AA	AA	AA	AG	AG	GG	GG	GG
AA	AA	AA	GG	GG	GG	GG	AG	AG	AG	AA	AA	AA
CT	CC	CC	TT	TT	--	TT	--	CT	CT	CC	CC	CC
--	TT	TT	CT	CT	--	TT	CT	CT	TT	TT	TT	TT
--	GG	GG	CC	--	CC	CG	GG	CG	CG	GG	GG	GG
AG	GG	GG	AG	AA	AA	AG	--	AG	AG	GG	GG	GG
CT	CC	CC	CT	TT	CT	CC	CT	CT	CT	CC	CC	CC
AT	AA	AA	AT	AT	--	AA	AT	AT	AT	AA	AA	AA
--	AA	AA	AC	CC	CC	AA	AC	AC	AC	AA	AA	AA
GG	AG	GG	AG	--	--	AG	AG	AG	AG	AG	AG	AG
CC	CT	CC	--	CC	CC	CT	CT	CT	CT	CT	CC	CC
--	CT	CC	CT	CC	CC	CT	--	CT	CT	CT	CC	CC
GG	GT	GG	--	GG	GG	GT	GG	GT	--	GT	GT	GT
CT	CT	CT	CT	CC	TT	CC	CC	TT	CT	CT	CT	CT
AG	GG	GG	GG	AG	GG	AG	AG	GG	AG	GG	AG	AG
AG	AG	GG	AG	AA	GG	AA	AA	GG	--	AG	AG	AG
CG	CG	CG	--	--	CG	GG	GG	CG	CG	GG	CG	CG
AG	GG	GG	AG	AA	GG	AA	AA	GG	AG	AG	AG	AG
AG	AA	AA	AG	GG	AA	GG	AG	AA	AG	AG	GG	GG
TT	TT	TT	GT	GT	TT	GT	TT	TT	TT	GT	GT	GT
AG	AA	AA	AG	AG	AA	GG	AG	AA	AG	AG	GG	GG
AG	AG	AA	AG	AG	AA	GG	--	AA	AG	AG	GG	GG
GG	GT	GG	GT	GG	GG	GT	GG	GG	GG	GT	GT	GT
CT	--	CC	CT	CT	CC	TT	CT	CC	CT	CT	TT	TT
CT	CC	TT	CT	CT	TT	CC	CT	TT	CT	CT	CC	CC
CT	CT	CC	CC	CT	CC	CT	CT	CC	CT	CC	CT	CT
AG	GG	AG	AG	AG	AG	AG	AG	AG	AG	GG	GG	GG
CT	TT	CT	CT	CC	CT	CT	CT	CT	CT	TT	TT	TT
AG	GG	AG	AG	AG	AG	AG	AA	AG	AG	GG	GG	GG
CT	CT	CT	CT	CT	TT	CC	CT	CT	TT	TT	TT	TT
CG	CG	CG	CG	CG	CC	GG	CG	CG	--	--	CC	CC
--	CT	CT	CT	CT	TT	CC	CC	CT	--	TT	CT	CC
AC	AC	AA	AC	--	AC	AC	AC	CC	AC	AA	AC	AC
--	AG	GG	AG	AG	AG	AG	AG	AA	AG	AG	AG	AG
--	CT	TT	CT	TT	CT	CT	CC	CC	CT	CT	CT	CT
--	AG	AA	AG	AA	AG	AG	GG	GG	AA	AG	AG	--
--	AG	AA	AG	--	AG	AG	GG	GG	AA	AG	AG	AG
TT	CT	TT	TT	TT	CT	--	--	CT	TT	CT	TT	TT
GG	AA	GG	AG	--	AG	AG	AA	AA	GG	AG	GG	GG
AA	AG	AA	AG	AA	AA	AA	AG	--	AA	AA	AA	AA
TT	GT	TT	GT	TT	GT	TT	GT	GT	TT	TT	TT	TT
TT	GT	TT	GT	TT	TT	TT	TT	GT	TT	TT	TT	TT
AA	CC	AA	AC	AA	CC	AC	AC	CC	AA	AC	AA	AA
AA	--	AA	AG	--	GG	AG	AG	GG	AA	AG	AA	AA

CC	CG	CC	CG	--	GG	CG	CG	GG	CC	CG	CC	CC
--	CG	CC	CG	CC	GG	CG	CC	GG	CG	CG	CC	CC
CT	CT	TT	CT	TT	--	CT	CT	CC	CT	CT	TT	TT
CT	CT	CC	CT	CC	TT	CT	CC	TT	CT	TT	CT	CT
AT	AT	AA	AT	AA	TT	AT	--	TT	AT	TT	AT	AT
GG	AG	AG	GG	AG	GG	AG	AG	GG	GG	GG	GG	GG
CT	CT	CC	CT	CC	TT	CT	CC	CT	CT	TT	CT	CT
CC	--	TT	TT	TT	--	CT	CT	CT	CT	CC	CT	CT
--	CT	CC	CT	CC	TT	CT	CT	CT	CT	TT	CT	CT
AG	AG	--	AG	GG	AA	AG	AG	AG	AG	AA	AG	AG
CC	CC	CC	CC	--	CC	CG	CC	CG	CC	CG	CC	CC
CG	CG	CG	CG	CG	CG	GG	CG	GG	GG	CG	CG	CG
--	GG	GG	GT	GG	GG	GT	--	GG	GG	GT	GG	GG
CG	CG	CG	CC	CC	CG	CC	CG	CC	CC	CG	CG	CG
AA	AG	AA	AG	AG	AA	AG	AA	GG	AG	AG	AA	AA
AC	AC	AC	AA	AA	AC	AA	AC	AA	AA	AC	AC	AC
AT	AT	AT	AA	AA	AT	AA	AT	AA	AA	AT	AT	AT
AA	AG	AA	AG	AG	AA	AG	AA	GG	AG	AG	AA	AA
TT	TT	TT	AT	AA	TT	AT	AT	AA	AT	TT	AT	AT
AA	AA	AA	AG	GG	AA	AG	AG	GG	AG	AA	AG	AG
CC	AC	AC	AC	CC	AC	AC	CC	CC	AC	AC	AC	AC
TT	TT	TT	CT	CC	TT	CT	CC	CC	CT	TT	CT	CT
GG	GG	GG	GT	TT	GG	GT	TT	TT	GT	GG	GT	GT
GG	GG	GG	GT	GT	GG	GT	TT	TT	GT	GT	GT	GT
TT	TT	TT	CT	CT	TT	CT	CC	CC	CT	CT	CT	CT
GG	GG	GG	CG	CG	GG	CG	CC	CC	--	CG	CG	CG
AA	AA	AA	AG	AG	AA	AG	GG	GG	--	AG	AG	AG
TT	TT	--	CT	CT	TT	CT	CC	CC	CT	CT	CT	CT
GG	GG	GG	GT	GT	GG	GT	TT	TT	GT	GT	GT	GT
CC	CC	CC	CT	CT	CC	CT	TT	TT	CT	CT	CT	CT
CC	CC	CC	CG	CG	CC	CG	GG	GG	CG	CG	CG	CG
AA	AA	AA	AG	AG	AA	AG	--	GG	AG	AG	AG	AG
CC	CC	--	AC	AC	CC	AC	AA	AA	AC	AC	AC	AC
CC	CC	CC	CT	CT	--	CT	TT	--	CT	CT	CT	CT
AG	AG	AG	GG	GG	AG	GG	GG	GG	GG	GG	GG	GG
--	CC	CC	AC	AC	CC	AC	AA	AC	AA	AC	AC	AC
TT	TT	TT	CT	CT	TT	CT	CC	CT	CT	CT	CT	CT
AA	AA	AA	AG	AG	AA	AG	GG	AG	AG	AG	AG	AG
CC	CC	CC	CG	CG	CC	CG	--	CG	CG	CG	CG	CG
--	--	--	GT	GT	TT	--	--	GT	--	GT	GT	GT
TT	TT	TT	CT	CT	TT	CT	CC	CT	--	CT	CT	CT
CT	CT	CT	TT	TT	CT	TT	TT	TT	TT	TT	TT	TT
GG	GG	GG	GG	GG	GG	GG	AG	GG	GG	GG	GG	GG
GG	GG	CG	CC	CG	GG	CG	CC	CG	CG	CG	CG	CG
AT	AT	AT	AA	AT	AT	AT	AA	AT	AT	AT	AT	AT
AG	AG	AA	AA	AA	AG	AA	AA	AA	AA	AA	AA	AA
AC	AC	CC	CC	CC	AC	CC	CC	CC	CC	CC	CC	CC

CT	CT	TT	TT	TT	CT	TT	TT	TT	TT	TT	TT	TT
CT	CT	CT	TT	CT	CT	CT	TT	CT	CT	CT	CT	CT
AT	--	AG	GG	AG	--	AG	GG	AG	AG	AG	AG	AG
AT	AT	AA	AA	AA	AT	AA	AA	AA	AA	AA	AA	AA
AA	AA	AG	GG	GG	AG	AG	GG	AG	AG	AG	AG	AG
AA	AA	AC	CC	CC	AC	AC	CC	AC	AC	AC	AC	AC
AG	AG	AA	AA	AA	AG	AA	AA	AA	AA	AA	AA	AA
--	AA	AG	AG	GG	AG	AG	GG	GG	AG	--	AG	AG
GG	--	AG	AG	AA	GG	--	AA	AA	AG	AA	AG	--
AA	AA	AG	AG	AG	AA	AG	GG	GG	--	GG	AG	AG
--	TT	GT	GT	GT	TT	GT	GT	GG	GT	GG	GT	GT
AG	AG	AG	AG	AG	AG	GG	AG	GG	AG	GG	AG	AG
TT	TT	CC	CT	CT	--	CC	CT	CC	CT	CC	CT	CT
GG	GG	CC	CG	CC	GG	CC	CG	CC	CG	CC	CG	CG
CT	CT	TT	TT	TT	CT	TT	TT	TT	TT	TT	TT	TT
CT	CT	TT	CT	TT	CT	TT	CT	TT	CT	TT	CT	CT
CC	CC	CT	CC	CC	CC	CT	CC	CT	CT	CT	CC	CC
GT	GG	GG	GG	--	GG	GG	GT	GG	GT	--	GT	GT
AG	GG	AG	GG	AG	AG	AG	AG	AG	AA	AA	AG	AG
--	--	AG	AA	AG	AG	AG	GG	AG	GG	GG	AG	--
--	AA	AG	AA	AA	--	AG	--	AG	--	AG	AA	AA
GT	GG	GT	GT	GT	GT	GT	--	GT	TT	GT	GG	GG
AA	AA	AG	AA	--	AG	AG	AA	AG	AG	AG	AA	AA
CC	CC	CT	CC	CT	CT	CT	CC	CT	CT	CT	CC	CC
AG	AG	AA	--	--	AA	AA	AG	AA	AA	AA	AA	AA
AG	AG	AG	AG	AG	AA	AG	AG	AA	AG	AG	AG	AG
GG	AG	AG	GG	AG	AG	AA	--	AA	AG	AG	AG	AG
GG	CG	CG	GG	CG	CG	CC	GG	CC	CG	CG	CG	CG
GG	CG	CG	GG	--	CG	CC	CC	CC	GG	CG	CG	CG
GG	AG	AG	GG	AG	AG	AA	AG	AA	GG	AA	AG	AG
GG	--	GG	GG	AG	AG	AG	AG	AG	GG	AG	GG	GG
TT	CT	CT	TT	CT	CT	CC	CT	CC	TT	CC	CT	CT
--	AC	CC	CC	CC	AC	AC	AC	AC	CC	AC	CC	CC
CC	CG	CG	CC	CC	CG	CG	CG	CG	CC	CG	CG	CG
CC	AC	AC	CC	CC	AC	CC	AC	AC	CC	AC	AC	AC
--	CC	CC	AA	AA	AC	AC	AC	CC	AA	CC	CC	CC
AA	AA	AG	AA	AA	AA	AA	AA	AG	AA	AG	AG	AG
AG	GG	GG	AA	--	AG	AG	AA	GG	AA	AG	GG	GG
GG	AG	AG	GG	GG	GG	AG	GG	AG	GG	AG	AG	AG
TT	TT	TT	CC	CC	CT	CT	CT	TT	CC	CT	TT	TT
TT	--	TT	CC	CC	CT	CT	CT	CT	CC	CT	--	TT
AG	AG	AG	AA	AA	AG	AA	--	AG	AA	AA	AG	AG
CT	CC	CT	--	CC	CT	CC	CT	--	CC	CC	CT	CT
AG	AG	AG	GG	GG	AG	AA	AA	AG	GG	AG	AA	--
AC	CC	CC	CC	CC	CC	AC	AC	AC	CC	AC	AC	AC
CC	CT	CT	CC	CC	CT	CT	CT	CC	CC	CC	CT	CT
GG	AG	AG	AA	--	AG	AG	GG	AG	--	AA	GG	GG

AA	AG	AG	AA	AA	AG	AG	AG	AA	AA	AA	AG	AG
AG	AG	AG	AA	AA	AG	AG	GG	AG	AG	AA	AG	AG
AC	AC	AA	CC	CC	AA	AC	AA	AC	AC	CC	AC	AC
AG	AG	AA	GG	GG	AA	AG	AA	AG	GG	GG	AG	AG
GT	GT	GG	TT	TT	GG	GT	GG	GT	TT	TT	GT	GT
AG	AA	AG	AA	AA	AA	AA	AG	AG	AA	AG	AA	AA
CT	CT	CC	TT	TT	CT	CT	CC	CT	TT	CT	CT	CT
CT	CC	CT	CC	--	CC	CC	CT	CT	CC	CT	CC	CC
AG	AG	AA	GG	GG	AG	AG	AA	AG	GG	AG	AG	AG
GG	CG	CG	GG	GG	GG	CG	CG	GG	GG	GG	CG	CG
TT	CT	CT	--	TT	TT	CT	CT	TT	TT	TT	CT	CT
AG	AG	GG	AG	GG	AA	GG	GG	AG	AA	AG	AG	AG
AG	AG	GG	AG	GG	AA	GG	GG	AG	AA	AG	AG	AG
CG	CG	GG	CG	GG	CC	GG	GG	CG	CC	CG	CG	CG
GG	AG	GG	AG	--	--	GG	GG	AG	AA	AG	AG	AG
AA	AA	AG	GG	AA	AG	AG	AG	AG	AA	GG	GG	GG
CC	CC	CT	TT	CC	CT	CT	CT	CT	CC	TT	TT	TT
AA	--	AG	--	AA	AG	AG	GG	AG	AA	GG	GG	GG
GG	GG	CG	CC	GG	CG	CG	CG	CG	GG	CC	CC	CC
AA	AA	AC	CC	AA	AC	AC	AC	AC	AA	CC	CC	CC
GG	GG	AG	AA	GG	AG	AG	AG	AG	GG	AA	AA	AA
TT	TT	GT	GG	TT	GT	GT	GT	GT	TT	GG	GG	GG
AA	AA	AG	GG	AA	AG	AG	AG	AG	AA	GG	GG	GG
AA	AA	AG	GG	AA	AG	AG	AG	AG	AA	GG	GG	GG
GG	GG	AG	--	GG	AG	AG	AG	AG	--	AA	AA	AA
CC	--	AC	AA	CC	AC	AC	AC	AC	CC	AA	AA	AA
GG	GG	AG	AA	GG	AG	AG	AG	AG	GG	AA	AA	AA
GG	GG	AG	AA	GG	--	AG	AG	AG	GG	AA	AA	AA
CC	CC	CT	TT	CC	CT	CT	CT	CT	CC	TT	TT	TT
CC	CC	CG	GG	CC	CG	CG	--	CG	CC	GG	GG	GG
CC	CC	AC	AA	CC	AC	AC	AA	AC	CC	AA	AA	AA
CC	CC	CT	TT	CC	CT	CT	CT	CT	CC	TT	TT	TT
GG	GG	GT	TT	GT	GT	GT	GT	GT	GG	TT	TT	TT
--	TT	CT	CC	TT	CT	CT	CT	CT	TT	CC	CC	CC
CG	GG	CG	CC	GG	CG	CG	CG	CG	GG	CC	CC	CC
AT	AA	AT	TT	AA	AT	AT	TT	AT	AA	TT	TT	TT
--	TT	CT	CC	TT	CT	TT	CT	CT	TT	CC	CC	CC
--	AA	AG	--	AA	AG	AG	GG	AG	--	GG	GG	GG
AG	GG	AG	AA	GG	AG	GG	AG	AG	GG	AA	AA	AA
--	TT	AT	AA	TT	AT	TT	AT	AT	--	AA	AA	AA
AG	AA	AG	GG	--	AG	AA	AG	AG	AA	GG	GG	GG
--	GG	AG	AA	--	AG	--	--	AG	GG	AA	AA	AA
CG	GG	CG	CC	GG	CG	GG	--	CG	GG	CC	CC	CC
AG	GG	AG	AA	GG	AG	GG	AG	AG	GG	AA	AA	AA
--	CC	CG	GG	CC	CG	CC	CG	CG	CC	GG	GG	GG
AA	AA	AG	--	AA	AG	AA	--	AG	AA	--	GG	GG
AC	CC	AC	--	CC	AC	CC	CC	AC	CC	AA	AA	AA

AG	AA	AG	GG	AA	AG	AA	AG	AG	AA	AG	GG	GG
CG	GG	CG	CC	GG	CG	GG	--	CG	--	CG	CC	CC
--	TT	CT	CC	TT	CT	TT	CT	CT	TT	CT	CC	CC
AT	AA	AT	TT	AA	AT	AA	AT	AT	AA	AT	TT	TT
CT	TT	CT	CC	TT	CT	TT	CT	CT	TT	CT	CC	CC
AT	TT	AT	AA	TT	AT	TT	AT	AT	TT	AT	AA	AA
AT	AA	AT	TT	AA	AT	AA	AT	AT	AA	AT	TT	TT
CG	CC	CG	GG	CC	CG	CC	CG	CG	CC	CG	GG	--
CT	TT	CT	CC	TT	CT	TT	CT	CT	TT	CT	CC	CC
AG	GG	AG	AA	GG	AG	GG	AG	AG	GG	AG	AA	AA
AC	CC	AC	AA	CC	AC	CC	AC	AC	CC	AC	AA	AA
GT	GG	GT	TT	GG	GT	GG	GT	GT	GG	GT	TT	TT
CT	TT	CT	CC	TT	--	TT	CT	CT	TT	CT	--	CC
CG	CC	CG	GG	CC	CG	CC	CG	CG	CC	CG	GG	GG
GT	GG	GT	TT	GG	GT	GG	GT	GT	--	GT	TT	TT
CT	CC	CT	TT	CC	CT	CC	CT	CT	CC	CT	TT	TT
AG	GG	GG	AA	GG	AG	GG	AG	AG	GG	AG	AA	AA
--	CC	CC	AC	CC	AC	CC	CC	AC	CC	AC	AA	AA
CT	TT	TT	CT	TT	CT	--	CT	CT	TT	CT	CC	--
AC	CC	CC	AC	CC	AC	CC	AC	AC	CC	AC	AA	AA
AG	GG	GG	AG	GG	AG	GG	AG	AG	GG	AG	AA	AA
GT	TT	TT	GT	TT	GT	TT	GT	GT	TT	GT	GG	GG
GT	TT	TT	GT	TT	GT	TT	GT	GT	TT	GT	GG	GG
AG	AA	AA	AG	AA	AG	AA	AG	AG	AA	AG	GG	GG
CT	TT	TT	CT	TT	CT	TT	CT	CT	TT	CT	CC	CC
CT	TT	TT	CT	TT	CT	TT	CT	CT	TT	CT	CC	CC
AG	GG	GG	AG	GG	AG	GG	AG	AG	GG	AG	AA	AA
CT	TT	TT	CT	TT	CT	TT	CT	CT	TT	CT	CC	CC
CT	CC	CC	CT	CC	CT	CC	CT	CT	CC	CT	TT	TT
AG	AA	AA	AG	AA	AG	AA	AG	AG	AA	AG	GG	GG
CT	TT	TT	CT	TT	CT	TT	--	CT	TT	CT	CC	CC
TT	CT	CT	CT	TT	CT	CT	CT	TT	CT	CT	--	TT
CT	--	TT	CT	CT	CT	CT	TT	CT	TT	CT	CC	CC
AG	GG	GG	AG	AG	AG	AG	GG	AG	GG	AG	AA	AA
AC	CC	CC	--	AC	CC	AC	CC	AC	CC	AC	AA	AA
CC	CC	CC	--	CC	CC	CG	CC	CC	CC	CG	CG	CG
GT	TT	TT	GT	GT	GT	GG	TT	GT	TT	GT	GT	GT
--	AA	AG	AG	AG	AG	GG	AA	AG	AA	AG	AG	AG
TT	CC	CT	CT	CT	CT	TT	CC	CT	CC	CT	CT	CT
AG	GG	GG	AG	GG	GG	AG	GG	GG	GG	AG	AG	AG
GG	AG	AG	AG	AG	AG	GG	AA	AG	AA	AG	AG	AG
GG	CG	CG	CG	CG	CG	GG	CC	CG	CC	CG	CG	CG
CT	TT	TT	CT	--	TT	CT	CT	TT	TT	CT	CT	CT
--	GT	GT	GT	GT	GT	TT	TT	GT	GG	GT	GT	GT
GG	--	AG	GG	GG	GG	GG	AG	AG	AG	GG	GG	GG
GG	AG	AG	AG	GG	GG	AG	--	GG	GG	GG	AG	AG
--	AC	CC	AC	AA	--	AC	CC	AC	AC	AA	AC	AC

TT	AT	AA	TT	TT	TT	TT	AA	AT	AT	TT	TT	TT
TT	AT	AA	TT	TT	TT	TT	AA	AT	AT	TT	TT	TT
AT	AT	TT	--	AA	AA	AA	--	AT	AT	AA	AA	AA
CT	CT	CC	TT	TT	TT	TT	CC	CT	CT	TT	TT	TT
AG	AG	AA	GG	GG	GG	GG	AA	AG	AG	AG	GG	GG
AC	AC	CC	AA	--	AC	AA	CC	AC	AC	AC	AA	AA
TT	CT	CC	TT	TT	CT	TT	CC	CT	CT	CT	TT	TT
AA	AG	AA	GG	AG	--	GG	AA	AA	AG	AG	GG	--
GT	GG	TT	GG	GT	GT	GG	TT	TT	GT	GT	GG	GG
AG	GG	AA	GG	AG	AG	GG	AA	AA	AG	GG	GG	GG
CG	CC	CG	CC	CG	CG	CC	CG	CG	CG	CC	CC	CC
AG	GG	AG	GG	AG	AG	GG	AG	AG	AG	GG	GG	GG
CG	CC	GG	CG	CG	GG	CG	CG	CG	CG	CC	CG	CG
AG	AA	AG	AG	AG	GG	AG	AG	AG	GG	AA	AG	AG
AG	GG	AG	AG	AG	AA	AG	AG	AG	AA	GG	AG	AG
AG	AA	AG	AG	AG	GG	AG	AG	AG	GG	AA	AG	AG
GT	GG	GT	GG	GT	GT	GG	GT	GT	--	GG	GG	GG
AG	GG	AG	AG	AG	AA	AG	AG	AG	AA	--	AG	AG
TT	TT	GT	TT	GT	GT	TT	GT	GT	GT	TT	TT	TT
CC	CG	CG	CG	CG	CC	CG	--	CG	CC	GG	CG	CG
--	CT	CT	CT	TT	CT	TT	CT	TT	TT	CT	CT	CT
GG	CG	GG	CC	GG	CC	CG	CC	CG	GG	CG	CC	CC
CC	--	CC	CG	CC	CG	CG	CG	CG	CC	CC	CG	CG
AA	AC	AA	AC	AA	AA	AA	AC	AA	AA	AC	AC	AC
CC	CT	CC	CT	CC	CC	CC	CT	CC	CT	CT	CT	CT
AA	AG	AA	AG	AA	AA	AA	AG	AA	AG	AG	AG	AG
CC	CT	CC	--	CC	CC	CC	CT	CC	CT	CT	CT	CT
GG	GG	GG	CG	GG	GG	CG	GG	GG	CG	GG	CG	CG
CC	CT	CT	CT	CC	CC	CC	CT	CC	CT	CT	CT	CT
AG	AG	AG	GG	AG	AA	AG	AG	AA	GG	AG	GG	--
GG	GT	GT	GT	GT	GG	GG	GG	GG	GT	GT	GT	GT
GT	TT	GT	GG	GT	TT	GT	TT	TT	GG	GT	GG	GG
GG	GG	CG	CG	--	GG	GG	GG	GG	CG	CG	CG	CG
CG	GG	CG	CC	CG	GG	CG	GG	GG	CC	CG	CC	CC
GG	GG	AG	AG	AG	GG	GG	GG	GG	AG	AG	AG	AG
CT	TT	CT	CC	CT	TT	--	TT	TT	CC	CT	CC	CT
GT	GG	GT	TT	GT	GG	GT	GG	GG	TT	GT	TT	TT
GG	GG	AG	AG	AG	GG	GG	GG	GG	AG	AG	AG	AG
AG	AA	AG	--	AG	AA	AG	AA	AA	GG	AG	GG	GG
CT	TT	CT	CC	CT	TT	CT	TT	TT	CC	CT	CT	CT
--	TT	AT	AT	AT	TT	TT	TT	TT	AT	AT	TT	TT
--	TT	GT	GT	GT	GT	TT	TT	TT	--	GT	TT	TT
CT	--	CT	TT	CT	CT	CT	CC	CC	TT	CT	CT	CT
AA	AA	AG	AG	AG	AG	AA	AA	AA	AG	AG	AA	AA
AT	TT	AT	AA	--	AT	AT	TT	TT	AA	AT	AT	AT
AA	AA	AG	AG	AG	AG	AA	AA	AA	AG	AG	AA	AA
TT	TT	CT	CT	CT	CT	TT	TT	TT	CT	CT	TT	TT

TT	TT	AT	AT	AT	AT	TT	--	TT	AT	AT	TT	TT
GT	GG	GT	TT	GT	GT	--	GG	GG	TT	GT	GT	GT
AG	AA	AG	GG	AG	AG	AG	AA	AA	GG	AG	AG	AG
CT	CC	CT	TT	CT	CT	CT	CC	CC	TT	CT	CT	CT
TT	CC	CT	TT	CT	CT	CT	CC	CT	TT	CT	CT	CT
CT	CC	CC	TT	CT	CT	CT	CC	CT	TT	CT	CT	CT
TT	GT	GG	TT	GT	GT	GT	GG	GT	TT	GT	TT	TT
GG	CG	CC	GG	CG	CG	CG	CC	CG	GG	CG	GG	GG
--	AC	CC	AA	AC	AC	AC	CC	AC	AA	AC	AA	AA
AG	AG	AA	GG	AG	AG	AG	AA	AG	GG	AA	GG	GG
--	AG	GG	AA	AG	AG	--	GG	AG	AA	GG	AG	AG
CC	CC	TT	CC	CC	CC	TT	CC	CT	TT	TT	CT	CT
CC	CC	AA	--	CC	CC	AA	--	AC	AA	AA	AC	AC
GT	--	TT	GT	GT	GT	TT	GT	GT	TT	TT	TT	TT
CG	CG	GG	CG	GG	CG	GG	CG	GG	GG	GG	CG	CG
AA	AA	GG	AA	AG	AA	GG	AA	AG	GG	GG	AG	AG
CT	CT	TT	CT	TT	CT	TT	CT	TT	TT	TT	CT	CT
GT	TT	TT	TT	GT	GT	TT	GT	GT	GT	TT	TT	TT
CT	--	CC	CT	CC	CT	CC	CC	CT	CC	CC	CT	CT
AC	AC	AA	AA	AC	AC	AA	AC	AC	AC	AA	AA	AA
GG	GG	AA	AG	AG	GG	AG	AG	GG	AG	AA	AG	AG
AA	AG	AA	AA	AG	AG	AA	AG	AG	AG	AA	AA	AA
GG	--	TT	GT	GT	GG	GT	TT	GG	GG	TT	GT	GT
TT	TT	CT	CT	TT	CT	CT	--	CT	TT	CC	TT	TT
AC	CC	AC	AA	AC	AC	AC	AC	AC	CC	AC	AC	AC
AT	--	AT	AT	AT	--	AA	AT	AT	AT	AT	AT	AT
GT	GT	GG	GG	--	GT	GG	GG	GT	--	GG	GT	GT
AA	AA	AG	AG	AG	AA	AA	AG	AA	AA	AG	AA	AA
CG	CG	CG	CG	CG	CG	GG	GG	CG	CG	CG	CG	CG
AG	AG	AG	AG	AG	AG	GG	AG	AG	AG	AG	AG	AG
GG	GG	CC	CC	CG	CC	GG	CC	CC	--	CG	CG	CG
GG	AG	AA	AA	AG	AA	GG	AA	AA	AA	AG	AG	AG
AG	AG	AA	AA	AG	AA	AG	AA	AA	AA	AA	--	AG
AA	AA	AA	AA	AA	AA	AA	AA	AA	--	AT	AA	AA
AA	AG	AA	AA	--	AA	AG	AA	AA	AA	AA	AG	AG
AG	AA	AG	AG	AA	AG	AA	AG	AG	AG	AG	AA	AA
AC	AC	AC	AA	AC	AC	AC	AA	AA	AC	AC	AC	AC
--	CC	AC	CC	CC	AC	CC	CC	CC	AC	CC	CC	CC
CC	AC	AC	AC	AC	AC	AC	CC	CC	AC	AC	AA	AA
--	GT	GT	GT	GT	GT	GT	GG	GG	GT	GT	TT	TT
--	AT	AT	AT	AT	AT	AT	--	AA	AT	AT	TT	TT
AA	AA	AG	AA	AA	AG	AA	AA	AA	AG	AA	AG	AG
CC	CC	AC	CC	CC	AC	CC	CC	CC	AC	CC	AC	AC
AA	AA	AT	AT	AT	AT	AT	AA	AA	TT	AT	TT	TT
CC	CC	CT	CT	CT	CC	CT	CC	CC	CT	TT	TT	--
GG	GG	GG	AG	AG	GG	AG	GG	GG	AG	AA	AA	AA
CC	CC	CC	CT	CT	CC	CT	CC	CC	CT	TT	TT	TT

TT	TT	TT	CT	CT	TT	CT	TT	TT	CT	CC	CC	CC
GG	GG	GG	AG	AG	GG	AG	GG	GG	AG	AA	AA	AA
CC	CC	CC	CT	CT	CC	CT	CC	CC	CT	TT	TT	TT
TT	--	TT	CT	--	TT	CT	TT	TT	CT	CC	CC	CC
CC	CC	CC	CT	CT	CC	CT	CC	CC	CT	TT	TT	TT
--	GG	GG	AG	AG	GG	AG	GG	GG	AG	AA	AA	AA
--	TT	TT	CT	CT	TT	--	TT	TT	CT	CC	CC	CC
GG	GG	GG	AG	AG	GG	AG	GG	GG	AG	AA	AA	AA
CC	CC	CC	CG	CG	CC	CG	CC	CC	CG	GG	GG	GG
AA	AA	AA	AC	AC	AA	AC	--	AA	AC	CC	CC	CC
CC	CC	CC	CT	CT	CC	CT	CC	CC	CT	TT	CT	CT
CC	CC	CC	CT	CT	CC	CT	--	CC	CT	TT	CT	CT
--	TT	TT	AT	AT	TT	AA	AT	TT	AT	AA	AT	AT
CC	CC	CC	CT	CT	CC	TT	CC	CC	--	TT	CT	CT
AA	AA	AA	AC	AC	AA	CC	AA	AA	AC	CC	AC	AC
TT	TT	TT	GT	GT	TT	GG	TT	TT	GT	GG	GT	GT
TT	--	TT	CT	CC	TT	CC	TT	TT	CT	CT	CT	CT
GG	GG	GG	CG	CG	GG	CC	GG	GG	CG	CG	CG	CG
GG	--	GG	CG	GG	GG	CC	GG	GG	CG	CG	CG	CG
CC	CC	CC	AC	CC	CC	AA	CC	CC	AC	AC	AC	AC
GG	CG	GG	CG	GG	GG	CC	GG	GG	CG	CG	CG	CG
TT	--	AT	AA	TT	--	TT	AA	AT	AT	AA	TT	TT
--	AA	AA	AA	AG	AG	GG	--	--	--	AA	GG	GG
AG	AA	AA	AA	AA	AG	GG	AA	AG	AG	AA	GG	GG
--	GG	AG	GG	GG	AG	AA	GG	AA	AG	GG	AG	AG
AG	AA	AG	AA	AA	AG	GG	AA	AG	--	AA	AG	AG
AC	AA	AC	AA	--	AC	CC	AA	AC	AC	AA	AC	AC
AG	AA	AG	AA	AA	--	GG	AA	AG	AG	AA	AG	AG
AG	AA	AG	AA	AA	AG	GG	AA	AG	AG	AA	AG	AG
GT	GG	GT	GG	GG	GT	TT	--	GT	GT	GG	GT	GT
GT	TT	GT	TT	--	GT	--	TT	GT	GT	TT	GT	GT
AC	CC	AC	CC	CC	AC	AA	CC	AC	AC	CC	AC	AC
CT	TT	CT	TT	TT	CT	CC	TT	CT	CT	CT	CT	CT
TT	--	CT	TT	TT	CT	CC	CT	CT	CT	--	CT	CT
GT	GG	GT	GG	GG	--	TT	GG	GT	GT	GT	GT	GT
TT	AA	AT	AA	AA	--	TT	--	AT	AT	AT	AT	AT
CG	GG	CG	GG	GG	CG	CC	GG	CG	CG	CG	CG	CG
--	AA	AC	AA	AA	AC	CC	AA	AC	AC	AC	AC	AC
TT	CC	CT	CC	CC	CT	TT	--	CT	CT	CT	CT	CT
CG	GG	CG	GG	GG	CG	CC	GG	CG	CG	CG	CG	CG
--	CC	CT	CC	CC	CT	TT	CC	CT	--	CT	CT	CT
AT	AA	AT	AA	AA	AT	TT	AA	AT	AT	AT	AT	AT
--	TT	CT	TT	TT	CT	CC	TT	--	CT	CT	CT	CT
AG	GG	AG	GG	GG	AG	AA	GG	AG	AG	AG	AG	AG
AT	--	AT	AA	AA	AT	TT	AA	AT	AT	AT	AT	TT
TT	AA	AT	AA	AA	AT	TT	AA	AT	AT	AT	AT	AT
CC	AA	AC	AA	AA	AC	CC	AA	AC	AC	AC	AC	AC

GT	TT	TT	TT	TT	GT	GG	TT	GT	GT	GT	GT	GT
AG	GG	GG	GG	GG	AG	AA	GG	AG	--	AG	AG	AG
GT	GG	GG	GG	GG	GT	TT	GG	GT	GT	GT	GT	GT
AG	AA	AA	AA	AA	AG	GG	AA	AG	AG	AG	AG	AG
CT	TT	TT	TT	TT	CT	CC	TT	CT	CT	CT	CT	CT
AG	AA	AA	AA	AA	AG	GG	AA	AG	AG	AG	AG	AG
CC	TT	TT	TT	TT	CT	CC	TT	CT	CT	CT	CT	CT
AA	CC	CC	CC	CC	AC	AA	--	AC	AC	AC	AC	AC
--	AA	AA	AA	AA	AG	GG	--	AG	AG	AG	AG	AG
CT	TT	TT	TT	TT	CT	CC	TT	CT	CT	CT	CT	CT
AG	GG	GG	GG	GG	AG	AA	GG	AG	AG	AG	AG	AG
TT	CC	CT	--	CT	CT	CT	CC	CT	CC	CT	TT	TT
GT	GG	GT	GG	--	GG	GT	GG	GT	GT	GG	GT	GT
--	GG	GG	GG	GG	AG	GG	GG	GG	GG	GG	AG	AG
--	TT	AT	TT	TT	TT	AT	AT	AT	AT	TT	AA	AA
AA	TT	AT	AT	AT	TT	AT	AT	AT	AT	TT	AA	AA
AA	CC	AC	AC	AC	CC	AC	AC	AC	--	AC	AA	AA
TT	GG	GT	GT	GT	GG	GT	--	GT	GT	GT	TT	TT
TT	TT	TT	CT	CT	CT	TT	CT	CT	CT	CT	CT	CT
--	AG	AG	--	AG	AA	AG	AA	AA	AG	AA	AG	AG
GG	--	GG	--	--	GG	CG	GG	GG	CG	GG	CG	CG
TT	--	TT	TT	CT	CT	CT	--	TT	CT	TT	CT	CT
GT	GT	GG	GG	GG	GG	GT	GG	GG	GG	GG	GG	GG
AG	AG	AA	AA	AG	AG	GG	--	AG	AG	AA	AG	AG
--	AA	AA	AA	AC	AC	AC	AA	AC	AC	AA	AC	AC
GG	GG	GG	GG	CG	CG	CG	GG	CG	CG	GG	CG	CG
TT	TT	TT	TT	GT	GT	GT	--	GT	GT	TT	GT	GT
CC	CT	CT	CC	--	CT	TT	CC	CT	CT	CT	TT	TT
GG	GG	AA	--	AG	AA	AG	AG	AA	AA	GG	AG	AG
CC	CC	--	--	--	CT	CT	--	CT	--	CT	CT	--
CG	CG	CC	CG	--	CC	CC	CG	CC	CC	CC	CC	CC
AC	AA	AC	AC	AA	AC	AA	AC	AC	AC	AA	AA	AA
GG	AG	AG	GG	AA	AG	AA	GG	AG	AG	AA	AG	AG
TT	CT	TT	TT	CT	TT	CT	TT	TT	TT	CT	CT	CT
GG	GG	CG	GG	CG	CG	CG	GG	CG	CG	CG	GG	GG
AA	AG	AG	AA	--	AG	GG	AA	AG	AG	GG	AG	AG
CG	CG	GG	CG	GG	GG	GG	CG	GG	GG	GG	GG	GG
CC	CG	CG	--	GG	CG	GG	CC	CG	CG	GG	GG	GG
CC	--	CT	CC	TT	--	TT	--	CT	CT	TT	TT	TT
GG	AG	AG	GG	--	AG	AA	GG	AG	AG	AA	AA	AA
AG	GG	AG	AG	GG	AG	GG	AG	AG	AG	GG	GG	GG
CC	CT	CT	CC	TT	--	TT	CC	CT	CT	TT	TT	TT
GG	AG	GG	GG	AG	GG	AG	GG	GG	GG	AG	AG	AG
GG	AG	AG	GG	AA	AG	AA	GG	AG	AG	AA	AA	AA
--	AC	AC	--	AA	--	AA	CC	AC	AC	AA	AA	AA
TT	--	AT	TT	AT	AT	AT	TT	AT	AT	AT	AT	AT
AA	--	AG	AA	--	AG	--	--	AG	AG	GG	GG	GG

AA	AC	AC	AA	CC	AC	CC	AA	AC	AC	CC	CC	CC
AA	AG	AG	AA	GG	AG	GG	AA	AG	AG	GG	GG	GG
--	AG	AA	AA	AG	AA	AG	AA	AA	AA	AG	AG	AG
CC	CC	CT	CT	CT	CT	CT	CC	CC	CT	CT	CT	CT
CC	CC	CG	CG	CG	CG	CG	CC	CC	CC	CG	CG	CG
TT	TT	CT	CT	CT	CT	CT	TT	TT	TT	CT	CT	CT
CG	CG	GG	--	GG	CG	GG	CG	CG	CG	GG	GG	GG
CT	CC	CT	CT	CT	CC	CT	CC	CC	CC	CT	CT	CT
GG	GT	GG	GG	GG	GT	GG	GG	GT	GT	GT	GG	GG

1-66	1-70	1-73	1-74	1-79	1-81	1-84	1-88	1-89	1-91	1-92	1-93	1-94
CC	AA	AC	AC	CC	AC	AA	AC	AA	AA	CC	CC	AC
GG	AA	AG	AG	--	AG	AG	AG	AA	AA	GG	GG	AG
--	TT	CT	CT	CC	CT	CC	CT	TT	--	CC	CC	CT
--	GG	GG	GG	AG	AG	GG	GG	GG	GG	AG	AG	AG
AG	AA	AG	AG	--	AA	GG	AG	GG	--	GG	GG	AG
GG	CG	GG	GG	--	CG	GG	GG	--	CG	GG	GG	GG
CT	TT	CT	CT	--	TT	CT	CT	CT	TT	CC	CC	CT
--	TT	GT	GT	GT	TT	GT	GT	GT	TT	GG	GG	GT
GT	TT	TT	GT	GT	TT	GT	GT	GT	TT	GG	GG	GT
GT	TT	TT	GT	GT	TT	GG	GT	GT	TT	GT	GG	GT
AG	AA	AG	AA	AG	AA	AG	AA	AG	AA	AG	GG	AG
--	CT	CT	CT	CC	CT	--	CT	CC	CT	CT	CC	CT
--	AC	AC	AA	AC	AA	AA	AC	AC	AA	AC	CC	AA
--	AA	AG	AG	--	AG	AA	AG	AA	AG	AG	AA	AG
CT	CT	CC	CT	--	CT	CC	CC	CT	CT	CC	CC	CC
AG	AG	AA	GG	AG	GG	AA	AG	AG	GG	AG	AA	AG
TT	TT	AT	AT	--	AT	--	AT	TT	AT	AT	TT	AT
CT	CT	CC	CT	CT	CT	--	CC	CC	CT	CC	CC	CT
TT	TT	GT	GT	--	GT	TT	GT	TT	GT	GT	TT	GT
--	CC	CG	CG	CC	CG	CG	CG	CC	CG	CG	CC	CC
--	AT	AT	AA	--	AT	TT	TT	TT	AT	AT	TT	AA
CT	CT	TT	CC	--	CT	CT	TT	TT	CT	CT	TT	CC
AT	AT	AA	TT	AT	AT	AT	AA	AA	AT	AT	AA	TT
AT	AT	AA	TT	AT	--	AT	AA	AA	AT	AT	AA	TT
AG	AG	AA	GG	AG	AG	--	AA	AA	AA	AG	AA	GG
--	TT	TT	GT	--	TT	GT	TT	TT	--	GT	TT	GT
GT	GT	TT	GG	GT	GT	GT	TT	TT	GT	GT	TT	GG
AT	AT	TT	AA	AT	AT	AT	TT	TT	--	AT	TT	AA
CT	CT	TT	CC	CT	CT	CT	TT	TT	CT	CT	TT	CC
CG	CG	CC	GG	CG	CG	CG	CC	CC	--	CG	CC	GG
CT	CT	CC	TT	CT	CT	CT	CC	CT	CT	CT	CC	TT
AC	AC	AA	CC	AC	AC	AC	AA	AA	CC	AC	--	CC
AG	AG	AA	GG	--	AG	--	AA	AA	GG	GG	AA	GG
GT	GT	GG	TT	--	GT	GT	GG	--	GG	GT	GG	TT
--	AG	AA	GG	AG	AG	GG	AA	--	AG	AG	AA	GG
GT	GT	TT	GG	--	GT	--	TT	TT	--	GT	TT	GG
AG	AG	GG	AA	AG	--	AG	GG	GG	AG	AG	GG	AA
--	CT	CC	TT	CT	CT	--	CC	CC	CT	CT	CC	TT
AT	AT	AA	TT	--	AT	AT	AA	AA	AT	AA	AA	TT
AG	AG	AA	GG	--	AG	AG	AA	AA	AG	AG	AA	GG
--	AC	--	CC	AC	AC	AC	AA	AA	AC	AC	AA	CC
AG	AG	--	GG	AG	AG	GG	AA	AA	AG	AG	--	GG

CT	CT	TT	CC	--	CT	--	TT	TT	CT	CT	TT	CC
CG	CG	--	GG	--	CG	GG	CC	CC	CG	CG	CC	GG
--	CT	TT	CC	CT	CT	CT	TT	TT	CC	CT	TT	CC
CT	CT	CC	TT	TT	CT	TT	CT	CC	TT	CT	CC	TT
--	AC	AA	CC	--	CC	AC	AC	AA	CC	AC	AA	CC
--	CT	CC	CT	--	TT	CC	CT	CT	TT	CT	CT	--
--	CC	TT	CT	--	CC	CT	CT	CT	CC	CT	CT	CT
--	AG	GG	AG	--	AA	AG	AG	AA	AG	AA	AG	AG
TT	GG	GG	TT	GG	GT	GT	GT	TT	GT	GT	GG	GT
--	GG	GG	AA	GG	AG	GG	AG	AA	AA	AG	GG	AG
--	TT	CT	CC	TT	CT	TT	CT	CC	CC	CT	TT	CT
GG	CC	CG	GG	CC	CG	CC	CG	GG	GG	CG	CC	GG
GG	AA	AG	GG	--	AG	AA	AG	GG	GG	AG	AA	AG
--	CC	AC	AA	--	AC	CC	AC	AA	AA	AC	CC	AC
GG	AG	AG	GG	AG	GG	AG	GG	GG	GG	GG	AG	GG
--	TT	TT	CT	TT	CT	--	CC	CC	CC	CT	TT	CT
--	CC	CC	CT	CC	CT	CC	TT	TT	TT	CT	CC	CT
CT	TT	TT	CT	--	CT	TT	CC	CC	CC	CT	TT	CT
AG	AA	AA	AG	AG	AG	AA	GG	GG	GG	AG	AA	AG
AC	AA	AA	AA	--	AC	AA	CC	CC	AC	AC	AA	AC
AG	AA	AA	AA	AA	AG	AA	GG	GG	AG	AG	AA	AG
--	GG	GG	GG	--	CC	GG	CG	CC	CG	CG	GG	CC
CG	CC	CC	CC	--	GG	CC	CG	GG	CG	CG	CC	CG
AG	AA	AA	AA	--	GG	AA	AG	AG	AG	AG	AA	AG
AC	CC	CC	CC	--	AA	CC	AC	AC	AC	AC	CC	AC
CT	TT	TT	TT	TT	CC	TT	CT	CT	CC	TT	CT	CT
CT	CC	CC	CC	CC	TT	TT	CT	CT	CT	CC	CT	CT
AT	TT	TT	TT	TT	AA	AT	AT	AT	AT	TT	AT	TT
GG	GG	GG	GG	--	AG	--	AG	AG	GG	GG	AG	GG
--	TT	AT	TT	--	AT	AT	TT	TT	AT	TT	--	--
--	GT	GG	GG	TT	GT	--	GG	TT	TT	GT	GT	TT
--	AG	AG	AG	--	GG	AG	AG	GG	GG	GG	AG	GG
TT	CT	CT	CT	TT	TT	CT	CT	TT	TT	TT	CT	TT
--	AG	--	GG	GG	GG	--	AG	--	GG	GG	AG	GG
--	GT	TT	GT	GG	GT	GT	TT	GG	GG	GT	GT	GG
--	GT	GT	GG	GG	GG	GT	GT	GG	GG	GG	GT	GG
CT	CT	CC	CT	TT	CT	TT	CC	TT	TT	CT	TT	TT
CT	CT	CC	--	TT	CT	--	CC	TT	TT	CT	TT	TT
AG	AG	GG	AG	AA	AG	GG	GG	AG	AA	AA	AA	AA
--	--	CC	CG	--	--	CC	CC	CG	GG	GG	GG	GG
AG	AG	GG	AG	AA	AG	AG	GG	--	AA	AA	AA	AG
CT	CT	TT	CT	--	CT	--	CT	CT	--	CC	CC	--
CT	CT	TT	CT	CC	CT	CT	CT	CT	CC	CC	CC	TT
CC	CC	CG	CC	CC	CC	CC	CC	CC	CC	CC	CC	CG
--	TT	AT	TT	--	AT	TT	TT	TT	TT	TT	TT	AT
GT	GT	GG	GT	GT	GT	GG	GT	GT	GG	GG	GG	GT
CT	CT	CC	CT	CT	CT	CT	CT	CT	CC	CC	CC	CT

CT	TT	CC	CT	CT	TT	--	CT	CT	CC	CC	CC	TT
GT	GT	GG	GT	--	GT	GT	GT	GT	GG	GG	GG	GT
CT	TT	CC	CT	CT	TT	CT	CT	CT	CC	CC	CC	TT
GT	GT	TT	GT	GT	GT	GT	GT	GT	TT	TT	TT	GT
CG	CG	GG	CG	CG	CG	CG	CG	CG	GG	GG	GG	CG
--	AG	AA	AG	AA	AG	GG	GG	GG	AG	AA	AG	--
--	AG	GG	AG	GG	AG	AA	AA	AA	AA	GG	AA	AA
AA	AG	GG	AG	--	AG	AA	AA	AA	AA	GG	AA	AA
TT	CT	CC	CT	CC	CT	TT	TT	TT	TT	CT	TT	TT
--	AG	AA	AG	--	AG	GG	GG	AG	AG	AG	GG	GG
--	GT	TT	GT	--	GT	--	GG	--	GG	GT	GG	GG
--	AG	GG	AG	AG	AG	AG	GG	GG	GG	AG	AG	AG
--	CT	CT	TT	CT	CT	TT	CT	CT	CC	CT	TT	TT
--	AC	AC	CC	AC	AC	--	CC	CC	CC	AC	CC	CC
CT	CC	CC	CT	CC	CC	CT	CT	CT	CT	CC	CT	CT
GG	GT	GT	GG	GT	GT	GG	GT	GT	GT	GT	GG	GG
--	CC	CC	CC	CC	CC	CC	CT	CT	CT	CC	CC	CC
GG	CG	CG	GG	CG	CG	--	GG	GG	GG	CG	GG	GG
--	AC	AC	CC	AC	AC	--	CC	CC	--	AC	CC	CC
--	TT	--	TT	--	--	--	CT	CT	CT	TT	TT	TT
--	AA	AA	AA	--	AA	AA	AG	AG	AG	AA	AA	AA
AA	AG	AG	AA	AG	AG	AA	AG	AG	AG	AG	AA	AA
CT	CC	CC	CT	CC	CC	CT	CT	CT	CT	CC	CT	CT
--	AT	AT	AA	AT	AT	AA	AA	AA	AA	AT	AA	AA
--	CT	CT	CC	--	CT	CC	CC	CC	CC	CT	CC	CC
--	AG	AG	AA	AG	--	--	AA	AA	AA	AG	AA	AA
CC	CC	CC	CC	--	CC	CC	CT	CT	CT	CC	CC	CC
--	AT	AT	TT	AT	AT	TT	AT	AT	AT	AT	TT	--
--	AT	AT	TT	AT	AT	AT	AT	--	AA	AT	TT	TT
--	CT	CT	TT	CT	CT	TT	CT	CT	CT	CT	TT	TT
--	CT	CT	CC	CT	CT	CC	CC	CC	CC	CT	CC	CC
--	GG	GG	GG	--	--	GG	GT	GT	GT	GG	GG	GG
--	CC	CC	CC	CC	CC	CC	CT	CT	CT	CC	CC	CC
--	AG	AG	AG	AG	--	AA	AA	AA	AA	AG	AG	AG
--	AG	AG	GG	AG	AG	GG	AG	AG	AG	AG	GG	GG
AA	AA	AA	AA	AA	AA	AA	AG	AG	AG	AA	AA	AA
CC	CT	CT	CC	--	CT	CC	CT	CT	CT	CT	CC	CC
CC	CC	CC	CC	--	CC	--	AC	AC	AC	CC	CC	CC
--	AT	AT	TT	AT	AT	TT	AT	AT	AT	AT	TT	TT
--	AG	AG	AA	--	AG	AA	AA	AA	--	AG	AA	AA
AA	AA	AA	AA	AA	AA	AA	AC	AC	AC	AA	AA	AA
TT	TT	TT	TT	TT	TT	TT	AT	AT	AT	TT	TT	TT
AC	CC	CC	AC	--	CC	AC	AA	AA	AA	CC	AC	AC
CC	AC	AC	CC	AC	AC	CC	CC	CC	--	AC	CC	CC
AG	AG	AG	AG	AG	AG	AG	GG	GG	GG	AG	GG	AG
AC	CC	CC	AC	CC	--	AC	AC	AC	AC	CC	AC	AC
--	AA	AA	AA	--	AA	AA	AG	AG	AG	AA	AA	AA

--	CG	CG	CC	CG	CG	CC	CC	CC	CC	CG	CC	CC
--	CT	CT	TT	--	CT	TT	CT	CT	CT	CT	TT	TT
CC	CT	--	CC	CT	CT	CC	CT	CT	CT	CT	CC	CC
--	--	CC	CC	--	CC	--	CT	CT	CT	CC	CC	CC
AA	AA	--	AA	AA	AA	AA	AG	AG	AG	AA	AA	AA
CC	CT	CT	CC	TT	CT	CC	CT	CT	CC	TT	CC	CC
--	TT	TT	TT	CT	TT	TT	CT	CT	CT	TT	TT	TT
--	AT	AT	AA	TT	AT	AA	AT	AT	AT	--	AA	AA
GG	AG	AG	GG	AG	AG	GG	GG	GG	GG	AG	GG	GG
--	AA	AA	AA	AC	AA	--	AC	AC	AC	AA	--	AA
--	--	--	CC	CT	CC	--	CT	CT	CT	CC	CC	CC
--	GT	GT	TT	GT	--	TT	TT	TT	TT	GT	TT	TT
--	TT	TT	TT	--	TT	TT	CT	CT	CT	TT	CT	TT
AC	AC	AC	AC	--	AC	AC	CC	CC	CC	AC	CC	AC
--	AA	--	AA	AG	AA	AA	AG	AG	AG	AA	AG	AA
--	GG	GG	GG	GG	GG	GG	GT	GT	GT	GG	GT	GG
AT	AT	AT	AT	TT	--	TT	TT	TT	TT	AT	TT	AT
--	AA	AA	AA	AG	AA	AA	AG	AG	AG	AA	AG	AA
--	CT	CT	CC	CT	CT	CC	CC	CC	CC	CT	CC	CC
--	CC	--	CC	CT	CC	CC	CT	CT	CT	CC	CT	--
--	AA	AA	AA	AC	AA	AA	AC	--	AC	AA	AC	AA
--	CC	CC	CC	CT	CC	CC	CT	CT	CT	CC	CT	CC
--	AT	AT	AA	--	AT	--	AT	AT	AT	AT	AA	--
GG	AG	AG	GG	--	AG	GG	AG	AG	AG	AG	AG	GG
--	CG	CG	GG	CG	CG	GG	GG	GG	GG	CG	GG	GG
--	TT	TT	TT	CT	TT	--	CT	CT	CT	TT	CT	TT
GG	GG	--	GG	--	GG	GG	AG	AG	AG	GG	AG	GG
AA	AA	AA	AA	AG	AA	AA	AG	AG	AG	AG	AG	AG
AA	AG	AG	AA	AA	AG	AA	AA	AA	AA	AG	AA	AA
--	AG	AG	GG	AG	AG	GG	GG	GG	GG	AG	GG	GG
--	--	CT	CT	CT	--	CT	TT	CT	TT	CT	CT	CT
--	GT	TT	GT	TT	--	TT	GT	--	GG	TT	GT	GT
--	GT	GT	--	GT	GT	--	TT	TT	TT	GT	TT	TT
--	AG	AA	AG	--	AG	AG	AG	AG	GG	AA	AA	AG
CC	CC	CT	CT	CT	CC	CT	CT	CT	CC	CT	CT	CT
GT	TT	GT	GT	GT	TT	GT	GT	GT	TT	GT	GT	GT
AG	--	GG	AG	--	GG	GG	AG	AG	AG	GG	AG	AG
--	AG	AG	AG	AG	AG	GG	AG	AG	AA	AG	AA	AG
--	CT	CT	CT	CC	CC	CC	CT	CC	CC	CC	--	CC
--	AT	AT	AT	AT	AA	AT	AT	AA	AA	AA	AA	AA
GG	CG	CG	CG	CG	CG	--	CG	CG	CG	CG	CC	CG
AC	AA	AA	AA	AA	AC	AA	AA	AC	--	AC	AA	AC
--	GG	AG	AG	AG	AG	AG	AG	AG	AG	AA	AA	AA
--	GG	GG	GG	GG	AG	GG	GG	AG	AG	AG	GG	AG
CC	CC	CC	CC	CC	CT	CC	CC	CT	CT	CT	CT	CT
--	AA	AA	AA	AG	AG	AG	GG	AG	GG	GG	GG	GG
CC	CT	CT	CT	CT	CT	CC	CT	CT	CC	CC	CC	CC

AG	AG	AG	AG	AG	GG	AG	GG	GG	GG	GG	GG	GG
--	AG	AG	AG	AG	GG	AA	GG	GG	AG	AG	GG	AG
CT	TT	TT	TT	TT	CT	CT	CT	CT	CC	CC	CC	CC
TT	TT	TT	TT	TT	AT	AT	AT	AT	AT	AA	AA	AA
--	CC	CC	CC	CC	AC	AA	AC	AC	AA	AA	AA	AA
--	CC	CC	CC	CC	CT	--	CT	--	TT	TT	--	TT
AA	AA	--	AA	AA	AG	GG	AG	AG	GG	GG	GG	GG
--	TT	TT	TT	--	CT	CT	CT	--	CC	CC	CC	CC
CT	CT	CT	CT	TT	CT	--	CT	--	TT	TT	--	TT
--	GG	GG	GG	AG	GG	AA	AG	AG	AA	AA	AA	AA
AG	AG	AA	AG	AG	AG	--	AA	AG	GG	AG	AG	GG
CT	TT	CC	CT	CT	CT	CT	CC	CT	TT	CT	--	TT
AG	AG	GG	AG	--	GG	AG	GG	AG	--	GG	AG	AG
CT	CT	CC	TT	CT	CT	CT	CC	CT	TT	CT	CT	TT
--	GG	GG	CG	CG	CG	GG	GG	GG	CG	--	GG	CG
--	CT	CC	TT	--	CC	TT	CC	CT	TT	--	CT	--
CT	CT	CC	CT	--	CC	CT	CC	--	CT	CC	CT	CC
AG	AG	GG	AA	AG	GG	AG	GG	AA	AA	AG	GG	AG
CT	CT	CC	TT	CT	CC	--	CT	TT	TT	TT	CT	CT
AG	AG	AA	AG	AG	AA	AG	AG	GG	GG	GG	GG	AG
CT	CT	CC	CT	CT	CC	CT	CT	TT	TT	TT	TT	CT
AG	AG	AG	AG	--	GG	AG	AG	AA	AA	AA	AA	AG
AG	AG	AG	AG	AG	GG	AG	GG	AG	AG	AG	AG	GG
GT	GT	GG	GT	GT	TT	GT	GT	GG	GT	GG	GG	GT
CG	CG	CG	CG	CG	CG	CC	CC	CG	CG	CG	CG	CC
--	CG	CC	CG	CG	CG	CG	CC	CC	CG	CC	CC	CC
CT	CT	--	CC	CT	CT	CT	CT	TT	CT	TT	TT	CT
CT	TT	CC	TT	CT	CT	CT	CT	CC	CT	CC	CC	CT
--	AA	TT	AA	--	AT	AT	AT	TT	AA	TT	TT	AT
AG	GG	AA	GG	AG	AG	GG	AG	AA	--	AG	AA	AG
CT	CC	TT	CC	--	CT	CT	CT	TT	CT	CT	TT	CT
CT	TT	CT	TT	CT	CT	CT	TT	CT	CT	TT	CT	TT
AC	AA	CC	AA	AC	AC	CC	AC	CC	AC	AC	--	--
--	GT	--	TT	GT	GT	--	GT	GT	GG	GT	TT	TT
GG	AG	AG	AA	--	AG	AA	AG	GG	GG	AG	--	AA
--	CT	CT	TT	--	CT	TT	CT	CC	CC	CT	TT	TT
--	AT	AT	AA	AT	AT	AA	AT	AT	TT	AT	AA	AA
--	AC	AC	CC	AC	AC	CC	AC	AC	AA	AC	CC	CC
CC	CC	CG	CG	--	CC	CG	CC	--	CC	CC	CG	CG
--	AA	AG	AG	AA	AA	AG	AA	AG	AA	AA	AG	AG
--	CT	CT	CC	CT	CT	CC	CC	CT	TT	CT	CC	--
--	CT	CT	CC	CT	CT	CC	CT	CT	--	CT	CC	CC
--	AG	AG	AA	AG	AG	AA	AG	AG	GG	AG	AA	AA
--	CT	CC	CT	--	CT	--	CT	CC	CC	CT	CT	CT
--	AG	AG	AA	--	AG	AA	AA	AG	GG	--	AA	AA
--	AG	AG	GG	--	AG	--	GG	AG	AA	AG	--	GG
TT	GT	GT	GG	--	GT	GG	GG	GT	TT	GT	GG	GG

--	TT	CT	TT	--	CT	--	TT	CT	CC	CC	TT	TT
CC	GG	CG	GG	GG	CG	GG	GG	CG	CC	CG	GG	GG
--	TT	GT	TT	TT	GT	TT	TT	GT	GG	GT	TT	TT
--	AA	AG	AA	--	AG	AA	AA	AG	GG	AG	AA	AA
--	CC	CT	CC	CC	CT	CC	CC	CT	TT	--	CC	CC
TT	GT	TT	GT	TT	GT	GT	GT	TT	TT	GT	GT	GT
--	--	CG	GG	--	CG	GG	GG	CG	CC	CG	GG	GG
--	AA	AG	AA	AA	--	AA	AA	AG	--	AG	AA	--
--	AG	GG	AG	--	AG	GG	AG	GG	GG	AG	AG	AG
--	AA	AC	AA	--	AC	AA	AA	AC	CC	AC	AA	AA
CC	CG	CC	CG	--	CG	CG	CG	CC	CC	CG	CG	CG
AA	GG	AG	GG	AG	AG	GG	GG	AG	AA	AG	GG	GG
CC	AA	AC	AA	--	AC	AA	AA	AC	CC	AC	AA	AA
--	TT	GT	TT	GT	GT	TT	TT	GT	GG	GT	TT	TT
--	CC	AC	CC	AC	AC	CC	CC	AC	AA	AC	CC	CC
CC	CT	--	CT	CC	CT	CC	CT	CC	CC	CT	CT	CT
GG	TT	GT	TT	GT	GT	TT	TT	GT	GG	GT	TT	TT
--	AG	AG	AG	--	GG	AG	AG	AG	GG	GG	AG	AG
--	CC	CT	CC	CT	CT	CC	CC	CT	TT	CT	CC	CC
--	CT	CC	CT	CC	CT	CT	CT	CC	--	CT	CT	CC
--	AA	AT	AA	AT	AT	AA	AA	AT	TT	AT	AA	--
--	CC	CG	CC	CG	CG	CC	CC	CG	GG	CG	CC	CC
TT	GG	GT	GG	GT	GT	GG	GG	GT	TT	GT	GT	GT
GG	AG	AG	AG	AG	GG	AG	AG	AG	GG	GG	GG	AG
GT	TT	GT	TT	TT	--	TT	TT	TT	TT	TT	GT	GT
--	CC	CT	CC	CC	CT	--	CC	CC	TT	CT	TT	CT
--	CC	AC	CC	CC	AC	CC	CC	CC	AA	AC	AA	AC
CT	--	--	CC	CC	CT	CC	CC	CC	CC	CT	CT	CC
CC	--	CG	GG	GG	CG	GG	GG	GG	CC	CG	CC	CG
TT	CC	CT	CC	CC	CT	CC	CC	CT	TT	CT	TT	--
--	GT	GT	GT	--	TT	TT	GT	TT	--	TT	TT	--
--	CT	TT	CT	CT	CT	TT	CT	--	CT	TT	--	CT
AT	AT	AT	AT	AT	TT	AT	AT	TT	TT	TT	TT	TT
CC	CC	CC	CC	CC	AC	CC	CC	AC	AC	AC	AC	CC
--	TT	CT	TT	TT	CT	CT	TT	TT	--	CC	CC	TT
AA	AA	AA	AA	--	--	AA	AA	AG	AA	AG	AG	AA
--	GG	GG	GG	--	AG	GG	GG	AG	AG	AG	AG	GG
CG	GG	CG	GG	--	GG	CG	GG	GG	GG	CG	CG	GG
--	CC	--	CC	--	CC	TT	CT	TT	--	TT	TT	CT
TT	--	TT	CC	CC	CT	TT	CC	TT	CT	CT	TT	CT
--	CC	TT	CC	CC	CT	TT	CC	--	CC	CT	CT	CT
CT	--	CC	TT	--	CT	CT	TT	CC	TT	CT	CT	CT
--	GG	TT	GG	GT	GT	GT	GT	TT	GG	GT	GT	GG
CT	TT	CC	TT	CT	CT	CT	CT	CC	TT	CT	CT	TT
AT	TT	AA	TT	AT	AT	AT	AT	AA	TT	AT	AT	--
--	GG	AA	GG	AG	--	AG	AG	AA	AG	AG	AG	GG
--	AA	CC	AA	AC	AC	AC	AC	CC	AC	CC	AC	AA

AG	--	AA	GG	AG	AG	AG	AG	AA	AA	AA	AG	GG
AG	GG	AA	GG	--	AG	GG	AG	--	--	AA	AG	GG
--	CC	TT	CC	CT	CT	CC	CT	TT	CT	TT	CT	CC
AG	AA	GG	AA	AG	AG	GG	AG	GG	AG	GG	--	--
--	TT	--	TT	--	GT	GT	GT	GG	GG	GG	GT	TT
CT	CC	--	CC	CT	CT	CT	CT	--	CT	TT	TT	CC
AG	AA	GG	AA	AG	AG	AG	AG	GG	AG	--	AG	AA
AG	AA	--	AA	--	AG	--	AG	GG	GG	GG	--	AA
AG	GG	AA	GG	AG	--	GG	AG	AA	GG	AA	AG	GG
CG	CC	GG	CC	CG	CG	CC	CG	CG	CG	GG	GG	CC
AG	GG	AA	GG	AG	AG	--	AG	AA	AG	AA	AG	GG
--	AC	AA	CC	AC	AC	AC	AC	AA	AA	AA	AA	CC
GG	AG	GG	AA	AG	AG	AG	AG	GG	AG	GG	AG	AA
--	AA	GG	AA	AG	AG	AG	AG	AG	AG	GG	AG	AA
--	TT	AA	TT	AT	AT	AT	AT	AT	AT	AA	AT	TT
TT	CC	TT	CC	--	CT	CT	CT	CT	CT	TT	CC	CC
--	CC	TT	CC	CT	CT	--	CT	TT	CT	TT	CT	CC
--	GG	CC	GG	CG	CG	CG	CG	CG	CG	CC	CG	GG
CC	TT	CC	TT	CT	CT	CT	CT	CT	CT	CC	CT	TT
--	CC	TT	CC	--	CT	CT	CT	CT	CT	TT	CT	CC
--	TT	CC	TT	--	CT	TT	CT	CT	CC	CC	CT	TT
AA	TT	AA	TT	--	AT	AT	AT	AT	AT	AA	AT	TT
--	AA	TT	AA	AT	TT	AT	AT	AT	TT	TT	AT	AA
--	TT	CC	TT	CT	CC	CT	CT	CT	CT	CC	CT	TT
--	CC	TT	CC	CT	TT	CT	CT	CT	CT	TT	CT	CC
--	AA	AT	AA	--	TT	AT	AT	AT	AT	TT	AT	AA
--	GG	AG	GG	--	AA	AG	AG	AG	AG	AA	AG	GG
--	--	GT	GG	GT	TT	GT	GT	--	GT	TT	GT	GG
CC	TT	CT	TT	CT	CC	--	CT	CT	--	CC	CC	--
--	CC	CG	CC	--	GG	GG	CG	CG	CG	GG	CG	CC
--	GG	AG	GG	AG	AA	AG	AG	AG	AG	AA	AG	GG
CC	TT	CT	TT	--	CC	CT	CT	--	CT	CC	CT	TT
--	TT	CT	TT	CT	CC	CT	CT	CT	TT	CC	CT	TT
--	TT	AT	TT	--	--	AT	AT	AT	TT	AA	AT	TT
--	AA	AG	AA	AG	GG	AG	AG	AG	AA	GG	AG	AA
--	GG	AG	AG	AG	GG	AG	AA	AG	AA	GG	GG	AA
--	GG	TT	GG	GT	GG	GT	TT	GT	TT	GG	GG	TT
--	GG	AA	GG	AG	--	GG	AA	AA	AA	GG	GG	AA
AG	AG	GG	AG	AG	AG	AG	GG	GG	--	AG	GG	--
CT	TT	TT	CT	--	CT	TT	CT	TT	CT	TT	TT	TT
--	AG	--	AG	AG	AG	--	AG	AA	AG	GG	AG	GG
--	GG	GG	GG	AG	GG	AG	AG	AG	GG	GG	AG	AG
--	--	GG	AG	AG	AG	AG	AA	AG	AG	GG	GG	AA
CC	CC	--	CG	CG	CG	CG	GG	CG	GG	CC	CC	GG
--	TT	TT	CT	CT	CT	--	CC	CT	CT	TT	TT	CC
AC	AC	AC	CC	AC	CC	AC	CC	AC	CC	AC	AC	CC
AG	AG	AG	AG	--	--	AA	AA	AG	AA	AG	AG	AA

AC	AC	CC	CC	AC	CC	AC	CC	AC	CC	AC	AC	CC
CC	CC	CC	CC	CC	AC	CC	AC	CC	CC	CC	CC	AC
CT	CT	CT	TT	CT	TT	CT	TT	CT	TT	CT	CT	TT
--	GG	GG	AG	AG	AG	GG	AA	GG	AG	GG	GG	AG
--	AA	AG	AG	AG	AG	AA	AG	AA	AG	AA	AA	AG
CC	CC	CT	CT	TT	CT	CC	CC	CC	CT	CC	CC	CT
TT	TT	CT	TT	--	--	TT	TT	TT	TT	TT	TT	TT
--	GG	CG	CG	CC	CG	GG	GG	GG	CG	GG	GG	CG
GG	GG	AG	AG	AA	AG	GG	GG	GG	AG	GG	GG	AG
--	CT	--	CT	TT	CT	CC	CC	CC	CT	CT	CC	CT
--	AA	AT	AA	AT	AT	--	AA	AA	AA	AT	--	AA
AC	AC	AC	AC	CC	AC	AA	AA	AA	CC	AC	AA	AC
AG	GG	AG	GG	GG	GG	--	AG	AG	--	AG	AG	GG
CT	CC	CT	CC	CC	CC	CC	CT	CT	CC	CT	CT	CC
--	CC	CT	CC	CC	CC	CC	CT	CT	CC	CT	CT	CC
--	GG	GT	GG	--	GG	GG	GT	GT	GG	GT	--	GG
TT	TT	CT	CT	CC	CT	CT	CT	CT	CC	CT	CT	CT
GG	GG	AG	AG	AG	GG	GG	GG	GG	AG	AG	GG	GG
--	AG	AG	AG	--	AG	--	AG	GG	AA	GG	GG	--
CG	GG	CG	CG	--	GG	--	GG	GG	GG	CG	CG	--
GG	AG	AG	AG	AA	AG	AG	AG	AG	AA	GG	GG	AG
AA	AG	AG	AG	GG	AG	AG	AG	AG	GG	AA	AA	AG
--	GT	TT	TT	GT	GT	GT	GT	GT	GT	TT	TT	GT
AA	AG	--	AG	--	AG	AG	AG	GG	GG	AA	AA	AG
--	AG	AG	AG	--	AG	AG	AG	GG	GG	AA	AG	AG
--	GT	GG	GG	GG	GG	GT	GT	GT	GT	GG	GT	GT
--	TT	CT	CT	CT	--	--	CT	TT	TT	CC	--	TT
--	CC	CT	CT	CT	TT	CT	CT	CC	--	TT	CC	CC
CC	CT	CT	CT	CT	CC	CT	CC	CT	CC	CC	CT	CT
--	GG	AA	AG	AG	AA	GG	AG	AG	AG	AA	GG	AG
CC	TT	CC	CT	--	CC	TT	CT	CT	CT	CC	TT	CT
--	GG	AA	AG	AG	AA	GG	AG	AG	AG	AA	GG	AG
CT	--	--	CT	--	CC	TT	CT	CC	CT	CC	CT	CT
CG	CC	GG	CG	CG	GG	--	CG	CG	CG	GG	CG	CG
CT	CT	CC	CT	CT	CC	TT	CT	CT	--	CC	CT	CT
AC	AC	CC	AA	AC	AC	AC	CC	CC	--	AC	AC	AC
AG	AG	AA	AG	AG	AG	AG	AA	AG	AG	GG	AG	--
CT	CT	CT	CT	--	CT	--	CC	CT	CT	TT	CT	TT
AG	AG	AG	AG	--	AG	--	GG	AG	AG	AA	AG	AG
AG	AG	AG	AG	--	AG	AG	GG	AG	AG	AA	AG	AG
--	TT	CT	TT	CT	TT	--	CT	CT	TT	TT	--	TT
--	AG	AG	AG	AG	GG	GG	AA	AG	--	GG	AA	AA
--	AG	--	AG	AA	AA	AA	AG	--	AA	AA	AG	AA
TT	GT	TT	GT	TT	TT	TT	GT	TT	GT	TT	GT	GT
--	GT	TT	GT	TT	TT	TT	GT	TT	GT	TT	GT	GT
AA	AC	AC	AC	AC	AA	AA	CC	AC	AC	AA	CC	--
AA	AG	AG	AG	--	--	AA	GG	AG	AG	AA	AG	--

--	CG	CG	CG	CG	CC	CC	GG	CG	CG	CC	CG	CG
--	CG	CG	CG	GG	CC	GG	GG	CG	CG	CC	CG	--
--	CT	CT	CT	CC	--	CT	CT	TT	TT	TT	CT	CT
CC	CT	CT	CC	--	CC	CT	--	CC	CC	CC	CT	CT
--	AT	AT	AA	TT	AA	AA	AA	AA	AA	AA	AT	AA
AG	GG	AG	AG	GG	AG	GG	AG	AG	AG	AG	AG	GG
CC	CT	CT	CC	--	CC	CT	CC	CC	CC	CC	CT	CT
--	CT	CT	TT	CC	--	TT	TT	TT	TT	TT	TT	CC
--	CT	--	CC	--	CC	TT	CC	CC	CC	CC	CT	CT
--	AG	AG	GG	AG	GG	AA	GG	GG	GG	GG	AG	AG
--	CC	CG	CC	--	CC	CG	CC	CC	CC	CC	CC	CG
--	CG	GG	GG	--	GG	GG	GG	GG	GG	GG	CG	GG
--	GG	GT	GG	--	GT	GT	GG	GG	GG	GG	--	--
--	CC	CG	CC	CC	CG	CG	CG	CC	CG	CC	CG	CG
--	AG	AA	AG	AG	AG	AA	AA	GG	AA	--	AA	AG
AC	AA	AC	AA	--	AC	AC	AC	AA	AC	AA	AC	AC
AT	AA	AT	AA	AA	AT	AT	AT	AA	AT	AA	AT	AT
AA	AG	AA	AG	--	AG	AA	GG	GG	GG	AG	--	AG
--	AT	TT	AT	AT	AT	--	AA	AA	--	AT	TT	AT
--	AG	AA	AA	AG	AG	AG	GG	AG	AA	GG	AA	AG
--	AC	AC	AC	AC	CC	CC	CC	AC	AC	CC	CC	CC
TT	CT	TT	TT	CT	CT	CT	CC	CT	TT	CC	TT	CT
GG	TT	GG	GG	GT	GT	GT	TT	GT	GG	TT	GG	GT
GG	TT	GG	GG	GT	GT	GT	TT	--	GG	TT	GG	GT
TT	CC	TT	TT	--	CT	CT	CC	CT	TT	CC	TT	CC
--	CC	GG	--	CG	CG	CG	CC	CG	GG	CC	GG	--
--	AG	--	AA	AG	AG	--	GG	AG	AA	GG	AA	AG
--	CT	TT	TT	CT	CT	--	CC	CT	TT	CC	TT	CT
GG	GT	GG	GG	GT	GT	GT	TT	GT	GG	TT	GG	GT
--	CT	CC	CC	CT	CT	CT	TT	CT	CC	TT	CC	CT
--	CG	--	CC	CG	CG	CG	CG	CG	CC	GG	CC	CG
AA	AG	AG	AA	AG	AG	AG	AG	AG	AG	GG	AA	AG
--	AC	AC	CC	--	--	--	AC	--	--	AA	--	AC
CC	CT	CT	CC	TT	--	CT	CT	CC	CT	TT	CT	CT
--	AG	AG	AG	--	GG	AG	GG	GG	AG	GG	AG	AG
CC	AC	AA	CC	--	AC	AC	AC	AC	AC	AA	CC	AC
TT	CT	CC	TT	CC	CT	CT	CT	CT	CT	CC	TT	CT
AA	AG	GG	AA	GG	AG	AG	AG	AG	AG	GG	AA	AG
--	CG	GG	CC	--	CG	--	CG	CG	CC	GG	CC	CG
--	GT	GG	TT	--	GT	GT	--	GT	GT	GG	TT	GT
--	CT	CC	TT	CC	CT	CT	CT	CT	CT	CC	TT	--
CT	CT	TT	CT	TT	TT	CT	TT	CT	CT	TT	--	CT
--	AG	AG	AG	AG	GG	AG	GG	GG	AG	AG	GG	GG
GG	CG	CC	CG	--	CG	GG	CG	GG	CG	CC	GG	CC
AT	AA	AA	AA	AA	AT	AT	AT	AT	AA	AA	AT	AA
AG	AG	AA	AG	AA	AA	AG	AA	AA	AG	AA	AG	AG
CC	AC	CC	AC	CC	CC	AC	CC	CC	AC	CC	AC	AC

TT	CT	TT	CT	--	TT	CT	TT	TT	CT	TT	CT	CT
--	TT	CT	TT	CT	CT	CT	CT	CT	TT	TT	CT	TT
--	GT	AG	GT	AG	--	AT	AG	AG	--	GG	AT	--
AA	AT	AA	AT	AA	AA	AT	AA	AA	AT	AA	AT	AT
GG	AG	AG	AG	AG	AG	AA	GG	AG	AG	GG	AA	AG
--	AC	AC	AC	AC	AC	AA	CC	AC	--	CC	AA	--
AA	AG	AA	AG	--	AA	AG	AA	AA	AG	AA	AG	AG
--	AG	AG	AG	--	AG	--	GG	--	--	GG	--	AG
--	AG	AG	AG	AG	AG	GG	AA	AG	AG	AA	GG	GG
--	AA	AG	AG	--	AG	--	GG	AG	--	--	--	AG
--	TT	GT	GT	GT	GT	GT	GG	TT	--	GT	--	GT
GG	AG	AG	GG	AG	AG	GG	GG	GG	GG	AG	AG	GG
--	TT	CT	CT	--	CT	CT	CC	CT	TT	CT	TT	CT
CC	GG	CG	CG	CC	CG	CG	CC	CG	GG	CG	GG	CG
TT	CT	TT	CT	TT	TT	CT	TT	TT	CT	--	CT	CT
TT	CT	TT	TT	--	CT	TT	TT	CT	CT	CT	CT	TT
CC	CC	CC	CT	CC	CC	CC	CT	CT	CC	CC	CC	CC
GT	GG	GG	GG	GT	GG	GT	GG	GG	GT	GG	GG	GG
--	GG	GG	AG	--	GG	AG	AG	AG	AG	GG	GG	GG
--	AA	AA	AG	AG	--	AG	AG	--	AG	AA	AA	AA
--	AA	AA	AG	AA	AA	AA	AG	AG	AA	AA	AA	AA
--	GT	GG	TT	GG	GG	--	GT	GT	GG	GT	GG	GG
AG	AA	AA	AG	AA	AA	--	AG	AG	AA	AA	AA	--
CT	CC	--	CT	--	CC	CC	CT	CT	CC	CC	CC	CC
--	AG	AG	AA	--	AG	AG	AA	--	AG	AG	AG	AG
--	AG	GG	AA	--	GG	AA	AG	AG	AG	AG	AG	AG
--	AG	GG	AA	--	GG	AA	AG	AG	AG	AG	AG	AG
CC	CG	GG	CC	CG	GG	CC	CG	CG	CG	CG	CG	CG
--	CG	GG	CC	--	GG	CC	CG	CG	CG	CG	CG	CG
AA	AG	GG	AA	AG	GG	AA	AA	AG	AG	AG	AG	AG
AG	AG	GG	AG	AG	GG	AG	AG	GG	AG	AG	AG	GG
--	CT	TT	CC	CT	--	--	CC	CT	CT	CT	CT	CT
AC	AC	CC	AC	AC	CC	CC	AC	--	AC	AC	--	AC
--	CG	CC	CG	CC	CC	CG	CG	CC	CC	CG	CG	CG
AC	AC	CC	AC	CC	CC	AC	AC	CC	CC	AC	AC	AC
--	AC	AA	CC	--	AA	CC	CC	AC	AA	AA	--	--
AG	AA	AA	AG	AA	AA	AG	AG	AG	AA	AA	AA	--
AG	AG	AA	GG	AA	AA	GG	GG	AG	AA	AA	GG	AG
AG	GG	GG	AG	GG	GG	GG	AG	AG	GG	GG	AG	GG
CT	CT	CT	TT	--	CC	TT	TT	CT	CC	CC	TT	CT
CT	TT	CT	TT	CT	--	TT	TT	TT	CC	CC	TT	CC
--	AG	AG	AG	--	AA	--	AG	AG	AA	AA	--	AG
CC	CT	CT	CT	CC	CC	--	CT	CT	CC	CC	CC	CC
--	AG	--	AG	--	AG	--	AG	GG	GG	AA	AG	--
--	AC	AC	CC	AC	AC	AC	CC	CC	CC	AC	CC	AC
CC	CC	CT	CT	CC	CC	--	CT	CC	CC	CT	CC	CT
--	AG	GG	AG	AG	--	AG	--	--	AA	AG	AA	GG

AA	AA	AG	AG	--	AA	AA	AG	AA	AA	AA	AG	AG
AG	AG	GG	GG	AG	AG	AG	GG	AA	AG	AG	AG	GG
AC	AC	AA	AA	AC	AC	AC	AA	CC	AC	AC	--	AA
AG	AG	AA	AA	AG	AG	AG	AA	GG	AG	AG	AG	AA
GT	GT	GT	GG	--	GT	GT	GG	TT	GG	GT	GT	GG
AG	AG	AA	AG	--	AG	AG	AG	AA	AG	AG	AA	AG
CT	CT	CT	CC	CT	CT	CT	CC	CT	CT	CT	CC	CC
CT	CT	CC	CT	--	CT	CT	CT	CT	CT	CC	CC	CT
--	AG	AG	AA	AG	AG	--	AA	AA	AG	GG	AG	AA
CG	GG	CG	CG	CG	GG	--	CG	CG	GG	GG	CG	CG
CT	TT	CT	CT	CT	TT	--	CT	CT	TT	TT	CT	CT
--	AG	AG	GG	AG	AG	AG	GG	GG	AG	AA	AG	--
--	AG	AG	GG	AG	AG	AA	GG	GG	AG	AA	AG	GG
--	CG	CG	GG	CG	CG	CC	GG	GG	--	CC	CG	CG
--	AG	AG	GG	--	AG	AA	GG	GG	AG	AA	AG	AG
AG	GG	--	AG	--	AG	--	GG	AG	GG	GG	AA	AG
CT	TT	CT	CT	CC	CT	CT	TT	CT	TT	TT	CC	CT
--	GG	AG	AA	--	AG	--	--	AG	GG	GG	AA	AG
--	CC	CG	CG	GG	CG	CG	CC	CG	CC	CC	GG	CG
AC	CC	AC	AC	AA	--	AC	CC	AC	CC	CC	AA	AC
AG	AA	AG	AG	GG	AG	GG	AA	--	AA	AA	GG	AG
GT	GG	GT	GT	TT	GT	--	GG	GT	GG	GG	TT	GT
AG	GG	AG	AG	AA	AG	AA	GG	AG	GG	GG	AA	AG
AG	GG	AG	AG	--	AG	--	GG	AG	GG	GG	AA	AG
--	AG	AG	AG	--	--	--	AA	AG	AA	AA	GG	--
AC	AA	AC	AC	CC	AC	AC	AA	AC	AA	AA	CC	AC
AG	AA	AG	AG	--	AG	AG	AA	AG	AA	AA	GG	AG
AG	AA	AG	AG	AG	AG	AG	AA	AG	AA	AA	GG	AG
CT	TT	CT	CT	CC	CT	--	TT	CT	TT	TT	CC	CT
CG	--	CG	CG	--	CG	CC	GG	CG	CG	GG	CC	CG
AC	AA	AC	AC	CC	AC	--	AA	--	AA	AA	CC	AC
--	TT	CT	CT	--	CT	CT	TT	CT	TT	TT	CC	--
GT	--	GT	GT	GG	GT	GT	TT	GT	TT	TT	GG	GT
CT	CC	CT	CT	TT	CT	CC	CC	CT	--	CC	TT	CT
CG	CC	CG	CG	GG	CG	CG	CC	CG	CC	CC	GG	CG
AT	TT	AT	AT	--	AT	AT	TT	AT	TT	TT	AA	AT
--	CC	CT	CT	--	CT	CC	CC	CT	CC	CC	TT	CT
--	GG	AG	AG	--	AG	--	GG	AG	GG	GG	AA	AG
AG	AA	AG	AG	--	AG	AG	AA	AG	AA	AA	GG	AG
--	AA	AT	AT	--	AT	--	AA	AT	AA	AA	TT	AT
--	GG	AG	AG	AA	AG	AG	GG	AG	GG	GG	AA	AG
AG	AA	AG	AG	GG	AG	--	AA	AG	AG	AA	GG	AG
CG	CC	CG	CG	GG	CG	CC	CC	CG	--	CC	GG	CG
AG	AA	AG	AG	GG	AG	AG	AA	AG	AA	AA	GG	AG
--	GG	CG	CG	--	--	CG	GG	CG	--	GG	CC	CG
--	GG	AG	AG	--	AG	--	GG	--	GG	GG	AA	AA
--	AA	AC	AC	CC	AC	AC	AA	AC	AA	AA	--	AC

--	GG	AG	AG	--	--	--	GG	AG	GG	GG	AA	AG
CC	CC	CG	CG	GG	CG	--	CC	CG	CC	CC	--	CC
--	CC	CT	CT	TT	CT	CT	CC	TT	CC	--	--	CC
--	TT	AT	AT	AA	AT	TT	TT	AT	TT	TT	AA	AT
--	CC	CT	CT	--	CT	CC	CC	CT	CC	CC	TT	CT
AA	AA	AT	AT	--	AT	AT	AA	AT	AA	AA	TT	AT
--	TT	AT	AT	AA	--	TT	TT	AT	TT	TT	AA	AT
GG	GG	CG	CG	--	CG	GG	GG	CG	GG	GG	CG	CG
CC	CC	CT	CT	TT	CT	CC	CC	CT	CC	CC	TT	CT
AA	AA	AG	AG	GG	AG	AA	AA	AG	AA	AA	GG	AG
AA	AA	AC	AC	CC	AC	AA	AA	AC	AA	AA	CC	AC
TT	TT	GT	GT	--	GT	TT	TT	TT	TT	TT	GG	GT
CC	CC	CT	CT	--	CT	CC	CC	CC	CC	CC	TT	CT
GG	GG	CG	CG	CC	CG	GG	GG	GG	GG	GG	CC	CG
TT	TT	GT	GT	--	GT	TT	TT	TT	TT	TT	GG	GT
TT	TT	CT	CT	CC	CT	TT	TT	TT	TT	TT	CC	CT
--	AA	--	AG	--	AG	AA	AA	AA	--	AA	GG	AG
--	--	AC	AC	--	--	AA	AA	--	AA	AA	CC	AC
--	CC	CT	CT	--	--	--	CC	CC	CC	CC	--	CT
--	AA	AC	AC	AC	AC	AA	AA	AA	AA	AA	CC	AC
--	AA	AG	AG	GG	AG	AA	AA	AA	AA	AA	GG	AG
--	GG	GT	GT	--	GT	GG	GG	GG	GG	GG	TT	--
--	GT	GT	GT	GT	--	GG	GG	GG	--	GT	TT	--
GG	AG	AG	AG	AA	AG	GG	GG	GG	GG	GG	AA	AG
--	CT	CT	CT	--	CT	CC	CC	CC	CC	CC	TT	CT
--	CT	CT	CT	--	CT	CC	CC	CC	CC	CC	TT	--
--	AG	AG	AG	--	AG	--	AA	AA	AA	AA	GG	AG
--	CT	CT	CT	CT	CT	CC	CC	CC	CC	CC	TT	CT
--	CT	CT	CT	CT	CT	TT	TT	TT	TT	TT	CC	CT
--	AG	AG	AG	AG	AG	GG	GG	GG	GG	GG	AA	AG
CC	CT	CT	CT	CT	CT	--	CC	CC	CC	CC	TT	CT
TT	CT	TT	CT	--	CT	TT	TT	TT	TT	TT	CT	TT
CC	CT	CC	CT	CT	--	CC	CC	CC	CC	CC	TT	TT
AA	GG	AA	AG	AG	GG	AA	AA	AA	AA	AA	GG	AG
--	CC	--	AC	AC	CC	AA	AA	AA	AA	AA	CC	AC
CG	CC	CG	CG	CC	CC	CG	CG	CG	CG	CG	CC	CC
--	TT	GG	GT	--	TT	GG	GG	GG	GG	GG	TT	TT
AG	AA	GG	AG	AG	AA	GG	GG	GG	GG	GG	AA	AG
CT	CC	TT	CT	CT	CT	TT	TT	TT	TT	TT	CC	CT
AG	GG	AG	AG	GG	GG	AG	AG	GG	AG	AG	GG	GG
--	AA	GG	AG	--	AG	GG	GG	AG	GG	GG	AG	AG
--	CC	GG	GG	CG	CG	GG	CG	--	GG	GG	CG	CG
CT	TT	CT	CT	--	TT	CT	TT	CT	CT	CT	TT	TT
--	GG	TT	TT	GG	GT	TT	GT	GT	TT	TT	GT	GT
GG	GG	--	GG	GG	AG	AG	GG	GG	GG	AG	AG	GG
--	GG	AG	AG	AG	GG	GG	GG	AG	GG	AG	AG	GG
AC	AA	AC	AC	AC	AC	AC	AA	AA	AA	AC	AC	AA

AT	TT	TT	AT	AT	AT	AT	AT	AT	TT	AT	AT	TT
AT	TT	TT	AT	AT	AT	AT	AT	AT	TT	AT	AT	TT
--	AA	AA	AT	--	AT	TT	AT	--	--	AT	AT	AA
--	TT	TT	CT	TT	CT	TT	CC	TT	TT	CT	CT	CC
AG	GG	GG	AG	AG	AG	AG	AA	GG	GG	AG	AG	AG
AC	AA	AA	AC	AC	AC	AC	CC	AA	AA	AC	AC	AC
CT	TT	TT	CT	CT	CT	CT	CC	TT	TT	CT	CT	--
AG	GG	GG	GG	--	AG	GG	AA	GG	GG	AG	AG	AG
GT	GG	GG	GG	--	GT	GT	TT	GG	GG	GT	GG	GT
AG	GG	GG	GG	--	--	AA	AA	GG	GG	AA	GG	AA
CC	CC	CC	CC	CG	CG	CG	CG	CC	CC	CG	CC	CG
GG	GG	GG	GG	AG	AG	AG	AG	GG	GG	AG	GG	AG
--	CG	CC	CG	CG	CG	CC	GG	CC	CC	GG	CC	GG
AG	AG	AG	AG	AG	AG	--	AG	AA	AA	GG	AA	GG
--	AG	AG	AG	--	AG	--	AG	--	GG	AA	GG	AA
AG	AG	AG	AG	AG	AG	AG	AG	AA	AA	GG	AA	GG
--	GG	GG	GG	GT	GT	GT	GT	GG	GG	GT	GG	GT
--	AG	AG	AG	AG	AG	AG	AG	GG	GG	AA	GG	AA
TT	TT	TT	TT	GT	GT	GT	GT	TT	TT	GT	TT	GT
--	CG	CG	CG	--	CG	CG	CG	GG	GG	CC	CG	CG
TT	CT	CT	TT	CT	CT	--	CT	TT	CT	CT	CT	TT
--	CG	CG	GG	--	CG	--	CG	CG	CG	CC	CG	GG
--	CC	CC	CC	CG	CC	CG	CC	CG	CC	CG	CC	CC
AA	AC	AC	AA	AC	AC	AA	AC	AA	AC	AC	AC	AA
--	CT	CT	CC	CT	CT	CC	CT	CT	CC	CT	CT	CC
AG	AG	AG	AA	--	AG	AA	AG	AG	AA	AG	AG	AA
--	CT	CT	CC	--	CT	CC	CT	CT	CC	CT	CT	CC
--	GG	GG	GG	--	GG	CG	GG	GG	GG	CG	GG	--
CT	CT	CT	CC	--	CT	CC	CT	CT	CC	CC	CT	--
AG	AG	AG	AA	--	AG	GG	GG	GG	AA	AG	AG	GG
GT	GT	GT	GG	--	GT	GG	GT	--	GG	GG	GT	GT
--	GT	GT	TT	GT	--	GT	GG	GT	TT	GT	TT	GT
CG	CG	--	GG	--	CG	--	CG	CG	--	GG	--	CG
CG	CG	--	GG	--	CG	CG	CC	CG	GG	CG	GG	CC
--	--	AG	GG	GG	AG	GG	AG	AG	GG	GG	GG	AG
--	TT	CT	TT	CT	CT	CT	CC	TT	TT	CT	--	CC
GT	GG	GT	GG	--	GT	GT	TT	GT	GG	GT	GG	TT
--	GG	AG	GG	GG	AG	GG	AG	AG	GG	GG	GG	AG
AG	AA	AG	AA	AG	AG	AG	GG	AG	AG	AG	AA	GG
CT	TT	--	TT	--	CT	CT	CC	CT	CC	CT	TT	CC
AT	TT	AT	TT	TT	AT	--	AT	AT	--	TT	--	AT
GT	--	GT	TT	--	GT	--	GT	GT	GT	TT	TT	GT
CT	CC	CT	CC	CT	CT	TT	TT	CT	--	CT	CC	--
AG	AA	AG	AA	AA	AA	AA	AG	AG	AG	AA	AA	AG
AT	TT	AT	TT	AT	TT	--	AA	AT	AT	AT	TT	--
AG	AA	AG	AA	AA	AA	AA	AG	AG	AG	AA	AA	AG
CT	TT	CT	TT	--	TT	TT	CT	CT	CT	TT	TT	CT

AT	TT	AT	TT	--	TT	--	AT	AT	AT	TT	TT	AT
GT	GG	GT	GG	GT	GG	GT	TT	GT	GT	GT	GG	TT
AG	AA	AG	AA	AG	AA	AG	GG	AG	--	AG	AA	GG
--	CC	CT	CC	CT	CC	--	TT	CT	CT	CT	CC	TT
--	CC	CT	CC	--	CT	CT	TT	CT	CT	CT	CC	TT
CT	CC	CT	CC	CT	CT	CT	TT	CT	CT	CT	CC	TT
GT	GG	GT	GG	--	GT	GT	TT	GT	GG	GT	GT	TT
--	CC	CG	CC	--	CG	CG	GG	CG	CC	CG	CG	GG
AC	CC	AC	CC	--	AC	AC	AA	AC	CC	AC	--	AA
AG	AA	AG	AG	GG	AG	AA	GG	AG	AA	AG	AG	GG
--	AG	AG	AG	--	AG	--	AA	AG	GG	AG	AG	AA
CT	TT	TT	CT	--	CT	CC	CC	CC	CT	TT	CC	TT
AC	AA	AA	AC	--	AC	--	CC	CC	AC	AA	CC	AA
--	TT	--	GT	--	TT	GT	GT	TT	TT	GT	GT	TT
CG	GG	GG	GG	--	GG	CG	CG	CG	CG	CG	CG	GG
--	GG	--	AG	AG	GG	--	AA	AG	AA	AG	AA	AG
CT	TT	TT	TT	CT	TT	CT	CT	CT	CT	CT	CT	CT
--	TT	TT	GT	GT	TT	GT	GT	TT	GT	TT	GT	TT
CT	CC	CC	CC	CT	CC	CT	CT	CT	CC	CT	CT	CT
AC	AC	AA	AC	AC	AC	AC	AC	AA	--	AA	AC	AA
AG	AG	AA	GG	--	AG	GG	AG	AG	AG	AG	GG	AG
--	AG	AA	AG	--	AG	--	AA	AA	AG	AA	AG	AA
--	GT	TT	GG	GG	GT	GG	GT	GT	GT	GG	GG	GT
CT	CT	--	TT	--	CT	TT	TT	CT	CT	TT	TT	CT
AC	AC	AA	CC	--	AC	AC	CC	AC	AA	CC	CC	AC
AT	AT	--	AA	AA	--	AT	AA	AT	TT	AA	AT	AT
--	GG	GT	GG	GG	GT	GT	GG	GT	GT	GG	GT	GT
--	AG	AG	AA	--	AG	AA	AA	AA	--	AA	AA	AA
CG	CG	CC	GG	--	--	CC	GG	CG	CC	GG	CG	GG
AG	AG	AA	GG	--	AA	AA	GG	AG	--	GG	AG	AG
CG	CC	GG	GG	CG	CG	--	CC	CC	CC	CC	GG	CG
AG	--	GG	GG	AG	AG	AG	AA	AA	AA	AA	--	--
--	AA	AG	AG	AG	--	AG	AA	AA	AA	AA	AG	--
--	AA	AT	AA	AA	AT	AA	AA	AT	AA	AT	AA	AT
AG	AA	AG	AG	--	AA	AG	AA	AA	AA	AA	AG	AA
--	AG	--	AA	AA	AG	AA	AG	AG	AG	AG	AA	AG
AC	AC	AC	AC	AC	AC	AC	AC	AC	AA	AC	AC	AA
--	AC	CC	CC	--	AC	--	AC	AC	CC	CC	CC	CC
--	AA	--	AC	AC	AC	--	AC	--	CC	AC	AC	CC
--	TT	GT	GT	GT	GG	TT	GT	GT	--	GT	GT	GG
TT	TT	AT	AT	--	AA	TT	AT	AA	AA	AT	--	AA
--	AG	AA	AA	AG	AA	AG	AG	AG	AA	AG	AA	AA
--	AC	CC	CC	CC	CC	AC	AC	AC	CC	AC	CC	CC
AT	TT	AT	AT	--	--	TT	AT	AT	AA	AT	AA	AA
CT	TT	CT	CT	--	CC	TT	CT	CT	CC	CC	CC	CC
AG	AA	AA	AG	AG	GG	AA	AG	AG	AG	GG	GG	GG
--	TT	TT	CT	--	CC	TT	CT	CT	CT	CC	CC	CC

--	CC	CC	CT	--	TT	CC	CT	TT	CT	TT	TT	TT
--	AA	AA	AG	--	GG	--	GG	AA	AG	AG	GG	GG
TT	TT	CT	CT	CT	CC	TT	CC	CT	CT	CT	CC	CC
--	CC	CT	CT	CT	TT	--	TT	CT	CT	CT	TT	TT
TT	TT	CT	CT	CT	CC	--	CC	CT	CT	CT	--	CC
--	AA	AG	AG	AG	GG	--	GG	AG	AG	AG	GG	GG
--	CC	CT	CT	CT	--	CC	TT	CC	CT	CT	TT	TT
--	AA	AG	AG	AG	--	AA	GG	--	AG	AG	GG	GG
--	GG	CG	CG	--	CC	--	CC	--	CG	CG	CC	CC
--	CC	AC	AC	AC	AA	CC	AA	AC	--	AC	AA	--
--	CT	CT	CT	CT	CC	TT	CC	CT	CT	CT	CC	CC
--	CT	CT	CT	CT	CC	TT	CC	TT	CC	CT	CC	CC
--	AT	AT	AT	AT	TT	AA	TT	AT	--	AT	TT	TT
--	--	CT	CT	CT	CC	TT	CC	CT	CT	CT	CC	CC
--	AC	AC	AC	AC	AA	CC	AA	AC	AC	AC	AA	AA
--	GT	GT	GT	GT	TT	GG	TT	GT	GT	GT	TT	TT
--	CT	CT	CT	--	TT	--	TT	CT	CT	CT	--	TT
--	CG	CG	CG	CG	GG	CC	GG	CG	CG	CG	GG	GG
--	CG	--	CG	CG	GG	CC	GG	CG	CG	CG	GG	GG
--	AC	AC	AC	AC	CC	AA	CC	AC	AC	AC	CC	CC
--	CG	CG	CG	CG	GG	CC	GG	CG	GG	CG	CG	GG
--	AA	AT	AT	AT	--	TT	AT	TT	AT	AT	AA	AA
--	AA	AG	AA	AG	AA	AG	AG	GG	AG	AG	--	AG
--	AA	AG	AA	AG	AA	AG	AG	AG	AG	AA	AA	AG
--	AG	AA	GG	AG	GG	AA	AG	GG	AG	--	GG	AG
--	AG	GG	AA	--	--	GG	AG	AA	AG	AA	AA	AG
--	AC	CC	AA	AC	AA	CC	AC	AC	AC	AA	AA	AC
AG	AG	GG	AA	AG	AA	GG	AG	AG	AG	AA	AA	AG
AG	AG	GG	AA	--	--	GG	AG	AG	AG	AA	AA	GG
--	GT	TT	GG	GT	GG	--	GT	GT	GT	GG	GG	GT
--	GT	--	TT	GT	TT	GG	GT	GT	GT	TT	--	TT
AC	AC	AA	CC	--	CC	AA	AC	AC	AC	CC	CC	AC
--	CT	CC	TT	--	TT	CC	CT	CT	CT	TT	--	CT
CT	CT	CC	TT	--	--	--	CT	--	CT	TT	--	TT
--	GT	TT	GG	GT	GG	TT	GT	GT	GT	GG	GG	GT
AT	AT	TT	AA	--	AA	TT	AT	AT	AT	AA	AA	--
CG	CG	CC	GG	CG	GG	CC	CG	CG	CG	GG	GG	CG
AC	AC	CC	AA	AC	AA	--	AC	AC	AA	AA	AA	AC
CT	CT	--	CC	CT	CC	TT	CT	CT	CT	CC	CC	CT
CG	CG	CC	GG	CG	--	CC	CG	CG	CG	GG	GG	GG
--	CT	TT	CC	--	--	TT	CT	--	--	CC	CC	CT
AT	AT	TT	AA	AT	AA	TT	AT	TT	AT	AA	AA	AT
--	CT	CC	TT	CT	TT	CC	CT	CT	CC	TT	TT	--
--	AG	AA	GG	AG	GG	--	AG	AG	AG	GG	GG	AG
AT	AT	TT	AA	AT	--	TT	AT	--	AT	AA	--	TT
AT	AT	TT	AA	--	AA	TT	AT	AT	AT	AA	AA	AT
AC	AC	CC	AA	AC	AA	CC	AC	AC	AC	AA	AA	AC

--	GT	GG	TT	GT	TT	GG	GT	GT	--	TT	TT	GT
AG	AG	--	GG	--	GG	--	AG	AG	AG	GG	GG	AG
GT	GT	TT	GG	GT	GG	TT	GT	GT	GT	GG	GG	--
--	AG	GG	AA	AG	AA	GG	AG	AG	AG	AA	AA	AG
--	CT	--	TT	CT	TT	CC	CT	CT	CT	TT	TT	CT
AG	AG	GG	AA	AG	AA	GG	AG	AG	AG	AA	AA	AG
CT	CT	CC	TT	--	TT	CC	CT	CT	CT	TT	TT	CC
AC	AC	AA	CC	--	CC	AA	AC	AC	--	CC	CC	AC
--	AG	GG	AG	--	AA	--	AG	--	AG	AA	AA	GG
CT	CT	CC	CT	CT	TT	CC	CT	CT	CT	TT	TT	CT
AG	AG	AA	AG	AG	GG	AA	AG	AA	AG	GG	GG	AG
CT	CT	TT	CT	CT	CT	TT	CT	--	TT	CC	CC	CT
--	GG	GT	GT	GT	GG	GT	GT	GT	--	GG	GG	GT
--	AG	GG	GG	AG	AG	AG	GG	AG	AG	GG	GG	--
--	TT	AT	AT	AA	AT	--	AT	AA	AT	TT	--	--
TT	TT	AT	AT	AA	AT	AA	AT	AA	AT	TT	TT	TT
--	CC	AC	AC	--	AA	AC	AC	AA	AC	CC	CC	CC
GG	GG	GT	GT	TT	TT	--	GT	TT	GT	GG	GG	GG
CT	TT	TT	CT	--	CT	TT	CT	CT	TT	TT	TT	CT
--	GG	--	AA	AG	AG	AG	AA	AG	--	GG	AG	AG
--	CG	--	GG	CG	CG	GG	GG	CG	GG	CG	GG	GG
--	CT	--	TT	TT	CT	TT	TT	--	TT	CT	TT	CT
GT	GT	GT	GG	GG	GG	GT	GG	GG	GT	GT	GT	GG
--	GG	GG	AA	--	GG	--	AA	GG	GG	GG	AA	AG
--	AC	--	AA	--	--	AA	AA	AA	AA	AC	AA	AC
GG	CG	CG	GG	GG	CG	--	GG	CG	GG	CG	--	CG
TT	GT	GT	TT	--	GT	TT	TT	TT	--	GT	--	GT
CT	TT	TT	CC	CC	TT	CT	CC	CT	CT	TT	CT	TT
--	AA	AG	AG	--	AG	GG	AG	AA	GG	AG	GG	AG
--	CT	CT	CC	CC	CT	CC	CC	CT	CC	CT	CC	CC
CG	--	CG	CG	CG	CC	CG	CG	CG	--	CC	CG	CC
--	AC	AA	AC	AC	AA	AA	AC	AC	AA	AA	AA	AA
AG	AG	AG	GG	GG	AA	AG	GG	GG	GG	AA	AG	AA
--	TT	CT	TT	--	CT	CT	TT	TT	TT	CT	CT	CT
--	CG	GG	GG	--	CG	GG	GG	GG	GG	CG	GG	CG
AG	AG	AG	AA	AA	GG	AG	AA	AA	AA	GG	AG	GG
--	GG	CG	CG	--	GG	CG	CG	CG	CG	GG	CG	GG
--	CG	CG	CC	CC	--	CG	CC	CC	CC	GG	CG	GG
CT	CT	CT	CC	--	TT	CT	CC	CC	CC	TT	CT	TT
AG	AG	--	GG	GG	AA	AG	GG	GG	GG	AA	AG	AA
GG	AG	GG	AG	AG	GG	GG	AG	AG	AG	GG	GG	GG
CT	CT	CT	CC	CC	--	--	CC	CC	CC	TT	CT	TT
AG	GG	AG	GG	GG	AG	AG	GG	GG	GG	AG	AG	AG
AG	AG	AG	GG	GG	AA	AG	GG	GG	AG	AA	AG	AA
--	AC	AC	CC	--	--	AC	CC	CC	AC	AA	AC	AA
TT	AT	TT	TT	TT	AT	TT	TT	TT	TT	AT	TT	AT
AG	AG	AG	AG	--	GG	AG	AA	AA	AG	GG	AG	GG

AC	AC	AC	AC	AA	CC	AC	AA	AA	AC	CC	--	CC
--	AG	AG	AG	--	--	GG	AA	AA	AG	GG	AG	GG
--	--	AG	AA	--	AG	--	AA	AA	AA	AG	AG	AG
--	CT	--	CT	CC	CT	CC	CC	--	CT	--	--	CT
CC	CG	CC	CG	CC	CG	--	CC	CC	CG	CG	CC	CG
TT	CT	--	CT	--	CT	TT	TT	CT	CT	CT	--	--
--	CG	CG	GG	CG	GG	--	CG	GG	GG	CG	CG	GG
CC	CC	CC	CT	CC	CT	CC	CC	CT	CT	CC	CC	CC
--	GT	GT	GG	GT	GG	GT	GT	GG	--	GT	GT	GT

1-98	1-99	1-100	1-101	1-103	1-104	1-106	1-108	1-110	2-2	2-3	2-4	2-9
AA	AA	AA	AA	AA	AC	AC	AC	AC	AA	CC	AA	AA
AA	AA	AA	AA	AA	AG	AG	AG	AG	AA	GG	AA	AA
TT	CT	TT	TT	TT	CT	CT	CT	CT	TT	CC	TT	TT
GG	AG	GG	GG	--	AG	GG	GG	AG	GG	AG	GG	GG
--	GG	AA	AA	AA	AG	AG	AG	AG	AA	GG	AA	--
CG	CG	--	CG	CG	CG	GG	GG	CG	CG	GG	CG	CG
TT	CT	TT	TT	TT	CT	CT	CT	CT	TT	CC	TT	TT
GT	GT	TT	GT	TT	GT	GT	GT	TT	TT	GG	GT	TT
GT	GT	TT	GT	TT	GT	GT	GT	TT	TT	GG	GT	TT
GT	GT	TT	GT	TT	GT	GT	GT	TT	TT	GG	GT	TT
AG	GG	AA	AG	AG	AG	AG	AG	AA	AA	GG	AG	AA
CT	CC	CT	CT	CT	CT	CC	CC	CT	CT	CC	CC	CT
AC	CC	AA	AC	AC	AA	AC	AC	AA	AA	CC	AC	AC
AG	AA	AG	AG	AG	AG	AA	AA	AG	AG	AA	AA	AG
CC	CC	CT	CC	CC	CT	CT	CT	CT	CT	CC	CT	CC
AG	AA	GG	AG	AG	AG	AG	AG	GG	GG	AA	AG	--
AT	TT	AT	AT	AT	TT	TT	TT	AT	AT	TT	TT	AT
CC	CC	CT	CC	CC	CT	CT	CT	CT	CT	CC	CT	CC
GT	GT	GT	GT	--	TT	TT	TT	GT	TT	TT	TT	GT
CG	CG	CG	CG	CG	CC	CC	CC	CG	CC	CC	CC	CG
AT	AT	AA	AT	--	AT	AT	AT	AA	AA	TT	AT	AT
CT	CT	CC	CT	--	CC	--	CT	CC	CC	TT	CT	CT
AT	AT	TT	AT	AT	TT	AT	AT	AT	TT	AA	AT	AT
AT	AT	TT	AT	AT	TT	AT	AT	TT	TT	AA	AT	AT
AG	AG	GG	AG	AG	GG	AG	AG	GG	GG	AA	AG	AG
GT	GT	GT	GT	GT	GT	TT	TT	GT	GT	TT	TT	GT
GT	GT	GG	GT	GT	GG	GT	GT	GG	GG	TT	GT	GT
--	AT	AA	AT	AT	AA	AT	AT	AA	AA	TT	AT	AT
CT	CT	CC	CT	--	CC	CT	CT	CC	CC	TT	CT	CT
CG	CG	GG	CG	--	GG	CG	CG	GG	GG	CC	CG	CG
CT	CT	TT	CT	CT	TT	CT	CT	TT	TT	CC	CT	CT
AC	AA	CC	AC	AC	CC	AC	AC	CC	CC	AA	AC	AC
--	AG	GG	AG	AG	GG	AG	AG	GG	GG	AA	AG	AG
TT	GT	TT	GT	GT	TT	GT	GT	TT	TT	GG	GT	GT
AG	AG	GG	AG	AG	GG	AG	AG	GG	GG	AA	AG	AG
--	GT	GG	GT	--	GG	GT	GT	GG	GG	TT	GT	GT
AG	AG	AA	AG	AG	AA	AG	AG	AA	AA	GG	AG	AG
CT	CT	TT	CT	CT	TT	CT	CT	TT	TT	CC	CT	CT
AT	AT	TT	AT	AT	TT	AT	AT	TT	TT	AA	AT	AT
AG	AG	GG	AG	AG	GG	AG	AG	GG	GG	AA	AG	AG
AC	AC	CC	AC	AC	CC	AC	AC	CC	CC	AA	AC	AC
AG	AG	GG	AG	--	GG	AG	AG	GG	GG	AA	AG	AG

CT	CT	CC	CT	CT	CC	CT	CT	CC	CC	TT	CT	CT
CG	CG	GG	CG	CG	GG	CG	CG	CG	GG	CC	CG	CG
CT	CT	CC	CT	TT	CC	CT	CT	CT	CC	TT	CT	CT
CT	CT	TT	CT	CT	TT	CT	CT	CT	TT	CT	TT	CT
AC	AC	CC	AC	--	CC	AC	AA	AC	CC	AC	CC	AA
CT	CT	TT	CT	--	TT	TT	CT	CT	CT	CT	TT	CC
CT	TT	CC	CT	TT	CC	CC	CT	CT	CT	TT	CT	TT
--	AG	GG	AG	AG	GG	AA	GG	GG	AG	AG	AA	AA
TT	GT	GG	GT	TT	GG	TT	GT	GT	GT	TT	TT	TT
AA	AG	GG	AG	AA	AG	AA	AG	AG	AG	AA	AG	AA
CC	CT	CT	CT	--	CT	CC	CT	CT	TT	CC	CT	--
GG	CG	CG	GG	GG	CG	GG	CG	CG	CC	GG	CG	GG
GG	AG	AG	GG	GG	AG	GG	AG	AG	AA	GG	AG	GG
AA	--	AC	AA	--	AC	AA	AC	AC	CC	AA	AC	AA
GG	GG	AG	GG	GG	AG	GG	AG	AG	AG	GG	GG	GG
CT	CC	CT	CC	CC	CT	CC	CT	CT	TT	CC	CC	CC
CT	TT	CT	TT	TT	CT	TT	CT	CT	--	TT	TT	TT
CT	CC	CT	CC	CC	CT	CC	CT	CT	TT	CC	CC	CC
AG	GG	AG	GG	GG	AG	GG	AG	AG	AA	GG	GG	GG
AA	CC	AC	AC	AC	AC	CC	AC	AC	AA	CC	CC	CC
--	GG	AG	AG	AG	AG	GG	AG	AG	AA	GG	GG	GG
CG	CG	CG	CG	--	CC	CG	CG	CG	GG	CC	CC	CC
CG	CG	GG	CG	CG	GG	CG	CG	CG	CC	GG	GG	GG
AG	AG	AG	AG	AG	GG	AG	AG	AG	AA	GG	GG	GG
AC	AC	AC	AC	AC	AA	AC	AC	AC	CC	AA	AA	AC
CT	CT	CT	CT	--	CC	CT	CT	CC	TT	CC	CC	CT
CT	--	CT	CT	CT	CT	CT	CT	TT	CC	TT	TT	CT
AT	AT	AT	AT	AT	AT	AT	AT	AA	AT	AA	AA	TT
AG	AG	AG	AG	--	AG	GG	AG	AG	GG	AG	AG	GG
TT	TT	TT	TT	--	TT	AT	TT	AT	TT	AT	AT	TT
GT	GT	GG	GG	--	GT	TT	GT	GG	GG	GT	GT	TT
AG	GG	AG	AG	--	AG	GG	--	--	AG	GG	AG	GG
CT	TT	CT	CT	CT	CT	TT	CT	CT	CT	TT	CT	TT
AG	GG	AG	AG	AG	AG	GG	AG	AG	AG	GG	AG	GG
GT	GT	TT	TT	TT	GT	GG	GT	GT	TT	GT	GT	GG
GG	GG	GT	GT	GT	GT	GG	GT	GG	GT	GG	GT	GG
CT	CT	CC	CC	CC	CT	TT	TT	CT	CC	CT	CT	--
--	CC	CC	CC	CC	CC	TT	TT	CT	CC	TT	CT	TT
AA	AG	GG	GG	GG	GG	AG	AA	AG	GG	AA	AG	AA
GG	CC	CC	CG	CG	CC	CG	GG	CG	CC	GG	CG	GG
AA	GG	GG	AG	AG	GG	AG	AA	AG	GG	AA	AG	AA
CC	TT	TT	CT	--	TT	--	CC	--	TT	CC	CT	CT
CC	TT	CT	CT	CT	TT	TT	CC	CT	TT	CC	CT	TT
CG	CG	CG	CC	CG	CG	CG	CC	CC	CG	CC	CG	CG
AT	AT	AT	TT	AT	AT	AT	TT	TT	AT	TT	AT	AT
GG	GT	GG	GT	--	GT	GT	GG	GT	GT	GG	GG	GT
CC	CC	CC	CT	CC	CT	CT	CC	CT	CT	CC	CC	CT

CT	CT	CT	CT	TT	TT	CT	CT	TT	TT	CC	TT	TT
GG	GG	GG	GT	GG	GT	GT	GG	GT	GT	GG	GT	GT
CT	CT	CT	CT	CC	TT	CT	CT	--	TT	CC	CT	TT
TT	TT	TT	GT	TT	GT	GT	TT	GT	GT	TT	GT	GT
GG	GG	GG	CG	--	CG	CG	GG	CG	CG	GG	CG	CG
GG	--	AG	AG	--	AA	GG	AA	GG	AA	GG	AA	AG
AG	AG	AG	AG	AA	GG	AA	GG	AA	GG	AA	GG	AG
AG	AG	AG	AG	AG	GG	AA	AG	AA	GG	AA	GG	GG
CT	TT	CT	CT	TT	CT	TT	CT	TT	CC	TT	CC	CC
GG	GG	AG	AG	GG	AG	GG	AG	GG	AA	GG	AA	AA
GT	GG	GT	GG	--	GT	GG	GT	GG	TT	GG	TT	TT
GG	AG	GG	AG	AG	AG	AG	AG	AG	GG	AG	GG	GG
CT	TT	CT	TT	CT	CT	CT	CT	TT	CC	TT	CC	CC
CC	CC	CC	CC	--	AC	CC	AC	CC	AC	CC	AC	AC
CT	CT	CT	CT	CT	CC	CT	CC	CT	CC	CT	CC	CC
TT	GG	GT	GG	GT	GT	GT	GT	GG	TT	GG	GT	TT
CT	CC	CT	CC	CT	CC	CT	CC	CC	CT	CC	CC	CT
GG	GG	GG	GG	GG	CG	GG	CG	GG	CG	GG	CG	CG
CC	CC	CC	CC	--	AC	CC	AC	CC	AC	CC	--	AC
CT	TT	CT	TT	--	TT	CT	TT	TT	CT	TT	TT	CT
AG	AA	AG	AA	AG	AA	AG	AA	AA	AG	AA	AA	AG
AG	AA	AG	AA	AG	AG	AG	AG	AA	GG	AA	AG	GG
CT	CT	CT	CT	CT	CC	CT	CC	CT	CC	CT	CC	CC
AA	AA	AA	AA	--	AT	AA	AT	AA	AT	AA	AT	AT
CC	CC	CC	CC	--	CT	CC	CT	CC	CT	CC	CT	CT
AA	AA	AA	AA	--	AG	AA	AG	AA	AG	AA	AG	AG
CT	CC	CT	CC	CT	CC	CT	CC	CC	--	CC	CC	CT
AT	TT	AT	TT	AT	AT	AT	AT	TT	AA	TT	AT	AA
AT	TT	AT	TT	AT	AT	TT	AT	TT	AA	TT	AT	AA
CT	TT	CT	TT	CT	CT	TT	CT	TT	CC	TT	CT	CC
CC	CC	CC	CC	CC	CT	CC	CT	CC	CT	CC	CT	CT
GT	--	--	GG	--	GG	GG	GG	GG	GT	GG	GG	GT
CT	CC	CT	CC	CT	CC	CC	CC	CC	CT	CC	CC	CT
AA	AG	AA	AG	--	AG	AG	AG	AG	AA	AG	AG	AA
AG	GG	AG	GG	AG	AG	GG	AG	GG	AA	GG	AG	AA
AG	AA	AG	AA	AG	AA	AA	AA	AA	AG	AA	AA	AG
CT	CC	CT	CC	CT	CT	CC	CT	CC	TT	CC	CT	TT
--	CC	--	CC	AC	CC	CC	CC	CC	AC	CC	CC	AC
AA	TT	AT	TT	AT	AT	TT	AT	TT	AA	TT	AT	AA
--	AA	AA	AA	AA	AG	AA	AG	AA	--	AA	AG	AG
AC	AA	AC	AA	AC	AA	AA	AA	AA	AC	AA	AA	AC
AT	TT	AT	AT	AT	TT	TT	TT	TT	AT	TT	TT	AT
AA	AC	AA	AA	--	CC	AC	CC	AC	AC	AC	CC	AC
CC	CC	CC	CC	CC	AC	CC	AC	CC	AC	CC	AC	AC
--	AG	GG	GG	--	AG	AG	AG	AG	GG	AG	AG	GG
AC	AC	AC	AC	AC	CC	AC	CC	AC	CC	AC	CC	CC
AG	AA	AG	AG	AG	AA	AA	AA	AA	AG	AA	AA	AG

CC	CC	CC	CC	CC	CG	CC	CG	CC	CG	CC	CG	CG
CT	TT	CT	CT	CT	CT	TT	CT	TT	CC	TT	CT	CC
CT	CC	CT	CT	CT	CT	CC	CT	CC	TT	CC	CT	TT
CT	CC	CT	CT	--	CC	CC	CC	CC	CT	CC	CC	CT
AG	AA	AG	AG	--	AA	AA	AA	AA	AG	AA	AA	AG
CT	CC	CT	CT	CT	CT	CC	CT	CC	TT	CC	CT	TT
CT	TT	CT	CT	CT	TT	TT	TT	TT	CT	TT	TT	CT
AT	AA	AT	AT	AT	AT	AT	AT	AA	TT	AA	AT	TT
GG	GG	GG	GG	GG	AG	GG	AG	GG	AG	GG	AG	AG
AC	AA	AC	AC	AC	AA	AA	AA	AA	AC	AA	AA	AC
CT	CC	CT	CT	CT	CC	CC	CC	CC	CT	CC	CC	CT
TT	TT	TT	TT	GT	GT	TT	GT	TT	GT	TT	GT	GT
CT	TT	CT	CT	--	TT	TT	TT	--	CT	TT	TT	CT
CC	AC	CC	CC	CC	AC	AC	AC	AC	CC	AC	AC	CC
AA	AA	AG	AG	AG	AA	AA	AA	AA	AG	AA	AA	AG
GT	GG	GT	GT	--	GG	GG	GG	GG	GT	GG	GG	GT
TT	AT	TT	TT	TT	AT	AT	AT	AT	TT	AT	AT	TT
AG	AA	AG	AG	AG	AG	AA	AA	AA	AG	AA	AA	AG
CC	CC	CC	CC	--	CT	CC	CT	CC	CT	CC	CT	CT
CT	CC	CT	--	--	CT	CC	CC	CC	CT	CC	CC	CT
AC	AA	AC	AC	AC	AC	AA	AA	AA	AC	AA	AA	AC
--	CC	CT	CT	CT	CT	CC	CC	CC	CT	CC	CC	CT
TT	AA	TT	AT	AT	TT	AA	AT	AA	--	AA	AT	TT
AG	GG	AG	AG	AG	AA	GG	AG	GG	AA	GG	AG	AA
GG	GG	GG	GG	--	CG	GG	CG	GG	GG	GG	CG	CG
--	TT	CT	CT	--	CT	--	TT	TT	CT	TT	TT	CT
AG	GG	AG	AG	AG	AG	GG	GG	GG	AG	GG	GG	AG
AG	AA	AG	AG	AG	AG	AA	AA	AA	AG	AG	AA	AG
AA	AA	AA	AA	AA	AG	AA	AG	AA	AG	AA	AG	AG
GG	GG	GG	GG	GG	AG	GG	AG	GG	AG	GG	AG	AG
TT	TT	CT	TT	TT	CT	TT	TT	TT	TT	CT	TT	TT
GG	GG	GT	GG	--	TT	GG	GT	GG	GT	GT	GT	GT
TT	TT	TT	TT	--	GT	TT	GT	TT	GT	TT	GT	GT
GG	GG	AG	GG	GG	AA	GG	AA	GG	AG	AG	AG	AG
CC	CC	CT	CC	CC	CT	CC	CT	CC	CC	CT	CC	CC
TT	TT	GT	TT	TT	GT	TT	GT	TT	TT	GT	TT	TT
AG	AG	AG	AG	AG	GG	AG	GG	AG	GG	AG	GG	GG
AG	AA	GG	AG	AA	AG	AG	GG	GG	AG	AA	AG	AA
CC	CT	CC	CC	CC	CC	CT	CC	CC	CT	CT	CC	CT
AA	AA	AT	AA	AA	AA	AA	AT	AT	AT	AA	AA	AA
CG	CC	GG	CG	CC	CG	CC	CG	GG	GG	CC	CG	CG
AC	AA	AC	AC	AA	AC	AA	AA	AC	AA	AA	AC	AC
AG	AA	GG	AG	AA	AA	AA	AG	GG	--	AA	AG	AG
GG	AG	GG	AG	AG	AG	AG	GG	GG	GG	AG	AG	AG
CT	CT	CC	CT	CT	CT	CT	CC	CC	CC	CT	CT	CT
AG	GG	AA	GG	GG	GG	GG	AG	AA	AG	GG	GG	AG
CT	CC	CC	CC	CC	CC	CC	CC	CC	CC	CC	CC	CT

GG	GG	AG	GG	GG	GG	GG	AG	AG	AG	GG	GG	GG
GG	AG	AA	AG	--	AG	AG	AA	AA	AG	AG	AG	GG
CC	CC	CT	CC	CC	CC	CC	CT	CT	TT	CC	CC	CT
AT	AA	AT	AA	AA	AA	AA	TT	TT	TT	AA	AT	AT
AA	AA	AA	AA	AA	AA	AA	CC	CC	CC	AA	CC	AC
TT	CT	TT	TT	TT	TT	TT	CC	CC	CC	TT	CC	--
GG	AG	GG	GG	GG	GG	GG	AA	AG	AG	GG	AA	--
CT	TT	CC	CC	--	CC	CC	TT	CT	CT	CC	TT	--
--	CT	TT	TT	TT	TT	TT	CT	CT	TT	TT	CT	CT
GG	GG	AA	AA	--	AA	AA	GG	AG	AG	AA	GG	GG
AG	GG	AA	AA	GG	AG	AA	GG	AG	GG	AG	AG	AA
CT	TT	CC	CC	TT	CT	CC	TT	--	TT	CT	CT	CC
AG	AG	GG	GG	AG	GG	GG	AG	AG	GG	AG	GG	GG
CC	TT	CC	CC	--	TT	CC	TT	CT	CT	CT	TT	--
GG	CG	GG	GG	CG	CG	GG	CG	GG	CG	GG	CG	--
--	TT	CC	CC	--	TT	CC	TT	CT	CC	CT	TT	CC
CC	CC	CC	CC	CT	CT	CC	CT	CT	CC	CT	CT	CC
GG	AG	GG	GG	AA	AA	GG	AA	AG	GG	AA	AA	GG
CC	CT	CC	CC	--	TT	CC	TT	CT	CC	CT	TT	CC
AA	AG	AA	AA	GG	GG	AA	GG	AG	AA	AG	GG	AG
CC	CT	CC	CT	TT	TT	CC	TT	CT	CC	CT	TT	CT
GG	AG	AG	AG	AA	AA	GG	AA	AG	AG	AG	AG	AG
GG	GG	--	AG	GG	AG	GG	AG	AG	GG	GG	GG	AG
TT	GT	GT	GT	--	GG	GT	GG	GT	GT	GT	TT	GT
CG	CC	CG	CG	CG	CG	CG	CG	CG	CG	CC	CC	CG
CG	CC	CG	CG	CC	CC	CG	CC	CG	--	CC	CG	CG
CT	TT	CT	CT	--	TT	CT	TT	TT	CT	CT	CC	CT
CT	CC	CT	CT	CT	CT	CT	CC	CC	CT	CT	TT	CC
--	TT	AT	AT	AT	AT	AT	TT	TT	AT	AT	AA	TT
AG	AA	AG	AG	AG	AG	AG	AA	AA	AG	AG	GG	AA
CT	TT	CT	CT	--	CT	CT	CT	TT	CT	CT	CC	TT
CT	CT	CT	CT	TT	TT	CT	TT	CT	CT	TT	TT	CT
AC	CC	AC	AC	AC	AC	AC	AC	CC	AC	AC	AA	CC
GT	GT	GT	GT	GT	GT	GT	GT	GG	GG	GT	GT	TT
--	AG	AG	AG	AG	AG	AG	AG	GG	GG	AG	AG	AA
CT	CT	CT	CT	--	CT	CT	CT	--	CC	CT	CT	TT
AT	AT	AT	AT	--	AT	AT	AT	TT	TT	AT	AT	AA
AC	AC	AC	AC	AC	AC	AC	AC	AA	AA	AC	AC	CC
CG	CG	CC	CG	CC	CG	CG	CG	CC	CC	CG	CG	CG
AG	AA	AA	AG	AA	AG	AG	AG	AA	AA	AG	AG	--
CT	CT	CT	CT	CT	CT	CT	CT	TT	TT	CT	CT	CC
--	CT	CT	CT	CT	CT	CT	CT	TT	TT	CT	CT	CC
--	AG	AG	AG	AG	AG	AG	AG	GG	GG	AG	AG	AA
CC	CC	CT	CC	CT	CC	CC	CC	CC	CC	CC	CC	CT
AG	AG	AA	AG	--	AG	AG	AG	GG	GG	AG	AG	AA
AG	AG	AG	AG	--	AG	AG	AG	AA	--	AG	AG	GG
GT	GT	GT	GT	GT	GT	GT	GT	TT	TT	GT	GT	GG

CT	CT	CT	CT	CT	CT	CT	CT	CC	CC	CT	CT	TT
CG	CG	CG	CG	CG	CG	CG	CG	CC	CC	CG	CG	GG
GT	GT	GT	GT	--	GT	GT	GT	GG	GG	GT	GT	TT
AG	AG	AG	AG	AG	AG	AG	AG	GG	GG	AG	AG	AA
CT	CT	CT	CT	--	CT	CT	CT	TT	TT	CT	CT	CC
TT	TT	--	TT	GT	TT	TT	TT	TT	TT	TT	TT	GT
CG	CG	CG	CG	CG	CG	CG	CG	CC	CC	CG	CG	GG
--	AG	AG	AG	--	AG	AG	AG	--	GG	AG	AG	--
GG	GG	AG	GG	AG	GG	GG	GG	GG	GG	GG	GG	AG
AC	AC	AC	AC	--	AC	AC	AC	CC	CC	AC	AC	AA
CC	CC	CG	CC	CG	CC	CC	CC	CC	CC	CC	CC	CG
AG	AG	AG	AG	AG	AG	AG	AG	AA	AA	AG	AG	--
AC	AC	AC	--	--	AC	AC	AC	--	CC	AC	AC	AA
GT	GT	GT	GT	GT	GT	GT	GT	GG	GG	GT	GT	TT
AC	AC	AC	AC	AC	AC	AC	AC	AA	AA	AC	AC	CC
CC	CC	CT	CC	CT	CC	CC	CC	CC	CC	CC	CC	CT
GT	GT	GT	GT	GT	GT	GT	GT	GG	GG	GT	GT	TT
AG	AG	GG	AG	GG	AG	AG	AG	GG	GG	AG	AG	AG
CT	CT	CT	CT	--	CT	CT	CT	TT	TT	CT	CT	--
CC	CC	CT	CC	CT	CC	CC	CC	CC	--	CC	CC	CT
AT	AT	AT	AT	AT	AT	AT	TT	TT	TT	AT	AT	--
CG	CG	CG	CG	CG	CG	CG	GG	GG	GG	CG	CG	CC
GT	GT	GT	GT	GT	GT	TT	TT	TT	TT	GT	GT	GG
AG	AG	GG	AG	--	AG	GG	GG	GG	GG	AG	AG	AG
GT	GT	GT	TT	TT	GT	GT	GT	GT	GT	GT	GT	TT
CT	CT	CT	CC	CT	CT	TT	TT	TT	CT	CT	CT	CC
AC	CC	AC	CC	AC	AA	AA	AA	AA	AC	AC	AC	CC
CC	CC	CC	CC	CT	CT	CT	CT	--	CC	CC	CC	CC
CG	GG	CG	GG	CG	CC	CC	CC	CC	CG	CG	GG	CG
CT	CC	CT	CC	--	TT	CT	TT	TT	CT	CT	CC	CT
GT	GT	GT	GT	TT	TT	TT	TT	TT	GT	GT	GT	TT
TT	CT	TT	CT	--	TT	CT	TT	TT	TT	TT	CT	CT
AT	AT	AT	AT	--	TT	TT	TT	TT	AT	AT	AT	TT
CC	CC	CC	CC	AC	AC	AC	AC	AC	CC	CC	CC	AC
CC	--	--	CT	--	CC	CT	CT	CC	CT	CT	TT	CT
AA	AA	AA	AA	AG	AG	AG	AG	AG	AA	AA	AA	AG
GG	GG	--	GG	AG	AG	AG	AG	AG	GG	GG	GG	AG
CG	GG	GG	CG	CG	GG	CG	GG	CG	GG	GG	GG	CG
TT	CT	CT	CT	--	CC	CT	CT	CT	CC	CC	CC	--
CT	CT	CT	CT	TT	CC	CT	TT	CT	CC	CC	CC	TT
CT	CT	CT	TT	--	CC	CT	TT	TT	CC	CT	CC	TT
CT	CT	CT	CC	--	TT	CT	CC	CC	TT	CT	TT	CT
GT	TT	GT	TT	TT	GG	GT	TT	TT	GG	TT	GG	GT
CT	CT	CT	CC	--	TT	CT	CC	CC	TT	CC	TT	CT
AA	AT	AT	AA	AA	TT	AT	AA	AA	TT	AA	TT	AT
AA	AG	AG	AA	AG	GG	AG	AA	AA	AG	AA	GG	AG
CC	AC	AC	CC	AC	AC	AC	CC	CC	AC	CC	AA	AC

--	GG	AG	AA	AG	AG	AG	AA	AA	AG	AA	GG	AG
AA	GG	AG	AA	AG	AG	AG	AA	AA	AG	AA	AG	AG
TT	CC	--	TT	CT	CT	CT	TT	TT	CT	TT	CT	CT
GG	AA	--	GG	--	AG	AG	GG	GG	AG	GG	AG	AG
GG	TT	GT	GG	GT	GT	GT	GG	GG	GT	GG	GT	GT
CT	CC	CT	TT	--	CT	--	TT	--	CT	TT	CT	CT
--	AA	AA	AG	AG	AG	AG	GG	GG	AG	GG	AG	AG
AG	AA	AG	AG	--	AG	AG	GG	GG	AG	GG	AG	--
AG	--	AG	AG	AG	AG	AG	AA	AA	AG	AA	AG	AG
CG	CC	CG	CG	CG	CG	CG	GG	GG	CG	GG	CG	CG
AG	GG	AG	AG	AG	AG	AG	AA	AA	AG	AA	AG	AG
AC	CC	AC	AC	--	AC	AC	AA	AA	AC	AA	AC	AC
AG	AA	AG	AG	AG	AG	AG	GG	GG	AG	GG	AG	AG
AG	AA	AG	AG	AG	AG	AG	GG	GG	AG	GG	AG	AG
AA	TT	AT	AT	AT	AT	AT	AA	AA	AT	AA	AT	AT
CT	CC	CT	CT	CT	CT	CT	TT	TT	CT	TT	CT	CT
CT	CC	CT	CT	--	CT	CT	TT	--	CT	TT	CT	CT
CG	GG	CG	CG	CG	CG	CG	CC	CC	CG	CC	CG	CG
CT	TT	CT	CT	CT	CT	CT	CC	CC	CT	CC	CT	CT
CT	CC	CT	CT	--	CT	CT	TT	TT	CT	TT	CT	CT
CT	TT	CT	CT	CT	CT	CT	CC	CC	CT	CC	CT	CT
AT	TT	AT	AT	AT	AT	AT	AA	AA	AT	AA	AT	AT
AT	AA	AT	AT	AT	AT	AT	TT	TT	AT	TT	AT	AT
TT	TT	CT	CT	CT	CT	CT	CC	CC	CT	CC	CC	CT
CT	CC	CT	CT	CT	CT	CT	TT	TT	CT	TT	TT	CT
--	AA	AT	AT	AT	AT	AT	TT	TT	AT	TT	TT	AT
AG	GG	AG	AG	AG	AG	AG	AA	AA	AG	AA	AA	AG
GT	GG	--	GT	GT	GT	GT	TT	TT	GT	TT	TT	GT
CT	TT	CT	CT	CT	CT	CT	CC	CC	CT	CC	--	CT
CG	CC	CG	CG	CG	CG	CG	GG	GG	CG	GG	GG	CG
AG	GG	AG	AG	AG	AG	AG	AA	AA	AG	AA	AA	AG
CT	TT	CC	CT	CC	CT	CT	CC	CC	CT	CC	CC	CT
CT	TT	CC	CT	CC	CT	CT	CC	CC	CT	CC	CC	CT
AT	TT	AA	AT	AA	AT	AT	AA	AA	AT	AA	AA	AT
AG	AA	GG	AG	--	GG	AG	GG	GG	AG	GG	GG	AG
AG	AA	GG	AG	GG	GG	AG	GG	GG	AG	GG	GG	AG
GT	TT	GG	GT	GT	GG	GT	GG	GG	--	GG	GG	GT
AG	AA	GG	AG	AG	GG	AG	GG	GG	AG	GG	GG	GG
GG	AG	AG	GG	--	AG	AG	AG	AG	AG	AG	AG	AG
TT	CT	TT	TT	CT	CT	TT	TT	TT	TT	TT	CT	TT
GG	AA	AA	AG	AG	AG	GG	AG	AG	AG	GG	AA	GG
GG	AG	AG	AG	AG	GG	GG	GG	AG	AG	GG	AG	AG
GG	AG	AA	AG	--	AG	GG	GG	AG	AA	GG	AG	AG
CC	CG	GG	CG	GG	CG	CC	CC	CG	GG	CG	CG	CG
TT	CT	CC	CT	CC	CT	TT	TT	CT	CC	CT	CT	--
AC	CC	CC	AC	CC	CC	AC	AC	AC	CC	AC	CC	AC
AG	AG	AA	AA	--	AG	AG	AG	AA	AA	AA	AG	AG

AC	CC	CC	AC	CC	CC	AC	AC	AC	AC	AC	CC	AC
--	CC	AC	AC	AC	CC	CC	CC	AC	AC	AC	CC	CC
CT	TT	TT	CT	TT	TT	CT	CT	CT	TT	CT	TT	CT
AG	AG	AG	AG	AA	AG	GG	GG	AG	AA	AG	AG	AG
AG	AG	AG	AG	--	AG	AA	AA	AG	GG	AG	AG	AG
CT	CT	CT	CT	TT	CT	CC	CC	CT	TT	CT	CT	CT
--	TT	TT	CT	--	TT	TT	TT	CT	CT	CT	TT	CT
CG	CG	CG	CG	CG	CG	GG	GG	CG	CC	CG	CG	CG
AG	AG	AG	AG	AG	AG	GG	GG	AG	AA	AG	AG	AG
CT	CT	CT	CT	--	CT	TT	CC	CT	TT	CT	CT	CT
AT	AA	AA	AT	--	AA	AT	AA	AT	AT	AT	AT	AA
AA	AC	AC	AC	--	AA	AC	AA	AC	CC	AC	CC	AC
AG	GG	GG	AG	--	AG	GG	AG	AG	GG	AG	GG	GG
CT	CC	CC	CT	--	CT	CC	CT	CT	CC	CT	CC	CC
CT	CC	CC	CT	--	CT	CC	CT	CT	CC	CT	CC	CC
GG	GG	GG	GT	GG	GT	GG	GT	GT	GG	GT	GG	GG
CC	CC	CT	CT	CT	TT	CT	CC	TT	CT	TT	CT	CC
AG	AG	GG	AG	--	GG	GG	AG	GG	AG	GG	GG	AG
AA	AA	AG	AG	--	GG	AG	AA	GG	AG	GG	GG	AA
GG	CG	GG	CG	--	CG	GG	GG	CG	CG	CG	CG	GG
AA	GG	AG	AG	AG	GG	AG	AA	GG	AG	GG	GG	AA
GG	AA	AG	AG	AG	AA	AG	GG	AA	AG	AA	AA	GG
GT	TT	GT	TT	--	TT	GT	GT	TT	TT	TT	TT	GT
GG	AA	AG	AG	AG	AA	AG	GG	AG	AG	AA	AA	GG
AG	AA	AG	AG	--	AA	AG	GG	AG	AG	AA	AA	--
GT	GG	GT	GG	GG	GG	GT	GT	GT	GG	GG	GG	GT
CT	CC	CT	CC	CT	CT	CT	TT	CT	CT	CC	--	CT
CT	TT	CT	--	--	CT	CT	CC	CT	CT	CT	TT	CT
CC	CC	CC	CC	CT	CT	CC	CT	CT	CC	CT	CC	CT
AG	AG	AA	AA	AG	AG	AG	AG	AG	AG	AG	AA	AG
CT	CT	CC	CC	CT	CT	CT	CT	CT	CT	CT	CC	CT
AG	AG	AA	AA	AG	AG	AG	AG	AG	AG	AG	AA	AG
CT	CT	CC	CC	--	CT	CT	CT	CT	CT	CT	CT	CT
CG	CG	GG	GG	--	CG	CG	GG	CG	CG	CG	CG	CG
CT	CT	CC	CC	CT	CT	CT	CC	CT	CT	CT	CT	CT
AC	AC	AC	--	--	AA	AC	CC	AC	AA	AA	AA	AC
AG	AG	AA	AA	GG	GG	AG	AA	AG	--	GG	GG	AG
CT	CT	CC	CC	--	TT	CT	CC	CT	TT	CT	TT	CT
AG	AA	GG	GG	AA	AA	AG	GG	AG	AA	AG	AA	AG
AG	AG	GG	GG	AA	AA	AG	GG	AG	AG	AG	AA	AG
TT	TT	TT	CT	--	TT	CT	CT	CT	CT	TT	TT	CT
AA	GG	AA	AA	--	GG	AA	AA	AA	AG	AG	GG	AG
AG	AA	AG	AA	--	AA	AG	AG	AG	AA	AG	--	--
GT	TT	GT	TT	TT	TT	GT	GT	GT	TT	GT	TT	TT
GT	TT	GT	TT	--	TT	GT	GT	GT	TT	GT	TT	TT
AC	AA	CC	AC	--	AA	AC	CC	CC	AC	AC	AC	AC
AG	AA	GG	GG	--	AA	AG	GG	--	AG	AG	GG	AG

CG	CC	GG	GG	CC	CC	CG	GG	GG	CG	CG	GG	CG
--	CC	GG	GG	CC	CC	CG	GG	GG	GG	CG	GG	CG
TT	TT	CC	CC	--	TT	CT	CC	CC	CC	CT	CC	CC
CC	CC	TT	TT	CC	CC	CT	TT	TT	TT	CT	TT	TT
AA	AA	AT	TT	--	AA	AT	AT	TT	--	AT	TT	TT
AG	AG	AG	GG	AG	AG	AG	AG	GG	GG	GG	GG	GG
CC	CC	CC	CT	--	CC	CT	CT	--	TT	CT	TT	CT
--	TT	TT	CT	TT	TT	CC	CT	CT	CC	CT	CC	CT
CC	CC	CC	CT	--	CC	TT	CT	CT	TT	CC	TT	CT
GG	GG	GG	AG	AG	GG	AA	AG	AG	AA	GG	AA	AG
CC	CC	CC	CC	--	CC	CG	CG	CG	CG	CC	CG	CC
CG	CG	GG	CG	CG	GG	CG	GG	GG	CG	GG	CG	CG
--	GG	GG	GG	--	GG	GT	GT	GG	--	GG	GT	GG
CG	CG	CC	CC	CG	CC	CC	CG	CC	CG	CC	CG	CG
AG	AA	GG	AG	AG	GG	AG	AG	GG	AA	GG	AA	AA
AC	AC	AA	AA	AC	AA	AA	AC	AA	AA	AA	AC	AC
AT	AT	AA	AA	AT	AA	AA	AT	AA	AT	AA	AT	AT
AG	AA	GG	AG	AG	GG	AG	AG	GG	AA	GG	AA	AG
AT	AT	AA	AT	AT	AA	AA	AT	AA	TT	AA	AT	AT
AG	GG	GG	AG	AG	GG	GG	AA	GG	AA	GG	AG	AG
CC	CC	CC	AC	CC	CC	CC	AC	CC	AC	CC	AC	CC
CT	CC	CC	CT	TT	CC	CC	TT	CC	TT	CC	CT	CT
GT	TT	TT	GT	GG	TT	TT	GG	TT	GG	TT	GT	GT
GT	TT	TT	GT	GG	TT	TT	GG	TT	GG	GT	GT	GT
CT	CC	CC	TT	--	CC	CC	TT	CC	TT	CT	CT	CT
--	CC	CC	GG	GG	CC	CC	GG	CC	GG	CG	CG	CG
AG	GG	AG	AA	--	GG	GG	AA	AG	AA	AG	AG	AG
CT	CC	CC	TT	TT	CC	CC	TT	CT	TT	CT	CT	CT
GT	TT	TT	GG	--	TT	TT	GG	GT	GG	GT	GT	GT
CT	TT	TT	CC	CC	TT	TT	CC	CT	CC	CT	CT	CT
CG	GG	GG	CC	--	GG	GG	CC	CG	CC	CG	CG	CG
AG	GG	GG	AA	AA	GG	GG	AA	AG	AA	AG	AG	AG
AA	AA	AA	CC	CC	AA	AA	CC	AC	CC	AC	AC	AC
TT	TT	TT	CC	--	TT	TT	CC	CT	CT	CT	CT	CT
GG	GG	GG	AG	--	GG	GG	AG	AG	AG	AG	GG	AG
AC	AA	AA	CC	--	AA	AA	CC	--	CC	AC	AC	AC
CT	CC	CC	TT	TT	CC	CC	CT	CT	TT	CT	CT	CT
AG	GG	GG	AA	AA	GG	GG	AG	AG	AA	AG	AG	AG
CG	GG	GG	CC	--	GG	GG	CG	CG	CC	CG	CG	--
GT	GG	GG	TT	--	GG	GG	GT	GT	TT	GT	GT	GT
CT	CC	CC	TT	CT	CC	CC	CT	CT	TT	CT	CT	CT
TT	TT	TT	CT	CT	TT	TT	CT	CT	CT	CT	TT	CT
GG	AG	AG	GG	--	AG	AG	AG	AG	GG	AG	GG	GG
CG	CC	CC	GG	--	CG	CC	CG	CG	GG	CG	CC	GG
AT	AA	AA	AT	--	AA	AA	AA	AA	AT	AA	AA	AT
AA	AA	AA	AG	AG	AG	AA	AG	AG	AG	AG	AA	AG
CC	CC	CC	AC	AC	AC	CC	AC	AC	AC	AC	CC	AC

TT	TT	TT	CT	CT	CT	TT	CT	CT	CT	CT	TT	CT
CT	TT	TT	TT	TT	TT	CT	TT	CT	CT	TT	TT	CT
GG	GG	GG	GT	--	GT	AG	GT	AT	AT	GT	GG	AT
AA	AA	AA	AT	AT	AT	AA	AT	AT	AT	AT	AA	AT
GG	GG	GG	AG	--	AG	AG	AG	AA	AA	AG	AG	AA
CC	CC	AC	AC	AC	AC	AC	AC	AC	AA	AA	AC	AA
AA	AA	AA	AG	AG	AG	AA	AG	AG	AG	AG	AA	AG
GG	GG	AG	AG	--	AA	AG	AG	AA	--	AA	AG	AA
AA	AA	AG	AG	--	GG	AG	AG	GG	GG	GG	AA	AG
GG	GG	AG	AG	--	AA	AG	AG	AA	AG	AA	GG	AG
GG	GG	GT	GT	--	TT	GT	GT	TT	GT	TT	GG	GT
GG	GG	AG	GG	AG	AG	AG	GG	AG	GG	AG	GG	GG
CC	CC	CT	CT	--	TT	CC	CT	--	CT	TT	CC	CT
CC	CC	CG	CG	GG	GG	CC	CG	GG	CG	CG	CC	CG
TT	TT	TT	CT	CT	CT	TT	CT	CT	CT	CT	TT	CT
TT	TT	CT	TT	CT	TT	TT	TT	CT	TT	TT	TT	TT
CC	CT	CC	CT	CT	CC	CT	CT	CT	CC	CC	CC	CC
GT	GG	GG	GT	GT	GT	GG	GT	GG	GG	GT	GT	GG
AG	AG	GG	AA	--	AG	AG	AA	AG	GG	AG	AG	GG
AG	AG	AA	GG	GG	AG	AA	GG	AG	AA	AG	AG	AA
AA	AG	AA	--	AG	AA	AG	AG	AG	AA	AA	AA	AA
GT	GT	GG	TT	TT	GT	GT	TT	GT	GG	GT	GT	GG
AA	AG	AA	AG	AG	AA	AG	AG	AG	AA	AA	AA	AA
CC	CT	CC	CT	--	CC	CT	CT	CT	CC	CC	CC	CC
AA	AA	AA	AA	--	AG	AA	AA	--	--	AG	AG	AG
AG	AG	AG	AA	AA	AG	AG	AA	AA	GG	GG	AG	AG
AG	AG	AG	AA	AA	AG	AG	AA	AA	AG	AG	AG	AG
CG	CG	CG	CC	CC	CG	CG	CC	CC	CC	CG	CG	CG
CG	CG	CG	CC	--	CG	CG	CC	CC	CC	CG	CG	CG
AG	AG	AG	AA	--	GG	AG	AG	AA	AA	AG	GG	AG
GG	GG	GG	AG	--	GG	GG	GG	--	AG	GG	GG	AG
--	CT	CT	CC	CC	TT	CT	CT	CT	CC	CT	TT	CT
CC	AC	CC	AC	AC	CC	CC	CC	CC	AC	CC	CC	AC
CC	CG	CC	CG	CG	CC	CC	CC	CC	CG	CC	CC	CG
CC	AC	CC	AC	AC	CC	CC	CC	CC	CC	CC	CC	CC
AC	CC	CC	CC	CC	AA	AC	AC	AC	AC	AC	AA	AA
AG	AG	AG	AG	AG	AA	AG	AG	AG	AG	AG	AA	AA
AG	GG	GG	GG	GG	AA	AG	AG	AG	AG	AG	AG	AA
AG	AG	AG	AG	AG	GG	AG	AG	AG	AG	AG	GG	GG
CT	TT	TT	TT	--	CC	CT	CT	CT	CT	CT	CT	CC
CT	TT	TT	CT	--	CC	CT	CT	CT	CT	CT	CT	--
AA	--	AG	AG	AG	AA	AA	AA	AA	AA	AA	AG	AA
CC	CT	CT	CT	CC	CC	CC	CC	CC	CC	CC	CT	CC
GG	AA	GG	GG	GG	AG	--	GG	GG	AA	GG	AG	AG
CC	AC	CC	CC	CC	AC	AC	CC	CC	AC	CC	CC	CC
CC	CT	CC	CC	CC	CC	CT	CC	CC	CT	CC	CT	CT
AA	AG	AA	AA	--	AA	GG	AG	AG	GG	AG	AG	AG

AA	AG	AA	AA	AA	AA	AG	AA	AA	AG	AA	AG	AG
AA	AG	AA	AA	AG	AA	GG	AG	AG	GG	AG	AG	AG
CC	AC	CC	CC	AC	CC	AA	AC	AC	AA	AC	AC	AA
GG	AG	GG	GG	AG	GG	AA	AG	AA	AA	AG	AG	AA
TT	GT	TT	TT	GT	TT	GG	GT	GG	GG	GT	GT	GT
AA	AA	AA	AA	AG	AA	AG	AA	AG	AG	AG	AG	AG
TT	CT	TT	CT	CT	TT	CC	TT	CT	--	CT	CC	CC
--	CC	CC	CC	CT	CC	CT	CC	CC	CT	CT	CT	CT
GG	AG	GG	AG	AG	GG	AA	GG	AG	AA	GG	AG	AG
GG	CG	GG	CG	--	GG	CG	GG	CG	--	GG	GG	GG
TT	--	TT	CT	--	TT	TT	TT	CT	CT	TT	TT	--
AG	AG	AA	GG	AG	AA	AA	AA	AG	GG	AA	AA	AG
AG	AG	AA	GG	AG	AA	AA	AA	AG	GG	AA	AA	AG
CG	CC	CC	GG	--	CC	CC	CC	--	GG	CC	CC	CG
AG	AG	AA	GG	--	AA	AA	AA	AG	GG	AA	AA	AG
AA	AA	AG	AG	AG	AG	GG	AG	--	AG	AA	GG	AG
CC	CC	CT	CT	TT	CT	TT	CT	TT	CT	CC	CT	CT
AA	AA	AG	AG	GG	AG	GG	AG	GG	AG	AA	AG	AG
GG	GG	CG	CG	CC	GG	CC	CG	CC	CG	GG	CG	CG
AA	AA	AC	AC	--	AA	CC	AC	CC	AC	AA	AC	AC
GG	GG	AG	AG	AA	GG	AA	AG	AA	AG	GG	AG	AG
TT	GT	GT	GT	GG	TT	GG	GT	GG	GT	TT	GT	GT
AA	AG	AG	AG	GG	AA	GG	AG	GG	AG	AA	AG	AG
AA	AG	AG	AG	GG	AA	GG	AG	GG	AG	AA	AG	AG
GG	AG	AG	AG	AA	GG	AA	AG	AA	AG	GG	AG	AG
CC	AC	AC	AC	AA	CC	AA	AC	AA	AC	CC	AC	AC
GG	AG	AG	AG	AA	GG	AA	AG	AA	AG	GG	AG	AG
GG	AG	AG	AG	AA	GG	AA	AG	AA	AG	GG	AG	AG
CC	CT	CT	CT	TT	CC	TT	CT	TT	CT	CC	CT	CT
CC	CG	CG	CG	GG	CC	GG	CG	--	CG	CC	CG	CG
CC	AC	AC	AC	AA	CC	AA	AC	AA	--	CC	AC	AC
CC	CT	CT	CT	TT	CC	TT	CT	TT	CT	CC	CT	CT
GG	GT	GT	GT	TT	GG	TT	GT	TT	GT	GG	GT	GT
TT	TT	CT	CT	--	TT	CC	CT	CC	CC	TT	CT	CT
GG	CG	CG	CG	CG	GG	CC	CG	CC	GG	GG	CG	CG
AA	AT	AT	AT	TT	AA	TT	AT	TT	AT	AA	AT	AT
TT	--	CT	CT	CC	TT	CC	CT	CC	CT	TT	CT	CT
AA	AA	AG	AG	--	AA	GG	AG	GG	AG	AA	AG	AG
GG	AG	AG	AG	AA	GG	AA	AG	AA	AG	GG	AG	AG
--	AT	AT	AT	AA	TT	AA	AT	AA	AT	TT	AT	AT
AA	AG	AG	AG	GG	AA	GG	AG	GG	AG	AA	AG	AG
GG	AG	AG	AG	AA	GG	AA	AG	AA	AG	GG	AG	AG
GG	CG	CG	CG	CC	GG	CC	CG	CC	CG	GG	CG	CG
GG	AG	AG	AG	--	GG	AA	AG	AA	AG	GG	AG	AG
CC	CG	CG	CG	--	CC	GG	CG	GG	CG	CC	CG	CG
--	AG	AG	AG	--	AA	GG	AG	AG	AG	AA	AG	AG
CC	AC	AC	AC	AC	CC	AA	AC	AC	AC	CC	AC	AC

AA	AG	AG	AG	--	AA	GG	AG	AG	--	AA	AG	AG
GG	CG	--	CG	CC	GG	CC	CG	CG	CG	GG	CG	--
TT	CT	CT	CT	--	TT	CC	CT	CT	CT	TT	CT	CT
AA	AT	AT	AT	TT	AA	TT	AT	AT	AT	AA	AT	AT
TT	CT	CT	CT	--	TT	CC	CT	CT	CT	TT	CT	CT
TT	AT	AT	AT	--	TT	AA	AT	AT	AT	TT	AT	AT
AA	AT	AT	AT	--	AA	TT	AT	AT	AT	AA	AT	AT
CC	CG	CG	CG	CG	CC	GG	CG	CG	CG	CC	CG	CG
TT	CT	CT	CT	CC	TT	CC	CT	CT	CT	TT	CT	CT
GG	AG	AG	AG	AA	GG	AA	AG	AG	AG	GG	AG	AG
CC	AC	AC	AC	AA	CC	AA	AC	AC	AC	CC	AC	AC
GG	GT	GT	GT	TT	GG	TT	GT	GT	GT	GG	GT	GT
TT	CT	CT	CT	CC	TT	CC	CT	CT	CT	TT	CT	CT
CC	CG	CG	CG	GG	CC	GG	CG	CG	CG	CC	CG	CG
GG	TT	GT	GG	TT	GG	TT	GT	GT	GT	GG	GT	GT
CC	CT	CT	CC	TT	CC	TT	CT	CT	CT	CC	CT	CT
GG	AG	AG	GG	AA	GG	AA	AG	AG	AG	GG	AG	AG
CC	AC	AC	CC	AA	CC	AA	AC	AC	AC	CC	AC	AC
--	CT	CT	TT	--	TT	CC	CT	CT	CT	TT	CT	CT
CC	AC	AA	CC	AA	CC	AA	AC	AC	AC	CC	AC	AC
GG	AG	AA	GG	AA	GG	AA	AG	AG	AG	GG	AG	AG
TT	GT	GG	TT	GG	TT	GG	GT	GT	GT	TT	GT	GT
TT	GT	GG	TT	GG	TT	GG	GT	GT	GT	TT	GT	GT
AA	AG	GG	AA	GG	AA	GG	AG	AG	AG	AA	AG	AG
TT	CT	CC	TT	--	TT	CC	CT	CT	CT	TT	CT	CT
TT	CT	CC	TT	CC	TT	CC	CT	CT	CT	TT	CT	CT
GG	AG	AA	GG	AA	GG	AA	AG	AG	AG	GG	AG	AG
TT	CT	CC	TT	CC	TT	CC	CT	CT	CT	TT	CT	CT
CC	CT	TT	CC	TT	CC	TT	CT	CT	CT	CC	CT	CT
AA	AG	GG	AA	GG	AA	GG	AG	AG	AG	AA	AG	AG
TT	CT	CC	TT	CC	TT	CC	CT	CT	CT	TT	CT	CT
CT	TT	TT	CT	--	TT	CT	TT	CT	TT	CT	CT	TT
TT	CT	CC	TT	CC	CT	CT	CT	CT	CT	TT	TT	CT
GG	AG	AA	GG	AA	AG	AG	AG	AG	AG	GG	GG	AG
CC	AC	AA	CC	AA	AC	AC	AC	AC	AC	AC	CC	AC
CC	CC	CG	CC	CG	CC	CG	CC	CG	CC	CC	CC	CC
TT	GT	GG	GT	--	GT	GT	GT	GT	GT	GT	TT	GT
AA	AG	GG	AG	AG	AA	AG	AG	AG	AG	AG	AA	AG
CC	CT	CT	CT	--	CC	CT	CT	CT	CT	CT	CT	CT
GG	GG	AG	GG	AG	GG	AG	GG	AG	GG	GG	GG	GG
AA	AG	GG	AG	--	AA	AG	AG	GG	--	AG	AG	AG
CG	CG	GG	CG	--	CC	CC	CG	--	CG	CG	CG	CG
TT	TT	CT	TT	CT	TT	TT	TT	--	TT	TT	TT	CT
GG	--	TT	GT	TT	GG	GG	GT	GT	GT	TT	GT	TT
AG	AG	GG	AG	--	AG	GG	AG	AG	AG	AG	AG	AG
GG	GG	AG	GG	GG	GG	GG	AG	AG	GG	AG	AG	AG
AC	AC	AC	AC	--	AC	AA	CC	CC	AC	CC	--	CC

AT	AT	--	AT	--	AT	TT	AT	AA	AT	AA	AT	AA
AT	AT	AT	AT	--	AT	TT	AT	AA	AT	AA	AT	--
AT	AT	AT	AT	AT	AT	AA	AT	TT	TT	TT	AT	TT
CT	CT	CT	CT	--	CC	TT	CT	CT	CT	CC	CT	CC
AG	AG	AG	AG	GG	AA	GG	AG	AG	AG	AA	AG	AA
AA	AC	AC	AC	AC	CC	AA	AC	AC	AC	CC	CC	CC
TT	TT	CT	CT	CT	CC	TT	CT	CT	CT	CC	CT	CC
GG	GG	AG	AG	--	AA	AG	AG	AG	GG	AA	AG	AA
GG	GG	GG	GT	GT	TT	GT	GT	GT	GG	GT	GT	TT
GG	GG	GG	AG	AG	AG	AG	AG	AG	GG	AG	AG	AA
CC	CC	CC	CG	--	CC	CG	CG	CG	CC	CC	CC	CG
GG	GG	GG	AG	GG	GG	AG	AG	AG	GG	GG	GG	AG
CC	CC	CG	CG	--	CC	CG	CG	CG	CC	CG	CG	CG
AA	AA	AG	AG	AG	AA	AG	AG	AG	AA	AG	AG	AG
GG	GG	AG	AG	AG	GG	AG	AG	--	GG	AG	AG	--
AA	AA	AG	AG	AG	AA	AG	AG	GG	AA	AG	AG	AG
GG	GG	GG	GT	GG	GG	GT	GT	GT	GG	GG	GG	GT
GG	GG	AG	AG	AG	GG	AG	AG	AA	GG	AG	GG	AG
TT	TT	TT	GT	--	TT	GT	GT	GT	TT	TT	TT	GT
GG	CG	CG	CG	--	GG	CC	CG	CC	CG	CG	GG	CG
TT	TT	CT	CT	CT	CT	CT	CT	CT	TT	TT	CT	CT
CG	CG	CC	CG	--	CG	CG	CG	GG	CG	GG	CC	CC
CG	CG	CG	CC	--	CC	CC	CC	CC	CG	CC	CG	CC
AA	AA	AC	AC	AC	AC	AC	AC	AA	AA	AA	AC	AC
CC	CC	CT	CT	--	CT	CT	CT	CC	CC	CC	CC	CC
AA	AA	AG	AG	AG	AG	AG	AG	AA	--	AA	AA	AA
CC	CC	CT	CT	CT	CT	CT	CT	CC	CC	CC	CC	CC
CG	CG	CG	GG	GG	CG	GG	GG	--	CG	GG	CG	GG
CC	CT	CT	CT	--	CT	CT	CC	CC	CC	CC	CC	CC
AG	GG	GG	AG	AG	GG	AG	AA	AG	AG	AA	AG	AA
GG	GT	GT	GT	GT	GT	GT	GG	GG	GG	GT	GG	GG
GT	GG	GG	GT	--	GG	GT	GT	GT	TT	GT	GT	TT
GG	CG	CG	CG	CG	CG	CG	GG	GG	GG	CG	GG	GG
CG	CC	CC	CG	CG	CC	CG	CG	CG	GG	CG	CG	--
GG	AG	AG	AG	AG	AG	AG	GG	GG	GG	AG	GG	GG
CT	CC	CC	CT	CT	CC	CT	CT	CT	TT	CT	CT	TT
GT	TT	TT	GT	--	TT	GT	GT	--	GG	GT	GT	GG
GG	AG	AG	AG	AG	AG	AG	GG	GG	GG	AG	GG	GG
AG	GG	GG	AG	AG	GG	AG	AG	AG	AA	AG	AG	AA
TT	CC	CC	CT	CT	CC	CT	CT	CT	TT	CT	CT	TT
TT	AT	AT	AT	AT	AT	AT	TT	TT	TT	AT	TT	TT
TT	GT	GT	GT	GT	GT	GT	TT	TT	TT	GT	TT	TT
CC	TT	TT	CT	--	TT	CT	CT	CT	CC	CT	CT	CC
AA	AG	AG	AG	AG	AG	AG	AA	AA	AA	AG	AA	AA
TT	AA	AA	--	AT	AA	AT	AT	AT	TT	AT	AT	TT
AA	AG	AG	AG	AG	AG	AG	AA	AA	AA	AG	AA	AA
TT	CT	CT	CT	CT	CT	CT	TT	TT	TT	CT	TT	TT

--	AT	AT	AT	--	AT	AT	TT	TT	TT	AT	TT	TT
GG	TT	TT	GT	GT	TT	GT	GT	GT	GG	GT	GT	GG
AA	GG	GG	AA	AA	GG	GG	AG	AG	AA	AG	GG	--
CC	TT	TT	CC	--	TT	TT	CT	CT	CC	CT	CT	--
CC	TT	TT	CT	--	TT	TT	CT	CT	CC	CT	CT	CC
CC	TT	TT	CT	--	TT	TT	CT	CT	CC	CT	CT	CC
GG	TT	TT	GT	GG	TT	TT	GT	GT	GG	GG	GT	GG
CC	GG	GG	CG	CG	GG	GG	CG	CG	CC	CC	CG	CG
CC	AA	AA	AA	AC	AA	AA	AC	AC	CC	CC	AC	AC
AA	GG	GG	GG	AG	GG	GG	AG	AG	AA	AA	AG	AG
AG	AA	AA	AA	AG	AA	AA	AG	AA	GG	GG	GG	AG
CT	TT	CC	TT	CT	CT	CT	CC	TT	CC	CT	CT	TT
AC	AA	CC	AA	AC	AC	AC	CC	AA	CC	AC	AC	AA
TT	TT	GT	TT	--	GT	TT	GT	TT	GT	TT	GT	TT
GG	GG	CG	GG	CG	GG	--	GG	--	CG	CG	GG	GG
GG	GG	AA	GG	AG	AG	AG	AG	GG	AA	AG	--	GG
TT	TT	CT	TT	CT	TT	CT	TT	TT	CT	CT	CT	TT
TT	TT	GT	TT	--	GT	TT	GT	TT	TT	TT	GT	GT
CC	CC	CT	CC	CT	CC	CT	CC	CC	CT	CT	CT	CC
AA	AA	AC	AC	AC	AC	AA	AC	AA	AA	AA	AC	AC
AA	AA	GG	AG	--	AG	AG	AG	AA	AG	AG	GG	AG
AA	AA	AG	AG	AG	AG	AA	AG	AA	AA	AA	AG	AG
TT	GT	GT	GT	--	GT	GT	GT	TT	GT	GT	GG	GT
CC	CT	CT	CT	--	CT	CT	CT	--	TT	CT	TT	CT
AC	AC	AC	AC	CC	CC	AC	AC	AA	CC	AC	CC	AC
AT	AT	AT	AT	--	AA	AT	AT	TT	AA	AT	AA	AT
GG	GG	GG	GT	--	GT	GT	GG	GT	GG	--	--	GG
AG	AA	AG	AG	--	AA	AA	AG	AG	AA	AA	AA	AG
--	CG	CG	CC	GG	CG	CG	CG	CC	GG	CG	GG	CG
AG	GG	AG	AA	AG	AG	AG	AG	AA	GG	AG	AG	AG
CC	CG	GG	CG	CG	GG	CG	CG	CG	CC	CG	GG	CG
AA	AG	GG	AG	AG	GG	AG	AG	AG	AA	AG	GG	AG
AA	AG	AA	AG	--	AG	AA	AG	AG	AA	AA	AG	AA
AA	AA	AT	AA	--	AT	AT	AA	AA	AA	AT	AT	AT
AA	AG	AA	AG	AG	AG	AA	AG	AG	AA	--	AG	AA
AG	AA	AG	AA	AA	AA	AG	AA	AA	AG	AG	AA	AG
AA	AC	AC	AC	AC	AC	AC	CC	AC	AA	AC	CC	AC
CC	CC	AC	CC	CC	AC	AC	AC	CC	CC	AC	AC	AC
AC	AC	AC	AC	AC	AC	AC	AA	AC	CC	AC	AC	CC
GG	GT	GT	GT	GT	GG	GT	TT	GG	GG	GT	GT	GG
AT	AT	AT	AT	AT	AA	AT	TT	AT	AA	--	AT	AA
AA	AA	AG	AA	AA	AA	AG	AG	AG	AG	AG	AG	AA
CC	CC	AC	CC	CC	CC	AC	AC	AC	CC	AC	AC	CC
AT	AT	AT	AT	AT	AA	AT	TT	AT	AT	AT	AT	AT
CT	CT	CT	CT	CT	CT	CC	TT	CT	CT	CT	CT	CC
AG	AG	AG	AG	AG	AG	GG	AA	AG	AG	AG	AG	AG
CT	CT	CT	CT	CT	CT	CC	TT	CT	CT	CT	CT	CT

CT	CT	CT	CT	--	CT	TT	CC	CT	CT	CT	CT	CT
AG	AG	AG	AG	--	AG	GG	AA	AG	AG	AG	GG	AG
CT	CT	CT	CT	CT	CT	CC	TT	CT	CT	CT	CC	CT
CT	CT	CT	CT	CT	CT	TT	CC	CT	CT	CT	TT	CT
CT	CT	CT	CT	CT	CT	CC	TT	CT	CT	CT	CC	CT
--	AG	AG	AG	AG	AG	GG	AA	AG	AG	AG	GG	AG
CT	CC	CT	CT	CT	CT	TT	CC	CT	CT	CT	TT	CT
AG	AG	--	AG	AG	AG	GG	AA	AG	AG	AG	GG	AG
CG	CG	CG	CG	--	CG	CC	GG	CG	CG	CG	CC	CG
AC	AC	AC	AC	AC	AC	AA	CC	AC	AC	AC	AA	AC
CT	CT	CT	CC	CT	CT	CC	TT	CT	CC	CT	CC	CT
CT	CT	CT	CC	CT	CT	CC	TT	CT	CC	CT	CC	CT
AT	AT	--	TT	AT	AT	TT	AA	AT	TT	AT	TT	AT
CT	CT	CT	CC	CT	CT	CC	TT	CT	CC	CT	CC	CT
AC	AC	AC	AA	AC	AC	AA	CC	AC	AA	AC	AA	AC
GG	GT	GT	TT	GT	GT	TT	GG	GT	TT	GT	TT	GT
--	CT	CT	TT	CT	CT	TT	CC	CT	TT	CT	TT	CT
CG	CG	CG	GG	CG	CG	GG	CC	CG	GG	CG	GG	CG
CG	CG	CG	GG	CG	CG	GG	CC	CG	GG	CG	GG	CG
AC	AC	AC	CC	AC	AC	CC	AA	AC	CC	AC	CC	AC
--	CG	CG	GG	CG	CG	GG	CC	CG	GG	CG	GG	CG
AT	AT	AT	AA	AT	AA	AA	AT	AT	AT	TT	AT	AA
AG	GG	AG	AA	--	AA	AA	AG	AG	AG	GG	AG	AA
AG	GG	AG	AA	AG	AA	AG	AG	AG	AG	GG	AG	AA
AA	AA	AG	GG	AG	GG	AG	AG	AG	AG	AG	AG	GG
AG	GG	AG	AA	AG	AA	AG	AG	AG	AG	AG	AG	AA
AA	CC	AC	AA	AC	AA	AC	AC	AC	AC	AC	AC	AA
AG	GG	AG	AA	AG	AA	AG	AG	AG	AG	AG	AG	AA
AG	GG	AG	AA	AG	AA	AG	AG	AG	AG	AG	AG	AA
GT	TT	GT	GG	GT	GG	GT	GT	GT	GT	GT	GT	GG
GT	GG	GT	TT	GT	TT	GT	GT	GT	GT	GT	GT	TT
AC	AA	AC	CC	AC	CC	AC	AC	AC	AC	AC	AC	CC
CT	CC	CT	TT	CT	TT	CT	CT	CT	CT	CT	CT	TT
CT	CC	CT	TT	--	TT	CT	CT	CT	CT	CT	CT	TT
TT	TT	GT	GG	GT	GG	GT	GT	GT	GT	GT	GT	GG
TT	TT	AT	AA	AT	AA	AT	AT	AT	AT	AT	AT	AA
CG	CC	CG	GG	CG	GG	CG	CG	CG	CG	CG	CG	GG
AC	CC	AC	AA	AC	AA	AC	AC	--	AC	AC	AC	AA
CT	TT	CT	CC	CT	CC	CT	CT	CT	CT	CT	CT	--
CG	CC	CG	GG	CG	GG	CG	CG	CG	CG	CG	CG	GG
CT	TT	CT	CC	--	CC	CT	CT	CT	CT	CT	CT	--
AT	TT	AT	AA	AT	AA	AT	AT	AT	AT	AT	AT	AA
CT	CC	CT	TT	CT	TT	CT	CT	CT	CT	CT	CT	TT
AG	AA	AG	GG	--	GG	AG	AG	AG	AG	AG	AG	GG
AT	TT	TT	AA	AT	AA	AT	AT	AT	AT	--	TT	AA
--	TT	AT	AA	AT	AA	AT	AT	AT	AT	AT	AT	AA
AC	CC	AC	AA	AC	AA	AC	AC	AC	AC	AC	AC	AA

GT	GG	GT	TT	GT	TT	GT	GT	TT	GT	GT	GT	TT
AA	AA	AG	GG	AG	GG	AG	AG	GG	--	AG	AG	GG
GT	TT	GT	GG	GT	GG	GT	GT	GG	GT	GT	GT	GG
AG	GG	AG	AA	AG	AA	AG	AG	AA	AG	AG	AG	AA
CT	CC	CT	TT	--	TT	CT	CT	--	CT	CT	CT	TT
AG	GG	AG	AA	AG	AA	AG	AG	AA	AG	AG	AG	AA
CT	CC	CT	TT	--	TT	CT	CT	TT	CT	CT	CT	TT
AC	AA	AC	CC	--	CC	AC	AC	CC	AC	AC	AC	CC
AG	GG	AG	AA	--	AA	AG	AG	AA	AG	AG	--	AA
CT	CC	CT	TT	CT	TT	CT	CT	TT	CT	CT	CT	TT
AA	AA	AG	GG	AG	GG	AG	AG	GG	AG	AG	AG	AG
CT	CC	CT	CT	--	CT	CT	CT	CC	CT	TT	CC	CT
GG	GG	GT	GG	GG	GG	GG	GG	GG	GT	GT	GG	GG
AG	GG	GG	AG	--	AG	AG	AG	GG	GG	AG	GG	AG
AA	TT	AA	AT	--	AT	AT	AT	TT	AT	AA	TT	AT
AT	TT	AA	AT	--	AT	AT	AT	TT	AT	AA	TT	AT
AC	CC	AA	AC	AA	AC	AC	AC	CC	AC	AA	CC	AA
TT	GG	TT	GT	--	GT	GT	GT	GG	GT	TT	GG	--
CT	TT	TT	CT	--	TT	CT	TT	CT	TT	TT	CT	CT
AA	AG	GG	AG	--	GG	AG	AG	AA	AG	GG	AG	AA
GG	GG	GG	CG	--	CG	CG	GG	GG	GG	CG	CG	CG
TT	TT	TT	CT	CT	CT	CT	TT	--	TT	CT	CT	CT
GG	GG	GG	GT	GG	GT	GG	GT	GG	GT	GT	GG	GG
AA	AA	AA	GG	GG	GG	AA	AG	AA	GG	GG	AG	AG
--	AA	AA	AC	AC	AC	AA	AA	AA	AC	AC	AC	AC
GG	--	GG	CG	CG	CG	GG	GG	GG	CG	CG	CG	CG
--	TT	TT	GT	GT	GT	TT	TT	TT	GT	TT	GT	GT
CC	CC	CC	TT	TT	TT	CT	CT	CC	TT	CT	CT	CT
AG	AG	AG	AG	AG	AG	GG	GG	AG	AG	GG	AA	AA
CT	CC	CC	--	CT	CC	CC	CC	CC	CT	CC	CT	CT
CC	CG	CG	CG	--	CG	CG	CG	CG	CC	CG	CC	CG
AC	AC	AA	AA	--	AA	AA	AA	AC	AC	AA	AC	AC
AG	GG	GG	AG	--	AG	AG	AG	AG	AG	AA	AG	GG
TT	TT	TT	CT	--	CT	CT	CT	TT	TT	CT	TT	TT
CG	GG	GG	GG	CG	GG	GG	GG	GG	CG	GG	CG	GG
AG	AA	AA	AG	--	AG	AG	AG	AA	AG	AG	AG	--
GG	CG	CG	CG	--	CG	CG	CG	CG	GG	CG	GG	CG
CG	CC	CC	CG	--	CG	CG	CG	CC	CG	CG	CG	CC
CT	CC	CC	CT	--	CT	CT	CT	CC	CT	CT	CT	--
AG	GG	GG	AG	--	AG	AG	AG	GG	AG	AG	AG	GG
AG	AG	AG	GG	GG	GG	GG	GG	AG	AG	GG	AG	AG
CT	CC	CC	CT	--	CT	CT	CT	CT	CT	TT	CT	CC
GG	GG	GG	AG	AG	AG	AG	AG	GG	GG	AG	GG	GG
AG	GG	GG	AG	AA	AG	AG	AG	AG	AG	AA	AG	GG
AC	CC	CC	AC	AA	AC	AC	AC	AC	AC	AA	AC	CC
AT	TT	TT	TT	AT	TT	TT	TT	AT	AT	AT	AT	TT
AG	AA	AA	--	--	AG	AG	AG	AG	AG	GG	AG	AA

AC	AA	AA	AC	--	AC	AC	AC	AC	AC	CC	AC	AA
AG	AA	AA	AG	--	AG	AG	AG	AG	AG	GG	AG	AA
AA	AA	AA	AG	AG	AG	AG	AG	AA	AA	AG	AA	AA
CT	CC	CC	CC	--	CC	CC	CC	CT	CT	CT	CT	--
CG	CC	CC	CC	CG	CC	CC	CC	CG	CG	CG	CG	CC
--	TT	TT	TT	CT	TT	--	TT	CT	CT	CT	CT	TT
GG	CG	CG	GG	GG	CG	GG	CG	GG	GG	GG	GG	CG
CT	CT	CC	CT	CT	CC	CC	CC	CT	CT	CT	CT	CC
GG	GG	GT	GG	--	--	GT	GT	GG	GG	GG	GG	GT

2-12	2-13	2-14	2-15	2-18	2-27	2-28	2-29	2-30	2-31	2-33	2-35	2-36
AC	AC	CC	AC	AC	AC	AC	AA	CC	AC	CC	AC	AA
AG	AG	GG	AG	AG	AG	AG	AA	GG	AG	--	AG	AA
CT	CT	CC	CT	CT	--	CT	TT	CC	CT	CC	CT	TT
--	GG	AG	GG	AG	GG	GG	--	AG	GG	GG	AG	GG
--	GG	GG	AG	AG	AG	AG	AA	GG	AA	--	AG	AA
CG	GG	GG	GG	CG	GG	CG	CG	GG	CG	GG	GG	CG
TT	CT	CC	CT	CT	CT	TT	TT	CC	TT	--	CC	TT
TT	GT	GG	GT	GT	GT	TT	GT	GG	TT	--	GG	TT
TT	TT	GT	GT	GT	GT	TT	GT	GG	TT	--	GG	TT
GT	TT	GT	GT	GT	GT	TT	GG	GG	TT	--	GG	GT
AG	AA	AG	AG	AG	AG	AA	GG	GG	AA	--	AG	AG
CT	--	--	CC	CT	--	CT	CC	CC	CC	CC	CC	CT
CC	AA	AC	AC	AC	AC	AC	CC	CC	AC	CC	AC	AC
AA	AG	AG	AA	AG	AA	AA	AA	--	AA	--	AA	AG
CC	CT	CC	CT	CT	CT	CT	CC	CC	CT	CC	CT	CC
AA	GG	AG	AG	GG	GG	AG	AA	AG	AG	GG	AG	AG
TT	TT	AT	TT	AT	TT	TT	--	TT	TT	--	TT	AT
CC	CT	CC	CT	CC	CT	CT	CC	CC	CT	CC	CT	CC
TT	TT	GT	TT	GT	TT	TT	TT	TT	TT	GT	--	TT
CC	CC	CG	CC	CC	CC	CC	CC	CC	CC	--	CC	CC
TT	AT	--	AT	AA	AT	AT	AT	TT	AT	--	AT	TT
--	CT	CT	CT	CC	TT	CT	TT	TT	CC	--	CC	--
--	AT	AT	AT	TT	AT	AT	AT	AA	TT	--	AT	AA
AT	AT	AT	AT	TT	AT	AT	AT	AA	TT	--	TT	AA
AG	AG	AG	AG	GG	AG	AG	AG	AA	GG	--	GG	AA
--	TT	GT	TT	GT	TT	TT	GT	TT	GT	--	GT	TT
GT	GT	GT	GT	GG	GT	GT	GT	TT	GG	TT	GG	TT
AT	AT	AT	AT	AA	AA	AT	AT	TT	AA	AT	AA	AT
CT	CT	CT	CT	CC	CC	CT	CT	TT	CC	--	--	TT
CG	CG	CG	CG	GG	--	CG	CG	--	GG	--	GG	CC
CT	CT	CT	CT	TT	CT	CT	CT	CC	TT	--	TT	CC
AC	AC	AC	AC	--	AC	AC	AC	AA	CC	--	CC	--
AG	AG	AG	AG	--	AG	AG	AG	AA	GG	--	GG	--
GT	TT	GT	GT	TT	GT	GT	GT	GG	TT	--	TT	GG
AG	GG	AG	AG	--	AG	AG	AG	AA	GG	--	GG	AA
GT	GT	GT	GT	GG	--	GT	GT	TT	--	--	GG	--
AG	AG	AG	AG	--	AG	AG	AA	GG	AA	--	AA	GG
CT	CT	CT	CT	TT	CT	CT	CT	CC	TT	--	TT	CC
AT	AT	AT	AT	TT	AT	AT	AT	AA	TT	--	TT	AA
AG	AG	AG	AG	GG	AG	AG	AG	AA	GG	--	GG	AA
AC	AA	AC	AC	CC	AC	AC	AC	AA	CC	AC	CC	AA
AG	AG	--	AG	GG	AG	AG	AG	AA	GG	--	GG	AA

CT	CT	CT	CT	CC	TT	CT	CT	TT	CC	--	CC	TT
CG	CG	CG	CG	GG	CG	CG	CG	CC	GG	--	CG	CC
CT	CT	CT	CT	CC	CT	CT	CT	TT	CC	--	CT	TT
--	TT	CC	CT	TT	TT	CT	CT	CT	CT	CT	CT	CT
AA	CC	AA	AC	CC	CC	AC	AC	AC	AA	--	CC	AC
CC	TT	CC	TT	CT	TT	CT	CC	--	CC	--	TT	CT
TT	CC	TT	CC	--	CT	CT	CT	CT	TT	--	CC	CT
--	AG	AG	GG	AG	AA	AA	GG	AG	AG	--	AG	GG
GG	GT	GT	GG	GT	GT	TT	GG	GT	GT	GT	TT	GG
--	AG	AG	AG	--	AG	AA	GG	AG	AG	--	--	GG
CT	CT	CT	CT	CT	CT	CC	TT	CC	CT	--	--	TT
CG	CG	GG	CG	CG	GG	GG	CC	GG	CG	CG	GG	CG
AG	AG	GG	AG	AG	AG	GG	AA	GG	GG	--	GG	AG
AC	AA	AA	AC	AC	AC	AA	CC	AA	AC	AC	AA	AC
AG	GG	GG	AG	--	GG	GG	AG	GG	GG	--	GG	AG
CT	CC	CC	CT	CT	--	CC	TT	CC	CT	--	CC	CT
CT	TT	CT	CT	CT	TT	CT	CC	TT	--	--	--	CT
CT	CC	CT	CT	CT	CC	CT	TT	CC	CT	--	CT	CT
AG	GG	AG	--	AG	GG	AG	AA	GG	AG	--	AG	AG
AC	CC	AC	AC	AC	CC	AC	AC	CC	AC	AC	AC	AA
AG	AG	--	AG	AG	GG	AG	AG	GG	AG	AG	AG	AA
CG	CG	CC	CG	--	CC	CG	CG	CC	CG	--	CG	GG
CG	CG	GG	CG	CC	GG	CG	CG	GG	CG	--	CG	CC
AG	AG	GG	AG	AG	GG	AG	AG	GG	AG	--	AG	AA
AC	AC	AA	AC	AC	AA	AC	AC	AA	AA	--	AC	CC
CT	CT	CC	TT	TT	CC	TT	CT	CC	CC	--	CT	CT
CT	--	TT	CC	CC	TT	CC	--	--	TT	--	CC	TT
AT	AT	AA	TT	TT	AT	TT	AT	AA	AA	--	TT	AA
GG	GG	AG	GG	GG	AG	GG	AG	AG	AG	--	--	AG
AT	TT	TT	TT	TT	AT	TT	TT	--	AT	--	TT	AT
GT	GT	GT	--	GT	GT	GT	GT	GT	GT	--	GG	GT
--	GG	AG	GG	GG	AG	GG	AG	GG	AG	--	AG	AG
CT	TT	CT	TT	TT	CT	TT	CT	TT	CT	--	CT	CT
AG	GG	AG	GG	GG	AG	GG	AG	GG	AG	--	AG	AG
GT	GT	GT	GT	TT	GT	GT	GT	GT	GT	--	TT	GG
--	GG	GT	GG	GT	GT	GG	GT	GG	GT	--	GT	GG
CT	CT	CT	CT	CT	CC	CT	CC	CT	CT	--	CC	TT
CT	CT	CT	CT	CC	CT	CT	CC	CT	CT	--	CC	TT
AG	AG	AG	AG	AG	AG	AG	GG	AG	AG	AA	GG	AA
CG	CG	CG	CG	GG	CG	CG	--	GG	--	--	CC	GG
AG	GG	GG	AG	AG	AG	AG	GG	AA	AG	--	GG	AA
CT	TT	--	--	CT	TT	CT	TT	CC	CT	--	--	--
CT	TT	TT	--	CT	TT	CC	TT	CC	CT	--	CT	CC
CG	CG	CG	CC	CG	CG	CG	CG	CC	CG	--	CC	CC
AT	AT	AT	TT	AT	AT	AT	AT	TT	AT	--	TT	TT
GG	GT	GT	GT	GG	GG	GG	GT	GG	GG	GG	GT	GG
CC	CT	CT	CT	CC	CT	CC	CT	CC	CC	--	CT	CC

CT	TT	--	CT	CT	TT	CT	TT	--	CT	--	CT	CT
GG	GT	GT	GT	GG	GT	GT	GT	GG	GG	--	GT	GG
CT	TT	TT	CT	CT	TT	CT	CT	CC	CC	--	CT	CT
TT	GT	GT	GT	TT	GT	TT	GT	TT	TT	TT	GT	TT
GG	GG	CG	CG	GG	CG	GG	CG	GG	GG	--	--	GG
AG	AG	AG	AA	GG	--	AG	--	AG	AA	--	AG	AA
AG	AG	AG	GG	AG	AG	AG	AA	AG	AA	AA	AG	GG
AG	GG	AG	GG	AA	AG	AG	AA	AG	AA	AA	AG	GG
CT	CC	CT	CC	TT	CT	CT	TT	CT	TT	--	CT	CC
AG	AA	AG	AA	GG	AG	AG	GG	AA	GG	--	AG	AA
GT	TT	TT	TT	GG	GT	GT	GT	GT	GG	--	GT	TT
AG	GG	GG	GG	AG	GG	GG	GG	AG	AG	GG	GG	GG
CT	CC	CC	CC	TT	TT	CT	CT	TT	TT	CT	TT	CC
AC	AC	AC	AC	CC	CC	CC	CC	--	CC	CC	CC	AC
CC	CC	CC	CC	CT	CT	CT	CT	CT	CT	CT	CT	CC
GT	TT	TT	GT	GG	--	GT	GT	GG	GG	--	--	TT
CC	CT	CT	CC	CC	CC	CT	CT	CC	CC	CC	CC	CT
CG	CG	CG	CG	GG	GG	GG	GG	GG	GG	--	GG	CG
AC	AC	AC	AC	CC	--	CC	CC	CC	--	--	CC	AC
--	CT	CT	TT	TT	TT	CT	--	TT	TT	TT	TT	CT
AA	AA	AG	AA	AA	AA	AG	AG	AA	AA	--	AA	AG
AG	GG	GG	AG	AA	AA	AG	AG	AA	AA	AG	AA	GG
CC	CC	CC	CC	CT	CT	CT	CT	CT	CT	CT	CT	CC
--	AT	AT	AT	AA	AA	AA	AA	AA	AA	AA	AA	AT
CT	CT	CT	CT	CC	CC	CC	CC	CC	CC	--	CC	CT
AG	AG	AG	AG	AA	AA	AA	AA	AA	AA	--	AA	AG
--	CT	CT	CC	CC	CC	CT	CT	CC	CC	CT	CC	CT
--	AA	--	AT	TT	TT	--	AT	TT	TT	--	TT	AA
AT	AA	AA	AT	TT	--	AT	TT	--	TT	--	--	AA
CT	CC	CC	CT	TT	TT	CT	CT	TT	TT	--	TT	CC
CT	CT	CT	CT	CC	CC	CC	CC	CC	CC	CC	CC	CT
--	GT	GT	GG	GG	GG	GT	GT	GG	GG	--	--	GT
CC	CT	CT	CC	CC	CC	CT	CT	CC	CC	--	CC	CT
--	AA	AA	AG	AG	AA	AA	--	AG	AA	--	AG	AA
AG	AA	AA	AG	GG	GG	AG	AG	GG	GG	--	GG	AA
AA	AG	AG	AA	AA	AA	AG	AG	AA	AA	--	AA	AG
CT	TT	TT	CT	CC	CC	CT	CT	CC	CC	--	--	TT
--	AC	AC	CC	--	CC	CC	AC	CC	--	--	--	AC
AT	AA	AA	AT	TT	TT	TT	AA	TT	TT	--	TT	AA
AG	AG	AG	AG	AA	AA	AA	AA	AA	--	--	--	AG
AA	AC	AC	AA	AA	AA	AA	AC	AA	AA	--	AA	AC
TT	AT	AT	TT	TT	TT	TT	AT	TT	TT	--	TT	AT
CC	AC	AC	CC	AC	AC	AC	AA	AC	AA	--	AC	AC
AC	AC	AC	AC	CC	CC	CC	--	CC	CC	--	CC	AC
AG	GG	GG	AG	AG	AG	AG	GG	AG	GG	--	AG	--
CC	CC	CC	CC	AC	AC	AC	AC	AC	AC	--	AC	CC
AA	AG	AG	AA	AA	AA	AA	AG	AA	AG	AG	AA	AG

CG	CG	CG	CG	CC	CC	CC	CC	CC	CC	--	CC	CG
CT	CC	CC	CT	TT	TT	TT	CT	TT	CT	--	TT	CC
CT	TT	TT	CT	CC	CC	CC	CT	CC	CC	TT	CC	TT
CC	CT	CT	CC	CC	CC	CC	--	CC	CT	--	CC	--
AA	AG	AG	AA	AA	AA	AA	AG	AA	AG	--	AA	AG
--	TT	TT	CT	CC	CC	CC	CT	CC	CT	--	CC	TT
--	CT	CT	TT	TT	TT	TT	CT	TT	CT	CT	TT	CT
AT	TT	TT	AT	AA	AA	AA	AT	--	AT	--	AA	TT
AG	AG	AG	AG	GG	GG	GG	GG	GG	GG	--	GG	AG
AA	AC	--	AA	AA	--	AA	AA	AA	AC	--	AA	AC
--	CT	CT	CC	CC	CC	CC	CT	CC	CT	--	CC	CT
GT	GT	GT	GT	TT	TT	TT	TT	TT	TT	--	TT	GT
TT	CT	CT	TT	TT	TT	TT	CT	TT	CT	--	--	CT
AC	CC	CC	AC	AC	AC	AC	CC	AC	CC	--	AC	CC
AA	AG	AG	AA	AA	AA	AA	AG	AA	AG	--	AA	AG
GG	GT	GT	GG	--	GG	GG	GT	GG	GT	GT	GG	GT
AT	TT	TT	AT	--	AT	AT	--	--	TT	--	AT	TT
AA	AG	AG	AA	--	AA	AA	AG	AA	AG	--	AA	AG
CT	CT	CT	CT	CC	CC	CC	CC	CC	CC	--	CC	CT
CC	CT	CT	CC	CC	CC	CC	CC	CC	--	--	--	CT
AA	AC	AC	AA	AA	AA	AA	AC	AA	AC	--	AA	AC
CC	CT	CT	CC	CC	CC	CC	CT	CC	CT	CT	CC	CT
AT	TT	TT	AT	AA	AA	AA	TT	AA	AT	TT	AA	TT
AG	AA	AA	AG	GG	GG	GG	AG	GG	AG	AA	GG	AA
CG	--	CG	CG	--	GG	GG	--	GG	GG	--	GG	CG
TT	CT	CT	TT	--	TT	TT	CT	TT	CT	--	TT	CT
AG	AG	AG	GG	GG	GG	GG	AG	GG	GG	GG	GG	--
AG	AG	AG	AA	AA	AG	AA	--	AA	AG	AA	AA	AA
AG	AG	AG	AG	AA	AA	AA	AA	AA	AA	--	AA	AG
AG	AG	AG	AG	GG	GG	GG	--	GG	GG	--	GG	AG
CT	CT	TT	TT	TT	CT	CT	CT	TT	CT	--	TT	--
TT	TT	GT	GT	GG	GT	GT	GT	GT	GT	--	GG	GT
GT	GT	GT	GT	TT	TT	TT	TT	TT	--	--	TT	GT
--	AG	AG	AG	--	AG	AG	AG	AG	AG	--	GG	AG
CT	CC	CC	CC	CC	CT	CT	CT	CT	CT	--	CC	CC
TT	TT	TT	TT	TT	GT	GT	GT	GT	GT	--	TT	TT
GG	GG	GG	GG	AG	AG	AG	GG	AG	AG	AG	AG	GG
AG	AA	AG	AA	GG	GG	GG	AA	AA	AG	--	AA	AA
CT	CT	CC	CT	CC	CC	CT	CC	CT	CC	--	CT	CT
AT	AA	AA	AA	AT	AT	AT	AA	AA	AA	--	AT	AA
CG	CC	--	CC	GG	--	CG	CC	CC	CC	--	CG	CC
AA	AA	--	AA	AC	AC	AA	AA	AA	AC	AA	AA	AC
AG	AA	GG	AA	GG	--	AG	AA	AA	AG	--	AG	AG
GG	AG	GG	AG	--	GG	GG	AG	AG	AG	--	GG	AG
CC	CT	CC	CT	CC	CC	CC	CT	CT	CT	--	CC	CT
AA	GG	AG	GG	AG	AA	AG	GG	AG	AG	--	AA	AG
CT	CC	CC	CC	CC	CT	CC	CC	CT	CT	--	CT	CT

AG	GG	AG	GG	AG	AG	AG	GG	GG	GG	--	AG	GG
AG	AG	AA	AG	AA	AG	AA	AG	GG	GG	--	AG	GG
TT	CC	CT	CC	CT	TT	CT	CC	CT	CT	--	TT	CT
TT	AT	AT	AA	AT	TT	TT	AA	AT	TT	--	TT	AT
CC	AC	AC	AA	AC	CC	CC	AC	AA	AC	CC	AC	AA
--	CT	CT	TT	CT	CT	CC	CT	CC	--	--	CT	TT
--	AG	AG	GG	AA	--	AA	AA	AA	AG	AG	AG	GG
--	CT	CT	CC	TT	CT	TT	TT	TT	--	--	CT	CC
--	CT	TT	TT	CT	CT	CT	TT	CT	CT	--	TT	TT
GG	AG	AG	AA	GG	--	GG	AG	GG	AG	--	AG	AA
--	AG	--	AG	AA	--	GG	AG	--	AG	--	GG	AG
TT	CT	TT	CT	CC	--	TT	CT	CT	CT	--	--	CT
AG	AG	AG	AG	GG	GG	AG	GG	GG	GG	--	AG	GG
--	CT	TT	CT	CC	CT	TT	CT	CT	CT	--	TT	CT
CG	GG	CG	GG	GG	GG	CG	CG	CG	CG	--	CG	CG
--	CT	TT	CT	CC	CT	TT	--	TT	CT	--	TT	CT
CC	CT	CT	CT	CT	CC	CT	CC	CT	CC	--	CT	CC
AG	AG	AA	AG	AG	AG	AA	AG	AA	AG	AA	AA	AG
CT	CT	--	CT	--	--	TT	TT	TT	CT	--	TT	CT
AG	AG	GG	AG	AG	AG	GG	AG	GG	AG	--	GG	AG
CT	CT	TT	CT	TT	--	TT	CT	TT	CT	--	CT	TT
AG	AG	AA	AG	AA	AG	AG	AG	AA	AA	--	AG	AA
GG	AG	AG	AG	--	GG	GG	GG	AG	AG	AG	AG	AG
GT	GT	GG	GT	GG	GT	GT	GT	GG	GG	GG	GT	GG
CC	CG	CG	CG	CG	CC	CC	CC	CG	CG	--	CG	CG
--	CG	CG	CG	CC	CC	CG	CC	CC	CC	CC	CG	CC
CT	--	CT	CT	TT	TT	CC	CT	TT	TT	CT	CT	TT
CT	CT	CT	CT	CC	CC	TT	--	CC	CC	--	CT	CC
AT	AT	AT	AT	TT	TT	AA	AT	TT	TT	TT	AT	TT
AG	AG	AG	AG	AA	AA	GG	AG	AG	AG	--	GG	AA
TT	CT	CT	CT	CT	TT	CC	CT	CT	CT	--	CC	TT
CT	CT	TT	CT	TT	CT	TT	TT	TT	TT	CT	TT	CT
CC	AC	AC	AC	--	CC	AA	AC	AC	AC	--	AA	CC
--	GG	GT	TT	GG	--	TT	--	--	GT	--	TT	GT
GG	GG	AG	AA	GG	AG	AA	AA	GG	AG	--	AA	AG
--	CC	CT	TT	CT	CT	TT	TT	CC	CT	--	TT	CT
TT	TT	AT	AA	AT	AT	AA	AA	TT	AT	--	AA	AT
AA	AA	AC	CC	AC	AC	CC	CC	AA	AC	--	CC	AC
CC	CC	CC	CG	CC	CC	CG	CG	CC	CC	--	CG	CG
AA	AA	AA	AG	AA	AA	AG	AG	AA	AA	--	AG	AG
TT	TT	--	CC	--	CT	CC	CC	--	CT	--	CC	CT
TT	TT	CT	CC	--	--	CC	CC	TT	CT	--	--	CT
GG	GG	AG	AA	--	AG	AA	AA	GG	AG	--	AA	AG
--	CC	CT	CT	CT	--	CT	CT	CC	CT	--	CT	CC
GG	GG	AA	AA	GG	AG	AA	AA	--	--	--	AA	AG
AA	AA	AG	GG	AG	AG	GG	GG	AA	GG	--	--	AG
TT	TT	--	GG	--	GT	GG	GG	TT	GT	--	GG	--

CC	CC	CT	TT	CT	CT	TT	TT	CT	CT	--	--	CT
CC	CC	CG	GG	CG	CG	GG	GG	CG	CG	--	CG	CG
GG	GG	GT	TT	GT	GT	TT	TT	GT	GT	TT	GT	GT
GG	GG	AG	AA	AA	--	AA	AA	AG	AG	AA	AG	AG
--	TT	CT	CC	CC	CT	CC	CC	--	--	--	CT	CT
--	TT	GT	GT	GT	GT	GT	GT	TT	GT	--	TT	TT
CC	CC	CG	GG	CG	CG	GG	GG	CG	GG	GG	CG	CG
--	--	--	AA	GG	AG	AA	--	AG	AG	--	AG	AG
GG	GG	AG	AG	AG	GG	AG	AG	GG	GG	--	GG	GG
CC	CC	AC	AA	AC	AC	AA	AA	AC	AC	--	--	AC
CC	CC	CG	CG	CG	CG	CG	CG	CC	CG	CG	CC	CC
AA	AA	AG	GG	AG	AG	GG	GG	AG	AG	GG	AG	AG
CC	CC	AC	AA	AC	CC	AA	AA	AC	AA	--	AC	AC
GG	GG	GT	GT	GT	GT	TT	TT	GT	GT	--	GT	GT
AA	AA	AC	AC	AC	AC	CC	CC	AC	AC	--	AC	AC
CC	--	CT	CT	CT	CC	CT	CT	CC	--	--	CC	CC
GG	GG	GT	GT	GT	GG	TT	TT	GT	GT	--	GT	GT
GG	GG	GG	GG	GG	GG	AG	AG	AG	GG	AG	AG	AG
TT	TT	CT	CT	CT	TT	CC	CC	CT	CT	CC	CT	CT
--	CC	CT	CT	CC	CC	CT	CT	CC	CT	--	CC	CC
TT	TT	AT	AT	AT	TT	AA	AA	AT	AT	AA	AT	AT
GG	GG	CG	CG	CG	GG	CC	CC	CG	CG	--	CG	CG
TT	TT	GT	GT	GT	TT	GG	GT	GT	GT	GG	GT	GT
GG	GG	GG	GG	GG	GG	AG	AG	AG	GG	AG	AG	AG
TT	GT	TT	TT	TT	GT	--	GT	GT	TT	TT	GT	GT
CT	TT	CT	CT	CT	TT	CC	CT	CT	CC	CC	CT	CT
AC	AA	AC	AC	--	AA	CC	AC	AC	CC	CC	AC	AC
CT	CT	CT	CT	CT	CT	--	CC	CC	CC	CC	CC	CC
CG	CC	CG	CG	CG	CC	CG	CG	CG	GG	GG	CG	CG
CT	TT	CT	CT	CT	CT	CT	CT	CT	CT	CC	CT	CT
TT	TT	TT	TT	--	--	TT	GT	GT	GT	--	GT	GT
CT	TT	CT	CT	CT	CT	CT	TT	TT	TT	TT	TT	TT
TT	AT	TT	TT	TT	TT	TT	AT	AT	AT	--	AT	AT
AC	CC	AC	AC	AC	AC	AC	CC	CC	CC	--	CC	CC
CT	TT	CT	CT	CC	CT	CT	CT	--	CT	--	--	CT
AG	AA	AG	AG	AG	AG	AG	AA	AA	AA	AA	AA	AA
AG	GG	AG	AG	AG	AG	AG	GG	GG	GG	--	GG	GG
GG	GG	GG	CG	GG	GG	CG	CG	GG	CG	--	CG	CG
CC	CC	CT	TT	CT	CC	TT	TT	CT	TT	--	CT	CT
CC	CC	CC	CT	CT	CT	TT	CT	CT	TT	--	CT	CT
CT	CC	CT	CT	CT	CT	TT	CT	TT	TT	TT	CT	CT
CC	TT	CT	CT	CC	TT	CC	CT	CT	CC	--	CT	CT
TT	GG	GT	GT	TT	GT	TT	GT	GT	GT	--	GT	GT
--	CT	CT	CT	CC	--	CC	CT	CT	CT	--	CT	CT
AA	AT	AT	TT	AA	--	AT	AT	AT	AT	AA	AT	AT
AA	AA	AG	GG	AA	AG	AG	AG	--	AG	--	AG	AG
CC	CC	AC	--	CC	AC	AC	AC	CC	AC	--	AC	AC

AA	AA	AG	GG	AA	AG	AG	AG	AA	AG	--	--	AG
AA	AA	AG	GG	AA	GG	AG	GG	AA	--	--	AG	AG
TT	TT	CT	CC	TT	CT	CT	CC	TT	CT	--	CT	CT
GG	GG	AG	AA	GG	AA	AG	GG	GG	AG	--	--	AG
GG	GG	GT	--	--	TT	GT	GT	GG	GT	--	TT	GT
TT	TT	CT	CC	TT	CC	CT	CT	TT	CT	--	--	CT
--	GG	AG	AA	GG	AA	AG	AG	GG	AG	--	AA	AG
GG	GG	AG	AA	GG	AA	AG	AG	GG	AG	--	AA	AG
--	AA	AG	GG	--	GG	AG	AG	--	AG	--	GG	AG
GG	GG	--	CC	GG	CC	CG	CC	GG	CG	--	CC	CG
AA	AA	AG	GG	AA	GG	AG	AG	AA	GG	--	GG	AG
AA	AA	--	CC	AA	CC	AC	AC	AA	AC	--	CC	AC
GG	GG	AG	AA	GG	AA	AG	AG	GG	AG	GG	AA	AG
GG	GG	AG	AA	GG	AA	AG	AG	GG	AG	--	AA	AG
AA	AA	AT	TT	AA	TT	AT	AT	AA	AT	AA	TT	AT
TT	TT	CT	CC	TT	CC	CT	CT	TT	CT	TT	CC	CT
TT	--	CT	CC	TT	CC	CT	--	TT	TT	--	--	CT
CC	CC	CG	GG	CC	GG	CG	CG	CC	CG	--	GG	CG
CC	CC	CT	TT	CC	TT	CT	CT	CC	CT	--	--	CT
TT	TT	CT	CC	TT	CC	CT	CT	TT	CT	--	CC	CT
CC	CC	CT	TT	CC	TT	CT	CT	CC	CT	--	TT	CT
AA	AA	AT	TT	AA	TT	AT	AT	AA	AT	--	TT	AT
TT	TT	AT	AA	TT	--	AT	AT	TT	AT	--	AA	AT
CC	CC	CT	CT	CC	TT	CT	CT	CC	CT	--	TT	CT
TT	TT	CT	CC	TT	--	CT	CT	TT	CT	--	CC	CT
TT	TT	AT	AA	TT	AA	AT	AT	TT	AT	--	AA	AT
AA	AA	AG	GG	AA	GG	AG	AG	--	AG	--	GG	--
TT	TT	--	GG	TT	GG	GT	GT	TT	GG	--	--	GT
CC	CC	CT	--	--	TT	CT	--	CC	CT	CC	TT	CT
GG	GG	CG	CC	GG	CC	CG	CG	GG	CG	--	CC	CG
AA	AA	AG	GG	AA	GG	AG	AG	AA	AG	--	GG	AG
--	CC	CT	TT	CC	TT	CT	CT	CC	CT	--	TT	CT
CC	CC	CT	TT	CC	TT	CT	CT	CC	CT	--	TT	CT
AA	AA	AT	TT	AA	TT	AT	AT	AA	AT	--	TT	AT
--	--	AG	AA	GG	AA	AG	AG	GG	AA	--	AA	AG
GG	GG	AG	AA	AG	AA	AG	AG	AG	AG	--	--	AA
GG	GG	GT	TT	GT	TT	GG	GT	TT	TT	--	TT	TT
--	GG	AG	AA	--	AA	GG	AG	AA	AG	--	AA	AA
AG	GG	AG	GG	--	GG	AG	GG	GG	--	--	--	GG
TT	TT	CT	TT	TT	CT	TT	CT	CT	TT	--	TT	TT
GG	AG	AG	AG	GG	AA	AG	AA	AG	--	--	AG	GG
GG	AG	GG	AG	GG	AG	AG	AG	GG	AG	--	GG	GG
GG	AG	AG	GG	GG	--	AG	AA	AG	AG	--	AG	GG
CC	CG	CG	CC	CC	GG	CG	GG	GG	CG	CG	GG	CC
TT	CT	CT	TT	--	CC	CT	CC	CC	--	--	CT	TT
AC	AC	CC	AC	CC	CC	AC	CC	CC	AC	--	CC	AC
AG	AA	AG	AG	AG	AA	AA	AA	AA	AA	--	AG	AA

CC	AC	CC	AC	CC	CC	AC	CC	AC	AC	--	CC	AC
CC	AC	AC	CC	CC	CC	AC	AC	AC	CC	--	CC	AC
TT	CT	TT	CT	CT	TT	CT	TT	CT	CT	--	TT	CT
AA	AG	AA	GG	GG	AA	AG	AA	GG	GG	AG	AA	AG
GG	AG	GG	AA	AA	GG	AG	GG	AA	AA	--	GG	AG
TT	CT	TT	CC	CC	TT	CT	TT	CC	CT	--	TT	CT
--	CT	CT	TT	TT	TT	CT	TT	TT	CT	TT	CT	CT
CC	CG	CC	GG	GG	CG	CG	GG	CG	CG	GG	CC	CG
AA	AG	AA	GG	GG	AG	AG	GG	AG	AG	--	AA	AG
TT	CT	TT	CC	TT	CC	CT	CC	CT	CT	--	TT	CT
AT	AA	AT	AA	AA	AA	AT	AA	AT	AT	--	AT	AT
CC	AA	CC	AA	AC	AA	AC	AA	AC	AC	--	--	CC
GG	AG	GG	AG	AG	AG	AG	AG	AG	GG	--	--	--
CC	CT	CC	CT	CT	CT	CT	CT	CT	CT	--	CC	CC
CC	CT	CC	CC	CT	CT	CT	CT	CT	CT	CC	CC	CC
GG	GT	--	GT	GT	GT	GT	GT	GG	GT	GG	GG	GG
TT	TT	TT	CC	TT	TT	CC	CC	CC	CT	--	CT	CC
GG	GG	GG	AG	GG	GG	AG	AG	AG	GG	GG	GG	AG
GG	GG	GG	AA	GG	GG	AA	AA	AA	AG	AG	AG	AA
CG	GG	CG	GG	GG	CG	GG	GG	GG	GG	--	GG	--
GG	AG	GG	AG	GG	AG	AA	AA	AA	AG	--	AG	AA
AA	AG	AA	AG	AA	AG	GG	GG	GG	AG	AA	AG	GG
TT	GT	TT	TT	TT	GT	GT	GT	GT	GT	TT	GT	GT
AA	AG	AA	AG	AA	AG	GG	GG	GG	AG	--	AG	GG
--	AG	AA	AG	AA	AA	GG	GG	GG	AG	AG	AG	AG
GG	GT	GT	GG	GG	GT	GT	GT	GT	GT	--	GT	GG
CT	CT	CT	CT	--	CC	TT	TT	TT	--	--	CT	--
CT	CT	CC	CT	CT	--	CC	CC	CT	CT	CC	CT	CT
CC	CC	CT	CT	CT	CC	CT	CT	CT	CC	--	CC	CT
AG	AG	GG	AG	AG	AA	GG	GG	AG	AG	--	GG	AG
CT	TT	TT	TT	CT	CC	TT	TT	CT	CT	--	--	CT
AG	GG	GG	GG	AG	AA	GG	GG	AG	AG	AG	GG	AG
--	TT	--	TT	CT	CC	TT	TT	CT	CT	--	TT	CC
CG	CC	CC	CC	--	--	CC	CC	GG	CG	--	CC	GG
CT	TT	TT	TT	CC	CT	TT	--	CC	CT	--	TT	CC
--	--	AC	AA	CC	AC	CC	AC	AA	AA	--	AC	AC
AG	AG	AG	GG	AA	AG	AA	--	--	GG	--	AG	AG
CT	CT	CT	TT	CC	CT	CC	--	TT	TT	--	CT	CT
AG	AG	AG	AA	GG	--	AG	GG	--	AA	--	AG	AA
AG	AG	AG	AA	GG	--	AG	AA	--	AA	--	AG	AA
--	TT	TT	TT	CT	CT	CT	CT	TT	TT	--	CT	TT
GG	AG	--	GG	--	--	AG	AG	GG	GG	--	AG	GG
AA	AA	AG	AA	AG	AG	AA	AA	AG	--	--	--	AG
TT	TT	GT	TT	GT	GT	TT	TT	GT	TT	--	TT	GT
TT	TT	GT	TT	GT	GT	TT	TT	GT	TT	--	TT	GT
AA	AA	AC	AA	CC	CC	AC	AC	--	AA	--	AC	AC
--	AA	AG	AA	GG	GG	AG	AG	AG	--	--	AG	AG

CG	CC	CG	CC	GG	GG	CG	CG	CG	CC	--	CG	CG
CG	CC	CG	CC	GG	GG	CG	CG	CG	CC	--	CG	CG
CT	TT	--	TT	CC	CC	CT	CT	CT	TT	--	CT	CT
CT	CC	CT	CC	TT	TT	CT	CT	CT	CC	--	CT	CT
AT	AA	AT	AA	TT	TT	AT	--	AT	AT	AA	AT	AT
GG	AG	GG	AG	GG	GG	AG	AG	GG	GG	GG	GG	GG
CT	CT	TT	CC	TT	TT	CT	TT	CT	CT	CT	TT	CT
--	CT	CC	--	--	--	CT	CT	CT	CT	CT	CC	CT
--	CT	TT	CC	CT	--	CT	--	CT	--	--	TT	CT
AG	AA	AA	GG	AG	AA	AG	AG	AG	AG	--	AA	AG
CG	CG	CG	CC	CG	CG	CG	CG	CC	CC	--	CG	CC
--	CG	CG	GG	GG	CG	GG	GG	--	CG	--	CG	CG
GT	GT	GT	GG	--	--	GT	GT	GG	GT	--	GT	GG
CC	CG	CG	CC	CC	CC	CC	CC	CG	CC	--	CC	CC
--	AA	AA	AG	--	--	AG	AG	AA	AG	--	GG	AG
AA	AC	AC	AA	AC	AA	AA	AA	AC	AC	AC	AA	AA
AA	AT	AT	AA	AT	AA	AA	AA	AT	AA	--	AA	AA
GG	AA	AA	AG	--	AG	AG	AG	AA	GG	--	GG	AG
--	TT	TT	AT	AT	AT	AT	AT	TT	AT	--	AA	AT
GG	AA	AA	AG	AG	AG	AG	AG	AA	AG	AA	GG	AG
CC	CC	AC	AC	--	AC	AC	--	AC	CC	--	CC	AC
CC	CC	TT	CT	TT	CT	CC	CT	TT	CC	--	CC	CT
TT	TT	GG	TT	GG	GT	TT	GT	GG	GT	GG	TT	GT
TT	TT	GG	TT	GG	GT	TT	GT	GG	GT	--	TT	GT
CC	CC	TT	CC	TT	CT	CC	CC	TT	--	--	CT	CT
CC	CC	GG	CC	GG	CG	CC	--	GG	--	--	--	CG
GG	GG	AA	--	AA	AG	GG	GG	AA	AG	AA	AG	AG
CC	CC	TT	CC	TT	CT	CC	CT	TT	CT	--	CT	CT
TT	TT	GG	TT	GG	GT	TT	GT	GG	GT	GG	GT	GT
TT	TT	CC	TT	CC	CT	TT	CT	CC	CT	CC	CT	CT
GG	GG	CC	GG	CC	CG	GG	CG	CC	CG	CG	CG	CG
GG	GG	AA	GG	AA	AG	GG	--	AA	AG	--	AG	AG
--	AA	AC	AA	CC	AC	AA	--	--	AC	--	AC	--
--	TT	--	TT	CC	CT	TT	--	CC	CT	--	CT	CT
--	GG	GG	GG	AG	GG	GG	GG	GG	GG	--	AG	GG
AA	AA	--	AA	--	AC	AA	CC	CC	AC	--	AC	AC
CC	CC	CT	CC	TT	CT	CC	CT	TT	CT	--	CT	CT
GG	GG	AG	GG	AA	AG	GG	AG	AA	AG	--	AG	AG
--	GG	CG	GG	CC	GG	GG	CG	--	CG	--	CG	CG
GG	GG	GT	GG	--	GT	GG	GT	TT	GT	--	GT	GT
--	CC	CT	CC	TT	CT	CC	CT	TT	CT	--	CT	CT
TT	TT	TT	TT	TT	TT	TT	TT	CT	TT	--	CT	TT
--	GG	GG	AG	GG	GG	AG	GG	AG	GG	GG	AG	GG
CC	CC	CC	CC	GG	--	CC	CG	CC	CG	GG	CG	CG
AA	AT	AA	AA	AT	AT	AA	AT	AA	AA	AT	AA	AT
AA	AA	AA	AA	AG	AA	AA	AA	AA	AA	--	AG	AA
CC	CC	CC	CC	AC	CC	CC	CC	CC	CC	--	AC	CC

TT	TT	TT	TT	CT	TT	TT	TT	TT	TT	--	CT	TT
TT	CT	TT	TT	--	CT	TT	CT	TT	TT	CT	TT	CT
GG	AG	GG	GG	AT	--	GG	AG	GG	GG	--	--	AG
AA	AA	AA	AA	AT	AA	AA	AA	AA	AA	AT	AT	AA
GG	GG	GG	GG	AA	GG	GG	AG	GG	AG	--	AG	GG
--	CC	--	CC	--	--	CC	AC	CC	AC	--	AC	CC
AA	AA	AA	AA	AG	AA	AA	AA	AA	AA	--	AG	AA
GG	GG	GG	AG	--	--	GG	GG	GG	--	--	AG	GG
AA	AA	AG	AG	GG	AA	AA	AG	AG	AG	--	AG	AA
--	GG	AG	GG	AA	GG	GG	AG	AG	AG	--	AG	GG
--	GG	GT	GT	TT	GG	GT	GT	TT	GT	TT	GT	GG
GG	GG	AG	AG	AG	GG	AG	GG	AG	AG	AG	GG	GG
CC	CC	--	CT	TT	CC	CT	CC	CC	CT	TT	CT	CT
CC	CC	CG	CG	GG	CC	CG	CC	CG	CG	--	CG	CG
TT	TT	TT	TT	CT	TT	TT	TT	TT	TT	CT	CT	TT
TT	TT	CT	CT	CT	TT	CT	TT	TT	CT	--	TT	CT
CC	CC	CC	CC	CT	CT	CC	CC	CT	CC	CC	CC	CT
GT	GG	GG	GT	GG	GG	GG	GG	GT	GG	GG	GG	GG
AG	GG	AG	AG	AG	GG	GG	GG	AA	GG	AA	GG	AG
--	AA	--	AG	AG	GG	AG	AA	--	AA	--	AA	AG
AA	AG	AA	AA	--	--	AA	AA	AA	AA	AA	AA	AG
GT	GT	GT	GT	GT	GT	GG	GG	TT	GG	--	GG	GT
--	AG	AA	AA	AG	AG	AA	AA	AG	AA	--	AA	AG
CC	CT	CC	CC	CT	CT	CC	CC	CT	CC	--	CT	CT
AG	AA	AG	AG	AG	AA	AG	AG	AA	AG	--	AA	AA
GG	AA	AG	AG	GG	AG	GG	AG	AA	GG	--	AG	AG
GG	AA	AG	AG	GG	AG	GG	AG	AA	GG	--	AA	AG
GG	CC	CG	CG	GG	CG	GG	CG	CC	GG	--	CC	CG
GG	CC	CG	CG	GG	CG	GG	CG	CC	GG	CC	CC	CG
GG	AA	AG	AG	GG	--	GG	AG	AG	GG	--	AA	AG
GG	AG	AG	AG	GG	GG	GG	AG	GG	GG	--	--	GG
TT	CC	CT	CT	TT	TT	TT	CT	--	--	CC	CC	CT
CC	CC	AC	AC	CC	CC	CC	AC	--	CC	AC	AC	AC
CC	CC	CG	CG	--	CC	CC	CG	CG	CC	--	CG	CG
CC	CC	AC	AC	CC	CC	CC	AC	AC	CC	--	AC	AC
AC	AC	AC	AC	AA	AC	AC	--	AC	AA	--	CC	CC
AA	AG	AA	AA	AA	AG	AA	AA	AA	AA	--	AG	AG
AG	AG	AG	AG	AA	AG	AG	AG	AG	AG	AG	GG	GG
GG	AG	--	GG	GG	GG	GG	GG	GG	GG	GG	AG	AG
CT	CT	CT	CT	CC	CT	CT	CT	TT	CT	--	TT	TT
CT	CT	CT	CT	CC	CT	CT	CT	CT	CT	CT	TT	TT
--	AA	AG	AG	--	AA	AG	AA	AG	AG	--	AG	--
--	CC	CT	CT	CC	CC	CT	CT	CC	CT	CC	CT	CT
--	GG	AG	AG	GG	AG	AG	GG	AG	AA	--	GG	--
AC	AC	AC	AC	CC	CC	CC	AC	AC	AC	AC	CC	AC
CT	CC	CC	CC	CC	CT	CT	CC	CC	CT	--	CC	CT
--	AG	AG	AG	--	--	AG	AG	--	GG	AA	AA	GG

AG	AA	AA	AA	AA	AG	AG	AA	AA	AG	--	AA	AA
GG	AG	AG	AG	AA	AG	GG	AA	AA	GG	--	AA	GG
AA	AC	AC	AC	CC	AC	AA	CC	CC	AC	--	CC	AA
AA	AG	AG	GG	GG	AG	AA	GG	GG	AG	--	GG	AA
--	GT	GT	TT	TT	GT	GT	--	TT	GT	--	TT	GT
AG	AG	AG	AA	--	AA	AG	AA	--	AA	--	--	AG
--	CC	CC	TT	TT	CC	CT	TT	TT	CT	--	TT	CC
CT	CT	CT	CC	CC	CC	CT	CC	--	CC	--	--	CT
--	AG	AA	GG	AG	AA	AG	GG	GG	AG	GG	GG	AA
--	CG	CG	GG	CG	CG	GG	GG	GG	CG	--	GG	CG
--	CT	CT	TT	CT	CT	TT	TT	CT	CT	--	CT	CT
AG	AG	AG	AA	AG	GG	AA	AA	AG	AG	--	AG	GG
AG	AG	AG	AA	AG	GG	AA	AA	GG	AG	AA	AG	GG
CG	CG	CG	CC	CG	GG	CC	CG	GG	CC	--	CG	GG
AG	AG	AG	AA	--	GG	AA	AA	GG	AG	--	AG	--
GG	GG	--	GG	GG	AA	GG	AG	AG	GG	--	AG	AG
TT	TT	CT	TT	TT	CC	TT	CT	TT	TT	--	CT	CT
--	--	AG	GG	GG	AA	GG	AG	GG	GG	--	AG	AG
CC	CC	CG	CC	CC	GG	CC	CG	CC	CC	--	CG	CG
CC	CC	AC	CC	CC	AA	CC	--	CC	CC	AA	AC	--
AA	AA	AG	--	AA	GG	AA	AG	AA	AA	GG	AG	AG
GG	GG	GT	GG	GG	TT	GG	GT	GG	--	--	GT	GT
GG	GG	AG	GG	GG	AA	GG	AG	--	GG	--	AG	AG
GG	GG	AG	GG	GG	AA	GG	AG	GG	GG	--	AG	--
--	AA	AG	AA	AA	GG	AA	AA	AA	AA	GG	AG	AG
AA	AA	AC	AA	AA	CC	AA	AC	AA	AA	--	AC	AC
AA	AA	AG	AA	AA	GG	AA	AG	AA	AA	--	AG	AG
AA	AA	AG	AA	AA	GG	AA	AG	AA	AA	--	AG	AG
TT	TT	CT	TT	TT	CC	TT	CT	TT	TT	--	CT	CT
GG	GG	CG	GG	GG	CC	GG	CG	GG	--	--	CG	CG
--	AA	AC	AA	AA	--	AA	AC	AA	--	--	AC	AC
TT	TT	CT	TT	TT	CC	TT	CT	TT	TT	--	CT	CT
--	TT	GT	TT	TT	GG	TT	GT	TT	TT	GG	GT	GT
CC	CC	CT	CC	CC	TT	CC	CT	CC	CC	--	CT	CT
CC	CC	CG	CC	CC	GG	CC	--	CC	CC	--	CG	CG
TT	TT	AT	TT	TT	AA	TT	--	TT	TT	--	AT	AT
CC	CC	CT	CC	CC	TT	CC	CT	CC	CC	--	CT	CT
--	GG	AG	GG	GG	AA	GG	--	GG	GG	--	AG	AG
AA	AA	AG	AA	AA	GG	AA	--	AA	AA	GG	AG	AG
AA	AA	AT	AA	AA	TT	AA	AT	AA	AA	--	AT	AT
GG	GG	AG	GG	GG	AA	GG	AG	GG	GG	--	AG	AG
AA	AA	AG	AA	AA	GG	AA	AG	AA	AA	--	--	AG
CC	CC	CG	CC	CC	GG	CC	CG	CC	CC	GG	--	CG
AA	AA	AG	AA	AA	GG	AA	AG	AA	AA	--	AG	AG
GG	GG	CG	--	--	CC	GG	CG	GG	--	--	--	CG
GG	GG	AG	GG	GG	AA	GG	AG	--	GG	--	AG	AG
AA	AA	AC	AA	--	CC	AA	AC	AA	AA	--	AC	AC

--	GG	AG	GG	--	AA	GG	AG	GG	GG	--	AG	AG
--	CC	CG	CC	CC	GG	CC	CG	CC	CC	--	CG	CG
--	CC	CT	CC	CC	--	CC	CT	CC	CC	--	CT	CT
TT	TT	AT	TT	TT	AA	TT	AT	TT	--	--	AT	AT
--	CC	CT	CC	CC	TT	CC	CT	CC	CC	TT	CT	CT
AT	AA	AT	AA	AA	TT	AA	AT	AA	AA	--	AT	AA
AT	TT	AT	TT	TT	AA	TT	AT	TT	TT	AA	AT	TT
CG	GG	CG	GG	GG	CC	GG	CG	GG	GG	--	CG	GG
CT	CC	CT	CC	CC	TT	CC	CT	CC	CC	TT	CT	CC
AG	AA	AG	AA	AA	GG	AA	AG	AA	AA	GG	AG	AA
AC	AA	AC	AA	AA	CC	AA	AC	AA	AA	--	AC	AA
GT	TT	GT	TT	TT	GG	TT	GT	TT	TT	--	GT	TT
CT	CC	CT	CC	CC	--	CC	CT	CC	CC	--	CT	CC
CG	GG	CG	GG	GG	CC	GG	CG	GG	GG	--	CG	GG
--	TT	GT	TT	TT	GG	TT	GT	TT	TT	--	GT	TT
CT	TT	CT	TT	TT	CC	TT	CT	TT	TT	CC	CT	TT
AG	AA	AG	--	--	GG	AA	AG	AA	AA	--	AG	AA
AC	AA	AC	AA	AA	--	AA	AC	--	--	--	AC	AA
CT	CC	CT	CC	CC	TT	CC	CT	CC	CC	--	CT	CC
AC	AA	AC	AA	AA	CC	AA	AC	AA	AA	--	AC	AA
AG	AA	AG	AA	AA	GG	AA	AG	AA	AA	--	AG	AA
GT	GG	GT	GG	--	TT	GG	GT	--	GG	--	GT	GG
GT	GG	GT	GT	--	TT	GG	GT	GG	GG	--	GT	GG
AG	AG	AG	GG	GG	AA	GG	AG	GG	GG	--	AG	GG
CT	CT	CT	CC	CC	TT	CC	CT	CC	CC	--	CT	CC
CT	CT	CT	CC	CC	TT	CC	CT	CC	CC	--	CT	CC
AG	AG	AG	AG	AA	GG	AA	AG	AA	AA	GG	AG	AA
CT	CT	CT	CT	CC	TT	CC	CT	CC	CC	--	CT	CC
CT	CT	CT	CT	TT	CC	TT	CT	TT	TT	CC	CT	TT
AG	AG	AG	AG	GG	AA	GG	AG	GG	GG	AA	AG	GG
CT	CT	CT	CT	CC	TT	CC	CT	CC	CC	--	CT	CC
CT	CT	TT	CT	TT	CT	TT	CT	TT	TT	--	--	TT
CT	TT	CT	CT	CT	--	CC	CT	--	CC	--	CT	CC
AG	GG	AG	AG	AG	GG	AG	AG	AG	AG	--	AG	AA
AC	CC	AC	AC	AA	CC	CC	AC	AC	AC	AC	AC	AA
CG	CC	CC	CG	CC	CC	CC	CC	CG	CG	--	CG	CG
GT	TT	GT	GT	GT	TT	TT	TT	GT	GT	GT	GT	GG
AG	AA	AG	AG	AG	AA	AA	AA	AG	--	--	AG	GG
--	CT	CT	CT	CT	CC	CC	CC	CT	CT	--	CT	TT
AG	GG	AG	AG	GG	GG	GG	GG	AG	AG	--	AG	AG
AG	AG	AG	AG	--	AA	AA	AA	AG	AG	AG	AG	--
CG	CG	CG	CG	CG	CG	CC	CC	CG	CG	--	--	GG
CT	TT	CT	CT	TT	TT	TT	TT	CT	CT	CT	CT	CT
TT	GT	GT	GT	GG	GG	GG	GG	GT	GT	TT	GT	TT
GG	AG	GG	GG	AG	AG	GG	GG	GG	AG	--	AG	--
AG	GG	GG	AG	AG	GG	AG	GG	GG	GG	--	GG	AG
AC	AC	AA	AC	--	CC	AC	AA	AA	AC	AC	AC	AC

AT	AT	--	AT	AT	AT	AT	AT	TT	AT	--	AT	AT
AT	AT	TT	AT	AT	AT	AT	AT	TT	AA	--	AT	AT
AT	--	AA	AT	AT	AT	AT	--	--	TT	--	AT	AT
CT	CT	CT	CT	TT	CC	CT	CT	TT	CT	--	CT	CT
AG	AG	AG	AG	AG	AG	GG	AG	GG	GG	--	AG	AG
AC	AC	AC	AC	AC	AC	AA	AC	AA	AA	CC	CC	AC
CT	CT	--	CT	CT	TT	TT	CC	TT	TT	--	CC	CT
AG	AG	AG	AG	GG	AA	GG	AG	AG	GG	--	AA	AG
GT	TT	TT	GT	GG	TT	GG	GT	GT	GG	TT	TT	GT
AG	AA	AA	AG	GG	AA	GG	AA	AA	GG	--	AA	AG
CC	CG	CG	CC	CC	CG	CC	CC	CC	CC	CG	CG	CC
GG	AG	AG	GG	GG	AG	GG	GG	GG	GG	GG	AG	GG
CG	GG	GG	CG	CC	GG	CC	CG	CC	CC	GG	GG	CG
AG	AG	GG	AG	AA	AG	AA	AA	AA	AA	--	GG	AG
AG	AA	AA	AG	GG	AG	GG	--	GG	GG	--	AA	AG
AG	GG	GG	AG	AA	AG	AA	AG	AA	AA	GG	GG	AG
GG	GT	GT	GG	GG	GT	GG	GG	GG	GG	--	GT	GG
GG	AA	AA	AG	GG	AG	GG	AG	GG	GG	--	AA	AG
TT	GT	GT	TT	--	GT	TT	TT	TT	TT	--	GT	TT
GG	CC	CG	CG	CG	GG	GG	GG	GG	GG	--	CG	CG
TT	TT	TT	CT	TT	TT	CT	TT	CT	--	--	--	CT
CG	CG	CG	GG	GG	GG	CC	GG	CC	CG	--	CG	CG
CG	CG	CG	CC	--	CC	CG	CC	CG	CC	--	CG	CC
AA	AA	AC	AC	AA	AA	AA	AC	AC	AA	--	AA	AC
--	CC	CT	CT	CC	CC	CC	CT	CT	CC	--	CC	--
AA	AA	AG	AG	AG	--	AA	AG	AG	AA	AG	AA	AG
CC	CC	CT	--	CT	CC	CC	CT	CT	CC	--	CC	CT
CG	CG	CG	GG	CG	--	CG	GG	GG	CG	--	--	--
CC	CC	CT	CT	CT	CC	CC	CT	CT	CC	--	CC	CT
AG	AG	GG	AG	GG	AA	AG	AG	GG	AG	GG	AA	AG
GG	GG	GT	GT	GT	GG	GG	GT	GT	GG	GT	GG	GT
GT	GT	GG	GT	GG	TT	GT	GT	GG	GT	GG	--	GT
GG	GG	CG	CG	--	--	GG	CG	CG	--	--	GG	CG
CG	CG	CC	CG	CC	GG	CG	CG	CC	CG	CC	GG	CG
GG	GG	AG	AG	AG	GG	GG	AG	AG	GG	--	GG	AG
CT	CT	--	CT	CC	TT	CT	CT	CC	CT	CC	TT	--
GT	GT	TT	GT	TT	GT	GT	GT	TT	GT	--	GG	GT
GG	GG	AG	AG	AG	GG	GG	AG	AG	GG	AG	GG	AG
AG	AG	--	AG	GG	AA	AG	AG	GG	AG	GG	AA	AG
CT	CT	CC	CT	CC	TT	CT	CT	CC	CT	--	TT	--
--	TT	AT	AT	AT	TT	TT	--	AT	TT	--	TT	AT
--	TT	GT	GT	GT	TT	TT	GT	GT	TT	--	TT	GT
CT	CT	TT	CT	TT	CC	CT	CT	--	CT	--	CT	CT
AA	AA	AG	AG	AG	AA	AA	AG	AG	AA	AG	AG	AG
AT	AT	AA	AT	AA	TT	AT	TT	AA	AT	AA	AT	AT
AA	AA	AG	AG	AG	AA	AA	AG	AG	AA	AG	AG	AG
TT	TT	CT	CT	CT	TT	TT	CT	CT	TT	--	CT	CT

TT	TT	AT	AT	AT	TT	TT	--	AT	TT	--	AT	AT
GT	GT	--	GT	TT	GG	GT	GT	TT	GT	TT	GT	GT
AG	AG	GG	AG	GG	AA	AG	AG	GG	AG	--	AG	AG
TT	CT	TT	CT	--	CC	CT	TT	TT	--	--	CT	CC
TT	CT	TT	CT	TT	CC	CT	CT	TT	CT	TT	CT	CT
TT	CT	TT	CT	TT	CC	TT	TT	--	CT	--	CT	CT
TT	GT	TT	GT	TT	GG	TT	GT	GT	TT	--	GT	GT
GG	CG	GG	CG	CG	CC	GG	CG	--	GG	--	CG	CG
AA	AC	AA	AC	AA	CC	AA	AC	AC	AA	--	AC	AC
AG	AG	GG	AG	AG	AA	GG	GG	AA	GG	--	AG	AG
AG	AG	AA	AG	--	AG	AG	--	GG	AA	--	GG	AG
CC	CT	CT	TT	TT	--	CT	CC	CT	CT	--	--	CT
CC	CC	AC	AA	AA	AA	AC	AC	AC	--	--	AC	AC
GT	TT	TT	TT	TT	GT	TT	TT	GT	TT	TT	GT	GT
CG	GG	CG	GG	GG	GG	CG	--	GG	GG	--	GG	GG
AA	GG	AG	GG	GG	AG	AG	AG	AG	AG	--	AG	AG
CT	TT	CT	TT	TT	TT	CT	CT	TT	CT	--	TT	TT
GT	TT	--	GT	--	GT	TT	TT	GT	TT	--	TT	TT
CT	CC	CT	CC	CC	CC	CT	CT	CC	CC	--	CC	CC
AC	AC	AA	AC	AC	AC	AA	AA	AC	AA	--	AA	AA
GG	AG	AG	AG	AG	AG	AG	AG	AG	AA	AA	AA	AA
AG	AG	AA	AG	AG	AG	AA	AA	AA	AA	--	AA	--
--	GT	TT	GT	GT	GT	GT	GT	TT	TT	--	TT	TT
TT	TT	CC	CT	--	--	CT	CT	CC	CC	--	--	CC
CC	AC	AA	AC	AC	AC	AC	CC	AA	AA	--	AA	AA
--	--	TT	AT	--	AT	AT	AA	TT	TT	TT	--	TT
GG	GG	GT	GT	GT	GG	GT	GG	GG	GT	--	GG	--
AA	AG	--	AG	AG	AG	AA	AA	AG	AG	AG	AG	AG
--	CG	CC	CC	CC	CG	CG	--	CC	CC	--	CG	CC
GG	AG	AA	AA	AA	AG	AG	GG	AG	AA	--	AG	AA
CG	CG	GG	GG	--	CG	CG	CC	CG	CC	--	CG	CC
AG	AG	GG	GG	AG	AG	AG	AA	AA	AA	--	AG	AA
AA	AA	AG	AG	--	AG	AG	AG	--	AA	--	AG	AA
AT	AT	AT	AT	AA	AA	AA	AA	AA	AA	AT	AA	AA
AA	AA	AG	AG	AA	AG	AG	AG	AG	AA	--	AG	AA
AG	AG	AA	AA	AG	AA	AA	AA	AA	AG	--	AA	AG
AC	AA	CC	CC	AA	AC	CC	AC	CC	AC	--	CC	AA
AC	--	AC	AC	--	CC	AC	--	AC	AC	--	AC	CC
AC	CC	AA	AA	CC	AC	AA	AC	AA	AC	--	AA	CC
GT	GG	GT	TT	--	TT	TT	--	TT	GT	--	TT	GG
AT	AA	AT	AT	AA	TT	TT	AA	TT	AT	--	--	AT
AG	AA	AA	AA	AA	AG	AG	AA	AG	AG	--	AG	AG
AC	CC	CC	CC	CC	AC	AC	CC	CC	AC	--	AC	AC
AT	AA	AT	AT	AA	AA	TT	AT	TT	TT	TT	TT	AT
CC	CC	CT	TT	CC	CT	TT	CT	TT	CT	--	TT	CC
GG	GG	AA	AA	GG	AG	AA	AG	AA	AG	AA	AG	GG
CC	CC	TT	TT	CC	CT	TT	CT	TT	CT	--	CT	CC

TT	TT	CC	CC	TT	CT	CC	CT	CC	CT	--	CT	TT
GG	GG	AA	AA	--	AG	AA	AA	AA	AG	--	AG	GG
CC	CC	TT	TT	CC	CT	TT	CT	TT	CT	--	CT	CC
--	TT	CC	CC	TT	CT	CC	CT	CC	CT	--	CT	TT
CC	CC	TT	TT	--	CT	TT	CT	TT	CT	--	--	CC
GG	GG	AA	AA	GG	AG	AA	AA	AA	AG	AA	AG	GG
TT	TT	CC	CC	--	CC	CC	CT	CC	CT	--	CT	TT
GG	GG	AA	--	GG	GG	AA	AG	AA	AG	--	AG	GG
--	CC	GG	GG	CC	CC	GG	--	GG	CG	GG	CG	CC
AA	AA	CC	CC	--	AA	CC	AC	CC	AC	--	AC	AA
CT	CC	TT	TT	CC	CC	TT	--	TT	CT	TT	CT	CC
CT	CC	TT	TT	CC	CC	TT	CT	TT	CT	TT	CT	CC
AT	TT	AA	AA	--	TT	AT	AT	AA	--	--	AT	TT
--	CC	TT	TT	CC	CC	CT	CT	TT	CT	--	CT	CC
AC	AA	CC	CC	AA	AA	AC	AC	CC	--	CC	AC	AA
GT	TT	GG	GG	TT	TT	GT	GT	GG	GT	--	GT	TT
CT	--	CC	--	TT	--	CT	CT	CC	--	--	CT	TT
CG	GG	CC	CC	GG	GG	CG	CG	CC	CG	--	CG	GG
CG	GG	--	CC	GG	GG	CG	CG	CC	CG	CC	CG	GG
AC	CC	AA	AA	CC	CC	AC	AA	AA	AC	--	AC	CC
CG	CG	CC	CC	GG	GG	CG	CC	CG	CG	--	CG	GG
AA	AT	AT	TT	AA	AA	AT	AA	AT	AT	--	TT	--
AA	AG	AG	GG	AA	AA	AA	GG	--	AA	--	AG	AA
AA	AG	AG	GG	AA	AG	AA	GG	AG	AG	--	AG	AA
GG	AG	AG	AG	GG	AG	GG	AA	AG	AG	--	AG	GG
AA	AG	AG	AG	AA	AG	AA	GG	AG	AG	--	AG	AA
AA	AC	AA	AC	AA	AC	AA	CC	AC	AC	--	AC	AA
AA	AG	AA	AG	AA	AG	AA	GG	AG	AG	--	AG	AA
AA	AG	AA	AG	AA	AG	AA	GG	AG	AG	GG	AG	AA
GG	GT	--	GT	GG	GT	GG	TT	GG	GT	--	GT	GG
TT	GT	TT	GT	TT	GT	TT	GG	GT	GT	--	--	TT
CC	AC	CC	AC	CC	AC	CC	AA	AC	AC	CC	AC	CC
TT	CT	TT	CT	TT	--	TT	CC	--	CT	CT	--	TT
--	CT	TT	CT	TT	CT	TT	CC	CT	CC	--	CT	TT
--	GT	GG	GT	GG	GT	GG	TT	TT	GT	--	GT	GG
AA	AT	AA	AT	AA	AT	AA	TT	AT	AA	--	AT	AA
GG	CC	GG	CG	GG	CG	GG	CC	CG	CG	--	CG	GG
AA	AC	AA	AC	AA	AC	AA	CC	AA	AC	--	AC	AA
CC	CT	CC	CT	--	CT	CC	TT	CT	CT	TT	CT	CC
GG	CG	GG	CG	GG	CG	GG	CC	CG	CG	--	CG	CG
CC	CT	--	CT	CC	CT	CC	TT	--	CC	--	CT	CC
AA	AT	AA	AT	AA	AT	AA	TT	AT	AT	--	AT	AA
TT	CT	TT	CT	TT	CT	TT	CC	CT	--	--	CT	TT
GG	AG	GG	AG	--	--	GG	AA	AG	AG	--	AG	GG
--	AT	AA	AT	AA	AT	AA	TT	TT	AT	--	TT	--
AA	AT	AA	AT	AA	--	AA	TT	AT	AA	--	AT	AA
AA	AC	AA	AC	AA	AC	AA	CC	AC	AC	--	AC	AA

TT	GT	TT	GT	TT	GT	TT	GG	TT	GT	--	GG	TT
--	AG	GG	AG	GG	GG	GG	--	AG	AG	AA	AA	GG
GG	GT	GG	GT	GG	GT	GG	TT	GT	GT	--	TT	GG
--	--	AA	AG	AA	AG	AA	GG	--	AG	--	GG	AA
TT	CT	TT	CT	TT	CT	TT	CC	CT	CT	--	CC	--
AA	AG	AA	AG	AA	AG	AA	GG	AG	AG	AG	GG	AA
TT	CT	--	CT	TT	--	TT	CC	CT	CT	--	CC	TT
CC	--	--	AC	CC	--	CC	AA	CC	AC	--	AA	CC
AA	AG	AA	AG	--	--	AA	GG	AG	--	AA	GG	AA
TT	CT	TT	CT	TT	CT	TT	CC	TT	CT	--	CC	TT
GG	AG	GG	AG	AG	AG	GG	AA	GG	GG	AA	AA	GG
--	CT	CC	CT	CT	CT	CC	TT	CC	CT	--	TT	CC
GG	GT	GG	GT	GG	--	GG	GG	GG	GT	--	GT	GG
--	GG	GG	GG	AG	AG	GG	AG	GG	GG	--	AG	GG
TT	AA	TT	AT	--	--	AT	AA	TT	AT	--	AA	TT
TT	AA	TT	AT	--	AA	AA	AA	TT	AT	--	AA	AT
--	AA	--	CC	AA	AA	AA	AA	CC	AC	--	AA	AC
--	TT	GG	GG	TT	--	TT	--	GG	GT	--	TT	GT
--	TT	--	TT	CT	TT	CT	--	TT	CT	--	TT	CT
AG	GG	AA	GG	--	GG	AA	AG	GG	AG	GG	GG	AG
CG	CG	--	CG	GG	--	GG	GG	CG	CG	CG	CG	CG
CT	TT	TT	CT	TT	TT	CT	TT	CT	CT	CT	CT	CT
GT	GT	GG	GT	GG	GT	GG	GG	GT	GG	--	GT	GG
GG	AG	AG	GG	AG	AG	AG	--	GG	GG	--	GG	AG
--	AA	AC	AC	AA	AA	AC	AA	AC	AA	--	AC	AC
CG	GG	--	CG	GG	--	CG	GG	CG	GG	--	CG	CG
GT	TT	GT	GT	TT	TT	GT	TT	GT	TT	--	GT	GT
TT	CT	CT	TT	CT	CT	CT	CC	CT	CC	--	CT	CT
AG	GG	AA	AA	GG	GG	AA	--	GG	AG	--	GG	AG
CT	CC	CT	CT	CC	CC	CT	CC	CC	CC	--	CC	CC
CC	CG	CC	CC	CG	CG	CC	CG	CG	CG	--	CG	CG
AA	AA	AA	AC	AA	AA	AC	AC	AA	AC	AA	AA	AC
AA	AG	AA	AG	AG	AG	AG	GG	AG	GG	--	AG	GG
CT	CT	CT	TT	CT	CT	TT	TT	CT	TT	--	CT	TT
CG	GG	CG	CG	GG	GG	CG	GG	GG	GG	GG	GG	GG
GG	AG	GG	AG	AG	GG	AG	AA	GG	AA	AG	AG	AA
GG	CG	GG	GG	CG	CG	GG	CG	CG	CG	CG	CG	CG
--	CG	--	--	--	--	CG	CC	GG	CC	--	CG	CC
--	--	TT	CT	CC	--	CT	CC	CT	CC	--	CT	CC
AA	AG	AA	AG	AG	AG	AG	GG	GG	GG	--	AG	GG
GG	GG	GG	AG	GG	GG	AG	AG	GG	AG	--	GG	AG
--	CT	TT	CT	--	CT	CT	CC	CC	CC	--	--	CC
AG	AG	AG	GG	AG	AG	GG	GG	AG	GG	--	AG	GG
AA	AG	AA	AG	AA	AA	AG	GG	AG	GG	--	--	GG
AA	AC	AA	AC	AC	AC	AC	CC	AC	CC	--	AC	CC
AT	TT	AT	AT	TT	TT	AT	TT	TT	TT	--	TT	TT
GG	AG	GG	AG	AG	AG	AG	AA	AG	AA	--	AG	AA

--	AC	CC	AC	AC	AC	AC	AA	AA	AA	--	AC	AA
--	AG	GG	AG	AG	AG	AG	AA	AG	AA	--	AG	AA
AG	AG	AG	AA	--	AG	AA	AA	AG	AA	AG	AG	AA
CT	CC	--	CT	CC	CC	CT	CC	CC	CC	CC	CC	CC
CG	CC	CG	CG	CC	CC	CG	CC	CC	CC	--	CC	CC
CT	TT	CT	CT	TT	TT	CT	TT	TT	--	--	--	TT
GG	CG	GG	GG	GG	CG	GG	CG	CG	CG	--	CG	CG
CT	CC	CT	CT	CC	CC	CT	CC	CC	CC	CC	CC	CC
GG	GT	GG	--	GT	GT	GG	GT	GT	GT	--	GT	--

2-37	2-41	2-42	2-43	2-46	2-47	2-48	2-50	2-51	2-52	2-53	2-54	2-57
CC	AA	AC	CC	CC	AC	AA	AA	AC	AC	AC	CC	AC
GG	AA	AG	GG	GG	AG	AA	AA	AG	AG	AG	GG	AG
CC	TT	CT	CC	CC	CT	TT	TT	CT	CT	CT	CC	CT
AG	GG	GG	AG	AG	GG	GG	--	GG	GG	GG	AG	GG
GG	AA	AG	GG	GG	AG	AA	GG	AG	AG	AG	GG	AG
GG	CG	GG	GG	GG	GG	CG	GG	GG	GG	--	GG	GG
CC	TT	CT	CC	CT	CT	TT	CC	CT	CT	CT	CC	CT
GG	TT	GT	GG	GT	GT	TT	GG	GT	GT	GT	GG	GT
GG	TT	GT	GG	GT	GT	GT	GG	GT	GT	GT	GG	GT
GG	TT	GT	GG	GT	GT	GT	GG	GG	GT	GT	GG	GT
GG	AA	AG	AG	AG	AG	AG	GG	GG	AG	AG	GG	AG
CC	CT	--	CC	CC	CC	CC	CC	CC	CC	CC	CC	CT
CC	AA	CC	AA	AA	AC	AC	CC	CC	AC	AC	CC	AA
AA	AG	--	AG	AG	AA	AA	AG	AA	AA	AA	AA	AG
CC	CT	CC	CT	CT	CT	CT	CC	CC	CT	CT	CC	CT
AA	GG	AG	GG	GG	AG	AG	AG	AA	AG	AG	AA	GG
TT	TT	AT	AT	AT	AT	TT	AT	TT	TT	TT	TT	AT
CC	CT	CC	CT	CT	CT	CT	CC	CC	CT	CT	CC	CT
TT	GT	GT	GT	GT	GT	TT	GT	GT	TT	TT	TT	GT
CC	CG	CG	CC	CG	CG	CC	CG	CG	CC	CC	CC	CG
TT	AA	AT	AT	AA	AA	AT	--	AT	AT	AT	TT	AT
TT	--	CT	CT	CC	CC	CC	CT	CT	CC	CT	TT	CT
AT	TT	AT	AT	AT	TT	TT	AT	AT	TT	AT	AA	AT
AA	TT	AT	AT	TT	TT	TT	AT	AT	TT	AT	AA	AT
AA	GG	AG	AG	GG	GG	GG	AG	AG	GG	AG	AA	AG
TT	GT	GT	TT	GT	GT	GT	GT	GT	GT	TT	TT	TT
TT	GG	GT	GT	GG	GG	GG	GT	GT	GG	GT	TT	GT
TT	AA	AT	AT	AA	AA	AA	AA	AT	AA	AT	TT	AT
TT	CC	CT	CT	CT	CC	CC	CT	CT	CC	CT	TT	CT
CC	GG	CG	CG	CG	GG	GG	CG	CG	GG	CG	CC	CG
CC	TT	CT	CT	CT	TT	TT	CT	CT	TT	CT	CC	CT
AA	--	AC	AC	--	CC	CC	AC	AC	CC	AC	AA	AC
AA	GG	--	AG	--	GG	--	AG	AG	GG	AG	AA	AG
GG	TT	GT	GT	TT	TT	TT	GT	GT	TT	GT	GG	GT
AA	GG	AG	AG	GG	GG	GG	AG	AG	GG	AG	AA	AG
TT	--	GT	GT	GT	GG	GG	--	GT	GG	GT	TT	GT
GG	--	AG	AG	AG	AA	AA	AG	AG	AA	AG	GG	AG
CC	TT	CT	CT	CT	TT	TT	CC	CT	TT	CT	CC	CT
AA	TT	AT	AT	AT	TT	TT	AT	AT	TT	AT	AA	AT
AA	GG	AG	AG	AG	GG	GG	AG	AG	GG	AG	AA	AG
AA	CC	AC	AC	--	CC	CC	AC	AC	CC	AC	AA	AC
AA	GG	AG	AG	AG	GG	GG	AG	AG	--	AG	AA	AG

TT	--	CT	CT	CT	CC	CC	CT	CT	CC	CT	TT	CT
CG	GG	CG	CG	CG	GG	GG	CG	CG	GG	CG	CC	CG
CT	CC	CT	CT	CT	CC	CC	CT	CT	CC	CT	TT	CT
CT	TT	CT	CT	CT	TT	TT	CT	CT	TT	CT	CC	CT
AC	CC	AC	AC	AC	CC	CC	AA	AC	CC	AC	AC	AC
CT	CT	CC	CT	CT	TT	TT	CC	CC	TT	CT	CT	CT
CT	CT	TT	CT	CC	CC	CT	TT	TT	CC	CT	CT	CT
AA	AA	AA	AG	AG	GG	GG	AG	AG	AG	GG	GG	AG
TT	TT	GT	GT	GT	GG	GG	GT	GT	GT	GT	GG	GT
AA	AA	AG	GG	AG	GG	--	AG	AG	AG	AG	GG	AG
CC	CC	CT	TT	CT	TT	TT	CT	CT	CT	CT	TT	CT
GG	GG	CG	CC	CG	CC	CG	CG	CG	CG	CG	CC	CC
GG	GG	AG	AA	AG	AA	AG	AG	AG	AG	AG	AA	AA
AA	AA	AC	CC	AC	CC	CC	CC	AC	AC	AC	CC	CC
GG	GG	GG	AG	AG	AG	AG	AG	GG	GG	AG	AG	AG
CC	CC	--	CT	CT	TT	TT	TT	CT	CT	CT	TT	TT
TT	TT	CT	CT	CT	CC	CC	CC	CT	CT	CT	CC	CC
CC	CC	CT	CT	CT	CT	TT	TT	CC	CT	CT	TT	TT
GG	GG	AG	AG	AG	AG	AA	AA	GG	AG	AG	AA	AA
CC	AC	AC	AC	AC	AC	AA	AA	CC	AC	AC	AA	AA
AG	AG	AG	AG	AG	AG	AA	AA	GG	AG	AG	AA	AA
CG	CG	CG	CG	CG	CG	GG	GG	CC	CG	CG	GG	GG
CG	CG	CG	CG	CG	CG	CC	CC	GG	CG	CG	CC	CC
AG	AG	AG	AG	AG	AG	--	AA	GG	GG	AG	AA	AA
AC	AC	AC	AC	AC	AC	CC	CC	AC	AA	AC	CC	AC
TT	CT	CC	CT	CT	CT	TT	TT	CT	CC	CT	CT	CT
CC	CT	TT	CT	CC	TT	CC	--	CT	TT	CT	CT	CT
TT	AT	AA	AT	TT	AA	TT	AT	AT	AA	AT	AT	AT
GG	GG	GG	GG	GG	AG	GG	GG	GG	AG	GG	GG	GG
TT	AT	AT	AT	TT	AT	AT	AT	AT	--	AT	AT	AT
GG	GT	TT	GG	GT	GT	TT	--	GG	GT	GG	GG	GG
AG	AG	GG	AG	AG	AG	GG	GG	AG	AG	AG	AG	AG
CT	CT	TT	CT	CT	CT	TT	TT	CT	CT	CT	CT	CT
AG	AG	GG	AG	AG	AG	GG	GG	AG	GG	AG	AG	AG
TT	GT	GG	TT	GT	GT	GG	GT	TT	GG	TT	TT	TT
GT	--	GG	GT	GT	GT	GG	GG	GT	GG	GG	GT	GT
CC	CT	TT	CT	CC	CT	TT	CT	CC	TT	CT	CC	CC
CC	CT	--	CT	CC	CT	--	CC	CC	TT	CT	CC	CC
GG	AG	AG	AG	GG	AA	AA	AG	GG	AA	AG	AG	GG
CC	CG	CG	CG	--	CG	CG	--	CC	GG	CG	CG	CC
GG	AA	--	AG	GG	AG	AG	AG	GG	AA	AG	AG	GG
TT	CC	CT	CT	TT	CT	CT	CT	CT	CC	CT	CT	TT
TT	CC	CT	CT	CT	CT	CT	CT	CT	CC	CT	CT	TT
CG	CC	CG	CG	CC	CC	CG	CC	CC	CC	CC	CG	CG
AT	TT	AT	AT	TT	TT	AT	--	TT	TT	AT	AT	AT
GT	GG	GG	GT	GT	GT	GG	GT	GT	GG	GG	GG	GT
CT	CC	CC	CT	CT	CT	CC	CT	CT	CC	CC	CC	CT

CT	CT	CC	TT	CT	CT	CT	CC	CT	CC	CT	CT	TT
GT	GG	GG	GT	GT	GT	GG	GT	GT	GG	GG	GG	GT
CT	CT	CC	TT	TT	CT	CC	TT	CT	CC	CT	CT	TT
GT	TT	TT	GT	GT	GT	TT	GT	GT	TT	TT	TT	GT
CG	GG	GG	CG	CG	CG	GG	CG	CG	--	GG	GG	CG
AG	GG	GG	AG	--	AG	GG	AA	AG	AG	AG	GG	AA
AG	AA	AA	AG	AG	AG	AG	AG	AG	AG	AG	AA	GG
AG	AA	AA	AA	AG	AG	AG	AG	AG	AG	AG	AA	GG
TT	TT	CT	TT	CT	CT	CT	CT	CT	TT	CT	TT	CT
GG	GG	AG	GG	AG	AG	AG	AG	AG	GG	AG	AG	AG
GG	GG	GT	GG	GT	GT	GT	GT	GT	GG	GT	GT	GT
AG	GG	GG	AG	AG	GG	AG	GG	GG	AG	AG	GG	AG
TT	CT	CT	TT	CT	CT	CT	CT	CT	TT	CC	CT	CT
CC	CC	CC	CC	AC	CC	AC	CC	CC	CC	AC	CC	AC
CT	CT	CT	CT	CC	CT	CC	CT	CT	CT	CC	CT	CC
GG	GT	GT	GG	GT	GT	TT	GT	GT	GG	TT	GT	GT
CC	CT	CT	CC	CC	CT	CT	CT	CT	CC	CT	CT	CC
GG	GG	GG	GG	CG	GG	CG	GG	GG	GG	CG	GG	CG
CC	CC	CC	CC	AC	CC	AC	CC	CC	CC	AC	CC	AC
TT	CT	CT	TT	TT	CT	CT	CT	CT	TT	CT	CT	TT
AA	AG	AG	AA	AA	AG	AG	--	AG	AA	AG	AG	AA
AA	AG	AG	AA	AG	AG	GG	AG	AG	AA	GG	AG	AG
CT	CT	CT	CT	--	CT	CC	CT	CT	CT	CC	CT	CC
AA	AA	AA	AA	AT	AA	AT	AA	AA	AA	AT	AA	AT
CC	CC	CC	CC	CT	CC	CT	CC	CC	CC	CT	CC	CT
AA	AA	AA	AA	AG	AA	AG	AA	AA	AA	AG	AA	AG
CC	CT	CT	CC	CC	CT	CT	CT	CT	CC	CT	CT	CC
AT	AT	--	TT	AT	AT	--	TT	AT	TT	AA	AT	AT
--	AA	AT	TT	AT	AT	AA	AT	AT	TT	AA	AT	AT
TT	CT	CT	TT	CT	CT	CC	CT	CT	TT	CC	CT	CT
CC	CC	CC	CC	CT	CC	CT	CC	CC	CC	CT	CC	CT
GG	GT	GT	GG	--	GT	GT	GT	GG	GG	GT	GT	GG
CC	CT	CT	CC	CC	CT	CT	CT	CC	CC	CT	CT	CC
AG	AA	AA	AG	AG	AA	AA	AA	AG	AG	AA	AA	AG
GG	AG	AG	GG	AG	AG	AA	AG	GG	GG	AA	AG	AG
AA	AG	AG	AA	AA	AG	AG	AG	AA	AA	AG	AG	AA
CC	CT	CT	CC	CT	CT	--	CT	CC	CC	TT	CT	CT
CC	AC	CC	CC	CC	AC	AC	AC	CC	CC	AC	AC	CC
TT	AT	AT	TT	AT	AT	AA	AT	TT	TT	AA	AT	AT
AA	AA	--	AA	AG	AA	AG	AA	AA	AA	AG	AA	AG
AA	AC	AC	AA	AA	AC	AC	AC	AA	AA	AC	AC	AA
TT	AT	AT	TT	TT	AT	AT	AT	TT	TT	AT	AT	TT
AC	--	AA	AC	CC	AA	AC	AA	AC	AC	AC	AA	CC
CC	CC	CC	CC	AC	CC	AC	CC	CC	CC	AC	CC	AC
AG	GG	GG	--	AG	GG	GG	GG	AG	AG	GG	GG	AG
AC	AC	AC	AC	CC	AC	CC	AC	AC	AC	CC	AC	CC
AA	AG	AG	AA	AA	AG	AG	AG	AA	AA	AG	AG	AA

CC	CC	CC	CC	CG	CC	CG	CC	CC	CC	CG	CC	CG
TT	CT	CT	TT	CT	CT	CC	CT	TT	TT	CC	CT	CT
CC	CT	CT	CC	CT	CT	TT	CT	CC	--	TT	CT	CT
CC	CT	CT	CC	CC	CT	CT	CT	CC	CC	CT	CT	CC
AA	AG	AG	AA	AA	AG	AG	AG	AA	AA	AG	AG	AA
CC	CT	CT	CC	CT	CT	TT	CT	CC	CC	TT	CT	CT
TT	CT	CT	TT	TT	CT	CT	CT	TT	TT	CT	CT	TT
AA	AT	AT	AA	AT	AT	TT	AT	AA	AA	TT	AT	AT
GG	GG	GG	GG	AG	GG	AG	GG	GG	GG	AG	GG	AG
AA	AC	AC	AA	AA	AC	AC	AC	AA	AA	AC	AC	AA
CC	CT	CT	CC	CC	CT	CT	CT	CC	CC	CT	CT	CC
TT	TT	TT	TT	GT	TT	GT	TT	TT	TT	GT	TT	GT
TT	CT	CT	TT	TT	CT	CT	CT	TT	TT	CT	CT	TT
AC	CC	CC	AC	CC	CC	CC	CC	AC	AC	CC	CC	AC
AA	AG	AG	AG	AA	AG	AG	AG	AA	AA	AG	AG	AA
GG	GT	GT	GT	GG	GT	GT	--	GG	GG	GT	GT	GG
AT	TT	TT	TT	AT	TT	TT	TT	AT	AT	TT	TT	AT
AA	AG	AG	AG	AA	AG	AG	AG	AA	AA	AG	AG	AA
CC	CC	CC	CC	--	CC	CT	CC	CC	CC	CT	CC	CT
CC	CT	CT	CT	--	CT	CT	CT	CC	CC	CT	CT	CC
AA	AC	AC	AC	AA	AC	--	AC	AA	AA	AC	AC	AA
CC	CT	CT	CT	CC	CT	CT	CT	CC	CC	CT	CT	CC
AA	AT	AT	AT	AT	AT	TT	--	AA	AA	TT	AT	AT
GG	AG	AG	AG	AG	AG	AA	AG	GG	GG	AA	AG	AG
GG	GG	GG	GG	--	GG	CG	--	GG	GG	CG	GG	CG
TT	CT	CT	CT	TT	CT	CT	--	TT	TT	CT	CT	TT
GG	AG	AG	AG	--	AG	AG	AG	GG	--	AG	AG	GG
AA	AG	AG	AG	AA	AG	AG	AA	AA	AA	AG	AG	AA
AA	AA	AA	AA	AG	AA	AG	AA	AA	AA	AG	AA	AG
GG	GG	GG	GG	AG	GG	AG	GG	GG	GG	AG	GG	AG
CT	CT	CT	CT	TT	CT	CT	TT	TT	TT	CT	TT	TT
GT	--	GT	GT	GT	GT	TT	--	GG	--	TT	GG	GT
TT	TT	TT	TT	GT	TT	GT	TT	TT	TT	GT	TT	GT
AG	AG	AG	AG	AG	AG	AA	AG	GG	GG	AA	GG	AG
CT	CT	CT	CT	CC	CT	CT	CT	CC	CC	CT	CC	CC
GT	TT	GT	TT	TT	GT	GT	GT	TT	TT	GT	TT	TT
AG	--	AG	AG	GG	AG	GG	AG	AG	AG	GG	AG	GG
GG	GG	AG	GG	GG	AA	GG	AA	GG	AA	AG	AG	GG
CC	CC	CC	CC	CC	CT	CC	CT	CC	CT	CT	CT	CC
AT	AT	AA	AT	AT	AA	AT	AA	AT	AA	AT	AT	AT
GG	GG	CG	GG	GG	CC	GG	CC	GG	CC	CG	CG	GG
AC	AC	AC	AC	AC	AA	AC	AA	AC	AA	AA	AA	AC
GG	GG	AG	GG	GG	AA	GG	AA	GG	AA	AG	AG	AG
AG	GG	AG	GG	GG	AG	GG	AG	GG	AG	GG	GG	GG
CT	CC	CT	CC	CT	CT	CC	CT	CC	CT	CC	CC	CC
GG	AA	AG	AA	GG	GG	AG	GG	AA	GG	AG	AG	AG
CC	CT	CT	CT	CC	CC	CC	CC	CT	CC	CC	CC	CC

GG	AG	GG	AG	GG	GG	AG	GG	AG	GG	AG	AG	AG
AG	AG	GG	AG	AG	AG	AA	AG	AA	AG	AA	AA	AA
CC	TT	CT	TT	CC	CC	CT	CC	CT	CC	CT	CT	CT
AA	AT	AT	TT	AT	AA	AT	AA	AT	AA	AT	AT	AT
AA	AC	AC	CC	AC	AA	AC	AA	AC	AA	AC	AC	AC
TT	CT	CT	CC	CT	TT	CT	TT	CT	TT	CT	CT	TT
GG	AG	AG	AG	AG	GG	AG	GG	AG	GG	AG	AG	GG
CC	CT	CT	CT	CT	CC	CT	CC	CT	CC	CT	CT	CC
TT	TT	TT	TT	CT	TT	TT	--	TT	TT	TT	TT	TT
AA	AG	--	AG	AG	AA	AG	AA	AA	AA	AA	AG	AA
AG	AA	GG	AA	GG	GG	AA	GG	AA	AA	AG	AA	GG
CT	CC	TT	CT	--	TT	CC	TT	CC	CC	CT	CC	TT
GG	GG	AG	GG	AG	AG	AG	AG	GG	GG	AG	GG	AG
TT	CC	TT	CC	TT	TT	CT	TT	CC	CC	TT	CC	TT
CG	GG	CG	--	CG	CG	GG	GG	GG	GG	CG	GG	CG
TT	CC	TT	--	--	TT	CT	CC	CC	CC	TT	CC	TT
CT	CC	CT	CC	CT	CT	CT	CC	CC	CC	CT	CC	CT
AA	--	AA	GG	AA	GG	AG	GG	GG	GG	AA	AG	AA
TT	CC	TT	CC	TT	CC	CT	CC	CC	CC	TT	CT	TT
GG	AA	GG	AG	GG	AA	AG	AA	AA	AA	GG	AG	GG
CT	CC	TT	CT	TT	CC	CT	CC	CC	CC	TT	CT	CT
AG	GG	AA	AG	AA	GG	AG	--	GG	GG	AA	AG	AG
GG	GG	AG	GG	AG	GG	AG	GG	GG	GG	AG	AG	GG
GT	GT	GG	GT	GG	TT	TT	TT	TT	TT	GG	GT	GT
CC	CG	CG	CC	CG	CC	CC	CC	CC	CC	CG	CG	CC
CC	CG	CC	CC	CC	CG	CG	CG	CG	CG	CC	CG	CC
CT	CT	TT	CT	TT	CC	CC	CC	CC	CC	TT	CT	CT
CT	CT	CC	CT	CC	TT	TT	TT	TT	TT	CC	CT	TT
AT	AT	TT	AT	TT	AA	AA	--	AA	--	TT	AT	AA
AG	AG	AA	AA	AG	GG	GG	GG	AG	GG	AA	AG	GG
CT	CT	TT	TT	CT	CC	CT	CC	CT	CC	TT	CT	CC
CT	CT	TT	CT	TT	TT	CT	TT	CT	TT	CT	CT	TT
AC	AC	AC	CC	AC	AA	AC	AA	AC	AA	CC	AC	AA
GG	GT	GG	GT	GG	TT	TT	--	GT	TT	GG	GG	GG
GG	AG	GG	AG	GG	AA	AA	AA	AG	AA	GG	GG	GG
CC	CT	CC	CT	CC	TT	TT	TT	CT	TT	CC	CC	CC
TT	AT	TT	AT	TT	AA	AA	AA	AT	AA	TT	TT	TT
AA	AC	AA	AC	AC	CC	CC	CC	AC	CC	AA	AA	AA
CC	CC	CC	CC	CC	CG	CG	CG	CC	CG	CC	CC	CC
AA	AG	AA	AA	AA	AG	AG	AG	AA	AG	AA	AA	AA
TT	CC	TT	CT	--	CC	CC	CC	CT	CC	TT	TT	TT
TT	CC	TT	CT	TT	CC	CC	CC	CT	CC	TT	TT	TT
GG	AA	GG	AG	GG	AA	AA	AA	AG	AA	GG	GG	GG
CC	CT	CC	CT	CC	CT	CT	CT	CT	--	CC	CC	CC
GG	AA	GG	AG	--	AA	AA	--	AG	AA	GG	GG	GG
AA	GG	AA	AG	AA	GG	GG	--	AG	GG	AA	AA	AA
TT	GG	TT	GT	TT	GG	GG	GG	GT	GG	TT	TT	TT

CC	TT	CC	CT	CC	TT	TT	TT	CT	TT	CC	CC	CC
CC	GG	CC	CG	CC	GG	GG	GG	GG	GG	CC	CC	CC
GG	TT	GG	GT	GG	TT	TT	TT	TT	TT	GG	GG	GG
GG	AA	GG	AG	--	AA	AA	AG	AA	--	GG	GG	GG
TT	CC	TT	CT	TT	CC	CC	CC	CC	CT	TT	TT	TT
TT	GT	TT	GT	TT	GT	GT	--	GT	TT	TT	TT	TT
CC	GG	CC	CG	CC	GG	GG	GG	GG	CG	CC	CC	CC
GG	AA	GG	AG	GG	AA	AA	AA	AA	AG	GG	GG	GG
GG	AG	GG	AG	GG	AG	AG	GG	AG	GG	GG	GG	GG
CC	AA	CC	AC	CC	AA	AA	AA	AA	AC	CC	CC	CC
CC	CG	CC	CG	CC	CG	CG	CG	CG	CC	CC	CC	CC
AA	GG	AA	AG	AA	GG	GG	GG	GG	AG	AA	AA	AA
CC	AA	CC	AC	CC	AA	--	AA	AA	AC	CC	CC	CC
GG	TT	GG	GT	GG	TT	TT	TT	TT	GT	GG	GG	GG
AA	CC	AA	AC	AA	CC	CC	CC	CC	AC	AA	AA	AA
CC	CT	CC	CC	CC	CT	CT	CT	CT	--	CC	CC	CC
GG	TT	GG	GG	GG	TT	TT	GT	TT	GT	GG	GG	GG
GG	AG	GG	GG	GG	AG	AG	GG	AG	AG	GG	GG	GG
TT	CC	TT	TT	TT	CC	CC	CT	CC	CT	TT	TT	TT
CC	CT	CC	CC	CC	CT	CT	CT	CT	CC	CC	CC	CC
TT	AA	TT	TT	TT	AA	AA	AT	AA	AT	TT	TT	TT
GG	CC	GG	GG	GG	CC	CC	CG	CC	CG	GG	GG	GG
TT	GG	TT	TT	TT	GG	GG	GT	GG	GT	TT	TT	TT
GG	AG	GG	GG	GG	AG	GG	GG	AG	AG	GG	GG	GG
GT	TT	TT	GT	GT	TT	TT	TT	TT	GT	TT	GT	GT
TT	CC	CT	TT	TT	CC	CT	CT	CC	CT	CT	TT	TT
AA	CC	AC	AA	AA	CC	AC	AC	CC	AC	AC	AA	AA
CT	CC	CT	CT	CT	CC	CT	--	CC	CC	CT	CT	CT
CC	GG	CG	CC	CG	CG	CG	CG	GG	CG	CG	CC	CC
TT	CC	CT	TT	CT	CT	CT	TT	CC	CT	CT	CT	TT
TT	GT	TT	TT	TT	GT	TT	TT	GT	GT	TT	GT	TT
TT	TT	TT	TT	CT	TT	TT	TT	CT	TT	CT	TT	CT
TT	AT	TT	TT	--	AT	TT	--	AT	AT	TT	AT	TT
AC	CC	AC	AC	AC	CC	AC	AC	CC	CC	AC	CC	AC
CC	CT	CT	CC	CT	CT	CC	--	TT	CT	CC	CT	CT
AG	AA	AG	AG	AG	AA	AG	AG	AA	AA	AG	AA	AG
AG	GG	AG	AG	AG	GG	AG	AG	GG	GG	AG	GG	AG
CG	CG	CG	CG	--	CG	GG	GG	GG	CG	GG	CG	GG
TT	CT	TT	TT	TT	CT	CC	CT	CC	TT	CC	TT	CT
TT	CC	TT	CT	TT	CT	CC	CT	CC	TT	CC	TT	CT
TT	CC	TT	CT	TT	CT	CC	CT	CC	TT	CC	TT	CT
CC	TT	CT	CT	CC	CT	TT	CT	TT	CC	TT	CC	CT
TT	GG	GT	GT	TT	GT	GG	TT	GG	TT	GG	GT	GT
CT	TT	CT	CT	CC	CT	TT	CC	TT	CC	TT	CT	CT
AT	TT	AT	AT	--	AT	TT	--	TT	AA	AT	AT	AT
AG	GG	AG	AG	AA	AG	AG	AA	GG	AA	AG	AG	AG
AC	AA	AC	AC	CC	AC	AC	CC	AA	CC	AC	AA	AC

AG	GG	AG	AG	AA	AG	AG	AA	GG	AA	AG	GG	AG
AG	GG	AG	AG	AA	AG	AG	AA	GG	AA	AG	GG	AG
CT	CC	CT	CT	TT	CT	CT	TT	CC	TT	CT	CC	CT
AG	AA	AG	AG	GG	AG	AG	--	AA	GG	AG	AA	AG
GT	TT	GT	GT	GG	GT	GT	GG	TT	--	GT	TT	GT
CT	CC	CT	CT	TT	CT	CT	TT	CC	TT	CT	CC	CT
AG	AA	AA	AG	--	AG	AG	--	AA	GG	AG	AA	AG
AG	AA	AG	AG	GG	AG	AG	GG	AA	GG	AG	AA	AG
AG	GG	AG	AG	AA	AG	AG	AA	GG	AA	AG	GG	AG
CG	CC	CG	CG	GG	CG	CG	GG	CC	GG	CG	CC	CG
AG	GG	AG	AG	--	AG	AG	AA	GG	AA	AG	GG	AG
AC	CC	AC	AC	AA	AC	AC	AA	CC	AA	AC	CC	AC
AG	AA	AG	AG	GG	AG	AG	GG	AA	GG	AG	AG	AG
AG	AA	AG	AG	GG	AG	AG	AG	AA	GG	AG	AG	AG
AT	TT	AT	AT	AA	AT	AT	AT	TT	AA	AT	AT	AT
CT	CC	CT	CT	TT	CT	CT	CT	CC	TT	CT	CT	CT
CT	CT	TT	CT	TT	TT	CT	--	CC	TT	CT	CT	CT
CG	CG	CG	CG	CC	CC	CG	CG	GG	CC	CG	CG	CG
CT	CT	CT	CT	CC	CC	CT	CC	TT	CC	CT	CT	CT
CT	CT	CT	--	TT	TT	CT	CT	CC	TT	CT	CT	CT
CT	CT	CT	CT	CC	CC	CT	--	TT	CC	CT	CT	CT
TT	AT	AT	AA	AA	AA	AT	AT	TT	AA	AT	AT	AT
AA	AT	AT	AT	TT	TT	AT	TT	AA	TT	AT	AT	AT
TT	CT	CT	CT	CC	CC	CT	CT	TT	CC	CT	CT	CT
CC	CT	CT	CT	TT	TT	CT	CT	CC	TT	CT	CT	CT
AA	AT	AT	AT	TT	TT	AT	AT	AA	TT	AT	AT	AT
GG	AG	AG	AG	AA	AA	AG	AG	--	AA	AG	AG	AG
GG	GT	--	--	TT	TT	GT	--	GG	TT	GT	GT	GT
TT	CT	--	CT	CC	CC	CT	CT	TT	CC	CT	CT	CT
CC	CG	CG	CG	--	GG	CG	CG	CC	GG	CG	CG	CG
GG	AG	AG	AG	AA	AA	AG	AG	GG	AA	AG	AG	AG
TT	CT	CT	CT	CC	CC	CT	CT	TT	CC	CT	CT	CT
TT	CT	CT	CT	CC	CC	CT	CT	TT	CC	CT	CT	CT
TT	AT	AT	AT	AA	AA	AT	AT	TT	AA	AT	AT	AT
--	AG	AA	AG	GG	GG	AG	AG	AA	GG	AG	AG	AG
AA	AG	AG	AG	GG	GG	AG	--	AA	GG	AG	AG	AG
TT	GT	GG	GT	GG	GG	GT	GT	TT	GG	GT	GT	GT
AA	AG	AG	AG	GG	GG	AA	AG	AA	GG	AA	AG	AG
GG	AG	GG	AG	AG	AG	GG	GG	GG	AG	GG	AG	GG
CT	TT	--	CT	CT	TT	TT	CT	CT	TT	CT	CT	CT
AG	AG	AA	AG	--	GG	AG	AA	AG	AG	AA	AG	AG
GG	AG	AG	GG	GG	GG	AG	GG	GG	AG	AG	GG	GG
AG	--	AA	AG	AG	GG	AG	AA	AG	AG	AA	AG	AG
CG	CG	GG	CG	CG	CC	CG	GG	CG	CG	CG	CG	CG
CT	CT	CC	CT	CT	TT	CT	CC	CT	CT	CT	CT	CT
CC	AC	CC	CC	CC	AC	AC	CC	CC	AC	CC	CC	CC
AG	AG	AA	AG	AG	AG	AG	AA	AA	AA	AG	AG	AA

CC	AC	CC	CC	CC	AC	AC	CC	AC	--	CC	CC	CC
CC	CC	AC	CC	CC	CC	CC	AC	AC	AC	CC	CC	AC
TT	CT	TT	TT	TT	CT	CT	TT	CT	CT	TT	TT	TT
AG	GG	AA	AG	AG	AG	GG	AA	GG	AG	AG	AG	AA
AG	AG	GG	AG	AG	AG	AA	GG	AA	AG	AG	AG	GG
CT	CT	TT	CT	CT	CT	CT	TT	CC	CT	CT	CT	TT
TT	CT	CT	TT	TT	CT	CT	CT	TT	CT	TT	TT	CT
CG	CG	CC	CG	CG	CG	CG	CC	GG	CC	CG	CG	CC
AG	AG	AA	AG	AG	AG	AG	AA	GG	AA	AG	AG	AA
CT	TT	TT	CT	TT	CT	CC	TT	CC	TT	CT	CT	TT
AT	AT	AT	AA	AT	AT	AA	--	AA	AT	AT	AT	AT
AA	CC	AA	AC	AC	AC	AA	CC	AA	CC	CC	AC	CC
AG	GG	AG	GG	AG	AG	AG	GG	AG	GG	GG	AG	GG
CT	CC	CT	CC	CT	CT	CT	CC	CT	CC	CC	CT	CC
CT	CC	CT	CC	CT	CT	CT	CC	CT	CC	CC	CT	CC
GT	GG	GT	GG	GT	GT	GT	GG	GT	GG	GG	GT	GG
CC	CC	TT	CT	CT	CT	CT	CT	CC	CT	CT	CT	CC
AG	AG	GG	GG	GG	GG	GG	GG	AG	GG	AG	GG	AG
AA	AA	GG	AG	AG	AG	AG	AG	AA	AG	AG	AG	AA
GG	GG	CG	--	GG	CG	GG	CG	CG	GG	CG	GG	GG
AA	AA	GG	AG	AG	GG	AG	GG	AG	AG	AA	AG	AA
GG	GG	AA	AG	AG	AA	AG	AA	AG	AG	GG	AG	GG
GT	GT	TT	GT	GT	TT	GT	TT	TT	GT	GT	GT	GT
GG	GG	AA	AG	AG	AA	AG	AA	AG	AG	GG	AG	GG
GG	GG	AA	AG	AG	AA	AG	AA	AG	AG	GG	AG	GG
GT	GT	GG	GT	GT	GG	GT	GG	GG	GT	GT	GT	GT
CT	--	CC	CT	CT	CC	CT	CC	CT	CT	TT	TT	TT
CT	CT	TT	CT	CT	TT	CT	TT	TT	CT	CC	CC	CC
CC	CT	CC	CC	CC	CC	CC	CC	CC	CC	CT	CT	CT
AA	AG	AA	AG	AG	AG	AG	AA	AG	AG	GG	GG	GG
CC	CT	CC	CT	CT	CT	CT	CC	CT	CT	TT	TT	TT
AA	AG	AA	AG	AG	AG	AG	AA	AG	AG	GG	GG	GG
CC	CC	CC	CT	CT	CT	CT	CC	CT	CC	TT	TT	TT
GG	GG	GG	CC	CG	CG	CG	CG	CG	--	CC	--	CC
CC	CC	CC	TT	CT	CT	CT	TT	CT	CC	CT	TT	CT
CC	CC	AA	AC	AC	AC	AC	--	AA	AC	CC	AC	CC
AA	AA	AA	AG	AG	AG	AG	GG	GG	AG	AA	AG	AA
CC	CC	CC	CT	CT	CT	CT	TT	TT	CT	CC	CT	CC
GG	GG	GG	AG	GG	AG	AG	AA	AA	AG	GG	AG	GG
GG	GG	GG	AG	GG	AG	AG	AA	AA	AG	AG	AG	AG
CT	CT	CT	TT	CT	TT	CT	TT	TT	TT	CT	CT	CT
AA	AA	AA	AG	AA	AG	AG	AG	GG	AG	AG	AG	AG
AG	AG	AG	AG	AG	AG	AA	AG	AA	AG	AA	AG	AA
GT	GT	GT	GT	GT	TT	TT	GT	GT	GT	TT	GT	TT
GT	GT	GT	GT	GT	GT	TT	GT	TT	GT	TT	GT	TT
CC	CC	CC	AC	AC	AA	AC	AC	AC	AC	AC	CC	AC
GG	GG	GG	AG	--	AA	AG	AA	AG	--	AG	GG	AG

GG	GG	GG	CG	GG	CC	CG	CC	CG	CG	CC	GG	CG
GG	GG	GG	CG	GG	CC	CG	CC	CG	CG	CG	GG	CG
CC	CC	CC	CT	--	TT	CT	TT	CT	CT	CT	CC	CT
TT	TT	TT	CT	--	CC	CT	CC	CT	CT	CT	TT	CT
AT	TT	TT	AT	TT	AA	AT	AA	AT	AT	AT	TT	AT
AG	GG	AG	GG	GG	AG	AG	AG	GG	GG	GG	GG	AG
CC	TT	CT	CT	CT	CC	CT	CC	CT	CT	CT	TT	CT
TT	CC	CT	CT	CT	TT	CT	TT	CT	CT	CT	CC	CT
CC	TT	CT	CT	--	CT	CT	CC	CT	CT	CT	TT	CC
GG	AA	AG	AG	AG	AG	AG	GG	GG	GG	AG	AA	GG
CC	CG	CG	CG	CG	CC	CG	CC	CC	CC	CC	CG	CC
GG	CG	GG	CG	GG	CG	GG	GG	GG	GG	GG	CG	CG
GG	GG	--	GT	GT	GG	GT	GG	GG	GG	GG	GG	GG
CG	CG	CG	CC	CC	CG	CC	CG	CC	CG	CC	CC	CC
AG	AA	AA	GG	GG	AA	GG	--	GG	AG	AG	AG	GG
AC	AC	AC	AA	AA	AC	AA	AC	AA	AA	AC	AC	AA
AT	AT	AT	AA	AA	AT	AA	AT	AA	AT	AA	AA	AA
AG	AA	AA	GG	GG	AA	GG	AA	GG	AA	AG	GG	GG
AT	TT	TT	AA	AA	TT	AA	TT	AA	TT	AT	AA	AA
AG	AG	AA	GG	GG	AA	AG	AG	GG	AA	AG	GG	GG
CC	--	AC	CC	CC	AC	AC	CC	CC	--	AC	AC	CC
CT	--	CT	CT	CC	TT	CT	CT	CC	TT	CT	CT	CC
GT	GT	GT	GT	TT	GG	GG	GT	GT	GG	GT	GT	TT
GT	TT	GT	GT	TT	GG	GG	--	GT	GG	GT	GT	TT
CT	CC	CT	CT	CC	TT	TT	CT	CT	TT	CT	CT	CC
CG	CC	CG	CG	CC	GG	GG	CG	CG	GG	CG	CG	CC
AG	--	AA	AG	GG	AA	AA	AG	AG	AA	AG	AG	GG
CT	CC	CT	CT	CC	TT	TT	CT	CT	TT	CT	CT	CC
GT	TT	GT	GT	TT	GG	GG	GT	GT	GG	GT	GT	TT
CT	--	CT	CT	TT	CC	CC	CT	CT	CC	CT	CT	TT
CG	GG	CG	CG	--	CC	CC	CG	CG	CC	CG	CG	GG
AG	GG	AG	AG	GG	AA	AA	AG	AG	AA	AG	AG	GG
AC	AA	AC	AC	AA	CC	CC	AC	AC	CC	AC	AC	AA
CT	TT	--	CT	TT	CC	CC	CC	CT	CC	CT	CT	TT
AG	GG	AG	GG	--	AG	AG	GG	AG	AG	GG	GG	GG
AC	AA	AC	AC	--	CC	CC	AA	AC	CC	AC	AC	AA
CT	CC	CT	CT	CC	TT	TT	CC	CT	TT	CT	CT	CC
AG	GG	AA	AG	GG	AA	AA	GG	AG	AA	AG	AG	AG
CG	GG	CC	CG	GG	CC	CC	--	CG	CC	CG	CG	CG
GT	GG	TT	GT	GG	TT	TT	GG	GT	TT	GT	GT	GT
CT	CC	TT	CT	CC	TT	TT	CC	CT	TT	CT	CT	CT
CT	TT	CT	TT	TT	CT	CT	TT	CT	CT	TT	TT	CT
AG	GG	GG	GG	AG	AG	GG	AG	AG	GG	GG	GG	AG
GG	CG	CG	CG	CG	CG	GG	CC	CG	GG	CG	CG	CG
AT	AT	AT	AT	AA	AA	AT	AA	AA	AT	AA	AT	AA
AG	AA	AA	AA	AG	AG	AG	AA	AG	AG	AA	AA	AG
AC	CC	CC	CC	AC	AC	AC	CC	AC	AC	CC	CC	AC

CT	TT	TT	TT	CT	CT	CT	TT	CT	CT	TT	TT	CT
CT	CT	CT	CT	TT	TT	CT	TT	CT	CT	TT	CT	TT
AT	AG	AG	AG	GT	GT	AT	GG	AT	AT	GG	AG	GT
AT	AA	AA	AA	AT	AT	AT	AA	AT	AT	AA	AA	AT
AA	AG	AG	AG	AG	AA	AA	GG	AA	AA	GG	AG	AG
AA	AC	AC	AC	AC	AA	AA	CC	AA	--	CC	AC	AC
AG	AA	AA	AA	AG	AG	AG	AA	AG	AG	AA	AA	AG
AA	AG	AG	GG	--	AA	AA	GG	AA	AA	GG	AG	AG
GG	--	AA	AA	AG	GG	GG	AA	GG	GG	AA	AA	AG
AG	AG	AG	GG	AA	AA	AA	GG	AA	AA	GG	GG	AG
GT	GG	GG	GG	TT	TT	TT	GG	TT	TT	GG	GG	GT
GG	GG	AG	GG	AG	AG	AG	GG	AG	AG	GG	GG	GG
CT	CC	CT	CC	TT	TT	TT	CC	TT	TT	CC	CC	CT
GG	CC	CG	CC	GG	GG	GG	CG	GG	GG	CC	CC	CG
CT	TT	TT	TT	CT	CT	CT	TT	CT	CT	TT	TT	CT
CT	TT	CT	TT	CT	CT	CT	CT	CT	CT	TT	TT	TT
CC	CC	CT	CC	CC	--	CC	CC	CT	CC	CT	CC	CT
GG	GT	GT	GG	GG	GT	GT	GT	GT	GG	GG	GT	GG
GG	AG	AA	GG	AG	AA	AG	AG	AA	GG	AG	AG	AG
AA	AG	GG	AA	AG	GG	AG	AG	GG	AG	AG	AG	AG
AA	AA	AG	AA	--	AG	AA	AA	AG	AA	AG	AA	AG
GG	GT	TT	GT	GT	TT	GT	GG	TT	GG	GT	GT	GT
AA	AA	AG	AA	AG	AG	AA	AA	AA	AA	AG	AA	AG
CC	CC	CT	CC	CT	CT	CC	CC	CC	CC	CT	CC	CT
AG	AG	AA	AG	--	AA	AG	AG	AG	AG	AA	AG	AA
AG	AG	AA	AG	AG	AG	GG	GG	GG	GG	AG	GG	AG
AG	AG	AA	AG	AG	AG	GG	GG	GG	GG	AG	GG	AG
CG	CG	CC	CG	CG	CG	GG	GG	GG	GG	CG	GG	CG
CG	CG	CC	CG	CG	CG	GG	GG	GG	GG	CC	GG	CG
AG	GG	GG	AG	AG	AG	AG	GG	GG	AG	AA	GG	AG
AG	GG	GG	AG	GG	GG	AG	GG	GG	AG	AG	GG	GG
CT	--	CT	CT	CT	CT	CT	--	TT	CC	CT	TT	CT
AC	CC	CC	AC	AC	CC	AC	--	CC	AC	CC	CC	CC
CG	CC	CC	CG	CG	CC	CG	CC	CG	CG	CC	CC	CC
AC	CC	CC	AC	AC	CC	AC	CC	AC	AC	CC	CC	CC
AC	--	CC	AC	AC	AC	AC	AA	AC	AC	AC	AA	AC
AA	AA	AG	AA	AA	AG	AA	AA	AA	AG	AG	AA	AG
AG	AA	GG	GG	AG	AG	AG	AG	AG	AG	AG	AA	AG
GG	GG	AG	GG	GG	AG	GG	GG	GG	AG	AG	GG	AG
CT	CC	TT	CC	CT	CT	CT	CT	CT	CT	CT	CC	CT
CT	--	TT	CT	--	CT	CC	CT	CT	CT	CC	CC	CT
AG	AA	AG	AA	AG	AA	AA	AG	AG	AA	AA	AA	AA
CT	CC	CT	CC	CT	CC	CC	--	CT	CC	CC	CC	CC
GG	AA	AA	--	--	AG	GG	GG	AG	AG	GG	AG	AA
AC	AC	AC	CC	AC	CC	CC	CC	CC	CC	CC	CC	AC
CC	CT	CT	CT	CC	CT	CC	CC	CT	CT	CC	CT	CT
AG	GG	GG	AG	AG	AG	AA	AG	AG	AG	AA	AG	GG

AA	AG	AG	AG	AA	AG	AA	AA	AG	AG	AA	AG	AG
AG	GG	AG	GG	AG	AG	AA	AG	AG	AG	AA	AG	GG
AC	AA	AC	AA	AC	AC	CC	AC	AC	AC	CC	AC	AA
AG	AG	AG	AA	AG	AG	AG	AG	AG	AG	GG	AG	AA
GT	GT	GT	GG	GT	GT	GT	GT	GG	--	TT	GT	GG
AG	AA	AA	AG	AG	AA	AG	AG	AG	AG	AA	AA	AG
CT	CT	CT	CC	CT	CT	CT	CC	CC	CC	TT	CT	CT
CT	CC	CC	CT	CT	CC	CT	CC	CT	CT	CC	CC	CT
AG	AG	AG	AG	AA	AG	AG	AG	AG	AA	GG	AG	AG
GG	CG	CG	CG	CG	CG	GG	--	GG	CG	GG	CG	GG
TT	TT	CT	CT	CT	CT	TT	TT	TT	CT	TT	CT	TT
AA	AA	AG	AG	GG	AG	GG	--	AG	--	AA	AG	AG
AA	AA	AG	AG	GG	AG	GG	AG	AG	GG	AA	AA	AG
CC	CC	CG	CG	GG	CG	GG	CG	CG	GG	CC	CC	CG
AA	AA	AG	AG	--	AG	GG	--	AG	GG	AG	AA	AG
AG	AG	AG	GG	--	AG	AG	AG	AG	GG	GG	AG	GG
CT	CT	CT	TT	CC	CT	CT	CT	CT	TT	TT	CT	TT
AG	--	AG	GG	AA	AG	AG	AG	AG	GG	GG	AG	GG
CG	CG	CG	CC	GG	CG	CG	CG	CG	CC	CC	CG	CC
AC	AC	AC	--	--	AC	AC	AC	AC	CC	CC	AC	CC
AG	AG	AG	AA	GG	AG	AG	AG	AG	AA	AA	AG	AA
GT	GT	GT	GG	TT	GT	GT	GT	GT	--	GG	GT	GG
AG	AG	AG	GG	AA	AG	AG	AG	AG	AG	GG	AG	GG
AG	AG	AG	GG	AA	AG	AG	AG	AG	AG	GG	AG	GG
AG	AG	GG	AA	GG	AG	AG	GG	AG	AG	AA	AG	AA
AC	AC	AC	AA	CC	AC	AC	AC	AC	AC	AA	AC	AA
AG	AG	AG	AA	GG	AG	AG	AG	AG	AG	AA	AG	AA
AG	AG	AG	AA	GG	AG	AG	AG	AG	AG	AA	AG	AA
CT	CT	CT	TT	CC	CT	CT	CT	CT	CT	TT	CT	TT
CG	CG	CG	GG	CC	CG	CG	CG	CG	CG	GG	CG	GG
AC	AC	AC	AA	CC	AC	AC	AC	AC	AC	AA	AC	AA
CT	CT	--	TT	CC	CT	CT	CT	CT	CT	TT	CT	TT
GT	GT	GT	TT	GG	GT	GT	GT	GT	GT	TT	GT	TT
CT	CT	CT	CC	TT	CT	CT	CC	CT	CT	CC	CT	CC
CG	CG	CG	CC	GG	CG	CG	CG	CG	CG	CC	CG	CC
AT	AT	TT	TT	AA	AT	AT	AT	AT	AT	TT	AT	TT
CT	CT	CT	CC	TT	CT	CT	CT	CT	CT	CC	CT	CC
AG	AG	AG	GG	AA	AG	AG	AG	AG	AG	GG	AG	--
AG	AG	AA	AA	GG	AG	AG	AG	AG	AG	AA	AG	AA
AT	AT	AT	AA	TT	AT	AT	AT	AT	AT	AA	AT	AA
AG	AG	AG	GG	AA	AG	AG	AG	AG	AG	GG	AG	GG
AG	AG	AG	AA	GG	AG	AG	AG	AG	AG	AA	AG	--
CG	CG	CG	CC	GG	CG	CG	CG	CG	CG	CC	CG	CC
AG	AG	AG	AA	GG	AG	AG	AG	AG	AG	AA	AG	AA
CG	--	CG	GG	--	CG	CG	GG	CG	--	GG	CG	GG
AG	AG	AG	GG	AA	AG	AG	AG	AG	AG	GG	AG	GG
AC	AC	AC	AA	CC	AC	AC	--	AC	AC	AA	AC	AA

AG	AG	AG	GG	--	AG	AG	AA	AG	--	GG	AG	GG
CG	CG	CG	CC	GG	CG	CG	GG	CG	CG	CC	CG	CC
CT	CT	CT	CC	TT	CT	CT	CT	CT	CT	CC	CT	CC
AT	AT	AT	TT	--	AT	AT	AT	AT	AT	TT	AT	TT
CT	CT	CT	CC	TT	CT	CT	CT	CT	CT	CC	CT	CC
AT	--	AT	AA	TT	AT	AT	TT	AT	AT	AA	AT	AA
AT	AT	AT	TT	AA	AT	AT	AT	AT	AT	TT	AT	TT
CG	CG	CG	GG	CC	CG	CG	CG	CG	CG	GG	CG	GG
CT	CT	CT	CC	--	CT	CT	CT	CT	CT	CC	CT	CC
AG	AG	AG	AA	GG	AG	AG	AG	AG	AG	AA	AG	AA
AC	--	AC	AA	CC	AC	AC	AC	CC	AC	AA	AC	AA
GT	GT	GT	TT	GG	GT	GT	TT	GG	GT	TT	GT	TT
CT	CT	CT	CC	TT	CT	CT	CT	TT	CT	CC	CT	CC
CG	CG	CG	GG	CC	CG	CG	--	CC	CG	GG	CG	GG
GT	GT	GT	TT	GG	GT	GT	GT	GG	GT	GT	GT	TT
CT	CT	CT	TT	CC	CT	CT	CT	CC	CT	TT	CT	TT
AG	--	--	AA	--	AG	AG	AG	GG	AG	AA	AG	AA
AC	AA	AC	AA	CC	AC	AC	--	CC	AC	AA	AC	AA
CT	CC	--	CC	TT	--	CT	CT	TT	CT	CC	CT	CC
AC	AA	AC	AA	CC	AC	AC	AC	CC	AC	AA	AC	AA
AG	AA	AG	AA	GG	AG	AG	AG	GG	AG	AA	AG	AA
GT	GG	GT	GG	TT	GT	GT	GT	TT	GT	GG	GT	GG
GT	GG	GT	GG	TT	GT	GT	GT	TT	GT	GG	GT	GG
AG	GG	AG	GG	--	AG	AG	AA	AA	AG	GG	AG	GG
CT	CC	CT	CC	TT	CT	CT	CT	TT	CT	CC	CT	CC
CT	CC	TT	CC	TT	CT	CT	CT	TT	--	CC	CT	CC
AG	AA	AG	AA	GG	AG	AG	AG	GG	AG	AA	AG	AA
CT	CC	CT	CC	TT	CT	CT	CT	TT	CT	CC	CT	CC
CT	TT	CT	TT	CC	CT	CT	CT	CC	CT	TT	CT	TT
AG	GG	AG	GG	AA	AG	AG	AG	AA	AG	GG	AG	GG
CT	CC	CT	CC	--	CT	CT	CC	TT	CT	CC	CT	CC
TT	TT	CT	CT	CT	TT	CT	TT	CT	CT	TT	CT	TT
CT	--	CT	CT	TT	CT	CT	CC	TT	CT	CC	CT	CC
AA	AA	AG	AG	GG	GG	AG	AA	GG	AG	AA	AA	AA
AA	AA	AC	AC	AC	CC	AC	AA	CC	AC	AA	AC	AA
CG	CG	CG	CG	CG	CC	--	CG	CC	CG	CG	CC	CG
GG	GG	GG	GT	GT	TT	GT	GG	GT	GT	GG	GT	GT
GG	GG	GG	AG	AG	AG	AG	GG	GG	AG	GG	AG	AA
TT	TT	TT	TT	CT	CT	CT	TT	TT	CT	TT	CT	CC
AG	AG	GG	AG	AG	AG	AG	AG	AG	AG	AG	GG	GG
GG	GG	AG	GG	AG	AG	AG	GG	GG	GG	GG	AG	AA
GG	GG	CG	GG	CG	CG	CG	GG	GG	GG	GG	CG	CC
CT	CT	TT	CT	CT	CT	--	CT	CT	TT	CT	TT	TT
TT	TT	GT	TT	GT	GT	GT	TT	TT	GT	TT	GT	GG
AG	AG	AG	AG	GG	AG	GG	GG	AG	GG	GG	GG	GG
AG	GG	GG	AG	GG	AG	AG	AG	GG	AG	AG	GG	AG
CC	AC	AC	CC	--	CC	CC	--	AC	AC	AC	AA	AC

AA	AT	AT	AT	TT	AA	AA	AA	AT	AT	AT	TT	AT
AA	AT	TT	AT	TT	AA	AA	AA	AT	AT	AT	TT	AT
TT	AT	AA	AT	--	TT	TT	TT	AA	AT	AT	AA	AT
CC	CT	TT	CT	CT	CC	CC	CC	TT	CT	CT	TT	CT
AA	GG	GG	AG	AA	AA	AA	AA	GG	AG	AG	GG	AG
CC	AA	AA	AC	CC	CC	CC	CC	AA	AC	AA	AC	AC
CC	TT	TT	CT	CC	CC	CC	CC	TT	CT	TT	CT	CT
AG	GG	AG	AG	AA	AA	AA	AA	GG	AG	GG	AG	AG
GT	GG	GT	GG	TT	TT	TT	TT	GG	GT	GG	GT	GT
AG	GG	--	GG	AA	AA	AA	AA	GG	AG	GG	AG	GG
CG	CC	CC	CC	CG	CG	CG	CG	CC	CG	CC	CG	CC
AG	GG	GG	GG	AG	AG	AG	AG	GG	AG	GG	AG	GG
GG	CC	CG	CC	GG	GG	GG	GG	CC	CG	CC	CG	CC
GG	AA	AG	AA	GG	GG	AG	GG	AA	AG	AA	AG	AA
AA	GG	AG	--	AA	AA	AG	AA	GG	AG	GG	AG	GG
GG	AA	AG	AA	GG	GG	AG	GG	AA	AG	AA	AG	AA
GT	GG	GG	GG	GT	GT	GT	GT	GG	GT	GG	GT	GG
AA	GG	AG	GG	AA	AA	AG	AA	AG	AG	GG	AG	GG
GT	TT	TT	TT	GT	GT	GT	GT	TT	GT	TT	GT	TT
CC	--	GG	CG	CC	CC	CG	--	CG	CC	CG	CC	GG
TT	CT	TT	CT	CT	CT	TT	TT	CT	CT	CT	CT	CT
GG	CG	CG	CG	CG	CG	GG	CG	CG	--	CG	GG	CG
CC	CC	CG	CC	CC	CC	CC	CG	CC	CC	CC	CC	CC
AA	AA	AA	AC	AC	AC	AA	AA	AC	AA	AC	AA	AC
CT	CC	CC	CT	CT	CT	CC	CC	CC	CC	CT	CC	CT
AG	AA	AA	AG	AG	AG	AA	AG	AA	AA	AG	AA	AG
CT	CC	CC	CC	CT	CT	CC	CC	CC	CC	CT	CC	CT
GG	CG	CG	GG	GG	GG	GG	CG	GG	CG	GG	GG	GG
CT	CC	CT	CC	CT	CT	CC	CC	CC	CC	CT	CC	CT
GG	AG	GG	AA	--	AG	AA	AG	AA	AG	GG	AA	GG
GT	GG	GT	GG	GG	GT	GG	GG	GG	GG	GT	GG	GT
GG	GT	GG	TT	GT	GT	TT	GT	TT	GT	GG	GT	GG
CG	GG	CG	GG	--	CG	GG	GG	GG	GG	CG	GG	CG
CC	CG	CC	--	CG	CG	GG	CG	GG	CG	CC	--	CC
AG	GG	AG	GG	GG	AG	GG	GG	GG	GG	AG	GG	AG
CC	CT	CC	TT	CT	--	--	--	TT	CT	CC	CT	CC
TT	GT	TT	GG	GT	GT	GG	GT	GG	GT	TT	GT	TT
AG	GG	AG	GG	GG	AG	GG	GG	GG	GG	AG	GG	AG
GG	AG	GG	AA	AG	AG	AA	AG	AA	AG	GG	AG	GG
CC	CT	CC	TT	--	CT	TT	--	TT	CT	CC	CT	CC
--	TT	AT	TT	TT	AT	TT	TT	TT	TT	AT	TT	AT
GT	TT	--	TT	--	GT	TT	TT	TT	TT	GT	TT	GT
TT	CT	TT	CC	CT	CT	CC	CT	CC	CT	TT	CT	TT
AG	AA	AG	AA	AA	AG	AA	AA	AA	AA	AG	AA	AG
AA	AT	AA	TT	AT	AT	TT	TT	TT	AT	AA	AT	AA
AG	AA	AG	AA	AA	AG	AA	AA	AA	AA	AG	AA	AG
CT	TT	CT	TT	TT	CT	TT	TT	TT	TT	CT	TT	CT

AT	TT	--	TT	TT	AT	TT	TT	TT	TT	AT	TT	AT
TT	GT	TT	GG	GT	GT	GG	GT	GG	GT	TT	GT	TT
AG	AG	GG	AG	GG	AG	AA	AG	AG	AG	GG	AG	GG
CT	CT	TT	--	--	CT	CC	CT	CT	CT	TT	CT	TT
TT	CT	TT	CT	--	CT	CC	CT	CT	CT	TT	CT	TT
TT	CT	TT	CT	TT	CT	CC	--	CT	CT	TT	CT	TT
TT	GT	TT	GT	TT	GT	GG	GT	GT	GT	TT	GT	TT
GG	CG	GG	CG	CG	CG	CC	GG	CG	GG	GG	CG	GG
AA	AC	AA	AC	AC	CC	CC	AA	AC	AA	AA	AC	AA
GG	AG	GG	AG	AG	AA	AA	GG	AG	GG	AG	AG	GG
AA	AG	AA	AG	AG	GG	GG	AG	GG	AA	AG	AG	AA
CT	CC	CT	CT	--	CT	CT	CT	TT	CC	CC	CT	CT
AC	CC	AC	AC	AA	AC	AC	AA	AA	CC	CC	AC	AC
GT	GT	TT	GT	TT	TT	TT	TT	TT	--	GT	GT	GT
GG	CG	CG	GG	GG	CG	CG	CG	GG	CG	GG	GG	GG
AG	AG	AG	GG	--	AG	--	AG	GG	AA	AG	AG	GG
TT	TT	CT	CT	TT	CT	TT	TT	TT	TT	TT	TT	TT
GT	TT	TT	GT	TT	TT	GT	TT	GT	GT	GT	GT	TT
CC	CC	CT	CT	CC	CT	CC	CT	CC	CC	CC	CC	CC
AC	AA	AA	AA	AA	AA	AC	--	AC	AA	AC	--	AA
AG	AA	AG	AG	AA	AG	AG	AG	AG	AA	AG	AG	AA
AG	AA	AA	AA	AA	AA	AG	--	AG	AA	AG	AG	AA
GG	TT	GG	GT	GT	GT	GT	--	GT	TT	GT	GT	TT
TT	CC	TT	CT	CT	CT	CT	CT	CT	CC	CT	TT	CC
CC	AA	CC	AC	AC	AC	AC	AC	AC	AA	AC	CC	AA
AT	TT	AA	AT	AT	AT	AT	AT	AT	AT	AT	AA	TT
GT	--	GG	GG	GG	GT	GG	GT	GG	GG	GG	GG	--
AA	AG	AA	AA	--	--	AG	AA	AG	AG	AG	AA	AG
CG	CC	GG	GG	CG	CG	CG	CG	CG	CG	CG	GG	CC
AG	AA	GG	GG	AG	AG	AG	AA	AG	AG	AG	GG	AA
GG	CC	CC	CG	CC	GG	CG	GG	CC	CG	CG	GG	GG
GG	AA	AA	AG	AA	GG	AG	GG	AA	--	AG	GG	GG
AG	AA	AA	AG	AA	AG	AA	AG	AA	--	AA	AG	AG
AT	AT	AT	AA	AA	AT	AA	AT	AA	--	AT	AT	AA
AG	AA	AA	AG	--	AG	AA	AG	AA	AG	AA	AG	AG
AA	AG	AG	AA	AG	AA	AG	AA	AG	AA	AG	AA	AA
CC	AC	AC	AC	AA	AC	AA	AC	AA	AC	AC	CC	AC
AC	AC	AC	CC	CC	CC	CC	CC	CC	CC	AC	AC	CC
AC	AA	AC	--	CC	AC	CC	AC	CC	AC	AC	AA	AC
GT	TT	--	GT	GT	GT	GG	--	GG	GT	GG	TT	GT
AT	TT	AA	AT	AT	AT	AA	AT	AA	AT	AA	TT	AT
AA	AG	AG	AA	AG	AA	AA	AA	AA	AA	AA	AG	AA
CC	AC	AC	CC	AC	CC	CC	CC	CC	CC	CC	AC	CC
AT	TT	AT	AT	TT	AT	AA	TT	AA	AT	AT	TT	AT
CT	TT	CT	CT	TT	CT	CC	TT	CC	CT	CT	TT	TT
AG	AA	AG	AG	AA	AA	GG	AA	AG	AG	AG	AA	AA
CT	TT	CT	CT	TT	TT	CC	TT	CT	CT	CT	TT	TT

CT	CC	CT	CT	CC	CC	CT	CC	CT	CT	CT	CC	CC
AG	AA	AG	AG	AA	AA	AG	AA	AG	AG	AG	AA	AA
CT	TT	CT	CT	TT	TT	CT	TT	CT	CT	CT	TT	TT
CT	CC	CT	CT	CC	CC	CT	CC	CT	CT	--	CC	CC
CT	TT	CT	CT	TT	TT	CT	TT	CT	CT	CT	TT	TT
AG	AA	AG	AG	--	AA	AG	AA	AG	AG	AG	AA	AA
CT	CC	CT	CT	CC	CC	CT	CC	CT	CT	CT	CC	CC
AG	--	AG	AG	AA	AA	AG	AA	AG	AG	AG	AA	AA
CG	GG	CG	CG	GG	GG	CG	GG	GG	CG	CG	GG	GG
AC	CC	AC	AC	CC	CC	AC	--	CC	AC	AC	CC	CC
CT	TT	CT	CT	TT	TT	CT	TT	TT	CT	CT	TT	TT
CT	TT	CT	CT	TT	TT	CT	TT	TT	CT	CT	TT	TT
AT	AA	AT	AT	--	AA	--	AA	AA	AT	AT	AT	AA
CT	TT	CT	TT	--	TT	CT	TT	TT	CT	CT	CT	TT
AC	CC	AC	CC	CC	CC	AC	CC	CC	AC	AC	AC	CC
GT	GG	GT	GG	GG	GG	GT	GG	GG	GT	GT	GT	GG
CT	CC	CT	CC	CC	CC	CT	CC	CC	CT	CT	CT	CC
CG	CC	CG	CC	CC	CC	CG	CC	CC	CG	CG	CG	CC
CG	CC	CG	CC	CC	CC	CG	CC	CC	CG	CG	CG	CC
AC	AA	AC	AA	AA	AA	AC	AC	AA	AC	AC	AC	AA
CG	CG	CG	CC	CC	CC	CG	CG	CC	CG	CG	CG	CC
TT	--	AT	AT	AT	AA	AT	--	AT	--	AA	TT	AT
GG	AA	AG	AG	AG	AA	AG	--	AA	AG	AA	GG	AG
GG	AA	AG	AG	AG	AG	AA	AA	AA	AA	AA	GG	AG
AA	AG	AG	AG	GG	AG	GG	GG	AG	GG	GG	AA	AG
GG	AG	AG	GG	AA	AG	AA	AA	AG	AA	AA	GG	GG
CC	AC	AC	CC	AA	AC	AA	AA	AC	AA	AA	CC	CC
GG	AG	AG	GG	AA	AG	AA	AA	AG	AA	AA	GG	GG
GG	AG	AG	GG	AA	AG	AA	AA	AG	AA	AA	GG	GG
TT	GT	--	TT	GG	GT	GG	GG	GT	GG	GG	TT	TT
GG	GT	GT	GG	TT	GT	TT	TT	GT	TT	TT	GG	GG
AA	AC	AC	AA	CC	AC	CC	CC	AC	CC	CC	AA	AA
CC	CT	CT	CC	TT	CT	TT	TT	CT	TT	TT	CC	CC
CC	CT	--	CC	TT	CT	TT	TT	CT	TT	TT	CC	CC
TT	GT	GT	TT	--	GT	GG	GG	GT	GG	GG	TT	TT
TT	AT	AT	TT	--	AT	AA	AA	AT	AA	AA	TT	TT
CC	CG	CG	CC	GG	CG	GG	GG	CG	GG	GG	CC	CC
CC	AC	AC	CC	AA	AC	AA	AA	AC	AA	AA	CC	CC
TT	--	CT	TT	CC	CT	CC	CC	CT	CC	CC	TT	TT
CC	CG	CG	CC	--	CG	GG	GG	CG	GG	GG	CC	CC
TT	CT	TT	TT	CC	CT	CC	CC	CT	CC	CC	TT	TT
TT	AT	AT	TT	AA	AT	AA	AA	AT	AA	AA	TT	TT
CC	CT	CT	CC	TT	CT	TT	TT	CT	TT	TT	CC	CC
AA	AG	GG	AA	GG	AG	GG	GG	AG	GG	GG	AA	AA
TT	AT	AT	TT	--	AT	--	AA	AT	AA	AA	TT	TT
TT	AT	AT	TT	AA	AT	AA	AA	AT	AA	AA	TT	TT
CC	AC	AC	CC	AA	AC	AA	AA	AC	AA	AA	CC	CC

GG	GT	GT	GG	TT	GT	TT	TT	GT	TT	GT	GG	GG
AA	AG	AG	--	GG	AG	GG	GG	AG	GG	AG	AA	AA
TT	GT	GT	TT	GG	GT	GG	GG	GT	GG	GT	TT	TT
GG	AG	AG	GG	AA	AG	AA	AA	AG	AA	AG	GG	GG
CC	CT	CT	CC	--	CT	TT	--	CT	TT	CT	CC	CC
GG	AG	AG	GG	AA	AG	AA	AA	AG	AA	AG	GG	GG
CC	CT	CT	CC	TT	CT	TT	TT	CT	TT	CT	CC	CC
AA	AC	AC	AA	CC	AC	CC	CC	AC	CC	AC	AA	AA
GG	AG	AG	GG	AA	AG	AA	AA	AG	AA	AG	--	GG
CC	CT	CT	CC	TT	CT	CT	TT	CT	TT	CT	CC	CT
AA	AG	AG	AA	GG	AG	--	GG	GG	GG	AG	AA	AG
TT	CT	CT	TT	CT	CT	CT	CC	CC	CC	CT	TT	CT
GT	GG	GG	GT	GG	GG	GG	GG	GG	GG	GT	GT	GT
AG	AG	AG	AG	AG	AG	AG	AG	GG	GG	AG	AG	GG
AA	AT	TT	AA	AA	TT	AT	--	TT	AT	AA	AA	AT
AA	AT	TT	AA	AA	TT	AT	AA	TT	AT	AA	AA	AT
AA	AC	CC	AA	AA	CC	AC	AA	CC	AC	AA	AA	AC
TT	GT	GT	TT	--	GG	GT	TT	GG	GT	TT	TT	GT
TT	TT	TT	CT	TT	TT	CT	--	TT	TT	CT	CT	TT
GG	AG	AG	AA	GG	GG	AG	--	GG	GG	AG	AA	GG
CG	GG	GG	GG	CG	CG	CG	CG	GG	CG	CG	GG	CG
CT	TT	TT	TT	CT	CT	CT	CT	TT	TT	CT	TT	CT
GG	GT	GT	GG	GT	GT	GG	GT	GT	GT	GT	GG	GT
AA	AG	AG	AA	AG	AG	--	AG	AG	AG	GG	AG	GG
AA	--	AA	AA	AC	AA	AC	AC	AA	AA	AC	AC	AC
GG	GG	GG	GG	CG	GG	CG	GG	GG	GG	CG	CG	CG
TT	TT	TT	TT	GT	TT	GT	GT	TT	TT	GT	GT	GT
CC	CT	CT	CC	CT	CT	CT	CT	CT	CT	TT	CT	TT
AG	GG	AA	AG	AG	GG	AA	AA	AG	GG	AG	AA	AG
CC	CC	CT	CC	CC	CC	CT	CT	CC	CC	CT	CT	CT
CG	CG	CC	CG	CG	CG	CC	CC	CG	CG	CC	CC	CC
AC	AC	AC	AC	--	AA	AC	AC	AC	AA	AA	AC	AC
GG	GG	GG	GG	GG	AG	AG	AG	AG	AG	AA	AG	AG
TT	TT	TT	TT	TT	CT	TT	--	TT	CT	CT	TT	TT
GG	GG	CG	GG	GG	GG	CG	CG	CG	GG	CG	CG	CG
AA	AA	AG	AA	AA	AG	AG	AG	AG	AG	GG	AG	AG
CG	CG	GG	CG	CG	CG	GG	GG	CG	CG	GG	GG	GG
CC	CC	CG	CC	CC	CG	CG	CG	CC	CG	GG	CG	CG
CC	CC	CT	CC	CC	CT	CT	CT	CC	CT	TT	CT	CT
GG	GG	AG	GG	GG	AG	AG	AG	GG	AG	AA	AG	AG
AG	AG	AG	AG	AG	GG	AG	AG	AG	GG	GG	AG	AG
CC	CC	CC	CC	--	CT	CT	CT	CT	CT	TT	CT	CT
GG	GG	GG	GG	GG	AG	GG	GG	GG	AG	AG	GG	GG
GG	GG	GG	GG	GG	AG	AG	--	AG	AG	AA	AG	AG
CC	CC	CC	CC	CC	AC	AC	--	AC	AC	AA	AC	AC
TT	--	TT	TT	TT	TT	AT	AT	AT	TT	AT	AT	AT
AA	AA	AA	AA	AA	AG	AG	AG	AG	AG	GG	AG	AG

AA	--	AA	AA	AA	AC	AA	AA	AC	AC	CC	AC	AC
AA	AA	AA	AA	AA	AG	AA	AA	AG	AG	GG	AG	AG
AA	AA	AA	AA	AA	AG	AA	AA	AA	AG	AG	AA	AA
CC	CC	CC	CC	CC	CC	CC	CC	CT	CC	CT	CT	CT
CC	CC	CC	CC	CC	CG	CC	CC	CG	CC	CG	CG	CG
TT	TT	TT	TT	TT	CT	TT	--	CT	TT	CT	CT	CT
CG	CG	CG	CG	--	GG	CG	CG	GG	GG	GG	GG	GG
CC	CC	CC	CC	CC	CT	CC	CC	CT	CT	CT	CT	CT
GT	GT	GT	GT	GG	GG	GT	GT	GG	GG	--	GG	GG

2-58	2-59	2-61	2-62	2-63	2-64	2-65	2-68	2-73	2-75	2-76	2-78	2-80
AA	--	AC	AC	AC	AC	CC	AC	AC	CC	CC	AC	--
AA	AA	AG	AG	AG	AG	GG	AG	AG	GG	GG	AG	AA
TT	--	CT	CT	--	CT	--	CT	CT	CC	--	CT	--
GG	--	GG	AG	GG	AG	AG	AG	AG	AG	AG	GG	--
AA	--	AA	AG	AG	AG	--	GG	AG	GG	AG	--	--
CG	--	CG	CG	--	CG	--	GG	CG	GG	CG	GG	--
TT	CT	TT	CT	CC	CT	CC	CC	CT	CC	CT	CT	--
TT	GT	TT	GT	GT	GT	GG	GG	GT	GG	GT	GT	TT
TT	GT	TT	GT	GT	GT	GT	GG	GT	GG	GT	GT	--
TT	--	TT	GT	GT	GT	GT	GG	GT	GG	GT	GT	--
AA	AG	AA	AG	AG	AA	AG	GG	AG	GG	AG	AG	--
CT	--	CT	CT	CC	CT	CC	CC	CT	CC	CT	--	--
AA	--	AA	AC	--	AA	AC	CC	AC	CC	AC	AC	AA
AG	--	AG	AG	AA	AG	AA	AA	AG	AA	AG	AA	AG
CT	--	CT	CC	CC	CT	CT	CC	CC	CC	CC	CT	CT
GG	AG	GG	AG	AA	GG	GG	AA	AG	AA	AG	AG	--
TT	--	AT	AT	TT	AT	AT	AT	AT	TT	AT	TT	--
CC	CT	CT	CC	CC	CT	CT	CC	CC	CC	CC	CC	CT
TT	TT	GT	TT	TT	GT	GT	GT	GT	TT	GT	TT	GT
CG	--	CG	CC	CC	CG	CG	CG	CG	CG	CG	CC	CG
AT	--	AA	TT	--	AA	AT	AT	AT	AT	AT	--	--
CT	--	CT	TT	CT	CC	--	CT	CT	CT	CT	CT	--
AT	AT	AT	AA	AT	TT	AT	AA	AT	AT	AT	AT	--
AT	AT	AT	AA	AT	TT	AT	AT	AT	AT	AT	AT	--
AG	AG	AG	AA	AG	GG	AG	AG	AG	AG	AG	AG	--
GT	--	TT	TT	GT	--	--	GT	GT	GT	GT	GT	GT
GT	--	GT	TT	GT	GG	GT	GT	GT	GT	GT	GT	--
AT	--	AT	TT	AT	AA	--	AT	AT	AT	AT	AT	--
CT	CT	CT	TT	CT	CC	CT	CT	CT	CT	CT	CT	CC
CG	--	CG	CC	CG	GG	CG	CG	CG	CG	CG	CG	GG
CT	CT	CT	CC	CT	TT	CT	CT	CT	CT	CT	CT	--
AC	AC	AC	AA	AC	CC	AC	AC	--	--	AC	AC	--
AG	--	AG	AA	AG	--	AG	AG	AG	AG	AG	AG	--
GT	GT	GT	GG	GT	TT	GT	GT	GT	GT	GT	GT	--
AG	--	AG	AA	AG	GG	AG	AG	AG	AG	AG	AG	--
TT	GT	GT	TT	GT	GG	GT	GT	GT	--	GT	--	--
AG	AG	AG	GG	AG	AA	AG	GG	AG	AG	AG	AG	--
CT	--	CT	CC	CT	TT	CT	CC	CT	CT	CT	CT	--
AT	AT	AT	AA	AT	TT	AT	AA	AT	AT	AT	AT	--
AG	AG	AG	AA	AG	GG	AG	AA	AG	AG	AG	--	--
AC	AC	AC	AA	AC	CC	AC	AA	AC	AC	AC	AC	--
AG	--	AG	AA	--	GG	AG	AA	AG	AG	AG	AG	--

CT	CT	CT	TT	CT	CC	--	TT	CT	--	CT	CT	--
CG	--	CG	CC	--	GG	--	CC	CG	--	CG	CG	--
CT	--	CT	TT	CT	CC	CT	TT	CT	CT	CT	CT	--
CT	--	CT	CC	--	TT	CT	CC	CT	--	CT	--	--
AC	AC	AC	AC	AC	--	AC	AA	AC	AA	AC	AC	--
CC	--	TT	CT	CT	TT	CT	CC	CT	--	CT	CT	--
TT	CT	CC	CT	CT	CC	CT	TT	CT	TT	CT	CT	--
GG	--	AG	AG	AG	GG	AA	AG	AG	AA	AG	GG	--
GG	TT	GT	GT	GT	GG	GT	GT	GT	TT	GT	GG	TT
GG	--	AG	AG	AA	GG	AG	AG	AG	AA	AG	GG	--
CT	--	CT	CT	--	TT	CT	TT	CT	CC	CT	TT	--
CG	--	CG	CG	GG	CG	CG	CC	CG	GG	GG	CC	CG
AG	--	AG	AG	GG	AG	AG	AA	AG	AG	GG	AA	--
AC	--	AC	AC	AA	AC	AC	CC	AC	AC	AA	CC	--
AG	--	AG	AG	GG	AG	GG	AG	AG	GG	GG	AG	--
CT	--	CT	CT	CT	CT	CT	TT	CT	CT	CC	--	CT
CT	--	CT	CT	CT	CT	CT	CC	CT	CT	TT	CT	CT
CT	--	CT	CT	CT	CT	CT	TT	TT	CT	CC	CT	CT
AA	--	AG	AG	AG	AG	AG	AG	AA	AG	GG	AG	AG
AA	AC	AC	AC	AC	AC	AC	AC	AC	AC	CC	AC	--
AA	--	AG	AG	AG	AG	AG	AG	AG	AG	GG	AG	AG
GG	CG	CG	CG	CG	CG	--	CG	CC	CG	CC	CG	CG
CC	CG	CG	CG	CG	CG	CG	CG	GG	CG	GG	CG	--
AA	AG	AG	AG	AG	AG	AG	AG	GG	AG	GG	AG	--
CC	--	AC	AC	AC	AC	AC	AC	AA	AC	AA	AC	--
CT	CT	CT	CT	CT	CT	CT	CT	CC	CT	CC	CT	--
CT	CT	CT	CT	CT	CT	--	CT	TT	CT	TT	CT	CT
AT	--	TT	AA	AT	AT	TT	AT	AA	AT	AA	AT	--
AG	--	GG	AG	AG	GG	--	AG	AG	AG	AG	GG	--
AT	--	TT	AT	TT	AT	TT	AT	AT	TT	AT	AT	TT
GT	--	GG	GG	TT	GG	GT	GG	GG	GT	TT	GG	--
GG	--	AG	AG	GG	AG	--	AG	AG	AG	GG	AG	--
TT	TT	CT	CT	TT	CT	TT	CT	CT	CT	TT	CT	--
GG	--	AG	AG	GG	GG	--	AG	AG	AG	GG	AG	--
GT	GT	TT	TT	GG	GT	GT	TT	GT	GT	GG	GT	--
GG	--	GT	GT	GG	GG	GG	GT	GT	GT	GG	GT	--
CT	CT	CC	CC	TT	CT	CT	CC	CT	TT	TT	CT	CT
CT	CT	CC	CC	TT	TT	CT	CC	CT	CT	CT	CT	--
AG	AG	GG	GG	AA	AA	AG	GG	AG	AG	AG	AG	AG
CG	CG	CC	CC	GG	GG	CG	CC	CG	CG	--	CG	--
AG	--	GG	GG	AA	AA	AG	GG	AA	AG	AG	AG	AG
CT	--	TT	TT	CC	CC	CT	TT	CC	CT	CT	CT	--
CT	--	CT	TT	CC	CC	CT	TT	CC	CT	TT	CC	CT
CC	CC	CC	CG	CC	CC	CC	CG	CC	CC	CG	CC	--
TT	--	TT	AT	--	TT	--	AT	TT	TT	AT	TT	AT
GT	--	GT	GT	GG	GG	GT	GT	GG	GT	GT	GG	GT
CT	CC	CT	CT	--	CC	CT	CT	CC	CT	CT	CC	CT

CT	--	CT	TT	CC	CC	CT	TT	CC	CT	CT	CC	--
GT	--	GT	GT	GG	GG	GT	GT	GG	GT	GT	GG	GT
CT	--	CT	TT	CC	CC	CT	TT	CC	CT	CT	CC	CT
GT	TT	GT	GT	TT	TT	GT	GT	TT	GT	GT	TT	GT
CG	--	CG	CG	--	GG	CG	CG	GG	CG	CG	GG	--
AG	--	GG	GG	AG	GG	--	AA	GG	AA	AG	AG	--
AG	AA	AA	AA	AG	AA	AA	GG	AA	GG	AG	AG	--
AG	--	AA	AA	AG	AA	AA	GG	AA	GG	AG	AG	--
CT	--	TT	TT	CT	TT	CT	CC	TT	CC	CT	CT	--
AG	--	GG	GG	AG	GG	AG	AA	AG	AA	AG	AG	--
GT	--	GG	GT	GT	GT	--	TT	GT	TT	GT	GT	--
GG	AG	GG	GG	AG	GG	GG	GG	GG	GG	GG	GG	GG
CT	--	CT	CT	CT	CT	CT	CC	CT	CC	CT	CT	--
CC	--	CC	CC	AC	--	--	AC	CC	AC	CC	CC	AC
CT	CT	CT	CT	CC	CT	CT	CC	CT	CC	CT	--	--
--	--	GT	GT	--	GT	GT	TT	GT	--	GT	GT	--
CC	CC	CT	CT	CC	CT	CT	CT	CT	CT	CT	--	--
GG	GG	GG	GG	CG	GG	GG	CG	GG	CG	GG	GG	CG
CC	--	CC	CC	AC	CC	CC	AC	CC	AC	CC	CC	--
TT	TT	CT	CT	TT	CT	CT	CT	CT	CT	CT	CT	CT
AA	--	AG	AG	AA	AG	AG	AA	AG	AG	AG	AG	AG
AA	AA	AG	AG	AG	AG	AG	AG	AG	GG	AG	AG	--
CT	CT	CT	CT	--	CT	CT	CC	CT	CC	CT	CT	--
AA	--	AA	AA	AT	AA	AA	AT	AA	AT	AA	AA	AT
CC	--	CC	CC	CT	CC	CC	CT	CC	CT	CC	CC	CT
AA	--	AA	AA	AG	AA	--	AG	AA	AG	AA	AA	--
CC	--	CT	CT	CC	CT	CT	CC	CT	CT	CT	CT	--
TT	--	AT	AT	AT	AT	AT	AT	AT	AA	AT	AT	--
TT	--	AT	AT	AT	AT	AT	AT	AT	AT	AT	AT	AT
TT	TT	CT	CT	CT	CT	CT	CT	CT	CC	CT	CT	CT
CC	--	CC	CC	CT	CC	CC	CT	CC	CT	CC	CC	CT
GG	--	GT	GT	--	GT	GT	GG	GT	GT	GT	GT	--
CC	--	CT	CT	CC	CC	CT	CC	CT	CT	CT	CT	--
AG	--	AA	AA	AG	AG	AA	AG	AA	AA	AA	AA	AG
GG	--	AG	AG	AG	GG	AG	AG	AG	--	AG	AG	--
AA	AA	AG	AG	AA	AA	AG	AA	AG	AG	AG	AG	AA
CC	--	CT	CT	CT	CC	CC	CT	CT	TT	CT	CT	--
CC	--	AC	AC	CC	CC	CC	CC	AC	AC	AC	AC	--
--	--	AT	AT	AT	TT	--	AT	AT	AA	AT	AT	AT
AA	--	AA	AA	AG	AA	--	AG	AA	AG	AA	AA	--
AA	AA	AC	AC	AA	AA	AA	AA	AC	AC	AC	AC	AA
TT	--	AT	AT	TT	TT	TT	TT	AT	AT	AT	AT	TT
AC	AC	AA	AA	CC	AC	AC	CC	AA	AC	AA	AA	--
CC	CC	CC	CC	AC	CC	--	AC	CC	AC	CC	CC	AC
AG	AG	GG	GG	AG	AG	AG	AG	GG	GG	--	GG	AG
AC	AC	AC	AC	--	AC	AC	CC	AC	CC	AC	AC	--
AA	--	AG	AG	AA	AA	AA	AA	AG	AG	AG	AG	--

CC	CC	CC	CC	CG	CC	CC	CG	CC	CG	CC	CC	CG
TT	--	CT	CT	CT	TT	TT	CT	CT	CC	CT	CT	CT
CC	--	CT	CT	CT	CC	--	CT	CT	TT	CT	CT	CT
CC	--	CT	CT	CC	CC	--	CC	CT	CT	CT	CT	--
AA	--	AG	AG	--	AA	--	AA	AG	AG	AG	AG	--
CC	--	CT	CT	CT	CC	CC	CT	CT	TT	CT	CT	--
TT	--	CT	CT	TT	TT	--	TT	CT	CT	CT	CT	--
AA	--	AT	AT	AT	AA	AA	AT	AT	TT	AT	AT	--
GG	GG	GG	GG	AG	GG	GG	AG	GG	AG	GG	GG	--
AA	--	AC	AC	AA	AA	AA	AA	AC	AC	AC	AC	--
CC	--	CT	CT	CC	CC	--	CC	CT	CT	CT	CT	--
TT	--	TT	TT	GT	TT	TT	GT	TT	GT	TT	TT	--
TT	--	CT	CT	TT	TT	--	TT	CT	CT	CT	CT	--
AC	AC	CC	CC	AC	AC	AC	AC	CC	CC	CC	CC	AC
AA	--	AG	AG	AA	AA	AA	AA	AG	AG	AG	AG	--
GG	--	GT	GT	--	GG	--	GG	GT	GT	GT	GT	--
AT	--	TT	TT	AT	AT	AT	--	TT	TT	TT	TT	AT
AA	--	AG	AG	AA	AA	AA	AA	AG	AG	AG	AG	--
CC	--	CC	CC	--	CC	--	CT	CC	CT	CC	CC	--
CC	--	CT	CT	CC	CC	--	CC	CT	CT	CT	CT	--
AA	--	AC	AC	AA	AA	AA	AA	AC	AC	AC	AC	--
CC	--	CT	CT	CC	CC	CC	CC	CT	CT	CT	CT	--
AA	--	AT	AT	AT	AA	AA	AT	AT	TT	--	AT	--
GG	GG	AG	AG	AG	GG	GG	AG	AG	AA	AG	AG	--
GG	--	GG	GG	CG	GG	GG	--	GG	--	GG	GG	CG
--	--	CT	CT	--	TT	--	TT	CT	CT	CT	CT	--
GG	--	GG	AG	--	GG	--	GG	AG	AG	AG	AG	--
AA	--	AA	AG	AA	AA	--	AA	AA	AG	AG	AG	--
AA	--	AA	AA	AG	AA	AA	AG	AA	AG	AA	AA	AG
GG	--	GG	AG	AG	GG	GG	AG	GG	AG	GG	GG	AG
CT	--	TT	CT	--	CT	--	CT	TT	CT	CT	CT	--
GT	--	GG	TT	GT	GT	GG	GT	GG	TT	GT	GT	--
TT	--	TT	GT	GT	TT	--	GT	TT	GT	TT	TT	--
AG	--	GG	AA	AG	AG	GG	AA	GG	AA	AG	AG	--
CT	CC	CC	CT	CC	CT	CC	CT	CC	CT	CT	CT	--
GT	TT	GT	GT	TT	GT	TT	GT	TT	GT	GT	GT	TT
AG	AG	AG	AG	GG	AG	AG	GG	AG	AG	AG	AG	--
AG	AG	AG	AG	GG	AG	AG	AA	AA	AA	AA	GG	--
CT	--	CC	CT	CC	CC	CT	CT	CT	CT	CC	CC	--
AT	AT	AA	AT	AT	AA	AT	AA	AA	AA	AA	AT	--
CG	GG	CG	CG	CG	CG	--	CC	CC	CC	CC	GG	CG
AA	--	AC	AA	--	AC	--	AA	AA	AA	AA	AC	--
AA	AG	AG	AG	--	AG	--	AA	AA	AA	AA	GG	--
AG	--	AG	GG	GG	AG	GG	AG	AG	--	AG	GG	AG
CT	--	CT	CC	CC	CT	CC	CT	CT	CT	CT	CC	CT
GG	AG	AG	AG	AG	GG	AG	GG	AG	GG	GG	AA	--
CC	CC	CT	CC	CC	CC	CC	CC	CT	CC	CC	CC	CT

GG	AG	GG	AG	AG	GG	AG	GG	AG	GG	GG	AG	GG
AG	--	GG	AA	AA	AG	AA	AG	AG	AG	AG	AA	--
CC	CT	CT	CT	CT	CC	CT	CC	TT	CC	CC	CT	--
AA	AT	AT	AT	AT	AA	AT	AA	TT	AA	AA	TT	AT
AA	--	AC	AC	CC	AA	AA	AA	CC	AA	AA	CC	AC
TT	--	CT	CT	--	TT	CC	TT	CC	TT	TT	CC	--
GG	--	AG	AG	AA	AG	--	AG	AA	--	GG	--	--
CC	--	CT	CT	--	CT	--	CT	TT	CC	CC	TT	CT
TT	--	CT	TT	--	TT	CT	CT	CT	TT	TT	CT	--
AA	AG	AG	AG	--	AG	AG	AG	GG	AA	AG	--	GG
AA	--	GG	AA	AA	AG	--	AG	GG	GG	AG	GG	--
CC	TT	TT	CC	--	CT	CT	CT	TT	TT	CC	TT	--
GG	--	AG	GG	--	AG	AG	AG	AG	--	GG	AG	--
--	--	TT	CC	CC	CT	CT	CT	TT	CT	CT	--	--
GG	CG	CG	GG	GG	GG	GG	--	CG	CG	CG	CG	--
CC	--	TT	CT	CT	CT	CT	CT	TT	CT	CT	CT	--
CC	CT	CT	CC	CT	CT	--	CT	CT	CC	CC	CC	--
GG	AA	AA	AG	AA	AG	AG	AG	AA	AG	AG	AG	AG
CC	--	TT	TT	TT	CT	CT	CT	TT	CT	CT	CC	CT
AA	GG	GG	GG	GG	AG	AA	AG	GG	AG	AG	AG	--
CC	TT	TT	TT	TT	CT	--	CT	CT	CT	CT	CT	--
GG	--	AG	AA	AA	AG	GG	AG	AG	AG	AG	AG	--
GG	AG	AG	AG	AG	AG	GG	AG	GG	GG	GG	GG	--
GT	GG	GT	GG	GG	GT	TT	GT	GT	GT	GT	GT	--
CC	--	CG	CG	CG	CG	CC	CG	CC	CC	CC	CC	--
CC	--	CG	CC	CC	CG	CG	CG	CC	CC	CC	CC	--
CT	CT	CT	TT	--	CT	CC	CT	CT	CT	TT	CT	--
CT	--	CT	CT	CC	CT	TT	CT	CT	CT	CC	CT	--
AT	--	AT	AT	--	AT	AA	AT	AT	AT	TT	AT	--
AG	GG	AG	AG	AA	AG	GG	AG	AG	AG	AA	AG	--
CT	--	CT	CT	TT	--	--	CT	CT	CT	TT	CT	--
TT	--	TT	TT	CT	CT	TT	CT	TT	TT	CT	CT	--
AC	--	AA	AC	CC	AC	AA	AC	AC	AC	CC	AC	AC
GG	--	TT	GG	GT	GG	GT	GT	GT	TT	GT	--	--
GG	--	AA	GG	AG	GG	AG	AG	AG	AA	AG	AA	--
CC	--	TT	CC	CT	CC	CT	CT	CT	TT	CT	TT	--
--	--	AA	TT	--	TT	AT	AT	AT	--	AT	AA	--
AA	CC	CC	AA	AC	AA	AC	AC	AC	CC	AC	CC	--
CC	CG	CG	CC	CG	CC	CC	CG	CG	CG	CC	CG	--
AA	--	AG	AA	AG	AA	AA	AG	AG	AG	AA	AG	--
TT	--	CC	TT	--	TT	CT	CT	CT	CC	CT	CC	--
TT	--	CC	TT	CT	TT	CT	CT	CT	CC	CT	CC	CC
GG	--	AA	GG	AG	GG	AG	AG	AG	AA	AG	AA	--
CC	--	CT	CC	CC	CC	CT	CC	CC	CT	CT	CT	CT
GG	--	AA	GG	AG	GG	AG	AG	AG	--	AG	AA	--
AA	--	AG	AA	--	AA	AG	AG	AG	GG	AG	GG	--
TT	--	GT	TT	GT	TT	GT	GT	GT	--	GT	GG	--

CC	--	CT	CC	CT	CC	CT	CT	CT	TT	CT	TT	--
CC	GG	CG	CC	CG	CC	CG	CG	CG	GG	CG	GG	GG
GG	TT	GT	GG	GT	GG	GT	GT	GT	TT	GT	TT	--
GG	--	AG	GG	AG	GG	AG	AG	AG	AA	AG	AA	--
TT	--	CT	TT	CT	TT	CT	CT	CT	CC	CT	CC	--
TT	--	TT	TT	TT	TT	GT	TT	TT	GT	GT	GT	--
CC	--	CG	CC	CG	CC	CG	CG	CG	GG	CG	GG	--
GG	--	AG	GG	AG	GG	AG	AG	AG	AA	AG	--	--
GG	--	GG	GG	GG	GG	AG	GG	GG	AG	AG	AG	--
CC	AA	AC	CC	AC	CC	AC	AC	AC	AA	AC	AA	--
CC	CG	CC	CC	CC	CC	CG	CC	CC	CG	CG	CG	CG
AA	--	AG	AG	AG	AA	AG	AG	AG	GG	AG	GG	--
CC	--	AC	AC	AC	CC	--	AC	AC	AA	AC	AA	--
GG	TT	GT	GT	GT	GG	GT	GT	GT	TT	GT	TT	--
AA	--	AC	AC	AC	AA	AC	AC	AC	AC	AC	CC	--
CC	--	CC	CC	CC	CC	CT	CC	CC	CT	CT	CT	CT
GG	TT	GT	GT	GT	GG	GT	GT	GT	TT	GT	TT	--
GG	--	AG	AG	AG	GG	GG	AG	AG	AG	GG	AG	AG
TT	--	CT	CT	CT	TT	CT	CT	CT	CC	CT	CC	--
CC	--	CC	CC	CC	CC	CT	CC	CC	CT	CT	CT	CC
TT	--	AT	AT	AT	TT	AT	AT	AT	AA	AT	AA	--
GG	--	CG	CG	CG	GG	CG	CG	CG	CC	CG	CC	--
TT	GG	GT	GT	GT	TT	GT	GT	GT	GT	GT	GG	--
GG	--	AG	AG	AG	GG	GG	AG	AG	GG	GG	--	--
GT	--	GT	GT	--	GT	--	GT	TT	--	TT	TT	GT
TT	--	CT	CT	TT	TT	TT	CT	CC	CT	TT	CC	--
AA	CC	AC	AC	AA	AA	AA	AC	CC	AC	AA	CC	AA
CT	--	CC	CC	CT	CT	CT	CC	CC	CT	CT	CC	CT
CC	--	CG	CG	CC	CC	--	GG	GG	CG	--	GG	--
TT	--	CT	CT	TT	CT	TT	CC	CC	CT	TT	CC	--
TT	--	GT	GT	TT	GT	TT	GT	GT	TT	TT	TT	--
TT	CT	TT	TT	TT	TT	TT	CT	CT	CT	TT	CT	--
TT	--	AT	AT	TT	AT	--	AT	AT	TT	TT	--	--
AC	CC	CC	CC	AC	CC	AC	CC	CC	AC	AC	AC	--
CC	--	CT	CT	CC	CT	--	TT	CT	CT	CC	CT	--
AG	--	AA	AA	AG	AA	AG	AA	AA	AG	AG	AG	AG
AG	--	AG	GG	AG	GG	--	GG	GG	AG	AG	AG	--
GG	--	CG	GG	CG	CG	--	--	GG	GG	CG	GG	--
CC	--	CT	TT	CT	TT	--	CT	CT	TT	TT	CT	--
CT	CT	CT	TT	CT	TT	CT	CC	CT	CT	TT	CC	--
CT	CT	CT	CT	CT	TT	CT	CC	CT	CT	TT	CC	CC
CT	--	CT	CT	CT	CC	CT	TT	CT	CT	CC	TT	--
GT	GG	GT	GT	GT	TT	TT	GG	GT	GT	TT	GG	--
CC	--	CT	CT	CT	CC	--	TT	CT	CC	CC	TT	--
AA	--	TT	AT	AT	AA	--	TT	AT	AA	AA	TT	--
AA	--	GG	AG	AG	--	AA	GG	AG	AA	AA	GG	--
CC	--	AA	AC	AC	AC	CC	AA	AC	CC	CC	AA	--

AA	--	GG	AG	AG	AG	--	GG	AG	AA	AA	GG	--
AA	--	GG	AG	AG	AG	--	GG	AG	AA	AA	GG	--
TT	--	CC	CT	CT	CT	TT	CC	CT	TT	TT	CC	--
GG	--	AA	AG	AG	AG	GG	AA	AG	GG	GG	AA	--
GG	--	TT	GT	GT	GT	GG	--	GT	GG	GG	TT	--
TT	--	CC	CT	--	CT	--	CC	CT	--	TT	--	--
GG	--	AA	AG	AG	AG	--	AA	AG	GG	GG	AA	--
AG	--	AA	AG	AG	AG	GG	AA	AG	GG	GG	--	--
AA	--	GG	AG	AG	AG	AA	GG	AG	AA	AA	GG	--
GG	CG	CC	CG	CG	CG	--	CC	CG	GG	GG	CC	--
AA	AG	GG	AG	AG	AG	AA	GG	AG	AA	AA	GG	AG
--	--	CC	AC	AC	AC	--	CC	AC	AA	AA	CC	--
GG	--	AA	AG	AG	AG	GG	AA	AG	GG	GG	AA	AG
GG	AG	AA	AG	AG	AG	GG	AA	AG	GG	GG	AA	--
AA	AT	TT	AT	AT	AT	AA	TT	AT	AA	AA	TT	AT
TT	--	CC	CT	CT	CT	--	CC	CT	TT	TT	CC	CT
TT	--	CC	CT	CT	CT	--	CC	CT	TT	TT	CC	--
CC	CG	GG	CG	CG	CG	CC	GG	CG	CC	CC	GG	--
CC	CT	TT	CT	CT	CT	CC	TT	CT	CC	CC	TT	--
--	--	CC	CT	CT	CT	--	CC	CT	TT	TT	CC	--
CC	--	TT	CT	CT	CT	CC	TT	CT	CC	CC	TT	--
AA	AT	TT	AT	AT	AT	AA	TT	AT	AA	AA	TT	--
TT	AT	AT	AT	--	AT	TT	AA	AT	TT	TT	AA	--
CC	--	CT	CT	CT	CT	CC	TT	CT	CC	CC	TT	--
TT	CT	CT	CT	CT	CT	TT	CC	CT	TT	TT	CC	--
TT	AT	AT	AT	AT	AT	TT	AA	AT	TT	TT	AA	--
AA	--	AG	AG	AG	AG	--	GG	AG	AA	AA	GG	--
TT	GT	GT	GT	GT	GT	--	GG	GT	TT	TT	GG	--
CC	--	CT	CT	CT	CT	--	TT	CT	--	CC	TT	--
GG	CG	CG	CG	CG	CG	GG	CC	CG	GG	GG	CC	CG
AA	AG	AG	AG	AG	AG	AA	GG	AG	AA	AA	GG	AG
CC	CT	CT	CT	CT	CT	--	TT	CC	CC	CC	--	--
CC	--	CT	CT	CT	CT	CC	TT	CC	CC	CC	TT	--
AA	--	AT	AT	AT	AT	AA	TT	AA	AA	AA	TT	--
GG	--	AG	AG	AG	AG	GG	AA	GG	GG	GG	AA	--
GG	AG	AG	AG	AG	AG	GG	AA	GG	GG	GG	AA	AG
GG	GT	GT	GT	GG	GT	GT	TT	GG	GG	GG	TT	--
GG	--	AG	AG	GG	AG	--	AA	AG	GG	GG	AA	--
AG	--	AG	AG	AG	GG	GG	GG	AG	AG	AG	GG	--
TT	CT	TT	CT	CT	CT	--	TT	CT	CT	TT	CT	--
AG	--	GG	AA	--	AG	--	GG	AA	--	AG	AA	--
AG	--	GG	AG	AG	AG	AG	GG	AG	AG	AG	AG	--
AA	--	GG	AA	--	AA	--	GG	AA	AA	AG	AA	--
GG	CG	CC	GG	GG	GG	GG	CC	GG	GG	CC	GG	--
CC	--	TT	CC	CC	CC	CC	TT	CC	CC	TT	CC	CT
CC	CC	AC	CC	CC	AC	CC	AC	CC	CC	AC	CC	--
AA	--	AA	AG	AG	AA	--	AG	--	AA	AG	AG	--

CC	--	AC	CC	CC	AC	CC	AC	CC	CC	AC	CC	--
AC	--	AC	CC	CC	AC	--	CC	AC	AC	CC	CC	--
TT	--	CT	TT	TT	CT	TT	CT	TT	TT	CT	TT	--
AA	--	AG	AG	AA	AG	AG	GG	AA	AA	GG	AG	AG
GG	--	AG	AG	GG	AG	AG	AA	GG	GG	AA	AG	--
TT	CT	CT	CT	TT	CT	CT	CT	TT	TT	CC	CT	--
CT	--	CT	TT	CT	CT	--	CT	CT	--	TT	TT	--
CC	--	CG	CG	CC	CG	CG	CG	CC	CG	GG	CG	--
AA	--	AG	AG	AA	AG	AG	AG	AA	AG	GG	AG	--
TT	CT	CT	CT	--	CT	--	CT	TT	CT	CT	CT	--
AT	AT	AT	AT	--	AT	--	AT	AT	AA	AA	AA	--
CC	--	AC	AC	CC	AC	--	AC	CC	AC	AC	AC	--
GG	--	AG	AG	GG	AG	--	AG	GG	--	GG	AG	--
CC	--	CT	CT	--	CT	--	CT	CC	CC	CC	CT	--
CC	CT	CT	CT	--	CT	--	CT	CC	CT	CC	CT	--
GG	--	--	GT	GG	GT	GG	GT	GG	GG	GG	GT	GT
TT	CC	CT	CT	CT	CT	TT	TT	CC	TT	CC	TT	--
GG	AG	AG	AG	AG	AG	--	GG	AG	GG	AG	GG	--
GG	--	AG	AG	AG	AA	GG	GG	AA	GG	AA	GG	--
CG	--	CG	GG	CG	GG	--	CG	GG	CG	GG	GG	--
GG	AG	AA	AA	AG	AA	GG	GG	AA	GG	AA	AG	GG
AA	AG	GG	GG	AG	GG	AA	AA	GG	AA	GG	AG	--
TT	--	TT	GT	TT	GT	TT	TT	GT	TT	GT	GT	GT
AA	--	AG	GG	AG	GG	AA	AA	GG	--	GG	AG	--
AA	--	AG	GG	AG	GG	--	AA	GG	--	GG	AG	--
GG	--	GG	GT	GG	GT	GG	GG	GT	GT	GT	GT	GT
CC	CT	CT	TT	CT	TT	CT	CC	TT	CT	TT	CT	--
TT	CT	CT	CC	CT	CC	CT	TT	CC	CT	CC	CT	--
CC	CT	CT	CT	CT	CT	CC	CC	CT	CC	CT	CC	--
AA	AG	AG	AG	AG	GG	AG	AA	GG	AG	GG	AG	--
CC	CT	CT	CT	CT	TT	--	CC	TT	CT	TT	CT	--
AA	--	AG	AA	AG	GG	GG	AA	GG	AG	GG	AA	--
CC	--	CT	CC	--	CT	--	CC	TT	CT	TT	CC	--
GG	--	CG	GG	CG	CG	--	GG	CG	CG	CC	GG	--
CC	CT	CT	CC	CT	CT	TT	CT	CT	CT	TT	CC	CT
AC	--	CC	AC	AC	AC	AC	AC	AC	AA	AC	CC	--
GG	--	AA	AG	AG	AG	--	AG	AG	GG	AG	AG	--
TT	--	CC	CT	CT	CT	--	TT	CT	TT	CT	CT	--
--	--	GG	AG	AA	AG	GG	AA	AG	AA	AG	AG	--
AA	--	GG	AG	AA	AG	--	AA	AG	AG	AG	AG	--
TT	--	CT	TT	TT	CT	--	TT	TT	TT	CT	CT	--
GG	--	AA	AG	GG	AG	--	GG	AG	AG	AG	AG	--
AA	--	AG	AG	--	AA	AG	AA	AG	AG	AA	AA	--
TT	TT	GT	GT	TT	TT	GT	TT	GT	GT	TT	TT	--
TT	--	GT	GT	TT	TT	GT	TT	GT	GT	TT	TT	--
AA	--	CC	AC	AA	AC	AC	AA	AC	AC	AC	AC	AA
AA	--	GG	AG	AA	AG	--	AA	AG	AG	AG	AG	--

CC	CC	GG	CG	CC	CG	--	CC	CG	CG	--	CG	--
CC	--	GG	CG	--	CG	GG	CC	CG	CG	CG	CG	--
TT	--	CC	CT	TT	CT	CC	TT	CT	TT	CT	CT	TT
CC	--	TT	CC	CC	CT	TT	CC	CT	CC	CT	CT	--
AT	--	TT	AA	AA	AT	AA	AA	AT	AA	AT	AT	--
GG	AG	GG	AG	AG	AG	AG	AG	GG	AG	AG	AG	--
CT	--	TT	CC	CC	CC	--	CC	CT	CC	TT	CT	--
CT	--	CC	TT	TT	TT	--	TT	CT	TT	CC	CT	--
CT	--	TT	CC	--	CC	--	CC	CT	CC	TT	CT	--
AG	--	AA	GG	GG	GG	GG	GG	AG	GG	AA	AG	--
CC	--	CG	CG	CC	CC	CC	CC	CG	CC	CG	CG	--
CG	--	CG	GG	CG	GG	--	CG	GG	--	CG	CG	--
GG	--	GT	GT	GG	GG	--	GT	GT	GT	GT	GT	--
CG	CG	CC	CG	CG	CG	CG	CC	CG	CG	CG	CG	--
AG	--	AG	AG	AG	AG	--	GG	GG	AG	AA	--	AA
AA	--	AA	AC	AC	AC	AC	AA	AA	AC	AC	AC	AC
AT	AT	AA	AT	AT	AT	AT	AA	AT	AT	AT	AT	AT
AA	--	AG	AG	AG	AG	--	GG	AG	AG	AA	AA	--
TT	AT	AT	AT	AT	AT	TT	AT	AT	AT	TT	TT	--
AA	--	AG	AG	AG	AG	AA	AG	AG	AG	AA	AG	AA
AC	CC	AC	CC	CC	CC	CC	AC	CC	CC	AC	CC	--
TT	CT	CT	CC	CT	CC	CT	CT	CT	CT	CT	CT	--
GG	GT	GT	TT	GT	TT	GT	GT	GT	GT	GT	GT	GG
GG	GT	GT	TT	TT	TT	GT	GG	GT	GG	GT	GT	GG
TT	CT	CT	CC	CC	CC	CT	TT	CT	TT	CT	CT	--
GG	CG	CG	CC	CC	CC	CG	GG	CG	GG	CG	CG	--
AA	--	AG	GG	GG	GG	AG	AA	AG	AA	AG	AG	--
CT	CT	CT	CC	CC	CC	CT	TT	CT	TT	CT	CT	--
GG	--	GT	TT	TT	TT	GT	GG	GT	GG	GT	GT	--
CC	CT	CT	TT	TT	TT	CT	CC	CT	CC	CT	CT	--
CC	CG	CG	GG	GG	--	CG	CC	CG	--	CG	CG	CG
AA	--	AG	GG	GG	GG	AG	AA	AA	AA	AG	AG	--
CC	--	CC	AA	--	AA	AC	CC	AC	CC	AC	AC	--
CC	--	CC	TT	TT	TT	CT	CC	CT	CC	CT	CT	CC
AG	--	AG	GG	GG	GG	AG	AG	AG	AG	AG	AG	AG
CC	AC	CC	AA	AA	AA	AC	CC	AC	--	--	--	--
CT	--	TT	CC	CC	CC	CT	TT	CT	TT	CT	CT	--
AG	AG	AA	GG	GG	GG	AG	AA	AG	AA	AG	AG	--
CG	--	CC	GG	GG	GG	CG	CC	CG	--	CG	CG	--
GT	--	TT	GG	GG	GG	GT	TT	GT	TT	GT	GT	--
CT	CT	TT	CC	CC	CT	CT	TT	CT	TT	CT	CT	--
CT	--	CT	TT	TT	TT	CT	CT	CT	CT	CT	CT	--
AG	--	GG	AG	AG	GG	--	GG	AG	GG	AG	AG	--
CG	CG	GG	CC	CC	CG	CG	CG	CG	GG	CG	GG	CG
AA	--	AT	AA	AA	AT	AA	AA	AT	AT	AA	AT	--
AG	AG	AG	AA	AA	AA	AG	AG	AG	AG	AG	AG	--
AC	AC	AC	CC	CC	CC	AC	AC	AC	AC	AC	AC	AC

CT	CT	CT	TT	TT	TT	CT	CT	CT	CT	CT	CT	CT
TT	TT	CT	TT	TT	CT	TT	TT	CT	CT	CT	CT	--
GT	--	AT	AG	GG	AG	--	GT	AT	AT	AT	AT	--
AT	--	AT	AA	AA	AA	AT	AT	AT	AT	AT	AT	AT
AG	--	AA	AG	AG	AG	AA	AA	AA	AA	AA	AA	AG
AC	--	AA	AC	AC	AC	--	AA	AA	AA	AA	AA	AC
AG	AG	AG	AA	AA	AA	AG	AG	AG	AG	AG	AG	AG
AG	--	AA	AG	AG	AG	AA	AA	AA	--	AG	AA	--
GG	--	AG	AG	AG	AG	--	GG	GG	GG	AG	GG	--
AA	--	AG	AG	AG	AG	AA	AA	AA	AA	AG	AA	--
TT	--	GT	GG	GT	GG	TT	TT	TT	--	GT	TT	--
AG	--	GG	GG	AG	GG	AG	AG	AG	AG	GG	AG	--
TT	--	CT	CC	CT	CC	--	TT	CT	TT	CT	TT	--
GG	--	CG	CC	CG	CC	GG	GG	CG	GG	CG	GG	CG
CT	CT	CT	TT	TT	TT	CT	CT	CT	CT	CT	CT	TT
CT	CT	TT	TT	CT	TT	CT	CT	TT	CT	TT	CT	--
CC	CC	CT	CC	CC	CT	CT	CT	CT	CT	CC	CC	CT
GG	GT	GT	GG	GT	GG	GT	GG	GG	GT	GG	GG	--
GG	--	AA	GG	AG	AG	AG	AG	AG	AA	GG	GG	--
AA	--	GG	AA	AA	AG	--	AG	AG	GG	AA	AA	--
AA	--	AG	AA	AA	AG	AG	AG	AG	AA	AA	AG	AG
GG	--	TT	GG	GT	GT	TT	GT	GT	TT	GG	GT	--
AG	AG	AG	AG	AA	AG	AA	AG	AG	AG	AA	AG	AG
CC	CT	CC	CT	--	CC	CT	CT	CT	CT	CC	CT	--
AG	--	AG	AA	AG	AG	--	AA	AA	AA	AG	AA	AG
GG	--	AG	AA	AG	AG	AA	AG	AG	AA	GG	AA	--
GG	--	AG	AA	AG	AG	AA	AA	AG	AA	GG	AA	--
CG	--	CG	CC	CG	CG	CC	CC	CG	--	GG	CC	CG
CG	--	CG	CC	CG	CG	--	CC	CG	CC	GG	CC	--
AG	--	GG	AA	GG	AG	AG	AA	AG	AA	GG	AA	AG
GG	--	GG	AG	GG	AG	GG	AG	GG	AG	GG	AG	--
CT	--	TT	CC	TT	CT	--	CC	CT	CC	CT	--	--
CC	--	CC	AC	CC	AC	CC	AC	CC	AC	AC	AC	--
CC	CG	CC	CG	--	CG	--	CG	CG	CG	CG	CG	--
CC	AC	CC	AC	CC	AC	CC	AC	AC	AC	AC	AC	CC
AC	--	AA	CC	AA	AC	AC	CC	CC	AC	AC	CC	--
AG	--	AA	AG	AA	AA	AG	AG	AG	AG	AA	AG	--
AG	GG	AA	GG	AA	AG	AG	GG	GG	AG	AG	GG	--
AG	--	GG	AG	GG	GG	AG	AG	AG	AG	GG	AG	--
CT	--	CC	TT	CC	CT	CT	TT	TT	CT	CT	TT	--
CT	--	CT	TT	CC	CT	CT	TT	TT	CT	CT	TT	--
AA	AG	AG	AG	AA	AG	AA	AG	AG	AA	AG	AG	--
CC	CC	CT	CT	CC	CC	CC	CC	CT	CC	CT	CC	--
AA	--	AA	GG	AA	AG	AG	AG	GG	AG	AG	AG	--
AC	--	CC	CC	CC	CC	AC	CC	CC	CC	CC	AC	--
CT	CT	CT	CC	CT	CT	CC	CT	CC	CT	CT	CC	CT
GG	--	AG	AA	GG	AG	AG	AG	AA	AG	AG	AG	AG

AG	--	AG	AA	AG	AG	AA	AG	AA	AG	AG	AA	--
GG	--	AG	AA	GG	AG	AG	AG	AA	AG	AG	AG	--
AA	AC	AC	AC	AA	AC	AC	AC	CC	AC	AC	AC	--
AG	AG	AG	AG	AA	AG	AG	AG	GG	AG	AG	AG	--
GT	--	GT	GT	GG	GT	--	TT	TT	GT	GT	GT	--
AG	AA	AA	AA	AG	AA	AG	AA	AA	AA	AA	AG	--
CT	CT	CT	CT	CC	CT	TT	TT	TT	CT	CT	CT	TT
CT	--	CC	CC	CT	CC	CC	CC	CC	CT	CC	CT	--
AG	AG	AG	AG	AA	AG	GG	GG	GG	--	GG	AG	--
GG	--	CG	CG	CG	CG	GG	GG	GG	CG	GG	CG	--
TT	--	CT	CT	CT	CT	--	TT	TT	CT	TT	CT	--
AG	AG	AA	AG	AG	AG	AA	AA	AA	GG	AA	GG	--
AG	AG	AA	AG	AG	AG	AG	AA	AA	GG	AA	GG	AA
CG	--	CC	CG	CG	CG	CG	CC	CC	GG	CC	GG	--
AG	--	AA	AG	AG	AG	AG	AA	AA	GG	AA	GG	--
GG	--	GG	GG	AG	AA	AG	GG	GG	AA	GG	AG	--
TT	TT	CT	TT	CT	CC	CT	TT	TT	CC	TT	CT	CT
GG	--	AG	GG	AG	AA	AG	GG	GG	AA	GG	AG	--
CC	--	CG	CC	CG	GG	CG	CC	CC	GG	CC	CG	--
CC	--	AC	CC	AC	AA	AC	CC	CC	AA	CC	AC	--
AA	--	AG	AA	AG	GG	AG	AA	AA	GG	AA	AG	--
GG	GG	GT	GG	GT	--	GT	GG	GG	--	GG	GT	GT
GG	--	AG	GG	AG	AA	AG	GG	GG	AA	GG	AG	--
GG	--	AG	GG	AG	AA	AG	GG	GG	AA	GG	AG	AG
AA	--	AG	AA	AG	GG	AG	AA	AA	GG	AA	AG	--
AA	AA	AC	AA	AC	CC	AC	AA	AA	CC	AA	AC	--
AA	--	AG	AA	AG	GG	AG	AG	AA	GG	AA	AG	--
AA	--	AG	AA	AG	GG	AG	AG	AA	GG	AA	AG	AG
TT	--	CT	TT	--	CC	CT	CT	TT	CC	TT	CT	CT
GG	--	CG	GG	CG	CC	CG	CG	GG	CC	GG	CG	--
AA	--	AC	AA	AC	CC	--	AC	AA	CC	AA	AC	AC
TT	--	CT	TT	--	CC	CT	CT	TT	CC	TT	CT	CT
TT	--	GT	TT	GT	GG	--	GT	TT	GG	TT	GT	GT
CC	--	CT	CC	CT	TT	CT	CT	CC	TT	CC	CT	--
CC	CC	CG	CC	CG	GG	CG	CG	CC	CG	CC	CG	--
TT	--	AT	TT	AT	AA	AT	AT	TT	AA	TT	AT	--
CC	--	CT	CC	CT	TT	CT	CT	CC	TT	CC	CT	--
--	--	AG	GG	AG	AA	AG	AG	GG	--	GG	AG	--
AA	--	AG	AA	AG	GG	AG	AG	AA	GG	AG	AG	AG
AA	--	AT	AA	AT	TT	AT	AT	AA	--	AT	AT	--
GG	--	AG	GG	AG	AA	AG	AG	GG	AA	AG	AG	AG
AA	--	AG	AG	AG	AG	AG	AG	AA	GG	AG	AG	--
CC	--	CG	CG	CG	GG	CG	CG	CC	GG	CG	CG	--
AA	--	AG	AG	AG	GG	--	AG	AA	GG	AG	AG	AG
GG	--	CG	CG	CG	CC	CG	CG	GG	CG	CG	CG	--
GG	--	AG	AG	AG	AA	AG	AG	GG	AA	AG	GG	--
AA	--	AC	AC	AC	CC	AC	AC	AA	CC	AC	AC	AC

GG	--	AG	AG	AG	AA	AG	AG	GG	AA	AG	AG	AG
CC	CG	CG	CG	CG	GG	CG	CG	CC	GG	CG	CG	CG
CC	--	CT	CT	CT	TT	CT	CT	CC	TT	CT	CT	--
TT	--	AT	AT	AT	AA	--	AT	TT	--	AT	TT	--
CC	--	CT	CT	CT	TT	--	CT	CC	TT	CT	CC	--
AA	--	AT	AT	AT	TT	AT	AT	AA	TT	AT	AA	AT
TT	--	AT	AT	AT	AA	AT	AT	TT	--	AT	TT	--
GG	GG	CG	CG	CG	CC	CG	CG	GG	CC	CG	GG	--
CC	CC	CT	CT	CT	TT	CT	CT	CC	TT	CT	CC	--
AA	AA	AG	AG	AG	GG	AG	AG	AA	GG	AG	AA	--
AA	--	AC	AC	AC	CC	AC	AC	AA	CC	AC	AA	--
TT	--	GT	GT	GT	GG	GT	GT	TT	GG	GT	TT	--
CC	--	CT	CT	CT	TT	CT	CT	CC	TT	CT	CC	--
--	--	CG	CG	CG	CC	CG	CG	GG	CC	CG	GG	--
TT	--	GT	GT	--	GG	--	GT	TT	GG	GT	TT	--
TT	TT	CT	CT	CT	CC	CT	CT	TT	CC	CT	TT	--
AA	--	AG	AG	AG	--	AG	AG	AA	--	AG	AA	--
AA	--	AC	AC	AC	CC	AC	AC	AA	CC	AC	AA	--
CC	--	CT	CT	CT	TT	CT	CT	CC	TT	CT	CC	--
AA	--	AC	AC	AC	CC	AC	AC	AA	CC	AC	AA	AA
AA	--	AG	AG	AG	GG	AG	AG	AA	--	AG	AA	--
GG	--	GT	GT	GT	TT	GT	GT	GG	TT	GT	GG	--
GG	--	GT	GT	TT	TT	--	GT	GG	--	GT	GG	--
GG	--	AG	AG	AG	AA	AG	AG	GG	AA	AG	GG	--
CC	--	CT	CT	CT	TT	CT	CT	CC	TT	CT	CC	CC
CC	--	CT	CT	CT	TT	CT	CT	CC	TT	CT	CC	--
AA	AG	AG	AG	AG	GG	AA	AG	AA	GG	AG	AA	--
CC	CT	CT	CT	CT	TT	CT	CT	CC	TT	CT	CC	--
TT	CT	CT	CT	CT	CC	CT	CT	TT	CC	CT	TT	--
GG	AG	AG	AG	AG	AA	AG	AG	GG	--	AG	GG	--
CC	--	CT	CT	CT	TT	CT	CT	CC	TT	CT	CC	CC
TT	--	CT	CT	TT	CT	TT	CT	TT	CT	CT	TT	--
CC	CT	CT	CT	--	TT	CT	CT	CT	--	CT	CT	--
AA	--	AG	AG	AG	GG	AG	GG	AG	GG	AG	AG	--
AA	--	CC	AC	AC	CC	CC	CC	AC	CC	AC	AC	AA
CG	CG	CC	CG	CC	CC	CC	CC	CC	--	CG	CC	--
GG	--	GT	GG	GG	TT	--	TT	GT	TT	GG	GT	--
GG	AG	AG	GG	GG	AA	AA	AA	AG	AA	GG	--	GG
TT	--	CT	TT	TT	CC	CC	CC	CC	CC	TT	CT	--
AG	AG	GG	AG	--	GG	GG	GG	GG	GG	AG	GG	--
GG	AG	AG	GG	GG	AA	--	AA	AA	AG	GG	AG	AG
GG	--	CG	GG	GG	CC	--	CC	CC	CG	GG	CG	--
CT	--	TT	CT	CT	TT	--	TT	TT	TT	CT	--	--
TT	--	GT	TT	TT	GG	GG	GG	GG	GT	GT	GT	GT
AG	AG	AG	AG	AG	GG	AG	GG	AG	AG	AG	GG	--
GG	AG	GG	AG	AG	GG	--	AG	AG	AG	AG	GG	--
AC	--	AC	CC	CC	AC	--	AC	CC	CC	CC	AA	--

AT	--	AT	AA	AA	AT	AA	AT	AA	AA	AA	TT	AT
TT	--	AT	AA	--	AT	AA	AT	AT	AA	AA	TT	--
AA	--	AT	TT	TT	AT	--	AT	AT	TT	TT	--	--
TT	--	CT	CT	CC	CT	CC	CT	CT	CC	CC	TT	--
GG	GG	AG	AG	AA	AG	AA	AG	AG	AA	AA	GG	--
AC	--	AC	AC	AC	AC	CC	AC	AC	CC	CC	AA	--
CT	--	CT	CT	--	CT	--	CT	TT	CC	CC	TT	--
AG	--	AG	AG	AG	AG	AA	AG	GG	AA	AA	GG	--
GT	GG	GT	GT	GT	GT	TT	GT	GG	TT	GT	GG	GT
AG	--	AG	AG	AG	AA	AA	AG	GG	--	AG	GG	--
CC	--	CG	CG	--	CG	CG	CC	CC	CG	CG	CC	CG
GG	GG	AG	AG	AG	AG	--	GG	GG	AG	AG	GG	AG
CG	CC	GG	CG	GG	GG	GG	CG	CC	CG	CG	CC	CG
AG	--	GG	AG	GG	GG	GG	AG	AA	AG	GG	AA	AG
AG	--	AA	AG	AA	AA	AA	GG	--	--	AA	GG	AG
AG	AA	GG	AG	GG	GG	GG	AA	AG	AG	GG	AA	AG
GG	GG	GT	GT	GT	GT	GT	GG	GG	GT	GT	GG	GT
AG	--	AA	AG	AA	AA	AA	GG	AG	AG	AA	GG	--
TT	--	GT	GT	GT	GT	GT	TT	TT	GT	GT	TT	GT
GG	--	CC	CC	CC	CC	CC	GG	CG	CG	CC	GG	--
TT	--	CT	CT	--	TT	--	CT	TT	CT	TT	--	--
CG	--	CG	CC	CG	GG	--	CC	CG	--	GG	CG	CG
CG	CG	CC	CG	--	CC	--	CG	CG	--	CC	CC	--
AA	AA	AC	AC	AA	AA	AA	AC	AA	AA	AA	AC	AA
CC	--	CT	CT	--	CC	--	CT	CT	--	CC	CT	--
AA	--	AG	AG	--	AA	AA	AG	AG	AA	AA	AG	--
CC	CC	CT	CT	CC	CC	CC	CT	CT	CC	CC	CT	--
CG	GG	CG	CG	CG	CG	GG	CG	GG	GG	GG	GG	--
CT	--	CC	CT	CC	CC	--	--	CT	CC	CC	CT	--
GG	AA	AG	AG	AG	AG	AA	GG	AG	AA	AA	AG	AG
GT	--	GG	GT	GG	GG	--	GT	GT	GG	GG	GT	GT
GG	--	GT	GT	GT	GT	GT	GG	GT	TT	TT	GT	GT
CG	CG	GG	CG	GG	GG	--	CG	CG	GG	--	CG	CG
CC	--	CG	CG	CG	CG	CG	CC	CG	GG	--	CG	--
AG	AG	GG	AG	GG	GG	GG	AG	AG	GG	GG	AG	--
--	--	CT	CT	CT	CT	--	CC	CT	TT	TT	CT	--
TT	--	GT	GT	GT	GT	GT	TT	GT	GG	GG	GT	GT
AG	AG	GG	AG	GG	GG	GG	AG	AG	GG	GG	AG	AG
GG	--	AG	AG	AG	AG	AG	GG	AG	AA	AA	AG	--
CC	--	CT	CT	CT	CT	--	CC	CT	TT	TT	CT	--
AT	--	TT	AT	TT	TT	TT	AT	AT	TT	TT	AT	--
GT	--	TT	GT	TT	TT	TT	GT	GT	TT	TT	GT	GT
TT	--	CT	CT	CT	CT	CT	TT	CT	--	CC	CT	--
AG	AG	AA	AG	AA	AA	AA	AG	AG	AA	AA	AG	AG
AA	--	AT	AT	AT	AT	AT	AA	AT	TT	TT	AT	--
AG	--	AA	AG	AA	AA	AA	AG	AG	AA	AA	AG	--
CT	CT	TT	CT	TT	TT	--	CT	CT	TT	TT	CT	--

AT	--	TT	AT	TT	TT	TT	AT	AT	TT	TT	--	AT
TT	--	GT	GT	--	GT	GT	TT	GT	GG	GG	GT	--
GG	--	AG	AG	AG	AG	AG	GG	AG	AA	AA	AA	AG
TT	--	CT	CT	CT	CT	CT	TT	CT	CC	CC	CC	CT
TT	--	CT	CT	--	CT	CT	TT	CT	CC	CC	CC	--
TT	--	CT	CT	TT	CT	CT	TT	CT	CC	CC	CC	--
TT	GT	GT	GT	TT	GT	--	TT	GT	GG	GG	GG	GT
GG	CG	CG	CG	GG	CG	--	GG	CG	CC	CC	CC	--
AA	AC	AC	AC	AA	AC	AC	AC	AC	CC	CC	AC	--
GG	AG	AG	AA	GG	AG	GG	AG	AA	AA	AA	AG	--
AG	--	AG	GG	AA	AA	--	AG	GG	GG	GG	--	--
CC	TT	CC	TT	CT	CT	CT	CT	CC	CT	CT	CT	--
CC	--	CC	AA	--	AC	AC	AC	CC	CC	AC	AC	--
GT	--	GT	TT	TT	GT	--	TT	GT	GT	TT	TT	--
CG	--	CG	GG	GG	CG	--	CG	CG	CG	CG	CG	--
AG	--	AA	GG	AG	AA	AG	AG	AA	AA	AA	AG	AG
TT	TT	CT	CT	TT	CT	CT	CT	CT	CT	CT	CT	--
GT	GT	GT	TT	TT	GT	TT	GT	TT	GT	GT	TT	GT
CC	CC	CT	CT	CC	CT	CC	CC	CT	CT	CC	CT	--
AC	AC	AC	AA	AA	AC	AA	AC	AA	AA	AC	AC	AC
AG	AG	GG	AG	AA	GG	AA	AG	AG	AG	AG	GG	--
AG	AG	AG	AA	AA	AG	--	AG	AA	AA	AG	AG	AG
GT	--	GG	GT	GT	GG	GT	GT	GT	--	GT	--	--
CT	--	TT	CT	CT	TT	--	CT	CT	TT	CT	TT	--
AC	AA	CC	AC	AC	CC	AA	AC	AC	CC	AC	CC	CC
AT	--	AA	AT	--	AA	TT	AT	AT	AA	AT	AT	--
GG	GT	GG	GT	GT	GG	GT	GT	GT	GG	GG	GT	--
AG	AG	AA	AA	AA	AA	AG	AG	AA	AA	AG	AA	--
CG	--	GG	CG	CG	GG	CC	CC	CG	GG	CG	CG	--
AG	--	GG	AG	AG	GG	--	AA	AG	GG	AG	AG	--
CC	--	CG	CG	CG	CC	CG	CG	CG	GG	CG	CC	--
AA	--	AG	AG	AG	AA	AG	AG	AG	GG	AG	AA	--
AA	AG	AG	AG	AG	AA	--	AA	AA	AG	AA	AA	--
AA	--	AT	AA	AA	AA	--	AA	AT	AT	AT	AA	--
AG	AG	AG	AG	AG	AA	AG	AA	AA	AG	AA	AA	--
AA	--	AA	AA	AA	AG	AA	AG	AG	AA	AG	AG	--
AC	AC	CC	AC	AC	AA	AC	AA	AC	CC	AC	AC	AC
CC	--	AC	CC	--	CC	CC	CC	AC	AC	AC	AC	AC
AC	--	AA	AC	AC	CC	--	CC	AC	AA	AC	--	--
GT	--	TT	GT	GT	GG	GT	GG	GT	GT	GT	GT	--
TT	--	TT	AT	--	AA	TT	AA	AT	AT	AT	--	--
AG	AA	AG	AA	AG	AA	AG	AA	AG	AA	AG	AG	AG
AC	--	AC	CC	CC	CC	AC	CC	AC	CC	AC	AC	AC
TT	AT	TT	AT	TT	AT	TT	AA	AT	AT	AT	AA	AT
TT	TT	TT	CT	TT	CT	TT	CT	CT	CT	CT	CC	--
AA	--	AA	AG	AA	AG	AG	AG	AG	GG	AG	GG	--
TT	--	TT	CT	TT	CT	CT	CT	CT	--	CT	CC	--

CC	--	CC	CT	--	CT	CT	CT	CT	TT	CT	--	--
AA	--	AA	AG	AA	AG	--	AG	AG	GG	GG	GG	--
TT	TT	TT	CT	TT	CT	CT	CT	CT	CC	CC	CC	--
CC	--	CC	CT	CC	CT	--	CT	CT	TT	TT	TT	--
TT	--	TT	CT	TT	CT	CT	CT	CT	CC	CC	CC	TT
AA	--	AA	AG	AA	AG	--	AG	AG	GG	GG	GG	--
CC	--	CC	CT	--	CT	CT	CT	CT	--	TT	TT	--
AG	--	AA	AG	--	AG	AG	AG	AG	GG	GG	GG	--
CG	--	GG	CG	GG	CG	CG	CG	CG	CC	CC	CC	--
AC	--	CC	AC	CC	AC	AC	AC	AC	AA	AA	AA	--
CT	--	CT	CT	TT	CT	CT	CT	CT	CC	CC	CC	--
CT	--	CT	CT	CT	CT	CT	CT	CT	--	CC	CC	--
AT	--	AT	AT	AT	AT	AT	AT	AT	TT	TT	TT	--
CT	--	CT	CT	--	CT	--	CT	CT	CC	CC	CC	--
AC	--	AC	AC	--	AC	AC	AC	AC	AA	AA	AA	--
GT	GG	GT	GT	GT	GT	GT	GT	GT	TT	TT	TT	--
CT	--	CT	CT	CT	CT	--	CT	CT	TT	TT	TT	--
CG	CC	CG	CG	CG	CG	CG	CG	CG	GG	GG	GG	CC
CG	--	CG	CG	CG	CG	--	CG	CG	--	GG	GG	--
AC	--	AC	AC	AC	AC	AC	AC	AC	CC	CC	CC	--
CG	--	CG	CG	CG	CG	CG	CG	CG	--	GG	GG	--
AT	--	AT	AA	AT	AA	AT	AT	TT	AA	AT	--	--
AG	--	AG	AA	AG	AA	AA	AG	AG	AG	AG	AG	AA
AG	--	AG	AA	AG	AA	--	AG	AG	AG	AG	--	--
AG	--	AG	GG	AG	GG	GG	AG	AG	AG	AG	AG	--
AG	AG	AG	AA	AG	AA	AA	AA	AA	AG	AG	AG	--
AC	AC	AC	AA	AC	AA	AA	AA	AA	AC	AC	AC	--
AG	--	AG	AA	AG	AA	AA	AA	AA	AG	AG	AG	--
AG	AG	AG	AA	AG	AA	AA	AA	AA	AG	AG	AG	AA
GT	--	GT	GG	GT	GG	--	GG	GG	GT	GT	GT	--
GT	GT	GT	TT	GT	TT	--	TT	TT	GT	GT	GT	--
AC	AC	AC	CC	AC	CC	CC	CC	CC	AC	AC	AC	--
CT	--	CT	TT	--	TT	TT	TT	--	CT	CT	CT	--
CT	--	CT	TT	CT	TT	TT	TT	TT	CT	CT	CT	--
GT	--	GT	GG	GT	--	GG	GG	GG	--	GT	GT	--
AT	--	AT	AA	AT	AA	--	AA	AA	AT	AT	AT	--
CG	--	CG	GG	CG	GG	GG	GG	GG	CG	CG	CG	--
AC	--	AC	AA	--	AA	--	AA	AA	AC	AC	AC	--
CT	CT	CT	CC	CT	CC	CC	CC	CC	CT	CT	CT	--
CG	--	CG	GG	CG	GG	--	GG	GG	CG	CG	CG	--
CT	CT	CT	CC	CT	CC	CC	CC	CC	CT	CT	CT	--
AT	AT	AT	AA	AT	--	AA	AA	AA	AT	AT	AT	--
CT	CT	CT	TT	CT	TT	--	TT	TT	CT	CT	CT	--
AG	AG	AG	GG	AG	GG	GG	GG	GG	AG	AG	AG	--
AT	AT	AT	AA	AT	AA	--	AA	--	AT	AT	AT	--
AT	--	AT	AA	AT	AA	AA	AA	AA	AT	AT	AT	--
AC	AC	AC	AA	AC	AA	AA	AA	AA	AC	AC	AC	--

GT	GT	GT	TT	GT	TT	TT	TT	GT	GT	GT	GT	--
AG	--	AG	GG	AG	GG	GG	GG	AG	AG	AG	AG	--
GT	--	GT	GG	GT	--	--	GG	GT	GT	GT	GT	--
AG	--	AG	AA	AG	AA	AA	AA	AG	AG	AG	AG	--
CT	--	CT	TT	CT	TT	--	TT	CT	CT	CT	CT	--
AG	--	AG	AA	AG	AA	AA	AA	AG	AG	AG	AG	AA
CT	--	CT	TT	CT	TT	TT	TT	CT	CT	TT	CT	--
AC	AC	AC	CC	AC	CC	CC	CC	AC	AC	CC	AC	--
AG	--	AG	AA	AG	AA	AG	AA	AG	AG	AA	AG	--
CT	CT	CT	TT	CT	CT	CT	TT	CT	CT	TT	CT	--
AG	AG	AG	GG	AG	AG	AG	GG	AG	AG	GG	AG	--
TT	CT	CT	CC	--	CT	CT	CC	CT	CT	CC	CT	--
GT	GT	GG	GG	GG	GT	GG	GG	GT	GT	GG	GT	--
AG	--	AG	GG	AG	GG	AG	GG	GG	GG	GG	GG	AG
AA	--	TT	AT	AT	AA	AT	AT	AT	AT	TT	AT	AT
AA	--	TT	AT	AT	AA	AT	AT	AT	AT	AT	AT	--
AA	--	CC	AC	AA	AA	AC	AC	AC	AC	AC	AC	--
TT	--	GG	GT	TT	TT	--	GT	GT	GT	GT	GT	GT
CT	--	TT	TT	CT	TT	TT	TT	TT	--	CT	CT	--
AG	--	GG	GG	AA	AG	AG	AG	GG	AG	AG	AG	--
CG	--	CG	CG	--	GG	GG	GG	CG	GG	CG	CG	--
CT	--	CT	CT	TT	TT	TT	TT	CT	TT	CT	CT	--
GG	GG	GT	GT	GG	GT	GT	GT	GT	GT	GG	GG	--
AG	--	AG	GG	AA	AA	AG	AG	AG	--	AG	AG	--
AC	--	AA	AC	AA	AC	AA	AA	AA	AA	AC	AC	--
CG	--	GG	CG	GG	CG	--	GG	GG	GG	CG	CG	--
GT	TT	TT	GT	--	GT	--	GT	TT	--	GT	GT	--
CT	--	CT	TT	CC	CT	CT	TT	CC	CC	CT	CT	--
AA	--	GG	AG	AA	AA	AG	AG	AG	AG	--	AA	--
CC	--	CC	CT	CT	CT	CT	CT	CC	CC	CT	CT	--
CG	--	CG	CC	--	CC	CC	CC	CG	CC	CC	CC	--
AC	AC	AA	AA	AA	AC	AA	AA	AC	AC	AA	AC	--
GG	--	AG	AA	AG	AG	AA	AA	GG	AG	AA	GG	AG
--	--	CT	CT	CT	TT	CT	CT	TT	TT	CT	--	--
GG	CG	GG	CG	CG	CG	CG	CG	GG	CG	CG	CG	--
AA	AG	AG	GG	GG	AG	GG	GG	AA	AG	GG	AG	AG
CG	--	CG	GG	GG	GG	GG	GG	CG	GG	GG	--	--
CC	--	CG	GG	CG	CG	GG	GG	CC	CG	GG	CG	--
CC	--	CT	TT	CT	CT	TT	TT	CC	CT	TT	CT	CT
GG	--	AG	AA	AG	AG	AA	AA	--	AG	AA	AG	AG
AG	--	GG	GG	GG	AG	GG	GG	AG	AG	GG	AG	--
CC	CT	CT	TT	CT	CT	TT	TT	CC	CT	TT	CC	--
GG	GG	AG	AG	AG	GG	AG	AG	GG	GG	AG	GG	AG
GG	AG	AG	AA	AG	AG	AA	AA	GG	AG	AA	GG	--
CC	--	AA	AA	AC	AC	AA	AA	CC	AC	AA	CC	AC
TT	--	AT	AT	TT	AT	--	AT	TT	AT	AT	TT	--
--	--	GG	GG	AG	AG	AG	GG	AA	AG	GG	AA	--

AA	--	CC	CC	AC	--	CC	CC	AA	AC	CC	AA	--
AA	--	GG	GG	AG	AG	GG	GG	AA	AG	GG	AA	AG
AA	--	AG	AG	AG	AA	AG	AG	AA	AA	AG	AA	AG
CC	--	CT	CT	CC	CT	CT	CT	CC	CT	CT	CC	--
CC	--	CG	CG	CC	CG	CG	CG	CC	CG	CG	CC	--
TT	CT	CT	CT	--	CT	--	CT	CT	CT	CT	TT	--
CG	--	GG	GG	CG	CG	CG	GG	GG	--	GG	CG	--
CC	--	CT	CT	CC	CC	CC	CT	CT	CC	CT	CC	--
GT	--	GG	GG	GT	GT	GT	GG	GG	GT	GG	GT	GT

2-81	2-82	2-84	3-4	3-5	3-8	3-10	3-14	3-16	3-17	3-18	3-23	3-25	3-26
AC	AC	AC	AC	CC	AC	AC	CC	AC	AC	AC	AC	AC	AC
AG	AG	AG	AG	GG	AG	AG	GG	AG	AG	AG	AG	AG	AG
CT	CT	CT	CT	CT	CT	CT	CC	CC	CT	CT	CT	CT	CT
AG	AG	AG	GG	--	--	--	AG	AG	AG	AG	AG	--	AG
AG	AG	AG	AG	AG	AG	AG	GG	GG	AG	AG	AG	GG	AG
--	CG	CG	GG	CG	CG	CG	CG	GG	CG	CG	CG	GG	CG
CT	TT	CT	CT	CT	CT	TT	CT	CC	CT	CT	CT	CT	CT
GT	TT	GT	GT	GT	GG	TT	GT	GG	GT	GG	GT	GT	GT
GT	TT	GT	GT	GT	GG	TT	GT	GG	GT	GG	GT	GT	GT
GT	TT	--	GT	GT	GG	TT	GT	GG	GT	GG	GT	GT	GT
AG	AA	--	AG	AG	GG	--	AG	GG	GG	GG	GG	AG	AG
CT	CT	CT	CC	CT	CC	--	CT	CC	CC	CC	CC	--	CT
AC	AA	--	AC	AC	CC	AA	AC	CC	CC	CC	CC	AC	CC
AG	AG	AG	AA	AG	AA	AG	AG	AA	AA	AA	AA	AA	AA
CC	CT	--	CT	CC	CC	CT	CC	CC	CC	CC	CC	CT	CC
AG	GG	--	AG	AG	AG	AG	AG	AA	AA	AA	AG	AG	AA
TT	AT	AT	TT	AT	AT	TT	AT	TT	TT	TT	TT	TT	TT
CC	CT	--	CT	CC	CT	CT	CC	--	CC	CC	CT	--	CC
TT	TT	TT	TT	TT	GT	TT	GT	GT	TT	TT	TT	GT	TT
CC	CC	--	CC	CC	CG	CC	CG	CG	CC	CC	CC	CG	CC
AT	AT	--	AT	--	--	--	AT	AT	TT	TT	AT	AA	TT
CT	CT	CT	CT	TT	CC	CC	CT	CT	TT	TT	CT	CC	TT
TT	AT	--	AT	AA	TT	--	AT	AT	AA	AA	AT	--	AA
AT	AT	AT	AT	AA	TT	TT	AT	AT	AA	AA	AT	TT	AA
AG	AG	AG	AG	AA	GG	--	AG	AG	AA	AA	AG	GG	AA
TT	TT	TT	TT	TT	GT	GT	GT	GT	TT	TT	TT	GT	TT
GT	GT	GT	GT	TT	GG	GG	GT	GT	TT	TT	GT	GG	TT
AT	AT	AT	AT	TT	AA	AA	AT	AT	TT	TT	AT	AA	TT
CT	CT	--	CT	TT	CC	CC	CT	CT	TT	TT	CT	--	TT
CG	CG	--	CG	CC	GG	GG	CG	CG	CC	CC	CG	GG	CC
CT	CT	CT	CT	CC	TT	TT	CT	CT	CC	CC	CT	TT	CC
AC	AC	--	--	--	--	CC	AC	AC	AA	AA	AC	CC	AA
AG	AG	AG	AG	AA	GG	--	AG	AG	AA	AA	AG	GG	AA
GT	GT	GT	GT	GG	TT	--	GT	GT	GG	GG	GT	TT	GG
AG	AG	AG	AG	AA	GG	GG	AG	AG	AA	AA	AG	GG	AA
GT	GT	--	GT	TT	GG	GG	GT	GT	TT	TT	GT	GG	TT
AG	AG	AG	AG	GG	AA	AA	AG	AG	GG	GG	AG	AA	GG
CT	CT	CT	CT	CC	TT	--	CT	CT	CC	CC	CT	TT	CC
TT	AT	AT	AT	AA	TT	TT	AT	AT	AA	AA	AT	TT	AA
GG	AG	AG	AG	AA	GG	--	AG	AG	AA	AA	AG	GG	AA
CC	AC	--	AC	AA	CC	CC	AC	AC	AA	AA	AC	CC	AA
GG	AG	GG	AG	AA	GG	GG	AG	AG	AA	AA	AG	GG	AA

CC	CT	CT	CT	TT	CC	--	CT	CT	TT	TT	CT	--	TT
GG	CG	GG	CG	--	GG	GG	CG	GG	CC	--	CG	GG	CC
CC	CT	CT	CT	TT	CC	CT	CT	CT	TT	TT	CT	CC	TT
TT	CT	CT	CT	CC	CT	CT	CT	CT	CC	CC	CT	TT	CC
CC	AC	AC	AC	AC	AC	AC	CC	AC	AC	AA	AC	CC	AA
TT	TT	--	CT	CT	CT	CT	CT	CT	CT	CC	CT	TT	CC
CT	CC	CT	CT	CT	CT	--	CT	TT	CT	TT	CT	CC	TT
AG	AG	--	AA	AG	GG	AA	GG	GG	GG	AA	AG	AG	AG
GT	TT	GT	TT	GT	GG	TT	GT	GG	GT	TT	TT	GT	GT
AA	AA	--	AA	AG	GG	AG	AG	GG	AG	AA	AA	AG	GG
CC	CC	CC	CC	CT	TT	CT	CT	TT	CT	CC	CC	TT	TT
GG	CG	--	CG	CG	CC	CG	CG	CC	CG	GG	GG	CG	CC
GG	AG	GG	AG	AG	AA	AG	AG	AG	AG	GG	GG	AG	AA
AA	AC	--	AA	AC	CC	AC	AC	AC	AC	AA	AA	AC	CC
GG	GG	GG	GG	AG	AG	--	AG	AG	AG	GG	GG	AG	AG
CC	CT	--	CC	CT	TT	CT	CT	CT	CT	CC	CC	CT	TT
TT	CT	TT	TT	CT	CC	CT	CT	CT	CT	TT	TT	CT	CC
CT	CT	--	CC	CT	CT	CT	CT	CT	CT	CC	CC	CT	TT
AG	AG	GG	GG	AG	AG	AG	AG	AG	AG	GG	GG	AG	AA
AC	AC	CC	CC	AC	AC	AC	AC	AC	AA	AC	CC	AC	AA
AG	GG	GG	GG	AG	AG	GG	AG	AG	AA	AG	GG	AG	AA
CG	CC	--	CC	CG	CG	CC	CG	CG	GG	CG	CC	CG	GG
CG	GG	--	GG	CG	CG	GG	CG	CG	CC	CG	GG	CG	CC
AG	GG	--	GG	AG	AG	GG	AA	AG	AA	AG	GG	AG	AA
AC	AA	--	AA	AC	AC	--	CC	AC	CC	AC	AA	AC	CC
TT	CC	CC	CC	--	CT	CC	TT	CT	TT	CT	CC	CT	TT
CC	TT	--	TT	CT	CC	--	CC	--	CC	CT	TT	CC	CT
TT	AA	--	AA	AA	TT	AA	AT	TT	TT	TT	AA	TT	AT
GG	AG	--	AG	GG	GG	AG	AG	GG	GG	GG	AG	GG	GG
TT	AT	--	AT	--	TT	AT	TT	TT	AT	AT	AT	TT	AT
GG	GG	--	GT	TT	GG	--	GT	TT	GG	GT	TT	GT	TT
AG	AG	--	GG	GG	AG	--	AG	GG	AG	GG	GG	GG	GG
CT	CT	TT	TT	TT	CT	TT	CT	TT	CT	TT	TT	TT	TT
AG	AG	--	GG	--	AG	--	AG	GG	AG	GG	GG	--	GG
TT	TT	GG	GT	GT	TT	GG	GT	TT	TT	GT	GG	GT	GG
GT	GT	--	GT	--	GT	--	GT	GT	GT	GG	GG	GG	GG
CC	CC	TT	CT	--	CC	CT	CT	CC	CC	CT	TT	CT	TT
CC	CC	--	CT	CC	CC	--	CT	CC	CC	CT	TT	CT	TT
GG	GG	AA	AG	GG	GG	AG	AG	GG	AG	AG	AG	AG	AA
CC	CC	--	CG	CC	CC	--	CG	CC	CG	GG	CG	CG	GG
GG	GG	AA	AG	GG	GG	AG	AG	GG	AG	AG	AG	AA	AA
TT	CT	--	CT	TT	TT	CT	CT	TT	CT	CT	CT	CC	CC
TT	CT	CC	CT	TT	TT	--	CT	TT	CT	CT	CT	CC	CC
CG	CC	CC	CG	CG	CG	CG	CG	CG	CG	CG	CG	CC	CC
AT	TT	--	AT	AT	--	AT	AT	AT	AT	AT	AT	TT	TT
GT	GT	GG	GG	GG	GT	GT	GG	GT	GG	GG	GT	GG	GG
CT	CT	CC	CC	CC	CT	CT	CC	CT	CC	CC	CT	CC	CC

--	CT	CT	CC	CT	TT	TT	CT	TT	CT	CC	TT	CT	CC
GT	GT	--	GG	GG	GT	GG	GG	GT	GG	GG	GT	GG	GG
TT	CT	CT	CC	CC	CT	TT	CT	TT	CC	CC	TT	CT	CC
GT	GT	TT	TT	TT	GT	GT	TT	GT	TT	TT	GT	TT	TT
CG	CG	--	GG	--	CG	CG	GG	CG	GG	GG	CG	GG	GG
AG	AG	--	GG	AA	AG	AG	AG	AG	GG	GG	AG	AG	AG
AG	AG	AA	AA	GG	GG	AG	AG	AG	AA	AA	AG	AG	AG
GG	AG	AA	AG	GG	GG	AG	AG	AG	AA	AA	AG	AG	AG
CC	CT	--	CT	CC	CT	CT	CT	CT	TT	CT	CT	CT	CT
AA	AG	GG	AG	AA	AG	GG	AG	AG	GG	AG	GG	AG	AG
--	GT	--	GT	--	GT	--	GT	GT	GG	GT	GG	GG	GT
GG	GG	AG	GG	AG	AG	AG	GG	AG	AG	GG	AG	AG	AG
CC	--	--	CT	CT	CT	TT	CT	CT	TT	CT	TT	TT	TT
AC	CC	--	CC	AC	AC	CC	CC	AC	CC	CC	CC	CC	CC
CC	CT	--	CT	CC	CC	CT	CT	CC	CT	CT	CT	CT	CT
GT	GT	GT	GT	GT	GT	--	GT	GT	GG	GT	GG	--	GG
CT	CT	CT	CT	CC	CC	CC	CT	CC	CC	CT	CC	CC	CC
CG	GG	--	GG	CG	CG	GG	GG	CG	GG	GG	GG	GG	GG
AC	CC	--	CC	AC	AC	CC	CC	AC	CC	CC	CC	CC	CC
CT	CT	CT	CT	--	TT	--	CT	TT	TT	CT	TT	TT	TT
AG	AG	--	AG	AA	AA	AA	AG	AA	AA	AG	AA	AA	AA
GG	AG	AG	AG	AG	AG	AA	AG	AG	AA	AG	AA	AA	AA
CC	CT	CT	CT	CC	CC	CT	CT	CC	CT	CT	CT	CT	CT
AT	AA	AA	AA	AT	AT	AA	AA	AT	AA	AA	AA	--	AA
CT	CC	--	CC	CT	CT	--	CC	CT	CC	CC	CC	CC	CC
AG	AA	--	AA	AG	AG	AA	AA	AG	AA	AA	AA	AA	AA
CT	CT	CT	CT	CC	CC	--	CT	CC	CC	CT	CC	CC	CC
AA	AT	--	AT	AT	AT	--	AT	AT	TT	AT	TT	TT	TT
AA	AT	AT	AT	AT	AA	TT	AT	AT	TT	AT	TT	TT	TT
CC	CT	CT	CT	CT	CC	TT	CT	CT	TT	CT	TT	TT	TT
CT	CC	--	CC	CT	CT	CC	CC	CT	CC	CC	CC	CC	CC
GT	GT	GT	GT	--	GT	GG	GT	GG	GG	GT	GG	GG	GG
CT	CT	--	CT	CC	CT	CC	CT	CC	CC	CT	CC	CC	CC
AA	AA	AA	AA	AG	AA	AG	AA	AG	AG	AA	AG	AG	AG
AA	AG	AG	AG	AG	--	GG	AG	GG	GG	AG	GG	GG	GG
AG	AG	AG	AG	AA	AG	AA	AG	AA	AA	AG	AA	AA	AA
TT	CT	--	CT	CT	TT	CC	CT	CT	CC	CT	CC	CC	CC
AC	AC	AC	AC	CC	AC	CC	AC	CC	CC	AC	CC	CC	CC
AA	AT	--	AT	AT	AA	--	AT	AT	TT	AT	TT	TT	TT
AG	AA	--	AA	AG	AG	AA	AA	AG	AA	AA	AA	AA	AA
AC	AC	AC	AC	AA	AC	AA	AC	AA	AA	AC	AA	AA	AA
AT	AT	AT	AT	TT	AT	TT	AT	TT	TT	AT	TT	TT	TT
AC	AA	AA	AA	--	AC	AC	AA	CC	AC	AA	AC	AC	AC
AC	CC	--	CC	AC	AC	CC	CC	AC	CC	CC	CC	CC	CC
GG	GG	GG	GG	AG	GG	AG	GG	AG	AG	GG	AG	AG	--
CC	AC	AC	AC	CC	CC	AC	AC	CC	AC	AC	AC	AC	AC
AG	AG	AG	AG	AA	AG	AA	AG	AA	AA	AG	AA	AA	AA

CG	CC	--	CC	CG	CG	CC	CC	CG	CC	CC	CC	CC	CC
CC	CT	CT	CT	CT	CC	TT	CT	CT	TT	CT	TT	TT	TT
TT	CT	--	CT	CT	TT	CC	CT	CT	CC	CT	CC	CC	CC
CT	CT	--	CT	CC	CT	CC	CT	CC	CC	CT	CC	CC	CC
AG	AG	--	AG	--	AG	AA	AG	AA	AA	AG	AA	AA	AA
TT	CT	CT	CT	--	TT	--	CT	CT	CC	CT	CC	CC	CC
CT	CT	CT	CT	TT	CT	TT	CT	TT	TT	CT	TT	TT	TT
TT	AT	AT	AT	AT	TT	--	AA	AT	AA	AT	AA	AA	AA
AG	GG	GG	GG	AG	AG	GG	GG	AG	GG	GG	GG	GG	GG
AC	AC	AC	AC	AA	AC	AC	AA	AA	AA	AC	AA	AA	AA
CT	CT	--	CT	--	CT	CC	CC	CC	CC	CT	CC	--	CC
GT	TT	TT	TT	GT	GT	TT	TT	GT	TT	TT	TT	TT	TT
CT	CT	--	CT	TT	CT	TT	TT	TT	TT	CT	TT	CT	TT
CC	CC	CC	CC	AC	CC	AC	AC	AC	AC	CC	AC	CC	AC
AG	AG	AG	AG	AA	AG	--	AA	AA	AA	AG	AA	AG	AA
GT	GT	GT	GT	--	GT	--	GG	GG	GG	GT	GG	GT	GG
TT	TT	--	TT	AT	TT	AT	AT	AT	AT	TT	AT	TT	AT
AG	AG	AG	AG	AA	AG	AA	AA	AA	AA	AG	AA	AG	AA
CT	CC	--	CC	CC	CT	CC	CC	CT	CC	CC	CC	CC	CC
CT	CT	--	--	CC	CT	--	CC	--	CC	CT	CC	CT	CC
AC	AC	AC	AC	AA	AC	--	AA	AA	AA	AC	AA	AC	AA
CT	CT	CT	CT	CC	CT	CC	CC	CC	CC	CT	CC	CT	CC
TT	AT	--	AT	AT	TT	AA	AA	AT	AA	AT	AA	AT	AA
AA	AG	AG	AG	AG	AA	GG	GG	AG	GG	AG	GG	AG	GG
CG	GG	--	GG	CG	CG	GG	GG	CG	GG	GG	GG	--	GG
CT	CT	--	CT	TT	CT	TT	TT	TT	TT	CT	TT	CT	TT
AG	AG	--	GG	--	AG	--	GG	AG	GG	AG	GG	AG	GG
AG	AG	AG	AA	AA	AG	AG	AA	AG	AA	AG	AA	AG	AA
AG	AA	AA	AA	AG	AG	AA	AA	AG	AA	AA	AA	AA	AA
AG	GG	--	GG	AG	AG	GG	GG	GG	GG	GG	GG	GG	GG
CT	CT	CT	TT	TT	TT	CT	TT	CT	CT	CT	TT	CT	TT
TT	GT	GT	GG	--	GT	--	GG	GT	GT	GT	GG	GT	GG
GT	TT	--	TT	GT	GT	--	TT	TT	TT	TT	TT	TT	TT
AA	AG	--	GG	AG	AG	--	GG	AG	AG	AG	GG	AG	GG
CT	CT	CT	CC	CC	CC	CT	CC	CT	CT	CT	CT	CT	CC
GT	GT	GT	TT	TT	TT	GT	TT	TT	GT	GT	GT	GT	GT
GG	--	AG	AG	--	GG	AG	AG	GG	AG	AG	AG	--	AG
AA	GG	GG	GG	AG	AA	AA	AA	AG	AG	AA	GG	AA	AG
CT	CT	--	CC	CT	CT	CT	CT	CT	CT	--	CC	CT	CC
AA	AT	AT	AT	AT	AA	AA	AA	AT	AT	AA	AT	AA	AA
CC	CG	GG	GG	CG	CC	--	CC	CG	CG	CC	GG	CC	CG
AA	AA	AC	AC	AA	AA	AA	AA	AA	AA	AA	AC	AA	AA
AA	AG	GG	GG	AG	AA	AA	AA	AA	AG	AA	AG	AA	AA
AG	GG	GG	GG	GG	AG	AG	AG	AG	GG	AG	AG	GG	AG
CT	CC	CC	CC	CC	CT	CT	CT	CT	CC	CT	CT	CC	CT
AG	AG	AA	AA	AG	AG	AG	GG	GG	AG	GG	AG	AG	GG
CT	CC	CT	CC	CC	CT	CT	CC	CC	CC	CT	CT	CC	CC

GG	AG	AG	AG	AG	GG	GG	GG	GG	AG	GG	GG	AG	GG
GG	AA	AG	AA	AG	GG	GG	AG	AG	AA	GG	GG	AA	GG
CT	CT	TT	CT	TT	CT	CT	CC	CC	CT	CT	CT	CT	CT
AT	AT	--	AT	TT	AT	AT	AA	AT	AT	AT	AT	AT	AT
AA	AC	AC	AA	CC	CC	AC	AA	AC	AC	AC	AC	CC	AC
TT	CT	CT	TT	CC	CC	--	CT	CT	TT	CT	CT	--	CT
GG	AG	AG	GG	AA	AA	GG	AG	AG	GG	AG	GG	AA	AG
CC	CT	--	CC	TT	CT	CT	CT	CT	CC	CT	CC	TT	CT
TT	TT	CT	TT	CT	TT	--	CT	CT	TT	CT	TT	CT	CT
AA	AG	AG	AA	AG	AG	--	GG	AG	AA	AG	AA	GG	AG
GG	AG	--	AG	--	AG	AG	AG	AA	AG	AG	GG	AA	AA
TT	CT	--	CT	CC	CT	CT	CT	CC	CT	CT	TT	CC	CC
AG	AG	--	AG	--	AG	--	AG	GG	AG	GG	AG	--	GG
TT	CT	--	CT	CC	TT	--	CT	CC	CT	CT	CT	CC	CC
CG	GG	--	GG	GG	CG	CG	GG	GG	GG	CG	CG	GG	GG
TT	CT	--	CT	CT	TT	CT	CT	CC	CT	CT	CT	--	CC
CT	CT	--	CC	CT	CT	--	CT	CC	CT	CC	CC	CT	CC
AG	AG	--	GG	AG	AA	AG	AA	GG	AG	AG	AG	AG	AG
CT	CT	--	CC	CT	TT	--	TT	CC	CT	CT	CT	CT	CT
AG	AG	AA	AA	AG	GG	AG	GG	AA	AG	AG	AG	AG	AG
CT	CT	CT	CC	CT	TT	TT	TT	CT	CT	CT	CT	CT	CT
AG	AG	AG	GG	AG	AG	AA	AA	AG	GG	AG	AG	AG	AG
GG	AG	AG	GG	AG	GG	AG	AG	AG	GG	GG	GG	AG	GG
GT	GT	--	TT	GT	GT	--	GT	GT	TT	GG	TT	GT	GT
CC	CG	CG	CC	CC	CC	CG	CC	CG	CC	CG	CC	CG	CC
CC	CC	CG	CG	--	CC	CC	CC	CG	CC	CC	CG	CC	CC
CT	CT	--	CC	CC	CT	TT	CT	CC	CT	TT	CC	TT	CT
CT	CT	--	TT	TT	CT	CC	CT	TT	CT	CC	TT	CC	CT
TT	AT	--	AA	AA	AT	TT	AT	AA	AT	TT	AA	TT	AT
AA	AG	GG	GG	AG	AG	--	AG	GG	AG	AA	GG	AA	AG
TT	CT	--	CC	CT	TT	--	TT	CC	CT	TT	CC	TT	CT
CT	CT	--	TT	CT	CT	TT	CT	TT	TT	CT	TT	CT	TT
CC	AC	AC	AA	AC	CC	CC	CC	AA	AC	CC	AA	CC	AC
TT	TT	GT	GT	GG	GT	GT	GT	GT	GT	GT	TT	GT	GT
AA	AA	--	AG	--	AG	--	AG	AG	AG	AG	AA	AG	AG
TT	TT	CT	CT	--	CT	--	CT	CT	CT	CT	TT	CT	CT
AA	AA	AT	AT	--	--	AT	AT	AT	AT	AT	AA	--	AT
CC	CC	AC	AC	AA	AC	AC	AC	AC	AC	AC	CC	AC	AC
CG	CG	CC	CG	CC	CC	--	CG	CC	CG	CG	CG	CC	CG
AG	AG	AA	AG	AA	AA	AA	AG	AA	AG	AG	AG	AA	AG
CC	CC	CT	CT	TT	CT	CT	CT	CT	CT	CT	CC	CT	CT
CC	CC	--	CT	TT	CT	CT	CT	CT	CT	CT	CC	CT	CT
AA	AA	AG	AG	GG	AG	AG	AG	AG	AG	AG	AA	AG	AG
CT	CT	--	CC	CC	CT	CT	CC	CT	CC	CC	CT	CT	CC
--	AA	--	AG	GG	AG	AG	AG	AG	AG	AG	AA	--	AG
GG	GG	AG	AG	AG	AG	AG	AG	AG	AG	AG	GG	AG	AG
GG	GG	--	GT	TT	GT	GT	GT	GT	GT	GT	GG	GT	GT

TT	TT	CT	CT	CC	CT	CT	CT	CT	CT	CT	TT	--	CT
GG	GG	CG	CG	CC	CG	CG	CG	CG	CG	CG	GG	CG	CG
TT	TT	GT	GT	GG	GT	GT	GT	GT	GT	GT	TT	GT	GT
AA	AA	--	AG	GG	AG	AG	AG	AG	AG	AG	AA	AG	AG
CC	CC	CT	CT	TT	CT	CT	CT	CT	CT	CT	CC	CT	CT
GT	GT	GT	TT	TT	GT	GT	TT	GT	TT	TT	GT	GT	TT
GG	GG	CG	CG	CC	CG	GG	CG	CG	CG	CG	GG	CG	CG
AA	AA	--	AG	GG	AG	AA	AG	AG	AG	AG	AA	--	AG
AG	AG	--	GG	GG	AG	--	GG	AG	GG	GG	AG	AG	GG
AA	AA	AC	AC	CC	AC	AC	AC	AC	AC	AC	AA	AC	AC
CG	CG	CG	CC	CC	CG	CG	CC	CG	CC	CC	CG	CG	CC
GG	GG	AG	AG	--	AG	AG	AG	AG	AG	AG	GG	AG	AG
AA	AA	--	AA	CC	AC	AC	AC	AC	AC	AC	AA	AC	AC
TT	TT	GT	TT	GG	--	GT	GT	GT	GT	GT	TT	--	GT
CC	CC	AC	CC	--	AC	AC	AC	AC	AC	AC	CC	AC	AC
CT	CT	CT	CT	CC	--	CT	CC	CT	CC	CC	CT	CT	CC
TT	TT	GT	TT	GG	GG	GT	GT	GT	GT	GT	TT	GT	GT
AG	AG	GG	AG	GG	GG	GG	AG	GG	AG	AG	AG	GG	AG
CC	CC	--	CC	--	TT	--	CT	--	CT	CT	CC	CT	CT
CT	CT	--	CT	CC	CC	CT	CC	CT	CC	CC	CT	CC	CC
AA	AA	--	AA	TT	TT	AT	AT	AT	AT	AT	AA	TT	AT
CC	CC	--	CC	GG	GG	CG	CG	CG	CG	CG	CC	GG	CG
GG	GG	GT	GG	GT	TT	GT	GT	GT	GT	GT	GG	TT	GT
AG	AG	--	AG	GG	GG	GG	AG	GG	AG	AG	AG	GG	AG
TT	TT	--	TT	--	GT	--	GT	TT	GT	GT	TT	GT	GT
CC	CC	CT	CC	CT	CT	CT	CT	CT	CT	CT	CT	CT	CT
CC	CC	AC	CC	AC	AC	AC	CC	AC	AC	AC	AC	AC	AC
CC	CC	CT	CC	CT	CC	CT	CC	CT	CC	CC	CC	CC	CC
GG	GG	CG	CG	CG	CG	CG	GG	CC	GG	CG	CG	CG	CG
CC	CC	TT	CT	CT	CT	CT	CC	TT	--	CT	CT	CT	CT
GT	GT	--	TT	--	GT	TT	GT	TT	GT	GT	GT	GT	GT
CT	TT	--	CT	CT	TT	CT	CT	TT	CT	TT	TT	TT	CT
AT	--	--	TT	TT	AT	TT	AT	TT	AT	AT	AT	AT	AT
CC	CC	AC	AC	AC	CC	AC	CC	AC	CC	CC	CC	CC	CC
TT	CT	CC	CT	CT	CT	CT	TT	CT	CT	CT	CT	TT	TT
AA	AA	AG	AG	AG	AA	AG	AA	AG	AA	AA	AA	AA	AA
GG	GG	AG	AG	AG	GG	--	GG	AG	GG	GG	GG	GG	GG
CG	CG	--	GG	CG	CG	GG	GG	CG	GG	CG	GG	GG	GG
TT	CT	--	CC	CT	TT	--	CT	CT	CC	CT	CC	CT	CT
CT	CT	CT	CC	TT	TT	CC	CC	CT	CC	CT	CT	CT	CT
CT	CT	CT	CT	TT	TT	CC	CC	CT	CC	CT	CT	CT	CT
--	TT	--	CT	--	--	--	TT	CT	TT	CT	CT	CT	CT
GT	GG	GT	GT	TT	TT	GG	GG	GT	GG	GT	GT	GT	TT
CT	TT	CT	CT	CC	CC	--	CT	CT	TT	CT	CC	CT	CC
AT	TT	AT	AT	AA	AA	TT	AT	AT	TT	AT	AA	AT	AA
AG	GG	AG	AG	AG	AA	GG	AG	AG	GG	AA	AA	AG	AA
AC	AA	AC	AC	AC	CC	AC	AC	AA	AA	CC	CC	AC	CC

AG	GG	--	AG	AG	AA	--	AG	GG	AG	AA	AA	AG	AA
AG	GG	--	AG	AG	AA	AG	AG	GG	AG	AA	AA	AG	AA
CT	CC	CT	CT	--	TT	CT	CT	CC	CT	TT	TT	CT	TT
AG	AA	--	AG	AG	GG	AG	AG	AA	AG	GG	GG	AG	GG
GT	TT	--	GT	GT	GG	GT	GT	TT	GT	GG	GG	--	GG
CT	CC	CT	CT	CT	TT	CT	CT	CC	CT	TT	TT	CT	TT
AG	AA	--	AG	AG	GG	AG	AG	AA	AG	GG	GG	AG	GG
AG	AA	AG	AG	GG	GG	AG	AG	AA	AG	GG	GG	AG	GG
AG	GG	--	AG	AG	AA	--	AG	GG	AG	AA	AA	--	AA
CG	CC	CG	CG	--	GG	CG	CG	CC	CG	GG	GG	CG	GG
AG	GG	AG	AG	AA	AA	AG	AG	GG	AG	AA	AA	AG	AA
AC	CC	AC	AC	--	--	--	AC	CC	AC	AA	AA	AC	AA
AG	--	--	AG	GG	GG	AG	AG	AA	AG	GG	GG	AG	GG
AG	AA	AG	AG	--	GG	AG	AG	AG	AG	GG	GG	AG	GG
AT	TT	AT	AT	AA	AT	AT	AT	AT	AT	AA	AA	AT	AA
CT	CC	CT	CT	TT	CT	CT	CT	CT	CT	TT	TT	CT	TT
CT	CC	--	CT	TT	CT	CT	CT	CT	CT	TT	TT	CT	TT
CG	GG	CG	CG	CC	CG	CG	CG	CG	CG	CC	CC	CG	CC
CT	TT	CT	CT	CC	CT	CT	CT	CT	CT	CC	CC	CT	CC
CT	CC	--	CT	TT	CT	TT	CT	CT	CT	TT	TT	CT	TT
CT	TT	CT	CT	CC	CT	--	CT	CT	CT	CC	CC	CT	CC
AT	TT	AT	AT	AA	AT	AT	AT	AT	AT	AA	AA	AT	AA
AT	AA	--	AT	--	AT	AT	AT	AT	AT	TT	TT	AT	--
CT	TT	--	CT	CC	CT	--	CT	CT	CT	CC	CC	CT	CC
CT	CC	--	CT	TT	CT	TT	CT	CT	CT	TT	TT	CT	TT
AT	AA	AT	TT	TT	AT	--	AT	AT	AT	TT	TT	AT	TT
AG	GG	AG	AA	AA	AG	AG	AG	AG	AG	AA	AA	AG	AA
GT	GG	GT	TT	TT	GT	GT	GT	GT	GT	TT	TT	GT	TT
CT	TT	--	CC	CC	CT	CT	CT	CT	CT	CC	CC	CT	CC
CG	CC	CG	GG	GG	CG	CG	CG	CG	CG	GG	GG	CG	GG
AG	GG	--	AA	AA	AG	AG	AG	AG	AG	AA	AA	AG	AA
CT	TT	--	CC	CC	CT	--	CT	CT	CT	CC	CC	CT	CC
CT	TT	CT	CC	CC	CT	CT	CT	CT	CT	CC	CC	CT	CC
AT	TT	--	AA	AA	AT	--	AT	AT	AT	AA	AA	AT	AA
AG	AA	AG	GG	GG	AG	--	AG	AG	AG	GG	GG	AG	GG
AG	AA	AA	GG	GG	AG	AA	AG	AG	AG	GG	GG	AG	GG
GT	TT	TT	GG	GG	GT	TT	GT	GT	GT	GG	GG	GT	GT
AG	AA	--	GG	GG	AG	AA	AG	AG	AG	GG	GG	AG	AG
AG	GG	--	AG	AG	GG	--	AG	AG	AG	AG	AG	GG	GG
CT	CT	--	TT	CT	TT	CT	CT	TT	TT	CT	CT	TT	TT
AG	AG	--	AG	--	AG	AG	AA	AG	AG	AA	AG	AG	AG
AG	GG	AG	AG	AG	AG	GG	AG	AG	AG	AG	GG	AG	AG
AA	AG	--	AG	--	AG	AG	--	AG	AG	AA	AG	AG	GG
GG	CG	GG	CG	GG	CG	CG	GG	CG	GG	GG	CG	CG	CC
CC	CT	CC	CT	CC	TT	CT	CC	CT	CC	CC	CT	CT	TT
CC	CC	--	AC	CC	AC	--	CC	AC	CC	CC	CC	AC	AC
AA	AG	--	AA	AA	AG	AG	AA	AG	AA	AA	--	AA	AG

CC	CC	--	AC	CC	AC	--	CC	AC	CC	CC	CC	AC	AC
AC	CC	AC	AC	AC	CC	--	AC	CC	AC	AC	CC	AC	CC
TT	TT	TT	CT	TT	CT	TT	TT	CT	TT	TT	TT	CT	CT
AA	AG	--	AG	AA	GG	AG	AG	GG	AA	AG	AG	AG	GG
GG	AG	--	AG	GG	AA	AG	AG	AA	GG	AG	AG	AG	AA
CT	CT	--	CT	TT	CC	CT	CT	CC	TT	CT	CT	CT	CC
CT	TT	--	CT	CT	--	--	CT	CT	CT	CT	TT	CT	TT
CG	CG	--	CG	--	GG	CG	CG	CG	CC	CG	CG	CG	GG
AG	AG	AA	AG	AA	GG	AG	AG	AG	AA	AG	AG	AG	GG
CT	CT	--	--	--	CC	CT	CT	CT	TT	CT	CT	--	CC
AT	AA	--	AA	--	AA	--	AT	AT	AT	AT	AA	--	AA
AC	AC	--	AC	--	AA	--	AC	AC	CC	AC	AC	AC	AC
AG	AG	--	--	--	AG	--	AG	AG	GG	AG	GG	AG	AG
CT	CT	--	CC	CC	CT	CC	CT	CC	CC	CT	CC	CT	CT
CT	CT	CT	CC	--	CT	--	CT	CC	CC	CT	CC	CT	CT
GT	GT	--	GG	GG	GT	GG	GT	GT	GG	GT	GG	GT	GT
CT	CT	CT	CT	CT	CT	CT	CT	TT	TT	CT	TT	TT	CT
GG	AG	--	GG	--	AG	--	AG	GG	GG	GG	GG	GG	GG
--	AG	AG	AG	AG	AG	AG	AG	GG	GG	GG	GG	GG	AG
GG	GG	--	CG	GG	CG	CG	CG	CG	--	CG	GG	--	GG
GG	AA	AG	GG	AG	AG	GG	AG	GG	GG	GG	AG	AG	GG
AA	GG	--	AA	AG	AG	AA	AG	AA	AA	AA	AG	AG	AA
TT	GT	--	TT	GT	TT	TT	TT	GT	TT	TT	GT	GT	TT
AA	GG	AA	AA	AG	AG	AA	AG	AG	AA	AA	AG	AG	AA
AA	GG	--	AA	AG	AG	AA	AG	AG	AA	AA	AG	AG	AA
GG	GT	--	GG	GT	GG	GG	GG	GG	GG	GG	GT	GT	GG
CC	TT	--	CC	CT	CT	CC	CT	CC	CC	CC	CT	CC	CC
TT	CC	TT	TT	CT	CT	TT	CT	CT	TT	TT	CT	TT	TT
CC	CT	CC	CC	CC	CT	CC	CC	CT	CC	CC	CC	CC	CC
AA	GG	AA	AA	AG	AG	AA	AG	AG	AG	AG	AG	AA	AG
CC	TT	CC	CC	CT	CT	CC	CT	CT	CT	CT	CT	CC	CT
AA	GG	--	AA	AG	AA	AA	AG	AG	AG	AG	AG	AA	AG
CC	TT	--	CC	CT	CT	--	CT	CT	CT	CT	CT	CC	CT
CG	CC	--	GG	CG	CG	CG	CG	CC	CG	CG	GG	GG	CG
CT	TT	CC	CC	CT	CT	CC	CT	TT	CC	CT	CC	CC	CT
AA	AA	--	AC	--	CC	AC	AC	AC	AC	AC	AC	AC	AC
GG	GG	--	AG	--	--	AG	AG	AG	AG	AG	AG	--	AG
TT	TT	TT	CT	CT	CC	CT	CT	CT	CT	CT	CT	CT	CT
AA	AA	--	AG	AG	GG	AG	AG	AG	AG	AA	AG	AG	AG
AG	AG	--	AG	AG	GG	AG	AG	GG	AG	AA	AG	AG	AG
TT	TT	--	TT	TT	CT	TT	CT	CT	TT	TT	TT	TT	TT
AG	AG	--	GG	AG	AA	--	AA	AA	GG	GG	AG	GG	AG
AG	AG	AG	--	AG	AA	AG	AG	AG	AA	AA	AG	AA	AG
TT	GT	GT	GT	GT	TT	GT	GT	GT	TT	TT	GT	TT	GT
GT	GT	--	GT	GT	TT	--	GT	GT	TT	TT	GT	--	GT
AA	AC	AC	AC	AC	AC	AC	CC	AC	AA	AA	AC	AA	AC
AA	AG	--	AG	AA	GG	--	GG	AG	AG	AG	AG	AA	AG

CC	--	--	CG	--	GG	CG	GG	CG	CG	CG	CG	--	CG
CC	CG	--	CG	CC	GG	--	GG	CG	CG	CG	CG	CC	CG
TT	CT	TT	CT	TT	CC	CT	CT	CT	CT	CT	CT	TT	TT
CC	CT	CC	CC	CC	--	CC	CT	CT	CC	CT	CT	CC	CC
AA	AT	--	AT	AA	TT	AA	AT	AT	AA	AT	AT	AA	AA
AG	GG	AG	AG	AG	GG	AG	AG	AG	AG	GG	GG	AG	AG
CC	CT	--	CT	--	TT	--	CT	CT	CC	CT	CT	CC	CT
TT	CT	CT	CT	TT	CC	--	CT	CT	TT	CT	CT	TT	CT
CC	CT	--	CT	CC	TT	TT	CT	CT	CC	CT	CT	CC	TT
GG	AG	AG	AG	GG	AA	AA	AG	AG	GG	AG	AG	GG	AA
CC	CC	--	CG	--	CG	--	CG	CG	CC	CG	CC	CC	CG
GG	CG	--	CG	GG	CG	CG	GG	CG	GG	CG	CG	GG	CG
GG	GG	--	GT	GG	GT	GT	GT	GT	GG	GT	GG	GG	GT
CC	CC	--	CG	CC	CG	CC	CC	CC	CC	CC	CG	CC	CC
AG	AG	AG	AA	GG	AG	--	GG	AG	AG	AG	AG	GG	GG
AA	AA	AA	AC	AA	AA	AA	AA	AA	AA	AA	AC	AA	AA
AA	AA	AT	AT	AA	AT	AA	AA	AA	AA	AA	AT	AA	AA
AG	AG	AG	AA	GG	AG	AG	GG	AG	AG	GG	AG	GG	GG
AT	AT	AT	AT	AA	AT	AT	AA	TT	AT	AT	AT	AA	AA
AG	AA	--	AG	GG	AG	AG	GG	AA	AA	AG	AG	GG	GG
AC	AC	CC	AC	CC	CC	AC	CC	AC	AC	CC	CC	CC	CC
CT	TT	--	CT	CC	CT	CT	CC	TT	TT	CT	CT	CC	CC
GT	GG	GT	GT	TT	GT	GT	TT	GG	GG	GT	GT	GG	TT
GT	GG	--	GT	TT	GT	GT	TT	GG	GG	GT	GT	GG	TT
CT	TT	CT	CT	CC	CT	CT	CC	TT	TT	CT	CT	TT	CC
CG	GG	CG	CG	CC	CG	CG	CC	GG	GG	CG	CG	GG	CC
AG	AA	--	--	GG	AG	--	GG	AA	AA	AG	AG	--	GG
CT	TT	CT	CT	CC	CT	--	CC	TT	TT	CT	CT	TT	CT
GT	GG	GT	GT	TT	GT	GT	TT	GG	GG	GT	GT	GG	TT
CT	CC	CT	CT	TT	CT	CT	TT	CC	CC	CT	CT	CC	CT
CG	CC	--	CG	--	CG	CG	GG	CC	CC	CG	CG	CC	CG
AG	AA	AG	AG	GG	AG	AG	GG	AA	AA	AG	AG	AA	AG
AC	CC	AC	AC	AA	AC	AC	AA	CC	CC	AC	AC	CC	AC
CT	CC	--	CT	--	CT	CT	TT	CC	CC	CT	CT	CC	CT
GG	AG	--	GG	--	AG	GG	GG	AG	AG	AG	AG	AG	GG
AC	CC	--	AC	AA	AC	AC	AA	CC	CC	AC	AC	--	AC
CT	TT	CT	CT	CC	CT	CT	CC	TT	TT	CT	CT	TT	CT
AG	AA	AG	AG	GG	AG	AG	GG	AA	AA	AG	AG	AA	AG
CG	CC	CG	GG	--	CG	--	GG	CC	CC	CG	CG	CC	CG
GT	TT	GT	GG	--	GT	GT	GG	TT	TT	GT	GT	TT	GT
CT	TT	--	CC	--	CT	CT	CC	TT	TT	CT	CT	TT	CT
TT	CT	CT	TT	TT	CT	TT	TT	CT	CT	CT	CT	CT	TT
GG	GG	AG	AG	--	AG	AG	AG	GG	GG	AG	--	GG	GG
CG	GG	GG	CC	CC	CG	CG	CC	GG	GG	CG	CG	GG	CG
AT	AT	--	AA	AA	AA	AT	AA	AT	AT	AA	AA	AT	AT
AA	AG	AG	AA	AA	AG	AA	AA	AG	AG	AG	AG	AG	AA
CC	AC	CC	CC	CC	AC	CC	CC	AC	AC	AC	AC	AC	CC

TT	CT	CT	TT	TT	CT	TT	TT	CT	CT	CT	CT	CT	TT
CT	CT	--	TT	TT	CT	--	TT	CT	TT	TT	TT	CT	CT
AG	AT	GT	GG	GG	AT	AG	AG	AT	GT	GT	GT	AT	AG
AA	AT	AT	AA	AA	AT	AA	AA	AT	AT	AT	AT	AT	AA
AG	AA	AA	GG	GG	AA	AG	AG	AA	AA	AG	AG	AA	AG
AC	AA	--	CC	CC	--	AC	AC	--	AA	AC	AC	AA	AC
AA	AG	AG	AA	AA	AG	AA	AA	AG	AG	AG	AG	AG	AA
AG	AA	--	GG	--	AG	AG	AG	AA	AA	AG	AG	AA	AG
AG	GG	--	AA	AA	AG	AG	AG	GG	GG	AG	AG	--	AG
AG	AA	--	GG	--	AG	AG	AG	AA	AA	AG	AG	AA	AG
GT	TT	--	GG	GG	TT	--	GT	TT	GT	GT	GT	TT	GT
AG	AG	--	GG	GG	AG	AG	AG	GG	GG	GG	GG	AG	AG
CT	--	--	CC	--	TT	CT	CT	CT	CT	CT	CT	TT	CC
CG	GG	CG	CG	CC	GG	CG	CG	CG	CG	CG	CG	GG	CC
TT	CT	CT	TT	TT	CT	TT	TT	CT	CT	CT	CT	CT	TT
CT	CT	--	CT	TT	CT	CT	CT	TT	TT	TT	TT	CT	TT
CC	CT	CT	CC	CC	CC	CT	CT	CC	CC	CC	CT	CC	CT
GT	GG	GG	GG	GT	GG	GG	GG	GT	GT	GT	GT	GG	GG
AG	AG	AG	GG	AG	GG	AA	AG	AG	AG	AG	AG	GG	AG
AG	AG	--	AA	AG	AA	GG	AG	AG	AG	AG	AG	AA	AG
AA	AG	--	AA	--	AA	AA	AG	AA	AA	AA	AG	AA	AG
GT	GT	--	GG	GT	GG	TT	GT	GT	GT	GT	TT	GG	GT
AA	AG	AG	AG	AA	AA	AG	AG	AA	AA	AA	AG	AA	AG
CC	CT	--	CT	--	CT	CT	CT	--	CC	CC	CT	CC	CT
AG	AA	--	AA	AG	AA	--	AA	AG	AG	AG	AA	AG	AA
AG	AG	AG	AG	AG	AG	AA	AA	AG	AG	AG	AA	GG	AA
AG	AG	AG	AG	AG	AA	AA	AA	AG	AG	AG	AG	GG	AA
CG	CG	--	CG	CG	CC	--	CC	CC	CG	GG	CG	GG	CC
CG	CG	--	CG	CG	--	--	CC	CC	CG	GG	CG	--	CC
AG	AG	AG	AG	GG	AA	AA	AA	AA	AG	GG	AG	GG	AA
AG	GG	--	GG	--	AG	AG	AG	AG	AG	GG	AG	GG	AG
CT	CT	--	CT	TT	CC	CC	CC	--	CT	TT	CT	TT	CC
AC	CC	--	CC	--	AC	AC	AC	AC	AC	CC	AC	CC	CC
CG	CC	--	CC	CC	CG	--	CG	CG	CG	CC	CG	CC	CC
AC	CC	CC	CC	CC	CC	AC	AC	AC	AC	CC	AC	AC	CC
AC	AC	--	AC	AC	AC	CC	AC	CC	AC	AA	AC	CC	AC
AA	AG	AG	AG	AG	AG	AG	AA	AG	AA	AA	AA	AA	AG
AG	AG	AG	AG	AG	AG	GG	AG	GG	AG	AA	AG	GG	AG
GG	AG	AG	AG	AG	AG	AG	GG	AG	GG	GG	GG	AG	AG
CT	CT	CT	CT	CT	CT	TT	CT	TT	CT	CT	CC	TT	CT
CT	CT	--	CT	CT	CT	TT	CT	TT	TT	CT	CC	TT	CC
AG	AA	AA	AA	AA	AA	AG	AG	AG	AG	AG	AA	AG	AA
CT	CC	--	CC	CC	CC	CT	CT	CT	CT	CT	CC	CT	CC
AA	AG	--	AG	AG	AA	AG	GG	AA	GG	AG	GG	AA	AG
AC	AC	--	CC	AC	AC	--	CC	AC	CC	AC	CC	AC	CC
CT	CC	CT	CT	CC	CT	CC	CC	CT	CC	CT	CC	CT	CT
GG	AG	--	AG	AG	GG	AG	AA	AG	AA	GG	AA	GG	AG

AG	AA	AG	AG	AA	AG	AA	AA	AG	AA	AG	AA	AG	AG
GG	AG	--	AG	--	AG	AG	AA	AG	AA	GG	AA	AG	AG
AA	AA	AA	AC	AC	AC	AC	CC	AC	CC	AA	CC	AC	AC
AA	AA	AA	GG	AG	AG	AG	GG	AG	GG	AG	GG	AG	GG
GG	GG	--	TT	GT	GT	--	TT	GT	TT	GT	TT	--	TT
AG	AG	--	AA	AG	AA	AA	AA	AA	AA	AG	AA	AA	AA
CC	CC	--	TT	TT	CT	TT	TT	CT	TT	CT	CT	CT	TT
CT	CT	CT	CC	CC	CC	CC	CC	CT	CC	CT	CT	CC	CC
AA	AA	AG	GG	GG	AG	GG	AG	AA	GG	AG	AG	AG	GG
CG	CG	CG	GG	GG	CG	--	CG	CG	GG	GG	GG	CG	GG
TT	CT	CT	TT	--	CT	TT	CT	CT	TT	TT	CT	CT	TT
AG	GG	GG	AA	AA	AG	AG	AG	GG	AA	AG	GG	AG	AA
AG	GG	AG	AA	AA	AG	AG	AG	GG	AA	AG	GG	AG	AA
CG	GG	--	CC	CC	GG	--	CG	GG	CC	CG	GG	--	CC
AA	GG	--	AA	AA	AG	AG	AG	GG	AA	AG	GG	--	AA
AG	AG	--	AG	AG	GG	--	AG	--	GG	AG	AG	AG	GG
CT	CT	CT	CT	CT	TT	CC	CT	TT	CT	CT	CT	CT	TT
AG	AG	AG	--	AG	GG	--	AG	GG	AG	AG	AG	AG	GG
CG	CG	--	CG	CG	CC	GG	CG	CC	CG	CG	CC	--	CC
AC	AC	--	AC	AC	CC	--	AC	CC	AC	AC	CC	--	CC
AG	AG	AA	AG	AG	AA	GG	AG	AA	AG	AA	AA	AG	AA
GT	GT	--	GT	GT	GG	TT	GT	GG	GT	GG	GG	GT	GG
GG	AG	--	AG	AG	GG	--	AG	GG	AG	GG	GG	AG	GG
AG	AG	GG	AG	AG	GG	AA	AG	GG	AG	GG	GG	AG	GG
AG	AG	--	AG	AG	AA	--	AG	AA	AG	AA	AA	AG	AA
AC	AC	AC	AC	AC	--	CC	AC	AA	AC	AA	AA	AC	AA
AG	AG	--	AG	AG	AA	GG	AG	AA	AG	AA	AA	AG	AA
AG	AG	AA	AG	AG	AA	GG	AG	AA	AG	AA	AA	AG	AA
CT	CT	TT	CT	CT	TT	CC	CT	TT	CT	TT	TT	CT	TT
CG	CG	GG	CG	CG	GG	CC	CG	GG	CG	GG	GG	CG	GG
AC	AC	--	AC	--	AA	--	AC	AA	AC	AA	AA	AC	AA
CT	CT	--	CT	CT	TT	CC	CT	TT	CT	TT	TT	CT	TT
GT	GT	--	GT	GT	TT	GG	GT	TT	GT	TT	TT	GT	TT
CT	CT	--	--	--	CC	TT	CT	CC	CT	CC	CC	CT	CC
CG	CG	--	CG	CG	CC	GG	CG	CC	CG	CC	CC	CG	CC
AT	AT	TT	AT	AT	TT	AA	AT	TT	AT	TT	TT	AT	TT
CT	CT	--	CT	CT	CC	CT	CT	CC	CT	CC	CC	CT	CC
AG	AG	--	AG	AG	GG	AA	AG	GG	AG	GG	GG	AG	GG
AG	AG	AA	AG	AG	AA	GG	AG	AA	AG	AA	AA	AG	AA
AT	AT	--	AT	--	AA	--	AT	AA	AT	AA	AA	AT	AA
AG	AG	GG	AG	AG	GG	AA	AG	GG	AG	GG	GG	AG	GG
AG	AG	--	AG	--	AA	--	AG	AA	AG	AA	AA	AG	AA
CG	CG	--	CG	CG	CC	--	CG	CC	CG	CC	CC	CG	CC
AG	AG	AA	AG	AG	AA	GG	AG	AA	AG	AA	AA	AG	AA
CG	CG	--	CG	CG	GG	CC	CG	GG	CG	GG	GG	--	GG
AG	AG	--	AG	AG	GG	--	AG	GG	AG	GG	GG	AG	GG
AC	AC	--	AC	AC	AA	CC	AC	AA	AC	AA	AA	AC	AA

AG	--	--	AG	AA	GG	--	AG	GG	AG	GG	GG	--	GG
CG	CG	--	CG	--	CC	--	CG	CC	CG	CC	CC	CG	CC
CT	CT	--	CT	CT	CC	TT	CT	--	CT	CC	CC	CT	CC
AT	AT	--	AT	AT	TT	AA	AT	TT	AT	TT	TT	AT	TT
CT	CT	--	CT	CT	CC	TT	CT	CC	CT	CC	CC	CT	CC
AT	AT	AA	AT	AT	AA	TT	AT	AA	AT	AA	AA	AT	AA
AT	AT	--	AT	AT	TT	AA	AA	TT	AT	TT	TT	AT	TT
CG	CG	GG	--	CG	GG	CC	CC	GG	CG	GG	GG	CG	GG
CT	CT	CC	CT	CT	CC	TT	TT	CC	CT	CC	CC	CT	CC
AG	AG	AA	AG	AG	AA	GG	GG	AA	AG	AA	AA	AG	AA
AC	AC	AA	AC	AC	AA	CC	CC	AA	AC	AA	AA	AC	AA
GT	GT	TT	GT	GT	TT	GG	--	TT	GT	TT	TT	GT	TT
CT	CT	--	CT	CT	CC	TT	TT	CC	CT	CC	CC	CT	CC
CG	CG	GG	CG	CG	GG	CC	CC	GG	CG	GG	GG	CG	GG
GT	GT	--	GT	GT	TT	--	GG	TT	GT	TT	TT	GT	TT
CT	CT	TT	CT	CT	TT	CC	CC	TT	CT	TT	TT	CT	TT
AG	AG	--	AG	AG	AA	--	GG	AA	AG	AA	AA	AG	AA
AC	AC	--	AC	AC	AA	CC	CC	AA	AC	AA	AA	AC	AA
CT	CT	CC	CT	CT	CC	--	TT	CC	CT	CC	CC	CT	CC
AC	AC	--	AC	AC	AC	CC	CC	AA	AC	AA	AA	AC	AA
AG	AG	--	AG	AG	AG	GG	GG	AA	AG	AA	AA	AG	AA
GT	GT	--	GT	GT	GT	--	TT	GG	GT	GG	GG	GT	GG
GT	GT	--	GT	GT	GT	GT	TT	GG	GT	GG	GG	TT	GG
AG	AG	--	AG	AG	AG	AA	AA	GG	AG	GG	GG	AA	GG
CT	CT	CC	CT	CT	CT	TT	TT	CC	CT	CC	CC	TT	CC
--	CT	--	CT	CT	CT	TT	TT	CC	CT	CC	CC	TT	CC
AG	AG	AA	AG	AG	AG	GG	GG	AA	AG	AA	AA	GG	AA
CT	CT	--	CT	CT	CT	CT	TT	CC	CT	CC	CC	TT	CC
CT	CT	TT	CT	CT	CT	TT	CC	TT	CT	TT	TT	CC	TT
AG	AG	GG	AG	AG	AG	GG	AA	GG	AG	GG	GG	AA	GG
CT	CT	--	CT	CT	CT	CC	TT	CC	CT	CC	CC	TT	CC
CT	CT	TT	CT	CT	CT	TT	CT	TT	CT	TT	TT	CT	TT
CT	TT	--	CT	TT	CT	CC	TT	CC	CT	CC	CC	TT	CC
AG	GG	AA	AG	GG	AG	AA	GG	AA	AG	AA	AA	GG	AA
AC	CC	--	AC	CC	AC	AA	CC	AA	AC	AA	AA	CC	AA
CG	CG	CG	CG	CC	CG	CG	CC	CG	CC	CG	CG	CC	CG
GT	GT	--	GT	GT	GT	--	TT	GT	GT	GT	GG	--	GG
AG	AG	GG	AG	AG	GG	GG	AA	AG	AG	AG	GG	AA	GG
CT	CT	--	CT	TT	TT	CT	CT	CT	--	CT	TT	CC	TT
AG	AG	--	AG	AG	AG	AG	GG	AG	GG	AG	AG	GG	AG
GG	AG	--	AG	GG	GG	AG	AG	AG	AG	AG	GG	AA	GG
GG	GG	--	CG	--	GG	--	CG	CG	CG	CG	GG	CC	GG
CT	CT	--	CT	CT	CT	CT	TT	TT	TT	CT	CT	TT	TT
TT	TT	--	GT	TT	TT	--	GT	GG	GT	GT	TT	GG	GT
GG	GG	--	AG	AG	AG	AG	AG	AG	GG	GG	AG	AG	GG
GG	GG	AG	AG	GG	AG	GG	GG	GG	AG	GG	AG	GG	GG
AA	AA	--	AC	CC	--	AC	AC	AC	AC	AA	CC	AA	AC

TT	TT	--	AT	AT	AT	AT	AT	AT	AT	TT	AA	TT	AT
TT	TT	--	AT	--	TT	AT	TT	AT	AT	TT	AA	--	AT
AA	AA	--	AT	TT	AA	--	AA	AT	AT	AA	TT	AA	AT
CC	TT	CT	CT	CC	TT	CT	TT	CT	CT	TT	CC	TT	CT
AA	GG	GG	AG	AA	GG	AG	GG	AG	AG	GG	AA	GG	AG
CC	AA	AA	AC	CC	AA	AC	AA	AC	AC	AA	CC	AA	AC
CC	TT	--	CT	--	TT	CT	TT	CT	CT	CT	CC	TT	CT
AA	GG	--	AG	AA	GG	--	GG	AG	AG	AG	AA	GG	AG
TT	GG	GG	GT	TT	GG	GT	GG	TT	GG	GT	TT	GG	GT
AA	GG	--	AG	AA	GG	AG	GG	AA	GG	AG	AA	GG	AG
CG	CC	CC	CC	CG	CC	CG	CC	CG	CC	CC	CG	CC	CC
AG	GG	GG	GG	AG	GG	AG	GG	AG	GG	GG	AG	GG	GG
GG	CC	CC	CG	GG	CC	GG	CC	GG	CC	CG	GG	CC	CG
GG	AA	--	AG	GG	AA	GG	AA	GG	AA	AA	GG	AA	AG
AA	GG	--	AG	AA	GG	AA	GG	--	GG	GG	AA	GG	AG
GG	AA	AA	AG	GG	AA	GG	AA	GG	AG	AA	GG	AA	AG
GT	GG	--	GG	GT	GG	GT	GG	GT	GG	GG	GT	GG	GG
AA	GG	--	GG	AA	GG	AA	AG	AA	AG	GG	AA	GG	AG
GT	TT	--	TT	GT	TT	GT	TT	GT	TT	TT	GT	TT	TT
CC	GG	--	GG	CC	GG	CC	CG	CC	CG	GG	CG	GG	GG
TT	CT	--	CT	TT	TT	TT	TT	TT	CT	TT	TT	TT	CT
CC	CG	--	CG	CG	GG	CG	GG	--	CC	--	GG	GG	CC
CG	CC	CC	CC	CG	CC	--	CC	CG	CG	CG	CC	CC	CG
AC	AC	AA	AC	AA	AA	AC	AA	AA	AC	AC	AA	AA	AC
CT	CT	--	CT	CC	CC	--	CC	--	CT	CT	CC	CC	CT
AG	AG	--	AG	AA	AA	AG	AA	AA	AA	AG	AA	AA	AA
CT	CT	CC	CT	CC	CC	CT	CC	CC	CC	CT	CC	CC	CC
CG	GG	--	GG	CG	CG	GG	GG	CG	GG	CG	GG	GG	CG
--	CC	--	CT	--	CT	CT	CC	CC	CC	CT	CC	CC	CC
GG	AA	--	AG	AG	GG	GG	AA	AG	AA	AG	AA	AA	AG
GT	GG	GG	GT	GG	GT	GT	GG	GG	GG	GT	GG	GG	GT
GG	TT	--	GT	GT	GG	--	TT	GT	TT	GT	TT	TT	GG
CG	GG	--	CG	GG	CG	CG	GG	GG	GG	CG	GG	GG	CG
CC	GG	--	CG	CG	CC	--	GG	CG	GG	CG	GG	CG	--
AG	GG	GG	AG	GG	GG	AG	GG	--	GG	AG	GG	GG	AG
CC	TT	--	CT	CT	CT	CC	TT	CT	TT	CT	TT	CT	CC
TT	GG	--	GT	GT	GG	TT	GG	GT	GG	GT	GG	GT	TT
AG	GG	GG	AG	GG	GG	AG	GG	GG	GG	AG	GG	GG	AG
GG	AA	--	AG	--	AG	--	AA	AG	AA	AG	AA	AG	GG
CT	TT	--	TT	CC	CT	--	TT	CT	TT	CT	TT	CT	CT
TT	TT	--	TT	TT	TT	AT	TT	TT	TT	AT	TT	TT	AT
GT	TT	--	TT	TT	TT	GT	TT	TT	TT	GT	TT	TT	GT
--	CC	--	CC	CT	CT	--	CC	CT	CC	CT	--	CT	CT
AG	AA	--	AA	AA	AA	AG	AA	AA	AA	AG	AA	AA	AG
AT	TT	TT	TT	--	AT	--	TT	AT	TT	AT	TT	AT	AT
AG	AA	--	AA	AA	AA	AG	AA	AA	AA	AG	AA	AA	AG
CT	TT	--	TT	TT	TT	CT	TT	TT	TT	CT	TT	TT	CT

AT	TT	--	TT	AT	TT	AT	TT	TT	TT	AT	TT	TT	AT
GT	GG	--	GG	--	GT	--	GG	GT	GG	GT	GG	GT	GT
AG	AA	AA	AA	GG	AG	GG	AA	GG	AA	AG	AA	AG	AG
CT	CC	--	CT	TT	CT	--	CC	TT	CC	CC	CC	CT	CT
CT	CC	--	CT	CT	CT	TT	CC	TT	CC	CC	CC	CT	CT
CT	CC	--	CT	CT	CT	--	CC	TT	CC	CC	CC	CT	CT
GT	GT	GT	GT	GT	GT	TT	GG	TT	GG	GG	GG	GT	GT
CG	CG	--	CG	CG	GG	GG	CC	GG	CC	CC	CC	CG	CG
CC	AC	--	AC	AC	AA	AA	CC	AA	CC	CC	CC	AC	AC
AA	AG	--	AG	AG	GG	AG	AA	AG	AG	AA	AA	AG	AG
GG	AG	--	AG	AG	AA	AG	GG	AG	AG	GG	GG	AG	AG
CT	CT	CC	CC	--	CT	CC	CC	CC	CT	CT	CT	CT	CT
AC	AC	--	CC	AC	AC	CC	CC	CC	AC	AC	AC	AC	AC
TT	GT	GT	GT	TT	GT	GT	GT	GT	GT	TT	GT	GT	TT
CG	CG	--	CG	CG	GG	CG	CG	CG	GG	CG	GG	CG	GG
AG	AA	--	AA	AG	AG	AA	AA	AA	AG	AG	AG	AA	AG
CT	CT	--	CT	CT	TT	CT	CT	CT	TT	CT	TT	CT	TT
TT	GT	--	GT	--	GT	GT	GT	GT	TT	TT	GT	GT	GT
CT	CT	CC	CT	CT	CC	CT	CT	CT	CC	CT	CT	CT	CC
AA	AC	--	AA	AC	AA	--	AC	AC	AA	AA	AC	AA	AC
AG	GG	AG	AG	GG	AA	AG	GG	GG	AA	AG	GG	AG	AG
AA	AG	AG	AA	AG	AA	AA	AG	AG	AA	AA	AG	AA	AG
GT	GG	--	GT	--	TT	--	GG	GG	GT	GT	GG	TT	GT
CT	TT	--	CT	--	CT	CT	TT	TT	CT	CT	TT	CC	CT
AC	CC	AC	AC	AA	AC	AC	CC	CC	AC	CC	CC	AA	AC
AT	AT	--	TT	TT	AT	--	AA	AA	AT	AA	AA	TT	AT
GT	GT	GT	GT	GT	--	GT	GG	GG	GT	--	GG	GT	GG
AA	AA	AG	AG	AG	AA	--	AA	AA	AA	AA	AA	AG	AG
CG	CG	--	CC	CC	GG	CG	GG	GG	CG	GG	GG	CC	CG
AG	AG	--	AA	--	GG	--	GG	GG	AG	--	GG	AA	AG
CG	--	--	CG	--	GG	GG	CG	CG	CG	CG	CC	GG	CG
AG	AG	--	AG	GG	GG	--	AG	AG	AG	AG	AA	GG	AG
AA	AG	--	AA	--	AG	AG	AG	--	AA	AG	AA	AG	AA
AT	AA	--	AA	AT	AT	--	AA	AT	AT	--	AA	AT	AT
AA	AG	--	AA	AG	AG	AG	AG	--	AA	AG	AA	AG	AA
AG	AA	--	AG	AA	AA	AA	AA	AG	AG	AA	AA	AA	AG
AC	AA	--	AA	CC	CC	CC	AC	CC	AC	AC	AC	CC	AA
AC	CC	--	CC	AC	AC	AC	CC	AC	AC	CC	CC	AC	CC
AC	CC	--	CC	AA	AA	AA	AA	AA	AC	AC	AC	AA	CC
GT	GG	--	GG	TT	TT	TT	TT	TT	GT	GT	GT	TT	GG
AT	AA	--	AA	AT	TT	AT	TT	TT	AT	AT	AT	TT	AA
AG	AA	AG	AA	--	AG	AA	AG	AG	AG	AA	AA	AG	AA
AC	CC	--	CC	AC	AC	CC	AC	AC	AC	CC	CC	AC	CC
AT	AA	AT	AA	AT	TT	AT	TT	TT	AT	AT	AT	AT	AA
CT	CC	--	CC	CT	TT	CT	CT	TT	CC	CT	CT	CT	CC
AG	GG	--	AG	AG	AA	GG	AG	AA	GG	AG	AG	GG	GG
CT	CC	--	CT	CT	TT	CC	CT	TT	CC	CT	CT	CC	CC

CT	TT	--	CT	--	CC	--	CT	CC	TT	CT	CT	TT	TT
AG	GG	--	AG	AA	AA	GG	AG	AA	GG	AG	AG	GG	GG
CT	CC	TT	CT	CT	TT	CC	CT	TT	CC	CT	CT	CC	CC
CT	TT	--	CT	CT	CC	--	CT	CC	TT	CT	CT	TT	TT
CT	CC	--	CT	CT	TT	CC	CT	TT	CC	CT	CT	CC	CC
AG	GG	--	AG	AG	AA	--	AG	AA	GG	AG	AG	GG	GG
CT	TT	--	CT	CT	CC	--	CT	CC	TT	CT	CT	TT	TT
AG	GG	--	AG	AG	AA	--	AG	AA	GG	AG	--	--	GG
CG	CC	GG	CG	CG	GG	--	CG	GG	CC	CG	CG	CC	CC
AC	AC	--	AC	AC	CC	AA	AC	CC	AA	AC	AC	AA	AA
CC	CT	--	CT	CT	TT	CC	CT	TT	CC	CT	CT	CC	CC
CC	--	--	CT	CT	TT	--	CT	TT	CC	CT	CT	--	CC
TT	AT	--	AT	AT	AA	--	AT	AA	TT	AT	AT	TT	TT
CC	CT	--	CT	CC	TT	--	CT	--	CC	--	CT	CC	CC
AA	AC	CC	AC	AC	CC	AA	AC	CC	AA	AC	AC	AA	AA
TT	GT	GG	GT	GT	GG	TT	GT	GG	TT	GT	GT	TT	TT
TT	CT	--	CT	CT	CC	--	CT	CC	TT	CT	CT	TT	TT
GG	CG	CC	--	CG	CC	GG	CG	CC	GG	CG	CG	GG	GG
GG	CG	--	CG	CG	CC	GG	CG	--	GG	CG	CG	GG	GG
CC	AC	AA	AC	AC	AA	--	AC	AA	CC	AC	AC	CC	CC
GG	CG	--	CG	--	CC	GG	CG	CC	GG	CG	CG	GG	GG
--	AT	--	AA	--	AA	AT	AT	AT	AT	TT	AT	AA	AT
AG	GG	--	AA	GG	AA	GG	AG	AG	AA	GG	AG	AA	AG
AA	GG	--	AG	GG	AA	--	AG	AG	AA	GG	AG	AG	AG
GG	AA	AG	AG	AA	GG	AA	GG	AG	GG	AG	AA	AG	AG
AA	GG	AG	AG	GG	AA	--	AA	AG	AA	AG	GG	AG	AG
AA	CC	AC	AC	CC	AA	CC	AA	AC	AA	AC	CC	AC	AC
AA	GG	--	AG	GG	AA	GG	AA	AG	AA	AG	GG	AG	AG
AA	GG	--	AG	GG	AA	GG	AA	AG	AA	AG	GG	AG	AG
GG	TT	GT	GT	TT	GT	TT	GG	GT	GG	GT	TT	GT	GT
TT	GG	--	GT	GG	GT	--	TT	GT	TT	GT	GG	GT	GT
CC	AA	AC	AC	AA	AC	AA	CC	AC	CC	AC	AA	AC	AC
TT	CC	--	--	CC	--	--	--	CT	TT	CT	CC	--	CT
TT	--	--	CT	CC	CT	--	TT	CT	TT	CT	CC	--	CT
GG	TT	GT	GT	TT	GT	TT	GG	GT	GG	GT	TT	GT	GT
AA	TT	AT	AT	TT	AT	--	AA	AT	AA	AT	TT	AT	AT
GG	CC	CG	CG	CC	CG	CC	GG	CG	GG	CG	CC	CG	CG
AA	CC	--	AC	CC	AC	--	AA	AC	AA	AC	CC	AC	AC
CC	TT	CT	CT	TT	CT	TT	CC	CT	CC	CT	TT	CT	CT
GG	CC	--	CG	CC	CG	CC	GG	CG	GG	CG	CC	CG	CG
CC	TT	--	CT	TT	CT	--	CC	CT	CC	CT	TT	--	CT
AA	TT	AT	AT	TT	AT	--	AA	AT	AA	AT	TT	AT	AT
TT	CC	--	CT	CC	CT	CC	TT	CT	TT	CT	CC	CT	CT
GG	AA	AG	AG	AA	AG	AA	GG	AG	GG	AG	AA	AG	AG
--	TT	--	AT	TT	TT	TT	AA	AT	AA	AT	TT	AT	AT
AA	TT	--	AT	TT	AT	--	AA	AT	AA	AT	TT	AT	AT
AA	CC	AC	AC	CC	AC	CC	AC	AC	AA	AC	CC	AC	CC

TT	GG	--	GT	GG	GT	GG	GT	GT	TT	GT	GG	GT	GG
GG	AA	--	AG	AA	AG	AA	AG	AG	GG	AG	AA	AG	AA
GG	TT	GT	GT	TT	GT	TT	GT	GT	GG	GT	TT	GT	TT
AA	GG	--	AG	GG	AG	GG	AG	AG	AA	AG	GG	AG	GG
TT	CC	--	CT	CC	CT	--	CT	CT	TT	CT	CC	CT	CC
AA	GG	--	AG	GG	AG	GG	AG	AG	AA	AG	GG	AG	GG
TT	CC	CT	CT	CC	CT	--	CT	CT	TT	CT	CC	CT	CC
CC	AA	AC	AC	AA	AC	AA	AC	AC	CC	AC	AA	AC	AA
AA	GG	--	AG	GG	AG	--	AG	AG	AA	AG	GG	AG	GG
TT	CC	--	CT	CC	CT	--	CT	CT	TT	CT	CC	CT	--
GG	AA	--	AG	--	AG	AG	AG	GG	GG	AG	AA	AG	AA
CC	TT	--	CT	TT	CT	--	CT	--	CC	CT	TT	--	TT
GG	GT	GT	GG	GT	GG	GT	GG	GG	GG	GT	GT	GG	GT
GG	AG	AG	AG	AG	AG	GG	AG	GG	AG	GG	AG	AG	AG
TT	AT	AT	TT	--	AT	AT	AT	TT	AA	AT	AA	AT	AA
TT	AT	AT	TT	AA	AT	AT	AT	TT	AA	AT	AA	TT	AA
CC	CC	--	CC	AA	CC	AC	AC	CC	AA	AC	AA	CC	AA
GG	GG	--	GG	TT	GG	GT	GT	GG	TT	GT	TT	GG	TT
TT	TT	--	TT	CT	TT	CT	TT	CT	TT	TT	TT	CT	CT
AG	AG	--	AG	--	AG	--	AG	--	GG	GG	GG	AG	AA
GG	GG	--	GG	GG	GG	--	GG	CG	CG	CG	CG	CG	GG
TT	TT	--	--	TT	TT	--	TT	CT	CT	CT	CT	CT	TT
GT	GG	GG	GT	GG	GT	GG	GT	GG	GT	GT	GT	GG	GG
--	AA	--	AA	--	AG	--	AG	AG	GG	GG	GG	AG	AG
AA	AA	--	AA	AA	AA	--	AA	AA	AC	AC	AA	AC	AA
--	GG	--	GG	--	GG	GG	GG	--	CG	CG	CG	GG	GG
GT	TT	--	TT	TT	TT	TT	TT	TT	TT	GT	TT	GT	TT
TT	CC	--	CC	CC	TT	--	CT	CT	CT	CT	CT	TT	CT
AG	AG	AG	AG	GG	AG	AG	GG	GG	GG	AA	GG	AG	GG
CT	CC	--	CC	--	CT	--	CC	CC	CC	CT	CC	CC	CC
CC	CG	CG	CG	CG	CC	CG	CG	CG	CG	CC	CG	CC	CG
AA	AC	AC	AC	AC	AA	--	AA	AA	AC	AC	AA	AA	AA
AA	GG	GG	GG	GG	AA	GG	AG	AG	GG	AG	AG	AA	AG
CT	TT	--	TT	TT	CT	TT	CT	CT	TT	TT	CT	CT	CT
CG	GG	--	GG	GG	CG	GG	GG	GG	GG	CG	GG	CG	GG
GG	AA	--	AA	AA	GG	AA	AG	AG	AA	AG	AG	GG	AG
GG	CG	CG	CG	CG	GG	CG	CG	CG	CG	GG	CG	GG	CG
GG	CC	--	CC	CC	GG	CC	CG	CG	CC	CG	CG	GG	CG
TT	CC	--	CC	CC	TT	CC	CT	CT	CC	CT	CT	TT	CT
AA	GG	--	GG	GG	AA	GG	AG	AG	GG	AG	AG	AA	AG
GG	AG	AG	AG	AG	GG	AG	GG	GG	AG	AG	GG	GG	GG
TT	CC	--	CC	CC	TT	CC	CT	CT	CC	CT	CT	TT	CT
AG	GG	GG	GG	--	AG	GG	AG	AG	GG	GG	AG	AG	AG
AA	GG	GG	GG	GG	AA	GG	AG	AG	GG	AG	AG	AA	AG
AA	CC	CC	CC	CC	AA	CC	AC	AA	CC	AC	AC	AA	AC
AT	TT	--	TT	TT	AT	--	TT	AT	TT	AT	TT	AT	TT
GG	AA	--	AA	--	GG	AA	AG	GG	AA	AG	AG	--	AG

CC	AA	--	AA	AA	CC	--	AC	CC	AA	AC	AC	--	AC
GG	AA	--	AA	--	--	AA	AG	GG	AA	AG	AG	GG	AG
AG	AA	--	AA	AA	AG	--	AG	AG	AA	AA	AG	AG	AG
CT	CC	--	CC	--	CT	CC	CC	CT	CC	--	CC	CT	CC
CG	CC	--	CC	CC	CG	CC	CC	CG	CC	CG	CC	CG	CC
CT	TT	--	CT	TT	CT	--	TT	CT	TT	CT	TT	CT	TT
GG	CG	CG	GG	CG	GG	CG	CG	GG	CG	GG	GG	GG	CG
CC	CC	--	CT	CC	CT	CT	CC	CT	CC	CT	CT	CT	CC
GT	GT	--	GG	GT	GG	--	GT	GG	GT	GG	GG	--	GT

3-29	3-31	3-32	3-33	3-36	3-37	3-38	3-40	3-41	3-44	3-45	3-46	3-47
AC	AA	AC	AC	AC	AA	AC	AC	CC	--	CC	AA	AA
AG	AA	AG	AG	AG	AA	AG	AG	GG	AG	GG	AA	AA
CT	TT	CT	CT	--	TT	CT	--	CC	--	CT	TT	TT
AG	GG	GG	AG	AG	AG	--	AG	AG	--	AG	AG	GG
AG	AA	AG	AG	--	AG	AG	AG	GG	--	AG	AG	AA
--	GG	--	CG	CG	CG	GG	CG	GG	--	CG	CG	CG
CC	CT	CT	CT	CT	CT	CT	--	CC	--	CT	CT	TT
GG	GT	GT	GT	GT	GT	GT	GG	GG	GT	GT	GG	TT
GG	GT	GT	GT	GT	GT	GT	GG	GT	GT	GT	GG	TT
GT	GT	GT	GT	GT	GT	GT	GG	GT	--	GT	GG	GT
AG	AG	AG	AG	--	AG	AG	AG	AG	--	AG	GG	AG
--	CC	--	CT	CT	--	CC	--	--	--	CT	CC	CT
AC	AC	AC	AC	AA	CC	AC	AC	AC	AC	AC	CC	AC
AA	AA	AA	AA	AG	AA	AA	AG	AA	--	AG	AA	AG
CT	CT	--	CC	CT	CC	CT	CC	CT	--	CT	CC	CC
GG	AG	AG	AA	AG	AA	AG	AG	--	--	GG	AG	AG
--	TT	TT	TT	--	TT	TT	AT	TT	--	AT	AT	AT
CT	CT	CT	CC	CT	CC	CT	CC	CT	CT	CC	CC	CC
GT	TT	TT	TT	TT	TT	TT	GT	TT	--	GT	GT	GT
CG	CC	CC	CC	CC	CC	CC	CG	CC	--	CG	CG	CG
AT	AT	AT	TT	AT	TT	AT	AT	AT	--	--	AT	AT
CT	CT	CT	TT	--	TT	CT	CT	--	--	CC	CT	CT
AT	--	AT	AA	AT	AA	--	AT	AT	--	TT	AT	AA
AT	AT	AT	AA	--	AA	AT	AT	AT	--	TT	AT	AA
AG	AG	AG	AA	AG	AA	AG	AG	AG	--	GG	AG	AA
TT	TT	TT	TT	--	TT	TT	GT	TT	GT	GT	GT	GT
GT	GT	GT	TT	GT	TT	GT	GT	GT	GG	GG	GT	GT
AT	AT	AT	TT	AT	TT	AT	AT	AT	AA	AA	AT	AT
CT	CT	CT	TT	CT	TT	CT	CT	CT	--	CC	CT	TT
CG	CG	CG	CC	CG	CC	CG	CG	CG	--	GG	CG	CC
CT	CT	CT	CC	CT	CC	CT	CT	CT	TT	TT	CT	CC
AC	AC	AC	AA	AC	AA	AC	AC	AC	CC	CC	AC	AA
--	AG	AG	AA	AG	AA	AG	AG	AG	--	GG	AG	AA
GT	GT	GT	GG	--	GG	GT	GT	GT	--	TT	GT	GG
--	AG	AG	AA	AG	AA	AG	AG	AG	--	GG	AG	AA
GT	GT	GT	TT	GT	TT	--	GT	GT	--	GG	GT	TT
AG	AG	AG	GG	AG	GG	AG	AG	AG	AA	AA	AG	GG
CT	CT	CT	CC	CT	CC	CT	CT	CT	TT	TT	CT	CC
AT	AT	AT	AA	AT	AA	AT	AT	AT	--	TT	AT	AA
AG	AG	AG	AA	AG	AA	AG	AG	AG	--	GG	AG	AA
AC	AC	AC	AA	AC	AA	AC	AC	AC	--	CC	AC	AA
AG	AG	--	AA	AG	AA	AG	AG	AG	--	GG	AG	AA

--	CT	CT	TT	--	TT	CT	CT	CT	--	CC	CT	TT
--	CG	--	CC	CG	--	CG	CG	CG	--	GG	CG	CC
CT	CT	CT	TT	CT	TT	CT	TT	CT	--	CC	CT	CT
CT	CT	TT	CC	CT	CC	CT	CC	CT	CT	TT	CT	CT
AC	AC	CC	AA	AC	AA	AC	AA	AC	AC	CC	AC	AC
CT	CT	TT	CC	CT	CC	CT	CC	--	CT	TT	CT	CT
CC	CT	CC	TT	--	TT	CT	CT	CT	--	CC	CT	CT
AG	AG	GG	AA	GG	AG	GG	GG	AG	--	GG	AA	AG
GT	GT	GG	TT	GG	GT	GG	GG	GT	GG	GG	TT	GT
--	AG	GG	AA	--	AG	GG	--	GG	--	GG	AA	AG
CT	CT	TT	CC	CT	CT	TT	TT	TT	--	TT	CT	CT
CG	CG	CC	GG	CG	CG	CC	CC	CC	--	CC	CG	CG
AG	AG	AA	GG	AG	AG	AA	AA	AA	--	AA	GG	AG
AC	AC	CC	AA	--	AC	CC	--	CC	--	CC	AA	AC
GG	GG	AG	GG	--	GG	AG	AG	AG	--	AG	AG	GG
CT	CT	CT	CT	CT	CT	TT	TT	TT	TT	TT	CT	CT
CT	CT	CT	CT	--	CT	CC	CC	CC	CC	CC	CT	CT
TT	CT	CT	CT	CT	CT	TT	TT	TT	--	TT	CT	CT
--	AG	AG	AG	AG	AG	AA	AA	AA	--	AA	AG	AG
AA	AC	AC	AC	AC	CC	AA	AA	AA	--	AA	AC	AC
AA	AG	AG	AG	AG	GG	AA	AA	AA	--	AA	AG	AG
GG	CG	CG	CG	CG	CC	GG	GG	GG	--	GG	CG	CC
CC	CG	CG	CG	CG	GG	CC	CC	CC	CG	CC	CG	GG
AA	AG	--	AG	AG	GG	AA	AA	--	--	AA	AG	GG
--	AC	AC	AC	CC	AC	CC	CC	CC	AC	AC	AC	AA
--	CT	CT	CT	--	CT	TT	TT	TT	--	CT	TT	CC
CT	CC	CT	CT	CC	CT	CC	CC	CC	--	CT	CC	TT
AT	AT	AT	AA	TT	AT	AT	TT	AT	--	AT	TT	AT
--	AG	GG	AG	GG	GG	AG	GG	GG	AG	GG	GG	GG
--	TT	--	AT	--	AT	TT	AT	AT	AT	AT	TT	TT
GT	GT	--	GG	GG	GG	GT	TT	--	--	GG	GT	GT
AG	AG	AG	AG	AG	AG	AG	GG	AG	--	AG	GG	AG
CT	CT	CT	CT	CT	CT	CT	TT	CT	CT	CT	TT	CT
--	AG	AG	AG	--	AG	GG	--	AG	AG	GG	GG	AG
GT	GT	TT	TT	TT	TT	GG	GG	GT	--	GT	GT	GT
GT	GT	GT	GT	GT	--	--	GG	GT	--	--	GG	GT
CT	TT	CC	CC	CC	CT	TT	TT	CT	--	CT	CT	CT
CT	CT	--	CC	--	CT	TT	--	TT	--	CT	CC	CT
AG	AG	GG	AG	GG	AG	AA	AA	AA	GG	AG	GG	AG
CG	CG	CC	CG	CG	--	GG	--	--	--	CG	CG	CG
--	AG	GG	AA	AA	AG	AA	AG	AA	AA	AG	AG	AA
CT	CT	TT	CC	CC	CT	CC	CT	CC	--	CT	CT	CC
CT	CT	CT	CC	--	CC	CC	CT	CC	--	CT	CT	CC
CC	CC	CC	CC	CC	CG	CC	CC	CC	CG	CC	CG	CC
--	TT	TT	TT	--	AT	--	AT	TT	AT	TT	AT	TT
GG	GT	GT	GG	GG	GG	GG	GT	GG	--	GT	GG	GG
CC	CT	CT	CC	CC	CC	CC	CT	CC	CC	CT	CC	CC

CC	CT	CT	CC	--	CC	CC	TT	CT	--	TT	CT	CC
GG	GT	GT	GG	--	GG	GG	GT	GG	--	GT	GG	GG
CT	CT	CT	CC	CT	CC	CC	TT	CT	--	TT	CT	CC
TT	GT	GT	TT	TT	TT	TT	GT	TT	TT	GT	TT	TT
--	CG	CG	GG	GG	--	GG	CG	GG	--	CG	--	GG
--	AG	AG	AG	AG	AG	--	AG	AG	--	AG	AA	AA
AA	AG	AG	AG	AG	AG	GG	AG	AG	--	AG	GG	GG
--	AG	AG	AG	AG	AG	GG	AG	AG	--	GG	GG	GG
TT	CT	CT	CT	CT	CT	CC	CT	CT	--	CC	CC	CC
--	AG	AG	AG	AG	AG	AA	AG	AG	--	AA	AA	AG
GG	GT	GT	GT	--	GT	TT	GT	GT	--	TT	GT	TT
AG	GG	GG	GG	AG	GG	GG	AG	AG	AG	GG	AG	GG
--	CT	CT	CT	--	TT	CC	CT	CT	--	CC	CT	CC
--	CC	CC	CC	AC	CC	AC	--	AC	--	AC	AC	AC
CT	CT	CT	CT	CC	CT	CC	CC	CC	CT	CC	CC	CC
--	GT	GG	GT	--	GG	TT	GT	GT	--	GT	GT	TT
CC	CT	CC	CT	CC	CC	CT	CC	CC	CC	CC	CC	CT
GG	GG	GG	GG	CG	GG	CG	CG	CG	--	CG	CG	CG
--	CC	--	CC	--	CC	AC	--	AC	--	AC	AC	AC
TT	CT	TT	CT	--	--	CT	TT	TT	--	TT	TT	CT
AA	AG	AA	AG	--	AA	AG	AA	AA	AA	AA	AA	AG
AA	AG	AA	AG	AG	AA	GG	AG	AG	--	AG	AG	GG
CT	CT	CT	CT	CC	CT	CC	--	CC	--	CC	CC	CC
--	AA	AA	AA	AT	AA	AT	AT	AT	--	AT	AT	AT
CC	CC	CC	CC	CT	CC	CT	CT	CT	--	CT	CT	CT
AA	AA	AA	AA	--	AA	AG	AG	AG	--	AG	AG	AG
--	CT	CC	CT	--	CC	CT	CC	CC	--	CC	CC	CT
--	AT	TT	AT	AT	--	--	--	AT	--	AT	AT	AA
--	AT	--	AT	AT	TT	--	AT	AT	--	AT	AT	AA
TT	CT	TT	CT	CT	TT	CC	CT	CT	--	CT	CT	CC
CC	CC	CC	CC	CT	CC	CT	CT	CT	--	CT	CT	CT
GT	GT	GG	GT	--	--	GT	GG	--	--	GG	--	GT
CT	CT	--	CT	--	CC	CT	CC	CC	--	CC	CC	CT
--	AA	AG	AA	--	AG	AA	AG	AG	AG	--	AG	AA
--	AG	GG	AG	AG	GG	AA	AG	AG	--	AG	AG	AA
--	AG	AA	AG	AA	AA	AG	AA	AA	--	AA	AA	AG
--	CT	CC	CT	CT	CC	TT	CT	CT	--	CT	CT	TT
AC	AC	CC	AC	--	CC	AC	CC	--	--	CC	--	AC
--	AT	TT	AT	AT	TT	--	AT	AT	--	AT	AT	AA
AA	AA	AA	AA	AG	AA	AG	AG	AG	--	--	AG	AG
AC	AC	AA	AC	AA	AA	AC	AA	AA	--	AA	AA	AC
AT	AT	--	AT	--	TT	AT	TT	TT	TT	TT	TT	AT
AA	AA	AC	AA	CC	AC	AC	CC	CC	AC	CC	CC	AC
CC	CC	CC	CC	AC	CC	AC	AC	AC	CC	AC	AC	AC
--	GG	AG	GG	AG	AG	GG	AG	AG	--	AG	AG	GG
AC	AC	AC	AC	CC	AC	CC	CC	CC	AC	CC	CC	CC
AG	AG	AA	AG	AA	AA	AG	AA	AA	--	AA	AA	AG

CC	CC	CC	CC	CG	CC	CG	CG	CG	CG	CG	CG	CG
CT	CT	TT	CT	CT	TT	CC	CT	CT	--	CT	CT	CC
CT	CT	CC	CT	CT	CC	TT	CT	CT	--	CT	CT	TT
CT	--	CC	CT	--	CC	CT	CC	--	--	--	CC	CC
AG	AG	AA	AG	--	--	AG	AA	AA	--	--	AA	AA
CT	CT	CC	CT	CT	CC	TT	CT	CT	CC	CT	CT	CT
CT	CT	TT	CT	--	TT	CT	TT	TT	--	TT	TT	TT
--	AT	AA	AT	AT	AA	--	AT	AT	--	AT	AT	AT
GG	GG	GG	GG	AG	GG	AG	AG	AG	--	AG	AG	AG
AC	AC	AA	AC	AA	AA	AC	AA	AA	--	AA	AA	AA
--	CT	--	CT	--	CC	CT	CC	--	--	CC	CC	CC
TT	TT	TT	TT	GT	TT	GT	GT	GT	--	GT	GT	GT
--	CT	--	CT	TT	--	TT	TT	TT	--	TT	TT	TT
CC	CC	AC	CC	AC	AC	CC	AC	AC	AC	AC	AC	AC
AG	AG	AA	AG	AA	AA	AG	AA	--	--	AA	AA	AA
GT	GT	GG	GT	--	--	GT	--	GG	--	GG	GG	GG
--	TT	--	TT	AT	--	TT	AT	AT	--	AT	AT	AT
AG	AG	AA	AG	--	AA	AG	AA	AA	--	AA	AA	AA
--	CC	CC	CC	CT	--	CT	CT	CT	--	CT	CT	CT
--	CT	--	CT	CC	CC	CT	CC	CC	--	CC	CC	CC
AC	AC	AA	AC	--	AA	AC	AA	AA	--	AA	AA	AA
CT	CT	CC	CT	CC	CC	CT	CC	CC	--	CC	CC	CC
AT	AT	AA	AT	AT	AA	TT	AT	AT	--	AT	AT	AT
AG	AG	GG	AG	AG	GG	AA	AG	AG	GG	AG	AG	AG
--	GG	GG	GG	CG	GG	CG	CG	CG	--	CG	CG	CG
CT	CT	--	CT	--	TT	CT	TT	TT	TT	TT	TT	TT
AG	AG	GG	AG	GG	GG	AG	--	GG	--	GG	GG	GG
AG	AG	AG	AG	--	AA	AG	AA	AA	--	AA	AA	AA
AA	AA	AA	AA	AG	AA	AG	AA	AG	--	AG	AG	AG
GG	GG	GG	GG	AG	GG	AG	GG	AG	--	GG	AG	AG
CT	--	--	CT	--	TT	--	TT	TT	--	--	TT	--
GT	GT	GT	GT	--	GG	GT	GG	GT	--	GG	GG	GT
TT	TT	TT	TT	--	TT	TT	TT	GT	--	TT	GT	GT
--	GG	GG	AG	--	GG	AG	GG	AG	--	GG	GG	AG
CT	CC	CC	CT	CC	CC	CT	CT	CT	CT	CC	CC	CC
GT	TT	TT	GT	TT	TT	GT	GT	GT	GT	TT	TT	TT
--	AG	AG	AG	GG	AG	GG	--	GG	--	GG	GG	GG
GG	AG	AG	GG	AG	GG	AG	AG	AG	AG	AA	AG	AA
--	CT	CT	CC	CT	CC	CT	CC	CC	CC	CT	CC	CC
--	AT	AA	AT	--	--	AT	AA	AA	--	AA	AA	AA
GG	CG	CG	GG	CG	GG	CG	CG	CG	--	CG	CG	CG
--	AA	AC	AC	AA	AC	AA	AC	AC	AA	AA	AC	AC
GG	AG	AG	GG	AG	GG	AG	AG	AG	--	AG	AG	AG
GG	GG	AG	GG	GG	GG	GG	AG	AG	GG	GG	AG	AG
CC	CC	CT	CC	CC	CC	CC	CT	--	--	CC	CT	CT
AA	AG	AG	AA	AA	AA	AA	AG	AG	--	AG	AG	AG
CT	CC	CT	CT	CT	CT	CT	CT	CT	CT	CC	CT	CT

AG	AG	GG	AG	AG	AG	AG	GG	GG	AG	AG	GG	GG
--	AA	GG	GG	AG	AG	--	AG	GG	--	AA	AG	GG
TT	CT	CT	CT	TT	TT	TT	CC	CT	CT	CT	CC	CC
TT	AT	AT	AT	TT	TT	TT	AA	AT	--	AT	AA	AA
--	AC	AC	AC	CC	CC	AC	AA	AA	AC	AC	AA	AA
CC	CT	CT	CT	--	CT	CT	TT	TT	CT	CT	TT	TT
--	--	AG	AG	AG	AG	GG	GG	GG	--	AG	GG	GG
CT	CT	TT	CT	--	CC	CC	CT	CC	--	CT	CC	CC
CT	TT	CT	CT	CT	--	TT	TT	TT	--	CT	TT	TT
--	AG	GG	AG	--	AA	AA	--	AA	AG	GG	AA	AA
AG	AG	GG	GG	--	--	AG	AG	AG	--	AA	AG	AG
--	CT	TT	TT	--	CT	CT	--	CT	CT	CC	CT	CT
AG	AG	AG	AG	--	--	AG	--	--	--	GG	GG	GG
CT	CT	TT	TT	--	CT	CT	CT	--	--	--	CT	CT
--	GG	CG	CG	CG	GG	--	CG	CG	--	GG	CG	CG
--	CT	TT	TT	--	CT	--	CT	CT	--	CT	CT	--
CT	CT	CT	CT	CT	CT	CT	CC	CC	CC	CT	CC	CC
AG	AG	--	AA	AA	AG	AG	AG	AG	--	AG	AG	AG
CT	CT	TT	TT	TT	CT	CT	CT	CT	--	CT	CT	CT
AG	AG	GG	GG	GG	AG	AG	AG	AG	AA	AG	AG	AG
CT	CT	TT	CT	TT	CT	CT	TT	CT	--	CT	TT	CT
AG	AG	AA	AG	AA	AG	GG	AA	AA	--	AG	AA	AG
--	AG	AG	GG	AG	AG	GG	AG	AG	--	AG	AG	GG
GG	GT	GG	GT	GT	GT	TT	GT	GG	--	GT	GG	GT
CG	CG	--	CC	--	CG	CC	CG	CG	CC	CG	CG	CC
CC	CG	CG	CC	--	CG	CG	CG	CC	--	CG	CC	CC
--	CT	TT	CT	--	CT	CC	CT	TT	CT	CT	TT	CT
CC	CT	CC	CT	--	CT	TT	CT	CC	CT	CT	CC	CT
TT	AT	TT	AT	--	AT	AA	AT	TT	--	AT	TT	AT
AA	AG	AA	AA	GG	AG	AG	AG	AA	--	GG	AA	AG
TT	CT	TT	TT	CC	CT	CT	--	CT	--	CC	TT	CT
CT	CT	TT	CT	CT	CT	CT	CT	TT	--	TT	CT	CT
--	AC	CC	CC	AC	--	AC	AC	AC	AC	AA	CC	AC
GT	TT	--	GT	--	TT	GT	TT	--	--	GG	GT	GT
--	AA	AG	AG	AG	--	AG	--	--	--	GG	AG	AG
CT	TT	CT	CT	CT	--	--	TT	TT	--	CC	CT	CT
AT	AA	AT	AT	AT	AA	AT	--	AA	--	TT	AT	AT
--	CC	AC	AC	AC	--	AC	CC	CC	--	AA	AC	AC
CC	CG	CG	CG	CG	CG	CC	CG	CG	CG	CC	CC	CG
AG	AG	--	AG	AG	AG	AA	AG	AG	AG	AA	AA	AG
--	CC	CT	CT	CT	--	CT	CC	CC	--	TT	CT	CT
TT	CC	CT	CT	CT	CC	CT	--	--	--	TT	CT	CT
AG	AA	AG	AG	AG	AA	AG	AA	AA	--	GG	AG	AG
CC	CT	--	CC	--	CT	CT	CT	CT	CT	CC	CT	CC
--	AA	AG	AG	AG	AA	AG	AA	AA	--	GG	AG	AG
--	GG	--	AG	AG	GG	AG	--	GG	--	--	AG	AG
--	GG	GT	GT	GT	GG	GT	GG	GG	--	--	GT	GT

CT	TT	CT	CT	CT	TT	CT	TT	TT	--	CC	CT	CT
CG	GG	CG	CG	CG	GG	CG	GG	GG	GG	CC	CG	CG
GT	TT	GT	GT	GT	TT	GT	TT	GT	--	GG	GT	GT
AG	AA	AG	AG	AG	AA	AG	AA	AG	--	GG	AG	AG
CT	CC	CT	CT	--	CC	CT	CC	CT	--	TT	CT	CT
TT	GT	TT	TT	TT	GT	GT	--	GT	--	TT	GT	TT
--	GG	--	CG	CG	GG	CG	--	--	--	CC	CG	CG
AG	AA	AG	AG	AG	AA	AG	AA	AG	--	GG	AG	AG
GG	AG	GG	GG	GG	--	AG	AG	AG	--	GG	AG	GG
AC	AA	AA	AC	AC	AA	AA	AA	AC	AA	CC	AC	AC
CC	CG	CG	CC	CC	CG	CG	CG	CG	CG	CC	CG	CC
AG	GG	GG	AA	--	GG	GG	GG	AG	--	AA	AG	AG
--	AA	AA	CC	AC	--	AA	AA	AC	--	CC	AC	AC
GT	TT	TT	GG	GT	TT	TT	TT	GT	--	GG	GT	GT
--	CC	CC	AA	AC	CC	CC	CC	AC	--	AA	AC	AC
CC	CT	CT	CC	--	--	CT	CT	CT	CT	CC	CT	CC
GT	TT	GT	GG	GT	TT	TT	TT	--	GT	GG	GT	GT
AG	AG	AG	GG	AG	AG	AG	AG	--	AG	GG	GG	AG
--	CC	--	TT	CT	CC	CC	--	CT	--	TT	CT	CT
CC	CT	CT	CC	--	CT	CT	CT	CC	CT	CC	CT	CC
AT	AA	AA	TT	AT	--	AA	--	TT	--	--	AT	AT
CG	CC	CC	GG	GG	CC	CC	CC	GG	--	GG	CG	CG
GT	GG	GG	TT	--	GG	GG	GG	TT	--	TT	GT	GG
AG	AG	AG	GG	AG	AG	AG	AG	GG	AG	GG	AG	AG
TT	TT	TT	GT	GT	TT	--	GT	GT	--	GT	TT	TT
CT	CC	CC	TT	CT	CC	CC	CT	TT	--	TT	CT	CC
AC	CC	CC	AA	CC	CC	CC	AC	AA	CC	AC	AC	CC
--	CC	CC	CT	--	CC	CC	CC	CT	--	CC	CC	--
--	CG	--	CG	--	GG	GG	CG	CC	--	CG	CG	GG
CC	CT	CT	CT	--	CC	CC	CT	TT	--	CT	CT	CC
GT	GT	GT	TT	--	GT	GT	GT	TT	GT	GT	GT	GT
CT	TT	CT	CT	CT	CT	CT	TT	CT	CT	TT	TT	CT
AT	--	--	TT	TT	AT	AT	--	TT	AT	AT	AT	AT
CC	CC	CC	AC	AC	CC	CC	CC	AC	CC	CC	CC	CC
TT	CT	CT	CC	CC	TT	CT	CT	CT	TT	TT	CT	CT
AA	AA	AA	AG	AG	AA	AA	--	AG	--	AA	AA	AA
GG	GG	GG	AG	--	GG	GG	GG	AG	GG	GG	GG	GG
--	--	--	CG	--	--	GG	CG	GG	--	GG	CG	--
--	CT	--	CT	CC	--	CC	TT	CT	--	CT	TT	CT
CC	CT	CT	CT	CC	CT	CT	CT	CT	--	TT	TT	CT
CC	CC	CT	CT	--	CT	CT	CT	CT	CT	TT	TT	CT
--	TT	CT	CT	TT	--	CT	CT	CT	--	CC	CC	CT
GG	GG	GT	GT	GT	GT	GT	GT	GT	GT	TT	TT	GT
TT	TT	CT	CT	CT	CT	CT	CT	CT	--	CC	CC	CT
TT	TT	AT	AT	--	AT	AT	--	AT	--	AA	AA	AT
GG	GG	--	AG	--	AG	AG	AG	--	--	AA	AA	AG
--	AA	AC	AC	AC	CC	AC	AC	AC	AC	CC	CC	AC

--	GG	--	AG	--	AA	AG	AG	AG	--	AA	AA	AG
AG	GG	AG	AG	--	AA	AG	AG	AA	AG	AA	AA	AG
CT	CC	CT	CT	CT	TT	CT	CT	TT	CT	TT	TT	CT
AG	AA	AG	AG	AG	GG	AG	AG	GG	--	GG	GG	AG
GT	TT	GT	GT	GT	--	GT	GT	GG	--	--	--	GT
--	CC	--	CT	CT	TT	CT	--	TT	--	TT	TT	CT
AG	AA	AG	AG	--	--	AG	--	GG	--	GG	GG	AG
--	AA	AG	AG	GG	--	AG	AG	GG	--	GG	GG	AG
--	GG	--	AG	--	AA	--	AG	AA	--	AA	AA	AG
CG	CC	CG	CG	CG	GG	CG	CG	GG	CG	--	GG	CG
AG	GG	AG	AG	AG	AA	AG	--	AA	AG	AA	AA	AG
AC	CC	AC	AC	AC	AA	AC	AC	AA	AC	AA	AA	AC
--	AA	AG	AG	AG	GG	AG	AG	GG	--	GG	GG	AG
AG	AA	AG	AG	AG	GG	AG	AG	GG	--	GG	GG	AG
AT	TT	AT	AT	AT	AA	AT	AT	AA	--	AA	AA	AT
CT	CC	CT	CT	CT	TT	CT	CT	TT	TT	TT	TT	CT
CT	CC	CT	CT	CT	TT	--	CT	TT	--	TT	TT	CT
--	GG	CG	CG	CG	--	CG	CG	CC	--	CC	CC	CG
CT	TT	CT	CT	CT	CC	CT	CT	CC	--	CC	CC	CT
CT	CC	CT	CT	CT	TT	CT	CT	TT	--	TT	TT	CT
--	TT	CT	--	CT	CC	CT	CT	CC	--	CC	CC	CT
AT	TT	AT	AT	AT	AA	AT	AT	AA	--	AA	AA	AT
AT	AA	AT	AT	AT	TT	AT	AT	TT	--	TT	TT	AT
CT	TT	CT	CT	CT	CC	CT	CT	CC	CC	CC	CC	CT
CT	CC	CT	CT	CT	TT	CT	CT	TT	--	TT	TT	CT
AT	AA	AT	AT	AA	TT	AT	AT	TT	--	TT	TT	AT
AG	GG	AG	AG	GG	AA	AG	AG	AA	--	AA	AA	AG
--	GG	GT	GT	--	TT	GT	GT	TT	--	TT	TT	GT
--	TT	CT	CT	--	CC	--	CT	CC	--	CC	CC	CT
GG	CC	CG	CG	CC	GG	CG	CG	GG	GG	GG	GG	CG
AA	GG	AG	AG	GG	AA	AG	AG	AA	--	AA	AA	AG
--	TT	CT	CT	TT	CC	CT	CT	--	--	CC	CC	CT
CC	TT	CT	CT	--	CC	CC	CT	CC	--	CC	CC	CT
AA	TT	AT	AT	TT	AA	AA	AT	AA	--	AA	AA	AT
GG	AA	AG	AG	--	GG	GG	AG	GG	GG	GG	GG	--
GG	AG	AG	AG	AA	GG	GG	AG	GG	--	GG	AG	AG
GG	GT	GT	GT	TT	GG	GG	GT	GG	GG	GG	TT	GT
--	AG	GG	AG	--	AG	GG	AG	AG	--	GG	AA	AG
AG	GG	AG	AG	GG	GG	AG	--	AG	--	AG	GG	AG
TT	TT	TT	CT	TT	TT	TT	CT	TT	CT	CT	TT	TT
--	GG	--	AG	--	AG	AG	AG	AG	--	AA	GG	--
--	GG	GG	GG	GG	AG	AG	GG	AG	--	AG	GG	AG
--	GG	--	AG	GG	AG	AG	--	AG	--	--	GG	AG
CC	CC	CC	CG	CC	CG	CG	CG	CG	--	GG	CC	CG
TT	TT	TT	CT	TT	CT	CT	CT	CT	--	CC	TT	CT
AC	AC	AC	CC	AC	AC	AC	CC	AC	--	CC	AC	AC
--	AA	--	AG	AG	--	AG	AG	AA	--	AA	AG	AA

AC	AC	AC	AC	--	AC	AC	CC	AC	--	CC	AC	CC
--	AC	AC	CC	--	AC	CC	CC	AC	--	AC	CC	AC
CT	CT	CT	CT	CT	CT	CT	TT	CT	TT	TT	CT	CT
GG	AG	AG	GG	GG	AG	GG	AG	AG	--	AG	GG	AG
AA	AG	AG	AA	AA	AG	AA	--	AG	--	AG	AA	AG
CC	CT	CT	CC	CC	CT	CC	CT	CC	--	CT	CC	CT
--	--	CT	CT	--	CT	TT	--	TT	--	--	TT	CT
--	CG	GG	CG	--	CG	GG	CG	GG	--	CG	GG	CG
AG	AG	GG	AG	GG	AG	GG	AG	GG	AA	AG	GG	AG
--	CT	--	CT	CC	CT	--	--	CC	--	CT	--	CT
AT	AT	--	AT	AT	AT	AA	--	AA	--	AA	AA	AT
AC	AC	AA	AC	AA	AC	AA	AA	AA	--	AC	AA	AC
AG	AG	--	AG	--	AG	AG	AG	AG	--	--	GG	AG
--	CC	--	CT	CT	CT	CT	CT	CT	--	CC	CC	CT
--	CC	--	CT	CT	CT	CT	CT	CT	--	CC	CC	CT
--	GT	GT	--	GT	--	GT	GT	--	--	GG	GG	GT
CC	CT	CT	CT	CC	TT	CT	TT	CC	--	CT	TT	CT
AG	GG	GG	GG	--	GG	GG	--	AG	--	AG	GG	AG
AA	AG	AG	AG	AG	AG	AG	GG	AA	--	AG	GG	AG
GG	CG	CG	GG	--	GG	--	CG	GG	--	CG	CG	CG
--	AG	GG	GG	AG	AG	AG	AG	AG	--	AG	GG	AG
GG	AG	AA	AA	AG	AG	AG	AG	AG	--	AG	AA	AG
TT	TT	TT	TT	TT	GT	GT	GT	TT	--	TT	TT	TT
--	AG	AA	AA	AG	AG	AG	AG	--	AG	AG	AA	AG
AG	AG	--	AA	AG	AG	AA	--	AG	--	AG	AA	AG
--	GG	GG	GG	GG	GT	GG	GT	GT	--	GG	GG	GT
CT	CC	CC	CC	--	--	CC	CT	TT	--	CT	CC	--
--	TT	TT	TT	CT	CT	TT	CT	CC	--	CT	TT	CC
CT	CC	CC	CC	CT	CC	CC	CC	CT	CT	CT	CC	CT
GG	AA	AG	AA	AG	AG	AA	AG	GG	GG	AG	AA	GG
TT	CC	CT	CC	CT	CT	CC	CT	TT	TT	CT	CC	TT
GG	AA	AA	AA	AG	AG	AA	AA	GG	--	AG	AA	AG
--	CT	--	CC	CT	--	CC	--	CT	--	CT	CC	CT
--	CG	GG	GG	CG	CG	GG	GG	CG	--	CG	GG	CG
TT	CT	CC	TT	CT	CT	CC	CT	CT	--	CT	CC	CT
AC	AC	--	AA	--	--	--	AC	AC	--	AA	AC	AC
--	AG	--	GG	--	AA	--	--	AG	--	GG	AG	AG
--	CT	CC	TT	--	CC	TT	CT	CT	CT	TT	CT	CT
AG	AG	GG	AA	--	GG	AA	AG	GG	--	AA	AG	AG
AG	AG	GG	AA	--	GG	AA	AG	GG	--	AA	GG	AG
CT	CT	CT	TT	CT	CT	TT	TT	CT	CT	TT	CT	TT
--	AA	AA	GG	AA	AA	GG	--	AA	--	GG	AA	AG
AA	AG	AG	AA	AG	AG	AA	AA	AG	--	AA	AG	AG
TT	GT	GT	TT	GT	GT	TT	TT	GT	TT	TT	GT	GT
--	GT	GT	TT	GT	GT	TT	TT	GT	--	TT	GT	GT
AC	CC	--	AA	CC	CC	AA	AA	AC	--	AA	CC	AC
AG	GG	GG	AA	--	GG	AA	AA	--	--	GG	GG	AG

CG	GG	GG	CC	GG	--	--	--	CG	CG	GG	--	CG
CG	GG	CG	CC	GG	--	CC	CC	CG	--	GG	GG	CG
--	CC	CC	TT	CC	CC	TT	TT	CT	--	CC	CC	CT
CT	TT	TT	CT	TT	TT	CT	CC	CT	--	TT	TT	TT
AT	TT	TT	AT	TT	TT	TT	AA	AA	TT	TT	TT	TT
AG	GG	AG	GG	GG	GG	GG	AG	AG	--	GG	GG	GG
CT	TT	CT	TT	TT	TT	TT	CC	CC	--	TT	TT	--
CT	CC	CT	CC	--	CC	CC	TT	TT	CT	CC	CT	CC
--	TT	CT	TT	--	TT	TT	CC	CC	--	TT	CT	TT
AG	AA	AG	AA	AG	AA	AA	GG	GG	AG	AA	AG	AA
--	CG	CG	CG	CG	CG	CG	CC	CC	CG	CG	CG	CG
CG	CG	GG	CG	--	CG	CG	GG	GG	--	CG	GG	CG
GG	GT	GT	GT	--	GT	GT	GG	GG	GT	GT	GT	GT
CC	CC	CC	CG	CC	CG	CG	CG	CG	--	CC	CC	CC
--	AG	GG	AG	AG	AG	--	AA	AA	--	AG	AG	AG
AA	AA	AA	AA	AA	AA	AC	AC	--	--	AA	AA	AA
AA	AA	AA	AT	AA	AT	AT	AT	AT	--	AA	AA	AA
GG	AG	GG	AA	AG	AA	AG	AA	AA	--	AG	AG	AG
AA	AT	AA	TT	AT	TT	AT	TT	TT	--	AT	AT	AT
GG	AG	GG	AA	AG	AA	AG	AA	AA	GG	AG	AG	AG
--	AC	CC	AC	AC	AC	AC	AC	AC	CC	AC	AC	AC
CC	CT	CC	TT	CT	TT	TT	TT	CT	--	CT	CT	CT
--	GT	TT	GG	TT	GG	GT	GT	GT	--	GT	GT	GT
TT	GT	TT	GG	--	GG	GT	GT	TT	--	GT	GG	GT
--	CT	CC	TT	CC	TT	CT	CT	CC	CT	CT	--	CT
CC	GG	CC	GG	--	GG	CG	CG	CC	CG	CG	GG	CG
GG	AA	--	AA	--	AA	AG	--	--	AG	AG	AA	AG
CC	TT	CC	TT	--	TT	--	CT	CC	--	CT	TT	CT
TT	GG	TT	GG	TT	GG	GT	GT	TT	--	GT	GG	GT
TT	CC	TT	CC	TT	CC	CT	CT	TT	CT	CT	CC	CT
GG	CC	GG	CC	GG	CG	CG	CG	GG	--	CG	CC	CG
--	AA	GG	AA	GG	AG	AG	AG	GG	AG	AG	AA	AG
--	CC	AA	CC	--	AC	AC	--	AA	--	AC	CC	AC
CT	CC	TT	CC	--	CT	CT	CT	TT	CT	CT	CC	CT
AG	AG	GG	AG	--	AG	GG	AG	GG	--	GG	AG	GG
--	CC	AA	CC	AA	AC	CC	--	--	AC	AC	CC	AC
CT	TT	CC	TT	CC	CT	CT	CT	CC	CT	CT	TT	CT
AG	AA	GG	AA	GG	AG	AG	AG	GG	AG	AG	AA	AG
CG	CC	GG	CC	--	CG	CG	CG	GG	CG	CG	CC	CG
GT	TT	GG	TT	GG	--	GT	GT	GG	--	GT	TT	GT
CT	TT	CT	TT	--	CT	CT	CT	CC	CT	CT	TT	CT
CT	CT	TT	CT	TT	CT	TT	CT	TT	--	TT	CT	TT
--	GG	--	GG	GG	AG	GG	AG	GG	--	GG	GG	GG
GG	CG	CC	GG	GG	CG	CG	CG	--	--	CG	GG	CG
AT	AA	AA	AT	AT	--	AT	AA	AT	AT	AT	AT	AT
AG	AG	AA	AG	AG	AG	AA	AA	AA	--	AA	AG	AA
AC	AC	CC	AC	AC	AC	CC	CC	CC	CC	CC	AC	CC

CT	CT	CT	CT	CT	CT	TT	TT	TT	TT	TT	CT	TT
CT	TT	TT	CT	CT	--	CT	TT	CT	--	CT	TT	CT
AT	GT	AG	AT	AT	AT	AG	GG	AG	--	AG	GT	AG
AT	AT	AA	AT	AT	AT	AA	AA	AA	--	AA	AT	AA
AA	AG	GG	AA	AA	AA	AG	GG	AG	GG	AG	AG	AG
AC	AC	CC	AA	--	--	AC	--	AC	--	AC	AC	AC
AG	AG	AA	AG	AG	AG	AA	AA	AA	AA	AA	AG	AA
AG	AG	GG	AA	--	AA	AG	GG	AG	--	AA	AG	AG
AG	AG	AA	GG	--	GG	AG	AA	AG	--	AG	AG	AG
--	AG	GG	AA	--	AA	AG	AG	--	--	AG	AG	AG
GT	GT	GG	TT	--	TT	GG	--	GT	--	GT	GT	GT
--	GG	GG	AG	AG	AG	GG	AG	AG	--	AG	AG	AG
--	CT	CC	TT	TT	--	CC	CT	CT	--	CT	TT	CT
GG	CG	CC	GG	GG	CG	CC	CG	CG	--	CG	GG	CG
CT	CT	TT	CT	CT	CT	TT	TT	TT	TT	TT	CT	TT
CT	TT	TT	CT	CT	TT	TT	TT	TT	TT	CT	CT	CT
CT	CT	CT	CT	CT	CC	CC	CC	CT	CC	CC	CC	CT
GG	GT	GG	GT	GT	GT	GT	GT	GG	--	GT	GG	GG
AG	AG	AG	AA	--	AG	AG	AA	AG	--	AG	GG	AG
AG	AG	AG	GG	--	AA	--	AG	AG	AG	AG	AA	AG
--	AG	AG	AG	AG	AA	AA	AA	AG	--	AA	AA	AG
--	TT	--	TT	TT	GT	GT	GT	GT	--	GT	GT	TT
--	AG	AG	AG	--	AA	AA	--	AG	--	AA	AG	AG
CT	CT	CT	CT	--	--	CC	CC	--	--	CC	CT	CT
--	AA	--	AA	--	AG	AG	AG	AA	AG	AG	AA	AA
AG	AG	AA	AG	AA	AG	AG	AG	AA	GG	AG	AG	AA
--	AG	--	AG	--	AG	AG	AG	AA	--	AG	AG	AA
CG	CG	CC	CG	CC	CG	CG	CG	CC	--	CG	CG	CG
--	CG	--	CG	CC	CG	CG	GG	CC	--	GG	CG	CG
AG	AG	AG	AG	AA	AG	AG	GG	AA	GG	GG	AA	AG
--	GG	--	GG	--	AG	AG	GG	AG	AG	GG	AG	GG
--	CT	CT	CT	--	CT	CT	--	--	CT	TT	CC	CT
AC	CC	--	CC	AC	AC	AC	--	AC	AC	CC	--	CC
CG	CC	CC	CC	CG	CG	CG	CC	CG	--	CG	CG	CC
CC	CC	CC	CC	AC	AC	AC	CC	AC	AC	AC	AC	CC
--	AC	AA	AC	AC	AC	AC	AA	CC	--	AC	CC	AC
AG	AG	AA	AG	--	AA	AA	AA	AG	--	AA	AG	AG
GG	GG	AA	AG	AG	AG	AG	AA	GG	AG	AG	GG	AG
AG	AG	GG	AG	AG	GG	--	GG	AG	--	GG	AG	AG
TT	TT	CC	CT	CC	CT	CT	CT	TT	--	CT	TT	CT
--	TT	CC	CT	CC	CT	CT	CT	TT	CT	CT	TT	CT
--	AG	AA	AG	--	AG	AG	--	AG	AG	AG	AG	AA
CT	CT	CC	CT	CC	CT	CT	CT	--	CT	CT	CT	CC
AA	GG	AG	AG	AG	GG	AG	AG	AG	--	AG	AG	AA
AC	CC	CC	CC	CC	AC	CC	AC	AC	AC	AC	CC	AC
CT	CC	CT	CT	CT	CC	CT	CC	CC	CT	CC	CT	CT
--	AA	AG	AG	AG	AG	AG	AG	AG	--	--	AG	AG

AG	AA	AG	AG	AG	AA	AG	AG	AA	--	AA	AG	AG
GG	AG	AG	AG	AG	AG	AG	GG	AG	AG	AA	AG	AG
AA	AC	CC	AC	--	AC	AC	AA	AC	AC	CC	AC	AC
AA	AG	GG	AG	AG	AG	AG	AA	AG	AG	GG	AG	AA
--	GT	--	GG	GT	GT	--	GG	TT	--	--	GT	GG
--	AG	AA	AG	AA	AG	AG	AG	AA	--	AA	AA	AG
CC	CT	TT	CC	--	CT	CC	CC	TT	--	TT	CT	CC
CT	CT	CC	CT	CT	CT	CT	CT	CC	--	CC	CC	CT
--	AA	GG	AA	AG	AG	AA	AA	AG	--	GG	AG	AA
CG	CG	GG	CG	GG	GG	CG	CG	CG	--	GG	--	CG
CT	CT	--	CT	--	--	CT	CT	CT	--	TT	CT	CT
--	AG	AA	AG	AG	AG	GG	--	AG	AG	AA	AG	GG
AG	AG	AA	AG	AG	AG	GG	GG	AG	--	AA	AG	GG
CG	CG	CC	CG	CG	--	CG	GG	CG	CG	CC	CG	GG
--	AG	--	AG	AG	--	AG	AG	AG	--	AA	AG	GG
--	AG	--	GG	--	AA	AG	AG	AA	--	GG	AA	--
CC	CT	CT	TT	CC	CC	CT	CT	CC	CT	TT	CC	CT
--	AG	--	GG	--	AA	AG	AG	AA	--	GG	AA	AG
GG	CG	CG	CC	GG	GG	CG	CG	GG	--	CC	GG	CG
--	AC	AC	CC	AA	AA	--	AC	AA	--	CC	AA	AC
GG	AG	AG	AA	GG	GG	AG	AG	GG	AG	AA	GG	AG
TT	GT	--	GG	TT	TT	GT	--	TT	GT	GG	TT	GT
--	AG	AG	GG	--	AA	AG	AG	AA	--	GG	AA	AG
--	AG	AG	GG	AA	AA	AG	AG	AA	AG	GG	AA	AG
--	AG	AG	AA	--	--	AG	AG	GG	--	AA	GG	--
CC	AC	AC	AA	CC	CC	AC	AC	CC	AC	AA	CC	AC
--	AG	AG	AA	GG	GG	AG	AG	GG	AG	AA	GG	AG
GG	AG	AG	AA	GG	GG	AG	AG	GG	--	AA	GG	AG
CC	CT	CT	TT	CC	CC	CT	CT	CC	CT	TT	CC	CT
CC	CG	CG	GG	CC	CC	CG	CG	CC	CG	GG	CC	CG
CC	AC	--	AA	--	CC	AC	AC	CC	AC	AA	CC	AC
--	CT	CT	TT	--	CC	CT	CT	CC	CT	TT	CC	CT
--	GT	GT	TT	--	GG	GT	GT	GG	--	TT	GG	GT
TT	CT	CT	CC	--	TT	CT	CT	--	--	CC	CT	CT
--	CG	CG	CC	GG	GG	CG	CG	GG	CG	CC	CG	CG
--	AT	AT	TT	--	AA	AT	AT	AA	AT	TT	AT	AT
--	CT	CT	CC	TT	TT	CT	CT	TT	--	CC	CT	CT
AA	AG	AG	GG	--	AA	AG	AG	AA	AG	GG	AG	AG
GG	AG	AG	AA	--	GG	AG	AG	GG	AG	AA	AG	AG
TT	AT	AT	AA	--	TT	AT	AT	TT	--	AA	AT	AT
AA	AG	AG	GG	AA	AA	GG	AG	AA	--	GG	AG	AG
--	AG	AG	AA	--	GG	AA	AG	GG	AG	AA	AG	AG
CG	CG	CG	CC	--	GG	CC	CG	GG	CG	CC	CG	CG
GG	AG	AG	AA	GG	GG	AA	AG	GG	AG	AA	AG	AG
--	CG	CG	GG	--	--	GG	CG	CC	CG	GG	CG	--
--	AG	AG	GG	--	AA	GG	AG	AA	--	GG	AG	AG
CC	AC	AC	AA	CC	CC	AA	AC	CC	AC	AA	AC	AC

--	AG	AG	GG	--	--	--	--	AA	AG	GG	AG	AG
--	CG	CG	CC	--	GG	CC	CG	GG	CG	CC	CG	CG
--	CT	CT	CC	--	TT	--	CT	TT	--	CC	CT	CT
--	AT	AT	TT	AA	AA	TT	AT	AA	AT	TT	AT	AT
--	CT	CT	CC	--	--	CC	CT	TT	CT	CC	CT	CT
TT	AT	--	AA	TT	TT	AA	AT	TT	--	AA	AT	AT
--	AT	AT	TT	AA	AA	TT	AT	AA	AT	TT	AT	AT
CC	CG	CG	GG	CC	CC	GG	CG	CC	CG	GG	CG	CG
TT	CT	CT	CC	TT	TT	CC	CT	TT	--	CC	CT	CT
GG	AG	AG	AA	GG	GG	AA	AG	GG	AG	AA	AG	AG
CC	AC	AC	AA	CC	CC	AA	AC	CC	--	AA	AC	AC
GG	GT	GT	TT	GG	GG	TT	GT	GG	--	TT	GT	GT
TT	CT	CT	CC	TT	TT	CC	CT	TT	--	CC	CT	CT
CC	CG	CG	GG	CC	CC	--	CG	CC	CG	GG	CG	CG
--	GT	--	TT	GG	GG	TT	GT	GG	GT	TT	GT	GT
CC	CT	CT	TT	CC	CC	TT	CT	CC	CT	TT	CT	CT
--	AG	AG	AA	--	GG	AA	AG	--	AG	AA	AG	AG
--	AC	AC	AA	CC	--	--	AC	CC	--	AA	AC	AC
TT	CT	CT	CC	--	TT	CC	CT	TT	--	CC	CT	CT
--	AC	AC	AA	CC	CC	AA	AC	CC	--	AA	AC	AC
--	AG	AG	AA	GG	GG	AA	AG	GG	AG	AA	AG	AG
TT	GT	GT	GG	--	TT	GG	GT	TT	--	GG	GT	GT
GT	GT	GT	GG	--	TT	GG	GT	TT	--	GG	GT	GT
AA	AG	AG	GG	AA	AA	GG	AG	AA	AG	GG	AG	AG
--	CT	CT	CC	TT	TT	CC	CT	TT	CT	CC	CT	CT
TT	CT	--	CC	TT	TT	CC	CC	TT	--	CC	CT	CT
GG	AG	AG	AA	--	GG	AA	AG	GG	--	AA	AG	AG
--	CT	CT	CC	TT	TT	CC	CT	--	--	CC	CT	CT
CC	CT	CT	TT	CC	CC	TT	CT	CC	CT	TT	CT	CT
--	AG	AG	GG	AA	AA	GG	AG	AA	--	GG	AG	AG
TT	CT	CT	CC	TT	TT	CC	CT	TT	CT	CC	CT	CT
CT	CT	TT	TT	CT	CT	TT	TT	CT	--	TT	TT	TT
TT	CT	CC	CC	CT	TT	CC	CC	TT	--	CC	CT	CT
GG	AG	AA	AA	AG	GG	AA	AG	AG	--	AA	AG	AG
--	AC	AA	AA	AC	CC	AA	AC	AC	--	AA	AC	AC
--	CG	CG	CG	CG	CC	CG	CG	CG	CC	CG	CC	CC
--	GT	--	--	GG	--	GG	GT	GT	--	GG	GT	GG
--	AG	GG	AG	GG	--	GG	AG	AG	AG	GG	AA	GG
--	CT	TT	CT	--	CT	TT	CT	CT	--	TT	CC	TT
GG	AG	AG	GG	AG	AG	AG	AG	AG	--	AG	GG	AG
--	AG	GG	AG	GG	AG	GG	AA	AG	AG	GG	AA	GG
--	CG	--	CG	GG	CG	GG	CC	CG	CG	GG	CC	GG
--	CT	--	TT	CT	CT	CT	--	CT	--	CT	TT	CT
GG	GT	GT	GG	TT	GT	TT	GT	GT	--	TT	GG	GT
--	GG	AG	GG	--	AG	AG	GG	AG	--	AG	GG	GG
GG	AG	GG	AG	GG	GG	GG	GG	AG	GG	AG	GG	AG
AA	AC	CC	AC	--	AC	AC	AC	CC	--	CC	AA	AC

TT	AT	AA	AT	--	--	AT	AA	AT	--	AA	TT	TT
AT	AT	AA	AT	--	AT	AT	--	TT	TT	AA	TT	TT
--	AT	--	--	AA	AA	AT	TT	AA	--	TT	AA	AA
CT	CT	CC	CC	--	TT	CT	CC	TT	TT	CC	TT	TT
AG	AG	AA	AA	GG	GG	AG	AA	GG	GG	AG	GG	GG
AC	AC	CC	CC	AC	AA	AC	CC	AA	--	AC	AA	AA
--	CT	CC	CC	CT	CT	TT	--	--	--	CT	TT	TT
AG	AG	AA	AA	AG	--	GG	AA	--	--	AG	AG	GG
GT	GT	TT	TT	GT	GT	GG	TT	GG	--	GT	GT	GG
--	AG	--	AA	--	AG	GG	--	GG	--	AG	AG	GG
CC	CC	CG	CG	CC	CC	CC	CG	CC	--	CC	CC	CC
GG	GG	AG	AG	GG	GG	GG	AG	GG	GG	GG	GG	GG
CC	CG	CG	GG	CC	CG	CC	CG	CC	--	CG	CG	CC
--	AG	AG	GG	AA	AA	AA	AG	--	--	AG	AG	AG
GG	AG	AG	AG	--	GG	GG	AG	GG	--	AG	AG	AG
AA	AA	AG	AG	AA	AA	AA	AG	AA	--	AG	AG	AG
GG	GG	GT	GT	--	GG	GG	GT	GG	--	GG	GG	GG
GG	GG	AG	AG	GG	GG	GG	AG	AG	--	AG	AG	AG
TT	TT	GT	GT	--	TT	TT	GT	TT	--	TT	TT	TT
GG	GG	--	CG	--	GG	GG	CG	CG	--	CG	CG	CG
CT	TT	CT	CT	CT	TT	CT	--	CT	CT	TT	CT	CT
--	CG	CG	CC	CG	CG	--	CG	CG	--	CG	CG	CG
--	CG	CC	CG	--	CG	CC	CC	CC	--	CG	CC	CC
AA	AA	AC	AC	AC	AA	AC	AC	AC	AA	AA	AC	AA
--	CC	CT	CT	CT	CC	CT	CT	CT	--	CC	CT	CC
--	AA	AG	AG	AG	AA	AG	AG	AG	--	AA	AG	AA
CC	CT	CT	CT	CT	CC	CT	CT	CT	--	CC	CT	CC
GG	CG	GG	CG	GG	CG	GG	GG	GG	GG	CG	GG	GG
--	CT	--	CT	--	CC	CT	CT	CT	--	CC	CT	--
AA	GG	AA	GG	AG	AG	AG	AG	AG	AA	AG	AG	AA
GG	GT	GG	GT	GT	GG	GT	GT	GT	GG	GG	GT	GG
TT	GG	TT	GG	GT	GT	GT	GT	GT	--	GT	GT	TT
--	CG	GG	CG	CG	GG	--	--	CG	--	GG	CG	GG
GG	CC	GG	CC	CG	CG	CG	CG	CG	GG	CG	CG	GG
--	AG	GG	AG	AG	GG	AG	AG	AG	--	GG	AG	GG
--	CC	TT	CC	CT	CT	CT	CT	CT	--	CT	CT	TT
--	TT	GG	TT	GT	GT	GT	GT	GT	--	GT	GT	GG
GG	AG	GG	AG	AG	GG	AG	AG	AG	--	GG	AG	GG
AG	--	--	GG	AG	AG	AG	AG	AG	--	AG	AG	AA
--	CC	--	CC	CT	--	--	CT	--	--	TT	CT	TT
TT	AT	--	AT	AT	TT	--	AT	AT	TT	TT	AT	--
TT	GT	TT	GT	--	TT	GT	GT	GT	--	TT	GT	TT
--	TT	--	TT	--	CT	--	--	CT	--	CT	CT	CC
AA	AG	AA	AG	AG	AA	AA	AG	AG	--	AA	AG	AA
TT	AA	TT	AA	AT	AT	TT	AT	AT	--	AT	AT	TT
AA	AG	AA	AG	AG	AA	AA	AG	AG	--	AA	AG	AA
TT	CT	TT	CT	--	TT	TT	CT	CT	TT	TT	CT	TT

--	AT	TT	AT	AT	TT	TT	AT	AT	--	TT	AT	TT
--	TT	GG	TT	GT	GT	GG	GT	GT	--	GT	GT	GG
AA	GG	AA	GG	AG	AG	AA	AG	AG	--	AG	AG	AG
--	TT	CC	TT	CT	CT	CC	CT	CC	CC	CT	CT	CT
--	TT	CC	TT	CT	CT	CC	CT	CC	--	TT	CT	CT
--	CT	CT	TT	CT	CT	CC	CT	--	--	TT	CT	CT
GG	GT	GT	GT	GT	GT	GG	GT	GG	GG	TT	GT	GT
--	CG	CG	CG	CG	CG	CC	CG	CC	--	GG	CG	CG
CC	AC	--	AC	CC	AC	CC	AC	CC	--	AA	AC	AC
AA	AG	AG	AG	AA	AA	AA	AG	AA	AG	GG	AG	AG
--	AG	AG	AG	--	GG	--	AG	GG	AG	AA	AG	AG
--	TT	TT	TT	TT	CT	CC	CT	CC	CT	CT	CC	CC
AA	AA	AA	AA	AA	AC	CC	AC	CC	AC	AC	CC	CC
--	TT	TT	TT	TT	TT	GT	GT	GT	--	GT	GT	GT
--	CG	--	GG	GG	CG	CG	GG	CG	--	GG	CG	CG
--	AG	GG	GG	GG	GG	AA	AG	--	--	AG	AA	AA
TT	CT	TT	TT	TT	TT	CT	TT	CT	--	TT	CT	CT
GT	TT	GT	TT	GT	TT	TT	--	GT	--	GT	GT	TT
CC	CT	CC	CC	--	CC	CT	CT	CT	CC	CC	CT	CT
--	AC	AC	AA	AC	AA	AA	AA	AC	--	AC	AC	AA
GG	GG	AG	AA	AG	AA	AG	AG	GG	AA	AG	AG	AG
AG	AG	AG	AA	AG	AA	AA	AA	AA	--	AG	AG	AG
--	GG	--	TT	GT	--	GT	GT	GT	--	GT	GT	GT
--	TT	TT	CC	CC	--	CT	CT	CT	--	CT	CT	CT
CC	CC	AC	AA	AC	AA	AC	AC	AC	AA	AC	AC	AC
--	AA	AT	TT	AT	TT	AT	AT	AT	AT	TT	AT	AT
GT	GG	GG	--	--	GT	--	GT	--	--	GT	--	GG
--	AA	AG	AG	AA	AG	AA	--	AA	--	AG	AG	AG
CG	GG	CG	CC	CG	CC	GG	CG	CG	CG	CC	CG	CG
--	GG	--	AG	AG	--	GG	AG	AG	--	AA	AG	AG
--	GG	GG	CC	--	CG	CG	CG	--	--	CG	CC	CG
AA	GG	GG	AA	--	--	AG	AG	GG	AG	AG	AA	AG
--	AG	AG	AG	AG	AG	AA	--	AG	--	AG	AA	AG
--	AT	AT	AA	--	--	AT	--	AT	--	AA	AA	AA
--	AG	AG	AG	AG	--	--	AG	AG	--	AG	AG	AG
--	AA	AA	AA	--	AA	AG	AA	AA	AA	AA	AA	AA
--	CC	CC	CC	AC	AC	AC	AC	CC	--	AA	AC	AC
--	AC	AC	AC	--	CC	AC	--	AC	--	CC	CC	AC
--	AA	AA	AA	AC	--	AC	--	AA	--	CC	AC	AA
--	TT	TT	TT	GT	GT	GT	GT	GT	--	GG	TT	TT
AA	TT	TT	TT	AT	AT	AT	--	--	--	--	TT	TT
AA	AG	AG	AG	AA	AA	AG	AA	AG	AG	AA	AG	AG
CC	AC	AC	AC	CC	CC	AC	CC	CC	--	CC	AC	AC
AA	TT	TT	TT	--	AT	AT	AT	TT	--	AA	TT	TT
CT	CT	TT	TT	CC	CT	CT	CT	TT	--	--	TT	TT
AG	AG	AA	AA	--	AG	AG	AG	AA	--	GG	AA	AA
CT	CT	TT	TT	CC	CT	CT	CT	TT	--	CC	TT	TT

CT	CT	--	CC	--	--	--	CT	CC	--	TT	CC	CC
AG	AG	AA	AA	--	AA	AG	AG	AA	--	GG	AA	AA
CT	CT	TT	TT	CC	TT	TT	CT	TT	--	CC	TT	TT
--	CT	--	CC	--	CC	CC	CT	CC	--	TT	CC	CC
CT	CT	TT	TT	CC	TT	TT	CT	TT	--	CC	TT	TT
AG	AG	AA	AA	--	AA	AA	AG	--	--	GG	AA	AA
CT	CT	--	CC	TT	CC	CC	CT	CC	--	TT	CC	CC
AG	AG	AA	AA	GG	AA	--	--	AA	--	GG	AA	AA
CG	CG	GG	GG	CC	GG	GG	CG	GG	--	CC	GG	GG
AC	AC	CC	CC	--	CC	CC	AC	--	--	AA	CC	CC
CT	CT	CT	TT	CC	TT	TT	CT	TT	TT	CC	TT	TT
--	CT	CT	TT	CC	TT	TT	CT	TT	--	CC	TT	--
AT	AT	AT	AA	--	AA	AA	AT	AA	--	TT	AA	AA
CC	CT	CT	TT	CC	TT	--	CT	--	TT	CC	TT	TT
AC	AC	AC	CC	AA	CC	CC	AC	CC	--	AA	CC	CC
GT	GT	GT	GG	TT	GG	GG	GT	GG	--	TT	GG	GG
CT	CT	CT	CC	--	CC	CC	CT	CC	CC	TT	CC	CC
CG	CG	CG	CC	GG	CC	CC	CG	CC	--	GG	CC	CC
--	CG	CG	CC	GG	--	--	CG	--	--	GG	CC	--
AC	AC	AC	AA	CC	AA	AA	AC	AA	AA	CC	AA	--
CG	CG	CG	CC	GG	CC	CC	CG	CC	CG	GG	CC	CC
AT	AT	AA	AT	AT	AA	AT	--	TT	--	AT	AT	AT
AG	AG	AA	AG	AG	AA	AG	AA	--	AG	AG	AG	AG
--	GG	AA	AG	AG	--	AG	--	GG	AG	AG	GG	AA
AA	AA	GG	AG	--	GG	--	AG	AA	AG	AG	AA	AG
--	GG	AA	AG	AA	AA	AG	AG	AG	AG	AG	GG	AG
--	CC	--	AC	AA	AA	AC	AC	AC	AC	AC	CC	AC
--	GG	AA	AG	--	AA	AG	AG	AG	AG	AG	GG	AG
GG	GG	AA	AG	AA	AA	AG	AG	AG	AG	AG	GG	AG
--	TT	GG	GT	--	GG	GT	GT	--	GT	GT	TT	GT
--	GG	TT	GT	TT	--	GT	GT	GT	--	GT	--	GT
AA	AA	CC	AC	CC	CC	AC	AC	AC	AC	AC	AA	AC
--	CC	TT	CT	TT	--	CT	CT	CT	--	CT	CC	CT
CC	CC	--	CT	--	TT	CT	CT	CT	CT	CT	CC	CT
TT	TT	--	GT	GG	GG	GT	GT	GT	GT	GT	TT	GT
--	TT	--	AT	AA	AA	AT	AT	AT	AT	AT	TT	AT
CC	CC	GG	CG	GG	GG	CG	CG	CG	CG	CG	CC	CG
--	CC	--	AC	--	--	--	AC	AC	--	AC	CC	AC
--	TT	CC	CT	CC	CC	CT	CT	CT	CT	CT	TT	CT
CC	CC	GG	CG	GG	GG	CG	CG	CG	--	CG	CC	CG
TT	TT	--	CT	--	CC	CT	CT	CT	--	CT	TT	CT
TT	TT	AA	AT	AA	AA	AT	AT	AT	--	AT	TT	AT
--	CC	TT	CT	--	--	CT	CT	CT	--	CT	CC	CT
--	AA	GG	AG	--	GG	AG	AG	--	--	AG	AA	AG
TT	TT	--	AT	AA	AA	AT	AT	AT	--	AT	TT	AT
--	TT	AA	AT	--	AA	TT	AT	AT	AT	AT	TT	AT
CC	CC	AA	AC	--	AA	AC	AC	AC	--	AC	CC	AC

GG	GG	TT	GT	TT	TT	GT	GT	GT	GT	GT	GG	GT
AG	AA	GG	AG	--	GG	--	AG	AG	--	AG	AA	AG
GT	TT	GG	GT	GG	--	TT	GT	GT	GT	GT	TT	GT
--	GG	AA	AG	--	AA	GG	AG	AG	AG	AG	GG	AG
--	CC	--	CT	TT	TT	CC	CT	CT	--	CT	CC	CT
AG	GG	AA	AG	AA	AA	GG	AG	AG	--	AG	GG	AG
CT	CC	CT	CT	--	TT	CC	CT	CT	CT	CT	CC	CT
AC	AA	AC	AC	CC	CC	AA	--	AC	AC	AC	AA	AC
AG	GG	AG	AG	--	AA	GG	AG	AG	--	AG	GG	AG
--	CC	CT	CT	TT	TT	CC	CT	CT	--	CT	CC	CT
AG	AA	AG	AG	GG	GG	AA	AG	AG	AG	AG	AA	AG
--	TT	CT	--	CC	--	--	CT	CT	CT	CT	--	CT
GT	GT	GG	GG	--	GG	GT	GG	GT	--	GG	GT	GG
--	AG	AG	AG	AG	GG	GG	AG	GG	--	AG	GG	AG
AT	--	AT	AT	AT	AT	AT	AT	AT	--	AT	AT	TT
AT	AA	AT	TT	AT	AT	AT	AT	AT	--	AT	AT	TT
--	AA	AC	AC	--	--	AC	AC	AC	--	AC	AC	CC
GT	TT	GT	GT	--	GT	GT	GT	GT	--	GT	GT	GG
CT	TT	CT	TT	TT	TT	--	CT	TT	--	TT	CT	CT
--	--	--	AG	AG	AG	AA	AG	GG	--	AG	AG	AA
GG	CG	GG	GG	--	GG	GG	CG	CG	--	GG	CG	GG
TT	CT	TT	TT	--	TT	TT	CT	CT	--	CT	CT	TT
GG	GT	GG	GT	GT	GT	GG	GG	GT	GG	GG	GG	GT
--	GG	--	AG	AG	--	--	--	--	--	AG	AG	GG
--	AC	AA	AA	--	AA	AA	--	AC	--	AC	AC	AC
--	--	GG	GG	--	GG	GG	GG	--	--	CG	CG	CG
TT	GT	TT	GT	--	--	TT	TT	GT	--	GT	GT	GT
CC	TT	CC	TT	CT	CC	CC	CC	TT	CC	CT	CC	TT
--	--	AG	AG	GG	AG	GG	AG	AG	--	AA	AG	AG
CT	CT	CC	CT	--	CC	CC	--	CT	CT	CT	CC	CT
--	CG	CG	CC	CG	CG	CG	CG	CC	--	CC	CG	CC
--	AA	AC	AC	AC	AC	AA	AC	AA	AC	AC	AC	AA
AG	AG	GG	AG	GG	GG	AA	GG	AA	AG	AG	GG	AA
--	CT	--	TT	TT	TT	CT	TT	CT	--	TT	TT	CT
CG	GG	GG	CG	GG	GG	GG	GG	CG	CG	CG	GG	CG
--	AG	AA	AG	--	AA	AG	AA	GG	--	AG	AA	GG
--	CG	--	GG	CG	CG	CG	CG	GG	--	GG	CG	GG
CG	CG	CC	--	--	CC	CG	--	--	--	CG	CC	--
--	CT	CC	CT	CC	CC	CT	CC	--	--	CT	CC	TT
AG	AG	GG	AG	--	GG	AG	GG	--	--	AG	GG	AA
AG	GG	AG	AG	AG	AG	GG	AG	GG	AG	AG	AG	GG
CT	CT	CC	CT	CC	CC	TT	CC	TT	CT	CT	CC	TT
GG	AG	--	GG	--	GG	AG	GG	AG	GG	GG	GG	AG
AG	AG	GG	AG	GG	GG	AA	GG	AA	AG	AG	GG	AA
AC	AC	CC	AC	--	CC	AA	CC	AA	--	AC	CC	AA
AT	TT	TT	AT	TT	TT	AT	TT	AT	AT	AT	TT	AT
--	AG	AA	AG	AA	--	GG	AA	GG	--	--	AA	GG

--	AC	AA	AC	--	AA	CC	--	--	AC	AC	AA	CC
--	AG	AA	AG	--	AA	GG	AA	GG	--	AG	AA	GG
--	AG	--	AA	--	AA	AG	AA	AG	--	AA	AA	AG
CT	CC	CC	CC	--	CC	CT	--	CT	--	CC	CC	CT
--	CC	CC	CC	--	CC	CG	CC	CG	--	CC	CC	CG
--	TT	--	TT	TT	--	CT	TT	CT	--	TT	CT	CT
--	--	CG	--	CG	--	--	CG	GG	--	CG	GG	GG
CT	CC	CC	CC	--	CC	CT	CT	CT	--	CC	CT	CT
--	GT	GT	GT	GT	GT	GG	--	--	--	GT	GG	--

3-48	3-49	3-50	3-52	3-54	3-56	3-57	3-59	3-63	3-66	3-67	3-68	3-69
AC	AA	AC	CC	AC	AC	AC	AC	AC	AC	AC	AA	AC
AG	AA	AG	GG	AG	AG	AG	AG	AG	AG	AG	AA	AG
CT	TT	CT	CC	CT	CT	CT	CT	CT	CT	CT	TT	CT
GG	--	AG	AG	--	GG	GG	AG	GG	AG	AG	GG	AG
--	--	AG	GG	--	--	--	AG	AG	GG	AG	AA	AG
CG	CG	CG	GG	GG	--	GG	CG	GG	GG	CG	CG	CG
TT	--	CT	CC	--	CT	CT	CT	CT	CT	CT	TT	CT
TT	TT	TT	GG	TT	GT	GT	GT	TT	GG	GT	TT	GT
TT	TT	TT	GG	GT	GT	GT	GT	TT	GG	GT	TT	GG
TT	TT	TT	GG	GT	--	GT	GT	TT	GG	GT	TT	GG
AG	AA	AA	GG	--	--	AG	AG	AA	GG	AG	AA	GG
CC	CT	CT	CC	CT	--	CC	CT	--	CC	CT	CT	CC
AC	--	AA	CC	AC	AC	AC	AC	AA	CC	AC	AC	CC
AA	AA	AG	AA	AG	--	AA	AG	AG	AA	AG	AA	AA
CT	CT	CT	CC	CC	--	CT	CT	CT	CC	CC	CC	CC
AG	AG	GG	AG	AG	AG	GG	GG	AG	AA	AG	AA	AA
TT	--	AT	AT	AT	AT	AT	AT	TT	TT	TT	TT	TT
CT	--	CT	CC	--	CT	CT	CT	CC	CC	CC	CC	CC
TT	TT	TT	GT	TT	GT	GT	GT	TT	GT	TT	TT	TT
CC	CC	CC	CG	CC	CG	CG	CG	CC	CG	CC	CC	CG
AT	AT	AT	AT	TT	--	AA	--	TT	AT	TT	TT	AT
CT	--	CT	CT	--	--	CC	CC	TT	CT	TT	TT	CT
AT	--	AT	AT	--	--	TT	TT	AA	AT	AA	AA	AT
AT	AT	AT	AT	--	TT	TT	--	AA	AT	AA	AA	AT
AG	AG	AG	AG	AA	--	GG	GG	AA	AG	AA	AA	AG
TT	--	TT	GT	TT	GT	GT	GT	TT	GT	TT	TT	GT
GT	GT	GT	GT	TT	GG	GG	GG	TT	GT	TT	TT	GT
AT	AT	AT	AT	TT	AA	AA	AA	TT	AT	TT	TT	AT
CT	CT	CT	CT	TT	CC	CC	CC	TT	CT	TT	TT	CT
CG	CG	CG	CG	--	GG	GG	GG	CC	CG	CC	CC	CG
CT	CT	CT	CT	CC	TT	TT	TT	CC	CT	CC	CC	CT
AC	--	AC	AC	--	--	AC	CC	AA	AC	AA	AA	AC
--	--	AG	AG	--	--	GG	GG	AA	AG	AA	AA	AG
GT	GT	GT	GT	GG	--	TT	TT	GG	GT	GG	GG	GT
AG	AG	AG	AG	--	--	GG	GG	AA	AG	AA	AA	--
GT	GT	GT	GT	--	--	GG	GG	TT	GG	TT	TT	--
AG	AG	AG	AG	GG	--	AA	AA	GG	AG	GG	GG	AG
CT	CT	CT	CT	--	--	TT	TT	CC	CT	CC	CC	CT
AT	AT	AT	AT	AA	TT	TT	TT	AA	AT	AA	AA	AT
AG	AG	AG	AG	AA	GG	GG	GG	AA	--	AA	AA	AG
AC	AC	AC	AC	AA	CC	CC	CC	AA	AC	AA	AA	AC
AG	AG	AG	AG	AA	--	GG	GG	AA	--	AA	AA	AG

CT	--	CT	CT	TT	--	CC	CC	TT	CT	TT	TT	CT
CG	CG	CG	CG	CC	GG	GG	GG	CC	CG	CC	CC	CG
CT	CT	CT	CT	--	CC	CT	CC	TT	CT	TT	TT	CT
CT	--	CT	CT	--	--	CT	--	CC	CT	CC	CC	CT
AC	CC	AC	AC	AA	AC	AC	CC	AA	AC	AA	AA	AC
TT	TT	TT	CC	--	--	CT	TT	CC	CT	CC	CC	CC
CC	CC	CC	TT	TT	CT	CT	--	CT	CT	TT	TT	TT
GG	GG	AG	GG	AG	--	AG	GG	AA	AG	AG	GG	AG
GT	GG	GT	GG	--	GT	GT	--	TT	GT	GT	GG	GT
AG	GG	--	GG	AA	--	AG	AG	AA	AG	AG	GG	AA
CT	TT	CC	TT	CC	CT	CT	CT	CC	CT	CT	TT	CC
CG	CG	GG	CC	--	--	GG	CG	GG	CG	CG	CC	GG
AG	AG	GG	AA	GG	--	GG	AG	GG	AG	AG	AA	GG
AC	CC	AA	CC	AC	AC	AC	AC	AA	AC	AC	CC	AA
AG	AG	GG	AG	GG	--	GG	AG	GG	GG	AG	AG	GG
CT	TT	CC	CT	CT	CT	CT	CT	CC	CT	CT	TT	--
CT	CC	TT	CT	CT	TT	CC	CT	TT	CT	CT	CC	TT
CT	--	CC	CT	CT	--	TT	CT	CC	CT	CT	TT	CC
AG	AA	GG	AG	GG	GG	AA	AG	GG	AG	AG	AG	GG
AC	AA	AC	AC	AC	CC	AA	AC	CC	AC	AC	AC	AC
AG	AA	AG	AG	AG	GG	AA	AG	GG	GG	AG	AG	AG
CG	GG	CG	CG	CG	CC	GG	CG	CG	CC	CG	CG	CG
CG	CC	CG	CG	CG	GG	CC	CG	CG	GG	CG	CG	CG
AG	AA	AG	AG	AG	GG	AA	AG	AG	GG	AG	AG	AG
AC	--	AC	AC	AC	AA	CC	AC	AC	AA	AC	AC	AC
CT	--	CT	TT	CT	--	TT	CT	CT	CC	CT	CT	CT
CT	--	CT	CC	CT	--	CC	CT	CT	TT	TT	CT	CT
AT	TT	AT	TT	--	AT	TT	AT	AT	AA	AA	AT	AA
GG	GG	GG	GG	AG	AG	GG	GG	GG	AG	AG	GG	AG
AT	TT	AT	TT	--	--	AT	AT	AT	AT	AT	AT	AT
GG	--	TT	GT	--	--	GT	GT	GT	GT	GG	GT	GG
AG	--	GG	GG	AG	AG	GG	AG	GG	GG	AG	GG	AG
CT	TT	TT	TT	CT	CT	TT	CT	TT	TT	CT	TT	CT
AG	--	GG	GG	--	AG	GG	AG	GG	GG	AG	GG	AG
TT	GT	GG	GT	GT	TT	GT	GT	TT	GT	TT	GT	TT
GG	--	GG	GG	--	GT	GG	GT	GT	GG	GT	GG	GT
CT	CT	TT	CT	CT	CC	CT	CT	CC	CT	CC	TT	CC
CT	CT	TT	CT	--	--	CT	CT	CC	CT	CC	TT	--
AG	AG	AA	AA	AG	AG	AG	AG	GG	AG	GG	AA	GG
CG	CG	GG	GG	--	--	CG	CG	CC	CG	CC	GG	CC
AG	AG	AA	AA	AG	AG	AG	AG	GG	AG	GG	AA	GG
CT	--	CC	CC	--	CT	CT	CT	TT	--	TT	CC	TT
CT	CT	CC	CC	--	CT	CT	CT	TT	CT	TT	CC	TT
CC	CC	CC	CC	CC	CC	CC	CG	CG	CC	CG	CC	CG
TT	TT	TT	TT	--	--	TT	AT	AT	TT	AT	TT	AT
GT	GT	GG	GG	GG	GG	GT	GG	GT	GG	GT	GG	GT
CT	CT	CC	CC	CC	CC	CT	CC	CT	CC	CT	CC	CT

CT	--	CC	CC	CT	--	CT	CT	CT	CC	TT	CC	TT
GT	GT	GG	GG	GG	--	GT	GG	GT	GG	GT	GG	GT
CT	CT	CC	CC	CT	--	CT	CT	CT	CC	TT	CC	TT
GT	GT	TT	TT	TT	TT	GT	TT	GT	TT	GT	TT	GT
CG	CG	GG	GG	GG	GG	CG	GG	CG	GG	CG	GG	CG
--	--	AG	AA	--	--	GG	AA	AA	AG	AG	GG	AA
AG	AG	AG	GG	AG	AA	AA	GG	GG	AG	AG	AA	GG
AG	AG	AG	GG	AG	AA	AA	GG	GG	AG	AG	AA	GG
TT	CT	CT	CC	CT	CT	TT	CC	CC	CT	CT	CT	CC
AG	--	AG	AA	AG	AG	GG	AA	AA	AG	AA	AG	AA
GT	GT	GT	GT	GT	GT	GG	TT	TT	GT	TT	GT	--
GG	GG	GG	AG	AG	--	AG	GG	AG	AG	GG	GG	GG
CT	TT	CC	CT	CT	--	CT	CC	CT	CC	CC	CT	CC
CC	--	AC	AC	AC	CC	CC	AC	AC	AC	AC	CC	AC
CT	CT	CC	CC	--	CT	CT	CC	CC	CC	CC	CT	CC
GT	--	TT	GT	GT	GT	GT	TT	GT	TT	TT	GT	TT
CT	CC	CT	CC	CC	CT	CT	CT	CC	CT	CT	CT	CT
GG	GG	CG	CG	CG	GG	GG	CG	CG	CG	CG	GG	CG
CC	--	AC	AC	AC	CC	CC	AC	AC	AC	AC	CC	AC
CT	--	CT	TT	--	CT	CT	CT	TT	CT	CT	CT	CT
AG	--	AG	AA	--	AG	AG	AG	AA	AG	AG	AG	AG
AG	AA	GG	AG	AG	AG	AG	--	AG	GG	GG	AG	GG
CT	CT	CC	CC	CC	CT	CT	CC	CC	CC	CC	CT	CC
AA	AA	AT	AT	--	AT	AA	AT	AT	AT	AT	AA	AT
CC	CC	CT	CT	CT	CT	CC	CT	CT	CT	CT	CC	CT
AA	AA	AG	AG	AG	AA	AA	AG	AG	AG	AG	AA	AG
CT	CC	CT	CC	CC	CT	CT	CT	CC	CT	CT	CT	CT
AT	TT	AA	TT	--	AT	AT	--	AT	AA	AA	AT	AA
AT	TT	--	AT	--	--	AT	AA	AT	AA	AA	AT	AA
CT	TT	CC	CT	CT	CT	CT	CC	CT	CC	CC	CT	CC
CC	CC	CT	CT	CT	CC	CC	CT	CT	CT	CT	CC	CT
GT	--	GT	GG	--	GT	GT	GT	GG	GT	GT	GT	GT
CT	--	CT	CC	--	CT	CT	CT	CC	CT	CT	CT	CT
AA	AA	AA	AG	AG	--	AA	--	AG	AA	AA	AA	AA
AG	--	AA	AG	AG	--	AG	AA	AG	AA	AA	AG	AA
AG	AA	AG	AA	AA	AG	AG	AG	AA	AG	AG	AG	AG
CT	--	TT	CT	CT	--	CT	TT	CT	TT	TT	CT	TT
AC	--	AC	CC	--	AC	AC	AC	CC	AC	AC	AC	AC
AT	--	AA	AA	AT	--	AT	AA	AT	AA	AA	AT	AA
AA	--	AG	AG	AG	--	AA	AG	AG	AG	AG	AA	AG
AC	AA	AC	AC	AA	AC	AC	AC	AA	AC	AC	AC	AC
AT	TT	AT	AT	TT	AT	AT	AT	TT	AT	AT	AT	AT
AA	AC	AC	AC	CC	AA	AA	AC	CC	AC	AC	AA	AC
CC	CC	AC	AC	AC	--	CC	AC	AC	AC	AC	CC	AC
GG	AG	GG	GG	AG	GG	GG	GG	AG	GG	GG	GG	GG
AC	AC	CC	CC	CC	--	AC	CC	CC	CC	CC	AC	CC
AG	AA	AG	AG	AA	--	AG	AG	AA	AG	AG	AG	AG

CC	CC	CG	CG	CG	CC	CC	CG	CG	CG	CG	CC	CG
CT	TT	CC	CC	CT	--	CT	CC	CT	CC	CC	CT	CC
CT	CC	TT	TT	CT	CT	CT	TT	CT	TT	TT	CT	TT
CT	--	CT	CT	--	CT	CT	CT	CC	CT	CT	CT	CT
AG	AA	AG	AG	AA	AG	AG	AG	AA	AG	AG	AG	AG
CC	CC	TT	TT	--	CT	CT	TT	CT	TT	TT	CT	TT
TT	--	CT	CT	--	--	CT	CT	TT	CT	CT	CT	CT
AA	--	TT	TT	--	--	AT	--	AT	TT	TT	AT	TT
GG	GG	AG	AG	AG	GG	GG	AG	AG	AG	AG	GG	AG
AA	AC	AC	AC	--	AC	AC	AC	AA	AC	AC	AC	AC
CC	CT	CT	CT	--	--	CT	CT	CC	CT	CT	CT	CT
TT	--	GT	GT	GT	--	TT	GT	GT	GT	GT	TT	GT
TT	CT	CT	CT	TT	TT	CT	CT	TT	CT	CT	CT	CT
AC	CC	CC	CC	AC	AC	CC	CC	AC	CC	CC	CC	CC
AA	AG	AG	AG	--	--	AG	AG	AA	AG	AG	AG	AG
GG	GT	GT	GT	GG	GG	GT	GT	GT	GT	GT	GT	GT
AT	--	TT	TT	AT	--	TT	AT	TT	TT	TT	TT	TT
AA	AG	AG	AG	AA	--	AG	AG	AG	AG	AG	AG	AG
CC	--	CT	CT	CT	CC	CC	CT	CT	CT	CT	CC	CT
CC	--	CT	CT	CC	CC	CT	CT	CT	CT	CT	CT	CT
AA	AC	AC	AC	--	--	AC	AC	AC	AC	AC	AC	AC
CC	--	CT	CT	--	--	CT	CT	CT	CT	CT	CT	CT
AA	--	TT	TT	--	--	AT	TT	TT	TT	TT	AT	TT
GG	AG	AA	AA	AG	GG	AG	AA	AA	AA	AA	AG	AA
GG	--	CG	CG	CG	GG	GG	CG	CG	CG	CG	GG	--
TT	CT	CT	CT	TT	--	CT	CT	CT	CT	CT	CT	CT
GG	AG	AG	AG	GG	GG	AG	AG	AG	AG	AG	AG	AG
AA	AG	AG	AG	--	--	AA	AG	AG	AG	AG	AG	AG
AA	AA	AG	AG	AG	AG	AA	AG	AG	AG	AA	AA	AG
GG	GG	AG	AG	--	AG	GG	AG	AG	AG	GG	GG	AG
TT	--	--	CT	CT	--	TT	CT	CT	CT	TT	CT	TT
GG	GT	TT	TT	--	--	GG	TT	TT	TT	GG	GT	TT
TT	TT	GT	GT	GT	GT	TT	GT	GT	GT	TT	TT	GT
GG	AG	AA	AA	--	--	GG	AA	AA	AA	GG	GG	AG
CC	CT	CT	CT	CT	CT	CC	CT	CT	CT	CC	CC	CC
TT	TT	TT	GT	GT	GT	TT	GT	GT	GT	TT	TT	TT
AG	AG	GG	GG	GG	--	AG	GG	GG	GG	GG	AG	GG
GG	GG	AG	GG	--	--	GG	AG	AG	AG	AG	AG	AG
CC	CC	CC	CC	CC	CT	CC	CC	CC	CT	CT	CC	CT
AT	AT	AA	AT	AA	AA	AT	AA	AA	AA	AT	AA	AT
GG	GG	CC	GG	CG	CC	GG	CG	CG	CC	CG	CG	CG
AC	AC	AA	AC	AC	--	AC	AC	AC	AA	AA	AC	AA
GG	AG	AA	GG	AG	AA	GG	AG	AG	AA	AG	AG	AG
GG	GG	AG	GG	AG	--	GG	AG	AG	AG	AG	AG	GG
CC	CC	CT	CC	CT	CT	CC	CT	CT	CT	CT	CT	CC
AG	AG	GG	AA	AG	AG	AA	AG	AG	GG	AG	AG	AG
CC	CC	CC	CT	CT	CT	CC	CC	CT	CC	CT	CC	CC

AG	AG	GG	AG	GG	GG	AG	GG	GG	GG	GG	GG	AG
AG	--	AG	AG	GG	--	AG	AG	AG	GG	GG	AG	AA
TT	CT	TT	TT	CT	CT	TT	CC	CC	CT	CT	CC	CT
TT	AT	TT	TT	AT	AT	TT	AA	AA	AT	AT	AA	AT
CC	AC	CC	CC	AC	AA	CC	AA	AA	AC	AC	AA	AC
CC	CT	CC	--	CT	--	CC	TT	TT	CT	CT	TT	CT
AG	AG	AA	AA	AG	--	AG	GG	GG	AG	AG	GG	AA
CT	CT	TT	TT	CT	--	CT	CT	CC	CT	CT	CC	TT
TT	TT	CT	--	--	--	CT	CT	TT	CT	CT	TT	CT
AG	AG	GG	--	AG	AA	AG	AG	AA	AG	AG	AA	GG
GG	--	AG	GG	--	--	GG	AG	AG	AG	AA	AG	AA
TT	CT	CT	TT	CT	TT	TT	CT	CT	CT	CC	CT	CC
AG	AG	GG	AG	AG	AG	AG	AG	AG	GG	GG	GG	GG
TT	TT	--	TT	--	TT	TT	CT	CT	CT	CC	CT	CC
CG	--	CG	CG	--	--	CG	GG	GG	CG	GG	CG	GG
TT	TT	CT	TT	TT	--	--	CT	CT	CT	CC	CT	CC
CT	CT	CC	CT	CT	--	CT	CT	CT	CC	CC	CC	CC
AA	--	AG	AA	--	AG	AA	AG	AA	AG	GG	AG	GG
TT	--	CT	TT	TT	CT	TT	CT	TT	CT	CC	CT	CC
GG	GG	AG	GG	GG	AG	GG	AG	GG	AG	AA	AG	AA
TT	TT	CT	TT	TT	CT	TT	CT	TT	CT	CT	CT	CC
AA	AA	AG	AA	AA	AG	AA	AG	AA	AG	AG	AG	GG
AG	--	GG	AG	AG	AG	AG	AG	AG	GG	AG	GG	GG
GG	GG	GT	GG	GG	GT	GG	GT	GT	GT	GT	GT	TT
CG	CC	CC	CG	CG	CG	CG	CG	CC	CC	CG	CC	CC
CC	--	CC	CC	--	--	CC	CG	CC	CC	CC	CC	CG
TT	--	CT	TT	TT	CT	TT	CT	CT	TT	CT	CT	CC
CC	CC	CT	CC	--	CT	CC	TT	CT	CC	CT	CT	TT
TT	TT	AT	TT	TT	--	TT	AA	AT	TT	AT	AT	AA
AG	AA	AG	AA	AG	AG	AA	GG	AG	AA	AG	AG	AG
CT	TT	CT	TT	CT	CT	TT	CC	CT	TT	CT	CT	CT
TT	CT	TT	CT	TT	CT	CT	TT	TT	CT	TT	TT	CT
AC	CC	AC	CC	--	AC	CC	AA	AC	CC	AC	AC	AC
TT	--	TT	GG	--	--	GG	--	GT	GG	GT	GT	GT
AA	AG	AA	GG	AG	AA	GG	AA	AG	GG	AG	AG	AG
TT	CT	TT	CC	CT	TT	CC	--	CT	CC	CT	CT	CT
AA	--	AA	TT	AT	--	TT	AA	AT	TT	AT	AT	AT
CC	AC	CC	AA	AC	CC	AA	CC	AC	AA	AC	AC	AC
CG	CG	CG	CC	CG	CG	CC	CG	CC	CC	CG	CG	CC
AG	--	AG	AA	AG	AG	AA	AG	AA	AA	AG	AG	AA
CC	--	CC	TT	--	--	TT	CC	CT	TT	CT	CT	CT
CC	CT	CC	--	--	--	TT	CC	CT	TT	CT	CT	CT
AA	AG	AA	GG	AG	--	GG	AA	AG	GG	AG	AG	AG
CT	--	CT	CC	--	--	CC	CT	CT	CC	CC	CC	CT
AA	AG	AA	GG	AG	--	GG	AA	AG	GG	AG	AG	AG
GG	AG	GG	AA	AG	--	AA	GG	AG	AA	AG	AG	AG
GG	GT	GG	TT	--	--	TT	GG	GT	TT	GT	GT	GT

TT	--	TT	CC	CT	--	CC	TT	CT	CC	CT	CT	CT
GG	CG	GG	CC	CG	GG	CC	GG	CG	CC	CG	CG	CC
TT	GT	TT	GG	GT	TT	GG	TT	GT	GG	GT	GT	GG
AA	--	AA	GG	AG	--	GG	AA	AG	GG	AG	AG	GG
CC	CT	CC	TT	CT	--	TT	CC	CT	TT	CT	CT	TT
GT	--	GT	TT	--	--	TT	GT	GT	TT	TT	TT	TT
GG	CG	GG	CC	CG	GG	CC	GG	CG	CC	CG	CG	CC
AA	--	AA	GG	--	--	GG	AA	AG	GG	AG	AG	GG
AG	--	AG	GG	GG	AG	GG	AG	AG	GG	GG	GG	GG
AA	AC	AA	CC	AC	--	CC	AA	AC	CC	AC	AC	CC
CG	CC	CG	CC	CC	CG	CC	CG	CG	CC	CC	CC	CC
GG	AG	GG	AG	--	--	AA	GG	AG	AA	AG	AG	AA
AA	AC	AA	AC	AC	AA	CC	AA	AC	CC	AC	AC	CC
TT	GT	TT	GT	--	TT	GG	TT	GT	GG	GT	GT	GG
CC	AC	CC	AC	AC	CC	AA	CC	AC	AA	AC	AC	AA
CT	--	CT	CT	--	CT	CC	CT	CT	CC	CC	CC	CC
TT	GT	TT	GT	GT	TT	GG	TT	GT	GG	GT	GT	GG
AG	AG	AG	GG	AG	--	GG	AG	GG	GG	AG	AG	GG
CC	--	CC	CT	CT	CC	TT	CC	CT	TT	CT	CT	--
CT	CC	CT	CT	--	CT	CC	CT	CT	CC	CC	CC	--
AA	--	AA	AT	AT	--	TT	AA	AT	TT	AT	AT	TT
CC	CG	CC	CG	CG	--	GG	CC	CG	GG	CG	CG	GG
GG	GT	GG	GT	GT	GG	TT	GG	GT	TT	GT	GT	TT
AG	AG	AG	GG	AG	--	GG	GG	GG	GG	AG	AG	GG
GT	--	TT	TT	GT	--	GT	TT	TT	GT	GT	GT	GT
CT	CT	CC	CT	CT	--	TT	CT	CT	TT	CT	CT	TT
AC	AC	CC	AC	AC	CC	AA	AC	CC	AA	AC	AC	AA
CC	--	CC	--	--	--	CT	CT	CC	CT	CC	CC	CT
CG	CG	CG	GG	CG	--	CC	CG	GG	CC	CG	CG	CC
CC	CT	CT	CC	CT	--	TT	CT	CC	TT	CT	CT	TT
GT	GT	GT	GT	GT	GT	TT	TT	GT	TT	GT	GT	GT
CT	--	TT	CT	--	--	TT	CT	CT	TT	TT	TT	TT
AT	AT	AT	AT	AT	AT	TT	TT	AT	TT	AT	TT	AT
CC	CC	CC	CC	CC	CC	AC	AC	CC	AC	CC	AC	CC
TT	TT	CT	TT	CT	--	CT	CT	TT	CC	TT	CC	CT
AA	--	AA	AA	--	AA	AG	AG	AA	AG	AA	AG	AA
GG	--	GG	GG	--	--	AG	AG	GG	AG	GG	AG	GG
CG	--	GG	--	CG	GG	CG	GG	GG	CG	GG	CG	GG
CT	--	CT	CT	--	TT	CT	CC	CT	TT	CT	TT	CT
CT	CC	CT	CT	CT	TT	CT	CC	CT	TT	CT	TT	CT
CT	CC	CT	CT	CT	CT	CC	CT	CT	CT	CT	CT	CC
CT	--	CT	CT	CT	--	TT	CT	CT	CT	CT	CT	TT
GT	GG	GT	GT	GT	GT	GG	GT	GT	GT	GT	GT	GG
CT	--	CT	CT	CT	--	TT	CT	CT	CT	CT	CT	TT
AT	--	AT	AT	AT	--	TT	AT	AT	AT	AT	AT	TT
AG	AG	AG	AG	--	GG	GG	AG	AG	AG	AG	AG	GG
AC	AC	AC	AC	AC	--	AA	AC	AC	AC	AC	AC	AA

AG	AG	AG	AG	AG	--	GG	AG	AG	AG	AG	AG	GG
AG	AG	AG	AG	AG	--	GG	AG	AG	GG	AG	AG	GG
CT	CT	CT	CT	CT	CC	CC	CT	CT	CC	CT	CT	CC
AG	--	AG	AG	AG	--	AA	AG	AG	AA	AG	AG	AA
GT	GT	GT	GT	GT	TT	TT	GT	GT	TT	GT	GT	TT
CT	CT	CT	CT	--	CC	CC	CT	CT	CC	CT	CC	CC
AG	AG	AG	AG	--	--	AA	AG	AG	AA	AG	AA	AA
AG	AG	AG	AG	AG	--	AA	AG	AG	AA	AG	AA	AA
AG	AG	AG	AG	AG	--	GG	AG	AG	GG	AG	GG	GG
CG	--	CG	CG	CG	--	CC	CG	CG	CC	CG	CC	CC
AG	AG	AG	AG	AG	GG	GG	AG	AG	--	AG	GG	GG
AC	AC	AC	AC	--	CC	CC	AC	AC	CC	AC	CC	CC
AG	AG	AG	AG	AG	AA	AA	AG	AG	AA	AG	AA	AA
AG	--	AG	AG	--	--	AA	AG	AG	AA	AG	AA	AA
AT	AT	AT	AT	TT	--	TT	AT	AT	TT	AT	TT	TT
CT	CT	TT	CT	CC	CC	CC	CT	CT	CC	CT	CC	CC
CT	--	--	CT	CC	--	CC	CT	CT	CC	CT	CC	CC
CG	CG	CC	CG	GG	GG	GG	CG	CG	GG	CG	GG	GG
CT	CT	CC	CT	TT	TT	TT	CT	CT	TT	CT	TT	TT
CT	CT	TT	CT	--	CC	CC	CT	CT	CC	CT	CC	CC
CT	CT	CC	CT	TT	TT	TT	CT	CT	TT	CT	TT	TT
AT	AT	AA	AT	TT	TT	TT	AT	AT	TT	AT	TT	TT
AT	AT	TT	AT	AA	AA	AA	AT	AT	AA	AA	AA	AA
CT	CT	CC	CT	TT	TT	TT	TT	CT	TT	TT	TT	TT
CT	CT	TT	CT	CC	CC	CC	CC	CT	CC	CC	CC	CC
AT	AT	TT	AT	AA	AA	AA	AA	AT	AA	AA	AA	AA
AG	AG	AA	AG	--	GG	GG	GG	AG	GG	GG	GG	GG
GT	GT	TT	GT	--	--	GG	GG	GT	GG	GG	GG	GG
CT	CT	CC	CT	--	--	TT	TT	CT	TT	TT	TT	TT
CG	CG	GG	CG	--	--	CC	CC	CG	CC	CC	CC	CC
AG	AG	AA	AG	GG	GG	GG	GG	AG	GG	GG	GG	GG
CT	--	CC	CC	TT	TT	TT	TT	CT	TT	TT	TT	--
CT	CT	CC	CT	TT	--	TT	TT	CT	TT	TT	TT	TT
AT	--	AA	AT	TT	TT	TT	TT	AT	TT	TT	TT	TT
AG	--	GG	AG	--	--	AA	--	AG	AA	AA	AA	AA
AG	--	GG	AA	AG	AA	AA	AA	AG	AA	AA	AA	AA
GT	GT	GG	TT	GT	TT	TT	TT	GT	TT	TT	TT	TT
AG	--	GG	AA	--	--	AG	AA	AG	AA	AA	AA	AA
AG	--	AG	GG	--	--	GG	GG	GG	GG	GG	GG	GG
TT	CT	TT	TT	--	TT	CT	CT	CT	TT	TT	CT	TT
AG	--	GG	GG	AG	GG	AA	AA	AG	AG	GG	AA	GG
AG	AG	GG	GG	GG	--	AG	AG	GG	AG	GG	AG	GG
AG	--	GG	AG	AG	--	AG	AG	AG	AG	GG	AA	GG
CG	GG	CC	CG	CG	CC	CG	CG	CG	CG	CC	CG	CC
CT	CC	CT	CT	CT	--	CT	CT	CT	CT	CT	CT	TT
AC	CC	AC	AC	--	AC	AC	AC	CC	AC	AC	CC	AC
AA	--	AA	AA	AG	--	AA	AA	AA	AA	AA	AG	AG

AC	CC	CC	AC	CC	CC	AC	AC	CC	AC	AC	CC	AC
AC	AC	AC	AC	CC	--	AC	AC	AC	AC	AC	CC	CC
CT	--	CT	CT	TT	CT	CT	CT	TT	CT	CT	TT	CT
AG	AA	AA	AG	AG	AG	GG	AG	AA	AG	AG	AG	GG
AG	--	GG	AG	AG	--	AA	AG	GG	AG	AA	AG	AG
CT	TT	TT	CT	CT	CC	CC	CT	TT	CT	CC	CT	CT
CT	--	CT	CT	--	--	TT	CT	CT	CT	TT	TT	CT
CG	CG	CC	CG	--	--	GG	CG	CC	CG	GG	CG	CG
AG	AG	AA	AG	AG	GG	GG	AG	AA	AG	GG	AG	AG
CT	--	TT	CT	CC	CC	CC	CT	TT	CT	CC	CT	CT
AT	--	AA	AT	AT	AT	AA	AT	AT	AT	AA	AA	AT
AA	AC	AC	AC	--	--	AC	AC	CC	AC	AC	AA	AC
AG	GG	GG	AG	AG	AG	AG	AG	GG	--	GG	AG	AG
CT	CT	CC	CT	CT	CT	CT	CT	CC	CC	CC	CT	CT
CT	CT	CC	--	--	CT	CT	CT	CC	CC	CC	CT	CT
GT	GT	GG	GT	GT	--	GT	GT	GG	GT	GG	GT	GT
CT	CC	CT	CT	CT	CC	CC	CC	TT	TT	CC	CC	CT
AG	AG	AG	AG	--	AG	AG	AG	AG	GG	AG	AG	AG
AG	--	AG	AG	AG	--	AA	AA	GG	GG	AA	AA	AG
CG	--	GG	CG	--	--	GG	GG	CG	CG	GG	GG	CG
AG	AA	AA	AG	--	AA	AA	AG	GG	GG	AA	AA	AG
GG	AG	GG	AG	AG	GG	GG	AG	AA	AA	GG	GG	AG
GT	TT	GT	TT	TT	GT	TT	TT	TT	TT	GT	GT	TT
GG	AG	GG	AG	AG	--	AG	AG	AA	AA	GG	GG	AG
GG	AG	--	--	--	--	AG	AG	AA	AA	GG	GG	AG
GT	GG	GT	GG	GT	--	GG	GG	GT	GG	GT	GT	GG
TT	CT	TT	CT	--	--	CT	--	CT	CC	TT	TT	CT
CC	CT	CC	CT	CC	CT	CT	TT	CT	TT	CC	CC	CT
CC	CT	CT	CT	CT	CT	CT	CC	CC	CC	CT	CT	CT
AG	AG	AG	AG	GG	AG	AG	AA	AG	AA	AG	GG	AG
CT	CT	CT	CC	TT	CT	CC	CC	CT	CC	CT	TT	CT
AG	AG	AG	AA	GG	AG	AA	AA	AG	AA	AG	GG	AG
CT	CT	CT	CC	TT	--	CC	--	CT	CC	CC	TT	CT
CG	--	CG	CG	--	CG	GG	GG	CG	CG	GG	CC	CG
CT	--	CT	CT	CT	CT	CC	CC	CT	CT	CC	TT	CT
AC	AC	CC	AC	--	--	AA	AA	AC	AA	AC	AC	AC
AG	AG	AA	AG	--	--	GG	GG	AG	GG	AG	AG	AG
CT	CT	CC	CT	CT	TT	CT	TT	CT	TT	CT	CT	CT
AA	GG	GG	AG	AG	--	AG	AA	AG	AA	AG	GG	AG
AA	GG	GG	AG	--	--	AG	AA	AG	AA	AG	GG	AG
TT	CT	CT	TT	CT	--	TT	TT	TT	TT	TT	CT	CT
GG	--	AA	AG	--	--	AG	GG	AG	GG	AG	AA	AG
AA	AG	AG	AG	--	AG	AG	AA	AG	AA	AG	AG	AA
TT	GT	GT	GT	TT	GT	GT	TT	GT	TT	GT	GT	TT
TT	--	GT	GT	TT	GT	GT	TT	GT	TT	GT	GT	TT
AC	CC	CC	AC	--	AC	AC	AA	AC	AA	AC	CC	AC
AG	GG	GG	AG	AG	--	AA	AA	AG	AA	AG	GG	AG

CG	--	GG	CG	CG	CC	CC	CC	CG	CC	CG	GG	CG
CG	GG	GG	CG	--	CC	CC	CC	CG	CC	CG	GG	CG
CT	CC	CC	CT	CT	TT	TT	TT	CT	CT	CT	CC	CT
CT	CT	TT	CT	CT	CC	CC	CC	CT	CT	CT	TT	CT
AT	AT	TT	AT	--	--	AA	AA	AT	AT	AA	TT	AT
GG	AG	GG	GG	AG	AG	AG	AG	GG	GG	AG	GG	AG
CT	CT	TT	CT	CT	CC	CC	CC	CT	CT	CC	TT	CT
TT	--	CT	CT	--	--	TT	TT	CT	CT	TT	CC	CT
CC	CT	CT	CT	CT	--	CC	CC	CT	CT	CC	TT	TT
AG	AG	AG	AG	AG	GG	GG	GG	AG	AG	GG	AG	AA
CG	CG	CC	CC	CG	CC	CC	CC	CC	CC	CC	CC	CG
GG	--	CG	CG	GG	--	GG	GG	CG	CG	GG	CG	CG
GT	GT	GG	GG	--	GT	GG	GT	GG	GG	GT	GG	GT
CC	CG	CC	CC	CG	CG	CG	CG	CG	CG	CC	CG	CC
AG	AG	AG	GG	--	AG	AA	AG	AG	AA	AG	AA	GG
AA	AC	AA	AA	AC	--	AC	AC	AC	AC	AA	AC	AA
AA	AT	AA	AA	AT	AT	AT	AT	AT	AT	AA	AT	AA
AG	AG	AG	GG	AA	AG	AA	AA	AG	AA	AG	AA	GG
AT	AT	AT	AA	--	AT	TT	--	AT	TT	AT	TT	AA
AG	AG	AG	GG	AA	AG	AA	AA	AG	AA	AG	AA	GG
AC	CC	AC	CC	AC	AC	AC	AC	AC	AC	AC	AC	CC
CT	CT	CT	CC	TT	--	TT	TT	TT	TT	CC	TT	CC
GT	GT	GT	TT	GT	GG	GG	GG	GG	GG	TT	GG	GT
GT	GT	GT	TT	--	GG	GG	GG	GG	GG	TT	GT	GT
CT	CC	CT	CC	CC	TT	TT	TT	TT	TT	CC	CT	CT
CG	--	CG	CC	CC	--	GG	GG	GG	GG	CC	CG	CG
AG	--	AG	GG	--	--	AA	AA	AA	AA	GG	AG	AG
CT	--	CT	CC	CC	CT	TT	TT	TT	TT	CC	CT	CT
GT	TT	GT	TT	TT	GG	GG	GG	GG	GG	TT	GT	GT
CT	TT	CT	TT	TT	CC	CC	CC	CC	CC	TT	CT	CT
CG	--	CG	GG	GG	CG	CC	CC	CC	CC	GG	CG	CG
AG	--	AG	GG	GG	AG	AA	AA	AA	AA	GG	AG	AG
AC	--	CC	--	--	AC	CC	CC	CC	CC	AA	AC	AC
CT	CT	CC	TT	--	CT	CC	CC	CC	CC	TT	CT	CT
GG	--	AG	GG	GG	AG	AG	AG	AG	AG	GG	AG	AG
AC	--	CC	AA	AA	AC	CC	CC	CC	CC	AA	AC	AC
CT	CC	TT	CC	CC	CT	TT	TT	TT	TT	CC	CT	CT
AG	GG	AA	AG	GG	AG	AA	AA	AA	AA	GG	AG	AG
CG	--	CC	CG	GG	CG	CC	CC	CC	CC	GG	CG	CG
GT	--	TT	GT	--	--	TT	TT	TT	TT	GG	GT	GT
CT	CC	TT	CT	--	CT	TT	TT	TT	TT	CC	CT	CT
TT	TT	CT	TT	TT	CT	CT	CT	CT	CT	TT	CT	CT
GG	AG	GG	GG	AG	AG	GG	GG	GG	GG	AG	AG	AG
CG	--	GG	CG	CC	CG	GG	GG	GG	CG	CG	CG	CG
AT	AA	AA	AT	AA	AA	AT	AT	AA	AA	AT	AA	AA
AA	AA	AG	AA	AA	AG	AG	AG	AG	AA	AA	AG	AG
CC	CC	AC	CC	CC	AC	AC	AC	AC	CC	CC	AC	AC

TT	TT	CT	TT	--	CT	CT	CT	CT	TT	TT	CT	CT
CT	--	TT	CT	--	CT	CT	TT	TT	CT	CT	TT	CT
AG	GG	GT	AG	GG	AT	AT	GT	GT	AG	AG	GT	AT
AA	AA	AT	AA	AA	AT	AT	AT	AT	AA	AA	AT	AT
GG	GG	AG	AG	GG	AG	AA	AG	AG	AG	AG	AG	AA
CC	CC	AC	AC	--	--	AA	AC	AC	AC	AC	AC	AA
AA	AG	AG	AA	AA	AG	AG	AG	AG	AA	AA	AG	AG
GG	--	AG	AG	GG	AG	AA	AG	AG	AG	AG	AG	AA
--	--	AG	AA	AA	AG	GG	AG	AG	AG	AG	AG	AG
GG	GG	AG	GG	--	--	AA	AG	AA	AG	AG	AG	AG
GG	--	GT	GG	--	--	TT	GT	TT	GT	GT	GT	GT
GG	GG	AG	GG	--	--	AG	AG	AG	GG	GG	GG	GG
CC	--	TT	--	CC	TT	TT	TT	TT	CC	CC	CT	CT
CC	CG	GG	CC	CC	GG	GG	GG	GG	CC	CC	GG	CG
TT	TT	CT	TT	TT	CT	CT	CT	CT	TT	TT	CT	CT
TT	CT	CT	TT	TT	CT	TT	CT	CT	TT	TT	CT	CT
CT	CC	CT	CT	CT	CC	CT	CC	CC	CT	CT	CT	CC
GG	GT	GG	GT	GG	GT	GT	GT	GG	GT	GT	GG	GT
AG	AG	AG	AA	AG	--	AA	AG	GG	AA	AA	AG	AG
AG	AG	AG	GG	AG	--	GG	AG	AA	GG	GG	AG	GG
AG	--	AG	AG	AG	AA	AA	AA	AA	AG	AG	AG	AA
GT	GT	--	TT	GG	GT	GT	GT	GG	TT	TT	GT	GT
AG	AA	AG	AG	--	--	AG	AA	AA	AG	AG	AG	AA
CT	CC	CT	CT	CC	CC	CT	CC	CC	CT	CT	CT	CC
AA	AG	AA	AG	AG	AG	AA	AG	AG	AA	AA	AA	AG
AG	--	AG	AG	GG	AG	AG	AG	AG	AA	AA	AG	AG
AG	AG	AG	--	GG	AG	AG	GG	AG	AA	AA	AG	AG
CG	CG	CG	CG	GG	CG	CG	--	CC	CC	CC	CG	CG
CG	--	CG	CG	GG	CG	CG	GG	CC	CC	CC	CG	CG
AG	GG	AG	AG	GG	--	AG	GG	AA	AA	AA	AG	AG
GG	--	--	AG	GG	GG	GG	GG	AG	AG	AG	GG	AG
CT	CT	CT	CT	--	--	CT	TT	CC	CC	CC	CT	CT
CC	--	CC	AC	--	AC	CC	CC	AC	AC	AC	CC	AC
CC	CC	CC	--	CC	CG	CC	CC	CG	CG	CG	CC	CG
CC	CC	CC	AC	CC	AC	CC	CC	CC	CC	CC	CC	AC
AC	AA	CC	AC	--	AC	AC	AA	AC	AC	AC	CC	AC
AG	AA	AG	AA	--	--	AG	AA	AG	AG	AG	AG	AA
AG	--	GG	AG	AA	AG	GG	AA	AG	AG	AA	GG	AG
AG	GG	AG	GG	--	--	AG	GG	AG	AG	GG	AG	GG
CT	CT	TT	CT	CC	CT	TT	CC	CT	CT	CC	TT	CC
CT	--	TT	CT	--	CT	TT	CT	CT	CT	CC	TT	CC
AA	AG	AG	AG	--	--	AG	AA	AA	AA	AA	AG	AA
CC	CT	CT	CT	--	--	CT	CC	CC	CC	CC	CT	CC
GG	GG	AG	AA	GG	--	AG	AG	AG	GG	AG	GG	GG
CC	CC	CC	AC	--	--	AC	CC	AC	CC	CC	CC	CC
CC	CC	CT	CT	CC	CT	CC	CT	CC	CC	CT	CC	CC
AA	--	AG	AG	--	--	AG	AG	AG	AA	GG	AA	--

AA	AA	AG	AG	AA	AG	AA	AG	AA	AA	AG	AA	AA
AA	AA	AG	AG	--	GG	AG	AG	AG	AA	AG	AA	AA
CC	CC	AC	AC	CC	--	AC	AC	AC	CC	AC	CC	CC
GG	--	AG	AG	GG	AA	AG	AG	AG	GG	AG	GG	GG
TT	--	GT	--	--	--	GT	GT	GT	TT	GT	TT	TT
AA	AA	AA	AA	--	AG	AG	AA	AG	AA	AG	AA	AA
TT	--	CT	CT	CT	--	CT	CT	CT	TT	CT	TT	CT
CC	CC	CC	CC	CC	CT	CT	CC	CT	CC	CT	CC	CC
GG	GG	AG	AA	--	AA	AG	AG	AG	GG	AG	GG	AG
GG	GG	CG	CG	CG	CG	GG	CG	GG	GG	GG	GG	CG
TT	TT	CT	CT	CT	--	TT	CT	TT	TT	TT	TT	CT
AG	AA	AG	GG	AG	AG	AG	AG	AG	AA	AG	AA	GG
AG	AA	AG	GG	AG	AG	AG	AG	AG	AA	AG	AA	GG
CG	--	CG	GG	--	CG	CG	CG	CG	CC	CG	CC	GG
AG	--	AG	GG	GG	AG	AG	--	AG	AA	AG	AA	GG
AG	--	AG	AA	AA	GG	AG	AG	GG	AA	AG	GG	AG
CT	TT	CT	CC	CC	TT	CT	CT	CT	CC	CT	TT	CT
AG	--	AG	AA	--	--	AG	AG	AG	AA	AG	GG	AG
CG	CC	CG	GG	--	CC	CG	CG	CG	GG	CG	CC	CG
AC	--	AC	AA	AA	--	AC	AC	AC	AC	AC	CC	AC
AG	AA	AG	GG	GG	--	AG	AG	AG	GG	AG	AA	AG
GT	GG	GT	TT	--	--	GT	GT	GT	TT	GT	GG	GT
AG	--	AG	AA	--	--	AG	AG	AG	AA	AG	GG	AG
AG	GG	AG	AA	AA	--	AG	AG	AG	AA	AG	GG	AG
AG	--	AG	GG	--	--	AG	AG	AG	GG	AG	AA	AG
AC	AA	AC	CC	CC	--	AC	AC	AC	CC	AC	AA	AC
AG	AA	AG	GG	GG	AA	AG	AG	AG	GG	AG	AA	AG
AG	AA	AG	GG	GG	AA	AG	AG	AG	GG	AG	AA	AG
CT	TT	CT	CC	CC	TT	CT	CT	CT	CC	CT	TT	CT
CG	GG	CG	CC	CC	GG	CG	CG	CG	CC	CG	GG	CG
AC	--	AC	CC	--	--	AC	AC	AC	CC	AC	AA	AC
CT	TT	CT	CT	--	TT	CT	CT	CT	CC	CT	TT	CT
GT	TT	GT	GT	GG	--	GT	GT	GT	GG	GT	TT	GT
CT	--	CT	CT	--	CC	CT	CT	CT	TT	CT	CC	CT
CG	CC	CG	CG	GG	--	CG	CG	CG	GG	CG	CC	CG
AT	--	AT	AT	--	--	AT	AT	AT	AA	AT	TT	AT
CT	CC	CT	CT	TT	--	CT	CT	CT	TT	CT	CC	CT
AG	GG	AG	AG	--	--	AG	AG	AG	AA	AG	GG	AG
AG	AA	AG	AG	GG	AA	AG	AG	AG	GG	AG	AA	AG
AT	AA	AT	AT	--	AA	AT	TT	AT	TT	AT	AA	AT
AG	GG	AG	AG	AA	GG	AG	AA	AG	AA	AG	GG	AG
AG	--	AG	AG	--	--	AG	GG	AG	GG	AG	AA	AG
CG	CC	CG	CG	GG	--	CG	GG	CG	GG	CG	CC	CG
AG	AA	AG	AG	GG	AA	AG	GG	AG	GG	AG	AA	AG
CG	--	CG	CG	--	--	CG	CC	CG	CC	CG	GG	CG
AG	--	AG	AG	AA	--	AG	AA	AG	AA	AG	GG	AG
AC	--	AC	AC	--	--	AC	CC	AC	CC	AC	AA	AC

AG	GG	AG	AG	--	--	AG	--	AG	AA	AG	GG	AG
CG	CC	CG	CG	--	--	CG	GG	CG	GG	CG	CC	CG
CT	--	CT	CT	--	--	CT	--	CT	TT	CT	CC	CT
AT	--	AT	AT	AA	--	AT	AA	AT	AA	AT	TT	AT
CT	--	CT	CT	--	--	CT	TT	CT	TT	CT	CC	CT
AT	AA	AT	AT	TT	AA	AT	TT	AT	AT	AT	AA	AT
AT	TT	AT	AT	--	--	AT	AA	AT	AA	AT	TT	AT
CG	GG	CG	CG	CC	GG	CG	CC	CG	CC	CG	GG	CG
CT	CC	CT	CT	TT	CC	CT	TT	CT	TT	CT	CC	CT
AG	AA	AG	AG	GG	AA	AG	GG	AG	GG	AG	AA	AG
AC	AA	AC	AC	CC	AA	AC	CC	AC	CC	AC	AA	AC
GT	TT	GT	GT	GG	TT	GT	GG	GT	GG	GT	TT	GT
CT	CC	CT	CT	TT	--	CT	TT	CT	TT	CT	CC	CT
CG	--	CG	CG	CC	GG	CG	CC	CG	CC	CG	GG	CG
GT	TT	GT	GT	GG	--	GT	GG	GT	GG	GT	TT	GT
CT	TT	CT	CT	CC	TT	CT	CC	CT	CC	CT	TT	CT
AG	--	AG	AG	GG	AA	AG	GG	AG	GG	AG	AA	AG
AC	AA	AC	AC	--	--	AC	CC	AC	CC	AC	AA	AC
CT	CC	CT	CT	TT	CC	CT	TT	CT	TT	CT	CC	CT
AC	AA	AC	AC	CC	--	AC	CC	AC	CC	AC	AA	AC
AG	AA	AG	AG	GG	--	AG	GG	AG	GG	AG	AA	AG
GT	--	GT	GT	--	GG	GT	TT	GT	TT	GT	GG	GT
GT	GG	GT	GT	--	--	GT	TT	GT	TT	GT	GG	GT
AG	GG	AG	AG	AA	GG	AG	AA	AG	AA	AG	GG	AG
CT	CC	CT	CT	TT	CC	CT	TT	CT	TT	CT	CC	CT
CT	--	CT	CT	--	CC	CT	TT	CT	TT	CT	CC	CT
AG	AA	AG	AG	GG	AA	AG	GG	AG	GG	AG	AA	AG
CT	CC	CT	CT	TT	CC	CT	TT	CT	TT	CT	CC	CT
CT	TT	CT	CT	CC	TT	CT	CC	CT	CC	CT	TT	CT
AG	GG	AG	AG	AA	GG	AG	AA	AG	AA	AG	GG	AG
CT	CC	CT	CT	--	CC	CC	TT	CT	TT	CT	CC	CT
CT	--	TT	TT	CT	--	TT	CT	CT	CT	CT	TT	CT
CT	--	CT	CT	CT	CC	CC	TT	CT	TT	CT	CC	CT
AG	AA	AA	AG	GG	AA	AA	GG	AA	GG	AG	AA	AG
AC	--	AA	AC	CC	AA	AA	CC	AA	AC	AA	AC	AC
CG	CG	CG	CC	CC	--	CG	CC	CG	CC	CG	CC	CG
GT	--	GT	GT	GT	GT	GG	TT	GG	GT	GG	GT	GT
AG	AG	AG	AG	AG	AG	AG	AA	GG	AG	GG	AG	AG
CT	CT	CT	CT	CT	CT	CT	CC	TT	CT	TT	CT	CT
AG	AG	AG	GG	--	--	AG	GG	GG	GG	AG	GG	AG
AG	AG	AG	AG	AG	AG	AG	AA	AA	AG	GG	AG	AG
GG	CG	CG	CC	CG	CG	CG	CC	CC	CG	GG	CG	CG
TT	CT	CT	TT	TT	TT	CT	TT	TT	TT	CT	TT	CT
GT	--	GT	GG	--	--	GT	GG	GG	GT	TT	GT	GT
AG	--	AG	GG	--	--	GG	AG	GG	AG	AG	AG	GG
GG	GG	GG	AG	AG	--	AG	AG	GG	AG	GG	GG	AG
AA	--	AC	AC	--	--	AC	CC	AA	CC	AC	AC	AC

AT	AT	AT	AT	AT	--	AT	AA	TT	AA	AT	AT	AT
AT	AT	AA	AT	--	--	AT	AA	TT	AA	AT	AT	AT
AT	--	AT	AT	AT	AA	AT	TT	AA	TT	AT	TT	--
CT	--	CT	CT	CT	--	CC	CC	TT	CC	CT	CC	CT
AG	AA	AG	AG	AG	AG	AA	AA	GG	AA	AG	AA	GG
AC	CC	AC	AC	AA	AC	CC	CC	AA	CC	AC	CC	AA
CT	--	CT	CT	TT	CT	CC	CT	TT	CC	CT	CC	TT
AG	AG	AG	AG	GG	AG	AA	AG	GG	AA	AG	AG	GG
GT	GT	GT	GT	GG	GT	TT	GT	GG	TT	TT	GT	GG
AG	--	AG	AG	--	--	AA	AG	GG	AA	AA	AG	GG
CC	CC	CC	CC	CC	--	CG	CG	CC	CG	CG	CG	CC
GG	GG	GG	GG	GG	GG	AG	AG	GG	AG	AG	AG	GG
CC	CG	CG	CG	CC	CC	GG	CG	CC	GG	GG	CG	CC
AA	AG	AG	AG	AA	AA	GG	AG	AA	GG	GG	AG	AA
GG	AG	AG	AG	--	--	AG	AG	GG	AA	AA	AG	GG
AA	AG	AG	AG	AA	AA	AG	AG	AA	GG	GG	GG	AA
GG	GG	GG	GG	GG	--	GT	GT	GG	GT	GT	GT	GG
GG	AG	GG	AG	GG	GG	AG	AG	GG	AA	AA	AA	AG
TT	TT	TT	TT	TT	TT	GT	GT	TT	GT	GT	GT	TT
GG	CG	GG	GG	--	--	CG	CG	GG	CC	CC	CC	CG
CT	--	TT	CT	TT	TT	TT	TT	CT	CT	CT	CT	CT
CC	--	GG	CG	GG	CG	CG	CG	CG	CG	CG	CC	CG
CG	CG	CC	CC	CC	--	CG	CG	CC	CC	CC	CG	CC
AC	AA	AA	AC	--	AA	AC	AC	AC	AC	AC	AC	AC
CC	--	CC	CT	CC	CC	CT	CT	CT	CT	CT	CT	CT
AA	AA	AA	AG	--	--	AG	AG	AG	AG	AG	AG	AG
CC	CC	CC	CT	CC	CC	CT	CT	CT	CT	CT	CT	CT
CG	GG	CG	GG	GG	--	GG	CG	GG	CG	GG	GG	GG
CC	CC	CC	CT	--	--	CT	CC	CC	CT	CT	CT	CT
AG	AA	AG	AG	AA	AA	AA	GG	AA	GG	AG	AG	AG
GG	GG	GG	GT	GG	--	GG	GT	GG	GT	GG	GT	GG
TT	TT	GT	GT	--	--	GT	GG	TT	GG	TT	TT	TT
GG	--	GG	GG	--	--	--	CG	GG	CG	GG	GG	--
GG	GG	CG	CG	--	--	CG	CC	GG	CC	GG	GG	GG
GG	--	GG	AG	GG	--	AG	AG	GG	AG	GG	GG	GG
TT	--	CT	CT	--	--	CT	CC	TT	CC	TT	TT	TT
GG	--	GT	GT	GG	GG	GT	GT	GG	TT	GG	GG	GG
GG	GG	GG	AG	GG	GG	AG	AG	GG	AG	GG	GG	GG
AA	AA	AG	AG	--	--	AG	GG	AA	GG	AA	AA	AA
TT	--	--	CT	TT	TT	CT	CC	TT	CC	TT	TT	TT
TT	--	TT	AT	--	--	AT	AT	TT	AT	TT	TT	TT
TT	TT	--	GT	--	--	GT	GT	TT	GT	TT	TT	TT
CC	--	CT	CT	--	--	CT	TT	CC	TT	CC	CC	CC
AA	AA	AA	AG	--	AA	AG	AG	AA	AG	AA	AA	AA
TT	--	AT	AT	--	--	AT	AT	TT	AA	TT	TT	TT
AA	AA	AA	AG	AA	AA	AG	AG	AA	AG	AA	AA	AA
TT	TT	TT	CT	TT	TT	CT	CT	TT	CT	TT	TT	TT

TT	--	TT	AT	TT	--	AT	AT	TT	AT	TT	TT	TT
GG	--	GT	GT	GG	GG	GT	TT	GG	TT	GG	GG	GG
AG	AA	AG	AG	AA	AA	AG	GG	AA	GG	AA	AA	AA
CT	--	CT	CT	CC	CC	CT	TT	CC	TT	CC	CC	CC
CT	CC	CT	CC	CC	CC	CT	TT	CC	TT	CC	CC	CC
CT	--	CT	CT	CC	CC	CT	TT	CC	TT	CC	CC	--
GT	--	GT	GT	GT	GG	GT	TT	GG	TT	GG	GG	GT
CC	CC	CG	CC	CG	--	CC	CG	CC	GG	CC	CC	GG
CC	CC	AC	CC	AC	CC	CC	AC	CC	AC	CC	CC	AA
AA	AG	GG	AA	AG	AA	AA	AG	AA	AG	AA	AA	GG
GG	AG	AA	GG	AG	--	GG	AG	AG	GG	AG	GG	AA
TT	CC	TT	CT	CT	TT	TT	CT	CC	CC	CT	CC	CT
AA	CC	AA	AC	AC	--	AA	AC	CC	CC	AC	CC	AC
TT	--	TT	TT	TT	TT	TT	GT	GT	GT	GT	GT	GT
GG	CG	GG	CG	CG	GG	GG	GG	CG	CG	GG	CG	GG
AG	AA	--	--	AG	GG	GG	AG	AA	AG	AG	AA	AG
TT	CT	TT	CT	TT	TT	TT	TT	CT	TT	TT	CT	TT
GT	--	GT	TT	--	--	TT	GT	GT	GT	GT	GT	GT
CC	CT	CC	CT	CC	--	CC	CT	CT	CC	CC	CT	CC
AC	AC	AC	AA	--	--	AA	AC	AC	AC	AC	AC	AC
AG	GG	AG	AG	AA	AG	AA	GG	GG	AG	AG	GG	AG
AG	AG	AG	AA	--	AA	AA	AG	AG	AG	AG	AG	AG
GG	--	GT	--	--	--	TT	GG	GG	GT	GG	GG	--
TT	--	TT	CC	CC	TT	CC	TT	TT	CT	TT	TT	TT
CC	CC	CC	AA	AA	CC	AA	AC	CC	AC	CC	CC	CC
AA	--	AT	AT	TT	--	TT	AT	AA	AT	AT	AA	AA
GG	--	GT	GG	GT	GG	GT	GT	GG	GG	GT	GG	GG
AA	--	AA	AG	AG	AA	AG	AA	AA	AG	AA	AA	AA
GG	--	CG	CG	--	--	CC	CG	GG	CG	CG	GG	GG
GG	--	AG	AG	AA	GG	AA	AG	GG	AG	AG	AG	GG
CG	--	GG	CC	--	CG	CC	GG	GG	CG	CG	CG	CC
AG	GG	GG	AA	--	--	AA	GG	GG	AG	AG	AG	AA
AA	--	AG	AG	--	--	AA	AG	AG	AG	AA	AA	AA
AT	AT	AT	AA	AT	AT	AA	AA	AT	AA	AT	AT	AA
AA	--	AG	AA	AA	AA	--	AG	AG	AG	AA	AA	--
AG	AA	AA	AG	AG	AG	AG	AA	AA	AA	AG	AG	AG
AC	--	CC	AA	AC	AC	AA	AC	AC	AC	AC	AC	AA
AC	--	AC	CC	AC	--	--	CC	CC	CC	AC	AC	CC
CC	--	AA	CC	AC	AC	AC	AC	AC	AC	AC	AC	CC
GG	TT	TT	GG	GT	--	GT	GT	GT	GT	GT	GT	GG
AA	TT	TT	AA	AT	AA	AT	AT	AT	AT	AA	AT	AA
AA	AG	AG	AA	AG	AA	AG	AA	AA	AA	AA	AG	AA
CC	--	AC	CC	AC	CC	AC	CC	CC	CC	AC	AC	CC
AA	TT	TT	AA	--	--	AT	AT	AT	AT	AA	AT	AA
CC	CT	TT	CT	CT	CC	CT	CT	CT	CT	CC	CT	CC
GG	AG	AA	AG	AG	GG	AG	GG	AG	AG	GG	AA	GG
CC	CT	TT	CT	CT	CC	CT	CT	CT	CT	CC	TT	CC

TT	--	CC	CT	CT	TT	CT	CT	CT	CT	TT	CC	TT
GG	AG	AA	AG	AG	--	AG	AG	AG	AG	GG	AA	GG
CC	CT	TT	CT	CT	CC	CT	CT	CT	CT	CC	TT	CC
TT	CT	CC	CT	CT	TT	CT	CT	CT	CT	TT	CC	TT
CC	--	TT	CT	CT	--	CT	CT	CT	CT	CC	TT	CC
GG	--	AA	AG	AG	--	AG	AG	AG	AG	GG	AA	GG
TT	CT	CC	CT	CT	--	CT	CT	CT	CT	TT	CC	TT
--	AG	AA	AG	AG	--	AG	AG	AG	AG	GG	AA	GG
CC	--	GG	CG	CG	--	GG	CG	CG	CG	CC	GG	CC
AA	AC	CC	AC	AC	AA	CC	AC	AC	AC	AA	CC	AA
CC	CT	TT	CT	CT	CC	TT	CT	CT	CT	CC	TT	CC
CC	CT	--	CT	CT	--	TT	CT	CT	CT	CC	TT	CC
TT	AT	AA	AT	AT	TT	AA	AT	AT	AT	TT	AA	TT
CC	--	TT	CT	--	CC	TT	CT	CT	CT	CC	TT	CC
AA	AC	CC	AC	AC	--	CC	AC	AC	AC	AA	CC	AA
TT	GT	GG	GT	GT	TT	GG	GT	GT	GT	TT	GG	TT
TT	CT	CC	CT	CT	--	CC	CT	CT	CT	TT	CC	TT
GG	CG	CC	CG	CG	GG	CC	CG	CG	CG	GG	CC	GG
GG	CG	--	--	CG	--	CC	CG	CG	GG	GG	CC	GG
CC	AC	AA	AC	AC	CC	AA	AC	AC	CC	CC	AA	CC
GG	CG	CC	CG	CG	--	CC	CG	CG	GG	GG	CC	GG
AT	AT	AT	TT	AT	--	AT	AA	AT	AT	AT	TT	AA
AG	AG	AG	GG	--	--	AG	AA	AG	AG	AG	GG	AA
AA	AA	AG	AG	AG	AG	AA	AG	GG	AG	AG	GG	AA
GG	--	AG	AG	--	AG	GG	AG	AA	AG	AG	AA	AG
AA	--	AG	AG	--	AG	AA	AG	GG	AG	AG	GG	AG
AA	AA	AC	AC	AA	AC	AA	AC	CC	AC	AC	CC	AC
AA	AA	AG	AG	--	AG	AA	AG	GG	AG	AG	GG	AG
AA	AA	AG	AG	AA	AG	AA	AG	GG	AG	AG	GG	AG
GG	GG	GT	GT	--	GT	GG	GT	TT	GT	GT	TT	GT
TT	TT	GT	GT	--	--	TT	GT	GG	GT	GT	GG	GT
CC	CC	AC	AC	CC	AC	CC	AC	AA	AC	AC	AA	AC
TT	TT	CT	CT	--	CT	TT	CT	CC	CT	CT	CC	CT
TT	--	CT	CT	--	CT	TT	CT	CC	CT	CT	CC	CT
GG	--	GT	GT	GG	GT	GG	GT	TT	GT	GT	TT	GT
AA	--	AT	AT	--	AT	--	AT	TT	AT	AT	TT	AT
GG	GG	CG	CG	GG	CG	GG	CG	CC	CG	CG	CC	CG
AA	--	AC	AC	AA	AC	AA	AC	CC	AC	AC	CC	--
CC	--	CT	CT	CC	CT	CC	CT	TT	CT	CT	TT	CT
GG	--	CG	CG	--	CG	GG	CG	CC	CG	CG	CC	CG
CC	--	CT	CT	--	CT	CC	CT	TT	CT	CT	TT	CT
AA	AA	AT	AT	AA	AT	AA	AT	TT	AT	AT	TT	AT
TT	--	CT	CT	--	--	TT	CT	CC	CT	CT	CC	CT
GG	--	AG	AG	--	AG	GG	AG	AA	AG	AG	AA	AG
AA	--	AT	AT	--	TT	AA	AT	TT	AT	AT	TT	AT
AA	AA	AT	AT	AA	AT	AA	AT	TT	AT	AT	TT	AT
AA	AA	AC	AC	AA	AC	AA	AC	CC	AC	AC	CC	AC

TT	--	GT	GT	--	GT	TT	GT	GG	GT	GT	GG	GT
GG	GG	AG	AG	--	AG	GG	AG	AA	AG	AG	AA	AG
GG	--	GT	GT	GG	GT	GG	GT	TT	GT	GT	TT	GT
AA	--	AG	AG	--	--	AA	AG	GG	AG	AG	GG	AG
TT	--	CT	CT	TT	CT	TT	CT	CC	CT	CT	CC	CT
AA	AA	AG	AG	AA	AG	AA	AG	GG	AG	AG	GG	AG
TT	--	CT	CT	--	--	TT	CT	CC	CT	CT	CC	CT
CC	--	AC	AC	--	AC	AC	AC	AA	AC	AC	AA	AC
AA	--	AG	AG	--	AG	AG	AG	GG	AG	AG	GG	AG
TT	--	CT	CT	--	CT	CT	CT	CC	CT	CT	CC	CT
GG	GG	AG	AG	GG	--	AA	AG	AA	AG	AG	AA	AG
CC	--	CT	CT	--	TT	TT	CT	TT	CT	CT	TT	--
GG	GG	GT	GT	GG	--	GT	--	GT	GT	GT	GT	GG
AG	GG	AG	GG	--	AG	AG	AG	GG	GG	AG	AG	AG
AT	--	AA	AT	AT	AT	AA	AT	TT	AA	AA	AT	--
AT	TT	AA	AT	--	AT	AA	AT	TT	AA	AA	AT	AA
AC	--	AA	AC	AC	AC	AA	AC	CC	AA	AA	AC	AA
GT	GG	--	GT	--	--	TT	GT	GG	TT	TT	GT	TT
CT	--	CT	CT	CT	TT	TT	CT	--	CT	TT	TT	CT
AA	--	AG	AA	AG	AG	GG	AA	AG	AG	GG	GG	AA
GG	GG	CG	GG	CG	--	CG	GG	CG	GG	CG	CG	GG
TT	TT	CT	--	CT	--	CT	TT	CT	TT	CT	CT	TT
GG	GT	GG	GG	GG	GT	GT	GG	GG	GT	GT	GT	GG
AA	AG	AG	AA	AG	--	GG	AA	AG	AG	AG	AG	--
AA	AA	AC	AA	--	--	AC	AA	AC	AA	AA	AA	AA
GG	--	CG	GG	CG	--	CG	GG	CG	GG	GG	GG	GG
TT	--	GT	TT	--	--	GT	TT	GT	TT	TT	TT	TT
CC	CT	CT	CC	TT	CC	TT	CC	CT	CT	CT	CT	CC
AG	GG	AG	AG	AG	AG	GG	AG	AA	GG	GG	GG	AA
CC	CC	CC	CC	CT	CC	CC	CC	CT	CC	CT	CC	--
CG	CG	CG	CG	CC	CG	CG	CG	CC	CG	CC	CG	CC
AA	AA	AA	AC	AA	AC	AA	AC	AA	AA	AA	AA	AC
AG	AG	AG	GG	AG	GG	AG	GG	AA	AG	AA	AG	AG
CT	--	CT	TT	CT	TT	CT	TT	CT	CT	CT	CT	TT
GG	GG	GG	GG	--	--	GG	GG	CG	GG	CG	GG	CG
AG	AG	AG	AA	GG	--	AG	AA	GG	AG	GG	AG	AG
CG	--	CG	CG	--	CG	CG	CG	GG	CG	GG	CG	GG
CG	CG	CG	CC	--	--	CG	CC	GG	CG	GG	CG	CG
CT	CT	CT	CC	--	CC	CT	--	TT	CT	TT	CT	CT
AG	AG	AG	GG	--	--	AG	GG	AA	AG	AA	AG	AG
GG	GG	GG	AG	GG	AG	GG	AG	GG	GG	GG	GG	AG
CT	CT	CT	CC	--	CC	CT	CC	TT	CT	TT	CT	CT
AG	AG	AG	GG	AG	GG	AG	GG	AG	AG	AG	AG	GG
AG	AG	AG	GG	AG	GG	AG	GG	AA	AG	AA	AG	AG
AC	AC	AC	CC	--	--	AC	--	AA	AC	AA	AC	AC
TT	TT	TT	TT	--	--	TT	TT	AT	TT	AT	TT	AT
AG	AG	AG	AA	AG	--	AG	AA	GG	AG	GG	AG	AG

AC	--	AC	AA	AC	--	AC	AA	CC	AC	CC	AC	AA
AG	AG	AG	AA	AG	--	AG	AA	GG	AG	GG	AG	AA
AG	AG	AG	AG	AG	AA	AG	AA	AG	AG	AG	AG	--
CC	CC	CC	CC	CC	--	CC	CC	CT	CC	CT	CC	CC
CC	CC	CC	CC	CC	CC	CC	CC	CG	CC	CG	CC	CC
TT	--	TT	CT	TT	--	TT	CT	CT	TT	CT	TT	TT
CG	--	CG	GG	CG	--	CG	--	GG	CG	GG	CG	CG
CT	CC	CC	CT	CC	--	CC	CC	CT	CT	CT	CC	CC
GG	GT	GT	GG	GT	GG	GT	GT	GG	GG	GG	GT	GT

3-70	3-71	3-75	3-78	4-4	4-5	4-6	4-7	4-8	4-11	4-12	4-13	4-15	4-16
CC	AA	CC	AC	AA	AC	AC	AC	AA	AA	AC	AA	CC	CC
AG	AA	GG	AG	AA	AG	AG	AG	AA	AA	AG	AA	GG	GG
CT	CT	CC	CT	TT	CT	CC	TT	TT	--	CT	CT	--	CC
AG	AG	GG	AG	GG	AG	AG	GG	GG	GG	--	GG	AG	AG
AG	AG	GG	AG	AA	AG	GG	AA	AA	--	GG	AG	--	--
CG	CG	GG	CG	CG	CG	GG	CG	CG	CG	--	GG	--	GG
CT	CT	CC	CT	TT	CT	CC	TT	TT	TT	--	CT	--	CC
GT	GT	GG	GT	TT	GT	GG	TT	TT	TT	GG	GT	GG	GT
GT	GT	GT	GT	TT	GT	GG	TT	TT	GT	GG	GT	GG	GT
GT	GT	GT	GT	TT	GG	GG	TT	TT	GT	GG	GT	GG	GT
AG	AG	AG	AG	AA	GG	GG	AA	AA	AA	GG	AG	GG	AG
CT	CT	CT	CT	CT	--	CC	CT	CC	CT	--	--	--	CC
AC	AC	AC	AC	--	CC	CC	AA	AC	AC	AC	AA	CC	AA
AG	AG	AG	AG	AG	AA	AA	AG	AA	AG	AA	AG	AA	AG
CC	CC	CC	CC	CT	CC	CC	CT	CT	CC	CT	CT	--	CT
GG	AG	AG	AG	GG	AA	AA	GG	AG	AG	--	GG	AA	GG
AT	AT	AT	AT	AT	TT	TT	AT	TT	AT	AT	AT	TT	AT
CT	CC	CC	CC	CT	CC	CC	CT	CT	CC	CT	CT	--	CT
GT	GT	GT	GT	GT	TT	GT	GT	TT	GT	GT	GT	TT	GT
CG	CG	CG	CG	CC	CC	CG	CG	CC	CG	CG	CG	CC	CC
AA	AT	TT	TT	AT	TT	AT	--	AT	AT	--	AA	--	AT
CC	CT	TT	TT	CT	TT	CT	CT	CT	--	--	CC	--	CT
TT	AT	AA	AA	AT	AA	AT	AT	AA	--	TT	TT	AT	AT
TT	AT	AA	AA	AT	AA	AT	AT	AA	AA	TT	TT	AT	AT
GG	AG	AA	AA	AG	AA	AG	AG	AA	AA	GG	GG	AG	AG
GT	GT	--	TT	TT	TT	GT	--	TT	TT	GT	GT	GT	TT
GG	GT	TT	TT	GT	TT	GT	GT	GT	TT	GG	GG	GT	GT
AA	AT	TT	TT	AT	TT	AT	AT	AT	TT	AA	AA	AT	AT
CC	CT	TT	TT	CT	TT	CT	CT	TT	TT	CC	CC	CT	CT
GG	CG	CC	CC	CG	CC	CG	CG	CC	CC	--	GG	CG	CG
TT	CT	CC	CC	CT	CC	CT	CT	CC	CC	TT	TT	CT	CT
CC	AC	AA	AA	--	AA	AC	AC	AA	AA	CC	AC	AC	AC
GG	AG	AA	AA	AG	AA	AG	AG	AA	AA	--	GG	--	AG
TT	GT	GG	GG	GT	GG	GT	GT	GG	GG	--	TT	GT	GT
GG	AG	AA	AA	AG	AA	AG	AG	AA	AA	GG	GG	--	AG
GG	GT	TT	TT	GT	TT	GT	GT	--	TT	--	GG	GT	GT
AA	AG	--	GG	AG	GG	AG	AG	GG	GG	AA	AA	AG	AG
TT	CT	CC	CC	CT	CC	CT	CT	CC	CC	TT	TT	CT	CT
TT	AT	AA	AA	AT	AA	AT	AT	AA	AA	--	TT	AT	AT
GG	AG	AA	AA	AG	AA	AG	AG	AA	AA	--	--	AG	AG
CC	AC	AA	AA	AC	AA	AC	AC	AA	AA	CC	CC	AC	AC
GG	AG	AA	AA	AG	AA	AG	AG	--	AA	--	--	AG	AG

CC	CT	TT	TT	CT	TT	CT	CT	TT	TT	--	--	--	CT
GG	CG	CC	CC	CG	CC	CG	CG	CC	CC	GG	GG	CG	GG
CC	CT	CT	CT	CT	TT	CT	CT	--	TT	CC	CC	CT	CT
TT	CT	CT	CT	CT	CC	CT	CT	CC	CC	--	TT	CT	CT
CC	AC	AC	AC	AC	AA	AA	AC	AA	AA	CC	CC	AC	AC
TT	CT	--	CT	CT	CC	CC	CT	CT	CT	--	TT	CT	TT
CC	CT	CT	CT	CT	TT	TT	CT	CT	CT	CT	CC	--	CC
AG	AA	AA	AG	AG	AG	GG	AG	GG	AG	AA	GG	--	GG
GT	TT	TT	GT	GT	GT	GT	GT	GT	GT	TT	GG	TT	GG
--	AA	AA	AG	AG	AG	AG	AA	AG	AG	AA	GG	AG	AG
CT	CC	CC	CT	CT	CT	CT	CC	CT	CC	--	TT	CT	CT
CG	GG	GG	GG	CG	CG	CG	GG	CG	GG	GG	CG	CG	CG
AG	GG	GG	GG	AG	AG	AG	GG	AG	GG	GG	AG	AG	AA
AC	AA	AA	--	AC	AC	AC	AA	--	AA	AA	AC	AC	CC
AG	GG	GG	GG	AG	GG	AG	GG	AG	GG	GG	AG	GG	AG
CT	CC	CC	CC	CT	CT	CT	CC	--	CC	CC	CT	CC	TT
CT	TT	TT	CT	CT	CT	CT	TT	CT	TT	TT	CT	TT	CC
CT	CC	CC	CT	CT	CT	TT	CC	CT	CC	--	CT	CC	TT
AG	GG	GG	AG	AG	AG	AA	GG	AG	GG	AG	--	--	AA
AC	CC	CC	AC	AC	AC	AA	AC	AC	CC	AC	AC	CC	AA
AG	GG	GG	AG	AG	GG	AA	AG	AG	GG	AA	AG	GG	AA
CG	CC	CC	GG	CG	CC	GG	CG	CG	CC	GG	CG	CC	GG
GG	GG	CG	CC	CG	GG	CC	CG	GG	GG	CC	CG	GG	CC
GG	GG	GG	AA	AG	GG	AA	GG	GG	GG	AA	AG	GG	AA
AA	AC	AA	CC	AC	AA	CC	AA	--	AA	--	AC	AA	CC
CC	CT	CC	TT	CT	CC	TT	CC	--	CC	--	CT	CC	TT
TT	CT	TT	CC	CT	TT	CC	CT	--	TT	CC	CT	TT	CC
AA	AT	AA	TT	AT	AA	TT	AT	AA	AA	TT	AT	AA	TT
AG	GG	AG	GG	GG	GG	GG	GG	AG	AG	GG	GG	--	--
AT	AT	AT	TT	AT	AT	TT	AT	AT	AT	TT	AT	--	TT
TT	GG	TT	GT	GT	GT	TT	--	--	TT	--	GT	--	TT
--	AG	GG	GG	AG	AG	GG	AG	AG	AG	--	AG	--	GG
TT	CT	TT	TT	CT	CT	TT	CT	CT	CT	TT	CT	TT	TT
GG	AG	GG	GG	AG	GG	GG	AG	AG	GG	--	AG	--	GG
GG	TT	GG	GT	GT	GG	GG	GT	TT	--	GG	GT	GT	GG
GG	GT	GG	GG	GT	GG	GG	GT	GT	GG	GG	GG	--	--
TT	CT	TT	CT	CT	TT	TT	CT	CC	TT	TT	CT	CC	TT
--	CT	TT	CT	CT	TT	TT	CT	--	TT	--	CT	--	CT
AA	AG	AA	AG	AG	AA	AA	AG	GG	AA	AA	AG	GG	AG
CG	CG	CG	GG	CG	GG	GG	CG	CC	GG	--	CG	--	CG
AG	AG	GG	AA	AG	AA	AA	AG	GG	AA	AA	AG	GG	AG
CT	CT	TT	CC	CT	CC	CC	CT	CT	CC	--	--	--	CT
CT	CT	TT	CT	CT	CT	CC	TT	CT	CC	--	CT	CT	CT
CC	CC	CG	CG	CG	CG	CC	CG	CG	CC	CC	CC	CG	CG
TT	TT	AT	AT	AT	AT	TT	AT	AT	TT	--	TT	AT	AT
GT	GT	GT	GG	GG	GG	GG	GT	GG	GG	--	GT	GG	GG
CT	CT	CT	CC	CC	CC	CC	CT	CC	CC	CC	CT	CC	CC

CT	CT	TT	CT	CT	CT	CC	CT	CT	--	--	CT	CC	CT
GT	GT	GT	GG	GG	GG	GG	GT	GG	GG	GG	GT	GG	GG
CT	CT	TT	CT	CT	CT	CC	CT	CT	CC	CC	CT	CC	CT
GT	GT	GT	TT	TT	TT	TT	GT	TT	TT	TT	GT	TT	TT
CG	CG	CG	GG	GG	GG	GG	CG	GG	GG	--	CG	--	GG
AG	AG	AA	GG	AG	GG	AG	AG	AA	--	AG	--	--	AA
AG	AG	GG	AA	AG	AA	AG	AG	GG	AA	AG	AG	AA	GG
AG	AG	GG	AA	AG	AA	AG	AG	GG	AA	AG	AG	AA	GG
CT	CT	CC	TT	TT	TT	CT	CT	CC	TT	CT	CT	--	CC
AG	AG	AA	AG	GG	GG	GG	AG	AA	GG	AG	--	GG	AA
GT	GG	TT	GT	GG	GG	GG	GT	TT	GG	GT	GG	--	--
GG	GG	AG	GG	AG	AG	AG	GG	GG	AG	--	AG	AG	GG
CT	CT	CT	CT	TT	TT	TT	CT	CC	TT	TT	TT	--	CC
CC	CC	AC	CC	AC	CC	CC	CC	AC	--	--	CC	--	AC
CT	CT	CC	CT	CT	CT	CT	CT	CC	CT	CT	CT	CT	CC
GT	GT	GT	GT	GT	GG	GG	GT	TT	GG	--	GG	--	TT
CT	CT	CC	CT	CC	CC	CC	CT	CT	CC	--	CC	CT	CT
GG	GG	CG	GG	CG	GG	GG	GG	CG	GG	GG	GG	GG	CG
--	CC	AC	CC	AC	CC	CC	CC	AC	CC	--	CC	--	AC
CT	CT	TT	CT	TT	TT	TT	CT	CT	TT	TT	TT	CT	CT
AG	AG	AA	AG	AA	AA	AA	AG	AG	--	AA	AG	AG	AG
AG	AG	AG	AG	AG	AA	AA	AG	GG	AA	--	AG	AG	GG
CT	CT	CC	CT	CC	CT	CT	CT	CC	CT	CT	CT	CT	CC
AA	AA	AT	AA	AT	AA	AA	AA	AT	AA	--	AA	--	AT
CC	CC	CT	CC	CT	CC	CC	CC	CT	CC	CC	CC	CC	CT
AA	AA	AG	AA	AG	AA	AA	AA	AG	AA	AA	AA	AA	AG
CT	CT	CC	CT	CC	CC	CC	CT	CT	CC	CC	CT	CT	CT
AT	AT	AT	AT	AT	TT	TT	AT	AA	TT	--	AT	AT	--
AT	AT	AT	AT	AT	TT	TT	AT	AA	--	--	--	AT	AA
CT	CT	CT	CT	CT	TT	TT	CT	CC	TT	TT	CT	CT	CC
CC	CC	CT	CC	CT	CC	CC	CC	CT	CC	CC	CC	--	CT
GT	GT	GG	GT	GG	GG	GG	GT	GT	--	--	GT	GT	GT
CT	CT	CC	CT	CC	CC	CC	CT	CT	CC	CC	CT	CT	CT
AA	AA	AG	AA	AG	AG	AG	AA	--	--	AG	--	AA	AA
AG	AG	AG	AG	AG	GG	GG	AG	AA	GG	--	AG	AG	AA
AG	AA	AA	AG	AA	AA	AA	AG	AG	AA	AA	AG	AG	AG
CT	CC	CT	--	CT	CC	CC	CT	TT	CC	--	CT	CT	TT
AC	CC	CC	AC	CC	CC	CC	AC	AC	CC	--	AC	AC	AC
AT	TT	AT	AT	AT	TT	TT	AT	AA	TT	--	AT	AT	AA
AA	AA	AG	AA	AG	AA	AA	AA	AG	AA	AA	AA	--	AG
AC	AA	AA	AC	AA	AA	AA	AC	AC	AA	AA	AC	AC	AC
AT	TT	TT	AT	TT	TT	TT	AT	AT	TT	TT	AT	AT	AT
AA	AC	CC	AA	CC	AC	AC	AA	AC	AC	AC	AA	AA	AC
CC	CC	AC	CC	AC	CC	CC	CC	AC	CC	CC	CC	--	AC
GG	AG	AG	GG	AG	AG	AG	GG	GG	AG	AG	GG	AG	GG
AC	AC	CC	AC	AC	AC	AC	AC	CC	AC	AC	AC	AC	CC
AG	AA	--	AG	AA	AA	AA	AG	AG	AA	AA	AG	--	AG

CC	CC	CG	CC	CG	CC	CC	CC	CG	CC	CC	CC	CC	CG
CT	TT	CT	CT	CT	TT	TT	CT	CC	TT	TT	CT	--	CC
CT	CC	CT	CT	CT	CC	CC	CT	TT	CC	CC	CT	--	TT
--	CC	CC	CT	CC	CC	CC	CT	CT	CC	--	CT	--	CT
AG	AA	AA	AG	AA	AA	AA	AG	AG	AA	--	AG	--	AG
CT	CC	CT	CT	CT	CC	CC	CT	TT	CC	--	CT	--	TT
CT	TT	CT	CT	TT	TT	TT	CT	CT	TT	TT	CT	--	CT
AT	AA	TT	AT	AT	AT	AA	--	--	--	--	AT	--	TT
GG	GG	AG	GG	AG	GG	GG	GG	AG	GG	GG	GG	GG	AG
AC	AA	AA	AC	AA	AC	AA	AC	AC	AA	AC	AC	AA	AC
--	CC	CT	CT	CC	CT	CC	CT	CT	--	--	CT	--	CT
TT	TT	GT	TT	GT	TT	TT	TT	--	TT	--	TT	--	GT
CT	TT	CT	CT	TT	CT	TT	CT	CT	TT	CT	CT	--	CT
CC	AC	CC	CC	AC	CC	AC	CC	CC	AC	CC	CC	AC	CC
AG	AA	AG	AG	AA	AG	AA	AG	AG	AA	AG	AG	--	AG
GT	GG	GT	GT	GG	GT	GG	GT	GT	GG	GT	GT	--	GT
TT	AT	TT	TT	AT	TT	AT	TT	TT	--	TT	TT	AT	TT
AG	AA	AG	AG	AA	AG	AA	AG	AG	--	AG	AG	AA	AG
CC	CC	CT	CC	CT	CC	CC	CC	CT	CC	--	CC	--	CT
CT	CC	CT	CT	CC	CT	CC	CT	CT	CC	CT	CT	--	CT
AC	AA	AC	AC	AA	AC	AA	AC	AC	AA	AC	AC	AA	AC
CT	CC	CT	CT	CC	CT	CC	CT	CT	CC	CT	CT	CC	CT
AT	AA	TT	AT	AT	AT	AA	AT	TT	AA	--	AT	--	TT
AG	GG	AA	AG	AG	AG	GG	AG	AA	GG	--	AG	GG	AA
GG	GG	CG	GG	CG	GG	GG	GG	--	GG	GG	GG	--	CG
CT	TT	CT	CT	TT	CT	TT	--	CT	TT	CT	CT	--	CT
AG	GG	AG	AG	GG	AG	GG	AG	AG	GG	AG	AG	--	AG
AG	AA	AG	AG	AA	AG	AA	AG	AA	AA	AG	--	AA	AG
AA	AA	AG	AA	AG	AA	AA	AA	AG	AA	AA	AA	AA	AG
GG	GG	AG	GG	AG	GG	GG	GG	AG	GG	--	GG	GG	AG
CT	CT	CT	TT	TT	TT	CT	TT	TT	TT	CT	CT	TT	CT
GT	GT	TT	GG	GT	GG	GT	GG	GT	--	--	GT	--	TT
TT	TT	GT	TT	GT	TT	TT	TT	GT	TT	TT	--	TT	GT
AG	AG	AA	AG	AG	GG	AG	GG	GG	GG	AG	AG	--	AA
CT	CT	CT	CC	CC	CC	CT	CC	CC	CC	--	CT	CC	CC
GT	GT	GT	TT	TT	TT	GT	TT	TT	TT	GT	GT	TT	TT
AG	AG	GG	AG	GG	AG	AG	AG	GG	AG	AG	AG	AG	GG
AG	AA	AG	AG	AA	AG	AG	AG	AG	AG	AG	AG	AA	AG
CT	CT	CC	CC	CT	CC	CT	CT	CC	CC	CT	CC	CC	CT
AT	AA	AA	AA	AA	AA	AT	AT	AA	AA	AT	AA	--	AA
CG	CC	CG	CG	CC	CG	CC	CG	CG	CG	--	CG	CG	CC
AA	AC	AA	AC	AA	AA	AA	AA	AC	AC	AA	AC	AC	AA
AG	AG	AA	AG	AG	AA	AA	AG	AG	AG	AA	AG	AG	AA
GG	AG	AG	AG	GG	AG	AG	GG	AG	AG	AG	AG	AG	AG
CT	CT	CT	CT	CC	CT	CT	CC	CT	CT	CT	CT	CT	CT
GG	AG	GG	AG	AA	GG	AG	AG	AG	GG	GG	AG	AG	AG
CC	CT	CC	CT	CT	CC	CT	CC	CT	CC	CC	CT	CT	CT

GG	GG	GG	GG	AG	GG	GG	AG	GG	GG	GG	GG	GG	GG
AG	GG	AG	GG	AG	AG	GG	AA	GG	AG	AG	GG	--	GG
CC	CT	CC	CT	CT	CC	CT	CT	CT	CC	CC	CT	TT	CT
AA	AT	AA	AT	AT	AA	AT	AT	AT	AA	AA	AT	TT	AT
AA	AC	AA	AC	AC	AA	AA	AC	AC	AA	AA	AC	CC	AC
TT	CT	TT	CT	CT	TT	TT	CT	CT	TT	TT	CT	--	CT
GG	AG	GG	AG	AG	GG	GG	AG	GG	GG	--	AG	--	AG
CC	CT	CC	CT	CT	CT	CC	CT	CC	CC	CC	CT	--	CT
TT	CT	TT	TT	TT	TT	TT	TT	TT	TT	--	CT	--	CT
AA	AG	AA	AA	AG	AG	AA	AG	AA	AA	AA	AG	AG	AG
GG	AG	AG	GG	AG	AA	AG	AA	AA	GG	--	GG	--	AG
TT	CT	--	TT	CT	CC	CT	CC	--	TT	CT	TT	--	CT
AG	GG	AG	AG	AG	GG	AG	AG	GG	AG	--	--	--	--
TT	CT	TT	TT	CT	CC	CT	CT	CC	TT	CT	TT	--	CC
CG	CG	GG	CG	GG	GG	GG	GG	GG	GG	--	CG	--	GG
TT	CT	CT	TT	CT	CC	CT	CT	CC	--	CT	TT	--	CC
CT	CC	CT	CC	CT	CC	CT	CT	CC	CT	CT	CT	CC	CC
AA	AG	AG	AG	AG	GG	AG	AG	AG	AG	AG	AA	--	GG
TT	TT	CT	CT	CT	CC	CT	CT	CT	CT	--	TT	--	CC
GG	GG	AG	AG	AG	AG	GG	AG	AG	AG	AG	GG	AA	AA
CT	TT	CT	CT	CT	CT	TT	CT	CT	CT	CT	TT	CC	CC
AG	AA	AG	AG	AG	AG	AA	AG	AA	AG	AG	AA	AG	GG
GG	AG	AG	GG	AG	AG	AG	AG	GG	AG	AG	AG	AG	GG
GT	GG	GT	GT	GT	GT	GG	GT	GG	GT	GT	GT	GT	TT
CC	CG	CG	CC	CG	CG	CG	CG	CG	CG	CG	CC	CG	CG
CC	CC	CG	CC	CG	CG	CC	CG	CC	CG	CG	CC	CC	CG
CT	TT	CT	CT	CT	CT	CT	CT	TT	CT	--	CT	--	CT
CC	CC	CT	CT	CT	CT	CT	CT	CC	CT	CT	CT	CC	CT
TT	TT	AT	AT	AT	AT	AT	AT	TT	AT	AT	AT	--	AT
AA	AA	GG	AA	AG	AG	AG	GG	AA	GG	GG	AG	AG	AG
TT	TT	CC	TT	CT	CT	CT	CC	TT	CC	--	TT	--	CT
CT	CT	TT	CT	CT	CT	TT	TT	CT	TT	TT	CT	TT	CT
CC	CC	AA	CC	AC	AC	AC	AA	CC	AA	AA	CC	AC	AC
GT	GT	GT	GT	GT	GT	GT	GT	TT	TT	GT	GT	GT	GG
AG	AG	AG	AG	AG	AG	AG	AG	AA	AA	AG	AG	AG	GG
CT	CT	CT	CT	CT	CT	CT	CT	TT	TT	--	--	--	CC
AT	AT	AT	AT	AT	AT	AT	AT	--	AA	AT	AT	AT	TT
AC	AC	AC	AC	AC	AC	AC	AC	CC	CC	AC	AC	AC	AA
CG	CG	CG	CC	CG	CG	CG	CC	CG	CG	CC	CC	CC	CC
AG	AG	AG	AA	AG	AG	AG	AA	AG	AG	AA	AA	AA	AA
CT	CT	CT	CT	CT	CT	CT	CT	--	CC	CT	CT	CT	TT
CT	CT	CT	CT	CT	CT	CT	CT	CC	--	TT	--	--	TT
AG	AG	AG	AG	AG	AG	AG	AG	AA	AA	AG	AG	AG	GG
CC	CC	CC	CT	CC	CC	CC	CT	CT	CT	--	CT	CT	CC
AG	AG	AG	AG	AG	AG	AG	--	AA	AA	AG	--	AG	GG
--	AG	AG	AG	AG	AG	AG	GG	GG	GG	AG	AG	--	AA
GT	GT	GT	GT	GT	GG	GT	GG	GG	GG	GT	GG	GT	TT

CT	CT	CT	CT	CT	CT	CT	TT	TT	TT	CT	TT	CT	CC
CG	CG	CG	CG	CG	CG	CG	GG	GG	GG	CG	GG	--	CC
GT	GT	GT	GT	GT	GT	GT	TT	TT	TT	GT	TT	GT	GG
AG	AG	AG	AG	AG	AG	AG	AA	AA	--	AG	AA	--	GG
CT	CT	--	CT	CT	CT	CT	CC	CC	CC	CT	CC	CT	TT
TT	TT	TT	GT	TT	TT	TT	GT	GT	GT	GT	GT	GT	TT
CG	CG	CG	CG	CG	CG	CG	GG	GG	GG	CG	GG	--	--
AG	AG	AG	AG	AG	AG	AG	AA	AA	AA	AG	AA	AG	--
GG	GG	GG	AG	GG	GG	GG	AG	--	AG	AG	AG	--	GG
AC	AC	--	AC	AC	AC	AC	AA	AA	AA	--	AA	AC	CC
CC	CC	CC	CG	CG	CC	CC	CG	CG	CG	CG	CG	CG	CC
AG	AG	AG	AG	GG	AA	AG	GG	GG	GG	AG	GG	AG	AA
AC	AC	CC	AC	AA	CC	AC	AA	AA	AA	AC	--	--	CC
GT	GT	GT	GT	TT	GG	GT	TT	TT	TT	GT	TT	GT	GG
AC	AC	AC	AC	CC	AA	AC	CC	CC	CC	AC	CC	AC	AA
CC	CC	CC	CT	CT	CC	CC	CT	CT	CT	CT	CT	CT	CC
GT	GT	GT	GT	TT	GG	GT	TT	TT	TT	GT	TT	GT	GG
AG	AG	AG	GG	AG	GG	AG	AG	AG	AG	GG	AG	GG	GG
CT	CT	CT	CT	CC	TT	CT	CC	CC	CC	CT	CC	--	TT
CC	CC	CC	CT	CT	CC	CC	CT	CT	CT	CC	CT	CT	--
AT	AT	AT	AT	AA	TT	AT	AA	AA	AA	AT	AA	AT	TT
CG	CG	CG	CG	CC	GG	CG	CC	CC	CC	CG	CC	CG	GG
GT	TT	GT	GT	GG	TT	TT	GG	GG	GG	GT	GG	GG	TT
AG	GG	AG	GG	AG	GG	GG	AG	AG	AG	GG	AG	AG	GG
TT	GT	TT	GT	TT	GT	GT	TT	TT	TT	TT	TT	--	TT
CC	TT	CC	TT	CC	TT	TT	CC	CC	CC	CT	CC	--	CT
CC	AA	CC	AA	CC	AA	AC	CC	CC	CC	AC	CC	CC	AC
CC	CT	CC	CT	CC	CT	CT	CC	CC	CC	CT	CC	--	CT
GG	CG	GG	CC	CG	CC	CG	GG	GG	GG	CG	GG	GG	CG
CC	CT	CT	TT	CT	TT	CT	CC	CC	CC	CT	CC	CC	CT
GT	TT	TT	TT	TT	TT	TT	GT	GT	GT	TT	GT	GT	TT
CT	CT	CT	TT	TT	TT	CT	TT	TT	CT	CT	CT	CT	CT
AT	TT	TT	--	--	TT	TT	AT	AT	AT	--	AT	--	TT
CC	AC	AC	AC	AC	AC	AC	CC	CC	CC	AC	CC	CC	AC
CT	CC	CC	CC	CC	CC	CC	CT	CT	CT	--	--	CT	CC
AA	AG	AG	AG	AG	AG	AG	AA	AA	AA	AG	--	AA	AG
GG	AG	AG	AG	AG	AG	AG	GG	GG	GG	AG	GG	GG	AG
CG	CG	GG	CG	GG	GG	GG	GG	CG	GG	--	GG	--	GG
CT	CT	--	CT	TT	CT	CT	CT	CT	CC	CC	CT	--	CT
CT	CC	CT	TT	TT	CT	TT	CT	TT	CC	CC	CT	CC	CT
CT	CC	TT	TT	TT	CT	TT	CT	TT	CT	TT	CT	CT	CC
CT	TT	CC	CC	CC	CT	CC	CT	CC	CT	--	--	CT	CT
GT	GG	TT	TT	TT	GT	TT	GT	TT	GT	TT	TT	GT	GT
CT	TT	CC	CC	CC	CT	CC	CC	CC	CT	--	--	CT	CT
AT	TT	AA	AA	AA	AT	AA	AA	AA	AT	AA	AA	AT	AT
AG	GG	AA	AA	AA	AG	AA	AA	AA	AG	AA	AA	AG	AG
AC	AA	CC	CC	CC	AC	CC	CC	CC	AC	--	CC	AC	AC

AG	GG	AA	AA	AA	AG	AA	AA	AA	AG	--	AA	AG	AG
AG	GG	AA	AA	AA	AG	AA	AA	AA	AG	AA	AA	AG	AG
CT	CC	TT	TT	TT	CT	TT	TT	TT	CT	TT	TT	CT	CT
AG	AA	GG	GG	GG	AG	GG	GG	GG	AG	GG	GG	AG	AG
GT	TT	GG	GG	GG	GT	GG	GG	GG	GT	--	--	--	GT
CT	CC	TT	TT	TT	CT	TT	--	TT	CT	--	--	--	CT
AG	AA	GG	GG	GG	AG	GG	GG	--	--	--	GG	AG	AG
AG	AA	GG	GG	GG	AG	GG	GG	GG	AG	GG	GG	AG	AG
AG	GG	AA	AA	AA	AG	AA	AA	AA	--	AA	--	AG	AG
CG	CC	GG	GG	GG	CG	GG	GG	GG	CG	GG	--	CG	CG
AG	GG	AA	AA	AA	AG	AA	AA	AA	AG	AA	AG	AG	AG
AC	CC	AA	AA	AA	AC	AA	AA	AA	AC	AA	AC	AC	AC
AG	AA	GG	GG	GG	AG	GG	GG	GG	AG	GG	AG	--	AG
AG	AA	GG	GG	GG	AG	GG	GG	GG	AG	GG	AG	AG	AG
--	TT	AA	AA	AA	AT	AA	AA	AA	AT	AA	AT	AT	AT
CT	CC	TT	TT	TT	CT	TT	TT	TT	CT	TT	CT	CT	CT
CT	CC	TT	TT	TT	CT	TT	TT	TT	CT	--	--	CT	CT
CG	GG	CC	CC	CC	CG	CC	CC	CC	CG	--	CG	CG	CG
CT	TT	CC	CC	CC	CT	CC	CC	CC	CT	CC	CT	CT	CT
CT	CC	TT	TT	TT	CT	TT	TT	TT	CT	TT	CT	CT	CT
CT	TT	CC	CC	CC	CT	CC	CC	CC	CT	--	CT	CT	CT
AT	TT	AA	AA	AA	AT	AA	AA	AA	AT	AA	AT	AT	AT
AT	AA	TT	TT	TT	AT	TT	TT	TT	AT	--	AT	AT	AT
CT	TT	CC	CC	CC	CT	CC	CC	CC	CT	--	CT	CT	CT
CT	CC	TT	TT	TT	CT	TT	TT	TT	CT	TT	CT	CT	CT
AT	AA	TT	TT	TT	AT	TT	TT	TT	AT	--	AT	AT	AA
AG	GG	AA	AA	AA	AG	AA	AA	AA	AG	AA	AG	AG	GG
GT	GG	TT	TT	TT	GT	TT	TT	TT	GT	TT	GT	GT	GG
CT	TT	CC	CC	CC	CT	CC	CC	CC	CT	CC	CT	--	TT
CG	CC	GG	GG	GG	CG	GG	GG	GG	CG	GG	CG	CG	CC
AG	GG	AA	AA	AA	AG	AA	AA	AA	AG	AA	AG	AG	GG
CT	TT	CC	CC	CC	CT	CC	CC	CC	CT	--	CT	--	TT
CT	TT	CC	CC	CC	CC	CC	CC	CC	CT	CC	CT	CT	TT
AT	TT	AA	AA	AA	--	AA	AA	AA	AT	--	AT	AT	TT
AG	AA	GG	GG	GG	GG	GG	GG	GG	AG	--	AG	AG	AA
AA	AA	GG	GG	GG	GG	GG	GG	GG	AA	--	AG	AG	AA
TT	TT	GG	GG	GG	GG	GG	GG	GG	TT	GG	GT	GT	TT
AA	AA	GG	GG	GG	GG	GG	GG	AG	AA	GG	GG	AG	AA
GG	GG	AG	AG	AG	AG	AG	AG	GG	GG	--	AG	--	GG
CT	TT	TT	CT	TT	CT	TT	TT	TT	TT	TT	CT	TT	CT
AA	GG	GG	AG	GG	AA	AG	GG	GG	GG	--	AA	--	AA
AG	GG	GG	GG	GG	GG	GG	GG	--	GG	AG	AG	--	AG
AA	GG	GG	AG	GG	AG	GG	--	GG	AA	--	--	--	AG
GG	CC	CC	CG	CC	CG	CC	CC	CG	GG	--	GG	CC	GG
CC	TT	TT	CT	TT	CT	TT	TT	CT	--	--	CC	--	CC
CC	AC	AC	CC	AC	CC	AC	AC	AC	CC	AC	CC	AC	CC
AA	AG	AG	AG	AA	AG	AG	AG	--	AA	--	AA	--	AA

CC	AC	AC	CC	AC	CC	CC	AC	AC	CC	AC	CC	--	CC
AC	CC	CC	CC	CC	CC	CC	CC	AC	AC	AC	AC	CC	AC
TT	CT	CT	TT	CT	TT	TT	CT	CT	TT	CT	TT	CT	TT
AG	GG	GG	AA	GG	AG	AG	GG	AG	AA	AG	AA	--	AA
AG	AG	AA	AG	AA	AG	AG	AA	AG	GG	AG	GG	AA	GG
CT	CT	CC	CT	CC	CT	CT	CC	CT	TT	CT	TT	CC	CT
TT	TT	TT	TT	TT	TT	TT	TT	CT	--	CT	CT	--	--
CG	CG	GG	CG	GG	CG	CG	GG	CG	CC	CG	CG	CG	CG
GG	AG	GG	AG	GG	AG	AG	GG	AG	--	AG	AG	AG	AG
CC	CT	CC	CT	CC	CT	CT	CC	CT	TT	CT	CT	--	CC
AT	AA	AA	AA	AA	AA	AA	--	AT	AT	--	AT	--	--
AC	AC	AA	AC	AC	AC	AC	AA	AC	CC	AC	AC	--	AA
AG	GG	AG	GG	GG	GG	GG	AG	AG	GG	AG	AG	--	AG
CT	CC	CT	CC	CC	CC	CC	CT	CT	CC	CC	CT	--	CT
CT	CC	CT	CT	CC	CC	CC	CT	CT	CC	--	CT	CC	CT
GT	GG	GT	GG	GG	GG	GG	GT	GT	GG	--	GT	--	GT
CT	TT	TT	CT	CT	TT	CT	CC	CT	TT	CC	CC	CC	TT
GG	GG	GG	AG	AG	GG	GG	AG	AG	GG	AG	AG	AG	GG
AG	GG	GG	AG	AG	GG	AG	AA	AG	GG	AA	AA	--	GG
GG	CG	CG	CG	CG	CG	GG	GG	CG	CG	GG	GG	--	CG
AG	GG	GG	AG	AG	GG	AG	AA	AG	GG	--	AA	AA	GG
AG	AA	AA	AG	AG	AA	AG	GG	AG	AA	GG	GG	GG	AA
GT	TT	TT	TT	TT	TT	GT	GT	TT	TT	TT	TT	GT	TT
AG	AA	AA	AG	AG	AA	AG	GG	AG	AA	AG	AA	GG	AA
AG	AA	AA	AG	AG	AA	AG	GG	AG	AA	--	--	--	AG
GT	GG	GG	GG	GG	GG	GT	GG	GG	GG	GG	GG	GT	GT
CT	CC	CC	CT	CC	CC	CT	CT	CT	CC	CT	CT	--	CT
CT	TT	TT	CT	TT	TT	CT	CT	CT	TT	CT	CT	CC	CT
CC	CC	CC	CT	CC	CT	CC	CT	CC	CC	CT	CT	CT	CC
AG	AA	AA	AG	AA	AG	AG	GG	AA	AG	AG	GG	GG	AG
CT	CT	CT	TT	CC	CT	CT	TT	CC	CT	CT	TT	TT	CT
AG	AG	AG	GG	AA	GG	AG	GG	AA	AG	AG	GG	GG	AA
CT	CT	CT	TT	CC	TT	CT	TT	CC	CT	CC	TT	--	CC
CG	CC	CG	CC	CG	--	CG	--	GG	CG	GG	CC	--	GG
CT	TT	CT	CT	CT	TT	CT	CT	CC	CT	--	TT	--	CC
AC	AA	AA	AA	AC	--	AC	AC	AC	AC	--	AC	--	CC
AG	GG	AG	GG	GG	AG	AG	AG	AG	AG	AG	AG	AG	AA
CT	TT	TT	TT	TT	CT	CT	CT	CT	CT	CT	CT	CT	CC
AG	AA	AG	AA	AA	AG	AG	AG	AG	AG	AG	AG	AG	GG
AA	AA	AG	AA	AA	AG	AG	GG	--	GG	AG	AG	AG	GG
TT	TT	CT	--	TT	TT	TT	CT	CT	CT	CT	CT	--	CT
GG	GG	AA	GG	GG	AG	AG	AA	AG	--	AG	--	AG	AG
AA	AG	AA	AA	AA	AA	AG	AG	--	AG	--	AA	--	--
TT	GT	TT	TT	TT	TT	GT	GT	TT	GT	TT	TT	TT	TT
TT	GT	TT	TT	TT	TT	GT	GT	TT	GT	--	TT	--	TT
AA	AC	AC	AA	AA	AA	AC	CC	AC	CC	--	AC	AA	AC
AA	AA	AG	AA	AA	--	AG	GG	AG	GG	GG	GG	AA	AG

--	CC	CG	CC	CC	--	CC	--	CG	GG	CG	GG	--	--
CC	CC	CG	CC	CC	CC	CC	GG	CG	CG	CG	--	CC	CG
TT	TT	CT	TT	TT	TT	TT	CC	CT	CT	--	CC	TT	CC
CC	CC	CT	CC	CC	CC	CC	TT	TT	CT	CC	--	CC	TT
AA	AA	AT	AA	AT	AA	AA	TT	TT	AT	AA	TT	--	TT
AG	AG	AG	GG	GG	AG	AG	GG	GG	AG	AG	GG	AG	GG
CC	CC	CT	CT	CT	CC	CC	TT	TT	CT	--	TT	--	CT
TT	TT	CT	CT	--	TT	TT	--	CC	CT	--	CC	TT	CT
CC	CC	TT	CT	CT	CC	CC	TT	TT	CT	CC	TT	--	CT
GG	GG	AG	AG	AG	GG	GG	AA	AA	AG	GG	AA	GG	AG
CG	CG	CG	CC	CC	CC	CC	CG	CG	CG	CC	CG	--	--
GG	GG	GG	CG	CG	CG	GG	CG	CG	GG	--	CG	--	CG
GT	GT	GT	GG	GG	GG	GG	GT	GT	GT	--	GT	--	--
CC	CC	CC	CG	CG	CG	CG	CC	CC	CG	CC	CG	CG	CG
GG	GG	AA	AA	AG	AA	AG	AG	AG	AG	AG	AA	--	AA
AA	AA	AC	AC	AC	AC	AC	AA	AA	AC	AA	AC	AC	AC
AA	AA	AA	AT	AT	AT	AT	AA	AA	AT	AA	AT	AT	AT
GG	AG	AG	AA	AG	AA	AG	AG	AG	AG	AG	AA	AA	AA
AA	AT	AT	AT	AT	TT	AT	TT	AT	AT	--	TT	TT	TT
GG	AG	AG	AG	AG	AA	AG	AG	AG	AA	AG	AA	AA	AA
CC	CC	AC	CC	CC	--	AC	CC	--	AC	AC	AC	AC	AC
CC	CT	CT	CT	CT	TT	TT	CT	CT	TT	--	CT	TT	TT
TT	GT	TT	GT	GG	GG	GG	GT	GT	GG	--	GT	GG	GG
TT	GT	TT	GT	GG	GG	GG	GT	GT	GG	GT	GT	GG	GG
CC	CT	CC	CT	TT	TT	TT	CT	CT	TT	--	CT	--	TT
CC	CG	CC	CG	GG	GG	CG	CG	CG	GG	CG	--	GG	GG
GG	AG	GG	AG	AA	AA	AG	AG	AG	AA	--	AG	--	AA
CC	CT	CC	CT	--	TT	CT	CT	CT	TT	CT	CT	--	TT
TT	GT	TT	GT	GG	GG	GT	GT	GT	GG	--	GT	GG	GG
TT	CT	TT	CT	CC	CC	CT	CT	CT	CC	CT	CT	CC	CC
GG	CG	GG	CG	CC	CC	CG	CG	CG	CC	--	CG	CC	CG
GG	AG	GG	AG	AA	AA	AG	AG	AG	AA	AG	AG	AA	AG
AA	AC	AA	AC	CC	--	AC	AC	AC	CC	AC	AC	CC	AC
TT	CT	TT	CT	CC	CC	CT	CT	CT	--	CT	CT	--	CT
GG	AG	--	AG	AG	AG	GG	AG	GG	AG	GG	GG	AG	AG
AA	AC	AA	AC	CC	--	AC	AC	AC	CC	AC	AC	--	AC
CC	CT	CC	CT	TT	TT	CT	CT	CT	TT	CT	CT	TT	CT
GG	AG	GG	AG	AA	AA	AG	AG	AG	AA	AG	AG	AA	AG
GG	CG	GG	CG	CG	CC	CG	CG	CG	--	CG	--	GG	CG
GG	GT	GG	GT	GT	TT	GT	GT	GT	TT	GT	--	GT	GT
CC	CT	CC	CT	CT	TT	CT	CT	CT	TT	CT	CT	CT	CT
TT	CT	TT	CT	TT	CT	TT	CT	TT	CT	TT	TT	TT	CT
AG	AG	AG	AG	GG	GG	GG	AG	GG	GG	GG	AG	--	AG
CC	CG	CC	CG	CG	GG	CG	CG	--	GG	CG	CC	CC	CC
AA	AA	AA	AA	AT	AT	AT	AA	AT	AT	AT	AA	AA	AA
AA	AG	AA	AG	AA	AG	AA	AG	AA	AG	AG	AA	AA	AA
CC	AC	CC	AC	CC	AC	CC	AC	CC	AC	AC	CC	CC	CC

TT	CT	TT	CT	TT	CT	TT	CT	TT	CT	CT	TT	TT	TT
TT	TT	TT	TT	CT	CT	CT	TT	CT	CT	TT	TT	TT	TT
GG	AT	GG	GT	AG	AT	AG	GT	AG	AT	GT	GG	GG	GG
AA	AT	AA	AT	AA	AT	AA	AT	AA	AT	AT	AA	AA	AA
AG	AA	GG	AG	AG	AA	AG	AG	AG	AA	AG	GG	GG	GG
AC	AA	CC	AC	AC	AA	AC	AC	AC	AA	AC	CC	CC	CC
AA	AG	AA	AG	AA	AG	AA	AG	AA	AG	AG	AA	AA	AA
AG	AA	GG	AG	AG	--	AG	AG	AG	AA	--	GG	GG	GG
AG	GG	AA	AG	AG	GG	AG	AG	AG	AG	AG	AA	AA	AA
GG	AA	GG	AA	AG	AA	AG	AG	AG	AG	AG	GG	--	GG
GG	--	GG	TT	GT	GT	GT	GT	GT	--	--	GG	--	GG
GG	GG	GG	AG	AG	GG	AG	GG	AG	GG	--	GG	GG	GG
CC	CT	CC	TT	CT	CT	CT	CT	CT	CT	CT	CC	--	--
CC	CG	CC	GG	CG	CG	CG	CG	CG	CG	CG	CC	CC	CC
TT	CT	TT	CT	TT	CT	TT	CT	TT	CT	CT	TT	TT	TT
TT	TT	TT	CT	CT	TT	CT	TT	CT	TT	TT	TT	TT	TT
CT	CC	CC	CT	CT	CT	CC	CT	CT	CC	CC	CC	CC	CT
GT	GT	GG	GG	GT	GG	GG	GG	GT	GT	GG	GG	GT	GT
AA	AG	GG	AG	AA	AG	GG	AG	AA	AG	--	GG	--	AA
GG	AG	AA	AG	GG	AG	AA	AG	GG	AG	AA	--	--	GG
AG	AA	AA	AG	--	AG	AA	--	AG	AA	AA	AA	AA	--
TT	GT	GG	GT	TT	GT	GG	GT	TT	GT	--	GG	GT	TT
AG	AA	AA	AG	AG	AG	AA	AG	AG	AA	--	AA	--	AA
CT	CC	CC	CT	CT	CT	CC	CT	CT	CC	CC	CC	--	CC
AA	AG	AG	AA	AA	AA	AG	AA	--	AG	AG	AG	--	AG
AA	AG	GG	AA	AA	AG	GG	AG	AG	AG	GG	GG	AG	AG
AG	AG	GG	AA	AA	AG	GG	AG	--	--	GG	GG	--	AG
CG	CG	GG	CC	CC	CG	GG	CG	CG	CC	GG	--	CG	CG
CG	CG	GG	CC	CC	CG	GG	CG	--	CC	--	CG	--	--
AG	AG	GG	AA	AA	AG	GG	AG	AG	AA	GG	AG	--	AG
AG	AG	GG	AG	--	GG	GG	GG	--	AG	--	GG	AG	--
CT	TT	TT	CC	CC	CT	TT	CC	CT	CC	--	--	CT	CT
AC	CC	CC	AC	AC	CC	CC	AC	CC	AC	CC	--	--	AC
CG	CC	CC	CG	CG	CC	CC	CG	CC	CG	--	CC	CG	CG
AC	CC	CC	AC	CC	CC	CC	AC	CC	AC	CC	CC	AC	AC
AC	AA	AA	CC	AC	CC	AA	CC	AC	AC	--	AC	AC	AC
AA	AA	AA	AG	AG	AG	AA	AG	AG	AG	--	AG	AA	AA
AG	AA	AA	AG	AG	GG	AG	GG	AG	AG	AG	AG	AG	AG
GG	GG	GG	AG	AG	AG	GG	AG	GG	AG	GG	AG	GG	GG
CT	CC	CC	CC	CT	TT	CT	TT	CT	CT	--	CT	CT	CT
CT	CC	CC	CC	CT	TT	CT	TT	CT	CT	TT	CT	--	CT
AG	AA	--	AA	AA	AG	AG	AG	AG	--	--	AA	AA	AG
CT	CC	CC	CC	CC	CT	CT	CT	CT	CC	CT	CC	CC	CT
AG	AG	AG	GG	AG	AA	AG	AA	GG	AA	GG	AG	AA	GG
AC	AC	--	CC	AC	AC	CC	CC	CC	AC	--	AC	CC	CC
CC	CC	CC	CC	CC	CT	CT	CT	CC	CT	CC	CC	CT	CC
AG	AG	AG	AA	AG	GG	GG	AG	AA	GG	--	AG	AG	AA

AA	AA	AA	AA	AA	AG	AG	AG	AA	AG	AA	AA	AG	AA
AG	AA	AA	AG	AG	GG	GG	AG	AG	--	--	AG	--	AA
AC	CC	CC	AA	AC	AA	AA	AC	AC	AA	AC	AC	AC	CC
AG	GG	GG	AA	AG	AA	AA	AG	AG	AA	--	AG	AG	GG
GT	TT	TT	GG	GT	GG	GG	GT	GT	GG	--	GT	GT	GT
AG	AA	AA	AG	AG	AG	AG	AA	--	AG	AG	AG	AA	AA
CT	TT	TT	CC	CT	CC	CC	TT	CT	CC	CT	CT	CT	--
CC	CC	CC	CT	CT	CT	CT	CC	--	CT	--	CT	--	--
GG	AG	GG	AG	AG	AA	AA	GG	AG	AA	--	AG	--	AG
GG	CG	GG	CG	GG	CG	CG	GG	GG	CG	--	GG	CG	CG
TT	CT	TT	CT	TT	CT	CT	TT	TT	CT	TT	TT	--	CT
AA	GG	AA	AG	AG	AG	AG	AG	AG	--	AA	GG	--	AG
AA	GG	AA	AG	AG	AG	AG	AG	AG	GG	AA	GG	--	AG
CC	GG	CC	CG	CG	CG	CG	CG	CG	GG	--	GG	--	CG
--	GG	AA	AG	AG	AG	AG	AG	AG	AG	--	GG	--	AG
AG	AG	AG	AG	AG	AA	GG	--	AA	AG	--	AG	AG	GG
CT	CT	CT	CT	CT	CC	TT	TT	CC	CT	CC	CT	CT	TT
AG	AG	AG	AG	AG	--	GG	GG	--	AG	--	AG	AG	GG
CG	CG	CG	CG	CG	GG	CC	CC	GG	CG	GG	CG	CG	CC
AC	AC	AC	AC	AC	AA	CC	CC	AA	AC	AA	AC	AC	CC
AG	AG	AG	AG	AG	GG	AA	AA	--	AG	--	AG	--	AA
GT	GT	GT	GT	GT	TT	GG	GG	--	GT	TT	GT	GT	GG
AG	AG	GG	AG	AG	AA	GG	GG	AA	AG	--	AG	AG	GG
AG	AG	AG	AG	AG	AA	GG	GG	AA	AG	AA	AG	AG	GG
AG	AG	AG	AG	AG	GG	AA	AA	GG	AG	GG	AG	AG	AA
AC	AC	AC	AC	AC	CC	AA	AA	CC	AC	CC	AC	AC	AA
AG	AG	AG	AG	AG	GG	AA	AA	GG	AG	GG	AG	--	AA
AG	AG	AG	AG	AG	GG	AA	AA	GG	AG	GG	AG	AG	AA
CT	CT	CT	CT	CT	CC	TT	TT	CC	CT	--	CT	CT	TT
CG	CG	CG	CG	CG	CC	GG	GG	CC	CG	CC	CG	CG	GG
AC	AC	AC	AC	AC	CC	AA	AA	CC	AC	CC	AC	AC	AA
CT	CT	TT	CT	CT	CC	TT	TT	CC	--	CC	CT	--	TT
GT	GT	GT	GT	GT	GG	TT	TT	GG	GT	--	GT	GT	TT
CT	CT	--	CT	CT	TT	CC	CC	--	CT	--	CT	--	CC
CG	CG	CG	CG	CG	GG	CC	CC	--	CG	GG	CG	GG	CC
AT	AT	AT	AT	AT	AA	TT	TT	AA	AT	--	AT	--	TT
CT	CT	CT	CT	CT	TT	CC	CC	TT	CT	--	CT	CT	CC
AG	AG	AG	AG	AG	AA	GG	GG	AA	AG	--	AG	AG	GG
AG	AG	AG	AG	AG	GG	AA	AA	GG	AG	GG	AG	AG	AA
AT	AT	AA	AT	AT	TT	AA	AA	TT	AT	--	AT	AT	AA
AG	AG	AG	AG	AG	AA	GG	GG	AA	AG	AA	AG	AG	GG
AG	AG	AG	AG	--	GG	AA	--	GG	AG	--	--	--	AA
CG	CG	CC	CG	CG	GG	CC	CC	GG	CG	GG	CG	CG	CC
AG	AG	AG	AG	AG	GG	AA	AA	AG	AG	GG	AG	AG	AA
CG	CG	CG	CG	CG	CC	GG	GG	--	CG	--	CG	CG	GG
AG	AG	AG	AG	AG	AA	GG	GG	--	AG	--	AG	--	--
--	AC	AC	AC	AC	CC	AA	AA	AC	AC	--	AC	AC	AA

AG	AG	AG	AG	AG	AA	GG	--	AG	AG	--	AG	AG	GG
CG	CG	CG	CG	CG	GG	CC	CC	CG	CG	GG	CG	--	CC
CT	CT	CC	CT	CT	TT	CC	CC	CT	CT	--	CT	CT	CC
AT	AT	TT	AT	AT	AA	TT	TT	AT	AT	--	AT	--	TT
CT	CT	CT	CT	CT	TT	CC	CC	CT	CT	TT	CT	--	CC
AT	AT	AT	AT	AT	TT	AA	AA	AT	AT	--	AT	AT	AA
AT	AT	AT	AT	AT	AA	TT	TT	AT	AT	--	AT	AT	TT
CG	CG	CG	CG	CG	CC	GG	GG	CG	CG	CC	CG	CG	GG
CT	CT	CT	CT	CT	TT	CC	CC	CT	CT	TT	CT	--	CC
AG	AG	AG	AG	AG	GG	AA	AA	AG	AG	GG	AG	AG	AA
AC	AC	AC	AC	AC	CC	AA	AA	AC	AC	CC	AC	AC	AA
GT	GT	GT	GT	GT	GG	TT	TT	GT	GT	GG	GT	GT	TT
CT	CT	CT	CT	CT	TT	CC	CC	CT	CT	TT	CT	CT	CC
CG	CG	CG	CG	CG	CC	GG	GG	CG	CG	--	CG	CG	GG
GT	GT	GT	GT	GT	GG	TT	TT	GT	GT	--	GG	GT	TT
CT	CT	CT	CT	CT	CC	TT	TT	--	CT	CC	TT	CT	TT
AG	AG	AG	AG	AG	GG	AA	AA	AG	AG	GG	AA	AG	AA
AC	AC	AC	AC	AC	CC	AA	AA	AC	AC	--	AA	--	AA
CT	CT	CT	CT	--	TT	CC	CC	CT	CT	--	CC	CT	CC
AC	AC	AC	AC	AC	--	AA	AA	AC	AC	--	AA	--	AA
AG	AG	GG	AG	AG	GG	AA	AA	AG	AG	GG	AA	AG	AA
GT	GT	--	GT	GT	TT	GG	GG	GT	GT	TT	GG	GT	GG
GT	GT	TT	GT	--	TT	GG	GT	GT	GT	--	GG	GT	GG
AG	AG	AA	AG	AG	AA	GG	AG	AG	AG	AA	GG	AG	GG
CT	CT	TT	CT	CT	TT	CC	CT	CT	CT	TT	CC	CT	CC
CT	CT	TT	CT	CT	TT	CC	CT	CT	CT	CT	CC	CT	CC
AG	AG	GG	AG	AG	GG	AA	AG	AG	AG	--	AA	AG	AA
CT	CT	TT	CT	CT	TT	CC	CT	CT	CT	--	CC	TT	CC
CT	CT	CC	CT	CT	CC	TT	CT	CT	CT	CC	TT	CT	TT
AG	AG	AA	AG	AG	AA	GG	AG	AG	AG	AA	GG	AG	GG
CT	CT	TT	CT	CT	TT	CC	CT	CT	CT	TT	CC	CT	CC
TT	TT	CT	TT	CT	CT	TT	TT	TT	CT	CT	TT	--	TT
CT	TT	TT	CT	CT	TT	CC	CT	CT	CT	--	CT	CT	CC
AG	GG	GG	AG	AG	GG	AA	AG	AG	GG	GG	AG	AG	AA
AA	CC	CC	CC	AC	CC	AA	AC	AC	CC	CC	AC	AA	AC
CG	CC	CC	CC	CG	CC	CG	CC	CC	CC	CC	CC	CG	CC
GG	TT	GT	TT	GT	TT	GG	GT	GT	TT	--	GT	--	GT
GG	AA	AG	AA	AG	AA	AG	AG	AG	AA	AG	AG	GG	AG
TT	CC	CT	CC	CT	CC	CC	CT	CT	CC	CT	CT	--	CT
AG	GG	GG	GG	AG	GG	GG	GG	GG	GG	AG	GG	AG	GG
GG	AA	AG	AA	AG	AG	AA	--	AG	AA	--	AG	AG	--
--	CC	CG	CC	CG	CG	CC	--	CG	CC	--	CG	CG	CG
CT	TT	TT	TT	CT	CT	TT	TT	TT	TT	CT	TT	--	--
TT	GG	GT	GG	GT	TT	GG	GT	GT	GG	GT	GT	GT	GT
GG	GG	GG	GG	GG	GG	GG	AG	GG	GG	--	AG	GG	GG
GG	AG	GG	GG	AG	AG	AG	GG	AG	AG	GG	GG	AG	GG
AA	AC	AA	AA	AC	AA	AC	AC	AC	AC	AC	AC	AC	AA

TT	AA	TT	TT	AT	TT	AT	AT	AT	AT	--	AT	AT	TT
TT	AA	TT	TT	AT	TT	AT	AT	AT	AT	AA	TT	AT	TT
--	TT	AT	AA	AT	AA	AA	AT	AT	TT	AT	AA	AT	AA
TT	CC	CT	TT	CT	TT	TT	CT	CT	--	CT	TT	CT	TT
GG	AA	AG	GG	AG	GG	GG	AG	AG	AA	AG	GG	AG	GG
AA	CC	AC	AA	AC	AA	AA	AC	AC	CC	AC	AA	AC	AA
TT	CC	CT	TT	CT	TT	TT	CT	CT	CC	CT	TT	--	--
GG	AA	AG	AG	AG	GG	GG	AG	AG	AA	AG	GG	--	GG
GG	TT	TT	GT	GT	GG	GG	GT	GT	TT	GT	--	GT	GG
GG	AA	AA	AA	AG	GG	GG	AG	AG	AA	AG	GG	AG	GG
CC	CG	CG	CG	CC	CC	CC	CG	CC	CG	CC	CC	CC	CC
GG	AG	AG	AG	GG	GG	GG	AG	GG	AG	GG	GG	GG	GG
CC	CG	GG	CG	CG	CC	CC	CG	CG	CG	CG	CC	CC	CC
AA	AG	GG	AG	AA	AA	AA	AG	AG	AG	AG	AA	--	AA
GG	AG	AA	AG	GG	GG	AG	AG	AG	AG	--	GG	--	GG
AA	AG	GG	AG	AA	AA	AG	AG	AG	AG	AG	AA	AA	AA
GG	GT	GT	GT	GG	GG	GG	GT	GG	GT	GG	GG	GT	GT
GG	AG	AA	AG	GG	AG	AG	AG	AG	AG	AG	GG	AG	AG
TT	GT	GT	GT	TT	TT	TT	GT	TT	GT	--	TT	GT	GT
GG	CG	CC	CG	GG	CG	CG	CG	CG	CG	CG	GG	--	CG
TT	TT	TT	TT	--	TT	CT	CT	CT	--	--	--	--	CT
--	CG	GG	GG	CG	CG	CC	CC	CG	GG	--	CG	--	CG
CC	CG	CC	CC	CG	CG	CG	CG	CC	CC	CG	CG	CG	CC
AA	AA	AA	AC	AA	AA	AA	AC	AC	AA	AA	AA	AC	AA
CC	CC	CC	CT	CC	CC	CC	CT	--	CC	CC	CC	--	CC
AA	AG	AA	AG	AA	AA	AA	AG	AA	AA	AA	AA	AG	AA
CC	CT	CT	CT	CC	CC	CC	CT	CC	CC	CC	CC	--	CC
GG	CG	--	GG	CG	CG	CG	GG	CG	GG	CG	CG	CG	--
CC	CT	CT	--	CC	CC	CC	CT	CC	CC	--	--	--	CC
AA	AG	GG	AG	AG	AG	AG	AG	AG	AA	AG	AG	GG	AG
GG	GT	GT	GT	GG	GG	GG	GT	GG	GG	GG	GG	GT	GG
TT	GT	GG	GT	GT	GT	GT	GT	GT	TT	GT	GT	GG	GT
GG	CG	CG	CG	GG	GG	GG	GG	--	GG	--	GG	CG	GG
GG	CG	CC	CG	CG	CG	CG	GG	CG	GG	CG	CG	CC	CG
GG	AG	AG	AG	GG	GG	GG	GG	GG	GG	--	GG	AG	GG
TT	CT	CC	CT	CT	CT	CT	TT	--	TT	--	--	--	CT
GG	GT	TT	GT	GT	GT	GT	GG	GT	GG	--	GT	TT	--
GG	AG	AG	AG	GG	GG	GG	GG	GG	GG	GG	GG	--	GG
AA	AG	GG	AG	AG	AG	AG	AA	AG	AA	AG	--	--	AG
TT	CT	CC	CT	CT	CT	TT	--	CT	TT	--	CC	--	CT
TT	AT	AT	AT	TT	TT	TT	TT	TT	TT	TT	TT	AT	TT
TT	GT	GT	GT	TT	TT	TT	TT	--	--	TT	TT	--	TT
CC	CT	TT	CT	CT	CT	CC	CC	CT	CC	--	--	--	CT
AA	AG	AG	AG	AA	AA	AA	AA	AA	AA	AA	AA	AG	AA
TT	AT	AA	AT	AT	AT	TT	TT	AT	TT	AT	AT	--	AT
AA	AG	AG	AG	AA	AA	AA	AA	AA	AA	AA	AA	AG	AA
TT	CT	CT	CT	TT	TT	TT	TT	TT	TT	TT	TT	CT	TT

TT	AT	TT	AT	TT	TT	TT	TT	TT	TT	--	--	AT	TT
GG	GT	TT	GT	GT	GT	GG	GG	GT	GG	--	GT	GT	GT
AA	AG	GG	AG	AG	AG	AA	AA	AG	AA	GG	AG	AG	AG
CC	CT	TT	CT	CT	CT	CC	CC	CT	CC	TT	CT	CT	CT
CC	CT	CT	CT	CT	CT	CT	CC	CT	CC	--	CT	CT	CT
--	CT	CT	CT	CT	CT	CT	--	--	CC	--	CT	CT	CT
GG	GT	GT	GT	GT	GT	GT	GG	TT	GG	TT	GT	GT	GT
CC	CG	--	CG	CG	CG	CG	CC	GG	CC	GG	CG	CG	CG
CC	AC	AC	AC	AC	CC	AC	CC	AA	CC	AA	AC	AC	AC
AA	AG	AG	AG	AG	AA	AG	AA	GG	AA	GG	AG	AG	AG
GG	AG	AG	AG	AG	GG	AG	GG	AA	GG	--	AG	AA	AG
CT	TT	TT	TT	TT	CC	CT	TT	TT	CC	TT	CT	CC	TT
AC	AA	AA	AA	AC	CC	AC	AA	AA	CC	--	AC	--	AA
TT	TT	TT	TT	TT	GT	GT	TT	TT	GT	--	TT	--	TT
CG	GG	GG	GG	CG	CG	GG	GG	GG	CG	--	CG	--	GG
AG	GG	GG	GG	AG	AG	AG	GG	GG	AG	GG	AG	AA	GG
CT	TT	TT	TT	CT	CT	TT	TT	TT	CT	TT	CT	CT	TT
TT	TT	TT	TT	TT	TT	GT	TT	TT	GT	--	TT	GT	TT
CT	CC	CT	CC	CT	CC	CC	CC	CC	CT	CC	CT	CT	CC
AA	AC	AA	AA	AA	AA	AC	AA	AA	AC	AA	AA	AC	AA
AG	AG	AG	AA	AG	AA	AG	AA	AA	GG	AA	GG	GG	AA
AA	AG	AA	AA	AA	AA	AG	AA	AA	AG	--	AA	AG	AA
GT	GT	GT	TT	GG	TT	GT	GT	TT	GG	--	GT	--	--
CT	CT	CT	CC	TT	CC	CT	CT	CT	TT	--	CT	--	CC
AC	AC	AC	AC	CC	AA	AC	AC	AC	CC	AA	AC	AC	AA
AT	AT	AT	AT	AA	TT	AT	AT	AT	AA	TT	--	AT	TT
GT	GT	GT	GT	GG	GT	GG	GT	--	GG	GT	--	GG	GT
AA	AG	AA	--	AA	AG	--	AA	AG	AA	--	AA	--	--
CG	CC	CC	CG	GG	CC	CG	CG	CG	GG	--	--	CG	CC
AG	AA	AG	AG	GG	AA	AG	AG	AG	GG	--	AG	--	AA
CG	CG	GG	GG	CG	GG	GG	CC	GG	GG	--	CC	--	CG
AG	AG	GG	GG	AG	GG	GG	AA	GG	AG	GG	--	AG	AG
AG	AG	AA	AG	AG	AG	AG	AA	AG	AA	--	--	--	AA
AA	AA	AA	AT	AT	AT	AT	AA	AT	AT	AT	AT	AT	AT
--	AG	AA	AG	AG	AG	AG	AA	--	AA	--	AA	--	--
AA	AA	AG	AA	AA	AA	AA	AG	AA	AG	--	AG	AA	AG
AC	AC	AA	AC	CC	CC	AC	AC	CC	AC	CC	AC	CC	AA
CC	CC	CC	CC	AC	AC	CC	AC	AC	AC	AC	AC	--	CC
CC	AC	CC	AC	AA	AA	AC	AC	--	AC	--	AC	--	CC
GT	GT	GG	GT	TT	TT	GT	GT	TT	GT	--	GT	--	GG
AT	AT	AA	AT	TT	TT	AT	AT	TT	AT	--	AT	--	AA
AG	AA	AA	AA	AG	AG	AA	AG	AG	AA	AG	AG	AG	AA
AC	CC	CC	CC	AC	AC	CC	AC	CC	CC	--	AC	CC	CC
AT	AT	AA	AT	TT	TT	AT	AT	TT	AT	TT	AT	TT	AA
CT	TT	CT	CT	TT	TT	CT	CT	TT	--	TT	CT	CT	CC
AG	AA	AG	AG	AA	AA	AG	GG	AA	AA	AA	AG	AG	AG
CC	TT	CT	TT	TT	TT	CT	CC	TT	TT	--	CT	CT	CT

TT	CC	CT	CC	CC	CC	CT	TT	CC	CC	--	CT	--	CT
GG	AA	AG	AA	AA	AA	AG	GG	AA	AA	--	AG	AG	AG
CC	TT	CT	TT	TT	TT	CT	CC	TT	TT	TT	CT	CT	CT
--	CC	CT	CC	CC	CC	CT	TT	CC	CC	--	CT	--	CT
CC	TT	CT	TT	TT	TT	CT	CC	TT	TT	TT	CT	CT	CT
GG	AA	AG	AA	AG	AA	AG	GG	AA	AA	--	AG	--	AG
TT	CC	CT	CC	CT	CC	CT	TT	CC	CC	--	CT	CT	CT
GG	AA	AG	AA	AG	AA	AG	GG	--	AA	--	AG	AG	AG
CC	GG	CG	GG	CG	GG	CG	CC	GG	GG	--	CG	CG	CG
AA	CC	AC	CC	AC	CC	AC	AA	CC	CC	CC	AC	AC	AC
CC	TT	CC	TT	CT	CT	CT	CC	TT	TT	TT	CT	CT	CT
CC	TT	CT	TT	CT	CT	CT	CC	CT	TT	--	CT	--	CT
TT	AA	TT	AA	AT	AT	AT	TT	AT	AA	--	AT	AT	--
CC	TT	CT	TT	--	CT	CT	CC	CT	TT	--	CT	--	CT
AA	CC	AC	CC	AC	AC	AC	AA	AC	CC	CC	AC	AC	AC
TT	GG	GT	GG	GT	GT	GT	TT	GT	GG	GG	GT	GT	GT
TT	CC	CT	CC	CT	CT	CT	TT	CT	CC	--	CT	CT	CT
GG	CC	CG	CC	CG	CG	CG	GG	CG	CC	CC	CG	CG	CG
GG	CC	CG	CC	CG	CG	CG	GG	CG	CC	--	CG	CG	CG
CC	AA	AC	AC	AC	AC	AC	CC	AC	AA	AA	AC	AC	AC
GG	CC	CG	CG	GG	CG	CG	GG	GG	CG	CC	CG	CG	CG
AT	AT	AT	AT	AA	TT	AA	TT	AT	AT	AT	--	--	AT
AG	AG	GG	AG	AA	GG	AA	GG	AG	GG	AG	GG	--	AG
AG	AG	GG	AG	--	GG	AG	GG	AG	GG	AG	--	--	AG
AG	AA	AA	AG	GG	AA	AG	AA	AG	AA	AG	AG	GG	AA
AG	GG	GG	AG	AG	GG	AG	AG	AA	GG	AG	AG	AA	GG
AC	CC	CC	AC	AC	CC	AC	AC	AA	CC	AC	AC	--	CC
AG	GG	GG	AG	AG	GG	AG	AG	AA	GG	--	AG	AA	GG
AG	GG	GG	AG	AG	GG	AG	AG	AA	GG	AG	AG	AA	GG
GT	TT	TT	GT	GT	TT	GT	GT	GG	TT	GT	GT	--	TT
GT	GG	GG	GT	GT	GG	GT	GT	TT	GG	--	GT	--	--
AC	AA	AA	AC	AC	AA	AC	AC	CC	AA	--	AC	CC	AA
CT	CC	CC	--	CT	CC	CT	CT	TT	--	CT	CT	TT	CC
CT	CC	CC	CT	CT	CC	CT	CT	TT	CC	--	CT	CT	CC
GT	TT	TT	GT	GT	TT	GT	GT	GG	TT	GT	GT	--	TT
AT	TT	TT	AT	AT	TT	AT	AT	AA	TT	AT	--	--	TT
CG	CC	CC	CG	CG	CC	CG	CG	GG	CC	CG	CG	GG	CC
AC	CC	CC	AC	AC	CC	AC	AC	AA	AC	AC	AC	--	CC
CT	TT	TT	CT	CT	TT	CT	CT	CC	TT	CT	CT	CC	TT
CG	CC	CC	CG	CG	CC	CG	CG	GG	CC	--	CG	GG	CC
CT	TT	TT	CT	CT	TT	CT	CT	CC	TT	--	CT	CC	--
AT	TT	TT	AT	AT	TT	AT	AT	AA	TT	AT	AT	AA	TT
CT	CC	CC	CT	CT	CC	CT	CT	TT	CC	CT	CT	--	CC
AG	AA	AA	AG	AG	AA	AG	AG	GG	AA	AG	AG	GG	AA
AT	TT	TT	AT	--	TT	--	AT	AA	TT	--	--	--	TT
AT	TT	TT	AT	AT	TT	AT	AT	AA	TT	AT	AT	AA	TT
AC	CC	CC	AC	AC	CC	AC	AC	AA	CC	AC	AC	AA	CC

GT	GG	GG	GT	GT	GG	GT	GT	TT	GG	GT	GT	TT	GG
AG	AA	AA	AG	AG	AA	AG	AG	GG	AA	--	AG	--	AA
GT	TT	TT	GT	GT	TT	GT	GT	GG	TT	--	GT	--	TT
AG	GG	GG	AG	AG	GG	AG	AG	AA	GG	AG	AG	--	GG
CT	CC	CC	CT	CT	CC	CT	CT	TT	CC	CT	CT	--	--
AG	GG	GG	AG	AG	GG	AG	AG	AA	GG	AG	AG	AA	GG
CT	CC	CC	CT	CT	CC	CT	CT	TT	CC	CT	CT	TT	--
AC	AA	AA	AC	AC	AA	AC	AC	CC	AA	AC	AC	--	AA
AG	GG	GG	AG	AG	GG	AG	AG	AA	GG	AG	AG	--	GG
CT	CC	CC	CT	CT	CC	CT	CT	TT	CC	CT	CT	--	CC
AG	AA	AA	AG	AG	AA	AG	AG	GG	AA	AG	AG	--	AA
CT	TT	CT	CT	CT	CT	CT	CT	CC	TT	CT	CT	--	--
GT	GT	GT	GG	GG	GG	GG	GT	GG	--	GT	GT	GG	GT
GG	AG	GG	AG	AG	AG	GG	AG	GG	GG	GG	GG	--	GG
AA	AA	TT	AT	AT	AT	TT	AA	TT	AT	--	AT	AT	AT
AA	AA	AT	AT	AT	AT	TT	AA	TT	AT	--	AT	AT	AT
AA	AA	AC	AC	AC	AC	CC	AA	CC	AC	--	AC	--	AC
TT	GT	GT	GT	GT	GT	GG	TT	GG	GT	--	GT	GT	GT
TT	TT	CT	TT	TT	CT	CT	CT	--	TT	--	CT	CT	CT
AG	GG	AA	AG	AG	AA	AG	AA	AA	AG	GG	AG	AG	AA
CG	CG	GG	GG	GG	GG	CG	GG	GG	GG	CG	GG	--	GG
TT	CT	TT	TT	TT	TT	CT	TT	TT	TT	CT	--	--	TT
GT	GT	GG	GT	GT	GG	GG	GT	GG	GT	GT	GT	GG	GT
GG	GG	AG	AA	AG	--	AG	AG	AA	AG	--	AG	--	AG
AC	AC	AC	AA	AA	AA	AC	AC	AA	AA	--	AA	--	AA
CG	CG	GG	GG	GG	GG	CG	CG	GG	--	CG	--	--	--
GT	GT	GT	TT	TT	TT	GT	GT	GT	TT	TT	TT	GT	TT
CT	TT	TT	CC	CT	CC	CT	TT	CT	CT	CT	CT	CT	TT
AA	AA	GG	AG	GG	AG	GG	AG	AA	GG	GG	GG	--	AG
CT	CT	CT	CC	CC	CC	CC	CT	CT	CC	CC	CC	CT	CT
CC	CC	CC	CG	CG	CG	CG	CC	CC	CG	CG	CC	--	CC
AC	AC	AA	AC	AA	AC	AA	AA	AC	AC	--	AA	AC	AA
AG	AG	AA	AG	AG	GG	AG	AA	AG	GG	AG	AA	AG	AA
TT	TT	CT	TT	CT	TT	CT	--	TT	TT	CT	CT	--	CT
CG	CG	CG	CG	GG	GG	GG	CG	CG	GG	--	CG	CG	CG
AG	AG	GG	AG	AG	AA	AG	GG	AG	AA	AG	GG	AG	GG
GG	GG	GG	CG	CG	CG	CG	GG	GG	CG	CG	GG	--	GG
CG	CG	GG	CC	CG	CC	CG	GG	CG	CC	CG	GG	CG	GG
CT	CT	TT	CC	CT	CC	CT	TT	CT	CC	CT	TT	CT	TT
AG	AG	AA	GG	AG	GG	AG	AA	AG	GG	AG	--	AG	AA
AG	AG	GG	AG	GG	AG	GG	GG	AG	AG	GG	GG	AG	GG
CT	CT	TT	CT	CT	CC	CT	TT	CT	CC	--	TT	CT	TT
GG	GG	AG	GG	AG	GG	AG	AG	GG	GG	AG	AG	GG	AG
AG	AG	AA	AG	AG	GG	AG	AA	AG	GG	AG	AA	AG	AA
AC	AC	AA	AC	AC	CC	AC	AA	AC	CC	--	AC	AC	AA
AT	AT	AT	AT	--	TT	TT	AT	AT	TT	--	TT	AT	AT
AG	AG	GG	AG	AG	AA	AG	GG	AG	AA	--	AG	AG	GG

AC	AC	CC	AC	AC	AA	AC	CC	AC	AA	AC	AC	AC	CC
AG	AG	GG	AG	AG	AA	AG	GG	AG	AA	AG	AG	AG	GG
AA	AA	AG	AA	AG	AA	AG	AG	AA	AA	AG	AG	--	AG
CT	CT	CT	CT	CC	CC	CC	CT	CT	CC	--	CC	CT	CT
CG	CG	CG	CG	CC	CC	CC	CG	CG	CC	--	CC	--	CG
CT	CT	CT	CT	TT	TT	TT	CT	CT	TT	--	--	--	CT
GG	GG	GG	GG	CG	--	CG	GG	CG	CG	CG	CG	--	GG
CT	CT	CT	CT	CC	CC	CC	CT	CC	CC	CC	CC	CT	CT
GG	GG	GG	--	GT	GT	GT	GG	GT	GT	GT	GT	--	GG

4-18	4-24	4-25	4-26	4-27	4-29	4-31	4-34	4-35	4-39	4-40	4-41	4-43
AC	AC	AC	CC	CC	AA	CC	CC	AC	AC	AC	AC	AC
AG	GG	AG	GG	GG	AA	GG	GG	AG	AG	AG	AG	AG
CC	CC	CT	CC	CC	CT	CT	CC	CT	TT	--	CT	CT
AG	GG	GG	AG	AG	--	--	AG	GG	GG	--	GG	GG
GG	AG	--	--	AG	--	AG	GG	AG	AA	--	AG	AG
GG	GG	GG	--	CG	CG	GG	GG	CG	CG	CG	GG	GG
CT	CT	TT	CC	CT	TT	CT	CC	TT	TT	TT	CT	CT
GT	GT	TT	GG	GT	TT	GT	GG	TT	TT	TT	GT	GT
GT	GT	TT	GG	GT	TT	GT	GG	TT	TT	TT	TT	GT
GT	GT	TT	GG	GT	TT	GT	GG	TT	TT	--	TT	GG
AG	AG	AA	--	AG	AA	AG	GG	AA	AA	AA	AA	GG
CC	--	--	--	CT	CT	CC	CC	CT	CT	CT	CT	CC
AC	AA	AA	AC	AC	AA	AC	--	AA	AA	--	AA	CC
AG	AG	AG	AG	AG	AG	AA	AA	AG	AG	AG	AG	AA
CT	CT	CT	--	CC	CT	CT	CC	CT	CT	CT	CT	CC
GG	GG	GG	AG	AG	GG	GG	AA	GG	GG	GG	GG	AA
AT	AT	AT	AT	AT	AT	AT	TT	AT	AT	AT	AT	TT
CT	CT	CT	CC	CC	CT	CT	CC	CT	CT	CT	CT	CC
GT	--	GT	GT	GT	GT	GT	TT	GT	GT	GT	GT	GT
CG	CG	CG	CG	CG	CG	CG	CC	CG	CC	CC	CG	CG
AA	AA	AA	AT	AT	AA	AA	TT	AA	AT	AT	AA	AT
--	CC	--	--	TT	CC	CT	TT	CC	CT	CT	CC	CT
TT	TT	AT	--	AA	TT	AT	AA	TT	TT	--	TT	AT
TT	TT	AT	AA	AA	TT	AT	AA	TT	AT	AT	TT	AT
GG	GG	AG	--	AA	GG	AG	AA	GG	AG	AG	GG	AG
GT	GT	TT	TT	TT	GT	TT	TT	GT	TT	--	GT	GT
GG	GG	GT	TT	TT	GG	GT	TT	GG	GT	GT	GG	GT
AA	AA	AT	TT	TT	AA	AT	TT	AA	AT	--	AA	AT
CC	CC	CT	TT	TT	CC	CT	TT	CC	CT	CT	CC	CT
GG	GG	CG	--	CC	GG	CG	CC	GG	CG	CG	GG	CG
TT	TT	CT	CC	CC	TT	CT	CC	TT	CT	CT	--	CT
CC	CC	AC	--	AA	CC	AC	AA	CC	AC	AC	CC	AC
GG	--	AG	--	AA	GG	AG	AA	GG	AG	AG	GG	AG
--	TT	GT	--	--	TT	GT	GG	TT	GT	GT	TT	GT
GG	GG	AG	--	AA	GG	--	AA	GG	AG	AG	GG	AG
GG	GG	GT	TT	TT	GG	GT	TT	GG	GT	--	GG	GT
AA	AA	AG	GG	GG	AA	AG	GG	AA	AG	--	AA	AG
TT	TT	CT	CC	CC	TT	CT	CC	TT	CT	CT	TT	CT
TT	TT	AT	AA	AA	TT	AT	AA	TT	AT	AT	TT	AT
GG	--	AG	AA	AA	GG	AG	AA	GG	AG	AG	GG	AG
CC	CC	AC	AA	AA	CC	AC	AA	CC	AC	AC	CC	AC
--	--	--	--	--	GG	AG	AA	--	AG	AG	GG	AG

--	CC	CT	TT	TT	CC	CT	TT	CC	CT	CT	CC	CT
GG	GG	CG	--	CC	GG	CG	CC	GG	GG	GG	GG	CG
CC	CC	CT	TT	TT	CC	CT	TT	CC	CC	--	CC	CT
--	TT	CT	--	CC	TT	CT	CC	TT	TT	--	TT	CT
CC	CC	AC	AA	AA	CC	AC	AA	CC	CC	CC	CC	AC
--	TT	CT	--	CC	CT	CT	CC	TT	TT	--	TT	CC
CC	CC	CT	TT	CT	--	CT	CT	--	CC	CC	CT	TT
GG	AG	AA	AG	GG	GG	AG	AG	AG	AG	--	AA	AG
GT	GT	TT	GT	GG	GG	GT	GG	GT	GT	GT	TT	GT
AG	AG	AA	--	GG	--	AG	GG	AA	AG	--	AG	AG
CT	CT	CC	CT	CT	CT	TT	TT	CC	CT	CT	CT	CT
CG	CG	GG	GG	CG	CG	CC	--	GG	GG	--	CG	CG
AG	AG	GG	--	AG	AG	AA	AA	GG	GG	GG	AG	AG
AC	AC	AA	--	AC	AC	CC	AC	AA	AA	--	AC	AC
AG	GG	GG	GG	AG	AG	AG	AG	GG	GG	GG	GG	AG
CT	CT	--	--	CT	CT	TT	CT	CT	CC	CC	CT	CT
CT	CT	CT	TT	CT	CT	CC	CT	CT	TT	--	CT	CT
CT	CT	CT	CC	CT	CT	TT	CC	CT	CC	--	CT	CT
AG	AG	AG	GG	AG	AG	AA	GG	AG	GG	GG	AG	AG
AC	AC	AC	--	AA	AA	AA	CC	AC	CC	CC	AC	AC
AG	AG	AG	GG	AA	AA	AA	GG	AG	GG	GG	AG	AG
CC	CG	CG	CG	GG	GG	CG	CC	CG	CC	CC	CC	CG
GG	CG	CG	CG	CC	CC	CG	GG	CG	GG	GG	GG	CG
GG	AG	AA	AG	AG	AA	AG	GG	AG	GG	GG	GG	AA
AA	AC	AC	AC	AC	CC	AA	AA	AC	AA	AA	AA	CC
CC	CT	CT	CT	CT	TT	CC	CC	CC	CC	--	CC	TT
TT	--	CT	--	CT	CC	TT	TT	TT	TT	--	TT	CC
AA	AT	AT	AT	AT	TT	AA	AT	AA	AA	AA	AA	TT
AG	AG	GG	--	GG	--	AG	AG	AG	AG	AG	AG	GG
AT	TT	AT	AT	AT	TT	AT	TT	AT	TT	--	AT	TT
GG	GT	GG	--	GG	GT	--	GG	GT	TT	--	TT	TT
AG	AG	AG	--	AG	GG	AG	GG	GG	GG	--	GG	GG
CT	CT	CT	TT	CT	TT	CT	CT	TT	TT	TT	TT	TT
AG	AG	AG	--	AG	GG	AG	AG	GG	AG	AG	GG	GG
TT	GT	TT	GG	TT	GG	GT	TT	GT	GT	GT	GG	GG
GT	GT	GT	--	GT	GG	GT	GT	GG	GT	GT	--	GG
CC	CT	CT	TT	CC	--	CT	CC	CT	CT	CT	TT	TT
--	CT	CT	--	CC	TT	CT	CC	CT	CC	--	TT	TT
GG	AG	AG	--	GG	AA	AG	GG	AG	AG	AG	AA	AA
CC	CG	CG	--	CC	GG	CG	CC	CG	CG	--	GG	GG
AG	AG	AG	AA	GG	AA	AG	GG	AG	AG	AG	AG	AA
CT	CT	CT	--	--	CC	--	TT	TT	CT	CT	CT	CC
CT	CT	CT	CC	TT	CC	CC	TT	TT	CT	CT	CT	CC
CC	CG	CG	CC	CG	CC	CC	CG	CG	CC	CC	CG	CC
TT	AT	AT	--	AT	TT	--	AT	--	TT	--	AT	TT
GT	GG	GG	--	GT	GG	GT	GT	GT	GT	GT	GG	GG
CT	CC	CC	CC	CT	CC	CT	CT	CT	CT	CT	CC	CC

--	CT	CT	CC	TT	CT	CT	CT	TT	TT	--	CT	CT
GT	GG	GG	--	GT	GG	GT	GT	GT	GT	GT	GG	GG
CT	CT	CT	CT	TT	CT	CT	CT	TT	TT	TT	CT	CT
GT	TT	TT	TT	GT	TT	GT	GT	GT	GT	GT	TT	TT
CG	GG	GG	--	CG	GG	CG	CG	CG	CG	CG	GG	GG
AG	AG	AG	--	AA	AG	AG	AG	AA	AA	--	AG	AG
AG	AG	AG	AG	GG	AG	AA	AG	GG	GG	GG	AG	AG
GG	AG	AG	AG	GG	AG	AA	AG	GG	GG	GG	AG	AG
CC	CT	CT	CT	CC	CT	TT	CT	CC	CC	CC	CT	CT
AA	AG	AG	AG	AA	AG	GG	AG	AA	AA	AA	AG	AG
TT	GT	GG	--	TT	GT	GG	GT	TT	TT	TT	GT	GG
GG	GG	AG	AG	GG	GG	AG	GG	GG	AG	AG	AG	AG
CC	TT	TT	--	CC	CT	TT	CT	CC	CT	--	CT	TT
AC	CC	CC	--	--	CC	CC	CC	AC	AC	--	AC	CC
CC	CT	CT	CC	CC	CT	CT	CT	CC	CC	CC	CC	CT
TT	--	GG	GT	TT	GT	--	GT	TT	TT	--	GT	GG
CT	CC	CC	CC	CT	CT	CC	CT	CT	CT	CT	CC	CC
CG	GG	GG	CG	CG	GG	GG	GG	CG	CG	CG	CG	GG
AC	--	CC	AC	AC	--	--	CC	AC	AC	AC	AC	CC
CT	TT	TT	TT	CT	CT	TT	CT	CT	CT	--	TT	TT
AG	AA	AA	AA	AG	AG	AA	AG	--	AG	AG	AA	AA
GG	AA	AA	AG	GG	AG	AA	AG	GG	GG	--	AG	AA
CC	CT	CT	CC	CC	CT	CT	CT	CC	CC	--	CC	CT
AT	AA	AA	AT	AT	AA	AA	AA	--	AT	AT	AT	AA
CT	CC	CC	CT	CT	CC	CC	CC	CT	CT	CT	CT	CC
AG	AA	AA	--	AG	AA	AA	AA	AG	AG	AG	AG	AA
CT	CC	CC	--	CT	CT	CC	CT	CT	CT	CT	CC	CC
AA	TT	TT	AT	AA	AT	TT	AT	AA	AA	--	AT	TT
--	TT	TT	AT	AA	AT	TT	AT	AT	AA	--	AT	TT
CC	TT	TT	CT	CC	CT	TT	CT	CT	CC	CC	CT	TT
CT	CC	CC	CT	CT	CC	CC	CC	CT	CT	CT	CT	CC
GT	GG	GG	--	GT	GT	GG	GT	GG	GT	GT	GG	GG
CT	CC	CC	--	CT	CT	CC	CT	CC	CT	CT	CC	CC
--	AG	AG	--	AA	AA	AG	AA	AG	AA	--	AG	AG
AA	GG	GG	AG	AA	AG	GG	AG	AG	AA	--	AG	AG
AG	AA	AA	AA	AG	AG	AA	AG	AA	AG	AG	AA	AG
TT	CC	CC	CT	TT	CT	CC	CT	CT	TT	TT	--	CT
--	CC	CC	--	AC	AC	CC	AC	CC	AC	AC	CC	AC
AA	TT	--	AT	AA	AT	TT	AT	AT	AA	--	AT	AT
AG	AA	--	--	AG	AA	AA	AA	AG	AG	AG	AG	AA
AC	AA	AA	AA	AC	AC	AA	AC	AA	AC	AC	AA	AC
AT	TT	TT	TT	AT	AT	TT	AT	TT	AT	AT	TT	AT
AC	AC	AC	--	AC	--	AC	AA	CC	AC	AC	CC	AA
AC	CC	CC	AC	AC	CC	CC	CC	AC	AC	AC	AC	CC
GG	AG	AG	--	GG	GG	AG	GG	AG	GG	--	AG	GG
CC	AC	AC	CC	CC	--	AC	AC	CC	CC	CC	CC	AC
AG	AA	AA	AA	AG	AG	AA	AG	AA	AG	AG	AA	AG

CG	CC	CC	CG	CG	CC	CC	CC	CG	CG	CG	CG	CC
CC	TT	TT	CC	CC	CT	TT	CT	CT	CC	CC	CT	CT
TT	CC	CC	CT	TT	CT	CC	CT	CT	TT	TT	CT	CT
CT	CC	CC	CT	CT	CT	CC	CT	--	CT	CT	--	CT
AG	AA	AA	--	AG	AG	AA	AG	AA	AG	AG	AA	AG
TT	CC	CC	--	TT	CT	CC	CT	CT	TT	--	CT	CT
CT	TT	TT	TT	CT	CT	TT	CT	TT	CT	--	TT	CT
TT	--	--	--	TT	AT	AA	AT	AT	TT	--	AT	AT
AG	GG	GG	AG	AG	GG	GG	GG	AG	AG	AG	AG	GG
AC	AA	AA	--	AC	AC	AA	AC	AA	AC	--	AA	AC
CT	CC	CC	--	CT	CT	CC	CT	CC	CT	CT	CC	CT
--	TT	TT	GT	GT	TT	TT	TT	GT	GT	GT	GT	TT
CT	TT	TT	--	CT	CT	TT	CT	TT	CT	CT	TT	CT
CC	AC	AC	AC	CC	CC	AC	CC	AC	CC	CC	AC	CC
AG	AA	AA	AA	AG	AG	AA	AG	AA	AG	AG	AA	AG
GT	GG	GG	GG	GT	GT	GG	GT	GG	GT	GT	GG	GT
TT	AT	AT	--	TT	--	TT	--	AT	TT	--	AT	TT
AG	AA	AA	AA	AG	AG	AG	AG	AA	AG	AG	AA	AG
--	CC	CC	CT	CT	CC	CC	CC	CT	CT	CT	CT	CC
CT	CC	CT	--	--	CT	CT	CT	CC	CT	CT	CC	CT
AC	AA	AC	--	AC	AC	AC	AC	AA	AC	AC	AA	AC
CT	CC	CT	CC	CT	CT	CT	CT	CC	CT	CT	CC	CT
TT	AA	AT	AT	TT	AT	AT	AT	AT	TT	--	AT	AT
AA	GG	AG	AG	AA	AG	AG	AG	AG	AA	AA	AG	AG
CG	GG	GG	--	CG	GG	GG	GG	CG	CG	CG	CG	GG
CT	--	CT	--	CT	CT	CT	CT	TT	CT	CT	TT	CT
AG	GG	AG	--	AG	AG	AG	AG	GG	AG	AG	GG	AG
AG	AA	--	--	AG	AG	AG	AG	AA	AG	AG	AG	AG
AG	AA	AA	AG	AA	AA	AA	AA	AG	AG	AG	AG	AA
AG	GG	GG	AG	GG	GG	GG	GG	AG	AG	AG	GG	GG
CT	TT	--	TT	CT	TT	CT	CT	CT	--	--	CT	CT
TT	GG	GG	--	GT	GG	GT	GT	TT	GT	GT	GT	GT
GT	TT	TT	GT	TT	TT	--	TT	GT	GT	GT	TT	TT
AA	GG	AG	AG	AG	GG	AG	AG	AA	GG	--	AG	AG
CT	CC	CC	CC	CT	CC	CT	CT	CT	CC	CC	CT	CT
GT	TT	TT	TT	GT	TT	GT	GT	GT	TT	TT	GT	GT
GG	AG	--	--	GG	AG	AG	AG	--	GG	--	GG	AG
GG	GG	AG	AG	AA	AG	AG	AG	AA	GG	GG	AG	AG
CC	CC	CC	--	CT	CT	--	CT	CT	CC	CC	CC	CT
AT	AT	AA	AT	AA	AT	AT	AT	AA	AT	AT	AT	AT
GG	CG	CG	CG	CC	CG	CC	CG	CC	GG	GG	GG	CG
AC	AA	AC	AA	AA	AA	AA	AA	AC	AC	--	AC	AA
AG	AG	AG	GG	AA	AG	AA	AG	AG	AG	AG	GG	AG
GG	GG	AG	AG	AG	GG	AG	GG	AG	GG	GG	GG	GG
CC	CC	CT	CT	CT	CC	CT	CC	CT	CC	CC	CC	CC
AG	AG	AG	AG	AG	AA	AG	AG	AG	AG	AG	AA	AG
CC	CC	CT	CT	CT	CT	CT	CC	CT	CC	CC	CT	CC

AG	AG	GG	GG	GG	AG	GG	AG	GG	AG	AG	AG	AG
--	--	GG	--	GG	AG	GG	AA	AG	AA	--	AA	AA
CT	CT	CT	CT	CT	TT	CT	CT	CC	CT	CT	CT	CT
AT	AT	AT	AT	AT	TT	AT	AT	AA	AT	AT	AT	AT
AC	AC	CC	--	AC	AC	AC	AC	AA	AC	--	AC	AC
CT	CT	CC	CT	CT	CT	CT	CT	TT	CT	CT	CT	CT
--	AG	AA	AG	AG	GG	AG	AG	GG	AG	AG	AG	AG
--	CC	TT	TT	CT	CC	CT	CT	CC	CT	CT	CT	CC
--	TT	CT	CT	TT	TT	TT	TT	TT	TT	--	TT	TT
--	AA	GG	AG	AG	AA	AA	AG	AA	AG	AG	AA	AA
AA	AA	AG	--	AG	--	AG	AG	AA	AG	--	AG	AG
CC	--	CT	--	CT	TT	CT	CT	CC	CT	CT	CT	CT
--	GG	AG	--	GG	AG	AG	AG	--	GG	--	AG	--
--	CT	CT	--	CT	TT	CT	TT	CC	CT	CT	CT	CT
--	CG	--	--	CG	CG	GG	CG	GG	CG	CG	GG	CG
--	CT	CT	CT	CT	--	--	--	CC	CT	CT	CT	CT
CC	CC	CT	CT	CT	CT	CC	CC	CC	CC	CC	CT	CC
GG	AG	AG	AG	AA	AG	GG	AG	GG	AG	AG	AG	AG
CC	CT	CT	CT	TT	CT	CC	CT	CC	CT	CT	CT	CT
AG	AG	AA	AG	GG	AG	AA	AG	AA	AG	AG	AG	AG
CT	CT	CC	CT	TT	CT	CC	CT	CC	TT	TT	CT	TT
AG	AG	GG	AG	AA	AG	GG	AG	GG	AA	AA	AG	AA
AG	GG	GG	AG	AG	GG	GG	GG	GG	AG	AG	AG	AG
GT	--	TT	GT	GG	GT	TT	GT	TT	GG	GG	GT	GG
CG	CC	CC	CG	CG	CC	CC	CC	CC	CG	CG	CG	CG
CG	CC	CG	--	CC	CC	CG	CC	CG	CC	--	CG	CC
CT	CT	CC	CT	TT	CT	CC	CT	CC	TT	TT	CT	TT
CT	CT	TT	--	CC	CT	TT	CT	TT	CC	CC	CT	CC
AT	TT	AA	--	TT	AT	AA	AT	AA	TT	TT	AT	TT
AG	AA	GG	--	AA	AG	AG	AG	GG	AG	AG	AG	AA
CT	TT	CC	--	TT	CT	CT	CT	--	CT	CT	CC	TT
CT	CT	TT	CT	TT	TT	CT	TT	TT	TT	TT	CT	CT
AC	CC	AA	AC	CC	AC	AC	AC	AA	AC	AC	--	CC
GT	GT	GG	--	GG	GT	GT	GG	GT	GG	--	TT	GT
AG	AG	GG	--	GG	AG	AG	GG	AG	GG	GG	AA	AG
CT	CT	CC	--	CC	CT	--	CC	CT	CC	CC	TT	CT
AT	AT	TT	--	TT	AT	AT	TT	AT	TT	--	--	AT
AC	AC	--	--	AA	AC	AC	AA	AC	AA	AA	CC	AC
CC	CC	CC	CG	CC	CC	CG	CC	CC	CC	CC	CG	CG
AA	AA	AA	AG	AA	AA	AG	AA	AA	AA	--	AG	AG
CT	CT	TT	CC	TT	CT	CT	TT	CT	TT	TT	CC	CT
CT	CT	TT	--	TT	CT	CT	TT	CT	TT	--	CC	CT
AG	AG	GG	AA	GG	AG	AG	GG	AG	GG	GG	AA	AG
CT	CT	CC	CT	CC	CT	--	CC	--	CC	--	CT	CC
AG	AG	GG	--	GG	AG	AG	GG	AG	AG	AG	AA	AG
AG	AG	AA	--	AA	AG	AG	AA	GG	AG	AG	GG	AG
GT	GT	TT	--	TT	GT	GT	TT	GG	GT	GT	GG	GT

CT	CT	CC	--	CC	CT	CT	CC	TT	CT	--	TT	CT
CG	CG	CC	GG	CC	CG	CG	CC	GG	CG	CG	GG	CG
GT	GT	GG	TT	GG	GT	GT	GG	TT	GT	GT	TT	GT
AG	AG	GG	--	GG	AG	AG	GG	AA	AG	AG	AA	AG
CT	CT	TT	CC	TT	CT	CT	TT	CC	CT	CT	CC	CT
GT	GT	TT	GT	TT	GT	TT	TT	GT	TT	TT	GT	TT
CG	CG	CC	--	CC	CG	CG	CC	GG	CG	CG	GG	CG
--	AG	--	--	GG	AG	AG	GG	AA	AG	--	AA	AG
AG	AG	GG	AG	GG	AG	GG	GG	AG	GG	--	AG	GG
AC	AC	CC	--	CC	AC	AC	CC	AA	AC	AC	AA	AC
CG	CG	CC	CG	CC	CG	CC	CC	CG	CC	CC	CG	CC
AG	AG	AA	--	AA	AG	GG	AA	GG	AG	--	GG	AG
AC	AC	CC	--	CC	AC	AA	CC	AA	AC	--	AA	AC
GT	GT	GG	TT	GG	GT	TT	GG	TT	GT	GT	TT	GT
AC	AC	AA	CC	AA	AC	CC	AA	CC	AC	AC	CC	AC
--	CT	CC	CT	CC	CT	CT	CC	CT	CC	--	CT	CC
GT	GT	GG	--	GG	GT	TT	GG	TT	GT	GT	TT	GT
AG	GG	GG	--	GG	GG	AG	GG	AG	AG	AG	AG	AG
CC	CT	TT	CC	TT	CT	CC	TT	CC	CT	CT	CC	CT
CT	CT	CC	CT	CC	CT	CT	CC	CT	CC	--	CT	CC
AA	AT	TT	AA	TT	--	AA	TT	AA	AT	AT	AA	AT
CC	CG	GG	CC	GG	CG	CC	GG	CC	CG	CG	CC	CG
GT	GT	TT	GG	TT	GT	GG	TT	GG	GT	GT	GG	GG
AG	GG	GG	AG	GG	GG	AG	GG	AG	AG	AG	AG	AG
GT	TT	GT	GT	GT	TT	TT	GT	TT	TT	--	TT	TT
CT	CT	TT	--	TT	CT	CC	TT	CC	CC	--	CT	CC
AC	AC	AA	AC	AA	AC	CC	AA	CC	CC	CC	AA	CC
--	CT	CT	CC	CT	CT	CC	CT	CC	CC	--	CT	CC
CG	CG	CC	--	CC	CG	GG	CC	GG	GG	--	CC	CG
CT	TT	CT	--	CT	CT	CC	TT	CT	CC	CC	TT	CT
GT	TT	TT	--	TT	TT	GT	TT	GT	GT	GT	TT	TT
TT	TT	CT	--	CT	CT	CT	CT	--	TT	--	TT	CT
AT	TT	--	--	TT	TT	AT	--	AT	AT	AT	--	TT
CC	AC	AC	AC	AC	AC	CC	AC	CC	CC	CC	AC	AC
CT	CC	CT	CC	CT	CT	TT	CT	CT	CT	CT	CC	CT
AA	AG	AG	AG	AG	AG	AA	AG	AA	AA	AA	AG	AG
GG	AG	AG	--	AG	AG	GG	AG	GG	GG	GG	AG	AG
GG	GG	GG	--	CG	CG	CG	GG	GG	GG	GG	CG	CG
CC	CT	CC	--	CT	CT	TT	CC	CT	CC	--	CT	TT
CC	CT	CC	CT	CT	CT	TT	CC	CT	CC	CC	TT	CT
CT	CT	CT	--	CT	CT	TT	--	CT	CC	--	TT	CT
CT	CT	CT	--	CT	TT	CT	CT	CT	TT	TT	CC	CT
GT	GT	GT	--	GG	GG	GT	GT	GT	GG	GG	GT	GT
CT	CT	CT	CT	TT	TT	--	CT	CT	TT	--	CT	CT
AA	AT	AT	--	TT	TT	AT	AT	AA	TT	TT	AT	AT
AA	GG	AG	GG	GG	GG	AG	AG	AA	AG	AG	AG	AG
CC	AA	AC	--	AA	AA	AC	AC	CC	AC	--	AC	AC

AA	GG	AG	--	GG	--	AG	AG	AA	AG	AG	AG	AG
--	GG	AG	--	GG	--	AG	AG	AA	AG	AG	AG	AG
TT	CC	CT	--	CC	CC	CT	CT	TT	CT	CC	CT	CT
GG	--	AG	--	AA	AA	AG	AG	GG	AG	AG	AG	AG
GG	TT	--	TT	TT	TT	GT	GT	GG	GT	--	GT	GT
TT	CC	CT	--	CC	CC	CT	CT	TT	CT	CT	CC	CT
--	AA	AG	--	AA	AA	AG	AG	--	AG	AG	AG	--
GG	AA	AG	--	AA	AA	AG	AG	GG	AG	--	AG	AG
AA	GG	AG	--	GG	--	AG	AG	AA	AG	AG	AG	AG
GG	CC	CG	--	CC	CC	CG	CG	GG	CG	--	CG	CG
AA	GG	AG	GG	GG	GG	AG	AG	AA	AG	AG	AG	AG
AA	--	AC	--	CC	CC	AC	AC	AA	AC	AC	AC	AC
GG	AA	AG	AA	AA	AA	AG	AG	GG	AG	GG	AG	GG
--	AA	AG	AA	AA	AA	--	AG	GG	AG	AG	AG	GG
AA	TT	AT	--	TT	TT	AT	AT	AA	AT	AT	AT	AA
TT	CC	CT	CC	CC	CC	CT	CT	TT	CT	CT	CT	TT
TT	CC	CT	CC	CC	CC	CT	CT	TT	CT	--	CT	TT
CC	GG	CG	--	GG	GG	CG	CG	CC	CG	CG	CG	CC
CC	TT	CT	TT	TT	TT	CT	CT	CC	CT	CT	CT	CC
TT	CC	CT	--	CC	CC	CT	CT	TT	CT	CT	--	TT
CC	TT	CT	--	TT	TT	CT	CT	CC	CT	CT	CT	CC
AA	TT	AT	TT	TT	TT	AT	AT	AA	AT	AT	AT	AA
TT	AA	AT	AA	AA	AA	AT	AT	TT	AT	AT	AT	TT
CC	TT	--	--	TT	--	CT	CT	CC	CT	CT	CT	CC
TT	CC	CT	CC	CC	CC	CT	CT	TT	CT	CT	CT	TT
TT	AA	AT	AA	AA	AA	AT	AT	TT	AT	AT	TT	TT
AA	GG	AG	--	GG	GG	AG	AG	AA	AG	AG	AA	AA
TT	GG	--	GG	GG	GG	GT	GT	TT	GT	--	TT	TT
CC	--	CT	--	TT	TT	CT	CT	--	CT	--	CC	CC
GG	CC	CG	--	CC	CC	CG	CG	GG	CG	CG	GG	GG
AA	GG	AG	--	GG	GG	AG	AG	AA	AG	AG	AA	AA
--	TT	CT	--	TT	--	--	CT	CC	CT	--	CC	CC
CC	TT	CT	TT	TT	TT	CT	CT	CC	CT	CT	CC	CC
AA	TT	AT	TT	TT	TT	AT	AT	AA	TT	TT	AA	AA
--	AA	AG	AA	AA	AG	--	AG	GG	AA	--	GG	GG
GG	AA	AG	--	AA	AG	AG	AG	GG	AA	AA	GG	GG
GG	TT	GT	GT	GT	GT	GT	GT	GG	TT	TT	GG	GG
--	AA	AG	AG	AG	AG	AG	AG	GG	AA	--	GG	GG
AG	AG	GG	--	AG	GG	GG	GG	AG	GG	--	AG	AG
CT	CT	CT	CT	TT	CT	TT	CT	TT	CT	CT	CT	CT
GG	AA	AA	--	GG	AA	GG	AG	GG	AG	AG	AA	AA
GG	GG	--	AG	GG	GG	GG	GG	GG	AG	AG	AG	AG
GG	AG	--	--	GG	AG	AG	AG	GG	AG	AG	--	AA
CC	CG	GG	--	CC	CG	CG	CG	CC	CG	CG	GG	GG
TT	CT	CC	--	TT	CT	CT	CT	TT	CT	--	CT	CC
AC	CC	CC	CC	AC	CC	AC	CC	AC	AC	AC	CC	CC
AG	AG	AA	--	AA	AG	AA	AG	AG	AG	AG	AG	AA

--	CC	CC	CC	AC	CC	AC	CC	AC	--	AC	CC	CC
CC	CC	AC	AC	AC	CC	AC	CC	CC	CC	CC	CC	AC
CT	TT	TT	TT	CT	TT	CT	TT	CT	CT	CT	TT	TT
GG	AG	AA	AA	AG	AG	AG	AG	GG	AG	AG	AG	AA
AA	AG	GG	GG	AA	AG	AG	AG	AA	AG	--	AG	GG
CC	CT	TT	TT	CC	CT	CT	CT	CC	CT	CT	CT	TT
TT	TT	CT	CT	TT	TT	CT	TT	TT	CT	CT	TT	--
GG	CG	CC	CC	GG	CG	CG	CG	GG	CG	CG	CG	CC
GG	AG	AA	--	GG	AG	AG	AG	GG	AG	AG	AG	AA
CC	CT	TT	--	CT	CT	CT	CT	--	CT	CT	--	--
AT	AA	AT	AT	AA	--	AT	AT	AA	AT	AT	--	AT
--	CC	CC	CC	AC	AC	--	AC	AA	AC	AC	AA	AC
AG	GG	GG	--	GG	GG	GG	AG	AG	AG	AG	AG	AG
CT	CC	CC	--	CC	CC	CC	CT	CT	CT	--	CT	CT
--	CC	--	CT	CC	CC	CC	CT	CT	CT	--	CT	CT
GT	GG	GG	--	GG	GG	--	GT	--	GT	GT	GT	GT
CC	CT	CT	CC	CT	CC	TT	CC	CC	CT	CT	CC	CC
AG	GG	GG	GG	--	AG	GG	AG	AG	GG	--	AG	AG
AG	AG	AG	--	--	AA	GG	AG	AA	AG	AG	AA	AA
CG	--	GG	GG	CG	CG	CG	CG	GG	GG	--	GG	GG
AG	AG	AG	AG	AG	AG	GG	AG	AG	AG	AG	AA	AA
AG	AG	AG	AG	AG	AA	AA	AG	AG	AA	AA	GG	GG
TT	GT	GT	GT	TT	TT	TT	TT	TT	TT	TT	GT	GT
AG	AG	AG	AG	AG	AA	AA	AG	AG	AA	AA	GG	GG
AG	AG	AG	--	AG	AG	AA	AG	AG	AA	--	--	GG
GG	GT	GT	--	GG	GT	GG	GG	GG	GG	GG	GT	GT
CT	CC	CT	--	CT	CT	CC	CT	CT	CC	--	TT	TT
CT	TT	CT	CC	CT	CT	TT	CT	CT	TT	--	CC	CC
CT	CC	CC	CT	CT	CC	CC	CT	CT	CC	CC	CT	CT
AG	AA	AG	GG	AG	AA	AA	AG	AG	AA	AA	GG	GG
CT	CC	CT	TT	TT	CC	CC	CT	CT	CC	CC	TT	TT
AG	AA	AG	GG	GG	AA	AA	AG	GG	AA	AA	GG	AG
CT	CC	CT	--	TT	CC	CT	CC	TT	CC	CC	TT	CT
CG	GG	CG	--	CC	--	CG	GG	CC	GG	--	CC	CG
CT	CC	CT	--	TT	CC	CT	CC	TT	CC	--	TT	CT
--	AA	AC	AC	AC	AC	CC	AC	CC	--	--	--	CC
GG	GG	AG	AG	AG	AG	AA	AG	--	AG	--	AG	AA
TT	TT	CT	--	CT	CT	CC	CT	CC	CT	--	CT	CC
AA	AA	AA	--	AG	AA	GG	AG	GG	AG	AG	AG	GG
AA	AA	AA	--	AG	AA	GG	AG	GG	AG	--	AG	GG
--	TT	TT	CT	TT	TT	CT	TT	CT	TT	--	TT	CT
GG	GG	GG	--	AG	GG	--	AG	AA	AG	AG	AG	AG
AA	--	AA	AG	AG	AA	AG	AG	AG	AG	AG	AG	AA
TT	TT	TT	GT	GT	TT	GT	GT	GT	GT	GT	GT	TT
TT	TT	TT	GT	GT	TT	GT	GT	GT	GT	GT	GT	TT
AA	AA	AA	--	AC	AA	CC	AC	CC	AC	--	AC	AC
--	AA	AA	GG	AG	AA	GG	AG	GG	AG	AG	AG	AG

CC	CC	--	--	--	--	--	CG	GG	CG	CG	CG	--
CC	CC	CC	GG	CG	CC	GG	CG	GG	CG	CG	CG	CG
TT	TT	TT	--	TT	TT	CC	CT	CC	CT	CT	CT	CT
CC	CC	CC	CT	CC	CC	TT	CT	CT	CT	CT	CT	CT
AA	AA	AT	AT	AA	AA	AT	AT	AT	AT	AT	AT	AT
AG	AG	GG	GG	AG	AG	AG	GG	AG	GG	GG	GG	AG
CC	CC	CT	--	CC	CC	CT	CT	CT	TT	--	CT	CT
TT	--	CT	CT	TT	TT	CT	CT	TT	CC	--	CT	CT
--	CC	CT	CT	CC	CT	CT	CT	CC	TT	--	CT	CT
GG	GG	AG	AG	GG	AG	AG	AA	GG	AA	--	GG	AG
CC	CG	CC	--	CC	CG	CG	CG	CC	CG	CG	CC	CC
GG	GG	CG	CG	GG	GG	GG	CG	GG	CG	CG	GG	GG
--	GT	GG	--	GG	GT	GT	GT	GG	GT	GT	GG	GG
CG	CG	CG	CC	CC	CG	CG	CC	CC	CG	CG	CG	CG
--	AG	AA	--	AG	AA	AA	AG	AG	AG	--	AA	AA
AC	AC	AC	AA	AA	AC	AC	AA	AA	AC	AC	AC	AC
AT	AT	AT	AA	AA	AT	AT	AA	AA	AT	AT	AT	AT
AA	AG	AA	--	AG	AA	AA	AG	GG	AG	--	AA	AA
TT	AT	AT	AT	AT	TT	TT	AT	AA	AT	AT	AT	AT
AA	AG	AG	AG	AG	AA	AA	AG	GG	AG	AG	AG	AG
AC	CC	CC	--	CC	AC	--	AC	CC	CC	CC	CC	CC
TT	CT	CC	CT	CC	TT	TT	CT	CC	TT	TT	CT	CT
GG	GT	TT	GT	TT	GG	GT	GT	TT	GG	GG	GT	GT
GG	GT	TT	GT	TT	GG	GT	GT	TT	GG	GG	GT	GT
TT	CT	CC	CT	CC	TT	CT	CT	CC	TT	--	CT	CT
GG	CG	CC	CG	CC	GG	CG	CG	CC	GG	--	CG	CG
--	AG	GG	--	GG	--	AG	AG	GG	AA	--	AG	AG
TT	CT	CC	TT	CC	TT	--	CT	CC	TT	TT	CT	CC
GG	GT	TT	--	TT	GG	GT	GT	TT	GG	--	GT	GT
CC	CT	TT	CC	TT	CC	CT	CT	TT	CC	CC	CT	TT
CC	CG	--	CC	GG	CC	CG	CG	GG	CC	CC	CG	GG
AA	AG	GG	AA	GG	AA	--	AG	GG	AA	AA	AG	GG
CC	AC	--	CC	AA	CC	AC	AC	AA	CC	--	AC	AA
--	CT	TT	CC	TT	CC	--	CT	TT	CC	--	CT	TT
AG	AG	GG	AG	GG	AG	--	GG	GG	AG	AG	AG	GG
CC	AC	--	--	AA	CC	AC	AC	AA	CC	CC	--	AA
TT	CT	CC	TT	CC	TT	CT	CT	CC	TT	TT	CT	CC
AA	AG	GG	AA	GG	AA	AG	AG	GG	AA	AA	AG	GG
CC	CG	GG	--	--	CC	CG	CG	GG	CC	--	CG	GG
TT	--	--	--	GG	--	GT	GT	GG	TT	--	GT	GG
TT	CT	CC	TT	CC	TT	CT	CT	CC	TT	--	CT	CC
CT	CT	TT	CT	TT	CT	CT	TT	TT	CT	CT	CT	TT
GG	AG	AG	--	AG	GG	AG	GG	AG	GG	GG	AG	AG
GG	CG	CC	GG	CC	GG	CG	GG	CC	CG	CG	CG	CC
AT	AA	AA	AT	AA	AT	AA	AT	AA	AA	AA	AA	AA
AG	AG	AA	AG	AA	AA	AG	AG	AA	AA	AA	AG	AA
AC	AC	CC	AC	CC	CC	AC	AC	CC	CC	CC	AC	CC

CT	CT	TT	CT	TT	TT	CT	CT	TT	TT	TT	CT	TT
CT	TT	CT	CT	TT	CT	TT	CT	TT	TT	TT	CT	TT
AT	GT	AG	--	GG	AG	GT	AT	GG	GG	GG	AT	GG
AT	AT	AA	AT	AA	AA	AT	AT	AA	AA	AA	AT	AA
AG	AG	AG	AA	GG	AG	AG	AA	GG	GG	GG	AA	GG
AC	AC	AC	--	CC	AC	AA	AA	CC	CC	--	AA	CC
AG	AG	AA	AG	AA	AA	AG	AG	AA	AA	AA	AG	AA
AG	AG	AG	AA	GG	AG	AA	AA	GG	GG	GG	AA	GG
AG	AG	--	--	AA	AG	--	GG	AA	AA	--	GG	AA
AG	AG	GG	--	GG	AG	--	AA	GG	GG	GG	AA	GG
GT	GT	GG	--	GG	GT	--	GT	GG	GG	GG	--	GG
GG	GG	GG	AG	GG	AG	AG	GG	AG	GG	--	AG	GG
TT	CT	CC	TT	CC	CT	TT	CT	CT	CC	CC	--	CC
GG	CG	CC	GG	CC	CG	CG	CG	CG	CC	CC	CG	CC
CT	CT	TT	CT	TT	TT	CT	CT	TT	TT	TT	CT	TT
CT	TT	TT	CT	TT	CT	TT	TT	CT	TT	TT	TT	TT
CC	CT	CC	CT	CT	CC	CT	CC	CT	CT	CT	CC	CT
GT	GT	GT	GT	GG	GG	GT	GT	GT	GT	GT	GG	GG
AG	AA	AG	AA	AG	GG	AA	AG	AA	AA	--	GG	AG
AG	AG	AG	--	--	AA	GG	AG	GG	GG	--	AA	AG
AA	AG	--	--	AG	AA	AG	AA	AG	AG	--	AA	AG
GT	TT	GT	--	GT	GG	TT	GG	TT	TT	--	GG	TT
--	AG	AA	AG	AG	AA	AG	AA	AG	AG	--	AA	AG
CC	CT	CC	CT	CT	CC	CT	CC	CT	CT	CT	CC	CT
AG	AA	AG	--	AG	AG	AA	AG	AA	AA	AA	AG	AA
AG	AA	AG	AA	AG	AG	AA	GG	AA	AA	AA	AG	AA
AG	AA	GG	--	AG	AG	--	GG	--	AA	AA	AG	AA
CG	CC	GG	--	CG	CG	CC	GG	CC	CC	--	CG	CC
CG	CC	GG	--	CG	CG	CC	GG	--	CG	CG	CC	CG
AG	AA	GG	AA	AG	AG	AA	GG	AA	AG	AG	AA	GG
--	AG	--	--	AG	AG	AG	GG	--	GG	GG	AG	GG
CT	CC	--	CC	CT	CT	CC	TT	CC	CT	--	CC	TT
--	AC	AC	--	AC	AC	AC	CC	AC	CC	CC	AC	CC
CG	CG	CG	CG	CG	CG	CG	CC	CG	CC	CC	CG	CC
AC	AC	AC	AC	AC	AC	AC	CC	AC	CC	CC	AC	CC
AA	AC	CC	AC	AC	AC	CC	AC	AC	AC	--	CC	AA
AA	AG	AA	AG	AA	AA	--	AA	AA	AG	AG	AG	AA
AA	AG	GG	GG	AG	AG	GG	AG	AG	AG	AG	GG	AA
GG	AG	AG	AG	GG	GG	AG	GG	GG	AG	AG	AG	GG
CC	CT	TT	--	CT	CT	TT	CT	CT	CC	CC	TT	CC
CC	CT	--	TT	CT	CC	TT	CT	CT	CC	--	TT	CC
AA	AA	AG	--	AG	AA	AG	AG	AG	AA	AA	AG	AA
CC	CC	CT	CT	CT	CC	CT	CT	CT	CC	CC	CT	CC
AG	AG	--	GG	AG	AG	AG	GG	AA	AG	--	GG	AG
--	AC	AC	CC	AC	AC	CC	CC	AC	CC	--	CC	AC
CT	CC	CT	CC	CC	CC	CT	CC	CT	CT	CT	CC	CC
AG	AG	--	AG	AG	AG	--	AA	--	GG	--	AG	AG

AG	AA	AG	AA	AA	AA	AG	AA	AG	AG	AG	AA	AA
AG	AG	GG	AG	AG	AG	AG	AA	GG	GG	GG	AG	AG
AC	AC	AA	AC	AC	AC	AC	CC	AA	AA	AA	AC	AC
AG	AG	AA	AG	AG	AG	AG	GG	AA	AA	AA	AG	AG
GT	GT	GT	GT	GT	GT	GT	TT	--	GG	--	GT	GT
AA	AA	AA	AG	AG	AA	AA	AG	AA	AG	AG	AG	AG
CT	TT	CT	CT	CT	CT	CT	CT	TT	CC	--	CT	CT
CC	CC	CC	CT	CT	CC	CC	CT	CC	CT	CT	CT	CT
AA	AG	AG	AG	AG	AG	AG	AG	GG	AA	--	AG	AG
CG	CG	CG	CG	GG	CG	CG	GG	GG	CG	CG	GG	GG
CT	CT	CT	--	TT	--	CT	TT	TT	CT	CT	TT	TT
GG	AG	AG	AG	AG	AG	AG	AG	AA	GG	GG	AG	AG
GG	AG	AG	AG	AG	AG	AG	AG	AA	GG	GG	AG	AG
GG	CG	CG	CG	CG	CG	CG	GG	CC	GG	GG	CC	CG
--	AG	AA	--	AG	AG	AG	GG	AA	GG	GG	AA	AG
AG	GG	AA	--	AA	AG	AG	AG	AG	AG	--	AG	AG
CT	TT	CC	--	CC	CT	CT	CT	CT	CT	CT	CT	CT
AG	GG	AA	--	AA	AG	AG	AG	AG	AG	AG	AG	AG
CG	CC	GG	GG	GG	CG	CG	CG	CG	CG	CG	CG	CG
AC	CC	AA	--	AA	AC	AC	AC	--	AC	--	AC	AC
AG	AA	GG	--	GG	AG	--	AG	AG	AG	AG	AG	AG
GT	GG	TT	TT	TT	GT	GT	GT	GT	GT	GT	GT	GT
AG	GG	AA	AA	AA	--	AG	AG	AG	AG	AG	AG	AG
AG	--	AA	AA	AA	AG	AG	AG	AG	AG	AG	AG	AG
AG	AA	--	--	GG	AG	AG	AG	AG	AG	AG	AG	--
AC	AA	CC	CC	CC	AC	AC	AC	AC	AC	AC	AC	AC
AG	AA	GG	--	GG	AG	AG	AG	AG	AG	AG	AG	AG
AG	AA	GG	GG	GG	AG	AG	AG	AG	AG	AG	AG	AG
CT	TT	CC	CC	CC	CT	CT	CT	CT	CT	CT	CT	CT
CG	GG	CC	CC	CC	CG	CG	CC	CG	CG	CG	CG	CG
--	AA	CC	--	CC	AC	AC	--	AC	AC	AC	AC	AC
CT	TT	CC	--	CC	CT	CT	CC	CT	CT	--	CT	CT
GT	TT	GG	GG	GG	GT	GT	GG	GT	GT	--	GT	GT
CT	CC	TT	--	TT	CT	CT	TT	CT	CT	CT	CT	CT
CG	CC	GG	GG	GG	CG	--	GG	CG	CG	CG	CG	CG
AT	TT	AA	--	AA	AT	AT	AA	AT	AT	--	AT	AT
CT	CC	TT	--	TT	CT	CT	TT	CT	CT	CT	CT	CT
AA	GG	AA	AA	AA	AG	AG	AA	AG	AG	--	AG	AG
AG	AA	AG	GG	GG	AG	AG	GG	AG	AG	AG	AG	AG
AT	AA	AT	TT	TT	AT	AT	TT	AT	AT	--	AT	AT
AG	GG	AG	--	AA	AG	AG	AA	AG	AG	AG	AG	AG
AG	AA	AG	GG	GG	--	AG	--	AG	AG	--	AG	AG
CG	CC	CG	--	GG	CG	CG	GG	CG	CG	CG	CG	CG
AG	AA	AG	GG	GG	AG	AG	GG	AG	AG	--	AG	AG
CG	--	CG	--	CC	CG	--	CC	CG	CG	CG	CG	CG
AG	GG	AG	--	AA	AG	AG	AA	AG	AG	AG	AG	AG
AC	AA	AC	CC	CC	AC	AC	CC	AC	AC	--	AC	AC

AG	--	AG	--	AA	AG	AG	AA	AG	AG	AG	AG	AG
CG	CC	CG	--	GG	CG	CG	GG	CG	CG	CG	CG	CG
CT	--	CT	TT	TT	--	CT	TT	CT	CT	CT	CT	CT
AT	--	AT	AA	AA	AT	AT	AA	AT	AT	--	AT	AT
CT	CC	CT	TT	TT	CT	CT	TT	CT	CT	CT	CT	CT
AT	AA	AT	TT	TT	AT	AT	TT	AT	AT	AT	AT	AT
AT	TT	AT	AA	AA	AT	AT	AA	AT	AT	AT	AT	AT
CG	GG	CG	CC	CC	CG	CG	CC	CG	CG	CG	CG	CG
CT	CC	CT	TT	TT	CT	CT	TT	CT	CT	CT	CT	CT
AG	AA	AG	GG	GG	AG	AG	GG	AG	AG	AG	AG	AG
AC	AA	AC	CC	CC	AC	AC	CC	AC	AC	AC	AC	AC
GT	TT	GT	GG	GG	GT	TT	GG	GT	GT	GT	GT	GT
CT	CC	CT	TT	TT	CT	CC	TT	CT	CT	CT	CT	CT
CG	GG	CG	CC	CC	CG	GG	CC	CG	CG	CG	CG	CG
GT	TT	GT	--	GG	GT	TT	GG	GT	GT	--	GT	GT
CT	TT	CT	CC	CC	CT	TT	CC	CT	CT	CT	CT	CT
AG	AA	AG	--	GG	AG	AA	GG	AG	AG	AG	AG	AG
AC	AA	AC	CC	CC	AC	AA	CC	AC	AC	--	AC	AC
CT	CC	CT	--	TT	CT	CC	TT	CT	CT	CT	CT	CT
AC	AA	--	CC	CC	AC	AA	CC	AC	AC	AC	AC	AC
AG	AA	AG	GG	GG	AG	AA	GG	AG	AG	AG	AG	AG
GT	GG	GT	TT	TT	--	GT	TT	GT	GT	GT	GT	GT
GT	GG	--	--	TT	GT	GG	TT	GT	GT	--	GT	GT
AG	GG	AG	AA	AA	AG	GG	AA	AG	AG	AG	AG	AG
CT	CC	CT	--	TT	CT	CC	TT	CT	CT	CT	CT	CT
--	CC	CT	--	TT	CT	CC	TT	CT	CT	CT	CT	CT
AG	AA	AG	GG	GG	AG	AA	GG	AG	AG	AG	AG	AG
CT	CC	CT	TT	TT	CT	CC	TT	CT	CT	CT	CT	CT
CT	TT	CT	--	CC	CT	TT	CC	CT	CT	CT	CT	CT
AG	GG	--	AA	AA	AG	GG	AA	AG	AG	AG	AG	AG
CT	CC	CT	TT	TT	--	CC	TT	CT	CT	CT	CT	CT
CT	TT	TT	CT	CT	TT	TT	CT	TT	TT	TT	CT	TT
--	CT	CT	--	TT	CT	CC	TT	CT	CT	CT	CT	CT
GG	AG	AG	--	GG	AG	AG	GG	AG	AG	AG	AA	AG
CC	AC	AC	CC	CC	AC	AC	CC	AC	CC	CC	AA	AC
CC	CG	CC	CC	CC	CC	CC	CC	CC	CC	CC	CG	CC
--	GT	GT	--	TT	GT	TT	TT	GG	TT	TT	GG	GT
AG	AG	AG	AA	AA	AG	AA	AA	AG	AA	--	GG	AG
CT	CT	CC	--	CT	CT	CC	CC	--	CC	--	TT	CT
GG	AG	GG	GG	GG	GG	GG	GG	GG	GG	GG	AG	GG
AG	GG	--	--	AG	AG	AA	AA	AG	AA	AA	GG	AG
CG	GG	CC	--	--	CG	CC	CC	CG	CC	CC	GG	CG
TT	CT	TT	--	TT	--	TT	TT	TT	TT	TT	CT	TT
GT	TT	GT	GG	GT	GT	GG	GG	GT	GG	--	TT	GT
GG	GG	--	AG	GG	GG	GG	AG	AG	AG	AG	AG	GG
AG	AG	GG	GG	AG	AG	AG	GG	GG	AG	AG	AG	AG
AC	AC	AA	AC	AC	AC	AC	AC	AA	CC	--	AC	AC

TT	AT	TT	--	AT	TT	--	AT	TT	AA	--	AT	TT
TT	AT	TT	TT	AT	TT	AT	AT	TT	AA	AA	AT	TT
AA	AT	AA	AT	AT	--	AT	AT	AA	TT	TT	TT	AA
--	CT	TT	CT	CT	TT	CT	CT	TT	CC	CC	CT	TT
GG	AG	GG	AG	AG	GG	AG	AG	GG	AA	AA	AG	GG
AC	CC	AA	AC	AC	AA	AC	AC	AA	CC	CC	AC	AA
CT	CC	TT	CT	CT	--	CT	CT	TT	CT	CT	CT	TT
AG	AG	GG	--	AG	GG	AG	AG	GG	AG	AG	AG	GG
GT	GT	GG	GT	GT	GG	GT	GT	GG	GT	GT	GT	GG
AG	AG	GG	AG	AG	GG	AG	AG	GG	AG	--	AG	GG
CC	CG	CC	CG	CC	CC	CG	CG	CC	CC	CC	CC	CC
GG	AG	GG	AG	GG	GG	AG	AG	GG	GG	GG	GG	GG
CG	CG	CC	CG	CG	CC	GG	GG	CC	CG	CG	CG	CC
AG	AG	AG	AG	AG	AA	GG	GG	AA	AG	--	AG	AA
AG	--	AG	AG	AG	GG	AA	AA	GG	AG	AG	AG	GG
AG	AG	AG	GG	AG	AA	GG	GG	AA	AG	AG	AG	AA
GG	GT	GG	GT	GG	GG	GT	GT	GG	GG	--	GG	GG
AG	AG	AG	AG	AG	GG	AA	AA	GG	AG	AG	AG	GG
TT	GT	TT	--	TT	TT	GT	GT	TT	TT	--	TT	TT
--	CG	CG	--	CG	GG	CC	CC	CG	CG	--	CG	CG
TT	TT	CT	--	CT	CT	CT	TT	CT	CT	CT	--	TT
CG	CG	CC	--	CG	CC	CG	CG	CC	CC	--	GG	GG
CG	CG	CG	--	CC	CG	CG	CG	--	CG	--	CC	CC
AC	AA	AC	AA	AC	AC	AC	AA	AA	AC	AC	AA	AA
CT	CC	CT	--	CT	CT	CT	CC	CC	CT	CT	CC	CC
AG	AA	AG	--	AG	AG	AG	AA	AA	AG	AG	AA	AA
CT	CC	CT	CC	CC	CC	--	CC	CC	CT	CT	CC	CC
CG	CG	GG	CG	CG	CG	CG	CG	CG	GG	--	GG	GG
CT	CC	CT	--	CC	CC	CT	CC	CC	CT	--	--	CC
AG	AG	AG	AG	AG	AG	GG	AG	AG	AG	AG	AA	AA
GT	GG	GT	GG	GG	GG	GT	GG	GG	GT	GT	GG	GG
GT	GT	GT	GT	GT	GT	GG	GT	TT	GT	GT	TT	TT
CG	GG	CG	--	GG	GG	CG	GG	GG	CG	--	GG	GG
CG	CG	CG	CG	CG	CG	CC	CG	GG	CG	CG	CG	GG
AG	GG	AG	GG	GG	GG	AG	GG	GG	AG	AG	GG	GG
CT	CT	--	CT	CT	CT	CC	CT	TT	CT	--	TT	TT
GT	GT	GT	GT	GT	GT	TT	GT	GG	GT	--	GG	GG
AG	GG	AG	GG	GG	GG	AG	GG	GG	AG	AG	GG	GG
AG	AG	AG	--	AG	AG	GG	AG	AA	AG	AG	AA	AA
CT	CT	CT	CT	CT	CT	CC	CT	TT	CT	CT	TT	TT
AT	--	AT	--	TT	TT	AT	TT	TT	TT	--	--	TT
GT	TT	--	--	TT	TT	GT	TT	TT	TT	--	TT	TT
CT	CT	CT	--	--	CT	TT	CT	--	CC	CC	CC	CC
AA	AA	AG	AA	AA	AA	AG	AA	AA	AA	--	AA	AA
--	--	AT	AT	--	AT	AA	AT	TT	TT	--	--	AT
AA	AA	AG	AA	AA	AA	AG	AA	AA	AA	AA	AA	AA
TT	TT	CT	TT	TT	TT	CT	TT	TT	TT	TT	TT	TT

TT	TT	--	--	TT	TT	AT	TT	TT	TT	TT	TT	TT
GG	GT	GT	GT	GT	GT	TT	GT	GG	GG	GG	GG	GT
AA	AG	AG	AG	GG	AG	GG	AG	AA	AA	AA	AA	AG
CT	--	CC	--	TT	CT	TT	CT	CC	CC	--	CC	CT
CT	--	CT	CT	TT	CT	TT	CT	CC	CC	CC	CC	CT
CT	CT	CT	--	CT	CT	--	CT	CC	CT	--	--	CT
GT	TT	GT	GT	GT	GT	TT	GT	GG	GT	GT	GG	GT
CG	GG	CG	CG	CG	--	GG	CG	CC	CG	CG	CC	CG
AC	AA	--	--	AC	AA	AA	AA	CC	AC	AC	CC	AC
AG	GG	AG	AG	AG	GG	GG	GG	AA	AG	AG	AA	AG
AG	AA	GG	AG	AG	AA	AA	AA	AG	GG	--	GG	AG
CT	TT	CT	CT	CT	TT	CT	CC	CC	CT	CT	CC	CC
AA	AA	AC	AC	AC	AA	--	CC	CC	AC	AC	CC	CC
TT	TT	GT	GT	TT	TT	TT	GT	GT	GT	GT	GT	GT
GG	GG	GG	--	CG	GG	CG	CG	--	GG	GG	CG	CG
GG	GG	AG	--	AG	AG	AG	AG	AA	AG	AG	AA	AG
TT	TT	TT	TT	CT	TT	CT	CT	CT	TT	TT	CT	TT
--	TT	GT	GT	TT	GT	TT	TT	GT	TT	--	GT	GT
CC	CT	CC	CC	CT	CC	CT	CT	CT	CC	--	CT	CC
AA	AC	AC	AC	AA	AC	AC	AA	AA	AA	AA	AC	AC
AG	GG	AG	AG	AG	AG	GG	AG	AG	AA	AA	GG	AG
AG	AG	AG	AG	AA	AG	AG	AA	AA	AA	--	AG	AG
GT	GG	TT	GT	GT	GT	GG	TT	GT	GT	GT	GG	GT
CT	--	CC	--	CT	CT	TT	CC	CT	CT	CT	--	CT
AC	CC	AA	AC	AC	AC	CC	AC	AC	AC	AC	CC	AC
--	AA	TT	--	AT	AT	AA	AT	AT	AT	--	AT	AT
GG	GT	GT	--	GT	--	--	GG	GT	GT	--	GT	GG
AG	AA	AG	AG	AA	AG	--	AG	AA	AA	AA	AG	AG
CG	CG	CC	CG	CG	CG	CG	CG	CG	CG	CG	CC	CG
AG	AG	AA	--	AG	AA	AG	AG	AG	AG	AG	--	AG
GG	GG	CG	--	CC	GG	CG	CG	GG	CG	CG	GG	GG
AG	GG	AG	AG	AA	GG	AG	AG	GG	AG	--	GG	GG
AA	AG	--	AG	AA	AG	AA	AG	AG	AG	AG	AG	AG
AT	AA	AA	--	AA	AT	AT	AA	AT	AA	--	AT	AT
AA	--	AG	AG	--	AA	AA	AG	--	AG	AG	--	AG
AG	AG	AA	AA	AG	AG	AG	AA	AA	AA	--	AA	AA
AC	AA	AC	AC	AA	AC	AC	AC	CC	AC	AC	CC	CC
AC	CC	--	CC	CC	--	AC	CC	AC	CC	--	CC	AC
AC	CC	AC	AC	CC	CC	AC	AC	--	AA	--	AC	AA
GT	GG	TT	GT	GG	GG	GT	GT	TT	TT	--	GT	TT
--	AA	TT	--	AA	AA	AT	AT	TT	TT	--	AT	TT
AA	AA	AG	AA	AA	AA	AG	AA	AA	AG	AG	AA	AG
AC	CC	AC	CC	CC	CC	AC	CC	AC	AC	AC	CC	AC
AA	AA	TT	TT	AA	--	AT	AT	AT	TT	TT	AT	AT
CC	CC	TT	CT	CT	CC	CT	TT	CT	TT	TT	CC	CC
GG	GG	AG	AG	AG	GG	AG	AA	AG	AA	AA	GG	GG
CC	CC	CT	CT	CT	CC	--	TT	CT	TT	TT	CC	CC

TT	TT	CT	--	CT	TT	CT	CC	TT	CC	--	--	TT
--	GG	AG	AG	AG	GG	AG	AA	AG	AA	--	GG	GG
CC	CC	CT	CT	CT	CC	CT	TT	CT	TT	TT	CC	CC
--	TT	--	CT	CT	--	CT	--	CT	CC	CC	TT	TT
CC	CC	CT	--	CT	CC	CT	TT	CT	TT	--	CC	CC
GG	GG	AG	--	AG	GG	AG	AA	AG	AA	AA	GG	GG
TT	TT	CT	--	CT	TT	CT	CC	CT	CC	--	--	TT
GG	GG	GG	--	AG	GG	AG	AG	AG	AA	--	GG	GG
CC	CC	CG	--	CG	CC	CG	CG	CG	GG	GG	CC	CC
AA	AA	AC	AA	AC	AA	AA	AC	AC	CC	--	AA	AA
CC	CC	CC	CC	CT	CC	CC	CT	CT	TT	TT	CC	CC
--	CC	--	--	CT	--	--	--	--	TT	--	CC	--
TT	TT	TT	--	AT	TT	TT	AT	AT	AA	AA	TT	TT
CC	CC	CC	CC	CT	CC	CC	CT	CT	TT	--	CC	CC
AA	AA	AA	AA	AC	AA	AA	AC	AC	CC	CC	AA	AA
TT	TT	TT	TT	GT	TT	TT	GT	GT	GG	GG	TT	TT
--	TT	TT	--	CT	TT	TT	CT	CT	CC	--	--	TT
GG	GG	GG	GG	CG	GG	GG	CG	CG	CC	CC	GG	GG
GG	GG	GG	--	CG	GG	--	CG	CG	CC	--	GG	GG
CC	CC	CC	CC	AC	CC	CC	AC	AC	AA	AA	CC	CC
GG	GG	GG	GG	CG	GG	GG	CG	CG	CC	--	GG	GG
AT	--	AT	--	AT	AT	TT	TT	AT	AA	--	AA	AT
--	AG	AA	--	AG	AA	GG	GG	AG	AA	--	AG	AG
AA	AG	--	--	--	AA	--	AG	AG	AA	AA	AG	AG
GG	AG	GG	--	AA	GG	AA	AG	AG	AG	AG	AG	AG
--	AG	AA	--	GG	AA	GG	AG	AG	AG	AG	AG	AG
AA	AC	AA	--	CC	AA	CC	AC	AC	AC	--	AC	AC
AA	AG	AA	GG	GG	AA	GG	AG	AG	AG	AG	AG	AG
AA	AG	AA	GG	GG	AA	GG	AG	AG	AG	AG	AG	AG
GG	GT	GG	TT	--	GG	TT	GT	GT	GT	GT	GT	GT
TT	GT	TT	--	GG	TT	--	GT	GT	GT	GT	GT	GT
CC	AC	CC	AA	AA	CC	AA	AC	AC	AC	AC	AC	AC
TT	--	TT	--	CC	TT	CC	CT	CT	CT	--	--	CT
TT	CT	TT	--	CC	--	--	CT	CT	CT	CT	CT	CT
GG	GT	--	--	TT	GG	TT	GT	GT	GT	--	GT	GT
--	AT	AA	--	TT	--	TT	AT	AT	AT	AT	AT	AT
GG	CG	GG	--	CC	GG	CC	CG	CG	CG	CG	CG	CG
AA	AC	AA	--	CC	AA	CC	AC	AC	AC	AC	AC	AC
CC	CT	CC	TT	TT	CC	TT	CT	CT	CT	CT	CT	CT
GG	CG	GG	--	CC	GG	CC	CG	CG	CG	CG	CG	CG
CC	CT	CC	--	TT	CC	TT	CT	CT	CT	--	CT	CT
AA	AT	AA	TT	TT	AA	TT	AT	AT	AT	AT	AT	AT
TT	CT	TT	CC	CC	TT	CC	CT	CT	CT	--	CT	CT
GG	AG	GG	AA	AA	GG	AA	AG	AG	AG	AG	AG	AG
AA	--	--	TT	TT	AT	TT	AT	TT	AT	AT	AT	AT
AA	AT	--	--	TT	AA	TT	AT	AT	AT	--	AT	AT
AA	AC	AA	CC	CC	AA	CC	AC	AC	AC	AC	AC	AC

TT	GT	TT	GG	GG	TT	GG	GT	GT	GT	GT	GT	GT
GG	AG	GG	--	AA	GG	AA	AG	AG	AG	--	AG	AG
GG	GT	GG	--	TT	GG	TT	GT	GT	GT	GT	GT	GT
--	AG	AA	GG	GG	AA	GG	AG	AG	AG	AG	AG	AG
TT	CT	TT	--	CC	TT	CC	CT	CT	CT	CT	CT	CT
AA	AG	AA	GG	GG	AA	GG	AG	AG	AG	AG	AG	AG
TT	CT	TT	CC	CC	TT	CC	CT	CT	CT	CT	CT	CT
CC	AC	CC	--	AA	CC	AA	AC	AC	AC	--	AC	AC
--	AG	AA	--	GG	AA	GG	AG	AG	AG	AG	AG	AG
TT	CT	TT	--	CC	TT	CC	CT	CT	CT	CT	CT	CT
GG	AG	GG	--	AA	GG	AA	AG	AG	AG	--	AG	AG
CC	CT	CC	--	CT	--	--	CT	CT	CC	--	CT	TT
--	GT	GG	GT	GT	GT	GT	GT	GT	GG	--	GT	GT
AG	GG	GG	AG	GG	AG	AG	AG	GG	GG	--	GG	AG
--	AT	TT	--	AT	--	--	AT	AT	TT	TT	AT	AT
AT	AT	TT	AA	AT	AA	AA	AT	AT	AT	TT	AT	AT
AC	AA	CC	--	AC	--	AA	AC	AC	AC	AC	AC	AC
GT	TT	GG	--	GT	TT	GT	GT	GT	GT	GT	GT	GT
TT	--	CT	CT	CT	TT	TT	CT	TT	CT	CT	CT	TT
AA	GG	AG	AG	AA	AG	GG	AA	AA	AA	--	AG	AG
GG	CG	CG	--	GG	CG	CG	GG	GG	GG	--	CG	GG
--	TT	CT	--	TT	CT	CT	TT	TT	TT	--	CT	TT
GG	GT	GG	GT	GG	GT	GT	GG	GG	GG	GG	GG	GT
AA	AG	AG	--	AA	GG	AG	AG	AA	AA	--	AG	AG
AA	AA	AC	--	AA	AC	AA	AC	AA	AA	--	AA	AA
--	GG	CG	--	GG	CG	GG	CG	GG	GG	--	GG	GG
--	--	GT	GT	TT	GT	TT	GT	TT	TT	TT	TT	TT
CC	CT	CT	--	CC	TT	CT	CT	CC	CC	CC	CC	CT
--	GG	AA	--	AG	AG	GG	AA	AG	AG	--	AG	GG
CC	CC	CT	CT	CC	--	--	CT	--	CC	--	CC	CC
CG	CG	CC	CC	CG	CC	CG	CC	CG	CG	CG	CG	--
AC	AA	AC	--	AC	AA	AA	AC	AC	AC	AC	AC	AA
GG	AG	AG	AA	GG	AA	AG	AG	AG	AG	AG	GG	AG
TT	CT	TT	--	TT	CT	CT	TT	--	CT	--	TT	CT
GG	GG	CG	CG	GG	CG	GG	CG	CG	GG	GG	GG	GG
AA	AG	AG	--	AA	GG	AG	AG	AG	AA	AG	AA	AG
CG	CG	GG	--	CG	GG	CG	--	CG	CG	CG	CG	CG
--	CG	CG	--	CC	--	--	CG	--	CC	--	CC	CG
CC	CT	--	--	CC	TT	CT	CT	CC	CC	CC	CC	CT
GG	AG	AG	--	GG	--	AG	AG	GG	GG	--	GG	AG
AG	GG	AG	GG	AG	GG	GG	AG	AG	AG	AG	AG	GG
CC	CT	CT	TT	CC	TT	CT	CT	CT	CT	CT	CC	CT
GG	AG	GG	AG	GG	AG	AG	GG	--	AG	AG	GG	AG
GG	AG	AG	AA	GG	AA	AG	AG	AG	AG	AG	GG	AG
CC	--	AC	AA	--	--	AC	AC	AC	AC	AC	CC	AC
TT	TT	AT	AT	TT	AT	TT	AT	AT	TT	--	TT	TT
--	AG	--	--	AA	GG	GG	AG	--	AG	AG	AA	AG

AA	AC	AC	--	AA	CC	CC	AC	AC	AC	--	AA	AC
--	AG	AG	--	AA	--	GG	AG	AG	AG	AG	--	AG
AA	AG	AA	AG	AA	AG	AG	AA	AA	AG	AG	AA	AG
--	CC	CT	CT	CC	CT	CT	CT	CT	CC	CC	CC	CC
CC	CC	CG	CG	CC	CG	CG	CG	CG	CC	--	CC	CC
TT	TT	CT	CT	TT	CT	CT	CT	CT	CT	CT	CT	CT
CG	CG	GG	CG	CG	GG	GG	GG	GG	GG	GG	--	GG
CC	CC	CT	--	CC	CT	CT	CT	CC	CT	CT	CT	CT
GT	GT	--	GT	GT	--	GG	GG	GT	GG	GG	--	GG

4-45	4-47	4-48	4-49	4-50	4-51	4-53	4-54	4-56	4-57	4-59	4-60	4-62
AC	AC	AC	AC	AC	AA	CC	CC	CC	AC	AA	CC	AA
AG	AG	AG	AG	AG	AA	GG	GG	GG	AG	AA	GG	AA
CT	CT	CC	--	CT	TT	CC	CC	CC	CT	TT	CC	TT
AG	AG	AG	--	GG	GG	AG	AG	AG	GG	GG	AG	GG
AG	AG	GG	AG	AG	AA	AG	--	GG	AG	AA	GG	GG
CG	CG	GG	CG	GG	CG	CG	--	GG	GG	CG	GG	GG
TT	CT	CC	CT	CT	TT	CT	CC	CC	CT	TT	CC	CC
TT	TT	GG	GT	GT	TT	TT	GG	GG	GT	TT	GG	GG
TT	TT	GG	--	GT	TT	TT	GG	GG	GT	TT	GG	GG
TT	TT	GT	GT	GT	TT	TT	GG	GG	GT	TT	GG	GG
AA	AA	AG	--	AG	AA	AA	GG	GG	AG	AA	AG	GG
CT	CT	CT	CT	CC	CT	CT	--	CC	CC	CT	CT	CC
AA	AA	AC	AC	AC	AA	AA	CC	CC	AC	AA	AC	CC
AG	AG	AG	--	AA	AA	AG	--	AA	AA	AG	AG	AA
CT	CT	CC	--	CT	CT	CT	CC	--	CT	CT	CC	CC
AG	GG	AG	--	GG	AG	AG	--	AA	AG	AA	AG	AA
TT	AT	AT	TT	AT	TT	TT	AT	TT	TT	TT	AT	TT
--	CT	CC	CT	CT	CT	CT	CC	CC	CT	CC	CT	CC
TT	GT	GT	--	GT	TT	TT	GT	TT	TT	TT	TT	TT
CC	CG	CG	CC	CG	CC	CC	CG	CC	CC	CC	CC	CC
--	AT	AT	--	AA	AT	AT	AT	TT	AT	TT	AT	TT
CT	CT	CT	--	CT	CT	CT	--	TT	CT	CT	CT	TT
AT	AT	AT	TT	AT	AT	AT	AT	AA	AT	AT	AT	AA
AT	AT	AT	--	AT	AT	AT	AT	AA	AT	AT	AT	AA
AG	AG	AG	--	AG	AG	AG	AG	AA	AG	AG	AG	AA
TT	TT	--	GT	TT	TT	TT	GT	TT	TT	GT	TT	TT
GT	GT	GT	GG	GT	GT	GT	GT	TT	GT	GT	GT	TT
AT	AT	AT	--	AT	AT	AT	AT	TT	AT	AA	AT	TT
CT	CT	CT	--	CT	CT	CT	--	TT	CT	CT	CT	TT
--	CG	CG	--	CG	CG	CG	CC	CC	CG	CG	CG	CC
CT	CT	CT	TT	CT	CT	CT	CT	CC	CT	CT	CT	CC
--	AC	AC	--	AC	AC	AC	--	AA	AC	AC	AC	AA
AG	AG	AG	--	AG	AG	AG	AA	AA	AG	AG	AG	AA
GT	GT	GT	--	GT	GT	GT	GT	GG	GT	GT	GT	GG
AG	AG	AG	--	AG	AG	AG	--	AA	AG	AG	AG	AA
GT	GT	GT	--	GT	GT	GT	GT	TT	GT	GT	GT	TT
AG	AG	AG	AA	AG	AG	AG	AG	GG	AG	AG	AG	GG
CT	CT	CT	--	CT	CT	CT	CT	CC	CT	CT	CT	CC
AT	AT	AT	--	AT	AT	AT	AT	AA	AT	AT	AT	AA
AG	AG	AG	GG	AG	AG	AG	AG	AA	AG	AG	AG	AA
AC	AC	AC	CC	AC	AC	AC	AC	AA	AC	AC	AC	AA
--	AG	AG	--	AG	AG	AG	--	AA	AG	AG	AG	AA

CT	CT	CT	--	CT	CT	CT	CT	TT	CT	CT	CT	TT
CG	CG	CG	--	CG	CG	--	CG	CC	CG	CG	CG	CC
CT	CT	CT	--	CT	CT	CT	CT	TT	CT	CT	CT	CT
CT	CT	CT	--	CT	CT	CT	CT	CC	CT	CT	CT	CT
AC	AC	AC	CC	AC	AC	CC	AC	AA	AC	AC	AC	AC
TT	CT	CT	TT	TT	CT	TT	CT	CT	CT	CT	CT	CT
CC	CT	TT	CT	CC	CT	CC	CT	CT	CT	CT	CT	CT
AG	AG	GG	--	AG	AG	GG	--	GG	AG	AA	GG	AA
GT	GT	GG	GT	GT	TT	GT	GT	GG	GT	TT	GG	GT
AG	AG	GG	--	AG	AA	AG	AG	GG	AG	AA	GG	AG
CT	CT	TT	CT	CT	CC	CT	CT	TT	CT	CC	TT	CT
CG	CG	CC	--	CG	GG	CG	CG	CC	CG	GG	CC	CG
AG	AG	AA	AG	AG	--	AG	AG	AA	GG	GG	AA	GG
AC	AC	CC	AC	AC	AA	CC	--	CC	AA	AA	CC	AC
AG	GG	AG	--	AG	GG	AG	AG	AG	GG	GG	AG	GG
CT	CC	TT	--	CT	CC	TT	TT	TT	CT	CC	CT	CC
CT	TT	CC	CT	CT	TT	CC	CC	CC	CT	CT	CT	TT
CT	CC	TT	CT	CT	TT	TT	TT	TT	CT	CT	CT	CC
AA	GG	AA	AG	AG	AA	AA	AA	AA	AG	AG	AG	GG
AA	CC	AA	AC	AC	AA	AA	AC	AA	AC	AC	AC	CC
AA	GG	AA	AG	AG	AA	GG	AG	AA	AG	AG	AG	GG
GG	CC	GG	--	CG	GG	CC	CG	GG	--	CG	CG	CC
CC	GG	CC	CG	CG	CC	GG	CG	CC	CG	CG	CG	GG
AA	AG	AA	AG	AG	AA	GG	AG	AA	AG	AA	AG	GG
CC	AC	CC	--	AC	CC	AA	AC	CC	AC	CC	AC	AA
TT	CT	TT	TT	CT	TT	CC	--	TT	CT	TT	TT	CC
--	CT	CC	--	CT	CC	TT	CT	CC	CT	CC	CC	TT
TT	AT	TT	AT	AT	TT	AA	AT	TT	AT	TT	TT	AT
AG	AG	GG	--	AG	GG	AG	AG	GG	AG	GG	GG	GG
TT	TT	AT	--	AT	TT	AT	TT	TT	AT	TT	TT	AT
GG	GG	TT	GT	GT	GT	GT	GT	GG	GT	GT	GT	GT
AG	AG	GG	--	GG	AG	GG	AG	AG	AG	GG	GG	AG
CT	CT	TT	TT	TT	CT	TT	CT	CT	CT	TT	TT	CT
AG	AG	GG	--	GG	AG	GG	--	AG	AG	GG	GG	AG
TT	TT	GG	--	GT	GT	GT	GT	TT	GT	GT	GT	GT
GT	GT	GG	--	GG	GT	GG	GT	GT	GT	GG	GG	GT
CC	CC	TT	CC	CT	CT	TT	CT	CC	CT	TT	CT	CT
CC	CC	TT	--	CT	CT	TT	CT	CC	CT	TT	CT	CT
GG	GG	AA	GG	AG	AG	AA	AG	GG	AG	AA	AA	AG
CG	CC	GG	CC	CG	CG	GG	CG	CC	--	CG	GG	CG
AG	GG	AG	--	AG	AG	AA	AG	GG	AA	AG	AA	AG
CT	TT	CT	--	CT	CT	CC	CT	CT	CC	CT	CC	CT
CT	TT	CT	--	TT	CT	CC	CT	CT	CC	CT	CC	CC
CC	CG	CG	--	CG	CG	CC	CG	CC	CC	CG	CC	CC
TT	AT	AT	--	AT	AT	TT	AT	TT	TT	AT	TT	TT
GT	GT	GG	--	GT	GG	GG	GG	GG	GG	GG	GG	GG
CT	CT	CC	CT	CT	CT	CC	CC	CC	CC	CC	CC	CC

TT	CT	CT	CT	TT	TT	CC	--	CT	CT	CT	CC	CC
GT	GT	GG	GT	GT	GT	GG	GG	GG	GG	GG	GG	GG
TT	CT	CT	CT	TT	TT	CC	CC	CT	CT	CC	CT	CC
GT	GT	TT	GT	GT	GT	TT	TT	TT	TT	TT	TT	TT
CG	CG	GG	CG	CG	CG	GG	--	GG	GG	GG	GG	GG
AG	AG	AA	--	AG	AG	GG	AA	AG	AA	AG	GG	AG
AG	AG	GG	AA	AG	GG	AA	GG	AG	GG	AG	AA	AG
AG	AG	GG	--	AG	GG	AA	GG	AG	GG	AG	AG	GG
CT	CT	CC	TT	CT	CC	TT	CC	CT	CC	CT	CT	CC
AG	GG	AG	GG	GG	AA	GG	AA	AG	AA	AG	AG	AA
GT	GG	GT	--	GG	TT	GG	--	GT	GT	GT	GT	TT
AG	AG	AG	AG	AG	GG	GG	GG	AG	AG	AG	GG	GG
CT	TT	CT	--	TT	CC	CT	CC	CT	CT	CT	CT	CC
AC	CC	AC	--	CC	AC	CC	AC	AC	AC	AC	CC	AC
CC	CT	CC	CT	CT	CC	CT	CC	CC	CC	CC	CT	CC
GT	GG	GT	--	GG	TT	GT	--	GG	GT	GT	GT	TT
CC	CC	CC	CC	CC	CT	CT	CC	CC	CC	CC	CT	CT
CG	GG	CG	GG	GG	CG	GG	CG	CG	CG	CG	GG	CG
AC	CC	AC	--	CC	AC	CC	AC	AC	AC	AC	CC	AC
TT	TT	TT	TT	TT	CT	CT	--	TT	--	TT	CT	CT
AA	AA	AA	AA	AA	AG	AG	AG	AA	AA	AA	AG	AG
AG	AA	AG	AA	AA	GG	AG	GG	AG	AG	AG	AG	GG
CC	CT	CC	CT	CT	CC	CT	CC	CC	CC	CC	CT	CC
AT	AA	AT	--	AA	AT	AA	--	AT	AT	AT	AA	AT
CT	CC	CT	--	CC	CT	CC	CT	CT	CT	CT	CC	CT
AG	AA	AG	--	AA	AG	AA	AG	AG	AG	AG	AA	AG
CC	CC	CC	--	CC	CT	CT	CT	CC	CC	CC	CT	CT
AT	TT	AT	TT	TT	AA	AT	--	AT	AT	AT	AT	AA
AT	TT	AT	--	TT	AA	AT	AA	AT	AT	AT	AT	AA
CT	TT	CT	--	TT	CC	CT	CC	CT	CT	CT	CT	CC
CT	CC	CT	CC	CC	CT	CC	CT	CT	CT	CT	CC	CT
GG	GG	GG	--	GG	GT	GT	GT	GG	GG	GG	GT	GT
CC	CC	CC	CC	CC	CT	CT	--	CC	CC	CC	CT	CT
AG	AG	AG	AG	AG	AA	AA	AA	AG	AA	AG	AA	AA
AG	GG	AG	--	GG	AA	AG	AA	AG	AA	AG	AG	AA
AA	AA	AA	AA	AA	AG	AG	AG	AA	AG	AA	AG	AG
CT	CC	CT	--	CC	--	CT	TT	CT	TT	CT	CT	TT
CC	CC	CC	--	CC	AC	AC	AC	CC	AC	CC	AC	AC
--	TT	AT	--	TT	AA	AT	--	AT	AA	AT	AT	AA
AG	AA	AG	--	AA	AG	AA	AG	AG	AG	AG	AA	AG
AA	AA	AA	--	AA	AC	AC	AC	AA	AC	AA	AC	AC
TT	TT	TT	TT	TT	AT	AT	AT	TT	AT	TT	AT	AT
CC	AC	CC	AC	AC	AC	AA	AC	CC	AC	CC	AA	AC
AC	CC	AC	--	CC	AC	CC	--	AC	AC	AC	CC	AC
AG	AG	AG	AG	GG	GG	GG	--	AG	GG	AG	GG	GG
CC	AC	CC	AC	AC	CC	AC	CC	CC	CC	CC	AC	CC
AA	AA	AA	AA	AG	AG	AG	AG	AA	AG	AA	AG	AG

CG	CC	CG	CC	CC	CG	CC	CG	CG	CG	CG	CC	CG
CT	TT	CT	TT	CT	CC	CT	CC	CT	CC	CT	CT	CC
CT	CC	CT	--	CT	TT	CT	TT	CT	TT	CT	CT	TT
--	CC	CC	--	CT	CT	CT	CT	CC	CT	CC	CT	CT
--	AA	AA	--	AG	AG	AG	AG	AA	AG	AA	AG	AG
CT	CC	CT	--	CT	TT	CT	TT	CT	TT	CT	CT	TT
TT	TT	TT	TT	CT	CT	CT	CT	TT	CT	TT	CT	CT
--	AA	AT	--	AT	TT	AT	--	AT	TT	AT	AT	TT
AG	GG	AG	GG	GG	AG	GG	AG	AG	AG	AG	GG	AG
AA	AA	AA	--	AC	AC	AC	AC	AA	AC	AA	AC	AC
--	CC	CC	--	CT	CT	CT	CT	CC	CT	CC	CT	CT
GT	TT	GT	TT	TT	GT	TT	GT	GT	GT	GT	TT	GT
TT	TT	TT	--	CT	CT	CT	CT	TT	CT	TT	CT	CT
AC	AC	AC	AC	CC	CC	CC	CC	AC	CC	AC	CC	CC
AA	AG	AA	AA	AG	AG	AG	AG	AA	--	AA	AG	AG
GG	GT	GG	--	GT	GT	GT	GT	GG	GT	GG	GT	GT
AT	TT	AT	--	TT	TT	TT	TT	AT	TT	AT	TT	TT
AA	AG	AA	--	AG	AG	AG	AG	AA	AG	AA	AG	AG
--	CC	CT	CT	CC	CT	CC	--	CT	CT	CT	CC	CT
--	CT	CC	--	CT	CT	CT	--	CC	CT	CC	CT	CT
AA	AC	AA	--	AC	AC	AC	--	AA	AC	AA	AC	AC
--	CT	CC	CC	CT	CT	CT	CT	CC	CT	CC	CT	CT
AT	AT	AT	--	AT	TT	AT	TT	AT	TT	AT	AT	TT
AG	AG	AG	--	AG	AA	AG	AA	AG	AA	AG	AG	AA
CG	GG	CG	--	GG	CG	GG	CG	GG	CG	CG	GG	CG
TT	CT	TT	--	CT	CT	CT	CT	TT	CT	TT	CT	CT
--	AG	GG	--	AG	GG	GG	AG	GG	GG	GG	AG	GG
AG	AG	AA	AG	AG	AA	AA	--	AA	AA	AA	AG	AA
AG	AA	AG	--	AA	AG	AA	AG	AG	AG	AG	AA	AG
AG	GG	AG	--	GG	AG	GG	AG	AG	AG	AG	GG	AG
--	CT	TT	--	CT	TT	TT	--	TT	TT	TT	CT	--
TT	GT	GT	GT	GT	GT	GG	GT	GT	GT	GT	GT	GT
GT	TT	GT	--	TT	GT	TT	GT	GT	GT	GT	TT	GT
AA	AG	AG	--	AG	AG	GG	AG	AG	AG	AA	AG	AG
CT	CT	CC	CT	CT	CC	CC	CC	CC	CC	CT	CT	CT
TT	GT	TT	GT	GT	TT	TT	TT	TT	TT	GT	GT	GT
--	AG	GG	--	AG	GG	AG	--	GG	GG	GG	AG	GG
AA	AG	GG	AG	AG	AG	AA	AA	AA	GG	AG	GG	AG
CC	CC	CC	--	CC	CC	CT	CT	CT	CC	CC	CC	CC
AT	AA	AT	--	AA	AA	AA	--	AA	AT	AA	AT	AA
CG	CC	CG	CG	CG	CC	CC	CG	CC	GG	CG	GG	CG
--	AA	AA	--	AC	AA	AA	AC	AA	AC	AA	AC	AC
AG	AA	AG	AG	AG	AA	AA	AG	AA	GG	AA	GG	AG
GG	AG	GG	--	AG	AG	AG	AG	AG	GG	AG	GG	AG
CC	CT	CC	CC	CT	CT	CT	CT	CT	CC	CT	CC	CT
AA	GG	AG	--	AG	GG	GG	AG	GG	AG	GG	AA	AG
CT	CC	CC	CC	CT	CC	CT	CT	CC	CC	CC	CT	CT

AG	GG	AG	AG	GG	GG	GG	GG	GG	AG	GG	AG	GG
AG	AG	AA	--	GG	AG	GG	GG	AG	AA	AG	AG	GG
TT	CC	CT	CT	TT	CC	CT	CT	CC	CT	CC	TT	CT
TT	AA	AT	AT	TT	AA	AT	AT	AT	AT	AA	TT	AT
CC	AA	AC	AC	AC	AA	AC	--	AC	CC	AC	CC	AC
--	TT	CT	--	CT	TT	CT	--	CT	CT	CT	CC	CT
--	GG	AG	--	AG	GG	AG	AG	AG	AG	AG	AA	AG
TT	CT	CT	CT	CT	CC	CT	CT	CT	CT	CT	TT	CT
CT	TT	TT	--	TT	TT	CT	CT	CT	CT	--	CT	CT
--	AG	AG	AG	AG	AA	AG	--	AG	AG	AG	GG	AG
AG	AA	GG	--	AA	AG	AA	AG	AG	AG	GG	AG	AA
--	CC	TT	--	CC	CT	CC	CT	CT	CT	TT	CT	CC
--	GG	AG	--	GG	AG	GG	AG	GG	GG	AG	GG	GG
CT	CC	CT	--	CC	CT	CT	TT	CT	CT	TT	CT	CC
GG	GG	GG	--	GG	GG	CG	CG	CG	CG	CG	CG	GG
--	CC	CT	--	CC	CT	CT	TT	CT	CT	TT	TT	CC
CT	CC	CT	--	CC	CT	CC	--	CC	CC	CT	CT	CC
AG	GG	GG	AG	AG	AG	AG	AA	AG	AG	AA	AG	GG
--	CC	CC	CT	CT	CT	CT	TT	CT	CT	TT	CT	CC
AG	AA	AA	--	AG	AG	AG	GG	AG	AG	GG	AG	AA
CT	CC	CC	CT	CT	CC	CT	TT	CT	CT	TT	CT	CC
AG	AG	GG	AG	AG	GG	AG	AA	AG	AG	AA	AG	GG
AG	AG	GG	--	AG	GG	GG	AG	GG	GG	AG	AG	GG
GT	GT	GT	GT	GT	TT	GT	GG	GT	GT	GG	TT	TT
CG	CG	CG	--	CG	CC	CC	CG	CG	CC	CG	CC	CC
CG	CG	CG	--	CG	CG	CC	CC	CC	CC	CC	CG	CG
CT	CT	CT	CT	CC	CC	CT	--	TT	CT	TT	CC	CC
CT	CC	CT	CT	TT	CT	CT	CC	CC	CT	CC	TT	TT
AT	TT	AT	AT	AA	AT	AT	TT	TT	--	TT	AA	AA
AG	AA	AG	AG	GG	AG	AG	AA	AA	AG	AA	GG	GG
CT	TT	CT	--	CC	CT	CT	CT	TT	CT	TT	CC	CC
CT	CT	CT	CT	TT	CT	TT	TT	CT	TT	CT	TT	TT
AC	CC	AC	AC	AA	AC	AC	AC	CC	AA	CC	AA	AA
GG	GT	GG	--	TT	GG	GG	GT	TT	GT	GT	GG	GG
GG	AG	GG	--	AA	GG	GG	AG	AA	AG	AG	GG	GG
--	CT	CC	--	TT	CC	CC	CT	TT	CT	CT	CC	CC
--	AT	TT	--	AA	TT	TT	--	AA	AT	AT	TT	TT
AA	AC	AA	--	CC	AA	AA	AC	CC	AC	AC	AA	AA
CC	CC	CC	CC	CG	CC	CC	CG	CG	CG	CC	CC	CC
AA	AA	AA	AA	AG	AA	AA	AG	AG	AG	AA	AA	AA
--	CT	TT	TT	CC	TT	TT	CT	CC	CT	CT	TT	TT
TT	CT	TT	--	CC	TT	TT	CT	CC	--	CT	TT	TT
GG	AG	GG	--	AA	GG	GG	AG	AA	AG	AG	GG	GG
CC	CT	CC	--	CT	CC	CC	CC	CT	CC	CT	CC	CC
GG	AG	GG	--	AA	GG	GG	AG	AA	AG	AG	GG	GG
--	AG	AG	--	GG	AA	AA	AG	GG	AG	GG	AA	AA
TT	GT	GT	--	GG	TT	GT	--	GG	TT	GG	TT	--

CC	CT	CT	CC	TT	CC	CT	CT	TT	CC	TT	CC	CC
CC	CG	CG	CC	GG	CC	CG	CG	GG	CC	GG	CC	CC
GG	GT	GT	GG	TT	GG	GT	--	TT	GG	TT	GT	GG
GG	AG	AG	--	AA	GG	AG	AG	AA	GG	AA	AG	GG
TT	CT	CT	--	CC	TT	CT	CT	CC	TT	CC	CT	TT
TT	GT	TT	TT	GT	TT	TT	TT	GT	TT	GT	TT	TT
--	CG	CG	--	GG	CC	CG	CG	GG	CC	GG	CG	CC
--	AG	AG	--	AA	GG	AG	AG	AA	GG	AA	AG	GG
--	AG	GG	--	AG	GG	GG	GG	AG	GG	AG	GG	GG
CC	AC	AC	CC	AA	CC	AC	AC	AA	CC	AA	AC	CC
CC	CG	CC	CC	CG	CC	CC	CC	CG	CC	CG	CC	CC
AA	AG	AG	--	GG	AA	AG	AG	GG	AA	GG	AG	AA
--	AC	AC	--	AA	CC	AC	--	AA	CC	AA	AC	CC
GG	GT	GT	--	TT	GG	GT	GT	TT	GG	TT	GT	GG
--	AC	AC	--	CC	AA	AC	AC	CC	AA	CC	AC	AA
CC	CT	CC	CC	CT	CC	CC	--	CT	CC	CT	CC	CC
GG	GT	GT	--	TT	GG	GT	GT	TT	GG	TT	GT	GG
GG	GG	AG	--	AG	AG	AG	GG	AG	GG	AG	AG	GG
--	CT	CT	--	CC	CT	CT	CT	CC	TT	CC	CC	TT
CC	CT	CC	CC	CT	CC	CC	--	CT	CC	CT	CT	CC
--	AT	AT	--	AA	AT	AT	--	AA	TT	AA	AA	TT
GG	CG	CG	GG	CC	CG	CG	CG	CC	GG	CC	CC	GG
TT	GT	GT	TT	GG	GG	GT	GT	GG	TT	GG	GG	TT
GG	GG	AG	--	AG	AG	AG	AG	AG	GG	AG	AG	GG
GT	TT	GT	--	TT	TT	GT	--	GT	GT	GT	TT	GT
TT	CT	CT	--	CC	CC	CT	CT	CT	TT	CT	CC	TT
AA	AC	AC	--	CC	CC	AC	AC	AC	AA	AC	CC	AA
--	CT	CC	--	CC	CC	CC	--	CC	CT	CC	CC	CT
CC	CG	GG	CG	GG	GG	CG	GG	CG	CC	CG	GG	CC
TT	CT	CC	CT	CC	CC	CT	CC	CT	TT	CT	CC	TT
TT	TT	GT	GT	GT	GT	GT	GT	GT	TT	GT	GT	TT
TT	CT	CT	TT	CT	CT	TT	CT	TT	TT	TT	CT	TT
--	TT	AT	--	AT	AT	AT	AT	AT	TT	AT	AT	TT
AC	AC	CC	CC	CC	CC	CC	CC	CC	AC	CC	CC	AC
CC	CT	CT	--	TT	TT	CT	--	CT	CC	--	CT	CC
AG	AG	AA	--	AA	AA	AA	AA	AA	AG	AA	AA	AG
AG	AG	GG	--	GG	GG	GG	GG	GG	AG	GG	GG	AG
CG	CG	GG	--	GG	CG	CG	CG	CG	GG	GG	GG	CG
--	CT	CC	--	CC	CT	TT	CT	TT	CT	CT	CT	CT
TT	CT	CT	--	CT	CT	TT	CT	TT	CT	CT	CT	CT
CC	CT	CT	--	CT	CC	TT	CT	TT	CT	CT	CT	CT
--	CT	CT	--	CT	TT	CC	CT	CC	CT	--	CT	CT
GG	GT	GT	GG	GT	GG	TT	GT	TT	GT	GT	GT	GT
TT	CT	CT	--	CT	TT	CC	--	CC	CT	CT	CT	CT
AT	AT	AT	AT	AT	TT	AA	AT	AA	AT	AT	AT	TT
GG	AG	AG	--	AG	GG	AA	AG	AA	AG	AG	--	GG
AA	AC	AC	--	AC	AA	CC	AC	AC	AC	AC	AC	AA

GG	AG	AG	--	AG	GG	AA	AG	AG	AG	AG	AG	GG
GG	AG	AG	--	AG	AG	AA	AG	AG	AG	AG	AG	GG
CC	CT	CT	TT	CT	CC	TT	CT	CT	CT	CT	CT	CC
AA	AG	AG	--	AG	AG	GG	AG	AG	AG	AG	AG	AA
--	GT	GT	--	GT	GT	GG	GT	GT	GT	GT	GT	TT
--	CT	CT	--	CT	CT	TT	CT	CT	CT	CT	CT	CC
AA	AG	AG	--	AG	AG	GG	AG	AG	AG	AG	AG	AA
AA	AG	AG	--	AG	AG	GG	AG	AG	AG	AG	AG	AA
GG	AG	AG	--	AG	AG	AA	--	AG	AG	AG	AG	GG
CC	CG	CG	--	CG	CG	GG	CG	CG	CG	CG	CG	CC
GG	AG	AG	AA	AG	AG	AA	AG	AG	AG	AG	--	GG
--	AC	AC	AA	AC	AC	AA	--	AC	AC	AC	AC	CC
--	AG	AG	GG	AG	AG	GG	AG	GG	AG	AG	AG	AA
AA	AG	AG	GG	AG	AG	GG	--	GG	AG	AG	AG	AA
AT	AT	AT	AA	AT	AT	AA	AT	AA	AT	AT	AT	TT
CT	CT	CT	--	CT	CT	TT	CT	TT	CT	CT	CT	CC
CT	CT	CT	--	CT	CT	TT	CT	TT	CT	CT	CT	CC
CG	CG	CG	CC	CG	CG	CC	CG	CC	CG	CG	CG	GG
CT	CT	CT	CC	CT	CT	CC	CT	CC	CT	CT	CT	TT
CT	CT	CT	--	CT	CT	TT	CT	TT	CT	CT	--	CC
CT	CT	CT	CC	CT	CT	CC	CT	CC	CT	CT	CT	TT
AT	AT	AT	AA	AT	AT	AA	AT	AA	AT	AT	AT	TT
--	AT	AT	--	AT	AT	TT	AT	TT	AT	AT	AT	AA
CT	CT	CT	--	CT	CT	CC	CT	CC	CT	CT	CT	CT
CT	CT	CT	TT	CT	CT	TT	CT	TT	CT	CT	CT	CT
AT	AT	AT	TT	AT	AT	TT	AT	TT	AT	AT	AT	AT
AG	AG	AG	--	AG	AG	AA	AG	AA	AG	AG	AG	AG
GT	GT	GT	--	GT	GT	TT	--	TT	--	GT	GT	GT
CT	CT	CT	--	CT	CT	CC	--	CC	CT	CT	CT	CT
--	CG	CG	GG	CG	CG	GG	CG	GG	CG	CG	CG	CG
AG	AG	AG	AA	AG	AG	AA	AG	AA	AG	GG	AG	AG
--	CT	CT	--	CT	CT	CC	CT	CC	CT	TT	CT	CT
CT	CT	CT	--	CT	CT	CC	CT	CC	CT	TT	CT	CT
AT	AT	AT	AA	AT	AT	AA	--	AA	AT	TT	AT	AT
AG	AG	AA	--	AG	AG	GG	AG	GG	AG	AA	AG	AG
AG	AG	AA	GG	AG	AG	GG	AG	GG	GG	AA	AG	AG
GT	GT	TT	GT	GT	GT	GG	GT	GG	GG	TT	GT	GT
AG	AG	AG	AG	AG	AG	GG	--	GG	GG	AA	AG	AG
AG	GG	GG	--	AG	AG	AG	AG	AG	AG	GG	GG	AG
CT	TT	CT	--	TT	CT	CT	CT	CT	TT	TT	CT	TT
--	AG	AG	--	AG	AG	AG	AG	AG	GG	GG	AG	--
AG	AG	GG	--	AG	GG	GG	--	GG	GG	GG	GG	GG
--	GG	AG	--	AG	AG	GG	--	AG	GG	AG	AG	AG
GG	CC	CG	CG	CG	CG	CC	GG	CG	CC	CG	CG	CG
--	TT	CC	CT	CT	CC	CT	CC	CT	TT	CT	CT	CC
CC	AC	CC	CC	AC	CC	AC	CC	CC	AC	CC	CC	CC
AG	AG	AA	--	AA	AA	AA	--	AG	AA	AG	AG	--

CC	AC	CC	--	AC	CC	AC	--	CC	AC	CC	CC	CC
CC	CC	AC	--	AC	AC	AC	AC	CC	AC	CC	CC	AC
TT	CT	TT	TT	CT	TT	CT	TT	TT	CT	TT	TT	TT
AG	GG	AG	AG	AG	AA	AG	AA	AG	AG	AG	AG	AA
AG	AA	AG	AG	AA	GG	AG	--	AG	AG	AG	AG	GG
CT	CC	CT	--	CC	TT	CT	--	CT	CT	CT	CT	CT
--	TT	TT	--	TT	CT	CT	--	TT	CT	TT	TT	TT
CG	GG	CG	CG	GG	CC	CG	CG	CG	CG	CG	CG	CG
AG	GG	AG	AG	GG	AA	AG	GG	AG	GG	AG	AG	AG
--	CC	CT	--	CC	TT	CT	--	CT	CC	CT	CC	CT
--	AA	AA	--	AA	AA	AT	--	AA	--	AA	AA	AA
AC	AC	AA	AA	AA	AC	AC	AA	AC	AA	CC	AA	AC
GG	GG	AG	AG	AG	GG	AG	AG	GG	AG	GG	AG	GG
CC	CC	CT	--	CT	CC	CT	CT	CC	CT	CC	CT	CC
CC	CC	CT	CT	CT	CC	CT	CT	--	CC	CC	CT	CC
GG	GG	GT	--	GT	GG	GT	GT	GG	GT	GG	GT	GG
CT	CC	TT	--	CT	CT	CT	CT	CC	CC	CT	CT	CT
GG	AG	AG	--	AG	AG	GG	AG	AG	AG	GG	GG	AG
AG	AA	GG	AG	AG	AG	AG	AG	AA	AA	AG	AG	GG
GG	CG	CG	--	CG	CG	GG	--	CG	GG	GG	CG	CG
GG	AG	GG	--	AG	AG	AG	AG	AG	AA	AG	GG	GG
AA	AG	AA	--	AG	AG	AA	AG	AG	GG	AG	AA	AA
TT	TT	TT	TT	TT	TT	TT	TT	TT	GT	GT	TT	TT
AA	AG	AG	AA	AG	AG	AA	AG	AG	GG	AG	AA	AA
AG	AG	AG	--	AG	AG	AA	AG	AG	GG	AG	AA	AA
GG	GG	GG	GG	GG	GG	GG	GG	GG	GT	GT	GG	GG
--	CT	CT	--	CT	CT	CC	--	CT	--	CT	CC	CC
TT	CT	CT	--	CT	CT	TT	CT	TT	CC	CT	TT	TT
CC	CT	CT	CT	CT	CT	CT	--	CC	CT	CC	CC	CC
AG	AG	GG	AG	AG	GG	AG	AG	AA	AG	AG	AA	AA
CT	CT	TT	CT	CT	TT	CT	CT	CC	CT	TT	CC	CC
AG	AG	GG	AG	GG	GG	AG	GG	AA	AG	GG	AA	AA
CT	CT	TT	--	TT	TT	CT	TT	CC	CT	TT	CC	CC
CG	CG	CC	--	CC	CC	CG	CC	GG	CG	CC	GG	GG
CT	CT	TT	--	TT	TT	CT	--	CC	CT	TT	CC	CC
--	CC	AC	AC	AA	AC	AC	AC	AA	AA	CC	AC	AA
AG	AA	AG	--	GG	AG	AG	AG	GG	AG	AA	AG	GG
CT	CC	CT	--	TT	CT	CT	CT	TT	CT	CC	CT	TT
AG	GG	AG	AG	AA	AG	AG	AG	AG	AG	GG	AG	AA
AA	GG	AG	AG	AA	AG	AG	--	AG	AG	GG	AA	AA
TT	CT	TT	--	TT	CT	TT	TT	TT	TT	CT	TT	TT
--	AA	AG	AG	GG	AG	GG	--	AG	AG	AA	GG	GG
AA	AG	AG	--	AA	AA	AA	--	AG	AA	AG	AA	AA
TT	GT	GT	TT	TT	TT	TT	TT	GT	TT	GT	TT	TT
TT	GT	GT	--	TT	TT	TT	TT	GT	TT	GT	TT	TT
AA	CC	AC	--	AA	AC	AA	--	AC	AA	CC	AA	AA
AA	GG	AG	AG	AA	AG	AA	--	AG	--	GG	AA	AG

--	GG	CG	CG	CC	CG	CC	--	CG	CC	GG	CC	CG
--	GG	CG	CG	CC	CG	CC	CC	CG	CC	GG	CC	CG
TT	CC	CT	--	CT	CT	TT	TT	CT	CT	CC	TT	CT
CC	TT	CT	--	CT	CT	CC	CC	CC	CT	TT	CC	CT
AA	TT	AT	--	AT	AT	AA	AA	AA	AT	AT	AA	AT
AG	GG	GG	AG	GG	AG	AG	AG	AG	GG	GG	AG	AG
CT	TT	CT	--	CT	CT	CC	CC	CC	CT	CT	CC	CT
TT	CC	CT	--	CT	CT	TT	--	TT	CT	CT	TT	CT
--	TT	CT	--	CT	CT	CC	CC	CC	TT	CT	CC	CT
GG	AG	AG	--	AG	AG	GG	GG	GG	AA	AG	GG	AG
CC	CG	CC	--	CC	CG	CC	CC	CC	CG	CC	CC	CG
GG	GG	GG	--	CG	GG	CG	GG	GG	CG	GG	GG	GG
GG	GT	GG	--	GG	GT	GG	GG	GG	GT	GG	GG	GT
CG	CC	CC	CG	CC	CG	CG	CC	CG	CG	CC	CC	CG
AG	GG	GG	AG	AG	AG	AG	--	AA	AA	AG	GG	AA
AC	AA	AA	AC	AA	AC	AC	AA	AC	AC	AA	AA	AC
AT	AA	AA	AT	AA	AT	AT	AA	AT	AT	AA	AA	AT
AG	GG	GG	--	AG	AG	AG	--	AA	AA	AG	AG	AA
AT	AA	AA	TT	AT	AT	AT	AT	TT	TT	AT	AT	TT
AG	GG	GG	AA	AG	AA	AG	AG	AA	AA	AG	AG	AA
CC	CC	CC	--	CC	AC	CC	AC	--	AC	AC	AC	AC
CT	CC	CC	--	CT	TT	CT	CT	CT	TT	CT	TT	TT
GT	TT	TT	--	GT	GG	GT	GT	GT	GG	GT	GG	GG
GT	GT	TT	--	GT	GG	GG	GT	GG	GG	GT	GG	GG
CT	CT	CC	CT	CT	TT	TT	CT	TT	TT	CT	TT	TT
CG	CG	CC	CG	CG	GG	GG	CG	GG	GG	CG	GG	GG
AG	AG	GG	--	AG	AA	AA	AG	--	AG	AG	AA	AA
CT	CT	CC	--	CT	TT	TT	CT	TT	TT	CT	TT	TT
GT	GT	TT	GT	GT	GG	GG	GT	GG	GG	GT	GG	GG
CT	CT	TT	CT	CT	CC	CC	CT	CC	CC	CT	CC	CC
CG	CG	GG	--	CG	CC	CC	CG	CC	CC	CG	CC	CC
AG	AG	GG	AG	AG	AA	AA	AG	AA	AA	AG	AA	AA
AC	AC	AA	--	AC	CC	CC	AC	CC	CC	AC	CC	CC
CT	CT	TT	--	CT	CC	CC	CT	CC	CC	CT	CC	CC
AG	GG	GG	--	AG	AG	AG	--	AG	--	GG	AG	AG
--	AC	AA	--	AC	CC	CC	AC	CC	CC	AC	CC	CC
CT	CT	CC	CT	CT	TT	TT	CT	TT	TT	CT	TT	TT
AG	AG	GG	AG	AG	AA	AA	AG	AA	AA	AG	AA	AA
CG	CG	GG	--	CG	CG	CC	CG	CC	CC	CG	CC	CC
GT	GT	GG	GT	GT	GT	TT	GT	TT	TT	GT	TT	TT
CT	CT	CC	--	CT	CT	TT	CT	TT	TT	CT	TT	TT
CT	TT	TT	--	CT	TT	CT	TT	CT	CT	TT	CT	CT
AG	GG	AG	--	AG	GG	GG	GG	GG	GG	GG	GG	GG
CG	CG	CG	--	CG	CG	GG	GG	GG	CG	CG	GG	CG
AA	AT	AA	AA	AA	AT	AT	AT	AT	AT	AT	AT	AA
AG	AA	AG	AA	AG	AA	AG	AG	AG	AA	AA	AG	AG
AC	CC	AC	--	AC	CC	AC	AC	AC	CC	CC	AC	AC

CT	TT	CT	--	CT	TT	CT	CT	CT	TT	TT	CT	CT
TT	CT	TT	--	TT	TT	CT	CT	TT	CT	CT	CT	TT
GT	AG	GT	--	GT	GG	AT	AT	GT	AG	AG	GT	GT
AT	AA	AT	AA	AT	AA	AT	AT	AT	AA	AA	AT	AT
AG	AG	AG	--	AA	GG	AA	AG	AG	AG	GG	AG	AG
--	AC	AC	--	AA	CC	AA	AC	AC	AC	CC	AC	AC
AG	AA	AG	--	AG	AA	AG	AG	AG	AA	AA	AG	AG
AG	AG	AG	--	AA	GG	AA	AG	AG	--	GG	AG	AG
AG	AG	AG	--	GG	AA	GG	--	AG	AG	AA	AG	GG
AG	AG	AG	--	AA	AG	AA	AG	AA	AG	GG	AG	--
--	GT	GT	--	TT	GT	TT	GT	TT	GT	GG	GT	TT
AG	AG	GG	--	AG	AG	AG	GG	AG	AG	GG	GG	AG
--	CT	CT	--	TT	CT	TT	--	TT	CT	CT	CT	TT
GG	CG	CG	CC	GG	CG	GG	GG	GG	CG	CG	CG	GG
CT	TT	CT	TT	CT	TT	CT	CT	CT	TT	TT	CT	CT
CT	CT	TT	--	CT	CT	CT	--	CT	CT	CT	TT	CT
CT	CC	CT	CT	CC	CC	CT	CT	CT	CT	CC	CT	CC
GT	GG	GT	--	GG	GT	GG	GT	GT	GG	GG	GT	GG
AA	GG	AG	--	GG	AG	AG	AA	AA	AG	GG	AA	GG
GG	AA	AG	--	AA	AG	AG	--	GG	AG	AA	GG	--
AG	AA	AG	--	AA	AA	AG	AG	AG	AG	AA	AG	AA
TT	GG	TT	--	GG	GT	GT	TT	TT	GT	GG	TT	GG
AG	AA	AG	AG	AA	AA	AG	AG	AG	AA	AA	AG	AA
CT	CC	CT	--	CC	CC	CT	CT	CT	CC	CC	CT	CC
AA	AG	AG	--	AG	AG	AA	AA	AA	AA	AG	AA	AG
AA	GG	AG	--	GG	AA	AG	AA	AA	GG	AG	AA	GG
AA	AG	AG	AG	GG	AG	AG	AG	AA	GG	AG	AA	GG
CC	CG	CG	--	GG	CG	CG	CG	CC	GG	CC	CC	GG
CG	CG	CG	--	GG	CG	CG	--	CC	GG	CC	CG	GG
AG	AG	AG	AA	GG	AG	AG	AG	AA	GG	AA	AG	GG
--	AG	AG	AG	GG	GG	GG	GG	AG	GG	AG	GG	GG
CT	CT	CT	--	TT	CT	CT	--	CC	TT	CC	CT	TT
CC	AC	AC	AC	CC	CC	AC	CC	AC	AC	AC	AC	CC
CC	CG	CG	--	CC	CC	CG	--	CG	CG	CG	CG	CC
CC	CC	AC	AC	CC	CC	AC	--	AC	AC	AC	AC	CC
AC	AA	AC	--	AA	AC	AC	AC	CC	--	AC	CC	AA
AG	AA	AA	--	AA	AG	AA	AG	AG	AA	AG	AG	AA
AG	AA	AA	--	AA	AG	AG	AG	GG	AG	AG	GG	AA
AG	GG	GG	AG	GG	AG	GG	AG	AG	GG	AG	AG	GG
CT	CC	CC	TT	CT	CT	CT	TT	TT	CT	CT	TT	--
CT	CC	CC	--	CT	CT	CT	--	TT	CT	CT	TT	--
--	AA	AA	AG	AA	AA	AG	--	AG	AG	AA	AG	AA
CC	CC	CC	CC	CC	CC	CT	CC	--	CT	CC	CT	CC
AG	GG	AG	--	AA	AG	GG	--	AG	AG	AA	AA	AG
CC	CC	AC	--	AC	AC	CC	AC	CC	AC	AC	AC	AC
CT	CC	CC	CC	CT	CC	CC	CT	CT	CT	CT	CT	CC
--	AG	AG	--	GG	AG	AA	--	AG	GG	GG	GG	AG

AG	AA	AA	AA	AG	AA	AA	AG	AG	AG	AG	AG	AA
AG	AG	AA	--	GG	AG	AA	AG	AG	GG	GG	GG	AG
CC	AC	CC	CC	AA	AC	CC	AC	AC	AA	AA	AA	AC
GG	AG	GG	--	AA	AG	GG	AG	AG	AA	AG	AA	AG
--	GT	TT	--	GG	GT	GT	--	GT	GG	GT	GT	GT
AA	AG	AA	--	AG	AG	AG	AA	AA	AG	AG	AA	AA
TT	CT	CT	--	CC	CT	CT	CT	CT	CT	CT	CT	TT
CC	CT	CC	--	CT	CT	CT	--	CC	CC	CT	CC	CC
GG	AG	AG	GG	AA	AG	AA	AG	AG	AG	GG	GG	GG
--	GG	CG	--	CG	GG	CG	--	CG	CG	GG	GG	GG
--	TT	CT	--	CT	TT	CT	--	CT	CT	TT	TT	TT
AA	AG	AG	--	GG	AG	GG	--	GG	AG	AA	AA	GG
AA	AG	AG	AA	GG	AG	GG	GG	GG	AG	AA	AA	GG
--	CG	CG	--	GG	CG	GG	--	GG	CG	CC	CC	GG
--	AG	AG	--	GG	AG	GG	--	GG	AG	AA	AA	GG
AA	AA	AA	--	AA	AG	AG	AG	GG	AG	AG	AA	AG
CC	CC	CC	--	CC	CT	CT	CT	TT	TT	CT	CC	CT
AA	AA	AA	--	AA	AG	AG	AG	GG	GG	AG	AA	AG
GG	GG	GG	CC	GG	CG	CG	CG	CC	CC	CG	GG	CG
AA	AA	AA	--	AC	AC	AC	--	CC	CC	AC	AA	AC
GG	GG	GG	--	AG	AG	AG	AG	AA	AA	AG	GG	AG
TT	TT	TT	GG	GT	GT	GT	GT	GG	GG	GT	TT	GT
AA	AA	AA	--	AG	AG	AG	AG	GG	GG	AG	AA	AG
AA	AA	AA	--	AG	AG	AG	AG	GG	GG	AG	AA	AG
GG	GG	GG	--	AG	AG	AG	AG	AA	AA	AG	GG	AG
CC	CC	CC	AA	AC	AC	AC	AC	AA	AA	AC	CC	AC
GG	GG	GG	AA	AG	AG	AG	AG	AA	AA	AG	GG	AG
GG	GG	GG	AA	AG	AG	AG	AG	AA	AA	AG	GG	AG
CC	CC	CC	--	CT	CT	CT	CT	TT	TT	CT	CC	CT
CC	CC	CC	GG	CG	CG	CG	CG	GG	GG	CG	CC	CG
CC	CC	CC	--	AC	AC	AC	--	AA	AA	--	CC	AC
CC	CC	CC	--	CT	CT	CT	CT	TT	TT	CT	CC	CT
GG	GG	GG	--	GT	GT	GT	GT	TT	TT	GT	GG	GT
TT	TT	TT	CT	CT	CT	CT	--	CC	CC	CT	TT	CT
GG	GG	GG	--	CG	CG	CG	CG	CC	CC	CG	GG	CG
AA	AA	AA	--	AT	AT	AT	AT	TT	TT	AT	AA	AT
--	TT	CT	--	CT	CT	CT	--	CC	CC	CT	TT	CT
--	AA	AG	GG	AG	AG	AG	AG	GG	GG	AG	AA	AG
GG	GG	AG	AA	AG	AG	AG	AG	AA	AA	AG	GG	AG
TT	TT	AT	AA	AT	AT	AT	AT	AA	AA	AT	TT	AT
AA	AA	AG	GG	AG	AG	AG	AG	GG	GG	AG	AA	AG
GG	GG	AG	--	AG	AG	AG	AG	AA	AA	AG	GG	AG
GG	GG	CG	--	CG	CG	CG	CG	CC	CC	CG	GG	CG
GG	GG	AG	--	AG	AG	AG	AG	AA	AA	AG	GG	AG
CC	CC	CG	--	CG	CG	CG	CG	GG	GG	CG	CC	CG
AA	AA	AG	--	AG	AG	AG	--	GG	GG	AG	AA	AG
CC	CC	AC	--	AC	AC	AC	--	AA	AA	AC	CC	AC

AA	AA	AG	--	AG	AG	AG	AG	GG	GG	AG	AA	AG
GG	GG	CG	--	CG	CG	CG	CG	CC	CC	CG	GG	CG
TT	TT	CT	--	CT	CT	CT	CT	CC	CC	CT	TT	CT
AA	AA	AT	TT	AT	AT	AT	AT	TT	TT	AT	AA	AT
--	TT	CT	--	CT	CT	CT	CT	CC	CC	CT	TT	CT
TT	AT	AT	--	AT	AT	AT	AT	AA	AA	AT	TT	AT
AA	AT	AT	--	AT	AT	AT	AT	TT	TT	AT	AA	AT
CC	CG	CG	GG	CG	CG	CG	CG	GG	GG	CG	CC	CG
TT	CT	CT	--	CT	CT	CT	--	CC	CC	CT	CT	CT
GG	AG	AG	--	AG	AG	AG	AG	AA	AA	AG	AG	AG
CC	AC	AC	AA	AC	AC	AC	AC	AA	AA	AC	AC	AC
GG	GT	GT	--	GT	GT	GT	GT	TT	TT	GT	GT	GT
TT	CT	CT	CT	CT	CT	CT	CT	CC	CC	CT	CT	CT
CC	CG	CG	CG	CG	CG	CG	CG	GG	GG	CG	CG	CG
GG	GT	GT	--	GT	GT	GT	GT	TT	TT	GT	GT	GT
CC	CT	CT	CT	CT	CT	CT	CT	TT	TT	CT	CT	CT
GG	AG	AG	--	AG	AG	AG	AG	AA	AA	AG	AG	--
CC	AC	AC	--	AC	AC	AC	AC	AA	AA	AC	AC	AC
TT	CT	CT	--	CT	CT	CT	CT	CC	CC	CT	CT	CT
CC	AC	AC	--	AC	AC	AC	AC	AA	AA	AC	AC	AC
GG	AG	AG	AG	AG	AA	AG	AG	AA	AA	AG	AG	AG
TT	GT	GT	GT	GT	GG	GT	GT	GG	GG	GT	GT	GT
TT	GT	GT	--	GT	GG	GT	GT	GG	GG	GT	GT	GT
AA	AG	AG	AG	AG	GG	AG	AG	GG	GG	AG	AG	AG
TT	CT	CT	--	CT	CC	CT	CT	CC	CC	CT	CT	CT
TT	CT	CT	CT	CT	CC	CT	CT	CC	CC	CT	CT	CT
GG	AG	AG	AG	AG	AA	AG	AG	AA	AA	AG	AG	AG
TT	CT	CT	CT	CT	CC	CT	CT	CC	CC	CT	CT	CT
CC	CT	CT	CT	CT	TT	CT	CT	TT	TT	CT	CT	CT
AA	AG	AG	--	AG	GG	AG	--	GG	GG	AG	AG	AG
TT	CT	CT	CT	CT	CC	CT	CC	--	CC	CT	CT	CT
CT	TT	TT	--	TT	TT	CT	TT	TT	TT	CT	TT	TT
TT	CT	CT	--	CT	CT	CT	--	CC	CC	CT	CT	CT
GG	AG	AA	AG	AG	AG	AA	GG	AA	AA	AG	AG	AG
CC	CC	AA	AC	AC	AC	AA	CC	AC	AC	AC	AC	AC
CC	CC	CG	CG	CC	CC	CG	CC	CC	CG	CG	CC	CC
--	TT	GG	--	TT	GT	GG	--	GT	GT	GT	GT	GT
AG	AA	GG	--	AG	AG	GG	AG	AG	AG	AG	AG	AG
CT	CC	TT	--	CT	CT	TT	CT	CT	CT	CT	CT	CT
GG	GG	AG	AG	AG	GG	AG	AG	GG	AG	AG	GG	GG
--	AA	AG	--	AG	AG	GG	AG	AG	AG	AG	AG	AG
CG	CC	CG	--	CG	CG	GG	CG	CG	CC	CG	CG	CG
--	TT	CT	--	CT	TT	CT	CT	TT	TT	CT	TT	TT
GT	GG	GT	TT	GT	GT	TT	GT	GT	GG	GT	TT	GT
AG	AG	GG	--	GG	GG	AG	GG	GG	GG	AG	AG	AG
GG	GG	GG	AG	GG	GG	GG	AG	GG	GG	AG	AG	GG
AC	AC	AA	CC	AA	AA	AC	AC	AA	AA	CC	AC	AC

AT	AT	TT	AA	TT	AT	AT	AT	TT	TT	TT	AT	AT
AT	AT	TT	AA	TT	AT	AT	--	TT	TT	TT	AT	AT
AT	AT	AA	--	AA	AT	AT	AA	AA	AA	AA	AT	AT
CT	CT	TT	--	TT	CC	CT	TT	TT	TT	TT	CT	CT
GG	AG	AG	AA	GG	AA	AG	AG	GG	GG	GG	AG	AG
AA	AC	AC	CC	AA	CC	AC	AC	AA	AA	AA	AC	AC
--	CT	CT	--	TT	CC	CT	CT	TT	TT	TT	CT	CT
GG	AA	AG	--	GG	AA	AG	--	GG	GG	GG	AG	AG
GG	TT	GT	TT	GG	TT	GT	TT	GT	GG	GG	GG	GT
GG	AA	AG	--	GG	AA	AG	--	AG	GG	GG	GG	AG
CC	CG	CC	--	CC	CG	CG	CG	CG	CC	CC	CC	CG
GG	AG	GG	AG	GG	AG	AG	AG	AG	GG	GG	GG	AG
CC	GG	CG	GG	CC	GG	CG	GG	CG	CC	CC	CG	CG
AA	GG	AG	GG	AA	GG	AG	GG	AG	AA	AA	AG	AG
GG	AA	AG	AG	GG	AA	AG	--	--	GG	GG	AG	AG
AA	GG	AG	--	AA	AG	AG	GG	GG	AA	AA	AG	AG
GG	GT	GG	GT	GG	GT	GT	GT	GT	GG	GG	GG	GT
GG	AA	AG	AG	GG	AG	AA	AA	AA	GG	GG	AG	AG
TT	GT	TT	GT	TT	GT	GT	GT	GT	TT	TT	TT	GT
CG	CC	GG	CG	GG	CG	CC	CC	CG	GG	GG	CG	CG
--	CT	CT	--	CT	TT	CT	--	TT	CT	TT	CT	CT
--	CG	CC	--	CG	CG	CC	CG	CG	CG	GG	CG	CC
CC	CC	CG	--	CC	CG	CG	CG	CG	CC	CC	CC	CG
AA	AC	AC	AC	AC	AA	AC	AA	AA	AC	AA	AC	AC
--	CT	CT	CT	CT	CC	CT	--	CC	CT	CC	CT	CT
AA	AG	AG	AG	AG	AA	AG	AG	AG	AG	AG	AG	AG
CC	CC	CT	--	CT	CC	CT	CT	CT	CT	CT	CT	CT
GG	GG	CG	--	GG	CG	GG	CG	CG	GG	CG	GG	CG
--	CC	CT	--	CC	CC	CT	CT	CT	CT	CT	CT	CC
AA	AA	GG	AA	AA	AA	AG	GG	GG	AG	AG	AG	AG
GG	GG	GG	--	GG	GG	GT	GT	GT	GT	GG	GT	GG
TT	TT	GT	TT	GT	TT	GT	GG	GG	GT	GT	GT	TT
--	GG	GG	--	GG	GG	CG	CG	CG	CG	GG	CG	GG
GG	GG	CG	--	CG	GG	CG	--	CC	CG	CG	CG	GG
GG	GG	GG	GG	GG	GG	AG	AG	AG	AG	GG	AG	GG
TT	TT	CT	--	CT	TT	CT	CC	CC	CT	CT	CT	TT
GG	GG	GT	--	GT	GG	GT	--	TT	GT	GT	GT	GG
GG	GG	GG	--	GG	GG	AG	AG	AG	AG	GG	AG	GG
--	AA	AG	--	AG	AA	AG	GG	GG	AG	AG	AG	AA
--	TT	CT	--	CT	TT	CT	--	CC	CT	CT	CT	TT
TT	TT	TT	--	TT	TT	AT	AT	--	AT	TT	AT	TT
TT	TT	TT	--	TT	TT	GT	GT	GT	GT	TT	GT	TT
CC	CC	CT	--	CT	CC	CT	--	--	CT	CT	CT	CC
AA	AA	AA	AG	AA	AA	AG	AG	AG	AG	AA	AG	AA
TT	TT	AT	--	AT	TT	AT	--	AA	AT	AT	AT	TT
AA	AA	AA	AG	AA	AA	AG	AA	AG	AG	AA	AG	AA
TT	TT	TT	--	TT	TT	CT	TT	CT	CT	TT	CT	TT

TT	TT	TT	AT	TT	TT	AT	--	AT	AT	TT	AT	TT
GG	GG	GT	--	GT	GG	GT	GT	TT	GT	GT	GT	GG
AA	AA	AG	--	AG	AA	AG	AG	GG	AG	AG	AG	AA
CC	CC	CT	CT	CT	CT	CT	CT	TT	CT	CT	CT	CC
CC	CT	CT	--	CT	CT	CT	CT	TT	CT	CT	CT	CC
--	CT	CT	CT	CT	CT	CT	--	TT	CT	CT	CT	CC
GG	GT	GT	GT	GT	GT	GT	GT	TT	GT	TT	GT	GG
CG	CG	CG	CG	CG	CG	CG	CG	GG	CG	GG	CG	CC
AC	AC	AC	--	AC	AC	CC	AC	AC	AC	AA	AC	CC
AG	AG	AG	--	AG	AG	AA	GG	AG	AG	GG	AG	AA
AG	AG	AG	--	AG	AG	GG	--	AG	AG	AA	AG	GG
CC	CT	CT	CT	CT	CC	CT	TT	CT	CT	CC	TT	CT
CC	AC	AC	AC	AC	CC	AC	AA	AC	AC	CC	AA	AC
GT	TT	GT	--	GT	GT	TT	TT	TT	TT	GT	TT	TT
CG	CG	GG	--	GG	CG	CG	--	CG	CG	CG	GG	CG
--	AG	AG	AG	AG	AA	AG	AG	AG	AG	AG	GG	AG
CT	CT	TT	TT	TT	CT	CT	TT	CT	CT	TT	TT	CT
GT	TT	GT	--	GT	GT	TT	GT	GT	TT	GT	TT	TT
CT	CT	CC	--	CC	CT	CT	CC	CT	CT	CC	CC	CT
AA	AA	AC	AC	AC	AC	AA	--	AC	AA	AC	AA	AA
AG	AG	AG	--	AG	GG	AG	AG	GG	AG	AG	AA	AG
AA	AA	AG	--	AG	AG	AG	AG	AG	AA	AG	AA	AA
--	GT	TT	--	GT	GT	GG	GT	GG	GT	GT	TT	GT
CC	CT	CC	--	CT	CT	TT	--	TT	CT	CT	TT	CT
AA	AC	AA	AC	AC	AC	CC	AC	AC	AC	AC	CC	AC
TT	AT	TT	--	AT	AT	AA	AT	AT	--	AT	AA	AT
GT	GT	GT	--	GG	GG	GG	--	GG	GT	GG	GG	GT
AG	AA	AG	--	AG	AG	AA	AG	AG	AA	AG	AA	AA
CC	CG	CC	--	CG	CG	GG	CG	CG	CG	CG	GG	CG
--	AG	AA	--	AG	AG	GG	--	GG	AG	AG	GG	AG
CG	CC	CG	CG	CG	GG	GG	--	CC	GG	CG	GG	GG
AG	AA	AG	AG	AG	GG	GG	AG	AA	GG	AG	GG	GG
--	AA	AG	AG	AG	AG	AG	AG	AA	AG	AA	AG	AG
AA	AA	AA	--	AT	AT	AT	--	AA	AT	AT	AT	AT
--	AA	AG	--	AG	AG	AA	AG	AA	AG	AA	AG	AG
AA	AA	AA	--	AA	AA	AG	AA	AG	AA	AG	AA	AA
AC	AC	AC	AC	CC	CC	AC	AC	AA	CC	AC	CC	CC
--	CC	--	--	AC	AC	AC	CC	CC	AC	AC	AC	AC
--	AC	AA	--	AA	AA	AC	AC	CC	AA	AC	AA	AA
TT	GT	TT	GT	TT	TT	GT	GT	GG	--	GT	TT	TT
TT	AT	AT	--	TT	TT	AT	--	AA	AT	AT	TT	TT
AG	AA	AA	AA	AG	AG	AG	AA	AA	AA	AG	AG	AA
AC	CC	AC	CC	AC	AC	AC	CC	CC	CC	AC	AC	AC
TT	TT	AT	--	TT	TT	AT	AT	AA	AT	AT	TT	AT
TT	TT	CT	TT	TT	TT	CT	CT	CC	CT	TT	TT	CT
AA	AA	AG	--	AA	AA	AG	AG	GG	AG	AA	AA	AG
TT	TT	CT	--	TT	TT	CT	CT	CC	CT	TT	TT	CT

--	CC	CT	--	CC	CC	CT	--	TT	CT	CC	CC	CT
AA	AA	AG	--	AA	AA	AG	--	GG	AG	AA	AA	AG
TT	TT	CT	TT	TT	TT	CT	CT	CC	TT	TT	TT	CT
--	CC	CT	--	CC	CC	CT	CT	TT	CC	CC	CC	CT
TT	TT	CT	--	TT	TT	CT	CT	CC	TT	TT	TT	CT
AA	AA	AG	--	AA	AA	AG	AG	GG	AA	AA	AA	AG
CC	CC	CT	--	CC	CC	CT	--	TT	CC	CC	CC	CT
--	AA	AG	--	AA	AA	AG	AG	GG	AA	AA	AA	AG
GG	GG	CG	--	CG	GG	CG	CG	CC	GG	GG	GG	CG
CC	CC	AC	--	AA	CC	AC	AC	AA	CC	AC	CC	AC
TT	TT	CT	TT	CC	TT	CT	CT	CC	TT	CT	TT	CT
--	TT	CT	--	CC	TT	CT	--	CC	TT	CT	TT	CT
AA	AA	AT	--	TT	AA	AT	AT	TT	AA	AT	AA	AT
TT	TT	CT	--	CC	TT	CT	--	CC	TT	CT	TT	CT
CC	CC	AC	CC	AA	CC	AC	AC	AA	CC	AC	CC	AC
GG	GG	GT	--	TT	GG	GT	GT	TT	GG	GT	GG	GT
CC	CC	CT	--	TT	CC	CT	--	TT	CC	CT	CC	CT
CC	CC	CG	CC	GG	CC	CG	CG	GG	CC	CG	CC	CG
--	CC	CG	--	GG	CC	CG	CG	GG	CC	CG	CC	CG
AA	AA	AC	--	CC	AA	AC	AC	CC	AA	AC	AA	AC
CC	CC	CG	--	GG	CC	CG	CG	CG	CC	CG	CC	CG
AT	AT	AT	TT	AT	AT	AT	AT	TT	TT	AT	TT	AA
GG	AG	AG	GG	AG	AA	AG	--	GG	GG	AA	GG	AA
GG	AA	AA	--	AG	AA	AG	--	--	AG	AA	GG	AA
AA	GG	AG	AG	GG	GG	AG	AG	AG	AG	GG	AA	GG
GG	AA	AG	--	AA	AA	AG	--	AG	AG	AA	GG	AA
CC	AA	AC	AC	AA	AA	AC	AC	AC	AC	AA	CC	AA
GG	AA	AG	AG	AA	AA	AG	--	AG	AG	AA	GG	AA
GG	AA	AG	AG	AA	AA	AG	AG	AG	AG	AA	GG	AA
TT	GG	GT	--	GG	GG	GT	GT	GT	GT	GG	TT	GG
GG	TT	GT	GT	TT	TT	GT	GT	GT	GT	TT	--	TT
AA	CC	AC	AC	CC	CC	AC	AC	AC	AC	CC	AA	CC
CC	TT	CT	--	TT	TT	CT	CT	CT	CT	TT	CC	TT
--	TT	CT	CT	TT	TT	CT	CT	CT	CT	TT	CC	TT
TT	GG	GT	--	GG	GG	GT	GT	GT	GT	GG	TT	GG
TT	AA	AT	--	AA	AA	AT	--	AT	AT	AA	TT	AA
CC	GG	CG	CG	GG	GG	CG	CG	CG	CG	GG	CC	GG
--	AA	AC	--	AA	AA	AC	AC	AC	AC	AA	CC	AA
--	CC	CT	CT	CC	CC	CT	CT	CT	CT	CC	TT	CC
CC	GG	CG	--	GG	GG	CG	--	CG	CG	GG	CC	GG
TT	CC	CT	--	CC	CC	CT	CT	CT	CT	CC	TT	CC
TT	AA	AT	--	AA	AA	AT	AT	AT	AT	AA	TT	AA
CC	TT	CT	CT	TT	TT	CT	CT	CT	CT	TT	CC	TT
AA	GG	AG	AG	GG	GG	AG	AG	AG	AG	GG	AA	GG
TT	AA	AT	AT	AA	AA	AT	AT	AT	TT	AA	TT	AA
TT	AA	AT	--	AA	AA	AT	AT	AT	AT	AA	AT	--
CC	AA	AC	AC	AA	AA	AC	AC	AC	AC	AA	AC	AA

GG	TT	GT	GT	TT	TT	GT	GT	GT	GT	GT	GT	TT
AA	GG	AG	AG	GG	GG	AG	AG	AG	AG	AG	AG	GG
TT	GG	GT	GT	GG	GG	GT	GT	GT	GT	GT	GT	GG
GG	AA	AG	AG	AA	AA	AG	AG	AG	AG	AG	AG	AA
--	TT	CT	--	TT	TT	CT	CT	CT	CT	CT	CT	TT
GG	AA	AG	AG	AA	AA	AG	AG	AG	AG	AG	AG	AA
CC	TT	CT	CT	TT	TT	CT	CT	CT	CT	CT	CT	TT
AA	CC	AC	--	CC	CC	AC	AC	--	AC	AC	AC	CC
--	AA	AG	AG	AA	AA	AG	AG	AG	AA	AG	AG	AA
CC	TT	CT	--	TT	TT	CT	CT	CT	CT	CT	CT	TT
AG	GG	AG	--	GG	GG	AG	AG	AG	AG	AG	AG	GG
--	CT	CT	--	CC	CC	CT	CT	CT	TT	CT	CT	CC
GT	GG	GT	--	GG	GG	GG	--	GT	GT	GG	GT	GG
GG	AG	GG	--	GG	GG	AG	AG	GG	AG	AG	GG	AG
AT	AT	AT	--	TT	AT	AT	--	AT	AT	AT	AA	AT
AT	AT	AT	AT	TT	AT	AT	--	AT	AT	AT	AA	AT
AC	AA	AC	--	CC	AC	AC	--	AC	AC	AC	AA	AA
GT	TT	GT	GT	GG	GT	GT	--	GT	GT	TT	TT	TT
CT	TT	CT	CT	CT	TT	TT	--	--	CT	TT	CT	TT
AG	GG	AA	--	AA	AG	AA	AA	AG	AA	AG	AG	GG
CG	CG	GG	CG	GG	GG	GG	GG	CG	GG	GG	CG	CG
CT	TT	TT	CT	TT	TT	TT	--	CT	TT	TT	CT	CT
GG	GT	GG	GG	GG	GT	GG	GG	GG	GG	GT	GG	GT
AA	AG	AA	--	AA	AG	AA	AA	AG	AA	AG	AG	GG
AA	AA	AC	--	AA	AA	AA	--	AC	--	AA	AC	AC
GG	GG	CG	--	GG	GG	GG	GG	CG	GG	GG	CG	CG
TT	TT	GT	--	TT	TT	TT	TT	GT	TT	TT	GT	GT
CC	CT	CT	CT	CC	CT	CC	CC	TT	CC	CT	TT	TT
AG	GG	AA	--	AG	GG	AG	AA	AG	--	GG	AG	AG
--	CC	CT	--	CC	CC	CC	CT	CT	CT	CC	CT	CT
CG	CG	CC	CG	CG	CC	CC	CC	CC	CC	CG	CG	CG
AA	AA	AC	--	AC	AA	AC	AC	AA	AC	AA	AA	AC
AG	AG	AG	--	AG	AA	AG	AG	AA	AG	AG	AG	GG
CT	CT	TT	--	TT	CT	TT	TT	CT	TT	CT	CT	TT
GG	GG	CG	GG	CG	CG	CG	--	CG	CG	GG	GG	GG
AG	AG	AG	--	AG	GG	AG	AG	GG	AG	AG	AG	AA
CG	CG	GG	CG	CG	GG	GG	--	GG	GG	CG	CG	CG
CG	CG	CG	--	CC	GG	CG	CG	GG	CG	CG	CG	CC
CT	CT	CT	CT	CC	TT	CT	CT	TT	CT	CT	CT	CC
AG	AG	AG	--	GG	AA	AG	AG	AA	AG	AG	AG	GG
GG	GG	AG	--	AG	GG	AG	AG	GG	AG	GG	GG	AG
CT	CT	CT	CT	CT	TT	CT	CT	TT	CT	CT	CT	CC
AG	AG	GG	AG	GG	AG	GG	GG	--	GG	AG	AG	GG
AG	AG	AG	AG	AG	AA	AG	AG	AA	AG	AG	AG	GG
AC	AC	AC	--	AC	AA	AC	--	AA	AC	AC	AC	CC
TT	TT	AT	--	AT	AT	AT	AT	AT	TT	TT	TT	TT
AG	AG	AG	--	AG	GG	AG	AG	GG	AA	AG	AG	AA

AC	AC	AC	AC	AC	CC	AC	AC	CC	AA	AC	AC	AA
AG	AG	AG	AG	AG	GG	AG	AG	GG	AA	AG	AG	AA
AG	AG	AA	AG	AA	AG	AA	AA	AG	AA	AG	AG	AA
CC	CC	CT	--	CT	CT	CT	CT	CT	CC	CC	--	CC
CC	CC	CG	CG	CG	CG	CG	CG	CG	CC	CC	CC	CC
--	TT	CT	--	CT	CT	CT	TT	CT	TT	CT	TT	CT
--	CG	GG	--	GG	GG	GG	--	GG	CG	GG	--	GG
CC	CC	CT	CT	CT	CT	CT	CC	CT	CC	CT	CC	CT
GT	GT	GG	--	GG	GG	GG	GT	GG	GT	GG	GT	GG

4-67	4-72	5-1	5-3	5-4	5-5	5-6	5-7	5-8	5-10	5-11	5-13	5-20	5-22	5-23
AC	AC	AC	AC	AA	AA	AC	AC	CC	AA	AA	AA	AC	CC	AC
AG	GG	AG	AG	AA	AA	AG	AG	AG	AA	AA	AA	AG	GG	AG
CT	CC	CT	TT	TT	TT	CT	TT	CC	TT	TT	TT	CT	CC	CT
GG	AG	AG	GG	GG	GG	AG	GG	AG	GG	GG	GG	AG	AG	AG
AG	GG	AG	AA	AA	AA	AG	--	GG	AA	AA	AA	AG	GG	AG
GG	GG	CG	CG	CG	CG	CG	CG	GG	CG	CG	CG	CG	GG	CG
CT	CC	CT	TT	TT	TT	CT	TT	CC	TT	TT	TT	CT	CC	CT
GT	GG	GT	TT	TT	TT	GT	TT	GG	TT	TT	TT	GT	GG	GT
GT	GG	GT	TT	GT	GT	GT	TT	GT	TT	GT	TT	GT	GG	GT
GT	GG	GT	TT	GT	GT	GG	TT	GG	TT	GT	--	GT	GG	GT
AG	GG	AG	AG	AG	AG	GG	AA	GG	AA	GG	AA	AG	GG	AG
CT	CT	--	CT	CC	CC	CC	CT	CC	CT	CC	CT	CT	CC	CT
AA	AC	AA	AC	CC	AC	CC	--	CC	AA	CC	AA	AC	CC	AC
AG	AG	AG	AG	--	AA	AA	AG	AA	AG	AA	AA	AG	AA	AG
CT	CC	CT	CC	CC	CT	CC	CT	CC	CT	CC	CT	CC	CC	CC
GG	AG	GG	AG	AA	GG	AA	GG	AA	GG	AA	AG	AG	AG	GG
AT	AT	AT	AT	AT	AT	TT	--	TT	AT	TT	TT	AT	AT	AT
CT	CT	CT	CC	CC	CT	CC	CT	CC	CT	CC	CT	CC	CC	CT
GT	GT	TT	GT	GT	GT	TT	TT	TT	GT	TT	TT	GT	GT	GT
CG	CG	CC	CG	CG	CG	CC	CC	CC	CG	CC	CC	CG	CG	CC
AA	AA	AT	TT	AT	AA	TT	AT	TT	AA	TT	--	AT	AT	AT
CT	CC	--	TT	CT	CC	TT	CT	TT	--	TT	--	CT	CT	CT
AT	TT	AT	AA	AA	TT	AA	AT	AA	TT	AA	TT	AT	AT	AT
AT	TT	AT	AA	AT	TT	AA	AT	AA	TT	AA	TT	AT	AT	AT
AG	GG	AG	AA	AG	GG	AA	AG	AA	GG	AA	--	AG	AG	AG
TT	GT	TT	TT	GT	GT	TT	TT	TT	GT	TT	GT	GT	GT	TT
GT	GG	GT	TT	GT	GG	TT	GT	TT	GG	TT	GG	GT	GT	GT
AT	AA	--	TT	AA	AA	TT	AT	--	AA	TT	AA	AT	AT	AT
CT	CC	CT	TT	TT	CC	TT	CT	TT	CC	TT	CC	CT	CT	CT
CG	GG	CG	CC	CC	GG	CC	CG	CC	GG	CC	GG	CG	CG	CG
CT	TT	CT	CC	CC	TT	CC	CT	CC	TT	CC	TT	CT	CT	CT
AC	CC	--	AA	AA	CC	AA	AC	--	CC	AA	CC	AC	AC	AC
AG	GG	--	AA	AA	GG	AA	--	AA	--	AA	GG	AG	AG	AG
GT	TT	GT	GG	GG	TT	GG	GT	GG	TT	GG	TT	GT	GT	GT
AG	GG	AG	AA	AG	GG	AA	AG	AA	GG	AA	GG	AG	AG	AG
GT	GG	--	TT	--	--	TT	GT	TT	GG	TT	GG	GT	GT	GT
AG	AA	AG	GG	AG	AA	--	AG	GG	AA	GG	AA	AG	AG	AG
CT	TT	CT	CC	CC	TT	CC	CT	CC	TT	CC	TT	CT	CT	CT
AT	TT	AT	AA	AA	TT	AA	AT	AA	TT	AA	TT	AT	AT	AT
AG	GG	--	AA	AA	GG	AA	AG	AA	GG	AA	GG	AG	AG	AG
AC	CC	AC	AA	AA	CC	AA	AC	AA	CC	AA	--	AC	AC	AC
AG	GG	AG	AA	--	GG	AA	AG	AA	AG	AA	GG	AG	AG	AG

CT	CC	CT	TT	TT	CC	TT	CT	TT	CC	TT	CC	CT	CT	CT
CG	GG	CG	CC	CC	GG	CC	CG	CG	GG	CC	GG	CG	CG	CG
CT	CC	CT	TT	CT	CC	TT	CT	--	CC	TT	CC	CT	CT	CT
CT	TT	CT	CC	CC	TT	CC	CT	CC	TT	CC	--	CT	CT	CT
AC	CC	AC	AA	AA	CC	AA	CC	AA	CC	AA	CC	AC	AC	AC
CT	TT	CT	CT	CC	TT	CC	TT	CC	CT	CC	--	CC	CC	CT
CC	CC	TT	CT	TT	CC	TT	CC	TT	CT	TT	CC	TT	TT	CT
AG	AA	AA	GG	AG	AG	AG	AG	AG	--	AG	AG	AG	GG	AA
TT	TT	GT	GG	GT	GT	GT	GT	GT	GG	GT	GT	GT	GG	TT
AA	AG	--	GG	AG	AG	AG	GG	AG	GG	AG	AG	AG	GG	AA
CC	CT	CT	CT	CT	CT	CT	TT	--	TT	CT	CT	CT	TT	CC
GG	CG	CG	CG	CG	CG	CG	CC	GG	CC	CG	CG	GG	CC	GG
GG	AG	AG	AG	AG	AG	AG	AA	GG	AA	AG	AG	GG	AA	GG
AC	AC	AC	AC	AC	AC	AC	CC	AA	CC	AC	AC	AA	CC	AA
GG	GG	--	AG	GG	GG	GG	AG	GG	AG	AG	AG	GG	AG	GG
CT	CC	--	TT	--	CT	CT	TT	CC	TT	CT	TT	CC	CT	CT
CT	TT	TT	CC	CT	CT	CT	CT	--	CC	CT	CC	TT	CT	CT
CT	CC	CC	TT	CT	CT	CT	CT	CC	CT	CT	TT	CC	CT	CT
AG	GG	GG	AA	AG	AG	AG	AG	GG	AG	AG	AA	GG	AG	AG
AC	CC	CC	AA	AC	AC	AC	AC	CC	AC	AC	AA	CC	CC	AC
AG	GG	AG	AA	AG	AG	AG	AG	GG	AG	AG	AA	GG	GG	AG
CG	CC	CG	GG	CG	CG	CG	CG	CC	CG	CG	GG	CC	CC	CG
CG	GG	CG	CC	GG	CG	CG	CG	GG	CG	CG	CC	GG	GG	CG
AG	GG	AG	AA	GG	AG	AG	AG	GG	AG	AG	AA	GG	GG	AG
AA	AA	--	CC	AA	AC	AC	AC	AA	AC	AC	CC	AA	AA	AC
CC	CC	CT	TT	CC	CT	CT	CT	CC	CT	TT	TT	CC	CC	CT
TT	CT	--	--	TT	CT	CT	CT	TT	CT	CC	CC	TT	TT	CT
AA	AT	AT	TT	AA	AT	AA	AT	AA	AT	TT	TT	AA	AA	AA
AG	GG	--	GG	GG	GG	AG	--	AG	GG	GG	--	AG	AG	AG
AT	TT	TT	TT	--	TT	AT	AT	AT	TT	TT	TT	AT	TT	AT
GG	TT	TT	GG	TT	GG	TT	GT	GG	GG	GT	--	TT	GT	GT
AG	AG	--	AG	--	AG	GG	AG	AG	AG	AG	--	GG	GG	AG
CT	TT	TT	CT	TT	CT	TT	CT	CT	CT	CT	TT	TT	TT	CT
AG	GG	--	AG	GG	AG	GG	AG	AG	AG	AG	GG	GG	GG	AG
TT	GG	GG	TT	GT	TT	GG	GT	GT	TT	GT	GG	GG	GT	GT
GT	--	--	GT	GT	GT	GG	GT	GT	GT	GG	--	GG	GT	GT
CT	TT	TT	CC	CT	CT	CC	CT	CT	CC	TT	TT	TT	CC	CT
CT	TT	--	CC	--	CT	CC	CT	CT	CC	TT	TT	TT	CC	CT
AG	AA	AA	GG	AG	AG	GG	AG	AG	GG	AA	AA	AA	GG	AG
CG	GG	GG	CC	CG	CG	CC	--	CG	CC	GG	GG	GG	CC	CG
AG	AA	AA	GG	AG	AG	GG	AG	AG	GG	AA	AA	AA	GG	AG
CT	CC	--	TT	CT	CT	TT	CT	CT	--	CC	CT	CC	TT	CT
CT	CT	--	CT	CT	CT	TT	CT	CT	CT	CC	CT	CC	TT	CT
CG	CG	CC	CC	CG	CG	CG	CG	CG	CC	CC	CG	CC	CG	CG
AT	AT	--	TT	AT	TT	AT	TT	AT	--	TT	AT	TT	AT	AT
GG	GG	GG	GT	GG	GG	GT	GG	GG	GT	GG	GG	GG	GG	GT
CC	CC	CC	CT	CC	CC	CT	CC	CC	CT	CC	CC	CC	CC	CT

CT	CT	--	CT	CT	CC	TT	CC	CT	CT	CC	CT	CC	CC	CT
GG	GG	GG	GT	GG	GG	GT	GG	GG	--	GG	GG	GG	GG	GT
CT	CT	CT	CT	CT	CC	TT	CC	CT	CT	CC	CC	CC	CC	CT
TT	TT	TT	GT	TT	TT	GT	TT	TT	GT	TT	TT	TT	TT	GT
GG	GG	--	CG	--	GG	CG	GG	GG	CG	GG	--	GG	GG	CG
AG	GG	AG	GG	AG	GG	GG	GG	GG	GG	AG	GG	AA	AG	AG
AG	AG	--	AA	AG	AA	AA	AA	AA	AA	AG	AA	AG	AG	AG
AA	AG	AG	AG	AG	AA	AA	AA	AA	AA	AG	AA	AG	AG	AG
TT	CT	CT	CT	CT	TT	TT	TT	TT	TT	CT	TT	CT	CT	CT
GG	AG	--	AG	--	AG	GG	GG	GG	GG	AG	GG	AG	AG	AG
GG	GT	GT	GT	--	GT	--	--	GG	--	GT	--	GT	GT	GT
AG	GG	GG	GG	GG	GG	GG	AG	AG	AG	AG	AG	AG	AG	AG
TT	CT	CT	CT	CC	CT	CT	TT	TT	TT	CT	TT	CT	CT	CT
CC	CC	CC	CC	AC	CC	CC	CC	CC	CC	AC	CC	AC	AC	AC
CT	CT	CT	CT	CC	CT	CT	CT	CT	CT	CC	CT	CC	CC	CC
GG	GT	GT	GT	TT	GT	GT	GG	GG	GG	GT	GG	GT	GT	TT
CC	CT	CT	CT	CT	CT	CT	CC	CC	CC	CC	CC	CC	CC	CT
GG	GG	GG	GG	CG	GG	GG	GG	GG	GG	CG	GG	CG	CG	CG
CC	CC	--	CC	AC	CC	CC	CC	CC	CC	AC	--	AC	AC	AC
TT	CT	CT	CT	CT	CT	CT	TT	TT	TT	TT	TT	TT	TT	CT
AA	AG	AG	AG	AG	AG	AG	AA	AA	AA	AA	AA	AA	AA	AG
AA	AG	AG	AG	GG	AG	AG	AA	AA	AA	AG	AA	AG	AG	GG
CT	CT	CC	CT	CC	CT	CT	CT	CT	CT	CC	CT	CC	CC	CC
AA	AA	--	AA	AT	AA	AA	AA	AA	AA	AT	AA	AT	AT	AT
CC	CC	CC	CC	CT	CC	CC	CC	CC	CC	CT	CC	CT	CT	CT
AA	AA	AA	AA	AG	AA	AA	AA	AA	AA	AG	AA	AG	AG	AG
CC	CT	CT	CT	CT	CT	CT	CC	CC	CC	CC	CC	CC	CC	CT
TT	AT	--	AT	AA	AT	AT	TT	TT	TT	AT	TT	AT	AT	AA
TT	AT	AT	AT	AT	--	--	TT	TT	TT	AT	TT	AT	AT	AA
TT	CT	CT	CT	CC	CT	CT	TT	TT	TT	CT	TT	CT	CT	CC
CC	CC	CC	CC	CT	CC	CC	CC	CC	CC	CT	CC	CT	CT	CT
GG	GT	GT	GT	GT	GT	GT	GG	GG	--	GG	GG	GG	GG	GT
CC	CT	CT	CT	CT	CT	CT	CC	CC	CC	CT	CC	CC	CC	CT
AG	AA	--	AA	AA	AA	AA	AG	AG	AG	AA	AG	AG	AG	AA
GG	AG	AG	AG	AA	AG	AG	GG	GG	--	AA	--	AG	AG	AA
AA	AG	AG	AG	AG	AG	AG	AA	AA	AA	AG	AA	AA	AA	AG
CC	CT	CT	CT	TT	CT	CT	CC	CC	CC	TT	CC	CT	CT	TT
CC	AC	AC	AC	--	AC	AC	CC	CC	CC	AC	CC	CC	CC	AC
TT	AT	AT	AT	AA	AT	AT	TT	TT	TT	AA	TT	AT	AT	AA
AA	AA	--	AA	AG	AA	AA	AA	AA	AA	AG	AA	AG	AG	AG
AA	AC	AC	AC	AC	AC	AC	AA	AA	AA	AC	AA	AC	AA	AC
TT	AT	AT	AT	AT	AT	AT	TT	TT	TT	AT	TT	AT	TT	AT
AC	AA	AA	AA	AC	AA	AA	AC	AC	AC	AC	AC	AC	CC	AC
CC	CC	CC	CC	AC	CC	CC	CC	CC	CC	AC	CC	AC	AC	AC
AG	GG	--	GG	GG	GG	GG	AG	AG	AG	GG	AG	GG	AG	GG
AC	AC	CC	AC	CC	AC	AC	AC	AC	AC	CC	AC	CC	CC	CC
AA	AG	AG	AG	AG	AG	AG	AA	AA	AA	AG	AA	AG	AA	AG

CC	CC	CC	CC	CG	CC	CC	CC	CC	CC	CG	CC	CG	CG	CG
TT	CT	CT	CT	CC	CT	CT	TT	TT	TT	CC	TT	CC	CT	CC
CC	CT	CT	CT	TT	CT	CT	CC	CC	CC	TT	CC	TT	CT	TT
CC	CT	CT	CT	CT	CT	CT	CC	CC	--	CT	--	CT	CC	CT
AA	AG	--	AG	AG	AG	AG	AA	AA	AA	AG	--	AG	AA	AG
CC	CT	CT	CT	TT	CT	CT	CC	CC	--	TT	CC	TT	CT	TT
TT	CT	--	CT	CT	CT	CT	CT	TT	TT	CT	TT	CT	TT	CT
AA	AT	--	AT	TT	AT	AT	AA	AA	AA	TT	AA	TT	AT	TT
GG	GG	GG	GG	AG	GG	GG	GG	GG	GG	AG	GG	AG	AG	AG
AA	AC	AC	AC	AC	AC	AC	AC	AA	AA	AC	AA	AC	AA	AC
CC	CT	CT	CC	CT	CT	CT	CT	CC	CC	CT	CC	CT	CC	CT
TT	TT	--	TT	GT	TT	TT	TT	TT	TT	GT	TT	GT	GT	GT
TT	CT	CT	--	CT	CT	CT	CT	TT	CT	CT	--	CT	TT	CT
AC	CC	CC	AC	CC	CC	CC	CC	AC	AC	CC	AC	CC	AC	CC
AA	AG	--	AA	AG	AG	AG	AG	AA	AA	AG	AA	AG	AA	AG
GG	GT	GT	GG	GG	GT	GT	GT	GG	GG	GT	--	GT	GG	GT
AT	TT	--	AT	TT	TT	TT	--	AT	AT	TT	AT	TT	AT	TT
AA	AG	--	AA	AG	AG	AG	AG	AA	AA	AG	AA	AG	AA	AG
CC	CC	--	CC	CT	CC	CC	CC	CC	CC	CT	--	CT	CT	CT
CC	CT	CT	CC	--	CT	CT	CT	CC	CC	CT	--	CT	CC	CT
AA	AC	AC	AA	AC	AC	AC	AC	AA	AA	AC	AA	AC	AA	AC
CC	CT	CT	CC	CT	CT	CT	CT	CC	CC	CT	CC	CT	CC	CT
AA	AT	--	AA	--	AT	AT	AT	AA	AA	TT	AA	TT	AT	TT
GG	AG	AG	GG	AA	AG	AG	AG	GG	GG	AA	GG	AA	AG	AA
GG	GG	GG	GG	CG	GG	GG	GG	GG	GG	CG	--	CG	CG	CG
TT	CT	--	TT	CT	CT	--	CT	TT	--	CT	--	CT	TT	CT
GG	AG	AG	GG	AG	AG	AG	AG	GG	GG	AG	--	AG	GG	AG
AA	AG	AG	AA	AA	AG	AG	AG	AA	AA	AG	AA	AG	AG	AG
AA	AA	AA	AA	AG	AA	AA	AA	AA	AA	AG	AA	AG	AG	AG
GG	GG	GG	GG	AG	GG	GG	GG	GG	GG	AG	GG	AG	AG	AG
TT	CT	--	TT	TT	TT	CT	CT	TT	--	TT	TT	CT	CT	CT
GG	GT	GT	GG	GT	GG	GT	GT	GG	GG	GT	GG	TT	TT	TT
TT	TT	TT	TT	GT	TT	TT	TT	TT	TT	GT	TT	GT	GT	GT
GG	AG	--	AG	--	GG	AG	AG	GG	GG	AG	GG	AA	AG	AA
CC	CT	CT	CC	CT	CC	CT	CT	CC	CC	CC	CC	CT	CC	CT
TT	GT	GT	TT	GT	TT	GT	GT	GT	TT	TT	TT	GT	TT	GT
AG	AG	AG	AG	GG	AG	--	AG	AG	AG	GG	--	GG	GG	GG
AG	AG	AG	AG	GG	AA	GG	AG	AA	GG	GG	AA	AG	AG	AG
CT	CC	CT	CC	CC	CT	CC	CT	CT	CC	CC	CT	CC	CT	CC
AT	AA	AT	AA	AT	AA	AT	AT	AA	AT	AT	AA	AA	AT	AA
CG	CG	CG	CG	GG	CC	GG	CG	CC	GG	GG	CC	CG	GG	CG
AA	AC	AA	AC	AC	AA	AC	AA	AA	AC	AC	AA	AC	AC	AC
AG	AG	AG	AG	--	AA	GG	AG	AA	AG	GG	AA	AG	GG	AG
GG	AG	GG	AG	GG	AG	GG	AG	AG	AG	GG	AG	AG	GG	AG
CC	CT	CT	CT	CT	CT	CC	CT	CT	CT	CC	CT	CT	CC	CT
AG	AG	GG	AG	AA	AG	AA	GG	GG	AG	AA	GG	AG	AA	AG
CC	CC	CC	CT	CT	CT	CT	CC	CC	CT	CT	CC	CT	CT	CT

AG	GG	GG	GG	AG	GG	AG	GG	GG	GG	AG	GG	GG	GG	GG
AA	AG	--	--	AG	GG	AG	AG	AG	GG	AG	AG	GG	GG	GG
CT	CC	CC	CT	TT	CT	TT	CC	CC	CT	TT	CC	CT	CT	CT
AT	AA	AA	AT	TT	AT	AT	AA	AA	AT	TT	AA	AT	AT	AT
AC	AA	--	AC	AC	AC	AC	AA	AA	AC	CC	AA	AC	AC	AC
CT	CT	--	CT	--	CT	TT	--	--	--	CC	TT	CT	CT	CT
AG	AG	--	AG	GG	AG	GG	GG	GG	AG	AA	GG	AG	AG	AG
CT	CT	--	CT	CC	TT	CC	CC	CC	CT	TT	CC	TT	CT	CT
TT	CT	--	CT	TT	CT	TT	TT	TT	TT	CT	TT	CT	TT	CT
AG	AG	AA	AG	AA	GG	AA	AA	AA	AA	GG	AA	GG	AA	AG
GG	AG	--	GG	AA	AG	GG	GG	GG	GG	GG	AG	AG	GG	AG
TT	TT	CT	TT	--	CT	TT	TT	TT	TT	TT	CT	CT	TT	CT
AG	GG	--	AG	--	GG	AG	AG	AG	AG	AG	GG	AG	AG	GG
TT	CT	--	TT	CC	CT	TT	--	TT	--	TT	CT	CC	TT	CT
CG	CG	CG	CG	GG	CG	CG	CG	CG	CG	CG	CG	GG	CG	CG
TT	--	CT	CT	CC	CT	TT	CT	TT	TT	TT	CT	CC	TT	CT
CT	CC	CC	CC	CC	CC	CT	--	CT	CT	CT	CC	CC	CT	CC
AG	AG	AG	AG	GG	AG	AA	AG	AA	AG	AA	AG	GG	AA	AG
CT	CT	--	CT	CT	CT	TT	CT	TT	CT	TT	--	CC	TT	CT
AA	AG	AG	AG	AG	AG	GG	AG	AG	AG	GG	AG	AA	GG	AG
CC	CT	CT	CT	CT	TT	TT	CT	CT	CT	TT	CT	CC	TT	CC
GG	AG	AG	AG	AG	AA	AA	AG	AG	AG	AG	AG	GG	AA	GG
GG	GG	GG	GG	GG	AG	AG	GG	GG	AG	GG	GG	GG	AG	GG
TT	GT	GT	GT	GT	GG	GG	GT	GT	GT	GT	GT	GT	GG	TT
CC	CC	CC	CC	CC	CG	CG	CC	CC	CC	CC	CC	CC	CG	CC
CG	CC	--	CG	CC	CC	CC	CC	CC	CG	CC	CC	CC	CC	CG
CC	CT	--	CC	CT	TT	TT	TT	CT	--	CT	CT	CT	CT	CT
TT	CT	CT	TT	CT	CC	CC	CC	CT	TT	CT	CT	CT	CT	CT
AA	AT	--	AA	--	TT	TT	TT	AT	AA	AT	TT	AT	AT	AT
GG	AG	AG	GG	AG	AA	AA	AA	AG	AG	AG	AA	AG	AG	AG
CC	CT	--	CC	CT	TT	TT	TT	TT	CT	CT	TT	CT	CT	CT
TT	TT	TT	TT	TT	CT	CT	CT	CT	CT	TT	CT	CT	CT	CT
AA	AC	AC	AA	AC	--	CC	CC	CC	AC	AC	CC	CC	AC	AC
TT	GG	--	GT	--	TT	GG	GG	GT	GG	GG	GT	GT	GT	GT
AA	GG	--	AG	GG	AA	GG	GG	AG	GG	GG	AG	AG	AG	AG
TT	CC	CT	CT	CC	TT	CC	CC	CT	CC	CC	--	CT	CT	CT
AA	TT	--	AT	TT	AA	TT	TT	AT	TT	TT	--	AT	AT	AT
CC	AA	AC	AC	AA	CC	AA	--	AC	--	AA	AC	AC	AC	AC
CG	CC	CC	CC	CC	CG	CC	CC	CG	CC	CC	CG	CC	CG	CG
AG	AA	--	AA	AA	AG	AA	AA	AG	AA	AA	AG	AA	AG	AG
CC	TT	CT	CT	TT	CC	TT	TT	CT	TT	TT	CT	--	CT	CT
CC	TT	CT	CT	TT	CC	TT	TT	CT	TT	TT	CT	CT	CT	CT
AA	GG	AG	AG	GG	AA	GG	GG	AG	GG	GG	AG	AG	AG	AG
CT	CC	CT	CT	CC	CT	CC	CC	CC	CC	CC	CC	CT	CC	CC
AA	GG	AG	AG	GG	AA	GG	--	AG	GG	GG	--	AG	AG	AG
GG	AA	AG	AG	AA	GG	AA	AA	AG	AA	AA	--	AG	AG	AG
--	TT	GT	--	TT	--	TT	TT	GT	TT	TT	GT	GT	GT	GT

TT	CC	--	CT	CC	TT	CC	CC	CT	CC	CC	CT	CT	CT	TT
GG	CC	CG	CG	CC	GG	CC	CC	CG	CC	CC	CG	CG	CG	GG
TT	GG	GT	GT	GG	TT	GG	GG	GT	GG	GG	GT	GT	GT	TT
AA	GG	AG	AG	GG	AA	GG	GG	AG	GG	GG	AG	AG	AG	AA
CT	TT	CT	CT	TT	CC	--	TT	CT	TT	TT	CT	CT	CT	CC
TT	TT	GT	GT	TT	GT	TT	TT	TT	TT	TT	TT	GT	TT	GT
CG	CC	--	CG	CC	GG	CC	CC	CG	CC	CC	CG	CG	CG	GG
AG	GG	AG	AG	GG	AA	GG	GG	AG	GG	AG	GG	AG	AG	AA
GG	GG	AG	AG	--	AG	GG	GG	GG	GG	GG	--	AG	GG	AG
AC	CC	AC	AC	CC	AA	CC	CC	AC	AC	AC	AC	AC	AC	AA
CC	CC	CG	CG	CC	CG	CC	CC	CC	CG	CC	CC	CG	CC	CG
AG	AA	AG	AG	AA	GG	AA	AA	AG	AG	AG	AG	AG	AG	--
AC	CC	--	AC	CC	AA	CC	CC	AC	AC	AC	--	AC	AC	AA
GT	GG	GT	GT	GG	TT	GG	GG	GT	GT	GT	GT	GT	GT	TT
AC	AA	AC	AC	AA	CC	AA	AA	AC	AC	AC	AC	AC	AC	CC
CC	CC	CT	CT	CC	CT	CC	CC	--	CT	CC	CC	CT	CC	CT
GT	GG	--	GT	GG	TT	GG	GG	GT	GT	GT	GT	GT	GT	TT
AG	GG	GG	GG	GG	AG	GG	GG	AG	GG	AG	AG	GG	AG	AG
CT	TT	CT	CT	TT	CC	TT	--	CT	CT	CT	--	CT	CT	CC
CC	CC	--	CT	CC	CT	CC	CC	CC	--	CC	CT	CT	CC	CT
AT	TT	AT	AT	TT	AA	TT	TT	AT	AA	AT	AA	AT	AT	AA
CG	GG	CG	CG	GG	CC	GG	GG	CG	CC	CG	CC	CG	CG	CC
GT	TT	GT	GT	TT	GG	GT	TT	GT	GG	GT	GG	GT	GT	GG
AG	GG	GG	GG	AG	AG	GG	GG	AG	AG	AG	AG	GG	AG	AG
GT	GT	--	GT	GT	TT	TT	GT	GT	--	GT	TT	TT	GT	TT
CT	TT	TT	TT	CT	CC	CT	TT	TT	CC	CT	CT	CC	CT	CC
AC	AA	AA	AA	--	CC	AC	AA	AA	CC	AC	AC	CC	AC	CC
CC	CT	CT	CT	CC	--	CT	CT	CT	--	CC	CT	CC	CC	CC
CG	CC	CC	CC	CG	CG	CG	--	CC	GG	CG	CG	CG	CG	GG
CT	CT	TT	TT	CT	CT	CT	TT	TT	CC	CT	CT	CT	CT	CT
GT	GT	--	TT	GT	GT	TT	TT	TT	GT	GT	TT	GT	GT	GT
TT	TT	--	TT	TT	TT	CT	TT	TT	TT	TT	CT	TT	CT	TT
TT	AT	--	TT	AT	--	TT	TT	TT	AT	AT	--	AT	AT	AT
AC	CC	AC	AC	CC	CC	AC	AC	AC	CC	CC	AC	CC	CC	CC
CC	CT	--	CC	CT	CT	--	CC	CC	CT	TT	CT	CT	TT	CT
AG	AA	AG	AG	AA	AA	AG	AG	AG	AA	AA	AG	AA	AA	AA
AG	GG	AG	AG	--	GG	AG	AG	AG	GG	GG	AG	GG	GG	GG
GG	GG	CG	GG	GG	CG	CG	--	GG	CG	CG	--	GG	CG	CG
CC	CC	CT	CC	CT	CT	TT	CT	CC	--	CT	--	CT	CT	CT
CC	CC	CT	CC	--	CT	TT	CT	CC	TT	CT	CT	CC	CT	TT
CC	CC	CT	CT	CC	CT	CT	CT	CT	CT	CT	CT	CC	CT	TT
TT	TT	--	CT	CT	CT	CT	TT	CT	CT	CT	--	TT	CT	CC
GG	GG	GT	GT	GT	GT	GT	GG	GT	GT	GT	GT	GG	GT	TT
TT	TT	--	CT	--	CT	CT	TT	CT	TT	CT	CT	TT	CT	CC
TT	TT	--	AA	AT	TT	TT	TT	AT	TT	AT	AT	TT	AT	AA
AG	AG	AG	AA	AG	GG	GG	GG	AG	GG	AG	GG	GG	AG	AA
AC	AC	CC	CC	AC	AA	AA	AA	AC	AA	AC	AA	AA	AC	CC

AG	AG	AA	AA	AA	GG	GG	GG	AG	GG	AG	GG	GG	AG	AA
AG	AG	--	AA	--	GG	GG	GG	AG	GG	AG	GG	GG	AG	AA
CT	CT	TT	TT	CT	CC	CC	CC	CT	CC	CT	CC	CC	CT	TT
AG	AG	--	GG	AG	AA	AA	AA	AG	AA	AG	AA	AA	AG	GG
GT	--	GG	GG	GT	TT	TT	--	GT	--	GT	--	TT	GT	GG
CT	CT	--	TT	CT	CC	CC	CC	CT	CC	CT	--	CC	CT	TT
AG	AG	--	GG	AG	AA	AA	AA	AG	AA	AG	--	AA	AG	GG
AG	AG	--	GG	AG	AA	AA	AA	AG	AA	AG	AA	AA	AG	GG
AG	AG	--	AA	--	GG	GG	GG	AG	GG	AG	--	GG	AG	AA
CG	CG	--	GG	CG	CG	CC	CC	CG	CC	CG	CC	CC	CG	GG
AG	AG	AA	AA	AG	AG	GG	GG	AG	GG	AG	GG	GG	AG	AA
AC	AC	--	AA	--	AC	CC	CC	AC	--	AC	CC	CC	AC	AA
AG	AG	GG	GG	AG	AG	AA	AA	AG	AA	AG	--	AA	AG	GG
AG	AG	GG	GG	AG	AG	AA	AA	AG	AA	AG	AA	AG	AG	GG
AT	AT	AA	AA	AT	AT	TT	TT	AT	TT	TT	TT	AT	AT	AA
CT	CT	TT	TT	CT	CT	CC	CC	CT	CC	CC	CC	CT	CT	TT
CT	CT	--	TT	CT	CT	CC	CC	--	--	CC	--	CT	CT	TT
CG	CG	CC	CC	CG	CG	GG	GG	CC	GG	GG	GG	CG	CG	CC
TT	CT	CC	CC	TT	CT	TT	TT	CC	TT	TT	TT	CT	CT	CC
CC	CT	TT	TT	CT	CT	CC	CC	TT	CC	CC	CC	CT	CT	TT
TT	CT	CC	CC	CT	CT	TT	TT	CC	TT	TT	TT	CT	CT	CC
TT	AT	AA	AA	AT	AT	TT	TT	AA	TT	TT	TT	AT	AT	AA
AA	AT	TT	TT	AT	AT	AA	AA	TT	AA	AA	AA	AT	AT	TT
TT	CT	CC	CC	CT	CT	TT	TT	CC	TT	TT	TT	CT	CT	CC
CC	CT	TT	TT	CT	CT	CC	CC	TT	CC	CC	CC	CT	CT	TT
AA	AT	TT	TT	AT	AT	AA	AA	TT	AA	AA	AA	AT	AT	TT
GG	AG	--	AA	AG	AG	GG	--	AA	GG	GG	GG	AG	AG	AA
GG	GT	--	TT	GT	GT	GG	GG	TT	GG	GG	GG	GT	GT	TT
TT	CT	CC	CC	CT	CT	TT	TT	CC	--	TT	TT	CT	CT	CC
CC	CG	GG	GG	CG	CG	CC	CC	GG	CC	CC	CC	CG	CG	GG
GG	AG	AA	AA	AG	AG	GG	GG	AA	GG	GG	GG	AG	AG	AA
TT	--	--	CC	CT	CT	TT	--	CC	TT	TT	TT	CT	CT	CC
TT	CT	CT	CC	CT	CT	TT	CT	CC	TT	TT	TT	CT	CT	CC
TT	AT	AT	AA	AT	AT	TT	AT	AA	TT	TT	TT	AT	AT	AA
AA	AG	AG	GG	AG	AG	AA	AG	GG	AA	AA	AA	AG	AG	GG
AA	AG	AG	GG	GG	AG	AA	AG	GG	AA	AA	AA	AG	AG	GG
TT	GT	GT	GG	GG	GT	TT	GT	GG	TT	TT	TT	GT	GT	GG
AA	AG	AG	GG	GG	AG	AA	AG	GG	AA	AA	AA	AG	AG	GG
AG	AG	--	AG	AG	--	GG	AG	AG	GG	GG	GG	AG	GG	AG
TT	CT	CT	TT	TT	TT	CT	TT	CT	CT	TT	CT	CT	TT	TT
GG	AG	--	AG	AG	GG	--	AG	AG	AA	AG	--	AG	AG	AG
GG	GG	--	AG	AG	GG	AG	AG	GG	AG	AG	GG	GG	AG	AG
GG	AG	--	AA	AG	AG	AA	GG	AG	AG	AG	--	AG	AG	AA
CC	CG	CG	GG	GG	CG	GG	CC	CG	CG	CG	CG	CG	CG	GG
CT	CT	CT	CC	CC	CT	CC	TT	CT	CT	CT	CT	CT	CT	CC
AC	CC	--	CC	CC	CC	CC	AC	CC	CC	CC	CC	CC	AC	CC
AA	AG	--	AG	AA	AA	AA	AG	AG	AG	AA	--	AG	AA	AA

AC	CC	CC	CC	CC	CC	CC	AC	CC	CC	CC	CC	CC	AC	CC
AC	CC	AC	CC	AC	AC	AC	CC	CC	CC	AC	AC	CC	AC	AC
CT	TT	TT	TT	TT	TT	TT	CT	TT	TT	TT	TT	TT	CT	TT
AG	AA	AA	AG	AG	AA	AA	GG	AG	AA	AA	AA	AG	AG	AA
AG	GG	GG	GG	AG	GG	GG	AA	AG	GG	GG	GG	AG	AG	GG
CT	TT	TT	TT	CT	CT	TT	CC	CT	TT	TT	TT	CT	CC	TT
CT	CT	--	CT	TT	TT	CT	TT	TT	CT	CT	CT	TT	TT	TT
CG	CC	CG	CC	CG	CG	CC	--	CG	CC	CC	CC	CG	GG	CG
AG	AA	--	AA	AG	AG	AG	--	AG	AA	AA	AA	AG	GG	AG
CT	TT	CT	TT	CT	CT	CT	CC	CT	TT	TT	--	CT	CC	CT
AT	AT	--	AT	AA	AA	AT	AA	--	AT	AT	--	AA	AA	AA
AC	CC	AC	CC	CC	AC	AC	AA	AC	CC	CC	CC	AC	--	AC
GG	--	--	GG	GG	GG	AG	--	GG	GG	GG	GG	GG	GG	GG
CC	CC	CC	CC	CC	CC	CT	CT	CC	CT	CC	--	CC	CC	CC
CC	CC	CC	CC	CC	CC	CT	CT	CC	CT	CC	CC	CC	CC	CC
GG	GG	--	GG	GG	GG	GT	GT	GG	GT	GG	GG	GG	GG	GG
TT	CT	CT	TT	CC	CC	CT	CC	TT	CC	CC	TT	TT	CC	TT
GG	GG	AG	GG	AG	AG	GG	AG	GG	AG	AG	GG	GG	AG	AG
GG	AG	AG	GG	AA	--	AG	AA	--	AA	AA	GG	GG	AA	GG
CG	CG	CG	CG	GG	GG	GG	GG	GG	GG	CG	CG	CG	GG	GG
GG	GG	AG	GG	AA	AA	AG	AA	AG	AA	AG	GG	GG	AA	AG
AG	AA	--	AA	GG	GG	AG	GG	AG	AG	AG	AA	AA	GG	AG
GT	TT	TT	TT	GT	GT	GT	GT	GT	TT	TT	TT	TT	GT	GT
AG	AA	AG	AA	GG	GG	AG	GG	AG	AG	AG	AA	AA	GG	AG
AG	AA	AG	AA	GG	GG	AG	--	AG	--	AG	AG	AA	GG	AG
GT	GG	GG	GT	GT	GT	GT	GT	GT	GG	GG	GT	GG	GT	GT
CT	CC	CT	CT	TT	TT	CT	TT	CT	CT	CT	TT	CC	CT	CT
CT	TT	CT	CT	CC	CC	CT	CC	CT	CT	CT	CC	TT	CT	CT
CC	CC	CT	CT	CT	CT	CC	CT	CC	CT	CT	CT	CC	CC	CC
AG	AA	AG	GG	AG	GG	AG	AG	AA	AG	AG	GG	AG	AA	AG
CT	CC	CT	TT	CT	CT	CC	CT	CC	CT	CT	TT	CT	CC	CT
GG	AA	GG	GG	AG	AG	AA	AG	AA	AG	AG	GG	AG	AA	AA
TT	CT	CT	TT	CT	CT	CC	CT	CC	--	CT	--	CT	CC	CC
CC	CG	CG	CG	CG	CG	GG	--	GG	CG	CG	--	CG	GG	GG
TT	CT	CT	CT	TT	CT	CC	CT	CC	CT	CT	TT	CT	CC	CC
AA	AC	AC	AA	--	AC	AA	AC	AA	AC	AC	AC	AA	CC	AA
GG	AG	--	AG	AA	AG	GG	AG	GG	AG	AG	AG	GG	AA	AG
TT	CT	TT	CT	CT	CT	TT	CT	TT	CT	CT	CT	TT	CC	CT
AA	AG	AA	GG	AG	AG	AA	--	AA	AA	AG	AG	AA	GG	AG
AA	AG	--	GG	AG	AG	AA	AG	AA	AA	AG	AG	AA	GG	AG
TT	CT	--	CT	CT	CT	TT	CT	TT	TT	CT	CT	TT	CT	TT
GG	AG	--	--	AG	AG	GG	AG	--	GG	AG	AA	GG	AA	AG
AA	AA	--	AG	AA	AA	AA	--	AA	AA	AA	AG	AA	AG	AG
TT	TT	TT	GT	TT	TT	TT	TT	TT	TT	TT	GT	TT	GT	GT
TT	TT	TT	GT	TT	TT	TT	TT	TT	TT	TT	--	TT	GT	GT
AA	AC	AA	CC	CC	AC	AA	AC	AA	AA	AC	CC	AA	CC	AC
AA	AG	--	GG	GG	AG	AA	AG	AA	--	AG	GG	AA	GG	AG

CC	CG	--	GG	GG	CG	CC	CG	CC	CC	CG	GG	CC	GG	CG
CG	CG	CC	GG	GG	CG	CC	CG	CG	CC	CG	GG	CC	GG	CG
CT	CT	TT	CC	CC	CT	TT	CT	CT	TT	CT	--	TT	CC	CT
CT	CT	CC	TT	TT	CT	CC	CT	CT	--	TT	TT	CC	TT	CT
AT	AA	AT	TT	TT	AA	AA	--	AT	AT	TT	TT	AA	TT	AT
GG	AG	GG	GG	GG	AG	AG	GG	GG	GG	GG	GG	AG	GG	GG
CT	CC	CT	TT	TT	CC	CT	--	CT	TT	TT	--	CC	TT	CT
CT	TT	--	CC	CC	TT	CT	CC	CT	CT	CC	CT	TT	CC	CT
CT	--	--	TT	--	CC	CT	TT	CT	CT	TT	CT	CC	TT	CT
AG	GG	AA	AA	AA	GG	AG	AA	AG	AA	AA	AG	GG	AA	AG
CC	CC	CG	CG	--	CC	CC	CG	CC	CG	CG	--	CG	CG	CC
CG	GG	--	CG	CG	GG	CG	CG	CG	CG	CG	GG	GG	CG	CG
GG	GG	GT	GG	GG	GG	GG	GT	GG	GT	GT	GG	GT	GT	GG
CC	CC	CC	CC	CG	CG	CC	CC	CG	CC	CC	CG	CG	CG	CC
GG	GG	AG	AG	--	AG	GG	--	AG	GG	GG	AG	AG	AA	AG
AA	AA	AA	AA	AC	AC	AA	AC	AC	AA	AA	AC	AC	AC	AA
AA	AA	AA	AA	AT	AT	AA	AA	AT	AA	AA	AT	AT	AT	AA
GG	GG	AG	AG	AG	AG	GG	GG	AG	AG	AG	AG	AG	AA	AG
AA	AA	AT	AT	AT	AT	AA	AA	AT	AT	AT	AT	AT	TT	TT
GG	GG	--	AG	AG	AG	GG	GG	AG	AG	AG	AG	AG	AG	AA
CC	AC	CC	AC	CC	CC	CC	CC	CC	--	AC	CC	CC	AC	AC
CC	CT	CT	CT	CT	CT	CC	CC	CT	CT	CT	CT	CT	CT	TT
GT	GT	GT	GT	GT	GT	TT	TT	GT	GT	GT	GT	GT	GT	GG
GT	GT	GT	GT	TT	GT	GT	TT	GT	GT	GT	GT	GT	GT	GG
CT	CT	CT	CT	CC	CT	CT	CC	CT	CT	CT	CT	CT	CT	TT
CG	CG	CG	CG	CC	CG	CG	CC	CG	CG	CG	CG	CG	CG	GG
AG	AG	AG	AG	GG	AG	AG	GG	AG	AG	AG	AG	AG	AG	AA
CT	CT	CT	CT	CC	CT	CT	CC	CT	CT	CT	CT	CT	CT	TT
GT	GT	GT	GT	TT	GT	GT	TT	GT	GT	GT	GT	GT	GT	GG
CT	CT	CT	CT	TT	CT	CT	TT	CT	CT	CT	CT	CT	CT	CC
CG	CG	CG	CG	GG	CG	CG	GG	CG	CG	CG	CG	CG	CG	CC
AG	AG	AG	AG	GG	AG	AG	GG	AG	AG	AG	AG	AG	AG	AA
AC	AC	AC	AC	AA	AC	CC	AA	AC	AC	AC	AC	AC	AC	CC
CT	CT	CT	CT	TT	CT	CC	TT	CT	CT	CT	CT	CT	CT	CC
GG	GG	--	GG	--	AG	GG	GG	AG	GG	GG	AG	AG	GG	AG
AC	AC	AC	AC	AA	AC	CC	AA	AC	AC	AC	AC	AC	AC	CC
CT	CT	CT	CT	CC	CT	TT	CC	CT	CT	CT	CT	CT	CT	TT
AG	AG	AG	AG	GG	AG	AA	AG	AG	AG	AG	AG	AG	AG	AA
CG	CG	--	CC	GG	CG	CC	CG	CG	CG	CG	CG	CG	CG	CC
GT	GT	GT	GT	GG	GT	TT	GT	GT	--	GT	GT	GT	GT	TT
CT	CT	CT	CT	CC	CT	TT	CT	CT	CT	CT	CT	CT	CT	TT
TT	TT	TT	TT	TT	CT	CT	TT	CT	TT	TT	CT	CT	TT	CT
GG	GG	GG	GG	AG	AG	GG	GG	AG	GG	GG	AG	GG	GG	GG
GG	CG	CG	CG	CC	CG	GG	CG	CG	CG	CG	CG	GG	CG	GG
AT	AT	AT	AT	AA	AA	AT	AT	--	AT	AT	AA	AT	AA	AT
AG	AA	AA	AA	AA	AG	AG	AA	AG	AA	AG	AG	AG	AA	AG
AC	CC	CC	CC	CC	AC	AC	CC	AC	CC	AC	AC	AC	CC	AC

CT	TT	TT	TT	TT	CT	CT	TT	CT	TT	CT	CT	CT	TT	CT
CT	TT	CT	CT	TT	CT	CT	CT	TT	CT	CT	TT	CT	TT	CT
AT	GG	AG	AG	GG	AT	AT	AG	GT	GG	AT	GT	AT	GG	AT
AT	AA	AA	AA	AA	AT	AT	AA	AT	AA	AT	AT	AT	AA	AT
AA	GG	AG	AG	GG	AG	AA	AG	AG	GG	AA	AG	AA	GG	AA
AA	CC	--	CC	AC	AC	--	AC	AC	--	AA	AC	AA	CC	AA
AG	AA	AA	AA	AA	AG	AG	AA	AG	AA	AG	AG	AG	AA	AG
AA	GG	--	GG	--	AG	AA	--	AG	--	AG	AG	AA	GG	AA
GG	AA	--	AA	AG	AG	GG	AG	AG	AG	AG	AG	GG	AA	GG
AA	GG	AG	GG	--	AG	AA	AG	AG	AG	AG	AG	AA	AG	AA
TT	GG	--	GG	GT	GT	TT	GT	GT	GT	GT	GT	TT	GT	TT
AG	GG	GG	GG	AG	GG	AG	AG	GG	AG	GG	GG	AG	AG	AG
TT	CC	--	CC	CT	CT	CT	CT	CT	CT	CT	--	TT	CT	TT
GG	CC	CC	CC	CG	CG	CG	CG	CG	CG	CG	CG	GG	CG	GG
CT	TT	TT	TT	TT	CT	CT	TT	CT	TT	CT	CT	CT	TT	CT
CT	TT	TT	TT	CT	TT	TT	CT	TT	CT	TT	TT	CT	CT	CT
CT	CT	CC	CC	CT	CC	CC	CC	CC	CC	CC	CC	CC	CT	CC
GT	GG	GG	GT	GT	GT	GG	GT	GT	GT	GG	GG	GT	GG	GT
AA	AG	GG	AG	AA	AG	GG	AG	AG	AG	AG	AG	AG	AG	AA
GG	AG	AA	AG	GG	AG	AA	--	AG	GG	AG	AG	AG	AG	GG
AG	AG	AA	AA	AG	AA	AA	AA	AA	AA	AA	AG	AA	AG	AG
GT	TT	GG	GT	--	GT	GG	GT	GT	GT	GG	GT	GT	GT	TT
AA	AG	--	AA	--	AA	AA	--	AA	AA	AA	AG	AA	AG	AG
CC	CT	--	CC	CT	CC	CC	CT	CT	--	CC	CT	CC	CT	CT
AG	AA	AG	AG	--	AG	AG	--	AA	AG	AG	--	AG	AA	AA
AG	AA	AG	AG	AG	AG	--	AG	AG	GG	GG	AA	AG	AA	AG
AG	AA	AG	AG	AG	GG	GG	AG	AG	GG	GG	AA	AG	AA	AG
GG	CC	CG	CG	GG	GG	GG	CG	CG	GG	GG	CC	CG	CC	CG
GG	CC	CG	CG	GG	GG	GG	CG	CG	--	GG	--	CG	CC	CG
GG	AA	AG	AG	GG	GG	GG	AG	AG	GG	GG	AA	AG	AA	AG
GG	AG	AG	GG	GG	GG	GG	GG	GG	GG	GG	AG	AG	AG	GG
TT	CC	--	CT	TT	CT	CT	CT	CT	TT	TT	CC	--	CC	CT
AC	CC	AC	CC	CC	AC	CC	CC	CC	CC	CC	AC	AC	AC	CC
CG	CC	CG	CC	CC	CG	CC	CC	CC	CC	CC	CG	CG	CG	CC
AC	CC	AC	CC	CC	AC	AC	CC	CC	CC	CC	AC	AC	AC	CC
AC	AC	--	CC	--	AC	CC	--	AC	AA	AA	CC	AC	AC	AC
AA	AG	AA	AG	AA	AA	AG	AG	AG	AA	AA	AG	AA	AG	AG
AG	AG	AG	GG	AA	AG	GG	GG	AG	AG	AG	GG	AG	AG	AG
GG	AG	GG	AG	GG	GG	AG	AG	AG	GG	GG	AG	GG	GG	AG
CT	CT	--	TT	CC	CT	TT	TT	CT	CT	CT	CT	CT	CT	CT
CT	CT	CT	TT	CC	CT	TT	TT	CT	CT	CT	CT	CT	CT	TT
AG	AA	--	AG	AA	AG	AG	AG	AA	AG	AG	AG	AG	AG	AG
CT	CC	--	CT	CT	CT	CT	CT	CC	CT	CT	CT	CT	CT	CT
AA	AA	AA	GG	--	GG	GG	AA	AG	AG	GG	GG	AG	AG	AA
AC	AC	--	CC	AC	CC	CC	AC	AC	AC	CC	CC	AC	AC	AC
CT	CT	CT	CC	CC	CC	CC	CT	CC	CC	CC	CC	CC	CC	CT
GG	GG	GG	AG	AG	AA	AA	GG	AG	--	AA	AA	AG	AG	GG

AG	AG	AG	AA	AA	AA	AA	AG	AA	AA	AA	AA	AA	AA	AG
GG	GG	GG	AG	AG	AA	AA	AG	AG	AA	AA	AG	AA	AG	GG
AA	AA	AA	CC	AC	CC	CC	AC	AC	CC	CC	AC	CC	AC	AA
AA	AA	AA	GG	AG	GG	GG	AG	AG	GG	GG	AA	GG	AG	AA
GG	GG	--	TT	GT	TT	TT	GT	GT	--	TT	GT	TT	GT	GG
AG	AG	AG	AA	AG	AA	AA	AA	AG	AA	AA	AG	AA	AG	AA
CT	CC	--	TT	--	TT	TT	TT	CT	TT	TT	CC	CT	CT	CT
CT	CT	CT	CC	CT	CC	CC	CC	CT	CC	CC	CT	CC	CT	CC
AG	AA	AA	GG	AG	GG	GG	AG	AG	GG	AG	AA	AG	AG	AG
GG	CG	--	GG	--	GG	GG	GG	GG	GG	CG	CG	CG	GG	CG
TT	TT	--	TT	--	TT	TT	--	TT	TT	CT	--	CT	TT	TT
AG	AA	--	AA	AA	AG	AG	AG	AG	AA	GG	GG	AG	AA	AA
AG	AA	GG	AA	AA	GG	AG	AG	AG	AA	GG	GG	AG	AA	AA
CG	CC	GG	CC	CC	GG	CG	CG	CC	CC	GG	--	CG	CC	CC
AG	AA	GG	AA	AA	GG	AG	--	AA	AA	GG	--	AG	AG	AA
AG	AA	--	AG	GG	GG	AG	AA	AG	AG	AG	--	GG	AG	GG
CT	CC	CT	CC	TT	TT	CT	CC	CT	CT	CT	CT	TT	CT	TT
AG	AA	AG	AA	GG	GG	AG	AA	AG	AG	AG	--	GG	AG	GG
CG	GG	CG	GG	CC	CC	CG	GG	CG	CG	CG	CG	CC	CG	CC
AC	AA	AC	AA	CC	CC	AC	AA	AC	AC	AC	AC	CC	AC	CC
AG	GG	--	GG	AA	AA	AG	--	AG	AG	AG	AG	AA	AG	AA
GT	TT	GT	--	GG	GG	GT	TT	GT	GT	GT	GT	GG	GT	GG
AG	AA	--	AA	GG	GG	AG	AA	AG	AG	AG	AG	GG	AG	GG
AG	AA	AG	AA	GG	GG	AG	AA	AG	AG	AG	AG	GG	AG	GG
AG	GG	AG	--	AA	--	AG	GG	AG	AG	AG	AG	AA	AG	AA
AC	CC	AC	CC	AC	AA	AC	CC	AC	AC	AC	AC	AA	AC	AA
AG	GG	AG	GG	AA	AA	AG	GG	AG	AG	AG	AG	AA	AG	AA
AG	GG	AG	GG	AA	AA	AG	GG	AG	AG	AG	AG	AA	AG	AA
CT	CT	CT	CC	TT	TT	CT	CC	CT	CT	CT	CT	TT	CT	TT
CG	CG	CG	CC	GG	GG	CG	CC	CG	CG	CG	CG	GG	CG	GG
AC	--	AC	CC	AA	AA	AC	CC	AC	AC	AC	AC	AA	AC	AA
CT	CT	CT	CC	TT	TT	CT	CC	CT	CT	CT	CT	TT	CT	TT
GT	GT	--	GG	TT	TT	GT	GG	GT	GT	GT	GT	TT	GT	TT
CT	CT	CT	TT	--	CC	CT	TT	CT	CT	CT	CT	CC	CT	CC
CG	CG	CG	GG	CC	CC	CG	GG	CG	CG	CG	CG	CC	CG	CC
AT	AT	--	AA	TT	TT	AT	AA	AT	AT	AT	AT	TT	AT	TT
CT	CT	CT	TT	CC	CC	CT	TT	CT	CT	CT	CT	CC	CT	--
AG	AG	AG	AA	GG	GG	AG	--	AG	AG	AG	AG	GG	AG	GG
AG	AG	--	GG	AA	AA	AG	GG	AG	AG	AG	AG	AA	AG	AA
AT	AT	AT	TT	AA	AA	AT	TT	AT	AT	AT	AT	AA	AT	AA
AG	AG	AG	AA	GG	GG	AG	AA	AG	AG	AG	AG	GG	AG	GG
AG	AG	AG	GG	AG	--	AG	GG	AG	AG	AG	AG	AA	AG	AA
CG	CG	--	GG	CC	CC	CG	GG	CG	CG	CG	CG	CC	CG	CC
AG	AG	AG	GG	AA	AA	AG	GG	AG	AG	AG	AG	AA	AG	AA
GG	CG	--	CC	GG	GG	CG	CC	CG	CG	CG	CG	GG	CG	GG
GG	AG	AG	AA	GG	GG	AG	AA	AG	AG	AG	AG	GG	AG	GG
AA	AC	AC	CC	AA	AA	AC	CC	AC	AC	AC	AC	AA	AC	AA

GG	AG	--	AA	GG	GG	AG	AA	AG	AG	AG	AG	GG	AG	GG
CC	CG	CG	GG	CC	CC	CG	GG	CG	CG	CG	CG	CC	CG	CC
CC	CT	CT	TT	CC	CC	CT	TT	CT	CT	CT	CT	CC	CT	CC
TT	AT	--	AA	TT	TT	AT	AA	AT	AT	AT	AT	TT	AT	TT
CC	CT	--	TT	--	CC	CT	TT	CT	CT	CT	--	CC	CT	CC
AA	AT	--	TT	AA	AA	AT	TT	AT	AT	AT	AT	AA	AT	AA
TT	AT	AT	AA	TT	TT	AT	AA	AT	AT	AT	AT	TT	AT	AT
GG	CG	CG	CC	GG	GG	CG	CC	CG	CG	CG	CG	GG	GG	CG
CC	CT	CT	TT	CC	CC	CT	TT	CT	CT	CT	CT	CC	CC	CT
AA	AG	AG	GG	AA	AA	AA	GG	AG	AG	AG	AG	AA	AA	AG
AA	AC	AC	CC	AA	AA	AA	CC	AC	AC	AC	AC	AA	AA	AC
TT	GT	GT	GG	TT	TT	TT	GG	GT	GT	GT	GT	TT	TT	GT
CC	CT	CT	TT	CC	CC	CC	TT	CT	CT	CT	CT	CC	CC	CT
CG	CG	CG	CC	GG	GG	GG	CC	CG	CG	CG	CG	GG	GG	CG
GT	GT	GT	GG	TT	TT	TT	GG	GT	GT	GT	GT	TT	TT	GT
CT	CT	TT	CC	TT	TT	TT	CC	CT	CT	CT	CT	TT	TT	CT
AG	AG	--	GG	AA	AA	AA	GG	AG	AG	AG	AG	AA	AA	AG
AC	AC	--	CC	AA	AA	AA	CC	AC	AC	AC	AC	AA	AA	AC
CT	CT	CC	TT	CC	CC	CC	CT	CT	CT	CT	CT	CC	CC	CT
AC	AC	AA	CC	AA	AA	AA	CC	AC	AC	AC	AC	AA	AA	AC
AG	AG	AA	GG	AA	AA	AA	GG	AG	AG	AG	AG	AA	AA	AG
GT	GT	--	TT	GG	GG	GG	TT	GT	GT	GT	GT	GG	GG	GT
GT	GT	GG	TT	GG	GG	GG	TT	GT	GT	GT	GT	GG	GG	GT
AG	AG	GG	AA	GG	GG	GG	AA	AG	AG	AG	AG	GG	GG	AG
CT	CT	CC	TT	CC	CC	CC	TT	CT	CT	CT	CT	CC	CC	CT
CT	CT	--	TT	--	CC	CC	TT	CT	CT	CT	CT	CC	CC	CT
AG	AG	--	GG	AG	AA	AA	GG	AG	AG	AG	AG	AA	AA	AG
CT	CT	CC	TT	CC	CC	--	TT	CT	CT	CT	CT	CC	CC	CT
CT	CT	TT	CC	TT	TT	TT	CC	CT	CT	CT	CT	TT	TT	CT
AG	AG	GG	AA	GG	GG	GG	AA	AG	AG	AG	AG	AG	GG	AG
CT	CT	CC	TT	CC	CC	CC	TT	CT	CT	CT	CT	CT	CC	CT
TT	TT	TT	--	TT	CT	TT	CT	CT	CT	CT	CT	CT	TT	CT
CT	CT	--	CT	CC	CT	CC	CT	CT	CT	CT	CT	CT	CC	CT
AG	AG	AA	AG	AA	AG	AG	AG	AG	AG	AA	AG	AG	AA	AG
AC	AC	AA	AC	AA	AC	CC	AC	AC	AC	AA	AC	AC	AA	AC
CC	CC	CG	CG	CG	CG	CC	CC	CG	CG	CG	CG	CG	CG	CG
GT	GT	--	GT	GG	GT	TT	GT	GT	GT	GG	--	GT	GG	GT
AG	GG	--	AG	GG	AG	AA	AG	AA	AG	GG	AG	AG	GG	AG
CT	CT	--	CT	TT	CT	CC	CT	CC	CT	TT	CT	CT	TT	CT
GG	AG	--	AG	AG	AG	GG	GG	GG	AG	AG	AG	AG	AG	AG
AG	AG	AG	AG	AG	AA	AA	--	AA	GG	GG	AG	AG	GG	AG
CG	CG	CG	GG	GG	CC	CC	--	--	GG	GG	--	CG	GG	CG
TT	CT	CT	CT	--	TT	TT	TT	TT	CT	TT	--	CT	CT	CT
GT	GT	GT	TT	--	GG	GG	GG	GG	TT	GT	GT	GT	TT	GT
GG	AG	AG	GG	--	AG	GG	GG	AG	GG	AG	AG	AG	AG	GG
AG	GG	AG	AG	AG	AG	AG	GG	AG	GG	AG	GG	AG	GG	AG
AC	AC	CC	AC	AC	CC	CC	AC	AC	AA	CC	--	CC	AC	AC

AT	AT	--	TT	--	AA	AT	AA	AT	TT	AA	AT	AA	AT	AT
AT	AT	--	TT	--	AA	AT	AA	AT	TT	AA	AT	AA	AA	AT
AT	AT	--	AT	--	TT	AT	TT	AT	AA	TT	AT	TT	TT	AT
CT	CT	CC	CT	CC	CC	CT	CC	CT	TT	CC	CT	CC	CC	CT
AG	AG	AA	AG	AA	AA	AG	AG	AG	GG	AA	AG	AA	AA	AG
AC	AC	AC	AC	AC	AC	AC	AC	AC	AA	CC	AC	CC	CC	AC
CT	CT	CT	CT	CT	CT	CT	CT	CT	TT	CC	--	--	CC	CT
AG	AG	AG	AG	AG	AG	AG	AG	GG	GG	AA	AG	AA	AA	AG
GT	GG	GT	GT	GT	GT	GT	GT	GG	GG	TT	GT	TT	TT	GT
AG	GG	AG	AG	AG	AG	AG	AG	GG	GG	AA	AG	AA	AA	AG
CC	CC	CG	CG	CG	CG	CC	CG	CC	CC	CG	CG	CG	CG	CC
GG	GG	AG	AG	AG	AG	GG	AG	GG	GG	AG	AG	AG	GG	GG
CG	CC	GG	CG	CG	CG	CG	CG	CG	CC	CG	GG	GG	CC	CG
AG	AA	GG	--	AG	AG	AG	AG	AG	AA	AG	AG	GG	AA	AG
GG	GG	AA	AG	--	AG	AG	AG	AG	GG	AG	AG	AA	GG	AG
AA	AA	GG	AG	AG	AG	AG	AG	AG	AG	AG	AG	GG	AA	AG
GG	GG	GT	GT	GG	GT	GG	GT	GG	GG	GT	GT	GT	GG	GG
GG	GG	AA	AG	AG	AG	AG	AG	AG	AA	AG	AG	AA	GG	AG
TT	TT	GT	--	GT	GT	TT	GT	TT	TT	GT	GT	GT	TT	TT
GG	GG	CC	CG	CC	CG	CG	CG	CG	CG	CG	CG	CC	GG	CG
TT	CT	--	CT	CT	CT	--	TT	TT	--	TT	--	CT	TT	CT
CG	CG	CG	CG	CC	CC	CC	GG	CG	--	GG	--	CC	GG	CG
CG	CC	CG	CC	CG	CG	CG	CC	CG	CG	CC	--	CG	CC	CC
AA	AC	AA	AC	AC	AC	AC	AA	AC	AC	AA	AA	AC	AA	AC
CC	CT	CC	CT	--	CT	CT	CC	CT	CT	CC	--	CT	CC	CT
AA	AG	--	AG	AG	AG	AG	AA	AG	AA	AA	--	AG	AA	AG
CC	CT	--	CT	CT	CT	CT	CC	CT	CC	CC	CC	CC	CC	CT
GG	GG	CG	CG	--	CG	CG	GG	CG	CG	GG	CG	GG	GG	GG
CC	CT	--	CT	CT	CT	CT	CC	CT	CC	CT	--	CC	CC	CT
AA	AG	AG	GG	GG	GG	GG	AA	GG	AG	AG	AG	AA	AA	AA
GG	GT	GG	GT	GT	GT	GT	GT	GT	GG	GT	GT	GG	GG	GG
TT	GT	--	GG	GG	GG	GG	GT	GG	GT	GT	GG	TT	GT	TT
GG	CG	--	GG	CG	CG	CG	CG	CG	GG	CG	CG	GG	CG	GG
GG	CG	--	CC	CC	CC	CC	CG	CC	CG	CG	CC	GG	CG	GG
GG	AG	AG	AG	AG	AG	AG	AG	AG	GG	AG	AG	GG	AG	GG
TT	CT	--	CC	CC	CC	CC	CT	CC	CT	CT	CC	TT	CT	TT
GG	GT	--	TT	TT	TT	GT	GT	TT	GT	GT	TT	GG	GT	GG
GG	AG	AG	AG	AG	AG	AG	AG	AG	GG	GG	AG	GG	AG	GG
AA	AG	--	GG	GG	GG	GG	AG	GG	AG	AA	--	AA	AG	AA
TT	CT	--	CC	CC	CC	CC	CT	CC	TT	TT	--	TT	CT	TT
TT	AT	AT	AT	AT	AT	AT	AT	AT	TT	TT	AT	TT	AT	TT
TT	GT	GT	GT	GT	GT	GT	GT	GT	TT	TT	GT	TT	GT	TT
CC	CT	--	TT	--	TT	TT	CT	TT	CC	CC	TT	CC	CT	CC
AA	AG	AG	AG	AG	AG	AG	AG	AG	AA	AA	AG	AA	AG	AA
TT	AT	--	AA	AA	AA	AA	AT	AA	TT	TT	AA	TT	AT	TT
AA	AG	AG	AG	AG	AG	AG	AG	AG	AA	AA	AG	AA	AG	AA
TT	CT	CT	CT	CT	CT	CT	CT	CT	TT	TT	CT	TT	CT	TT

TT	AT	AT	AT	AT	AT	AT	AT	AT	TT	TT	AT	TT	AT	TT
GG	GT	--	TT	TT	TT	TT	GT	TT	GG	GG	--	GG	GT	GG
AG	AG	GG	AG	AG	GG	GG	AG	GG	AA	AA	GG	AA	AG	AG
CT	CT	TT	CT	CT	CT	TT	CT	TT	--	CC	TT	CC	CT	CT
CT	CT	TT	CT	CC	CT	TT	CT	TT	CC	CC	TT	CC	CT	CT
CT	CT	CT	CT	CT	CT	TT	CT	TT	--	CC	--	CT	TT	TT
GG	GT	--	GT	TT	GT	GT	GT	TT	GG	GT	TT	GT	TT	TT
CC	CG	--	CG	--	CG	CG	CG	--	CC	CG	GG	CG	GG	GG
CC	CC	--	AC	AC	AC	AC	AC	AA	CC	AC	AA	AC	AA	AA
AA	AA	AA	AG	AG	AG	AG	AG	GG	AA	AG	GG	AG	GG	GG
GG	GG	--	AG	AA	AG	AG	GG	AA	GG	AG	--	AG	AA	AA
CT	CT	CT	CT	CT	CT	CT	TT	CT	TT	CT	CT	CT	CC	CT
AC	AC	AC	AC	AC	AC	AC	AA	AC	AA	AC	AC	AC	CC	AC
GT	GT	GT	GT	GT	TT	TT	TT	GT	TT	--	GT	TT	GT	GT
GG	GG	--	GG	GG	GG	CG	GG	GG	GG	GG	CG	CG	CG	GG
AG	AG	AG	AG	AG	GG	AG	GG	AG	GG	AG	AG	--	AG	AG
TT	TT	--	TT	TT	TT	CT	TT	TT	TT	TT	CT	CT	CT	TT
GT	GT	--	GT	GT	TT	TT	TT	GT	TT	GT	--	TT	TT	GT
CC	CC	CC	CC	CC	CC	CT	CC	CC	CC	CC	CT	CT	CT	CC
AC	AC	--	--	AA	AC	AA	AA	AC	AA	AC	AA	AC	AA	AC
AG	AG	AA	AG	AA	AG	AG	AA	AG	AA	AG	AG	GG	AG	AG
AG	AG	AA	AG	AA	AG	AA	AA	AG	AA	AG	AA	AG	AA	AG
GT	GT	--	GT	TT	GT	GT	--	GT	TT	GG	GT	GG	GT	GT
CT	CT	--	CT	CC	CT	CT	CC	CT	--	TT	CT	TT	CT	CT
AC	AC	AA	AC	AA	AC	AC	AA	AC	AA	CC	AC	CC	AC	AC
AT	AT	AT	AT	--	AT	AT	TT	AT	AT	AA	AA	AA	AT	TT
--	GG	GG	GG	GG	GG	--	GT	GG	GG	GG	GG	GG	GG	GT
AG	AG	AG	AG	AG	AG	AA	AG	AG	AG	AA	--	AA	AG	AG
CG	CG	CG	CG	CG	CG	CG	--	CG	CG	GG	--	GG	CG	CC
AG	AG	AG	AG	AG	AG	AG	--	AG	AG	GG	--	GG	AG	AA
CC	CG	--	CG	CG	CC	CC	CG	CC	CG	CC	GG	CG	GG	CG
AA	AG	GG	AG	AG	AA	AA	AG	AA	AG	AA	GG	AG	GG	AG
AA	AA	AG	AG	AG	--	AA	AA	--	AG	AA	AG	AA	AG	AA
AA	AA	AT	AA	--	AA	AA	AT	AA	--	AA	--	AT	AT	AT
AA	AA	AG	AA	AG	AA	AA	--	AA	AG	AA	AG	AA	AG	AA
AG	AG	--	AG	AA	AG	AG	AG	AG	AA	AG	AA	AG	AA	AG
AA	AA	CC	AA	CC	AA	AA	AC	AA	AC	AA	CC	AC	CC	AC
CC	CC	AC	CC	--	CC	CC	--	CC	--	CC	AC	AC	AC	AC
CC	CC	--	CC	AA	--	CC	CC	CC	AC	CC	AC	AC	AA	AC
GG	GG	--	GG	TT	GG	GG	GG	GT	GT	GT	TT	GT	GT	GG
AA	AA	--	AA	AT	AA	AA	AA	AT	AT	TT	AT	AT	AT	AA
AA	AA	AG	AA	AA	AA	AA	AA	AG	AA	AG	AG	AG	AA	AA
CC	CC	AC	CC	CC	CC	CC	CC	AC	CC	AC	AC	AC	CC	CC
AA	AA	TT	AA	AT	AT	AA	AA	AT	AT	TT	AT	AT	AT	AA
CC	CT	CT	CC	CT	CT	CT	CC	CT	CT	CT	CT	CT	CT	CC
GG	AA	AG	GG	AG	AA	AG	GG	AG	AG	AG	AG	AG	AA	AG
CC	TT	CT	CC	CT	TT	CT	CC	CT	CT	CT	CT	CT	TT	CT

TT	CC	CT	TT	CT	CC	CT	--	CT	CT	CT	CT	--	CC	CT
GG	AA	--	GG	AA	AA	AG	GG	AG	--	AG	AG	GG	AA	AG
CC	TT	CT	CC	TT	TT	CT	CC	CT	CT	CT	CT	CC	TT	CT
TT	CC	--	TT	CC	CC	CT	TT	CT	CT	CT	CT	TT	CC	CT
CC	TT	CT	CC	TT	TT	CT	CC	CT	CT	CT	CT	CC	TT	CT
GG	AA	--	GG	AA	AA	AG	--	AG	AG	AG	AG	GG	AA	AG
TT	CC	CT	TT	--	CC	CT	TT	CT	CT	CT	CT	TT	CC	CT
GG	AA	AG	GG	--	AA	AG	GG	AG	AG	AG	AG	GG	AA	AG
CC	GG	CG	CC	GG	GG	CG	CC	CG	CG	CG	CG	CC	GG	CG
AA	CC	AC	AA	CC	CC	AC	AA	AC	AC	AC	AC	AA	CC	AC
CC	TT	CT	CC	TT	TT	CT	CC	CT	CT	CT	CT	CC	TT	CT
CC	TT	--	CC	TT	TT	CT	CC	CT	CT	CT	CT	CC	TT	CT
TT	AA	AT	TT	AA	AA	AT	TT	AT	AT	AT	AT	TT	AA	AT
CC	--	CT	CC	TT	TT	CT	CC	CC	CT	--	CT	CC	TT	--
AA	CC	AC	AA	AC	CC	AC	AA	AC	AC	AC	AC	AA	CC	AC
TT	GG	GT	TT	GG	GG	GT	TT	GT	GT	GT	GT	TT	GG	GT
TT	CC	CT	TT	CC	CC	CT	TT	CT	CT	CT	CT	TT	CC	CT
GG	CC	CG	GG	CC	CC	CG	GG	CG	CG	CG	CG	CG	CC	CG
GG	CC	CG	GG	CC	CC	CG	GG	CG	CG	CG	CG	CG	CC	CG
CC	AA	AC	CC	AA	AA	AC	CC	AC	AC	AC	AC	AC	AA	AC
GG	CC	CG	CG	CC	CC	CG	GG	CG	GG	CG	CG	CG	CC	CG
AT	AT	AT	AT	AT	AT	TT	--	TT	AA	AT	AT	AT	TT	AT
GG	AG	AG	AG	--	AG	GG	GG	GG	AA	AA	--	AG	GG	AG
GG	GG	AG	--	AG	AA	--	--	GG	AA	AA	AG	AG	GG	AG
AA	AA	AG	GG	GG	GG	AG	GG	--	--	GG	AG	AG	AA	GG
GG	GG	AG	AA	AA	AA	AG	AA	AA	AA	AA	AG	AA	AG	AA
CC	CC	AC	AA	AA	AA	AC	AA	AA	AA	AA	AC	AA	AC	AA
GG	GG	--	AA	AA	AA	AG	AA	AA	AA	AA	AG	AA	AG	AA
GG	GG	AG	AA	AA	AA	AG	AA	AA	AA	AA	AG	AA	AG	AA
TT	TT	--	GG	GG	GG	GT	GG	GG	GG	GG	GT	GG	GT	GG
GG	GG	--	TT	TT	TT	GT	TT	TT	TT	TT	GT	TT	GT	TT
AA	AA	--	CC	CC	CC	AC	CC	CC	CC	CC	AC	CC	AC	CC
CC	CC	CT	TT	TT	TT	CT	TT	TT	TT	TT	CT	TT	CT	TT
CC	CC	--	--	TT	TT	CT	TT	TT	--	TT	--	TT	CT	TT
TT	TT	--	GG	GG	GG	GT	--	GG	GG	GG	GT	GG	GT	GG
TT	TT	--	AA	AA	AA	AT	AA	AA	AA	AA	AT	AA	AT	AA
CC	CC	--	GG	GG	GG	CG	GG	GG	GG	GG	CG	GG	CG	GG
CC	CC	AC	AA	AA	AA	AC	AA	AA	AA	AA	AC	AA	AC	AA
TT	TT	CT	CT	--	CC	CT	CC	CC	CC	CC	CT	CC	CT	CC
CC	CC	CG	GG	GG	GG	CG	GG	GG	GG	GG	CG	GG	CG	GG
TT	TT	CT	CC	CC	CC	CT	CC	CC	CC	CC	CT	--	CT	CC
TT	TT	AT	AA	AA	AA	AT	AA	AA	AA	AA	AT	AA	AT	AA
CC	CC	CT	TT	TT	TT	CT	TT	TT	TT	TT	CT	TT	CT	TT
AA	AA	AG	GG	GG	GG	AG	GG	GG	GG	GG	AG	GG	AG	GG
TT	TT	AT	AA	AA	AA	--	--	AA	AA	AA	--	AA	AT	AT
TT	TT	--	AA	AA	AA	AT	--	AA	AA	AA	AT	AA	AT	AA
CC	CC	AC	AA	AA	AA	AA	AA	AA	AA	AA	AC	AA	AC	AA

GG	GG	--	TT	TT	TT	TT	TT	TT	TT	TT	GT	TT	GT	GT
AA	AA	--	GG	GG	GG	GG	--	GG	--	GG	--	GG	AG	AG
TT	TT	--	GG	GG	GG	GG	GG	GG	GG	GG	TT	GG	GT	GT
GG	GG	AG	AA	AA	AA	AA	AA	AA	AA	AA	GG	AA	AG	AG
CC	CC	CT	TT	TT	CT	TT	TT	TT	--	TT	--	TT	CT	CT
GG	GG	AG	AA	AA	AG	AA	AA	AA	AA	AA	GG	AA	AG	AG
CC	CC	CT	TT	TT	CT	TT	CT	TT	TT	TT	--	TT	CT	CT
AA	AA	AC	CC	CC	AC	CC	AC	--	CC	CC	AA	--	AC	AC
GG	GG	--	AA	AA	AG	AA	AG	AA	AA	AA	--	AA	AG	AG
CC	CC	TT	TT	TT	CT	TT	CT	TT	TT	TT	CC	TT	CT	CT
AA	AA	GG	AG	GG	AG	GG	AG	GG	GG	AG	AA	GG	AG	AG
TT	TT	CT	CT	CC	TT	CT	--	CC	CC	CT	--	CC	TT	CT
GT	GT	GG	GG	GG	GT	GG	GG	GG	GG	GG	GT	GG	GT	GG
AG	AG	AG	AG	GG	GG	AG	GG	GG	GG	AG	AG	AG	AG	AG
AA	AT	AT	AT	TT	AT	AT	TT	AT	TT	AT	--	AT	AA	AT
AA	AT	AT	TT	TT	AT	AT	TT	AT	TT	AT	AA	AT	AA	AT
AA	AC	AC	CC	CC	AC	AC	CC	AC	AC	AC	AA	AC	AA	AC
TT	GT	GT	GG	GG	GT	GT	--	GT	GT	GT	TT	GT	TT	GT
CT	TT	CT	TT	CT	TT	CT	TT	TT	TT	CT	CT	TT	TT	TT
AA	AG	--	GG	--	GG	AG	--	AG	AG	AA	AG	AG	GG	GG
GG	GG	CG	GG	GG	CG	CG	CG	GG	GG	GG	CG	GG	CG	CG
TT	TT	CT	TT	TT	CT	CT	CT	TT	TT	TT	CT	TT	CT	CT
GG	GT	GT	GT	GG	GT	GG	GT	GG	GT	GG	GG	GT	GT	GT
AG	AG	--	AG	AA	GG	AG	--	AA	AG	AA	AG	--	GG	GG
AA	AA	AC	AA	AC	AC	AC	--	AA	AA	AA	AC	AA	AA	AC
GG	GG	CG	GG	CG	CG	CG	--	GG	--	GG	CG	GG	GG	CG
TT	TT	GT	TT	GT	GT	GT	TT	TT	TT	TT	GT	TT	TT	GT
CT	CT	TT	CT	TT	TT	CT	CT	CC	CC	CC	CT	CT	CT	CT
GG	GG	AG	GG	AG	AG	AG	AG	AG	AG	AA	AA	GG	GG	GG
CC	CC	CT	CC	CT	CT	CC	CC	CT	--	CT	CT	--	CC	CC
CG	CC	--	CG	CC	CC	CG	CG	CC	CG	CC	--	CC	CG	CG
AA	AA	AA	AA	AA	AA	AC	AC	AC	AC	AC	AC	AC	AC	AA
AG	AA	AA	AG	AA	AA	GG	GG	AG	GG	AG	AG	AG	GG	AG
CT	CT	CT	CT	CT	CT	TT	TT	TT	TT	TT	--	TT	TT	CT
GG	CG	CG	GG	CG	CG	GG	GG	CG	GG	CG	CG	CG	GG	GG
AG	GG	--	AG	GG	GG	AA	AA	AG	AA	AG	AG	AG	AA	AG
CG	GG	--	CG	GG	GG	CG	CG	GG	CG	GG	--	GG	CG	CG
CG	GG	--	CG	GG	GG	CC	CC	CC	CC	CG	CG	CG	CC	CG
CT	TT	--	CT	TT	TT	CC	CC	CT	CC	CT	CT	CT	CC	CT
AG	AA	--	AG	AA	AA	GG	GG	AG	GG	AG	AG	AG	GG	AG
GG	GG	GG	GG	GG	GG	AG	AG	AG	AG	AG	AG	AG	AG	GG
CT	TT	--	CT	TT	TT	CC	CC	CT	CC	CT	CT	CT	CC	CT
AG	AG	AG	AG	AG	AG	GG	GG	GG	GG	GG	GG	GG	GG	AG
AG	AA	AA	AG	AA	AA	GG	GG	AG	GG	AG	AG	AG	GG	AG
AC	AA	AA	AC	AA	AA	CC	CC	AC	CC	AC	--	AC	CC	AC
TT	AT	AT	TT	AT	AT	TT	TT	AT	TT	AT	AT	AT	TT	TT
AG	GG	--	AG	GG	GG	AA	AA	AG	--	AG	--	AG	AA	AG

AC	CC	AC	AC	CC	CC	AA	AA	AC	AA	AC	AC	AC	AA	AC
AG	GG	--	AG	GG	AG	AA	AA	AG	AA	AG	AG	AG	AA	AG
AG	AG	AG	AG	AG	AG	AA	AA	AA	AA	AA	--	AA	AA	AG
CC	CT	--	CC	CT	CC	CC	CC	CT	CC	CT	CT	CT	CC	CC
CC	CG	CC	CC	CG	CC	CC	CC	CG	CC	CG	CG	CG	CC	CC
TT	CT	--	TT	TT	TT	CT	--	CT	TT	CT	CT	CT	TT	TT
CG	GG	--	CG	GG	CG	GG	CG	GG	CG	GG	--	GG	GG	CG
CC	CT	--	CC	CT	CC	CT	CC	CT	CC	CT	CT	CT	CT	CC
GT	GG	--	GT	GG	GG	GG	GT	GG	GT	GG	--	GG	GG	GT

5-24	5-25	5-28	5-30	5-32	5-35	5-38	5-39	5-40	5-41	5-43	5-44	5-45
AA	AA	AC	AC	AC	CC	AA	AC	AC	AC	AC	AC	AC
AA	AA	AG	AG	AG	GG	AA	AG	AG	AG	AG	AG	AG
CT	TT	CT	CT	CT	CC	TT	CT	CT	CT	CT	CT	CT
AG	GG	GG	AG	AG	AG	--	GG	AG	AG	AG	AG	AG
GG	AA	AG	AG	AG	--	--	AG	AG	AG	--	AG	AG
GG	CG	GG	CG	CG	CG	CG	GG	--	CG	CG	CG	CG
CC	CT	CT	CT	CT	CT	--	CT	CT	TT	--	CT	CT
GG	GT	GT	GT	GT	GT	TT	GT	GT	TT	TT	GT	GT
GG	GT	GT	GT	GT	GT	TT	GT	GT	TT	GT	GG	GT
GG	GT	GT	GT	GT	GT	TT	GT	GT	GT	GT	GG	GT
GG	AA	AG	AG	AG	AG	--	AG	AG	AG	AG	--	AG
CC	CT	CC	CT	CT	CT	CT	CC	--	CC	CC	--	CT
CC	AA	CC	AC	AC	AC	AA	AC	AA	AC	AC	--	AC
AA	AG	AA	AA	AG	AG	AG	AA	AG	AA	AA	AA	AG
CC	CT	CC	CC	CC	CC	CT	CT	--	CT	CT	CC	CC
AA	GG	AA	AA	AG	AG	GG	AG	GG	AG	AG	--	AG
TT	AT	TT	TT	TT	AT	AT	TT	AT	TT	TT	--	TT
CC	--	CC	CC	CC	CC	CT	CT	CT	CT	CT	--	CC
TT	GT	GT	TT	TT	GT	GT	TT	TT	GT	TT	TT	TT
CC	CG	CG	CC	CC	CG	CC	CC	CC	CG	CC	CG	CC
TT	--	AT	TT	TT	AT	AT	AT	AT	AA	AT	--	TT
CT	CT	CT	TT	TT	CT	TT	CT	--	CC	CC	CT	TT
AT	AA	AT	AA	AA	AT	AA	AT	AT	TT	--	AT	AA
AT	AA	AT	AA	AA	AT	AA	AT	AT	TT	TT	AT	AA
AG	AG	AG	AA	AA	AG	AA	AG	AG	GG	GG	AG	AA
GT	GT	GT	TT	TT	GT	TT	TT	TT	GT	GT	GT	TT
GT	GT	GT	TT	TT	GT	TT	GT	GT	GG	GG	GT	TT
AT	AT	AT	TT	TT	AA	TT	AT	AT	AA	AA	AT	TT
CT	--	CT	TT	TT	CT	--	CT	CT	CC	CC	CT	TT
CG	CC	CG	CC	CC	CG	CC	CG	CG	GG	GG	CG	CC
CT	CC	CT	CC	CC	CT	CC	CT	CT	TT	TT	--	CC
AC	AA	AC	AA	AA	AC	--	AC	AC	CC	CC	AC	--
AG	--	AG	AA	AA	AG	AA	AG	AG	GG	GG	AG	AA
GT	GG	GT	GG	GG	GT	GG	GT	GT	TT	TT	GT	GG
AG	AG	AG	AA	AA	AG	AA	AG	AG	GG	GG	--	AA
GT	TT	TT	TT	TT	GT	TT	GT	GT	GG	--	GT	--
AG	GG	AG	GG	GG	AG	GG	AG	AG	AA	AA	AG	GG
CT	CC	CT	CC	CC	CT	CC	CT	CT	TT	TT	CT	CC
AT	AA	AT	AA	AA	AT	AA	AT	AT	--	TT	AT	AA
AG	AA	AG	AA	AA	AG	AA	--	AG	GG	GG	--	AA
AC	AA	AC	AA	AA	AC	AA	AC	AC	CC	CC	AC	AA
AG	AA	AG	AA	AA	AG	--	AG	AG	GG	--	AG	AA

CT	TT	CT	TT	TT	CT	TT	CT	CT	CC	CC	--	TT
CG	CC	CG	CC	CC	CG	--	CG	CG	GG	GG	GG	CC
CT	TT	CT	TT	TT	TT	CT	CT	CT	CC	--	CT	TT
CT	--	CT	CC	CC	CC	CT	CT	CT	CT	--	CT	CC
AC	AA	AC	AA	AA	AA	AC	AC	CC	AC	CC	AC	AA
CC	CT	CT	CC	CC	CC	--	CT	TT	CT	TT	CT	CC
TT	CT	CT	TT	TT	TT	--	CT	CC	TT	CT	CT	TT
GG	AA	GG	AA	AA	AG	AG	--	AG	--	AA	--	AA
GT	TT	GG	TT	TT	GT	GT	TT	GT	GT	GT	TT	TT
AG	AA	GG	AA	AA	AG	AA	AA	AG	AG	--	AG	AA
CT	CC	TT	CC	CC	CT	CC	CC	CT	CT	CT	CT	--
CG	GG	CC	GG	GG	CG	GG	GG	CG	CG	CG	CG	GG
AG	GG	AA	GG	GG	AG	GG	GG	AG	AG	AG	--	GG
AC	AA	CC	AA	AA	AA	--	AA	AC	AC	AA	--	AA
AG	GG	AG	GG	GG	GG	GG	GG	GG	GG	GG	GG	GG
TT	CC	CT	CC	CC	CC	CT	CC	CT	CT	CC	--	CT
CC	TT	CT	TT	TT	TT	CT	TT	CT	--	TT	CT	CT
TT	CC	CT	CC	CC	CC	CT	CC	CC	CT	CC	CT	CT
AA	GG	AG	GG	GG	GG	AA	GG	GG	AG	GG	AG	AG
AA	AC	AC	CC	CC	CC	AA	CC	CC	AC	CC	AC	AC
AA	AA	AG	GG	GG	GG	AA	GG	GG	AG	GG	AG	AG
CG	GG	CG	CC	CC	CC	GG	CC	CC	CG	CC	CG	CG
CG	CC	CG	GG	GG	GG	CC	GG	GG	CG	GG	CG	CG
AG	AA	AG	GG	GG	GG	AA	GG	GG	AG	--	AG	AG
AC	CC	AC	AA	AA	--	CC	AC	AC	AC	AA	AC	AC
CT	TT	CT	CC	CC	CC	TT	CT	CT	CT	CC	CT	CT
CT	CC	CT	CT	TT	TT	CC	CT	CT	CT	--	CT	CT
AT	TT	AT	AT	AA	AT	TT	AT	TT	AT	AT	--	AT
GG	--	GG	GG	GG	GG	GG	AG	GG	AG	--	--	AG
AT	TT	AT	AT	TT	AT	TT	TT	TT	TT	TT	AT	TT
GT	GG	GT	TT	GT	GT	--	TT	GT	GG	TT	GT	TT
GG	AG	GG	GG	GG	AG	AG	GG	GG	AG	--	AG	GG
TT	CT	TT	TT	TT	CT	CT	TT	TT	CT	TT	CT	TT
GG	AG	GG	GG	GG	AG	AG	GG	GG	AG	GG	AG	GG
GT	TT	GT	GG	GT	GT	TT	GG	GT	TT	--	GT	GG
GG	GT	GG	GG	GG	GT	GT	GG	--	GT	GG	GT	GG
CT	CC	CT	TT	CT	CT	CC	TT	CT	CC	TT	CT	TT
CC	CT	CT	TT	TT	CT	--	TT	CC	CC	CC	--	TT
AG	AG	AG	AA	AA	AG	GG	AA	GG	AG	AG	AA	AA
CG	CG	CG	GG	GG	CG	CC	GG	CC	CG	CG	--	GG
AG	AA	AG	AA	AA	AG	GG	AA	GG	AG	--	AA	AA
CT	CC	CT	CC	CC	CT	--	CT	TT	CT	CT	--	CC
CC	CC	CT	CC	CC	CC	TT	CT	TT	CT	CT	--	CC
CC	CC	CC	CC	CC	CC	CG	CG	CG	CC	CG	CC	CC
TT	TT	TT	TT	TT	TT	AT	AT	AT	--	AT	--	TT
GG	GG	GT	GG	GG	GG	GT	GT	GT	GT	GG	GG	GG
CC	CC	CT	CC	CC	CC	CT	CC	CT	CT	CC	CC	CC

CC	CC	CT	CC	CC	CC	TT	CT	CT	CT	CT	--	CC
GG	GG	GT	GG	GG	GG	GT	GG	GT	GT	GG	--	GG
CC	CC	CT	CC	CT	CC	TT	CT	CT	CT	--	CC	CC
TT	TT	GT	TT	TT	TT	GT	TT	GT	GT	TT	TT	TT
GG	--	CG	GG	GG	GG	CG	GG	CG	CG	GG	--	GG
AG	AG	GG	AG	AG	GG	AG	AA	AG	AG	--	--	AA
AG	AG	AG	AG	AG	AG	--	GG	AG	AG	AG	GG	GG
AG	AG	AG	AG	AG	AG	AG	AG	AG	AG	AG	GG	GG
CT	TT	CT	CT	CT	CT	CT	CT	CT	CT	CT	CT	CC
AG	GG	AG	AG	AG	AG	AG	AG	AG	AG	AG	AG	AA
GT	GG	GT	GT	GT	GT	GT	GT	GG	GT	GT	--	GT
AG	AG	AG	AG	AG	GG	GG	AG	AG	AG	GG	--	AG
CC	TT	CT	CT	CT	CT	--	TT	TT	TT	TT	CT	CT
AC	CC	AC	AC	AC	CC	--	CC	CC	CC	CC	AC	AC
CC	CT	CC	CC	CC	CT	CT	CT	CT	CT	CT	CC	CC
GT	GG	GT	GT	GT	GT	GT	GG	--	GG	GG	--	--
CC	CC	CC	CC	CC	CT	CT	CC	CC	CC	CC	CC	CC
CG	GG	CG	CG	CG	GG	GG	GG	GG	GG	GG	CG	CG
AC	--	AC	AC	AC	CC	--	CC	CC	CC	--	AC	AC
TT	--	TT	TT	TT	CT	CT	TT	TT	TT	TT	--	TT
AA	AA	AA	AA	AA	AG	AG	AA	AA	AA	AA	--	AA
AG	AA	AG	AG	AG	AG	AG	AA	AA	AA	AA	AG	AG
CC	CT	CC	CC	CC	CT	CT	CT	CT	CT	CT	CC	CC
AT	AA	AT	AT	AT	AA	--	AA	AA	AA	AA	--	AT
CT	CC	CT	CT	CT	CC	CC	CC	CC	CC	CC	--	CT
AG	AA	AG	AG	AG	AA	AA	AA	AA	AA	AA	AA	AG
CC	CC	CC	CC	CC	CT	CT	CC	CC	CC	CC	--	CC
AT	TT	AT	AT	AT	AT	AT	TT	TT	TT	TT	AT	AT
AT	TT	AT	AT	AT	AT	AT	TT	--	--	TT	AT	AT
CT	TT	CT	CT	CT	CT	CT	TT	TT	TT	TT	CT	CT
CT	CC	CT	CT	CT	CC	CC	CC	CC	CC	CC	CT	CT
GG	GG	GG	GG	GG	GT	GT	GG	GG	--	GT	--	GG
CC	CC	CC	CC	CC	CT	CT	CC	CC	CC	--	CC	CC
AG	AG	AG	AG	AG	AA	AA	AG	AG	AG	AA	AG	AG
AG	GG	AG	AG	AG	AG	AG	GG	GG	GG	--	AG	AG
AA	AA	AA	AA	AA	AG	AG	AA	AA	AA	AG	AA	AA
CT	CC	CT	CT	CT	CT	CT	CC	CC	CC	CT	CT	CT
CC	--	CC	CC	CC	AC	AC	CC	--	CC	--	--	CC
AT	TT	AT	AT	--	AT	AT	TT	TT	--	AT	AT	AT
AG	AA	AG	AG	AG	AA	AA	--	AA	AA	--	AG	AG
AA	AA	AA	AA	AA	AC	AC	AA	AA	AA	AC	AA	AA
TT	TT	TT	TT	TT	AT	AT	TT	TT	TT	AT	TT	TT
CC	AC	CC	CC	CC	AA	AA	AC	AC	AC	--	CC	CC
AC	CC	AC	AC	AC	CC	--	CC	CC	CC	CC	AC	AC
AG	AG	AG	AG	AG	GG	GG	AG	AG	--	GG	--	AG
CC	AC	CC	CC	CC	AC	AC	AC	AC	AC	AC	--	CC
AA	AA	AA	AA	AA	AG	AG	AA	AA	AA	AG	AA	AA

CG	CC	CG	CG	CG	CC	CC	CC	CC	CC	CC	CG	CG
CT	TT	CT	CT	CT	CT	CT	TT	TT	TT	CT	CT	CT
CT	CC	CT	CT	CT	CT	CT	--	CC	CC	CT	--	CT
CC	--	CC	CC	CC	CT	CT	CC	CC	CC	CT	--	CC
AA	--	AA	AA	AA	AG	--	AA	AA	AA	AG	--	AA
CT	CC	CT	CT	CT	CT	CT	CC	CC	CC	CT	CT	CT
TT	TT	TT	TT	TT	CT	CT	TT	TT	TT	CT	--	TT
AT	AA	AT	AT	AT	AT	AT	AA	AA	AA	AT	--	AT
AG	GG	AG	AG	AG	GG	GG	GG	GG	GG	GG	AG	AG
AA	AA	AA	AA	AA	AC	AC	AA	AA	AA	AC	--	AA
CC	CC	CC	CC	CC	CT	CT	CC	CC	CC	CT	CC	CC
GT	TT	GT	GT	GT	TT	TT	TT	TT	TT	TT	GT	GT
TT	--	TT	TT	TT	CT	--	TT	TT	TT	CT	--	TT
AC	AC	AC	AC	AC	CC	CC	AC	AC	AC	CC	AC	AC
AA	AA	AA	AA	AA	AG	AG	AA	AA	--	AG	--	AA
GG	GG	GG	GG	GG	GT	GT	GG	GG	GG	GT	GG	GG
AT	AT	AT	AT	AT	TT	TT	AT	AT	AT	TT	AT	AT
AA	AA	AA	AA	AA	AG	AG	AA	AA	AA	AG	AA	AA
CT	--	CT	CT	CT	CC	CC	CC	CC	CC	CC	--	CT
CC	--	CC	CC	CC	CT	CT	CC	CC	CC	CT	--	CC
AA	AA	AA	AA	AA	AC	AC	AA	--	AA	AC	--	AA
CC	CC	CC	CC	CC	CT	CT	CC	CC	CC	CT	CC	CC
AT	AA	AT	AT	AT	AT	AT	AA	AA	AA	AT	AT	AT
AG	GG	AG	AG	AG	AG	AG	GG	GG	GG	AG	AG	AG
CG	GG	CG	CG	CG	GG	GG	GG	GG	GG	GG	--	--
TT	--	TT	TT	TT	--	--	TT	TT	TT	CT	--	--
GG	GG	GG	GG	GG	GG	GG	GG	GG	GG	AG	AG	GG
AA	AG	AA	AA	AA	AA	AA	AA	AA	AA	AG	AG	AG
AG	AA	AG	AG	AG	AA	AA	AA	AA	AA	AA	AG	AG
GG	GG	AG	AG	AG	GG	GG	GG	GG	GG	GG	AG	AG
CT	CT	CT	CT	TT	TT	--	TT	CT	CT	TT	--	CT
GT	GT	TT	TT	GT	GG	GG	GG	GT	GT	GG	--	TT
TT	TT	GT	GT	GT	TT	TT	TT	TT	--	TT	GT	GT
AG	AG	AA	AA	AG	GG	GG	GG	AG	AG	GG	--	AA
CT	CT	CT	CT	CC	CC	CC	CC	CT	CT	CC	CT	CC
GT	GT	GT	GT	TT	TT	TT	GT	GT	GT	TT	GT	TT
GG	AG	GG	GG	GG	AG	AG	AG	AG	AG	AG	--	GG
AG	AA	AA	AA	GG	AG	AG	AA	AG	AG	AG	AA	AG
CT	CT	CT	CT	CC	CC	CC	CC	CC	CT	CC	CC	CC
AT	AA	AA	AT	AT	AA	AA	AA	AA	AT	AA	AA	AA
CG	CC	CC	CG	CG	CG	CG	CG	CG	CG	CG	--	CG
AA	AA	--	AA	AA	AC	AC	AC	AC	AA	AC	--	AC
AG	--	AA	AG	AG	AG	AG	AG	GG	AG	--	AG	AG
GG	AG	AG	GG	GG	AG	AG	AG	GG	GG	AG	AG	AG
CC	CT	CT	CC	CC	CT	CT	CT	CC	CT	CT	CT	CT
AA	AG	AG	AG	AG	AG	GG	AG	AG	GG	AG	AG	AG
CT	CT	CT	CC	CC	CT	CC	CT	CC	CC	CT	CT	CT

AG	GG	GG	AG	AG	GG	GG	GG	AG	GG	GG	GG	GG
AA	GG	GG	AG	AG	GG	GG	GG	AA	AG	GG	--	AG
CT	CT	CT	TT	CC	CT	CC	CT	CT	CC	CT	CT	CC
AT	AT	AT	TT	AA	AT	AA	AT	AT	AA	AT	AT	AA
AC	AC	CC	CC	AA	AC	AA	AC	AC	AA	AC	--	AA
CT	CT	CC	CC	TT	CT	--	CC	CT	TT	CT	CT	TT
AG	AG	AA	AA	GG	AG	--	AG	AG	GG	AG	AG	GG
CT	CC	TT	CT	CC	TT	CC	--	CT	CC	CT	CT	CC
TT	TT	CT	CT	TT	CT	TT	TT	TT	TT	CT	--	TT
AG	AA	GG	AG	AA	GG	AA	AG	AG	AA	AG	AG	AA
GG	AG	AG	AG	AA	GG	AA	AG	AG	AG	AG	AG	GG
TT	CT	CT	CT	CC	TT	CC	CT	CT	CT	CT	CT	TT
AG	AG	GG	AG	GG	AG	--	AG	--	AG	--	AG	AG
TT	--	CT	CT	--	TT	--	CT	CT	--	--	--	TT
CG	--	CG	GG	GG	CG	--	GG	CG	GG	CG	CG	CG
TT	CT	CT	CT	CC	TT	--	CT	CC	CC	CT	--	TT
CT	CT	CC	CT	CC	CT	CC	CC	CC	CC	--	CT	CT
AA	AG	AG	AG	GG	AG	GG	AG	GG	GG	AA	--	AA
TT	CT	CT	CT	CC	CT	CC	CT	CC	CC	TT	--	CT
GG	AG	AG	AG	AA	AG	AA	AG	AA	AA	GG	GG	AG
TT	CT	CT	CT	CC	CT	CC	CT	CT	CC	CT	TT	CT
AA	AG	AG	AA	GG	AG	GG	AG	AG	GG	AG	AG	AG
AG	AG	AG	GG	GG	AG	--	GG	AG	GG	--	GG	GG
GG	GT	GG	GT	TT	GT	TT	GT	GT	TT	GT	GT	GT
CG	CG	CG	CC	CC	CG	CC	CC	CG	CC	CG	CC	CC
CC	CG	CC	CC	CC	CG	CG	CC	CG	CG	CG	CC	CC
CT	CT	TT	CT	CT	CT	CC	CT	CT	CC	CT	CT	CT
CT	TT	CC	CT	CT	CT	TT	CT	TT	CT	CT	--	CT
AT	AA	TT	AT	AT	AT	--	AT	AA	AT	AT	--	AT
AG	GG	AA	AG	AG	AG	GG	AG	GG	AG	--	AG	AG
CT	--	TT	CT	CT	CT	CC	CT	CC	CT	CT	CT	TT
TT	TT	TT	TT	TT	CT	TT	TT	TT	CT	CT	TT	CT
AC	AA	AC	AC	AC	AC	--	AC	AA	AC	AC	AC	CC
GT	GG	TT	GG	GT	TT	GT	GT	GT	GG	GG	GT	GG
AG	GG	AA	GG	AG	AA	AG	AG	AG	GG	GG	AG	GG
CT	CC	TT	CC	CT	TT	CT	CT	CT	CC	CC	CT	CC
AT	TT	AA	TT	AT	AA	AT	AT	AT	TT	TT	--	TT
AC	AA	CC	AA	AC	CC	AC	AC	AC	AA	AA	AC	AA
CG	CC	CG	CC	CC	CG	CG	CG	CC	CC	--	--	CC
AG	AA	AG	AA	AA	AG	AG	AG	AA	AA	AA	--	AA
CT	TT	CC	TT	CT	CC	CT	CT	CT	TT	--	CT	TT
CT	TT	CC	TT	CT	CC	CT	CT	CT	TT	TT	--	TT
AG	--	AA	GG	AG	AA	AG	AG	AG	GG	GG	AG	GG
CC	CC	CT	CC	CT	CT	CC	CC	CT	CC	--	CT	CC
AG	--	AA	GG	AG	AA	--	AG	AG	GG	--	AG	GG
AG	--	GG	AA	AG	GG	AG	AG	AG	AA	AA	AG	AA
GT	TT	--	TT	GT	GG	--	GT	GT	TT	TT	--	TT

CT	CC	TT	CC	--	TT	CT	CT	CT	--	CC	CT	CC
CC	CC	GG	CC	CG	GG	CG	CG	CG	CC	CC	CG	CC
GG	GG	TT	GG	GT	TT	GT	GT	GT	GG	GG	GT	GG
GG	GG	AA	GG	AG	AA	AG	AG	AG	GG	GG	AG	GG
TT	TT	CC	TT	CT	CC	CT	CT	CT	TT	TT	CT	TT
TT	TT	GT	TT	GT	GT	TT	TT	GT	--	TT	GT	TT
CC	CC	GG	CC	CG	GG	CG	CG	CG	CC	CC	--	CC
GG	GG	AA	GG	AG	AA	AG	AG	AG	GG	GG	AG	GG
GG	--	AG	GG	AG	AG	--	GG	AG	GG	GG	AG	GG
CC	CC	AA	CC	AC	AA	AC	AC	AA	CC	--	AC	CC
CC	CC	CG	CC	CG	CG	CC	CC	CG	CC	CC	CG	CC
AA	AA	GG	AA	AG	GG	AG	AG	GG	AA	AA	AG	AA
CC	CC	AA	CC	AC	AA	AC	AC	AA	CC	--	--	CC
GG	GG	TT	GG	GT	TT	GT	GT	TT	GT	GG	GT	GG
AC	AA	CC	AA	AC	CC	AC	AC	--	AC	AA	AC	AA
CT	CC	CT	CC	CT	CT	CC	CC	CT	CC	CC	CT	CC
GT	GG	TT	GG	GT	TT	GT	GT	TT	GT	GG	GT	GG
GG	GG	GG	GG	GG	AG	AG	AG	AG	AG	GG	GG	GG
CT	TT	CC	TT	CT	CC	--	CT	CC	CT	TT	--	TT
CT	CC	CT	CC	CT	CT	--	CC	CT	CC	CC	--	CC
AT	TT	AA	TT	AT	AA	AT	AT	AA	AT	TT	AT	TT
CG	GG	CC	GG	CG	CC	CG	CG	CC	CG	CG	CG	GG
GT	TT	GG	TT	GT	GT	GT	GT	GG	GT	GT	GT	TT
GG	GG	AG	GG	GG	GG	AG	AG	AG	AG	GG	GG	GG
TT	GT	TT	GT	TT	TT	GT	GT	GT	TT	--	--	GT
CT	TT	CC	TT	CT	CT	CC	CT	CT	CC	CT	CT	TT
AC	AC	CC	AA	AC	AC	CC	AC	AC	CC	CC	AC	AC
--	--	CC	CT	CC	CT	CC	CC	CC	CC	CC	CT	CT
CG	CG	GG	CC	CC	CG	GG	CG	CG	GG	GG	CG	CG
CT	CT	CC	TT	TT	CT	CC	CT	CT	CC	CC	CT	CT
TT	TT	GT	TT	TT	TT	GT	GT	GT	GT	GT	--	TT
CT	CT	CT	TT	TT	CT	CT	TT	CT	TT	TT	CT	CT
TT	--	AT	TT	TT	TT	AT	AT	AT	AT	TT	--	TT
AC	AC	CC	AC	AC	AC	CC	CC	CC	CC	CC	AC	AC
CT	CT	CT	CT	CC	CT	CT	CT	TT	CT	--	--	CC
AG	AG	AA	AG	AG	AG	--	AA	AA	AA	AA	AG	AG
AG	AG	GG	AG	AG	AG	GG	GG	GG	GG	--	AG	AG
GG	CG	GG	GG	CG	CG	GG	CG	GG	GG	CG	--	--
CT	CT	CC	CC	CT	CT	CC	TT	CC	CT	TT	CC	TT
CC	CT	CC	CC	CT	CT	CC	CT	CC	CT	CC	CC	TT
CC	CC	CC	CC	CT	CT	CC	CT	CC	TT	CC	CC	TT
TT	TT	TT	TT	CT	CT	TT	CT	CT	CC	TT	--	CC
GG	GG	GG	GG	GT	GT	GG	GG	GT	TT	GG	--	TT
TT	--	TT	TT	CT	CT	--	TT	CC	CC	TT	--	CC
TT	TT	TT	TT	TT	AT	TT	TT	AA	AA	TT	--	AA
GG	GG	GG	GG	GG	AG	AG	--	AA	AA	--	--	AA
AA	AC	AC	AA	AA	AA	AC	AA	CC	AC	AA	AA	AC

GG	AG	AG	GG	GG	GG	AG	GG	AA	AG	GG	GG	AG
AG	AG	AG	GG	GG	GG	AG	GG	AA	--	--	--	AG
CT	CT	CT	CC	CC	CC	CT	CC	TT	CT	CC	CC	CT
AG	AG	AG	AA	AA	AA	AG	AA	GG	--	AA	AA	AG
GT	GT	GT	TT	TT	--	GT	TT	--	GT	TT	--	GT
CT	CT	CT	CC	CC	CC	CT	CC	TT	CT	CC	CC	CT
AG	AG	AG	AA	AA	AA	AG	AA	GG	AG	AA	--	AG
AG	AG	AG	AA	AA	AA	AG	AA	GG	AG	AA	--	AG
AG	AG	AG	GG	GG	--	AG	GG	AA	AG	GG	--	AG
CG	CG	CC	CC	CC	CC	CG	CC	--	CG	CC	CC	CG
AG	AG	GG	GG	GG	GG	AG	GG	AA	AG	--	--	AG
AC	AC	CC	CC	CC	CC	AC	CC	AA	AC	--	--	AC
AG	AG	AA	AA	AA	AA	AG	AA	GG	AG	AA	AA	AG
AG	AG	AA	AA	AA	AA	AG	AA	GG	AG	AA	--	AG
AT	AT	TT	TT	TT	TT	AT	TT	AA	AT	TT	--	AT
CT	CT	CC	CC	CC	CC	CT	CC	TT	CT	CC	CC	CT
CT	CT	CC	CC	CC	CC	CT	CC	TT	CT	CC	--	CT
CG	--	GG	GG	GG	GG	--	GG	CC	CG	GG	--	CG
CT	TT	TT	TT	TT	TT	CT	TT	CC	CT	--	--	CT
CT	CC	CC	CC	CC	CC	--	CC	TT	CT	CC	--	CT
CT	TT	TT	TT	TT	TT	CT	TT	CC	CT	--	--	CT
AT	TT	TT	TT	TT	TT	AT	TT	AA	AT	TT	TT	AT
AT	AA	AA	AA	AA	AA	AT	AA	TT	AT	AA	--	AT
CT	--	TT	TT	TT	TT	CT	CT	CC	CT	TT	--	CT
CT	CC	CC	CC	CC	CC	CT	CC	TT	CT	--	CC	CT
AT	AA	AA	AA	AA	AA	AT	AA	TT	AT	AA	--	AT
AG	GG	GG	GG	GG	GG	AG	GG	AA	AG	GG	GG	AG
GT	GG	GG	GG	GG	GG	--	GG	TT	GT	GG	--	GT
CT	--	TT	TT	TT	TT	CT	TT	--	CT	--	--	CT
CG	CC	CC	CC	CC	CC	CG	CC	GG	CG	CC	CC	CG
AG	GG	GG	GG	GG	GG	AG	GG	AA	AG	GG	GG	AG
CT	TT	TT	TT	TT	TT	CT	TT	--	CT	CT	--	CT
CT	TT	TT	TT	--	TT	CT	TT	CC	CC	TT	--	CT
AT	TT	TT	TT	TT	TT	AT	TT	AA	AA	--	TT	AT
AG	AA	AA	AA	AA	AA	AG	AA	GG	GG	AG	--	AG
AG	AA	AA	AA	AA	AA	AG	AA	GG	GG	AA	--	AG
GT	TT	GT	TT	TT	TT	GT	GT	GG	GG	GT	TT	GT
AG	AA	AG	AA	AG	AA	--	AG	GG	GG	AG	--	AG
GG	GG	GG	GG	AG	GG	AG	AG	AG	AG	AG	--	AG
TT	--	CT	TT	CT	CT	--	CT	CT	CT	TT	--	CT
AG	AG	AG	AG	AA	AG	GG	AG	AG	AA	--	AG	AG
AG	AG	AG	AG	AG	GG	--	GG	GG	AG	AG	AG	GG
AG	AG	AA	AG	AA	AG	--	AG	AG	AA	AG	--	AG
CG	CG	GG	CG	GG	CG	CC	CG	CG	GG	CG	CG	CG
CT	TT	CC	TT	CC	CC	TT	CT	CT	CC	CT	CT	CT
AC	AC	CC	AC	CC	CC	AC	CC	CC	CC	AC	AC	CC
AA	--	AA	AG	AG	AA	--	AG	AA	AA	AA	--	AA

AC	AC	CC	AC	CC	CC	AC	CC	CC	CC	AC	--	CC
AC	CC	AC	CC	CC	AC	--	CC	AC	AC	CC	AC	AC
CT	CT	TT	CT	TT	TT	TT	TT	TT	TT	CT	CT	TT
AG	GG	AA	GG	AG	AA	AG	AG	AA	AA	GG	AG	AA
AG	AA	GG	AA	AG	GG	AG	AG	GG	GG	AA	AG	GG
CT	CC	TT	CC	CT	TT	CT	CT	TT	TT	CC	CT	CT
CT	TT	CT	CT	TT	CT	CT	TT	TT	CT	TT	--	TT
CG	GG	CC	CG	CG	CC	CG	CG	CG	CC	GG	CG	CG
AG	GG	AA	AG	AG	AA	AG	AG	AG	AA	GG	--	AG
CT	--	CT	CT	CT	TT	TT	CT	CT	TT	CC	CT	CT
AT	AA	--	AT	AA	--	AT	AA	AA	AT	AT	AT	AA
AC	AA	AC	AC	AC	CC	CC	AC	AC	CC	AA	AC	AC
AG	AG	GG	AG	GG	GG	GG	GG	--	AG	AG	AG	GG
CT	--	CC	CT	CT	CC	CC	CC	CC	CT	CT	CT	CC
CT	CT	CC	CT	CT	CC	--	CC	CC	CT	CT	CT	CC
GT	GT	GG	GT	GT	GG	GG	--	GG	GT	--	GT	GG
CT	CC	CC	TT	CC	TT	TT	TT	CC	CT	CC	TT	TT
GG	--	AG	GG	AG	GG	GG	GG	AG	GG	AG	--	GG
AG	AA	AG	GG	AA	GG	GG	GG	AA	AG	AG	GG	GG
GG	GG	GG	CG	GG	CG	CG	CG	GG	GG	CG	CG	CG
AG	AA	AG	GG	AA	GG	GG	GG	AA	AG	AG	GG	GG
AG	GG	AG	AA	GG	AA	AA	AA	GG	AG	AG	AA	AG
GT	GT	GT	TT	GT	TT	TT	TT	GT	GT	TT	--	GT
AG	GG	AG	AA	GG	AA	AA	AA	AG	AG	AG	--	AG
AG	GG	AG	AA	AG	AA	--	AG	AG	AG	AG	--	AG
GT	GT	GT	GG	GG	GG	GG	GG	GT	GT	GT	GG	GT
CT	TT	CT	CC	CC	CC	CC	CT	CT	CT	TT	--	CT
CT	CC	TT	CT	TT	TT	TT	CT	CT	CC	CC	TT	CT
CC	CT	CC	CC	CC	CC	CC	CT	CC	CT	CT	CC	CC
AG	GG	AA	AG	AA	AG	AA	AG	AG	GG	GG	AA	AG
TT	CT	CC	CT	CC	TT	CC	CT	CT	TT	TT	CC	CT
GG	AG	AA	AG	AG	GG	AA	AG	AG	GG	GG	AG	AG
TT	CT	CC	TT	CT	TT	--	CT	CT	TT	--	--	CT
CG	CG	GG	CC	CG	CC	--	CG	CG	CC	CG	CG	CG
CT	CT	CC	TT	CT	TT	CC	CT	CT	TT	CT	CT	CT
AA	AC	AC	CC	AC	AA	AC	AC	--	AC	AC	AC	CC
AG	AG	AG	AA	AG	GG	--	--	AG	AG	--	AG	AA
CT	CT	CT	CC	CT	TT	TT	CT	CT	CT	CT	--	CT
AG	AG	AG	GG	--	AA	--	AG	AG	AG	AG	--	AG
AG	AG	AG	GG	AG	AA	AA	GG	AG	AG	AG	AA	AG
TT	--	CT	CT	TT	TT	TT	CT	--	CT	--	--	CT
AG	GG	AG	AA	GG	GG	--	AA	AG	AG	AA	--	AG
AG	AA	AG	AG	AA	AA	AA	AG	AG	AA	AA	--	AA
GT	TT	GT	GT	TT	TT	TT	GT	GT	TT	TT	TT	TT
GT	TT	GT	GT	TT	TT	TT	GT	--	TT	TT	--	TT
AC	AA	CC	CC	AA	AA	AA	CC	AC	AC	AC	AA	AC
AG	AA	GG	GG	AA	AA	AA	GG	--	AG	--	--	AG

CG	CC	GG	GG	CC	CC	--	GG	CC	CG	CC	CC	CG
CG	CC	GG	GG	CC	CC	CC	GG	CC	GG	--	--	CG
CT	TT	CT	CC	CT	TT	--	CC	TT	CC	CT	TT	CT
CT	CC	CT	TT	CT	CT	CC	TT	CC	TT	CT	CC	CT
AT	AA	AT	AT	AT	AT	AT	TT	AA	AT	AT	--	AT
GG	AG	AG	AG	AG	GG	GG	GG	AG	GG	AG	AG	AG
CT	CC	CT	CT	CT	TT	--	TT	CC	CT	CT	CC	CT
CC	CT	CT	CT	CT	CC	CT	CC	TT	CT	CT	--	CT
TT	CT	CT	CT	CT	TT	--	TT	CT	CT	--	CC	CC
AA	AG	AG	AG	AG	AA	AA	AA	AG	AG	AG	GG	GG
CG	--	CG	CG	CG	CG	CG	CG	CC	CC	CC	CC	CC
CG	CG	GG	GG	GG	CG	CG	CG	CG	CG	GG	GG	GG
GT	GG	GT	GT	GT	GT	GT	GT	GG	GG	GG	--	GG
CC	CG	CG	CG	CG	CC	CG	CG	CC	CG	CG	CC	CC
AG	--	AA	AG	AA	GG	AA	AA	AG	AG	AG	AG	AA
AA	AC	AC	AC	AC	AA	AC	AC	AA	AC	AC	AA	AC
AA	AT	AT	AT	AT	AA	AT	AT	AA	AT	AT	AA	AA
AG	AG	AA	AG	AA	GG	AA	AA	AG	AA	AG	AG	AG
TT	AT	TT	AT	TT	AA	TT	TT	AA	TT	AT	AT	AT
AA	AG	AA	AG	AA	AG	AA	AA	AG	AA	AA	AG	AG
AC	CC	AC	CC	AC	CC	AC	--	CC	AC	CC	CC	AC
TT	CT	CT	TT	TT	CT	TT	TT	CT	TT	--	--	CT
GG	GT	GT	GG	GG	GT	GG	GG	GT	GG	GG	TT	TT
GG	GT	GT	GG	GG	GT	GG	GG	GT	GG	GG	--	TT
TT	CT	CT	TT	TT	CT	TT	TT	CT	--	TT	--	CC
GG	CG	CG	GG	GG	CG	GG	GG	CG	GG	--	CC	CC
AA	AG	AG	AA	AA	AG	--	AA	AG	AA	AA	--	GG
TT	CT	CT	TT	TT	CT	TT	TT	CT	TT	TT	--	CC
GG	GT	GT	GG	GG	GT	GG	GG	GT	GG	GG	--	TT
CC	CT	CT	CC	CC	CT	CC	CC	CT	CC	CC	TT	TT
CC	CG	CG	CC	CC	CG	CC	CC	CG	CC	CC	--	GG
AA	AG	AG	AA	AA	AG	AA	AA	AG	AA	--	--	GG
CC	AC	AC	CC	CC	AC	CC	CC	AC	--	CC	AC	AA
CC	CT	CT	CC	CC	CT	--	CC	CT	CC	CC	--	TT
AG	AG	AG	AG	AG	AG	AG	AG	--	AG	AG	--	GG
CC	--	AC	CC	CC	AC	--	CC	AC	CC	--	AC	AA
TT	TT	CT	TT	TT	CT	TT	TT	CT	TT	TT	CT	CC
AA	AA	AG	AA	AA	AG	AA	AA	AG	AA	--	AG	GG
CC	CC	CG	CC	CC	CG	--	CC	CG	CC	CG	CG	GG
TT	TT	GT	TT	TT	GT	TT	TT	GT	TT	GT	GT	GG
TT	TT	CT	TT	TT	CT	TT	TT	CT	TT	CT	CT	CC
CT	CT	CT	CT	CT	CT	CT	CT	CT	CT	TT	TT	TT
GG	GG	AG	GG	GG	AG	--	GG	AG	GG	GG	--	AG
GG	GG	CC	GG	GG	CG	GG	GG	CG	GG	CG	CG	CC
AT	AT	AA	AT	AT	AA	AT	AT	AA	AT	AT	AT	AA
AG	AG	AA	AG	AA	AG	AG	AG	AG	AA	AA	AA	AA
AC	AC	CC	AC	CC	AC	AC	AC	AC	CC	CC	CC	CC

CT	CT	TT	CT	TT	CT	CT	CT	CT	TT	TT	--	TT
CT	CT	CT	CT	CT	TT	CT	CT	CT	CT	CT	CT	TT
AT	AT	AG	AT	AG	GT	AT	AT	AT	GG	AG	AG	GG
AT	AT	AA	AT	AA	AT	AT	AT	AT	AA	AA	AA	AA
AG	AA	AG	AA	AG	AG	AA	AA	AA	GG	AG	AG	GG
AC	AA	AC	AA	AC	AC	AA	AC	AA	CC	AC	--	CC
AG	AG	AA	AG	AA	AG	AG	AG	AG	AA	--	AA	AA
AG	AA	AA	AA	AG	AG	AG	AG	AA	GG	--	--	GG
AG	GG	AA	GG	AG	AG	AG	AG	GG	AA	AG	AA	AA
AG	AA	GG	AA	AG	AG	AG	AG	AG	GG	--	GG	GG
GT	TT	GG	TT	GT	GT	GT	GT	GT	GG	GT	--	GG
GG	AG	GG	GG	AG	GG	GG	GG	GG	GG	GG	GG	AG
CT	TT	CC	CT	CT	CT	CT	CT	CT	CC	CT	--	CT
GG	GG	CC	CG	CG	GG	CG	CG	CG	CC	CG	CC	CG
CT	TT	TT	CT	TT	CT	CT	CT	CT	TT	TT	--	TT
CT	CT	TT	TT	CT	CT	TT	TT	TT	TT	--	TT	CT
CC	CT	CT	CC	CT	CC	CT	CC	CT	CT	CC	CT	CT
GT	GG	GG	GT	GT	GG	GG	GT	GG	GT	GG	--	GT
AG	AA	AG	AG	AA	GG	AG	AG	AG	AG	GG	AG	AG
AG	GG	AG	AG	AG	AA	AG	AG	AG	AG	--	AG	AG
AA	AG	AG	AA	AG	AA	AG	AA	AG	AG	AA	AG	AG
GT	TT	GT	GT	TT	GG	--	GT	GT	TT	--	--	TT
AA	AG	AG	AG	AG	AG	AG	AA	AG	AG	--	AG	AG
CC	--	CT	CT	CT	CT	CT	CT	CT	CT	CC	CT	CT
AG	AA	AA	AA	AA	AA	AA	AA	AA	AA	AG	--	AA
AG	AG	AG	AG	AA	AG	--	AA	AG	AA	GG	AG	AA
AG	AG	AG	AG	AG	AG	AG	AA	AG	AA	GG	AG	AA
CG	CG	CG	CG	CG	CG	CG	CC	CG	CC	GG	CG	CC
CG	CG	CG	CG	--	CC	--	CC	CG	CC	GG	CG	CC
AG	AG	AG	AG	AG	AA	AG	AA	AG	AA	GG	AG	AA
AG	GG	GG	GG	AG	AG	--	AG	GG	AG	--	--	AG
CT	CT	CT	CT	CT	CC	CT	CC	CT	CC	TT	CT	CC
AC	CC	CC	CC	AC	AC	CC	AC	CC	AC	CC	--	AC
CG	--	CC	CC	CG	CG	CC	CG	CC	CG	CC	--	CC
AC	CC	CC	CC	AC	AC	CC	AC	CC	AC	CC	CC	CC
AC	AC	CC	CC	AC	CC	--	CC	AC	CC	AA	CC	AC
AA	AG	AG	AG	AA	AG	AG	AG	AG	AG	AA	AG	AG
AG	AG	GG	GG	AG	GG	GG	GG	AG	GG	AG	GG	AG
GG	AG	AG	AG	GG	AG	AG	AG	AG	AG	AG	--	AG
CC	--	TT	TT	CT	TT	TT	TT	CT	TT	TT	TT	CT
CC	TT	TT	TT	CT	TT	--	TT	CT	--	TT	--	CT
AA	AG	AG	AG	AG	AG	AG	AG	AA	AG	AG	--	AA
CC	CT	CT	--	--	CT	CT	CT	CC	CC	--	CT	CC
AG	GG	AA	GG	AG	AA	--	AG	AG	AA	AG	GG	AA
CC	CC	AC	CC	CC	AC	AC	CC	CC	AC	AC	--	AC
CT	CC	CT	CC	CT	CT	CT	CT	CT	CT	CC	--	CT
AG	AA	GG	AA	AG	GG	GG	AG	AG	GG	AA	AA	GG

AG	AA	AG	AA	AG	AG	AG	AG	AG	AG	AA	AA	AG
AG	AG	AG	AA	AG	AG	AG	AG	AG	GG	AA	--	GG
AC	AA	AC	CC	AC	AC	AC	AC	AC	AA	CC	CC	AA
AG	AA	AG	AG	AG	AG	AG	AG	AG	AG	AG	GG	AA
GT	--	GT	GT	GT	GT	GG	GT	GT	GT	GT	--	GG
AG	AG	AA	AG	AA	AA	AA	AA	--	AA	--	--	--
CT	CC	CT	CT	CT	CT	CT	CT	CC	CT	CT	TT	CC
CT	--	CC	CT	CC	CC	--	CC	CT	CC	--	--	CT
AG	AA	AG	AG	AG	AG	AG	AG	AA	AG	AG	GG	AG
GG	CG	CG	GG	CG	CG	CG	GG	CG	CG	CG	--	GG
TT	CT	CT	TT	TT	CT	CT	TT	CT	CT	--	--	TT
AG	GG	AA	AG	AA	AG	AG	AA	GG	AG	AG	--	AA
AG	GG	AA	AG	AA	GG	AG	AA	GG	AG	AG	AA	AA
CG	GG	CC	CG	CC	CG	CG	CC	CG	GG	CG	--	CC
AG	GG	AA	AG	--	AG	--	AA	AG	GG	AG	--	AA
AG	--	AA	AG	AG	AG	AG	AG	AG	GG	--	AG	--
CT	TT	CC	CT	CT	CT	CT	CT	CT	TT	--	CC	CT
AG	GG	AA	AG	AG	AG	AG	AG	AG	GG	AG	AA	AG
CG	CC	GG	CG	CG	CG	CG	CG	CG	CC	CG	--	CG
AC	CC	AA	AC	AC	AC	AC	AC	--	CC	AC	--	AC
AG	AA	GG	AG	AG	AA	AG	AA	AG	AA	AG	--	AG
GT	GG	TT	GT	GT	GG	GT	GG	GT	GG	GT	TT	GT
AG	GG	AA	AG	AG	GG	AG	GG	AG	GG	--	--	AG
AG	GG	AA	AG	AG	GG	AG	GG	AG	GG	AG	AA	AG
AG	AA	GG	AG	AG	AA	--	AA	AG	AA	AG	--	AG
AC	AA	CC	AC	AC	AA	AC	AA	AC	AA	AC	CC	AC
AG	AA	GG	AG	AG	AA	AG	AA	AG	AA	GG	GG	AG
AG	AA	GG	AG	AG	AA	AG	AA	AG	AA	--	GG	AG
CT	TT	CC	CT	CT	TT	CT	TT	CT	TT	CT	CC	CT
CG	GG	CC	CG	CG	GG	CG	GG	CG	GG	CG	CC	CG
AC	AA	CC	AC	AC	AA	AC	AA	AC	AA	AC	--	AC
CT	TT	CC	CT	CT	TT	CT	TT	CT	TT	CT	--	TT
GT	TT	GG	GT	GT	TT	GT	TT	GT	TT	GT	--	TT
CT	CC	TT	CT	CT	CC	CT	CC	CT	CC	--	--	CC
CG	CC	GG	CG	CG	CC	CG	CC	CG	CC	CC	GG	CC
AT	TT	AA	AT	AT	TT	AT	TT	AT	TT	TT	--	TT
CT	CC	TT	CT	CT	CC	CT	CC	CT	CC	CC	--	CC
AG	GG	AA	AG	AG	GG	--	GG	AG	GG	GG	--	GG
AG	AA	GG	AG	AG	AA	AG	AA	AG	AA	AA	GG	AA
AT	AA	TT	AT	AT	AA	AT	AA	AT	AA	--	--	AA
AG	GG	AA	AG	AG	GG	AG	GG	AG	GG	GG	AG	GG
AG	AA	GG	AG	AG	AA	AG	AA	AG	AA	AA	--	--
CG	CC	GG	CG	CG	CC	CG	CC	CG	CC	CC	--	CC
AG	AA	GG	AG	AG	AA	AG	AA	AG	AA	AA	GG	AA
CG	GG	CC	CG	CG	GG	CG	GG	CG	--	--	--	GG
AG	GG	AA	AG	AG	GG	AG	GG	--	GG	--	--	GG
AC	AA	CC	AC	AC	AA	--	AA	AC	AA	--	CC	AA

AG	GG	AA	AG	AG	GG	AG	GG	AG	GG	--	--	GG
CG	CC	GG	CG	CG	CC	CG	CC	CG	CC	--	--	CC
CT	CC	TT	CT	CT	CC	CT	CC	CT	--	CC	TT	CC
AT	TT	AA	AT	AT	TT	AT	TT	AT	TT	TT	AA	TT
CT	CC	TT	CT	CT	CC	CT	CC	CC	CC	CC	--	CC
AT	AT	TT	AT	AT	AA	AT	AA	AA	AA	--	TT	AA
AT	AT	AT	AT	AT	TT	AT	TT	TT	TT	--	--	TT
CG	CG	CG	CG	CG	GG	CG	GG	GG	GG	GG	--	GG
CT	CT	CT	CT	CT	CC	CT	CC	CC	CC	CC	--	CC
AG	AG	AG	AG	AG	AA	AG	AA	AA	AA	AA	AG	AA
AC	AC	AC	AC	AC	AA	AC	AA	AA	AA	AA	CC	AA
GT	GT	GT	GT	GT	TT	GT	TT	TT	TT	TT	GG	TT
CT	CT	CT	CT	CT	CC	CT	CC	CC	CC	CC	TT	CC
CG	CG	CG	CG	CG	GG	CG	GG	GG	GG	GG	CC	GG
GT	GT	GT	GT	GT	TT	GT	TT	TT	TT	TT	GG	TT
CT	CT	CT	CT	TT	TT	CT	TT	TT	TT	TT	CC	TT
AG	AG	AG	AG	AA	AA	AG	AA	AA	AA	--	GG	AA
AC	AC	AC	AC	AA	AA	AC	AA	AA	AA	--	CC	AA
CT	CT	CT	CT	CC	CC	--	CC	CC	CC	CC	--	--
AC	AC	AC	AC	AA	AA	AC	AA	AA	AA	AA	--	AA
AG	AG	AG	AG	AA	AA	AG	AA	AA	AA	AA	--	AA
GT	GT	GT	GT	GG	GG	GT	GG	GG	GG	GG	--	GG
GT	GT	GG	GT	GG	GG	GT	GG	GG	GG	GG	--	GG
AG	AG	AG	AG	GG	GG	AG	GG	GG	GG	GG	AA	GG
CT	CT	CT	CT	CC	CC	CT	CC	CC	CC	--	TT	CC
CT	CT	CT	CT	CC	CC	CT	CC	CC	CC	CC	--	CC
AG	AG	AG	AG	AA	AA	AG	AA	AA	AA	AA	GG	AA
CT	CT	CT	CT	CC	CC	--	CC	CC	CC	CC	--	CC
CT	CT	CT	CT	TT	TT	CT	TT	TT	TT	TT	CC	TT
AG	AG	AG	AG	GG	GG	AG	GG	GG	GG	GG	AA	GG
CT	CT	CT	CT	CC	CC	CT	CC	CC	CC	--	TT	CC
CT	CT	TT	CT	TT	TT	CT	TT	TT	TT	--	CT	TT
CT	CT	CT	CT	CC	CC	CT	CC	CC	CC	CT	--	CC
AG	GG	AG	AG	AA	AA	AG	AA	AA	AA	AG	GG	AA
AC	CC	AC	AC	AA	AA	AC	AA	AA	AC	AC	CC	AA
CG	CC	CC	CG	CG	CG	CG	CG	CG	CG	CG	--	CG
GT	--	GT	GT	GG	GG	GT	GT	GT	GT	GT	--	GG
AG	AG	AG	AG	AG	GG	AG	AA	AG	AG	AG	AA	GG
CT	CT	CT	CT	CT	CT	CT	CC	CT	--	CT	--	TT
AG	GG	GG	AG	GG	GG	AG	GG	AG	AG	GG	--	AG
AG	--	AG	AG	AG	AA	AG	--	AG	AG	AG	AA	GG
CG	CG	CC	CG	CG	CC	CG	CC	CC	CG	--	CG	GG
CT	TT	TT	CT	TT	TT	CT	TT	TT	CT	TT	--	CT
GT	GT	GG	GT	GT	GG	TT	GG	GG	GT	GT	GT	TT
AG	GG	AG	GG	GG	AG	AG	AG	GG	AG	GG	--	AG
AG	GG	GG	GG	AG	AG	GG	GG	AG	GG	GG	GG	AG
CC	AA	AC	AA	AC	AC	AC	AC	AC	AC	AA	--	CC

AA	TT	AT	TT	AT	AT	AT	AT	AA	AT	--	TT	AA
AT	TT	AT	TT	AT	AT	AT	AT	AT	AT	TT	AT	AA
AT	AA	AT	AA	AT	AT	AT	AT	--	AT	AA	AT	TT
CT	TT	CT	TT	CT	CT	CT	CT	--	CT	TT	CT	CC
AG	GG	GG	GG	AG	AG	GG	AG	AA	AG	GG	AG	AA
AC	AA	AA	AC	AC	AC	AA	AC	AC	AC	AA	AC	AC
CT	--	TT	CT	CT	CT	--	CT	CT	CC	TT	CT	CT
AG	GG	GG	AG	AG	AG	GG	AG	AG	AA	GG	AG	AG
GT	GG	GG	GT	GT	GT	GG	GT	GT	TT	GG	GT	GG
AG	GG	GG	AG	--	GG	GG	AG	GG	AA	AG	AG	GG
CG	CC	CC	CC	CC	CC	CC	CC	CC	CG	CC	CC	CC
AG	GG	GG	GG	GG	GG	GG	GG	GG	AG	GG	GG	GG
CG	CC	CC	CC	CG	CC	CG	CG	CC	GG	CG	CG	CC
AG	AA	AA	AA	AG	AA	AG	AG	AA	GG	AG	--	AA
AG	GG	GG	GG	AG	GG	--	AG	GG	AA	--	AG	GG
AG	AA	AA	AA	AG	AA	AG	AG	AA	GG	AG	AG	AA
GT	GG	GG	GG	GG	GG	GG	GG	GG	GT	GG	GG	GG
AG	GG	GG	GG	AG	AG	AG	AG	GG	AG	AG	AG	AG
GT	TT	--	TT	TT	TT	TT	TT	TT	GT	TT	TT	TT
CG	GG	GG	GG	CG	CG	CG	GG	GG	CG	CG	CG	CG
TT	--	TT	TT	TT	TT	CT	CT	CT	TT	--	CT	TT
GG	--	GG	GG	--	GG	CC	CG	--	GG	CC	CG	CG
CC	CC	CC	CC	CG	CC	CG	CC	CC	--	CG	--	CG
AC	AA	AC	AA	AA	AC	AC	AC	AC	AA	AC	AC	AA
CT	CC	CT	CC	CC	CT	CT	CT	CT	CC	CC	CT	CC
AG	AA	AG	AA	AA	AG	AG	AG	AG	AA	AA	--	AA
CT	CC	CT	CC	CC	CT	CT	CT	CT	CC	CC	CT	CC
GG	GG	GG	GG	CG	GG	--	GG	GG	GG	GG	GG	CG
CT	CC	CT	CC	CT	CT	CC	CT	CT	CC	CC	CT	--
AG	AG	AG	AA	GG	AG	AG	AG	AG	AA	AA	AG	AA
GT	GG	GT	GG	GT	GT	GG	GT	GT	GT	GG	GT	GG
GT	GT	GT	TT	GG	GT	GT	GT	GT	GG	TT	GT	TT
CG	--	CG	GG	CG	CG	GG	CG	CG	--	GG	CG	--
CG	CG	CG	GG	CC	CG	CG	CG	CG	CC	GG	CG	GG
AG	GG	AG	GG	AG	AG	GG	AG	AG	AG	GG	AG	GG
CT	CT	CT	TT	CC	CT	CT	CT	CT	CC	TT	CT	TT
GT	GT	GT	GG	TT	GT	GT	GT	GT	TT	GT	GT	GG
AG	GG	AG	GG	AG	AG	GG	AG	AG	AG	GG	AG	GG
AG	--	AG	AA	GG	AA	AG	AG	AG	GG	AA	AG	AA
CT	--	CT	TT	CC	TT	CT	CT	CT	CC	--	--	--
AT	TT	AT	TT	AT	TT	--	AT	AT	AT	TT	--	TT
GT	TT	GT	TT	GT	TT	TT	GT	GT	GT	TT	GT	TT
CT	CT	CT	CC	TT	CC	CT	CT	CT	TT	CC	CT	CC
AG	AA	AG	AA	AG	AA	AG	AG	AG	AG	AA	AG	AA
AT	AT	AT	TT	--	TT	--	AT	AT	AA	TT	--	TT
AG	AA	AG	AA	AG	AA	AG	AG	AG	AG	AA	AG	AA
CT	TT	CT	TT	CT	TT	CT	CT	CT	CT	TT	CT	TT

AT	TT	AT	TT	AT	TT	AT	AT	AT	AT	--	--	TT
GT	GT	GT	GG	TT	GG	TT	GT	GT	TT	GG	GT	GG
AG	GG	AG	AA	GG	AA	GG	AG	AG	GG	AA	AG	AA
CT	TT	CT	CC	TT	CT	TT	CT	CT	TT	--	CT	CC
CT	TT	CT	CC	TT	CT	TT	CT	CC	TT	CC	CT	CC
CT	--	CT	CC	--	CT	CT	CT	CC	TT	CC	CT	CC
GT	TT	GT	GG	TT	GT	GT	GT	GG	TT	GG	GT	GT
CG	GG	CG	CC	GG	CG	--	CG	CC	GG	CC	--	CG
AC	AA	AC	CC	AA	AC	AC	AC	CC	AA	CC	--	AC
AG	GG	AG	AA	GG	AG	AG	AG	AA	GG	AA	--	AG
AG	AA	AG	GG	AG	AG	AG	GG	GG	AA	--	--	AG
CC	TT	CT	CC	CC	CT	CT	CT	CC	CT	--	TT	CT
CC	AA	AC	CC	CC	AC	AC	AC	CC	AC	AA	AA	AC
GT	--	TT	GT	GT	GT	GT	TT	--	TT	TT	--	GT
CG	GG	GG	CG	CG	GG	--	CG	CG	CG	--	GG	GG
AG	AG	GG	AA	AA	AG	AG	GG	AA	GG	GG	GG	AG
TT	TT	TT	CT	CT	TT	TT	TT	TT	TT	TT	TT	TT
GT	GT	TT	GT	GT	GT	GT	TT	TT	TT	TT	--	GT
CC	CC	CC	CT	CT	CC	CC	CC	CC	CC	CC	--	CC
AC	AC	AA	AC	AC	AC	--	AA	AA	AA	AA	--	AC
AG	AG	AA	AG	GG	GG	AA	AA	AA	AA	AA	AA	AG
AA	AG	AA	AG	AG	AA	AA	AA	AA	AA	AA	AA	AG
TT	GT	TT	GT	GG	GT	--	TT	TT	TT	TT	GT	GT
CC	--	CC	CT	TT	CT	--	CC	CC	CC	CT	CT	CT
AA	AC	AC	AC	CC	AC	AC	AA	AA	AA	AC	AC	AC
TT	AT	AT	AT	AA	AT	AT	TT	TT	TT	AT	AT	AT
GT	GG	GG	GG	GG	GT	--	GT	GT	GG	GG	GT	GT
AG	AG	AG	AG	AA	AA	AG	AG	AG	AG	--	--	AG
CC	CG	CG	CG	GG	CG	CG	CC	CC	CG	--	--	CC
AA	--	AG	AG	--	--	AG	AA	AA	AG	--	AG	AA
CG	CC	CG	CG	CG	CG	CG	CG	CG	GG	GG	CG	CC
AG	--	AG	AG	AG	AG	AG	AG	AG	GG	GG	AG	AA
AG	AA	AA	AG	AA	AG	AG	AA	AG	AG	AG	AA	AA
AA	--	AT	AA	AT	AA	--	AT	AA	AT	AT	AT	AT
AG	--	AA	AG	AA	AG	AG	AA	AG	AG	AG	--	AA
AA	AG	AG	AG	AG	AA	AA	AG	AA	AA	AA	AG	AG
AC	AA	AC	AA	AA	AC	CC	AC	AC	CC	CC	AC	AC
CC	--	AC	CC	CC	CC	--	AC	CC	AC	CC	--	AC
AC	CC	AC	CC	CC	AA	AA	AC	AA	AA	AA	--	AC
GT	GG	GT	GG	GG	TT	TT	GT	TT	TT	--	--	GT
AT	AA	AT	AA	AA	TT	TT	AT	TT	--	TT	--	AT
AA	AA	AG	AA	AA	AA	AG	AG	AG	AG	AG	--	AG
CC	CC	AC	CC	CC	AC	AC	--	AC	AC	AC	CC	AC
AT	AA	AT	AA	AA	AT	TT	AT	TT	TT	AT	AA	AT
CT	CC	CT	CC	CC	CT	TT	CT	CT	TT	CT	--	CT
AG	GG	AG	GG	GG	AG	AA	AG	AG	AG	AG	GG	AG
CT	CC	CT	CC	CC	CT	TT	CT	CT	CT	CT	CT	CT

CT	--	CT	TT	TT	CT	--	CT	CT	CT	CC	--	CT
AG	AG	AG	GG	GG	AG	--	AG	AG	AG	AG	--	AG
CT	CT	CT	CC	CC	CT	TT	CT	CT	CT	CT	CT	CT
CT	CT	CT	TT	TT	CT	--	CT	CT	CT	CT	CT	CT
CT	CT	CT	CC	CC	CT	TT	CT	CT	CT	--	CT	CT
AG	AG	AG	GG	GG	AG	--	AG	AG	AG	AG	AG	AG
CT	CT	CT	TT	TT	CT	CC	CT	CT	CT	--	--	CT
AG	AG	AG	GG	GG	AG	AA	AG	AG	AG	AG	AG	AG
CG	CG	CG	CC	CC	CG	GG	CG	CG	--	CG	--	CG
AC	AC	AC	AA	AA	AC	CC	AC	AC	AC	--	AC	AC
CT	CT	CT	CT	CC	CT	TT	CT	CT	CT	CT	CT	CC
CT	CT	CT	CT	CC	CT	--	CT	CT	CT	CT	--	CC
AT	AT	AT	AT	TT	AT	--	AT	AT	AT	--	AT	TT
CT	CC	CT	CC	CC	CT	--	CT	CT	CT	TT	CT	CC
AC	AC	AC	AC	AA	AC	CC	AC	AC	AC	AC	AC	AA
GT	GT	GT	GT	TT	GT	GG	GT	GT	GT	GT	GT	TT
CT	CT	CT	CT	TT	CT	CC	CT	CT	CT	--	CT	TT
CG	CG	CG	CG	GG	CG	CC	CG	CG	CG	CG	CG	GG
GG	CG	CG	CG	GG	CG	--	CG	CG	CG	CG	CG	GG
CC	AC	AC	AC	CC	AC	AA	AC	AC	AC	AC	AC	CC
GG	CG	CG	CG	GG	CG	CG	CC	CG	CG	CG	CG	GG
TT	TT	AT	TT	AA	AT	TT	AT	TT	AA	AT	AT	AT
GG	GG	AG	GG	AA	AG	GG	AA	GG	--	AG	--	AG
GG	GG	AG	AG	AG	AA	--	AA	GG	AA	AG	--	AG
AA	AA	AG	AG	AG	GG	AA	GG	AA	GG	--	--	AG
GG	GG	AG	AG	AG	AA	GG	AG	GG	AA	AG	--	AG
CC	CC	AC	AC	AC	AA	CC	AC	CC	AA	AC	--	AC
GG	GG	AG	AG	AG	AA	GG	AG	GG	AA	AG	--	AG
GG	GG	AG	AG	AG	AA	GG	AG	GG	AA	AG	AA	AG
TT	TT	GT	GT	GT	GG	--	GT	TT	GG	--	--	GT
GG	--	GT	GT	GT	TT	--	--	GG	TT	GT	--	GT
AA	AA	AC	AC	AC	CC	AA	AC	AA	CC	AC	CC	AC
CC	--	CT	CT	CT	TT	--	CT	CC	TT	CC	--	CT
CC	CC	CT	CT	CT	TT	--	CT	CC	TT	--	--	CT
TT	TT	GT	GT	GT	GG	--	GT	TT	GG	GT	GG	GT
TT	TT	AT	AT	AT	AA	TT	AT	TT	AA	AT	--	AT
CC	CC	CG	CG	CG	GG	CG	CG	CC	GG	CG	GG	CG
CC	--	AC	AC	AC	AA	CC	AC	CC	AA	AC	--	AC
TT	TT	CT	CT	CT	CC	TT	CT	TT	CC	CT	CC	CT
CC	CC	CG	CG	CG	GG	--	CG	CC	GG	CG	GG	CG
TT	TT	CT	CT	CT	CC	TT	CT	TT	CC	--	--	CT
TT	--	AT	AT	AT	AA	TT	AT	TT	AA	AT	AA	AT
CC	CC	CT	CT	CT	TT	CC	CT	CC	TT	CT	TT	CT
AA	--	AG	AG	AG	GG	AA	AG	AA	GG	AG	GG	AG
TT	TT	TT	AT	AT	AA	TT	--	TT	AA	AT	--	AT
TT	TT	AT	AT	AT	AA	--	AT	TT	AA	--	--	AT
CC	CC	AC	AC	AC	AA	CC	AC	CC	AA	AC	AA	AC

GG	GG	GT	GT	GT	TT	GG	GT	GG	TT	GT	TT	GT
AA	AA	AG	AG	AG	GG	--	AG	AA	GG	--	GG	AG
TT	TT	GT	GT	GT	GG	TT	GT	TT	GG	GT	--	GT
GG	GG	AG	AG	AG	AA	GG	AG	GG	AA	AG	AA	AG
CC	--	CT	CT	CT	TT	--	CT	CC	TT	--	--	CT
GG	GG	AG	AG	AG	AA	GG	AG	GG	AA	GG	AA	AG
CC	CC	--	CT	CT	TT	--	CT	CC	TT	CC	--	CT
AA	AA	AC	AC	AC	CC	--	AC	AA	CC	--	--	AC
GG	--	AG	AG	AG	AA	--	AG	GG	AA	GG	--	--
CC	CC	CT	CT	CT	TT	CC	CT	CC	TT	CC	TT	CT
AA	AG	AG	AG	AG	GG	AA	AG	AA	GG	AA	--	AG
CT	CT	CT	CT	CT	CT	--	TT	CT	CC	TT	CT	CT
GG	GG	GG	GG	GG	GT	GT	GT	GT	GG	GT	--	GT
AG	AG	AG	AG	AG	GG	AG	AG	AG	GG	AG	GG	AG
AT	AT	TT	AT	AT	AT	--	AA	AA	TT	AA	AT	AA
AT	TT	TT	AT	AT	AT	AA	AA	AA	TT	AA	AT	AA
AC	CC	AC	AC	AC	AC	AA	AA	AA	CC	AC	AC	AA
GT	GG	GT	GT	GT	GT	--	TT	--	GT	GT	GT	TT
CT	TT	CT	CT	TT	CT	CT	CT	TT	TT	--	--	CT
AA	AG	AA	AA	GG	AG	AA	AG	AG	AG	AG	--	AA
GG	GG	GG	GG	CG	CG	GG	CG	GG	GG	CG	--	GG
TT	TT	TT	TT	CT	CT	TT	TT	TT	TT	CT	--	TT
GT	GT	GG	GG	GT	GG	GT	GG	GT	GT	--	GT	GG
AG	AA	AA	AA	GG	AG	AG	AA	AG	AG	AG	--	AA
AA	AA	AA	AA	AC	AA	--	AA	AA	AA	--	--	AA
GG	GG	GG	GG	CG	--	GG	GG	GG	GG	GG	--	GG
TT	TT	TT	TT	GT	TT	--	TT	--	TT	TT	--	TT
CT	CC	CT	CC	TT	CC	CT	CC	TT	CT	CT	CT	CC
GG	AA	AA	AG	AG	AG	--	AG	AG	--	GG	--	AG
CC	CT	CT	CC	CT	CC	CC	CC	--	--	CC	CT	CC
CG	--	CC	CC	CC	CG	CG	CG	CC	CG	CC	CG	CG
AA	AC	AC	AC	AA	AC	AA	AC	AC	AA	AA	--	AC
AG	AG	AG	AG	AA	GG	AG	GG	AG	AG	AA	AA	GG
CT	TT	TT	TT	CT	TT	CT	TT	TT	CT	TT	CT	TT
GG	CG	CG	CG	CG	GG	GG	GG	CG	GG	CG	CG	GG
AG	AG	AG	AG	GG	AA	AG	--	AG	AG	GG	GG	AA
CG	GG	GG	GG	GG	CG	CG	CG	GG	CG	GG	--	CG
CG	CG	CG	CG	GG	CC	--	CC	CG	CG	CG	--	CC
CT	CT	CT	CC	TT	CC	--	CC	--	CT	CT	--	CC
AG	AG	AG	GG	AA	GG	AG	GG	AG	AG	AG	--	GG
GG	AG	AG	AG	GG	AG	GG	AG	AG	GG	GG	--	AG
CT	CT	CC	CT	TT	CC	CT	CC	CT	CT	TT	--	CC
AG	GG	GG	GG	AG	GG	AG	GG	GG	AG	AG	AG	GG
AG	AG	GG	AG	AA	GG	AG	GG	AG	AG	AA	AA	GG
AC	AC	CC	AC	AA	CC	--	CC	AC	AC	--	--	CC
TT	AT	TT	AT	AT	TT	--	TT	AT	TT	AT	--	TT
AG	--	AA	AG	GG	AA	AG	AA	AG	AG	GG	--	AA

AC	AC	AA	AC	CC	AA	AC	AA	--	AC	CC	CC	AA
AG	AG	AA	AG	GG	AA	AG	AA	AG	AG	GG	--	AG
AG	AA	AA	AA	AG	AA	AG	AA	AA	AG	AG	AG	AA
CC	CT	CC	CT	--	CC	--	--	--	CC	CT	--	CT
CC	CG	CC	CG	CC	CC	CC	--	CG	CG	CG	CG	CG
TT	CT	TT	CT	TT	TT	TT	TT	CT	CT	--	--	CT
CG	GG	CG	GG	CG	GG	--	GG	GG	GG	GG	--	GG
CC	CT	CC	CT	CC	CT	CC	CT	CT	CT	CT	CT	CT
GT	--	GT	GG	GT	GG	GT	GG	GG	GG	GT	--	GG

5-47	5-48	5-52	5-53	5-55	5-56	5-58	5-60	5-61	5-63	5-64	5-66	5-68
AC	AA	AC	AC	AA	CC	AA	AC	AC	CC	AA	AC	CC
AG	AA	AG	AG	AA	--	AA	AG	AG	GG	AG	AG	GG
CT	TT	CT	CT	CT	CC	CT	CT	CT	CC	CT	CT	CC
GG	GG	AG	GG	GG	AG	GG	AG	GG	AG	AG	AG	AG
AG	AA	AG	AG	AG	--	--	AG	AG	GG	AG	AG	GG
GG	CG	CG	GG	GG	GG	GG	CG	GG	GG	--	CG	GG
CT	TT	CT	CT	CT	--	CT	CT	CT	CC	CT	CT	CC
GT	TT	GT	GT	GT	GG	GT	GT	GT	GG	GT	GT	GT
GT	TT	GT	GT	GT	GG	GT	GT	GT	GT	GT	GT	GT
GT	TT	GT	GT	GT	GG	--	GT	GT	GT	GT	GT	GT
AA	--	AG	AG	AG	--	AG	AG	AG	AG	GG	AG	AG
CT	CT	CT	CC	CC	--	--	CT	CC	CT	CC	CT	CC
AA	AA	AC	AC	CC	--	--	--	AC	AC	CC	AA	AC
AG	AG	AG	AA	AA	--	AA	AG	AA	AG	AA	AG	AA
CT	CT	CC	CT	CC	--	CC	CC	CT	CC	CC	CT	CT
GG	GG	AG	AG	AA	AG	AA	AG	AG	AA	AA	GG	AG
AT	--	AT	TT	TT	AT	--	AT	TT	TT	TT	TT	AT
CT	--	CC	CT	--	--	--	CC	CT	CC	CC	CT	CT
GT	GT	TT	TT	TT	GT	TT	GT	TT	TT	TT	TT	GT
CG	CC	CC	CC	CC	CG	CC	CG	CC	CC	CC	CC	CG
--	--	--	AT	TT	--	TT	AT	AT	TT	TT	AT	AA
CC	TT	--	CT	TT	--	--	--	CT	TT	TT	CT	CC
TT	AA	AA	AT	AA	AT	--	--	AT	AA	AA	AT	TT
TT	AA	AA	AT	AA	--	AA	AT	AT	AA	AA	AT	TT
--	AA	AA	AG	AA	AG	AA	AG	AG	AA	AA	AG	GG
GT	TT	--	TT	TT	--	--	GT	TT	TT	TT	TT	GT
GG	TT	TT	GT	TT	GT	TT	GT	GT	TT	TT	GT	GG
AA	--	TT	AT	TT	AT	TT	AT	AT	TT	TT	AT	AA
CC	TT	TT	CT	TT	CT	TT	CT	CT	TT	TT	CT	CC
GG	CC	CC	CG	CC	CG	CC	CG	CG	CC	CC	CG	GG
TT	CC	CC	CT	CC	CT	CC	CT	CT	CC	CC	CT	TT
CC	AA	AA	AC	AA	--	AA	AC	AC	AA	AA	AC	CC
GG	--	AA	AG	AA	AG	--	AG	AG	AA	AA	AG	GG
TT	GG	GG	GT	GG	--	GG	GT	GT	GG	GG	GT	TT
GG	--	AA	AG	AA	--	--	AG	AG	AA	AA	AG	GG
GG	TT	TT	GT	TT	--	--	GT	GT	TT	TT	GT	GG
AA	GG	GG	AG	GG	AG	GG	AG	AG	GG	GG	AG	AA
TT	CC	CC	CT	CC	--	--	CT	CT	CC	CC	CT	TT
TT	AA	AA	AT	AA	AT	AA	AT	AT	AA	AA	AT	TT
GG	AA	AA	AG	AA	AG	AA	AG	AG	AA	AA	AG	GG
CC	AA	AA	AC	AA	AC	AA	AC	AC	AA	AA	AC	CC
GG	AA	--	AG	AA	AG	--	AG	AG	--	AA	AG	GG

CC	TT	TT	CT	TT	--	CT	CT	CT	TT	TT	CT	CC
GG	CC	CC	CG	CC	CG	CC	CG	CG	CC	--	--	GG
CC	TT	TT	CT	TT	--	--	CT	CT	TT	TT	CT	CC
--	CC	CC	CT	CC	--	--	CT	CT	CC	CC	CT	TT
CC	AA	AA	AA	AA	AA	AA	AC	AA	AA	AA	AC	CC
TT	CC	CT	CC	CT	--	CT	CC	CC	CC	CC	CT	TT
CC	TT	CT	TT	CT	CT	CT	TT	TT	TT	CT	CT	CC
--	AG	GG	--	GG	GG	--	AG	GG	AG	GG	GG	AG
GG	GT	GG	GG	GG	GG	GG	GT	GG	GT	GG	GT	GT
GG	AG	GG	GG	GG	--	--	AG	GG	AG	AG	AG	AG
TT	CT	TT	TT	CT	TT	CT	CT	TT	CT	CT	CT	CT
CC	CG	--	CC	CG	--	CG	CG	CC	CG	CG	CC	CG
AA	AG	AA	AA	AG	AA	AG	AG	AA	AG	AG	AA	AG
CC	AC	CC	CC	AC	--	--	AC	CC	AC	AC	CC	AC
AG	AG	AG	AG	AG	AG	--	GG	AG	AG	GG	AG	AG
TT	TT	TT	TT	CT	TT	--	CC	TT	CT	CC	TT	TT
CC	CC	CT	CT	CT	--	CT	TT	CT	CT	TT	CC	CC
TT	TT	CT	CT	CT	--	CT	--	CT	CT	CC	TT	TT
AA	AA	AG	AG	AA	--	AA	GG	AG	AG	GG	AA	AA
AA	AA	AC	AC	AA	AA	AA	CC	AC	AC	CC	AA	AA
AA	AA	AG	GG	AA	AA	AA	GG	GG	AG	GG	AA	AA
GG	GG	CG	CC	GG	--	GG	CC	CC	CG	CC	GG	GG
CC	CC	CG	GG	CC	CC	--	GG	GG	CG	GG	CC	CC
AA	AA	AG	GG	AG	AA	AG	GG	GG	AG	GG	AA	AA
CC	CC	AC	AA	AC	AC	AC	AA	AA	AC	AA	CC	CC
CT	TT	CT	CC	CT	CT	--	CT	CC	CT	CC	TT	CT
CT	--	CT	TT	CT	--	CT	CT	TT	CC	TT	CC	CT
AT	TT	AA	AA	AT	--	AT	AT	AA	TT	AA	TT	AA
GG	GG	AG	AG	AG	AG	AG	GG	AG	GG	AG	GG	AG
AT	TT	--	AT	--	--	--	AT	AT	TT	TT	TT	AT
--	--	GT	--	GT	--	GT	GT	GG	GT	GG	GT	GT
AG	GG	GG	AG	GG	--	GG	GG	AG	GG	AG	AG	GG
CT	TT	TT	CT	TT	TT	TT	TT	CT	TT	CT	CT	TT
AG	GG	--	AG	GG	GG	GG	GG	AG	GG	AG	AG	GG
GT	GT	GT	TT	GT	GT	GT	GT	TT	GT	TT	GT	GT
GG	--	--	GG	--	--	--	GG	GT	GT	GT	GT	GG
CT	CC	CT	CT	CT	CC	--	CT	CT	CC	CC	CT	CT
--	--	CT	CT	CT	--	--	--	CT	CC	CC	CT	CT
AG	GG	AG	AG	GG	GG	GG	AG	AG	GG	GG	AG	AG
CG	CC	--	CG	CC	--	--	CG	CG	CC	CC	CG	CG
AG	GG	AG	AA	GG	--	GG	AG	AA	GG	GG	AG	AG
CT	TT	CT	CC	TT	--	--	CT	CC	TT	TT	CT	CT
TT	TT	CT	CC	TT	--	--	CT	CC	TT	TT	CT	CT
CG	CG	CC	CC	CG	CG	CG	CC	CC	CG	CG	CG	CC
--	--	TT	TT	AT	AT	AT	TT	TT	AT	TT	AT	TT
GT	GT	GT	GG	GG	GG	--	GT	GG	GG	GT	GG	GT
CT	CT	CT	CC	CC	CC	CC	CT	CC	CC	CT	CC	CT

TT	TT	CT	CC	CT	--	--	TT	CC	CT	CT	CC	CT
GT	GT	GT	GG	GG	--	GG	GT	GG	GG	GT	GG	GT
TT	TT	CT	CC	CT	CC	CT	TT	CC	CT	CT	CC	CT
GT	GT	GT	TT	TT	TT	TT	GT	TT	TT	GT	TT	GT
CG	CG	CG	GG	GG	GG	GG	CG	GG	GG	CG	GG	CG
AG	AG	AA	--	AA	--	--	GG	GG	GG	AA	AG	AG
AA	AG	GG	AA	GG	--	GG	AA	AA	AA	GG	AG	AG
AA	AG	AG	AG	GG	--	GG	AA	AG	AA	GG	AG	AG
TT	CT	CT	CT	CC	CT	CC	TT	CT	CT	CC	CT	CT
AG	AG	AG	AG	AA	AG	AA	GG	AG	AG	AG	AG	AG
GT	GT	GT	TT	TT	GT	TT	GG	TT	GT	GT	GT	TT
GG	GG	AG	GG	GG	GG	GG	GG	GG	GG	AG	GG	GG
CT	CT	CT	CC	CT	--	CT	CT	CC	CT	CT	CT	CT
CC	--	AC	AC	AC	--	AC	CC	AC	CC	AC	CC	--
CT	CT	CC	CC	CC	CT	CC	CT	CC	CT	CC	CT	CT
GT	--	GT	TT	GT	--	GT	GT	TT	GT	GT	GT	GT
CT	CT	CC	CT	CC	CT	--	CT	CT	CT	CC	CT	CT
GG	GG	CG	CG	CG	GG	CG	GG	CG	GG	CG	GG	GG
CC	CC	AC	AC	AC	--	AC	CC	AC	CC	AC	CC	CC
CT	CT	--	CT	--	CT	--	CT	CT	CT	TT	CT	CT
AG	AG	AA	AG	--	--	--	AG	AG	AG	AA	AG	AG
AG	AG	AG	GG	AG	--	AG	AG	GG	AG	AG	AG	AG
CT	CT	CC	CC	CC	--	CC	CT	CC	CT	CC	CT	CT
AA	AA	AT	--	AT	--	AT	AA	AT	AA	AT	AA	AA
CC	CC	CT	CT	CT	--	CT	CC	CT	CC	CT	CC	CC
AA	AA	AG	AG	AG	--	AG	AA	AG	AA	AG	AA	AA
CT	CT	CC	CT	CC	CT	CC	CC	CT	CT	CC	CT	CT
AT	--	AT	--	AT	--	--	AT	--	AT	AT	AT	AT
AT	AT	AT	AA	AT	--	--	TT	AA	AT	AT	--	AT
CT	CT	CT	CC	CT	CT	CT	TT	CC	CT	CT	CT	CT
CC	CC	CT	CT	CT	CC	CT	CC	CT	CC	CT	CC	CC
GT	GT	--	GT	GG	--	--	GG	GT	GT	GG	GT	GT
CT	CT	CC	CT	CC	CT	CC	CC	CT	CT	CC	CT	CT
AA	AA	--	AA	AG	--	--	AG	AA	AA	AG	AA	AA
AG	AG	AA	AA	AG	AG	AG	GG	AA	AG	AG	AG	--
AG	AG	AG	AG	AA	AG	AA	AA	AG	AG	AA	AG	AG
CT	CT	TT	TT	CT	CT	CT	CC	TT	CT	CT	CT	CT
AC	AC	AC	AC	CC	--	CC	CC	AC	AC	CC	AC	AC
AT	AT	AA	AA	AT	AT	AT	--	AA	AT	AT	AT	--
AA	AA	AG	AG	AG	--	--	AA	AG	AA	AG	AA	AA
AC	AC	AC	AC	AA	--	AA	AA	AC	AC	AA	AC	AC
AT	AT	AT	AT	TT	AT	TT	TT	AT	AT	TT	AT	AT
AA	AA	AC	AC	CC	AA	CC	AC	AC	AA	CC	AA	AA
CC	CC	AC	AC	AC	--	AC	CC	AC	CC	AC	CC	CC
GG	GG	GG	GG	AG	--	AG	AG	GG	AG	AG	GG	GG
AC	AC	CC	CC	CC	AC	CC	AC	CC	AC	CC	AC	AC
AG	AG	AG	AG	AA	AA	AA	AA	AG	AG	AA	AG	AG

CC	CC	CG	CG	CG	CC	CG	CC	CG	CC	CG	CC	CC
CT	CT	CC	CC	CT	CT	CT	TT	CC	CT	CT	CT	CT
CT	--	TT	TT	CT	CT	CT	CC	TT	CT	CT	CT	CT
--	CT	CT	CT	CC	CT	--	CC	CT	CT	CC	CT	CT
AG	AG	AG	AG	AA	AG	--	AA	AG	AG	AA	AG	AG
CT	--	TT	TT	CT	CT	CT	CC	TT	CT	CT	CT	CT
CT	CT	CT	CT	TT	CT	--	TT	CT	CT	TT	CT	CT
AT	AT	TT	--	AT	--	AT	AA	TT	AT	AT	AT	AT
GG	GG	AG	AG	AG	GG	AG	GG	AG	GG	AG	GG	GG
AC	--	AC	AC	AA	AC	AA	AA	AC	AC	AA	AC	AC
CT	CT	CT	CT	CC	CT	--	CC	CT	CT	CC	CT	CT
TT	TT	GT	GT	GT	TT	GT	TT	GT	TT	GT	TT	TT
CT	--	CT	CT	TT	CT	--	TT	CT	CT	TT	CT	CT
CC	CC	CC	CC	AC	CC	AC	AC	CC	CC	AC	CC	CC
AG	AG	AG	AG	AA	--	--	AA	AG	AG	AA	AG	AG
GT	GT	GT	GT	GG	GT	--	--	GT	GT	GG	GT	GT
TT	TT	TT	TT	AT	--	TT	AT	TT	TT	AT	TT	TT
AG	AG	AG	AG	AA	AG	--	AA	AG	AG	AA	AG	AG
CC	CC	CT	CT	CT	CC	--	CC	CT	CC	CT	CC	CC
CT	CT	CT	CT	CC	CT	CC	--	CT	CT	CC	CT	CT
AC	AC	AC	AC	AA	--	AA	AA	AC	AC	AA	AC	AC
CT	CT	CT	CT	CC	CT	CC	CC	CT	CT	CC	CC	CT
AT	--	TT	TT	--	--	AT	AA	TT	AT	AT	AT	AT
AG	AG	AA	AA	AG	AG	AG	GG	AA	AG	AG	GG	AG
--	GG	CG	CG	CG	--	CG	GG	CG	GG	CG	GG	GG
CT	CT	CT	CT	TT	CT	TT	TT	CT	CT	TT	TT	--
AG	AG	AG	AG	GG	AG	GG	GG	AG	AG	GG	GG	GG
AG	AG	AG	AG	--	--	--	AA	AG	AA	AA	AA	AA
AA	AA	AG	AG	AG	AA	AG	AA	AG	AA	AG	AA	AA
GG	GG	AG	AG	AG	--	--	GG	AG	GG	AG	GG	GG
TT	CT	TT	CT	TT	CT	--	--	CT	TT	--	TT	TT
GG	GT	GT	TT	GT	GT	GT	GG	TT	GG	GT	GG	GG
TT	TT	GT	GT	GT	--	GT	--	GT	TT	GT	TT	TT
GG	GG	AG	AA	GG	--	--	--	AA	GG	AG	GG	GG
CC	CC	CC	CT	CC	CC	CC	CC	CT	CC	CC	CC	CC
TT	TT	TT	GT	TT	TT	TT	TT	GT	TT	TT	TT	TT
AG	AG	GG	GG	GG	AG	GG	AG	GG	AG	GG	AG	AG
AA	AG	AA	AG	AG	GG	AG	GG	AG	AG	AG	GG	AG
--	CC	--	CT	CC	CC	CC	CC	CT	CC	CT	CC	CC
AA	AA	AA	AA	AA	AT	AA	AT	AA	AA	AT	AT	AA
CG	CG	CC	CG	CG	GG	CG	GG	CG	CG	CG	GG	CC
AC	AC	AA	AC	AC	--	--	AC	AC	AC	AA	AC	AA
AG	AG	AA	AG	AG	AG	AG	GG	AG	GG	AG	AG	AA
AG	AG	AG	AG	AG	GG	AG	GG	AG	GG	GG	GG	AG
CT	CT	CT	CT	CT	--	--	CC	CT	CC	CC	CC	CT
AG	GG	GG	AG	AG	AG	AG	AA	AG	AA	AG	AG	GG
CT	CC	CC	CT	CT	CC	CT	CT	CT	CT	CT	CC	CC

GG	GG	GG	GG	GG	AG	GG	AG	GG	AG	AG	AG	GG
GG	AA	AG	GG	GG	--	GG	AG	GG	AG	AG	AA	AG
CT	CT	CC	CT	CT	CT	CT	TT	CT	TT	TT	CT	CC
AT	AT	AT	AT	AT	TT	AT	TT	AT	TT	TT	TT	AA
AC	AC	AC	AC	AC	--	AC	CC	AC	CC	CC	CC	AA
CT	CT	CT	CT	CT	--	CT	CC	CT	CC	CC	CC	TT
AG	AG	--	AG	GG	--	--	AA	AG	AA	AA	AA	GG
CT	TT	CT	CT	CC	--	CC	TT	CT	TT	TT	TT	CC
CT	--	CT	--	TT	CT	--	CT	CT	CT	CT	CT	TT
GG	GG	AG	AG	AA	--	--	GG	AG	GG	GG	GG	AA
AG	--	AG	GG	AA	--	AA	AG	GG	GG	--	AA	GG
CT	--	CT	TT	CC	--	CC	CT	TT	TT	CT	CC	TT
AG	AG	AG	AG	GG	AG	GG	AG	AG	AG	GG	GG	AG
CT	TT	CT	CT	CC	--	CC	CT	CT	TT	TT	CC	CT
--	CG	GG	GG	GG	--	--	GG	GG	CG	CG	GG	--
CT	TT	CT	CC	--	--	--	CC	CC	TT	TT	CC	CT
CT	CT	CT	CC	CC	CT	--	CC	CC	CT	CT	CC	CT
AG	AG	AG	GG	AG	AA	AG	GG	GG	AA	AA	GG	GG
CT	CT	CT	CC	--	--	--	CC	CC	TT	TT	CC	CC
AG	AG	AG	AA	AG	GG	AG	AA	AA	GG	GG	AA	AA
CT	CT	CT	CC	CT	TT	CT	CC	CC	TT	TT	CC	CC
AG	AG	AG	GG	AG	AA	AG	GG	GG	AG	AA	GG	GG
AG	GG	AG	GG	GG	AG	GG	GG	GG	AG	AG	GG	GG
GG	GT	GT	TT	GT	GG	GT	TT	TT	GT	GT	TT	TT
CG	CC	CG	CC	CC	CG	CC	CC	CC	CG	CC	CC	CC
CC	--	CG	CG	--	CC	CC	CG	CG	CG	CG	CG	CG
CT	CT	TT	--	CT	--	--	CC	CC	CT	CC	CC	CC
CT	CT	CC	TT	CC	--	--	TT	TT	CT	TT	TT	TT
AT	AT	TT	AA	TT	--	TT	AA	AA	AT	AA	AA	AA
AG	AG	AA	GG	AA	AA	AA	GG	GG	AG	GG	GG	GG
CT	CT	TT	--	TT	TT	TT	CC	CC	CT	CC	CC	CC
CT	TT	CT	TT	CT	CT	CT	TT	TT	TT	TT	TT	TT
AC	AC	CC	AA	CC	--	CC	AA	AA	AC	AA	AA	AA
TT	--	GT	GT	GG	--	GG	GG	GT	TT	TT	GT	GT
AA	GG	AG	AG	GG	GG	GG	GG	AG	AA	AA	AG	AG
--	--	CT	CT	CC	CC	CC	CC	CT	TT	--	CT	CT
AA	TT	AT	AT	TT	--	--	TT	AT	AA	AA	AT	AT
CC	AA	AC	AC	AA	AA	AA	AA	AC	CC	CC	AC	AC
CG	CC	CG	CC	CC	--	CC	CC	CC	CG	CG	CG	CG
AG	--	AG	AA	AA	AA	--	AA	AA	AG	AG	AG	AG
CC	--	CT	TT	TT	--	TT	TT	TT	CC	CC	CT	CT
CC	TT	CT	TT	TT	--	--	TT	TT	CC	CC	CT	CT
AA	GG	AG	GG	GG	GG	GG	GG	GG	AA	AA	AG	AG
CT	CC	--	CC	CC	--	--	CC	CC	CT	CT	CC	CC
AA	--	AG	GG	--	GG	--	GG	GG	AA	AA	AG	AG
GG	AA	AG	AA	AA	AA	AA	AA	AA	GG	GG	AG	AG
GG	--	GT	TT	TT	--	TT	TT	TT	GG	GG	GT	GT

TT	CC	CT	CC	CC	--	CC	CC	CC	TT	TT	CT	CT
GG	CC	CG	CC	CC	CC	CC	CC	CC	GG	GG	CG	CG
TT	GG	GT	GG	GG	--	GG	GG	GG	TT	TT	GT	GT
AA	GG	AG	GG	GG	--	GG	GG	GG	AA	AA	AG	AG
CC	TT	CT	TT	TT	--	TT	TT	TT	CC	CC	CT	CT
GT	TT	TT	TT	TT	--	--	TT	TT	GT	GT	TT	TT
GG	CC	CG	CC	CC	CC	CC	CC	CC	GG	GG	CG	CG
AA	GG	--	GG	GG	--	GG	GG	GG	AA	AA	AG	GG
AG	GG	GG	GG	GG	--	GG	GG	GG	AG	AG	GG	GG
AA	CC	AC	CC	CC	CC	CC	CC	CC	AA	AA	AC	CC
CG	CC	CC	CC	CC	CC	CC	CC	CC	CG	CG	CC	CC
GG	AA	AG	AA	AA	--	--	AA	AA	GG	GG	AG	AA
--	--	AC	CC	CC	--	CC	CC	CC	AA	--	AC	CC
TT	GG	GT	GG	GG	GG	GG	GG	GG	TT	TT	GT	GG
CC	AA	AA	AA	AA	AA	AA	AA	AA	CC	CC	AC	AA
CT	--	--	CC	CC	--	CC	CC	CC	CT	CT	CC	CC
TT	GG	GG	GG	GG	GG	GG	GG	GG	TT	TT	GT	GG
AG	GG	GG	GG	GG	--	GG	GG	GG	AG	AG	AG	GG
CC	--	TT	TT	TT	TT	--	TT	TT	CC	CC	CT	--
CT	--	--	CC	CC	--	--	CC	CC	CT	CT	CC	CC
AA	TT	TT	TT	TT	--	TT	TT	TT	AA	AA	AA	TT
--	GG	GG	GG	GG	--	GG	GG	GG	CC	CC	CC	GG
GG	TT	TT	TT	TT	TT	TT	GT	TT	GG	GG	GT	TT
GG	GG	GG	GG	GG	GG	GG	GG	GG	AG	AG	GG	GG
TT	GT	GT	GT	GT	GT	GT	TT	GT	TT	TT	TT	GT
CT	TT	TT	TT	TT	--	TT	CT	TT	CC	CC	CT	TT
AC	AA	AA	AA	AA	AA	AA	AC	AA	CC	CC	AC	AA
CT	CT	CT	CC	CT	CT	CT	CT	CC	CC	CC	CT	CT
CG	--	CC	CG	CC	--	--	CG	CG	GG	GG	CG	CC
CT	CT	TT	CT	TT	--	--	CT	CT	CC	CC	CT	TT
TT	TT	TT	--	TT	--	TT	TT	GT	GT	GT	TT	TT
TT	CT	TT	TT	TT	--	--	--	TT	CT	CT	CT	CT
TT	TT	TT	--	TT	TT	TT	TT	AT	AT	--	TT	TT
AC	AC	AC	CC	AC	AC	AC	AC	CC	CC	CC	AC	AC
CC	CT	CT	CT	CC	--	CC	CT	CT	TT	TT	CC	CT
AG	AG	AG	AA	AG	AG	AG	AG	AA	AA	AA	AG	AG
AG	AG	AG	GG	AG	AG	AG	AG	GG	GG	GG	AG	AG
GG	CG	GG	CG	GG	CG	GG	GG	CG	CG	CG	GG	CG
CC	TT	CC	CT	CT	--	CT	CC	CT	CT	--	CT	TT
CC	TT	CC	CT	CT	CT	CT	CC	CT	CT	TT	CT	TT
CC	TT	CC	CT	CT	CT	CT	CC	CT	CT	CT	CT	TT
TT	--	TT	CC	CT	CT	CT	CT	CT	CT	CT	CT	CC
GG	TT	GT	GT	GT	GT	GT	GT	GT	GT	GT	GT	TT
CT	--	CT	TT	CT	CT	CT	CT	TT	CT	CT	CT	CT
AT	AA	AT	TT	AA	AT	AA	AA	TT	AT	AT	AT	AT
AG	--	AG	AG	AA	AG	AA	AA	AG	GG	AG	AG	AG
AC	CC	AC	AC	CC	AC	CC	CC	AC	AA	--	AC	AC

AG	AA	AG	AG	AA	AG	--	AA	AG	AG	AG	AG	AG
AG	AA	AG	AG	AA	--	--	AA	AG	AG	AG	AG	AG
CT	TT	CT	CT	TT	CT	TT	TT	CT	CT	CT	CT	CT
AG	GG	AG	GG	GG	AG	GG	GG	--	AG	AG	AG	AG
GT	--	GT	GT	GG	GT	--	GG	GT	GT	GT	GT	GT
CT	--	CT	CT	TT	--	TT	TT	CT	CT	CT	CT	CT
AG	GG	--	AG	GG	--	--	GG	AG	AG	AG	AG	AG
AG	GG	AG	AG	GG	--	--	GG	AG	AG	AG	AG	AG
AG	AA	AG	AG	AA	--	--	AA	AG	AG	AG	AG	AG
CG	GG	CG	CG	GG	--	--	GG	CG	CG	CG	CG	CG
AG	AA	AG	AG	AA	AG	--	AA	AG	AG	AG	AG	AG
AC	AA	AC	AC	AA	--	AA	AC	AC	AC	AC	AC	AC
AG	GG	GG	AG	GG	AG	GG	GG	AG	AG	AG	AG	AG
AG	AG	AG	AG	GG	--	--	GG	AG	AG	AG	AG	AG
AT	AT	AT	AT	AA	AT	AA	AA	AT	AT	AT	AT	AT
CT	CT	CT	CT	TT	CT	TT	TT	CT	CT	CT	CT	CT
CT	CT	CT	CT	CT	--	CT	TT	CT	CT	CT	CT	CT
CG	CG	CG	CG	CG	--	CG	CC	CG	CG	CG	CG	CG
CT	CT	CT	CT	CT	CT	CT	CC	CT	CT	CT	CT	CT
CT	CT	CT	CT	--	CT	CT	TT	CT	CT	CT	CT	CT
CT	CT	CT	CT	CT	CT	CT	CC	CT	CT	CT	CT	CT
AT	AT	AT	AT	AT	AT	AT	AA	AT	AT	AT	AT	AT
AT	--	AT	AT	AT	AT	AT	TT	AT	AT	AT	AT	AT
CT	CT	CT	CC	CT	CT	CT	CC	CT	CT	CT	CT	CT
CT	CT	CT	CT	CT	CT	CT	TT	CT	CT	CT	CT	CT
AT	AT	AT	AT	AT	AT	AT	TT	AT	AT	AT	AT	AT
AG	AG	AG	AG	AG	AG	AG	--	AG	AG	AG	AG	AG
GT	GT	GT	GT	GT	GT	GT	TT	GT	GT	GT	GT	GT
CT	CT	CT	CT	CT	--	CT	CC	CT	CT	CT	--	CT
CG	CG	CG	CG	CG	--	CG	GG	CG	CG	CG	CG	CG
AG	AG	AG	AG	AG	AG	AA	AA	AG	AG	AG	AG	AG
CT	CT	TT	CT	CT	CT	--	--	CT	CT	CT	CT	CT
CT	CT	TT	CT	CT	--	CT	CC	CT	CT	CT	CT	CT
AT	AT	TT	AT	AT	AT	AT	AA	AT	AT	AT	AT	AT
AG	AG	AA	AG	AG	--	AG	GG	AG	AG	AG	AG	--
AG	AG	AG	AG	AG	--	AG	GG	AG	GG	AG	AG	AG
GT	GT	GT	GT	GT	GT	GT	GT	GT	GG	GT	GT	GT
AG	AG	AG	AG	AG	--	AG	AG	AG	GG	AG	AG	AG
GG	GG	GG	GG	GG	--	--	GG	GG	AG	GG	GG	GG
CT	TT	CT	CT	CT	TT	CT	TT	CT	CT	CT	CT	TT
AG	AG	AG	AA	AA	--	AA	GG	AA	AG	AG	AG	AG
GG	AG	GG	AG	AG	GG	AG	GG	AG	GG	GG	GG	AG
AG	--	AG	AA	AA	--	AA	GG	AA	AG	AG	AG	AG
CG	CC	CG	GG	GG	CC	GG	CG	GG	CG	--	CG	CC
CT	TT	CC	CC	CC	--	CC	CT	CC	CT	CC	CT	TT
CC	AC	CC	CC	CC	AC	CC	AC	CC	CC	CC	--	AC
AA	AG	--	--	AA	AG	--	AA	AA	AA	AA	AA	AG

CC	AC	CC	CC	CC	--	--	AC	CC	CC	CC	CC	AC
AC	CC	AC	AC	AC	--	AC	AC	AC	AC	AC	AC	CC
TT	CT	TT	TT	TT	CT	TT	CT	TT	TT	TT	TT	CT
AA	GG	AA	AA	AA	GG	AA	AG	AA	AA	AA	AA	GG
GG	AA	GG	GG	--	AG	GG	AG	GG	GG	GG	GG	AA
--	--	TT	TT	TT	TT	TT	CT	TT	TT	TT	TT	CC
CT	TT	CT	CT	CT	CT	CT	CT	CT	CT	CT	TT	TT
CC	GG	CC	CC	CC	--	--	CG	CC	CC	CC	GG	GG
AA	GG	AA	AA	AA	AA	AA	AG	AA	AG	AA	GG	GG
TT	--	TT	TT	TT	TT	TT	CT	TT	CT	TT	CC	CC
AT	AA	AT	AT	AT	AT	AT	AT	AT	AA	--	AA	AA
CC	AA	CC	CC	CC	--	CC	AC	CC	AA	CC	AA	AA
GG	AG	GG	--	GG	--	GG	AG	GG	AG	GG	AG	AG
CC	CT	CC	CC	CC	--	CC	CT	CC	CT	CC	CT	CT
CC	CT	CT	CC	CC	--	CC	CC	CC	CT	CC	CT	CT
GG	GT	GG	GG	GG	--	GG	GG	GG	GT	GG	GT	GT
TT	TT	CT	CT	CT	CC	CT	CT	CT	CT	CT	CT	TT
GG	GG	GG	GG	AG	AG	AG	GG	GG	GG	GG	GG	GG
GG	AG	AG	AG	AG	--	AA	AG	AG	AG	AG	AG	GG
CG	--	GG	GG	CG	--	CG	GG	GG	GG	GG	GG	CG
GG	AG	AG	AG	AG	AA	AG	AG	AG	AG	AG	AG	GG
AG	AG	AG	AG	AG	--	AG	AG	AG	AG	AG	AG	AA
GT	TT	GT	TT	TT	TT	TT	GT	TT	GT	GT	GT	TT
AG	AA	AG	AA	AG	AG	AG	AG	AA	AG	AG	AG	AA
AG	AA	AG	AA	AG	--	AG	AG	AA	AG	AG	AA	AA
GT	GG	GG	GG	GG	--	GG	GT	GG	GT	GT	GG	GT
CT	CC	CC	CC	CT	CT	--	--	--	CT	CT	CC	TT
CT	CT	--	TT	CT	--	CT	CT	TT	CT	CT	TT	CC
CC	CC	CC	CC	CT	CT	CT	CC	CC	CT	CC	CC	CT
AG	AG	AA	AA	AG	GG	AG	AG	AA	AG	AG	AA	GG
CT	CT	CT	CC	TT	TT	TT	CT	CC	CT	CT	CC	TT
AA	AG	AG	AA	GG	GG	GG	AG	AA	AG	AG	AA	GG
CT	CT	CT	CC	TT	TT	TT	CT	CC	CT	CT	CC	TT
CG	CG	CG	GG	--	--	CG	CG	GG	CG	CG	--	CC
CT	CT	CT	CC	CT	--	CT	CT	CC	CT	CT	CC	TT
AC	AC	--	AA	AC	AC	--	AA	AA	AC	AC	AA	CC
AG	AA	GG	GG	AG	AG	AG	GG	GG	AG	AA	GG	AA
CT	CC	TT	TT	CT	CT	CT	TT	TT	CT	CC	TT	CC
AG	GG	AA	AA	AG	--	--	AA	AA	AG	GG	AA	GG
GG	--	AA	AG	AA	--	AA	AA	AG	AG	GG	AA	GG
CT	CT	TT	TT	TT	CT	--	TT	TT	TT	CT	TT	CT
--	--	AG	AG	GG	--	--	GG	AG	AG	AA	AG	AA
AG	--	--	AG	AA	AG	AA	AA	AG	AG	AG	AG	AA
GT	TT	GT	GT	TT	GT	TT	TT	GT	GT	GT	GT	TT
GT	GT	GT	GT	TT	GT	TT	TT	GT	GT	GT	GT	--
CC	--	AC	AC	AA	--	AA	AA	AC	AC	CC	AC	AC
GG	AG	AG	AG	AG	--	--	--	AG	AG	GG	AG	AG

GG	CG	CG	CG	CG	GG	CG	CC	CG	CG	GG	CG	CG
GG	CG	CG	CG	CG	--	--	CC	CG	CG	CG	CG	CG
CC	CT	CT	CT	CT	--	CT	TT	CT	CT	CT	CT	CT
TT	CT	CT	TT	CT	TT	CT	CC	TT	CC	CT	CT	CT
TT	AT	AT	TT	AT	--	AT	AA	TT	AA	AT	AT	TT
GG	AG	GG	GG	AG	GG	AG	AG	GG	AG	AG	GG	GG
TT	CT	CT	TT	CT	TT	CT	CC	TT	CC	CT	CT	TT
CC	CT	TT	--	CT	--	CT	TT	CC	TT	TT	CT	CC
TT	CT	CC	TT	CT	--	--	CC	TT	CC	CC	CT	TT
AA	AG	GG	AA	AG	--	AG	GG	AA	GG	GG	AG	AA
CG	CG	CC	CG	CG	CG	CG	CC	CG	CC	CC	CC	CG
CG	GG	GG	CG	GG	--	--	GG	CG	GG	GG	CG	CG
GG	GT	GG	GT	GT	GT	GT	GG	GT	GG	GG	GG	GT
--	CG	CG	CC	CC	CG	CC	CG	CC	CC	CC	CC	CG
AG	AA	AG	GG	AG	--	AG	AG	GG	GG	GG	GG	AG
AA	AC	AC	AA	AA	AC	AA	AC	AA	AA	AA	AA	AC
AA	AT	AT	AA	AA	AT	AA	AT	AA	AA	AA	AA	AT
AG	AA	AG	GG	AG	--	AG	AG	GG	AG	GG	AG	AG
AA	TT	AT	AT	AT	--	AT	TT	AT	AT	AA	AT	AT
GG	AA	AA	AG	AG	AG	AG	AA	AG	AG	GG	AG	AG
CC	AC	AC	AC	AC	CC	AC	--	AC	CC	CC	AC	CC
CC	CT	TT	CT	CT	TT	CT	TT	CT	CT	CT	CT	CT
TT	GT	GG	GG	GT	GG	GT	GG	GG	GT	GT	GG	GT
--	GT	GT	GG	GT	--	--	GG	GG	GT	GT	GG	GT
CC	CT	CT	TT	CT	TT	CT	TT	TT	CT	CT	TT	CT
CC	CG	CG	GG	CG	--	CG	GG	GG	CG	CG	GG	CG
GG	AG	--	--	AG	--	AG	AA	AA	AG	AG	AA	AG
CC	--	CT	TT	CT	--	CT	TT	TT	CT	CT	TT	CT
TT	GT	GT	GG	GT	GG	GT	GG	GG	GT	GT	GG	GT
TT	CT	CT	CC	CT	CC	CT	CC	CC	CT	CT	CC	CT
GG	CG	CG	CC	CG	CC	CG	CC	CC	CG	CG	CC	CG
GG	AG	AG	AA	AG	AA	AG	AA	AA	AG	AG	AA	AG
AC	AC	AC	CC	AC	--	AC	CC	CC	AC	AC	CC	AC
CT	CT	CT	--	CT	--	--	CC	CC	CT	CT	CC	CT
AG	AG	GG	AG	AG	--	--	AG	AG	AG	AG	AG	AG
AC	--	AC	CC	CC	CC	--	CC	CC	AC	AC	CC	AC
CT	CT	CT	TT	TT	TT	TT	TT	TT	CT	CT	TT	CT
AG	AG	AG	AA	AA	AG	AA	AA	AA	AG	AG	AA	AG
CG	CG	CG	CC	CC	--	--	CC	CC	CG	CG	CC	CG
GT	GT	GT	TT	--	GT	--	TT	TT	GT	GT	TT	GT
CT	CT	CT	TT	TT	CT	TT	TT	TT	CT	CT	TT	CT
CT	CT	TT	CT	CT	TT	CT	CT	CT	CT	CT	CT	CT
AG	AG	GG	GG	GG	--	--	GG	GG	AG	AG	AG	AG
CG	CG	CG	GG	GG	CG	GG	GG	GG	CG	CG	CG	CG
AA	AA	AT	AT	AT	AT	AT	AT	AT	AA	AA	AA	AA
AG	AG	AA	AG	AG	AA	AG	AG	AG	AG	AG	AG	AG
AC	AC	CC	AC	AC	CC	AC	AC	AC	AC	AC	AC	AC

CT	CT	TT	CT	CT	TT	CT	CT	CT	CT	CT	CT	CT
TT	TT	CT	CT	TT	CT	TT	CT	CT	TT	TT	TT	TT
GT	AT	GG	GT	GT	AG	GT	AT	GT	AT	GT	GT	GT
AT	AT	AA	AT	AT	AA	AT	AT	AT	AT	AT	AT	AT
AG	AA	GG	AG	AG	AG	AG	AA	AG	AA	AG	AG	AA
AC	AA	--	AC	AC	AC	--	--	AC	AA	AC	AC	AA
AG	AG	AA	AG	AG	AA	AG	AG	AG	AG	AG	AG	AG
AG	AA	GG	AG	AG	AG	AG	AA	AG	AA	AG	AG	AA
AG	GG	AA	AG	AG	AG	AG	GG	AG	GG	AG	GG	GG
AG	--	GG	AG	AG	--	AG	--	AG	AA	AG	AA	AG
GT	TT	GG	TT	--	--	GT	TT	TT	TT	GT	TT	GT
GG	AG	GG	AG	GG	AG	--	AG	AG	AG	GG	AG	GG
CT	--	CC	TT	--	CT	CT	--	TT	TT	CT	TT	CT
CG	CG	CC	GG	CG	CC	CG	GG	GG	GG	CG	GG	CG
CT	CT	TT	CT	CT	--	CT	CT	CT	CT	CT	CT	CT
TT	TT	TT	CT	CT	TT	CT	CT	CT	TT	TT	CT	TT
CC	CC	CT	CT	CT	CT	CT	CT	CT	CT	CC	CC	CT
GT	GG	GG	GT	GG	GT	GG	GG	GT	GG	GT	GT	GT
AG	GG	AG	AA	AG	--	AG	AG	AA	AG	AG	AG	AA
AG	AA	AG	GG	AG	--	AG	AG	GG	AG	AG	AG	GG
AA	AA	AG	AG	AG	--	--	AG	AG	AG	AA	AA	--
GT	GG	GT	TT	GT	GT	GT	GT	TT	GT	TT	GT	TT
AA	AA	AG	AG	AG	AG	AG	AG	AG	AG	AG	AA	AG
CC	--	--	CT	CT	CT	CT	CT	CT	CT	CT	CC	CT
AG	AG	AA	AA	AA	AA	AA	AA	AA	AA	AA	AA	AA
--	GG	AG	AA	AA	--	AA	AG	AA	AG	AA	AA	AG
GG	GG	GG	AA	AA	AG	AA	AG	AA	AG	AA	AA	AG
GG	GG	GG	CC	CC	CG	CC	CG	CC	CG	CC	CC	CG
GG	--	GG	CC	CC	CG	CC	CC	CC	CG	CC	CC	CG
GG	GG	GG	AA	AA	AA	AA	AA	AA	AG	AA	AA	AG
GG	GG	GG	AG	AG	AG	AG	AG	AG	--	AG	AG	GG
TT	--	TT	CC	CC	--	CC	CC	CC	CT	CC	CC	CT
CC	CC	--	AC	AC	--	--	AC	AC	CC	CC	AC	CC
CC	CC	CC	CG	CG	--	--	CG	CG	CC	CC	CG	--
CC	CC	CC	AC	AC	CC	AC	AC	AC	CC	CC	AC	CC
AA	AA	AC	CC	CC	--	CC	CC	CC	AC	AC	CC	AC
AA	AA	AA	AG	AG	AG	AG	AG	AG	AG	AG	AG	AG
AA	AG	AG	AG	GG	AG	GG	GG	AG	AG	AG	GG	AG
GG	GG	GG	GG	AG	AG	AG	AG	GG	AG	AG	AG	AG
CC	CT	CT	CT	CT	CT	--	CT	CT	CT	CT	CT	TT
CC	CT	CT	CT	CT	--	CT	CT	CT	CT	CT	CT	TT
AA	--	AG	AG	AA	--	AA	AA	AG	AA	AA	AA	AG
CC	CT	CT	CT	CC	--	--	CC	--	CC	CC	CC	CT
AG	AG	AG	GG	--	GG	--	AG	GG	GG	AG	AG	--
CC	AC	AC	CC	AC	--	--	CC	CC	CC	AC	CC	AC
CT	CT	CC	CC	CT	CC	CT	CT	CC	CC	CC	CT	CT
--	--	AG	--	GG	--	--	AG	AA	AA	AA	AG	GG

AG	AG	AA	AA	AG	AA	AG	AG	AA	AA	AA	AG	AG
GG	GG	AG	AA	GG	AG	GG	AA	AA	AA	AA	GG	GG
AA	AC	CC	CC	AA	AC	AA	AC	CC	CC	CC	AA	AA
AA	AG	GG	GG	AG	AG	AG	AG	GG	GG	GG	AA	AA
--	GT	GT	TT	GT	GT	--	GT	TT	TT	TT	--	--
AG	AA	AA	AA	AA	AG	AA	AG	AA	AA	AA	AG	AA
CC	CT	CT	TT	CT	--	CT	CT	TT	TT	TT	CC	CT
CT	CC	CC	CC	CC	CC	CC	CT	CC	CC	CC	CT	CC
AA	AG	AG	GG	AG	--	AG	AG	GG	GG	GG	AA	AG
CG	CG	CG	GG	CG	CG	CG	GG	GG	GG	GG	CG	GG
--	CT	CT	CT	CT	--	--	--	CT	TT	TT	CT	TT
GG	AG	AG	GG	AA	--	--	--	GG	AA	AG	GG	AA
GG	AG	AG	GG	AA	AG	AA	AG	GG	AA	AG	GG	AA
GG	CG	--	GG	CG	--	CG	CG	GG	CC	CG	GG	CC
GG	AG	AG	GG	AG	GG	AG	AG	GG	AA	--	GG	AA
AG	AG	GG	AG	GG	AG	GG	AG	AG	GG	AG	AG	--
CT	CT	TT	CT	TT	CT	TT	CT	CT	TT	CT	CT	TT
AG	AG	GG	AG	GG	AG	--	AG	AG	GG	AG	AG	GG
CG	CG	CC	CG	CC	CG	CC	CG	CG	CC	CG	CG	CC
AC	AC	CC	AC	CC	AC	--	AC	AC	--	AC	AC	CC
AG	AG	AA	AG	AG	--	AG	AG	AG	AA	AG	AG	AA
GT	GT	GG	GT	GT	--	GT	GT	GT	GG	GT	GT	GG
AG	AG	GG	AG	AG	--	AG	AG	AG	GG	AG	AG	GG
AG	AG	GG	AG	AG	--	AG	AG	AG	GG	AG	AG	GG
AG	AG	AA	AG	AG	AG	AG	AG	AG	AA	AG	AG	AA
AC	AC	AA	AC	AC	AC	AC	AC	AC	AA	AC	AC	AA
AG	AG	AA	AG	AG	AG	AG	AG	AG	AA	AG	AG	AA
AG	AG	AA	AG	AG	AG	AG	AG	AG	AA	AG	AG	AA
CT	CT	TT	CT	CT	CT	CT	CT	CT	TT	CT	CT	TT
CG	CG	GG	CG	CG	CG	CG	CG	CG	GG	CG	CG	GG
AC	AC	--	AC	AC	--	AC	AC	AC	AA	AC	AC	AA
CT	CT	TT	CT	CT	--	CT	CT	CT	TT	CT	CT	TT
GT	GT	TT	GT	GT	GT	GT	GT	GT	GT	GT	GT	TT
CT	CT	CC	CT	CT	--	CT	CT	CT	CT	TT	CT	CC
CG	CG	CC	CG	CG	CG	CG	CG	CG	CG	CG	CG	CC
AT	AT	TT	AT	AT	--	AT	AT	AT	AT	AT	AT	TT
CT	CT	CC	CT	CT	CT	CT	CT	CT	CT	CT	CT	CC
AG	GG	GG	AG	AG	--	--	AG	AG	AG	AG	AG	GG
AG	AG	AA	AG	AG	AG	AG	AG	AG	AG	AG	AG	AA
AT	AT	AA	AT	AT	AT	AT	AT	AT	AT	AT	AT	AA
AG	AG	GG	AG	AG	--	AG	AG	AG	AG	AG	AG	GG
AG	AG	AA	AG	AG	--	AG	AG	AG	AG	AG	AG	AA
CG	CG	CC	CG	CG	--	CG	CG	CG	CG	CG	CG	CC
AG	AG	AA	AG	AG	--	AG	AG	AG	AG	AG	AG	AA
CG	CG	GG	CG	CG	CG	CG	CG	CG	CG	CG	CG	GG
AG	AG	GG	AG	AG	--	AG	AG	AG	AG	AG	AG	GG
AC	AC	AA	AC	AC	--	AC	AC	AC	AC	AC	AC	AA

AG	AG	GG	AG	AG	--	AG	AG	AG	AG	AG	AG	GG
CG	CG	CC	CG	--	CG	CG	CC	CG	CG	CG	CG	CC
CT	CT	--	CT	CT	--	--	CT	CT	CT	CT	CT	CC
AT	AT	TT	AT	AT	AT	AT	AT	AT	AT	AT	AT	TT
CT	CT	CC	CT	CT	--	--	CT	CT	CT	CT	CT	CC
AT	AT	AA	AT	AT	--	AT	AT	AT	AT	AT	AT	AA
AT	AT	TT	AT	AT	AT	AT	AT	AT	AT	AT	AT	TT
CG	CG	GG	CG	CG	CG	CG	CG	CG	CG	CG	CG	GG
CT	CT	CC	CT	CT	CT	CT	CT	CT	CT	CT	CT	CC
AG	AG	AA	AG	AG	AG	AG	AG	AG	AG	AG	AG	AA
AC	AC	AA	AC	AC	--	AC	AC	AC	AC	AC	AC	AA
GT	GT	TT	GT	GT	GT	GT	GT	GT	GT	GT	GT	TT
CT	CT	CC	CT	CT	CT	CT	CT	CT	CT	CT	CT	CC
CG	CG	GG	CG	CG	CG	CG	CG	CG	CG	CG	CG	GG
GT	GT	TT	GT	GT	GT	--	GT	GT	GT	GT	GT	TT
CT	CT	TT	CT	CT	CT	CT	CT	CT	CT	CT	CT	TT
--	AG	AA	AG	AG	AG	AG	GG	AG	AG	AG	AG	AA
AC	AC	AA	AC	AC	AC	--	--	AC	AC	AC	AC	AA
CT	CT	CC	CT	CT	--	CT	TT	CT	CT	CT	CT	CC
AC	AC	AA	AC	AC	AC	--	CC	AC	AC	AC	AC	AA
AG	AG	AA	AG	AG	AG	AG	GG	AG	AG	AG	AG	AA
GT	--	GG	GT	GT	GT	GT	TT	GT	GT	GT	GT	GG
GT	GT	GG	GT	GT	GT	--	--	GT	GT	GT	GT	GG
AG	AG	GG	AG	AG	AG	AG	AA	AG	AG	AG	AG	GG
CT	CT	CC	CT	CT	CT	CT	TT	CT	CT	CT	CT	CC
CT	CT	CC	CT	CT	CT	--	TT	CT	CT	CT	CT	CC
AG	AG	AA	AG	AG	--	--	GG	AG	AG	AG	AG	AA
CT	CT	CC	CT	CT	CT	CT	TT	CT	CT	CT	CT	CC
CT	CT	TT	CT	CT	CT	CT	CC	CT	CT	CT	CT	TT
AG	AG	GG	AG	AG	AG	AG	AA	AG	AG	AG	AG	GG
CT	CT	--	CT	CT	CT	CT	TT	CT	CT	CT	CT	CC
CT	TT	TT	CT	CT	CT	--	CT	CT	CT	CT	CT	CT
--	CT	CC	CT	CT	--	--	CT	CT	CT	CT	CT	CT
GG	AG	AG	AG	AG	AG	AG	AG	AG	AG	AG	AG	AG
CC	AC	AC	AC	AC	AC	AC	AC	AC	AC	AC	AC	AC
CC	CC	CG	CG	CG	CG	CG	CG	CG	CG	CG	CC	CG
GT	GT	GT	GT	GT	GT	GT	GT	GT	GT	GT	TT	GT
AG	AG	AG	AA	AG	AG	--	AG	AA	AG	GG	AA	AG
CT	CT	CC	CC	CT	--	CT	CT	CC	CT	TT	CC	CT
AG	GG	AG	GG	AG	--	AG	AG	GG	AG	AG	GG	AG
AG	AG	AG	AA	AG	AG	--	AG	AA	AG	GG	AA	AG
CG	CG	CG	--	CG	CG	CG	CG	CC	CG	GG	CC	CG
CT	TT	--	TT	CT	CT	CT	CT	TT	CT	CT	TT	CT
GT	GG	TT	GG	GT	GT	GT	GT	GG	GT	TT	GG	GT
AG	AG	AG	--	GG	--	--	GG	AG	GG	AG	AG	GG
GG	GG	AG	AG	AG	AG	AG	GG	AG	AG	GG	AG	GG
AA	AC	CC	CC	AC	--	AC	AA	CC	AC	AC	CC	AA

TT	AT	AA	AA	AT	--	AT	TT	AA	AT	AT	AT	TT
TT	AT	AA	AT	AT	--	AT	TT	AT	AT	AT	AT	TT
AA	AA	TT	AT	AT	TT	AT	AA	AT	AT	AT	AT	AA
TT	TT	CC	CT	CT	--	--	CT	CT	CT	CT	CT	TT
--	GG	AA	AG	AG	AA	AG	AG	AG	AG	AG	AG	GG
AA	AA	CC	AC	AC	CC	AC	AC	AC	AC	AC	AC	AA
TT	TT	--	CT	CT	CC	CT	CT	CT	CT	CT	--	TT
GG	GG	AA	AG	AG	--	AG	AG	AG	AG	AA	AG	GG
GG	GG	TT	GT	GT	TT	GT	GT	GT	GT	TT	GT	GG
--	--	--	AG	AG	--	--	AG	AG	GG	AG	AG	GG
CC	CC	CG	CG	CC	CG	CC	CC	CG	CC	CC	CG	CC
GG	GG	AG	AG	GG	AG	GG	GG	AG	GG	GG	AG	GG
CC	CC	GG	CG	CG	GG	CG	CG	CG	CC	CG	GG	CG
AA	AA	--	AG	AG	GG	AG	AG	AG	AA	AG	GG	AG
--	GG	AA	--	AG	--	--	AG	AG	GG	AG	AA	AG
AA	AA	GG	AG	AG	GG	AG	AG	AG	AA	AG	GG	AG
GG	GG	GT	GT	GG	GT	GG	GG	GT	GG	GG	GT	GG
GG	GG	AA	AG	AG	AA	AG	AG	AG	GG	AG	AA	AG
TT	TT	GT	GT	TT	GT	--	TT	GT	TT	TT	GT	TT
--	--	CC	CG	GG	--	--	CG	CG	GG	CG	CC	CG
TT	TT	CT	CT	CT	TT	CT	TT	CT	--	CT	TT	TT
CG	GG	CG	CG	--	GG	GG	CG	CG	GG	CG	GG	CG
CG	--	CC	CC	CC	--	CC	CC	CC	CC	CC	CC	CG
AA	AA	AC	AC	AA	AA	AA	AA	AC	AA	AC	AA	AA
CC	--	--	CC	CC	CT	--	CC	CC	--	CT	CC	CC
--	--	AG	AA	--	--	--	AA	AA	AA	AG	AA	AA
CC	CC	CT	CC	CC	CT	CC	CC	CC	CC	CT	CC	CT
CG	CG	GG	GG	CG	--	CG	GG	GG	GG	CG	GG	GG
--	--	CT	CC	CC	CT	CC	CC	CC	CC	CT	CC	CT
AG	GG	AG	AA	AG	AG	AG	AA	AA	AA	GG	AA	AG
GG	GT	GT	GG	GG	GT	GG	GG	GG	GG	GT	GG	GT
GT	GG	GT	TT	GT	--	GT	TT	TT	TT	GG	TT	GT
GG	CG	CG	GG	CG	--	CG	GG	GG	GG	CG	GG	CG
CG	CC	CG	GG	CC	CG	CC	GG	GG	GG	CC	CG	CG
GG	AG	AG	GG	AG	--	AG	GG	GG	GG	AG	AG	AG
CT	--	--	TT	CC	CT	--	--	TT	TT	CC	CT	CT
GT	TT	GT	GG	TT	--	--	GG	GG	GG	TT	GT	GT
GG	AG	AG	GG	AG	AG	AG	GG	GG	GG	AG	AG	AG
AG	--	AG	AA	GG	--	GG	AA	AA	--	GG	AG	AG
CT	--	CT	TT	CC	CT	CC	TT	TT	TT	CC	CT	CT
--	AT	AT	--	AT	AT	AT	TT	TT	TT	AT	AT	AT
TT	GT	GT	TT	GT	--	GT	TT	TT	TT	GT	GT	GT
--	TT	CT	CC	TT	--	--	CC	CC	--	TT	CT	CT
AA	AG	AG	AA	AG	AG	AG	AA	AA	AA	AG	AG	AG
AT	AA	AT	AT	AA	--	AA	TT	AT	TT	AA	AT	AT
AA	AG	AG	AA	AG	AG	--	AA	AA	AA	AG	AG	AG
TT	CT	CT	TT	CT	CT	CT	TT	TT	TT	CT	CT	CT

TT	--	AT	TT	AT	AT	AT	TT	TT	TT	AT	AT	AT
GT	TT	GT	GT	TT	GT	TT	GG	GT	GG	TT	GT	GT
AG	GG	AG	AG	GG	AG	GG	AG	AG	AA	GG	AG	AG
CT	--	CT	CT	TT	--	--	CT	CT	CC	TT	CT	CT
CT	TT	CT	--	TT	CT	TT	CT	CT	CC	TT	CT	CT
TT	TT	CT	--	TT	CT	--	CT	CT	CC	TT	CT	CT
TT	TT	GT	GT	TT	GT	--	GT	GT	GG	TT	GT	GT
CG	GG	CG	CG	GG	--	GG	CC	CG	CC	GG	CG	CG
AC	AA	AC	AA	AC	CC	AC	CC	AA	CC	AA	AC	AC
AG	GG	AG	--	AG	AA	AG	AA	GG	AA	GG	AG	AG
AG	AA	AG	AA	AG	--	AG	GG	AA	GG	AA	GG	AG
TT	TT	CT	CC	CC	CC	CC	CT	CC	CT	TT	CT	CC
AA	AA	AC	CC	AC	CC	AC	AC	CC	AC	AA	AC	CC
TT	TT	GT	GT	TT	GT	TT	TT	GT	GT	TT	TT	--
GG	GG	GG	CG	CG	CG	CG	--	CG	GG	GG	CG	CG
AG	GG	AG	AG	AG	--	--	AA	AG	AG	GG	AG	AA
TT	TT	CT	TT	CT	CT	CT	CT	TT	TT	TT	CT	CT
GT	TT	GT	TT	--	--	--	GT	TT	GT	TT	TT	GT
CC	CC	CT	CC	CT	CT	CT	CT	CC	CC	CC	CT	CT
AC	AA	AC	AA	AA	--	--	AC	AA	AC	AA	AA	AC
GG	AA	GG	AA	AG	AG	AG	GG	AA	AG	AG	GG	GG
AG	AA	AG	AA	AA	--	--	AG	AA	AG	AG	AG	AG
GG	--	GG	TT	GT	--	GT	GG	TT	GT	GT	GG	GG
CT	CC	TT	CC	CT	TT	CT	TT	CC	CT	CT	TT	TT
AC	AA	CC	AA	AC	CC	AC	AC	AA	AC	AC	CC	CC
AT	TT	AA	TT	AT	AA	AT	AT	--	AT	--	AA	AA
GT	GT	GG	GG	GT	--	GT	GT	GG	GG	--	GT	GG
AA	AG	AA	AG	AA	AA	AA	AA	AG	AG	AG	AA	AA
CG	--	GG	CG	CG	--	CG	CG	CG	CG	CG	CG	GG
AG	AA	GG	AG	AG	GG	AG	AG	AG	AG	AG	AG	GG
CC	CC	CC	CC	GG	--	GG	GG	CC	CC	GG	CG	CC
AA	AA	AA	AA	--	--	GG	--	AA	AA	GG	AG	AA
AA	AA	--	AA	--	AG	AG	AG	AA	AA	AG	AG	AA
AA	AA	AA	AT	AT	AT	AT	AT	AT	AA	AT	AA	--
AA	--	AA	AA	AA	AG	AA	AG	--	AG	AG	AG	AA
AG	AG	AG	AG	AG	--	AG	AA	AG	AA	AA	AA	AG
AA	AA	AA	AC	AC	CC	AC	CC	AC	AC	CC	AC	AA
CC	--	CC	AC	AC	AC	AC	AC	AC	CC	AC	CC	CC
--	CC	AA	AC	AC	AC	--	--	AC	AC	AA	AC	CC
GG	--	TT	GT	GT	--	GT	GT	GT	GT	TT	GT	GG
AA	AA	TT	AT	AT	AT	AT	--	AT	AT	TT	AT	AA
AG	AA	AG	AG	AG	AA	AG	AA	AG	AG	AG	AA	AA
CC	CC	CC	AC	AC	CC	AC	CC	AC	CC	--	CC	CC
AT	AA	TT	AT	AT	AT	AT	AT	AT	TT	TT	AT	AA
CT	CC	CT	CT	CT	CT	CT	CT	CT	TT	TT	TT	CC
AG	GG	AG	AG	AG	AG	AG	AA	AG	AA	AA	AA	GG
CT	CC	CT	CT	CT	CT	CT	TT	CT	TT	TT	TT	CC

CT	--	CT	--	CT	CT	CT	CC	CT	CC	--	CC	TT
AG	GG	AG	AG	AG	--	AG	AA	AG	AA	AA	AA	GG
CT	CC	CT	CT	CT	CT	CT	TT	CT	TT	TT	TT	CC
CT	TT	CT	CT	CT	--	--	CC	CT	CC	CC	CC	TT
CT	CC	CT	CT	CT	CT	CT	TT	CT	TT	TT	TT	CC
AG	GG	AG	--	--	--	AG	--	AG	AA	AA	AA	GG
CT	TT	CT	CT	CT	--	CT	CC	CT	CC	CC	CC	TT
AG	GG	AG	AG	AG	AG	AG	--	AG	AA	AA	AA	GG
CG	CC	CG	CG	CG	CG	CG	--	CG	GG	GG	GG	CC
AC	AA	AC	AC	AC	AC	--	CC	AC	CC	CC	CC	AA
CT	CT	CT	CT	CT	CT	CT	TT	CT	TT	TT	TT	CC
CT	--	CT	CT	CT	--	CT	TT	CT	TT	TT	TT	CC
AT	AT	AT	AT	AT	AT	--	AA	AT	AA	AA	AA	TT
CT	--	CT	CT	CT	CC	CT	--	CT	TT	--	TT	CC
CC	AC	AC	AC	AC	--	AC	CC	AC	CC	CC	CC	AA
GG	GT	GT	GT	GT	GT	GT	GG	GT	GG	GG	GG	TT
CC	CT	CT	CT	CT	--	--	CC	CT	CC	CC	CC	TT
CC	CG	CG	CG	CG	CG	CG	CC	CG	CC	CC	CC	GG
--	CG	CG	CG	CG	--	CG	CC	CG	CC	--	--	GG
AA	AC	AC	AC	AC	AC	AC	AA	AC	AA	AA	AA	CC
CC	CG	CG	CG	CG	CG	--	CC	CG	CC	CC	CC	GG
AA	--	AA	TT	AA	--	AA	AT	TT	AT	TT	TT	--
AA	AG	AA	AG	AG	--	AG	AG	AG	AG	GG	GG	AA
AA	--	AA	AG	AG	AG	AG	--	AG	AG	GG	AG	AA
--	GG	GG	AG	AG	--	AG	AA	AG	AA	AA	AG	GG
AA	AA	AA	AG	AG	AG	--	AG	AG	GG	GG	AG	AA
AA	AA	AA	AC	AC	AC	AC	AC	AC	CC	CC	AC	AA
AA	AA	AA	AG	AG	AG	AG	AG	AG	GG	GG	AG	AA
AA	AA	AA	AG	AG	--	AG	AG	AG	GG	GG	AG	AA
GG	GG	GG	GT	GT	GT	GT	GT	GT	TT	TT	GT	GG
TT	TT	TT	GT	GT	GT	GT	--	GT	GG	GG	GT	--
CC	CC	CC	AC	AC	--	AC	AC	AC	AA	AA	AC	CC
TT	TT	TT	CT	--	--	CT	CT	CT	CC	CC	CT	TT
TT	--	TT	CT	CT	--	CT	CT	CT	CC	CC	CT	TT
GG	--	GG	GT	GT	GT	GT	GT	GT	TT	TT	GT	GG
AA	AA	--	AT	AT	--	AT	AT	AT	TT	TT	AT	AA
GG	GG	GG	CG	CG	--	CG	CG	CG	CC	CC	CG	GG
AA	AA	AA	AC	AC	AC	AC	AC	AC	CC	CC	AC	AA
--	--	CC	CT	CT	CT	CT	CT	CT	TT	TT	CT	CC
GG	GG	GG	--	CG	--	CG	CG	CG	CC	CC	CG	GG
CC	CC	CC	--	CT	--	--	CT	CT	TT	TT	CT	CC
AA	AA	AA	AT	AT	AT	AT	AT	AT	TT	--	AT	AA
TT	TT	TT	CT	CT	--	CT	CT	CT	CC	CC	CT	TT
GG	GG	GG	AG	AG	AG	AG	AG	AG	AA	AA	AG	GG
AA	--	--	AT	AT	TT	--	AT	TT	TT	TT	AT	AA
--	AA	AA	AT	AT	AT	AT	AT	AT	TT	TT	AT	AA
AA	AA	AA	AC	AC	AC	AC	AC	AC	CC	CC	AC	AA

TT	TT	TT	GT	GT	GT	GT	GT	GT	GG	GG	GT	TT
GG	--	GG	AG	AG	AG	AG	AG	AG	AA	AA	AG	GG
GG	--	GG	GT	GT	--	GT	GT	GT	TT	TT	GT	GG
AA	AA	AA	AG	AG	AG	AG	AG	AG	GG	GG	AG	AA
TT	TT	TT	--	CT	CT	CT	CT	CT	--	CC	CT	TT
AA	AA	AA	AG	AG	AG	AG	AG	AG	GG	GG	AG	AA
--	TT	TT	CT	CT	--	CT	CT	CT	CC	CC	CT	--
CC	CC	CC	AC	AC	AC	AC	AC	AC	AA	AA	AC	CC
AA	AA	AA	AA	AG	--	AG	AG	AA	GG	GG	AG	AA
CT	TT	TT	TT	CT	--	CT	CT	TT	CC	CT	CT	TT
AG	GG	AG	GG	GG	AG	GG	AG	GG	AA	AG	AG	GG
CT	CC	CT	CC	--	--	CC	CT	--	TT	CT	CT	CC
GG	GG	GT	GG	GT	--	GT	GG	GG	GT	GT	GT	GG
AG	GG	GG	GG	GG	GG	--	AG	GG	AG	GG	GG	GG
AT	--	AT	TT	AT	AT	--	AT	TT	AA	AT	AT	AT
AT	TT	AT	TT	AT	--	AT	AT	TT	AA	AT	AA	AT
AC	CC	AC	CC	AC	--	AC	AC	CC	AA	AC	AA	AC
GT	GG	GT	--	--	GT	GT	GT	GG	TT	GT	TT	GT
CT	CT	TT	TT	CT	CT	CT	CT	TT	CT	CT	CT	CT
AA	AG	AG	AG	AA	AG	AA	--	AG	AG	AG	AG	AG
GG	CG	GG	GG	GG	CG	--	GG	GG	CG	CG	CG	CG
CT	CT	TT	TT	TT	CT	--	TT	TT	CT	CT	CT	CT
GG	GG	GT	GT	GG	GG	GG	GG	GT	GG	GG	GG	GG
GG	AG	GG	AG	AA	--	--	AA	AG	AG	AG	AG	--
AC	AC	--	--	AA	--	--	AC	AA	AA	AC	AC	AC
CG	CG	CG	--	GG	--	--	--	GG	GG	CG	--	CG
GT	GT	GT	TT	TT	GT	--	GT	TT	--	GT	GT	GT
TT	TT	TT	CT	CC	CT	--	CT	CT	CT	CT	CT	CT
AG	AG	AA	GG	AG	--	AG	AA	GG	GG	AA	AA	AA
CT	CT	CT	CC	--	--	--	--	CC	CC	CT	CT	--
CC	CC	CC	CG	CG	CC	CG	CC	CG	CG	CC	CC	CC
AA	AA	AC	AA	AC	AC	AC	AC	AA	AA	AC	AA	AC
AA	AA	AG	AG	GG	AG	GG	AG	AG	AA	AG	AA	AG
CT	CT	TT	CT	TT	--	TT	TT	CT	CT	TT	CT	TT
CG	CG	CG	GG	GG	CG	GG	CG	GG	GG	CG	CG	CG
--	GG	AG	AG	AA	AG	AA	AG	AG	AG	AG	GG	AG
GG	GG	--	CG	CG	--	CG	GG	CG	CG	GG	GG	GG
GG	GG	CG	CG	CC	--	--	CC	CG	CG	CG	GG	CG
TT	TT	CT	CT	CC	--	--	CT	CT	CT	CT	TT	CT
AA	--	AG	AG	GG	AG	--	AG	AG	AG	AG	AA	AG
GG	GG	AG	GG	AG	AG	AG	AG	GG	GG	AG	GG	AG
--	TT	CC	CT	CC	CT	--	CT	CT	TT	CT	TT	CC
AG	AG	GG	AG	GG	--	GG	--	AG	AG	GG	AG	GG
AA	AA	GG	AG	GG	AG	GG	AG	AG	AA	AG	AA	GG
AA	AA	CC	AC	CC	--	CC	AC	AC	AA	AC	AA	CC
AT	--	TT	TT	TT	AT	--	AT	TT	AT	AT	AT	TT
GG	--	AA	AG	--	AG	AA	AG	AG	GG	--	GG	AA

CC	CC	AA	AC	AA	AC	--	AC	AC	CC	AC	CC	AA
--	--	AA	AG	AA	AG	AA	AG	AG	GG	AG	GG	AA
AG	AG	AA	AG	AA	--	AA	AA	AG	AG	AA	AG	AA
CT	CT	CC	CC	CC	--	--	CC	CC	CT	CT	CT	CC
CG	CG	CC	CC	CC	CG	CC	CC	CC	CG	CG	CG	CC
CT	CT	TT	TT	--	CT	TT	TT	TT	CT	CT	CT	TT
GG	GG	CG	CG	--	GG	CG	CG	CG	GG	GG	GG	CG
CT	CT	CC	CC	CC	CT	--	CC	CC	CT	CT	CT	CC
GG	--	GT	GT	GT	GG	GT	GT	GT	--	GG	GG	GT

5-69	6-5	6-6	6-9	6-10	6-11	6-13	6-14	6-15	6-18	6-20	6-21	6-23	6-27
AC	CC	AA	AC	AA	CC	AC	AA	CC	AA	AC	AC	AC	CC
AG	GG	AA	AG	AA	GG	--	AA	GG	AG	AG	AG	AG	GG
CT	CC	TT	CT	TT	CC	CC	CT	CC	CT	CT	CT	CT	CT
AG	AG	GG	GG	GG	AG	AG	GG	AG	AG	GG	GG	AG	--
AG	AG	AA	AG	AA	GG	GG	AG	GG	AG	AG	AG	AG	AG
CG	CG	CG	CG	CG	CG	GG	GG	GG	CG	CG	GG	CG	GG
CT	CT	TT	TT	TT	CT	CC	CT	CC	CT	TT	CT	CT	CT
GT	GT	TT	TT	TT	GT	GG	GT	GG	GT	GT	GT	GT	GT
GT	TT	GT	TT	TT	GT	GG	GT	GG	GT	GT	GT	GT	GT
GT	TT	GT	TT	TT	GT	GG	GT	GG	GT	GT	GT	GT	GT
AG	AA	AG	--	AA	AG	GG	AG	GG	AG	AG	AG	AG	AG
CT	CT	CC	CT	CT	CT	CC	CC	CC	--	CT	CC	CT	CC
AA	AA	AC	AA	AC	AC	CC	AC	CC	AC	AC	CC	AC	--
AG	AG	AG	AG	AG	AG	AG	AA	AA	AG	AG	AA	AG	AA
CT	CT	CT	CT	CC	CC	CT	CT	CC	CC	CC	CC	CC	CT
GG	GG	GG	GG	AG	AG	GG	AG	AA	AG	AG	AA	AG	AG
AT	TT	AT	AT	AT	TT	--	TT	TT	AT	AT	TT	AT	AT
CT	CT	CT	CT	CC	CC	CT	CT	CC	CC	CC	CC	CC	CT
GT	TT	GT	GT	GT	TT	GT	TT	TT	TT	GT	GT	GT	GT
CG	CC	CG	CG	CG	CC	CG	CC	CC	CC	CC	CG	CG	CG
AA	AT	AA	AA	AT	--	AA	AT	TT	TT	TT	AT	AT	AA
CC	CT	CC	CC	CT	TT	--	CC	TT	TT	TT	CT	CT	CT
TT	AT	TT	TT	AT	AA	TT	AT	AA	AA	AA	AT	--	AT
TT	AT	TT	TT	AT	AA	TT	AT	AA	AA	AA	AT	AT	AT
GG	AG	GG	GG	AG	AA	--	AG	AA	AA	AA	AG	AG	AG
GT	TT	TT	GT	GT	TT	GT	TT	TT	TT	TT	GT	GT	TT
GG	GT	GG	GG	GT	TT	GG	GT	TT	TT	TT	GT	GT	GT
AA	AT	AA	AA	AA	TT	AA	AT	TT	TT	TT	AT	AT	AT
CC	CT	CC	CC	CT	TT	CC	CT	TT	TT	TT	CT	CT	CT
GG	CG	GG	GG	CG	CC	GG	CG	CC	CC	CC	CG	CG	CG
TT	CT	TT	TT	CT	CC	TT	CT	CC	CC	CC	CT	CT	CT
CC	AC	CC	CC	AC	AA	CC	AC	AA	AA	AA	AC	AC	AC
GG	AG	GG	GG	AG	AA	--	AG	AA	AA	AA	AG	AG	AG
TT	GT	TT	TT	GT	GG	TT	GT	GG	GG	GG	GT	GT	GT
GG	AG	GG	GG	AG	AA	GG	AG	AA	--	AA	AG	AG	AG
GG	GT	GG	GG	GG	TT	--	GT	TT	--	TT	--	GT	GT
AA	AG	AA	AA	AG	GG	AA	AG	GG	GG	GG	AG	AG	AG
TT	CT	TT	TT	CT	CC	TT	CT	CC	CC	CC	CT	CT	CT
TT	AT	TT	TT	AT	AA	TT	AT	AA	AA	AA	AT	AT	AT
GG	AG	GG	GG	AG	AA	--	AG	AA	AA	AA	AG	AG	AG
CC	AC	CC	CC	AC	AA	CC	AC	AA	AA	AA	AC	AC	AC
GG	AG	--	GG	AG	--	GG	AG	AA	--	AA	AG	AG	AG

CC	CT	CC	CC	CT	TT	CC	CT	TT	TT	TT	CT	CT	CT
GG	CG	GG	GG	CG	CC	GG	CG	CC	CC	--	CG	CG	CG
CC	CT	CC	CT	CT	TT	--	--	--	TT	TT	CT	CT	CT
TT	CT	TT	--	CT	CC	TT	CT	CC	CC	CC	CT	CT	CT
AC	CC	CC	AC	AC	AA	AC	AC	AA	AA	AA	AC	AC	AC
CT	TT	TT	CT	CT	CC	CT	CT	TT	CC	CC	CT	CC	CT
CT	CC	CC	CT	CT	TT	CT	CT	CC	--	TT	CT	TT	CT
AG	GG	AG	AG	AG	AG	AA	AG	AA	GG	AG	AG	AG	AG
GT	GG	GT	GT	GT	GT	TT	GT	TT	GG	GT	GT	GT	GT
AG	GG	AG	AG	AA	AG	--	AG	AA	GG	AG	AG	AG	AG
CT	TT	CT	CT	CT	CT	CT	CT	--	TT	CT	CT	CT	CT
CG	CC	CG	CG	CG	GG	CG	CG	GG	CC	CG	CG	CG	CC
AG	AA	AG	AG	AG	GG	AG	AG	GG	AA	AG	AG	AG	AA
AC	CC	AC	CC	AC	AA	AC	AC	AC	CC	--	AA	CC	CC
GG	AG	GG	--	AG	GG	GG	GG	AG	AG	GG	GG	AG	AG
CT	TT	CC	TT	CT	CC	--	TT	CT	TT	CT	CC	TT	TT
CT	CC	TT	CC	CT	TT	CT	CT	CT	CC	CT	TT	CC	CC
CT	TT	CC	TT	CT	CC	CT	CC	CT	TT	CT	CC	TT	TT
AG	AA	GG	AA	AG	GG	--	GG	AG	AA	AG	GG	AA	AA
AC	AA	CC	AA	AC	CC	AC	CC	AC	AA	AC	CC	AA	AA
AG	AG	GG	AG	GG	GG	AG	GG	AG	AA	AG	GG	AA	AG
CG	CG	CC	CG	CC	CC	--	CC	CG	GG	CG	CC	GG	CG
CG	CG	GG	CG	GG	GG	GG	GG	CG	CC	CG	GG	CC	CG
AA	GG	GG	AG	GG	GG	AG	GG	AG	AA	AG	GG	AA	AG
CC	AA	AA	AC	AA	--	AC	AA	AC	CC	AC	AA	CC	AC
TT	CC	CC	CT	CC	CC	CT	CC	CT	TT	CT	CC	CT	CT
CC	TT	TT	TT	TT	TT	CT	TT	CT	CC	CT	CT	CT	CT
TT	AA	AA	AA	AA	AA	TT	AA	TT	TT	AT	AT	AT	AT
GG	AG	AG	AG	AG	AG	--	AG	GG	GG	AG	GG	GG	GG
TT	AT	AT	AT	AT	TT	--	AT	TT	TT	TT	AT	AT	AT
GT	GG	GT	--	GG	--	GG	GG	GT	TT	GT	GG	GT	TT
AG	AG	AG	AG	AG	AG	AG	AG	GG	--	GG	AG	AG	GG
CT	CT	CT	CT	CT	CT	CT	CT	TT	TT	TT	CT	CT	TT
AG	AG	AG	AG	AG	AG	AG	AG	GG	GG	GG	AG	AG	GG
GT	TT	GT	TT	TT	GT	TT	TT	GT	GG	GT	TT	GT	GG
GT	GG	GT	--	GT	GT	GT	GT	GG	--	GG	GT	GT	GG
CT	CT	CT	CC	CC	CC	CC	CC	CT	TT	CT	CC	CT	TT
CT	CT	CC	CT	CC	CC	CC	CC	CT	TT	CT	CC	CT	CT
AG	AG	AG	AG	GG	GG	GG	GG	AG	AA	AG	GG	AG	AG
CG	CG	CG	CG	CC	CC	CC	CC	CG	GG	CC	CC	CG	CG
AG	AG	AG	AG	AG	GG	AG	GG	AG	AA	GG	GG	AG	AG
CT	CT	CT	CT	CT	--	CT	TT	CT	CT	TT	CT	TT	CT
CT	CT	CT	CT	CT	TT	CT	--	CT	CT	CT	CT	TT	CT
CG	CC	CG	CC	CC	CG	CG	CG	CC	CG	CG	CC	CG	CG
AT	TT	AT	TT	TT	AT	AT	AT	TT	AT	AT	TT	AT	TT
GG	GT	GG	GT	GT	GT	GG	GT	GT	GG	GG	GT	GT	GG
CC	CT	CC	CT	CT	CT	CC	CT	CT	CC	CC	CT	CT	CC

CT	CT	CC	CT	CT	TT	CT	CC	CT	CT	CT	CT	TT	CC
GG	GT	GG	GT	GT	GT	GG	GT	GT	--	GG	GT	GT	GT
CT	CT	CC	CT	CT	TT	--	CT	CT	CT	CT	CT	TT	CT
TT	GT	TT	GT	GT	GT	TT	GT	GT	TT	TT	GT	GT	GT
GG	CG	GG	CG	CG	CG	--	CG	CG	GG	GG	CG	CG	CG
AG	GG	AA	GG	AA	AA	--	--	AA	AG	AG	AA	AG	AG
AG	AA	GG	AA	GG	GG	GG	GG	AG	AG	AG	GG	AG	AG
AG	AA	GG	AA	GG	GG	GG	AG	AG	AG	AG	GG	GG	AG
CT	TT	CC	TT	CT	CC	--	CT	CT	CT	CT	CC	CC	CT
AG	GG	AA	GG	AG	AA	AA	GG	AG	AG	AG	AA	AA	AG
GT	GG	TT	GG	GT	--	--	GG	GT	GT	GT	TT	TT	GT
GG	AG	GG	GG	AG	GG	GG	AG	AG	AG	AG	GG	GG	GG
CT	TT	CC	CT	CT	CT	CC	TT	CT	CT	CT	CC	CC	CC
CC	CC	AC	CC	AC	AC	CC	CC	AC	AC	AC	AC	AC	AC
CT	CT	CC	CT	CC	CC	CC	CT	CC	CC	CC	CC	CC	CC
GT	--	TT	GT	GT	GT	TT	GG	GT	GT	GT	TT	TT	TT
CT	CC	CT	CT	CC	CC	CT	CC	CC	CC	CC	CT	CT	CT
GG	GG	CG	GG	CG	CG	CG	GG	CG	CG	CG	CG	CG	CG
CC	CC	CC	--	AC	AC	--	CC	AC	--	AC	AC	AC	--
CT	TT	CT	--	TT	TT	CT	TT	TT	TT	TT	CT	CT	CT
AG	AA	AG	AG	AA	AA	AG	AA	AA	AA	--	AG	AG	AG
AG	AA	GG	AG	AG	AG	GG	AA	AG	--	AG	GG	GG	GG
CT	CT	CC	CT	CC	CC	CC	CT	CC	CC	CC	CC	CC	CC
AA	AA	AT	--	AT	AT	AT	AA	AT	AT	AT	AT	AT	AT
CC	CC	CT	CC	CT	CT	CT	CC	CT	CT	CT	CT	CT	CT
AA	AA	AG	AA	AG	AG	AG	AA	AG	AG	AG	AG	AG	AG
CT	CC	CT	CT	CC	CC	CT	CC	CC	CC	CC	CT	CT	CT
AT	TT	AA	AT	AT	AT	--	--	AT	AT	AT	AA	AA	AA
AT	TT	AA	AT	AT	AT	--	TT	AT	AT	AT	--	AA	AA
CT	TT	CC	CT	CT	CT	--	TT	CT	CT	CT	CC	CC	CC
CC	CC	CT	CC	CT	CT	--	CC	CT	CT	CT	CT	CT	CT
GT	GG	GT	GT	GG	GG	GT	GG	GG	GG	GG	GT	GT	GT
CT	CC	CT	CT	CC	CC	CT	CC	CC	CC	CC	CC	CT	CT
AA	AG	AA	--	AG	AG	--	AG	AG	AG	AG	AG	AA	AA
AG	GG	AA	AG	AG	AG	AA	GG	AG	AG	AG	AG	--	AA
AG	AA	AG	AG	AA	AA	AG	AA	AA	AA	AA	AA	AG	AG
CT	CC	TT	CT	CT	CT	TT	CC	CT	CT	CT	CT	TT	TT
AC	CC	AC	AC	CC	CC	AC	--	CC	CC	CC	CC	AC	AC
AT	TT	AA	AT	AT	AT	AA	TT	AT	AT	AT	AT	AA	AA
AA	AA	AA	AA	AG	AG	--	--	AG	AG	AG	AG	AG	AG
AC	AA	AC	--	AA	AA	AC	AA	AA	AA	AA	AA	AC	AC
AT	TT	AT	AT	TT	TT	AT	TT	TT	TT	TT	TT	AT	AT
AA	AC	AC	AA	CC	CC	--	AC	CC	CC	CC	CC	AC	AC
CC	CC	AC	CC	AC	AC	AC	CC	AC	AC	AC	AC	AC	AC
GG	AG	GG	GG	AG	AG	--	AG	AG	AG	AG	AG	GG	GG
AC	AC	CC	AC	CC	CC	CC	AC	CC	CC	CC	CC	CC	CC
AG	AA	AG	AG	AA	AA	AG	AA	AA	AA	AA	AA	AG	AG

CC	CC	CG	CC	CG	CG	CG	CC	CG	CG	CG	CG	CG	CG
CT	TT	CC	CT	CT	CT	CC	TT	CT	CT	CT	CT	CC	CC
CT	CC	TT	CT	CT	CT	TT	CC	CT	CT	CT	CT	TT	TT
CT	CC	CT	--	CC	CC	CT	CC	--	CC	CC	CC	CT	CT
AG	AA	AA	AA	AA	AA	AG	AA	AA	AA	AA	AA	AG	AG
CC	CC	TT	CC	CT	CT	--	CC	CT	CT	CT	CT	TT	TT
TT	TT	CT	TT	TT	TT	CT	TT	TT	TT	TT	TT	CT	CT
AA	AA	TT	AA	AT	AT	TT	AA	AT	AT	AT	AT	TT	TT
GG	GG	AG	GG	AG	AG	AG	GG	AG	AG	AG	AG	AG	AG
AA	AA	AC	AA	AA	AA	--	AA	AA	AA	AA	AA	AC	AC
CC	CC	CT	CC	CC	CC	--	CC	CC	CC	CC	CC	--	CT
TT	TT	GT	TT	GT	GT	GT	TT	GT	GT	GT	GT	GT	GT
TT	--	CT	--	TT	--	CT	TT	TT	TT	TT	TT	CT	CT
AC	AC	CC	AC	AC	AC	CC	AC	AC	AC	AC	AC	CC	CC
AA	AA	AG	AA	AA	AA	--	AA	AA	AA	AA	AA	AG	AG
GG	GG	GT	GG	GG	GG	GT	GG	--	GG	GG	GG	GT	GT
AT	AT	TT	AT	AT	AT	--	AT	AT	AT	AT	AT	TT	TT
AA	AA	AG	AA	AA	AA	AG	AA	AA	AA	AA	AA	AG	AG
CC	CC	CT	--	CT	--	--	CC	CT	CT	CT	CT	CT	CT
CC	CC	CT	--	CC	CC	CT	CC	CC	CC	CC	CC	CT	CT
AA	AA	AC	--	AA	AA	AC	AA	AA	AA	AA	AA	AC	AC
CC	CC	CT	CC	CC	CC	CT	CC	CC	CC	CC	CC	CT	CT
AA	AA	TT	AA	AT	AT	--	--	AT	AT	AT	AT	TT	TT
GG	GG	AA	GG	AG	AG	AA	GG	AG	AG	AG	AG	AA	AA
GG	GG	CG	GG	CG	CG	CG	GG	CG	CG	CG	CG	CG	CG
TT	TT	CT	TT	TT	TT	CT	TT	TT	TT	TT	TT	CT	TT
GG	GG	AG	--	GG	AG	AG	--	GG	GG	GG	GG	AG	GG
AA	AA	AG	AA	AA	AG	AG	AA	AA	AA	AA	AA	AG	AA
AA	AA	AG	AA	AG	AG	AG	AA	AG	AG	AG	AG	AG	AG
GG	GG	AG	GG	AG	AG	AG	GG	AG	AG	AG	AG	AG	AG
CT	TT	CT	--	TT	CT	TT	TT	--	TT	CT	TT	CT	TT
GT	GG	TT	GG	GT	TT	GT	GG	GT	GT	TT	GT	TT	GT
TT	TT	GT	TT	GT	GT	GT	TT	GT	GT	GT	GT	GT	GT
AG	GG	AA	GG	AG	AA	AG	GG	GG	AG	AA	AG	AA	AG
CT	CC	CC	CC	CC	CT	CC	CC	CC	CC	CT	CC	CT	CT
GT	TT	TT	TT	TT	GT	GT	TT	TT	TT	GT	TT	GT	GT
AG	AG	GG	--	GG	GG	GG	AG	GG	--	GG	GG	GG	GG
AA	AG	AG	AA	AA	GG	AG	AG	AA	GG	GG	AG	GG	AG
CT	CC	CC	CT	CT	CC	CC	CC	CC	CC	CC	CC	CC	CC
AA	AA	AT	AA	AA	AT	AT	AT	AA	AT	AT	AA	AT	AA
CC	CG	CG	CC	CC	GG	--	GG	CC	GG	GG	GG	GG	CG
AA	AC	AA	AA	AA	AC	AC	AC	--	AC	AC	AC	AC	AC
AG	AG	AG	AA	AA	GG	--	GG	AA	GG	AG	GG	GG	AG
GG	AG	GG	AG	AG	GG	GG	GG	AG	GG	AG	GG	GG	AG
CC	CT	CC	CT	CT	CC	CC	CC	CT	CC	CT	CC	CC	CT
AG	AG	AG	GG	AG	AA	AA	AA	GG	GG	AG	AA	AA	AG
CC	CT	CT	CT	CT	CT	CT	CT	CC	CC	CT	CT	CT	CT

AG	GG	AG	GG	GG	AG	AG	AG	GG	GG	GG	AG	AG	GG
AA	AG	--	AG	GG	AG	AA	AG	GG	AG	GG	AG	AG	GG
TT	CC	TT	CC	CT	TT	TT	TT	CT	CC	CT	TT	TT	CT
TT	AA	TT	--	AT	TT	AA	TT	AT	AA	AT	TT	TT	AT
CC	AA	CC	AA	AC	CC	AC	CC	AC	AC	AC	CC	CC	AC
CC	TT	CC	TT	CT	CC	CT	CC	CT	CT	CT	CC	CC	CT
AA	GG	AA	AG	GG	AA	AG	AA	AG	AG	AG	AA	AA	AG
TT	CC	TT	CT	CC	TT	--	TT	CT	CT	CT	TT	TT	CT
CT	TT	CT	--	TT	CT	--	CT	CT	CT	CT	CT	CT	CT
GG	AA	GG	--	AG	GG	AA	GG	AG	AG	AG	GG	GG	AG
AA	AG	AG	AG	AG	GG	--	AA	AA	AA	AG	AA	AA	AA
CC	CT	CT	CT	CT	TT	TT	CC	CT	CC	CT	CC	CC	CC
GG	AG	AG	AG	GG	AG	--	GG	GG	AG	AG	GG	GG	GG
CC	CT	TT	CT	CT	TT	CT	CC	CT	CT	CT	CC	CC	CC
GG	GG	CG	--	CG	CG	CG	GG	CG	GG	GG	GG	GG	GG
CT	CT	TT	CT	CT	TT	CT	--	CT	CT	CT	TT	CC	CC
CT	CT	CC	CT	CC	CT	CC	CC	CC	CT	CT	CT	CC	CC
AG	AG	AG	AG	AG	AG	AG	GG	AG	AG	AG	AA	GG	GG
CT	CT	CT	CT	CT	CT	--	CC	CT	CT	CT	TT	CC	CC
AG	AG	AG	AG	AG	AG	AG	AA	AG	AG	GG	GG	AA	AA
CT	CT	CT	CT	CT	CC	CT	CC	CT	CC	TT	TT	CC	CT
AG	AG	AG	AG	AG	GG	--	GG	AG	GG	AG	AA	GG	AG
AG	GG	GG	AG	GG	GG	GG	GG	GG	GG	GG	AG	GG	AG
GT	--	GT	GG	GT	TT	GG	TT	GG	TT	GT	GG	TT	GT
CG	CC	CC	CG	CC	--	CG	CC	CG	CC	CC	CG	CC	CG
CG	CG	CC	CC	CC	CG	CC	CG	CC	CG	CC	CC	CG	CG
TT	CC	CT	--	CC	CC	TT	CC	TT	--	CT	TT	CT	TT
CC	TT	CT	CT	TT	TT	CC	TT	CC	TT	CT	CC	CT	CC
TT	AA	AT	AT	AA	AA	TT	AA	TT	AA	TT	TT	AT	TT
AA	GG	AG	AG	GG	GG	AA	GG	AA	GG	AA	AA	AG	AA
TT	CC	CT	CT	CC	--	TT	CC	TT	--	TT	TT	CT	TT
CT	TT	TT	TT	TT	TT	CT	TT	CT	TT	CT	CT	TT	CT
CC	AA	AC	AC	AA	AA	CC	--	CC	AA	AC	CC	AC	CC
TT	TT	GT	GT	GT	GT	GG	GT	GG	GG	TT	GT	TT	GG
AA	AA	AG	AG	AG	AG	--	AG	GG	GG	--	AG	AA	GG
TT	TT	CT	CT	CT	CT	CC	CT	CC	--	TT	CT	TT	CC
AA	AA	AT	AT	AT	AT	TT	AT	TT	TT	AA	AT	--	TT
CC	CC	AC	AC	AC	AC	AA	AC	AA	AA	CC	AC	CC	AA
CG	CG	CG	CG	CC	CG	CC	CC	CC	CC	CG	CG	CG	CC
AG	AG	AG	AG	AA	AG	AA	AA	AA	AA	AG	AG	AG	AA
CC	CC	CT	CT	CT	CT	--	CT	TT	CT	CC	CT	CC	TT
CC	CC	CT	CT	CT	CT	TT	CT	TT	TT	CC	CT	CC	TT
AA	AA	AG	AG	AG	AG	GG	AG	GG	AG	AA	AG	AA	GG
CT	CT	CC	CC	CT	CC	CC	CT	CC	CC	CT	CC	CT	CC
AA	AA	GG	AG	AG	AG	--	--	GG	AG	AA	AG	AA	GG
GG	GG	--	AG	AG	AG	AA	AG	AA	AG	--	AG	GG	AA
GG	GG	GT	GT	GT	GT	TT	GT	--	GT	GG	GT	GG	TT

TT	TT	CT	CT	CT	CT	--	CT	CC	CT	TT	CT	TT	CC
GG	GG	CG	CG	CG	CG	CC	CG	CC	CG	GG	CG	GG	CC
TT	TT	GT	GT	GT	GT	GG	GT	GG	GT	TT	GT	TT	GG
AA	AA	AG	AG	AG	AG	GG	AG	GG	AG	AA	AG	AA	GG
CC	CC	CT	CT	CT	CT	TT	CT	TT	CT	CC	CT	CC	TT
GT	GT	--	TT	GT	TT	--	GT	TT	TT	GT	TT	GT	TT
GG	GG	CG	CG	CG	CG	CC	CG	CC	CG	GG	CG	GG	CC
AA	AA	AG	AG	AG	AG	--	--	GG	AG	AA	AG	AA	GG
AG	AG	GG	GG	AG	GG	GG	AG	GG	--	AG	GG	AG	GG
AA	AA	AC	AC	AC	AC	--	AC	CC	AC	AA	AC	AA	CC
CG	CG	CC	CC	CG	CC	CC	CG	CC	CC	CG	CC	CG	CC
GG	GG	AG	AG	AG	AG	AA	AG	AA	AG	GG	AG	GG	AA
AA	AA	AC	--	AC	AC	--	AC	CC	AC	AA	AC	AA	CC
TT	TT	GT	GT	GT	GT	GG	GT	GG	GT	TT	GT	TT	GG
CC	CC	--	AC	AC	AC	AA	AC	AA	AC	CC	AC	CC	AA
CT	CT	CC	CC	CT	CC	CC	CT	CC	CC	CT	CC	CT	CC
TT	GT	GT	GT	GT	GT	GG	GT	GG	--	TT	GT	TT	GG
AG	GG	AG	AG	GG	AG	--	GG	GG	AG	AG	AG	AG	GG
CC	CT	CT	CT	CT	CT	TT	CT	TT	CT	CC	CT	CC	TT
CT	CT	CC	CC	CT	CC	CC	CT	CC	CC	CT	CC	CT	CC
AA	AT	AT	AT	AT	AT	TT	AT	TT	AT	AA	AT	AA	TT
CC	CG	CG	CG	CG	CG	GG	CG	GG	CG	CC	CG	CC	GG
GG	GT	GT	GT	GT	GT	TT	GT	TT	GT	GG	GT	GG	TT
AG	GG	AG	AG	GG	GG	GG	AG	GG	AG	AG	AG	AG	GG
TT	TT	GT	GT	TT	GT	GT	GT	GT	GT	TT	GT	TT	GT
CC	CT	CT	CT	CT	TT	TT	CT	TT	CT	CT	CT	CC	TT
CC	AC	CC	CC	AC	AC	AA	AC	AC	AC	AC	AC	CC	AA
CT	CT	CC	CC	CT	CT	CT	CC	CC	CC	CC	CC	CC	CT
CG	CG	GG	GG	CG	CG	CG	CG	CG	CG	CG	CG	GG	CC
CT	CT	CC	CC	CT	CT	CT	CT	CT	CT	CT	CT	CC	CT
TT	TT	GT	GT	TT	TT	TT	GT	GT	GT	GT	GT	GT	GT
CT	CT	CT	CT	TT	CT	CT	TT	CT	TT	CT	TT	CT	CT
TT	TT	AT	AT	TT	--	--	AT	AT	AT	AT	AT	AT	AT
AC	AC	CC	CC	AC	AC	AC	CC	CC	CC	CC	CC	CC	CC
CT	CT	TT	TT	CC	CC	CC	CT	TT	CT	TT	CT	TT	TT
AG	AG	AA	AA	AG	--	AG	AA	AA	AA	AA	AA	AA	AA
AG	AG	GG	--	AG	AG	AG	GG	GG	GG	GG	GG	GG	GG
CG	CG	GG	--	GG	GG	--	GG	GG	GG	--	CG	CG	CG
CT	--	CT	CT	CC	CT	CC	CT	TT	--	CC	CT	TT	CT
CT	CT	TT	TT	CC	CT	CC	TT	TT	CT	CC	CT	TT	CT
TT	TT	TT	CT	CC	CT	CC	TT	TT	CT	CC	--	TT	CT
CC	CC	CC	CT	TT	CT	TT	CC	CC	CT	TT	CT	CC	TT
TT	TT	TT	GT	GG	GT	GT	TT	TT	GT	GG	GT	TT	GG
CC	CC	CC	CT	TT	CT	CC	CC	--	CT	TT	CT	CC	TT
AA	AA	AA	AT	TT	AT	AA	AA	AA	AT	TT	AT	AA	TT
AA	AA	AA	AG	GG	AG	AA	AA	AA	AG	AG	AG	AA	GG
CC	CC	CC	AC	AA	AC	CC	CC	CC	AC	AC	AC	CC	AA

AA	AA	AA	--	GG	AG	AA	AA	AA	AG	AG	AG	AA	GG
AG	AA	AA	AG	GG	AG	--	--	AA	AG	AG	AG	AA	GG
CT	TT	TT	CT	CC	CT	TT	TT	TT	CT	CT	CT	TT	CC
AG	GG	GG	AG	AA	AG	GG	GG	GG	AG	AG	AG	--	AA
GT	GG	GG	GT	TT	GT	GG	GG	GG	GT	GT	GT	GG	TT
CT	TT	TT	CT	CC	--	TT	TT	TT	CT	CT	CT	TT	CC
AG	GG	GG	AG	AA	AG	GG	GG	GG	AG	AG	AG	GG	AA
AG	GG	GG	AG	AA	AG	--	GG	GG	AG	AG	AG	GG	AA
AG	AA	AA	AG	GG	AG	AA	AA	AA	AG	AG	AG	AA	GG
CG	GG	GG	CG	CC	CG	GG	GG	GG	CG	CG	CG	GG	CC
AG	AA	AA	AG	GG	AG	--	AA	AA	AG	AG	AG	AA	GG
AC	AA	AA	AC	CC	AC	AA	AA	AA	AC	AC	AC	AA	CC
AG	GG	GG	GG	AA	AG	GG	GG	GG	AG	AG	AA	GG	AA
AG	GG	GG	AG	AA	AG	GG	GG	GG	AG	AG	AA	GG	AA
AT	AA	AA	AT	TT	AT	AA	AA	AA	AT	AT	TT	AA	TT
CT	TT	TT	CT	CC	CT	TT	TT	TT	CT	CT	CC	TT	CC
CT	TT	--	--	CC	CT	TT	--	TT	CT	CT	CC	TT	CC
CG	CC	CC	CG	GG	CG	CC	CG	CC	CG	CG	GG	CC	GG
CT	CC	CC	CT	TT	CT	CC	CC	CC	CT	CT	TT	CC	TT
CT	TT	TT	CT	CC	CT	TT	TT	TT	CT	CT	CC	TT	CC
CT	CC	CC	CT	TT	CT	CC	CC	CC	CT	CT	TT	CC	TT
AT	AA	AA	AT	TT	AT	--	AA	AA	AT	AT	TT	AA	TT
AT	TT	TT	AT	AA	AT	TT	TT	TT	AT	AT	AA	TT	AA
CT	CC	CC	CT	TT	CT	CC	CC	CC	CT	CT	TT	CC	TT
CT	TT	TT	CT	CC	CT	TT	TT	TT	CT	CT	CC	TT	CC
AT	TT	TT	AT	AA	AT	TT	TT	TT	AT	AT	AA	TT	AA
AG	AA	AA	AG	GG	AG	--	AA	AA	AA	AG	GG	AA	GG
GT	TT	TT	GT	GG	GT	--	TT	TT	TT	GT	GG	TT	GG
CT	CC	CC	CT	TT	CT	CC	CC	CC	CC	CT	TT	CC	CT
CG	GG	GG	CG	CC	CG	GG	GG	GG	GG	CG	CC	GG	CC
AG	AA	AA	AG	GG	AG	AA	AA	AA	AA	AG	GG	AA	GG
CT	CC	CC	CT	TT	CT	CC	CC	CC	CC	CT	TT	CC	TT
CT	CC	CC	CT	TT	CT	CC	CC	CC	CC	CT	TT	CC	TT
AT	AA	AA	AT	TT	AT	AA	AA	AA	AA	AT	TT	AA	TT
AG	GG	GG	AG	AA	AG	GG	GG	GG	GG	AG	AA	GG	AA
AG	GG	GG	AG	AA	AG	GG	GG	GG	GG	AA	AA	AG	AA
GT	GT	GG	GT	GT	GT	GG	GG	GG	GG	TT	TT	GT	TT
AG	AG	GG	AG	AG	AG	GG	GG	GG	GG	AA	AA	AG	AA
GG	GG	--	AG	AG	GG	AG	AG	AG	AG	GG	GG	AG	GG
CT	CT	TT	TT	CT	TT	TT	CT	CT	TT	CT	TT	CT	CT
AG	AG	GG	AG	AG	AG	AG	AG	AA	AG	--	GG	AA	AA
GG	GG	GG	--	GG	AG	AG	GG	AG	AG	AG	GG	AG	AG
AG	AG	GG	--	AG	AG	AG	--	AA	AG	AA	GG	AA	AA
CG	CG	CC	CC	CG	CG	CG	CG	GG	CG	GG	CG	GG	GG
CT	CT	TT	TT	CT	TT	CT	CT	CC	CT	CC	CT	CC	CC
CC	CC	AC	AC	CC	AC	AC	CC	CC	AC	CC	AC	CC	CC
AG	AG	AG	--	AG	AG	AG	AG	AG	--	AA	AA	AA	AA

CC	CC	AC	AC	CC	AC	AC	CC	CC	AC	CC	AC	CC	CC
CC	CC	CC	AC	CC	CC	CC	CC	CC	AC	AC	AC	AC	AC
TT	TT	CT	CT	TT	CT	CT	TT	TT	CT	TT	CT	TT	TT
AG	AA	GG	AG	AG	GG	GG	AG	AG	AG	AA	AG	AA	AG
AG	GG	AA	AG	AG	AA	AG	--	AG	AG	GG	AG	GG	AG
CT	TT	CC	CT	CT	CC	CT	CT	CT	CT	TT	TT	TT	CT
TT	CT	TT	CT	TT	TT	CT	TT	TT	CT	CT	TT	CT	TT
CG	CC	GG	CG	CG	CG	CC	CG	CG	CG	CC	CG	CC	GG
AG	AA	GG	AG	AG	AG	AA	AG	AA	AG	AA	AG	AA	GG
CT	TT	CT	CT	CT	CT	TT	TT	TT	TT	TT	CT	CT	CC
AA	AT	AT	AT	AA	AT	--	AT	AT	AT	--	AA	AA	AA
AA	CC	AC	AC	AC	AC	CC	CC	CC	CC	CC	AC	AC	AC
AG	--	AG	AG	GG	AG	--	AG	GG	GG	GG	GG	GG	AG
CT	CC	CT	CT	CC	CT	CC	CT	CC	CC	CT	CC	CC	CT
CT	CT	CT	CT	CC	CT	CC	CT	CC	CC	CT	CC	CC	CT
GT	GG	GT	GT	GG	GT	--	GT	GG	GG	GT	GG	GG	GT
CC	TT	TT	CT	CT	CC	CT	CC	CT	TT	CT	CT	CT	CT
AG	GG	GG	GG	AG	AG	AG	AG	AG	GG	GG	AG	AG	AG
AA	GG	GG	AG	AG	AA	AG	AG	AA	GG	AG	AG	AG	AG
GG	CG	CG	GG	CG	GG	CG	CG	GG	CG	GG	CG	GG	CG
AA	GG	AG	AG	AG	AA	AA	AG	AA	GG	AG	AG	AA	AG
GG	AA	AG	AG	AG	GG	AG	AG	GG	AA	AG	AG	GG	AG
GT	TT	GT	GT	TT	GT	GT	TT	GT	TT	--	TT	GT	TT
GG	AA	AG	AG	AG	GG	GG	AG	GG	AA	AG	AG	GG	AG
GG	AA	AG	AG	AG	GG	GG	AG	GG	AA	AG	AG	GG	AG
GT	GG	GT	GT	GG	GT	GT	GG	GG	GG	GT	GG	GT	GG
TT	CC	CT	CT	CT	TT	TT	CT	--	CC	TT	CT	TT	CT
CC	TT	CT	--	CT	CT	CC	CT	CT	TT	CC	CC	CC	CT
CT	CC	CC	CC	CT	CC	CT	CT	CT	CC	CT	CT	CT	CT
GG	AG	AG	AG	AG	AG	GG	AG	AG	AA	GG	GG	GG	AG
TT	CT	CT	CT	CT	CT	TT	TT	CT	CC	TT	TT	TT	CT
GG	AG	AG	AA	AG	AA	GG	GG	AG	AA	GG	GG	GG	AG
TT	CT	CT	--	TT	CC	TT	CT	CT	CC	--	TT	TT	CT
CG	CG	CC	GG	CC	GG	GG	CG	CG	GG	--	CC	--	CG
CT	CT	--	CC	TT	CC	--	CT	CT	CC	TT	TT	CT	CT
AC	AA	CC	AC	--	CC	AA	CC	AA	CC	--	AC	AC	AA
AG	GG	AA	AG	AG	AA	AG	AA	GG	AA	AG	AG	AG	GG
CT	TT	CC	CT	CT	CC	CC	CC	TT	CC	CT	CT	CT	TT
AG	AA	GG	AG	AG	GG	AG	GG	AA	GG	AG	AG	AG	AA
AG	AA	GG	AA	AG	AG	GG	GG	AA	GG	AG	AG	AG	AA
TT	TT	CT	TT	TT	CT	--	CT	TT	CT	CT	CT	CT	TT
AG	AG	AG	--	AG	AG	AA	AA	GG	AA	AA	AG	AA	GG
AG	AG	AA	AA	AA	AA	AG	AG	AA	AG	AG	AA	AG	AG
GT	GT	TT	TT	TT	TT	GT	GT	TT	GT	GT	TT	GT	GT
GT	GT	TT	TT	TT	TT	GT	GT	TT	GT	GT	TT	GT	GT
AC	AC	AC	AA	AA	AC	CC	CC	AA	CC	CC	AC	CC	AC
AG	AG	AG	AA	AA	AG	GG	GG	AG	GG	GG	AG	GG	AG

CG	CG	CG	--	CC	CG	GG	GG	CG	GG	GG	CG	GG	CG
CG	CG	CG	CC	CC	CG	GG	GG	CG	GG	GG	CG	GG	CG
CT	CT	CT	TT	TT	CT	CC	CC	CT	CC	CC	CT	CT	CT
CT	CT	CT	CT	CC	CT	TT	TT	CT	TT	TT	CT	CT	CT
AT	AT	AT	AT	AA	AT	TT	TT	AT	TT	TT	AT	AT	AT
GG	GG	AG	GG	AG	AG	GG	GG	GG	GG	GG	AG	AG	GG
CT	CT	CT	CT	CT	CT	TT	TT	--	CT	TT	CT	CC	CT
CT	CT	CT	CT	--	CT	CC	CC	CT	CT	CC	CT	TT	CT
TT	CT	CT	CT	CT	CT	--	TT	CT	CT	TT	CT	CC	CT
AA	AG	AG	AG	AG	AG	AA	AG	AG	AG	AG	AG	GG	AG
CG	CC	CG	--	CC	CG	CC	--	CC	CG	CC	CG	CC	CC
CG	CG	GG	CG	CG	GG	CG	GG	CG	GG	CG	GG	GG	GG
GT	GG	GT	GG	GG	GT	--	GT	GG	GT	GG	GT	GG	GG
CG	CG	CC	CG	CC	CG	CG	CC	CC	CC	CC	CG	CG	CC
AA	AA	AG	AG	AG	AG	--	AA	GG	GG	GG	AA	--	GG
AC	AC	AA	AC	AA	AC	AC	AC	AA	AA	AA	AC	AC	AA
AT	AT	AA	AT	AA	AT	AT	AA	AA	AA	AA	AT	AT	AA
AA	AA	GG	AG	AG	AG	GG	AG	GG	GG	GG	AG	AG	GG
TT	AT	AA	AT	AT	AT	AT	AT	AA	AA	AA	AT	AT	AA
AA	AG	GG	AG	GG	AG	AG	AG	GG	GG	GG	AG	AG	GG
AC	AC	CC	CC	CC	CC	--	CC	CC	CC	CC	CC	CC	AC
TT	CT	CC	TT	CC	CT	CT	CC	CT	CC	CT	CT	CT	CT
GT	GT	TT	--	TT	GT	GT	TT	GT	TT	GT	GT	GT	GT
GT	GT	TT	GG	TT	GT	GT	TT	GT	TT	GG	GT	GT	GT
CT	CC	--	TT	CC	CT	CT	CC	CT	CC	TT	CT	CT	CT
CG	CC	CC	GG	CC	CG	CG	CC	CG	CC	GG	CG	CG	CG
AG	GG	GG	--	GG	AG	AG	GG	AG	GG	AA	AG	AG	AG
CT	CC	CT	TT	CC	CT	CT	CC	CT	CC	TT	CT	CT	CT
GT	TT	GT	GG	TT	GT	GT	TT	GT	TT	GG	GT	GT	GT
CT	TT	CT	CC	TT	CT	CT	TT	CT	TT	CC	CT	CT	CT
CG	GG	CG	CC	GG	CG	CG	GG	CG	--	CC	CG	CG	CG
AG	GG	AG	AA	GG	AG	AG	GG	AG	GG	AA	AG	AG	AG
AC	AA	AC	CC	AA	AC	AC	AA	AC	AA	CC	AC	AC	AC
CT	TT	CT	CC	TT	CT	CT	TT	CT	TT	CC	CT	CT	CT
AG	GG	--	AG	GG	AG	GG	GG	GG	GG	AG	AG	AG	GG
AC	AA	AC	CC	AA	AC	CC	AA	AC	AA	CC	AC	AC	AC
CT	CC	CT	TT	CC	CT	TT	CC	CT	CC	TT	CT	CT	CT
AG	GG	AG	AA	GG	AG	AA	GG	AG	GG	AA	AG	AG	AG
CG	GG	CG	CC	GG	CG	--	GG	CG	GG	CC	CG	CG	CG
GT	GG	GT	TT	GG	GT	GT	GG	GT	GG	TT	GT	GT	GT
CT	CC	CT	TT	CC	CT	CT	CC	CT	CC	TT	CT	CT	CT
CT	TT	CT	CT	TT	CT	TT	TT	TT	TT	CT	CT	CT	TT
AG	AG	AG	--	AG	AG	--	AG	GG	--	--	GG	AG	GG
CG	CC	CG	GG	CC	GG	CG	CC	CG	CC	GG	GG	CG	CG
AT	AA	AA	AT	AA	AT	AT	AA	AT	AA	AT	AT	AT	AT
AG	AA	--	AG	AA	AG	AA	AA	AA	AA	AG	AG	AG	AA
AC	CC	AC	AC	CC	AC	CC	CC	CC	CC	AC	AC	AC	CC

CT	TT	CT	CT	TT	CT	TT	TT	TT	TT	CT	CT	CT	TT
CT	TT	CT	TT	TT	CT	CT	TT	CT	TT	CT	CT	CT	CT
AT	GG	AT	GT	GG	AT	--	GG	GG	GG	AT	AT	AT	AG
AT	AA	AT	AT	AA	AT	AA	AA	AA	AA	AT	AT	AT	AA
AA	GG	AA	AG	GG	AA	AG	GG	GG	GG	AG	AA	AA	GG
AA	CC	AC	AC	CC	AA	CC	CC	CC	CC	--	AA	AA	CC
AG	AA	AG	AG	AA	AG	AA	AA	AA	AA	AG	AG	AG	AA
AA	GG	--	--	GG	AA	--	GG	GG	GG	AG	AA	AA	AG
GG	AA	AG	GG	AA	GG	AA	AA	AA	AA	AG	--	GG	AG
AA	GG	AG	AA	GG	AA	--	--	AG	GG	AG	AA	AA	AG
TT	GT	GT	TT	GT	TT	GG	GG	GT	GG	GT	TT	TT	GT
AG	AG	GG	--	AG	GG	GG	GG	AG	GG	GG	AG	AG	AG
TT	CT	CT	--	CT	CT	--	CC	CT	CC	CT	--	TT	CC
GG	CG	CG	GG	CG	CG	CC	CC	CG	CC	CG	GG	GG	CC
CT	TT	CT	CT	TT	CT	TT	TT	TT	TT	CT	CT	CT	TT
CT	CT	TT	CT	CT	TT	TT	TT	CT	TT	TT	CT	CT	TT
CT	CT	CT	CT	CT	CC	CT	CC	CT	CC	CC	CC	CC	CT
GT	GT	GT	GG	GT	GG	GT	GT	GT	GT	GT	GT	GG	GT
AA	AA	AG	AG	AA	GG	AA	AG	AA	AG	AG	AG	GG	AA
GG	GG	AG	AG	GG	AA	--	AG	GG	AG	AG	AG	AA	GG
AG	AG	AG	--	AA	--	AG	AA	AG	AA	AA	AA	AA	AG
TT	TT	--	GT	TT	GG	--	GT	TT	GT	GT	GT	GG	TT
AG	AG	AG	AG	AG	AA	AA	AA	AG	AA	AA	AG	AA	AG
CT	CT	CT	CT	CT	CC	CT	CC	CT	CC	CC	CT	CC	CT
AG	AA	AA	--	AA	AG	--	--	AA	AG	AA	AA	AG	AA
AG	AA	AA	AG	AA	AG	AA	GG	AG	AG	AA	AG	GG	AA
AG	AA	AA	AG	AA	AG	--	GG	GG	AG	AG	AG	GG	AA
CG	CC	CC	CG	CC	CG	CG	GG	GG	CG	CG	CG	GG	CC
CG	CG	CC	--	CC	CG	CG	GG	GG	CG	CG	CG	GG	CC
AG	AG	AA	AA	AA	AG	AG	GG	GG	AG	AG	AG	AG	AA
AG	GG	AG	AG	AG	AG	--	GG	GG	AG	GG	GG	AG	AG
CT	CT	CC	CC	CC	CT	--	TT	TT	CT	CT	CT	CT	CC
AC	CC	AC	AC	AC	AC	--	CC	CC	AC	CC	CC	AC	AC
CG	CC	CG	CG	CG	CG	CC	CC	CC	--	CC	CC	CG	CG
AC	CC	AC	AC	AC	AC	CC	AC	CC	CC	CC	CC	AC	AC
AC	AC	CC	AC	CC	AA	AC	CC	AA	AC	AC	AC	AC	CC
AA	AG	AG	AG	AG	AA	AG	AA	AA	AG	AG	AG	AA	AG
AG	AG	GG	AG	GG	AA	AG	GG	AA	AG	AG	AG	AA	GG
GG	AG	AG	GG	AG	GG	AG	AG	GG	AG	AG	AG	GG	AG
CT	CT	TT	CT	TT	CC	CC	TT	CC	CT	CT	CT	CC	TT
CT	CT	TT	CT	TT	CC	--	TT	CC	CT	CT	CT	CC	TT
AG	AA	AG	AG	AG	AA	--	--	AA	AA	AA	AA	AA	--
CT	CC	CC	CT	CT	CC	CT	CT	CC	CC	CC	CC	CC	CT
AA	AG	AG	AG	AA	GG	--	GG	GG	AG	AA	--	AG	GG
AC	CC	AC	AC	AC	CC	CC	CC	CC	CC	AC	AC	AC	CC
CT	CC	CC	CC	CT	CC	CC	CC	CC	CT	CT	CT	CC	CC
AG	AA	GG	AG	GG	AA	AA	AA	AA	AG	GG	GG	AG	AG

AA	AA	AG	AA	AG	AA	AA	AA	AA	AG	AG	AG	AA	AA
AA	AA	GG	AG	GG	AA	AA	AA	AA	AG	AG	GG	AG	AG
CC	CC	AA	AC	AA	CC	AC	CC	AC	AC	AC	AA	AC	AC
GG	GG	AG	AG	AA	GG	AG	GG	AG	AG	AG	AA	AG	AG
TT	TT	GT	GT	GG	--	GT	TT	GT	--	GT	GG	GT	GT
AA	AA	AA	AG	AG	AA	AG	AA	AG	AA	AA	AG	AA	AG
TT	CT	CT	CT	CC	TT	CT	TT	CT	CT	CC	CC	TT	CT
CC	CT	CC	CT	CT	CC	--	CC	CT	CC	CT	CT	CC	CT
GG	AG	AG	AG	AA	GG	AG	GG	AG	AA	AA	AA	GG	AG
GG	GG	CG	GG	CG	GG	GG	GG	GG	CG	CG	GG	GG	GG
TT	TT	CT	--	CT	TT	--	TT	TT	--	TT	TT	TT	TT
AA	AG	GG	AG	GG	AA	AA	AA	AA	AG	AG	AG	AG	AG
AA	AG	GG	AG	GG	AA	AA	AA	AA	AG	AG	AG	AG	AG
CC	CG	GG	CG	GG	CC	--	CC	CC	CG	--	CG	CG	CG
AA	AG	GG	AG	GG	AA	AA	AA	AA	AG	--	AG	AG	AG
AA	GG	AG	GG	AG	AA	AA	AA	GG	AG	AG	AG	AA	AG
CC	TT	CT	TT	CT	CC	CT	CC	TT	CT	CT	CT	CC	CT
AA	GG	--	--	AG	AA	--	--	GG	AG	AG	AG	--	AG
GG	CC	CG	CC	CG	GG	CG	GG	CC	CG	CG	CG	GG	CG
AA	CC	AC	CC	AC	--	AC	AA	CC	AC	AC	AC	AA	AC
GG	AA	AG	AA	AG	GG	AG	GG	AA	AG	AG	AG	GG	AG
TT	GG	GT	GG	GT	TT	GT	TT	GG	GT	GT	GT	TT	GT
AA	GG	AG	GG	--	AA	AG	AA	GG	AG	AG	AG	AA	AG
AA	GG	AG	GG	AG	AA	AG	AA	GG	--	AG	AG	AA	AG
GG	AA	AG	AA	AG	GG	AG	GG	AA	AG	AG	AG	GG	AG
CC	AA	AC	AA	AC	CC	AC	CC	AA	AC	AC	AC	CC	AC
GG	AA	AG	AA	AG	GG	--	GG	AA	AG	AG	AG	GG	AG
GG	AA	AG	AA	AG	GG	AG	GG	AA	AG	AG	AG	GG	AG
CT	TT	CT	TT	CT	CC	CT	CC	TT	CT	CT	CT	CC	CT
CG	GG	CG	GG	CG	CC	--	CC	GG	CG	CG	CG	CC	CG
AC	AA	AA	AA	AC	CC	--	CC	AA	AC	--	AC	CC	AC
CT	TT	TT	TT	CT	CC	CT	CC	TT	CT	CT	CT	CC	CT
GT	TT	TT	--	GT	GG	GT	GG	TT	GT	GT	GT	GG	GT
CT	CC	--	CC	CT	TT	--	TT	CC	CT	CT	CT	TT	CT
CG	CC	CC	CC	CG	GG	CG	GG	CC	CG	CG	CG	GG	CG
AT	TT	TT	TT	AT	AA	--	AA	TT	AT	AT	AT	AA	AT
CT	CC	CC	--	CT	TT	CT	TT	CC	CT	CT	CT	TT	CT
AG	GG	GG	GG	AG	AA	AG	--	GG	AG	AG	AG	AA	AG
AG	AA	AA	--	AG	GG	AG	GG	AA	AG	AG	AG	GG	AG
AT	AA	--	AA	AT	TT	AA	TT	AA	AT	AT	AT	TT	AT
AG	GG	GG	--	AG	AA	AG	AA	GG	AG	AG	AG	AA	AG
AG	AA	AA	AA	AG	GG	AG	GG	AA	AG	AG	AG	GG	AG
CG	CC	CC	CC	CG	GG	CG	GG	CC	CG	CG	CG	GG	CG
AG	AA	AA	AA	AG	GG	AG	GG	AA	AG	AG	AG	GG	AG
CG	GG	GG	CG	CG	CC	--	CC	GG	CG	CG	CG	CC	CG
AG	GG	GG	AG	AG	AA	AG	AA	GG	AG	AG	AG	AA	AG
AC	AA	AA	AC	AC	CC	CC	CC	AA	AC	AC	AC	CC	AC

AG	GG	GG	AG	AG	AA	AG	AA	GG	AG	AG	AG	--	AG
CG	CC	CC	CG	CG	GG	CG	GG	CC	CG	CG	CG	GG	CG
CT	CC	CC	CT	CT	TT	CT	TT	CC	CT	CT	CT	TT	CT
AT	TT	TT	AT	AT	AA	--	AA	TT	AT	AT	AT	AA	AT
CT	CC	CC	CT	CT	TT	CT	TT	CC	CT	CT	CT	TT	CT
AT	AA	AA	--	AT	TT	AT	TT	AA	AT	AT	AT	TT	AT
AT	TT	TT	AT	AT	AA	--	AA	TT	AT	AT	AT	AA	AT
CG	GG	GG	CG	CG	CC	CG	CC	GG	CG	CG	CG	CC	CG
CT	CC	CC	CT	CT	TT	CT	TT	CC	CT	CT	CT	TT	CT
AG	AA	AA	AG	AG	GG	AG	GG	AA	AG	AG	AG	GG	AG
AC	AA	AA	AC	AC	CC	AC	CC	AA	AC	AC	AC	CC	AC
GT	TT	TT	GT	GT	GG	GT	GG	TT	GT	GT	GT	GG	GT
CT	CC	CC	CT	CT	TT	CT	TT	CC	CT	CT	CT	TT	CT
CG	GG	GG	CG	CG	CC	--	CC	GG	CG	CG	CG	CC	CG
GT	TT	TT	GT	GT	GG	GT	GG	TT	GT	GT	GT	GG	GT
CT	TT	TT	CT	CT	CC	CT	CC	TT	CT	CT	CT	CC	CT
AG	AA	AA	AG	AG	GG	--	GG	AA	AG	AG	AG	GG	AG
AC	AA	AA	AC	AC	CC	AC	CC	AA	AC	AC	AC	CC	AC
CT	CC	CC	CT	CT	TT	--	TT	CC	CT	CT	CT	TT	CT
AC	AA	AA	AC	AC	CC	AC	CC	AA	AC	AC	AC	CC	AC
AG	AA	AA	AG	AG	GG	AG	GG	AA	AG	AG	AG	GG	AG
GT	GG	GG	GT	GT	TT	GT	TT	GG	GT	GT	GT	TT	GT
GT	GG	GG	GT	GT	TT	GT	TT	GG	GT	GT	GT	TT	GT
AG	GG	GG	AG	AG	AA	AA	AA	GG	AG	AG	AG	AA	AG
CT	CC	CC	CT	CT	TT	--	TT	CC	CT	CT	CT	TT	CT
CT	CC	CC	CT	CT	TT	CT	TT	CC	CT	CT	CT	TT	CT
AG	AA	AA	AG	AG	GG	--	GG	AA	AG	AG	AG	GG	AG
CT	CC	CC	CT	CT	TT	CT	TT	CC	CT	CT	CT	TT	CT
CT	TT	TT	CT	CT	CC	CT	CC	TT	CT	CT	CT	CC	CT
AG	GG	GG	AG	AG	AA	AG	AA	GG	AG	AG	AG	AA	AG
CT	CC	CC	CT	CT	TT	TT	TT	CC	CC	CT	CT	TT	CT
TT	TT	TT	CT	CT	CT	CT	CT	TT	TT	TT	TT	CT	CT
CT	CC	CC	CT	TT	TT	--	CT	CT	CC	CT	CC	TT	TT
AG	AA	AA	AG	GG	GG	AG	AG	AG	AA	AG	AA	GG	GG
AC	AA	AC	AC	CC	CC	CC	AC	AC	AA	AC	AA	CC	CC
CC	CG	CC	CG	CC	CC	CC	CG	CC	CG	CC	CG	CC	CC
GT	GG	GT	GT	TT	TT	TT	GT	GT	GT	GT	GG	TT	TT
AG	GG	AG	AG	AA	AA	AA	AG	AG	AG	AG	GG	AA	AA
CT	TT	CT	CT	CC	CC	CC	CT	CT	CT	CT	TT	CC	CC
GG	GG	GG	AG	GG	GG	GG	AG	GG	AG	GG	GG	GG	GG
AG	AG	AA	AG	AA	AA	AA	AG	AG	AG	AG	AG	--	AA
GG	CG	CC	CG	CC	--	CC	CG	CG	CG	CG	CG	CC	CC
--	TT	TT	CT	TT	TT	TT	CT	TT	TT	--	TT	TT	TT
TT	GT	GG	GT	GG	GG	--	GT	GT	GG	GT	GT	GG	GG
GG	AG	AG	AG	AG	AG	AG	GG	AG	AG	GG	GG	GG	GG
AG	AG	GG	GG	AG	AG	GG	GG	AG	AG	AG	GG	AG	GG
AC	CC	AC	CC	CC	AC	--	AA	CC	AC	AC	AA	AC	AA

AT	AA	AA	--	AA	AT	AT	TT	AA	AT	AT	TT	AT	TT
AT	AA	AA	AA	AA	AT	--	TT	AA	AT	AT	TT	TT	TT
AT	TT	TT	AT	TT	AT	AT	--	TT	AT	AT	AA	AA	AA
CT	CC	CC	CT	CC	CT	CC	TT	CC	CT	CT	TT	TT	TT
AA	AA	AG	AG	AA	AG	AG	GG	AA	AG	AG	GG	GG	AG
CC	CC	AC	AC	CC	AC	AC	AA	CC	AC	AC	AA	AA	AC
CC	CC	CT	CT	CT	CT	--	TT	CC	CT	--	TT	TT	CT
AA	AA	AG	AG	GG	AG	AG	AG	AA	AG	GG	GG	GG	AG
TT	GT	GT	GT	GG	GT	GT	GT	TT	GT	GG	GT	GT	GT
AA	AG	GG	AG	GG	AG	AG	AG	AA	AG	AG	AG	AG	AG
CG	CG	CC	CC	CC	CC	CG	CG	CG	CG	CG	CG	CG	CG
AG	AG	GG	GG	GG	GG	AG	AG	AG	AG	AG	AG	AG	AG
GG	CG	CC	CG	CC	CG	CG	CG	CG	CG	CG	CG	CG	CG
GG	AG	AA	AG	AA	AG	AA	AG	AG	AG	GG	AG	AG	AG
AA	--	GG	AG	GG	AG	--	AG	AG	AG	AA	AG	AG	AG
GG	AG	AA	AG	AA	AG	AG	AG	AG	AG	GG	AG	AG	AG
GT	GT	GG	GG	GG	GG	GT	GT	GT	GT	GT	GT	GT	GT
AA	AA	GG	AG	GG	AG	AG	AA	AG	AG	AA	AG	AG	AG
GT	GT	TT	--	TT	TT	GT	GT	GT	GT	GT	GT	GT	GT
CC	CC	GG	CG	--	GG	CG	CC	CC	CG	CC	CC	CC	CC
CT	CT	CT	--	TT	--	--	CT	CT	TT	--	TT	CT	TT
CG	CC	CC	--	CG	CG	--	CC	CC	CG	CG	CG	CG	GG
CC	CG	CG	CG	CG	CC	--	CG	CG	CC	CG	CG	CC	CC
AC	AC	AC	AA	AA	AC	AC	AC	AC	AA	AA	AA	AC	AA
CT	CT	CT	--	CT	CT	CC	CT	CT	--	--	CC	CT	CC
AG	AG	AG	AG	AG	AG	AG	AG	AG	AG	AA	AA	AG	AA
CT	CT	CT	CT	CT	CT	CT	CT	CT	--	CC	CC	CT	CC
GG	CG	CG	CG	CG	GG	GG	CG	GG	CG	CG	CG	GG	CG
CT	CT	CT	CT	CT	CT	CC	CT	CT	CT	CT	CC	CT	CC
AG	GG	GG	GG	AG	AG	AA	AG	AG	GG	AG	AG	AA	AG
GT	GT	GT	GT	GG	GT	GG	GT	GT	GT	GT	GG	GG	GG
GT	GG	GG	GG	TT	GT	TT	GT	GT	GG	GT	GT	TT	GT
CG	CG	CG	CG	GG	CG	GG	CG	CG	CG	CG	GG	GG	GG
CG	CC	CC	CC	GG	CG	GG	CG	CG	CC	CG	CG	GG	CG
AG	AG	AG	AG	GG	AG	GG	AG	AG	AG	AG	GG	GG	GG
CT	CC	CC	--	TT	CT	TT	CT	CT	CC	CT	CT	TT	CT
GT	TT	TT	TT	GG	GT	GG	GT	GT	TT	GT	GT	GG	GT
AG	AG	AG	AG	GG	AG	GG	AG	AG	AG	AG	GG	GG	GG
AG	GG	GG	--	AA	AG	AA	AG	AG	GG	AG	AG	AA	AG
CT	CC	CC	--	TT	CT	TT	CT	CT	--	CT	CT	TT	CT
AT	AT	AT	AT	TT	AT	TT	AT	AT	AT	AT	TT	TT	TT
GT	GT	GT	GT	TT	GT	--	GT	GT	GT	GT	TT	TT	TT
CT	TT	TT	--	CC	CT	CC	CT	CT	TT	CT	CT	CC	CT
AG	AG	AG	AG	AA	AG	AA	AG	AG	AG	AG	AA	AA	AA
AT	AA	AA	AA	TT	AT	TT	AT	AT	AA	AT	AT	TT	AT
AG	AG	AG	AG	AA	AG	AA	AG	AG	AG	AG	AA	AA	AA
CT	CT	CT	CT	TT	CT	CT	CT	CT	CT	CT	TT	TT	TT

AT	AT	AT	AT	TT	AT	AT	--	AT	AT	AT	TT	TT	TT
GT	TT	TT	TT	GG	GT	GT	GT	GT	TT	GT	GT	GG	GT
AA	GG	GG	GG	AA	AG	AG	AG	AG	GG	AG	AG	AA	GG
CC	TT	TT	TT	CC	CT	CT	CT	CC	TT	CT	CT	CC	TT
CC	TT	TT	CT	CC	CT	CT	CT	CC	TT	CT	CT	CC	CT
CC	TT	TT	CT	CC	CT	CT	CT	--	--	--	CT	CC	CT
GG	TT	TT	GT	GG	GT	GT	GT	GG	TT	GT	GT	GG	GT
CC	GG	GG	CG	CC	CG	CG	CC	CC	GG	CG	CG	CC	CG
CC	AA	AA	AC	CC	AC	--	CC	CC	AA	AC	AC	CC	AC
AA	GG	GG	AG	AA	AG	AA	AA	AA	AG	AG	AG	AA	AG
GG	AA	AA	AG	GG	AG	--	GG	GG	AG	AG	AG	GG	AG
CC	CC	TT	TT	CC	TT	TT	CC	CC	TT	TT	TT	CT	TT
CC	CC	AA	AA	CC	AA	--	CC	CC	AA	AA	AA	AC	AA
GT	GT	TT	TT	GT	TT	TT	GT	GT	TT	TT	TT	TT	TT
CG	CG	GG	--	CG	GG	--	GG	CG	GG	GG	GG	CG	GG
AG	AA	GG	AG	AG	GG	GG	AG	AG	--	GG	GG	AG	GG
CT	CT	TT	CT	TT	TT	TT	TT	TT	TT	TT	CT	CT	TT
TT	GT	TT	TT	GT	TT	TT	GT	GT	GT	GT	TT	TT	TT
CC	CT	CC	CT	CC	CC	CT	CC	CC	CT	CC	CT	CT	CC
AA	AC	AA	AC	AC	AA	--	AA	AC	AC	AC	AC	AA	AA
AA	GG	AG	GG	AG	AA	AG	AA	AG	GG	AG	GG	AG	AA
AA	AG	AG	AG	AG	AA	AA	AA	AG	AG	AG	AG	AA	AA
TT	GG	GG	GG	GT	GT	GG	TT	GT	GG	GT	GG	GT	TT
CC	TT	TT	--	CT	CT	--	CC	--	--	CT	TT	CT	CC
AA	CC	CC	CC	AA	AC	CC	AC	AA	CC	AC	CC	CC	AA
TT	AA	AA	--	TT	AA	AA	AT	TT	AA	AT	AA	AA	AT
GT	GG	GG	--	GT	GG	--	GG	GT	GG	GG	GG	GG	GT
AG	AA	AA	--	AG	AA	AA	AG	AG	AA	AG	AA	AA	AG
CC	GG	GG	GG	CC	GG	GG	CG	CC	GG	CG	GG	GG	CC
AA	GG	GG	--	AA	--	GG	AG	AA	--	AG	GG	GG	AA
CG	CG	CG	CG	CG	CG	CC	CG	CG	CG	CC	CG	CG	CG
AG	AG	AG	AG	AG	AG	AA	AG	AG	AG	AA	AG	AG	AG
AG	AA	AA	AA	AA	AG	AA	AA	AG	AA	AA	AA	AG	--
AA	AT	AT	--	AT	--	AA	AT	AA	AT	--	AT	AA	AA
AG	AA	AA	--	AA	AG	--	AA	AG	AA	AA	AA	AG	AG
AA	AG	AG	AG	AG	AA	AA	AG	AA	AG	AG	AG	AA	AA
AC	AC	AA	AA	AC	AC	AC	AA	AC	AC	AA	AC	AC	AC
CC	AC	CC	CC	AC	CC	AC	CC	CC	AC	CC	AC	CC	CC
CC	AC	CC	--	AC	AC	AA	CC	AC	--	--	AC	AC	AC
GT	GT	GG	--	GT	TT	TT	GG	GT	GG	GG	GT	GT	TT
AT	AT	AA	--	AT	--	TT	AA	AT	--	AA	AT	AT	TT
AG	AG	AA	AA	AA	AA	AG	AA	AA	AA	AA	AG	AA	AG
AC	AC	CC	CC	AC	AC	AC	CC	CC	CC	CC	AC	CC	AC
AT	AT	AA	--	AA	AT	TT	AA	AT	AA	AA	AT	AT	TT
CT	CT	CC	CC	CC	CT	TT	CC	CT	CC	CC	CC	CT	TT
AG	AG	GG	GG	GG	AG	AA	GG	AA	GG	GG	GG	AG	AA
CT	CT	CC	CC	CC	CT	TT	CC	TT	CC	CC	CC	CT	TT

--	CT	TT	--	TT	CT	CC	TT	CC	TT	--	TT	CT	CC
AG	AG	GG	GG	GG	AG	--	GG	AA	GG	AG	GG	AG	AA
CT	CT	CC	CC	CC	CT	TT	CC	TT	CC	CT	CC	CT	TT
CT	CT	TT	TT	TT	CT	CC	TT	CC	TT	CT	TT	CT	CC
CT	CT	CC	CC	CC	CT	TT	CC	TT	CC	CT	CC	CT	TT
AG	AG	GG	GG	GG	AG	AA	GG	AA	GG	AG	GG	AG	AA
CT	CT	TT	TT	TT	CT	--	TT	CC	TT	CT	TT	CT	CC
AG	AG	GG	GG	GG	AG	AA	GG	AA	GG	AG	GG	AG	AA
CG	CG	CC	CC	CC	CG	GG	CC	GG	CC	CG	CC	CG	GG
AC	AC	AA	AA	AA	AC	--	AA	CC	AA	AC	AA	AC	CC
CT	CT	CC	CC	CC	CT	TT	CC	TT	CC	CT	CT	CT	TT
CT	CT	CC	--	CC	CT	TT	CC	TT	CC	CT	CT	CT	TT
AT	AT	TT	TT	TT	AT	AA	TT	AA	TT	AT	AT	AT	AA
CT	CT	CC	CC	CC	CT	--	CC	TT	CC	CT	CT	CC	TT
AC	AC	AA	AA	AA	AC	CC	AA	CC	AA	AC	AC	AC	CC
GT	GT	TT	TT	TT	GT	GG	TT	GG	TT	GT	GT	GT	GG
CT	CT	TT	--	TT	CT	--	TT	CC	TT	CT	CT	CT	CC
CG	CG	GG	GG	GG	CG	CC	GG	CC	GG	CG	CG	CG	CC
CG	CG	GG	GG	GG	CG	CC	GG	--	GG	--	CG	--	CC
AC	AC	CC	CC	CC	AC	AA	CC	AA	CC	AC	AC	AC	AA
CG	CG	GG	GG	GG	CG	--	GG	CC	GG	CG	CG	CG	CG
AT	AT	AT	AT	AT	TT	AA	AT	AT	AA	AT	TT	AT	AT
AG	AG	AG	AG	AG	GG	--	AA	AG	--	AG	GG	AG	AG
AG	--	AG	AA	AG	AG	AA	AG	AG	AA	AG	AG	AG	AG
AG	AG	AG	GG	AG	AG	GG	GG	AG	GG	AG	AG	AG	GG
AG	AG	AG	AA	AA	AG	AA	AG	AG	AA	AG	AG	AG	AA
AC	AC	--	AA	AA	AC	--	AC	AC	AA	AC	AC	AC	AA
AG	AG	AG	--	AA	AG	--	AG	AG	AA	AG	AG	AG	AA
AG	AG	AG	AA	AA	AG	AA	AG	AG	AA	AG	AG	AG	AA
GT	GT	GT	GG	GG	GT	GG	GT	GT	GG	GT	GT	GT	GG
GT	GT	GT	TT	TT	GT	TT	GT	GT	TT	GT	GT	GT	TT
AC	AC	AC	CC	CC	AC	CC	AC	AC	CC	AC	AC	AC	CC
CT	CT	CT	TT	TT	CT	--	CT	CT	TT	CT	CT	CT	TT
CT	CT	CT	--	TT	CT	TT	CT	CT	TT	CT	CT	CT	TT
GT	GT	GT	GG	GG	GT	GG	GT	GT	GG	GT	GT	GT	GG
AT	AT	AT	AA	AA	AT	--	AT	AT	AA	AT	AT	AT	AA
CG	CG	CG	GG	GG	CG	GG	CG	CG	GG	CG	CG	CG	GG
AC	AC	AC	AA	AA	AC	AA	AC	AC	AA	--	AC	AC	AA
CT	CT	CT	CC	CC	CT	CC	CT	CT	CC	CT	CT	CT	CC
CG	CG	CG	GG	GG	CG	GG	CG	CG	GG	CG	CG	CG	GG
CT	CT	TT	CC	CC	CT	--	--	CT	CC	CT	CT	CT	CC
AT	AT	AT	AA	AA	AT	--	AT	AT	AA	AT	AT	AT	AA
CT	CT	CT	TT	TT	CT	TT	CT	CT	TT	CT	CT	CT	TT
AG	AG	AG	GG	GG	AG	GG	AG	AG	GG	AG	AG	AG	GG
AT	AT	AT	--	AA	--	AA	AT	AT	AA	AT	AT	AT	AA
AT	AT	--	AA	AA	AT	AA	TT	AT	AA	--	AT	AT	AA
AC	AC	AC	AA	AA	AC	AC	AC	AC	AA	AC	AC	AC	AA

GT	GT	GT	TT	TT	GT	TT	GT	GT	TT	GT	GT	GT	TT
AG	AG	AG	GG	GG	AG	--	AG	AG	GG	AG	AG	AG	GG
GT	GT	GT	GG	GG	GT	GG	GT	GT	GG	GT	GT	GT	GG
AG	AG	AG	AA	AA	AG	AA	AG	AG	AA	AG	AG	AG	AA
CT	CT	CT	TT	TT	CT	TT	CT	CT	--	--	CT	CT	--
AG	AG	AG	AA	AA	AG	AA	AG	AG	AA	AG	AG	AG	AA
CT	CT	CT	TT	TT	CT	TT	CT	CT	TT	CT	CT	CT	TT
AC	AC	--	CC	CC	AC	--	AC	AC	--	AC	AA	AC	CC
AG	AG	--	AA	AA	AG	AA	AG	AG	AA	AG	GG	AG	AA
CT	CT	CT	TT	TT	CT	TT	CT	CT	TT	CT	CC	CT	TT
AG	AG	AG	GG	GG	AG	GG	AG	AG	GG	AG	AA	AG	GG
CT	CT	CT	--	CC	CT	CT	CT	CT	CC	TT	TT	CC	--
GG	GG	GT	GG	GG	GT	GG	GG	GT	GG	GT	GT	GG	GG
AG	AG	GG	GG	GG	GG	AG	AG	GG	GG	AG	AG	GG	GG
TT	AT	AT	TT	TT	AT	TT	TT	AT	TT	--	AA	TT	AT
TT	AT	AT	--	TT	AT	AT	TT	AT	TT	AA	AA	TT	AT
CC	AC	AC	CC	CC	AC	AC	CC	AC	CC	--	AA	AC	AC
GG	GT	GT	--	GG	GT	GT	GG	GT	GG	GT	TT	GT	GT
CT	CT	TT	CT	TT	CT	TT	CT	CT	TT	CT	TT	CT	--
AG	AG	--	AG	AG	AG	--	AA	AA	AG	AG	AG	AA	AA
CG	CG	GG	CG	GG	CG	CG	GG	GG	GG	CG	GG	GG	GG
CT	CT	TT	CT	TT	CT	CT	TT	TT	TT	CT	TT	TT	CT
GG	GG	GG	GG	GG	GG	GT	GG	GG	GT	GG	GT	GG	GG
AG	AG	AA	AG	AA	AG	GG	AG	AA	AG	AG	AG	AA	AG
AC	AC	--	AC	AA	AC	AC	--	AA	--	AC	AA	AA	AC
CG	CG	GG	CG	GG	CG	CG	CG	GG	CG	--	GG	GG	--
GT	GT	TT	GT	TT	GT	GT	GT	TT	GT	GT	TT	TT	GT
CT	CT	CC	CT	CC	CT	TT	TT	CC	TT	CT	CT	CC	CT
AA	AA	AG	AG	AG	AA	AG	AG	AG	AG	AA	AG	AG	AA
CT	CT	CC	CC	CC	CT	CT	CT	CC	CT	CT	CC	CC	CT
CG	CG	CG	CG	CG	CC	CC	CC	CG	CC	CC	CG	CG	--
AC	AC	AC	AC	AC	AC	--	AA	AC	AA	AC	AC	AC	AC
GG	GG	GG	GG	GG	AG	AG	AA	GG	AA	AG	GG	GG	GG
TT	TT	TT	--	TT	TT	CT	CT	TT	CT	TT	TT	TT	TT
GG	GG	GG	GG	GG	CG	CG	CG	GG	CG	CG	GG	GG	GG
AA	AA	AA	AA	AA	AG	GG	GG	AA	GG	AG	AA	AA	AA
CG	CG	CG	CG	CG	GG	GG	GG	CG	GG	--	CG	CG	GG
CC	CC	CC	CC	CC	CG	GG	GG	CC	GG	CG	CC	--	CG
CC	CC	CC	--	CC	CT	--	TT	CC	TT	CT	CC	CC	CT
GG	GG	GG	GG	GG	AG	AA	AA	GG	AA	AG	GG	GG	AG
AG	AG	AG	AG	AG	AG	GG	GG	AG	GG	AG	AG	AG	AG
CC	CC	CC	CC	CC	CT	TT	TT	CC	TT	CT	CC	CC	CC
GG	GG	GG	GG	GG	GG	AG	AG	GG	AG	GG	GG	GG	GG
GG	GG	GG	GG	GG	AG	--	AA	GG	AA	AG	GG	GG	GG
CC	AC	CC	CC	CC	AC	--	AA	CC	AA	AC	CC	CC	CC
TT	AT	TT	TT	TT	AT	AT	AT	TT	AT	AT	TT	TT	TT
AA	AG	AA	AA	AA	AG	GG	GG	AA	GG	AG	AA	AA	AA

AA	AC	AA	AA	AA	AC	--	CC	AA	CC	AC	AA	AA	AA
AA	AG	AA	AA	AA	AG	GG	AG	AA	GG	AG	AA	AA	AA
AA	AA	AA	--	AA	AA	AG	AG	AA	AG	AA	AA	AA	AA
CC	CT	CC	CC	CC	CT	CT	CC	CC	CT	CT	CC	CC	CC
CC	CG	CC	CC	CC	CG	CG	CC	CC	CG	CG	CC	CC	CC
TT	CT	TT	TT	TT	CT	CT	--	CT	--	CT	TT	TT	--
GG	GG	CG	CG	CG	GG	GG	CG	GG	GG	CG	CG	CG	CG
CT	CT	CC	CC	CC	CT	CT	CC	CT	CT	CC	CC	CT	CC
GG	GG	GT	GT	GT	--	GG	GT	GG	--	GT	GT	GG	GT

6-28	6-30	6-32	6-33	6-34	6-35	6-36	6-38	6-39	6-40	6-45	6-49	6-50
CC	AC	AC	CC	AC	CC	AA	CC	CC	AC	AC	AC	AC
AG	AG	AG	GG	GG	GG	AA	GG	GG	AG	AG	AG	AG
CT	CT	CT	CC	CC	CC	TT	CC	CC	CT	CT	CT	CT
GG	GG	GG	AG	AG	AG	GG	AG	AG	AG	GG	GG	GG
AG	AG	AG	AG	--	GG	AA	GG	GG	AG	AG	AG	AG
GG	GG	GG	CG	GG	CG	CG	GG	--	CG	GG	GG	GG
CT	CT	CT	CT	CC	CT	TT	CC	--	CT	CT	CT	CT
GT	GT	GT	TT	GG	GT	TT	GG	GG	GT	GT	GT	GT
GT	GT	GT	TT	GG	GT	TT	GG	GG	GT	TT	GT	GT
GT	GT	GT	TT	GG	GT	TT	GG	GG	GT	TT	GT	GT
AG	AG	AG	AA	GG	AG	AA	GG	GG	AG	AA	AG	AG
CC	CC	CC	CC	--	CT	CT	CC	CC	CT	CT	CC	CC
AA	AC	CC	AC	AC	AC	AA	CC	CC	AC	AA	AC	AC
AG	AA	AA	AA	AA	AG	AG	AA	AA	--	AG	AA	AA
CT	CT	CC	CT	CT	CC	CT	CC	CC	CC	CT	CT	CT
GG	AG	AA	AG	AG	AG	GG	AA	AA	AG	AG	AG	AG
AT	TT	TT	TT	TT	AT	TT	TT	TT	AT	TT	TT	TT
CT	CT	CC	CT	CT	CC	CT	CT	--	CC	CC	CT	CT
TT	TT	TT	TT	TT	GT	TT	TT	TT	TT	TT	GT	TT
CC	CC	CC	CC	CC	CG	CC	CC	--	CC	CC	CG	CC
AT	AT	TT	AT	AA	AT	AT	AA	--	--	TT	AA	AA
CT	CT	TT	CT	CC	CT	CT	CC	CT	TT	TT	CC	CC
AT	AT	AA	AT	TT	AT	AT	TT	AT	AA	AA	TT	TT
AT	AT	AA	AT	TT	AT	AT	TT	AT	AA	AA	TT	TT
AG	AG	AA	AG	GG	AG	AG	GG	AG	AA	AA	GG	GG
TT	TT	TT	TT	GT	GT	TT	GT	GT	TT	TT	GT	GT
GT	GT	TT	GT	GG	GT	GT	GG	GT	TT	TT	GG	GG
AT	AT	TT	AT	AA	AT	AT	AA	AT	TT	TT	AA	AA
CT	CT	TT	CT	CC	CT	CT	CC	CT	TT	TT	CC	CC
CG	CG	CC	CG	GG	CG	CG	GG	CG	CC	CC	GG	GG
CT	CT	CC	CT	TT	CT	CT	TT	CT	CC	CC	TT	TT
AC	AC	AA	AC	CC	AC	AC	CC	--	AA	AA	CC	CC
AG	AG	AA	AG	GG	AG	AG	GG	AG	AA	AA	GG	GG
GT	GT	GG	GT	TT	GT	GT	TT	GT	GG	GG	TT	TT
AG	AG	AA	AG	GG	AG	AG	GG	--	AA	AA	GG	GG
GT	GT	TT	GT	GG	GT	GT	GG	GT	--	TT	GG	GG
AG	AG	GG	AG	AA	AG	AG	AA	GG	GG	GG	AA	AA
CT	CT	CC	CT	TT	CT	CT	TT	CT	CC	CC	TT	TT
AT	AT	AA	AT	TT	AT	AT	TT	AT	AA	AA	TT	TT
AG	AG	AA	AG	GG	AG	AG	GG	AG	AA	AA	GG	GG
AC	AC	AA	AC	CC	AC	AC	CC	AC	AA	AA	CC	CC
AG	AG	AA	AG	GG	AG	AG	GG	AG	AA	AA	GG	GG

CT	CT	TT	CT	CC	CT	CT	CC	--	TT	TT	CC	CC
CG	CG	CC	CG	GG	CG	CG	GG	CG	CC	CC	GG	GG
CT	CT	TT	CT	CC	CT	--	CC	CT	TT	TT	CC	CC
CT	CT	CC	CT	TT	CT	--	TT	CT	CT	CC	TT	TT
AC	AC	AA	AC	CC	AC	AC	CC	AC	AC	AA	AC	CC
CT	CT	CC	TT	CT	CC	CT	TT	CT	CT	CT	CT	CT
CT	CT	--	CC	CT	TT	CT	CT	CT	CT	CT	CT	CT
AG	AG	GG	AA	GG	AG	AG	GG	AG	GG	AG	AG	AG
GT	GT	GT	TT	GT	GT	GT	GG	GT	GG	GT	GG	GT
--	AG	AG	AG	AG	GG	AG	GG	AG	GG	AG	GG	AG
TT	CT	CT	--	CT	TT	CT	TT	--	TT	CT	TT	CC
CC	CG	CG	GG	CG	CC	CG	CC	GG	CC	CG	CC	GG
AA	AG	AG	GG	AG	AA	AG	AA	GG	AA	AG	AG	GG
CC	AC	AC	AA	AC	CC	--	CC	AA	CC	AC	AC	AC
AG	AG	AG	GG	AG	AG	AG	AG	--	AG	GG	AG	GG
CT	CT	CT	CC	CT	TT	TT	TT	CT	TT	CT	CT	CT
CT	CC	CT	TT	CC	CC	CC	CC	CT	CC	CT	CT	CT
CT	TT	TT	CT	TT	TT	TT	TT	CT	TT	CT	CT	CT
AG	AA	AA	AG	AA	AA	AA	AA	AG	AA	AG	AG	AG
AC	AA	AA	AC	AA	AA	AA	AA	AC	AA	CC	AC	AC
AG	AA	AA	AG	AA	AA	AA	AA	AG	AA	GG	AG	GG
CG	GG	GG	CG	GG	GG	GG	GG	CG	GG	CC	CG	CC
CG	CC	CC	CG	CC	CC	CC	CC	CG	CC	GG	CG	GG
AG	AA	AA	AG	AA	AA	AA	AA	AA	AA	GG	AA	GG
AC	CC	AC	AC	CC	CC	CC	CC	--	AC	AA	AC	AC
CT	TT	CT	CT	TT	TT	TT	TT	TT	CT	CC	CT	CT
CT	CC	CT	CT	CC	CC	CC	CC	CC	CT	TT	CT	CC
AT	TT	AT	AT	AT	TT	TT	TT	TT	AA	AA	AT	TT
GG	GG	GG	AG	GG	GG	GG	GG	--	--	AG	AG	GG
AT	AT	AT	TT	AT	TT	TT	TT	TT	AT	AT	TT	TT
GT	TT	GT	GT	GT	TT	TT	GT	--	--	GG	GG	TT
GG	GG	AG	AG	AG	GG	GG	AG	GG	GG	AG	AG	GG
TT	TT	CT	CT	CT	TT	TT	CT	TT	TT	CT	CT	TT
GG	GG	AG	AG	AG	GG	GG	AG	GG	--	AG	AG	GG
GT	GG	GT	GT	GT	GG	GG	GT	GT	GT	TT	TT	GG
--	GG	GT	GT	--	GG	GG	GG	--	GT	GT	GT	GG
CT	TT	CT	CT	CT	TT	TT	TT	CT	CT	CT	CC	TT
--	TT	CT	CT	CT	--	CT	TT	CT	CT	CT	CC	TT
AG	AA	AG	AG	AG	AA	AG	AA	GG	AG	AG	GG	AA
CG	CG	CG	GG	CG	GG	CG	GG	CC	CG	GG	CC	GG
AG	AG	AG	AG	AG	AA	AG	AA	GG	AG	AA	GG	AA
CT	CT	CT	CT	CT	CC	CT	CC	TT	CT	CC	TT	CC
CT	CT	CT	CT	CT	CC	TT	CC	--	CT	CC	TT	CC
CC	CG	CG	CC	CG	CC	CG	CC	CG	CG	CC	CG	CC
TT	AT	AT	AT	AT	TT	AT	TT	AT	AT	TT	AT	TT
GT	GG	GG	GT	GG	GG	GT	GG	GT	GT	GG	GT	GG
CT	CC	CC	CT	CC	CC	CT	CC	CT	CT	CC	CT	CC

CT	CT	CT	TT	CT	CT	TT	CC	TT	TT	CC	TT	CT
GT	GG	GG	GT	GG	GG	GT	GG	--	GT	GG	GT	GG
TT	CT	CT	TT	CT	CT	TT	CC	TT	TT	CC	TT	CT
GT	TT	TT	GT	TT	TT	GT	TT	GT	GT	TT	GT	TT
CG	GG	GG	CG	GG	--	CG	GG	CG	CG	GG	CG	GG
--	AA	AG	AG	AG	AA	AA	AG	GG	AG	GG	GG	AG
AG	GG	AG	AG	AG	GG	GG	AG	AA	AG	AA	AA	AG
AG	GG	AG	AG	GG	GG	GG	AA	AA	AG	AA	AA	AG
CT	CT	CT	CT	CC	CC	CC	TT	TT	CT	TT	TT	CT
AG	AG	AG	AG	AA	AA	AG	GG	GG	AG	GG	GG	AG
GT	GT	GT	GT	TT	TT	GT	GG	--	GT	GG	GG	GT
AG	AG	AG	AG	GG	AG	AG	AG	AG	GG	AG	AG	AG
CT	CT	CT	TT	CC	CT	TT	TT	TT	CT	TT	TT	CC
AC	AC	AC	CC	AC	AC	CC	CC	--	CC	CC	CC	AC
CC	CC	CC	CT	CC	CC	CT	CT	CT	CT	CT	CT	CC
GT	GT	GT	GG	TT	GT	GG	GG	--	GT	GG	GG	TT
CC	CC	CC	CC	CC	CC	CC	CC	CC	CT	CC	CC	CT
CG	CG	CG	GG	CG	CG	GG	GG	GG	GG	GG	GG	CG
AC	AC	AC	CC	AC	CC	CC	CC	--	CC	CC	CC	AC
TT	TT	TT	TT	TT	TT	TT	TT	TT	TT	TT	TT	CT
AA	AA	AA	AA	AA	AA	AA	AA	AA	AA	AA	AA	AG
AG	AG	AG	AA	AG	AG	AA	AA	AA	AA	AA	AA	GG
CC	CC	CC	CT	CC	CC	CT	CT	CT	CT	CT	CT	CC
AT	AT	AT	AA	AT	AT	AA	AA	AA	AT	AA	AA	AT
CT	CT	CT	CC	CT	CT	CC	CC	--	CT	CC	CC	CT
AG	AG	AG	AA	AG	AG	AA	AA	AA	AA	AA	AA	AG
CC	CC	CC	CC	CC	CC	CC	CC	CC	CC	CC	CC	CT
AT	AT	AT	TT	AT	AT	TT	TT	TT	TT	TT	TT	AA
AT	AT	AT	TT	AT	AT	TT	TT	--	TT	TT	TT	AA
CT	CT	CT	TT	CT	CT	TT	TT	TT	TT	TT	TT	CT
CT	CT	CT	CC	CT	CT	CC	CC	CC	CC	CC	CC	CT
GG	GG	GG	GG	GG	GG	GG	GG	GG	GG	GG	GG	GG
CC	CC	CC	CC	CC	CC	CC	CC	CC	CC	CC	CC	CC
AG	AG	AG	AG	AG	AG	AG	AG	AG	AG	AG	AG	AG
AG	AG	AG	GG	AG	AG	GG	GG	GG	GG	GG	GG	AG
AA	AA	AA	AA	AA	AA	AA	AA	AA	AA	AA	AA	AA
CT	CT	CT	CC	CT	CT	CC	CC	--	CC	CC	CC	CT
CC	AC	CC	CC	CC	CC	CC	CC	CC	CC	CC	CC	CC
AT	AA	AT	TT	AT	AT	TT	TT	--	TT	TT	TT	AT
AG	AG	AG	AA	AG	AG	AA	AA	AA	AA	AA	AA	AG
AA	AC	AA	AA	AA	AA	AA	AA	AA	AA	AA	AA	AA
TT	AT	TT	TT	TT	TT	TT	TT	TT	TT	TT	TT	TT
CC	AC	CC	AC	CC	CC	AC	AC	AC	AC	AC	AC	CC
AC	AC	AC	CC	AC	AC	CC	CC	CC	CC	CC	CC	AC
AG	GG	AG	AG	AG	AG	AG	AG	AG	AG	AG	AG	AG
CC	CC	CC	AC	CC	CC	AC	AC	AC	AC	AC	AC	CC
AA	AG	AA	AA	AA	AA	AA	AA	AA	AA	AA	AA	AA

CG	CG	CG	CC	CG	CG	CC	CC	CC	CC	CC	CC	CG
CT	CC	CT	TT	CT	CT	TT	TT	TT	TT	TT	TT	CT
CT	TT	CT	CC	CT	CT	CC	CC	--	CC	CC	CC	CT
CC	CT	CC	CC	CC	CC	CC	CC	CC	CC	CC	CC	CC
AA	AG	AA	AA	AA	AA	AA	AA	AA	AA	AA	AA	AA
CT	TT	CT	CC	CT	CT	CC	CC	--	CC	CC	CC	CT
--	CT	TT	TT	TT	TT	TT	TT	TT	TT	TT	TT	TT
AT	TT	AT	AA	AT	AT	AA	AA	AA	AA	AA	--	AT
AG	AG	AG	GG	AG	AG	GG	GG	GG	GG	GG	GG	AG
AA	AC	AA	AA	AA	AA	AA	AA	AA	AA	AA	AA	AA
--	CT	CC	CC	CC	CC	CC	CC	--	CC	CC	CC	CC
GT	GT	GT	TT	GT	GT	TT	TT	TT	TT	TT	TT	GT
--	CT	TT	TT	TT	TT	TT	TT	--	TT	TT	TT	TT
AC	CC	AC	AC	AC	AC	CC	AC	AC	AC	AC	AC	AC
AA	AG	AA	AA	AA	AA	AG	AA	--	AA	AA	AA	AA
GG	GT	GG	GG	GG	GG	GT	GG	GG	GG	GG	GG	GG
AT	TT	AT	AT	TT	AT	TT	AT	AT	AT	AT	AT	AT
AA	AG	AA	AA	AA	AA	AG	AA	AA	AA	AA	AA	AA
CT	CT	CT	CC	CT	CT	CC	CC	--	--	CC	CC	CT
CC	CT	CT	CC	CC	CC	CT	CC	--	CC	CC	CC	CC
AA	AC	AC	AA	AA	AA	AC	AA	--	AA	AA	AA	AA
CC	CT	CT	CC	CC	CC	CT	CC	CC	CC	CC	CC	CC
AT	TT	TT	AA	AT	--	AT	AA	--	AA	AA	AA	AT
AG	AA	AA	GG	AG	AG	AG	GG	GG	GG	GG	GG	AG
CG	CG	CG	GG	CG	--	GG	GG	GG	GG	GG	GG	CG
TT	CT	CT	TT	TT	TT	CT	--	TT	TT	TT	TT	--
GG	AG	AG	GG	GG	GG	AG	GG	GG	--	AG	GG	GG
AA	AG	AG	AA	AA	AA	AG	AA	AA	AA	AG	AA	AA
AG	AG	AG	AA	AA	AG	AA	AA	--	AG	AA	AA	AG
AG	AG	AG	GG	GG	AG	GG	GG	GG	AG	GG	GG	AG
TT	TT	CT	TT	CT	CT	CT	CT	TT	CT	CT	TT	CT
GT	GT	TT	GG	GT	TT	GT	GT	--	TT	GT	GG	TT
GT	GT	GT	TT	TT	GT	TT	TT	TT	GT	TT	TT	GT
AG	AG	AG	GG	AG	AA	GG	AG	GG	AA	AG	AG	AA
CC	CC	CC	CC	CT	CT	CC	CT	CC	CT	CT	CC	CT
TT	TT	TT	TT	GT	GT	TT	GT	TT	GT	TT	TT	GT
GG	GG	GG	AG	GG	GG	--	AG	AG	--	AG	AG	GG
AG	AA	GG	AG	AG	AA	AG	AA	AA	GG	AG	AG	AG
--	CT	CC	CC	CC	CT	CT	CT	CC	--	CT	CT	CC
AA	AA	AT	AA	AA	AA	AT	AA	--	AA	AT	AT	AA
CG	CC	GG	CG	CC	CC	CG	CC	CC	CG	CC	CG	GG
AC	AA	AC	AC	AA	AC	AA	AA	AA	AC	AA	AA	AC
AG	AG	GG	AG	AA	AG	AG	AA	AG	AG	AA	AG	AG
AG	GG	AG	AG	AG	AG	GG	AG	GG	AG	AG	GG	GG
CT	CC	CT	CT	CT	CT	CC	CT	CC	CT	CT	CC	CC
AG	AA	AG	GG	GG	AG	AG	GG	AA	AG	GG	AG	AG
CT	CT	CT	CC	CC	CT	CC	CC	CT	CT	CC	CC	CC

GG	AG	GG	GG	GG	GG	AG	GG	AG	GG	GG	AG	AG
GG	AG	GG	AG	AA	GG	AA	AG	AA	--	GG	AG	AA
CT	TT	CT	CC	CT	CT	CT	CC	CT	CT	CT	TT	CT
AT	TT	AT	AA	TT	AT	AT	AA	AT	AT	AT	TT	TT
AC	CC	AC	AA	CC	AA	CC	AA	AC	AC	AC	CC	CC
CT	CC	CT	TT	CC	TT	CC	TT	CT	CT	CT	CC	CC
AG	AA	AG	GG	AA	--	AA	GG	AG	GG	AG	AA	AA
CT	TT	CT	CT	TT	CC	TT	CC	CT	CC	CT	TT	TT
CT	CT	CT	TT	CT	TT	CT	TT	TT	TT	CT	CT	CT
AG	GG	AG	AG	GG	AA	GG	AA	--	AA	AG	GG	GG
GG	AA	GG	AA	GG	AA	AA	AG	AG	AG	GG	AA	AG
TT	CC	TT	CC	TT	CC	CC	CT	CT	CT	TT	CC	CT
AG	GG	AG	GG	AG	GG	GG	AG	AG	AG	GG	GG	AG
TT	CC	CT	--	TT	CC	CC	CT	CT	CT	CT	CC	TT
CG	GG	GG	GG	CG	--	GG	GG	--	GG	CG	GG	CG
CT	CC	CT	CC	TT	CC	CC	TT	CT	CT	CT	CT	CT
CT	CC	CT	CC	CT	CC	CT	CT	CT	CT	CC	CT	CC
AG	GG	AG	AG	AA	AG	GG	AA	AG	AG	GG	AA	AG
--	CC	CT	CT	TT	CT	CC	TT	--	CT	CC	TT	CT
AG	AA	AG	AG	GG	AG	AA	GG	AG	AG	AA	GG	AG
CT	CC	CT	CT	TT	CT	CC	TT	CT	CT	CC	TT	CT
AG	AG	AG	AG	AG	AG	GG	AA	AA	AG	GG	AA	AG
AG	AG	AG	GG	GG	GG	GG	AG	AG	AG	GG	AG	GG
GT	GT	GT	GT	TT	GT	TT	GT	GG	GT	TT	GG	GT
CG	CG	CG	CC	CC	CC	CC	CC	CG	CG	CC	CG	CC
CG	CG	CG	CC	CG	CC	CG	CC	--	CG	CG	CC	CC
CT	CT	CT	CT	CC	TT	CC	TT	CT	CT	CC	TT	CT
CT	CT	CT	CT	TT	CC	TT	CC	CT	CT	TT	CC	CT
AT	AT	AT	AT	AT	TT	AT	TT	AT	AA	AT	TT	AT
AG	AG	AG	AG	AG	AA	AG	AA	AG	GG	AG	AA	AG
CT	CT	CT	CT	CT	CT	CT	TT	CT	--	CT	TT	CT
CT	CT	CT	TT	CT	TT	CT	CT	TT	TT	CT	CT	TT
AC	AC	AC	AC	AC	AC	AC	CC	AC	AA	AC	CC	AC
GT	TT	TT	GG	GT	GT	TT	TT	--	TT	GG	GT	TT
AG	AA	AA	GG	AG	AG	AA	AA	AG	AA	GG	AG	AA
--	TT	TT	CC	CT	CT	TT	TT	CT	TT	CC	CT	TT
AT	AA	AA	TT	AT	AT	AA	AA	--	--	TT	AT	AA
AC	CC	CC	AA	AC	AC	CC	CC	AC	CC	AA	AC	CC
CC	CG	CG	CG	CC	CC	CG	CG	CG	CG	CC	CC	CG
AA	AG	AG	AG	AA	AA	AG	AG	AG	AG	AA	AA	AG
CT	CC	CC	CT	CT	CT	CC	CC	CT	CC	TT	CT	CC
CT	CC	CC	CT	CT	CT	CC	CC	CT	CC	TT	CT	CC
AG	AA	AA	AG	AG	AG	AA	AA	AG	AA	GG	AG	AA
CT	CT	CT	CC	CT	CT	CT	CT	--	--	CC	CT	CT
AG	AA	AA	AG	AG	AG	AA	AA	AG	AA	GG	AG	AA
AG	GG	GG	AG	AG	AG	GG	GG	AG	--	AA	AG	GG
GT	GG	GG	GT	GT	GT	GG	GG	GT	GG	TT	GT	GG

CT	TT	TT	CT	CT	CT	CT	TT	CT	TT	CC	CT	TT
CG	GG	GG	CG	CG	CG	GG	GG	CG	GG	CC	CG	GG
GT	TT	TT	GT	GT	GT	TT	TT	GT	TT	GG	GT	TT
AG	AA	AA	AG	AG	AG	AA	AA	AG	AA	GG	GG	AA
CT	CC	CC	CT	CT	CT	CC	CC	--	CC	TT	TT	CC
GT	GT	GT	TT	GT	GT	GT	GT	TT	GT	TT	TT	GT
CG	GG	GG	CG	CG	--	GG	GG	--	GG	CC	CC	GG
AG	AA	AA	AG	AG	AG	AA	AA	AG	AA	GG	GG	AA
AG	AG	AG	GG	AG	AG	AG	AG	--	AG	GG	GG	AG
AC	AA	AA	AC	AA	AC	AA	AA	AC	AA	CC	CC	AA
CG	CG	CG	CC	CG	CG	CG	CG	--	CG	CC	CC	CG
AG	GG	GG	AG	GG	AG	GG	GG	AA	GG	AA	AA	GG
AC	AA	AA	AC	AA	AC	AA	AA	--	AA	CC	CC	AA
GT	TT	TT	GT	TT	GT	TT	TT	--	--	GG	GG	TT
AC	CC	CC	AC	CC	AC	CC	CC	AA	CC	AA	AA	CC
CT	CT	CT	CC	CT	CT	CT	CT	CC	CT	CC	CC	CT
GT	TT	TT	GT	TT	GT	TT	TT	GG	TT	GG	GG	TT
GG	AG	AG	AG	AG	GG	AG	AG	GG	AG	GG	GG	AG
CT	CC	CC	CT	CC	CT	CC	CC	--	--	TT	TT	CC
CT	CT	CT	CC	CT	CT	CT	CT	CC	CT	CC	CC	CT
AT	AA	AA	AT	AA	AT	AA	AA	TT	AA	TT	TT	AA
CG	CC	CC	CG	CC	CG	CC	CC	GG	CC	GG	GG	CC
GT	GG	GG	GT	GG	GT	GG	GG	TT	GT	TT	TT	GG
AG	AG	AG	AG	AG	GG	AG	AG	GG	GG	GG	GG	AG
TT	TT	TT	GT	TT	TT	TT	TT	GT	TT	GT	GT	TT
CC	CC	CT	CT	CT	CT	CC	CC	TT	CT	TT	TT	CC
CC	CC	AC	AC	AC	AC	CC	CC	AA	AC	AA	AA	CC
CC	CC	CT	CC	CC	CT	CC	CC	--	CT	CT	CT	CC
GG	GG	CG	CG	CG	CG	GG	GG	CC	CC	CG	CC	GG
CC	CT	CT	CT	CT	CT	CC	CT	CT	TT	CT	TT	CC
GT	TT	TT	GT	GT	TT	GT	TT	TT	TT	TT	TT	GT
CT	CT	CT	TT	TT	CT	CT	CT	CT	TT	CT	TT	CT
AT	TT	TT	AT	AT	TT	AT	TT	--	TT	TT	TT	AT
CC	AC	AC	CC	CC	AC	CC	AC	AC	AC	AC	AC	CC
TT	CT	CT	CT	TT	CT	TT	CT	CT	CC	CC	CT	CT
AA	AG	AG	AA	AA	AG	AA	AG	AG	AG	AG	AG	AA
GG	AG	AG	GG	GG	AG	GG	AG	AG	AG	AG	AG	GG
CG	CG	GG	GG	CG	GG	GG	GG	GG	--	CG	CG	CG
TT	TT	CT	CC	CT	CT	CT	CT	--	CC	CT	TT	CT
TT	TT	CT	CC	CT	CC	CT	CT	CC	CC	CT	TT	CT
TT	TT	CT	CC	CT	CC	CT	CT	CT	CC	CC	CT	CT
CC	CC	CT	TT	CT	TT	CT	CT	CT	--	TT	CT	CT
TT	TT	GT	GT	GT	GG	GT	GT	GT	GT	GG	GT	GT
CC	CC	CT	CT	CT	CT	CT	CT	CT	CT	TT	CT	CT
AA	AA	TT	AT	TT	AT	AT	AT	AT	AT	TT	AT	AT
AA	AA	GG	AG	GG	AG	AG	AG	AG	AG	GG	AG	AG
CC	AC	AA	AC	AA	AC	AC	AC	AC	AC	AA	AC	AC

AA	AG	GG	AG	GG	AG	AG	AG	--	AG	GG	AG	AG
AA	AG	GG	AG	GG	AG	AG	AG	--	AG	GG	AG	AG
TT	CT	CC	CT	CC	CT	CT	CT	CT	CT	CC	CT	CT
GG	AG	AA	AG	AA	AG	AG	AG	AG	AG	AA	AG	AG
--	GT	TT	GT	TT	GT	GT	GT	GT	GT	--	GT	GT
--	TT	CC	CT	CC	CT	CT	CT	CT	CT	--	CT	CT
GG	GG	AA	AG	AA	AG	AG	AG	AA	AG	AA	AG	AG
GG	GG	AA	AG	AA	AG	--	AG	AG	AG	AA	AG	AG
AA	AA	GG	AG	GG	AG	AG	AG	AG	AG	GG	AG	AG
GG	GG	CC	CG	CC	CG	CG	CG	CG	CG	CC	CG	CG
AA	AA	GG	AG	GG	AG	AG	AG	AG	AG	GG	AG	AG
AA	AA	CC	AC	CC	AC	AC	AC	AC	AC	CC	AC	AC
GG	GG	AA	AG	AA	AG	AG	AG	AG	AG	AA	AG	AG
GG	GG	AA	AG	AA	AG	AG	GG	AG	AG	AA	AG	AG
AA	AA	TT	AT	TT	AT	AT	AA	AT	AT	TT	AT	AT
TT	TT	CC	CT	CC	CT	CT	TT	CT	CT	CC	CT	CT
TT	TT	CC	CT	CC	CT	CT	TT	CT	CT	CC	CT	CT
CC	CC	GG	CG	GG	CG	CG	CC	CG	CG	GG	CG	CG
CC	CC	TT	CT	TT	CT	CT	CC	CT	CT	TT	CT	CT
TT	TT	CC	CT	CC	CT	TT	TT	CT	CT	CC	CT	CT
CC	CC	TT	CT	TT	CT	CC	CC	CT	CT	TT	CT	CT
AA	AA	AT	AT	TT	AT	AA	AA	AT	AT	TT	AT	AT
TT	TT	AT	--	AA	AT	TT	TT	--	AT	AA	AT	AA
CC	CC	CT	CT	TT	CT	CC	CC	CT	CT	TT	CT	TT
TT	TT	CT	CT	CC	CT	TT	TT	CT	CT	CC	CT	CC
TT	TT	AT	AT	AA	AT	TT	TT	AT	AT	AA	AT	AA
AA	AA	AG	AG	GG	AG	AA	AA	AG	AG	GG	AG	GG
--	TT	GT	GT	GG	GT	TT	TT	GT	GT	GG	GT	GG
CC	CC	CT	CT	TT	CT	CC	CC	CT	CT	TT	CT	TT
GG	GG	CG	CG	CC	CG	GG	GG	CG	CG	CC	CG	CC
AA	AA	AG	AG	GG	AG	AA	AA	AG	AG	GG	AG	GG
CC	CC	CT	CT	TT	--	CC	CC	CT	CT	TT	CT	TT
CC	CC	CT	CT	TT	TT	CC	CC	CT	CT	TT	CT	TT
AA	AA	AT	AT	TT	TT	AA	AA	AT	AT	TT	AT	TT
GG	GG	AG	AG	AA	AA	GG	GG	AG	AG	AA	AG	AA
GG	GG	AG	AG	AA	AA	GG	GG	AG	AG	AA	AG	AG
GG	GG	GT	GT	TT	TT	GG	GT	GT	GT	TT	GT	GT
GG	GG	AG	AG	AA	AA	GG	AG	AA	AG	AG	AG	AG
AG	AG	AG	AG	GG	GG	AG	GG	--	AG	GG	GG	GG
TT	TT	CT	TT	CT	TT	TT	TT	TT	CT	TT	TT	TT
--	AG	AA	AG	AA	AG	AG	AG	--	AA	GG	GG	AG
GG	AG	AG	AG	AG	AG	AG	AG	--	AG	GG	GG	AG
GG	AG	AA	AG	AA	AG	AG	AG	GG	AA	GG	AG	AG
CC	CC	GG	CG	GG	CG	CG	CC	CC	GG	CC	CG	CC
TT	TT	CC	TT	CC	TT	CT	TT	CT	CC	TT	CT	TT
AC	AC	CC	AC	CC	AC	AC	AC	--	CC	AC	AC	AC
AG	AG	AA	AG	AA	AG	AA	AG	--	AA	AG	AA	AG

AC	AC	CC	AC	CC	AC	AC	AC	--	CC	AC	AC	AC
CC	CC	AC	CC	AC	CC	AC	CC	AC	AC	CC	AC	CC
CT	CT	TT	CT	TT	CT	CT	CT	CT	TT	CT	CT	CT
GG	GG	AA	AG	AA	GG	AG	GG	AG	AA	GG	AG	GG
AG	AA	GG	AG	AG	AA	AG	AA	AG	GG	AA	AG	AA
CT	CC	TT	CT	CT	CC	CT	CC	--	TT	CC	CT	CC
CT	TT	TT	TT	TT	TT	CT	CT	CT	CT	--	CT	TT
--	GG	CG	CG	CG	GG	CG	CC	--	CC	CG	CG	GG
AA	GG	AG	AG	AG	GG	--	AA	AG	AA	AA	AG	GG
TT	CT	CT	CT	CT	--	CT	TT	CT	TT	TT	CC	CC
AT	AA	AA	AA	AA	AA	AT	AT	AT	AT	AT	AA	AA
CC	AC	AC	AC	AC	AA	AC	CC	AC	AC	CC	AA	AC
GG	GG	GG	--	GG	AG	AG	GG	--	AG	GG	AG	AG
CC	CC	CC	CC	CT	CT	CT	CC	CT	CT	CC	CT	CT
CC	CC	CC	CC	CT	CT	CT	CC	CT	CT	CC	CT	CT
GG	GG	GG	GG	GT	GT	GT	GG	GT	GT	GG	GT	GT
CT	TT	TT	CT	CT	TT	CC	CT	TT	CT	CT	CT	TT
AG	GG	AG	AG	AG	GG	AG	AG	--	GG	AG	GG	GG
AG	GG	GG	GG	AG	AG	AA	AG	GG	AG	AG	AG	GG
CG	CG	GG	CG	CG	GG	GG	CG	--	GG	CG	GG	CG
AA	GG	AG	GG	AG	AG	AA	AG	GG	AG	AG	AG	GG
GG	AG	AG	AA	GG	AG	GG	AG	AG	AG	AG	AG	AA
GT	GT	GT	TT	GT	GT	GT	TT	GT	GT	TT	GT	TT
GG	AG	AG	AG	AG	AG	GG	AG	AG	AG	AG	AG	AA
--	AG	AG	AG	AG	AG	GG	AG	--	AG	AG	AG	AA
GT	GT	GT	GT	GT	GT	GT	GG	GT	GT	GG	GT	GG
TT	CT	CT	CT	CT	CT	TT	CT	--	CT	CT	CT	CC
CT	CT	CT	CT	CT	TT	CC	CT	CT	CT	CT	TT	CT
CT	CC	CC	CC	CC	CC	CT	CT	CC	CC	CT	CC	CC
AG	AG	AG	AG	AG	AA	AG	AA	AG	AG	AG	AA	AG
CT	CT	CT	CT	CT	CC	CT	CC	CT	CT	CT	CC	CT
AG	AG	AG	AG	AG	AA	AG	AA	AG	AG	AG	AA	GG
--	CT	CT	CT	CT	CC	CT	CT	--	CT	TT	CT	TT
CG	CG	CG	CG	CG	GG	CG	CG	GG	CG	CC	CG	CC
CT	CT	CT	TT	CT	CC	CT	CT	CT	CT	TT	CT	TT
CC	AC	CC	AA	AA	AA	AC	AC	--	AC	AC	CC	AC
AA	AG	AA	GG	GG	GG	AG	AG	AA	AG	AG	AA	AG
CC	TT	CC	TT	TT	CT	CT	CT	CC	CT	CT	CC	CT
GG	AA	GG	AA	AA	AG	AG	AG	GG	GG	AG	GG	AG
GG	AA	GG	AA	AA	AG	AA	AA	--	GG	AG	GG	GG
CT	TT	CT	TT	TT	TT	TT	TT	CT	--	CT	CT	CT
AG	GG	AA	AG	GG	AG	GG	GG	--	AG	AG	AA	AA
--	AA	--	AG	AA	AG	AA	AA	--	AG	AA	AG	AG
TT	TT	GT	GT	TT	GT	TT	TT	GT	TT	TT	GT	GT
TT	TT	GT	GT	TT	GT	TT	TT	--	GT	TT	GT	GT
AC	AA	CC	AC	AA	AC	AA	AA	CC	AC	AC	CC	CC
AG	AA	GG	AG	AA	AG	AA	AA	--	AG	AG	AG	GG

CG	CC	GG	CG	CC	CG	CC	CC	--	CG	CG	--	GG
CG	CC	GG	CG	CG	CG	CC	CG	GG	CG	CG	CG	GG
CT	TT	CT	CT	CT	TT	TT	CT	CC	CT	CC	CT	CC
CT	CC	CT	CT	CT	CC	CC	CT	TT	CT	TT	CT	TT
AT	AA	AT	AT	AT	AA	--	AT	TT	AT	TT	AT	TT
AG	AG	GG	GG	GG	AG	GG	GG	GG	AG	GG	AG	GG
CT	CC	CT	CT	CT	CC	CT	CT	TT	CT	TT	CC	TT
CT	TT	TT	CT	CT	TT	CT	CT	CC	CT	--	TT	CC
CT	CC	CC	CT	CT	CC	CT	CT	TT	CT	TT	CC	TT
GG	GG	GG	AA	AG	GG	AG	AG	AA	AG	AA	GG	AA
CC	CC	CC	CG	CC	CC	CC	CC	--	CG	CC	CC	CG
GG	GG	GG	CG	GG	GG	CG	CG	CG	GG	CG	CG	CG
GG	GG	GG	GT	GG	GG	GG	GT	GG	GT	GG	GG	GG
CC	CC	CG	CG	CG	CG	CC	CG	CC	CG	CG	CG	CC
AG	AG	AA	AG	AA	AA	AG	AG	AG	AG	AG	AA	AA
AA	AA	AC	AC	AC	AC	AA	AC	AA	AC	AC	AC	AC
AA	AA	AT	AT	AT	AT	AA	AT	AA	AT	AT	AT	AA
AG	AG	AA	AG	AA	AA	AG	AG	AG	AG	AA	AA	AG
AT	AT	TT	AT	TT	TT	AT	AT	AT	TT	TT	TT	AA
AG	AG	AA	AG	AA	AA	AG	AG	AG	AA	AA	AG	GG
CC	AC	AC	CC	AC	CC	AC	CC	CC	AC	AC	CC	CC
CC	CT	TT	CT	TT	CT	TT	CT	CT	TT	CT	CT	CC
TT	GT	GG	TT	GG	GT	GT	GG	GT	GG	GT	GT	TT
TT	GG	GG	TT	GG	GT	GT	GG	GT	GG	GT	GT	TT
CC	TT	TT	CC	TT	CT	CT	TT	CT	TT	CT	CT	CC
CC	GG	GG	CC	GG	CG	CG	GG	CG	GG	CG	CG	CC
GG	AA	AA	GG	--	AG	AG	AA	AG	AA	AG	AG	GG
CC	TT	TT	CC	TT	CT	--	TT	CT	TT	CT	CT	CC
--	GG	GG	TT	GG	GT	GT	GG	GT	GG	GT	GT	TT
TT	CC	CC	TT	CC	CT	CT	CC	CT	CC	CT	CT	TT
GG	CC	CC	GG	CC	CG	CG	--	CG	CC	CG	CG	GG
--	AA	AA	GG	AA	AG	AG	AA	AG	AA	AG	AG	GG
AA	CC	AC	AA	CC	AC	AC	CC	AC	--	AC	AC	AA
TT	CC	CT	TT	CC	CT	CT	CC	CT	CC	CT	CT	TT
GG	AG	AG	GG	AG	AG	AG	AG	GG	AG	GG	AG	GG
AA	CC	AC	AA	CC	AC	AC	CC	--	CC	AC	AC	AA
CC	TT	CT	CC	TT	CT	CT	TT	CT	TT	CT	CT	CC
GG	AA	AG	GG	AA	AG	AG	AA	AG	AA	AG	AG	GG
GG	CC	CG	GG	CC	CG	CG	CC	CG	CC	CG	CG	GG
GG	TT	GT	GG	TT	GT	GT	TT	GT	TT	GT	GT	GG
CC	TT	CT	CC	TT	CT	CT	TT	CT	TT	CT	CT	CC
TT	CT	CT	TT	CT	CT	CT	CT	TT	CT	TT	CT	TT
AG	GG	AG	AG	GG	AG	AG	GG	GG	GG	GG	AG	AG
CC	GG	CG	CC	CG	CG	CG	CG	CG	GG	CG	CG	CC
AA	AT	AA	AA	AA	AA	AA	AT	AT	AT	AT	AA	AA
AA	AG	AG	AA	AG	AG	AG	AA	AA	AG	AA	AG	AA
CC	AC	AC	CC	AC	AC	AC	CC	CC	AC	CC	AC	CC

TT	CT	CT	TT	CT	CT	CT	TT	TT	CT	TT	CT	TT
TT	TT	CT	TT	TT	TT	TT	CT	CT	CT	CT	TT	TT
GG	GT	AT	GG	GT	GT	GT	AG	AG	AT	AG	GT	GG
AA	AT	AT	AA	AT	AT	AT	AA	AT	AT	AA	AT	AA
GG	AG	AA	GG	AG	AA	AG	AG	AG	AA	AG	AG	GG
CC	AC	AA	CC	AC	AA	AC	AC	AC	AA	AC	AA	CC
AA	AG	AG	AA	AG	AG	AG	AA	AA	AG	AA	AG	AA
GG	AG	AA	GG	AG	AA	AG	AG	AG	AA	AG	AA	GG
AA	AG	GG	AA	AG	GG	AG	AG	AG	GG	AG	GG	AA
GG	AG	AA	GG	AG	AA	AG	AG	AG	AA	AG	AA	GG
GG	GT	TT	GG	GT	TT	GT	GT	GT	TT	GT	TT	GG
GG	GG	AG	GG	GG	AG	GG	AG	AG	AG	AG	GG	GG
--	CT	TT	CC	CT	CT	CT	CT	--	TT	CT	CT	CT
CG	CG	GG	CC	CG	CG	CG	CG	CG	GG	CG	CG	CG
TT	CT	CT	TT	CT	CT	CT	TT	TT	CT	TT	CT	TT
CT	TT	CT	TT	TT	TT	TT	TT	TT	CT	CT	TT	CT
CT	CC	CT	CC	CC	CT	CT	CT	CT	CT	CT	CC	CT
GT	GG	GG	GG	GT	GG	GG	GG	GG	GG	GG	GG	GT
AA	GG	AG	GG	AG	AG	AG	AG	AG	AG	AG	GG	AA
GG	AA	AG	AA	AG	AG	AG	AG	--	AG	AG	AA	AG
AG	AA	AG	AA	AA	AG	AG	AG	--	--	--	AA	AG
TT	GG	GT	GG	GT	GT	GT	GT	GT	GT	GT	GT	TT
AG	AA	AA	AA	AA	AG	AG	AG	--	AG	AG	AA	AG
CT	CC	CC	CC	CC	CT	CT	CT	CC	--	CT	CC	CC
AA	AG	AG	AG	AG	AA	AA	AA	AG	AA	AA	AG	AG
AA	GG	GG	GG	GG	AA	AA	AG	AG	AA	AG	AG	AG
AA	GG	GG	GG	GG	AA	AA	AG	AG	AA	AG	AG	AG
CC	GG	GG	CG	GG	CG	CC	CG	CG	CC	CG	CG	CG
CC	GG	CG	CG	GG	CG	CC	CG	--	CC	CG	CG	GG
AA	GG	AG	AG	GG	AG	AA	AG	AG	AA	AG	AG	GG
AG	GG	AG	AG	GG	AG	AG	GG	AG	AG	AG	AG	GG
CC	TT	CT	CT	TT	CT	CC	CT	CT	CC	CT	CT	TT
AC	CC	AC	AC	CC	AC	AC	CC	AC	AC	AC	AC	AC
CG	CC	CG	CG	CC	CG	CG	CC	CG	CG	CG	CG	CG
AC	CC	AC	AC	CC	CC	AC	AC	AC	AC	CC	AC	AC
CC	AC	AC	AC	AA	AA	AC	CC	AC	CC	AA	AC	AC
AG	AA	AA	AA	AA	AA	AG	AG	AA	AG	AA	AA	AA
GG	AG	AG	AG	AA	AA	AG	GG	AA	GG	AA	AG	AG
AG	AG	GG	GG	GG	GG	GG	AG	GG	AG	GG	GG	GG
TT	CT	CT	CT	CT	CC	CT	TT	CC	TT	CC	CT	CT
TT	CT	CT	CT	CT	CC	CT	CT	CC	TT	CC	CT	CT
AG	AA	AG	AG	AG	AA	AG	AG	AA	AG	AA	AG	AG
CC	CT	CT	CT	CT	CC	CT	--	CC	CT	CC	CT	CT
--	AG	AG	AA	AA	AG	AG	GG	GG	AG	AG	AA	AG
CC	AC	AC	AC	AC	AC	AC	CC	CC	AC	AC	AC	AC
CT	CC	CC	CT	CT	CC	CC	CC	CC	CC	CC	CT	CC
AG	AG	AG	GG	GG	AG	AG	AG	AA	AG	AG	GG	AG

AG	AA	AA	AG	AG	AA	AA	AA	AA	AA	AA	AG	AA
AG	AG	AG	GG	AG	AA	AG	AG	AA	AG	AG	GG	AG
AC	AC	AC	AA	AC	CC	AC	AC	CC	AC	AC	AA	AC
AG	AG	AG	AA	AG	GG	AG	AG	AG	AG	AG	AA	AG
GT	GT	GT	GG	GT	TT	GT	GT	GT	GT	GT	GG	GG
AA	AG	AG	AG	AA	AA	AG	AG	AG	AG	AG	AG	AG
CT	CC	CT	CC	CT	CT	CC	TT	CT	CT	TT	CC	CC
CC	CT	CT	CT	CC	CC	CT	CC	CT	CT	CC	CT	CT
AG	AA	AG	AA	AG	AG	AA	GG	--	AA	GG	AA	AA
CG	CG	GG	CG	CG	CG	CG	GG	GG	CG	GG	CG	CG
CT	CT	TT	CT	CT	CT	CT	TT	--	CT	TT	CT	CT
AG	AG	AG	AG	AG	AG	GG	AA	AG	GG	AA	GG	AG
AG	AG	AG	AG	AG	AG	GG	AA	AG	GG	AA	GG	AG
CG	CG	CG	CG	CG	CG	GG	CC	--	GG	CC	GG	CG
AG	AG	AG	AA	GG	AG	GG	AA	GG	GG	AA	GG	AG
AG	AA	AA	AA	AG	AA	GG	AA	AG	AG	--	AG	AA
CT	CC	CC	CC	TT	CT	TT	CC	CT	TT	CT	CT	CC
AG	AA	AA	AA	GG	AG	--	AA	AG	GG	AG	AG	AA
CG	GG	GG	GG	CC	CG	CC	GG	CG	CC	CG	CG	GG
AC	AA	AA	AA	CC	AC	CC	AA	AC	CC	AC	AC	AA
AG	GG	GG	GG	AA	AG	AA	GG	AG	AA	AG	AG	GG
GT	TT	TT	TT	GG	GT	GG	TT	GT	GG	GT	GT	TT
AG	AA	--	AA	GG	AG	GG	AA	AG	GG	AG	AG	AA
AG	AA	AA	AA	GG	AG	GG	AA	AG	GG	AG	AG	AA
AG	GG	GG	GG	AA	AG	AA	GG	--	AA	AG	AG	GG
AC	CC	CC	CC	AA	AC	AA	CC	AC	AA	AC	AC	CC
AG	GG	GG	GG	AA	AG	AA	GG	AG	AA	AG	AG	GG
AG	GG	GG	GG	AA	AG	AA	GG	AG	AA	AG	AG	GG
CT	CC	CC	CC	TT	CT	TT	CC	CT	TT	CT	CT	CC
CG	CC	CC	CC	GG	CG	GG	CC	CG	GG	CG	CG	CC
AC	CC	CC	CC	AA	--	AA	CC	--	AA	AC	AC	CC
CT	CC	CC	CC	TT	CT	TT	CC	TT	TT	CT	CT	CC
GT	GG	GG	GG	TT	GT	TT	GG	TT	TT	GT	GT	GG
CT	TT	TT	TT	CC	CT	CC	TT	CC	CC	CT	CT	TT
CG	GG	GG	GG	CC	CG	CC	GG	--	CC	CG	CG	GG
AT	AA	AA	AA	TT	AT	TT	AA	TT	TT	AT	AT	AA
CT	TT	TT	TT	CC	CT	CC	TT	CC	CC	CT	CT	TT
AG	AA	AA	AA	GG	AG	GG	AA	--	GG	AG	AG	AA
AG	GG	GG	GG	AA	AG	AA	GG	AA	AA	AG	AG	GG
AT	TT	TT	TT	AA	AT	AA	TT	AA	AA	AT	AT	TT
AG	AA	AA	AA	GG	AG	GG	AA	GG	GG	AG	AG	AA
AG	GG	GG	GG	AA	AG	AA	--	--	AA	AG	AG	GG
CG	GG	GG	GG	CC	CG	--	GG	--	CC	CG	CG	GG
AG	GG	GG	GG	AA	AG	AA	GG	AA	AA	AG	AG	GG
CG	CC	CC	--	GG	CG	GG	CC	--	GG	CG	CG	CC
AG	AA	AA	--	GG	AG	GG	AA	--	GG	AG	AG	AG
AC	CC	CC	CC	AA	AC	AA	CC	--	AA	AC	AC	AC

AG	AA	AA	AA	GG	AG	GG	AA	GG	GG	AG	AG	AG
CG	GG	GG	GG	CC	CG	CC	GG	CC	CC	CG	CG	CG
CT	TT	TT	TT	CC	CT	CC	TT	CC	CC	CT	CT	CT
AT	AA	AA	AA	TT	AT	TT	AA	--	TT	AT	AT	AT
CT	TT	TT	--	CC	CT	CC	TT	--	--	CT	CT	CT
AT	TT	TT	TT	AA	AT	AA	TT	AA	AA	AT	AT	AT
AT	AA	AA	AA	TT	AT	TT	AA	TT	TT	AT	AT	AT
CG	CC	CC	CC	GG	CG	GG	CC	GG	GG	CG	CG	CG
CT	TT	TT	TT	CC	CT	CC	TT	CC	CC	CT	CT	CT
AG	GG	GG	GG	AA	AG	AA	GG	AA	AA	AG	AG	AG
AC	CC	CC	CC	AA	AC	AA	CC	AA	AA	AC	AC	AC
GT	GG	GG	GG	TT	GT	TT	GG	TT	TT	GT	GT	GT
CT	TT	TT	TT	CC	CT	CC	TT	CC	CC	CT	CT	CT
CG	CC	CC	CC	GG	CG	GG	CC	GG	GG	CG	CG	CG
GT	GG	GG	GG	TT	GT	TT	GG	--	TT	GT	GT	GT
CT	CC	CC	CC	TT	CT	TT	CC	TT	TT	CT	CT	CT
--	GG	GG	GG	AA	AG	AA	GG	AA	AA	AG	AG	AG
AC	CC	CC	CC	AA	AC	AA	CC	AA	AA	AC	AC	AC
CT	TT	TT	TT	CC	CT	CC	TT	--	CC	CT	CT	CT
AC	AC	CC	CC	AC	AC	AA	CC	AC	AA	AC	AC	AC
AA	AG	GG	GG	AG	AG	AA	GG	AG	AA	AG	AG	AG
GG	GT	TT	TT	GT	GT	GG	TT	GT	GG	GT	GT	GT
GG	GT	TT	TT	GT	GT	GG	TT	GT	GG	GT	GT	GT
GG	AG	AA	AA	AG	AG	GG	AA	AG	GG	AG	AG	AG
CC	CT	TT	TT	CT	CT	CC	TT	CT	CC	CT	CT	CT
CC	CT	TT	TT	CT	CT	CC	TT	CT	CC	CT	CT	CT
AA	AG	GG	GG	AG	AG	AA	GG	AG	AA	AG	AG	AG
CC	CT	TT	TT	CT	CT	CC	TT	CT	CC	CT	CT	CT
TT	CT	CC	CC	CT	CT	TT	CC	CT	TT	CT	CT	CT
GG	AG	AA	AA	AG	AG	GG	AA	AG	GG	AG	AG	AG
CC	CT	TT	TT	CT	CT	CC	TT	CT	CC	CT	CT	CT
TT	TT	CT	CT	TT	TT	TT	CT	TT	TT	TT	CT	TT
CC	CC	TT	TT	TT	CT	CC	TT	CT	--	CT	CT	CC
AA	AA	GG	GG	GG	AG	AA	GG	AG	AA	AG	AG	AA
AA	AA	CC	CC	CC	AC	AA	CC	AC	AA	AC	AC	AA
CG	CG	CC	CC	CC	CC	CG	CC	CC	CG	CC	CG	CG
GG	GG	TT	TT	TT	GT	GG	GT	GT	GG	GT	GT	GG
GG	GG	AA	AA	AA	GG	GG	AG	AA	GG	AG	AG	GG
TT	TT	CC	CC	CT	TT	TT	CT	CC	TT	CT	CT	TT
AG	AG	GG	GG	GG	AG	AG	AG	GG	AG	GG	AG	AG
GG	GG	AA	AG	AG	GG	GG	GG	--	GG	AG	AG	GG
GG	GG	CC	CG	CG	GG	CG	GG	--	--	CG	CG	GG
CT	CT	TT	CT	TT	CT	CT	CT	TT	CT	TT	CT	CT
TT	TT	GG	TT	GT	TT	GG	TT	GG	GT	GT	GT	TT
AG	AG	AG	AG	AG	AG	GG	AG	AG	GG	AG	GG	AG
AG	GG	AG	AG	AG	GG	GG	GG	GG	GG	AG	AG	AG
CC	AC	CC	AC	CC	AC	AA	AC	AC	AA	CC	AC	CC

AT	AT	AA	AT	AA	AT	TT	AT	AT	TT	AA	AT	AA
AT	AT	AA	AT	AA	AA	TT	AT	AT	TT	AA	AT	AA
AT	TT	TT	AT	TT	TT	AA	TT	AT	AA	TT	AT	TT
CT	CC	CC	CT	CC	CC	TT	CT	CT	--	CC	CT	CC
AG	AA	AA	AG	AA	AA	AG	AG	AG	GG	AA	AG	AA
AC	CC	CC	AC	AC	CC	AC	AC	AC	AA	CC	AC	CC
CT	CC	CC	CT	CT	CC	CC	CT	CT	TT	CC	CT	CC
GG	AG	AA	AG	AG	AA	AA	GG	AG	AG	AG	AG	AA
GG	GT	TT	GT	GT	GT	TT	GG	GT	GT	GT	GT	TT
GG	AG	AA	AG	AG	AG	AA	GG	AG	AA	AG	AG	AG
CC	CG	CG	CG	CG	CC	CG	CC	CG	CG	CG	CC	CG
GG	AG	AG	AG	AG	GG	AG	GG	AG	AG	AG	GG	AG
CC	CG	CG	GG	GG	CC	GG	CC	GG	GG	GG	CG	CG
AA	AG	AG	GG	GG	AA	GG	AA	GG	GG	GG	AG	AG
GG	AG	AG	AA	AA	GG	AA	GG	--	AA	AA	AG	AA
AA	AG	AG	GG	GG	AA	GG	AA	GG	GG	GG	AG	GG
GG	GT	GT	GT	GT	GG	GT	GG	GT	GT	GT	GG	GT
GG	AG	AG	AA	AA	GG	AA	GG	AA	AG	AA	AG	AA
TT	GT	GT	GT	GT	TT	GT	TT	GT	GT	GT	TT	TT
CG	CG	CG	CC	CC	GG	CC	GG	CC	CG	CC	CG	CG
CT	TT	CT	TT	TT	TT	CT	TT	CT	--	CT	TT	TT
CG	GG	CC	GG	GG	GG	CC	CG	CC	CG	CG	--	CG
--	CC	CG	CC	CC	CC	CG	CG	--	--	CC	CG	CG
AC	AA	AC	AA	AA	AA	AC	AA	AC	AA	AC	AA	AA
CT	CC	CT	CC	CC	CC	CT	CC	CT	CC	CT	CC	CC
AG	AA	AG	AA	AA	AA	AG	AA	AG	AA	AG	AA	AA
CT	CC	CT	CC	CC	CC	CT	CC	--	CC	CT	CC	CC
CG	CG	GG	CG	GG	GG	GG	CG	CG	GG	GG	GG	CG
CC	CC	CT	CC	CC	CC	CT	CC	CT	CC	CT	CC	CC
AG	AG	AG	AG	AG	AA	AG	AG	AA	AA	AG	AA	AG
GG	GG	GT	GT	GG	GG	GT	GG	GG	GG	GT	GG	GG
GT	GT	GT	GG	GT	TT	GT	GT	TT	TT	GT	TT	GT
GG	GG	CG	CG	GG	GG	CG	GG	GG	GG	CG	GG	GG
CG	CG	CG	CC	CG	GG	CG	CG	GG	GG	CG	GG	CG
GG	GG	AG	AG	AG	GG	AG	GG	GG	GG	AG	GG	GG
CT	CT	CT	CC	CC	TT	CT	CT	--	TT	CT	TT	CT
GT	GT	GT	TT	TT	GG	GT	GT	--	GG	GT	GG	GT
GG	GG	AG	AG	AG	GG	AG	GG	GG	GG	AG	GG	GG
--	AG	AG	GG	GG	AA	AG	AG	--	--	--	AA	AG
CC	CT	CT	CC	CC	TT	CT	CT	--	--	CT	TT	CT
AT	TT	AT	AT	AT	TT	AT	TT	--	TT	AT	TT	TT
GT	TT	GT	GT	GT	TT	GT	TT	TT	TT	GT	TT	TT
TT	CT	CT	TT	TT	CC	CT	CT	CC	CC	CT	CC	CT
AG	AA	AG	AG	AG	AA	AG	AA	AA	AA	AG	AA	AA
AA	AT	AT	AA	AA	TT	AT	AT	--	TT	AT	TT	AT
AG	AA	AG	AG	AG	AA	AG	AA	AA	AA	AG	AA	AA
CT	TT	CT	CT	CT	TT	CT	TT	TT	TT	CT	TT	TT

AT	TT	AT	AT	AT	TT	AT	TT	TT	TT	AT	TT	TT
TT	GT	GT	TT	TT	GT	GT	GT	--	--	GT	GG	GT
GG	AG	AG	GG	GG	AG	AA	AG	AA	AA	AG	AA	AG
TT	CT	CT	TT	TT	CT	CC	CT	CC	CC	CT	CC	CT
TT	CT	CT	TT	TT	CT	CC	CT	CT	CC	CT	CC	CT
TT	CT	CT	TT	TT	CT	CT	CT	CT	CC	CT	CT	CT
TT	GT	GT	TT	TT	TT	GT	TT	GT	GG	GT	GT	GT
GG	CG	CC	GG	CG	GG	CG	GG	CG	CC	CG	CG	CG
AA	AC	CC	AA	AC	AA	AC	AA	AC	CC	AC	AC	AC
GG	AG	AA	GG	AG	GG	AG	AG	AG	AA	AG	AG	AG
AA	AG	GG	AG	AG	AA	AG	AG	AG	GG	AA	AG	AA
CT	CT	TT	TT	CT	CT	TT	CC	TT	TT	CT	TT	TT
AC	AC	AA	AA	AC	AC	AA	CC	--	AA	AC	AA	AA
GT	GT	TT	TT	TT	GT	TT	GT	--	--	TT	TT	TT
GG	GG	GG	GG	CG	GG	GG	CG	GG	GG	CG	GG	GG
AG	AG	GG	AG	AG	AG	GG	AA	GG	GG	AG	GG	GG
TT	TT	TT	TT	TT	CT	TT	CT	TT	CT	CT	TT	TT
GT	GT	GT	GT	TT	GT	TT	GT	--	--	TT	TT	TT
CT	CC	CC	CC	CC	CT	CC	CT	CC	CT	CT	CC	CC
AA	AC	AC	AC	AA	AC	AC	AC	AA	AA	AA	AA	AA
AG	AG	AG	AG	AA	GG	AG	GG	AA	AG	AG	AA	AG
AA	AG	AG	AG	AA	AA	AG	AG	AA	AA	AA	AG	AG
GT	TT	GT	GT	TT	GT	GG	GG	--	GT	GT	GT	GT
CT	CC	CT	CT	CC	CT	TT	CT	--	CT	CT	CT	CT
AC	AA	AC	AC	AA	AC	CC	AC	AC	AC	AC	CC	CC
AA	TT	AT	AT	TT	AT	AA	AT	AT	AT	--	AA	AA
GG	GT	GG	GG	GT	GT	GG	GT	--	GT	GT	GG	GT
AA	AG	AG	AG	AG	--	AA	AA	--	--	AA	AA	AA
GG	CC	CG	CG	CC	CG	--	CG	CG	CG	CG	GG	CG
GG	AA	AG	AG	AA	AG	GG	AG	--	AG	AG	GG	AG
CC	CC	CG	CC	CG	CG	CC	CG	CG	GG	GG	CG	CG
AA	AA	AG	AA	AG	AG	AA	AG	AG	GG	GG	AG	AG
AA	AA	AA	AA	AG	AA	AA	AA	AG	AG	AG	AG	AG
AA	AA	AT	AA	AT	AT	AA	AT	AA	AT	AT	AT	AT
AA	AA	AA	AA	AG	AA	AA	AA	AG	AG	AG	AG	AG
AG	AG	AG	AG	AA	AG	AG	AG	AA	AA	AA	AA	AA
AC	AA	AC	AA	CC	AC	AC	AC	AC	AC	CC	CC	CC
AC	CC	AC	CC	AC	AC	AC	AC	--	CC	AC	AC	AC
AC	CC	AC	CC	AA	AC	AC	AC	AC	AC	AC	AA	AA
GT	GG	GT	GG	--	GT	GT	GT	--	GT	GG	TT	TT
AT	AA	AT	AA	TT	AT	AT	AT	AT	AT	AA	TT	TT
AG	AA	AG	AA	AG	AG	AG	AG	AA	AA	AA	AG	AG
AC	CC	AC	CC	AC	--	AC	AC	CC	CC	CC	AC	AC
AT	AA	AT	AA	TT	AT	AT	AT	AT	AT	AA	TT	TT
CT	CT	CT	CT	TT	CT	CT	CT	CT	CT	CT	TT	TT
AG	AG	AG	AG	AA	AG	AG	AG	AG	AG	AG	AA	AA
CT	CT	CT	CT	TT	CT	CC	CT	CT	CT	CT	TT	TT

CT	CT	CT	CT	CC	CT	TT	CT	CT	CT	CT	CC	CC
AG	AG	AG	AG	AA	AG	GG	AG	AG	AG	AG	AA	AA
CT	CT	CT	CT	TT	CT	CC	CT	CT	CT	CT	TT	TT
--	CT	CT	CT	CC	CT	TT	CT	--	CT	CT	CC	CC
CT	CT	CT	CT	TT	CT	CC	CT	CT	CT	CT	TT	TT
AG	AG	AG	AG	AA	AG	GG	AG	--	AG	AG	AA	AA
CT	CT	CT	CT	CC	CT	TT	CT	--	CT	CT	CC	CC
AG	AG	AG	AG	AA	--	GG	AG	AG	AG	AG	AA	AA
CG	CG	CG	CG	GG	CG	CC	CG	CG	CG	CG	GG	GG
AC	AC	AC	AC	CC	AC	AA	AC	--	AC	AC	CC	CC
CT	TT	CT	CT	TT	CT	CC	CT	CT	CT	CT	TT	TT
CT	TT	CT	CT	TT	CT	CC	CT	CT	CT	CT	TT	TT
TT	AA	AT	AT	AA	AT	TT	AT	--	AT	AT	AA	AA
CC	TT	CT	CT	TT	CC	CC	CT	CT	CT	CT	TT	TT
AA	CC	AC	AC	CC	AC	AA	AC	AC	AC	AC	CC	CC
TT	GG	GT	GT	GG	GT	TT	GT	GT	GT	GT	GG	GT
TT	CC	CT	CT	CC	CT	TT	CT	--	CT	CT	CC	CT
GG	CC	CG	CG	CC	CG	GG	CG	CG	CG	CG	CC	CG
GG	CC	CG	CG	CC	CG	GG	CG	CG	CG	GG	CC	CG
CC	AA	AC	AC	AA	AC	CC	AC	AC	AC	AC	AA	AC
GG	CC	CG	CG	CC	CG	GG	CG	CG	CG	CG	CC	CG
TT	--	AA	TT	AT	AT	AA	AA	AA	AA	AA	TT	TT
GG	AG	AA	GG	AG	AG	AA	AA	AG	--	AA	GG	GG
GG	AG	AA	GG	AG	--	AG	--	--	AA	AA	GG	GG
--	AG	GG	AA	AG	AG	AG	AG	AG	AG	GG	AA	AA
AG	AG	AA	GG	AG	AG	AG	AG	--	AG	AG	GG	AG
AA	AC	AA	CC	AC	AC	AC	AC	AC	AC	AC	CC	AC
AA	AG	AA	GG	AG	AG	AG	AG	AG	AG	AG	GG	AG
AA	AG	AA	GG	AG	AG	AG	AG	AG	AG	AG	GG	AG
GG	GT	GG	TT	GT	GT	GT	GT	GT	GT	GT	TT	GT
--	GT	TT	GG	GT	GT	GT	GT	GT	GT	GT	GG	GT
CC	AC	CC	AA	AC	AC	AC	AC	AC	AC	AC	AA	AC
TT	CT	TT	--	CT	CT	CT	CT	CT	CT	CT	CC	CT
TT	CT	TT	CC	CT	CT	CT	CT	CT	CT	CT	CC	CT
GG	GT	GG	TT	GT	GT	GT	GT	GT	GT	GT	TT	GT
AA	AT	AA	TT	AT	AT	AT	AT	AT	AT	AT	TT	AT
GG	CG	GG	CC	CG	CG	CG	CG	CG	CG	CG	CC	CG
AA	AC	AA	CC	AC	AC	AC	AC	--	--	AC	CC	AC
CC	CT	CC	TT	CT	CT	CT	CT	--	CT	CT	TT	CT
GG	CG	GG	CC	CG	CG	CG	CG	CG	CG	CG	CC	CG
CC	CT	CC	TT	CT	CT	CT	CT	CT	CT	CT	TT	CT
AA	AT	AA	TT	AT	AT	AT	AT	AT	AT	AT	TT	AT
TT	CT	TT	CC	CT	CT	CT	CT	CT	CT	CT	CC	CT
GG	AG	GG	AA	AG	AG	AG	GG	AG	AG	AG	AA	AG
AA	AT	AA	TT	AT	AT	AT	AT	AT	TT	AT	TT	AT
AA	AT	AA	TT	AT	AT	AT	AA	--	AT	AT	TT	AT
AA	AC	AA	CC	AC	AC	AC	AA	AC	AC	AC	CC	AC

TT	GT	TT	GG	GT	GT	GT	TT	GT	GT	GT	GG	GT
GG	AG	GG	AA	AG	AG	AG	GG	AG	AG	AG	AA	AG
GG	GT	GG	TT	GT	GT	GT	GG	GT	GT	GT	TT	GT
AA	AG	AA	GG	AG	AG	AG	AA	AG	AG	AG	GG	AG
TT	CT	TT	CC	CT	CT	CC	TT	CT	CT	CT	CC	CT
AA	AG	AA	GG	AG	AG	AG	AA	AG	AG	AG	GG	AG
TT	CT	TT	CC	CT	CT	CT	TT	CT	CT	CT	CC	CT
CC	AC	CC	AC	AC	AC	AC	CC	AA	AC	AC	AA	AC
AA	AG	AA	GG	AG	AG	AG	AA	GG	AG	AG	GG	AG
TT	CT	TT	CC	CT	CT	CT	TT	CC	CT	CT	CT	CT
GG	AG	GG	AA	GG	AG	AG	GG	AG	AG	AG	AG	AG
CC	CC	CC	TT	CC	--	CT	--	CT	CT	CT	CT	TT
GG	GG	GG	GT	GG	GT	GG	GG	GT	--	GT	GG	GT
GG	GG	GG	AG	GG	GG	AG	GG	GG	AG	GG	AG	AG
TT	TT	TT	AA	TT	AT	AT	TT	--	AT	AT	TT	AA
TT	TT	TT	AA	TT	AT	AT	AT	AA	AT	AT	TT	AA
CC	CC	CC	AA	CC	CC	AC	AC	--	AC	AC	CC	AA
--	GG	GG	TT	GG	GG	GT	GT	TT	GT	GT	GG	TT
CT	CT	CT	CT	TT	TT	TT	CT	TT	CT	TT	TT	TT
GG	AA	AG	AA	AG	GG	GG	AA	AG	--	AG	AG	AG
CG	GG	CG	GG	GG	CG	CG	GG	--	GG	GG	GG	GG
CT	TT	CT	--	TT	CT	CT	TT	TT	TT	TT	TT	TT
GT	GG	GT	GG	GT	GT	GT	GG	GT	GG	GT	GT	GT
GG	AA	GG	AA	GG	GG	GG	AA	--	AA	AG	AG	AG
AC	AA	AC	AA	AC	AC	AC	AC	AA	AA	AA	AC	AA
CG	GG	CG	GG	CG	CG	CG	CG	--	GG	GG	CG	GG
GT	TT	GT	TT	--	GT	GT	GT	--	TT	TT	GT	TT
TT	CC	TT	CC	TT	CT	CT	TT	CT	CC	CT	CT	CT
AG	AA	AG	AG	AG	GG	AA	AG	--	AG	AG	AA	AA
CT	CT	CC	CC	CT	CC	CT	CT	--	CC	CT	CT	CT
CC	CC	CG	CG	CC	CG	CC	CC	CG	CG	CC	CC	CC
AA	AA	AA	AC	AA	AA	--	AA	AC	AC	AC	AC	AC
AA	AA	AG	GG	AA	AG	AG	AA	GG	GG	AG	AG	AG
CT	CT	CT	TT	CT	CT	TT	CT	--	TT	--	TT	TT
CG	CG	GG	GG	CG	GG	CG	CG	GG	GG	CG	CG	CG
GG	GG	AG	AA	GG	AG	AG	GG	AA	AA	AG	AG	AG
GG	GG	CG	CG	GG	CG	GG	GG	CG	CG	GG	GG	GG
--	GG	CG	CC	GG	CG	CG	GG	--	CC	CG	CG	CG
TT	TT	CT	CC	TT	CT	CT	TT	CT	CC	CT	CT	CT
AA	AA	AG	GG	AA	AG	AG	AA	AG	GG	AG	AG	AG
GG	GG	GG	AG	GG	GG	AG	GG	GG	AG	AG	AG	AG
TT	TT	CT	CC	TT	CT	CT	TT	CC	CC	CT	CT	CT
AG	AG	AG	GG	AG	AG	GG	AG	GG	GG	GG	GG	GG
AA	AA	AG	GG	AA	AG	AG	AA	GG	GG	AG	AG	AG
AA	AA	AC	CC	AA	AC	AC	AA	CC	--	AC	AC	AC
AT	AT	TT	TT	AT	TT	AT	AT	TT	TT	AT	AT	AT
GG	GG	AG	AA	GG	AG	AG	GG	--	AA	AG	AG	AG

CC	CC	AC	AA	CC	AC	AC	CC	--	AA	AC	AC	AC
GG	GG	AG	AA	GG	AG	AG	GG	AA	AA	AG	AG	AG
AG	AG	AG	AA	AG	AG	AA	AG	--	AA	AA	AA	AA
CT	CT	CT	CC	CT	CC	CT	CT	--	CC	CC	CT	CT
CC	CG	CG	CC	CG	CC	CG	CG	CC	CC	CC	CG	CG
TT	CT	CT	TT	CT	TT	CT	CT	--	--	TT	CT	CT
CG	GG	GG	CG	GG	CG	GG	GG	--	CG	CG	GG	GG
CC	CC	CT	CC	CT	CC	CT	CT	CC	CC	CC	CT	CT
GT	GT	GG	GT	GT	GT	GG	GG	GT	GT	GT	GT	GG

6-51	6-52	6-54	6-57	6-62	7-1	7-2	7-3	7-4	7-8	7-9	7-10	7-11	7-12	7-13
AC	AC	CC	CC	CC	AC	AC	CC	AA	AA	AC	AC	AC	AC	AC
AG	AG	GG	AG	AG	AA	AG	GG	AA	AA	AG	AG	AG	AG	AG
CC	CT	CC	CT	CT	TT	CC	CC	TT	CT	CT	CT	CT	CT	CT
AG	GG	AG	GG	AG	GG	AG	AG	GG	GG	GG	GG	AG	GG	GG
AG	AG	GG	AG	AG	AA	GG	GG	AA	AG	GG	AG	AG	--	AG
CG	--	GG	--	CG	CG	GG	GG	CG	GG	CG	GG	CG	GG	GG
CT	TT	CC	CT	CT	TT	CT	CC	TT	CT	TT	CT	CT	CT	CT
GT	TT	GG	GT	GT	TT	GT	GT	TT	GT	TT	GT	GT	TT	GT
GT	TT	GG	GT	GT	TT	GT	GT	TT	GT	TT	GT	GT	TT	GT
GT	TT	GT	GT	GT	TT	GT	GT	TT	GT	TT	GT	GT	TT	GT
AG	AA	AG	AG	AG	AA	AG	AG	AA	GG	AA	AG	AG	AA	AG
CT	CT	CT	CC	CT	CT	CT	CT	CT	CC	CT	CC	CT	CT	CC
AC	AA	CC	AC	AA	AA	CC	AC	AA	CC	AA	AA	AA	AA	AC
AG	AG	AA	AA	AG	AG	AA	AG	AG	AA	AG	AG	AG	AG	AA
CC	CT	CC	CT	CT	CT	CC	CC	CT	CC	CT	CT	CT	CT	CT
AG	AG	AA	GG	GG	GG	AA	AG	GG	AA	GG	GG	GG	AG	AG
AT	TT	TT	AT	AT	AT	TT	AT	AT	TT	AT	AT	AT	TT	TT
CC	CT	CC	CT	CT	CT	CC	CC	CT	CC	CT	CT	CT	CT	CT
GT	TT	TT	GT	GT	GT	TT	GT	TT	GT	GT	GT	GT	TT	GT
CG	CC	CC	CG	CG	CG	CC	CG	CC	CG	CC	CG	CG	CC	CG
AT	AT	TT	AA	AT	AA	TT	AT	--	AT	AT	AA	AA	AT	AA
CT	CT	TT	CC	CT	CC	TT	CC	CT	CT	CT	CC	CC	CT	CC
AT	AT	AA	TT	AT	TT	AA	TT	AT	AT	AT	TT	TT	AT	TT
AT	AT	AA	TT	AT	TT	AA	TT	AT	AT	AT	TT	TT	AT	TT
AG	AG	AA	GG	AG	GG	AA	GG	AG	AG	AG	GG	GG	AG	GG
GT	TT	TT	GT	TT	GT	TT	GT	--	GT	TT	GT	GT	TT	GT
GT	GT	TT	GG	GT	GG	TT	GG	GT	GT	GT	GG	GG	GT	GG
AT	AT	TT	AA	AT	AA	TT	AA	AT	AT	AT	AA	AA	AT	AA
CT	CT	TT	CC	CT	CC	TT	CC	CT	CT	CT	CC	CC	CT	CC
CG	CG	CC	GG	CG	GG	CC	GG	CG	CG	CG	GG	GG	CG	GG
CT	CT	CC	TT	CT	TT	CC	TT	CT	CT	CT	TT	TT	CT	TT
AC	AC	AA	CC	AC	CC	AA	CC	AC	AC	AC	CC	CC	AC	CC
AG	AG	AA	GG	AG	GG	AA	GG	AG	AG	AG	GG	GG	AG	GG
GT	GT	GG	TT	GT	TT	GG	TT	GT	GT	GT	TT	TT	GT	TT
AG	AG	AA	GG	AG	GG	AA	GG	AG	AG	AG	GG	GG	AG	GG
GT	GT	TT	GG	GT	GG	TT	GG	GT	GT	GT	GG	GG	GT	GG
AG	AG	GG	AA	AG	AA	GG	AA	AG	AG	AG	AA	AA	AG	AA
CT	CT	CC	TT	CT	TT	CC	TT	CT	--	CT	TT	TT	CT	TT
AT	AT	AA	TT	AT	TT	AA	TT	AT	AT	AT	TT	TT	AT	TT
AG	AG	AA	GG	AG	GG	AA	GG	AG	AG	AG	GG	GG	AG	GG
AC	AC	AA	CC	AC	CC	AA	CC	AC	AC	AC	CC	CC	AC	CC
AG	AG	AA	GG	AG	GG	AA	GG	--	--	AG	GG	GG	AG	GG

CT	CT	TT	CC	CT	CC	TT	CC	CT	CT	CT	CC	CC	CT	CC
CG	CG	CC	GG	CG	GG	CC	GG	CG	--	CG	GG	GG	CG	GG
CT	CT	CT	CC	CT	CC	TT	CC	CT	CT	CT	CC	CC	CC	CC
CT	CT	CT	TT	CT	TT	CC	TT	CT	CT	CT	TT	TT	TT	TT
AC	AC	AC	CC	AC	CC	AA	CC	AC	AC	AC	CC	CC	CC	CC
CC	TT	CT	CT	CT	TT	CT	TT	CT	CT	CT	CT	CT	TT	TT
TT	CC	CT	CT	CT	CT	CT	CC	CT	CT	CT	CT	CT	CC	CT
GG	GG	GG	AG	AG	AA	AG	AA	--	AA	AA	GG	AG	GG	GG
GG	GG	GT	TT	GT	TT	GT	TT	GT	TT	TT	GG	GT	GG	GG
GG	GG	AG	AA	AG	AA	AG	AA	AG	AG	AA	GG	AA	AG	GG
TT	CT	CT	CC	CT	CC	CT	CC	CT	CT	CC	TT	CC	CT	TT
CC	CG	CG	GG	CG	GG	CG	GG	CG	CG	GG	CC	GG	CG	CC
AA	AG	AG	GG	AG	GG	AG	GG	AG	AG	GG	AA	GG	AG	AA
CC	AC	AC	AA	AC	AA	AC	AA	AC	AC	AA	CC	AA	AC	CC
AG	AG	AG	GG	AG	GG	AG	GG	AG	--	GG	AG	GG	AG	AG
TT	CT	CT	CT	CT	CC	CT	CC	CT	CT	CC	CT	CC	CT	TT
CT	CC	CC	CT	CT	TT	CT	TT	CT	CT	TT	CT	CT	CT	CC
CT	TT	TT	CT	CT	CC	CT	CC	CT	CT	CC	CT	CT	CT	--
AG	AA	AA	AG	AG	GG	AG	GG	AG	AG	GG	AG	AG	AG	AA
AC	AA	AA	CC	AC	CC	AC	CC	AC	CC	CC	AC	AC	AC	AA
AG	AA	AA	GG	AG	GG	AG	AG	AG	GG	GG	AG	AG	AG	AG
CG	GG	GG	CG	GG	CC	CG	CG	CG	CC	CC	CG	CG	CG	GG
CG	CC	CC	CG	CC	GG	CG	CG	CG	GG	GG	GG	CG	CG	CC
AG	AA	AG	AG	AA	GG	AG	AG	AG	GG	GG	GG	AG	AG	AA
AC	CC	AC	CC	CC	AA	AC	AC	AC	AA	AC	AA	AC	AC	CC
CT	TT	CC	TT	TT	CC	CT	CT	CT	CC	CT	CC	CT	CT	TT
CT	CC	TT	CC	CC	CT	CT	CT	--	CT	CT	TT	CT	CT	CC
AT	TT	AA	TT	TT	AT	AA	AT	AT	AT	AT	AA	AT	AT	TT
AG	GG	AG	GG	GG	AG	AG	--	--	AG	AG	AG	AG	GG	GG
TT	TT	AT	TT	TT	TT	AT	TT	AT	TT	TT	AT	TT	AT	AT
TT	GT	GT	TT	TT	GT	GT	GT	GG	GT	TT	TT	TT	TT	--
GG	GG	GG	--	GG	AG	GG	GG	AG	AG	GG	GG	GG	GG	AG
TT	TT	TT	TT	TT	CT	TT	TT	CT	CT	TT	TT	TT	TT	CT
GG	GG	GG	GG	GG	AG	GG	GG	AG	--	GG	GG	GG	GG	AG
GG	GT	GT	GG	GG	GT	TT	GT	GT	GG	GG	GG	GG	GG	GT
GG	GG	GG	GG	GG	GT	GT	GG	GT	GG	GT	GG	GG	GG	GG
CT	CT	CT	TT	TT	CT	CC	CT	CT	TT	CT	TT	TT	TT	CT
CT	CT	CT	CT	TT	CT	CC	CT	CT	TT	CT	CT	--	TT	CT
AG	AG	AG	AG	AA	AG	GG	AG	AG	AA	AG	AG	AA	AG	AG
CG	--	CG	CG	GG	CG	CC	CG	CG	GG	CG	CG	CG	CG	CG
AG	AG	GG	AG	AA	AG	GG	AG	AG	AA	AG	AG	AG	AG	AG
CT	TT	TT	CT	CC	CT	TT	CT	CT	CC	CT	CT	CT	CT	CT
CT	TT	TT	CT	CC	CT	TT	CT	CT	CC	CT	CT	CT	CT	CT
CC	CG	CG	CG	CC	CG	CG	CC	CG	CC	CG	CG	CG	CC	CC
TT	AT	AT	AT	TT	AT	AT	TT	AT	--	TT	AT	AT	TT	TT
GT	GT	GT	GG	GG	GG	GT	GT	GG	GG	GT	GG	GG	GT	GT
CT	CT	CT	CC	CC	CC	CT	CT	CC	CC	CT	CC	CC	CT	CT

CT	TT	TT	CT	CC	CT	TT	TT	--	CC	CT	CC	CT	TT	CT
GT	GT	GT	GG	GG	GG	GT	GT	GG	GG	GT	GG	GG	GT	GT
CT	TT	TT	CT	CC	CT	TT	TT	CC	CC	CT	CC	CT	TT	CT
GT	GT	GT	TT	TT	TT	GT	GT	TT	TT	GT	TT	TT	GT	GT
CG	CG	CG	GG	GG	GG	CG	CG	GG	--	CG	GG	GG	CG	CG
GG	AA	AG	AA	AA	AA	GG	AA	GG	AA	GG	AG	AA	AG	GG
AA	GG	AG	GG	GG	GG	AA	GG	--	GG	AA	AA	GG	AG	AA
AA	GG	AG	GG	GG	GG	AA	GG	AA	AG	AA	AA	GG	AG	AA
TT	CC	CT	CT	CC	CT	TT	CC	CT	CT	TT	TT	CC	CT	TT
GG	AG	AA	AG	AA	AG	GG	AA	AG	AG	GG	GG	AA	AG	GG
GG	GT	TT	GT	TT	GT	GG	TT	--	GT	GG	GG	TT	GT	GG
AG	AG	GG	GG	GG	AG	AG	AG	GG	AG	AG	AG	GG	GG	AG
TT	CT	CC	CT	CC	CT	CT	CT	CT	CT	TT	TT	CC	CC	TT
CC	AC	AC	CC	AC	AC	CC	AC	--	AC	CC	CC	AC	AC	CC
CT	CC	CC	CT	CC	CC	CT	CC	CT	CC	CT	CT	CC	CC	CT
GG	GT	TT	GT	GT	GT	GT	GT	GT	GT	GG	GG	TT	TT	GG
CC	CC	CT	CT	CC	CC	CT	CC	CT	CC	CC	CC	CT	CT	CC
GG	CG	CG	GG	CG	CG	GG	CG	GG	CG	GG	GG	CG	CG	GG
CC	AC	AC	CC	AC	AC	CC	AC	CC	--	CC	CC	AC	AC	CC
TT	TT	CT	CT	TT	TT	CT	TT	CT	TT	TT	TT	CT	CT	TT
AA	AA	AG	AG	AA	AA	AG	AA	AG	--	AA	AA	AG	AG	AA
AA	AG	GG	AG	AG	AG	AG	AG	AG	AG	AA	AA	GG	GG	AA
CT	CC	CC	CT	CC	CC	CT	CC	CT	CC	CT	CT	CC	CC	CT
AA	AT	AT	AA	AT	AT	AA	AT	AA	AT	AA	AT	AT	AT	AA
CC	CT	CT	CC	CT	CT	CC	CT	CC	CT	CC	CT	CT	CT	CC
AA	AG	AG	AA	AG	AG	AA	AG	AA	AG	AA	AA	AG	AG	AA
CC	CC	CT	CT	CC	CC	CT	CC	CT	CC	CC	CC	CT	CT	CC
TT	AT	AA	AT	AT	AT	AT	AT	AT	--	TT	TT	AA	AA	TT
TT	AT	AA	AT	AT	AT	AT	AT	AT	AT	TT	TT	AA	AA	TT
TT	CT	CC	CT	CT	CT	CT	CT	CT	CT	TT	TT	CC	CC	TT
CC	CT	CT	CC	CT	CT	CC	CT	CC	CT	CC	CC	CT	CT	CC
GG	GG	GT	GT	GG	GG	GT	GG	GT	GG	GG	GG	GT	GT	GG
CC	CC	CT	CT	CC	CC	CT	CC	CT	CC	CC	CC	CT	CT	CC
AG	AG	AA	AA	AG	AG	AA	AG	AA	AG	AG	AG	AA	AA	AG
GG	AG	AA	GG	AG	AG	AG	AG	AG	AG	GG	GG	AA	AA	GG
AA	AA	AG	AA	AA	AA	AG	AA	AG	AA	AA	AA	AG	AG	AA
CC	CT	TT	CC	CT	CT	CT	CT	CT	CT	CC	CC	TT	TT	CC
CC	CC	CC	CC	CC	CC	AC	CC	AC	CC	--	CC	AC	AC	CC
TT	AT	AT	TT	AT	AT	AT	AT	AT	AT	TT	TT	AA	AA	TT
AA	AG	AG	AA	AG	AG	AA	AG	AA	AG	AA	AA	AG	AG	AA
AA	AA	AA	AA	AA	AA	AC	AA	AC	AA	AA	AA	AC	AC	AA
TT	TT	TT	TT	TT	TT	AT	TT	AT	TT	TT	TT	AT	AT	TT
AC	CC	CC	AC	CC	CC	AA	CC	AA	CC	AC	AC	AC	AC	AC
CC	AC	AC	CC	AC	AC	CC	AC	CC	AC	CC	CC	AC	AC	CC
AG	AG	AG	AG	AG	AG	GG	AG	--	AG	AG	AG	GG	GG	AG
AC	CC	CC	AC	CC	CC	AC	CC	AC	CC	AC	AC	CC	CC	AC
AA	AA	AA	AA	AA	AA	AG	AA	--	AA	AA	AA	AG	AG	AA

CC	CG	CG	CC	CG	CG	CC	CG	CC	CG	CC	CC	CG	CG	CC
TT	CT	CT	TT	CT	CT	CT	CT	CT	CT	TT	TT	CC	CC	TT
CC	CT	CT	CC	CT	CT	CT	CT	CT	CT	CC	CC	TT	TT	CC
CC	CC	CC	CC	CC	CC	CT	CC	--	CC	CC	CC	CT	CT	CC
AA	AA	AA	AA	AA	AA	AG	AA	AG	AA	AA	AA	AG	AG	AA
CC	CT	CT	CC	CT	CT	CT	CT	CT	CT	CC	CC	TT	TT	CC
TT	TT	TT	TT	TT	TT	CT	TT	CT	TT	TT	TT	CT	CT	TT
AA	AT	AT	AA	AT	AT	AT	AT	AT	AT	AA	AA	TT	TT	AA
GG	AG	AG	GG	AG	AG	GG	AG	GG	AG	GG	GG	AG	AG	GG
AA	AA	AA	AA	AA	AA	AC	AA	AC	AA	AA	AA	AC	AC	AA
CC	CC	CC	CC	CC	CC	CT	CC	CT	CC	CC	CC	CT	CT	CC
TT	GT	GT	TT	GT	GT	TT	GT	TT	GT	TT	TT	GT	GT	TT
TT	TT	TT	TT	TT	TT	CT	TT	CT	TT	TT	TT	CT	CT	TT
AC	AC	AC	AC	AC	AC	CC	AC	CC	AC	AC	AC	CC	CC	AC
AA	AA	AA	AA	AA	AA	AG	AA	AG	AA	AA	AA	AG	AG	AA
GG	GG	GG	GG	GG	GG	GT	GG	GT	GG	GG	GG	GT	GT	GG
AT	AT	AT	AT	--	AT	TT	AT	TT	AT	AT	AT	TT	TT	AT
AA	AA	AA	AA	AA	AA	AG	AA	AG	AA	AA	AA	AG	AG	AA
CC	CT	CT	CC	CT	CT	CC	CT	CC	CT	CC	CC	CT	CT	CC
CC	CC	CC	CC	CC	CC	CT	CC	CT	--	CC	CC	CT	CT	CC
AA	AA	AA	AA	AA	AA	AC	AA	AC	--	AA	AA	AC	AC	AA
CC	CC	CC	CC	CC	CC	CT	CC	CT	CC	CC	CC	CT	CT	CC
AA	AT	AT	AA	AT	AT	AT	--	AT	AT	AA	AA	TT	TT	AA
GG	AG	AG	GG	AG	AG	AG	AG	AG	AG	GG	GG	AA	AA	GG
GG	CG	CG	GG	CG	CG	GG	CG	GG	CG	GG	GG	CG	--	GG
TT	TT	TT	TT	TT	TT	CT	TT	CT	TT	TT	TT	CT	CT	TT
GG	GG	GG	GG	GG	GG	--	GG	AG	AG	GG	GG	AG	AG	GG
AA	AA	AA	AA	AA	AA	AG	AA	AG	AG	AA	AA	AG	AG	AA
AA	AG	AG	AA	AG	AG	AA	AG	AA	AG	AA	AG	AA	AG	AA
GG	AG	AG	GG	AG	AG	GG	AG	GG	AG	GG	--	GG	AG	GG
CT	TT	TT	TT	TT	TT	CT	TT	CT	CT	CT	TT	CT	CT	CT
GT	GT	GT	GG	GT	GT	GT	GT	GT	TT	GT	GT	GT	TT	GT
TT	GT	GT	TT	GT	GT	TT	GT	TT	GT	TT	GT	TT	GT	TT
AG	AG	AG	GG	AG	AG	AG	AG	--	AA	AG	AG	AG	AA	AG
CT	CC	CC	CT	CC	CC	CT	CC	CT	CT	CT	CC	CT	CT	CC
GT	TT	TT	GT	TT	TT	GT	TT	TT	GT	GT	TT	GT	GT	TT
AG	GG	GG	AG	GG	GG	AG	GG	AG	GG	AG	GG	GG	GG	AG
AG	AG	AG	GG	AG	GG	AG	AG	AA	GG	AG	AA	AG	AA	GG
CC	CC	--	CC	CT	CC	CT	CT	CT	CC	CC	CT	CC	--	CC
AA	AA	AT	AT	AT	AT	AT	AA	AA	AT	AA	AA	AA	AA	AT
CG	CG	CG	GG	CG	GG	CG	CC	CC	GG	CG	CG	CG	CC	CG
AC	AC	AA	AC	AA	AC	AA	AA	AA	AC	AC	AC	AA	AA	AC
AG	AG	AG	GG	AG	GG	AG	AA	AA	GG	--	GG	AA	AG	AG
AG	AG	GG	GG	GG	GG	GG	AG	AG	GG	AG	GG	AG	GG	AG
CT	CT	CC	CC	CC	CC	CC	CT	CT	CC	CT	CC	CT	CC	CT
AG	AG	AG	AA	AG	AA	AG	GG	GG	AG	AG	AA	GG	AG	AG
CT	CT	CC	CT	CC	CC	CC	CC	CC	CC	CT	CT	CC	CC	CT

GG	GG	AG	AG	AG	AG	AG	GG	GG	AG	GG	AG	GG	AG	GG
GG	GG	AA	AG	AA	AA	AA	AG	AG	AA	AG	AG	AG	AA	GG
CT	CT	CT	TT	CT	CT	CT	CC	CC	CT	CC	TT	CC	CT	CT
AT	AT	AT	TT	AA	AT	AT	AA	AA	AT	AA	TT	AA	TT	AT
AC	AC	AC	CC	AA	AC	AC	AC	AC	AC	AA	CC	AA	CC	AC
CT	CT	CT	CC	TT	CT	CC	CT	CT	CT	TT	CC	CT	CC	CT
AG	AA	AG	AA	GG	AG	AA	AG	AG	AA	GG	AA	AG	AA	AG
CT	TT	CT	TT	CC	CT	TT	CT	CT	TT	CC	TT	CT	TT	CT
CT	CT	TT	CT	CT	TT	CT	CT	CT	CT	TT	CT	CT	CT	CT
AG	GG	AG	GG	AG	AG	GG	AG	--	GG	AA	GG	AG	GG	AG
AG	AG	AG	AA	GG	AG	AA	AG	AG	AG	--	AG	AG	AA	AA
CT	CT	CT	CC	TT	CT	CC	CT	CT	CT	CT	CT	CT	CC	CT
AG	AG	GG	GG	AG	GG	GG	GG	GG	--	GG	AG	GG	GG	GG
CT	CT	CT	CC	CT	TT	CC	CT	CT	CT	CC	CT	CT	CC	CC
GG	GG	GG	GG	GG	CG	GG	CG	CG	GG	CG	GG	CG	GG	GG
CT	CT	CC	CC	CT	TT	CC	CT	CT	CC	TT	CT	CT	CC	CC
CT	CT	CC	CC	CT	CT	CC	CC	CC	CC	CT	CT	CC	CC	CC
AG	AG	GG	GG	AG	AA	GG	AG	AG	GG	AG	AG	AG	GG	GG
CT	CT	CC	CC	CT	TT	CC	CT	CT	CC	CT	--	CT	CC	CC
AG	AG	AA	AA	AG	GG	GG	AG	AG	AA	AG	AG	AG	AA	AA
TT	CT	CC	CC	CT	TT	TT	CT	CT	CC	CT	CT	CC	CC	CC
AG	AG	GG	GG	AG	AA	AA	AG	AG	AG	AG	AG	AG	GG	GG
GG	AG	GG	AG	AG	AG	AG	GG	GG	AG	AG	AG	AG	GG	GG
GT	GT	TT	GT	TT	GG	GG	GT	GT	GT	GT	GT	GT	GT	GT
CC	CG	CC	CG	CC	CG	CG	CG	CC	CG	CG	CG	CG	CG	CC
CC	CG	CG	CG	CG	CC	CC	CG	CC	CG	CG	CC	CG	CG	CC
CT	CT	CC	CT	CC	TT	TT	CT	CT	CT	CT	TT	CT	CT	CT
CT	CC	TT	CT	TT	CC	CC	CT	CT	CT	CT	CC	CT	TT	CT
AT	TT	AA	AT	AA	TT	TT	AT	TT	AA	AT	TT	AT	AA	AT
AG	AA	GG	AG	GG	AA	AA	AG	AA	GG	AG	AA	AG	GG	AA
CT	TT	CC	CT	CC	TT	TT	CT	TT	CC	CT	TT	CT	CC	TT
TT	CT	TT	CT	TT	CT	CT	CT	CT	TT	CT	CT	CT	TT	CT
AC	CC	AA	AC	AA	--	CC	AC	CC	AA	AC	CC	AC	AA	CC
GT	GT	TT	GT	GT	GT	GT	GT	--	GT	TT	TT	TT	GT	GT
AG	AG	AA	AG	AG	AG	AG	AG	GG	AG	AA	AA	AA	AG	AG
CT	CT	TT	CT	CT	CT	CT	CT	CC	CT	TT	TT	TT	CT	CT
AT	AT	AA	AT	AT	AT	AT	AT	TT	AT	AA	AA	AA	AT	AT
AC	AC	CC	AC	AC	AC	AC	AC	AA	AC	CC	CC	CC	AC	AC
CG	CG	CG	CG	CG	CG	CC	CG	CC	CC	CG	CG	CG	CC	CC
AG	AG	AG	AG	AG	AG	AA	AG	AA	AA	AG	AG	AG	AA	--
CT	CT	CC	CT	CT	CT	CT	CT	TT	CT	CC	CC	CC	CT	CT
CT	CT	CC	CT	CT	CT	CT	CT	TT	CT	CC	CC	CC	CT	CT
AG	AG	AA	AG	AG	AG	AG	AG	GG	AG	AA	AA	AA	AG	AG
CC	CC	CT	CC	CC	CC	CT	CC	CC	CT	CT	CT	CT	CT	CT
AG	AG	AA	AG	AG	AG	AG	AG	GG	AG	AA	AA	AA	AG	AG
--	AG	GG	AG	AG	--	AG	AG	--	AG	GG	GG	GG	AG	AG
GT	GT	GG	GT	GT	GT	GT	GT	TT	GT	GG	GG	GG	GT	GT

CT	CT	TT	CT	CT	CT	CT	CT	--	CT	TT	TT	CT	CT	CT
CG	CG	GG	CG	CG	CG	CG	CG	CC	CG	GG	GG	CG	GG	CG
GT	GT	TT	GT	GT	GT	GT	GT	GG	GT	TT	TT	GT	TT	GT
AG	AG	AA	AG	AG	AG	AG	AG	GG	AG	AA	AA	AG	AA	AG
CT	CT	CC	CT	CT	CT	CT	CT	TT	CT	CC	CC	CT	CC	CT
TT	TT	GT	TT	TT	TT	GT	TT	TT	GT	GT	GT	TT	GT	GT
CG	CG	GG	CG	CG	CG	CG	CG	CC	CG	GG	GG	CG	GG	CG
AG	AG	AA	AG	AG	AG	AG	AG	GG	AG	AA	AA	AG	AA	AG
AG	GG	AG	GG	GG	GG	AG	GG	GG	AG	AG	AG	GG	AG	AG
AA	AC	AA	--	AC	AC	AC	AC	CC	AA	AA	AA	AC	AA	AC
CG	CC	CG	CC	CC	CC	CG	CC	CC	CG	CG	CG	CC	CG	CG
GG	AG	GG	AG	AG	AG	AG	AG	AA	AG	GG	GG	AG	--	AG
AA	AC	AA	AC	AC	AC	AC	AC	CC	AC	AA	AA	AC	AA	AC
TT	GT	TT	GT	GT	GT	GT	GT	GG	GT	TT	TT	GT	TT	GT
CC	AC	CC	AC	AA	AC	AC	AC	AA	AC	CC	CC	AC	CC	AC
CT	CC	CT	CC	CC	CC	CT	CC	CC	CT	CT	CT	CC	CT	CT
TT	TT	TT	GT	GG	GT	GT	GT	--	GT	TT	TT	GT	TT	GT
AG	AG	AG	AG	GG	AG	GG	AG	GG	GG	AG	AG	AG	AG	GG
CC	CC	CC	CT	TT	CT	CT	CT	TT	CT	CC	CC	CT	CC	CT
CT	CT	CT	CC	CC	CC	CT	CC	CC	CT	CT	--	CC	CT	CT
AA	AA	AA	AT	TT	AT	AT	AT	TT	AT	AA	AA	AT	AA	AT
CC	CC	CC	CG	GG	CG	CG	CG	GG	CG	CC	CC	CG	CC	CG
GG	GG	GG	GT	TT	GT	GT	GT	TT	GT	GG	GG	GT	GG	GT
AG	AG	AG	AG	GG	AG	GG	GG	GG	GG	AG	GG	AG	AG	GG
TT	TT	TT	GT	GT	GT	TT	TT	GT	--	GT	GT	GT	TT	TT
CC	CC	CC	CT	TT	CT	CT	CT	CT	CT	CT	TT	CT	CC	CT
CC	CC	AC	AC	AA	AC	AA	AC	AC	AC	AC	AA	AC	CC	AC
CC	CC	CT	CT	CT	CC	CT	CT	CC	CT	CC	CT	CC	CC	CT
GG	GG	CG	CG	CC	CG	CC	CG	CG	CG	CG	CC	CG	GG	CG
CC	CC	CT	CT	TT	CT	CT	CT	CC	CT	CT	TT	CT	CC	CC
GT	GT	TT	TT	TT	GT	GT	TT	GT	TT	GT	TT	GT	GT	GT
CT	CT	CT	CT	TT	TT	TT	CT	CT	CT	TT	TT	CT	CT	CT
AT	AT	TT	TT	TT	AT	AT	TT	AT	TT	AT	TT	AT	AT	AT
CC	CC	AC	AC	AC	CC	CC	AC	CC	AC	CC	AC	CC	CC	CC
TT	TT	CT	CT	CC	TT	TT	CC	--	CC	TT	CC	CT	TT	TT
AA	AA	AG	AG	AG	AA	AA	AG	AA	AG	AA	AG	AG	AA	AA
GG	GG	AG	AG	AG	GG	GG	AG	GG	AG	GG	AG	AG	GG	GG
GG	CG	GG	CG	GG	--	CG	GG	GG	GG	GG	GG	GG	GG	CG
CC	TT	CC	TT	CT	CC	TT	CT	CT	CT	CT	--	CT	CT	TT
CC	TT	CC	TT	CT	CC	TT	CT	CT	CT	CT	CC	CT	CT	TT
CT	TT	CC	TT	CT	CT	TT	CT	CT	CT	CT	CC	CT	CT	TT
CT	CT	TT	CC	CT	CT	CC	CT	CT	CT	CC	TT	CT	CT	CC
GT	GT	GG	TT	GT	GT	TT	GT	GT	TT	TT	GG	GT	GT	TT
CT	CT	TT	CC	CT	CT	CC	CT	CT	CC	CC	TT	CT	CT	CC
AT	AT	TT	AA	TT	AT	AA	AT	AT	AA	AA	AT	AT	AT	AA
AG	AG	GG	AA	GG	AG	AA	AG	AG	AA	AA	AG	AG	AG	AA
AC	AA	AA	CC	AA	AC	CC	AC	AC	CC	CC	AC	AC	AC	CC

AG	AG	GG	AA	GG	AG	AA	AG	AG	AA	AA	AG	AG	AG	AA
AG	AG	GG	AA	GG	AG	AA	AG	AG	AA	AA	AG	AG	AG	AA
CT	CT	CC	TT	CC	CT	TT	CT	CT	TT	TT	CT	CT	CT	TT
AG	AG	AA	GG	AA	AG	GG	--	AG	GG	GG	AG	AG	--	GG
GT	GT	TT	GG	TT	GT	GG	GT	GT	--	GG	GT	GT	GT	GG
CT	CT	CC	TT	CC	CT	TT	CT	CT	TT	TT	CT	CT	CT	TT
AG	AG	AA	GG	AA	AG	GG	AG	AG	GG	GG	AG	AG	AG	GG
AG	AG	AA	GG	AA	AG	GG	AG	AG	GG	GG	AG	AG	AG	GG
AG	AG	GG	AA	GG	AG	AA	AG	AG	AA	AA	AG	AG	AG	AA
CG	CG	CC	GG	CC	CG	GG	CG	CG	GG	GG	CG	CG	CG	GG
AG	AG	GG	AA	GG	AG	AA	AG	AG	AA	AA	AG	AG	AG	AA
AC	AC	CC	AA	CC	AC	AA	AC	AC	AA	AA	AC	AC	AC	AA
AG	AG	AA	GG	AG	AG	GG	AG	AG	GG	GG	AG	AG	AG	GG
AG	AG	AA	GG	AA	AG	GG	--	AG	GG	GG	AG	AG	AG	GG
AT	AT	TT	AA	TT	AT	AA	AT	AT	AA	AA	AT	AT	AT	AA
CT	CT	CC	TT	CC	CT	TT	CT	CT	TT	TT	CT	CT	CT	TT
CT	CT	CC	TT	CC	CT	TT	CT	CT	TT	TT	CT	CT	CT	TT
CG	CG	GG	CC	GG	CG	CC	CG	CG	CC	CC	CG	CG	CG	CC
CT	CT	TT	CC	TT	CT	CC	CT	CT	CC	CC	CT	CT	CT	CC
CT	CT	CC	TT	CC	CT	TT	CT	CT	TT	TT	CT	CT	CT	TT
CT	CT	TT	CC	TT	CT	CC	CT	CT	CC	CC	CT	CT	CT	CC
AT	AT	TT	AA	TT	AT	AA	AT	AT	AA	AA	AT	AT	AT	AT
AT	AT	AA	TT	AA	AT	TT	AT	AT	TT	TT	AA	AT	AT	AT
CT	CT	TT	CC	TT	CT	CC	CT	CT	CC	CC	TT	CT	CT	CT
CT	CT	CC	TT	CC	CT	TT	CT	CT	TT	TT	CC	CT	TT	CT
AA	AT	AA	TT	AA	AT	TT	AT	AT	TT	TT	AA	AT	TT	AT
GG	AG	GG	AA	GG	AG	AA	AG	AG	AA	AA	GG	AG	AA	AG
GG	GT	GG	GT	GG	GT	TT	GT	GT	TT	TT	GG	GT	TT	GT
TT	CT	TT	CT	TT	CT	CC	CT	CT	CC	CC	TT	CT	CC	CT
CC	CG	CG	CG	CC	CG	GG	CG	CG	GG	GG	CC	CG	GG	CG
GG	AG	AG	AG	GG	AG	AA	AG	AG	AA	AA	GG	AG	AA	AG
TT	CT	CT	CT	TT	CC	CC	CT	CC	CC	CC	TT	CT	CC	CT
TT	CT	CT	CT	TT	CT	CC	CT	CC	CC	CT	TT	CT	CC	CT
TT	AT	AT	AT	TT	AT	AA	AT	AA	AA	AT	TT	AT	AA	AT
AA	AG	AG	AG	AA	AG	GG	AG	GG	GG	AG	AA	AG	GG	AG
AA	AG	AG	AG	AA	AG	GG	AG	GG	GG	AG	AA	AG	GG	AG
TT	GT	GT	GT	TT	GT	GG	GT	GG	GG	GT	TT	GT	GG	TT
AA	AG	AG	AG	AA	AG	GG	AG	GG	--	AG	AA	AG	GG	AA
GG	AG	AG	GG	GG	AG	AG	GG	AG	--	GG	GG	GG	AG	GG
CT	CT	TT	TT	CT	TT	TT	TT	CT	CT	TT	TT	TT	TT	CT
AG	AG	AG	AG	AG	--	GG	GG	AG	AG	GG	AG	GG	AG	AG
GG	GG	AG	--	GG	GG	GG	GG	GG	GG	GG	AG	GG	AG	GG
AG	AG	AG	AG	AG	GG	GG	GG	AG	AA	GG	AG	GG	AG	AG
CG	CG	CG	CG	CG	CC	CC	CC	CG	GG	CC	CC	CG	CG	CG
CT	CT	CT	CT	CT	CT	TT	TT	CT	CC	TT	TT	CT	CT	CT
CC	CC	AC	AC	CC	AC	AC	AC	CC	CC	AC	AC	CC	AC	CC
AG	AG	AA	AG	AA	AA	AG	AG	AG	--	AG	AG	AG	AA	AG

CC	CC	AC	AC	CC	AC	AC	AC	CC	AC	AC	AC	CC	AC	CC
CC	CC	AC	CC	AC	AC	CC	CC	CC	AC	CC	CC	CC	AC	CC
TT	TT	CT	CT	TT	CT	CT	CT	TT	CT	CT	CT	TT	CT	TT
AG	AG	AG	GG	AA	AG	GG	GG	AG	AG	GG	GG	AG	AG	AG
AG	AG	AG	AA	GG	AG	AA	AA	AG	AA	AA	AA	AG	AG	AG
CT	CT	CT	CC	TT	TT	CC	CC	CT	CT	CC	CT	CT	CT	CT
TT	TT	CT	TT	CT	CT	TT	TT	TT	CT	TT	CT	TT	CT	TT
CG	CG	CG	GG	CC	CC	GG	CG	CG	CG	GG	CG	CG	CG	CG
AG	AG	AG	GG	AA	AA	GG	AG	AG	GG	AG	AG	AG	AG	AG
CT	CT	CC	CT	TT	TT	CC	CT	CT	--	CT	CT	CT	CT	CT
AA	AA	AA	AA	AT	AT	--	AT	AA	AA	AT	AT	AA	AT	AA
AC	AC	AA	AC	AC	CC	AA	AC	AC	AA	AC	AC	AC	AC	AC
GG	GG	GG	GG	GG	GG	AG	AG	GG	AG	AG	AG	GG	AG	GG
CC	CC	CC	CC	CC	CC	CT	CT	CC	CT	CT	CT	CC	CT	CC
CC	CC	CC	CC	CC	CC	CT	CT	CC	CT	CT	CT	CC	CT	CC
GG	GG	GG	GG	GG	GG	GT	GT	GG	GT	GT	GT	GG	GT	--
TT	CT	TT	TT	TT	CT	TT	CT	CC	CC	CT	TT	CC	CC	CT
GG	AG	GG	GG	GG	AG	GG	AG	AG	AG	AG	GG	GG	AG	AG
GG	AG	GG	AG	GG	AG	GG	AG	AA	AA	AG	GG	--	AA	AG
CG	CG	CG	GG	CG	CG	CG	GG	GG	GG	CG	CG	GG	GG	CG
GG	AG	GG	AG	GG	AG	GG	AG	AA	AA	AG	GG	AA	AA	AG
AA	AG	AA	AG	AA	AG	AA	AG	GG	GG	AG	AA	GG	GG	AG
TT	TT	GT	GT	TT	TT	TT	TT	GT	GT	TT	TT	GT	GT	TT
AA	AG	AG	AG	AA	AG	AA	AG	GG	--	AG	AA	GG	GG	AG
AA	AG	AA	AG	AA	AG	AA	AG	--	GG	AG	AA	GG	GG	--
GG	GG	GG	GT	GG	GG	GG	GG	GT	GT	GG	GT	GT	GT	GG
CC	CT	CC	CT	CC	CT	CC	CT	TT	TT	CT	CT	TT	TT	CT
CT	CT	TT	CT	TT	CT	TT	CT	CC	CT	CT	CT	CT	CT	CT
CC	CC	CC	CC	CC	CT	CC	CT	CT	CT	CT	CC	CT	CT	CT
AG	AG	AA	AG	AA	AG	AA	GG	GG	AG	AG	AG	AG	AG	AG
CT	CT	CC	CT	CC	CT	CC	TT	TT	CT	CT	CT	CT	CT	CT
AG	AG	AA	AG	AA	AA	AA	AG	GG	AG	AG	AG	AG	AG	AG
CT	CT	CC	CC	CC	CC	CC	CT	--	CT	CT	CT	CT	CT	CT
CC	CG	GG	CG	GG	GG	GG	CG	CG	CG	CG	CG	CG	CG	CG
TT	CT	CC	CT	CC	CC	CC	CT	--	CT	CT	CT	CT	CT	CT
AA	AA	AA	AC	AC	AC	AC	AC	AA	CC	AC	AC	--	AC	AA
GG	GG	GG	AG	AG	AG	AG	AG	GG	AA	AG	AG	AG	AG	GG
TT	TT	TT	CT	CT	CT	CT	CT	TT	CC	CT	CT	CT	CT	TT
AA	AA	AA	AG	AG	AG	GG	AG	AA	GG	AG	AG	AG	AG	AA
AA	AA	AA	AG	AG	AG	GG	AG	AA	GG	AG	AG	AG	AG	AG
TT	TT	TT	TT	CT	CT	TT	TT	TT	CT	CT	TT	TT	TT	TT
AG	GG	AG	AG	AG	AA	AG	AG	GG	AA	AG	AG	AG	AG	AG
AG	AG	AG	--	AA	AG	AG	AG	--	AG	AA	AG	AG	AG	AG
GT	GT	GT	GT	TT	GT	GT	GT	TT	GT	TT	GT	GT	GT	GT
GT	GT	GT	GT	TT	GT	GT	GT	TT	GT	TT	GT	GT	GT	GT
AC	AC	AC	AC	AC	CC	AA	AC	AA	CC	AC	AC	AC	AC	AC
AG	AG	AG	AG	AG	GG	AA	AG	--	GG	AG	AG	AG	AG	GG

CG	CG	CG	CG	GG	GG	CC	CC	CC	GG	CG	CG	CG	CG	GG
CG	CG	CG	CG	GG	GG	CC	CC	CC	GG	CG	CG	CG	CG	GG
CT	CT	CT	CT	CC	CC	TT	TT	TT	CC	CT	CC	CT	CT	CC
CT	CC	CT	CT	TT	TT	CC	CC	CT	TT	CT	TT	CT	CT	TT
AT	AA	AT	AT	TT	AT	AA	AA	AT	TT	AT	TT	AT	AT	TT
GG	AG	GG	GG	GG	AG	AG	AG	GG	GG	AG	GG	GG	GG	GG
CT	CC	CT	CT	TT	CT	CC	CC	CT	TT	TT	TT	CT	CT	TT
CT	TT	CC	CT	CC	CT	TT	TT	CT	CC	CC	CC	CT	CT	CT
CT	CC	TT	CT	TT	CT	CC	CC	CT	TT	TT	TT	CT	CT	CT
AG	GG	AG	AG	AA	AG	GG	GG	AG	AA	AA	AA	AG	AG	AG
CC	CC	CG	CG	CG	CG	CC	CG	CC	CG	CG	CG	CC	CC	CG
CG	GG	GG	GG	CG	GG	GG	GG	GG	--	CG	CG	CG	CG	GG
GG	GG	GT	GT	GT	GT	GG	GT	GG	GT	--	GT	GG	GT	GT
CC	CC	CG	CG	CG	CG	CG	CG	CC	CG	CC	CG	CC	CG	CC
GG	AG	AG	AG	AA	AG	AA	AG	GG	AG	GG	AA	AG	AA	GG
AC	AA	AC	AC	AC	AC	AC	AC	AA	AC	AA	AC	AA	AC	AA
AA	AA	AT	AT	AT	AT	AT	AT	AA	AT	AA	AT	AA	AT	AA
GG	AG	AG	AG	AA	AG	AA	AG	GG	AG	GG	AA	AG	AA	GG
AA	AT	AT	AT	AT	AT	TT	AT	AA	AA	AA	TT	AT	TT	AA
GG	AG	AG	AG	AG	AG	AA	AG	GG	GG	GG	AA	AG	AA	GG
CC	AC	CC	CC	CC	CC	AC	CC	CC	CC	CC	AC	AC	AC	CC
CC	CT	CT	CT	CC	CT	TT	CT	CC	CC	CC	TT	CT	TT	CC
TT	GT	GT	GT	GT	GT	GG	GT	TT	TT	TT	GG	GT	GG	GT
TT	GT	GT	GT	GT	GT	GG	GT	TT	TT	TT	GG	GG	GG	GT
CC	CT	CT	CT	CT	CT	TT	CT	CC	CC	CC	TT	TT	TT	CT
CC	CG	CG	CG	CG	CG	GG	CG	CC	--	CC	GG	GG	GG	CG
GG	AG	AG	AG	AG	AG	AA	AG	--	GG	GG	AA	AA	AA	AG
CC	CT	CT	CT	CT	CT	TT	CT	CC	CC	CC	TT	TT	TT	CT
TT	GT	GT	GT	GT	GT	GG	GT	TT	TT	TT	GG	GG	GG	GT
TT	CT	CT	CT	CT	CT	CC	CT	TT	TT	TT	CC	CC	CC	CT
GG	CG	CG	CG	CG	CG	CC	CG	GG	GG	GG	CC	CC	CC	CG
GG	AG	AG	AG	AG	AG	AA	AG	--	GG	GG	AA	AA	AA	AG
AA	AC	AC	AC	AC	AC	CC	AC	AA	AA	AA	CC	CC	CC	AC
TT	CT	CT	CT	CT	CT	CC	CT	TT	TT	TT	CC	CC	CC	CT
GG	GG	AG	AG	GG	AG	AG	AG	--	GG	GG	AG	AG	AG	--
AA	--	AC	AC	AC	AC	CC	AC	AA	AA	AA	CC	CC	CC	AC
CC	CT	CT	CT	CT	CT	TT	CT	CC	CC	CC	TT	TT	TT	CT
GG	AG	AG	AG	AG	AG	AA	AG	GG	GG	GG	AA	AA	AA	AG
GG	CG	CG	CG	CG	CG	CC	CG	GG	--	GG	CC	CC	CC	CG
GG	GT	GT	GT	GT	--	TT	GT	GG	GG	GG	TT	--	TT	GT
CC	CT	CT	CT	CT	CT	TT	CT	CC	CC	CC	CT	TT	TT	CT
TT	TT	CT	CT	TT	CT	CT	CT	TT	TT	TT	CT	CT	CT	TT
AG	GG	AG	AG	GG	AG	GG	AG	AG	AG	GG	AG	GG	GG	GG
CC	CG	GG	CG	CG	CC	GG	CG	CC	CC	CG	CG	GG	GG	CG
AA	AT	AT	AA	AT	AA	AT	AA	AA	AT	AT	AA	AT	AT	AT
AA	AA	AG	AG	AA	AA	AG	AA	AA	AA	AA	AA	AG	AG	AA
CC	CC	AC	AC	CC	CC	AC	CC	CC	CC	CC	CC	AC	AC	CC

TT	TT	CT	CT	TT	TT	CT	TT	TT	TT	TT	TT	CT	CT	TT
TT	CT	CT	TT	TT	TT	CT	TT	TT	CT	CT	TT	CT	CT	CT
GG	AG	AT	GT	GG	GG	AT	GG	GG	AG	AG	GG	AT	AT	AG
AA	AA	AT	AT	AA	AA	AT	AA	AA	AA	AA	AA	AT	AT	AA
GG	GG	AA	AG	GG	GG	AA	GG	GG	AG	AG	GG	AA	AA	AG
CC	CC	AA	AA	CC	AC	AA	CC	--	CC	AC	CC	AA	AA	CC
AA	AA	AG	AG	AA	AA	AG	AA	AA	AA	AA	AA	AG	AG	AA
GG	GG	AA	AA	AG	--	AA	GG	GG	GG	AG	GG	AA	AA	GG
AG	AA	GG	GG	AG	AG	GG	AA	AA	AA	AG	AA	GG	GG	AA
AG	GG	AA	AA	AG	AG	AA	GG	--	GG	AG	GG	AA	AA	GG
GT	GG	TT	TT	GT	GT	TT	GG	GG	GG	GT	GG	TT	TT	GG
AG	GG	AG	AG	AG	AG	AG	GG	--	GG	AG	GG	AG	AG	GG
CT	CC	TT	TT	CT	CC	TT	CC	--	CC	CT	CC	TT	TT	CC
CG	CC	GG	GG	CG	CG	GG	CG	CG	CC	CG	CC	GG	GG	CC
TT	TT	CT	CT	TT	TT	CT	TT	TT	TT	TT	TT	CT	CT	TT
CT	CT	CT	CT	CT	CT	CT	CT	CT	TT	CT	TT	CT	CT	CT
CC	CC	CC	CT	CT	CC	CC	CT	CC	CC	CT	CC	CC	CC	CC
GG	GG	GT	GT	GT	GT	GT	GG	GG	GG	GT	GT	GG	GT	GG
AG	GG	AG	AA	AA	AG	AA	AG	AG	GG	AA	AG	GG	AG	GG
AG	AA	AG	GG	GG	AG	GG	AG	--	AA	GG	AG	AA	AG	AA
AG	AA	AA	AG	AG	AA	AA	AG	AA	--	--	AA	AA	AA	AA
GT	GG	GT	TT	TT	GT	GT	GT	GG	GG	TT	GG	GG	GT	GG
AG	AA	AA	AG	AG	AA	AA	AG	AA	AA	AG	AA	AA	AA	--
CT	CC	CC	CT	CT	--	CC	CT	CC	CC	CT	CC	CC	CC	CC
AA	AG	AG	AA	AA	AA	AG	AA	AG	AG	AA	AG	AG	AG	AG
AG	GG	AG	AA	AA	AA	AG	AG	GG	GG	AA	GG	GG	AG	AG
AA	AG	AG	AA	AA	AA	AG	AG	GG	GG	AA	GG	GG	AG	AG
CC	CG	CG	CC	CC	CC	CG	CG	GG	GG	CC	GG	CG	GG	CG
CC	CG	CG	CC	CC	--	CG	CG	CG	GG	CC	GG	CG	GG	CG
AA	AG	AG	AA	AA	AA	AG	AG	AG	AG	AA	AG	AG	GG	AG
AG	AG	AG	AG	AG	AG	--	GG	AG	GG	AG	AG	GG	GG	AG
CC	CT	TT	CC	CC	CC	CT	CT	CT	CT	CC	CT	CT	TT	CT
AC	AC	CC	AC	CC	AC	AC	CC	AC	CC	AC	AC	CC	CC	AC
CG	CG	CC	CG	CC	CG	CG	CC	CG	CC	CC	CG	CC	CC	CG
AC	AC	CC	AC	CC	AC	AC	CC	AC	CC	AC	AC	CC	AC	AC
CC	AC	AA	CC	AC	CC	AC	AC	AC	AC	CC	AC	AC	CC	AA
AG	AA	AA	AG	AG	AG	AA	AG	AA	AG	AG	AA	AG	AA	AA
AG	AG	AA	GG	AA	GG	AG	GG	AG	AG	GG	AG	AG	GG	AA
AG	GG	GG	AG	AG	AG	GG	AG	GG	AG	AG	GG	AG	AG	GG
CT	CT	CC	TT	CC	TT	CT	TT	CT	TT	TT	CT	TT	TT	CC
CT	CC	CC	TT	--	TT	CC	TT	CT	TT	CT	CT	TT	TT	CC
AA	AA	AA	AG	AA	AG	AA	AG	AA	AG	AA	AG	AG	AG	AA
CC	CC	CC	--	CT	CT	CC	CT	CT	CT	CC	CT	CT	CT	CC
AG	GG	GG	AA	GG	AA	AG	GG	AG	GG	AA	AA	AA	AG	GG
CC	CC	CC	AC	CC	AC	AC	CC	--	CC	AC	AC	AC	CC	CC
CT	CC	CC	CT	CC	CT	CC	CC	CT	CC	CT	CT	CT	CT	CC
AG	AA	AA	AG	AA	GG	AG	AA	AG	--	GG	AG	GG	AG	AG

AG	AA	AA	AG	AA	AG	AG	AA	AG	AA	AG	AG	AG	AG	AA
AG	AA	AA	AG	AA	GG	GG	AA	AG	AG	GG	AG	GG	AG	AG
AC	CC	CC	AC	CC	AA	AA	CC	AC	AC	AA	AC	AA	AC	AC
AG	GG	GG	AG	GG	AA	AA	GG	AG	AG	AA	AG	AA	AA	AG
GT	TT	TT	GT	TT	GG	GG	TT	GT	GT	GG	GT	GG	GG	GT
AA	AA	AG	AA	AA	AG	AG	AA	AA	AA	AG	AA	AG	AG	AG
CT	TT	CC	CT	TT	CC	CC	TT	CT	TT	CC	CT	CC	CC	CT
CC	CC	CT	CC	CC	CT	CT	CC	CC	CC	CT	CC	CT	CT	CC
AG	GG	AA	AG	GG	AA	AA	GG	AG	GG	AA	AG	AA	AA	GG
CG	GG	CG	CG	GG	CG	CG	GG	CG	GG	CG	CG	CG	CG	GG
CT	TT	CT	CT	CT	--	CT	TT	CT	TT	CT	CT	CT	CT	TT
GG	AA	GG	AG	GG	AG	GG	AA	AG	AA	GG	AG	GG	GG	AA
GG	AA	GG	AG	GG	AG	GG	AA	AG	AA	GG	AG	GG	GG	AA
GG	CC	GG	CG	GG	CG	GG	CC	CG	CC	GG	GG	GG	GG	CC
GG	AA	GG	AG	GG	AA	--	AA	AG	AA	GG	GG	GG	GG	AA
AA	GG	GG	AG	GG	AG	AG	AG	--	AG	GG	AG	AG	AG	AA
CC	TT	TT	CT	TT	CT	CT	CT	CC	CT	TT	CT	CT	CT	CC
AA	GG	GG	AG	GG	AG	GG	AG	--	AG	GG	AG	AG	AG	AA
GG	CC	CC	CG	CC	CG	CC	CG	GG	CG	CC	CG	CG	CG	GG
AA	CC	CC	AC	CC	AC	CC	AC	AA	AC	CC	AC	AC	AC	AA
GG	AA	AA	AG	AA	AG	AA	AG	GG	AG	AA	AG	AG	AG	GG
TT	GG	GG	GT	GG	GT	GG	GT	TT	GT	GG	GT	GT	GT	TT
AA	GG	GG	AG	GG	AG	GG	AG	AA	AG	GG	AG	AG	AG	AA
AA	GG	GG	AG	GG	AG	GG	AG	AA	AG	GG	AG	AG	AG	AA
GG	AA	AA	AG	AA	AG	AA	AG	GG	AG	AA	AG	AG	AG	GG
CC	AA	AA	AC	AA	AC	AA	AC	CC	AC	AA	AC	AC	AC	CC
GG	AA	AA	AG	AA	AG	AA	AG	GG	AG	AA	AG	AG	AG	GG
GG	AA	AA	AG	AA	AG	AA	AG	GG	AG	AA	AG	AG	AG	GG
CC	TT	TT	CT	TT	CT	TT	CT	CC	CT	TT	CT	CT	CT	CC
CC	GG	GG	CG	GG	CG	GG	CG	CC	CG	GG	CG	CG	CG	CC
CC	AA	AA	AC	AA	AC	AA	AC	CC	AC	AA	AC	AC	AC	CC
CC	TT	TT	CT	TT	CT	TT	CT	CC	CT	TT	CT	CT	CT	CC
GG	TT	TT	GT	TT	GT	TT	GT	GG	GT	TT	GT	GT	GT	GG
TT	CC	CC	CT	CC	CT	CC	CT	TT	CT	CC	CT	CT	CT	TT
GG	CC	CC	CG	CC	CG	CC	CG	GG	CG	CC	CG	CG	CG	GG
AA	TT	TT	AT	TT	AT	TT	AT	AA	AT	TT	AT	AT	AT	AA
TT	CC	CC	CT	CC	CT	CC	CT	TT	CT	CC	CT	CT	CT	TT
AA	GG	GG	AG	GG	AG	GG	AG	AA	AG	GG	AG	AG	AG	AA
GG	AA	AA	AG	AA	AG	AA	AG	GG	AG	AA	AG	AG	AG	GG
TT	AA	AA	AT	AA	AT	AA	AT	TT	AT	AA	AT	AT	AT	TT
AA	GG	GG	AG	GG	AG	GG	AG	AA	AG	GG	AG	AG	AG	AA
GG	AA	AA	AG	AA	AG	AA	AG	--	AG	AA	AG	AG	AG	GG
GG	CC	CC	CG	CC	CG	CC	CG	--	CG	CC	CG	CG	CG	CG
GG	AA	AA	AG	AA	AG	AA	AG	GG	AG	AA	AG	AG	AG	AG
CC	GG	GG	CG	GG	CG	GG	CG	--	CG	GG	CG	CG	CG	CG
AA	GG	GG	AG	GG	AG	GG	AG	AA	AG	GG	AG	AG	AG	AG
CC	AA	AA	AC	AA	AC	AA	AC	CC	AC	AA	AC	AC	AC	AC

AA	GG	GG	AG	GG	AG	GG	AG	AA	AG	GG	AG	AG	AG	AG
GG	CC	CC	CG	CC	CG	CC	CG	GG	CG	CC	CG	CG	CG	CG
TT	CC	CC	CT	CC	CT	CC	CT	TT	CT	CC	CT	CT	CT	CT
AA	TT	TT	AT	TT	AT	TT	AT	--	AT	TT	AT	AT	AT	AT
--	CC	CC	CT	CC	CT	CC	CT	TT	CT	CC	CT	CT	CT	CT
TT	AA	AA	AT	AA	AT	AA	AT	TT	AT	AA	AT	AT	AT	AT
AA	TT	TT	AT	TT	AT	TT	AT	AA	AT	TT	AT	AT	AT	AT
CC	GG	GG	CG	GG	CG	GG	CG	CC	CG	GG	CG	CG	CG	CG
TT	CC	CC	CT	CC	CT	CC	CT	TT	CT	CC	CT	CT	CT	CT
GG	AA	AA	AG	AA	AG	AA	AG	GG	AG	AA	AG	AG	AG	AG
CC	AA	AA	AC	AA	AC	AA	AC	CC	AC	AA	AC	AC	AC	AC
GG	TT	TT	GT	TT	GT	TT	GT	GG	GT	TT	GT	GT	GT	GT
TT	CC	CC	CT	CC	CT	CC	CT	TT	CT	CC	CT	CT	CC	CT
CC	GG	GG	CG	GG	CG	GG	CG	CC	CG	GG	CG	CG	GG	CG
GG	TT	TT	GT	TT	GT	TT	GT	GG	GT	TT	GT	GT	TT	GT
CC	TT	TT	CT	TT	CT	TT	CT	CC	CT	TT	CT	CT	TT	CT
GG	AA	AA	AG	AA	AG	AA	AG	GG	AG	AA	AG	AG	AA	AG
CC	AA	AA	AC	AA	AC	AA	AC	CC	AC	AA	AC	AC	AA	AC
TT	CC	CC	CT	CC	CT	CC	CT	TT	CT	CC	CT	CT	CC	CT
CC	AA	AA	AC	AA	AC	AA	AC	CC	AC	AA	AC	AC	AA	AC
GG	AA	AA	AG	AA	AG	AA	AG	GG	AG	AA	AG	AG	AA	AG
TT	GG	GG	GT	GG	GT	GG	GT	TT	GT	GG	GT	GT	GG	GT
TT	GG	GG	GT	GT	GT	GG	GT	TT	GT	GG	GT	GT	GG	GT
AA	GG	GG	AG	GG	AG	GG	AG	AA	AG	GG	AG	AG	GG	AG
TT	CC	CC	CT	CC	CT	CC	CT	TT	CT	CC	CT	CT	CC	CT
TT	CC	CC	CT	CC	CT	CC	CT	TT	CT	CC	CT	CT	CC	CT
GG	AA	AA	AG	AA	AG	AA	AG	GG	AG	AA	AG	AG	AA	AG
TT	CC	CC	CT	CC	CT	CC	CC	TT	CT	CC	CT	CT	CC	CT
CC	TT	TT	CT	TT	CT	TT	CT	CC	CT	TT	CT	CT	TT	CT
AA	GG	GG	AG	GG	AG	GG	AG	AA	AG	GG	AG	AG	GG	AG
TT	CC	CC	CT	CC	CT	CC	CT	TT	CT	CC	CT	CT	CC	CT
CT	TT	TT	TT	CT	CT	TT	TT	CT	TT	TT	TT	CT	TT	TT
TT	CC	CC	CT	CT	CC	CC	CC	TT	CT	CC	CC	CT	CT	CT
GG	AA	AA	AG	AG	AA	AA	AA	GG	AG	AA	AA	AG	AG	AG
CC	AA	AA	AC	AC	AA	AA	AA	CC	CC	AA	AA	AC	AC	AC
CC	CG	CG	CC	CG	CG	CG	CG	CC	CC	CG	CC	CG	CC	CC
GT	GG	GG	GT	GT	--	GG	GG	TT	TT	GG	GT	GT	GT	GT
AG	GG	GG	GG	AG	GG	AG	AG	AA	AA	GG	AG	AG	AG	AG
CT	TT	TT	TT	CT	--	CT	CT	CC	CC	TT	CT	CT	CT	CT
GG	AG	AG	AG	AG	AG	GG	GG	GG	GG	AG	GG	AG	GG	GG
AG	GG	AG	GG	AG	GG	AG	AG	AA	AA	GG	AG	AG	AG	AG
CG	GG	CG	GG	CG	GG	CG	CG	CC	CC	GG	CG	CG	CG	CG
TT	CT	CT	CT	CT	CT	TT	TT	--	TT	CT	TT	CT	TT	TT
GT	TT	GT	TT	TT	TT	GT	GT	GG	GT	TT	GT	GT	GT	GT
AG	AG	AG	AG	GG	AG	GG	GG	GG	GG	AG	GG	GG	GG	GG
AG	GG	AG	GG	AG	AG	AG	AG	GG	AG	AG	AG	AG	AG	GG
CC	AC	CC	AC	AA	CC	AC	AC	AA	AC	AC	AC	AC	AC	AA

AA	AT	AA	AT	TT	AA	AT	AT	TT	AT	AT	AT	TT	AT	TT
AA	AA	AA	AT	TT	AA	AT	AT	TT	AT	AT	AT	TT	AT	TT
TT	TT	TT	AT	AA	TT	AT	AT	AA	AT	AT	AT	AA	AT	AT
CC	CC	CT	CT	TT	CC	CT	CT	CT	CT	CT	TT	TT	CT	CT
AA	AA	AG	GG	GG	GG	AG	AG	AG	AG	AG	GG	GG	AG	AG
CC	AC	AC	AA	AC	AA	AC	AC	AC	AC	AC	AA	AA	AC	CC
CC	CT	CT	TT	CT	TT	CT	TT	CT	CT	CT	TT	TT	TT	CC
AA	AG	GG	GG	AG	GG	AG	GG	--	AG	AG	AG	GG	GG	AA
TT	GT	GG	GG	GT	GG	GT	GG	TT	GT	GT	GT	GG	GG	TT
AG	AG	GG	GG	AA	GG	AG	GG	AA	--	AG	AG	GG	AG	AA
CG	CG	CC	CC	CG	CC	CC	CC	CG	CC	CC	CG	CC	CG	CG
AG	AG	GG	GG	AG	GG	GG	GG	AG	GG	GG	AG	GG	AG	AG
CG	CG	CC	CC	GG	CC	CG	CC	GG	CG	CG	GG	CC	CG	CG
AG	AG	AA	AA	GG	AA	AA	AA	GG	AG	--	GG	AA	AG	AG
AG	AG	GG	GG	AA	GG	GG	GG	AA	AG	GG	AA	GG	AG	AG
AG	AG	AA	AA	GG	AA	AA	AA	AG	AA	AA	GG	AA	AG	AG
GT	GT	GG	GG	GT	GG	GG	GG	GT	GG	GG	GT	GG	GT	GT
AG	AG	GG	GG	AA	GG	GG	AG	AG	GG	GG	AA	GG	AG	AG
GT	GT	TT	TT	GT	TT	TT	TT	GT	TT	TT	GT	TT	GT	GT
CG	CG	GG	GG	CC	GG	GG	CG	CG	GG	GG	CC	GG	CC	CG
TT	CT	CT	CT	TT	TT	TT	TT	TT	TT	CT	CT	CT	CT	CT
GG	CC	CG	--	CG	CG	--	CG	GG	CG	CC	CG	CC	CG	CG
CC	CG	CC	CG	CG	CG	CG	CG	CC	CG	CC	CC	CG	CC	CC
AA	AC	AC	AC	AA	AA	AC	AA	AC	AA	AC	AC	AC	AC	AC
CC	CT	CT	CT	CC	CC	CT	CC	CT	CC	CT	CT	CT	CT	CT
AG	AG	AG	AG	AA	AA	AG	AA	AG	AA	AG	AG	AG	AG	AG
CT	CT	CT	CT	CC	CC	CT	CC	--	CC	CT	CT	CT	CT	CT
GG	CG	CG	CG	CG	GG	CG	CG	GG	CG	GG	GG	CG	GG	GG
CT	CT	CT	CT	CC	CT	CT	CC	CT	CC	CT	CC	CT	CT	CT
AG	GG	GG	GG	AG	AG	AG	AG	AG	AG	AG	AA	GG	GG	GG
GT	GT	GT	GT	GG	GT	GG	GG	GT	GG	GT	GG	GT	GG	GT
GT	GG	GG	GG	GT	GT	GT	GT	GT	GT	GT	TT	GG	GT	GG
CG	CG	CG	CG	GG	CG	GG	GG	CG	GG	CG	GG	CG	GG	CG
CG	CC	CC	CC	CG	CG	CG	CG	CG	CG	CG	GG	CC	CG	CC
AG	AG	AG	AG	GG	AG	GG	GG	AG	GG	AG	GG	AG	GG	AG
CT	CC	CC	CC	CT	CT	CT	CT	CT	CT	CT	TT	CC	CT	CC
GT	TT	TT	TT	GT	GT	GT	GT	GT	GT	GT	GG	TT	GT	TT
AG	AG	AG	AG	GG	AG	GG	GG	AG	GG	AG	GG	AG	GG	AG
AG	GG	GG	GG	AG	AG	AG	AG	AG	AG	AG	AA	GG	AG	GG
CT	CC	CC	CC	CT	CT	CT	TT	CT	CT	CT	TT	CC	CT	CC
AT	AT	AT	AT	TT	AT	TT	TT	AT	TT	AT	--	AT	TT	AT
GT	GT	GT	GT	TT	GT	TT	TT	GT	TT	GT	TT	--	TT	GT
CT	TT	TT	TT	CT	CT	CT	CT	CT	CT	CT	CC	TT	CT	TT
AG	AG	AG	AG	AA	AG	AA	AA	AG	AA	AG	AA	AG	AA	AG
AT	AA	AA	AA	AT	AT	AT	AT	AT	AT	AT	TT	AA	AT	AA
AG	AG	AG	AG	AA	AG	AA	AA	--	--	AG	AA	AG	AA	AG
CT	CT	CT	CT	TT	CT	TT	TT	CT	TT	CT	TT	CT	TT	CT

AT	AT	AT	AT	TT	AT	TT	TT	AT	--	AT	TT	AT	TT	AT
GT	TT	TT	TT	GT	GT	GT	GT	--	GT	GT	GG	--	GT	TT
AG	GG	GG	GG	GG	AG	AG	AG	AG	AG	AG	AA	GG	AG	GG
CT	TT	TT	CT	TT	CT	CT	CT	CT	CT	CT	CC	--	CT	TT
CT	TT	TT	TT	TT	CT	CT	CT	CT	CT	CT	CC	TT	CT	CT
CT	TT	TT	TT	TT	CT	TT	CT	CT	CT	CT	CC	TT	CT	CT
GT	TT	TT	TT	TT	GT	TT	GT	GT	TT	GT	GG	TT	GT	GT
CG	GG	GG	GG	GG	CG	GG	CG	--	GG	CC	CG	GG	CG	CG
CC	AA	AA	AA	AA	AC	AA	AC	AC	AA	CC	AC	AA	AC	AC
AA	GG	GG	GG	GG	AG	GG	AG	AG	GG	AA	AG	GG	AG	AG
GG	AA	AA	AA	AA	AG	AA	AA	AG	AA	GG	AG	AA	AG	AG
TT	CT	CT	CT	CT	TT	CT	CT	CT	CT	CC	TT	CT	TT	CT
AA	AC	AC	AC	AC	AA	AC	AC	AC	AC	CC	AA	AC	AA	AC
TT	TT	TT	TT	GT	TT	GT	GT	GT	GT	GT	TT	GT	TT	GT
GG	CG	CG	CG	CG	GG	GG	GG	GG	GG	GG	GG	GG	GG	GG
GG	AG	AG	AG	AA	GG	AG	AG	AG	GG	AG	GG	AG	GG	GG
TT	CT	CT	CT	CT	TT	TT	CT	TT	TT	TT	TT	TT	TT	TT
GT	TT	GT	TT	GT	TT	GT	TT	TT	TT	GT	GT	TT	GT	TT
CC	CT	CT	CT	CT	CC	CC	CT	CC	CC	CC	CC	CC	CC	CC
AC	AA	AC	AA	AC	AA	AC	AA	AA	AA	AC	AC	AA	AC	AA
AG	AG	GG	AG	GG	AA	AG	AG	AA	AA	AG	AG	AA	AG	AA
AG	AA	AG	AA	AG	AA	AG	AA	AA	AA	AG	AG	AA	AG	AA
GT	TT	GT	GT	GG	TT	GT	GT	TT	TT	GT	GG	GT	GT	TT
--	CC	CT	CT	TT	CC	CT	CT	--	CC	CT	TT	CT	--	CC
AC	AA	AC	AC	CC	AA	AC	AC	AA	AA	AA	CC	AC	AC	AA
AT	TT	AT	AT	AA	TT	AT	AT	--	TT	TT	AA	AT	AT	TT
GG	GT	GT	GT	GG	GT	GG	GT	--	GT	GT	GG	--	GG	GT
AG	AG	AG	AA	AA	AG	AG	AA	AG	AG	AG	AA	AA	AG	AG
CG	CC	CC	CG	GG	--	CG	CG	CG	CC	CC	GG	CG	CG	CC
AG	AG	AA	AG	GG	AA	AG	AG	AG	AA	AA	GG	AG	AG	AA
CC	CC	CG	CG	CG	GG	CG	CG	CG	GG	CC	CG	GG	CC	CC
AA	AA	AG	AG	AG	GG	AG	AG	AG	GG	AA	AG	GG	AA	AA
AA	AA	AA	AG	AG	AA	AA	AG	AA	AG	--	AG	AG	AA	--
AA	AA	AT	AA	AA	AT	AT	AT	AT	AT	AA	AA	AT	AA	AA
AA	AA	AA	AG	AG	AA	--	AG	AG	AG	AG	AG	AG	AA	AA
AG	AG	AG	AA	AA	AG	AG	AA	AA	AA	AA	AA	AA	AG	AA
AA	AA	AA	AC	CC	AC	AC	CC	CC	CC	AC	AC	CC	AC	AC
CC	CC	CC	CC	AC	AC	AC	AC	AC	AC	CC	CC	AC	AC	CC
CC	--	CC	AC	AA	AC	AC	AA	AA	AA	AC	AC	AA	AC	AC
GG	GG	GG	GT	TT	GT	GT	TT	TT	TT	--	GT	TT	GT	TT
AA	AT	AA	AT	TT	AT	AT	TT	--	TT	AT	AT	TT	AT	TT
AA	AG	AA	AA	AG	AG	AG	AG	AG	AG	AA	AA	AA	AG	AG
CC	AC	CC	CC	AC	AC	AC	AC	AC	AC	CC	CC	AC	AC	AC
AA	AT	AA	AT	TT	AT	AT	AT	TT	TT	AT	AT	AT	AT	TT
CC	CT	CC	CT	TT	CT	CT	CT	TT	TT	CT	CT	CT	CT	TT
GG	AG	GG	AG	AA	AG	AG	AG	AG	AA	AG	AG	AG	AG	AA
CC	CT	CC	CT	TT	CT	CT	CT	CT	TT	CT	CT	CT	CT	TT

TT	CT	TT	CT	CC	--	CT	CT	CT	CC	CT	CT	CT	CT	CC
GG	GG	GG	AG	AA	AG	AG	AG	AG	AA	--	AG	AG	AG	AA
CC	CC	CC	CT	TT	CT	CT	CT	CT	TT	CT	CT	CT	CT	TT
TT	TT	TT	--	CC	--	TT	CT	CT	--	CT	CT	CT	CT	--
CC	CC	CC	CT	TT	CT	CC	CT	CT	TT	CT	CT	CT	CT	TT
GG	GG	GG	AG	AA	AG	GG	AG	AG	AA	AG	AG	AG	AG	AA
TT	TT	TT	CT	CC	CT	TT	CT	CT	CC	--	CT	CT	CT	CC
GG	GG	GG	AG	AA	AG	GG	AG	--	--	AG	AG	AG	AG	AA
CC	CC	CC	CG	GG	CG	CC	CG	CG	GG	CG	CG	CG	CG	GG
AA	AA	AA	AC	CC	AC	AA	AC	AC	--	AC	AC	AC	AC	CC
CC	CC	CC	CT	TT	CT	CC	CT	CT	TT	CT	CT	CT	CT	CT
CC	CC	CC	CT	TT	CT	CC	CT	CT	TT	CT	CT	CT	CT	CT
TT	TT	TT	AT	AA	AT	TT	AT	AT	AA	AT	AT	AT	AT	AT
CC	CC	CC	CC	TT	--	CC	CT	CT	--	CT	CT	--	CT	CT
AA	AA	AA	AC	CC	AC	AA	AC	AC	CC	AC	AC	AC	AC	AC
TT	TT	TT	GT	GG	GT	TT	GT	GT	GG	GT	GT	GT	GT	GT
TT	TT	TT	CT	CC	CT	TT	CT	CT	CC	CT	CT	CT	CT	CT
GG	GG	GG	CG	CC	CG	GG	CG	CG	CC	CG	CG	CG	CG	CG
GG	GG	GG	CG	CC	CG	GG	CG	CG	CC	CG	CG	CG	CG	CG
CC	CC	CC	AC	AA	AC	CC	AC	AC	AA	AC	AC	AC	AC	AC
GG	GG	GG	CG	CC	CG	GG	GG	CG	CC	CG	CG	CG	CG	CG
AT	TT	AA	AT	TT	AA	AT	AT	TT	--	AA	AA	AT	TT	TT
AG	GG	AA	AG	GG	AA	AG	AG	--	AA	AA	AG	AG	GG	GG
GG	GG	AA	AG	GG	--	AG	AG	--	AA	AA	AG	AG	GG	GG
AA	AA	GG	AG	AA	GG	GG	AA	AG	GG	GG	AG	AG	AA	AA
GG	GG	AG	AG	GG	AA	AA	GG	AG	AA	AA	AG	AG	GG	AG
CC	CC	AC	CC	CC	AA	AA	CC	AC	AA	AA	AC	AC	CC	AC
GG	--	AG	GG	GG	AA	AA	GG	AG	AA	AA	AG	AG	GG	AG
GG	GG	AG	GG	GG	AA	AA	GG	AG	AA	AA	AG	AG	GG	AG
TT	TT	GT	TT	TT	GG	GG	TT	GT	GG	GG	GT	GT	TT	GT
GG	GG	GT	GG	GG	TT	TT	GG	GT	TT	TT	GT	GT	GG	--
AA	AA	AC	AA	AA	CC	CC	AA	AC	CC	CC	AC	AC	AA	AC
CC	CC	CT	CC	CC	TT	TT	CC	CT	--	TT	CT	CT	CC	CT
CC	CC	CT	CC	CC	TT	TT	CC	CT	TT	TT	CT	CT	CC	CT
TT	TT	GT	TT	TT	GG	GG	TT	GT	GG	GG	GT	GT	TT	GT
TT	TT	AT	TT	TT	AA	AA	TT	AT	AA	AA	AT	AT	TT	AT
CC	CC	CG	CC	CC	GG	GG	CC	CG	GG	GG	CG	CG	CC	CG
CC	CC	AC	CC	CC	--	AA	CC	AC	AA	AA	AC	AC	CC	AC
TT	TT	CT	TT	TT	CC	CC	TT	CT	CC	CC	CT	CT	TT	CT
CC	CC	CG	CC	CC	GG	GG	CC	CG	GG	GG	CG	CG	CC	CG
TT	TT	CT	TT	TT	CC	CC	TT	CT	CC	CC	CT	CT	TT	CT
TT	TT	AT	TT	TT	AA	AA	TT	AT	AA	AA	AT	AT	TT	AT
CC	CC	CT	CC	CC	TT	TT	CC	CT	TT	TT	CT	CT	CC	CT
AA	AA	AG	AA	AA	GG	GG	AA	AG	GG	GG	AG	AG	AA	AG
TT	TT	AT	TT	TT	AA	AA	TT	AT	AA	--	AT	AT	TT	AT
TT	TT	AT	TT	TT	AA	AA	TT	AT	AA	AA	AT	AT	TT	AT
CC	CC	AC	CC	CC	AA	AA	CC	AC	AA	AA	AC	AC	CC	AC

GG	GG	GT	GG	GG	TT	TT	GG	GT	TT	TT	GT	GT	GG	GT
AA	AA	AG	AA	AA	GG	GG	AA	AG	GG	GG	AG	AG	AA	AG
TT	TT	GT	TT	TT	GG	GG	TT	GT	GG	GG	GT	GT	TT	GT
GG	GG	AG	GG	GG	AA	AA	GG	AG	AA	AA	AG	AG	GG	AG
CC	CC	CT	CC	CC	--	TT	CC	--	TT	TT	CT	CT	CC	CT
GG	GG	AG	GG	GG	AA	AA	GG	AG	AA	AA	AG	AG	GG	AG
CC	CC	CT	CC	CC	TT	TT	CC	CT	TT	TT	CT	CT	CC	CT
AA	AA	AC	AA	AA	CC	CC	AA	AC	CC	CC	AC	AC	AA	AC
GG	GG	AG	GG	AG	AA	AA	--	--	--	AA	AG	AG	GG	AG
CC	CT	CT	CC	CT	TT	TT	CC	CT	TT	TT	CT	CT	CC	CT
AA	AG	AG	AG	AG	AG	GG	AA	AG	GG	GG	AG	AA	AA	AG
TT	CT	CT	CT	CT	CT	CC	CT	CT	CC	CC	CT	CT	CT	CT
GT	GT	GG	GT	GT	GG	GG	GG	GT	GG	GG	GT	GT	GT	GT
AG	GG	AG	GG	GG	AG	GG	GG	GG	GG	AG	GG	GG	GG	AG
AA	AT	AT	AT	AT	AT	TT	TT	AT	TT	AA	AT	AT	AT	AA
AA	AT	AT	AT	AT	--	TT	TT	AT	TT	AA	AT	AT	AT	AA
AA	AC	AC	AC	AC	AA	CC	CC	AC	CC	AA	AC	AC	AC	AA
TT	GT	GT	GT	GT	TT	GG	GG	GT	GG	TT	GT	GT	GT	TT
TT	CT	TT	CT	TT	TT	TT	TT	TT	CT	TT	TT	CT	CT	TT
GG	AG	GG	AA	AG	GG	GG	AG	AG	AA	GG	AG	AG	AG	GG
CG	CG	CG	GG	GG	CG	CG	GG	GG	GG	CG	CG	CG	CG	CG
CT	CT	CT	TT	TT	CT	CT	CT	TT	TT	CT	CT	CT	CT	CT
GT	GG	GT	GG	GT	GT	GT	GT	GT	GG	GT	GT	GG	GG	GT
GG	AG	GG	AA	AG	AG	GG	GG	AG	AA	GG	GG	AG	AG	GG
AC	AA	AC	AA	AA	AA	AC	AC	AA	AA	AC	AC	AC	AC	AC
CG	CG	CG	GG	GG	GG	CG	CG	GG	GG	CG	CG	CG	CG	CG
GT	TT	GT	TT	GT	TT	GT	GT	TT	TT	GT	GT	GT	GT	GT
CT	CC	CT	CC	TT	CT	TT	TT	CC	CC	TT	TT	CT	TT	CT
GG	AG	AA	AG	AG	GG	AG	AG	AG	AG	AG	AG	AG	AG	AG
CC	CT	CC	CC	CT	CC	CT	CT	CC	CC	--	CT	--	CT	CC
CG	CC	CG	CG	CC	CG	CC	CC	--	CG	CC	CC	CG	CC	CG
AC	AC	AC	AC	AC	--	AA	AA	AC	--	AA	AA	AA	AA	AC
GG	AG	GG	GG	AG	AA	AA	AA	GG	GG	AA	AA	AG	AA	GG
TT	TT	TT	TT	TT	CT	CT	CT	TT	TT	CT	CT	CT	CT	TT
GG	CG	GG	GG	CG	CG	CG	CG	GG	GG	CG	CG	GG	CG	GG
AA	AG	AA	AA	AG	GG	GG	GG	AA	AA	GG	GG	AG	GG	AA
CG	GG	CG	CG	GG	CG	GG	GG	CG	CG	GG	GG	CG	GG	CG
CC	CG	CC	CC	CG	CG	GG	GG	CC	CC	GG	GG	--	GG	CC
CT	CT	CC	--	CT	CT	TT	TT	CC	CC	TT	TT	CT	TT	CC
AG	AG	GG	GG	AG	AG	AA	AA	GG	GG	AA	AA	AG	AA	GG
AG	AG	AG	AG	AG	GG	GG	GG	AG	AG	GG	GG	GG	GG	AG
CC	CT	CC	CC	CT	TT	TT	TT	CC	CC	TT	CT	CT	TT	CC
GG	GG	GG	GG	GG	AG	AG	AG	GG	GG	AG	AG	AG	AG	GG
GG	AG	GG	GG	AG	AA	AA	AA	GG	GG	AA	AG	AG	AA	GG
CC	AC	CC	CC	AC	AA	AA	AA	CC	CC	AA	AC	AC	AA	CC
TT	AT	TT	TT	AT	AT	AT	AT	--	TT	AT	TT	TT	AT	TT
AA	AG	AA	AA	AG	--	GG	GG	--	AA	GG	AG	--	GG	AA

AA	AC	AA	AA	AC	CC	CC	CC	AA	AA	CC	AC	AC	CC	AA
AA	AG	AA	AA	AG	GG	GG	GG	AA	AA	GG	AG	AG	GG	AA
AA	AA	AA	AA	AA	AA	AG	AG	AA	AA	AG	AG	AG	AG	AA
CC	CT	CC	CC	CT	CT	CT	CT	--	CC	CT	CC	CC	CC	CC
CC	CG	CC	CC	CG	CG	CG	CG	CC	CC	CG	CC	CC	CG	CC
CT	CT	TT	TT	CT	CT	TT	CT	TT	TT	CT	TT	TT	CT	TT
GG	GG	CG	CG	CG	GG	CG	GG	CG	CG	GG	--	CG	GG	CG
CT	CT	CT	CC	CC	CT	CC	CT	CC	CC	CC	CC	CC	CT	CC
GG	GG	GG	GT	GT	GG	GT	GG	GT	GT	GT	GT	GT	GG	GT

7-14	7-15	7-16	7-20	7-21	7-23	7-26	7-30	7-31	7-32	7-33	7-35	7-39
AA	CC	CC	AA	CC	AA	CC	AC	AC	AA	CC	CC	AC
AA	GG	GG	AA	GG	AA	GG	AG	AG	AA	GG	GG	AG
TT	CC	CC	TT	CC	CT	CC	CT	CT	TT	CC	CT	CT
GG	AG	AG	GG	AG	AG	AG	GG	AG	GG	AG	GG	GG
AA	GG	GG	AA	GG	AG	GG	AG	AG	AA	GG	AG	AG
--	GG	--	CG	GG	CG	GG	GG	CG	CG	GG	CG	GG
TT	CC	CC	TT	CC	CT	CC	CT	CT	TT	CC	TT	CT
TT	GG	GG	TT	GG	GT	GT	GT	GT	GT	GG	TT	GT
TT	GG	GG	TT	GT	GT	GT	GT	GT	GT	GG	TT	GT
TT	GG	GG	TT	GT	GT	GT	GG	GT	GT	GG	TT	GT
AA	--	GG	AA	AG	GG	AA	GG	AG	AG	GG	AG	AG
CC	CC	CC	CT	CT	CC	CT	CC	CT	CC	CC	CC	CT
AC	CC	CC	AA	AC	CC	AA	CC	AC	AC	CC	AC	AA
AA	AA	AA	AG	AG	AA	AG	AA	AG	AA	AA	AA	AG
CC	CC	CC	CT	CC	CC	CT	CC	CT	CT	CC	CT	CT
AA	AA	AG	GG	AG	AA	GG	AA	AG	AG	AA	AG	GG
TT	TT	TT	AT	AT	TT	AT	TT	TT	AT	TT	TT	AT
CC	CC	CT	CT	CC	CC	CT	CC	CT	CT	CT	CT	CC
GT	TT	TT	GT	GT	TT	GT	TT	TT	GT	TT	GT	GT
CG	CC	CC	CC	CG	CC	CG	CC	CC	CG	CC	CG	CG
AT	TT	AT	--	AT	TT	--	AT	AT	AA	AT	AA	AT
CT	TT	CT	CT	CT	TT	CC	CT	CT	CC	CT	CC	CT
AT	AA	AT	AT	AT	AA	TT	AT	AT	TT	--	TT	AT
AT	AA	AT	AT	AT	AA	TT	AT	AT	TT	AT	TT	AT
AG	AA	AG	AG	AG	AA	GG	AG	AG	GG	AG	GG	AG
GT	TT	TT	TT	GT	TT	GT	GT	TT	GT	TT	GT	GT
GT	TT	GT	GT	GT	TT	GG	GT	GT	GG	GT	GG	GT
AT	TT	AT	AT	AT	TT	AA	AT	AT	AA	AT	AA	AT
CT	TT	CT	CT	CT	TT	CC	CT	CT	CC	CT	CC	CT
CG	CC	CG	CG	CG	CC	GG	CG	CG	GG	CG	GG	CG
CT	CC	CT	CT	CT	CC	TT	CT	CT	TT	CT	TT	CT
AC	AA	AC	AC	AC	AA	CC	AC	AC	CC	AC	CC	AC
AG	AA	AG	GG	AG	AA	GG	AG	AG	GG	AG	GG	AG
GT	GG	GT	GT	GT	GG	TT	GT	GT	TT	GT	TT	GT
AG	AA	AG	AG	AG	AA	GG	AG	AG	--	AG	GG	AG
GT	TT	GT	GT	GT	TT	GG	GT	GT	GG	GT	GG	GT
AG	--	AG	--	AG	GG	AA	AG	AG	AA	AG	AA	AG
CT	CC	CT	CT	CT	CC	TT	CT	CT	TT	CT	TT	CT
AT	AA	AT	AT	AT	AA	TT	AT	AT	TT	AT	TT	AT
AG	AA	AG	AG	AG	AA	GG	AG	AG	GG	AG	GG	AG
AC	AA	AC	AC	AC	AA	CC	AC	AC	CC	AC	CC	AC
AG	--	AG	AG	AG	AA	GG	AG	AG	GG	AG	GG	AG

CT	--	CT	CT	CT	TT	CC	--	CT	CC	CT	CC	CT
CG	CC	CG	CG	CG	CC	GG	CG	CG	GG	CG	GG	CG
CT	TT	CT	--	CT	TT	CC	CT	CT	CC	CT	CC	CT
CT	CC	CT	--	CT	CC	TT	CT	TT	TT	CT	TT	CC
AC	AA	AC	AA	AC	AA	CC	AC	CC	CC	AC	CC	AA
CT	CT	TT	TT	CT	CT	TT	CT	TT	TT	CT	TT	CC
CT	CT	CC	CC	CT	CT	CC	CT	CC	CT	CT	CC	TT
GG	AG	GG	AG	GG	AG	AG	AG	AG	AG	GG	AA	GG
GG	GT	GG	GG	GG	GT	GT	GT	GT	GG	GG	TT	GG
GG	AG	GG	GG	AG	AG	GG	AG	GG	GG	GG	AA	GG
TT	CT	TT	TT	CT	CT	TT	CT	TT	TT	TT	CC	TT
CC	CG	CC	CC	CG	CC	CC	CG	CC	CC	CC	CG	CC
AA	AG	AA	AA	AG	AA	AA	AG	AA	AA	AA	AG	AA
CC	AC	CC	CC	CC	CC	CC	AC	CC	CC	CC	AC	CC
AG	GG	AG	GG	AG	AG	AG	AG	AG	AG	AG	GG	AG
TT	CC	TT	TT	TT	TT	TT	CT	TT	TT	TT	CT	TT
CC	TT	CC	CC	CT	CC	CC	CT	CC	CC	CC	CT	CC
TT	CC	TT	TT	CT	TT	TT	CT	TT	TT	TT	CT	TT
AG	GG	AA	AA	AG	AA	AA	AG	AA	AA	AA	AA	AA
AC	CC	AA	AA	AC	AA	AA	AC	AA	AA	AA	AA	AA
AG	GG	AA	AA	GG	AA	AA	AG	AA	AA	AA	AA	AA
CC	CC	CG	GG	CC	GG	GG	CG	GG	GG	GG	GG	GG
GG	GG	CG	CC	GG	CC	CC	CC	CC	CC	CC	CC	CC
GG	GG	AG	AA	GG	AG	AA	AA	AA	AA	AA	AA	AA
AA	AA	AC	CC	AA	AC	CC	CC	CC	CC	CC	CC	AC
CC	CC	CT	TT	CC	CT	TT	TT	TT	TT	CC	TT	CT
TT	TT	CT	CC	TT	CT	CC	CC	CC	CC	TT	CC	CT
AA	AA	AT	TT	AA	AT	TT	TT	AT	TT	AA	TT	AT
AG	AG	AG	--	AG	GG	GG	GG	AG	GG	AG	GG	AG
TT	AT	TT	--	AT	AT	TT	TT	TT	TT	AT	TT	AT
TT	GT	GT	--	TT	GT	TT	GG	GT	GT	GT	GT	GG
GG	AG	GG	--	GG	AG	GG	AG	GG	GG	--	AG	AG
TT	CT	TT	TT	TT	CT	TT	CT	TT	TT	CT	CT	CT
GG	AG	GG	GG	AG	AG	GG	AG	GG	GG	AG	AG	AG
GT	GT	GT	GG	GT	GT	GG	TT	GT	GT	GT	GT	TT
GT	GT	GG	GG	GT	GT	GG	GT	GG	GG	GT	GT	GT
CC	CT	CT	TT	CT	CT	TT	CC	CT	CT	CT	CT	CC
CC	CT	CT	TT	CT	CT	TT	CC	CT	CT	CT	CT	CC
GG	AG	AG	AA	GG	AG	AA	GG	GG	AG	AG	AG	GG
CC	CG	CG	GG	CC	CG	CC	CC	CC	CC	CG	CG	CC
GG	AG	AG	AA	GG	AG	GG	GG	GG	GG	AG	AG	GG
TT	CT	CT	CC	TT	CT	TT	TT	TT	TT	CT	CT	CT
TT	CT	CT	CC	TT	CT	TT	TT	TT	TT	CT	CT	CT
CG	CG	CC	CC	CG	CG	CG	CC	CG	CG	CG	CG	CC
AT	AT	TT	AT	AT	AT	AT	TT	AT	AT	AT	AT	TT
GT	GG	GT	GG	GT	GG	GT	GT	GG	GT	GG	GG	GT
CT	CC	CT	CC	CT	CC	CT	CT	CC	CT	CC	CC	CT

TT	CT	CC	CT	--	CT	TT	CT	CT	--	CC	CT	TT
GT	GG	GG	GG	GT	GG	GT	GT	GG	GG	GG	GG	GT
TT	CT	CC	CT	--	CT	TT	CT	CC	CC	CC	CT	TT
GT	TT	TT	TT	GT	TT	GT	GT	TT	TT	TT	TT	GT
CG	GG	GG	--	CG	GG	CG	CG	GG	GG	GG	GG	CG
AG	GG	AG	AG	GG	AG	AG	AA	AA	--	GG	AG	AA
AG	AA	AG	AG	AA	AG	AG	GG	GG	GG	AA	AG	GG
AA	AA	AG	AG	AA	AA	AG	GG	GG	GG	AA	AG	GG
TT	TT	CT	CC	TT	TT	CT	CC	CC	CC	TT	CT	CC
GG	--	AG	AG	GG	GG	AG	AA	AA	AA	GG	AG	AA
GG	GT	GT	GT	GG	GG	GT	TT	TT	TT	GT	GT	TT
AG	GG	AG	AG	AG	AG	AG	GG	GG	GG	GG	AG	GG
TT	CT	CT	CT	TT	TT	CT	CC	CC	CC	CT	TT	CC
CC	CC	AC	AC	CC	CC	AC	AC	AC	AC	CC	CC	AC
CT	CT	CC	CC	CT	CT	CC	CC	CC	CC	CT	CT	CC
GG	GT	GT	GT	GG	GG	GT	TT	TT	TT	GG	GG	TT
CC	CT	CC	CC	CC	CC	CC	CT	CT	CT	CC	CC	CT
GG	GG	CG	--	GG	GG	CG	CG	CG	CG	GG	GG	CG
--	CC	AC	AC	CC	CC	AC	AC	AC	AC	CC	CC	AC
TT	CT	TT	TT	TT	TT	TT	CT	CT	CT	TT	TT	CT
AA	AG	AA	AA	AA	AA	AA	AG	AG	AG	AA	AA	AG
AA	AG	AG	AG	AA	AA	AG	GG	GG	GG	AA	AA	GG
CT	CT	CC	CC	CT	CT	CC	CC	CC	CC	CT	CT	CC
AA	AA	AT	AT	AA	AA	AT	AT	AT	AT	AA	AA	AT
CC	CC	CT	CT	--	CC	CT	CT	CT	CT	CC	CC	CT
AA	AA	AG	--	AA	AA	AG	AG	AG	AG	AA	AA	AG
CC	CT	CC	CC	CC	CC	CC	CT	CT	CT	CC	CC	CT
TT	AT	AT	--	TT	TT	AT	AA	--	AA	TT	TT	AA
TT	AT	AT	AT	TT	TT	AT	AA	AA	AA	TT	TT	AA
TT	CT	CT	CT	TT	TT	CT	CC	CC	CC	TT	TT	CC
CC	CC	CT	CT	CC	CC	CT	CT	CT	CT	CC	CC	CT
GG	GT	GG	GG	GG	GG	GG	GT	GT	GT	GG	GG	GT
CC	CT	CC	CC	CC	CC	CC	CT	CT	CT	CC	CC	CT
AG	AA	AG	AG	AG	AG	AG	AA	AA	AA	AG	AG	AA
GG	AG	AG	AG	GG	GG	AG	AA	AA	AA	GG	GG	AA
AA	AG	AA	AA	AA	AA	AA	AG	AG	AG	AA	AA	AG
CC	CT	CT	CT	CC	CC	CT	TT	TT	TT	CC	CC	TT
CC	AC	CC	CC	CC	CC	CC	AC	AC	AC	CC	CC	AC
TT	AT	AT	AT	TT	TT	AT	AA	AA	AA	TT	TT	AA
AA	AA	AG	AG	AA	AA	AG	AG	AG	AG	AA	AA	AG
AA	AC	AA	AA	AA	AA	AA	AC	AC	AC	AA	AA	AC
TT	AT	TT	TT	TT	TT	TT	AT	AT	AT	TT	TT	AT
AC	AA	CC	--	AC	AC	CC	AC	AC	AC	AC	AC	AC
CC	CC	AC	AC	CC	CC	AC	AC	AC	AC	CC	CC	AC
AG	GG	AG	--	AG	AG	AG	GG	GG	GG	AG	AG	GG
AC	AC	CC	CC	AC	AC	CC	CC	CC	CC	AC	AC	CC
AA	AG	AA	AA	AA	AA	AA	AG	AG	AG	AA	AA	AG

CC	CC	CG	CG	CC	CC	CG	CG	CG	CG	CC	CC	CG
TT	TT	CT	CT	TT	TT	CT	CC	CC	CC	TT	TT	CC
CC	CT	CT	CT	CC	CC	CT	TT	TT	TT	CC	CC	TT
CC	CC	--	CC	CC	CC	CC	CT	CT	CT	CC	CC	CT
AA	AA	AA	AA	AA	AA	AA	AG	AG	AG	AA	AA	AG
CC	CC	CT	CT	CC	CC	CT	TT	TT	TT	CC	CC	TT
TT	TT	TT	TT	TT	TT	TT	CT	CT	CT	TT	TT	CT
AA	AA	AT	AT	AA	AA	AT	TT	TT	TT	AA	AA	TT
GG	GG	AG	AG	GG	GG	AG	AG	AG	AG	GG	GG	AG
AA	AA	AA	AA	AA	AA	AA	AC	AC	AC	AA	AA	AC
CC	CC	CC	CC	CC	CC	CC	CT	CT	CT	CC	CC	CT
TT	TT	GT	GT	TT	TT	GT	GT	GT	GT	TT	TT	GT
TT	TT	TT	TT	TT	TT	TT	CT	CT	CT	TT	TT	CT
AC	AC	AC	AC	AC	AC	AC	CC	CC	CC	AC	AC	CC
AA	AA	AA	--	AA	AA	AA	AG	AG	AG	AA	AA	AG
GG	GG	GG	GG	GG	GG	GG	GT	GT	GG	GG	GG	GT
AT	AT	AT	AT	AT	AT	AT	TT	TT	AT	AT	AT	TT
AA	AA	AA	AA	AA	AA	AA	AG	AG	AA	AA	AA	AG
CC	CC	CT	CT	CC	CC	CT	CT	CT	CT	CC	CC	CT
CC	CC	CC	CC	CC	CC	CC	CT	CT	CC	CC	CC	CT
AA	AA	AA	AA	AA	AA	AA	AC	AC	AA	AA	AA	AC
CC	CC	CC	CC	CC	CC	CC	CT	CT	CC	CC	CC	CT
AA	AA	AT	--	AA	AA	AT	TT	TT	AT	AA	AA	TT
GG	GG	AG	AG	GG	GG	AG	AA	AA	AG	GG	GG	AA
GG	GG	CG	CG	GG	GG	CG	CG	CG	CG	GG	GG	CG
TT	TT	TT	TT	TT	TT	TT	CT	CT	TT	TT	TT	CT
GG	GG	GG	GG	AG	GG	GG	AG	AG	GG	GG	GG	AG
AA	AA	AA	AA	AG	AA	AA	AG	AG	AA	AA	AA	AG
AA	AA	AG	AG	AA	AA	AG	AG	AG	AG	AA	AA	AG
GG	GG	AG	AG	GG	GG	AG	AG	AG	AG	GG	GG	AG
TT	--	TT	TT	CT	TT	CT	CT	TT	TT	TT	CT	TT
GG	GG	GT	GT	GT	GG	TT	TT	GT	GT	GG	GT	GT
TT	TT	GT	GT	TT	TT	GT	GT	GT	GT	TT	TT	GT
GG	GG	AG	AG	AG	GG	AA	AA	AG	AG	GG	AG	AG
CC	CC	CC	CC	CT	CT	CT	CT	CC	CC	CC	CT	CC
TT	TT	TT	TT	GT	GT	GT	GT	TT	TT	TT	GT	TT
AG	AG	GG	GG	AG	AG	GG	GG	GG	GG	AG	AG	GG
AG	AG	AG	AG	AG	AA	AG	AG	AG	AG	AA	AG	AG
CT	CC	CC	CC	CT	--	CT	--	CT	CT	CT	CT	CT
AT	AA	AT	AT	AA	AA	AA	AT	AT	AT	AA	AT	AT
CG	CG	CG	CG	CC	CC	CC	GG	GG	CG	CC	CG	CG
AA	AC	AA	AA	AA	AA	AA	AC	AC	AA	AA	AA	AA
GG	AG	AG	--	AA	AA	AA	AG	GG	AG	AA	AG	AG
GG	AG	GG	GG	AG	AG	AG	AG	GG	GG	AG	GG	AG
CC	CT	CT	CC	CT	CT	CT	CT	CC	CC	CT	CC	CT
AA	AG	GG	AG	GG	GG	AG	AG	AA	AG	GG	AA	GG
CT	CC	CT	CC	CC	CC	CT	CT	CT	CC	CC	CT	CC

AG	GG	GG	AG	GG	GG	GG	GG	AG	AG	GG	AG	GG
AG	AG	AG	AA	AG	AG	GG	AG	AA	AA	AG	AG	AG
TT	CT	CC	CT	CC	CC	CT	CC	CT	CT	CC	TT	CC
TT	AT	AA	AT	AA	AT	AT	AA	AT	AT	AA	TT	AT
AC	AC	AA	CC	AA	AC	AC	AA	AC	AA	AA	CC	AC
TT	CT	--	CC	TT	CT	TT	TT	CT	TT	TT	CC	CT
--	AG	--	AA	GG	AG	GG	GG	AG	GG	AG	AA	AG
CC	CT	CC	TT	CC	CT	CC	CC	CT	CC	CT	TT	CT
TT	CT	TT	CT	TT	TT	TT	TT	TT	--	CT	CT	CT
AA	AG	AA	GG	AA	AG	AA	AA	AG	AA	AG	GG	AG
AA	GG	AG	AG	AA	AG	AG	AG	AA	GG	AG	AG	AG
CC	TT	CT	CC	CC	CT	CC	CT	CC	TT	CT	CT	CT
GG	AG	AG	AG	GG	GG	AG	GG	GG	AG	AG	GG	AG
CC	TT	CT	CT	--	CT	CT	CT	CC	TT	TT	CT	CT
GG	CG	GG	GG	GG	CG	GG	CG	GG	CG	CG	CG	GG
CT	TT	CT	CT	CC	CT	CT	CT	CC	TT	TT	CT	CT
CT	CT	CT	CT	CC	CC	CT	CC	CC	CC	CT	CT	CT
AG	AA	AG	AA	GG	AG	AG	AG	GG	AA	AA	AA	AG
CT	TT	CT	--	CC	CT	CT	CT	CC	TT	TT	TT	CT
AG	GG	AG	GG	AA	AG	AG	AG	AA	GG	GG	GG	GG
CT	TT	CT	CT	CC	CT	CT	CT	CC	TT	TT	TT	TT
AA	AA	AG	AG	GG	AG	AG	AG	GG	AA	AA	AA	AA
AG	AG	AG	--	GG	GG	AG	GG	GG	AG	AG	AG	AG
GG	GG	GT	GT	GT	GT	GT	GG	TT	GG	GG	GG	GG
CG	CG	CG	CC	CC	CC	CG	CG	CC	CG	CC	CG	CG
CC	CC	CG	CC	CC	CC	CG	CC	CG	CC	CC	CC	CC
TT	TT	CT	CT	CT	CT	CT	TT	CC	TT	CT	TT	TT
CC	CC	CT	CT	CT	CT	CT	CC	TT	CC	CT	CC	CT
TT	TT	AT	AT	TT	AT	AT	TT	AA	TT	AT	AT	AT
AA	AA	AG	AG	AA	AG	AG	AA	GG	AA	AG	AG	AG
TT	TT	CT	CT	TT	CT	CT	TT	CC	TT	CT	CT	CT
CT	CT	CT	TT	CT	TT	CT	CT	TT	TT	TT	TT	TT
CC	CC	AC	CC	CC	AC	AC	CC	AA	CC	AC	AC	AC
GT	TT	GT	GT	--	GG	GG	TT	GT	GT	GT	GT	GT
AG	AA	AG	AG	AA	GG	GG	AA	AG	AG	AG	AG	AG
CT	TT	CT	CT	TT	CC	CC	TT	CT	CT	CT	CT	CT
AT	AA	AT	AT	AA	TT	TT	AA	AT	AT	AT	AT	AT
AC	CC	AC	AC	CC	AA	AA	CC	AC	AC	AC	AC	AC
CC	CG	CC	CG	CG	CC	CC	CG	CG	CG	CG	CC	CG
AA	AG	AA	AG	AG	AA	AA	AG	AG	AG	AG	AA	AG
CT	CC	CT	--	CC	TT	CT	CC	CT	CT	CT	CT	CT
CT	CC	CT	CT	CC	TT	TT	CC	CT	CT	CT	CT	CT
AG	AA	AG	AG	AA	GG	AG	AA	AG	AG	AG	AG	AG
CT	CT	CT	CC	CT	CC	CC	CT	CC	CC	CC	CT	CC
AG	AG	AG	AA	AA	GG	AG	AA	AG	AG	AG	AG	AG
AG	AG	AG	AG	GG	AA	AG	GG	AG	AG	AG	AG	AG
GT	GT	GT	GT	GG	GT	GT	GG	GT	GT	GT	GT	GT

CT	CT	CT	CT	TT	CT	CT	TT	CT	CC	CT	CT	CT
CG	CG	CG	CG	GG	CG	CG	GG	CG	CG	CG	CG	CG
GT	GT	GT	GT	TT	GT	GT	TT	GT	GT	GT	GT	GT
AG	AG	AG	AG	AA	AG	AG	AA	AG	AG	AG	AG	AG
CT	CT	CT	CT	CC	CT	CT	CC	CT	CT	CT	CT	CT
TT	GT	GT	--	GT	TT	TT	GT	TT	TT	TT	GT	TT
CG	CG	CG	CG	GG	CG	CG	GG	CG	CG	CG	CG	CG
AG	AG	AG	AG	AA	AG	AG	AA	AG	AG	AG	AG	AG
GG	AG	AG	GG	AG	GG	GG	AG	GG	GG	GG	AG	GG
CC	AC	AC	AC	AA	AC	AC	AA	AC	AC	AC	AC	AC
CC	CG	CG	CC	CG	CC	CC	CG	CC	CC	CC	CG	CC
AA	AG	AG	AG	GG	AG	AG	GG	AG	AG	AG	AG	AG
CC	AC	AC	AC	AA	AC	AC	--	AC	AC	AC	AC	AC
GG	GT	GT	GT	TT	GT	GT	TT	GT	GT	GT	GT	GT
AA	AC	AC	AC	CC	CC	AC	CC	AC	AC	AC	AC	AC
CC	CT	CT	CC	CT	CT	CC	CT	CC	CC	CC	CT	CC
GG	GT	GT	GT	TT	TT	GT	TT	GT	GT	GT	GT	GT
GG	GG	GG	AG	AG	AG	AG	AG	AG	AG	AG	GG	AG
TT	CT	CT	CT	CC	CC	CT	CC	CT	CT	CT	CT	CT
CC	CT	CT	CC	CT	CT	CC	CT	CC	CT	CC	CT	CC
TT	AT	AT	AT	AA	AA	AT	AA	AT	AA	AT	AT	AT
GG	CG	CG	CG	CC	CC	CG	CC	CG	CC	CG	CG	CG
TT	GT	GT	GT	GG	GG	GT	GG	GT	GG	GT	GT	GT
AG	GG	GG	AG	AG	AG	AG	AG	AG	AG	AG	GG	AG
GT	--	TT	GT	TT	TT	TT	TT	GT	--	GT	TT	GT
CT	CT	CT	CT	CT	CC	CC	CC	CC	CC	CT	CT	CT
AC	AC	AC	AC	AC	CC	CC	CC	CC	CC	AC	AC	AC
CC	CT	CT	CC	CT	CC	CC	CC	CC	CC	CC	CT	CC
CG	CG	CG	CG	CG	GG	GG	CG	GG	GG	CG	CG	CG
CT	CT	CT	CT	CT	CC	CC	CT	CC	CC	CT	CT	CT
GT	TT	TT	GT	TT	GT	GT	GT	GT	TT	TT	TT	GT
--	CT	CT	TT	CT	CT	CT	TT	CT	CT	TT	CT	TT
AT	TT	TT	AT	TT	AT	AT	AT	AT	TT	--	TT	AT
CC	AC	AC	CC	AC	CC	CC	CC	CC	AC	AC	AC	AC
CT	CT	CT	CT	TT	TT	CT	CT	CT	CT	CC	CT	CC
AA	AG	AG	AA	AG	AA	AA	AA	AA	AG	AG	AG	AG
GG	AG	AG	GG	AG	GG	GG	GG	GG	AG	AG	AG	AG
GG	GG	GG	GG	CG	GG	GG	CG	CG	GG	GG	CG	CG
CT	CT	CC	CC	CT	CT	CT	CT	CT	CC	CT	CT	TT
CT	CT	CC	CC	CT	TT	CT	TT	CT	CT	CT	CT	TT
CT	CT	CC	CC	TT	TT	CT	TT	CT	CT	CT	CT	TT
CT	CT	TT	TT	CC	CC	CT	CC	CT	CT	CT	CT	CC
GT	GT	GG	GG	TT	TT	GT	TT	GG	GT	GT	GT	TT
CT	CT	TT	CT	--	CC	CT	CC	CT	CT	CT	CT	CC
AT	AT	TT	AT	AA	AA	AT	AA	TT	AT	AA	AT	AA
AA	--	AG	AG	--	AA	AG	AA	GG	AG	AA	AG	AA
CC	CC	AC	AC	AC	CC	AC	CC	AA	AC	CC	CC	CC

AA	AA	AG	AG	AG	AA	AG	AA	GG	AG	AA	AA	AA
--	AA	AG	AA	AG	AA	AG	AA	GG	AG	AA	AA	AA
TT	TT	CT	CT	CT	TT	CT	TT	CC	CT	TT	TT	TT
GG	GG	AG	AG	AG	GG	AG	GG	AA	AG	GG	GG	GG
GG	GG	GG	GT	GT	GG	GT	GG	TT	GT	GG	GG	GG
TT	TT	CT	CT	CT	TT	CT	TT	CC	CT	TT	TT	TT
--	GG	AG	AG	AG	GG	AG	GG	AA	--	GG	GG	GG
GG	GG	AG	AG	AG	GG	AG	GG	AA	AG	GG	GG	GG
AA	AA	AG	--	AG	AA	AG	AA	GG	AG	AA	AA	AA
GG	GG	CG	CG	CG	GG	CG	GG	CC	CG	GG	GG	GG
AA	AA	AG	AG	AA	AA	AG	AA	GG	AG	AA	AA	AA
AA	AA	AC	--	AC	AA	AC	AA	CC	AC	AA	AA	AA
GG	GG	AG	AG	GG	GG	AG	GG	AA	AG	GG	GG	GG
GG	GG	AG	AG	GG	GG	AG	GG	AA	AG	GG	GG	GG
AA	AA	AT	AT	AA	AA	AT	AA	TT	AT	AA	AA	AA
TT	TT	CT	CT	TT	TT	CT	TT	CC	CT	TT	TT	TT
--	TT	CT	--	TT	TT	CT	TT	CC	CT	TT	TT	TT
CC	--	CG	CG	CC	CC	CG	CC	GG	CG	CC	CC	CC
CC	CC	CT	CT	CC	CC	CT	CC	TT	CT	CC	CC	CC
--	TT	CT	CT	TT	TT	CT	TT	CC	CT	TT	TT	TT
CC	CC	CT	CT	CC	CC	CT	CC	TT	CT	CC	CC	CC
AA	AA	AT	AT	AA	AA	AT	AA	TT	AT	AA	AA	AA
TT	TT	AT	AT	TT	TT	AT	AT	AA	AT	TT	TT	TT
CC	CC	CT	CT	CC	CC	CT	CT	TT	CT	CC	CC	CC
TT	TT	CT	CT	TT	TT	CT	CT	CC	CT	TT	TT	TT
TT	TT	AA	AT	TT	TT	TT	AT	AA	AT	TT	TT	TT
--	AA	GG	AG	AA	AA	AA	AG	GG	AG	AA	AA	AA
TT	--	GG	GT	TT	TT	--	GT	GG	GT	TT	TT	TT
CC	CC	TT	CT	CC	CC	CC	CT	TT	CT	CC	CC	CC
GG	GG	CC	CC	GG	GG	GG	CG	CC	CG	GG	GG	GG
AA	AA	GG	AG	AA	AA	AA	AG	GG	AG	AG	AA	AA
CC	CC	TT	CT	CC	CC	--	CT	TT	CT	CT	CC	CC
CC	CC	TT	CT	CC	CC	CC	CT	TT	CT	CT	CC	CC
AA	AA	TT	AT	AA	AA	AA	AT	TT	AT	AT	AA	AA
GG	GG	AA	AG	GG	GG	GG	AG	AA	AG	AG	GG	GG
GG	GG	AA	--	GG	GG	GG	AG	AA	AG	AG	GG	GG
GG	GG	GT	GT	GG	GG	GG	GT	TT	GT	GT	GG	GT
--	GG	AG	AA	GG	GG	GG	AG	AA	AG	AG	GG	AG
AG	AG	GG	GG	AG	AG	AG	GG	GG	AG	GG	AG	AG
TT	TT	TT	TT	CT	TT	CT	TT	TT	TT	TT	CT	TT
GG	GG	AG	--	AG	AG	AG	AG	AG	AG	AG	AA	GG
GG	GG	AG	AG	GG	AG	GG	AG	AG	AG	AG	AG	GG
AG	GG	AG	AG	AG	GG	AG	AG	AG	AG	AA	AA	GG
CG	CC	CG	CC	CG	CC	CG	CC	CG	CG	GG	GG	CC
CT	TT	CT	TT	CT	TT	CT	TT	CT	CT	CC	CC	TT
CC	AC	AC	AC	CC	AC	CC	AC	AC	AC	CC	CC	AC
AA	AG	AA	AG	AG	AG	AA	AG	AA	AA	AA	AA	AA

CC	AC	AC	AC	CC	AC	CC	AC	AC	AC	CC	CC	AC
AC	CC	AC	CC	AC	CC	AC	CC	AC	AC	AC	CC	AC
TT	CT	CT	CT	TT	CT	TT	CT	CT	CT	TT	TT	CT
AA	GG	AG	GG	AA	GG	AA	GG	AG	AG	AA	AG	AG
GG	AA	AG	AA	GG	AA	GG	AA	AG	AA	GG	AG	AG
TT	CC	CT	CC	TT	CC	CT	CC	CT	CC	TT	CT	CT
CT	TT	CT	TT	CT	TT	TT	CT	CT	TT	CT	TT	CT
CC	GG	CG	CG	CC	CG	CG	CG	CG	GG	CC	CG	CG
AA	AG	AG	AG	AA	AG	AG	AG	AG	GG	AA	AG	AG
TT	CT	CT	CT	TT	TT	CT	CT	CT	CC	TT	CT	CT
AT	AT	AT	AA	AT	AT	AA	AT	AT	AA	AT	AA	AT
CC	AC	AA	AC	CC	CC	AC	AC	AC	AA	CC	AA	AC
GG	AG	AG	GG	GG	GG	GG	AG	AG	AG	GG	AG	AG
CC	CT	CT	CC	CC	CC	CC	CT	CT	CT	CC	CT	CT
CC	CT	CT	CC	CC	CC	CC	CT	CT	CT	CC	CT	CT
GG	GT	GT	GG	GG	GG	GG	GT	GT	GT	GG	GT	GT
CC	CC	CT	CC	TT	TT	CT	CT	CC	CT	CT	CT	CC
AG	AG	AG	AG	GG	GG	GG	AG	AG	AG	GG	GG	AG
AA	AA	AG	AA	GG	GG	AG	AG	AA	AG	AG	AG	AA
GG	GG	CG	GG	CG	--	GG	CG	CG	CG	GG	GG	CG
AA	AA	AG	AA	GG	GG	AG	AG	AG	AG	AG	AG	AG
GG	GG	AG	GG	AA	AA	AG	AG	AG	AG	AG	AG	AG
GT	GT	TT	GT	TT	TT	GT	TT	TT	TT	TT	GT	TT
GG	GG	AG	GG	AA	AA	AG	AG	GG	AG	AA	AG	AG
AG	GG	AG	GG	AA	AA	AG	AG	AG	AG	AG	AG	AG
GG	GT	GG	GT	GG	GG	GT	GT	GG	GG	GT	GT	GG
CT	TT	TT	TT	CC	CC	CC	TT	CT	CT	CT	CT	CT
CT	CC	CC	CC	TT	CT	TT	CC	CT	CT	CT	CT	CT
CT	CT	CT	CT	CC	CC	CC	CT	CT	CT	CC	CC	CT
AG	AG	GG	GG	AA	AG	AA	GG	AG	AG	AG	AG	AG
CT	CT	TT	TT	CC	CT	CC	TT	CT	CT	CT	CT	CT
AG	AG	GG	GG	AA	GG	AA	AG	AG	AG	AG	AG	AG
CT	CT	TT	--	CC	TT	--	CT	CC	CT	CT	CT	CT
CG	CG	CC	CC	CG	CC	GG	CG	CG	CG	CG	CG	CG
CT	CC	TT	--	CT	TT	CT	CT	CT	CT	TT	CT	CT
AC	AA	CC	CC	AC	CC	AC	AC	AC	AC	AC	AA	AC
AG	GG	AA	--	AG	AA	AG	AG	AG	AG	AG	GG	AA
CC	TT	CC	CC	CT	CT	CT	CC	CT	CT	CT	TT	CC
GG	AA	GG	--	AG	AG	AG	GG	AG	AA	AG	AA	GG
GG	AA	GG	--	AG	AG	AG	GG	AG	AA	AG	AA	GG
CT	TT	CT	CT	TT	CT	TT	CT	TT	TT	CT	TT	CT
--	GG	AA	AG	GG	AG	AG	AA	AG	GG	AG	GG	AA
AG	AG	AG	AA	AA	AA	AG	AG	AG	AA	AA	AA	AG
GT	GT	GT	TT	TT	TT	GT	GT	GT	TT	TT	TT	GT
GT	GT	GT	TT	TT	TT	GT	GT	GT	TT	TT	TT	GT
CC	AC	CC	AC	AA	AC	AC	CC	AC	AA	AC	AA	CC
GG	AG	GG	AG	AA	AG	AG	GG	AG	AA	AG	AA	GG

GG	CG	GG	--	CC	CG	CG	GG	CG	CC	--	CC	GG
CG	CG	GG	CG	CC	CG	CG	GG	CG	CC	CG	CC	GG
CT	--	CC	CT	TT	CT	CT	CC	CT	TT	CT	TT	CC
CT	CT	TT	CT	CC	CT	CT	CT	CT	CC	TT	CC	TT
AT	AT	TT	AT	AA	AT	AT	AT	AA	AA	TT	AA	TT
AG	GG	GG	AG	AG	AG	GG	AG	AG	AG	GG	AG	AG
CT	CT	TT	CT	CT	CT	--	CT	CC	CT	TT	CC	CT
CT	CT	CT	TT	CT	CT	TT	CT	TT	CT	CT	TT	CT
CT	CT	CT	--	CT	TT	CC	CT	CC	CT	CT	CT	CT
AG	AG	AG	GG	AG	AA	AG	AG	GG	AA	AG	AG	AG
CG	CC	CC	CC	CC	CG	CG	CG	CC	CG	CC	CC	CG
GG	CG	CG	GG	CG	CG	GG	GG	GG	CG	CG	CG	GG
GG	GG	GG	GG	GG	GT	GT	GT	GG	GT	GG	GG	GT
CC	CG	CG	CC	CG	CG	CG	CG	CC	CG	CC	CG	CC
AG	AA	AG	AG	AA	AA	AG	AA	AG	AA	GG	AG	AG
AA	AC	AC	AA	AC	AC	AC	AC	AA	AC	AA	AC	AA
AA	AT	AT	AA	AT	AT	AT	AT	AA	AT	AA	AT	AA
AG	AA	AG	AG	AA	AA	AG	AA	AG	AA	GG	AG	AG
AT	TT	AT	--	AT	TT	AT	TT	AT	TT	AT	AT	AA
AG	AA	AG	AG	AG	AA	GG	AG	AG	AA	AA	AG	GG
AC	CC	CC	AC	CC	AC	CC	AC	AC	AC	AC	CC	CC
CT	TT	CT	CT	CT	TT	CC	CT	CT	TT	TT	CT	CC
GT	GG	GG	GT	GT	GG	TT	GT	GT	GG	GG	GT	TT
GT	GG	GG	GT	GT	GG	TT	GT	TT	GG	GG	GT	TT
CT	TT	TT	CT	CT	TT	CC	CT	CC	TT	TT	CT	CC
CG	GG	GG	CG	CG	GG	CC	CG	CC	GG	GG	CG	CC
AG	AA	AA	AG	AG	AA	GG	AG	GG	AG	AA	AG	GG
CT	TT	TT	CT	CT	TT	CC	CT	CC	TT	TT	CT	CC
GT	GG	GG	GT	GT	GG	TT	GT	TT	GG	GG	GT	TT
CT	CC	CC	CT	CT	CC	TT	CT	TT	CC	CC	CT	TT
CG	CG	CC	CG	CG	CC	GG	CG	GG	CC	CC	CG	GG
AG	AG	AA	AG	AG	AA	GG	AG	GG	AG	AA	AG	GG
AC	AC	CC	CC	AC	AC	AA	AC	AA	AC	CC	AC	AA
CT	CT	CC	--	CT	CT	TT	CT	TT	CT	CC	CT	TT
GG	AG	AG	GG	AG	AG	GG	GG	GG	GG	AG	AG	GG
AC	AC	CC	--	AC	AC	AA	--	AA	AC	CC	AC	AA
CT	CT	TT	CT	CT	CT	CC	CT	CC	CT	TT	CT	CC
AG	AG	AA	AG	AG	AG	GG	AG	GG	AG	AA	AG	GG
CG	CG	CC	CG	CG	CG	GG	CG	GG	CG	CC	CG	GG
--	GT	TT	GT	GT	GT	GG	GT	GG	GT	TT	GT	GG
CT	CT	TT	CT	CT	CT	CC	CT	CC	CT	TT	CT	CC
TT	CT	CT	TT	CT	CT	TT	TT	TT	TT	CT	CT	TT
GG	AG	GG	GG	AG	AG	AG	GG	AG	GG	GG	AG	AG
CG	CG	GG	CG	CG	CG	CC	CG	CC	CG	GG	CC	CC
AT	AA	AT	AT	AA	AA	AA	AT	AA	AA	AT	AA	AA
AA	AG	AG	AA	AG	AG	AA	AA	AA	AA	AG	AA	AA
CC	AC	AC	CC	AC	AC	CC	CC	CC	CC	AC	CC	CC

TT	CT	CT	TT	CT	CT	TT	TT	TT	TT	CT	TT	TT
CT	TT	TT	CT	CT	TT	TT	CT	TT	TT	CT	TT	TT
AG	--	GT	AG	AT	GT	GG	GG	GG	GG	AT	GG	GG
AA	AT	AT	AA	AT	AT	AA	AA	AA	AA	AT	AA	AA
AG	AG	AG	AG	AA	AG	GG	GG	AG	GG	AA	GG	AG
AC	AC	AC	CC	AA	AC	CC	CC	AC	CC	AA	CC	AC
AA	AG	AG	AA	AG	AG	AA	AA	AA	AA	AG	AA	AA
AG	AG	AG	--	AA	AG	GG	GG	AG	GG	AA	AG	AG
AG	AG	AG	AA	GG	AG	AA	AG	AG	AA	GG	AG	AG
AG	AA	AG	GG	AA	AG	GG	AG	AG	GG	AA	AG	AG
GT	TT	TT	GG	GT	GT	GG	GT	GT	GG	TT	GT	GT
AG	AG	AG	AG	GG	GG	GG	AG	AG	GG	AG	AG	AG
CT	TT	TT	CT	CT	CT	CC	CT	CT	CC	TT	CT	CC
CG	GG	GG	CG	CG	CG	CC	CG	CG	CC	GG	CG	CC
TT	CT	CT	TT	CT	CT	TT	TT	TT	TT	CT	TT	TT
CT	CT	CT	CT	TT	TT	TT	CT	CT	CT	CT	CT	TT
CT	CT	CC	CT	CC	CT	CC	CC	CT	CC	CC	CC	CT
GT	GG	GG	GG	GT	GG	GT	GG	GG	GG	GG	GG	GT
AA	AG	GG	AG	AG	AA	AG	GG	AG	GG	GG	AG	AA
GG	AG	AA	AG	AG	GG	AG	AA	AG	AA	AA	AG	GG
--	AG	AA	AG	AA	AG	AA	AA	AG	AA	AA	AG	AG
GT	GT	GG	GT	GG	GT	GT	GT	GT	GG	GG	GT	TT
AG	AG	AA	AG	AA	AG	AA	AA	AG	AA	AA	AG	AA
CT	CT	CC	CT	CC	CT	CC	CC	CT	CC	CC	CT	CC
AA	AA	AG	--	AG	AA	AG	AG	AA	AG	--	AA	AG
AG	AG	GG	AG	GG	AG	AG	AG	AG	GG	GG	AA	AG
AG	--	GG	AA	GG	AG	AG	AG	AG	GG	GG	AA	AG
CG	GG	GG	CC	GG	CG	CG	CG	CG	GG	GG	CC	CG
CG	GG	GG	CC	GG	GG	CG	CG	CG	GG	CG	CC	CG
AG	GG	GG	AA	GG	GG	AG	AG	AG	GG	AG	AA	AG
GG	GG	GG	GG	GG	GG	AG	AG	AG	GG	--	AG	AG
CT	TT	TT	CC	TT	TT	CT	CT	CC	TT	CT	CT	CT
CC	CC	CC	AC	CC	CC	AC	AC	AC	CC	AC	CC	AC
CG	CC	CC	CG	CC	CC	CG	CG	CG	CC	CG	CC	CG
AC	AC	CC	AC	CC	CC	AC	AC	AC	CC	AC	CC	AC
CC	AC	AA	CC	AA	AA	AC	AC	CC	AA	AC	AA	AC
AG	AA	AA	AG	AA	AA	AA	AA	AG	AA	AA	AG	AA
GG	AG	AA	GG	AA	AA	AA	AG	AG	AA	AG	AA	AG
AG	GG	GG	AG	GG	GG	GG	GG	AG	GG	GG	GG	GG
TT	CT	CC	--	CC	CC	CC	CT	CT	CC	CT	CC	CT
TT	CT	CT	TT	CC	CC	CC	CT	CT	CC	CT	CC	CT
AG	AG	AA	AG	--	AA	AA	AG	AA	AA	AG	AA	AG
CT	CT	CC	CC	CC	CC	CC	CT	CC	CC	CT	CC	CT
AA	AA	AA	--	GG	AG	AG	AA	--	AG	AG	AG	GG
AC	AC	AC	AC	CC	CC	CC	AC	AC	AC	CC	CC	CC
CT	CT	CT	CT	CC	CT	CT	CT	CC	CC	CT	CT	CC
GG	GG	GG	GG	AA	AG	AG	GG	AG	AG	AG	AG	AA

AG	AG	AG	AG	AA	AG	AG	AG	AA	AA	AG	AG	AA
--	AG	GG	GG	AA	AG	AG	AG	GG	--	GG	AG	AA
AC	AC	AA	AA	CC	AC	AA	AC	AA	AA	AA	AC	CC
AG	AG	AA	AA	GG	AG	AA	AG	AA	AA	AG	AG	GG
--	--	GG	GG	TT	GT	GG	GT	GG	GG	GT	GT	TT
AA	AA	AG	AG	AA	AA	AG	AA	AG	AG	AG	AA	AA
CT	TT	CC	CC	TT	CT	CC	TT	CC	CC	CT	CT	TT
CT	CC	CC	CT	CT	CC	CT	CC	CT	CT	CT	CT	CC
AA	GG	AG	AA	AG	AG	AA	GG	AA	AA	GG	AA	GG
CG	GG	CG	CG	GG	CG	CG	GG	CG	CG	GG	CG	GG
CT	TT	CT	CT	TT	CT	--	TT	CT	CT	TT	CT	TT
AG	AA	AG	GG	AG	GG	GG	AG	GG	AG	AA	GG	AA
AG	AA	AG	GG	AG	GG	GG	AG	GG	AG	AA	GG	AA
CG	CC	CG	GG	CG	GG	GG	CG	GG	CG	CC	GG	CC
AG	AG	AG	--	AG	GG	GG	AG	GG	AG	AA	GG	AA
AG	GG	GG	GG	GG	GG	AG	AG	AA	AA	GG	AG	AA
CT	TT	TT	TT	TT	TT	CT	CT	CC	CC	TT	CT	CC
AG	GG	GG	GG	GG	GG	AG	AG	AA	AA	GG	AG	AA
CG	CC	CC	CC	CC	CC	CG	CG	GG	GG	CC	CG	GG
AC	CC	CC	CC	CC	CC	AC	AC	AA	AA	CC	AC	AA
AG	AA	AA	AA	AA	AA	AG	AG	GG	GG	AA	AG	GG
GT	GG	GG	GG	GG	GG	GT	GT	TT	TT	GG	GT	TT
AG	GG	GG	GG	GG	GG	AG	AG	AA	AA	GG	AG	AA
AG	GG	GG	GG	GG	GG	AG	AG	AA	AA	GG	AG	AA
AG	--	AA	AA	AA	AA	AG	AG	GG	GG	AA	AG	GG
AC	AA	AA	AA	AA	AA	AC	AC	CC	CC	AA	AC	CC
AG	AA	AA	AA	AA	AA	AG	AG	GG	GG	AA	AG	GG
AG	AA	AA	AA	AA	AA	AG	AG	GG	GG	AA	AG	GG
CT	TT	TT	TT	TT	TT	CT	CT	CC	CC	TT	CT	CC
CG	GG	GG	GG	GG	GG	CG	CG	CC	CC	GG	CG	CC
AC	AA	AA	AA	AA	AA	AC	AC	CC	CC	AA	AC	CC
CT	TT	TT	TT	TT	TT	CT	CT	CC	CC	TT	CT	CC
GT	TT	TT	TT	TT	TT	GT	GT	GG	GG	TT	GT	GG
CT	CC	CC	CC	CC	CC	CT	CT	TT	TT	CC	CT	TT
CG	CC	CC	CC	CC	CC	CG	CG	GG	GG	CC	CG	GG
AT	TT	TT	TT	TT	TT	AT	AT	AA	AA	TT	AT	AA
CT	CC	CC	--	CC	CC	CT	CT	TT	TT	CC	CT	TT
AG	GG	GG	--	GG	GG	AG	AG	AA	AA	GG	AG	AA
AG	AA	AA	AA	AA	AA	AG	AG	GG	GG	AA	AG	GG
AT	AA	AA	--	AA	AA	AT	AT	TT	TT	AA	AT	TT
AG	GG	GG	GG	GG	GG	AG	AG	AA	AA	GG	AG	AA
AG	--	AA	AA	AA	AA	AG	AG	GG	GG	AA	AG	GG
CG	CC	CC	CC	CC	CC	CG	CG	GG	GG	CC	CG	GG
AG	AA	AA	AA	AA	AA	AG	AG	GG	GG	AA	AG	GG
CG	GG	GG	GG	GG	GG	CG	CG	CC	CC	GG	CG	CC
--	GG	GG	GG	GG	GG	AG	AG	AA	AA	GG	AG	AA
AC	AA	AA	AA	AA	AA	AC	AC	CC	CC	AA	AC	CC

--	GG	GG	GG	GG	GG	AG	AG	AA	AA	GG	AG	AA
CG	CC	CC	CC	CC	CC	CG	CG	GG	GG	CC	CG	GG
CT	CC	CC	CC	CC	CC	CT	CT	TT	TT	CC	CT	TT
AT	TT	TT	TT	TT	TT	AT	AT	AA	AA	TT	AT	AA
CT	CC	CC	CC	CC	CC	CT	CT	TT	TT	CC	CT	TT
AT	AA	AA	--	AA	AA	AT	AT	TT	TT	AA	AT	TT
AT	TT	TT	TT	TT	TT	AT	AT	AA	AA	TT	AT	AA
CG	GG	GG	GG	GG	GG	CG	CG	CC	CC	GG	CG	CC
CT	CC	CC	CC	CC	CC	CT	CT	TT	TT	CC	CT	TT
AG	AA	AA	AA	AA	AA	AG	AG	GG	GG	AA	AG	GG
AC	AA	AA	AA	AA	AA	AC	AC	CC	CC	AA	AC	CC
GT	TT	TT	GT	TT	TT	GT	GT	GG	GG	TT	GT	GG
CT	CC	CC	CT	CC	CC	CT	CT	TT	TT	CC	CT	TT
CG	GG	GG	CG	GG	GG	CG	CG	CC	CC	GG	CG	CC
GT	TT	TT	GT	TT	TT	GT	GT	GG	GG	TT	GT	GG
CT	TT	TT	CT	TT	TT	CT	CT	CC	CC	TT	CT	CC
AG	AA	AA	--	AA	AA	AG	AG	GG	GG	AA	AG	GG
AC	--	AA	AC	AA	AA	AC	AC	CC	CC	AA	AC	AC
CT	--	CC	CT	CC	CC	CT	CT	TT	TT	CC	CT	CT
AC	--	AA	AC	AA	AA	AC	AC	CC	CC	AA	AC	AC
AG	AA	AA	AG	AA	AA	AG	AG	GG	GG	AA	AG	AG
GT	GG	GG	GT	GG	GG	GT	GT	TT	TT	GG	GT	GT
GT	GG	GG	GT	--	GG	GT	GT	TT	TT	GG	GT	GT
AG	GG	GG	AG	GG	GG	AG	AG	AA	AA	GG	AG	AG
CT	CC	CC	CT	CC	CC	CT	CT	TT	TT	CC	CT	CT
CT	CC	CC	CT	CT	CC	CT	CT	TT	TT	CC	CT	CT
AG	AA	AA	AG	AG	AA	AG	AG	GG	GG	AA	AG	AG
CT	CC	CC	CT	CT	CC	CT	CT	TT	TT	CC	CT	CT
CT	TT	TT	CT	CT	TT	CT	CT	CC	CC	TT	CT	CT
AG	GG	GG	AG	AG	GG	AG	AG	AA	AA	GG	AG	AG
CT	CC	CC	CT	CT	CC	CT	CT	TT	TT	CC	CT	CT
CT	TT	TT	CT	CT	TT	CT	TT	CT	CT	TT	TT	TT
CT	CC	CC	CT	CT	CC	CT	CT	TT	CT	CC	CT	CT
AG	AG	AA	AG	AG	AA	AA	AG	GG	AG	AA	AG	AG
AC	AC	AC	AC	AC	AA	AA	AC	CC	AC	AA	CC	AC
CG	CG	CC	CG	CG	CG	CG	CC	CC	CG	CC	CC	CC
GT	GT	GT	GT	GT	GG	GG	GT	TT	GT	GT	GT	GT
AG	AG	AG	AA	AG	GG	GG	AG	AA	AG	AG	AG	AG
CT	CT	CT	CT	CT	TT	TT	CT	CC	CT	CT	CT	CT
AG	AG	GG	AG	AG	AG	AG	GG	GG	AG	GG	AG	GG
AG	AG	AG	AG	--	AG	GG	AG	AA	AG	AG	AG	AG
CG	CG	CG	CG	CG	CG	GG	CG	CC	CG	CG	CG	CG
CT	TT	TT	CT	CT	CT	CT	TT	CT	CT	TT	CT	TT
GT	GG	GT	--	GT	GT	TT	GT	GT	GT	GT	GT	GT
AG	GG	AG	GG	GG	AG	GG	AG	GG	AG	AG	GG	GG
AG	AG	AG	AG	AG	GG	AG	GG	GG	AG	AG	AG	AG
CC	AC	CC	--	--	AC	AC	AC	AA	CC	CC	AC	AC

AA	TT	AT	AT	AT	AT	AT	AT	TT	AA	AA	AT	AT
AA	TT	AT	AT	AT	AT	AT	AT	TT	AA	AA	AT	AT
AT	AA	AT	AT	AT	AT	AT	AT	AA	TT	TT	AT	AT
CT	TT	CT	CT	CT	TT	CC	CT	TT	CC	CC	CT	CT
GG	GG	AG	AG	AG	AG	AA	AG	GG	AA	AA	AG	AG
AA	AA	AC	AC	AC	AA	CC	AC	AA	CC	CC	AC	AC
TT	TT	CT	CT	CT	TT	CC	CT	TT	CC	CC	CT	CT
GG	GG	AG	AG	AG	GG	--	GG	GG	AA	AA	AG	GG
GG	GG	GT	GT	GT	GG	TT	GG	GG	TT	TT	GT	GG
--	GG	AG	AG	AG	GG	AA	GG	AG	AA	AA	AG	GG
CC	CC	CG	CC	CC	CC	CG	CC	CG	CG	CG	CG	CC
GG	GG	AG	GG	GG	GG	AG	GG	AG	AG	AG	AG	GG
CC	CG	CG	CG	CG	CC	GG	CC	CG	GG	CG	GG	CC
AA	AG	AG	AG	AG	AA	GG	AA	AG	GG	AG	GG	AG
GG	AG	AG	--	AG	GG	AA	GG	AA	AA	AG	AA	AG
AG	AG	AG	AG	AG	AG	GG	AA	GG	GG	AA	GG	AG
GG	GG	GT	GG	GG	GG	GT	GG	GT	GT	GG	GT	GG
AG	AG	AG	AG	AG	AG	AA	GG	AA	AA	GG	AA	AG
TT	TT	GT	TT	TT	TT	GT	TT	TT	GT	TT	GT	TT
CG	CG	CC	CG	GG	CG	CG	GG	CC	CC	GG	CC	CG
--	CT	TT	--	CT	TT	CT	TT	TT	TT	CT	CT	CT
CC	CC	CG	CG	CG	GG	CC	CG	CG	CG	CG	CG	CC
CG	CG	CG	CC	CC	CC	CG	CG	CG	CG	CC	CC	CG
AC	AC	AA	AC	AC	AA	AA	AA	AA	AA	AC	AC	AC
CT	CT	CC	CT	CT	CC	CC	CC	CC	CC	CT	CT	CT
AG	AG	AA	AG	AG	AA	AA	AA	AA	AA	AA	AG	AG
CT	CT	CC	CT	CT	CC	CC	CC	CC	CC	CC	CT	CT
CG	GG	CG	GG	GG	GG	GG	CG	CG	CG	GG	CG	CG
CT	CT	CC	CT	CT	CC	CC	CT	CC	CC	CC	CT	CT
GG	AG	AG	AG	AG	AG	AA	GG	AG	AG	AA	GG	GG
GT	GT	GG	GT	GT	GT	GG	GT	GG	GG	GG	GG	GT
GG	GT	GT	GT	GG	GT	TT	GG	GT	GT	TT	GT	GG
CG	CG	GG	--	CG	CG	GG	CG	GG	GG	GG	GG	CG
CC	CG	CG	CG	CC	CG	GG	CC	CG	CG	GG	CG	CC
AG	AG	GG	AG	AG	AG	GG	AG	GG	GG	GG	GG	AG
CC	CT	CT	TT	CC	CT	TT	CC	CT	CT	TT	CT	CC
TT	GT	GT	GT	TT	GT	GG	TT	GT	GT	GG	GT	TT
AG	AG	GG	AG	AG	AG	GG	AG	GG	GG	GG	GG	AG
GG	AG	AG	AG	GG	AG	AA	GG	AG	AG	AA	AG	GG
CC	CT	CT	--	CC	CT	TT	CC	CT	CT	TT	CT	CC
TT	--	TT	AT	AT	AT	TT	AT	TT	TT	TT	TT	AT
GT	GT	TT	GT	GT	GT	TT	GT	TT	TT	TT	TT	GT
TT	CT	CT	CT	TT	CT	CC	TT	CT	CT	CC	CT	TT
AG	AG	AA	AG	AG	AG	AA	AG	AA	AA	AA	AA	AG
AA	AT	AT	AT	AA	AT	TT	AA	AT	AT	TT	AT	AA
AG	AG	AA	AG	AG	AG	AA	AG	AA	AA	AA	AA	AG
CT	CT	TT	CT	CT	CT	TT	CT	TT	TT	TT	TT	CT

AT	AT	TT	AT	AT	AT	TT	AT	TT	TT	TT	TT	AT
TT	GT	GT	TT	TT	GT	GG	TT	GT	GT	GG	GT	TT
AG	AG	AG	AG	GG	AG	AA	GG	AG	AG	AA	AG	GG
--	CT	CT	CT	TT	CT	CC	TT	CT	--	CC	CT	TT
CT	CT	CT	CC	TT	CT	CC	TT	TT	CT	CC	CT	TT
CT	CT	CT	CC	TT	CT	CC	TT	TT	CT	CC	--	TT
GT	GT	TT	GG	TT	GT	GG	TT	TT	GT	GG	GT	TT
CG	CG	GG	CC	GG	CG	CC	GG	GG	CG	CC	CG	GG
AC	AC	AA	CC	AC	AC	CC	AA	AA	AA	CC	AC	AA
AG	AA	GG	AA	AG	AG	AA	GG	GG	GG	AA	AG	GG
AG	GG	AA	GG	AG	GG	GG	AA	AA	AA	GG	AG	AA
CC	CT	TT	--	CT	TT	CT	CT	TT	CT	CC	CC	CC
CC	AC	AA	AA	AC	AA	AC	AC	AA	AC	CC	CC	CC
GT	TT	TT	TT	TT	TT	TT	GT	TT	TT	GT	GT	GT
CG	CG	GG	--	CG	GG	CG	CG	GG	CG	CG	CG	CG
AG	AG	GG	GG	AG	GG	AG	AG	GG	AG	AA	AA	AA
CT	CT	TT	TT	CT	TT	CT	TT	CT	CT	CT	CT	CT
TT	GT	TT	TT	TT	TT	TT	GT	TT	TT	GT	GT	GT
CT	CC	CC	CC	CT	CC	CT	CC	CT	CT	CT	CT	CT
AA	AC	AA	AC	AA	AA	AA	AC	AA	AA	AC	AC	AC
AG	AG	AA	AG	AG	AA	AG	AG	AG	AA	GG	GG	GG
AA	AG	AA	AG	AA	AA	AA	AG	AA	AA	AG	AG	AG
GT	GT	TT	GT	GT	TT	GT	GT	GT	TT	GG	GG	GG
CT	CT	CC	CT	CT	CC	CT	CT	TT	CC	TT	TT	TT
AC	AC	AA	AC	AC	AA	AC	AC	CC	AA	CC	CC	CC
AT	AT	TT	AT	AT	TT	AT	AT	AA	TT	AA	AA	AA
GT	GG	GT	GG	GT	GT	GT	GG	GG	GT	GG	GG	GG
AA	AG	AG	AG	AA	AG	AA	AG	AA	AG	AA	AA	AA
CG	CG	CC	CG	CG	CC	CG	CG	GG	CC	GG	GG	GG
AG	AG	AA	AG	AG	AA	AG	AG	GG	AA	GG	GG	GG
GG	CC	CC	GG	CG	GG	CC	GG	GG	GG	GG	CG	CG
GG	AA	AA	GG	AG	GG	AA	GG	GG	GG	GG	AG	AG
AG	AA	AA	AG	AG	AG	AG	AG	AG	AG	AG	AA	AA
AT	AA	AA	AA	AA	AT	AA	AT	AT	AT	AT	AT	AT
AG	AA	AA	AG	AG	AG	--	AG	AG	AG	AG	AG	AA
AA	AG	AG	AA	AA	AG	AA	AA	AA	AA	AA	AA	AG
CC	AA	AC	CC	AC	AC	AC	AC	AC	AC	CC	CC	AA
AC	CC	AC	AC	CC	AC	CC	CC	CC	CC	AC	AC	CC
AA	CC	AC	AA	AA	AC	AC	AC	AC	AC	AA	AA	CC
TT	GG	GT	TT	TT	GT	GT	GT	GT	GT	TT	TT	GG
TT	AA	AT	TT	TT	AT	AT	AT	AT	AT	TT	TT	AA
AG	AA	AG	AG	AG	AG	AA	AA	AA	AA	AG	AG	AA
AC	CC	AC	AC	AC	AC	CC	CC	CC	CC	AC	AC	CC
TT	AA	AT	TT	TT	AT	AT	AT	AT	TT	TT	TT	AA
TT	CC	CT	TT	TT	CT	CT	TT	CT	CT	CT	CT	CC
AA	GG	AG	AG	AA	AG	AG	AA	AG	AG	AG	AG	GG
TT	CC	CT	CT	TT	CT	CT	TT	CT	CT	CT	CT	CC

CC	TT	CT	CT	CC	CT	CT	CC	CT	CT	CT	CT	TT
AA	GG	AG	AG	AA	AG	AG	AA	AG	AG	AG	AG	GG
TT	CC	CT	CT	TT	CT	CT	TT	CT	CT	CT	CT	CC
CC	--	CT	CT	CC	CT	CT	CC	CT	CT	CT	CT	TT
TT	CC	CT	--	TT	CT	CT	TT	CT	CT	CT	CT	CC
AA	GG	AG	AG	AA	AG	AG	AA	AG	AG	AG	AG	GG
CC	TT	TT	--	CC	CT	CT	CC	CT	CT	CT	CT	TT
AA	GG	GG	AG	AA	AG	AG	AA	AG	AA	AG	AG	GG
GG	CC	CC	CG	GG	CG	CG	GG	CG	GG	CG	CG	CC
CC	AA	AA	AC	CC	AC	AC	CC	AC	CC	AC	AC	AA
CT	CC	CC	CT	TT	CC	CT	TT	CT	TT	CT	CT	CC
CT	--	--	CT	TT	CC	--	TT	CT	TT	CT	CT	CC
AT	TT	AT	--	AT	TT	AT	AA	AT	AA	AT	AT	TT
CT	CC	CT	CT	CT	CC	TT	TT	CT	TT	CT	CT	CC
AC	AA	AC	AC	AC	AA	CC	CC	AC	CC	AC	AC	AA
GT	TT	GT	GT	GT	TT	GG	GG	GT	GG	GT	GT	TT
CC	--	CT	CT	CT	TT	CC	CC	CT	CC	CT	CT	TT
CG	GG	CG	CG	CG	GG	CC	CC	CG	CC	CG	CG	GG
CG	GG	CG	CG	CG	GG	CC	CC	CG	CC	CG	CG	CG
AC	CC	AC	AC	AC	CC	AA	AA	AC	AA	AC	AC	AC
CG	GG	CG	CG	CG	GG	CC	CC	CG	CC	CG	CG	CC
AT	AA	AA	AT	AT	AT	TT	TT	AT	TT	AT	AT	AT
AG	AA	AA	AG	AG	AG	GG	GG	GG	GG	AG	AG	AG
AG	--	AA	AA	AG	AG	GG	GG	AG	AG	AG	AG	AG
AG	GG	AG	GG	AG	AG	AA	AA	AA	AG	AG	AG	AG
AG	AA	AG	AA	AG	AG	GG	GG	GG	AG	AA	AG	AG
AC	AA	AC	AA	AC	AC	CC	CC	CC	AC	AA	AC	AC
AG	AA	AG	--	AG	AG	GG	GG	GG	AG	AA	AG	AG
AG	AA	AG	AA	AG	AG	GG	GG	GG	AG	AA	AG	AG
GT	GG	GT	GG	GT	GT	TT	TT	TT	GT	GG	GT	GT
GT	TT	GT	TT	GT	GT	GG	GG	GG	GT	TT	GT	GT
AC	CC	AC	CC	AC	AC	AA	AA	AA	AC	CC	AC	AC
CT	--	CT	--	CT	CT	CC	CC	CC	CT	CT	CT	CT
CT	TT	CT	TT	CT	CT	CC	CC	CC	TT	CT	CT	CT
GT	GG	GT	GG	GT	GT	TT	TT	TT	GT	GT	GT	GT
AT	AA	AT	AA	AT	AT	TT	TT	TT	AT	AT	AT	AT
CG	GG	CG	GG	CG	CG	CC	CC	CC	CG	CG	CG	CG
AC	AA	AC	AA	AC	AC	CC	CC	CC	AC	AC	AC	AC
CT	CC	CT	CC	CT	CT	TT	TT	TT	CT	CT	CT	CT
CG	--	CG	GG	CG	CG	CC	CC	CC	CG	CG	CG	CG
CT	CC	CT	--	CT	CT	TT	TT	TT	CT	CT	CT	CT
AT	AA	AT	AT	AT	AT	TT	TT	TT	AT	AT	AT	AT
CT	TT	CT	TT	CT	CT	CC	CC	CC	CT	CT	CT	CT
AG	GG	AG	GG	AG	AG	AA	AA	AA	AG	AG	AG	AG
AT	AA	AT	AA	AT	AT	TT	TT	TT	AT	AT	AT	AT
AT	AA	AT	AA	AT	AT	TT	TT	TT	AT	AT	AT	AT
AC	AA	AC	AA	AC	AC	--	CC	CC	AC	AC	AC	AC

GT	TT	GT	TT	GT	GT	GG	GG	GG	GT	GT	GT	GT
AG	GG	AG	GG	AG	AG	AA	AA	AA	AG	AG	AG	AG
GT	GG	GT	GG	GT	GT	TT	TT	TT	GT	GT	GT	GT
AG	AA	AG	AA	AG	AG	GG	GG	GG	AG	AG	AG	AG
CT	TT	CT	TT	CT	CT	CC	CC	CC	CT	CT	CT	CT
AG	AA	AG	AA	AG	AG	GG	GG	GG	AG	AG	AG	AG
CT	TT	CT	TT	CT	CT	CC	CC	CC	CT	CT	CT	CT
AC	CC	AC	--	AC	AC	AA	AA	AA	AC	AC	AC	AC
AG	AA	AG	AA	AG	AG	GG	GG	AA	--	AG	AG	AG
CT	CT	CT	TT	CT	CT	CT	CC	CT	CT	CT	CT	CT
AG	AG	AG	GG	AG	AG	AG	AA	AG	AG	AG	AG	AG
TT	CT	CT	CC	CT	CT	CT	TT	CC	CT	CC	CT	CT
GT	GG	GT	GG	GT	GT	GT	GT	GG	GT	GT	GG	GT
AG	AG	GG	GG	GG	AG	GG	AG	GG	GG	GG	AG	GG
AT	AT	AT	TT	--	AA	AT	AA	TT	--	AT	AT	AT
AT	AA	AT	TT	AA	AA	TT	AA	TT	TT	AT	AT	AT
AC	AA	CC	CC	AA	AA	CC	AA	CC	CC	AC	AC	AA
GT	TT	GG	GG	TT	TT	GG	TT	GG	GG	GT	GT	TT
TT	CT	TT	TT	TT	CT	CT	TT	CT	TT	TT	CT	CT
AG	AG	AG	AA	GG	AG	AG	AG	AG	GG	GG	AA	AG
GG	CG	GG	GG	CG	CG	CG	CG	CG	CG	CG	GG	CG
TT	CT	TT	CT	CT	TT	CT	--	CT	CT	CT	TT	CT
GT	GG	GT	GG	GT	GG	GG	GG	GT	GT	GT	GG	GG
AG	AG	AG	AG	GG	AA	AG	AG	GG	GG	GG	AA	AG
AA	AC	AA	--	AA	AA	AC	AC	--	AC	AC	AA	AC
GG	CG	GG	CG	CG	GG	CG	CG	CG	CG	CG	GG	CG
TT	GT	TT	--	TT	TT	GT	GT	GT	--	GT	TT	GT
CT	TT	CT	CT	CT	CT	CT	CT	TT	TT	TT	CC	CT
GG	AG	GG	AA	GG	GG	AA	AA	AG	GG	GG	AG	AA
CC	CT	CC	CT	CC	CC	CT	CT	CT	CC	CC	CC	CT
CG	CC	CG	CC	CG	CG	CC	CG	CC	CG	CG	CG	CC
AA	AA	AA	AC	AA	AA	AC	AC	AA	AA	AA	AC	AC
AG	AA	AG	AG	AG	AG	AG	GG	AA	AG	AG	GG	AG
CT	CT	CT	TT	CT	CT	TT	TT	CT	CT	CT	TT	TT
GG	CG	GG	CG	GG	GG	CG	GG	CG	GG	GG	GG	CG
AG	GG	AG	AG	AG	AG	AG	AA	GG	AG	AG	AA	AG
CG	GG	CG	GG	CG	CG	GG	CG	GG	CG	CG	CG	GG
CG	GG	CG	CG	CG	CG	CG	CC	GG	CG	CG	CC	CG
CT	TT	CT	CT	CT	CT	CT	CC	TT	CT	CT	CC	CT
AG	AA	AG	AG	AG	AG	AG	GG	AA	AG	AG	GG	AG
GG	GG	GG	AG	GG	GG	AG	AG	GG	GG	GG	AG	AG
CT	TT	CT	CT	CT	CT	CT	CC	TT	CT	CT	CC	CT
AG	AG	AG	GG	AG	AG	GG	GG	AG	AG	AG	GG	GG
AG	AA	AG	AG	AG	AG	AG	GG	AA	AG	AG	GG	AG
AC	AA	AC	AC	AC	AC	AC	CC	AA	AC	AC	CC	AC
TT	AT	TT	AT	TT	TT	AT	TT	AT	TT	TT	TT	AT
AG	GG	AG	AG	AG	AG	AG	AA	GG	AG	AG	AA	AG

AC	CC	AC	AC	AC	AC	AC	AA	CC	AC	AC	AA	AC
AG	GG	AG	AG	AG	AG	AG	AA	GG	AG	AG	AA	AG
AG	AG	AG	AA	AG	AG	AA	AA	AG	AG	AG	AA	AA
CC	CT	CC	CT	CC	CC	CT	CC	CT	CC	CC	CC	CT
CC	CG	CC	CG	CC	CC	CG	CC	CG	CC	CC	CC	CG
TT	TT	TT	CT	TT	TT	CT	TT	CT	TT	TT	TT	CT
CG	CG	CG	GG	CG	CG	GG	--	GG	CG	CG	CG	GG
CC	CC	CC	CT	CC	CC	CT	CT	CT	CC	CC	CT	CT
GT	GT	GT	GG	GT	GT	GG	GG	GG	GT	GT	GG	GG

7-43	7-46	7-47	7-48	7-51	7-53	P-F	P-M	GP-F	GP-M
AA	AC	AC	AC	AC	CC	AC	AC	AC	AC
AA	AG	AG	AG	AG	GG	AG	AG	AA	GG
TT	CT	CT	CT	CT	CC	CT	CT	TT	--
GG	AG	GG	GG	GG	AG	GG	AG	GG	--
AA	AG	AG	AG	AG	GG	AG	AG	--	--
GG	GG	GG	GG	GG	GG	CG	GG	CG	GG
CT	CC	CT	CT	CT	CC	CT	CT	TT	--
GT	GG	GT	GG	GT	GG	GT	GT	TT	GG
GT	GG	GT	GG	GT	GG	GT	GT	GT	--
GT	GG	GT	GG	GT	GG	GT	GT	TT	GG
AG	GG	AA	GG	AG	GG	AG	AG	AA	--
CC	CC	CT	CC	CC	CC	CT	CC	CT	CC
AC	CC	AC	CC	AC	AC	AC	AC	AA	CC
AA	AA	AG	AA	AA	AA	AG	AA	AG	--
CT	CC	CC	CC	CT	CT	CC	CT	CT	--
AG	AA	AG	AA	AG	AG	AG	AG	GG	AA
TT	TT	AT	TT	TT	TT	AT	TT	AT	--
CT	CC	CC	CC	CT	CT	CC	CT	CT	--
TT	TT	GT	TT	TT	TT	GT	TT	GT	--
CC	CC	CC	CC	CC	CC	CG	CC	CG	CG
AT	TT	TT	TT	AT	AT	AT	AT	AA	--
CT	TT	TT	TT	CT	CT	CT	CT	CC	--
AT	AT	AA	AA	AT	AT	AT	AT	AT	AA
AT	AA	AA	AA	AT	AT	AT	AT	TT	--
AG	AA	AA	AA	AG	AG	AG	AG	GG	--
TT	TT	TT	TT	TT	TT	GT	TT	GT	TT
GT	TT	TT	TT	GT	GT	GT	GT	GG	TT
AT	TT	TT	TT	AT	AT	AT	AT	AA	TT
CT	TT	TT	TT	CT	CT	CT	CT	CC	TT
CG	CG	CC	CC	CG	CG	CG	CG	GG	CG
CT	CC	CC	CC	CT	CT	CT	CT	CT	CC
AC	AA	AA	AA	AC	AC	AC	AC	AC	--
AG	AA	AA	AA	AG	AG	AG	AG	GG	--
GT	GG	GG	GG	GT	GT	GT	GT	GT	--
AG	AA	AA	AA	AG	AG	AG	AG	AG	--
GT	GT	TT	TT	GT	GT	GT	GT	--	TT
AG	AG	GG	GG	AG	AG	AG	AG	AA	--
CT	CT	CC	CC	CT	CT	CT	CT	TT	CC
AT	AT	AA	AA	AT	AT	AT	AT	TT	AA
AG	AG	AA	AA	AG	AG	AG	AG	GG	--
AC	AC	AA	AA	AC	AC	AC	AC	CC	AA
AG	AG	AA	AA	AG	AG	AG	AG	AG	AG

CT	CT	TT	TT	--	CT	CT	CT	CT	--
CG	CG	CC	CC	CG	CG	CG	CG	CG	--
CT	CT	TT	TT	CT	CT	CT	CT	CT	TT
CT	CT	CC	CC	TT	CT	CT	CT	TT	--
AC	AC	AA	AA	CC	AC	AC	AC	AC	AA
CT	CT	CC	CC	TT	CT	CT	CT	CT	--
CT	CT	CT	CT	CC	CC	CT	CT	CC	TT
AG	GG	AA	AG	GG	AG	AG	AG	AG	GG
GG	GG	TT	GT	GG	GT	GT	GT	TT	GG
GG	GG	AA	GG	GG	AG	AG	AG	AA	--
TT	CT	CC	TT	TT	CT	CT	CT	CC	TT
CC	CG	GG	CC	CC	GG	CG	CG	GG	--
AA	AG	GG	AA	AA	GG	AG	AG	AG	--
CC	AC	AA	AC	CC	AA	AC	AC	AC	CC
AG	AG	GG	AG	AG	GG	GG	AG	GG	AG
TT	CT	CC	CT	TT	CC	CT	CT	CT	--
CT	CT	TT	CT	CC	TT	CT	CT	CT	--
CT	CT	CC	CT	TT	CC	CT	CT	CC	TT
AG	AG	GG	AG	AA	GG	AG	AG	AG	AA
AC	AC	CC	AC	AA	CC	AC	AC	AC	--
AG	AG	GG	AG	AA	GG	AG	AG	AG	--
CG	CG	CC	CG	GG	CC	CG	CG	CC	--
CG	CG	GG	CG	CC	GG	CG	CG	GG	CC
AG	AG	GG	AG	AA	AA	AG	AG	GG	AA
AC	AC	AC	AC	AC	CC	AC	AC	AA	--
CT	CT	CT	CT	CT	TT	CT	CT	CT	--
CT	CT	CT	CT	CT	CC	CT	CT	TT	CC
AT	AT	AT	AT	AT	TT	AT	AT	AT	--
AG	GG	AG	GG	GG	GG	GG	AG	AG	--
TT	TT	TT	AT	AT	TT	AT	TT	AT	TT
GT	GT	GG	GT	TT	GG	GT	GT	GG	GT
AG	GG	AG	AG	GG	AG	AG	GG	AG	--
CT	TT	CT	CT	TT	CT	CT	TT	CT	TT
GG	GG	AG	AG	GG	AG	AG	GG	AG	GG
GG	GT	TT	GT	GG	TT	GT	GT	TT	GG
GG	GG	GT	GG	GG	GT	GT	GG	--	GG
CT	CT	CC	TT	TT	CC	CT	CT	CT	TT
CT	CT	CT	TT	TT	CC	CT	CT	CT	--
AG	AG	AG	AA	AA	GG	AG	AG	GG	AA
CG	CG	CG	GG	GG	CC	CG	CG	CG	--
AG	AG	AG	AA	AA	GG	AG	AG	AG	AG
CT	CT	TT	CC	CC	TT	CT	CT	CT	--
CT	CT	TT	CC	CC	TT	CT	CT	TT	CC
CC	CC	CG	CC	CC	CG	CG	CC	CG	CC
TT	TT	AT	TT	AT	AT	AT	TT	AT	--
GT	GG	GT	GG	GG	GT	GG	GT	GT	GG
CT	CC	CT	CC	CT	CT	CC	CT	CT	CC

CT	CC	TT	CC	TT	TT	CT	CT	TT	--
GT	GG	GT	GG	GT	GT	GG	GT	GT	--
CT	CC	TT	CC	TT	CT	CT	CT	TT	CC
GT	TT	GT	TT	GT	GT	TT	GT	GT	TT
CG	GG	CG	GG	CG	CG	GG	CG	CG	--
AA	AG	AA	AA	AG	AA	AG	AG	AA	--
GG	AG	GG	GG	AG	GG	AG	AG	AG	AA
GG	AG	GG	GG	AG	GG	AG	AG	GG	AG
CC	CT	CC	CC	CT	CC	CT	CT	CC	TT
AA	AG	AA	AG	AG	AA	AG	AG	AA	--
TT	GT	TT	GT	TT	TT	GT	GT	GT	GG
GG	AG	GG	AG	GG	GG	AG	GG	AG	AG
CC	CT	CC	CT	CC	CT	CT	CT	CC	TT
AC	CC	AC	AC	AC	AC	CC	AC	AC	--
CC	CT	CC	CC	CC	CC	CC	CT	CC	CT
TT	GT	TT	GT	TT	TT	GT	GT	TT	GG
CT	CT	CT	CC	CT	CT	CT	CC	CT	CC
CG	GG	CG	CG	CG	CG	GG	CG	CG	GG
AC	CC	AC	AC	AC	AC	CC	AC	AC	--
CT	CT	CT	TT	CT	CT	CT	TT	CT	--
AG	AG	AG	AA	AG	AG	AG	AA	AG	AA
GG	AG	GG	AG	GG	GG	AG	AG	GG	AA
CC	CT	CC	CC	CC	CC	CC	CT	CC	CT
AT	AA	AT	AT	AT	AT	AA	AT	AT	--
CT	CC	CT	CT	CT	CT	CC	CT	CT	--
AG	AA	AG	AG	AG	AG	AA	AG	AG	--
CT	CT	CT	CC	CT	CT	CT	CC	CT	--
AA	AT	AA	AT	AA	AA	AT	AT	AA	--
AA	AT	AA	AT	AA	AA	AT	AT	--	--
CC	CT	CC	CT	CC	CC	CT	CT	CC	TT
CT	CC	CT	CT	CT	CT	CC	CT	CT	CC
GT	GT	GT	GG	GT	GT	GT	GG	GT	--
CT	CT	CT	CC	CT	CT	CT	CC	CT	--
AA	AA	AA	AG	AA	AA	AG	AA	AA	--
AA	AG	AA	AG	AA	AA	AG	AG	AA	--
AG	AG	AG	AA	AG	AG	AG	AA	AG	AA
TT	CT	TT	CT	TT	TT	CT	CT	TT	--
AC	AC	AC	CC	AC	AC	AC	CC	AC	--
AA	AT	AA	AT	AA	AA	AT	AT	AA	--
AG	AA	AG	AG	AG	AG	AA	AG	AG	--
AC	AC	AC	AA	AC	AC	AC	AA	AC	AA
AT	AT	AT	TT	AT	AT	AT	TT	AT	--
AC	AA	AC	CC	AC	AC	AC	AC	AC	AC
AC	CC	AC	AC	AC	AC	CC	AC	AC	--
GG	GG	GG	AG	GG	GG	AG	GG	GG	--
CC	AC	CC	CC	CC	CC	CC	AC	CC	AC
AG	AG	AG	AA	AG	AG	AG	AA	AG	AA

CG	CC	CG	CG	CG	CG	CC	CG	CG	CC
CC	CT	CC	CT	CC	CC	CT	CT	CC	TT
TT	CT	TT	CT	TT	TT	CT	CT	TT	CC
CT	CT	CT	CC	CT	CT	CT	CC	--	CC
AG	AG	AG	AA	AG	AG	AG	AA	AG	--
TT	CT	TT	CT	TT	TT	CT	CT	TT	--
CT	CT	CT	TT	CT	CT	CT	TT	--	--
TT	AT	TT	AT	TT	TT	AT	AT	TT	--
AG	GG	AG	AG	AG	AG	GG	AG	AG	GG
AC	AC	AC	AA	AC	AC	AC	AA	AC	--
CT	CT	CT	CC	CT	CT	CT	CC	CT	--
GT	TT	GT	GT	GT	GT	TT	GT	GT	TT
--	CT	CT	TT	CT	CT	CT	TT	CT	--
CC	CC	CC	AC	CC	CC	AC	CC	CC	--
AG	AG	AG	AA	AG	AG	AG	AA	AG	--
GT	GT	GT	GG	GT	GT	GT	GG	GT	--
TT	TT	TT	AT	TT	TT	AT	TT	--	--
AG	AG	AG	AA	AG	AG	AG	AA	AG	--
CT	CC	CT	CT	CT	CT	CC	CT	CT	--
CT	CT	CT	CC	CT	CT	CT	CC	CT	--
AC	AC	AC	AA	AC	AC	AC	AA	AC	AA
CT	CT	CT	CC	CT	CT	CT	CC	CT	--
TT	AT	TT	AT	TT	TT	AT	AT	TT	--
AA	AG	AA	AG	AA	AA	AG	AG	AA	GG
CG	GG	CG	CG	CG	CG	GG	CG	CG	--
CT	CT	CT	TT	CT	CT	CT	TT	CT	--
AG	AG	AG	GG	AG	AG	AG	GG	AG	--
AA	AG	AG	AA	AG	AG	AG	AA	AG	AA
AA	AA	AG	AG	AG	AG	AA	AG	AG	AA
GG	GG	AG	AG	AG	AG	GG	AG	AG	--
TT	CT	CT	TT	TT	CT	CT	TT	CT	TT
GG	GT	TT	GT	GT	TT	GT	GT	TT	--
TT	TT	GT	GT	GT	GT	TT	GT	GT	--
GG	AG	AA	AG	AG	AA	AG	AG	AA	--
CC	CT	CC	CC	CC	CT	CT	CC	CT	--
TT	GT	TT	TT	TT	GT	GT	TT	GT	TT
GG	AG	GG	GG	GG	GG	GG	AG	GG	--
AG	AG	AG	GG	AG	GG	AG	AG	AA	--
CT	CC	CT	CC	CT	CC	CT	CC	CT	--
AT	AA	AT	AT	AT	AT	AA	AT	AA	AT
CG	CG	CG	GG	CG	GG	CG	CG	CC	GG
AA	AC	AA	AC	AA	--	AC	AA	AA	--
AG	AA	AG	AG	AG	AG	AG	AG	AA	--
GG	AG	GG	GG	GG	GG	GG	AG	AG	GG
CC	CT	CC	CC	CC	CC	CC	CT	CT	CC
AG	GG	AG	AG	AG	AG	AG	AG	GG	AA
CC	CC	CC	CC	CC	CC	CT	CC	CT	--

AG	GG	AG	AG	AG	AG	GG	AG	GG	--
AG	AG	AA	AA	AA	AA	AG	AG	AG	--
TT	CC	CT	CT	TT	CT	CT	CT	CT	TT
TT	AA	AT	AT	TT	TT	AT	AT	AT	--
CC	AA	AC	AC	CC	CC	AC	AC	AA	CC
CC	TT	CT	CT	CC	CC	CT	CT	TT	CC
--	GG	AG	AG	AA	AA	AG	AG	--	AA
TT	CT	CT	CT	TT	TT	CT	CT	CT	--
CT	TT	TT	TT	CT	CT	CT	TT	TT	CT
GG	AG	AG	AG	GG	GG	AG	AG	AA	--
AG	AG	GG	AA	AG	GG	AG	AG	AG	--
CT	CT	TT	CC	CT	TT	CT	CT	CC	--
AG	GG	AG	GG	GG	AG	AG	GG	GG	--
CT	CT	TT	CC	CT	TT	CT	CT	CC	TT
GG	CG	CG	GG	CG	CG	GG	CG	--	CG
CT	CT	TT	CT	CT	TT	CT	CT	CT	--
CT	CC	CT	CT	CC	CT	CT	CC	CT	--
AG	AG	AA	AG	AG	AA	AG	AG	GG	AA
CT	CT	TT	CT	CT	TT	CT	CT	CT	--
AG	AG	AG	AG	GG	GG	AG	AG	AG	--
CT	CT	CT	CT	TT	TT	CT	CT	CC	TT
AG	AG	AG	AG	AA	AA	AG	AG	GG	AA
GG	GG	GG	AG	AG	AG	AG	GG	GG	--
TT	TT	GT	GT	GG	GT	GT	GT	TT	GG
CC	CC	CC	CG	CG	CC	CG	CC	CC	CG
CG	CG	CC	CG	CC	CC	CC	CG	CG	CC
CC	CC	CT	CT	TT	CT	CT	CT	CT	TT
--	TT	CT	CT	CT	CT	CT	CT	TT	CC
AA	AA	AT	AA	AT	AT	AT	AT	AA	TT
GG	GG	AG	GG	AG	AG	AG	AG	AG	AA
CC	CC	--	CC	CT	CT	CT	CT	CC	TT
TT	TT	TT	TT	TT	TT	CT	TT	CT	--
AA	AA	AC	AA	AC	AC	AC	AC	AA	--
GT	TT	GT	TT	GT	GG	GT	GT	GG	--
AG	AA	AG	AA	AG	GG	AG	AG	AG	AA
CT	TT	CT	TT	CT	CC	CT	CT	CT	--
AT	AA	AT	AA	AT	TT	AT	AT	AT	AA
AC	CC	AC	CC	AC	AA	AC	AC	AC	--
CC	CG	CG	CG	CG	CC	CC	CG	CC	CG
AA	AG	AG	AG	AG	AA	AA	AG	AA	--
CT	CC	CT	CC	CT	TT	CT	CT	TT	--
CT	CC	CT	CC	CT	TT	CT	CT	CT	--
AG	AA	AG	AA	AG	GG	AG	AG	AG	--
CT	CT	CC	CT	CC	CC	CT	CC	CT	CT
AG	AA	AG	AA	AG	GG	AG	AG	GG	--
AG	GG	AG	GG	AG	AA	AG	AG	AG	--
GT	GG	GT	GG	GT	TT	GT	GT	GT	GG

CT	TT	CT	TT	CT	CC	CT	CT	CT	--
CG	GG	CG	GG	CG	CC	CG	CG	CC	GG
GT	TT	GT	TT	GT	GG	GT	GT	GT	TT
AG	AA	AG	AA	AG	GG	AG	AG	GG	AA
CT	CC	CT	CC	CT	TT	CT	CT	CT	--
GT	GT	TT	GT	TT	TT	GT	TT	GT	GT
CG	GG	CG	GG	CG	CC	CG	CG	CG	--
AG	AA	AG	AA	AG	GG	AG	AG	--	--
AG	AG	GG	AG	GG	GG	AG	GG	GG	--
AC	AA	AC	AA	AC	CC	AC	AC	AC	AA
CG	CG	CC	CG	CC	CC	CG	CC	CG	CG
AG	GG	AG	GG	AG	AA	AG	AG	AG	GG
AC	AA	AC	AA	AC	CC	AC	AC	CC	--
GT	TT	GT	TT	GT	GG	GT	GT	GG	TT
AC	CC	AC	CC	AC	AA	AC	AC	AC	--
CT	CT	CC	CT	CC	CC	CT	CC	CC	--
GT	TT	GT	TT	GT	GG	GT	GT	GG	TT
GG	AG	AG	AG	AG	GG	GG	AG	GG	AG
CT	CT	CT	CC	CT	TT	CT	CT	TT	--
CT	CT	CC	CT	CC	CC	CT	CC	--	CT
AT	AT	AT	AA	AT	TT	AT	AT	TT	AA
CG	CG	CG	CC	CG	GG	CG	CG	GG	--
TT	GT	TT	GG	GT	TT	GT	GT	TT	GG
GG	GG	GG	AG	AG	GG	GG	AG	GG	--
GT	TT	GT	--	GT	GT	GT	TT	--	--
TT	CT	TT	CC	CT	TT	CT	CT	TT	CC
AA	AC	AA	AC	AC	AA	AC	AC	AA	CC
CT	CT	CT	CT	CC	CT	CC	CT	--	--
CC	CG	CC	CG	CG	CC	CG	CG	CC	GG
TT	CT	TT	CT	CT	TT	CT	CT	CT	--
TT	TT	TT	TT	GT	TT	TT	GT	GT	GT
TT	CT	TT	CT	TT	CT	CT	TT	TT	CT
TT	TT	TT	TT	AT	TT	TT	AT	AT	AT
AC	AC	AC	AC	CC	AC	CC	AC	AC	CC
CC	CT	CC	CT	CT	CT	CT	CT	CC	--
AG	AG	AG	AG	AA	AG	AA	AG	AG	AA
AG	AG	AG	AG	GG	AG	GG	AG	AG	GG
GG	GG	CG	GG	CG	CG	CG	GG	CG	GG
TT	CC	CT	CT	CT	CT	CT	CT	TT	CC
TT	CC	CT	CT	CT	CT	CT	CT	TT	--
TT	CC	CT	CT	CT	CT	CT	CT	CT	--
CC	TT	CT	CT	CT	CT	CT	CT	CC	TT
TT	GG	GT	GT	GT	GT	GT	GT	TT	--
--	TT	CT	CT	CT	TT	CT	CT	CT	--
AA	TT	AT	AT	AT	TT	AT	AT	AA	TT
AA	GG	AG	AG	AG	GG	AG	AG	AA	GG
CC	AA	AC	AC	AC	AA	AC	AC	AC	AA

AA	GG	AG	AG	AG	GG	AG	AG	AG	GG
--	GG	AG	AG	AG	GG	AG	AG	AA	--
TT	CC	CT	CT	CT	CC	CT	CT	TT	--
GG	AA	AG	AG	AG	AA	AG	AG	GG	--
GG	TT	GT	GT	GT	TT	GT	GT	GT	TT
TT	CC	CT	CT	CT	CC	CT	CT	CT	--
GG	AA	AG	AG	AG	AA	AG	AG	AG	--
GG	AA	AG	AG	AG	AA	AG	AG	GG	AA
AA	GG	AG	AG	AG	GG	AG	AG	AG	GG
GG	CC	CG	CG	CG	CC	CG	CG	GG	CG
AA	GG	AG	AG	AG	GG	AG	AG	AA	--
--	CC	AC	AC	AC	CC	AC	AC	AA	--
GG	AA	AG	AG	AG	AA	AG	AG	GG	--
GG	AA	AG	AG	AG	AA	AG	AG	AG	AA
AA	TT	AT	AT	AT	TT	AT	AT	AA	TT
TT	CC	CT	CT	CT	CC	CT	CT	CT	CC
TT	CC	CT	CT	CT	CC	CT	CT	TT	CC
CC	GG	CG	CG	CG	GG	CG	CG	CG	GG
CC	TT	CT	CT	CT	TT	CT	CT	CC	TT
TT	CC	CT	CT	CT	CC	CT	CT	TT	--
CC	TT	CT	CT	CT	TT	CT	CT	CT	--
AA	TT	AT	AT	AT	TT	AT	AT	AA	TT
TT	AA	AT	TT	AT	AA	AT	AT	AT	--
CC	TT	CT	CC	CT	TT	CT	CT	CT	CT
TT	CC	CT	TT	CT	CC	CT	CT	CT	CC
TT	AA	AT	TT	AT	AA	AT	AT	TT	AA
AA	GG	AG	AA	AG	GG	AG	AG	AG	GG
TT	GG	GT	TT	GT	GG	GT	GT	GT	--
CC	TT	CT	CC	CT	TT	CT	CT	CC	TT
GG	CC	CG	GG	CG	CC	CG	CG	CG	--
AA	GG	AG	AA	AG	GG	AG	AG	AA	--
CC	TT	CT	CC	CT	TT	CT	CT	CT	CT
CC	TT	CT	CC	CT	TT	CT	CT	CT	TT
AA	TT	AT	AA	AT	TT	AT	AT	AA	TT
GG	AA	AG	GG	AG	AA	AG	AG	GG	--
GG	AA	AG	GG	AG	AG	AG	AG	AG	--
GG	TT	GT	GG	GT	GT	GT	GT	GG	TT
GG	AA	AA	GG	AG	AG	AG	AG	GG	AA
AG	GG	GG	AG	AG	GG	AG	GG	AG	GG
TT	CT	CT	TT	CT	CT	TT	CT	CT	--
AG	AG	AG	AG	--	AG	AG	AG	AG	--
AG	GG	GG	AG	AG	GG	AG	GG	AG	AG
AG	AG	AG	AG	AA	AA	AG	AG	GG	--
CG	CG	CG	CG	GG	GG	CG	CG	CG	GG
CT	CT	CT	CT	CC	CC	CT	CT	TT	--
CC	CC	CC	AC	CC	CC	CC	AC	AC	--
AG	AG	AG	AA	AA	AA	AG	AA	AG	--

CC	CC	CC	AC	CC	CC	CC	AC	AC	CC
CC	CC	CC	AC	AC	AC	AC	CC	CC	--
TT	TT	TT	CT	TT	TT	TT	CT	CT	TT
AG	AG	AG	AG	AA	AA	AG	AG	GG	AA
AG	AG	AG	AA	GG	GG	AG	AG	AG	GG
CT	CT	CT	CC	TT	TT	CT	CT	CT	TT
TT	TT	TT	TT	TT	CT	CT	TT	--	--
CG	GG	CG	GG	CG	CC	CG	CG	GG	--
AG	GG	AG	GG	AG	AG	AG	AG	AG	--
CT	CT	CT	CC	CT	CC	CT	CT	CT	--
AA	AT	AA	AA	AA	AA	AT	AA	AT	AT
AC	AC	AC	AA	AC	AA	AC	AC	AA	CC
GG	AG	GG	AG	GG	AG	GG	AG	AG	--
CC	CT	CC	CC	CC	CT	CC	CT	CT	--
CC	CT	CC	CC	CC	CT	CC	CT	CT	--
GG	GT	GG	GT	GG	GT	GG	GT	GT	--
CT	CC	CT	CT	CC	CT	CT	CT	TT	CC
GG	AG	AG	AG	AG	AG	GG	AG	AG	AG
AG	AA	AG	AG	AA	AG	AG	AG	GG	AA
GG	GG	CG	GG	GG	CG	CG	GG	CG	--
AG	AA	GG	AG	AA	AG	AG	AG	GG	AA
AG	GG	AA	AG	GG	AG	AG	AG	AG	GG
GT	GT	TT	GT	GT	TT	GT	TT	TT	GT
AG	GG	AA	AG	GG	AG	AG	AG	AA	GG
AG	GG	AA	AG	GG	AG	AG	AG	AA	--
GT	GT	GT	GT	GT	GG	GT	GG	GG	GT
CT	TT	CT	CT	TT	CT	CT	CT	CC	--
CT	CC	CT	CT	CC	CT	CT	CT	CT	--
CC	CT	CC	CC	CT	CT	CC	CT	CT	CT
AG	AG	AG	AA	AG	AG	AG	AG	AA	GG
CT	CT	CT	CC	CT	CT	CT	CT	CT	TT
AG	AG	AG	AA	AG	AG	AG	AG	AA	--
CT	CT	CT	CC	CT	CT	CT	CT	CC	--
CG	CG	CG	GG	CG	CG	CG	CG	CG	CC
CT	CT	CT	CC	CT	TT	CT	CT	CT	CT
--	AA	CC	AC	AC	CC	AC	AC	--	--
AG	GG	AA	AG	AG	AA	AG	AG	AA	--
CT	TT	CC	CT	CT	CC	CT	CT	CT	--
AA	AA	GG	AG	AG	GG	AG	AG	GG	--
AA	AA	GG	AG	AG	GG	AG	AG	GG	--
TT	TT	CT	TT	TT	CT	TT	CT	CT	TT
AG	GG	AA	GG	AG	AG	AG	AG	AA	--
AG	AA	AG	--	AG	AA	AG	AA	AG	--
GT	TT	GT	TT	GT	TT	GT	TT	GT	TT
GT	TT	GT	TT	GT	TT	GT	TT	GT	--
AC	AA	CC	AA	AC	AC	AC	AC	CC	--
AG	AA	GG	AA	AG	AG	AG	AG	GG	AA

CG	CC	GG	CC	--	CG	CG	CG	GG	--
CG	CC	GG	CC	CG	CG	CG	CG	GG	CC
CT	CT	CC	TT	CT	CT	CT	CT	CT	TT
CT	CT	TT	CC	CT	CT	CT	CT	TT	CC
AT	AT	TT	AT	AT	AT	AT	AT	AT	AA
GG	GG	GG	GG	GG	AG	AG	GG	GG	AG
CT	CT	TT	CT	CC	CT	CT	CT	TT	--
CT	CT	CT	CC	CT	CC	CT	CT	CT	TT
CT	CT	CT	TT	CT	TT	CT	CT	TT	CC
AG	GG	AG	AA	AG	AA	AG	AG	AG	GG
CC	CC	CC	CG	CG	CG	CC	CG	CG	--
CG	GG	CG	CG	GG	CG	CG	GG	CG	--
GG	GG	GG	GT	GT	GG	GG	GT	GT	--
CG	CC	CC	CC	CC	CC	CC	CG	CC	CG
AG	AG	GG	AG	GG	GG	AG	AG	GG	--
AC	AA	AA	AC	AA	AA	AA	AC	AA	AC
AT	AA	AA	AA	AA	AA	AA	AT	AA	AT
AG	GG	GG	GG	GG	AG	AG	AG	GG	AA
TT	AA	AA	AA	AA	AT	AT	AT	AA	--
AA	GG	AG	GG	GG	AG	AG	AG	GG	AA
AC	CC	AC	CC	CC	AC	AC	CC	CC	AC
CT	CC	CT	CC	CC	CT	CT	CT	CT	--
GT	TT	GT	TT	GT	TT	GT	GT	TT	GG
GT	TT	GT	TT	GG	TT	GT	GT	TT	GG
CT	CT	CT	CC	TT	CC	CT	CT	CC	TT
CG	CG	CG	CC	GG	CC	CG	CG	CG	GG
AG	AG	AG	GG	AA	GG	AG	AG	GG	--
CT	CT	CT	CC	TT	CC	CT	CT	CC	--
GT	GT	GT	TT	GG	TT	GT	GT	TT	GG
CT	CT	CT	TT	CC	TT	CT	CT	TT	CC
CG	CG	CG	GG	CC	GG	CG	CG	CG	--
AG	AG	AG	GG	AA	GG	AG	AG	AG	AA
AC	AC	AC	AA	CC	AA	AC	AC	AC	--
CT	CT	CT	TT	CC	TT	CT	CT	TT	CC
GG	AG	GG	GG	AG	GG	GG	AG	GG	--
AC	AC	AC	AA	CC	AA	AC	AC	AC	--
CT	CT	CT	CC	TT	CC	CT	CT	CT	TT
AG	AG	AG	GG	AA	GG	AG	AG	AG	AA
CG	CG	CG	GG	CC	GG	CG	CG	GG	CC
GT	GT	GT	GG	TT	GG	GT	GT	GG	--
CT	CT	CT	CC	TT	CC	CT	CT	CC	TT
TT	CT	TT	TT	CT	TT	TT	CT	TT	--
GG	GG	GG	AG	GG	AG	AG	GG	AG	GG
CG	GG	CG	CC	GG	CC	CG	CG	CG	GG
AT	AT	AT	AA	AT	AA	AT	AA	AA	AT
AA	AG	AA	AA	AG	AA	AA	AG	AA	AG
CC	AC	CC	CC	AC	CC	CC	AC	CC	AC

TT	CT	TT	TT	CT	TT	TT	CT	TT	CT
CT	CT	CT	CT	CT	TT	CT	TT	TT	--
AG	AT	AG	AG	AT	GG	AG	GT	AG	--
AA	AT	AA	AA	AT	AA	AA	AT	AA	AT
GG	AA	AG	AG	AA	GG	AG	AG	AG	AA
CC	AA	AC	AC	AA	AC	AC	AC	CC	--
AA	AG	AA	AA	AG	AA	AA	AG	AA	AG
GG	AA	AG	AG	AA	AG	AG	AG	GG	--
AA	GG	AG	AG	GG	AG	AG	AG	AG	--
GG	AA	AG	AG	AA	AG	AG	AG	GG	--
GG	TT	GT	GT	GT	GT	GT	GT	GG	TT
GG	AG	AG	GG	GG	AG	AG	GG	GG	AG
CC	TT	CC	CT	CT	CC	CT	CT	CC	--
CC	GG	CC	CG	CG	CC	CG	CG	CC	--
TT	CT	TT	TT	CT	TT	TT	CT	TT	CT
TT	CT	TT	CT	TT	TT	CT	TT	TT	TT
CT	CC	CT	CC	CC	CC	CC	CT	CT	CC
GG	GT	GT	GT	GG	GT	GT	GG	GT	--
AG	AG	AA	AG	GG	AG	AG	AG	AA	--
AG	AG	GG	AG	AA	AG	AG	AG	GG	--
AA	AA	AG	AA	AA	AA	AA	AG	AG	--
GG	GT	TT	GT	GG	GT	GT	GT	TT	--
AA	AA	AG	AA	AA	AA	AA	AG	AG	--
CC	CC	CT	--	CC	CC	CC	CT	CT	--
AG	AG	AA	AG	AG	AG	AA	AG	AA	--
GG	AA	AA	AG	GG	AG	AG	AG	AA	--
GG	AA	AA	AG	GG	AG	AG	AG	AA	AG
GG	CC	CC	CG	GG	CG	CG	CG	CC	--
GG	CC	CC	GG	GG	CG	CG	CG	CG	--
GG	AA	AA	GG	GG	AG	AG	AG	AA	GG
GG	AG	AG	GG	GG	AG	AG	GG	AG	--
TT	CC	CC	TT	TT	CT	CT	CT	CC	--
CC	AC	AC	CC	CC	CC	AC	CC	AC	--
CC	CG	CG	CC	CC	CC	CG	CC	CG	CC
CC	AC	AC	CC	CC	CC	AC	CC	AC	CC
AA	AC	CC	AC	AA	AA	AC	AC	CC	AC
AA	AG	AG	AA	AA	AA	AA	AG	AG	--
AA	AG	AG	AG	AA	AA	AG	AG	GG	AA
GG	AG	GG	AG	GG	GG	GG	AG	AG	--
CC	CT	CT	CT	CC	CC	CT	CT	TT	--
--	CT	CT	CT	CC	CC	CT	CT	TT	--
AA	AA	AG	AA	AA	AA	AG	AA	AG	--
CC	CC	--	CC	CC	CC	CT	CC	CT	--
GG	AG	GG	AG	AA	AG	AG	AG	GG	--
CC	AC	CC	AC	AC	CC	AC	CC	CC	--
CC	CC	CC	CC	CT	CT	CC	CT	CC	CT
AA	AA	AA	AG	AG	AG	AG	AG	AG	--

AA	AA	AA	AA	AG	AG	AA	AG	AG	AG
AA	AA	AA	AG	AG	GG	AG	AG	AA	GG
CC	CC	CC	AC	AC	AA	AC	AC	AC	--
GG	GG	GG	AG	AG	AA	AG	AG	GG	AA
TT	GT	TT	GT	GT	GG	GT	GT	TT	--
AA	AA	AA	AG	AA	AG	AG	AA	AG	AG
TT	CT	TT	CT	CT	CC	CT	CT	CT	CC
CC	CC	CC	CT	CC	CT	CT	CC	CT	CT
AA	AG	GG	AG	AG	AA	AG	AG	GG	AA
CG	CG	CG	GG	CG	CG	GG	CG	CG	CG
CT	CT	CT	TT	CT	CT	TT	CT	--	TT
GG	AG	AG	AA	AG	AG	AG	AG	AG	--
GG	AG	AG	AA	AA	AG	AG	AG	AG	--
GG	CG	CG	CC	CC	CG	CG	CG	CG	--
GG	AG	AG	AA	--	AG	AG	AG	AA	GG
GG	AG	AA	AA	AG	GG	AG	AG	AA	--
TT	CT	CC	CC	CT	TT	CT	CT	CT	TT
GG	AG	AA	AA	AG	GG	AG	AG	AG	GG
CC	CG	GG	GG	CG	CC	CG	CG	CG	CC
CC	AC	AA	AA	AC	CC	AC	AC	AC	--
AA	AG	--	GG	AG	AA	AG	AG	AG	--
GG	GT	TT	TT	GT	GG	GT	GT	TT	--
GG	AG	AA	--	AG	GG	AG	AG	AG	--
GG	AG	AA	AA	AG	GG	AG	AG	AA	--
AA	AG	GG	GG	AG	AA	AG	AG	GG	AA
AA	AC	CC	CC	AC	AA	AC	AC	AC	--
AA	AG	GG	GG	AG	AA	AG	AG	GG	AG
AA	AG	GG	GG	AG	AA	AG	AG	GG	AA
TT	CT	CC	CC	CT	TT	CT	CT	CC	--
GG	CG	CC	CC	CG	GG	CG	CG	CC	GG
AA	AC	CC	CC	AC	AA	AC	AC	AC	--
TT	CT	CC	CC	CT	TT	CT	CT	CC	--
TT	GT	GG	GG	GT	TT	GT	GT	GG	TT
CC	CT	TT	TT	CT	CC	CT	CT	TT	--
CC	CG	GG	GG	CG	CC	CG	CG	GG	CC
TT	AT	AA	AA	AT	TT	AT	AT	AT	--
CC	CT	TT	TT	CC	CC	CT	CT	CT	CT
GG	AG	AA	AA	GG	GG	AG	AG	AG	GG
AA	AG	GG	GG	AA	AA	AG	AG	GG	AG
AA	AT	TT	TT	AA	AA	AT	AT	TT	--
GG	AG	AA	AA	GG	GG	AG	AG	AA	AG
AA	AG	GG	GG	AA	AA	AG	AG	AG	--
CC	CG	GG	GG	CC	CC	CG	CG	GG	CC
AA	AG	GG	GG	AA	AA	AG	AG	GG	AA
GG	CG	CC	CC	GG	GG	CG	CG	CC	CG
GG	AG	AA	AA	GG	GG	AG	AG	AG	GG
AA	AC	CC	CC	AA	AA	AC	AC	AC	AA

GG	AG	AA	AA	GG	GG	AG	AG	AA	--
CC	CG	GG	GG	CC	CC	CG	CG	CG	--
CC	CT	TT	TT	CC	CC	CT	CT	TT	CC
TT	AT	AA	AA	TT	TT	AT	AT	AA	--
CC	CT	TT	TT	CC	CC	CT	CT	CT	CC
AA	AT	TT	TT	AA	AA	AT	AT	TT	AA
TT	AT	AA	AA	TT	TT	AT	AT	AA	AT
GG	CG	CC	CC	GG	GG	CG	CG	CG	GG
CC	CT	TT	TT	CC	CC	CT	CT	TT	CC
AA	AG	GG	GG	AA	AA	AG	AG	GG	AA
AA	AC	CC	CC	AA	AA	AC	AC	CC	AA
TT	GT	GG	GG	TT	TT	GT	GT	GT	TT
CC	CT	TT	TT	CC	CC	CT	CT	TT	--
GG	CG	CC	CC	GG	GG	CG	CG	CG	GG
TT	GT	GG	GG	TT	--	GT	GT	GG	TT
TT	CT	CC	CC	TT	CT	CT	CT	CC	TT
AA	AG	GG	GG	AA	AG	AG	AG	GG	--
AA	AC	CC	--	AA	AC	AC	AC	CC	AA
CC	CT	TT	TT	CC	CT	CT	CT	TT	CC
AA	AC	CC	CC	AA	AC	AC	AC	AC	--
AA	AG	GG	GG	AA	AG	AG	AG	GG	AA
GG	GT	TT	TT	GG	GT	GT	GT	TT	--
GG	GT	TT	TT	GG	GT	GT	GT	TT	--
GG	AG	AA	AA	GG	AG	AG	AG	AG	GG
CC	CT	TT	TT	CC	CT	CT	CT	CT	CC
CC	CT	TT	TT	CC	CT	CT	CT	CT	CC
AA	AG	GG	GG	AA	AG	AG	AG	GG	--
CC	CT	TT	TT	CC	CT	CT	CT	CT	--
TT	CT	CC	CC	TT	CT	CT	CT	CC	TT
GG	AG	AA	AA	GG	AG	AG	AG	AA	GG
CT	CT	TT	TT	CC	CT	CT	CT	TT	CT
--	TT	CT	CT	TT	CT	CT	TT	CT	--
CT	CT	TT	--	CC	CT	CT	CT	TT	CT
AG	AG	GG	AG	AA	AG	AG	AG	GG	--
AA	AC	CC	--	AA	AC	AC	AC	CC	--
CG	CC	CC	CG	CG	CG	CC	CG	CC	CG
GG	TT	TT	GT	GG	GT	GT	GT	TT	--
GG	AA	AA	AG	GG	AA	AG	AG	AA	--
--	CC	CC	CT	TT	CC	CT	CT	CC	TT
AG	GG	GG	AG	AG	GG	GG	AG	GG	AG
GG	AA	AG	AG	GG	AA	AG	AG	AA	--
GG	CC	CG	CG	GG	CC	CG	CG	CG	--
CT	CT	TT	CT	CT	TT	TT	CT	TT	CT
TT	GT	GT	GT	TT	GG	GT	GT	GT	--
AG	GG	AG	GG	AG	GG	GG	AG	AG	--
AG	GG	AG	GG	AG	AG	AG	GG	AG	GG
CC	AA	CC	AA	CC	AA	AC	AC	CC	--

AA	TT	AA	TT	AA	TT	AT	AT	AA	--
AA	TT	AA	TT	AA	TT	AT	AT	AA	TT
TT	AA	TT	AA	AT	AA	AT	AT	AT	--
CC	CT	CC	TT	CT	TT	CT	CT	CC	CT
AG	AG	AA	GG	AG	GG	AG	AG	AG	GG
AC	CC	AC	AA	AC	AA	AC	AC	CC	AA
CT	CC	CT	TT	CT	CT	CT	CT	CT	--
--	AA	AG	GG	AG	AG	AG	AG	AG	GG
GT	TT	GT	GG	GG	GT	GT	GT	GT	GG
AG	AA	AG	AG	GG	AG	AG	AG	AA	--
CG	CG	CG	CC	CC	CC	CC	CG	CG	--
AG	AG	AG	GG	GG	GG	GG	AG	AG	--
CG	GG	CG	CG	CG	CG	CG	CG	GG	CG
AG	GG	AG	AG	AG	AG	AG	AG	GG	AA
AG	AA	AA	AG	AG	AG	AG	AG	AA	GG
AG	GG	GG	AG	GG	AG	AG	AG	GG	AG
GT	GT	GT	GG	GT	GG	GG	GT	GT	--
AG	AA	AA	AG	AA	GG	AG	AG	AA	GG
GT	GT	GT	TT	GT	TT	TT	GT	GT	GT
CG	CG	CC	CG	CC	GG	CG	CG	CC	--
TT	TT	CT	TT	--	TT	CT	TT	CT	--
GG	CG	CC	GG	CC	CG	CG	CG	CC	--
CC	CG	CG	CC	CG	CG	CC	CG	CG	CC
AA	AC	AC	AA	AA	AC	AC	AA	AC	AA
CC	CT	CT	CC	CC	CT	CT	CC	CT	CC
AA	AG	AG	AA	AA	AG	AG	AA	AG	AA
CC	CT	CT	CC	CC	CT	CT	CC	CT	--
GG	CG	GG	GG	CG	CG	GG	CG	CG	--
CC	CT	CT	CT	CC	CT	CT	CC	CT	--
AA	GG	AG	AG	AG	GG	AG	AG	GG	AA
GG	GT	GT	GT	GG	GT	GT	GG	GT	GG
TT	GG	GT	GT	GT	GG	GT	GT	GG	TT
GG	--	CG	CG	GG	CG	CG	GG	CG	--
GG	CC	CG	CG	CG	CC	CG	CG	CC	--
GG	AG	AG	AG	GG	AG	AG	GG	AG	--
TT	CC	CT	CT	CT	CC	CT	CT	--	TT
GT	TT	GT	GT	GT	TT	GT	GT	TT	--
AG	AG	AG	AG	GG	AG	AG	GG	AG	AG
AG	GG	AG	AG	AG	GG	AG	AG	GG	--
--	CC	CT	CT	CT	CC	CT	CT	CC	--
AT	AT	AT	AT	TT	AT	AT	TT	AT	--
GT	GT	GT	GT	TT	GT	GT	TT	GT	TT
CT	TT	CT	CT	CT	TT	CT	CT	TT	--
AG	AG	AG	AG	AA	AG	AG	AA	AG	--
AT	AA	AT	AT	AT	AA	AT	AT	AA	AT
AG	AG	AG	AG	AA	AG	AG	AA	AG	AA
CT	CT	CT	CT	TT	CT	CT	TT	CT	--

AT	AT	AT	AT	TT	AT	AT	TT	AT	TT
GT	TT	GT	GT	GT	TT	GT	GT	TT	GG
AG	GG	AG	GG	GG	GG	AG	AG	GG	AA
CT	TT	CT	TT	TT	TT	CT	CT	CT	--
CT	TT	CT	TT	CT	TT	CT	CT	TT	CC
CT	TT	CT	TT	CT	TT	CT	CT	TT	CT
GT	TT	GT	TT	GT	TT	GT	GT	TT	GG
CG	GG	CG	GG	CG	GG	CG	CG	GG	CC
AC	AC	AA	AA	AC	AA	AC	AC	AA	AC
AG	AG	GG	GG	AG	GG	AG	AG	GG	AA
AG	AG	AA	AA	AG	AA	AG	AG	AA	GG
CC	CC	CC	CT	CT	TT	CT	CT	CC	--
CC	CC	CC	AC	AC	AA	AC	AC	CC	AA
GT	GT	--	TT	TT	TT	GT	TT	GT	GT
CG	CG	CG	CG	CG	GG	GG	CG	CG	--
AA	AG	AA	AA	AG	GG	AG	AG	AA	--
CT	CT	CT	CT	CT	TT	TT	CT	CT	TT
GT	TT	GT	GT	TT	TT	GT	TT	GT	TT
CT	CT	CT	CT	CT	CC	CC	CT	CT	CC
AC	AA	AA	AC	AA	AA	AC	AA	AC	AC
GG	AG	AG	GG	GG	AA	AG	AG	GG	AG
AG	AA	AA	AG	AG	AA	AG	AA	AG	AA
GG	TT	GT	GG	GT	GT	GT	GT	GG	GT
TT	CC	CT	TT	CT	CT	CT	CT	TT	--
CC	AA	AC	CC	AC	CC	AC	AC	CC	AA
AT	TT	AT	AA	TT	AA	AT	AT	AT	TT
--	GT	GT	GT	GT	GG	GT	GG	GG	--
AA	AG	AA	AA	AG	AA	AA	AG	AA	--
CG	CC	CG	CG	CC	GG	CG	CG	CG	CC
AG	AA	AG	AG	AA	GG	AG	AG	GG	--
CC	GG	CG	GG	CC	GG	CG	CG	GG	--
AA	GG	AG	GG	AA	GG	AG	AG	GG	--
AA	AG	AA	AG	AA	AG	AA	AG	AG	--
AA	AT	AT	AT	AA	AT	AT	AA	AT	--
AA	AG	AA	AG	AA	AG	AA	AG	AG	--
AG	AA	AG	AA	AG	AA	AA	AG	AA	--
AA	AC	AC	CC	AA	AC	AC	AC	CC	--
CC	CC	AC	AC	CC	CC	AC	CC	AC	--
AC	AC	AC	AA	CC	AC	AC	AC	AA	--
GT	GT	GT	TT	GG	GT	GT	GT	TT	GG
AT	AT	AT	TT	AA	AT	AT	AT	TT	AA
AA	AA	AG	AG	AA	AA	AG	AA	AG	AA
CC	CC	AC	AC	CC	CC	AC	CC	AC	CC
AT	AT	AT	TT	AA	AT	AT	AT	TT	--
CT	CT	CT	TT	CC	CT	CT	CT	CT	CC
AG	AA	GG	AA	GG	AG	AG	AG	AG	GG
CT	TT	CC	TT	CC	CT	CT	CT	TT	CC

CT	CC	TT	CC	CT	CT	CT	CT	CT	--
--	AA	GG	AA	AG	AG	AG	AG	AG	GG
CT	TT	CC	TT	CT	CT	CT	CT	TT	CC
CT	CC	TT	CC	CT	CT	CT	CT	CC	--
CT	TT	CC	TT	CT	CT	CT	CT	CT	--
AG	AA	GG	AA	AG	AG	AG	AG	AG	--
CT	CC	TT	CC	CT	CT	CT	CT	CT	--
AG	AA	GG	AA	AG	AG	AG	AG	AG	--
CG	GG	CC	GG	CG	CG	CG	CG	GG	CC
AC	CC	AA	CC	AC	AC	AC	AC	AC	AA
CT	TT	CC	TT	CT	CT	CT	CT	CT	CC
CT	TT	CC	TT	CT	CT	CT	CT	CT	CC
AT	AA	TT	AT	AT	AT	AT	AT	AT	--
CT	TT	CC	CT	CT	CT	CT	CT	CT	--
AC	CC	AA	AC	AC	AC	AC	AC	AC	AA
GT	GG	TT	GT	GT	GT	GT	GT	GG	TT
CT	CC	TT	CT	CT	CT	CT	CT	CT	TT
CG	CC	GG	CG	CG	CG	CG	CG	CG	GG
CG	CC	GG	CG	CG	CG	CG	CG	CG	--
AC	AA	CC	AC	AC	AC	AC	AC	AA	--
CG	CC	CG	GG	CG	CG	CG	CG	CG	--
AT	AT	AT	AA	TT	AA	AT	AT	--	AA
AG	GG	AG	--	GG	AA	AG	AG	GG	--
AG	AG	AA	--	GG	AA	AG	AG	AG	AA
AG	AG	GG	GG	AA	AG	AG	AG	AA	--
AG	AG	AA	AA	GG	AG	AG	AG	AG	AA
AC	AC	AA	AA	CC	AC	AC	AC	AC	--
AG	AG	AA	AA	GG	AG	AG	AG	GG	AA
AG	AG	AA	AA	GG	AG	AG	AG	AG	AA
GT	GT	GG	GG	TT	GT	GT	GT	TT	GG
GT	GT	TT	TT	GG	GT	GT	GT	GG	TT
AC	AC	CC	CC	AA	AC	AC	AC	AA	--
CT	CT	TT	TT	CC	CT	CT	CT	CC	TT
CT	CT	TT	TT	CC	CT	CT	CT	CT	--
GT	GT	GG	GG	TT	GT	GT	GT	TT	--
AT	AT	AA	AA	TT	AT	AT	AT	AT	AA
CG	CG	GG	GG	CC	CG	CG	CG	CC	GG
AC	AC	AA	AA	CC	AC	AC	AC	AC	--
CT	CT	CC	--	TT	CT	CT	CT	CT	CC
CG	CG	GG	GG	CC	CG	CG	CG	CC	CG
CT	CT	CC	CC	TT	CT	CT	CT	CT	--
AT	AT	AA	AA	TT	AT	AT	AT	AT	--
CT	CT	TT	TT	CC	CT	CT	CT	CC	--
AG	AG	GG	GG	AA	AG	AG	AG	AG	--
AT	AT	AA	AA	TT	AT	AT	AT	TT	--
AT	AT	AA	AA	TT	AT	AT	AT	TT	--
AC	AC	AA	AA	CC	AC	AC	AC	AC	AA

GT	GT	TT	TT	GG	GT	GT	GT	GG	TT
AG	AG	GG	GG	AA	AG	AG	AG	AG	--
GT	GT	GG	GG	TT	GT	GT	GT	TT	--
AG	AG	AA	AA	GG	AG	AG	AG	AG	--
CT	CT	TT	TT	CC	CT	CT	CT	CT	--
AG	AG	AA	AA	GG	AG	AG	AG	AG	--
CT	CT	TT	TT	CC	CT	CT	CT	CC	--
AC	AC	CC	CC	AA	AC	AC	AC	AA	--
AG	AG	AA	AA	GG	AG	AG	AG	GG	--
CT	CT	TT	CT	CC	CT	CT	CT	CC	TT
AA	AG	GG	AG	AA	AG	AG	AG	AG	--
TT	CT	CT	CT	TT	TT	CT	CT	CT	--
GG	GT	GT	GG	GG	GT	GG	GT	GT	--
AG	GG	GG	AG	AG	AG	AG	GG	AG	--
TT	AT	AT	AT	AT	AA	AT	AT	AT	--
TT	TT	AT	AT	AT	AA	AT	AT	AA	--
CC	CC	AC	AC	AC	AA	AC	AC	AA	--
GG	GG	GT	GT	GT	TT	GT	GT	TT	GG
TT	CT	TT	CT	CT	TT	TT	CT	TT	--
GG	AA	AG	AA	AA	AG	AG	AG	GG	AA
CG	GG	GG	GG	GG	GG	CG	GG	CG	--
CT	TT	TT	TT	TT	TT	CT	TT	CT	--
GG	GT	GT	GG	GG	GT	GG	GT	GT	GG
AG	GG	AG	AA	AA	AG	AG	AG	GG	--
AC	AC	AA	AA	AA	AA	AC	AA	AC	--
CG	CG	GG	GG	GG	GG	CG	GG	CG	--
GT	GT	TT	TT	TT	TT	GT	TT	GT	TT
CT	TT	TT	CC	CC	CT	CT	CT	TT	CC
AA	AG	AA	AG	AG	GG	AG	AG	AG	--
CT	CT	CT	CC	CC	CC	CT	CC	CT	--
CC	CC	CC	CG	CG	CG	CG	CC	CC	--
AC	AA	AC	AC	AC	AA	AA	AC	AA	--
AG	AA	AG	GG	GG	AG	AG	AG	AA	--
TT	CT	TT	TT	TT	CT	TT	CT	CT	--
CG	CG	CG	GG	GG	GG	CG	GG	CG	GG
AG	GG	AG	AA	AA	AG	AG	AG	GG	AA
GG	GG	GG	CG	CG	CG	CG	GG	GG	--
CG	GG	CG	CC	CC	CG	CG	CG	GG	--
CT	TT	CT	CC	CC	CT	CT	CT	TT	--
AG	AA	AG	GG	GG	AG	AG	AG	AA	GG
AG	GG	AG	AG	AG	GG	GG	AG	GG	AG
CT	TT	CT	CC	CC	CT	CT	CT	TT	CC
GG	AG	GG	GG	GG	AG	GG	AG	AG	GG
AG	AA	AG	GG	GG	AG	AG	AG	AA	GG
AC	AA	AC	CC	CC	AC	AC	AC	AA	CC
AT	AT	AT	TT	TT	TT	AT	TT	AT	TT
AG	GG	AG	AA	AA	AG	AG	AG	GG	--

AC	CC	AC	AA	AA	AC	AC	AC	CC	AA
AG	GG	AG	AA	AA	AG	AG	AG	GG	--
AA	AG	AA	AA	AA	AG	AA	AG	AG	--
CT	CT	CT	CC	CC	CC	CT	CC	CT	--
CG	CG	CG	CC	CC	CC	CG	CC	CG	CC
CT	TT	CT	TT	TT	TT	CT	TT	CT	--
CG	CG	GG	CG	CG	--	CG	GG	CG	CG
CC	CC	CT	CC	CC	CC	CT	CC	CT	CC
GT	GT	GG	GT	GT	GT	GT	GG	GG	--

Supplementary Table 2: Original morphological measurements for all F2 individuals.

Family	ID	Sex	CS	PC1	PC2	PC3	PC4
1	1	F	6.375759862	-0.014678551	-0.031501780	0.015585872	-0.007632606
1	2	F	6.186264470	-0.020676515	0.000620000	0.015270307	0.008017977
1	3	M	6.177851657	0.012247254	-0.019821264	0.000576000	-0.005986272
1	4	F	6.154091864	0.014436606	-0.001585171	-0.004937207	-0.005726766
1	5	M	5.820769369	0.008731976	-0.011687610	-0.001759741	0.001720826
1	6	M	6.950851320	-0.001465295	-0.020240951	0.006007078	-0.013680659
1	7	F	7.611645894	-0.052022483	-0.014561625	-0.021319813	0.033035633
1	8	M	7.397594823	-0.022409060	-0.029795230	-0.000376000	0.012966330
1	12	M	6.429423457	0.005930965	-0.010230111	-0.006619265	-0.001849925
1	14	F	7.097030860	-0.025975617	-0.026148405	0.015195338	0.014426432
1	15	M	6.874550355	-0.006022666	-0.015837599	-0.014364147	0.005580124
1	16	F	6.403783910	-0.039778088	-0.039861456	-0.028071741	-0.004636067
1	17	F	6.584191740	-0.005322625	0.017235346	0.006442456	0.010578155
1	20	F	6.222314907	-0.048036624	-0.026522721	-0.001052860	0.005604895
1	23	M	6.408937024	0.002697016	-0.011981027	-0.000464000	-0.014210797
1	24	M	6.114906684	-0.005536539	-0.008196195	-0.008902676	-0.005088441
1	25	M	6.171601624	0.002643717	-0.019278739	-0.002681257	-0.021518807
1	28	F	5.652540863	0.019686424	-0.050129238	-0.011057232	-0.015223153
1	29	M	5.412792945	0.030146344	-0.037695143	-0.003375490	0.014636445
1	31	F	6.165653819	-0.016572708	-0.008264523	0.003662301	-0.002659895
1	32	M	5.779694375	-0.004681651	-0.010219508	-0.008794066	0.011872656
1	33	F	5.206326046	0.003421167	-0.003924480	0.006513738	0.004686051
1	35	M	6.114327126	-0.004070603	0.002085847	0.009296835	-0.010188036
1	37	M	6.917479963	-0.024178016	0.004678827	-0.010056482	0.000186000
1	40	F	6.746959992	-0.030108578	-0.001105708	0.003294360	-0.002932716
1	43	F	7.769051263	-0.018360944	0.026474965	0.009719966	-0.005466747
1	45	F	7.118959891	-0.033591919	-0.025063000	-0.004476710	-0.009859931
1	46	M	6.209034431	-0.004679623	0.002656290	-0.003665387	-0.015401837
1	47	F	6.493294936	-0.013105068	0.006637618	-0.004568397	0.006448216
1	48	F	6.316753846	-0.012703409	-0.011842858	0.005269888	-0.012383362
1	55	F	6.716662973	-0.001570433	0.006405026	0.005717889	0.003219552
1	56	F	7.474011288	-0.046313703	-0.006198679	-0.009468968	0.019614631
1	62	F	5.967631527	-0.027555133	-0.001363537	-0.012630450	0.002020942
1	63	F	6.657208093	-0.010457651	0.002709973	0.009777448	-0.010251424
1	65	F	6.520972308	-0.009114101	0.007239181	0.006116709	0.017529893
1	66	F	7.194091109	-0.024036030	-0.011133109	0.006048072	0.012012257
1	70	M	6.563289089	-0.021000434	0.009642759	-0.028429419	0.018407956
1	73	F	7.709410133	-0.026004105	0.005346949	0.000399000	-0.008671184
1	74	M	6.663605897	-0.013095878	0.001209540	-0.031669133	-0.000142000
1	79	F	6.655288503	-0.021558533	-0.002451338	0.011580670	-0.004367364
1	81	F	7.083408074	-0.029354556	0.003136732	0.013155754	0.001211602
1	84	M	6.740605845	0.002284824	-0.001609709	-0.010895636	-0.011633842
1	88	F	7.013297463	-0.029105620	-0.001526470	0.004815109	-0.007837423

1	89	F	5.901841214	0.013912595	0.031686828	0.009857604	0.002221516
1	91	M	6.266475356	0.020446020	0.007233271	0.006616487	-0.016199814
1	92	F	6.547914090	-0.014668597	0.007371925	0.006507336	-0.012628638
1	93	M	5.801901118	0.032931514	-0.001855556	-0.001171274	0.005815817
1	94	M	6.766839859	0.002115488	0.005591281	-0.009398039	-0.001109955
1	98	F	6.348529713	-0.031528335	-0.007094103	-0.001228611	0.002854601
1	99	F	6.566720749	-0.021900098	0.000233000	-0.004064989	-0.004055837
1	100	F	6.334608811	-0.018644940	0.003942414	0.006140172	0.016921987
1	101	M	6.381144002	0.002052623	-0.013201992	-0.006471301	-0.005680285
1	103	M	5.726627197	-0.000146000	-0.014361273	-0.022348382	0.014302108
1	104	M	6.247424436	0.025901893	0.009819496	0.001142938	0.010133422
1	106	F	6.667970338	-0.005431119	-0.016629421	0.010426163	-0.003719064
1	108	M	5.856701631	0.009139530	-0.011842767	-0.007813995	0.006139161
1	110	M	5.895094854	0.013302603	-0.005194693	0.005447785	0.000304000
2	3	M	5.982324013	0.002181185	0.005118155	0.003147084	0.000580000
2	4	M	6.205926209	-0.025077544	-0.021321650	0.006388909	0.022060267
2	6	F	6.011483586	-0.006279443	0.014196562	-0.020510827	0.004349199
2	9	M	6.175588647	0.004101663	0.004297583	-0.016294066	-0.011354684
2	13	F	5.843397214	-0.009625428	-0.011473846	0.006111609	-0.014923386
2	14	F	6.747460204	-0.036790852	0.007416533	-0.002804530	-0.005822709
2	15	F	6.061319601	-0.014712466	0.012440992	0.002730985	-0.002512910
2	18	M	6.572375621	0.011961051	-0.003194945	-0.005696843	0.000177000
2	27	F	6.615685123	-0.004111896	-0.000004780	0.014816509	0.008913496
2	28	F	6.570648559	-0.015349143	-0.003776179	-0.000633000	-0.007576239
2	29	F	5.915030876	0.004077407	0.004771349	-0.008991821	0.006210564
2	30	F	6.674969086	-0.023222202	0.001720884	0.013343507	-0.005593845
2	31	F	6.442098922	-0.004957603	-0.025000141	-0.003683958	-0.020604495
2	33	M	5.919867050	0.002547361	0.003628662	-0.002005913	-0.013050740
2	36	F	5.870166528	0.027713818	-0.007062441	0.012494546	0.010128107
2	37	M	6.485430724	0.008178082	0.019424497	-0.015869256	-0.008802574
2	41	F	7.406953322	-0.027718871	0.009501751	0.000286000	-0.000994000
2	42	F	7.367651409	-0.028041477	0.008405559	0.006674377	-0.005007357
2	43	F	7.100067780	0.010837689	0.018176914	0.017714758	0.001378451
2	46	M	5.962570975	0.007631430	0.006392015	-0.001679048	0.013949305
2	47	M	6.108566918	-0.015401576	0.012990800	-0.015668131	-0.005862867
2	48	M	6.015111046	-0.016613486	0.003480762	-0.004974649	-0.005421634
2	50	F	6.028541414	-0.009101384	0.005078884	0.000174000	-0.009543565
2	51	M	6.539773932	-0.018516122	0.016247865	-0.017144917	-0.002053313
2	52	M	6.703440952	0.008381953	0.000474000	-0.008416020	-0.010868542
2	53	F	7.187796594	-0.010849982	0.006270084	0.006481182	0.014452253
2	54	F	6.609973589	0.006536639	0.010764946	0.010946721	0.015378469
2	57	M	5.818571102	0.013104888	0.004253819	-0.003217327	0.010680431
2	58	M	5.678614124	0.016579499	-0.001107186	-0.003281800	-0.012012533
2	59	M	5.943892820	-0.000432000	0.017052769	-0.008416461	-0.008083524
2	61	M	6.305980054	0.029870876	0.016793432	-0.001421320	-0.002813057
2	62	F	5.775393567	0.021849272	0.002821099	0.011066893	0.001260076
2	63	F	6.187632345	-0.004303631	-0.006750708	0.013273301	0.012742945
2	64	F	5.983242772	-0.019023603	-0.012262700	-0.019733767	-0.011971395

2	65	M	6.202315046	-0.000145000	0.000083500	-0.001292061	0.003168066
2	68	M	6.018134842	-0.016379856	0.004571808	-0.011838847	-0.001297688
2	73	M	6.863859009	0.019251966	0.001863665	-0.000897000	0.000318000
2	75	M	6.312483762	0.001435587	0.001234423	-0.009285468	0.005286404
2	76	M	5.655467209	0.021273957	-0.002037452	-0.013395161	-0.008435498
2	78	M	6.464976229	-0.005315830	0.003649498	-0.011677290	-0.010381765
2	80	M	5.571265291	0.040005466	0.023701320	-0.002785068	-0.000426000
2	81	F	7.497562446	-0.034675567	0.022343958	-0.007888642	-0.011313293
2	82	M	6.162968305	0.022020602	0.009985553	-0.017673671	0.003948600
2	84	M	6.172419793	0.002604069	0.011035822	-0.028559398	0.007271983
3	4	F	6.459747518	-0.010277085	0.007269856	0.007429359	-0.012994313
3	5	F	5.953702245	-0.020247519	-0.033169128	0.024233637	0.005433318
3	8	M	6.219471023	-0.001034888	0.004400435	-0.010927499	0.000909000
3	10	F	6.116958567	-0.023405292	0.012809563	-0.002872463	-0.007898538
3	14	F	7.375565369	-0.012266271	-0.004905709	0.016004046	0.001546033
3	16	M	5.948641895	-0.003147237	0.018146128	-0.012328898	-0.008307910
3	17	M	6.242176188	0.037547327	0.000981000	0.003317721	-0.000847000
3	18	M	5.723748441	0.020029954	0.021879356	-0.004639637	-0.007039112
3	23	M	5.488254120	0.024224059	-0.004487273	-0.012384198	0.002130762
3	25	M	6.688158983	-0.007754424	0.002321314	-0.009955258	-0.007612845
3	26	F	5.991518589	0.011656305	0.012187372	0.014385781	-0.004564636
3	29	M	6.103031367	-0.005710384	0.007947641	-0.004734620	-0.007190665
3	31	M	5.725124121	0.008101098	-0.003521498	-0.009547050	-0.012657311
3	32	F	5.891979523	0.029728760	0.009638223	0.025688902	-0.008211493
3	33	M	5.542216103	0.010709576	-0.004003352	-0.003989117	-0.001006896
3	36	M	5.804259047	0.031541445	0.005350438	0.001469058	0.016087032
3	37	M	6.319804246	0.002290394	-0.009321736	-0.009863456	-0.005934602
3	38	F	5.629524248	-0.015963910	-0.010189133	-0.002356713	0.013753752
3	40	F	6.945049176	-0.026536344	0.008901159	-0.007841107	-0.005758714
3	41	F	6.650303320	-0.021752704	-0.010938504	-0.000696000	0.017535553
3	44	F	7.080462849	-0.019305614	-0.005782755	-0.009705337	0.022473013
3	45	F	6.410768490	0.015361284	0.003998967	0.016580782	-0.003923726
3	46	M	5.702035640	0.015291118	0.021875020	-0.007970155	-0.014655574
3	47	F	6.230572581	-0.003302384	-0.000675000	0.018741518	-0.010525184
3	48	F	5.736360761	0.045627992	0.004836550	-0.005283426	-0.015887900
3	49	F	6.772149125	-0.021093998	0.021255420	-0.002561005	-0.003232803
3	50	M	5.783121543	0.000270000	0.012035393	-0.018907717	-0.005793886
3	52	F	6.957185483	-0.019800515	0.001751739	-0.002174456	-0.011481192
3	54	F	7.219892091	-0.023915700	-0.008634367	-0.003162077	-0.001891108
3	56	M	5.213008903	0.063862432	-0.030834036	-0.033155375	-0.005937391
3	57	M	6.110917294	0.010744245	-0.003000474	-0.013205759	-0.009105726
3	59	M	6.261816965	-0.010371812	0.025395199	-0.025561630	-0.007849482
3	63	M	6.145941410	-0.003477067	0.001278302	-0.006368838	-0.007249136
3	66	F	6.314199439	-0.032361711	0.013741216	-0.001282126	-0.010608220
3	67	F	6.926786715	-0.005660820	-0.013631219	0.015836429	-0.001522413
3	68	M	6.174481056	0.011911660	0.016714056	-0.006245062	0.002196504
3	69	M	6.293643121	-0.005985384	-0.018720096	-0.006196457	0.000712000
3	70	F	6.146995943	0.030376373	0.006910789	0.019673591	-0.003866841

3	71	M	6.268515564	0.002204770	−0.003820246	−0.003692415	0.005260538
3	75	F	6.992541629	−0.018071490	0.026580595	0.015346414	−0.013271417
3	78	M	5.767935746	0.025485821	0.034326143	−0.005899903	0.005823203
4	4	F	5.662065882	−0.005103102	−0.003648859	0.010934002	−0.016421616
4	5	M	6.417989865	0.001980282	−0.005054490	0.000144000	−0.026294456
4	6	F	6.677576678	−0.005064002	0.000584000	0.008745693	−0.013584516
4	7	M	5.428033438	0.032609775	−0.011581073	−0.012474356	−0.010607207
4	8	F	6.212900610	−0.024636902	−0.009956267	0.008295009	−0.010318153
4	11	M	5.966924704	0.011934206	0.009876294	−0.011265567	0.002059430
4	12	M	6.104254060	0.005733496	−0.008711307	−0.006561603	0.000486000
4	13	F	6.616911438	−0.029200270	−0.004315841	0.004129367	−0.004883479
4	15	F	6.624787272	−0.015384417	−0.026194858	0.007534188	−0.017203785
4	16	F	5.971852059	0.003432619	−0.002746431	0.010817655	0.006794410
4	18	M	6.427026184	0.018673194	−0.011100137	0.002824757	−0.005946887
4	24	M	6.109094803	0.029567310	0.002118448	−0.011013841	−0.006464831
4	25	F	6.594283496	−0.006795481	−0.002449576	0.019219448	0.001101050
4	27	F	6.469839492	−0.016791977	0.003267611	0.013022096	0.005161690
4	29	F	6.988372813	−0.020910642	0.015786811	−0.001377380	−0.005143767
4	31	M	6.552880566	−0.002184322	−0.000230000	−0.004127953	−0.010545605
4	34	M	6.477644734	0.003320240	−0.003644923	−0.005481242	−0.000975000
4	35	F	6.457045681	−0.014745658	0.010167216	0.014497969	−0.003016084
4	39	F	7.362534507	−0.023769776	0.015049194	0.018637858	−0.004505570
4	40	F	6.665156543	−0.001070580	−0.005873018	0.024027307	−0.007871181
4	43	F	5.781439284	0.030980595	−0.000245000	−0.010361287	0.011391441
4	45	M	7.653542394	−0.012443921	0.002693113	0.013910476	−0.005867618
4	50	M	6.869651200	−0.007251160	0.004288014	−0.012308842	−0.009453840
4	51	F	7.139791124	−0.035604752	−0.003548577	0.012454479	−0.001190196
4	53	M	5.931120215	0.008793473	0.017494076	−0.015642128	−0.012247524
4	54	M	6.426151737	0.017572236	−0.000587000	0.007701245	−0.008060517
4	56	M	6.282148671	0.031744845	0.014138987	−0.001048103	0.002589919
4	57	F	6.511175945	−0.023161392	−0.000181000	0.005092831	0.006756473
4	59	F	7.657919152	−0.019565404	0.007425726	0.010527923	−0.003554550
4	60	M	6.044595243	0.003490176	−0.022453026	0.001629748	0.011938252
4	62	M	6.171634992	0.011031330	−0.007673704	0.001038699	−0.022003356
4	67	M	5.769602146	0.030188882	0.012193281	−0.011199456	−0.001418979
4	72	F	6.488973822	−0.011014186	0.026262809	0.018557529	0.004439098
5	1	F	6.004607987	−0.020139542	−0.002857436	0.000143000	−0.004886284
5	3	F	6.180134603	0.000470000	0.006465386	0.023114543	−0.007193386
5	4	F	5.913031345	0.004942777	−0.000151000	0.014055018	−0.002297279
5	5	M	6.052725155	0.009525140	0.009951780	−0.016760157	0.013722366
5	6	M	6.285952983	0.003161356	0.010412028	−0.019010988	0.015329650
5	7	F	4.822696938	0.059437241	−0.051684887	0.004682534	−0.016796479
5	8	M	6.294948671	0.058588213	0.016679792	0.004533474	0.000815000
5	10	F	6.066708270	0.023389954	0.020166748	0.012856847	−0.002904132
5	11	M	6.516978438	0.006877718	0.007653020	−0.008538284	0.009969291
5	13	M	5.999524946	−0.006711553	−0.013820956	−0.020064400	0.024613053
5	20	M	6.122131808	−0.008650043	−0.002091466	−0.006892413	−0.014780794
5	22	F	6.087504056	−0.004786764	−0.008981663	0.012276709	−0.002990038

5	23	M	6.008482760	0.012118139	0.002059213	−0.011627933	−0.004824965
5	24	M	5.939064619	0.007798105	0.000534000	−0.007262355	0.002251062
5	25	M	6.104451139	0.023047185	0.018302067	0.006056623	−0.000838000
5	28	F	7.482055097	−0.025445599	0.001887423	0.009998563	−0.006074745
5	30	M	5.841989569	0.019807147	0.011355742	0.003320961	0.011587438
5	32	F	6.584168309	−0.000136000	0.018573482	0.001617168	−0.013906859
5	35	M	5.141504209	0.031287478	0.019875360	−0.021218089	0.020265295
5	38	M	5.808426585	0.006073241	−0.005777342	0.005620181	−0.010966598
5	39	M	5.713015054	0.027293835	0.003694715	0.004652611	−0.008729443
5	40	M	6.164858740	0.013120492	0.015283782	−0.011072422	0.009725300
5	41	F	6.366685676	−0.013148896	−0.007311268	0.015497381	−0.004472853
5	43	F	5.543664112	0.016090151	0.006039806	−0.002293335	0.002481644
5	44	F	6.204734311	−0.042195791	−0.000404000	−0.015600303	−0.011278729
5	45	F	4.950015463	0.039263361	−0.063558170	−0.007293665	−0.009104964
5	47	M	6.649135430	0.041435573	0.009013137	−0.007058273	0.004805018
5	48	M	6.974663280	0.009255658	0.032144819	−0.005013000	0.000170000
5	52	F	6.706486989	−0.004882142	0.008471296	0.013311151	−0.001099500
5	53	M	6.336529184	−0.014121709	−0.013975709	−0.002196228	0.001322064
5	55	M	6.294548754	0.014244836	−0.002734062	−0.000901000	0.010571171
5	56	F	6.564001809	0.005213531	−0.002986019	0.016493307	0.007262129
5	58	M	5.810995809	−0.007974266	−0.005990164	0.000552000	−0.003530390
5	63	M	5.937154737	−0.003130240	0.000208000	−0.012300699	0.000052400
5	64	M	5.499855428	0.017894918	−0.016615498	0.001980503	−0.006168269
5	66	M	6.191774462	0.017124035	0.002230992	0.003046948	−0.008328059
5	68	M	6.024865676	0.025550201	−0.002451357	−0.015166318	0.002988554
5	69	M	6.067803749	0.012104983	0.015349924	−0.020989464	−0.000697000
6	5	F	5.567110845	0.046995788	0.019293694	0.011848074	−0.003027434
6	6	M	5.509678424	0.027621838	0.003916239	−0.009033151	0.016497803
6	9	M	5.912107097	0.019544165	0.009394464	−0.007091289	−0.003302930
6	10	F	5.227447731	0.024619576	−0.024213395	−0.002828402	0.017365930
6	11	M	6.273802080	0.022941313	−0.004096726	0.008714881	0.001540456
6	13	F	5.736062146	−0.022545901	−0.023903193	0.001791771	0.000072100
6	14	F	5.285412330	0.017944099	−0.001835736	−0.000398000	0.000796000
6	15	F	6.160313966	0.002548566	0.014844288	0.015183621	−0.002153830
6	18	F	6.006988832	0.001485526	0.003819440	0.017779548	0.006127556
6	20	M	5.959175510	0.028705126	0.007221711	−0.003483753	0.007134031
6	21	M	7.427984823	0.000168000	0.006455292	0.002209257	−0.017448316
6	23	M	7.230453001	−0.007767409	−0.005467448	0.008010875	0.000935000
6	27	F	5.706026256	0.031767609	0.007848693	0.023986049	0.024823361
6	28	F	7.058317514	−0.012651013	0.002224847	0.007918560	0.009026506
6	30	M	6.725344618	−0.021945203	−0.019229544	−0.011535395	−0.005291987
6	32	M	6.375457072	−0.006350005	−0.010485221	−0.014876400	0.010487011
6	33	F	6.551517782	0.005666675	−0.002903488	0.020217988	0.002585410
6	34	M	7.066085803	−0.006804837	0.003239639	−0.005956735	−0.009845232
6	35	M	6.619464430	0.013470253	0.000413000	0.003519970	0.012311052
6	36	M	5.474552719	0.056446116	0.001524480	−0.001426409	0.026396072
6	38	F	6.468685272	−0.008016028	0.015869513	0.002103858	0.014126037
6	39	F	6.645301182	0.003391190	0.011125218	0.005276128	0.017893069

6	40	M	5.713842809	0.021927879	-0.014665797	-0.010068855	0.020924197
6	45	F	6.200927660	0.014224760	-0.011194896	0.022793431	0.013570077
6	49	M	6.007955330	0.036034101	0.003015038	0.006493424	0.008774856
6	50	F	6.378676959	-0.018494782	-0.013623730	0.001876037	0.017293815
6	51	F	6.705787530	-0.019228943	0.013152316	0.004843728	-0.002418112
6	52	F	6.854176884	-0.020344916	0.001479731	0.018422979	0.005193733
6	54	M	5.945880571	0.011789520	0.007586357	-0.005715158	-0.005938942
6	57	M	5.656016492	0.009877472	-0.001564020	-0.013733421	-0.001454939
6	62	F	5.771203049	-0.008478477	0.002335779	0.006552368	0.007785899
7	1	F	5.311458074	0.027299190	-0.013843398	0.011829891	0.000207000
7	2	M	5.544770545	0.020864410	-0.015989321	0.005326572	0.003077660
7	3	F	5.047470000	-0.023994929	-0.031536674	-0.015504644	0.006617288
7	4	F	5.643498694	-0.036254024	-0.000633000	0.006745530	0.001403472
7	8	F	6.050022024	-0.004189785	-0.003493448	0.023155174	-0.000105000
7	9	F	5.371620693	0.048017259	-0.018755898	0.035719592	0.006866591
7	10	F	6.242587400	0.002384524	0.004323457	0.010369827	0.012282256
7	11	M	5.800035571	-0.009304883	0.016874319	-0.018682997	-0.008555233
7	12	M	5.755827994	-0.005329075	0.003799270	-0.000021900	-0.010917650
7	13	F	6.269446914	-0.020501144	0.004215946	0.009307584	0.015169765
7	14	F	5.882072981	0.000090100	-0.002290342	0.009519789	-0.004069076
7	15	M	5.075149719	0.063986010	-0.024711160	-0.011738533	-0.016269527
7	16	M	6.716263328	-0.008036241	-0.002565667	-0.002664351	-0.000080700
7	20	F	6.157897835	0.025492421	0.018800189	0.012640653	0.009204515
7	21	M	6.333982315	-0.003497477	-0.003854221	-0.009083217	0.003456625
7	23	M	6.273068039	0.028604143	-0.007857195	-0.000330000	0.000046700
7	26	F	6.029308695	0.017653945	-0.009816301	0.014465168	-0.008726084
7	30	F	6.930222811	-0.015629212	0.007260622	0.006300612	0.001150811
7	31	F	6.756885997	-0.035242793	0.000285000	0.004359526	0.010540367
7	32	F	6.298411783	-0.024101723	0.021085862	0.004846803	0.000781000
7	33	M	6.701492989	0.000369000	0.010975568	-0.012958608	0.005955688
7	35	F	7.001391017	-0.024518432	0.022252826	-0.006946959	0.017020644
7	39	F	6.333505164	-0.013131603	0.043121264	-0.005494904	0.012235802
7	43	F	6.887453443	-0.036874987	-0.000574000	0.002878291	0.021050234
7	46	M	6.046996525	-0.008719721	0.010644162	-0.016739302	0.000798000
7	47	F	5.176840341	0.003106084	-0.004096805	0.009049697	0.017946138
7	48	F	5.716515365	-0.012557802	-0.001059131	-0.005175577	-0.001513674
7	51	M	5.688384529	0.010408181	0.014176620	-0.002271981	-0.008143082
7	53	F	5.642804476	0.015415792	-0.009388433	0.017472008	0.012071477

PC5	PC6	PC7	PC8	PC9
-0.001750412	0.003538749	0.002917953	0.007978889	0.003057161
0.005223916	-0.000538000	0.007314102	0.013137734	-0.004774249
-0.000755000	0.010929943	0.008640798	0.001966355	0.004508456
0.018019790	0.001587243	0.000181000	-0.012401372	0.000038900
-0.003147655	0.002612456	-0.007972339	0.012453428	-0.014027205
-0.007778271	0.003222541	-0.006127631	0.000281000	-0.001384993
0.005333378	-0.010182465	0.010476987	-0.006699034	0.006804154
0.009884887	0.011300531	-0.013664069	0.006873199	0.003325618
-0.002226761	-0.009009946	-0.007086090	0.002292624	-0.009576216
-0.004017615	-0.003833956	0.000619000	0.003919845	0.003130707
0.008135856	0.010066499	-0.004123714	0.011884013	-0.010993027
0.009618992	0.013720215	-0.013181051	0.011144903	0.000648000
0.015920396	-0.006964681	-0.004574371	0.004267790	-0.003979423
0.015179766	0.006654118	0.010654342	-0.003214325	-0.000321000
-0.006613251	0.008306232	0.000867000	0.001571114	-0.000185000
-0.005621591	0.009863395	0.001411280	0.001670733	-0.002025261
-0.004567547	-0.000735000	-0.003735310	-0.002545988	-0.006957481
-0.005631192	-0.004919748	0.002407961	0.006000432	-0.000201000
0.004489140	0.008899793	-0.003699717	-0.006547926	0.025219635
0.007942108	-0.003096153	0.005602353	-0.000437000	0.000406000
-0.000107000	-0.008644757	0.005517863	0.016938862	0.004087889
0.010382478	-0.004068723	0.003389269	-0.000285000	-0.004256386
-0.011342473	0.001476026	0.001731524	-0.000807000	-0.000954000
0.005022965	-0.012103465	-0.002223024	-0.009646893	-0.000424000
-0.000677000	0.003995328	0.001907374	0.000907000	-0.007588650
-0.000757000	-0.001765413	0.007202532	0.005416479	-0.000758000
0.004511218	0.003250928	-0.004988877	-0.000536000	-0.006368222
-0.011773386	-0.007032269	-0.004583143	-0.000193000	-0.013591818
-0.011868817	-0.006624856	-0.001343934	-0.001926067	-0.003441830
-0.004551662	0.001848116	0.005066776	0.003234901	-0.001364330
-0.008619329	-0.008835757	0.004475609	-0.006363419	0.011243738
0.004851997	0.002635428	-0.001072672	-0.000642000	-0.000588000
0.013076804	0.000323000	-0.008230875	0.010348467	-0.013297082
-0.007435142	0.003425826	-0.005262388	0.004914161	-0.002783650
-0.030109734	-0.024704321	0.010701408	-0.000094000	0.000501000
-0.013627699	-0.004755190	-0.005448775	-0.001087838	-0.003239470
-0.003186427	0.006659659	-0.004662398	-0.007534954	-0.010919215
0.009194049	-0.004373253	-0.003200802	0.003588472	0.003668194
0.009152285	0.013198710	-0.009389073	0.002784114	-0.003638919
0.006771887	0.003190290	0.008461272	-0.006735744	0.004550932
-0.002574290	-0.002522617	-0.012239136	-0.003323469	-0.001529777
-0.006991134	0.009430970	-0.012049952	-0.003039688	-0.002079443
0.003853532	0.003283621	0.006506846	-0.011105803	0.008409082

0.008095173	0.003599056	0.003563896	-0.008629752	-0.005411379
-0.008223297	0.009628332	0.006485702	-0.003988894	0.003258232
0.001101274	-0.004175785	0.008413955	-0.000613000	-0.001785865
-0.009650427	0.004783834	0.007218603	-0.003698686	-0.004944059
-0.013322035	0.002523618	-0.002586726	-0.000350000	0.006315255
0.001251536	-0.003230444	-0.002891727	0.004163300	-0.008697009
0.001296680	-0.000730000	0.003437866	0.003153366	-0.003921255
0.004222380	0.001039200	0.005724012	-0.003981244	0.001665191
-0.001530505	0.003960773	0.001852832	-0.000054100	-0.003982891
-0.001499779	-0.001235773	-0.007131452	0.014507487	-0.013274769
-0.003243109	0.001212262	-0.002508823	0.005497175	0.007197445
-0.014473439	-0.004018906	0.004889139	0.001026684	-0.005855334
-0.001993154	-0.008258362	0.002657512	-0.004954737	0.001223177
0.004883882	0.003734335	0.008109053	0.004147874	0.002230101
-0.016038144	0.003776771	0.006147954	0.000746000	0.006171241
0.001330237	0.004578812	0.007911134	-0.013378959	0.019558149
0.003791179	-0.000741000	0.009805191	0.002933939	0.008578636
0.004613578	0.003223478	-0.002954705	0.004028467	0.003954371
-0.001543649	-0.006976206	-0.002374654	-0.004008060	0.003873460
0.007076913	-0.008870216	-0.008904490	0.002761770	-0.001565817
0.005289107	0.001584404	0.005402624	-0.000628000	-0.006314648
-0.010461903	-0.006725122	-0.001455625	-0.001159785	-0.000651000
-0.003679980	-0.006496889	-0.004443345	-0.002009670	-0.002841999
0.006075896	-0.004373604	-0.004496474	-0.000894000	-0.000772000
-0.003347057	-0.000721000	0.004498307	0.007869723	-0.002135209
0.009097445	-0.011494461	-0.005318222	0.007079740	-0.002281205
-0.009324221	0.008811388	0.008963631	-0.000479000	-0.000743000
0.004042852	-0.000975000	-0.003912716	0.006197246	-0.001433511
0.005796688	0.006840247	0.007278275	-0.003215071	-0.007435372
0.001412453	-0.008358666	-0.004348005	-0.001806467	0.008854394
-0.004274760	0.003699196	-0.001912578	0.002879714	-0.009057983
-0.003445758	0.006633487	-0.006324345	0.002747282	-0.001204877
-0.002416187	0.011097869	-0.005462004	0.008720910	-0.005150900
0.004055974	0.005636952	0.003947923	0.009915427	0.004957402
-0.004693282	0.017013313	0.003892734	0.011067854	0.003166071
-0.012215384	0.012446900	0.011845032	-0.006558239	-0.001883257
-0.001068365	0.009537876	0.007634566	0.003688218	0.001801851
-0.002525325	-0.005776473	-0.000229000	0.005413849	-0.000092900
-0.008262595	0.008704894	-0.007320932	-0.002596318	0.001856631
-0.005589631	0.002098636	-0.006653054	0.008750973	-0.005618865
-0.000526000	0.010356964	0.000067900	0.004896748	0.000438000
0.004187595	-0.004938318	0.004666640	0.015664883	0.004120960
-0.003890702	-0.004943100	0.005850211	0.002082045	0.004710107
-0.001600790	-0.001625104	0.001899347	0.002387472	-0.007296303
0.005580134	0.015399774	-0.000274000	0.001163653	0.002005784
0.013079354	0.002793587	0.001780144	-0.000220000	-0.003411572
-0.007536476	0.007085570	0.006268300	0.011621253	-0.006668938
-0.005175362	0.002950178	0.020849287	-0.000277000	0.001272446

-0.009703925	0.004985509	0.006500150	0.003770542	0.001426293
-0.011696748	-0.005797085	-0.010608636	-0.007456475	-0.004214059
-0.009139661	0.009893540	-0.008286673	0.003933525	0.011400225
-0.004965979	0.007715092	0.000371000	0.000695000	-0.005644049
0.006478323	-0.000330000	0.007100653	0.002938127	0.003570083
-0.011598657	0.011837878	0.008318889	-0.005994694	0.004651873
0.000319000	0.016258971	0.003875975	-0.001780278	-0.005213803
-0.001899742	0.013040292	-0.013048882	0.002343064	0.009977424
0.004526775	0.007809128	-0.001358997	-0.000488000	0.004017086
-0.012282540	-0.007703821	-0.003595699	0.003247710	0.000550000
-0.001513606	-0.005119833	0.000911000	-0.005243265	0.004753338
0.012522991	-0.000413000	0.005616369	-0.005904235	0.017383014
0.003785556	-0.004565405	-0.009394386	0.004269311	-0.002762886
0.002141312	-0.005988802	0.004000262	-0.005296002	-0.009099275
-0.008870622	0.000213000	-0.013806562	-0.000437000	-0.005169702
0.003580837	-0.000115000	-0.002056965	0.006937098	-0.000739000
-0.009523784	-0.000849000	-0.004627624	-0.005497480	0.003051976
0.004596978	-0.011334754	0.002864740	-0.000308000	-0.002011996
-0.006432723	-0.007254174	0.003051235	-0.012262266	-0.003672846
-0.006182974	-0.001488318	0.004835393	-0.009188291	-0.000971000
0.008207560	-0.006252039	-0.020607014	-0.005018363	0.004940126
-0.004824861	-0.009129061	-0.004719522	-0.005836573	-0.000065600
-0.002827706	0.000176000	0.000781000	0.000756000	-0.004370521
0.008189456	-0.004933627	0.003989874	0.003674754	-0.003660155
0.008894481	-0.008099408	-0.002772501	0.006218965	0.002195301
-0.001440046	-0.004771324	0.002455716	-0.003674968	-0.005193096
0.001244082	0.002221331	-0.002546462	0.008024772	-0.000870000
-0.003669338	0.000028700	-0.001836103	-0.006441291	0.003466245
0.002989082	0.001871549	-0.001505210	-0.001232918	-0.000585000
0.015478946	0.005445641	-0.000519000	-0.000133000	-0.000442000
0.015806234	-0.003031916	0.001815416	-0.003530523	-0.003191633
0.020303747	-0.010678277	-0.006373901	0.011325065	-0.010335905
-0.003089556	-0.011963709	-0.001789334	-0.009507143	-0.002743787
-0.004688759	0.001477346	-0.007823832	0.002001325	0.000203000
0.015337158	0.001020292	-0.011690185	0.003243161	0.003105701
0.012597446	-0.002911748	-0.005629701	-0.005242133	0.004269287
0.004587583	0.010506202	-0.000641000	-0.006097059	-0.008933784
0.003834452	-0.001787319	-0.000830000	-0.004205590	0.001590784
-0.012066577	-0.011810054	-0.006452302	-0.002379017	0.005138510
0.025131155	-0.001099658	-0.005794798	-0.002622291	-0.005216300
0.002437571	0.003493991	-0.012013072	-0.003619737	0.003511008
-0.006218211	-0.002012468	0.005767247	-0.007262725	-0.000927000
-0.013265597	-0.006176177	-0.002768821	0.001564828	0.001696940
0.000616000	-0.002110074	0.010404298	0.005682027	0.001974618
0.006256501	0.006042703	-0.005336504	0.001990247	-0.003921958
-0.008967470	-0.001099772	-0.000149000	0.004837335	-0.006551070
-0.007308652	0.002746522	-0.002776210	-0.009606937	-0.001050910
0.000573000	0.003779194	-0.009629591	0.000244000	0.000180000

-0.005281922	-0.016571444	-0.005175972	0.002407525	-0.001584926
-0.003082521	-0.004084325	-0.001959419	0.004852108	0.009161756
0.011444650	-0.009578979	-0.006752991	0.000827000	-0.004672077
0.002310678	-0.002327817	0.006706903	-0.005997186	0.002777398
-0.012879952	0.002048988	0.005649700	-0.013721334	-0.000666000
0.003530630	-0.007868802	0.005499766	0.008059127	-0.001738382
-0.013296525	-0.015615254	0.014650623	0.001625160	-0.000717000
0.002101175	-0.001266251	0.003539991	0.005859019	0.000511000
-0.004345704	-0.009590004	-0.000057200	0.005904067	0.002399672
-0.014457121	-0.004346475	0.001272074	0.001200912	0.007930087
0.006308864	-0.001892804	0.003639103	-0.005829718	0.003924113
0.003843438	-0.002190662	0.003027202	-0.006765761	-0.002336166
-0.005546197	-0.004598859	-0.007625316	0.001588531	0.000501000
0.001337890	-0.002255071	0.000851000	-0.009658495	-0.005245104
0.006622277	-0.004749422	0.001871576	-0.000523000	0.000230000
0.009687597	-0.003528045	0.000069600	-0.002299191	0.007659534
-0.000070300	-0.007078865	-0.007546338	0.008226170	0.010680181
-0.010316323	0.001827503	0.002105344	0.004685162	-0.002573859
0.004698956	-0.007397823	0.002096019	-0.002361033	0.002927735
0.000638000	-0.005200932	-0.001296394	-0.001527097	0.003120719
-0.000040500	-0.003152959	0.000768000	-0.004519152	-0.004553497
-0.004023088	0.000680000	-0.003144214	0.004775156	0.004691518
-0.003082630	0.000370000	-0.014256464	-0.005315176	0.004871582
0.001688305	-0.007131579	-0.012068621	-0.000075100	-0.000137000
-0.004042468	0.011668988	-0.001740899	0.000045600	0.007499910
-0.009622270	0.001726041	0.001037963	-0.006523668	0.001650460
-0.004296738	0.006810167	-0.000137000	0.002414656	-0.002471362
0.007151327	-0.009031749	0.005308127	-0.002243000	0.002509385
0.002290074	0.004977367	-0.005222204	-0.002317591	0.006337413
-0.008590212	0.005429201	-0.001025417	0.002630071	0.002329008
0.014407535	-0.004443995	-0.000099000	0.006759038	0.002710383
0.002608067	-0.000337000	-0.005922314	-0.000971000	0.005056382
-0.006474195	0.011822358	-0.003185436	-0.002526521	0.011710469
-0.008048957	-0.006792293	0.004512414	-0.001872199	0.003209212
-0.004197071	-0.010144458	0.003247976	0.003757301	0.007955934
0.001433890	-0.005358207	-0.006478158	0.002407486	-0.006726398
0.008196288	0.011227858	0.006021872	0.009716306	-0.003779426
0.009011807	-0.008592652	0.002162857	0.004248587	0.006297637
0.002197761	-0.012801911	-0.004828874	0.003088021	0.004358780
-0.006590855	0.006913941	-0.001978091	0.009804194	0.004203444
0.001863232	0.004354254	0.004306818	0.007497383	0.011765647
0.022344987	-0.009513831	0.011420320	0.004547795	-0.000622000
-0.002812655	-0.007881156	0.000359000	-0.001060458	0.002850096
0.006606021	0.008015109	0.008865768	-0.001348136	0.011475920
-0.009670684	0.003474453	0.008094645	0.010379374	0.011063974
0.009306348	-0.003228422	0.006051088	-0.016002026	0.004963619
-0.005995075	0.006402546	-0.007547275	-0.011013441	-0.007627608
0.003734918	-0.012954628	-0.011355196	0.003358440	-0.002305413

-0.007732106	-0.005453229	-0.003898592	0.003705370	0.010345096
-0.003325951	-0.003700605	-0.006658470	0.008887987	0.005506177
0.001580243	0.008068387	0.001769799	0.012893274	0.001140621
-0.004555422	0.008392952	0.001580182	-0.001868705	0.002055111
-0.006274964	-0.012570689	-0.003031382	-0.003298481	-0.002882332
0.009731098	0.004216641	0.000626000	-0.003423541	0.001361102
-0.006732054	0.002486589	-0.000464000	0.004731747	-0.002943534
-0.010017902	0.000860000	-0.001518311	-0.004407224	0.000990000
-0.000731000	-0.000378000	0.001859726	-0.001493124	-0.004184286
0.001683467	-0.007272254	-0.003845501	0.017141550	0.009575226
0.004329084	0.003855307	-0.003611518	-0.003614431	-0.002255183
0.011935266	0.005966037	0.007941392	-0.000646000	-0.010229035
0.003603195	-0.008932496	-0.002857234	0.001003701	0.005996682
-0.001118510	-0.022518520	0.007423524	0.006320548	0.000934000
0.008143495	0.010846590	-0.005353118	0.000435000	0.012324494
0.006058870	0.001411411	-0.002151428	0.002267511	0.006964861
-0.001149330	-0.001661712	-0.001457695	-0.004447439	0.010346239
0.004311153	-0.004095836	-0.012834634	-0.003374835	-0.000267000
-0.000039700	0.004850972	0.004872477	0.009163269	0.003081298
-0.005966060	0.001323494	0.011382349	0.008713193	-0.004875424
0.003395495	0.007598852	0.006436935	0.013930852	0.006884044
-0.002138815	-0.010313257	0.000605000	-0.008706801	-0.002535055
-0.006785971	0.005919534	0.001868649	0.002844465	0.003912860
-0.000762000	0.001099104	-0.003019710	-0.000686000	0.002249447
0.014876129	-0.012449949	-0.002320856	-0.003133991	0.011043922
0.015234768	0.002213676	0.001205650	-0.001705036	0.003055348
0.008044120	0.008475999	0.010769999	0.003872566	-0.006365457
0.005885804	-0.007825932	-0.006328476	-0.003959605	-0.000897000
0.004319464	-0.000837000	0.000920000	-0.001983759	-0.000802000
-0.008598764	-0.002213522	0.013416177	0.001439068	-0.004502357
-0.005492753	-0.005000136	-0.006216460	-0.008827049	0.000635000
0.005770868	0.002248918	0.000376000	0.001578126	-0.008394837
0.016608106	-0.008575283	-0.001415133	-0.003498790	-0.003680766
0.012953321	-0.006139376	-0.001909856	-0.003329726	0.005536264
-0.006212122	0.004431130	-0.003149237	0.003555088	0.000018500
-0.007930536	-0.005690807	0.006464086	-0.006002744	-0.007333793
-0.013553541	0.002005853	-0.001921850	0.003098394	-0.000508000
-0.003281848	0.010768935	0.003115389	-0.005040796	-0.005346747
0.007553081	-0.001827374	0.005362355	-0.005875089	-0.010998349
-0.003716906	-0.003093572	-0.006651872	-0.001022470	0.001972299
-0.000813000	0.012228117	-0.011842095	-0.005326817	-0.008018207
-0.002912716	0.013041123	-0.006135994	-0.008281611	0.000713000
-0.000310000	0.008741387	-0.004830593	-0.000020400	0.002869265
-0.002159428	0.001921262	-0.001052134	0.001152957	0.004309490
-0.007853074	0.007552725	-0.007244367	-0.000975000	-0.003615073
-0.004522606	0.006433437	-0.011504838	-0.016147498	-0.003191070
-0.008306020	0.005701101	0.003317336	-0.001608949	-0.004362331
-0.001561218	-0.002631194	-0.006274094	-0.010553569	0.001544188

-0.004214269	0.003531562	0.002772985	-0.008332299	-0.002812104
-0.006100549	0.006204331	-0.003165017	0.002213558	-0.007207202
0.002811914	0.009344684	-0.007812960	-0.012159875	-0.009071576
-0.012017129	0.005531916	-0.000617000	-0.005407823	0.001055309
-0.005898457	-0.003706061	-0.006483994	-0.001618265	-0.003018120
-0.008367278	0.002355283	0.000574000	-0.006293008	-0.003609021
0.004802043	0.005497044	-0.009630793	-0.006810857	0.001090305
-0.014436701	-0.011500174	0.004897201	-0.001639063	-0.000451000
-0.007008683	-0.010194768	-0.002800594	-0.001332113	-0.013591919
0.000138000	-0.004062256	0.012877096	0.005528276	-0.005825411
0.012681490	0.006392342	0.004908245	0.001477019	-0.004010025
0.000454000	-0.012506946	-0.000623000	0.003177778	-0.010107472
0.005678571	0.000250000	0.004196811	-0.002298082	-0.006825929
-0.004498038	0.004676448	0.006055207	0.001020708	-0.002481092
-0.003855296	0.006573579	-0.000765000	-0.009432378	-0.002078697
-0.010361895	0.002077836	0.003722999	-0.004889177	-0.004613196
0.005476494	0.005279301	-0.001773925	-0.004772176	-0.005466249
-0.002448286	0.002440603	-0.005894012	-0.002338562	0.001335510
0.003704466	-0.013221732	-0.000457000	0.002314120	0.007110192
0.009233437	-0.002906123	-0.001505794	-0.002415632	0.000645000
0.010688452	0.006610282	0.012655478	-0.003022420	-0.010213399
0.006581042	0.002515815	-0.004869018	-0.016947707	0.003699178
0.018506998	0.006540664	0.013669803	-0.000953000	-0.006643487
0.010380215	0.008338126	-0.003752103	-0.007004277	0.009239918
-0.001388358	0.006973095	-0.012143977	-0.000239000	0.004533498
0.006981585	0.000628000	-0.002939409	0.002192006	0.003543257
-0.000080300	0.003415789	0.006643946	-0.000672000	-0.003538755
0.003418140	0.010465685	0.001564784	-0.007759374	-0.003153723
0.005694847	0.006310881	0.005462577	-0.005051030	-0.002672231
-0.005322776	0.001642315	0.012148286	0.006186587	-0.002391750
-0.004941173	-0.005167290	0.007184733	-0.004473541	0.002609964
0.014198563	0.004684490	0.014939329	-0.005089075	0.000480000
-0.003800068	0.006469851	0.010024333	0.000308000	-0.004140623
-0.006766655	0.007484931	0.007488593	0.006641642	0.004362827
0.003453813	-0.007953320	0.001899555	0.000045500	-0.003606140
-0.012579202	-0.006576768	0.010187624	-0.003682006	-0.002930491
0.000698000	0.000814000	0.008841088	-0.006919488	-0.006057887
-0.003798732	0.000775000	-0.003300541	-0.005791574	-0.004943491

PC10	PC11	PC12	PC13	PC14
0.002233530	-0.005151570	-0.001614170	-0.001975364	-0.006602179
-0.002031675	0.003172861	-0.003834362	0.001437460	-0.002938043
-0.000943000	-0.001039977	-0.005239809	0.005473818	-0.001431692
0.027391476	-0.003206213	-0.010921736	-0.002551289	-0.000700000
-0.001657985	0.001054443	0.001296261	-0.004009343	-0.005481290
-0.008289042	0.008656859	-0.003536432	0.003167968	-0.001631848
0.007070589	-0.001652283	0.003690382	-0.002205527	-0.001167900
-0.000182000	-0.009302474	-0.002048853	0.004215696	0.003955841
0.008276069	0.006508029	-0.005571915	0.002524843	0.000483000
-0.003785448	-0.001085598	-0.005805410	0.004280315	-0.004758860
-0.002876793	-0.002676164	0.004191058	-0.000366000	-0.000666000
-0.004702734	-0.001292865	-0.008704419	-0.003709051	-0.002696847
-0.004953077	-0.002548794	-0.006520393	-0.002113138	-0.005259716
-0.003032189	-0.007521376	-0.004325069	0.000473000	-0.002125340
0.000161000	-0.006038683	0.000504000	0.001801943	-0.000566000
-0.000571000	-0.006819331	0.004792450	0.002698005	0.001077703
-0.001494952	-0.000167000	-0.004925819	-0.005935549	-0.002028513
-0.001879563	0.004134858	0.006628519	0.004645409	-0.002419281
0.016215779	0.005008754	0.002665397	0.011761109	-0.007794926
0.000257000	0.000015300	-0.009425509	-0.001067427	0.004714434
0.006274747	-0.002771491	0.002892811	-0.005644582	-0.002505266
0.001693698	-0.003812789	0.001289325	-0.003941058	0.002475419
-0.008083426	-0.001224041	0.000663000	0.006050904	-0.001181152
-0.007584459	0.001673516	-0.003732424	0.001218065	0.000232000
-0.003768068	0.003271453	-0.000910000	0.006758778	-0.000370000
0.002055521	0.001900442	-0.000468000	0.004178531	-0.002679929
0.002132720	-0.001957232	0.003054129	0.004146431	-0.007518624
-0.002630782	0.003033919	0.001084465	0.001376994	-0.006096831
0.000662000	-0.005821363	-0.004833504	-0.000411000	-0.003407482
0.002776486	0.005288365	0.002554429	-0.002310909	-0.003445910
0.013705827	0.002225277	-0.003363978	-0.000456000	-0.002624703
-0.007915594	0.006084537	-0.004791998	0.002552685	-0.001217901
-0.001996046	-0.000907000	-0.007147397	0.000627000	-0.004336853
0.003019176	-0.004013552	-0.002094635	-0.002891293	-0.000928000
-0.002182350	0.003505290	0.004612621	0.000028200	-0.003866693
0.005151009	0.006249330	0.002556341	-0.003507043	-0.004607342
0.004590130	0.013218916	-0.005406180	0.003097355	0.002537202
0.001868716	0.004670320	-0.000946000	-0.003078284	-0.003047528
-0.001502029	0.004820822	-0.002989339	0.004748983	0.002835755
-0.005337733	0.000537000	0.005499518	-0.001840748	-0.003732885
0.000499000	0.006580926	0.000187000	0.004795661	0.001157666
0.003493628	0.000952000	-0.004519339	-0.000797000	-0.000274000
-0.006026403	-0.003317737	-0.005654641	-0.004350984	-0.002960559

-0.009189744	-0.000046200	-0.006183573	-0.003296833	-0.002235650
0.005344463	0.000543000	-0.004237099	0.001861136	0.000924000
-0.004176374	0.001046438	-0.002335185	-0.005186797	0.000480000
-0.006213101	0.000177000	0.002167255	-0.001355779	-0.000061700
0.001252216	-0.000563000	-0.004535560	-0.000670000	0.003099169
0.004875624	0.000070100	0.002323194	0.001385582	-0.003254114
0.002040374	0.005919770	-0.003674971	0.001271447	-0.003247955
-0.004222499	-0.001004995	-0.002276260	-0.003216183	-0.000086100
-0.000256000	0.003186782	-0.001066844	0.000317000	0.001184701
0.002664007	0.005782086	-0.000190000	0.004378616	0.000919000
-0.002197440	0.000474000	-0.001980475	-0.004975430	-0.000920000
0.016451704	-0.000540000	0.002969717	-0.004472389	-0.003053130
-0.000649000	0.007596606	-0.001821110	-0.008986444	-0.001239323
0.000339000	0.001738575	-0.003597896	-0.003163523	-0.001834957
0.004386224	-0.000019200	-0.000667000	-0.002838943	-0.002494360
-0.005789464	-0.006970249	0.004599173	-0.002386822	-0.004428489
-0.000677000	0.002967088	-0.002626964	0.002543965	0.003316570
0.004510225	-0.003941047	0.002967057	0.001980494	0.005901098
0.004493114	0.001245588	0.000766000	-0.001519349	0.004108605
-0.001711699	-0.003797604	-0.000052800	0.003535644	-0.000460000
-0.005563489	-0.002971662	-0.002667421	-0.008112120	0.000890000
-0.003879109	-0.000829000	-0.000572000	0.001229727	-0.003003234
-0.000135000	-0.012749798	0.000300000	0.002360542	-0.004588418
0.003232957	-0.005000514	0.000877000	-0.001774449	0.002181649
0.004388740	-0.001907599	-0.000075800	-0.002611575	0.001546280
-0.003067947	-0.001092010	0.002527915	0.002227721	-0.000461000
-0.001004526	0.002662840	0.000021900	-0.002601570	-0.004045131
0.003090891	0.000392000	-0.000066200	-0.002640636	-0.001076181
0.001222879	0.006154722	0.004794624	0.005946892	0.002458416
-0.005499187	-0.004730247	-0.005977587	-0.000361000	0.000411000
-0.000000664	0.000566000	0.004632801	0.000113000	-0.002124096
0.007308033	-0.003312989	-0.004577425	0.007170186	-0.007183065
0.000009100	-0.002397602	0.005558103	0.003465473	-0.001787777
0.003118749	0.002744787	0.000985000	-0.001366895	-0.004869605
0.007792948	0.000873000	-0.002378172	0.001003105	-0.000294000
-0.001314754	-0.004202370	0.001026536	0.002558750	-0.001450972
0.001246944	0.002229881	0.001133210	-0.000176000	0.002197338
-0.005022865	-0.002901765	0.001262453	0.002998410	-0.001103564
-0.001904235	0.004808092	0.008268417	-0.001476364	-0.000368000
-0.003739522	0.004328557	-0.000735000	0.002276476	0.004146198
0.005547889	0.001837187	-0.000495000	-0.000744000	-0.000170000
0.000289000	0.002407520	-0.004170280	-0.002038058	-0.000153000
-0.001885584	0.001563064	-0.010276490	-0.008305586	0.002296762
-0.006095649	0.002094129	0.000676000	0.002899009	0.002551651
-0.000381000	-0.002394055	-0.003036116	0.000390000	-0.001039214
-0.008203018	-0.002509370	0.000923000	-0.000570000	-0.004544424
-0.003274775	-0.004001573	-0.005899103	-0.002185128	-0.000657000
-0.001141463	0.000682000	-0.006945227	0.002884748	-0.003309753

-0.000603000	-0.001586615	0.008891658	0.001272463	-0.005969641
-0.004033879	0.001295267	-0.001407460	0.001833933	0.004706859
0.003684023	0.004162223	0.002727696	0.002329977	-0.000158000
0.001077003	-0.006830672	0.001680670	-0.003838902	-0.004314156
-0.002782003	-0.005049839	-0.001148100	0.001218774	0.000846000
-0.007148768	-0.000613000	-0.006885450	0.000627000	-0.001763484
0.005139433	0.000221000	-0.003363577	-0.005161590	0.001043125
-0.011206589	0.003797531	-0.001966364	-0.004447673	0.004257692
0.005469479	0.005153369	0.002377760	-0.004299385	-0.001454375
-0.003732762	-0.008346983	0.006748702	0.001543445	-0.000648000
-0.001194050	0.003054364	-0.000562000	-0.001688737	0.000619000
-0.010509125	0.006895718	0.001196445	-0.007632100	-0.000023900
-0.005494554	-0.005857387	-0.001103238	0.009173418	0.007019559
-0.003499235	0.005205131	-0.002607719	-0.000598000	-0.002468066
-0.004363264	-0.004706516	-0.002994925	-0.003506873	-0.002426696
0.001526092	-0.002505138	-0.002065724	-0.001160817	-0.001887686
-0.000244000	0.008868847	-0.004269652	0.001235040	-0.003859892
-0.000041600	-0.001922266	0.004936124	-0.000341000	-0.004254634
-0.005479342	-0.004930477	-0.002249179	0.000722000	-0.003720621
-0.002406659	-0.001176801	0.004039941	0.002482411	-0.006565650
-0.004319906	-0.002207327	-0.001186494	-0.007053375	-0.000804000
-0.005871466	-0.001965305	-0.000271000	0.000508000	-0.002649185
0.000818000	-0.001796222	0.002161551	-0.001629179	-0.002132915
-0.001119047	-0.001219134	-0.000040800	0.003223507	-0.001946227
0.000210000	0.000131000	0.007690851	-0.003673243	-0.003790443
-0.006297178	0.001471682	-0.003602007	0.000706000	-0.001199777
0.003592861	0.000524000	0.004497430	-0.005276043	-0.000513000
-0.009932404	-0.002912484	-0.000277000	0.000053400	-0.000178000
-0.000911000	0.000014900	-0.001200321	-0.001223479	-0.000828000
-0.003557164	0.002443803	-0.002713532	-0.004887203	-0.001613790
0.008790466	0.000055800	-0.008201957	-0.003320630	-0.005177395
0.003947507	0.007224981	-0.004930575	-0.002951874	-0.002400367
0.006077637	-0.002930046	0.000975000	-0.005842148	-0.002053701
0.011188045	-0.000564000	0.003963795	-0.000842000	-0.000162000
-0.003046297	-0.006568397	-0.000283000	0.004968527	-0.006427276
0.000239000	-0.000692000	0.000137000	-0.003429885	-0.000179000
0.003404998	0.002988006	0.001746473	-0.003902411	-0.000539000
-0.001730744	0.010044636	-0.002329567	0.002940564	-0.001420293
-0.003525008	-0.000419000	0.002991918	0.003139358	-0.000155000
0.000467000	0.000805000	0.002644444	0.000339000	0.002262532
0.005860190	0.002277531	0.011310339	-0.000428000	0.000579000
-0.004717553	-0.001447367	-0.006000045	-0.001057387	-0.000700000
0.000388000	0.000927000	0.000048300	-0.000184000	-0.000083300
-0.001884576	0.007610504	0.003090098	-0.004368284	-0.002606154
-0.000212000	-0.002563718	0.002718710	-0.001042666	0.005384163
0.001947546	-0.001140732	0.000581000	-0.000024400	0.004463171
0.000879000	0.001364289	0.000141000	0.000873000	0.004350800
-0.001879992	-0.003379669	0.001186296	0.002007545	-0.002397283

-0.001735031	0.000971000	-0.000474000	0.000790000	0.000709000
0.005085305	-0.008886838	-0.004994216	0.002947703	0.002781836
-0.007162351	-0.002297903	0.005501218	0.006625027	0.001310941
0.002844815	0.000134000	0.004703205	0.001398507	0.003119190
-0.000407000	0.011764741	-0.001813278	-0.000775000	-0.001103856
0.005916738	0.000875000	0.002998097	0.002168524	-0.002905770
-0.003311239	0.000793000	-0.000369000	-0.000141000	0.000398000
-0.002071055	-0.004192066	-0.001757189	-0.000954000	0.000328000
-0.002673774	-0.001998310	-0.001403155	-0.002342800	0.003322023
0.001709856	-0.005684750	-0.007153520	0.002704247	-0.002975373
-0.000244000	-0.000212000	-0.001945794	0.002719426	0.004849684
-0.000888000	-0.005049403	-0.000187000	0.001329052	0.000198000
0.002597774	0.003001826	-0.005469467	-0.000222000	0.001290369
0.004616244	-0.004245997	0.005864698	-0.001598340	0.003355222
-0.000777000	0.002687698	-0.000928000	0.005684266	0.001468087
-0.008587969	0.002703421	-0.007362535	0.000516000	-0.002561213
-0.007046704	-0.000837000	0.004822294	-0.002868583	-0.001284545
-0.000879000	0.001464411	-0.004989645	0.002007194	0.000060300
-0.002004625	0.001734074	-0.000663000	0.003920662	-0.003032184
-0.000391000	-0.004672376	0.001357609	-0.002667721	-0.003143684
0.000444000	-0.001450793	-0.003707823	0.000698000	0.003494280
-0.005106854	-0.002571516	0.000295000	-0.000307000	0.002453226
0.011396709	0.005035252	0.002700208	-0.002516880	0.003686542
0.003707723	-0.006474237	0.001676230	-0.000212000	0.000310000
0.003673039	0.001189992	-0.001133390	0.001878688	0.001087349
0.001164434	-0.001912338	0.001781591	-0.001964483	0.003743607
0.002190756	0.001585103	0.000958000	0.003150747	0.002921774
-0.002258722	0.006900661	0.003366039	0.004826424	0.000490000
0.005877927	-0.005157161	0.000647000	0.004374794	0.002706452
-0.001374338	-0.000698000	-0.006596404	-0.002419928	-0.000648000
-0.005399178	-0.003665831	0.001166091	0.003831345	0.000156000
0.002668528	0.001793349	0.004995024	0.004418105	0.005862957
-0.006066261	-0.000058200	0.003606302	-0.002668554	0.002859210
0.005088071	0.001595813	0.004070601	0.000753000	0.001365752
-0.005378006	0.000587000	0.001479981	0.001528140	0.005161800
0.005425217	0.004124735	0.001653072	0.006402115	-0.001351752
-0.001581863	-0.000660000	0.001885089	0.001929860	-0.001709829
-0.001426515	-0.003390640	-0.002090147	0.003960971	0.003073677
0.002340493	-0.006666820	-0.001153992	0.003404364	0.001072736
0.002688602	-0.004833242	0.000808000	0.005941495	-0.000369000
-0.000895000	-0.001579886	-0.003796634	-0.003224208	-0.000629000
0.001195929	0.007265375	0.002939596	0.002462158	-0.000035900
-0.004740730	0.000759000	-0.000641000	0.006095650	-0.007578294
-0.001309059	0.009565772	-0.000796000	-0.001422950	-0.000483000
0.001704298	0.001798457	0.004900114	0.000257000	-0.003694979
-0.001792364	0.002375285	0.004718685	0.001780809	-0.003519738
-0.002408156	0.009555042	0.002773292	-0.000970000	-0.004409424
0.006847975	0.000072900	0.006121000	-0.004154420	-0.003516336

-0.000966000	-0.000896000	0.001168179	0.002760625	-0.002347810
0.001334074	-0.000380000	0.003494395	-0.004445172	0.002439360
0.001288389	0.004934536	-0.001627968	-0.001413100	0.000566000
0.002468060	-0.000096000	0.000618000	0.003788334	-0.001475758
0.001303389	0.009639804	0.005431144	-0.007997490	0.000723000
0.008446620	-0.007279616	0.003958146	-0.004219575	-0.001134144
-0.000261000	0.004012900	0.001271903	-0.005944784	0.003720516
-0.001758441	0.001351385	-0.002692931	-0.000499000	0.001542890
-0.006033539	-0.003595503	0.001389000	-0.005664030	0.002462414
-0.002854171	-0.003265715	0.001028260	-0.002224175	0.001360773
-0.003288817	0.003984728	-0.004978689	-0.007092438	0.002246599
-0.003158064	-0.003327069	0.002164185	0.001627333	-0.000704000
-0.004517267	-0.000411000	0.001839598	0.003257193	0.003612155
0.001691489	-0.001586942	-0.008459081	0.006025473	0.008804256
-0.000327000	-0.003537369	0.000796000	-0.000756000	-0.001276192
0.001763391	-0.003593715	-0.003858964	0.004329007	0.000039600
0.006314532	0.005524340	0.001396616	0.004524452	0.005959625
0.004466548	0.004207064	0.007141403	-0.004521433	0.005188635
0.013315744	0.008137725	0.003429002	0.000574000	-0.001016498
-0.005593766	-0.001025924	0.002754646	-0.005890854	-0.000890000
-0.001159083	0.006418111	0.007056580	-0.003152916	0.004171172
0.000540000	-0.000432000	0.004858695	-0.003901282	-0.001257310
-0.004311658	-0.002542966	-0.004654467	-0.005807414	-0.002710361
-0.010416412	0.000208000	-0.001228995	0.000266000	-0.000896000
-0.004590179	0.009500224	-0.007280082	0.001136438	-0.003570943
-0.004582793	-0.002503466	0.005480764	-0.003761151	0.003703713
-0.007796664	0.004426382	0.004914729	0.006456645	-0.001138145
-0.002839878	-0.002063878	-0.003920785	-0.001469958	-0.001086816
0.000401000	-0.002293782	0.001256242	0.007502576	0.002083644
-0.000852000	0.001941745	-0.002058635	0.000095700	0.007452559
0.000043000	-0.000673000	-0.000941000	-0.002093039	0.002231981
0.003222057	0.004083562	-0.002173319	0.001772719	0.002101775
0.005015693	-0.005329227	0.002264621	-0.002446119	0.002804882
0.001109895	0.002795875	-0.001193788	-0.001303394	0.001853778
-0.009037942	0.002070518	0.002327580	0.003412894	0.000730000
-0.010340613	0.000050500	0.009181982	0.006624258	-0.002587015
0.001725996	-0.005938451	0.005684323	-0.000263000	0.003226035
-0.008550916	0.000882000	0.006153856	0.005258565	0.001259438
0.000643000	0.000309000	-0.001143280	0.002927770	-0.001612858
0.001933829	-0.000952000	-0.003227723	0.007996194	0.000543000
0.002235253	-0.002785341	0.004590347	-0.002343635	0.000694000
0.003290154	0.000866000	0.001786611	0.003574013	-0.001917028
0.000094700	0.001279055	0.001098354	0.002922228	-0.003579357
0.002225071	0.005790335	-0.000559000	0.001514579	0.000096000
-0.001221227	0.000861000	0.002485405	0.002918811	-0.003370380
0.000021100	0.002678010	0.003366062	0.001860193	-0.001742808
0.001326477	-0.002724654	-0.000964000	0.002875341	0.002424390
-0.002118762	0.000674000	0.005387434	-0.004809838	-0.004071477

-0.003049621	-0.006637536	0.003418724	0.005994938	0.002280833
-0.010039124	0.009907048	0.004192237	-0.004651411	-0.000472000
-0.004869468	0.010877404	-0.010567456	-0.001970386	0.002448060
0.000332000	-0.001202554	0.000325000	-0.000033200	0.008897523
-0.003915290	-0.006698328	-0.000510000	0.003598725	-0.000521000
0.003805651	-0.000153000	0.001344616	0.002055468	0.002446365
0.005746073	-0.006041723	0.002198622	-0.002957261	-0.004082404
0.005599788	0.001915927	-0.003017622	-0.004719642	0.000099400
0.002583348	0.003359382	-0.003620799	0.003522649	0.000540000
0.003172830	-0.005010175	-0.005354897	0.001678147	0.001348609
-0.003460761	-0.010708944	-0.000031500	-0.004137184	0.000023700
0.009848715	-0.001010339	-0.000869000	0.001154701	0.001685144
0.003508602	0.000827000	-0.003143090	-0.005029731	0.005323016
0.002582232	-0.003492984	0.006592900	0.002517745	0.005764278
0.004824716	-0.013151605	-0.004969576	0.008168942	-0.001908275
-0.000256000	-0.002689001	-0.005187345	0.005708025	0.005471813
0.006277027	-0.001001548	-0.001871814	-0.000296000	-0.000702000
0.002827126	-0.002537903	0.001948445	0.000175000	0.006578303
0.004952735	0.002187994	0.002080129	0.003656052	0.000509000
0.002966764	0.001716562	0.000948000	0.000857000	-0.000325000
-0.007665894	-0.004415486	0.003611935	-0.001924208	0.006278310
-0.005650433	-0.003823141	-0.004076151	-0.001602542	0.004652382
0.015457910	0.002322587	-0.001730265	0.002315508	0.002955655
-0.001237554	0.000618000	-0.000544000	0.006756239	-0.008328313
0.000913000	-0.000401000	-0.001321750	-0.006109622	0.006726614
-0.007360949	0.005439871	0.008873024	-0.005855164	0.002294440
-0.001156295	-0.002248522	0.003340277	-0.000291000	0.003948251
0.000427000	-0.002403541	0.006802243	-0.002273427	0.002134125
0.005239474	0.000760000	-0.003291810	-0.001889495	0.005559253
0.000368000	-0.002654398	0.007653968	-0.000607000	0.004653558
0.014454442	0.000325000	-0.002475096	-0.001222004	0.003243638
0.010027781	0.001804511	0.005598417	0.001971220	-0.002213086
-0.002562937	-0.000960000	0.005599227	-0.002750136	0.003527454
-0.000927000	-0.002274979	-0.001183760	-0.007685933	0.002693566
0.005434032	0.000947000	0.003347378	-0.003686032	-0.002075807
0.008626977	-0.009139985	-0.002281121	-0.000657000	-0.003608259
0.001421433	-0.010663640	-0.002189818	0.000855000	0.003561063
0.001828575	0.002716572	0.001599767	-0.008751162	0.000193000

PC15	PC16	PC17	PC18	PC19
0.006156641	0.002166878	0.001303135	-0.004068121	-0.000291000
-0.000730000	-0.005303280	0.000177000	-0.001671215	0.001416208
-0.003823223	-0.003791055	-0.000468000	0.005190844	0.002715238
0.001384192	-0.005256719	0.000671000	-0.000081700	0.000733000
0.001258512	0.002227033	-0.000312000	-0.003397075	0.001588086
0.002787465	-0.001008528	-0.003544015	-0.001191752	0.001907993
0.002562851	-0.002391369	-0.001291408	-0.002614825	-0.002414824
0.000675000	-0.004009563	-0.000951000	0.001433530	0.002712594
0.003002137	0.001249962	-0.001370933	-0.005857972	-0.002825145
0.006925423	0.000311000	0.002557569	-0.000118000	-0.004265218
0.003153105	-0.004536842	-0.001685412	-0.001478215	-0.000177000
-0.001825172	0.001705990	0.001164957	-0.004432719	0.001199501
-0.003035653	-0.002796972	0.001647005	0.001220388	0.001797318
-0.005995107	-0.000966000	0.000496000	0.002540609	-0.004755723
-0.000068100	0.002503495	0.000506000	0.001298302	-0.000703000
0.002022710	-0.003573394	-0.002065909	0.002432705	0.002669329
-0.000645000	-0.000568000	-0.005948728	-0.005352368	0.000380000
-0.003604480	-0.002265836	-0.000090300	0.001566523	-0.000547000
-0.000262000	-0.002125023	-0.000676000	-0.002510110	0.001309762
0.000549000	-0.000695000	-0.000559000	0.002670295	-0.002665436
-0.003622477	0.002685483	-0.000671000	0.002613214	0.005786492
0.003696364	0.005462863	0.001808803	-0.005918476	0.000480000
-0.001117119	-0.001698236	0.002598296	-0.000821000	0.002380498
0.007113167	-0.001695781	0.002810755	0.001374725	0.002080135
0.001641149	-0.000385000	-0.001447950	0.002263943	0.001380765
-0.005059373	0.001094824	-0.000757000	-0.001133187	-0.000330000
0.002238440	0.000225000	-0.004326649	-0.002060374	0.001706719
0.000265000	-0.001592259	-0.002288293	0.002756404	0.000804000
0.001281818	-0.001916057	0.001887374	-0.001153594	0.000518000
-0.005814338	-0.001872975	0.000958000	0.004547966	0.001914793
0.005507837	-0.001020514	0.000028600	0.000027400	0.004769738
0.003609427	0.003042972	-0.001411995	0.002579703	0.001602152
0.000673000	0.006311156	0.002497719	-0.000123000	-0.000387000
-0.000695000	0.001209286	0.000203000	-0.003062343	0.003407973
-0.001051357	0.001233451	-0.003497226	0.000037600	0.004174569
0.000954000	0.000475000	0.002919589	0.001508950	-0.004806869
-0.006786085	-0.002882054	-0.001012978	-0.005133889	-0.003246495
0.002929182	-0.000759000	-0.002833366	0.002726382	0.000961000
-0.000609000	0.003211223	-0.001089348	0.000054300	-0.001121454
-0.000755000	-0.002828793	0.000272000	-0.000429000	-0.002895105
-0.001537074	-0.000234000	-0.001817587	-0.001205693	-0.000918000
-0.001336181	-0.002056509	-0.003095513	-0.000582000	0.000839000
-0.003661560	-0.000289000	-0.000119000	-0.000867000	-0.003015581

-0.000371000	-0.000156000	-0.001840155	-0.001030443	0.003305406
-0.002356313	-0.000207000	-0.001244334	-0.000142000	0.002445883
-0.001686590	-0.000029000	0.002096269	-0.000834000	0.006878532
0.002577548	0.002841387	0.001102939	0.002559597	0.002515947
-0.002238027	0.001354953	-0.001116866	0.003527751	0.001316239
-0.004301872	-0.005359597	0.003913385	-0.001442881	-0.000236000
-0.000098800	0.002170234	0.002811644	0.001484075	0.002481675
-0.006176584	0.001623018	0.001496926	0.000613000	0.001946755
0.000705000	0.003398616	0.004522561	0.004882487	0.002472598
-0.006294664	-0.001668762	-0.001072215	-0.000822000	-0.000367000
-0.002231581	0.000209000	-0.003040907	-0.000124000	0.003048260
-0.000116000	-0.000355000	0.003562506	-0.001576717	0.002825979
-0.004011690	-0.003262212	0.000731000	-0.003685601	0.005951922
-0.005968312	0.000510000	-0.001675422	0.001783535	-0.002048875
-0.005798539	-0.001645947	-0.000974000	0.002773410	-0.002269131
0.000332000	-0.001984271	-0.000946000	0.001165192	0.001658265
-0.001291174	0.002548595	0.005545382	-0.005842425	0.004486932
0.000426000	-0.004587225	0.000521000	0.003040367	0.002399240
-0.004253672	-0.003465064	0.003719455	-0.003907074	0.001712792
-0.001642791	0.000308000	0.000355000	0.000269000	0.002022149
-0.002342880	-0.002358330	0.004496462	-0.002089691	0.000960000
-0.001413610	0.001920994	0.001495892	0.003332379	0.006722947
0.002827963	-0.001168672	-0.001972907	-0.001024256	0.002748030
-0.000920000	0.001091300	0.001728404	-0.002095212	0.004354718
-0.000317000	0.001351989	-0.003041387	-0.001729353	-0.002682520
-0.000847000	0.000045500	0.001240071	-0.000048500	-0.003473798
-0.000284000	-0.002367070	0.003446607	0.000868000	0.000132000
-0.002777892	-0.000219000	-0.000154000	-0.001881003	0.002054639
0.002124174	-0.001390704	0.004886519	-0.000728000	0.002038546
0.000276000	0.004149241	0.003107226	-0.001671365	0.002025577
0.006794575	-0.002142833	0.000310000	-0.001924522	0.004952189
0.004472413	-0.003833845	0.001227830	0.001859689	0.002002062
-0.004646190	-0.002900649	0.000965000	0.000947000	-0.004135375
0.001036275	-0.000742000	0.000466000	0.001941075	0.001438099
0.000654000	0.002410510	0.000186000	0.001303250	-0.000649000
-0.005690863	-0.004534250	-0.003622272	0.001212812	-0.000677000
0.003312314	-0.006007748	-0.003694555	0.000652000	0.002151109
0.004189837	-0.006983247	-0.004003543	0.003695317	0.003465449
-0.000921000	-0.003642953	0.001135076	0.002403708	-0.000397000
0.002886228	-0.002628800	0.004936521	-0.001870447	0.000538000
-0.000536000	-0.006436587	0.000839000	0.000020600	-0.000557000
-0.000888000	-0.000766000	0.001078223	-0.000609000	-0.001731224
-0.001574723	-0.003362168	0.000430000	0.000660000	-0.002973042
-0.001880573	-0.006098432	0.002368430	-0.003841558	-0.001311343
0.003797187	0.002790993	-0.001422676	0.000224000	0.003253498
0.000714000	0.001342972	0.003301889	0.004692816	0.002215858
0.000661000	-0.003023849	-0.002533217	-0.000683000	0.001480398
0.005458395	-0.003472339	0.004553553	0.001098666	-0.002668337

-0.000486000	0.005573625	0.003710333	-0.006937796	0.001575046
0.000554000	0.000599000	0.001199080	0.001556420	0.000807000
-0.000517000	-0.003700366	-0.000137000	-0.001208401	0.005511509
-0.000250000	0.000560000	0.001437802	0.000936000	-0.000122000
-0.002408688	-0.002575824	0.000969000	0.004946850	0.001926123
-0.005219741	0.003440343	0.000439000	-0.005014949	-0.000860000
-0.003416790	-0.005351115	0.003840439	0.003235818	0.005850509
0.006164686	-0.005063295	0.000927000	0.002058395	0.000007970
0.003180317	-0.001791646	0.005494526	-0.005033395	0.001535457
0.000463000	-0.002885354	0.001443500	-0.005132932	-0.002642081
0.004048327	-0.000938000	0.001393429	-0.000216000	-0.000424000
-0.001544097	-0.004459769	-0.000620000	-0.001991409	0.002992220
0.002435455	-0.001544229	0.004984993	0.002593156	0.005370147
-0.001057205	0.000841000	0.002621822	-0.002637112	0.000154000
0.001649373	-0.001193790	-0.002584432	0.000920000	0.001575866
0.004361073	0.000572000	-0.002852818	0.003344675	-0.002285153
0.002215300	0.000590000	0.000338000	-0.001239360	-0.002080335
-0.004267855	-0.001066076	0.003273233	0.003117068	0.003221528
-0.000073800	-0.000392000	-0.000502000	0.003426861	0.000049700
-0.003740692	-0.000518000	0.001157816	-0.000673000	-0.002120442
-0.000424000	-0.002328715	-0.001244627	-0.001581964	-0.001564908
-0.005994341	0.002681941	0.000191000	-0.003195001	-0.004259654
-0.001508249	0.003551355	0.002004810	-0.004000722	-0.004209886
-0.002377980	-0.000171000	0.002687537	-0.002261257	0.000459000
0.000229000	0.001919112	-0.001678212	0.004536676	-0.000494000
-0.002668256	0.000746000	0.001533937	0.000136000	-0.000953000
-0.003190094	0.000192000	0.001253670	-0.003240099	0.000497000
-0.004087557	0.000517000	-0.000397000	-0.000852000	-0.003123276
0.001175988	-0.001413856	0.003833091	-0.000163000	0.000106000
0.002406841	-0.004700483	0.002480437	0.001288799	0.001421723
-0.003586386	-0.000646000	-0.003995239	0.003334681	0.002764264
0.000734000	0.000902000	-0.001644718	0.002503162	-0.005711735
-0.001341856	-0.000526000	0.001468864	0.002956144	0.001067871
-0.003630313	0.001535040	0.000440000	-0.003416014	0.004244325
-0.001844165	-0.005473002	0.006534345	-0.002208842	-0.004372203
-0.001587036	-0.001161731	0.000120000	0.000185000	0.001018384
0.002732278	-0.003474238	-0.003189317	-0.000941000	0.001715017
-0.000567000	0.001741227	0.008067288	0.004016978	-0.000956000
0.002713169	0.003378626	0.002717574	-0.000170000	0.001866850
0.000802000	-0.001862509	0.002286120	0.002177710	-0.005093200
-0.003705053	-0.003230736	0.001205906	-0.000306000	-0.001815526
0.006174563	-0.001378575	0.000667000	-0.001559028	-0.004053640
-0.000447000	0.001973322	-0.003273394	0.002420386	0.000083500
0.002942303	0.006443072	0.000459000	0.000891000	0.000689000
-0.000764000	0.001371830	0.003425651	-0.006717499	0.005517612
-0.002365282	-0.000532000	-0.001337911	-0.003418349	-0.001585226
0.000215000	0.000432000	-0.001980133	-0.000664000	-0.001271357
0.002898445	0.001028768	-0.001484043	0.000848000	0.000560000

0.001830660	0.003960294	-0.001340018	-0.000811000	0.001529873
0.002084053	-0.002506462	0.002857583	0.002069172	-0.001853775
0.000256000	-0.003817919	0.000589000	0.003414469	-0.001776593
-0.002649089	0.001173915	0.001153715	-0.002405452	-0.002324732
0.003497797	0.001170768	0.002948704	0.000853000	-0.000421000
0.000369000	0.000983000	0.000229000	-0.000894000	0.000368000
0.002557891	-0.004543172	0.001387604	-0.002016671	-0.001923228
-0.001036206	0.000992000	-0.000716000	0.000936000	-0.001210362
0.002986706	-0.004859582	0.002651786	0.001310767	-0.001354467
0.003039683	-0.000750000	0.002568715	0.003587074	-0.004223502
-0.004871953	-0.002446209	-0.000731000	0.002763084	-0.000431000
-0.003035362	0.000705000	-0.001633632	0.002765645	-0.002120013
-0.000545000	-0.002267040	0.000962000	0.000449000	-0.001686744
-0.002087035	-0.000908000	0.005816410	-0.000394000	-0.002171434
0.004693897	0.001215240	0.001140736	0.001181422	-0.002329056
-0.001678167	-0.001906285	-0.002530014	0.000641000	-0.003100301
-0.001297525	-0.002677563	0.000055400	0.001381355	-0.003740941
0.001314408	0.000778000	-0.001252871	-0.001355789	-0.000800000
-0.005380826	0.006796102	0.000144000	-0.001011057	0.000082500
0.001857765	-0.000842000	0.001584020	0.001738604	-0.002613606
-0.003613276	-0.000452000	-0.001193169	0.000155000	0.000948000
-0.000336000	0.001256676	0.001001608	0.000662000	-0.000756000
0.003234202	-0.003050365	0.003327650	0.003934641	-0.002077985
-0.005416969	-0.002546935	0.002434672	-0.000191000	0.000349000
0.001029062	-0.000432000	-0.001296101	0.000634000	-0.000717000
-0.007867695	0.002712626	0.001136774	0.001732052	-0.000974000
0.000104000	-0.005562784	-0.002309204	-0.001937773	-0.001580050
-0.002311481	0.000222000	-0.001066971	0.003116238	-0.001256963
-0.005078211	-0.000809000	0.000782000	-0.000440000	-0.001519575
-0.003964133	-0.000109000	-0.001418851	0.002214837	-0.001537655
-0.003746469	0.003837194	-0.002634866	-0.000521000	-0.000575000
-0.001554540	0.002325433	0.000369000	0.002535191	-0.001039625
0.001740500	0.001093865	0.006211278	0.004952502	-0.001035573
-0.001687658	0.000056600	-0.004000290	0.002275260	-0.001880937
0.001056132	0.001467863	-0.001085752	0.001574312	-0.002640503
0.002974778	0.001563036	0.003530179	0.000844000	-0.000860000
0.001147224	-0.001552164	-0.001355378	0.000849000	-0.001843403
0.002895883	0.001419651	0.000685000	-0.002278525	0.000430000
-0.000903000	-0.001824840	-0.000460000	-0.002093358	-0.001018500
0.005393093	-0.003663386	-0.003472550	0.001360702	-0.000168000
-0.000113000	-0.002669650	-0.001557779	0.001524762	-0.000961000
0.004896407	-0.003242513	-0.000031600	-0.000890000	-0.001712608
0.007251617	-0.001640608	-0.001621789	-0.007323362	-0.000488000
-0.003479842	0.000280000	-0.004419196	-0.007286334	0.001488628
-0.002125879	0.001951302	-0.001827193	-0.001759564	-0.002786747
0.004424370	-0.001556666	-0.005276408	-0.001870234	-0.000654000
0.005864478	-0.000417000	-0.005168174	-0.005670571	-0.001144849
-0.002982755	-0.000104000	-0.000830000	0.000471000	0.000946000

0.000979000	0.001094793	-0.001406120	-0.000973000	-0.001770471
-0.000002310	0.002192879	-0.000610000	0.000358000	-0.000116000
0.003068402	0.000841000	-0.004526237	-0.002851049	-0.000739000
0.001639623	-0.000204000	-0.001566475	-0.002978340	-0.002947924
0.003083455	-0.001957918	0.005178910	0.004829595	-0.002341766
0.007082304	0.001896666	-0.004135332	0.000821000	0.000961000
-0.002201888	-0.004354818	-0.001126843	-0.001332775	-0.000620000
0.006221104	0.002715061	-0.002493670	-0.002845883	-0.002532517
-0.000297000	0.004912721	0.000859000	-0.001890378	-0.001036591
-0.001582029	0.009781367	0.000436000	-0.002689937	-0.001153084
-0.001914301	0.001977161	-0.001137968	-0.001955734	-0.002368014
-0.000267000	-0.000070000	0.001656612	-0.002532347	-0.004211860
0.001443975	0.001794377	0.000980000	0.001987088	0.000683000
0.002384043	-0.001271417	-0.000004100	0.001543893	0.004243104
-0.001071988	-0.001180790	0.003360119	-0.008006741	-0.000103000
0.001404979	0.002204488	0.002205954	-0.002488503	-0.000788000
-0.000744000	-0.000041600	-0.000085800	-0.003013262	0.001341287
-0.001915093	0.002048310	-0.007366438	-0.000406000	0.000656000
-0.000232000	0.003798154	0.000602000	0.003468009	-0.002971789
0.001175181	-0.001596836	-0.000973000	-0.001131026	-0.003507402
0.000755000	0.005288255	-0.000684000	0.000436000	-0.000148000
-0.001760295	-0.002081724	0.001836345	-0.004099932	0.000144000
0.005666906	0.001678180	0.000144000	0.001405769	0.002805247
-0.004328512	0.002223206	-0.004194716	0.000134000	0.002529323
-0.000712000	0.004671625	-0.001858439	0.002131282	0.005674219
0.002216848	-0.000704000	-0.004071777	0.002119604	-0.000550000
-0.003322933	-0.000344000	-0.001056317	0.005659840	0.001791544
0.000727000	0.003334087	-0.004276238	-0.002298478	-0.001297289
-0.001342318	0.001055426	-0.003544574	-0.001679626	0.000062400
0.001076017	0.001115478	-0.003120583	-0.003518130	0.001518189
0.004510764	0.001594038	-0.004735886	-0.001007499	-0.002151822
-0.003469854	0.002642897	-0.001635381	0.001033133	-0.000282000
0.001052258	-0.003912734	-0.002442955	-0.005142913	-0.000046500
-0.002013598	0.000590000	-0.005135494	-0.000384000	0.002455385
-0.001416814	0.000591000	-0.003235990	0.000601000	0.000815000
-0.004074597	-0.009594819	-0.003486390	-0.000294000	0.003424242
-0.003400838	0.003163489	-0.003162908	-0.002866987	-0.000277000
0.002069523	0.005216361	0.000931000	0.000731000	0.001789406
0.000610000	0.000001070	0.000652000	-0.001466894	-0.000285000
-0.002549063	0.002528133	0.002824587	0.000115000	0.002955691
-0.003176418	0.007155461	0.003225948	0.003327579	-0.000096500
0.000565000	0.007154977	-0.000736000	-0.000025900	-0.001763034
0.001297132	-0.000898000	-0.000434000	0.000944000	-0.003729964
0.001286499	0.000803000	-0.002536232	0.003352492	-0.000987000
0.000621000	0.000276000	0.000387000	0.002843908	0.000727000
-0.002216422	0.003635344	-0.001643019	0.004257063	0.000987000
0.001678560	0.004195605	0.001586774	-0.000187000	-0.000613000
-0.001061624	-0.001657800	-0.006067720	0.000399000	-0.001466676

-0.002708585	0.003575055	0.001661562	-0.000825000	-0.002230217
0.002744570	0.000668000	0.003345097	0.003040406	-0.003099299
-0.001369676	0.000609000	0.005254328	-0.002011618	0.004948705
0.000023500	-0.003368284	-0.001611508	-0.003102363	0.001728884
-0.004529460	-0.000135000	-0.003137752	0.003513590	0.001592244
-0.002413858	-0.003332247	0.000484000	-0.000457000	-0.002812526
0.001771075	0.005427012	0.002118908	0.002556065	0.002104948
-0.002248955	0.005155195	-0.003907746	0.005730516	0.000750000
0.000252000	-0.001428998	-0.001622603	0.003692533	-0.000962000
0.001700292	-0.001489549	0.003370632	-0.002494582	-0.002665389
-0.003588692	0.004768662	-0.000901000	0.003465232	-0.001906859
-0.000353000	0.002704038	-0.004048888	0.000321000	0.000586000
-0.006853608	0.001681589	-0.004318752	0.000938000	-0.002000660
0.003666263	-0.001332206	-0.001119035	-0.000007830	0.003237815
-0.003956394	0.003450314	0.001619253	-0.001115007	0.000002660
0.005188160	0.005956384	-0.000543000	-0.000097900	-0.001705713
0.002090632	0.002165085	-0.001621320	0.002175761	0.001941560
0.001877165	0.001788359	-0.005489512	0.000192000	-0.001857952
-0.001409993	-0.000278000	-0.000347000	-0.000810000	0.008464024
-0.000327000	0.001909198	-0.006528513	0.001424674	-0.000659000
0.003101594	0.000371000	-0.004315297	-0.000168000	0.003349148
0.001464373	0.000030900	0.001728568	-0.001057007	-0.000510000
0.002338964	0.004363664	-0.003691516	0.003756392	0.001847995
0.000862000	0.008071211	0.000756000	-0.000224000	-0.003807481
0.002937624	-0.002615754	0.001170956	-0.000800000	-0.003180784
0.005496169	0.002774032	0.001020222	0.004556857	-0.000287000
0.001436766	0.004016805	0.000606000	-0.002434651	-0.000248000
0.004357407	-0.001616925	-0.001171887	0.003113203	0.000041000
0.003359514	-0.004670278	-0.001404537	0.000920000	-0.001991418
-0.000246000	0.004008835	0.003239237	0.000703000	0.000708000
-0.001785443	0.001915510	0.009073378	0.002577778	-0.002653697
0.003601985	0.003081648	-0.000758000	-0.001888735	-0.000626000
0.003961175	0.002973399	0.002819758	-0.000321000	-0.000174000
-0.001792169	0.000835000	-0.000550000	-0.000742000	-0.001625017
-0.000911000	-0.000010900	0.000634000	0.001170882	-0.004454006
0.002024405	-0.005249573	-0.002053171	0.000642000	-0.003556765
0.001812119	0.001397293	-0.004343615	0.001294833	-0.002199828
0.002147162	0.002169013	0.002847901	0.003177579	-0.001684740

PC20	PC21	PC22	PC23	PC24
0.001719603	-0.005946156	0.000521000	0.002784372	-0.001331489
-0.000204000	0.001158445	-0.002014184	0.002749617	-0.001729173
-0.001192783	0.000252000	0.000912000	0.000681000	0.000543000
-0.000241000	-0.001873958	0.000847000	-0.000656000	0.005593588
0.000315000	0.001455515	-0.003708015	0.003844006	-0.003519530
0.004184676	0.000118000	-0.001774267	-0.003004005	-0.000904000
0.000510000	-0.004978197	0.000876000	-0.002026190	-0.001056781
0.001848511	-0.001462804	-0.000872000	-0.002484684	-0.001884025
0.001060881	-0.000741000	-0.003397975	0.001235269	-0.001079236
0.000393000	-0.004148095	-0.002357878	-0.000287000	0.002391966
-0.003069149	-0.001782223	-0.002115177	0.002573081	0.000111000
0.001102001	0.000714000	0.002371362	-0.004071875	0.000772000
-0.002607002	0.000145000	-0.000755000	-0.003640900	0.001235712
-0.001167899	-0.001846168	-0.002336127	0.003430503	0.001321461
0.000996000	-0.002455200	-0.002409232	0.001205376	-0.000445000
-0.000389000	0.001685426	-0.003395834	0.001386972	0.001758883
0.001453876	-0.002305812	0.001044243	-0.000696000	0.000683000
-0.005948187	0.000454000	0.002685260	-0.002964191	0.006901867
-0.000216000	-0.001332882	-0.003039445	-0.000436000	-0.000269000
-0.002612093	-0.002938109	-0.001652129	-0.001422191	-0.001656330
-0.001119108	0.001375215	0.001445000	-0.003618606	0.001881060
0.001064261	-0.002852559	-0.002227441	-0.001592223	0.001107783
-0.001028362	0.000191000	0.000568000	0.001860700	0.000282000
0.005589894	-0.001539737	0.000574000	0.000180000	0.001483609
0.001133402	-0.004235303	0.002702418	-0.002096859	-0.000174000
-0.000678000	-0.000542000	-0.000980000	-0.000809000	0.002287947
-0.001616444	0.004282150	0.000602000	0.001673755	-0.005980445
-0.001465814	0.001088212	-0.001798793	-0.000076300	0.001236912
-0.000486000	0.001132858	0.003328268	0.000186000	-0.000240000
0.001582548	-0.001159578	-0.000583000	-0.001583021	0.000896000
0.005640606	0.001003859	0.000961000	0.001571785	-0.001659063
0.000187000	-0.000964000	0.001626664	0.000599000	0.000012200
0.002575700	0.003192117	0.002590102	0.001783128	0.000909000
0.002241953	-0.000608000	0.000857000	0.001735821	0.001476379
-0.000226000	-0.000738000	0.003663562	0.001352108	0.002165047
0.000471000	-0.000252000	0.002315180	-0.001148308	-0.002430583
0.005193832	0.003011964	-0.000068100	-0.002130211	-0.003044266
0.000967000	0.002768731	-0.000000878	0.001148977	-0.000344000
0.000157000	-0.000830000	-0.001571858	-0.001220539	-0.000648000
0.003200514	-0.000907000	-0.002553714	0.001181250	0.001556127
-0.001144650	-0.002581387	0.000143000	0.000152000	-0.000511000
-0.000144000	-0.000564000	-0.001684128	0.003230021	0.001117389
0.000389000	-0.000855000	-0.001008423	-0.000606000	-0.000649000

0.002780892	-0.000643000	-0.000922000	0.001958675	-0.000767000
0.000443000	-0.002734186	-0.001516223	0.000618000	-0.002541096
-0.001038759	-0.000206000	0.000356000	-0.000331000	0.000534000
0.000405000	-0.001327027	0.001170027	0.005140348	-0.003000429
-0.002582217	-0.002751024	-0.000995000	0.000760000	-0.002581371
-0.002496398	0.000142000	-0.003451112	0.001334967	0.000605000
-0.000547000	-0.002904186	0.003443328	0.000585000	0.000321000
-0.000275000	-0.003768262	0.004793452	0.000862000	0.000498000
-0.000494000	0.002449281	0.000916000	0.004704474	-0.000460000
0.001560714	0.000569000	-0.001192832	0.003789671	0.001528112
0.003515932	0.000812000	0.000120000	0.000805000	-0.001340443
-0.001399085	-0.000954000	-0.001186754	-0.000526000	-0.002013088
-0.001649638	0.001395308	-0.002155304	0.001511903	-0.002659154
-0.003861037	0.003182276	-0.001336492	0.005353864	0.000710000
0.000444000	-0.001251785	-0.000297000	0.000167000	-0.003028594
-0.001338702	0.002946574	0.001518912	0.003811802	-0.003166532
-0.000798000	-0.000991000	0.000453000	0.002855595	0.002851419
0.001172186	-0.002343573	0.000221000	-0.001063465	-0.002981997
-0.000111000	-0.001289396	-0.002851989	0.003500851	0.000474000
0.003086512	0.000471000	-0.001862025	0.002375507	0.003021040
0.002428393	0.001544626	-0.001661339	-0.000446000	0.001262417
-0.003057751	-0.001951355	-0.003892520	0.002782293	-0.000576000
0.003913289	-0.001424599	0.003016788	0.001854869	0.000717000
-0.003547236	0.000416000	-0.002980726	0.003429470	0.003099717
0.002863078	-0.001795711	0.003430970	-0.000397000	0.002041225
0.000969000	-0.001334190	-0.001629267	0.001212060	-0.000093100
0.000338000	0.001799585	-0.000907000	-0.000268000	0.000873000
0.002760213	-0.002924315	0.000182000	0.000035100	-0.001877348
0.001544612	-0.004816894	0.005072781	-0.001770319	-0.000408000
0.002600865	-0.002911866	-0.003200742	0.000558000	0.001196508
-0.000431000	-0.002585608	0.000713000	0.001120604	0.000717000
-0.001459031	-0.003963016	-0.001620669	-0.002429413	0.000903000
-0.004769757	-0.004234132	0.003406040	-0.001600866	0.002117139
-0.000479000	0.000262000	0.000915000	0.000920000	0.000930000
-0.000272000	0.000270000	0.000102000	-0.001968104	0.001763132
-0.000679000	-0.002034990	-0.002747558	0.000583000	-0.000945000
0.003690415	0.000748000	0.003513519	0.001658344	0.005721356
-0.002275389	-0.009163730	-0.000414000	-0.002618122	-0.000609000
0.002028713	0.002078192	-0.001109943	0.001989254	0.000362000
-0.001793212	0.001286145	-0.000929000	0.000252000	0.001023473
0.001673643	0.000042500	-0.001971747	0.004298696	0.000090000
0.003266316	-0.002281661	-0.001053848	0.002505749	-0.001610047
0.000178000	-0.002564989	-0.002076708	-0.000018300	-0.003041149
0.000804000	-0.001878688	-0.000303000	0.002827552	0.001731210
-0.001468948	-0.002041431	-0.000357000	0.003069594	-0.002660125
0.000971000	0.000686000	0.000439000	-0.000248000	-0.001427945
-0.001010381	-0.002295657	0.001995620	-0.000914000	-0.001256118
-0.002812588	0.002851835	-0.001707492	-0.002016806	-0.000075100

0.000166000	0.000979000	0.003943307	-0.003518763	0.002971920
0.001002481	-0.001846145	-0.002111575	0.001374691	-0.000021100
0.001318578	0.002277642	-0.001572288	-0.000976000	0.000063200
0.000268000	-0.000241000	-0.001547173	0.000341000	0.000851000
0.004800297	-0.001106051	-0.002360164	-0.000785000	-0.002790465
-0.000649000	-0.001421343	0.001209940	-0.000630000	-0.002761952
0.003237900	0.001106935	-0.001510432	-0.001041349	0.001265669
0.001094889	0.001776508	0.001688694	-0.002357787	0.002437374
0.001509785	-0.002126410	0.007003527	0.002645142	0.001141181
-0.000867000	-0.003188047	0.001060678	-0.002077748	0.002347713
0.003102371	0.001729416	0.004019516	0.001131365	0.003834312
-0.000482000	-0.000447000	0.000330000	0.001730948	-0.002153204
-0.001792401	-0.000284000	-0.001374986	0.003459236	-0.000247000
-0.001318008	0.002060805	0.002314971	0.000646000	0.000239000
-0.002678460	0.002014165	-0.000413000	-0.000270000	0.000464000
-0.000979000	0.001380734	0.001765392	0.003980823	-0.002058863
-0.000587000	-0.000447000	0.001644040	0.001752076	0.004137729
-0.000119000	0.003213802	-0.001747819	-0.001655716	0.000117000
-0.004035779	0.004905817	-0.002145396	0.003492877	0.001924701
-0.001299842	-0.001295572	-0.001140284	-0.002472165	0.001595123
0.001157408	-0.001442864	-0.002506233	0.000600000	0.001779667
-0.002504190	0.000331000	0.001913352	0.000137000	-0.002848695
-0.001319067	-0.000230000	0.000730000	0.002330638	0.000712000
-0.001830910	-0.001350191	-0.001170168	-0.000644000	-0.003174751
-0.002746347	-0.007565558	0.000036700	-0.002290433	-0.002135252
0.001631565	-0.003039434	0.000131000	-0.000835000	-0.001039450
0.000897000	0.000706000	-0.001458743	-0.003525366	-0.001755148
-0.000265000	-0.000028300	-0.000443000	-0.000371000	0.002404738
0.000081000	0.001228991	0.001031630	0.000578000	0.001500930
0.004329835	0.002300270	0.001821967	0.000264000	0.002698681
-0.004634472	-0.001624956	0.001842969	-0.001274971	0.000425000
0.001227342	0.001814641	0.000076600	0.000308000	0.000423000
-0.002306053	-0.001092210	-0.001166836	-0.002310650	-0.001153134
-0.002926808	-0.001670087	-0.002060833	-0.001447235	-0.002475148
-0.005581228	0.004824129	0.014685183	0.002856818	-0.010629008
0.000526000	-0.001115251	-0.000956000	-0.000490000	-0.000098600
-0.001193312	-0.002331014	0.001057720	-0.002939142	0.000476000
0.001029047	-0.001175448	-0.000116000	-0.003943735	0.001566295
0.001738834	0.000174000	0.001756061	0.000283000	-0.000011200
0.001230270	-0.001391108	0.001220432	0.001900688	0.002884472
-0.002901243	-0.000409000	-0.000954000	-0.002568274	-0.001065288
0.000078700	0.000955000	-0.000563000	0.000917000	-0.003049020
-0.002628776	0.001016890	-0.000588000	0.004450623	0.000283000
-0.000958000	-0.000137000	-0.000938000	0.000052800	-0.001626180
0.000732000	0.000381000	-0.001783419	-0.001990632	-0.003724983
0.003297278	0.001808176	-0.000083500	0.000959000	-0.001637638
0.004345580	-0.002159725	-0.000732000	0.001099624	0.000306000
-0.002933479	-0.001362102	0.000833000	-0.004123555	-0.000610000

0.003917384	0.000712000	0.000789000	0.001938061	-0.001325893
0.002331679	0.001166001	0.000836000	-0.001883758	-0.001608699
-0.000122000	-0.004700427	-0.001575647	0.001204473	-0.002144644
-0.000070200	-0.002198989	0.000162000	0.000114000	0.001198745
-0.001838466	-0.000797000	-0.000603000	-0.003018923	-0.001478381
0.000514000	-0.001882797	-0.000122000	0.000504000	-0.000194000
0.001108792	0.001654164	-0.001392032	0.000028100	-0.000712000
0.001433420	0.001086827	-0.003798481	-0.001922038	-0.003858120
0.000921000	-0.000461000	0.001120306	0.000643000	0.001509852
-0.001421767	0.001870030	0.003336148	-0.000489000	0.001508073
-0.003262946	-0.001470118	0.002598120	0.001381306	0.000078600
0.002782879	-0.000900000	0.005116825	-0.001244420	0.002960022
-0.000584000	-0.001095020	-0.000161000	0.000259000	0.001289220
0.001041128	0.000299000	0.000480000	0.001383699	-0.004014239
-0.005250195	-0.000342000	-0.002170611	0.000578000	-0.003083169
0.000645000	0.001178678	-0.001537295	0.001730899	0.000483000
-0.000911000	-0.000040400	0.000282000	-0.001073129	-0.000167000
0.000530000	0.003603046	0.001487144	-0.001895919	-0.002269283
0.003137520	-0.000089400	0.001022816	0.003184267	-0.000505000
-0.004037197	-0.002380018	0.000544000	0.000731000	0.000578000
-0.003002116	0.000420000	0.003271249	0.002430669	0.003257477
0.000185000	-0.000030300	0.000220000	-0.002650750	0.000999000
-0.001418266	-0.001619974	0.002183889	0.002300811	-0.000249000
-0.002732022	-0.000929000	-0.000748000	-0.001568622	0.004104043
-0.000169000	0.000053700	0.000383000	0.000222000	0.001682518
0.002035499	0.005029703	-0.002974941	-0.004122885	-0.004414553
0.000404000	0.002548077	-0.000455000	-0.000030000	-0.000941000
0.000148000	-0.001104734	-0.001204008	-0.000745000	0.000305000
0.001912470	-0.001181813	-0.001716409	-0.000544000	-0.001570268
0.002386397	0.000215000	0.001411955	0.001513297	0.000261000
0.006693545	-0.001630876	0.000652000	-0.002057892	-0.002301381
0.002281097	0.003180118	-0.000601000	-0.000712000	-0.001530651
-0.000093800	-0.000238000	-0.000519000	-0.002245373	0.000806000
0.000899000	-0.000274000	0.000566000	0.000125000	-0.000972000
-0.000687000	0.001606226	-0.004321220	0.001424809	-0.000186000
0.002278088	-0.000720000	0.002299439	0.001070215	-0.001854604
0.002028779	0.003634088	-0.000713000	-0.001012030	0.001184323
0.000947000	0.001699981	0.000149000	-0.000068000	-0.000123000
-0.002011928	0.003791380	-0.001718937	-0.004539406	0.000938000
-0.000655000	-0.001230372	-0.000197000	-0.002495288	-0.003912843
-0.004049124	0.001074709	0.000739000	-0.002317943	-0.000991000
0.001700654	0.001034116	-0.001255809	-0.001313085	0.000097500
0.001385428	0.005020149	-0.002726275	-0.004764712	-0.001128440
-0.003242746	0.002182667	0.001054473	0.001750361	0.001053975
0.003814210	0.001229608	0.000708000	0.001183813	0.000750000
0.003049457	-0.000133000	-0.004333143	0.000592000	-0.002676978
-0.002429961	-0.001044986	-0.002803511	-0.002953184	0.000439000
-0.000643000	0.000452000	-0.000599000	0.004575037	-0.000250000

-0.001487735	-0.000767000	-0.001673809	-0.001619562	0.000992000
-0.002310239	0.000249000	0.001506849	0.001830526	-0.001802938
-0.002220312	0.001493262	-0.002621435	-0.001578729	-0.001924599
-0.000275000	-0.001783528	-0.000235000	0.000080300	0.000244000
-0.000387000	-0.000956000	0.001082333	-0.000682000	-0.001949809
-0.001686692	0.001800002	-0.003743423	-0.003956742	0.002091881
0.001342173	0.003717024	-0.000370000	-0.003577133	0.001498836
-0.000439000	-0.001216827	-0.000543000	-0.002039801	0.001551866
-0.000545000	-0.001033990	-0.000173000	-0.002025140	-0.000408000
0.001681629	-0.001154216	0.000175000	-0.003156056	-0.002138341
-0.002112898	0.000061100	-0.001555254	-0.001530002	0.000118000
-0.000683000	-0.001515241	0.004312104	-0.002018129	-0.000282000
0.000350000	0.005063236	0.000955000	-0.001228196	0.000799000
-0.001264078	0.005204104	0.003413118	-0.003666746	0.000782000
-0.001528227	0.002454456	-0.000127000	-0.000744000	0.001543542
-0.003518852	0.003162193	-0.002529278	-0.000403000	0.003166709
0.000581000	-0.001363323	0.002511269	0.000204000	-0.002776139
0.002517865	0.000051500	0.005537011	0.003871771	0.000625000
0.001370190	-0.002016548	-0.001973262	0.003687869	-0.001243480
-0.000010600	0.000752000	0.000184000	-0.000899000	0.002668351
-0.006856056	0.003762082	-0.000035900	0.000542000	0.002050352
-0.000861000	-0.002645233	0.000297000	0.003603430	0.000608000
-0.004957809	0.002843087	-0.001694270	0.006295452	-0.000383000
0.001288178	-0.001807392	0.002535444	-0.001561055	0.000988000
0.001793756	0.002326301	0.000494000	-0.003014288	0.000880000
-0.001832965	0.000254000	-0.001521068	0.000344000	-0.000774000
0.001960812	-0.001134317	-0.001369072	-0.000528000	-0.001454855
-0.004650801	-0.000629000	-0.000545000	-0.000948000	0.002524262
-0.000526000	-0.000823000	0.000890000	0.001039102	0.003728202
-0.003062537	-0.002184256	0.002739766	-0.002106318	-0.003327446
-0.001233906	-0.001073814	-0.000115000	0.002681859	0.000642000
-0.002030951	0.001412571	0.000102000	-0.003568914	0.000238000
0.000027600	0.002516246	-0.002062791	0.000331000	0.002464848
-0.002029345	-0.000864000	0.001328033	-0.003498673	0.000338000
-0.001516733	0.000971000	0.001651133	-0.002815730	0.002042136
0.005183431	0.004840596	-0.000192000	0.000048100	-0.000435000
0.000897000	0.000061900	0.001847436	0.002017886	0.001627786
0.000322000	0.000627000	-0.000013100	-0.000290000	-0.000211000
-0.000592000	-0.002463573	-0.002991590	-0.001672723	-0.000430000
-0.001244229	0.002573149	-0.001047392	-0.001341722	-0.001089030
-0.000448000	0.004188106	-0.003080816	0.000885000	-0.001308692
0.000841000	-0.000612000	-0.002116155	0.000574000	-0.000773000
0.000670000	0.003454418	-0.001419243	-0.001563700	-0.000675000
0.006256462	-0.002268797	0.003118528	-0.001060024	-0.001261110
-0.003417782	0.001894828	0.000561000	0.000979000	-0.001044301
0.000536000	0.003977175	0.000933000	-0.000247000	0.001384025
0.001245290	0.001352797	-0.000112000	0.000616000	-0.002118340
-0.000514000	-0.001029416	0.005211825	-0.000366000	-0.002940038

-0.000818000	-0.003186149	-0.003912166	0.001434910	0.003432804
0.000544000	0.000249000	-0.002754873	-0.002535802	0.000478000
-0.005123616	-0.002439027	0.000473000	-0.001651412	0.000241000
-0.003587833	-0.001843715	0.001684430	-0.001411182	0.000773000
-0.002381708	0.002440605	0.004148968	-0.001572422	0.001324432
-0.001453181	0.001636956	0.000568000	-0.000950000	-0.000131000
0.004595041	0.000148000	0.000886000	-0.001032956	-0.000883000
-0.001738812	-0.002433041	0.000205000	-0.002071194	-0.000759000
0.000852000	0.001292724	-0.002669803	-0.002441894	-0.000478000
-0.000345000	0.000156000	-0.000940000	-0.000070700	-0.002960746
0.004057484	-0.001682638	-0.001339246	0.002998737	0.002393403
-0.000372000	0.003339699	0.000605000	0.000052100	-0.002068699
0.000387000	-0.001641544	0.000363000	-0.004583172	-0.002928052
0.000529000	-0.000901000	0.001215320	0.001233289	0.000650000
0.006166781	0.003713017	0.000093700	-0.003444019	0.002198322
-0.001162630	0.000540000	0.001637678	0.000519000	0.001849588
-0.000994000	0.001231696	0.001481330	-0.002581452	0.002312398
-0.000123000	0.001819431	0.000038700	0.002805093	0.001038593
0.001964843	-0.000555000	-0.002372218	0.000613000	0.000770000
-0.001578823	0.003506220	0.003489849	0.000504000	0.001808992
0.001668648	-0.000383000	0.004933328	-0.001700184	0.000511000
-0.000906000	-0.001096913	-0.002011175	0.000613000	-0.001776732
-0.002122959	-0.000156000	0.000492000	0.001507975	-0.002894078
0.001301175	-0.002077565	0.000191000	0.003058168	0.000095100
0.001467423	-0.000662000	0.001417207	-0.000987000	-0.001901750
0.004480062	-0.001919021	-0.002032818	-0.001287969	0.001643574
-0.002071590	0.002854824	-0.000966000	0.002473296	-0.001170648
-0.003694172	0.002962135	-0.001363046	-0.000149000	-0.001714143
0.003948675	0.005437550	0.002203290	0.003483067	-0.001382880
0.002375419	-0.002528836	-0.000587000	-0.001534457	0.000602000
-0.001145550	0.003011984	-0.000582000	-0.002915743	0.001044397
-0.002016999	0.003182849	-0.000393000	0.002048063	0.004944976
-0.002764566	0.001506921	0.001628377	-0.001005846	0.000269000
0.001289879	-0.001141623	0.001118460	-0.000274000	-0.000382000
0.004765784	0.003659847	-0.001838359	-0.000677000	0.002172801
0.004686368	0.002597916	0.000233000	-0.001239514	-0.000269000
0.000328000	-0.002043180	0.000082100	0.001745795	0.001295259
0.001013008	0.001524414	0.000388000	-0.002385947	0.002234088

PC25	PC26	PC27	PC28	PC29
0.002592185	0.000071900	-0.000400000	-0.000366000	-0.000087900
-0.001000000	-0.000422000	-0.001011011	0.000686000	0.001806283
-0.000115000	-0.000439000	0.002885171	0.000642000	-0.000586000
0.002326996	0.000036400	0.003024205	0.002760155	0.000068600
-0.000549000	0.002731307	0.001268680	0.002288691	0.000343000
0.002072532	-0.003285437	0.002237618	0.001057398	-0.002028461
0.003997343	-0.000451000	-0.000635000	0.002533525	-0.003040168
0.001191424	-0.000653000	-0.000005090	0.002478363	-0.001411476
0.002607043	-0.002116902	0.001105703	0.000471000	0.000573000
-0.000079000	0.000790000	0.000157000	0.001856389	-0.001037766
0.000100000	-0.001155497	0.000628000	-0.000500000	0.000460000
-0.000203000	-0.000938000	0.002668888	-0.000502000	-0.001288290
-0.000972000	-0.000771000	0.000969000	-0.001026983	-0.001075459
0.000707000	0.002213349	-0.000200000	-0.000682000	-0.001192597
-0.001187701	-0.001856426	-0.001170604	0.000000055	-0.001625659
0.001094288	-0.004986007	0.000449000	0.000469000	-0.000427000
0.001902896	0.000068200	-0.000520000	0.001210841	0.000289000
0.000938000	0.002109118	-0.000964000	0.003517941	0.000344000
-0.003113223	0.003175206	-0.001595269	-0.000611000	0.000342000
-0.001752252	-0.001689789	-0.001535838	0.000625000	0.000497000
0.003295174	-0.000997000	0.002107394	0.000380000	0.003598969
0.003954903	-0.000108000	0.000218000	-0.003129685	-0.000160000
0.001401177	-0.001312127	0.000766000	-0.000428000	0.001116683
0.002118719	-0.000129000	-0.001931048	-0.000741000	0.001770366
-0.002385944	0.002479859	-0.001785972	0.000849000	-0.000003470
0.001273327	-0.000486000	0.000612000	-0.000115000	0.000442000
-0.001096001	0.004278157	-0.001206559	0.001045226	0.000444000
-0.001134277	0.001499963	-0.000788000	-0.000524000	0.000596000
0.000128000	-0.000186000	0.000578000	-0.000487000	0.001405200
-0.000072800	-0.001048175	0.000200000	-0.001527905	-0.000372000
0.000395000	-0.001146567	-0.000802000	0.001601547	0.000361000
0.000505000	0.001422255	0.000734000	0.000699000	0.000763000
-0.002125820	0.001701825	0.002006074	-0.002811410	0.002170970
-0.001898789	0.000288000	-0.000125000	-0.000614000	0.000709000
0.001271952	0.000318000	-0.000159000	-0.001172285	-0.001156690
-0.000730000	-0.000438000	-0.000405000	0.000665000	-0.000600000
0.003301280	0.001201575	0.000244000	0.001891754	0.001599919
0.001173214	0.001057165	0.000437000	-0.000385000	-0.000982000
-0.000379000	-0.000696000	0.000104000	-0.002582638	0.002135020
0.003230793	0.000588000	-0.003320653	-0.003193338	0.000053600
0.001774237	-0.000120000	-0.000021300	0.002602853	-0.000218000
0.001211922	0.001348518	0.000966000	-0.001288835	0.000992000
0.000072300	-0.000962000	0.000616000	0.001467013	0.000135000

-0.000029800	-0.000309000	0.000870000	0.000999000	0.001332133
-0.001486978	-0.001551879	0.000598000	-0.000751000	0.000401000
-0.002282955	-0.001066505	0.000357000	0.001218401	0.000854000
-0.000404000	-0.001710017	0.003601446	0.003296516	0.002607077
0.001440432	-0.000343000	-0.001315898	-0.002048596	0.000003800
0.002729482	0.001327241	0.000417000	-0.002196049	-0.000997000
-0.002175003	-0.002078321	0.000867000	0.001539309	0.000571000
-0.002728591	-0.001249252	-0.000462000	-0.001997021	-0.001023482
0.002027116	0.001909595	-0.001424755	0.001577359	0.001369984
-0.003268968	-0.004139819	-0.002241239	-0.000773000	0.000655000
-0.001086654	-0.001717451	0.001507901	0.000350000	-0.000129000
-0.001699384	-0.001126722	-0.002553992	0.000547000	-0.001129954
-0.001146400	0.001529238	-0.000011700	-0.001695011	0.000248000
0.001424946	-0.000765000	0.000289000	0.000732000	-0.000219000
0.000718000	-0.003385717	0.000824000	0.001169331	-0.000860000
-0.000087500	-0.000762000	0.001409744	0.000940000	0.001511287
0.000348000	-0.000315000	-0.001606432	-0.002687501	-0.000115000
-0.001969654	0.001626600	-0.000684000	-0.000551000	-0.003125677
-0.002221766	-0.001694241	0.001084445	-0.000485000	-0.000762000
0.000046700	0.000254000	-0.001251621	0.000294000	-0.000343000
0.001116161	0.001290599	-0.002105858	0.001261096	-0.001251209
0.001090281	-0.003113273	0.000944000	-0.000930000	-0.001724117
-0.001595348	0.000727000	0.001038562	-0.000489000	0.000107000
0.001659895	0.001259278	0.000508000	-0.000997000	0.001612381
-0.000186000	0.001989406	0.000707000	-0.000330000	0.000897000
0.000380000	-0.002911079	0.001129265	0.000404000	-0.000435000
-0.002808609	0.001062189	-0.000904000	0.001001419	-0.001104890
0.001210576	-0.001671799	-0.000502000	0.001109731	-0.001554047
-0.001498098	0.003100350	0.000622000	-0.000112000	0.000868000
-0.001607931	0.002329621	0.001123989	-0.002365351	-0.000856000
0.003862579	-0.001786163	-0.000506000	-0.001993054	-0.000242000
-0.001187759	0.000101000	-0.003585215	-0.003115493	-0.000943000
-0.001089932	-0.001678460	0.000390000	0.004924546	0.000441000
-0.001045443	0.002738070	0.000124000	-0.001343120	-0.001155056
-0.002179291	-0.000334000	-0.000763000	-0.000090200	0.001699101
-0.001062541	-0.001630815	0.001524452	0.000038100	0.000322000
0.001372675	-0.000742000	-0.000613000	-0.000180000	0.000327000
0.002642146	-0.000245000	-0.000865000	-0.000655000	0.001805744
-0.000683000	-0.000623000	0.001392126	0.000193000	0.001392209
-0.001143163	0.000040600	-0.000510000	-0.000563000	-0.001832634
0.001399670	-0.002618244	0.000247000	0.000843000	-0.000747000
-0.000251000	0.000516000	0.001340470	0.001547018	-0.000227000
0.000276000	0.001257952	-0.000735000	0.000664000	-0.000649000
-0.001423142	-0.000968000	-0.000124000	0.001721493	0.001474212
-0.001234564	0.001957473	0.003218553	0.001840557	-0.001112761
0.000910000	0.000206000	0.000453000	-0.000055100	-0.000675000
-0.000745000	-0.003421358	0.000155000	-0.002895030	-0.000780000
0.002554014	-0.002131556	0.004061354	-0.002020317	-0.001894058

-0.000610000	-0.002322293	0.002521812	0.001537915	0.003467171
-0.002262211	-0.000109000	-0.000329000	0.002464483	0.001551947
-0.003449144	-0.001244740	0.002908744	-0.000046800	0.000589000
-0.000667000	0.000735000	0.001489318	0.003157832	0.003059152
0.002336197	-0.000515000	0.001541877	0.001041675	0.000581000
0.000651000	0.003045535	0.002136190	-0.000106000	0.000497000
0.001426951	0.000905000	-0.001010870	-0.000859000	0.001663189
-0.000754000	-0.000236000	-0.000174000	-0.000325000	-0.000777000
-0.003259896	0.001169849	0.001062363	0.001363207	-0.002696113
0.000508000	0.000535000	0.002392583	-0.001616758	-0.002573763
-0.001509001	-0.001467222	0.001540465	0.000065800	0.000347000
0.000160000	-0.000058500	-0.001227675	-0.000046700	0.002231826
0.001794842	0.002398172	-0.001379992	0.000352000	0.000477000
-0.002029273	0.000927000	-0.000163000	-0.000481000	0.000267000
0.000749000	0.002888734	0.000106000	-0.001143767	-0.000635000
0.000086800	0.001314505	-0.000090900	0.001192003	-0.001558597
0.000926000	0.001618951	0.001320306	-0.000635000	0.001297371
-0.000721000	-0.001326567	-0.001892284	0.000356000	-0.001336456
0.001255110	-0.001355124	-0.002146966	-0.000690000	-0.000186000
-0.001620006	-0.000740000	0.000764000	0.000694000	0.000023600
-0.000882000	0.000057200	0.002863762	0.001160930	0.001551417
0.001114167	0.000768000	0.002004806	-0.001116380	0.000353000
-0.000147000	0.000574000	-0.000864000	0.000848000	0.000998000
0.000014600	0.001310123	0.001696361	0.000619000	-0.001325683
0.002355000	-0.001849374	-0.000185000	-0.000754000	0.001674162
-0.000797000	-0.001789135	0.000150000	-0.001247723	0.001648670
0.002090392	0.001714623	-0.000710000	-0.001740204	0.001032721
0.002414629	0.000674000	-0.001237612	0.000375000	0.000845000
0.002506797	0.002853951	0.001903623	-0.001221531	-0.002559966
0.002717626	-0.000673000	-0.001376917	0.003697920	-0.003185056
0.000949000	0.001886237	0.002132662	0.001195566	-0.000255000
-0.000462000	-0.002488196	-0.002789837	0.004881297	-0.000181000
-0.000254000	-0.001078429	-0.000241000	0.001946592	-0.000051400
0.002504564	0.001282574	0.000181000	0.001077735	0.000943000
0.005992718	-0.001747199	-0.003742275	-0.002843325	-0.000815000
-0.001465509	0.001792847	0.001386984	-0.001298661	0.002011487
-0.000411000	-0.001388964	0.000149000	-0.000550000	-0.001055512
0.000347000	0.001250198	0.000252000	0.002208479	-0.001146392
0.000298000	0.000413000	0.001872673	-0.000947000	0.001555364
0.001064083	0.000811000	0.000583000	-0.001195998	-0.002013182
0.002339312	0.000122000	0.002107592	-0.000211000	0.000734000
-0.003572848	-0.000294000	0.000483000	-0.001044833	0.001959890
0.000810000	-0.000878000	-0.000332000	0.001166609	0.002433476
-0.002388947	0.003175328	0.000645000	-0.001358038	0.002079887
-0.002100091	0.002269357	0.001137235	0.001583887	-0.000606000
-0.000071100	0.001182190	-0.000477000	-0.000355000	-0.001878886
-0.000458000	-0.000825000	-0.000442000	-0.000787000	-0.002015330
-0.004433614	0.001874637	-0.004185474	0.000982000	0.001703246

0.000177000	0.000661000	0.000069800	0.003532449	-0.001148441
-0.001642212	-0.001666396	0.000926000	0.000439000	0.001832916
-0.003131877	-0.001464986	0.002336301	-0.000782000	0.002027989
0.001411481	-0.001360383	0.000127000	0.000490000	0.001215841
-0.000687000	-0.000430000	-0.001348888	0.000156000	0.000373000
0.001436269	0.001182103	0.001433251	0.000639000	0.000664000
0.001235141	0.001159127	-0.001997153	-0.001298333	0.001022405
-0.001421082	0.000321000	-0.002161938	-0.000500000	-0.000242000
0.000810000	0.003521006	-0.000569000	0.000042400	-0.001104204
-0.002017204	0.000283000	-0.000490000	0.000034500	0.001484930
0.000284000	0.002791174	-0.000281000	-0.000362000	0.001448846
-0.003575423	-0.002069363	0.000217000	0.000515000	-0.000420000
0.000912000	-0.000501000	-0.000091000	-0.000528000	-0.000823000
-0.002133761	0.001455994	0.001707133	-0.001300561	0.000111000
-0.003262339	0.000964000	0.000117000	0.004231681	-0.001918874
0.000586000	0.000138000	0.001690579	0.000851000	0.001386252
0.000171000	0.002660550	0.002471237	0.000349000	0.002532500
0.004983429	0.000753000	0.005570871	0.003650299	-0.003480873
0.000977000	-0.000679000	-0.003894906	0.000128000	-0.000771000
-0.001970612	0.002073208	0.000939000	0.000942000	-0.001482273
-0.003580807	-0.001437584	0.000929000	0.000377000	0.000599000
-0.000911000	0.001037196	0.000088200	0.000174000	-0.001170504
0.000192000	0.000130000	0.000462000	-0.001116288	0.001002316
-0.001063899	-0.000016900	0.000444000	-0.001866920	0.000745000
0.000307000	0.001251523	-0.001100504	-0.000098200	-0.000743000
0.002991005	-0.001660066	0.003784595	0.001494333	-0.000122000
0.000909000	-0.000990000	-0.000189000	-0.001510439	0.000830000
0.002548944	-0.002822153	-0.000014000	0.000652000	-0.000594000
-0.002070268	0.000223000	-0.000528000	0.000636000	-0.000493000
0.000553000	0.000467000	-0.004465164	0.000763000	-0.000415000
-0.000855000	0.002018102	0.000819000	0.000639000	0.000987000
-0.000320000	0.001151737	0.000924000	0.000619000	0.000109000
-0.000836000	0.000278000	-0.002000150	-0.000050900	0.002264241
-0.000382000	0.002975918	-0.000238000	0.000805000	-0.001448803
0.002320049	-0.000297000	-0.001256173	0.001105245	-0.000537000
0.000712000	-0.000104000	0.000535000	0.000974000	0.002060318
-0.001252422	-0.001228802	-0.001995459	-0.000719000	0.003504485
-0.000816000	-0.001726556	0.000421000	-0.000940000	0.001407047
-0.002590074	-0.002093276	-0.000738000	0.000593000	0.001016103
0.003848119	-0.000306000	-0.000401000	-0.001057876	0.001630002
-0.002931158	0.001561773	0.000975000	-0.000533000	-0.000321000
-0.004929948	-0.002314665	0.004761262	0.001192702	0.001018598
-0.000813000	0.000077900	0.002404281	-0.003025580	-0.002997739
0.000219000	0.001424339	-0.001084983	-0.001529732	0.000876000
-0.002682945	0.000168000	-0.001740454	0.000884000	0.000087500
-0.000587000	-0.000034800	-0.002405520	-0.000828000	-0.000633000
0.000003800	-0.000492000	-0.004271801	-0.000654000	-0.000003600
-0.000756000	0.001773411	-0.002081438	-0.001741853	-0.001063395

-0.001659510	-0.000355000	-0.000136000	-0.001278825	-0.001250491
-0.002353347	0.002924384	0.000243000	-0.000961000	-0.000335000
0.001050434	-0.000194000	0.000172000	-0.000328000	0.000505000
-0.001843904	0.001382270	0.000237000	0.000228000	0.000822000
0.001754226	-0.001190285	0.001185628	0.000196000	-0.000788000
0.002313816	0.000758000	-0.002012400	0.002175176	0.000696000
-0.002024543	0.001657410	-0.002702070	0.001842465	0.001022045
0.001012228	0.002378972	-0.001710834	-0.002109370	0.002294789
0.001091530	0.000826000	-0.001929309	-0.000688000	-0.000780000
0.001167751	-0.002535526	-0.001729855	-0.002128800	-0.000737000
0.002542602	0.001006044	0.000686000	-0.001270717	0.000134000
0.002308151	-0.001678802	0.000169000	-0.000147000	0.000228000
-0.001618868	0.000923000	0.000320000	0.000546000	-0.000357000
0.003187618	0.003403451	0.000206000	-0.000436000	0.002216515
-0.000792000	0.000332000	-0.001931926	0.001932995	0.001326736
-0.000454000	-0.001094072	0.000152000	0.000267000	0.001000321
0.000970000	-0.002208239	-0.001757936	-0.001471449	0.000462000
-0.000463000	-0.000738000	-0.000880000	0.000839000	-0.001678260
-0.000175000	0.003289456	-0.000426000	-0.002644644	0.000186000
0.000846000	0.002072924	0.001051169	-0.000987000	-0.002496638
0.002050908	-0.001649409	0.001322019	0.000826000	0.000222000
-0.000611000	-0.001065819	-0.000604000	-0.000204000	0.000141000
-0.000886000	0.000872000	-0.001631923	-0.000398000	-0.000346000
-0.000299000	-0.001221760	0.000385000	0.001443484	-0.000357000
-0.002863609	-0.001941954	-0.002498974	0.001065542	-0.003154717
0.001965059	0.001250730	-0.001798621	-0.000861000	0.000296000
0.002574662	0.007348409	-0.001261682	-0.002193628	-0.000699000
-0.000293000	-0.001160216	0.000229000	-0.001253396	-0.000616000
0.000033000	-0.001280056	0.001542508	0.000030800	-0.000122000
0.000496000	0.000431000	0.002233255	-0.000891000	-0.002178746
-0.003185939	0.000007110	0.002362025	-0.000283000	-0.001550888
-0.001571985	0.000069400	-0.000698000	-0.001764437	-0.001743511
-0.001079190	0.002342983	-0.000416000	0.001641565	-0.000308000
-0.000852000	0.000802000	-0.002020023	-0.001568676	-0.001814463
0.000113000	-0.002102358	-0.000773000	-0.000658000	-0.002175457
-0.002070272	-0.001412084	-0.001647808	0.000560000	-0.003017224
0.002660945	-0.000340000	0.001082005	0.002414506	-0.001792326
0.000043500	0.000259000	0.001642616	0.000245000	-0.002265035
0.000141000	0.002627245	-0.001809768	0.004037598	0.000081200
-0.000448000	-0.000849000	-0.002051592	-0.000051300	-0.003614044
-0.001013338	-0.001486193	-0.003467063	0.000256000	-0.000979000
-0.001370322	0.000656000	0.001120369	0.000608000	0.001943094
0.000603000	-0.000448000	0.001764110	-0.002273075	-0.003506370
0.000753000	0.001988478	-0.001736702	0.001520764	0.000016000
-0.000432000	-0.001311221	0.000455000	-0.001342854	0.000802000
0.002506148	-0.000882000	0.004201272	-0.001589202	0.000723000
-0.000658000	-0.002205844	-0.003398505	0.001482240	0.001618918
0.001085027	0.000351000	-0.000464000	0.001344139	0.001038793

0.000050200	0.003236251	-0.000066400	0.002706628	0.000551000
-0.000831000	0.000013100	0.002162719	-0.002773220	0.001651307
0.001002950	-0.001413680	0.000262000	-0.000549000	0.001229460
-0.001402125	-0.001948449	-0.001638562	0.000048200	-0.000664000
-0.002250864	0.004795960	-0.000516000	0.000247000	-0.002329503
-0.002150441	0.001158613	0.000752000	-0.000045200	-0.002615932
-0.002666615	-0.002208451	0.002275447	-0.002100857	-0.002671363
0.002518574	0.000865000	-0.000402000	0.000638000	0.001888904
0.002107838	0.002067581	-0.001375653	0.001032299	0.001161384
0.000373000	-0.004941646	-0.004085399	-0.000543000	0.000791000
0.000901000	-0.002662179	0.001167037	-0.002695991	-0.000308000
-0.004521344	-0.002463783	0.000924000	-0.004575036	0.001732612
-0.003421121	-0.000366000	-0.000045800	-0.003617444	0.000588000
-0.000211000	0.000249000	-0.000676000	-0.002170872	-0.000883000
0.006624224	0.002282988	-0.000946000	-0.000872000	0.002961217
-0.000168000	-0.000366000	-0.000253000	-0.000829000	0.000752000
0.000459000	-0.000647000	-0.000069000	-0.000611000	0.000930000
0.002768311	0.000030700	0.001015283	-0.000692000	-0.000031000
0.003605393	0.000136000	0.001674565	-0.001841796	0.001382840
0.000866000	-0.002249745	0.000306000	-0.001066686	0.001118152
-0.002194553	-0.000624000	-0.001432642	-0.001067460	0.001601306
-0.001474368	-0.000130000	-0.001630448	-0.000402000	-0.000540000
-0.002067643	-0.001193373	0.001604261	-0.000211000	-0.002112286
0.001333271	-0.004159987	-0.002023166	-0.000770000	-0.000525000
0.002103051	-0.001554084	0.000437000	0.001976417	0.002462128
0.000467000	-0.000398000	0.002156445	-0.002329817	-0.000627000
-0.001256040	0.001334398	0.001010887	0.001414479	-0.003296768
0.002008136	-0.000440000	0.002446028	-0.001101891	-0.001613465
0.001015481	0.001771248	0.000189000	-0.001897497	0.002444159
-0.001181934	0.001122246	-0.001466578	0.001733125	0.000598000
0.000039600	-0.000934000	-0.000314000	-0.002145375	-0.001610631
0.001115307	-0.000863000	-0.001150530	0.001160284	0.000115000
-0.000700000	0.000679000	-0.000127000	0.002408022	0.000073700
-0.001208183	0.001742565	-0.000326000	0.000239000	-0.001039464
-0.003575998	0.001507685	0.001420051	-0.000939000	0.000470000
-0.002998644	0.000887000	0.001983815	-0.002023338	0.001253509
-0.000089800	0.000877000	0.000286000	-0.001235750	-0.001122949
0.001151822	-0.001496594	-0.001495988	-0.003311453	-0.001939936

PC30	PC31	PC32	PC33	PC34
0.001245197	-0.001209198	0.001147252	0.000522000	-0.000310000
0.001053092	-0.000480000	-0.002266007	-0.001391410	-0.000310000
0.003060510	-0.001094391	-0.000890000	-0.002139453	0.000283000
0.000804000	-0.001684429	0.001197337	0.000910000	0.001075447
0.000949000	0.000357000	0.000804000	0.000479000	-0.000734000
0.001712408	0.001339339	0.000672000	0.002053375	0.000447000
-0.000871000	-0.001199608	-0.001552318	0.001157457	-0.000189000
0.000816000	0.000025500	-0.000753000	-0.000534000	-0.001089403
0.002222242	-0.002308996	-0.001591212	0.001393551	0.000957000
-0.000293000	-0.001405904	-0.000038900	0.000371000	0.001810282
0.003042651	0.000732000	-0.000144000	-0.002220816	-0.000521000
0.000432000	-0.001849728	0.000146000	-0.000578000	0.000084800
-0.000607000	-0.000541000	-0.000167000	0.000755000	-0.000429000
-0.000217000	0.000595000	-0.001076198	0.002267372	0.000714000
0.001489738	-0.000235000	0.001418756	0.000446000	-0.001509794
-0.000537000	0.000416000	0.002573690	0.001110999	0.001182225
-0.000286000	0.001729844	0.001614157	0.001784251	0.002411960
-0.000676000	-0.000661000	0.001716534	0.001145994	0.000846000
-0.000703000	0.002007272	0.000905000	-0.001495929	0.000563000
-0.001037350	-0.000616000	-0.001095277	0.002428185	0.000758000
-0.001271898	-0.000007370	0.000823000	-0.000595000	-0.000061000
0.000845000	0.000632000	0.000046200	0.000164000	-0.000389000
-0.000495000	-0.003140794	0.001795837	0.000451000	0.001291469
-0.001011512	-0.001231192	-0.001475675	-0.001385799	-0.000243000
-0.002749061	0.001882207	-0.000764000	-0.000505000	0.003624293
0.002002109	0.001219858	-0.000115000	-0.000151000	0.000942000
-0.000684000	-0.000376000	0.001466251	0.002444772	0.000385000
0.000301000	0.000106000	0.000730000	0.000800000	0.000346000
-0.000114000	0.001471769	0.001122346	0.001386974	-0.000420000
-0.002444059	0.001537139	0.000779000	-0.001091334	0.000496000
-0.000086800	0.000275000	-0.000702000	-0.000558000	-0.001097943
-0.000987000	0.001210168	0.000197000	0.000770000	0.000032800
0.000806000	-0.002117081	-0.000883000	-0.000640000	0.000974000
0.001534049	-0.001681412	-0.000750000	-0.001141902	-0.000179000
0.000094500	-0.001178454	0.001485189	-0.000458000	-0.000256000
0.000740000	0.000108000	0.000479000	0.001282619	-0.001487686
-0.000042000	0.000348000	0.000753000	0.000164000	0.001449322
-0.001761771	-0.000960000	-0.001266302	-0.000170000	0.000595000
0.000773000	-0.000416000	0.000126000	-0.001152426	0.000284000
-0.000439000	-0.002298730	0.000430000	0.002575569	-0.000288000
-0.003122393	-0.001130315	-0.001012690	-0.002899471	0.001866536
-0.000975000	0.000069300	0.000676000	-0.000529000	-0.001725277
-0.002498900	-0.000192000	0.000438000	-0.001222270	0.000108000

-0.000452000	0.000058400	0.000914000	-0.001781629	0.000168000
0.000661000	-0.001447130	-0.001780007	0.000591000	-0.000308000
-0.000607000	-0.000513000	0.000452000	0.000869000	-0.000008940
0.001606979	0.001747344	0.001684727	0.001587109	-0.000217000
-0.001476391	0.001338363	0.001232603	-0.000690000	-0.000479000
-0.000998000	0.001931625	0.001431464	-0.000035800	-0.001082050
0.000998000	0.003563648	-0.001236822	0.001459595	-0.000723000
-0.000875000	0.000239000	0.000230000	0.000066900	0.000073200
-0.002355253	0.002700121	0.000398000	0.001853708	0.001176232
-0.002317716	0.000378000	0.000670000	0.001337766	-0.001947449
-0.000014400	0.000056000	0.001488487	0.000193000	0.000792000
-0.001473826	-0.000966000	-0.002197409	-0.000118000	0.000525000
0.002142751	-0.000217000	0.001624101	0.001496355	0.000960000
0.001344550	0.000376000	-0.000838000	-0.000953000	0.001619134
0.000515000	-0.000654000	-0.001048925	-0.000042900	-0.000589000
0.001010623	0.000670000	-0.000371000	-0.001009525	-0.000004330
-0.000011800	0.000678000	0.000380000	0.001342175	0.000605000
-0.000376000	0.001275474	0.001288963	0.001126355	0.001212843
-0.002485390	0.001486772	0.000892000	-0.001123426	0.000248000
0.000026300	0.000405000	0.000788000	-0.000332000	-0.000544000
-0.002524152	-0.000815000	0.000631000	-0.001236750	-0.000243000
-0.000986000	0.001921963	-0.000189000	-0.000725000	-0.000558000
-0.000513000	0.000847000	-0.000209000	-0.000995000	-0.000883000
-0.002127125	0.000851000	0.000738000	-0.001745340	0.001353556
0.000666000	0.000129000	-0.001004700	-0.000598000	0.000367000
0.000581000	-0.001151946	-0.000264000	0.000965000	-0.001172929
0.000655000	-0.000770000	-0.002286494	-0.001105007	-0.000592000
0.001417659	-0.000562000	-0.000145000	0.001635361	0.001267702
-0.000166000	0.002499363	-0.000308000	0.001575147	0.000986000
0.000989000	0.002082948	-0.001317405	0.003340804	-0.000790000
-0.000123000	0.000326000	-0.002009797	0.001560259	-0.000252000
0.000002370	0.000159000	-0.000833000	0.001549351	-0.001247864
-0.000203000	-0.001360895	-0.000301000	0.001447002	0.000486000
0.000308000	-0.000145000	0.001972635	-0.000267000	-0.000059800
-0.001140480	-0.000115000	0.001066850	-0.002284749	-0.000187000
0.001154347	-0.002215006	-0.000910000	0.000625000	0.000500000
0.000333000	-0.000273000	0.001062053	-0.001551619	-0.001074303
-0.000098900	-0.000920000	0.000773000	-0.000672000	0.000354000
0.000477000	0.000926000	-0.003155908	0.000002190	0.000129000
0.000473000	-0.000453000	-0.001347340	0.000890000	0.000034200
-0.001259756	0.000071600	0.001431808	0.003202014	0.000836000
0.001692402	0.000847000	-0.000631000	-0.000580000	0.001970492
0.000286000	0.002462571	-0.001811106	-0.002444159	-0.000402000
-0.000174000	0.000991000	0.000050700	-0.000231000	-0.000035200
-0.000931000	-0.003744549	-0.000891000	-0.000476000	0.001537930
-0.000665000	-0.000852000	-0.002233870	0.000639000	-0.001218072
-0.001960136	-0.000975000	-0.000967000	-0.000384000	0.001355283
0.002074772	0.002250723	-0.001378250	0.000836000	-0.000989000

-0.000660000	0.001947238	-0.002353601	-0.000545000	-0.000429000
-0.000661000	0.000901000	-0.000473000	0.000213000	0.000009180
-0.002925311	-0.000020800	-0.001122667	-0.000976000	-0.001203151
-0.002322564	0.000785000	0.001152578	-0.000063400	0.000755000
-0.001422650	0.002121531	-0.001907964	0.000206000	0.000474000
-0.000321000	-0.000152000	-0.000267000	0.001645746	-0.002799065
0.000541000	-0.001323814	-0.001178896	0.000210000	-0.001367049
-0.000883000	0.000905000	0.002196654	-0.000327000	-0.000139000
-0.001168922	-0.000867000	0.000985000	0.000014200	-0.000064500
0.001220334	0.001358357	0.001483766	-0.001469820	0.001560611
0.001507945	-0.002356869	0.000311000	0.000535000	0.000164000
0.000798000	-0.000603000	0.000438000	0.001446941	0.000088900
0.003543198	-0.002291665	-0.001682440	-0.000827000	0.001281522
0.000661000	0.000692000	0.000381000	0.001356591	0.001906981
0.001428223	0.001440943	0.000674000	-0.000105000	-0.002467940
-0.001364409	0.000909000	-0.001622855	0.000208000	0.000058400
-0.000123000	-0.000450000	-0.000578000	-0.000784000	-0.000274000
0.002337174	0.001447347	-0.000813000	-0.002087672	0.000147000
0.001935750	-0.000462000	-0.000142000	0.000061800	-0.000800000
0.001627919	-0.000117000	-0.002220707	-0.000963000	-0.000307000
-0.000348000	0.000310000	0.000014100	0.001932696	-0.000144000
0.000387000	-0.000255000	-0.000459000	0.000043500	0.000400000
0.001736900	0.000881000	-0.000484000	0.000821000	-0.000243000
0.000926000	-0.002181781	0.000811000	-0.000478000	-0.000032400
0.001423059	0.000086000	0.001331937	0.000202000	-0.000245000
0.000978000	0.000342000	-0.001053235	-0.001355281	0.000044000
0.003446090	-0.000209000	-0.001037975	-0.000455000	0.000528000
-0.000557000	-0.000346000	0.001018203	-0.000921000	0.000681000
-0.000356000	0.001528701	-0.000105000	-0.000723000	-0.000704000
0.001578869	0.001949902	0.000528000	-0.001046007	-0.001082990
0.000712000	0.000273000	0.000572000	-0.001317031	-0.000942000
0.001790767	0.002702582	-0.000527000	-0.001402170	0.000726000
-0.000754000	-0.000487000	-0.000358000	-0.000454000	0.000505000
0.000638000	0.000156000	0.000413000	0.000717000	-0.001231049
0.000900000	-0.001493839	0.000017600	-0.000040800	0.000093700
0.001532600	-0.000508000	-0.000485000	0.000717000	-0.000805000
0.001441104	-0.001584783	-0.000409000	0.000631000	-0.000644000
0.001455750	0.000592000	0.000578000	-0.000204000	-0.000287000
-0.002002437	-0.000130000	-0.002063209	-0.000100000	-0.000077300
-0.002056635	0.001527763	-0.001069376	-0.000045900	0.000170000
0.001977189	0.000317000	-0.000939000	-0.000763000	-0.000742000
0.000451000	0.001911636	0.000806000	0.000759000	0.000656000
0.001447539	0.001292025	0.000477000	-0.000703000	0.000091300
-0.000718000	-0.000072800	-0.000687000	0.000996000	0.000355000
0.002303058	0.000667000	0.002387740	0.000310000	-0.000544000
0.001974553	0.002090466	0.000556000	0.000457000	-0.000094400
-0.000403000	-0.001964446	0.000052800	0.001892592	0.000075900
0.002004058	0.001337134	-0.001265948	0.000207000	-0.000195000

0.001531724	-0.002291341	0.000401000	-0.000006500	-0.001938342
0.000123000	-0.000393000	0.000714000	-0.001683160	0.001158854
-0.001368958	-0.001346669	0.002343580	0.001214538	0.000528000
-0.000334000	-0.001224348	0.000260000	-0.000659000	0.000508000
-0.000109000	-0.001169887	0.001999334	-0.000955000	-0.000773000
-0.000328000	-0.001268254	0.000651000	-0.000277000	-0.001344030
0.000846000	-0.000022900	-0.000643000	-0.002014828	0.001180738
-0.001613936	0.001561871	0.001096586	-0.001278310	0.000779000
0.000489000	-0.001137350	0.001762289	-0.000034700	-0.000810000
0.001576348	-0.000483000	0.000731000	0.000809000	0.001494040
-0.000009270	-0.001988313	0.000173000	0.000352000	-0.000090800
-0.001093766	0.000516000	-0.002136457	0.001589628	-0.001326749
-0.000260000	-0.002558751	0.000136000	0.000238000	-0.001090748
0.000354000	0.000424000	0.001380975	-0.000821000	-0.001085924
-0.001322970	-0.001554982	0.000657000	-0.000754000	-0.000347000
0.000257000	-0.001508547	-0.000137000	-0.000928000	-0.001684543
0.000787000	0.000779000	0.000409000	0.001172469	0.000412000
-0.003793512	-0.001488567	-0.001161386	0.000661000	0.001075937
0.000147000	-0.001015620	-0.000001750	0.000218000	-0.001005426
0.000550000	0.000581000	-0.000910000	0.000491000	-0.000588000
0.001811198	-0.000158000	0.001772177	-0.000839000	0.000240000
0.000495000	0.000994000	-0.000882000	0.000714000	0.001313453
-0.001095114	0.001152991	-0.001314662	-0.000238000	0.000192000
0.000587000	0.000843000	-0.000733000	-0.001210391	0.001379376
-0.000016500	-0.000264000	0.000097600	0.000462000	-0.000679000
-0.001719416	-0.001216777	0.000764000	0.003147151	-0.000150000
0.000853000	0.000780000	-0.000055200	-0.001210990	-0.000273000
0.000863000	-0.001292440	0.000291000	-0.000174000	-0.001380244
0.000238000	-0.000217000	0.000182000	0.001373969	-0.001079191
-0.000842000	-0.001030099	-0.001765030	0.000070900	-0.000741000
0.000461000	-0.000005540	0.000261000	-0.002838229	-0.001731095
0.001184293	-0.000245000	0.000478000	-0.000086300	-0.000268000
0.000685000	0.000127000	0.000954000	0.000344000	0.000520000
-0.001307880	0.000736000	0.000503000	-0.001009722	-0.000721000
0.000180000	-0.001121291	0.000137000	-0.000362000	-0.000690000
0.000782000	0.000926000	0.001046425	0.000725000	-0.001001473
-0.001316026	-0.002067606	-0.001053047	0.000148000	-0.000406000
0.000588000	-0.000703000	0.000441000	-0.000024900	-0.000260000
-0.001345347	0.000132000	0.001949835	0.000675000	-0.000976000
-0.000985000	-0.001108192	-0.000026000	0.001201334	-0.001430724
0.000365000	-0.001537538	0.001836969	-0.000241000	0.000487000
-0.001163869	-0.001633157	0.000886000	-0.001354510	-0.000343000
-0.004069820	-0.000118000	0.002244261	-0.000989000	0.001684327
-0.000760000	-0.000512000	0.000471000	0.001200108	-0.001464685
0.001585934	-0.000374000	0.001149775	-0.000285000	-0.000236000
0.001314352	0.001360924	-0.000439000	-0.000040900	0.000471000
-0.000443000	-0.000857000	0.000766000	-0.002335757	0.001222061
-0.003229411	-0.001458062	-0.000422000	0.000981000	0.001989758

-0.001448920	-0.000672000	-0.002761568	-0.001552466	0.000673000
-0.001753683	-0.000026400	-0.000035900	0.000041100	0.000307000
0.000413000	0.001813561	-0.001177735	0.001388644	0.000342000
0.001445636	0.000945000	-0.000379000	-0.000143000	0.000626000
0.001991441	0.000347000	0.000913000	-0.000706000	0.002100717
0.000503000	0.000809000	0.001376618	-0.000082800	-0.001341647
-0.001554219	-0.000166000	-0.001712198	0.000741000	0.000645000
0.000892000	-0.000425000	-0.000810000	0.000318000	0.000382000
-0.000319000	-0.002437097	0.000622000	0.000217000	-0.000925000
-0.000774000	-0.000598000	0.000521000	-0.000766000	0.000088700
-0.000768000	0.000887000	-0.001303699	-0.000679000	0.000737000
-0.000578000	0.000283000	0.001722397	-0.001158408	-0.001538640
-0.000071300	-0.000172000	-0.000222000	-0.001447259	0.000208000
-0.001183018	0.000087100	0.000103000	0.001442314	-0.001722063
-0.000358000	-0.002787910	-0.000234000	0.001631274	0.000684000
-0.002131075	0.002962301	-0.002381588	0.000659000	-0.000212000
0.000967000	0.000459000	-0.000630000	-0.001067452	0.001598690
0.000251000	0.000053400	-0.000207000	-0.000054100	-0.001079384
-0.000824000	-0.000628000	-0.001818542	0.001018599	0.000581000
0.000841000	0.000727000	-0.001698313	-0.000104000	-0.000286000
0.000498000	0.000666000	0.000199000	0.000438000	0.000839000
0.000612000	0.001332618	-0.001432792	-0.000480000	-0.000232000
-0.000306000	-0.000271000	-0.001322210	-0.001387395	0.000606000
-0.000887000	0.000446000	-0.000814000	-0.001664546	-0.000219000
0.002689967	0.001983494	0.001392954	0.002901083	0.000329000
0.001368466	-0.000755000	0.001208395	0.001040037	0.001141932
-0.002176550	-0.001224153	-0.001250997	-0.000475000	-0.000624000
-0.000374000	0.000162000	-0.002223814	0.000940000	-0.002086672
0.001347078	0.000816000	0.000874000	-0.000055100	-0.001415890
0.000118000	0.002771056	-0.000423000	-0.002104540	-0.000606000
-0.000052500	0.000154000	-0.001703312	0.000640000	-0.000037800
0.000196000	-0.002473871	0.000673000	0.000528000	0.000525000
0.000026200	0.000879000	-0.002112924	0.000081500	-0.000735000
0.001304401	0.001226469	-0.000491000	-0.001020561	-0.000459000
0.002306008	0.000705000	-0.000612000	0.002447500	0.001557110
0.000781000	-0.000821000	-0.002410867	0.000992000	0.001172823
-0.001243549	0.001060871	-0.002247414	-0.000007030	-0.001359749
-0.000146000	-0.001575489	0.001435683	-0.000459000	0.000991000
-0.001299416	-0.000550000	-0.000640000	-0.000885000	-0.001404489
-0.001720220	0.000629000	-0.000153000	0.000942000	-0.001668899
0.000054300	0.000145000	-0.000661000	-0.001537182	0.000305000
0.002061971	-0.000916000	0.000878000	-0.001457770	-0.001204651
0.000356000	0.001222119	0.000587000	0.000242000	-0.000534000
-0.000428000	0.002071158	0.000455000	0.000147000	-0.001196937
0.000317000	0.001150767	0.000496000	0.000024300	-0.001181633
0.000057900	-0.002231957	-0.000720000	-0.000290000	0.000568000
0.001344388	0.000224000	0.000834000	-0.000281000	-0.000041500
-0.003913567	0.001965698	-0.000477000	-0.000640000	-0.000285000

-0.001409566	-0.000665000	0.000420000	0.001907383	0.001498580
-0.000677000	0.001360904	0.001415897	0.000452000	-0.001089204
-0.002119561	-0.000336000	0.000681000	-0.001723449	-0.001094468
-0.000370000	0.000278000	0.000296000	0.000800000	-0.000797000
-0.000280000	0.000638000	0.001342937	0.000730000	0.000429000
0.001259972	-0.000732000	0.000028900	-0.001355743	-0.000179000
0.000010400	0.000059400	-0.000201000	0.000572000	0.001876269
0.000281000	-0.001676129	0.001417493	0.001074755	0.000129000
-0.000331000	0.000115000	0.001810109	-0.001248081	-0.000445000
-0.004474589	0.001237400	0.001603790	-0.000326000	-0.001167106
-0.002885201	0.001044472	0.002136286	0.000601000	0.000330000
-0.001740552	-0.000573000	-0.001345300	0.000804000	-0.000137000
0.002007370	-0.000976000	-0.000521000	-0.001210467	0.001420575
0.000772000	-0.000735000	0.000206000	0.000100000	0.000243000
0.002036431	0.003772827	-0.000997000	0.000504000	0.002978177
-0.001629982	-0.000408000	-0.001358671	0.000726000	-0.000234000
-0.000558000	-0.001252197	-0.001498723	0.000046000	-0.001136828
0.001038687	0.000733000	0.000276000	0.000291000	0.000580000
0.001125839	-0.000849000	0.000108000	-0.001309364	0.001816746
-0.001354265	-0.000072200	-0.000160000	-0.001050936	0.001460787
0.000823000	0.001103321	0.000716000	0.000009200	0.000219000
0.000013200	-0.000061400	0.000950000	-0.000289000	0.000504000
0.000653000	0.000892000	0.000010600	0.000756000	0.001343124
-0.000664000	0.000977000	0.001642628	-0.002459034	-0.000017000
-0.000874000	0.000999000	-0.000403000	-0.000166000	0.000658000
-0.000144000	-0.001373198	-0.002948181	0.000598000	-0.000279000
0.000271000	-0.000245000	0.000531000	-0.001977748	-0.000432000
0.000195000	0.002100745	0.000957000	-0.001341908	-0.000739000
-0.000877000	0.000085900	0.000515000	0.000605000	-0.000216000
0.000333000	-0.000101000	0.000949000	-0.000073400	-0.001068341
0.000271000	-0.000281000	-0.000483000	-0.000309000	0.000860000
-0.000129000	-0.000537000	0.000828000	0.000968000	-0.001104419
0.000195000	-0.000960000	-0.001203444	-0.000342000	0.000090000
-0.001499053	0.000759000	0.000325000	-0.000051500	-0.000269000
0.000151000	-0.001985161	0.002324323	-0.000388000	-0.000424000
0.000986000	-0.000117000	0.001457500	0.000048500	-0.000732000
0.001219257	-0.000566000	0.000244000	-0.001167843	0.000862000
0.000174000	-0.001564853	0.000200000	0.000679000	-0.000895000

PC35	PC36	PC37	PC38	Snout length	Orbit diameter	Head length
−0.000188000	−0.000286000	0.000112000	−0.000671000	3.010	3.640	12.120
0.000753000	−0.000920000	0.000545000	0.000934000	2.615	3.690	11.640
0.001005646	−0.000063600	−0.002024944	−0.000260000	3.345	4.010	13.590
−0.000437000	−0.000542000	0.000665000	−0.000806000	3.385	3.310	*
−0.001162187	0.000427000	−0.000021000	−0.001391891	3.030	3.785	11.860
−0.000255000	0.000484000	0.000345000	0.001010732	3.530	4.020	14.235
0.000556000	0.000780000	−0.001657308	0.000811000	4.100	3.990	*
0.001631895	−0.000204000	0.000817000	0.000064700	4.060	4.335	15.680
0.000632000	0.000054800	−0.000430000	0.000152000	3.185	3.885	13.610
0.000475000	−0.000243000	−0.000194000	0.000627000	3.565	3.875	13.685
−0.001232597	−0.000731000	−0.000810000	0.000467000	3.580	4.555	14.430
−0.000223000	0.000143000	−0.000286000	0.000013700	3.240	3.635	12.230
0.000008320	0.000622000	−0.000525000	−0.001103950	3.280	3.920	13.050
0.001134354	0.000705000	−0.001023776	0.000943000	2.790	3.585	11.720
−0.000908000	−0.000721000	0.000289000	0.001029637	3.195	4.145	13.800
0.000579000	−0.001328360	−0.000557000	−0.000242000	3.030	3.870	12.825
−0.000104000	−0.001521836	0.000244000	−0.000028300	3.195	3.885	13.510
−0.001190862	0.000670000	0.000358000	0.000362000	3.725	4.155	13.710
−0.000986000	0.001008677	0.000168000	0.000049000	3.580	3.620	12.435
−0.000250000	−0.000673000	0.000628000	0.000216000	2.810	3.610	11.680
0.000040500	−0.001041767	−0.001411052	−0.000531000	2.710	3.790	*
−0.000474000	0.000890000	−0.000371000	−0.000149000	2.475	3.335	10.480
−0.000233000	−0.001063913	0.000821000	−0.000713000	3.150	3.930	13.100
−0.000456000	−0.000962000	0.000573000	−0.001498080	3.215	4.100	13.520
−0.000291000	0.000652000	−0.000140000	0.000149000	3.895	3.915	13.995
−0.001288782	0.000156000	−0.000137000	0.000487000	3.585	4.185	14.435
−0.000731000	−0.000562000	−0.000714000	0.000208000	3.790	4.260	14.870
−0.000723000	−0.001272260	0.000471000	0.000130000	3.080	3.680	12.790
−0.000769000	−0.000545000	−0.001120677	0.001086189	3.070	3.770	12.845
0.001308031	0.001409731	−0.000141000	0.000521000	3.235	3.730	12.370
−0.001144948	−0.000628000	−0.000342000	−0.000620000	3.590	3.925	13.000
0.000074300	0.000847000	0.000017100	−0.000257000	3.495	4.020	14.160
0.000513000	−0.000246000	0.000566000	−0.000742000	2.800	3.765	*
−0.000836000	0.000701000	−0.000721000	−0.000513000	3.100	4.000	12.595
0.000440000	0.000968000	−0.000035700	0.000548000	3.195	3.705	12.450
0.000311000	−0.000577000	0.000188000	−0.001586524	3.825	4.055	14.455
−0.001327797	0.000219000	0.001130795	−0.000174000	3.020	4.010	*
−0.000511000	0.000016500	0.000077900	−0.000113000	4.110	4.460	14.785
0.000304000	0.001119907	0.000129000	−0.000839000	3.430	4.230	13.725
0.000129000	0.001080316	0.000220000	0.001771024	2.810	3.830	12.340
0.000420000	−0.000253000	−0.000229000	0.000117000	3.860	3.850	14.280
0.000867000	−0.001102355	−0.001458502	0.000804000	3.645	4.155	14.055
−0.000821000	−0.000628000	−0.000642000	−0.000054000	3.285	3.930	13.085

0.000391000	0.001839415	0.001322338	0.000393000	3.025	3.665	12.210
0.001135945	0.000348000	-0.000088300	0.000161000	3.180	3.875	12.820
-0.000882000	0.001703765	-0.000127000	0.000327000	3.345	3.825	12.445
0.001755229	0.000032400	0.000814000	-0.000417000	2.980	3.920	12.770
0.001119111	0.000604000	0.000684000	0.000313000	3.350	4.180	*
-0.001192383	0.000575000	-0.001426708	0.000387000	3.245	3.950	12.750
0.000095200	-0.001271020	0.000852000	0.000558000	2.960	3.695	11.885
0.000350000	0.000262000	0.000341000	0.001532224	3.190	3.950	13.045
0.001515096	-0.000944000	-0.000363000	-0.000365000	3.260	4.055	*
0.000638000	-0.000193000	0.000415000	0.000286000	3.055	3.795	12.175
-0.000289000	-0.000449000	0.000258000	-0.000936000	3.145	3.880	12.400
0.001475191	0.001138016	0.000196000	-0.001399707	3.055	3.900	12.535
-0.000024400	0.000189000	-0.000136000	0.001797581	3.290	4.045	13.080
-0.000055200	0.000059500	0.000002760	-0.002124214	3.045	3.730	12.055
-0.000321000	-0.001573636	0.000672000	0.000702000	2.855	3.595	11.790
-0.001598572	-0.000514000	0.000293000	0.000254000	2.825	3.670	12.125
0.000712000	-0.000305000	-0.000309000	-0.000321000	3.385	4.045	12.640
0.001291890	-0.000207000	0.000196000	-0.000657000	3.895	4.045	13.700
-0.000576000	0.000640000	0.000611000	0.000256000	3.190	3.760	12.030
0.000116000	0.000869000	0.001698039	-0.000749000	3.205	3.975	12.795
0.000040500	0.000568000	0.001250443	0.000903000	3.005	3.730	11.795
0.000818000	0.000206000	-0.000601000	0.000110000	3.765	4.010	14.200
-0.000436000	0.001726697	0.000035000	0.000789000	3.100	3.845	12.825
-0.000232000	0.001601515	-0.001016275	0.000102000	3.375	4.015	13.190
0.000103000	-0.000650000	-0.000238000	-0.000134000	2.900	3.785	12.190
-0.000128000	-0.000110000	0.000094400	-0.000678000	2.935	3.855	12.615
0.000150000	0.000983000	-0.001501821	-0.000013700	3.370	4.175	13.200
0.000095400	0.001062013	-0.000112000	-0.000040100	2.830	3.670	11.820
0.000198000	0.000749000	-0.000701000	-0.000141000	3.390	4.020	13.035
0.000248000	-0.001004190	-0.001325924	-0.000647000	3.700	4.120	13.640
-0.000470000	-0.000588000	0.000931000	-0.000178000	3.675	4.075	13.985
0.000027700	-0.000497000	0.000069300	0.000568000	3.485	3.990	13.325
-0.001106484	-0.000615000	-0.001112843	-0.000600000	3.835	4.095	14.250
-0.000584000	-0.000942000	0.000717000	0.001486016	3.105	3.770	11.985
0.000364000	-0.000277000	0.000754000	-0.000770000	3.060	3.655	11.785
-0.001518566	0.001240326	0.000717000	-0.001173081	3.090	3.730	12.335
0.000599000	-0.000555000	0.000138000	0.000233000	3.100	3.735	12.010
-0.000919000	0.002446188	0.000113000	-0.001104329	4.370	3.970	14.415
0.001690006	-0.000146000	-0.000624000	-0.000318000	4.200	4.480	15.070
-0.000225000	0.000283000	-0.000482000	0.000569000	3.815	4.225	14.335
0.001206442	-0.000607000	0.000117000	-0.000865000	3.805	3.980	13.765
-0.001676597	0.000519000	0.000305000	-0.000199000	3.155	3.725	12.255
0.000419000	-0.000178000	-0.000786000	-0.000579000	3.025	3.560	11.635
0.000377000	0.000180000	-0.001341159	0.000867000	3.225	3.895	12.820
-0.000869000	-0.001475393	0.000184000	0.001494003	3.475	3.715	13.045
-0.000725000	-0.000138000	-0.000074000	-0.000359000	2.960	3.610	12.155
-0.000924000	0.000253000	0.001033382	-0.001228101	3.020	3.560	12.055
-0.001719026	0.000561000	0.000196000	-0.000080000	2.880	3.800	12.080

0.000830000	0.000676000	-0.000027000	-0.000469000	3.170	3.760	12.345
-0.002067996	0.000462000	-0.000078200	-0.000351000	3.385	3.840	12.670
-0.001825566	-0.001867287	0.000286000	0.000406000	3.850	4.240	14.675
0.000840000	-0.001862568	-0.001090855	-0.000745000	3.465	4.160	13.035
0.001741596	-0.000112000	-0.000386000	0.000869000	2.925	3.795	12.190
-0.000129000	-0.000364000	0.000104000	0.000671000	3.500	4.020	13.575
0.000124000	0.000990000	-0.000538000	0.001228522	3.105	3.820	11.965
0.000306000	0.001190318	-0.000576000	-0.000826000	4.000	4.025	13.905
0.001218590	0.001414827	0.000824000	-0.000577000	3.510	3.895	13.295
0.001978755	-0.001794038	0.001507280	0.000852000	3.765	3.950	13.465
0.000477000	-0.000713000	0.000058100	-0.000060300	3.055	3.800	12.635
0.000321000	-0.000614000	-0.000371000	-0.000884000	2.875	3.705	11.560
-0.002083867	-0.000005650	0.000023100	0.001585345	3.480	4.160	13.410
0.001351831	-0.000198000	0.000555000	0.000041900	2.975	3.770	12.100
0.000553000	-0.000408000	0.000270000	-0.000038400	3.715	4.290	14.305
0.000258000	0.000230000	-0.001469961	0.000236000	2.975	3.610	12.080
-0.001164079	-0.000184000	0.002041156	0.000059600	3.735	4.290	13.700
-0.000974000	-0.000079400	-0.001246747	-0.000136000	3.285	4.125	12.965
-0.000432000	0.000069200	0.000427000	-0.000665000	3.130	3.935	12.845
-0.000368000	0.000744000	-0.000352000	0.000256000	3.440	4.180	13.830
0.001066231	-0.000008880	-0.000426000	0.001450727	3.090	3.530	11.865
0.000536000	0.000661000	-0.001608060	-0.001762914	3.000	3.845	12.495
0.000424000	-0.000193000	0.000514000	0.000429000	3.010	3.895	12.380
0.000399000	-0.000248000	0.000315000	-0.000769000	3.225	3.710	12.250
0.000478000	0.000653000	0.000029400	0.000637000	2.890	3.785	12.080
0.000393000	-0.000295000	-0.000068600	-0.000893000	3.275	3.925	13.010
-0.000352000	-0.000056400	-0.000207000	0.001295017	3.395	4.160	13.435
-0.000892000	-0.000497000	-0.000355000	0.000290000	2.750	3.610	11.490
0.000701000	-0.000419000	0.000331000	-0.001159271	3.465	3.860	12.875
0.000046300	0.000700000	0.001148808	0.000645000	3.325	3.845	12.630
0.001308763	-0.000339000	0.000444000	-0.000417000	3.670	4.140	14.015
-0.000588000	0.000187000	0.001011692	0.000007030	3.030	4.125	12.730
0.000669000	-0.000972000	-0.000184000	-0.000916000	3.005	3.670	11.875
-0.001170287	-0.000370000	0.000453000	0.000518000	2.875	3.565	11.630
-0.000095100	0.000944000	0.001473842	0.000512000	3.425	3.845	13.285
0.000184000	-0.000777000	0.000601000	0.000630000	3.360	4.070	12.995
-0.000447000	0.000316000	0.000297000	-0.001024165	3.130	3.540	12.485
-0.000422000	-0.000057900	0.001204528	0.000049700	3.695	4.100	13.940
-0.000396000	0.000483000	0.000601000	0.000043300	3.900	4.330	14.385
-0.003382449	-0.000520000	0.000811000	-0.000866000	3.785	4.055	13.550
0.000940000	-0.001067851	0.000691000	-0.000680000	3.635	3.915	13.725
-0.000297000	0.000947000	-0.000547000	-0.000904000	2.870	4.050	12.635
0.000529000	0.000749000	0.000821000	0.000214000	2.995	4.025	12.625
0.000247000	-0.000188000	0.000054200	0.000197000	3.035	3.725	11.630
-0.001094353	0.000270000	-0.001001414	-0.000779000	3.770	4.130	14.195
0.002344004	-0.000859000	0.000505000	0.000771000	3.170	3.975	12.855
-0.000806000	0.000058100	-0.000248000	-0.001214406	3.655	4.005	13.625
0.000094300	0.000193000	-0.000224000	0.001061885	3.015	4.020	12.300

-0.000563000	0.000136000	-0.001355356	0.000015600	3.300	3.680	13.060
0.000929000	-0.000778000	0.000201000	0.000885000	3.090	3.790	12.455
-0.000480000	0.001335305	-0.000652000	0.000483000	3.655	4.010	13.265
-0.000055600	-0.000339000	0.000361000	0.000251000	2.760	3.450	11.275
-0.000671000	-0.001411127	-0.000127000	-0.001498520	3.280	4.140	13.100
0.000428000	0.000058200	-0.000105000	-0.000866000	3.245	3.980	12.905
-0.000005640	-0.001469301	-0.000424000	0.001558139	3.055	4.040	12.370
-0.000937000	0.000498000	0.001298742	0.000167000	3.045	3.715	12.430
0.001033826	0.000306000	0.000853000	0.000321000	3.255	3.905	12.805
0.000210000	0.000718000	-0.001297454	0.000268000	3.125	3.960	12.315
0.001282013	-0.000167000	0.000444000	0.000130000	3.090	4.045	12.815
-0.000497000	0.000372000	0.000849000	0.000075300	3.050	4.110	13.255
0.001236742	-0.000269000	-0.001271119	0.000374000	2.715	3.705	11.525
-0.000754000	-0.000463000	0.000394000	0.000019300	3.845	4.355	14.280
0.001248125	0.000566000	-0.000900000	0.001102411	3.660	3.905	13.835
0.001302571	-0.001455161	0.000177000	-0.000657000	3.065	3.880	12.620
0.000520000	-0.000120000	-0.000379000	-0.000397000	2.985	3.680	11.925
-0.000956000	-0.000137000	-0.001823014	0.001891510	3.735	3.985	13.695
0.000445000	0.000719000	0.000430000	0.000028100	3.600	4.220	14.090
0.000052900	0.000404000	0.000463000	-0.000672000	3.710	4.065	13.495
0.000214000	-0.000337000	-0.000435000	0.000112000	3.155	3.990	12.300
0.000132000	-0.000362000	-0.000348000	0.000169000	3.710	4.085	13.760
0.000092300	-0.000516000	0.000690000	-0.000215000	3.260	3.925	12.515
-0.000303000	-0.001291751	0.001772639	0.000221000	3.600	3.925	13.105
-0.000984000	0.000580000	0.001171401	-0.000232000	3.805	4.240	14.330
-0.001136667	0.001488515	0.001421186	-0.000183000	3.805	4.245	14.485
0.000505000	-0.000022700	0.000854000	0.000364000	3.650	4.190	13.770
0.000017600	0.001518158	0.000533000	0.000076000	3.280	3.895	12.905
0.001410943	0.000326000	-0.000154000	-0.001128574	3.845	4.095	14.155
-0.000547000	0.000640000	-0.000434000	0.000458000	3.590	4.195	13.490
0.000653000	-0.001260990	-0.000696000	-0.000232000	3.525	3.930	13.060
0.001113556	0.001248931	0.000485000	0.000562000	3.935	4.325	14.855
0.000167000	-0.000250000	0.000870000	0.000487000	3.270	3.805	12.580
-0.000274000	0.000160000	0.000243000	-0.000164000	3.525	3.930	13.265
0.001315522	-0.000214000	-0.000005690	-0.000312000	3.100	4.010	12.955
-0.000257000	0.000061700	-0.000647000	0.000543000	2.805	3.650	11.700
0.000199000	-0.000982000	-0.001157867	0.001092078	2.860	3.780	11.730
-0.000373000	0.000789000	0.000785000	0.000120000	3.025	3.815	11.700
-0.002365430	-0.001335215	0.000570000	0.000073100	2.835	3.715	11.790
0.000059400	-0.000129000	0.000770000	-0.000324000	3.235	3.565	12.525
-0.000466000	-0.000162000	-0.000696000	-0.001481329	3.155	3.940	12.920
0.000026400	0.001041781	-0.000343000	0.001061972	3.110	3.550	11.920
0.001513135	0.000639000	-0.001067711	0.000048600	4.120	4.010	14.750
-0.000550000	0.000084100	-0.000550000	0.000054700	3.575	3.950	12.795
-0.000293000	0.000287000	0.000266000	-0.000244000	4.265	4.200	13.880
0.000278000	-0.000997000	-0.000050900	-0.000513000	3.260	3.875	12.825
0.001384000	0.001052240	-0.000322000	-0.000718000	3.520	3.725	12.750
0.001660155	-0.001196172	-0.000110000	-0.000212000	3.090	3.850	12.220

0.000526000	-0.000052400	0.001012724	0.001099872	3.300	3.555	12.195
-0.000482000	-0.000198000	0.000488000	0.000165000	2.940	3.670	11.790
0.000481000	-0.000007850	-0.000174000	0.000048900	3.295	3.715	12.385
-0.000959000	0.000660000	-0.000330000	-0.000253000	3.470	4.260	13.860
-0.001331463	0.000113000	-0.001753840	0.001005951	3.095	3.585	12.195
0.001563036	0.001803288	0.001752459	-0.000048500	3.160	3.825	12.685
-0.000091900	0.001791485	-0.000525000	0.000108000	3.070	3.660	11.765
-0.000315000	-0.001072064	-0.000394000	0.000570000	2.830	3.615	11.425
0.000797000	0.000275000	0.001219135	-0.000190000	3.220	3.700	12.605
-0.001572761	-0.001188824	0.000251000	-0.000400000	3.240	4.010	12.990
-0.000320000	-0.000420000	0.000178000	-0.001391502	2.975	3.870	12.375
0.000755000	0.001168598	-0.001194230	-0.000179000	3.000	3.690	12.375
-0.000032800	-0.000168000	0.000266000	-0.000254000	3.010	3.660	12.005
0.001493261	0.000236000	-0.000563000	-0.000866000	2.955	3.825	11.670
-0.000385000	-0.000134000	0.000257000	0.000024100	4.070	4.425	14.410
-0.001184354	-0.000574000	-0.000434000	-0.001701265	3.800	4.295	14.315
-0.000163000	-0.000854000	-0.001792618	-0.000638000	3.740	4.080	13.460
-0.001434840	0.000260000	-0.002510873	-0.000305000	3.500	3.860	13.105
0.000870000	0.000048900	0.000674000	0.000313000	3.430	3.970	13.030
-0.000331000	-0.001289595	0.000357000	-0.000946000	3.480	4.030	13.545
0.000406000	0.000622000	-0.000224000	-0.000302000	2.815	3.550	11.525
-0.000102000	-0.001383765	0.000114000	0.000169000	3.275	3.970	12.605
-0.000367000	0.000923000	0.000534000	-0.000094100	3.145	3.555	11.355
0.000313000	-0.000242000	0.000506000	0.000164000	3.685	3.675	13.140
-0.000085000	-0.000381000	0.000404000	-0.000513000	3.190	4.060	13.110
0.000118000	-0.001101439	-0.000542000	-0.000704000	3.265	4.210	13.130
-0.000665000	-0.000927000	-0.000270000	-0.001426019	3.335	3.790	12.410
0.001740115	-0.000198000	0.000401000	0.001334732	3.170	3.775	12.445
0.000475000	-0.000085000	-0.000327000	-0.000392000	3.495	3.785	12.710
0.000218000	0.000237000	0.000575000	0.000549000	3.165	3.410	11.855
-0.000763000	0.000446000	-0.000323000	0.000095900	3.420	3.760	13.010
0.000311000	-0.000611000	-0.000446000	0.000501000	2.745	3.540	11.605
-0.000473000	0.000192000	0.000199000	0.000536000	2.890	3.475	11.545
-0.000322000	0.000047900	-0.000553000	0.000407000	3.085	3.730	11.805
0.000166000	-0.000464000	0.001413243	-0.000152000	3.160	3.510	12.030
0.001293971	-0.000864000	0.000767000	-0.000830000	3.585	3.885	13.430
-0.000812000	0.000251000	0.000377000	0.000548000	4.185	4.185	14.665
-0.000681000	-0.001413442	0.000088400	0.000299000	4.110	4.265	15.050
-0.000391000	-0.001618230	0.000379000	-0.000659000	3.305	3.525	12.080
-0.000005390	-0.000278000	-0.001744226	-0.000153000	3.845	4.090	13.905
-0.000003070	0.001278455	-0.001043115	0.000927000	3.370	4.110	13.910
0.000917000	0.000405000	0.001393042	0.001162175	4.095	4.325	14.600
0.000170000	0.000625000	-0.001092646	0.000814000	3.530	3.775	13.375
-0.001172167	0.000126000	-0.000057900	0.000242000	3.440	4.115	13.815
-0.000385000	0.000603000	-0.000524000	0.000081200	3.785	4.270	14.405
-0.000880000	0.000933000	-0.000086000	0.000238000	3.160	3.765	13.005
-0.000072800	0.000168000	-0.000727000	-0.000479000	3.205	4.075	13.050
0.000063800	-0.000293000	0.000643000	-0.000086000	3.825	3.955	14.265

0.000297000	0.000690000	0.000923000	0.000491000	3.780	3.825	13.205
0.000585000	-0.000160000	0.001289545	0.000704000	3.170	3.790	12.605
0.001277835	-0.000011500	0.000187000	-0.000239000	3.855	3.895	13.550
-0.000781000	-0.001050835	-0.000650000	0.000686000	3.445	3.775	12.930
-0.000295000	0.000170000	-0.000678000	-0.000796000	3.290	3.640	12.645
0.001284369	-0.000045100	0.000857000	-0.000931000	3.855	3.890	13.625
-0.000167000	-0.000851000	0.001114861	0.000670000	3.000	3.520	12.265
-0.001347744	-0.000156000	0.000729000	0.001192253	2.505	3.530	11.060
-0.000872000	-0.001279901	0.000446000	0.000723000	2.690	3.570	11.585
-0.001206070	-0.000861000	0.000522000	-0.000377000	3.085	3.685	11.725
0.000061800	-0.000571000	-0.000468000	-0.000054900	3.035	3.745	12.190
0.000615000	0.001245348	0.000801000	-0.000440000	2.245	3.600	10.270
0.000295000	-0.000276000	-0.000745000	0.000460000	2.465	3.310	10.620
0.000074700	0.000737000	0.000345000	-0.000789000	3.150	3.690	11.980
0.000368000	0.001717013	-0.000468000	-0.000971000	2.990	3.690	11.870
0.000341000	0.000014400	0.000161000	-0.000165000	3.025	3.880	12.560
0.000425000	-0.000337000	-0.000759000	0.000781000	2.790	3.605	11.775
0.000304000	-0.001238541	0.000833000	-0.000956000	2.680	3.560	11.265
-0.000400000	0.001232399	0.000146000	-0.000346000	3.305	3.600	12.035
-0.000173000	-0.000092500	-0.000383000	0.001121413	2.610	3.565	11.545
-0.000638000	-0.001743961	-0.000581000	0.001185045	2.905	4.010	12.355
-0.000416000	-0.000695000	-0.001234451	-0.000405000	3.855	4.350	14.580
-0.000173000	-0.000069800	0.000866000	0.000098700	3.330	3.915	13.500
0.000276000	-0.000442000	-0.001201946	0.000650000	3.650	4.045	13.525
0.001397903	0.001668179	-0.001959744	0.000586000	3.515	3.920	13.635
-0.000353000	0.000190000	-0.000592000	-0.000508000	3.100	3.780	12.565
0.000369000	0.000933000	0.000497000	-0.000217000	3.415	3.990	13.075
-0.000457000	-0.000765000	-0.000409000	-0.000430000	3.285	3.690	12.750
-0.000790000	-0.000722000	0.000482000	0.001243184	2.955	3.720	11.725
-0.000067800	-0.001021428	-0.002212518	-0.000967000	3.680	4.450	14.165
-0.000630000	-0.001341241	-0.000119000	0.000868000	3.165	4.190	13.355
-0.000219000	0.000211000	-0.000964000	0.000545000	3.240	3.865	12.675
0.000477000	0.000293000	0.000688000	-0.000061400	3.335	3.840	12.975
-0.001678275	0.001626733	0.002036413	0.000088000	2.960	3.835	12.055
0.000283000	0.000413000	0.000005930	-0.000731000	2.445	3.465	10.855
0.001106034	0.000700000	-0.000221000	-0.000131000	2.630	3.640	11.110
-0.000954000	0.001184143	-0.000404000	-0.000655000	2.990	3.805	12.350
-0.000630000	0.000406000	-0.001476357	-0.000101000	2.750	3.525	11.480

Upper jaw length	Lower jaw length	Body depth	Length of dorsal fin base	Length of anal fin base	Width of caudal peduncle	Length of caudal peduncle	Standard length	Left plate number	Right plate number
2.920	3.755	9.215	10.145	8.955	2.285	6.185	48.99414405	7	7
2.495	3.465	8.060	9.445	7.355	2.685	5.280	47.65617338	6	7
3.010	3.875	9.115	10.950	9.870	2.140	4.160	48.04941252	6	7
2.785	3.690	8.470	10.210	9.700	1.975	3.960	47.71071435	6	5
2.990	3.805	7.970	10.755	8.150	2.065	3.650	44.81949992	10	11
3.260	4.305	9.545	10.805	10.320	2.355	5.450	53.91162449	5	6
2.855	4.180	9.260	9.065	9.510	3.030	8.950	60.06180560	7	9
3.365	4.715	9.205	10.890	9.460	3.385	7.340	57.04448754	7	8
3.240	4.195	8.675	11.925	8.570	1.890	3.660	50.11693036	7	7
3.005	4.045	9.075	10.405	8.515	3.075	7.770	55.01028480	7	7
3.505	4.490	8.955	11.205	9.840	2.680	6.045	53.82946129	6	7
2.505	3.640	8.720	10.560	7.070	2.645	8.100	50.58763130	6	6
2.930	3.880	9.150	9.820	8.605	2.780	5.210	50.34391267	6	6
2.750	3.415	7.765	7.695	8.015	2.340	7.220	48.68789277	6	6
3.155	3.850	8.515	11.765	10.560	2.140	4.800	49.63547518	7	7
2.585	3.635	7.720	10.625	8.970	2.245	4.895	47.85182458	6	7
3.155	4.045	9.035	11.710	10.170	1.945	4.305	47.34244837	9	8
2.535	3.680	9.275	11.220	8.590	2.385	4.120	44.23483429	7	6
2.140	3.240	7.460	8.595	8.185	2.015	3.355	42.80877726	7	7
2.675	3.555	8.150	9.465	8.635	2.295	5.360	47.79519468	8	10
2.395	3.230	8.450	9.780	8.560	1.840	4.460	45.32646008	7	5
2.505	3.235	7.260	7.985	7.765	1.735	4.125	40.24182406	9	7
3.060	3.720	7.620	11.365	10.345	2.100	4.405	47.17381364	7	8
2.995	4.115	8.700	10.175	9.170	2.570	5.980	53.61272683	7	7
2.770	4.000	7.975	11.005	7.580	2.325	6.175	52.02217935	6	6
3.280	3.830	8.855	9.765	7.820	2.150	5.400	59.56527469	5	5
3.060	4.090	9.500	11.825	9.540	3.135	8.375	54.92628259	6	6
2.930	3.675	7.950	11.915	8.995	1.700	3.610	48.04597348	5	5
2.420	3.685	8.610	10.425	9.430	2.040	5.410	50.58634870	10	9
2.670	3.550	9.140	10.635	9.115	2.030	5.710	48.76949279	6	6
3.050	4.060	8.785	11.610	10.800	2.245	4.420	52.03357134	6	6
3.300	4.220	8.710	10.325	8.420	3.250	9.130	58.53014407	7	6
2.550	3.445	7.735	9.885	7.385	2.050	5.705	46.23852221	8	8
2.880	3.825	8.470	12.010	9.810	1.970	5.250	51.28258877	9	9
2.655	3.810	8.365	10.815	9.445	1.970	4.780	50.14811719	7	7
3.080	4.585	8.965	9.195	9.515	2.715	6.475	55.98712392	7	8
3.230	3.885	7.380	11.600	9.375	2.210	6.235	51.43350362	6	6
3.400	5.045	10.260	13.260	9.770	2.670	6.870	59.71558558	7	7
3.035	4.145	7.745	11.340	8.560	2.180	5.610	52.23335000	6	6
2.985	3.745	8.510	8.600	8.480	2.615	6.880	51.00471295	6	7
3.205	4.135	8.170	10.630	7.680	3.155	8.955	54.84156222	8	7
3.445	4.115	8.260	8.760	9.515	2.990	5.345	52.40698702	6	5
2.970	3.950	8.810	10.355	10.775	2.310	6.360	53.97396241	7	7

2.735	3.700	7.950	9.695	9.805	1.865	3.040	44.70070052	6	6
3.190	4.050	8.285	11.855	10.510	1.995	3.200	47.97708477	6	6
2.840	4.030	8.950	10.835	10.030	2.430	5.150	50.52944225	7	7
3.105	3.950	8.310	10.355	10.260	2.185	2.770	44.56438870	6	6
3.170	4.305	8.470	12.900	10.705	2.030	4.700	52.88284506	8	8
2.760	3.795	8.135	10.410	8.525	2.010	7.160	49.69649098	7	7
2.820	3.720	8.145	10.720	8.465	2.250	5.225	50.98472967	8	8
2.975	4.180	8.715	10.610	8.430	2.505	3.215	48.86579860	6	6
2.600	3.905	9.360	11.470	8.655	2.245	4.725	49.35227880	5	5
3.160	3.940	8.935	7.130	8.990	2.250	4.580	45.02156976	7	7
2.900	3.930	8.530	9.485	8.630	2.455	4.420	48.06060639	4	4
2.960	3.895	8.165	9.055	8.390	2.285	5.470	51.23097408	7	7
2.855	4.100	8.260	10.680	8.995	2.385	4.840	45.75959281	7	6
2.560	3.595	7.630	10.060	7.165	1.845	4.590	45.57902011	6	6
2.955	3.855	7.910	10.960	9.470	1.935	4.410	46.06924097	7	8
2.575	3.260	8.110	7.085	9.400	2.250	5.420	48.05893869	5	5
3.095	4.065	8.040	10.750	10.045	1.930	4.380	47.17531817	6	5
3.195	4.140	8.520	11.835	10.755	1.885	4.175	48.23877886	6	6
2.780	3.710	8.350	9.930	10.045	1.555	4.455	45.52628815	6	6
2.875	3.835	8.005	11.150	8.935	2.255	6.225	52.04909630	6	6
2.775	3.665	8.235	9.885	9.650	2.235	5.155	47.13772503	8	9
3.585	4.620	9.430	12.440	10.830	2.315	4.565	51.26532442	7	6
2.845	3.690	8.715	10.705	9.565	2.535	5.300	50.82728608	7	6
3.055	4.020	8.885	10.755	9.825	2.295	5.445	51.31660177	7	7
2.870	3.635	8.115	10.755	9.395	1.910	3.880	46.33743796	4	4
2.920	3.615	8.875	10.750	8.255	2.255	5.805	51.76729540	8	7
3.050	3.915	9.785	12.415	10.995	2.165	5.065	50.26092506	6	6
3.005	3.740	8.370	10.770	9.015	1.920	3.970	45.71122816	5	6
2.970	3.950	8.635	9.425	8.550	2.310	3.540	45.89961861	6	6
3.450	4.350	9.080	12.550	11.125	1.960	3.390	50.26349464	8	7
3.325	4.140	9.085	12.295	10.050	2.185	6.180	57.50187753	5	7
3.225	4.040	9.415	13.030	10.300	2.390	6.315	56.84246506	6	5
2.900	3.640	9.230	11.585	10.190	2.375	4.800	54.61856970	6	5
2.790	3.770	8.610	9.855	8.690	2.155	4.215	46.51855471	4	4
2.340	3.635	7.470	11.460	9.300	2.110	4.805	47.72846600	8	8
3.025	3.880	7.485	10.375	10.825	2.020	4.930	46.82167488	6	6
2.485	3.525	7.710	10.295	9.325	2.040	4.885	46.98856802	7	6
3.400	4.600	8.770	12.235	9.655	2.320	5.440	51.62252675	5	6
3.210	4.805	8.920	13.165	11.710	2.265	4.310	52.30919365	7	8
3.340	4.320	8.735	9.345	8.515	2.385	5.585	56.66802794	7	5
2.950	4.020	8.440	10.855	9.575	2.415	4.815	51.39774086	6	7
2.765	3.975	8.625	10.185	8.155	2.300	4.145	45.52925539	4	6
3.030	3.725	8.485	10.790	9.650	1.660	3.440	43.92606886	5	5
2.805	3.765	8.065	11.275	9.630	1.985	3.670	46.11179121	7	6
3.285	3.960	8.420	10.960	10.040	2.235	2.710	48.26558266	7	7
2.820	3.655	8.490	9.265	8.245	2.375	3.930	44.33552053	4	4
2.690	3.570	8.650	9.635	8.935	2.390	5.625	47.89433018	6	6
2.690	3.650	9.470	10.695	10.230	2.030	5.725	46.28372450	6	6

2.140	3.090	8.205	10.715	9.850	2.090	5.025	48.26391200	7	7
3.010	3.990	7.570	11.040	8.445	2.230	4.880	47.60543791	7	7
3.055	4.145	9.180	13.130	10.845	2.330	4.420	53.47396969	5	5
2.740	3.965	8.415	10.940	10.590	2.255	5.100	49.28551146	5	5
2.980	3.810	8.660	9.925	9.260	1.985	3.650	43.87304904	7	7
3.430	4.225	8.455	12.400	11.365	2.220	4.840	50.04342799	6	7
2.765	3.865	7.790	10.175	9.720	2.125	2.510	42.68936403	6	5
2.855	3.910	8.495	13.725	12.845	2.050	7.245	58.77833587	6	6
2.970	3.940	8.745	11.525	10.835	2.000	3.590	47.94322284	7	6
2.815	3.820	8.410	12.015	10.950	1.760	4.285	48.34765239	7	7
2.740	3.770	8.935	11.485	8.705	2.185	4.820	49.49911307	6	7
2.385	3.550	8.795	7.335	7.485	2.525	5.500	46.06129802	5	5
3.510	4.280	8.390	11.225	9.130	2.415	4.230	48.73981688	6	7
2.850	3.700	8.545	10.935	9.225	2.205	5.195	47.09049008	6	7
3.120	4.335	9.350	12.185	11.015	2.495	6.120	56.32173248	6	6
2.725	3.695	8.400	11.365	9.910	2.100	3.850	46.14712371	7	7
3.260	4.240	9.260	12.555	10.535	1.830	3.100	48.16821981	5	5
2.885	4.005	9.010	11.230	10.005	2.145	2.960	43.95629132	6	7
3.120	3.960	8.295	10.065	9.740	2.210	2.990	42.67841673	7	7
3.310	4.245	9.335	12.495	11.535	2.285	5.390	51.74369997	6	10
2.875	3.955	8.345	10.340	9.195	1.960	3.805	45.81041196	7	7
3.140	3.950	8.595	11.215	9.880	1.895	4.480	47.27353803	6	6
2.905	3.845	8.340	11.000	9.835	1.940	3.750	44.60201188	6	5
2.735	3.675	8.705	10.145	8.785	1.900	2.670	44.81734871	5	5
2.760	3.670	8.580	9.600	8.330	2.070	4.025	43.14565976	5	6
2.830	4.105	8.600	10.060	8.875	2.165	3.300	44.91969569	7	5
3.025	4.150	9.055	11.600	10.375	2.105	4.120	49.35890477	5	5
2.595	3.480	7.400	9.325	8.720	1.900	4.585	43.93905307	9	10
3.115	3.860	8.620	11.965	10.380	2.065	6.000	54.10397822	7	6
2.725	3.640	8.695	9.775	9.040	2.950	6.010	52.19298901	7	7
3.100	4.365	8.535	9.985	9.215	3.120	7.410	55.24789878	6	6
2.745	3.950	9.630	11.160	7.775	2.425	3.410	48.77137215	5	5
2.700	3.640	8.340	10.305	9.685	2.015	3.435	43.88922372	5	5
2.495	3.445	7.980	10.210	9.305	1.985	4.085	48.12039194	6	6
3.060	3.935	9.880	11.115	9.715	2.110	1.900	43.59983239	4	4
3.005	3.925	8.485	11.160	10.245	2.215	5.550	51.94736132	5	5
2.820	3.630	7.970	10.990	10.090	1.810	4.015	45.11562131	7	8
3.160	4.280	9.305	11.105	10.155	2.530	6.320	54.26761089	6	6
3.095	4.450	9.120	11.980	10.515	2.330	6.080	55.94266436	6	6
3.375	4.385	9.515	9.940	9.065	1.885	2.350	41.19991413	7	7
2.835	4.150	8.910	11.720	10.415	1.960	4.160	48.04134485	6	6
3.120	4.140	8.890	12.645	11.345	1.990	4.430	48.87314361	5	5
2.980	3.920	8.290	12.015	10.205	1.880	4.175	47.84741386	7	7
2.730	3.520	8.340	*	*	*	*	49.03349143	9	7
3.085	4.100	9.135	10.540	9.575	2.665	5.825	53.45937346	8	7
3.150	3.900	8.275	11.730	10.320	1.655	3.255	47.74503393	7	6
3.440	4.340	8.275	9.810	10.005	2.140	5.685	49.56921205	7	8
2.475	3.600	8.375	11.155	9.240	2.635	2.920	46.21400387	3	6

3.270	4.180	8.590	10.935	9.475	2.315	4.280	48.52215188	7	7
2.530	3.650	8.875	11.440	10.090	2.430	5.275	53.20051906	6	6
3.080	3.950	7.850	10.340	8.850	1.865	2.120	44.45210550	3	4
2.490	3.350	7.845	9.275	8.480	1.860	4.005	43.82212955	6	7
3.100	3.980	9.750	13.220	11.055	1.950	4.805	49.58843421	6	6
2.890	3.955	9.750	11.600	9.480	2.340	5.205	51.53028779	7	7
2.755	3.775	9.435	11.000	9.635	1.960	2.870	42.09240243	6	6
2.740	3.945	8.350	10.350	8.575	2.255	5.835	48.04967687	6	6
3.175	3.830	8.695	11.280	9.930	2.090	3.650	46.28293275	4	6
2.765	3.775	8.750	11.115	9.795	2.085	4.260	47.41836130	6	6
3.035	3.820	8.765	9.815	8.990	2.655	6.650	51.26092444	6	6
3.000	3.820	9.565	10.865	9.895	2.490	6.655	51.12970095	7	7
2.900	3.515	7.900	10.275	8.425	1.940	3.960	46.10155607	9	9
3.350	4.685	9.395	11.050	10.880	2.270	4.565	49.39967325	4	4
3.220	4.440	9.365	10.215	9.495	2.565	2.965	47.12097560	8	8
2.840	3.890	8.925	9.870	8.655	2.535	5.255	50.10632243	6	5
2.365	3.480	8.150	10.160	8.940	2.170	5.450	50.04799389	6	7
2.930	4.210	8.560	13.150	10.450	1.975	5.255	53.94800820	8	8
3.345	4.570	9.870	*	*	*	*	50.77876510	6	6
3.180	4.195	9.225	11.080	10.450	2.240	4.880	50.25462322	4	4
2.735	3.495	8.165	10.820	9.320	2.185	5.195	49.39052995	6	7
3.140	4.155	9.180	12.560	11.000	2.395	5.935	56.43754325	6	6
2.950	3.870	8.295	11.220	9.475	1.905	4.510	51.61014826	7	6
3.115	4.000	7.995	10.180	9.035	2.075	2.910	44.94555435	5	6
3.205	4.275	8.955	10.170	11.070	2.540	6.015	59.32165308	7	7
3.225	4.480	8.615	13.150	12.655	2.055	5.260	53.35973843	8	8
2.785	3.820	7.930	9.955	9.715	2.460	6.925	55.42917656	6	5
3.140	3.920	8.175	10.805	9.585	1.945	3.630	45.76387208	6	6
3.240	4.300	8.425	11.310	8.755	2.385	4.125	49.43620079	6	6
3.410	4.465	8.375	11.940	10.710	2.385	2.965	48.49568018	6	6
2.865	3.930	8.190	9.400	8.715	2.485	5.645	50.32350365	8	7
3.215	4.320	8.660	10.590	10.430	2.855	6.280	59.44798718	6	5
2.440	3.765	8.035	9.500	9.000	2.345	4.665	47.35397133	6	6
2.935	3.985	9.110	12.080	10.605	1.805	3.750	47.47106024	6	6
3.085	4.090	8.630	11.370	9.935	1.975	2.905	44.48867080	7	7
2.795	3.515	7.350	10.915	8.260	1.955	3.960	49.90980000	7	8
2.240	3.455	8.090	9.760	8.815	2.100	4.990	46.65112782	6	6
2.580	3.445	8.425	9.775	8.615	2.040	3.905	47.12527939	6	5
2.660	3.595	8.380	10.100	8.480	2.350	4.065	45.34356734	6	5
2.915	3.740	8.200	11.040	9.760	2.095	4.015	47.25951919	8	8
2.940	3.965	9.050	11.020	10.260	2.075	4.140	49.19134533	6	4
2.610	3.530	9.335	8.245	7.145	1.925	2.645	36.94107072	6	7
3.250	4.480	9.960	12.250	10.865	1.960	1.815	45.97923837	7	6
2.955	3.915	8.725	11.120	10.240	1.875	2.420	45.69397577	6	5
3.030	4.440	8.195	10.160	10.255	2.410	4.905	50.50433771	5	4
3.025	3.555	8.005	8.090	8.840	2.475	4.905	46.98966327	5	5
2.860	3.920	8.185	11.650	9.525	1.920	4.775	47.44386771	7	8
2.905	3.725	9.000	10.355	7.985	2.250	5.065	46.99039872	6	7

2.855	3.755	8.180	11.320	9.560	1.865	3.920	46.44154098	7	5
3.010	3.725	8.060	10.590	9.140	1.875	3.815	46.15194519	6	6
2.810	3.790	8.460	11.455	9.550	1.800	2.895	46.86110985	7	7
3.170	4.110	8.680	12.690	11.225	2.450	6.700	57.62508788	6	5
2.870	3.840	8.540	10.235	8.815	1.940	3.035	44.94556901	6	5
2.510	3.810	8.590	11.500	10.345	2.245	4.355	50.46231659	7	8
2.510	3.785	7.190	9.790	8.935	1.960	2.690	39.80149795	6	6
2.780	3.505	7.885	10.630	8.880	1.685	4.110	44.76532222	6	7
3.075	3.900	8.665	10.270	10.070	1.795	3.075	43.73803971	7	7
2.995	4.020	8.975	11.420	9.915	1.950	4.070	47.81668391	8	8
2.825	3.805	8.755	10.045	9.005	2.175	5.965	48.62334395	7	6
2.510	3.600	8.980	9.105	8.475	1.940	3.800	42.63809213	11	10
2.505	3.605	8.335	11.035	9.080	2.080	6.220	48.11573446	7	6
2.355	3.520	9.700	9.305	7.535	1.885	3.705	38.15963335	6	6
3.365	4.285	9.325	12.080	11.715	2.555	2.720	50.70858823	5	4
3.420	4.535	9.420	12.815	11.650	2.215	4.005	53.65181623	5	5
3.115	4.190	8.335	11.360	9.880	2.150	4.500	51.66018116	5	5
3.060	4.100	8.410	10.155	9.210	2.225	5.925	49.56948440	8	7
3.400	4.245	8.200	10.950	9.050	1.990	3.990	49.46500104	6	6
2.955	3.870	9.400	11.005	10.055	2.095	5.200	50.77360953	5	5
2.390	3.470	7.910	9.855	8.530	1.940	5.005	45.71441170	6	6
3.220	4.005	8.740	10.415	9.650	2.105	5.005	45.96601186	5	5
2.580	3.800	8.390	9.725	8.285	2.325	4.425	42.64036865	5	5
3.120	4.130	8.595	11.155	10.075	2.065	3.970	47.57851613	7	6
3.195	4.350	9.470	10.980	9.125	2.425	3.660	46.50986500	5	5
3.230	4.195	8.695	10.775	10.810	2.085	3.625	47.39149525	5	5
2.595	3.685	7.815	10.030	9.060	1.835	1.710	42.52143277	7	6
3.025	3.900	8.275	9.490	8.790	2.090	3.365	42.73531120	5	6
2.995	3.945	7.965	10.760	9.365	1.830	3.345	45.92586912	5	5
2.505	3.490	8.035	8.640	8.000	1.815	3.715	40.68285082	6	5
3.120	4.160	8.760	10.865	9.185	1.955	3.550	48.39946445	7	6
2.525	3.330	8.030	9.665	7.415	2.080	6.235	44.64506038	6	6
2.595	3.365	7.940	8.555	8.230	2.135	3.660	40.57094769	5	5
2.675	3.665	8.430	9.315	8.600	2.225	4.410	47.07365702	4	5
2.660	3.530	7.830	9.160	8.740	2.090	4.270	45.69009720	5	6
2.940	4.110	8.045	10.235	9.505	2.340	3.300	45.80234252	5	6
3.260	4.670	9.440	14.090	12.295	1.990	4.670	57.31182383	6	5
3.435	4.620	8.690	11.685	10.670	2.655	6.605	55.89967713	4	4
2.800	3.745	7.570	8.210	7.335	2.720	3.525	43.71949284	4	4
3.135	4.380	8.830	11.770	9.515	2.815	6.025	54.64387478	6	6
3.040	4.070	8.050	11.480	10.485	2.470	6.855	52.50940164	7	8
3.130	4.585	7.905	10.215	10.420	2.445	6.025	49.85783031	5	5
2.690	4.070	8.390	10.480	9.325	2.190	4.810	49.61386738	5	4
3.220	4.320	8.810	8.680	10.615	2.380	5.970	54.66920715	7	6
3.175	4.480	8.620	11.635	9.960	2.430	4.490	51.25143322	6	6
2.950	4.075	7.225	9.350	9.100	1.630	1.640	42.22433863	6	6
2.885	3.965	7.915	10.205	9.210	2.805	5.990	50.32096592	6	6
2.975	4.300	8.810	10.315	10.555	2.265	4.615	51.61256753	7	7

2.985	3.965	7.670	8.930	9.030	2.260	3.910	44.43292016	5	5
2.705	3.800	8.175	9.685	7.905	1.945	4.715	47.91371322	7	8
3.320	4.240	8.335	10.630	8.970	2.195	3.015	46.26050621	8	9
2.720	3.685	7.660	9.885	9.380	2.360	6.260	50.05243586	8	8
2.840	3.820	8.370	10.775	9.810	2.610	6.030	51.19379972	5	6
2.915	4.045	7.835	10.975	9.430	2.240	5.810	52.75031426	8	8
2.955	3.930	7.970	10.440	9.705	1.860	3.660	45.59737428	6	6
3.010	3.540	7.790	10.415	8.690	1.670	3.730	44.11597257	7	7
2.670	3.425	7.340	9.785	7.435	1.970	4.015	44.22516183	8	8
2.530	3.470	8.770	9.240	8.155	1.955	3.245	40.67338298	6	5
2.925	3.985	8.265	8.365	8.925	1.845	4.020	42.75016682	6	7
2.355	3.205	7.450	8.405	6.385	1.840	5.215	39.70037818	11	11
2.720	3.450	7.080	8.470	7.920	1.755	5.955	43.91295083	8	6
2.560	3.500	8.060	9.580	8.995	1.850	4.580	46.79492117	6	8
2.485	3.605	7.520	8.245	8.685	1.715	2.620	40.45077472	6	6
2.895	3.685	7.695	10.380	8.995	2.120	4.720	48.21275122	8	8
2.770	3.640	7.775	10.875	9.575	1.715	3.965	44.89846037	7	7
2.605	3.370	7.420	10.100	9.470	1.620	4.315	44.68288678	10	9
2.855	3.695	8.125	9.300	7.695	2.320	6.410	48.64480928	5	6
2.285	3.355	7.950	9.365	8.130	2.085	4.345	45.07844574	6	6
2.785	3.695	9.080	9.100	9.550	1.615	1.715	38.54944389	7	6
3.485	4.475	9.100	10.385	11.245	2.295	5.115	52.32778161	6	7
2.910	3.960	8.455	10.015	9.320	2.140	3.225	47.38262102	5	5
3.155	4.430	8.610	9.520	9.640	2.230	4.960	48.65784000	8	9
2.815	4.140	8.805	10.950	10.205	2.140	3.705	48.43530701	6	7
2.880	3.765	8.720	9.985	8.840	1.910	4.040	46.71877110	7	7
2.935	3.975	8.360	10.820	10.195	2.355	5.555	53.59628170	6	6
2.790	3.800	7.855	9.730	10.260	2.225	6.485	52.75874868	7	7
2.600	3.625	7.355	9.720	8.440	2.030	5.185	48.85098031	7	6
3.380	4.425	8.625	11.730	10.915	2.300	4.750	52.14439365	6	6
3.150	3.930	8.540	10.990	10.890	2.115	6.735	54.74907758	7	6
2.720	3.705	8.020	10.265	9.965	2.095	4.170	48.98765833	6	6
2.695	3.765	8.145	9.410	9.375	2.590	7.110	54.07489854	7	7
2.825	3.755	8.015	11.085	10.550	1.810	4.615	46.83680687	8	9
2.440	3.185	7.105	7.825	6.960	1.925	4.240	40.08054198	5	7
2.370	3.360	7.685	10.030	9.100	1.710	4.885	43.79688232	6	6
2.895	3.935	7.725	9.990	10.775	1.440	3.155	43.63458939	6	6
2.630	3.335	7.830	8.925	8.300	1.920	4.445	43.34022381	6	6

Total plate number
14
13
13
11
21
11
16
15
14
14
13
12
12
12
14
13
17
13
14
18
12
16
15
14
12
10
12
10
19
12
12
13
16
18
14
15
12
14
12
13
15
11
14

12
12
14
12
16
14
16
12
10
14
8
14
13
12
15
10
11
12
12
12
17
13
13
14
8
15
12
11
12
15
12
11
11
8
16
12
13
11
15
12
13
10
10
13
14
8
12
12

14
14
10
10
14
13
11
12
13
14
13
10
13
13
12
14
10
13
14
16
14
12
11
10
11
12
10
19
13
14
12
10
10
12
8
10
15
12
12
14
12
10
14
16
15
13
15
9

14
12
7
13
12
14
12
12
10
12
12
14
18
8
16
11
13
16
12
8
13
12
13
11
14
16
11
12
12
12
15
11
12
12
14
15
12
11
11
16
10
13
13
11
9
10
15
13

12
12
14
11
11
15
12
13
14
16
13
21
13
12
9
10
10
15
12
10
12
10
10
13
10
10
13
11
10
11
13
12
10
9
11
11
11
8
8
12
15
10
9
13
12
12
12
14

10
15
17
16
11
16
12
14
16
11
13
22
14
14
12
16
14
19
11
12
13
13
10
17
13
14
12
14
13
12
13
12
14
17
12
12
12
12

Supplementary Table 3: Synopsis of morphometric variation nine-spined stickleback morphology among F2-hybrids, pure marine and pure pond fish. Given are means, sample sizes (N), standard deviations, minimum and maximum values, and coefficient of variation for each trait.

Trait	F2 cross<br>Mean	SD	Minimum	Maximum	Range	CV	N	Wild popula<br>Marine (HE<br>Minimum
Snout length	3.28760	0.37290	2.24500	4.37000	2.12500	0.11343	283	1.69000
Orbit diameter	3.88484	0.23043	3.31000	4.55500	1.24500	0.05931	283	2.87000
Head length	12.83685	0.94203	10.27000	15.68000	5.41000	0.07338	276	8.75000
Upper jaw length	2.91117	0.28074	2.14000	3.58500	1.45000	0.09644	283	2.28000
Lower jaw length	3.90111	0.32895	3.09000	5.04500	1.95500	0.08432	283	2.64000
Body depth	8.47848	0.59129	7.08000	10.26000	3.18000	0.06974	283	5.84000
Dorsal fin base length	10.57477	1.18067	7.08500	14.09000	7.00500	0.11165	280	7.69000
Anal fin base length	9.42641	1.03989	6.38500	12.84500	6.46000	0.11032	280	6.82000
Caudal peduncle width	2.18486	0.31474	1.44000	3.38500	1.94500	0.14406	281	1.13000
Caudal peduncle length	4.69598	1.31641	1.64000	9.13000	7.49000	0.28033	281	2.12000
Standard length	48.53819	4.23448	36.94000	60.06000	23.12073	0.08724	283	32.38000
L plate number	6.31802	1.21949	3	11	8	0.19302	283	8
R plate number	6.32509	1.27731	4	11	7	0.20194	283	
Total plate number	12.64311	2.35272	7	22	15	0.18609	283	

ations

L)				Pond (RYT)						
Maximum	Range	CV	N	Mean	SD	Minimum	Maximum	Range	CV	N
2.68000	0.99000	0.13431	16	4.87937	0.93554	3.00000	6.81000	3.81000	0.19173	63
3.72000	0.85000	0.08022	16	5.54476	0.66338	4.07000	6.81000	2.74000	0.11964	63
11.53000	2.78000	0.08549	16	19.76937	2.93601	13.85000	26.04000	12.19000	0.14851	63
3.04000	0.76000	0.10639	16	5.56810	0.87828	3.62000	7.05000	3.43000	0.15773	63
3.60000	0.96000	0.11117	16	6.44508	0.98167	4.19000	8.37000	4.18000	0.15231	63
9.12000	3.28000	0.13701	16	13.14714	1.88468	9.46000	16.65000	7.19000	0.14335	63
11.30000	3.61000	0.12847	16	15.47746	2.66029	10.84000	20.65000	9.81000	0.17188	63
10.51000	3.69000	0.14500	16	13.76524	2.33743	9.27000	17.52000	8.25000	0.16981	63
1.87000	0.74000	0.15600	16	2.28635	0.33965	1.28000	2.89000	1.61000	0.14856	63
6.25000	4.13000	0.25538	16	5.68603	1.01920	1.97000	7.66000	5.69000	0.17925	63
48.70000	16.32000	0.13638	16	66.05206	8.56172	48.42000	81.42000	33.00000	0.12962	63
18	10	0.20653	26	6.69697	1.18545	4	9	5	0.17701	33

Supplementary Table 4: Significant QTL regions detected with the coarse-mapping.

LG, nearest QTL, position, LOD score, LOD threshold (genome-wide level), percentage of variation explained (PVE), 1.5 unit confidence interval (CI), and high density range used for fine mapping are shown for each trait.

Trait	LG	Nearest QTL	Position (cM)	LOD	LOD Threshold	PVE (%)	1.5 CI (cM)	High density range
**PC1**	15	12340	22.66	4.95	4.40	6.70	17.63-27.30	17.63-27.30
**PC3**	7	2995	14.06	7.82	4.40	8.00	0.00-19.95	0.00-19.95
	8	15355	72.81	4.75	4.40	4.80	67.06-78.77	67.06-78.77
**PC6**	7	4772	6.98	7.72	4.60	12.00	0.00-14.06	0.00-14.06
**PC11**	7	21266	19.95	6.52	4.20	10.40	0.00-19.95	0.00-19.95
**PC13**	5	12142	24.54	4.51	4.40	7.30	19.12-29.04	19.12-29.04
**PC14**	4	16781	81.33	5.09	4.40	8.20	76.16-86.16	76.16-86.16
**PC16**	17	21707	31.00	4.52	4.50	7.30	21.33-36.07	21.33-36.07
**PC20**	17	6207	46.35	4.58	4.30	7.40	41.32-50.85	41.32-50.85
**PC33**	14	11049	89.77	4.55	4.40	7.40	84.61-94.28	84.61-94.28
**Lower jaw length**	19	27323	105.58	5.88	4.30	6.80	92.39-113.69	92.39-113.69
**Caudal peduncle length**	15	20163	12.29	6.05	4.50	8.50	12.09-17.29	7.09-17.63
**Body depth**	4	29958	56.10	5.25	4.40	8.40	43.86-61.10	48.50-61.99
**Snout length**	20	18998	46.08	4.8	4.50	6.90	41.04-51.08	41.05-51.68
**Left side plate number**	8	8763	78.77	4.57	4.40	5.60	72.81-78.77	62.18-83.38
	12	33698	74.43	4.68	4.40	5.80	68.19-74.43	68.19-79.26
	20	37804	51.68	5.19	4.40	6.50	46.08-56.68	45.01-56.08
	21	8707	84.00	6.34	4.40	8.00	78.44-89.00	68.99-90.09
**Right side plate number**	20	18998	46.08	8.1	4.40	11.60	41.04-51.08	45.01-56.08
	21	20949	68.99	7.07	4.40	10.00	62.65-68.99	68.99-90.09
**Total plate number**	20	18998	46.08	7.34	4.50	10.40	36.04-51.08	45.01-56.08
	21	20949	68.99	7.76	4.50	11.00	62.65-68.99	68.99-90.09

Supplementary Table 5: Results of ANOVAs testing for differences in traits means between individuals with different genotypes for given QTL marker (Marker). Given are trait means, standard deviations (SD), minimum and maximum trait values, as well as denominator (ddf) and numerator (ndf) degrees of freedom with F-test values and associated P-values.

Trait	Marker	Genotype	N	Mean	SD	Minimum	Maximum	ddf	ndf	F	P
Lower jaw length	27323	AA	66	0.330	0.982	−1.53	2.70	131	1	16.40	< 0.001
		CC	67	−0.329	0.893	−1.93	2.05				
Caudal peduncle length	13320	CC	77	−0.233	0.817	−1.86	1.63	125	1	28.54	< 0.001
		GG	50	0.669	1.080	−1.20	3.18				
Body depth	11319	GG	50	−0.558	0.794	−2.07	1.59	119	1	20.30	< 0.001
		AA	71	0.237	1.054	−1.69	2.94				
Snout length	21583	CC	66	0.369	0.959	−1.33	3.21	118	1	12.22	0.001
		TT	54	−0.247	0.963	−1.93	2.28				
Left plate number	22134	CC	57	6.053	1.093	3	8	133	1	5.09	0.026
		TT	78	6.513	1.225	4	11				
	11482	TT	65	5.923	0.973	4	8	117	1	15.20	< 0.001
		CC	54	6.833	1.551	4	11				
	18769	CC	70	5.814	1.011	4	9	129	1	22.18	< 0.001
		AA	61	6.771	1.309	4	11				
Right plate number	22134	CC	57	6.158	1.099	4	9	133	1	2.60	0.109
		TT	78	6.500	1.297	4	11				
	11482	TT	65	5.800	1.078	4	8	117	1	19.05	< 0.001
		CC	54	6.815	1.455	4	11				
	18769	CC	70	5.814	1.011	4	8	129	1	20.34	< 0.001
		AA	61	6.803	1.481	4	11				
Total plate number	22134	CC	57	12.211	1.980	8	17	133	1	4.38	0.038
		TT	78	13.013	2.349	8	21				
	11482	TT	65	11.723	1.941	8	16	117	1	18.66	< 0.001
		CC	54	13.648	2.895	8	22				
	18769	CC	70	11.629	1.859	8	17	129	1	24.13	< 0.001
		AA	61	13.574	2.649	8	22				
